# An Approach to Modify 14-Membered Lactone Macrolide Antibiotic Scaffolds

**DOI:** 10.1021/acs.joc.1c02799

**Published:** 2022-01-12

**Authors:** Anna Janas, Krystian Pyta, Maria Gdaniec, Piotr Przybylski

**Affiliations:** Faculty of Chemistry, Adam Mickiewicz University, Uniwersytetu Poznanskiego 8, 61-614 Poznan, Poland

## Abstract

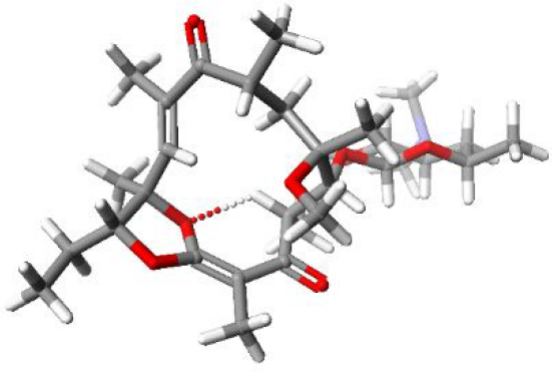

A ketolide derivative with (12*R*)-configuration was obtained via a novel ketene acetal in acidic conditions. The structure of this atypical β-keto ketene acetal intermediate within the macrocyclic system has been determined by NMR and X-ray methods. The use of basic conditions at an elevated temperature yielded new, doubly α,β-unsaturated ketone macrolide derivatives with (4*E*)-configuration as two conformational isomers of folded-in or folded-out conformations.

Lactone macrolides with different sizes of the macrocyclic rings, such as, clarithromycin (**1**), erythromycin, azithromycin, or leucomycins, are used worldwide as agents in antibacterial therapy.^1,2^ The resistance of different bacterial strains to these antibiotics prompted medicinal chemists to design novel modifications on the basis of their structural alterations mainly within the hydroxyl or ketone groups from aglycone and/or saccharide.^3^ Modifications and the total synthesis of 14-membered clarithromycin **1** and its congeners have been performed widely with the conserved stereochemistry at carbon C(12) and the ketone at the C(3) position, ketolide group antibiotics.^4−7^ Recently, it has been shown that contraction of the aglycone ring of classical lactone antibiotics contributed to the formation of effective antibacterial agents against multiresistant Gram-negative pathogens.^8^ It should be mentioned that the aglycone ring of 14-membered lactone macrolides is much more strained compared to 15- and 16-membered ones and hence is more prone to intramolecular reactions.^9^ Thus, all the above-mentioned transformations of 14-membered lactone aglycones or saccharide parts belonging to **1** or erythromycins, hindered often by intramolecular ketalizations, were performed for years via identical intermediates, contributing to the generation of a huge number of comparably substituted macrolide structures.^3,10−14^ Taking into account the above facts, here, we propose another type of approach to aglycone modifications of erythromycin-like antibiotics via novel intermediates, offering the opportunity for greater semisynthetic structural diversification of 14-membered macrolide scaffolds.

Clarithromycin (**1**) was transformed into **5** via multistep synthesis according to a previous report (Scheme 1).^15^ The synthesis of product **5** has been confirmed by X-ray crystallography (Figure S1). During attempts of an alternative preparation of **5**, 2′-acetylated **2** was isolated and its X-ray structure was also determined (Figure S2). Structures of **5** and 2′-acetylated **2** show *E*-configuration of the double bond C(10)=C(11). In the next step, **5** was treated with CDI (1,1′-Carbonyldiimidazole) in basic conditions yielding carbamate **6**, widely used in the syntheses of telithromycin-like ketolides.^16^ The isolated product **6** was converted into a novel-type structure for lactone macrolides, i.e., β-keto ketene acetal intermediate **7** (Scheme 2), at basic conditions and elevated temperature. The yield of this reaction was good (72%), and the structure of **7** was proven by NMR. This type of structure is unexpected and rare since, within 1,3-ketoester systems, usually, the ketone group is the favorable site of enolization.^17^ Similar types of ketene acetals, i.e., synthons used for heterocyclization, were obtained in a different way, not involving 1,3-ketoesters.^18^ To the best of our knowledge, only two examples of such ketene acetals within the 1,3-ketoester moiety have been characterized by X-ray crystallography in the literature.^19,20^ After smooth and efficient methanolysis of the acetyl group of **7**, derivative **8** was formed (Scheme 2). The X-ray structure of **8** and the spectral characteristics (Supporting Information) allowed us to unambiguously confirm the unique structure of the lactone macrolides, containing the protected lactone in a bicyclic system within a β-keto ketene acetal moiety (Figure 1) and the altered absolute configuration at C(12) when compared to **1**. The newly formed ketene acetal moiety has *Z*-configuration and is conjugated with C(3)-ketone in an *s*-*trans* arrangement. This type of structure can be alternatively formed via stereospecific S_N_1- or S_N_2-type mechanisms due to the nucleophilic attack of the anion localized at the oxygen of the ketene acetal on the electrophilic carbon atom C(12). On the one hand, when one takes into account steric crowding within the C(9)–C(13) portion and the macrocyclic ring strain, where tertiary carbocation at C(12) is being attacked by the enolate oxygen, the S_N_1 mechanism should be favored. On the other hand, the basic conditions and the nature of the leaving group should favor the S_N_2-type mechanism.

**Figure 1:**
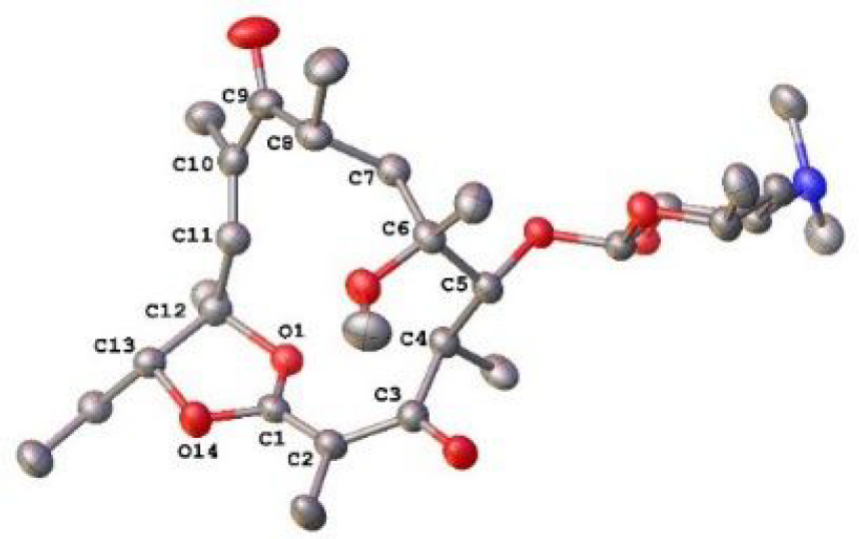
ORTEP diagram of **8**. Displacement ellipsoids were drawn at the 50% probability level.

**Scheme 1:**
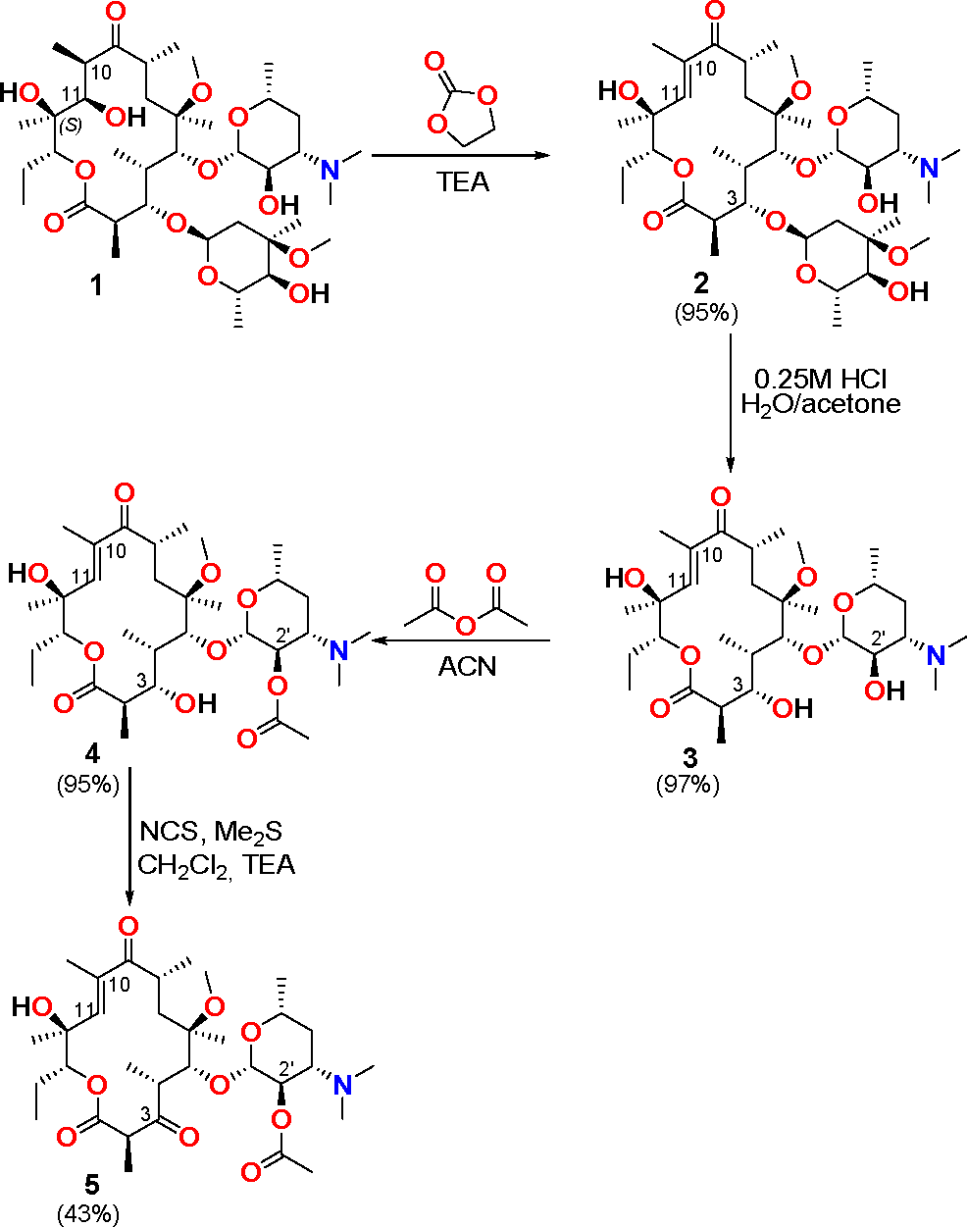
Synthetic Route to Clarithromycin Derivative **5**

**Scheme 2:**
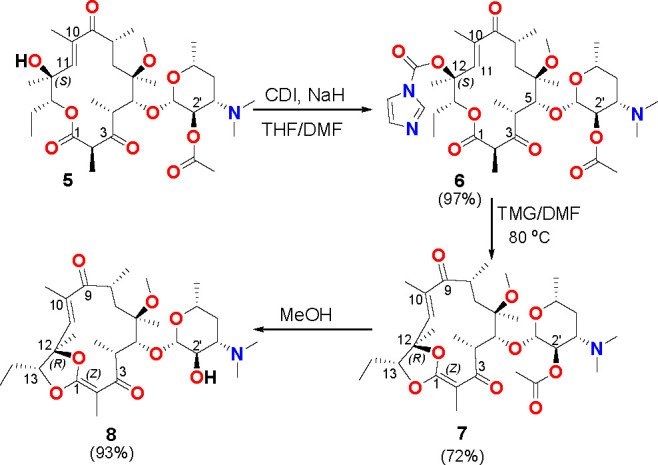
Synthesis of Novel Clarithromycin Intermediates **7** and **8**

Compound **7** is sensitive to acidic conditions as shown in Scheme 3. The treatment of **7** with an acidic methanol solution afforded derivative **9**, possessing an inverted absolute configuration (12*R*) relative to its epimer **5** (12*S*) as well as **1**, erythromycin, azithromycin, telithromycin, or their many congeners. At these experimental conditions, protonation of the ketone group at C(3) enables the nucleophilic attack on the electrophilic carbon C(1), followed by the ketene acetal C(1)–OC(12) cleavage with the retention of (12*R*) absolute configuration relative to **7** (Scheme S1). The stereochemistry of **9** was evidenced by ^1^H–^1^H NOESY (Figure S3) and chemical shift differences found in ^1^H and ^13^C NMR spectra compared to that of **5** (Tables S1 and S2). The altered stereochemistry at C(12) enables structure stabilization via the H-bond between C(12)-hydroxyl and the lactone group, as was evidenced by the shift of the *v*(O_12_–H) band toward lower wavenumbers (∼3100 cm^–1^) and the reduced intensity of the band (Figure S4). This intramolecular H-bond impacts the conformation of the whole aglycone, which is well reflected in chemical shift differences of **5** and **9** in ^1^H and ^13^C NMR spectra (Tables S1 and S2). The DFT calculated structure of **9** stabilized by an intramolecular H-bond is shown in Figure 2.

**Figure 2:**
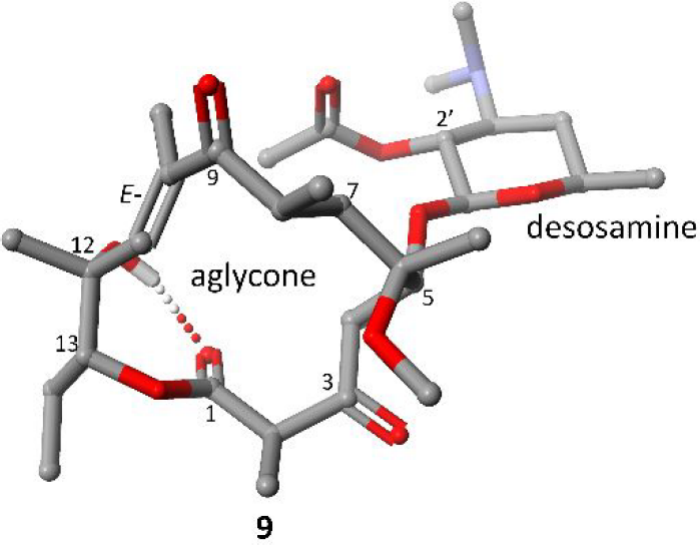
Calculated structure of **9** on the basis of assumed contacts found in the NOESY spectrum using an XC functional: BLYP-D3 with basis set TZ2P (ADF package; see the Supporting Information).

**Scheme 3:**
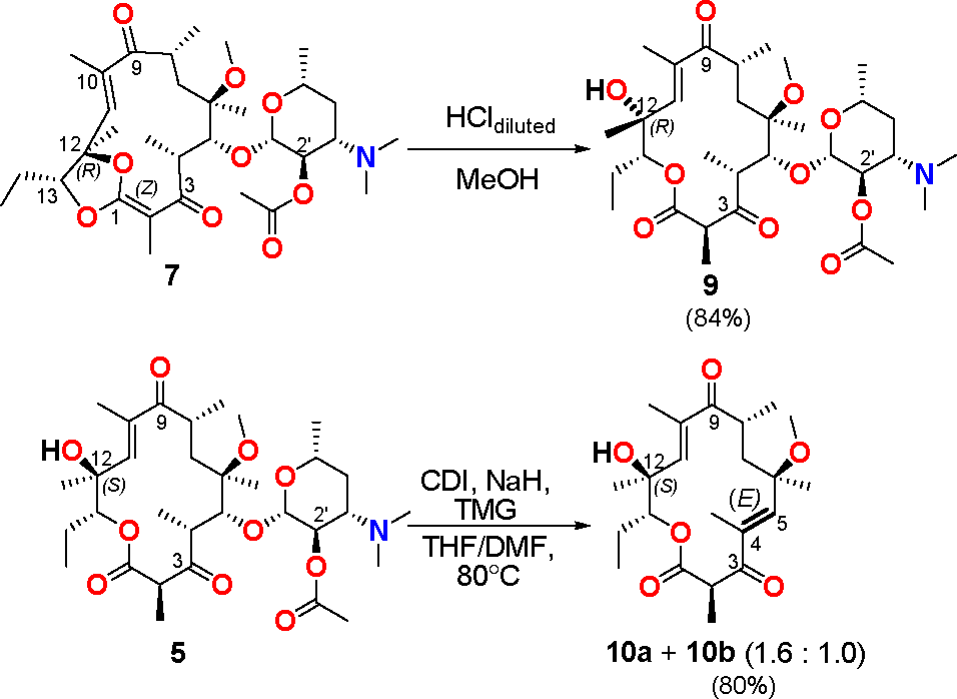
Synthesis of Clarithromycin Derivatives **9** and **10**

An attempt at shortening the synthetic path leading to **7** from **5** afforded an 80% yield of novel-type product **10**, lacking desosamine saccharide (Scheme 3). The unexpected elimination of the saccharide portion from the C(5) position of **5** is realized via the E1cB mechanism, as noted earlier for 16-membered lactones.^21^ Broadening of the ν(C=O) band at ∼1670 cm^–1^ in the FT-IR spectrum of **10** showed that, in addition to unsaturated ketone at C(9), another unsaturated one at C(3) also exists (Figure S5). However, this type of unsaturated derivative **10** is formed as the mixture of two compounds (**10a** and **10b**) having an identical 4*E*-configuration of the conjugated double bond with the ketone at C(3), as proved by 1D and 2D NMR spectra (Tables S1 and S2; Figure S6). Initial attempts to separate **10a** and **10b** by the HPLC method with C_18_ and chiral columns and different mobile phases were unsuccessful (Figure S7). Further efforts at HPLC separation of **10a** and **10b** on a C_8_ column with the reversed stationary phase were successful (Figure S8); hence, **10a** and **10b** can be described as the two different conformational isomers instead of “conformers”. Moreover, the occurrence of these two conformational isomers was evidenced independently on the solvent used (CDCl_3_, CD_3_CN, DMSO-*d*_6_; Supporting Information). ^1^H–^1^H NOESY contacts for each of the conformational isomers in the mixture allowed one to assume the initial structures for the DFT calculations (Figure S6). As shown in Figure 3, when one takes into account the mutual arrangement of the methyl groups of aglycone, **10a** has the folded-out structure, in contrast to **10b**, which is rather compact and has a folded-in structure.^22,23^ The E1cB elimination of the mycaminose saccharide requires the formation of enolate, and hence, the epimerization at C(2) is possible. In NOESY spectra of **10a** and **10b**, the strong contact H(2)···H(5) and the weak one H(2)···H(11) were noted (Figure S6). A comparison of the calculated structures of **10a** and **10b** and their C(2*S*) epimers (Figure S9) shows that the presence of the above-mentioned ^1^H–^1^H contacts in the NOESY spectra excludes epimerization at C(2). In the structure of **10b**, the two antiparallel oriented α,β-unsaturated ketone moieties are stabilized via a mutual π–π stacking interaction, where distances of C(4)···C(11) and C(5)···C(10) are 3.7 and 3.6 Å, respectively, as calculated by the DFT method (Figure 3). In contrast, these α,β-unsaturated ketone moieties in **10a** are much more distant from each other, as shown in Figure 3 (left, top).

**Figure 3:**
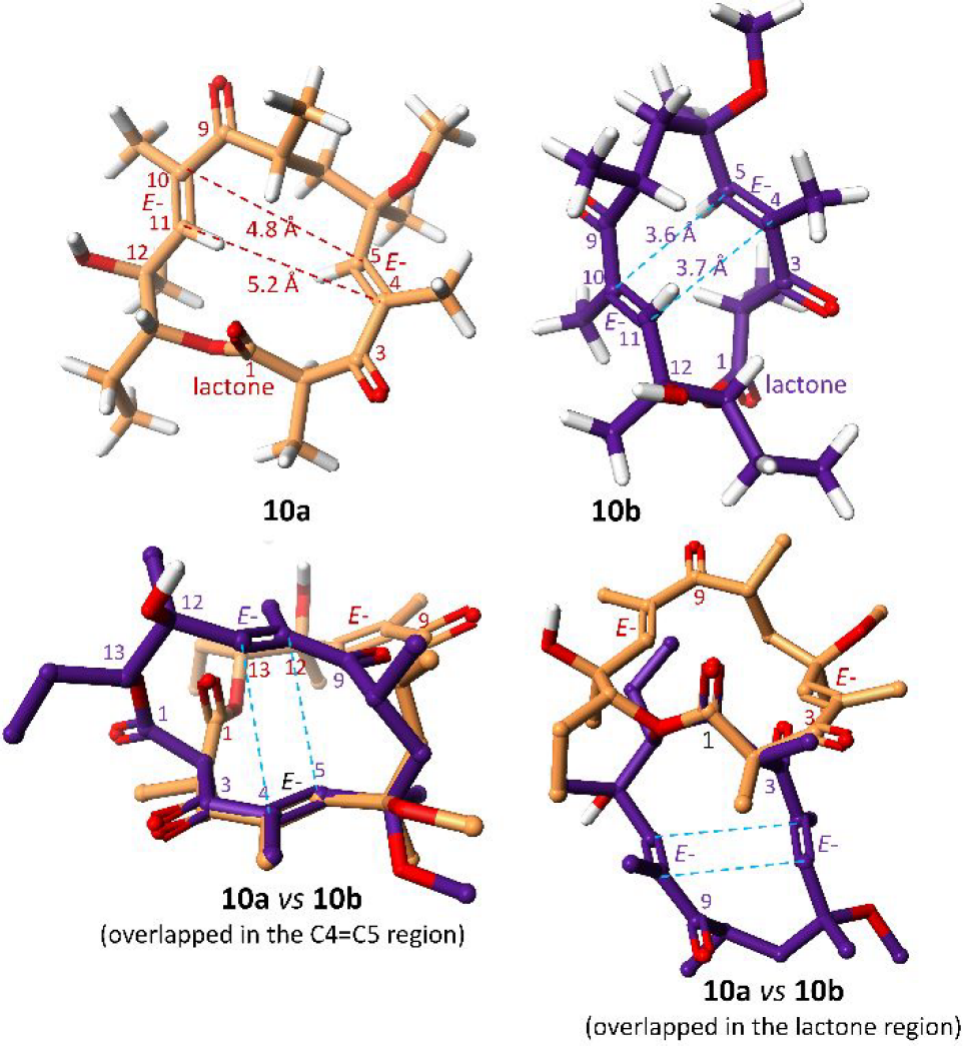
Calculated structures of **10a** (folded-out) and **10b** (folded-in) on the basis of assumed contacts found in the NOESY spectrum using an XC functional: BLYP-D3 with basis set TZ2P (ADF package; see the Supporting Information).

In conclusion, we have developed a new synthetic approach leading to modifications of the 14-membered lactone aglycone of known antibiotics. Our approach enables inversion of the configuration at C(12) and formation of doubly α,β-unsaturated ketone aglycones of 14-membered macrolide antibiotics. Further studies on the utility of these transformations for structural diversification of known macrolide antibiotic scaffolds are currently underway.

## Supporting Information Available

Experimental procedures, copy of all ^1^H and ^13^C NMR spectra with 2D NMR experiments and signal assignments collected in Tables S1 and S2, FT-IR and ATR spectra, details of DFT calculations with Δ*G*°s, *xyz* coordinates and calculated spectra, and X-ray structural details of 2′-acetylated **2**, **5**, and **8** (PDF)
Parallel execution process information for compounds **9** and **10** (PDF)

## Accession Codes

CCDC 2111470, 2111471, and 2111770 contain the supplementary crystallographic data for this paper. These data can be obtained free of charge via www.ccdc.cam.ac.uk/data_request/cif, or by emailing data_request@ccdc.cam.ac.uk, or by contacting The Cambridge Crystallographic Data Centre, 12 Union Road, Cambridge CB2 1EZ, UK; fax: +44 1223 336033.
