# Supplementary material for: An Approach to Modify 14-Membered Lactone Macrolide Antibiotic Scaffolds
Source: J Org Chem. 2022 Jan 12;87(5):3758–61. doi: 10.1021/acs.joc.1c02799 (PMC8902751; doi:10.1021/acs.joc.1c02799)

Supporting Information

An approach to modify 14-membered lactone macrolide antibiotic scaffolds

Anna Janas, Krystian Pyta, Maria Gdaniec, Piotr Przybylski*

Faculty of Chemistry, A. Mickiewicz University, Uniwersytetu Poznańskiego 8, 61-614 Poznań, Poland

*E-mail: piotrp@amu.edu.pl, tel. +48 61 8291693

Table of contents:

Scheme S1. *Mechanism proposal of the transformation of* ***7*** *into* ***9*** *in acidic conditions.* S3

Figure S1. *The molecule of* ***5*** *with the atom numbering scheme. Displacement ellipsoids were drawn at the 50% probability level.* S4

Figure S2. *The asymmetric unit in the crystal structure of 2'-acetylated* ***2***. *Only O and N atoms are labeled. Hydrogen atoms are omitted for clarity. Displacement ellipsoids were drawn at the 50% probability level.* S4

Figure S3. *NOESY contacts observed for protons: H(21) and H(2) for compound* ***9***. S5

Figure S4. *Comparison of FT-IR spectra of* ***5*** *(pink line),* ***9*** *(blue line),* ***10*** *(red line) measured in* $CDCl_3$ *solution in the range 400 – 4000* cm^{-1}. S5

Figure S5. *Comparison of FT-IR spectra of* ***5*** *(pink line),* ***9*** *(blue line),* ***10*** *(red line) measured in* $CDCl_3$ *solution in the range 1150 – 2000* cm^{-1}. S6

Figure S6. *NOESY contacts for* ***10a*** *(ΔG° = -7734.42 kcal/mol) and* ***10b*** *(ΔG° = -7724.69 kcal/mol), which allowed to the determine structures of these conformational isomers, calculated and visualized by ADF-Amsterdam Density Theory /package ADF ver. 2017.114, with recent revision r67691/, XC functional GGA:BLYP D3 with basis set TZ2P.[1–4]* S7

Figure S7. *HPLC separations, with the standard method with* C_{18} *column on the reversed phases, for* ***7***, ***8***, ***9*** *and* ***10*** *(see Experimental). Additional separations for* ***9*** *with the use of* C_8, *150×4.6 mm (3.5 µm, carbon load 13%, pH 1 to 12) were performed. For* ***10*** *an additional separation with the use of chiral column (Shodex ORpak CDBS – 453, 4.6 mm x 150 mm, β-cyclodextrin) was performed.* S8

Figure S8 *HPLC chromatogram for* ***10***. *Separation with the use of C8, 150×4.6 mm (3.5 µm, carbon load 13%, pH 1 to 12) column at 25 ºC was used. The flow rates was 0.5 mL/min with injection volumes of 5 µL. Mixtures of* H_2O/CH_3CN*/buffer in 15 min gradient from 85/5/10 to 30/60/10, next the process was continuing for 5 min. Buffer: 10 mmol ammonium acetate and 133 mmol* NH_3 *in 1L of* H_2O. *The analytical wavelengths was* λ_{max} *= 220 and 241 nm.* S9

Figure S9. *Comparison between DFT calculated structures of C2-epimers of a)* ***10a*** *and b)* ***10b****; the crossed-out* ^1H-^1H *NOESY contacts on C(2S) epimers are visible in experimental spectra of C(2R)-epimers.* S10

1. Experimental section **S11**

1.1. Syntheses and characterization of novel clarithromycin derivatives S11

1.2. Details of HPLC, 1D and 2D NMR and FT-IR characterization of 1–10. ....S15

1.3. Single molecule DFT calculations [XC functional: BLYP-D3; basis set: TZ2P] by ADF package. S17

Figure S10. *Calculated structure of* ***9*** *(ΔG° = -11891.49 kcal/mol; XYZ coordinates) and IR spectrum (no imaginary frequencies detected in the range 4000-400* cm^{-1}*; 1 imaginary frequency was detected at* ν *= -16* cm^{-1}*; Intensity = -0.56 km/mole → rescanned* ν *= 36* cm^{-1}*; Intensity = 0.54 km/mole).* S17

Figure S11. *Calculated structure of* ***10a*** *of C(2R) configuration (ΔG° = -7734.42 kcal/mol; XYZ coordinates) and IR spectrum (no imaginary frequencies detected in the range 4000-400* cm^{-1}*).* S20

Figure S12. *Calculated structure of C(2S)-epimer of **10a** (ΔG° = -7733.59 kcal/mol; XYZ coordinates) and IR spectrum (no imaginary frequencies detected in the range 4000-400 cm^{-1}).* *S22*
Figure S13. *Calculated structure of **10b** of C(2R) configuration (ΔG° = -7724.69 kcal/mol; XYZ coordinates) and IR spectrum (no imaginary frequencies detected in the range 4000-400 cm^{-1}).* *S24*
Figure S14. *Calculated structure of C(2S)-epimer of **10b** (ΔG° = -7732.41 kcal/mol; XYZ coordinates) and IR spectrum (no imaginary frequencies detected in the range 4000-400 cm^{-1}; 1 imaginary frequency was detected at ν = -52 cm^{-1}; Intensity = -1.27 km/mole → rescanned ν = 53 cm^{-1}; Intensity = 1.26 km/mole).* *S26*
1.4 X-Ray crystallography S28
Figure S15. *Superposition of molecules A and B of 2'-acetylated **2** (r.m.s. = 0.16 Å)* *S29*
Figure S16. *Comparison of the conformation of molecule B in 2'-acetylated **2** (a) with the conformation of clarithromycin molecule in its acetonitrile solvate (b) (refcode: CIWJIC)*[6] *S29*
Figure S17. *Two symmetry independent chains via O-H···O hydrogen bonds extended along [010] in 2'-acetylated **2**. The OH groups of the cladinose part are bonded to acetonitrile molecules via O-H···N interaction.* *S30*
Figure S18. *(a) Crystal packing in **8** and (b) hydrogen-bonded chains along [100]* *S31*
Figure S19. *(a) Crystal packing in **5** and (b) hydrogen-bonded chains along [010]* *S32*
Figure S20. *Overlay of the molecules of **8** (blue) and **5** (magenta). The superimposed fragment is labelled (r.m.s.=0.175 Å). O and N atoms are represented as spheres, hydrogen atoms are omitted for clarity.* *S32*

2. References **S32**

3. ^{1}H and ^{13}C{^{1}H} NMR data **S34**

Table S1. ^{1}H NMR data of **2** – **10**, assigned on the basis of ^{1}H-^{13}C HSQC, ^{1}H-^{13}C HMBC, ^{1}H-^{1}H COSY and ^{1}H-^{1}H NOESY spectra. The coupling constants (*J*) are expressed in Hz. S34
Table S2. ^{13}C{^{1}H} NMR data of all synthesized new clarithromycins **2** – **10**, assigned on the basis of ^{1}H-^{13}C HSQC, ^{1}H-^{13}C HMBC, ^{1}H-^{1}H COSY and ^{1}H-^{1}H NOESY spectra. S42

4. Copy of ^{1}H-NMR, ^{13}C{^{1}H}-NMR and FT-IR spectra of 2 – 10 and ^{1}H-^{1}H COSY, ^{1}H-^{13}C HSQC, ^{1}H-^{13}C HMBC for 8-10 and ^{1}H-^{1}H NOESY for 9 and 10. .... **S44**

Table S3. Contacts observed in the ^{1}H-^{1}H NOESY spectra for **9**. Contacts for vicinal and geminal protons were omitted. S71
Table S4. Contacts observed in the ^{1}H-^{1}H NOESY spectra for **10a**. Contacts for vicinal and geminal protons were omitted. S77
Table S5. Contacts observed in the ^{1}H-^{1}H NOESY spectra for **10b**. Contacts for vicinal and geminal protons were omitted. S78

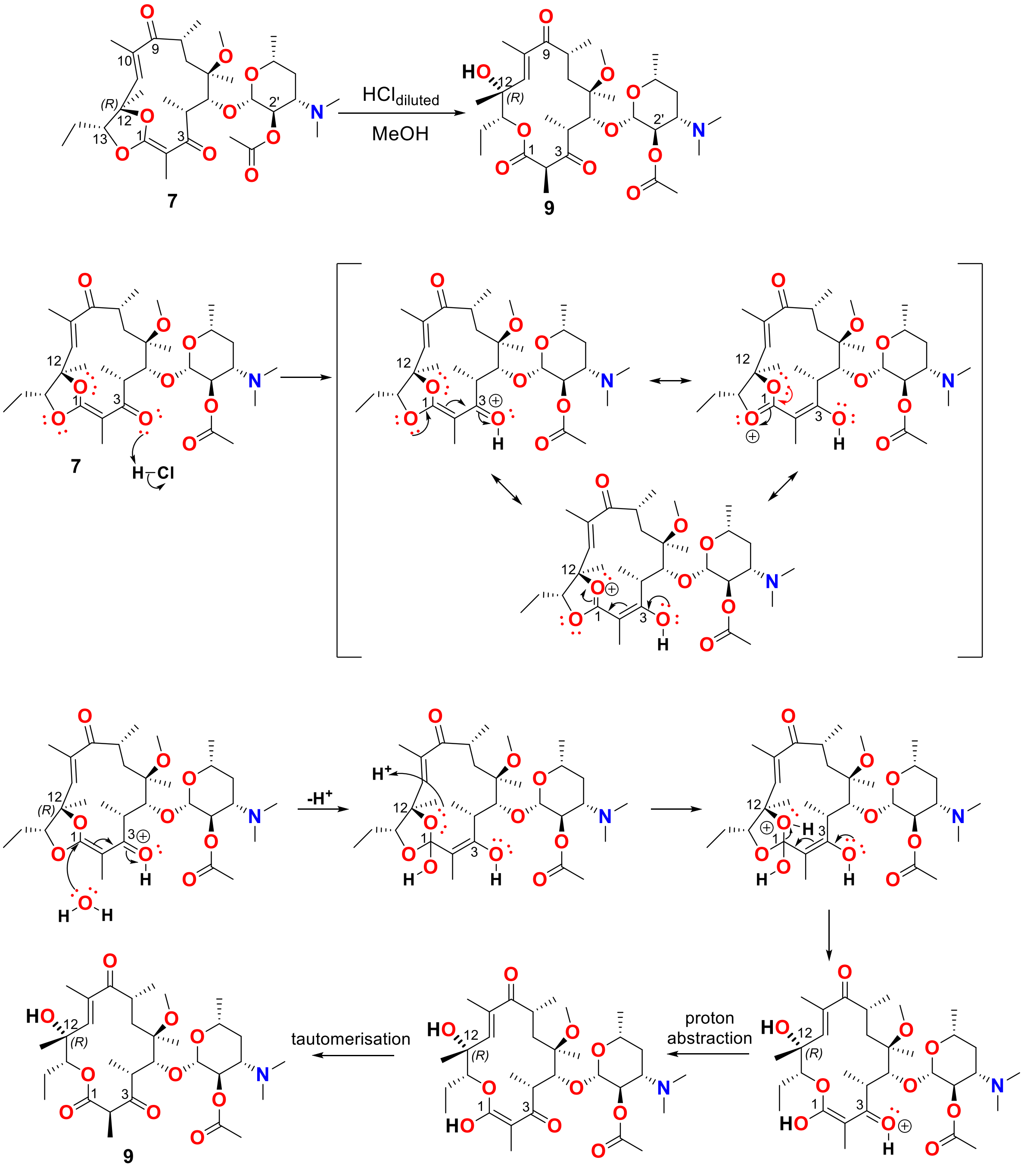

Scheme S1 Mechanism proposal of the transformation of **7** into **9** in acidic conditions.

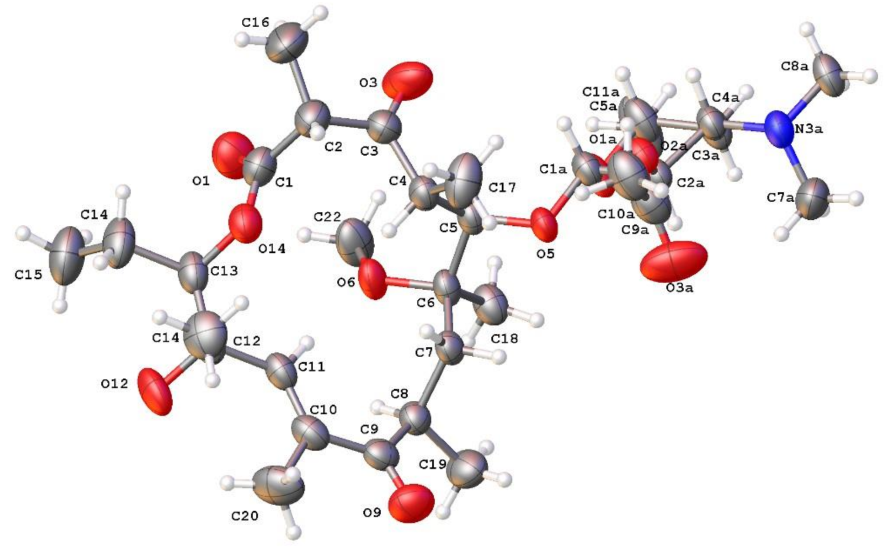

Figure S1. The molecule of **5** with the atom numbering scheme. Displacement ellipsoids were drawn at the 50% probability level.

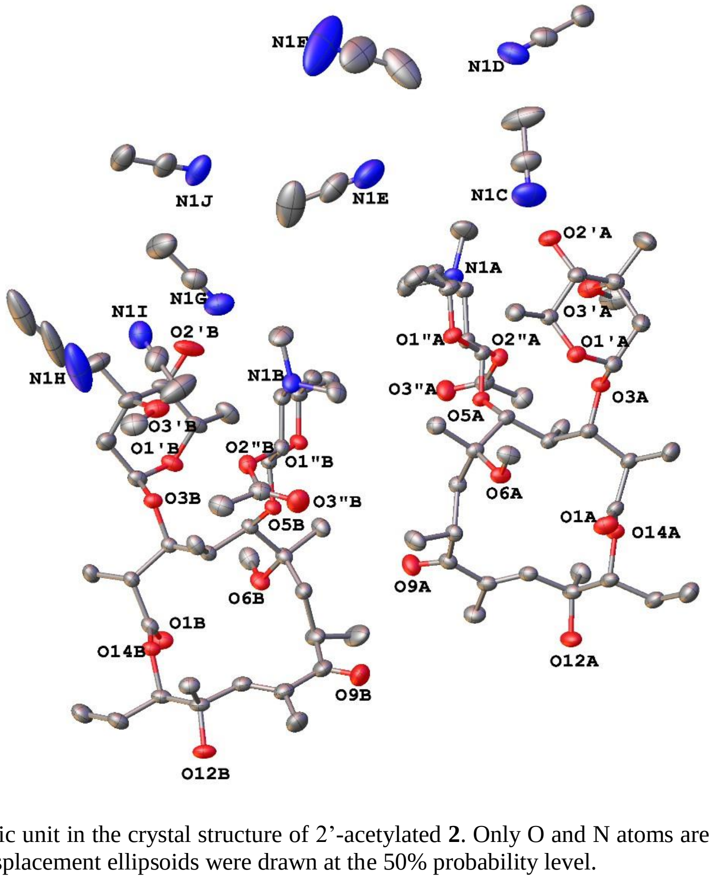

Figure S2. The asymmetric unit in the crystal structure of 2’-acetylated **2**. Only O and N atoms are labeled. Hydrogen atoms are omitted for clarity. Displacement ellipsoids were drawn at the 50% probability level.

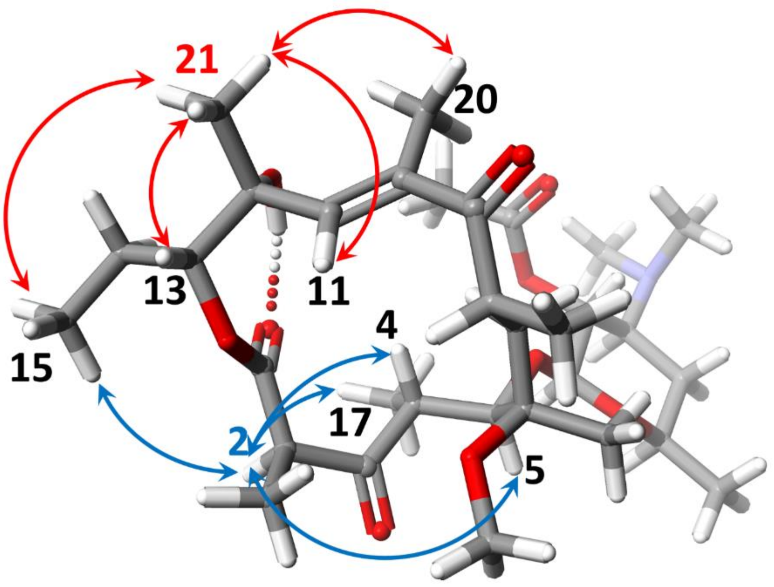

Figure S3. NOESY contacts observed for protons: H(21) and H(2) for compound **9**.

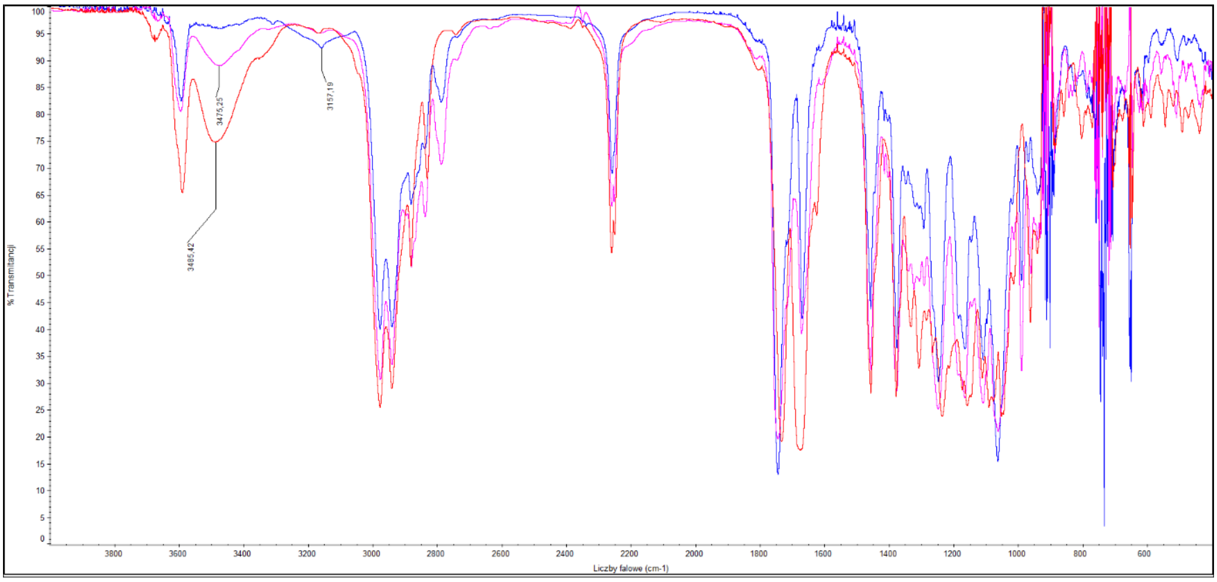

Figure S4. Comparison of FT-IR spectra of **5** (pink line), **9** (blue line), **10** (red line**)** measured in $CDCl_3$ solution in the range 400 – 4000 cm^{-1}.

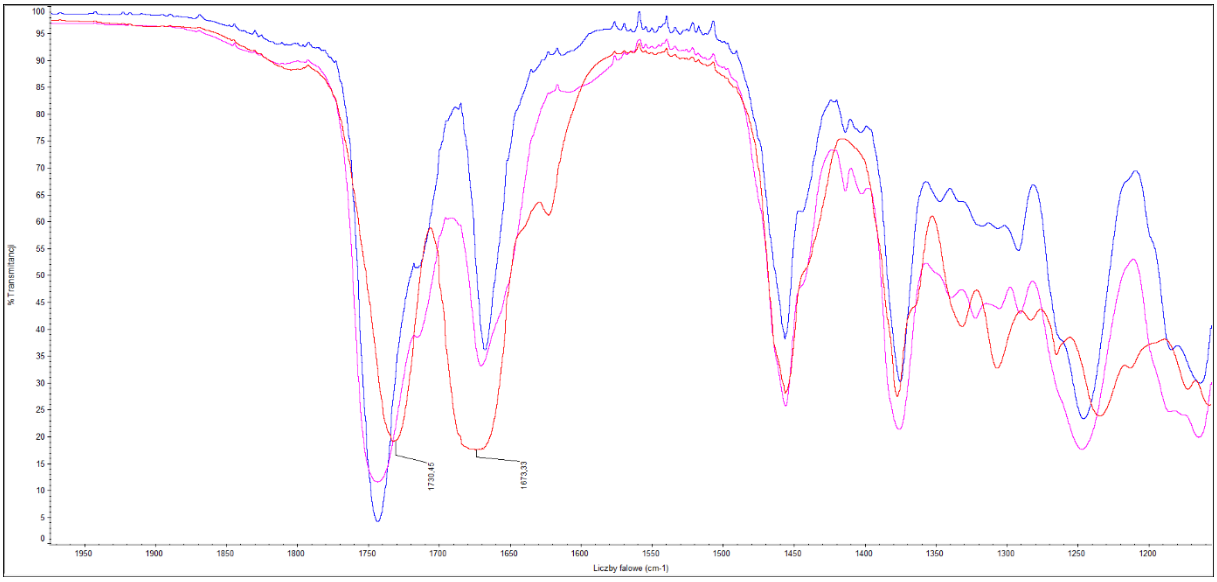

Figure S5. Comparison of FT-IR spectra of **5** (pink line), **9** (blue line), **10** (red line**)** measured in $CDCl_3$ solution in the range 1150 – 2000 cm^{-1}.

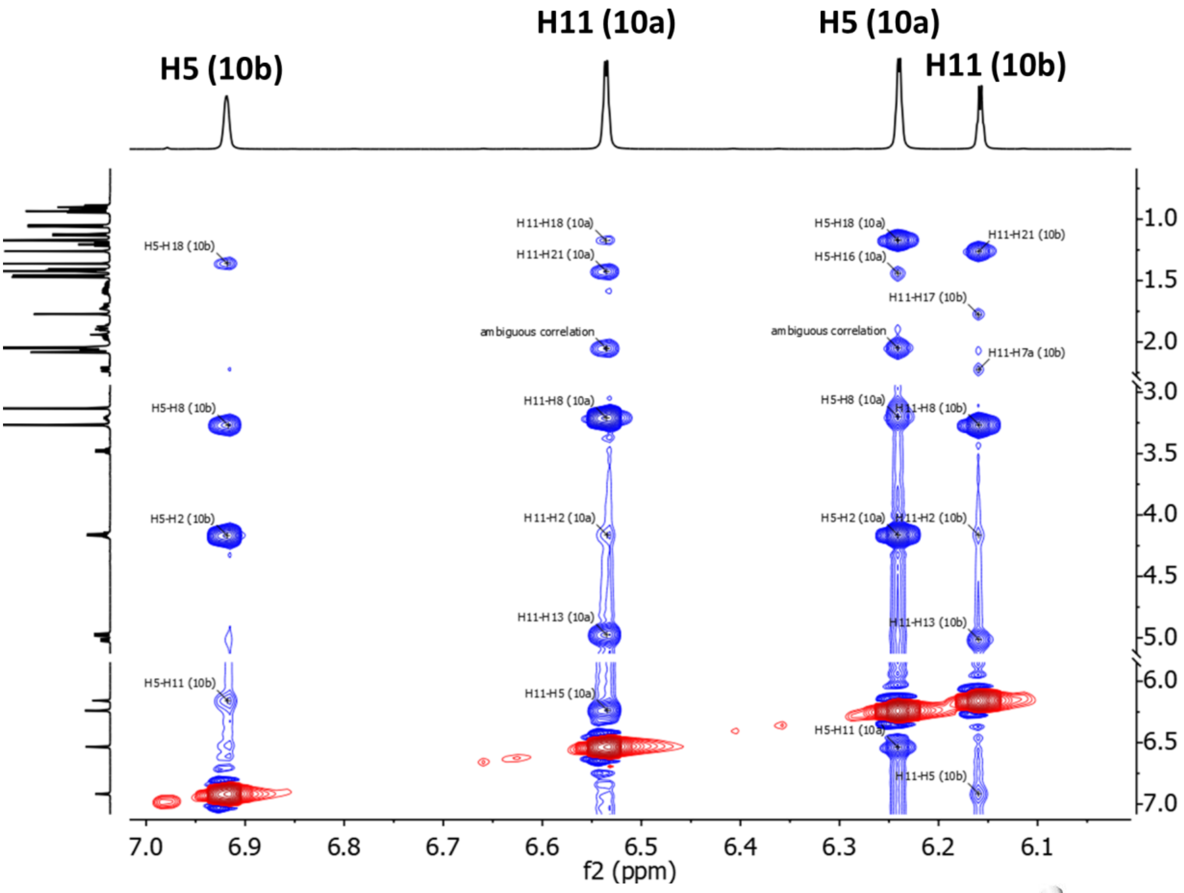

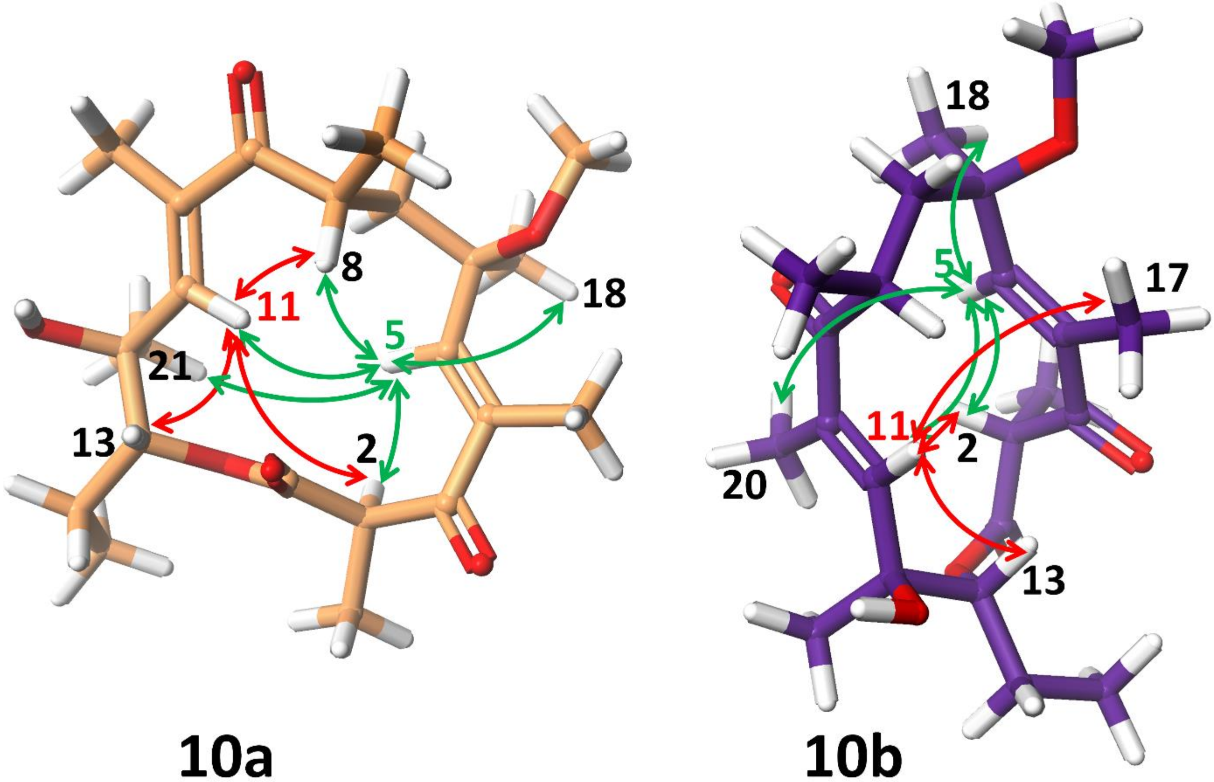

10a **10b**

Figure S6. NOESY contacts for **10a** (ΔG° = -7734.42 kcal/mol) and **10b** (ΔG° = -7724.69 kcal/mol), which allowed to the determine structures of these conformational isomers, calculated and visualized by ADF-Amsterdam Density Theory /package ADF *ver*. 2017.114, with recent revision r67691/, XC functional GGA:BLYP D3 with basis set TZ2P.[1–4]

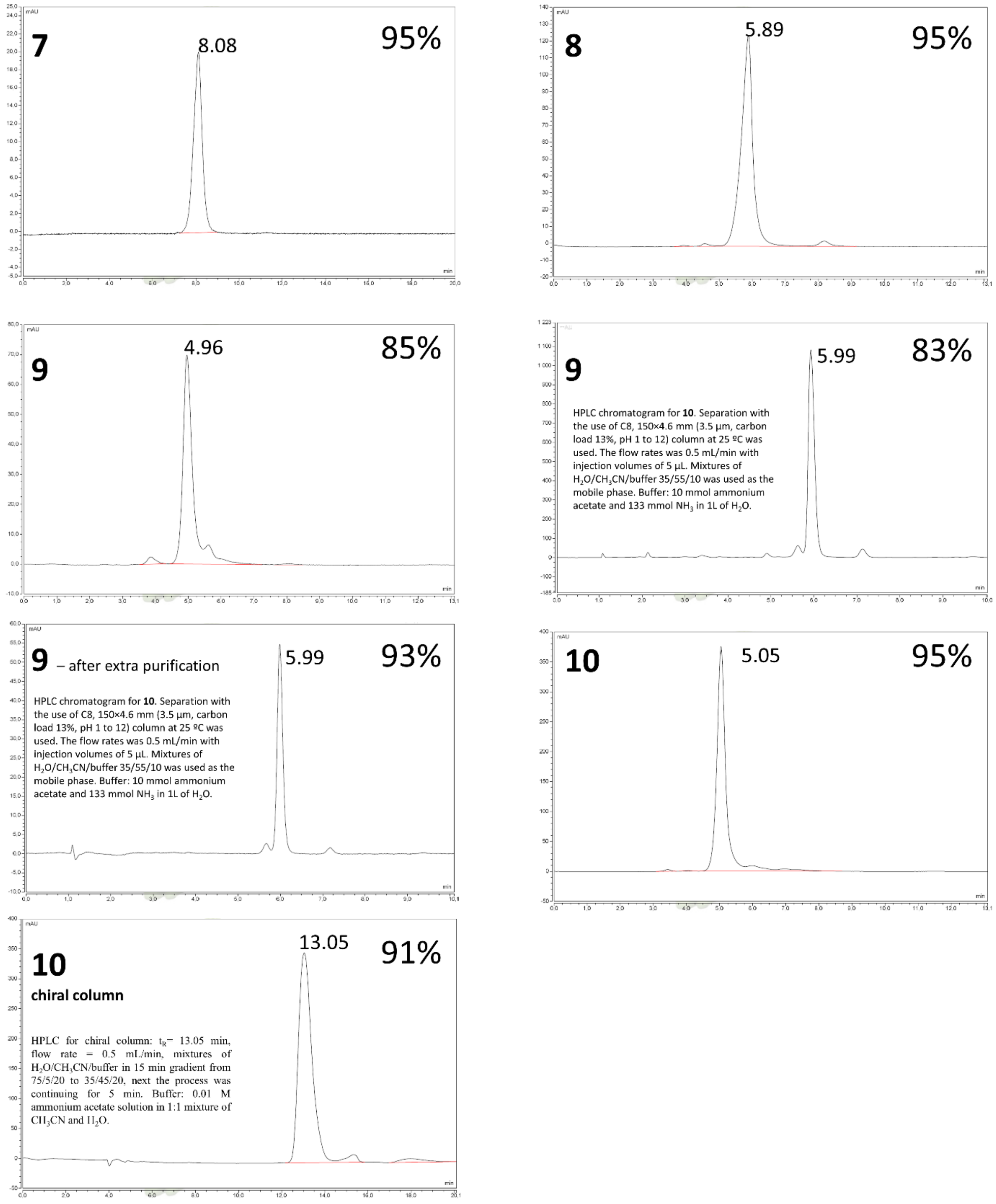

Figure S7. HPLC separations, with the standard method with C_{18} column on the reversed phases, for **7**, **8**, **9** and **10** (see Experimental). Additional separations for **9** with the use of C_8, 150×4.6 mm (3.5 µm, carbon load 13%, pH 1 to 12) were performed. For **10** an additional separation with the use of chiral column (Shodex ORpak CDBS – 453, 4.6 mm x 150 mm, β-cyclodextrin) was performed.

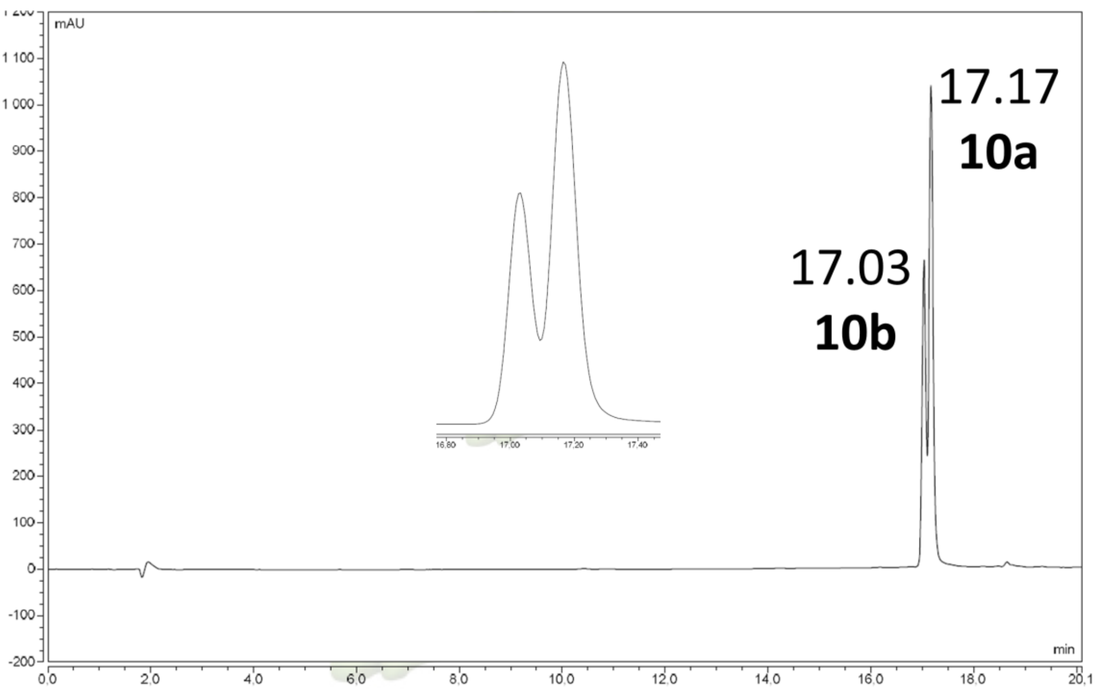

Figure S8 HPLC chromatogram for **10**. Separation with the use of C_8, 150×4.6 mm (3.5 µm, carbon load 13%, pH 1 to 12) column at 25 ºC was used. The flow rates was 0.5 mL/min with injection volumes of 5 µL. Mixtures of H_2O/CH_3CN/buffer in 15 min gradient from 85/5/10 to 30/60/10, next the process was continuing for 5 min. Buffer: 10 mmol ammonium acetate and 133 mmol NH_3 in 1L of H_2O. The analytical wavelengths was λ_{max} = 220 and 241 nm.

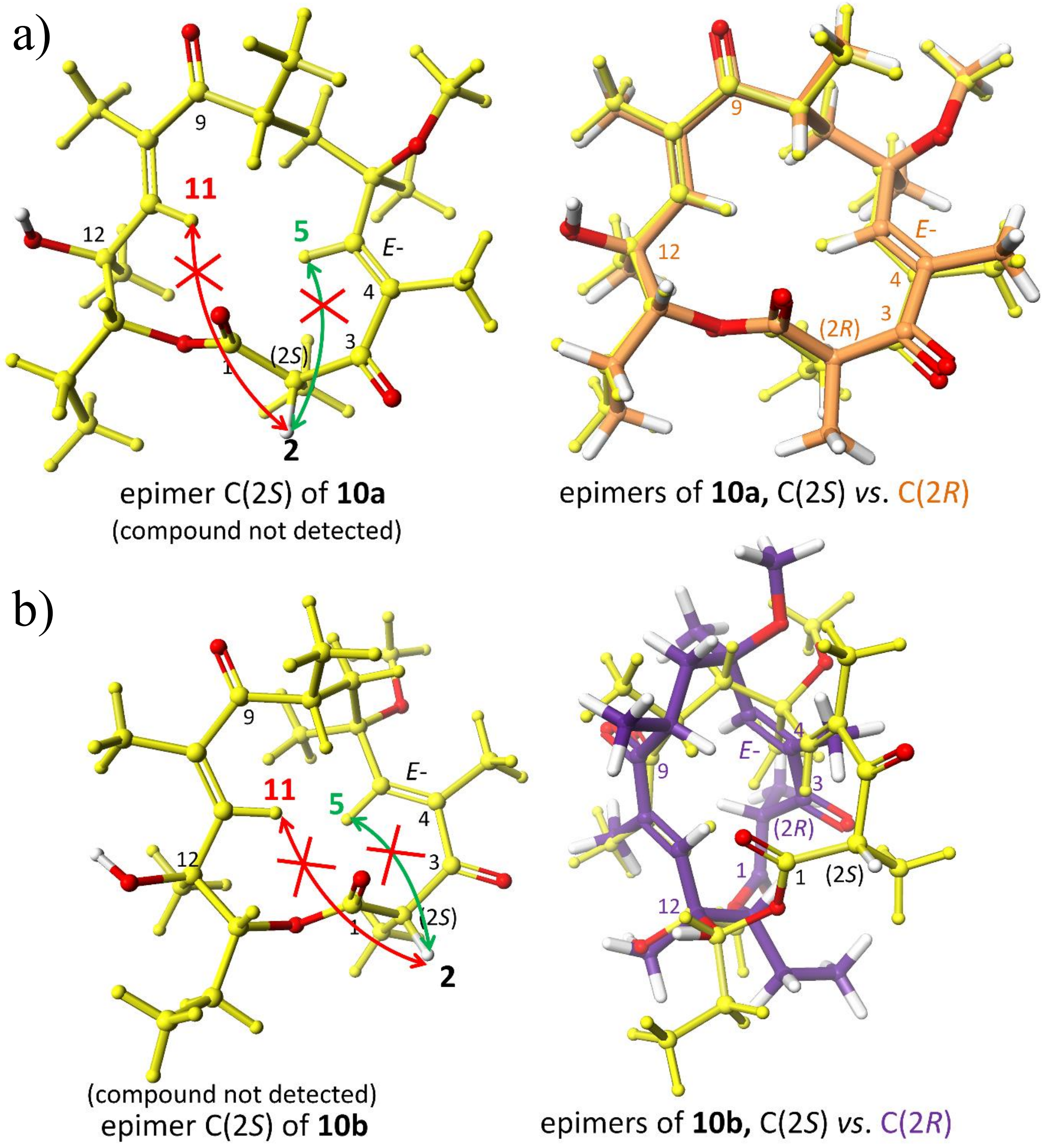

Figure S9. Comparison between DFT calculated structures of C2-epimers of a) **10a** and b) **10b**; the crossed-out ^{1}H-^{1}H NOESY contacts on C(2*S*) epimers are visible in experimental spectra of C(2*R*)-epimers.

1. Experimental section

1.1. Syntheses and characterization of novel clarithromycin derivatives

(3*R*,4*S*,5*S*,6*R*,7*R*,9*R*,13*S*,14*R*,*E*)-6-(((2*S*,3*R*,4*S*,6*R*)-4-(dimethylamino)-3-hydroxy-6-methyltetrahydro-2*H*-pyran-2-yl)oxy)-14-ethyl-13-hydroxy-4-(((2*R*,4*R*,5*S*,6*S*)-5-hydroxy-4-methoxy-4,6-dimethyltetrahydro-2*H*-pyran-2-yl)oxy)-7-methoxy-3,5,7,9,11,13-hexamethyloxacyclotetradec-11-ene-2,10-dione (compound **2**): Clarithromycin (**1**) was purchased from Prospecta (>98%). **1** (250.0 mg, 0.33 mmol) was dissolved in 10 mL TEA and anhydrous ethylene carbonate (133 µL, 2 mmol) was added. The mixture was refluxed at 110 °C (an oil bath) and stired for 12 hours. After that, the solvent was evaporated to dryness and the residue was dissolved in 50 mL of EtOAc and extracted five times with 50 mL of saturated $NaHCO_3$. The separated organic layer was evaporated. Then, the obtained solid state product was dissolved in 3 mL CH_2Cl_2 and the obtained solution was added dropwise into the 50 mL hexane. Obtained precipitate was filtered and dried under the vacuum (231 mg, 95%). HRMS (MALDI-TOF) m/z: $[M + H]^+$ Calcd for $C_{38}H_{68}NO_{12}^+$ 730.4737; Found 730.4726; FT-IR (ATR): a broad band 3446 cm^{-1} $\nu(O_{12}\text{-}H)+\nu(O_{2'}\text{-}H)+\nu(O_{4''}\text{-}H)$, 1733 cm^{-1} $\nu(C_1{=}O)_{lactone}$, 1668 cm^{-1} $\nu(C_9{=}O)$, 1258 cm^{-1} ν(C-O), 1165 cm^{-1} ν(C-O), 1051 cm^{-1} ν(C-O), 1011 cm^{-1} ν(C-O); Anal. Calcd for $C_{38}H_{67}NO_{12}$: C, 62.53; H, 9.35; N, 1.92. Found: C, 62.54, H, 9.36; N, 1.90. HPLC: t_R= 14.17 min (H_2O:CH_3CN:0.1% NH_4OH 10:65:25); ^1H and $^{13}C\{^1H\}$ NMR (Tables S1 and S2).

^1H NMR (500 MHz, $CDCl_3$, 25°C) δ 6.62 (s, 1H), 5.00 (dd, *J*= 10.5, 2.6 Hz, 1H), 4.85 (d, *J*= 4.8 Hz, 1H), 4.35 (d, *J*= 7.2 Hz, 1H), 4.02 (dd, *J* = 9.3, 6.2 Hz, 1H), 3.97 (d, *J*= 8.6 Hz, 1H), 3.66 (d, *J*= 7.7 Hz, 1H), 3.46 (dqd, *J* = 10.2, 6.0, 4.8 Hz, 1H), 3.30 (m, 1H), 3.30 (s, 3H), 3.22 (s, 3H), 3.18 (dd, *J*= 10.2, 7.2 Hz, 1H), 3.00 (d, *J*= 9.2 Hz, 1H), 2.87 (m, 1H), 2.42 (dd, *J* = 10.3, 7.2 Hz, 1H), 2.36 (d, *J*= 15.2 Hz, 1H), 2.26 (s, 6H,), 2.04 (dd, *J*= 14.9, 4.3 Hz, 1H), 2.00 (s, 3H), 1.92 (m, 1H), 1.92 (m, 1H), 1.63 (m, 1H), 1.57 (dd, *J* = 15.2, 4.9 Hz, 1H), 1.50 (ddt, *J* = 14.2, 7.3, 2.9 Hz, 1H), 1.40 (s, 3H), 1.39 (m, 1H), 1.39 (s, 3H), 1.28 (d, *J* = 6.2 Hz, 3H), 1.24 (d, *J* = 6.9 Hz, 3H), 1.23 (s, 3H), 1.21 (d, *J*= 6.1 Hz, 3H), 1.18 (m, 1H), 1.14 (d, *J*= 6.7 Hz, 3H), 1.09 (d, *J* = 7.4 Hz, 3H), 0.90 (d, *J* = 7.3 Hz, 1H).

$^{13}C\{^1H\}$ NMR (126 MHz, $CDCl_3$, 25°C) δ 207.5, 175.3, 142.5, 138.9, 103.2, 96.5, 80.3, 80.0, 79.3, 78.7, 78.1, 73.5, 72.7, 71.0, 69.0, 65.8, 65.6, 50.8, 45.2, 49.6, 40.5, 40.5, 40.4, 37.5, 35.2, 28.8, 22.4, 22.3, 21.7, 21.6, 21.0, 18.8, 18.7, 15.7, 13.4, 10.8, 9.7.

(3*R*,4*S*,5*S*,6*R*,7*R*,9*R*,13*S*,14*R*,*E*)-6-(((2*S*,3*R*,4*S*,6*R*)-4-(dimethylamino)-3-hydroxy-6-methyltetrahydro-2*H*-pyran-2-yl)oxy)-14-ethyl-4,13-dihydroxy-7-methoxy-3,5,7,9,11,13-hexamethyloxacyclotetradec-11-ene-2,10-dione (compound **3**): **2** (230 mg, 0.31 mmol) was dissolved in 5 mL acetone and 0.25M HCl was added. The mixture was stired for 18 hours at room temperature and after that 10 mL saturated $NaHCO_3$ was added to stop the reactions. Organic layer was extracted three times with 50 mL of saturated $NaHCO_3$.The separated organic layer was evaporated and white solid was obtained (175 mg, 97%). HRMS (MALDI-TOF) m/z: $[M + H]^+$ Calcd for $C_{30}H_{54}NO_9^+$ 572.3793; Found 572.3783; FT-IR (ATR): a broad band 3446cm^{-1} $\nu(O_3\text{-}H) + \nu(O_{12}\text{-}H) + \nu(O_{2'}\text{-}H)$; 1729 cm^{-1} $\nu(C_1{=}O)_{lactone}$, 1668 cm^{-1} $\nu(C_9{=}O)$, 1257 cm^{-1} ν(C-O), 1163 cm^{-1} ν(C-O), 1070 cm^{-1} ν(C-O), 1047 cm^{-1} ν(C-O); Anal. Calcd for $C_{30}H_{53}NO_9$: C, 63.02; H, 9.34; N, 2.45. Found: C, 63.01; H, 9.33; N, 2.47. HPLC: t_R= 8.32 min; ^1H and $^{13}C\{^1H\}$ NMR (Tables S1 and S2).

^{1}H NMR (500 MHz, $CDCl_3$) δ 6.47 (q, J = 1.5 Hz, 1H), 4.95 (dd, J = 11.0, 2.2 Hz, 1H), 4.59 (bs, 1H), 4.50 (d, J = 7.6 Hz, 1H), 4.02 (d, J = 10.5 Hz, 1H), 3.92 (d, J = 2.0 Hz, 1H), 3.54 (dqd, J = 12.5, 6.2, 2.1 Hz, 1H), 3.24 (dd, J = 10.3, 7.6 Hz, 1H), 3.18 (m, 1H), 3.10 (s, 3H), 2.71 (dq, J = 10.5, 6.8 Hz, 1H), 2.51 (m, 1H), 2.26 (s, 6H), 2.03 (d, J = 1.2 Hz, 3H), 1.95 (dqd, J = 15.2, 7.5, 2.1 Hz, 1H), 1.68 (m, 1H), 1.66 (m, 1H), 1.63 (m, 1H), 1.55 (m, 1H), 1.36 (s, 3H), 1.33 (d, J = 6.8 Hz, 3H), 1.32 (m, 1H), 1.29 (m, 1H), 1.26 (s, 3H), 1.26 (d, J = 6.2 Hz, 3H), 1.19 (d, J = 6.5 Hz, 3H), 1.03 (d, J = 7.0 Hz, 3H), 0.91 (t, J = 7.3 Hz, 3H).
^{13}C{^{1}H} NMR (126 MHz, $CDCl_3$) δ 207.7, 176.7, 141.5, 138.7, 106.8, 92.0, 80.9, 79.2, 76.8, 73.5, 70.5, 69.7, 65.6, 48.2, 44.3, 40.3, 38.3, 36.9, 36.4, 28.3, 21.4, 20.8, 20.4, 20.2, 16.2, 15.8, 12.9, 10.5, 7.6.

(2*S*,3*R*,4*S*,6*R*)-4-(dimethylamino)-2-(((3*R*,4*S*,5*S*,6*R*,7*R*,9*R*,13*S*,14*R*,*E*)-14-ethyl-4,13-dihydroxy-7-methoxy-3,5,7,9,11,13-hexamethyl-2,10-dioxooxacyclotetradec-11-en-6-yl)oxy)-6-methyltetrahydro-2*H*-pyran-3-yl acetate (compound **4**): **3** (170 mg, 0.30 mmol) was dissolved in 5 mL ACN and acetic anhydride (55 µl, 0.60 mmol) was added. The mixture was stirred at 25°C for 24 hours and after that the solvent was evaporated to dryness; the obtained residue was dissolved in 50 mL of EtOAc and extracted twice with 50 mL of saturated $NaHCO_3$. The separated organic layer was evaporated and white solid was obtained (175 mg, 95%). HRMS (MALDI-TOF) m/z: $[M + H]^+$ Calcd for $C_{32}H_{56}NO_{10}^+$ 614.3899; Found 614.3913; FT-IR (ATR): a broad band 3480 cm^{-1} $\nu(O_3\text{-}H)$ + $\nu(O_{12}\text{-}H)$; 1740 cm^{-1} $\nu(C_{9'}{=}O)_{acetyl}$, 1731 cm^{-1} $\nu(C_1{=}O)_{lactone}$, 1668 cm^{-1} $\nu(C_9{=}O)$, 1236 cm^{-1} ν(C-O), 1162 cm^{-1} ν(C-O), 1107 cm^{-1} ν(C-O), 1047 cm^{-1} ν(C-O); Anal. Calcd for $C_{32}H_{55}NO_{10}$: C, 62.62; H, 9.03; N, 2.28. Found: C, 62.63; H, 9.04; N, 2.30. HPLC: t_R= 6.67 min; ^{1}H and ^{13}C{^{1}H} NMR (Tables S1 and S2).
^{1}H NMR (600 MHz, $CDCl_3$) δ 6.42 (q, J = 1.5 Hz, 1H), 5.02 (dd, J = 10.8, 2.3 Hz, 1H), 4.72 (m, 2H), 3.88 (d, J = 3.0 Hz, 1H), 3.79 (d, J = 10.4 Hz, 1H), 3.51 (dqd, J = 11.0, 6.2, 2.1 Hz, 1H), 3.16 (m, 1H), 3.10 (s, 3H), 2.65 (m, 2H), 2.24 (s, 6H), 2.11 (s, 3H), 2.04 (d, J = 1.3 Hz, 3H), 1.95 (m, 1H), 1.82 (qd, J = 7.2, 3.1 Hz, 1H), 1.73 (ddd, J = 12.9, 4.4, 2.0 Hz, 1H), 1.60 (dd, J = 15.3, 10.0 Hz, 1H), 1.56 (m, 1H), 1.48 (dd, J = 15.5, 4.7 Hz, 1H), 1.39 (s, 3H), 1.36 (m, 1H), 1.31 (d, J = 6.8 Hz, 3H), 1.29 (s, 3H), 1.26 (d, J = 6.2 Hz, 3H), 1.18 (d, J = 6.7 Hz, 3H), 0.92 (d, J = 7.2 Hz, 3H), 0.91 (t, J = 7.4 Hz, 3H).
^{13}C{^{1}H} NMR (151 MHz, $CDCl_3$) δ 207.6, 175.8, 170.2, 140.8, 139.6, 102.6, 87.8, 80.0, 79.4, 77.2, 73.4, 71.9, 69.2, 64.1, 48.9, 44.3, 40.7, 37.7, 37.5, 37.2, 30.2, 21.4, 21.3, 21.2, 20.8, 20.3, 17.1, 15.5, 13.5, 10.6, 8.0.

(2*S*,3*R*,4*S*,6*R*)-4-(dimethylamino)-2-(((3*R*,5*R*,6*R*,7*R*,9*R*,13*S*,14*R*,*E*)-14-ethyl-13-hydroxy-7-methoxy-3,5,7,9,11,13-hexamethyl-2,4,10-trioxooxacyclotetradec-11-en-6-yl)oxy)-6-methyltetrahydro-2*H*-pyran-3-yl acetate (compound **5**): *N*-Chlorosuccinimide (NCS) (60 mg, 0.45 mmol) was dissolved in 3 mL CH_2Cl_2 and cooled for 5 minutes in an ice bath at -20 °C. After that, dimethyl sulfide (45 µl, 0.61 mmol) was added drop by drop and stirred for 15 minutes in an ice bath at -20 °C. Then, dissolved earlier in 3 mL CH_2Cl_2 **4** (170 mg, 0.28 mmol) was added dropwise. The mixture was stirred at -15 – -20°C for 35 minutes. Afterward TEA (45 µl, 0.60 mmol) was added and the reaction mixture was stirred to reach a temperature of +15 °C (approx. 4.5 hours). After that the solvent was evaporated and residue was dissolved in 50 mL of diethyl ether and extracted five times with 50 mL of water. The separated organic layer was evaporated and the synthesized derivative **5** was purified using column chromatography and silica gel with dichloromethane/acetone (4:1) as an eluent. The product was obtained as white solid (73.2

mg, 43%). HRMS (MALDI-TOF) m/z: $[M + H]^+$ Calcd for $C_{32}H_{55}NO_{10}^+$ 612.3742; Found 612.3729; FT-IR (ATR): 1739 cm^{-1} $\nu(C_{9'}=O)_{acethyl}$, 1731 cm^{-1} $\nu(C_1=O)_{lactone}$, 1713 cm^{-1} $\nu(C_3=O)$, 1669 cm^{-1} $\nu(C_9=O)$, 1229 cm^{-1} ν(C-O), 1162 cm^{-1} ν(C-O), 1102 cm^{-1} ν(C-O), 1057 cm^{-1} ν(C-O); Anal. Calcd for $C_{32}H_{53}NO_{10}$: C, 62.83; H, 8.73; N, 2.29. Found: C, 62.85; H, 8.72; N, 2.30. HPLC: t_R= 10.36 min; ^1H and $^{13}C\{^1H\}$ NMR (Tables S1 and S2).

^1H NMR (500 MHz, $CDCl_3$, 25°C) δ 6.49 (s, 1H), 4.98 (dd, *J*= 9.8, 2.9 Hz, 1H), 4.71 (dd, *J*= 10.5, 7.6 Hz, 1H), 4.34 (d, *J* = 7.6 Hz, 1H), 4.12 (d, *J* = 8.2 Hz, 1H), 3.72 (q, *J* = 6.9 Hz, 1H), 3.52 (m, 1H), 3.15 (m, 1H), 3.04 (p, *J* = 7.5 Hz, 1H), 2.85 (s, 3H), 2.64 (m, 1H), 2.23 (s, 6H), 2.03 (s, 3H), 2.01 (s, 3H), 1.95 (ddd, *J* = 14.4, 7.5, 2.9 Hz, 1H), 1.82 (dd, *J* = 14.4, 6.9 Hz, 1H), 1.71(m, 1H), 1.55 (ddd, *J* = 14.3, 9.9, 7.3 Hz, 1H), 1.51 (m, 1H), 1.46 (s, 3H), 1.34 (d, *J*= 6.9 Hz, 3H), 1.29 (m, 1H), 1.28 (s, 3H), 1.23 (d, *J*= 6.1 Hz, 3H), 1.14 (d, *J* = 6.8 Hz, 3H), 1.11 (d, *J*= 7.4 Hz, 3H), 0.92 (t, *J*= 7.4 Hz, 1H).

$^{13}C\{^1H\}$ NMR (126 MHz, $CDCl_3$, 25°C) δ 207.2, 204.6, 169.9, 169.9, 142.1, 139.0, 101.9, 81.5, 81.1, 78.5, 73.8, 71.7, 69.2, 63.7, 51.3, 50.5, 47.3, 40.8, 40.3, 38.6, 30.5, 22.6, 22.1, 21.5, 21.1, 21.1, 19.1, 14.9, 14.1, 13.7, 11.1.

(2*R*,3*S*,7*R*,9*R*,10*R*,11*R*,13*R*,*E*)-10-(((2*S*,3*R*,4*S*,6*R*)-3-acetoxy-4-(dimethylamino)-6-methyltetrahydro-2*H*-pyran-2-yl)oxy)-2-ethyl-9-methoxy-3,5,7,9,11,13-hexamethyl-6,12,14-trioxooxacyclotetradec-4-en-3-yl 1*H*-imidazole-1-carboxylate (compound **6**): **5** (70 mg, 0.11 mmol) was dissolved in 4 mL mixture of THF:DMF in 3:1 ratio and 1,1'-Carbonyldiimidazole (CDI) (46.5 mg, 0.28 mmol) and sodium hydride (7.23 mg, 29 mmol) were added. The mixture was stirred at 25°C for 1h and after that 1 mL of water was added and the mixture was diluted in 30 mL of diethyl ether and extracted three times with 30 mL of water. The separated organic layer was evaporated and white solid was obtained (79 mg, 97%). HRMS (MALDI-TOF) m/z: $[M + H]^+$ Calcd for $C_{36}H_{56}N_3O_{11}^+$ 706.3909; Found 706.3891; FT-IR (ATR): a broad band 3475 cm^{-1} $\nu(O_{12}$-H); 1745 cm^{-1} $\nu(C_{9'}=O)_{acetyl} + \nu(C_{23}=O) + \nu(C_1=O)_{lactone}$, 1713 cm^{-1} $\nu(C_3=O)$, 1673 cm^{-1} $\nu(C_9=O)$, 1238 cm^{-1} ν(C-O), 1159 cm^{-1} ν(C-O), 1093 cm^{-1} ν(C-O), 1056 cm^{-1} ν(C-O); Anal. Calcd for $C_{36}H_{55}NO_{11}$: C, 61.26; H, 7.85; N, 5.95. Found: C, 61.27; H, 7.86; N, 5.96. HPLC: t_R= 11.67 min; ^1H and $^{13}C\{^1H\}$ NMR (Tables S1 and S2).

^1H NMR (500 MHz, $CDCl_3$, 25°C) δ 8.08 (d, *J*= 1.1 Hz, 1H), 7.36 (t, *J* = 1.5 Hz, 1H), 7.06 (d, *J* = 1.7 Hz, 1H), 6.79 (s, 1H), 5.68 (dd, *J*= 9.9, 2.9 Hz, 1H), 4.71 (dd, *J* = 10.5, 7.6 Hz, 1H), 4.34 (d, *J*= 7.6 Hz, 1H), 4.11 (d, *J*= 8.6 Hz 1H), 3.73 (m, 1H), 3.48 (m, 1H), 3.15 (m, 1H), 3.01 (m, 1H), 2.77 (s, 3H), 2.63 (m, 1H), 2.22 (s, 6H), 2.03 (s, 3H), 1.85 (s, 3H), 1.84 (m, 1H), 1.82 (s, 3H), 1.79 (m, 1H), 1.70 (m, 1H), 1.67 (m, 1H), 1.62 (m, 1H), 1.35 (d, *J* = 6.8 Hz, 3H), 1.31 (m, 1H), 1.29 (s, 3H), 1.23 (d, *J*= 6.4 Hz, 3H), 1.21 (d, *J*= 7.6 Hz, 3H), 1.11 (d, *J* = 7.4 Hz, 3H), 0.94 (t, *J* = 7.5 Hz, 1H).

$^{13}C\{^1H\}$ NMR (126 MHz, $CDCl_3$, 25°C) δ 205.1, 204.1, 169.9, 169.0, 146.1, 138.6, 138.3, 137.2, 131.0, 117.2, 102.1, 84.7, 81.0, 78.7, 77.2, 71.7, 69.3, 63.7, 51.2, 50.4, 47.5, 40.8, 40.4, 39.1, 30.4, 22.8, 21.5, 21.1, 21.1, 20.3, 19.0, 15.2, 14.1, 13.4, 10.6.

(2*S*,3*R*,4*S*,6*R*)-4-(dimethylamino)-2-(((1*R*,2*E*,5*R*,7*R*,8*R*,9*R*,11*Z*,14*R*)-14-ethyl-7-methoxy-1,3,5,7,9,11-hexamethyl-4,10-dioxo-13,15-dioxabicyclo[10.2.1]pentadeca-2,11-dien-8-yl)oxy)-6-methyltetrahydro-2*H*-pyran-3-yl acetate (compound **7**): **6** (75 mg, 0.105 mmol) was dissolved in 3 mL DMF and 1,1,3,3-tetramethylguanidine (TMG) (25.1 µl, 0.2 mmol) was added. The mixture was vigorously stirred at 80 °C, heating using an oil bath. After 3h, the mixture was dissolved in 50 mL of EtOAc and extracted three times with 50 mL of water. The separated organic layer was evaporated to dryness and the

synthesized derivative **7** was purified using column chromatography with silica gel and dichloromethane/acetone (6:1) as an eluent. The product was obtained as white solid (47 mg, 72%). HRMS (MALDI-TOF) m/z: $[M + H]^+$ Calcd for $C_{32}H_{52}NO_9^+$ 594.3637; Found 594.3622; FT-IR (ATR): 1811 cm^{-1} combination bands, 1744 cm^{-1} $\nu(C_{9'}{=}O)_{acetyl}$, 1655 cm^{-1} $\nu(C_9{=}O)_{C(1)=C(2)}$, 1652 cm^{-1} $\nu(C{=}C)$, 1604 cm^{-1} $\nu(C_3{=}O)$, 1235 cm^{-1} ν(C-O), 1162 cm^{-1} ν(C-O), 1133 cm^{-1} ν(C-O), 1057 cm^{-1} ν(C-O); Anal. Calcd for $C_{32}H_{53}NO_{10}$: C, 62.83; H, 8.73; N, 2.29. Found: C, 62.82; H, 8.73; N, 2.30. HPLC: t_R= 8.08 min; ^{1}H and ^{13}C{^{1}H} NMR (Tables S1 and S2).
^{1}H NMR (500 MHz, $CDCl_3$, 25ºC) δ 6.33 (s, 1H), 4.70 (dd, *J* = 10.5, 7.6 Hz, 1H), 4.45 (d, *J* = 7.6 Hz, 1H), 4.29 (dd, *J*= 9.2, 4.5 Hz, 1H), 4.04 (d, *J* = 9.4 Hz, 1H), 3.91 (dq, *J* = 9.4, 7.3 Hz, 1H), 3.51 (m, 1H), 3.22 (m, 1H), 2.86 (s, 3H), 2.65 (m, 1H,), 2.23 (s, 6H), 2.14 (m, 1H), 2.04 (s, 3H), 1.93 (s, 3H), 1.75 (s, 3H), 1.74 (s, 3H), 1.70 (m, 1H), 1.68 (m, 1H), 1.64 (m, 1H), 1.32 (s, 3H), 1.29 (m, 1H), 1.24 (m, 1H), 1.20 (d, *J* = 6.1 Hz, 3H), 1.13 (d, *J* = 6.6 Hz, 3H), 1.11 (t, *J* = 7.3, 1H), 1.07 (d, *J* = 7.2 Hz, 3H).
^{13}C{^{1}H} NMR (126 MHz, $CDCl_3$, 25ºC) δ 208.4, 200.8, 170.2, 165.0, 136.9, 136.6, 102.2, 89.2, 87.1, 84.5, 84.1, 78.6, 71.9, 69.0, 63.7, 51.6, 42.8, 40.8, 39.9, 37.9, 30.5, 23.9, 22.6, 21.6, 21.1, 19.9, 17.6, 14.9, 13.6, 11.1, 10.3.

(1*R*,2*E*,5*R*,7*R*,8*R*,9*R*,11*Z*,14*R*)-8-(((2*S*,3*R*,4*S*,6*R*)-4-(dimethylamino)-3-hydroxy-6-methyltetrahydro-2*H*-pyran-2-yl)oxy)-14-ethyl-7-methoxy-1,3,5,7,9,11-hexamethyl-13,15-dioxabicyclo[10.2.1]pentadeca-2,11-diene-4,10-dione (compound **8**): **7** (50 mg, 0.09 mmol) was dissolved in 1 mL MeOH and stirred at RT. After 3h, the precipitate was filtered off, yield 15 mg of white solid (32 %). HRMS (MALDI-TOF) m/z: $[M + H]^+$ Calcd for $C_{30}H_{50}NO_8^+$ 552.7194; Found 552.7181; FT-IR (ATR): a broad band at 3361 cm^{-1} $\nu(O_{2'}\text{-}H)$; 1812 cm^{-1} combination bands, 1674 cm^{-1} $\nu(C_9{=}O)$, 1638 cm^{-1} $\nu(C{=}C)$, 1578 cm^{-1} $\nu(C_3{=}O)$, 1158 cm^{-1} ν(C-O), 1108 cm^{-1} ν(C-O), 1060 cm^{-1} ν(C-O); Anal. Calcd for $C_{30}H_{49}NO_8$: C, 65.31; H, 8.95; N, 2.54. Found: C, 65.34; H, 8.93; N, 2.55. HPLC: t_R= 5.89 min; ^{1}H and ^{13}C{^{1}H} NMR (Tables S1 and S2). ^{1}H NMR (600 MHz, $CDCl_3$, 25ºC) δ 6.40 (s, 1H), 4.41 (d, *J*= 7.3 Hz, 1H), 4.28 (dd, *J*= 9.4, 4.4 Hz, 1H), 4.14 (d, *J* = 9.9 Hz, 1H), 4.01 (dq, *J*= 9.9, 6.9 Hz, 1H), 3.54 (dqd, *J*= 12.3, 6.1, 2.1 Hz, 1H), 3.34 (m, 1H), 3.15 (dd, *J* = 10.2, 7.3 Hz, 1H), 2.92 (s, 3H), 2.48 (ddd, *J*=12.3, 10.1, 4.0 Hz, 1H), 2.32 (dd, *J*= 14.9, 2,9 Hz, 1H), 2.26 (s, 6H), 1.91 (s, 3H), 1.77 (s, 3H), 1.76 (s, 3H), 1.67 (m, 2H), 1.65 (m, 1H), 1.36 (s, 3H), 1.29 (dd, *J* = 15.0, 8.8 Hz, 1H), 1.24 (d, *J*= 6.9 Hz, 3H), 1.24 (d, *J* = 6.1 Hz, 3H), 1.22 (m, 1H), 1.15 (d, *J*= 6.5 Hz, 3H), 1.11 (t, *J* = 7.3 Hz, 1H).
^{13}C{^{1}H} NMR (150 MHz, $CDCl_3$, 25ºC) δ 208.0, 200.5, 164.6, 137.7, 135.6, 104.0, 89.4, 87.1, 84.7, 83.8, 78.4, 70.6, 69.3, 65.8, 51.8, 42.9, 40.6, 40.2, 37.0, 28.3, 23.8, 22.8, 21.2, 19.7, 17.2, 15.2, 13.1, 11.1, 10.1.

(2*S*,3*R*,4*S*,6*R*)-4-(dimethylamino)-2-(((3*R*,5*R*,6*R*,7*R*,9*R*,13*R*,14*R*,*E*)-14-ethyl-13-hydroxy-7-methoxy-3,5,7,9,11,13-hexamethyl-2,4,10-trioxooxacyclotetradec-11-en-6-yl)oxy)-6-methyltetrahydro-2*H*-pyran-3-yl acetate (compound **9**): **7** (50 mg, 0.084 mmol) was dissolved in 2 mL CH_3OH and HCl (168 μL of 0.01 mol/L HCl_{aq}) was added. The mixture was stirred for 2 hours at room temperature and after that 10 mL saturated $NaHCO_3$ was added to stop the reactions and 50 mL of ethyl acetate was added. Organic layer was extracted three times with 50 mL of saturated $NaHCO_3$. The separated organic layer was evaporated to dryness and the synthesized derivative **9** was purified using column chromatography and silica gel with dichloromethane/acetone (6:1) as an eluent. The product was obtained as white solid (43.2 mg, 84%). HRMS (MALDI-TOF) m/z: [M

+ H]+ Calcd for $C_{32}H_{54}NO_{10}^+$ 612.3742; Found 612.3729; FT-IR ($CDCl_3$ solution): a broad band at 3471 cm^{-1} $\nu(O_{12}\text{-}H)$; 1745 cm^{-1} $\nu(C_{9'}{=}O)_{acetyl}+\nu(C_1{=}O)_{lactone}$, 1715 cm^{-1} $\nu(C_3{=}O)$, 1668 cm^{-1} $\nu(C_9{=}O)$, 1245 cm^{-1} ν(C-O), 1163 cm^{-1} ν(C-O), 1106 cm^{-1} ν(C-O), 1062 cm^{-1} ν(C-O); Anal. Calcd for $C_{32}H_{53}NO_{10}$: C, 62.83; H, 8.73; N, 2.29. Found: C, 62.82; H, 8.75; N, 2.27. HPLC: t_R= 4.96 min; ^1H and $^{13}C\{^1H\}$ NMR (Tables S1 and S2).

^1H NMR (600 MHz, $CDCl_3$, 25ºC) δ 6.76 (s, 1H), 4.86 (m, 1H), 4.73 (dd, *J*= 10.7, 7.5 Hz, 1H), 4.32 (d, *J* = 7.6 Hz, 1H), 4.06 (d, *J* = 6.2 Hz 1H), 3.66 (m, 1H), 3.54 (m, 1H,), 3.40 (m, 1H), 3.02 (s, 3H), 2.97 (m, 1H), 2.69 (m, 1H), 2.26 (s, 6H), 2.06 (s, 3H), 2.05 (s, 3H), 1.90 (m, 1H), 1.84 (m, 2H), 1.73 (m, 1H), 1.73 (m, 1H), 1.40 (d, *J* = 6.8 Hz, 3H), 1.37 (s, 3H), 1.31 (m, 1H), 1.24 (d, *J*= 6.1 Hz, 3H,), 1.22 (bs, 3H), 1.10 (m, 3H), 1.09 (d, *J* = 7.1 Hz, 3H), 0.96 (t, *J* = 7.5 Hz, 1H).

$^{13}C\{^1H\}$ NMR (151 MHz, $CDCl_3$, 25ºC) δ 206.3, 205.2, 169.9, 144.5, 139.1, 101.1, 88.5, 83.3, 79.1, 76.9, 75.2, 71.5, 69.0, 63.5, 50.8, 48.0, 40.8, 38.6, 34.0, 30.8, 23.4, 22.8, 21.9, 21.5, 21.2, 19.9, 19.1, 15.7, 12.7, 10.7.

(3*R*,5*E*,7*R*,9*R*,11*E*,13*S*,14*R*)-14-ethyl-13-hydroxy-7-methoxy-3,5,7,9,11,13-hexamethyloxacyclotetradeca-5,11-diene-2,4,10-trione (compound **10**): **5** (50 mg, 0.084 mmol) was dissolved in 4 mL mixture of THF:DMF in 3:1 ratio and 1,1'-Carbonyldiimidazole (CDI) (46.5 mg, 0.28 mmol), sodium hydride (7.26 mg, 29 mmol) and 1,1,3,3-tetramethylguanidine (TMG, 33 mg, 29 mmol) were added. The mixture was stirred at 80°C for 1h The mixture was vigorously stirred at 80 °C, heating using an oil bath. and after that 1 mL of water was added and the mixture was diluted in 30 mL of diethyl ether and extracted three times with 30 mL of water. The separated organic layer was evaporated and white solid was obtained (34 mg, 80%). HRMS (MALDI-TOF) m/z: [M + Na]+ Calcd for $C_{22}H_{34}O_6Na^+$ 417.2248; Found 417.2264; FT-IR ($CDCl_3$ solution): a broad band at 3486 cm^{-1} $\nu(O_{12}\text{-}H)$; 1732 cm^{-1} $\nu(C_1{=}O)_{lactone}$, 1674 cm^{-1} $\nu(C_3{=}O)+\nu(C_9{=}O)$, 1234 cm^{-1} ν(C-O), 1157 cm^{-1} ν(C-O), 1098 cm^{-1} ν(C-O), 1050 cm^{-1} ν(C-O); Anal. Calcd for $C_{22}H_{34}NO_6$: C, 66.98; H, 8.69. Found: C, 67.02; H, 8.68. HPLC: t_R= 5.05 min; ^1H and $^{13}C\{^1H\}$ NMR (Tables S1 and S2).

10a: ^1H NMR (600 MHz, $CDCl_3$, 25ºC) δ 6.54 (q, *J*= 1.6 Hz, 1H), 6.24 (q, *J* = 1.5 Hz, 1H), 4.98 (dd, *J*= 10.9, 2.2 Hz, 1H), 4.16 (q, *J*= 7.0 Hz, 1H), 3.21 (dqd, *J* =8.5, 6.6, 4.7 Hz, 1H), 3.13 (s, 3H), 2.05 (m, 1H), 2.05 (d, *J*= 1.4 Hz, 3H), 2.04 (d, *J* = 1.4 Hz, 3H), 1.96 (m, 1H), 1.88 (m, 1H), 1.59 (m, 1H), 1.46 (d, *J* = 6.9 Hz, 3H), 1.42 (s, 3H), 1.17 (s, 3H), 1.05 (d, *J* = 6.6 Hz, 3H), 0.93 (t, *J* = 7.4 Hz, 1H).

$^{13}C\{^1H\}$ NMR (150 MHz, $CDCl_3$, 25ºC) δ 206.9, 196.1, 172.1, 142.9, 142.4, 140.5, 139.4, 80.6, 78.3, 73.8, 50.0, 46.7, 45.5, 35.2, 24.9, 21.2, 20.4, 19.5, 14.1, 12.6, 12.3, 10.6.

10b: ^1H NMR (600 MHz, $CDCl_3$, 25ºC) δ 6.92 (m, 1H), 6.16 (q, *J*= 1.4 Hz, 1H), 5.02 (dd, *J*= 10.8, 2.2 Hz, 1H), 4.17 (q, *J* = 6.8 Hz, 1H), 3.37 (m, 1H), 3.37 (s, 3H), 2.22 (dd, *J*= 14.8, 5.6 Hz, 1H), 2.08 (d, *J* = 1.5 Hz, 3H), 1.88 (m, 1H), 1.77 (d, *J*= 1.5 Hz, 3H), 1.71 (ddd,*J*= 14.8, 4.9, 0.8 Hz, 1H), 1.54 (m, 1H), 1.41 (d, *J* = 6.8 Hz, 3H), 1.36 (s, 3H), 1.26 (s, 3H), 1.13 (d, *J*= 6.8 Hz, 3H), 0.90 (t, *J*= 7.4 Hz, 1H).

$^{13}C\{^1H\}$ NMR (150 MHz, $CDCl_3$, 25ºC) δ 207.0, 194.8, 170.6, 148.9, 141.8, 138.9, 137.9, 79.5, 78.6, 73.7, 49.8, 48.7, 43.1, 35.4, 23.5, 21.8, 21.3, 19.9, 13.7, 12.9, 12.8, 10.5.

1.2. Details of HPLC, 1D and 2D NMR and FT-IR characterization of 1–10.

The FT-IR spectra of **1**–**8** were recorded using ATR technique and **9** and **10** in $CDCl_3$ solution measurements were performed at spectrometer equipped with a DTGS detector

and two-columnar purge gas generator at resolution 1 cm^{-1}, NSS = 150, range 4000-400 cm^{-1}. The Happ-Genzel apodization function was used.
The ^{1}H and $^{13}C\{^{1}H\}$ NMR measurements of **1–10** analogs were performed in $CDCl_3$ and/or DMSO-d_6 on 600 MHz and 500 MHz spectrometers.
For 600 MHz spectrometer: the operating frequencies for ^{1}H measurements were 600.08 MHz; pulse width corresponding to the flip angle of 45^0; spectral width swh = 9842.5 Hz; acquisition time at= 0.2 sec; relaxation delay d_1=1.0 s; T = 293.0 K, TMS was used as the internal standard. No window function or zero filling were used. Digital resolution was 0.2 Hz/point. $^{13}C\{^{1}H\}$ NMR spectra were recorded at the operating frequency 150.454 MHz; pulse width corresponding to the flip angle of 60^0; sw = 19000 Hz; at = 1.8 s; d_1=1.0 s; T = 293.0 K and TMS as the internal standard. Line broadening parameters of 0.5 or 1 Hz were applied.
For 500 MHz spectrometer: the operating frequencies for ^{1}H measurements were 500.25 MHz; pulse width corresponding to the flip angle of 45^0; spectral width swh = 11029.4 Hz; acquisition time at= 0.2 sec; relaxation delay d_1=1.0 s; T = 293.0 K, TMS was used as the internal standard. No window function or zero filling were used. Digital resolution was 0.2 Hz/point. $^{13}C\{^{1}H\}$ NMR spectra were recorded at the operating frequency 125.79 MHz; pulse width corresponding to the flip angle of 60^0; sw = 35714.3 Hz; at = 2.0 s; d_1=0.92 s; T = 293.0 K and TMS as the internal standard. Line broadening parameters of 0.5 or 1 Hz were applied.
The ^{1}H and $^{13}C\{^{1}H\}$ NMR resonances in solution were unambiguously assigned on the basis of the HMBC, HSQC, COSY and NOESY spectra.
For HPLC separations the C18, 250×4.6 mm (5 µm, carbon load 17%, pH 2 to 13) column at 25 ºC was used. The flow rates was 0.75 mL/min with injection volumes of 5 µL. Mixtures of H_2O:CH_3CN:0.1% NH_3(aq) 10:65:25 was used as the mobile phase. The analytical wavelengths was λ_{max} = 220 and 241 nm.

1.3. Single molecule DFT calculations [XC functional: BLYP-D3; basis set: TZ2P] by ADF package.

Models of macrolide derivatives **9**, **10a** and **10b** were initially built for single molecule. DFT calculations on the basis of our x-ray determined structure of **5** (Fig. S20, see X-ray studies section) and *via* removing of saccharide moiety at C(5) for **10a** and **10b** as well as *via* inversion of configuration at C(12) for **9**. Next the key mutual relative arrangements of the *ansa*-bridge groups and C(17)-substituent were assumed according to our experimental ^{1}H-^{1}H NOESY spectra. Such prepared structures have been initially optimized by molecular mechanics and then subjected to DFT calculations at 298.15 K (geometry optimization and frequencies) using XC functional GGA:BLYP D3 with basis set TZ2P by Amsterdam Density Theory package (ADF *ver.* 2017.114, with recent revision r67691).[1–4] Optimized structures of **9**, **10a** and **10b** were displayed in Figs 2 and 3 as well as the output files for them were attached.

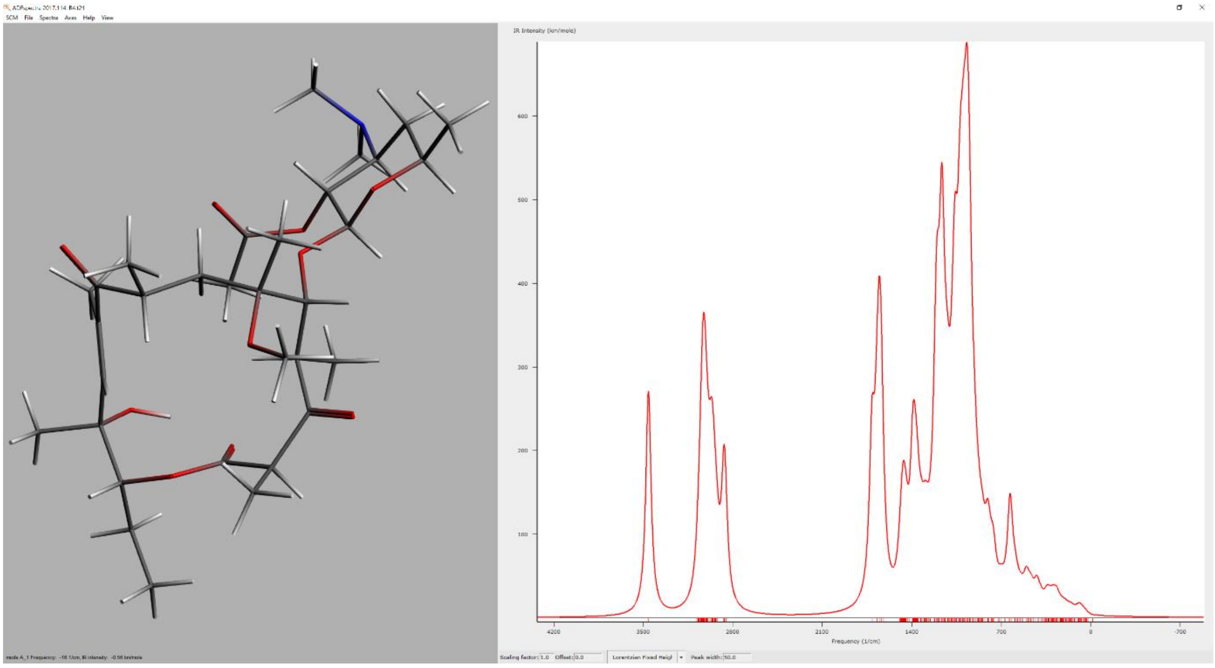

Figure S10. Calculated structure of **9** (ΔG° = -11891.49 kcal/mol; XYZ coordinates) and IR spectrum (no imaginary frequencies detected in the range 4000-400 cm^{-1}; 1 imaginary frequency was detected at ν = -16 cm^{-1}; Intensity = -0.56 km/mole → rescanned ν = 36 cm^{-1}; Intensity = 0.54 km/mole).

<u>XYZ coordinates of **9**:</u>

O	14.04415587	32.64292656	26.70224323
C	14.29994135	32.79027405	28.06726232
C	15.77808824	32.40004827	28.27038766
C	16.18883088	32.55737847	29.74991361
O	14.44652992	28.03005671	22.87010689
N	17.58922920	32.22644264	30.07965386
O	14.09846153	34.15938483	28.45171280
C	10.80526910	30.96501430	25.59712127
C	13.61516874	34.62019296	21.96403622
C	11.43499359	29.11823455	23.84947720

O	11.49011572	33.21091883	24.15168458
C	10.32592161	29.94405078	24.49924216
O	11.42340498	29.19683615	22.50702723
C	12.67701919	32.58858232	26.21883165
C	12.69491171	33.49118222	24.92704201
O	9.94129581	31.53398284	26.24747590
C	12.53840325	30.18178780	27.16104270
C	14.53339320	28.14284410	20.48775392
O	15.92551065	31.00718214	27.85053288
C	13.94470463	30.20807975	21.78526140
C	13.81402846	28.68253461	21.74771335
C	12.28576994	31.11224095	25.94152853
C	13.73681819	33.18545308	22.49403268
C	18.60509645	32.80823089	29.18934880
C	13.91209976	33.07112569	24.03490007
C	15.82949225	34.00034277	30.18814677
C	14.36680109	34.36146047	29.87051896
C	14.02021642	35.81480378	30.18005811
C	15.03213618	30.94459000	22.07794891
C	9.34888097	30.62115488	23.51670358
O	15.88131510	33.05325275	21.37127872
C	12.29033600	28.31594431	21.65064789
C	16.39302092	30.43804596	22.49672484
C	14.95315216	32.45132759	21.90036869
C	12.82114921	34.96495518	25.35619731
C	11.94519519	26.83705111	21.86213924
C	17.84098343	30.79844144	30.32275090
C	10.30619645	33.97900007	24.44021597
C	10.44084420	26.53420061	21.74760702
H	13.04798263	30.73815276	21.46976882
H	16.69115149	30.91029571	23.44278744
H	16.40845746	29.35594158	22.62427777
H	17.13569285	30.74450059	21.74961052
H	14.45511712	35.23753928	22.30139415
H	13.62197863	34.62934410	20.86813854
H	12.67929444	35.07248622	22.30733484
H	12.82922438	32.62855980	22.23298796
H	14.78341472	33.65769938	24.34293401
H	14.17155317	32.03417503	24.26015666
H	13.74167981	35.08066638	25.93354190
H	11.99345968	35.27552995	26.00182974
H	12.86451647	35.62721131	24.48757565
H	10.44108943	35.03789432	24.18518615
H	9.99321815	33.88187196	25.48790271
H	9.52170389	33.55898849	23.80636505
H	11.94342718	28.63287065	20.66045858
H	12.49148796	26.27253354	21.09687160
H	12.33052302	26.50208778	22.82825485
H	9.86882532	27.04013667	22.53378545

H	10.03667665	26.85974429	20.78014564
H	10.26040539	25.45764604	21.84448683
H	14.44280982	27.05371645	20.42343016
H	14.12781787	28.59815293	19.57781490
H	15.59464324	28.39625835	20.55307253
H	12.89689684	30.75017130	25.11128111
H	11.94856696	30.51566486	28.02237461
H	13.59729453	30.18174867	27.42598043
H	12.25053377	29.15668139	26.91318922
C	16.43363308	30.77905176	26.58600900
H	14.65484080	36.48895233	29.59307063
H	14.17600335	36.02539357	31.24452199
H	12.97332102	36.01957461	29.93191385
H	13.69817328	33.69335916	30.44589065
H	16.47932618	34.72145985	29.67550773
H	16.01641441	34.09311191	31.26460273
H	18.64300066	32.33291595	28.19125452
H	18.42576357	33.87896154	29.04958136
H	19.59056627	32.69554799	29.65673254
H	17.82381532	30.18079645	29.40617624
H	17.08910788	30.40251840	31.01487899
H	18.82593200	30.68611916	30.79349515
H	13.64188854	32.14961676	28.68182717
H	11.99371498	33.01915381	26.96103395
H	16.37993591	33.03719944	27.61680441
H	15.56724886	31.86580212	30.33658483
H	13.83844467	28.13352306	23.63510094
O	12.22595447	28.44146983	24.50457375
H	9.76961831	29.20477848	25.09685485
H	16.99908209	29.02520799	25.49234132
H	16.12526289	28.67408940	27.02904717
H	15.25634686	29.30224559	25.61249081
C	16.20229881	29.34306883	26.16806419
O	16.97401108	31.62406264	25.90175544
H	9.87950026	31.34183541	22.88907697
H	8.57400382	31.14220522	24.08525489
H	8.87712591	29.87708529	22.86804275

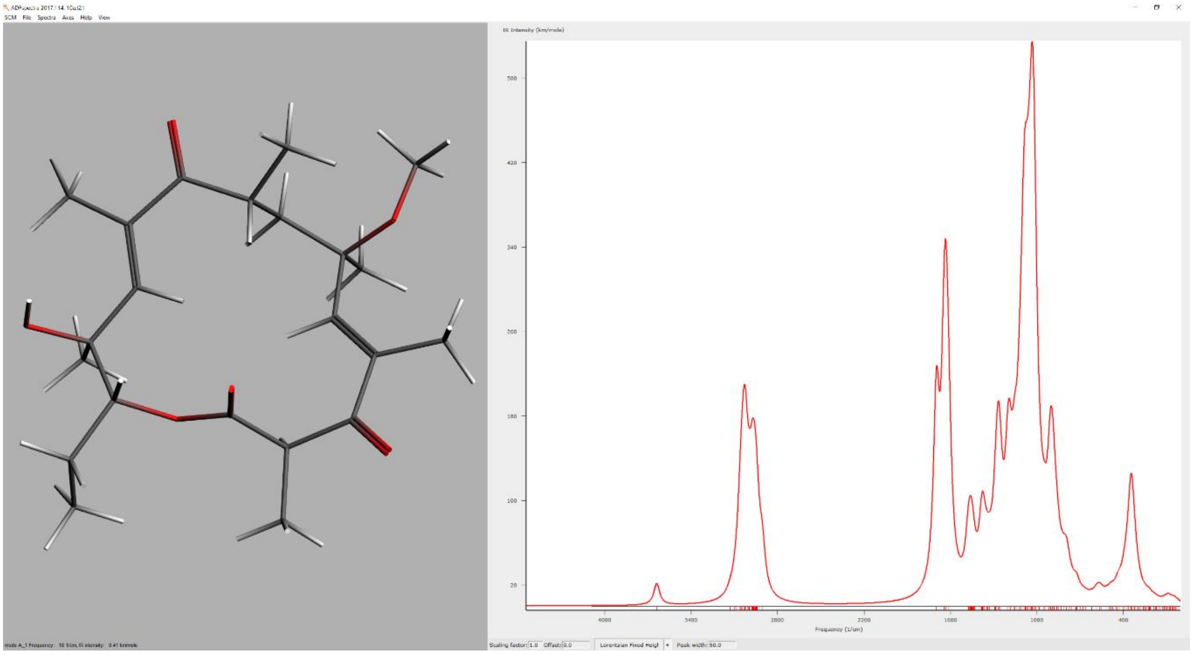

Figure S11. Calculated structure of **10a** of C(2*R*) configuration (ΔG° = -7734.42 kcal/mol; XYZ coordinates) and IR spectrum (no imaginary frequencies detected in the range 4000-400 cm^{-1}).

XYZ coordinates of **10a**:

C	17.72284541	18.38923055	11.71894985
C	12.81471618	21.48932202	14.18034021
C	15.82625057	24.72334059	10.40758221
C	13.81644430	19.86427305	12.48686250
O	15.28926275	25.08018881	13.41583659
C	13.48117798	20.10001472	13.95934440
O	14.85511118	18.97016695	12.38932509
C	14.91663503	22.71903016	13.72821189
C	15.96823653	23.81430676	13.60986354
O	11.61668195	21.54012686	14.43304754
C	12.86258455	23.98262804	14.59031785
O	17.01195951	18.28539325	9.43658592
C	16.67707581	20.41743793	10.64837183
C	16.66926561	18.89348015	10.71529986
C	13.63216971	22.75722480	14.14456148
C	16.28956802	23.37907921	11.00326559
C	16.92976652	23.49492692	12.42115522
C	17.56808658	21.23723302	10.05124152
C	12.55077403	18.95564420	14.43199559
O	18.13543122	23.45843692	9.46210496
C	15.20669897	18.43415677	11.05957450
C	18.81391808	20.80428782	9.30935616
C	17.36927020	22.73791088	10.09941134
C	16.78230502	23.80883101	14.93710916
C	14.90504896	16.92294961	11.04574475
C	16.07650882	26.27784649	13.49653748
H	13.82249686	16.82361882	10.88851997

H	15.83797889	20.87844662	11.15607742
H	19.69710042	21.25346136	9.78141087
H	18.92532724	19.72099022	9.27893073
H	18.79637577	21.19472882	8.28458429
H	16.65648142	25.43528461	10.36241664
H	15.46369016	24.57695435	9.38351468
H	15.02147591	25.14614598	11.01161158
H	15.41619136	22.72039188	11.07317810
H	17.72081679	24.25394486	12.38489646
H	17.43284409	22.54962414	12.66565090
H	17.15059646	22.80079936	15.15678017
H	16.13854077	24.13078473	15.76245422
H	17.64898337	24.47649407	14.87352126
H	16.95124305	26.25640400	12.83172445
H	16.41002278	26.48544709	14.52364598
H	15.41496980	27.08552703	13.17006967
H	11.61460777	18.96753815	13.86710635
H	13.04836002	17.99020970	14.29658456
H	12.30570761	19.08752435	15.49022182
H	14.55385694	18.92068041	10.32561954
H	15.39021666	16.50819185	10.15425684
C	15.28779123	16.10969201	12.29799652
H	16.57404018	18.79758441	8.73252849
H	12.27222515	23.74181197	15.48113049
H	12.14298993	24.27594576	13.81497758
H	13.52119614	24.82561191	14.78595804
H	15.34005242	21.74814388	13.47423017
O	13.23432284	20.35547231	11.54072972
H	17.84732525	17.30830830	11.61304529
H	14.41618934	20.05075463	14.52999140
H	17.41157102	18.61584342	12.74282408
H	18.68548206	18.86955092	11.52396725
H	14.84979798	16.54743206	13.20077802
H	16.36921179	16.04769077	12.44726123
H	14.90807600	15.08575368	12.20028006

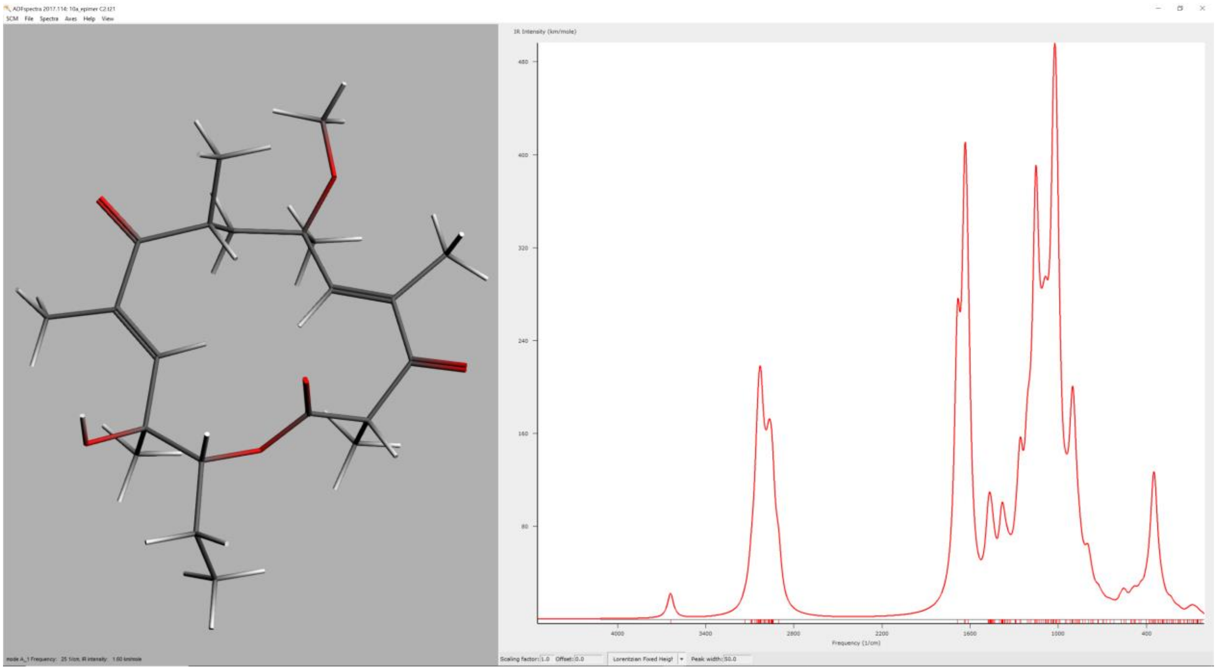

Figure S12. Calculated structure of C(2*S*)-epimer of **10a** (ΔG° = -7733.59 kcal/mol; XYZ coordinates) and IR spectrum (no imaginary frequencies detected in the range 4000-400 cm^{-1}).

XYZ coordinates of C(2*S*)-epimer of **10a**:

C	17.75996120	18.45183789	11.52942969
C	13.05129621	21.29257928	14.53850665
C	15.57069545	24.72901709	10.41096813
C	13.94599348	19.78308708	12.64821386
O	15.18464106	24.99646781	13.46368856
C	13.66984568	19.91571776	14.14788303
O	14.94999529	18.88152623	12.41491574
C	15.00275267	22.62542079	13.78991364
C	15.97383274	23.78490147	13.59702391
O	11.91645147	21.29610837	15.00426535
C	13.16518788	23.73508832	15.17393730
O	16.90719744	18.35457200	9.29396535
C	16.57803858	20.45739305	10.55290574
C	16.62802130	18.93351998	10.60221556
C	13.83374708	22.57801887	14.46210414
C	16.12336613	23.40232580	10.96652419
C	16.85752952	23.54055925	12.33596882
C	17.41123650	21.31135352	9.92170976
H	12.83310513	19.22174415	14.31268579
O	17.85828915	23.55357784	9.30700923
C	15.20808967	18.41893411	11.03746924
C	18.62899660	20.92491908	9.11143558
C	17.16251006	22.80416358	9.98939571
C	16.89399729	23.83526750	14.85158469
C	14.95738650	16.89849710	10.98680234
C	15.88170246	26.25031091	13.51285877
H	13.87437239	16.76003849	10.86765406

H	15.74249344	20.88381263	11.09529852
H	19.52295814	21.39060166	9.54605559
H	18.76744413	19.84569517	9.05606012
H	18.54838169	21.33135352	8.09605479
H	16.36901246	25.47136501	10.30994576
H	15.14111087	24.57164171	9.41475235
H	14.79538787	25.11981918	11.07246688
H	15.28201098	22.71141136	11.09347617
H	17.59791976	24.34576074	12.25267008
H	17.43256349	22.62428476	12.52844104
H	17.35386943	22.85592595	15.02304292
H	16.30424785	24.09873668	15.73553383
H	17.69972090	24.56700886	14.72415335
H	16.70877946	26.30378511	12.79103111
H	16.26731482	26.47065817	14.51917087
H	15.13988148	27.00856799	13.24599934
H	15.06938316	18.44233001	14.94954323
H	15.72772202	20.08746553	14.91591800
H	14.51589024	19.64684843	16.13234107
H	14.48488651	18.91391505	10.37903121
H	15.42377352	16.52825153	10.06643590
C	15.41985203	16.06666917	12.19959114
H	16.41675197	18.86814995	8.62648019
H	12.78971827	23.40250085	16.14807471
H	12.28891650	24.06959709	14.60397234
H	13.83698721	24.58255556	15.29428341
H	15.37657711	21.70390436	13.35085890
O	13.32348718	20.36537765	11.78047652
H	17.90965446	17.37622611	11.40248412
C	14.82314943	19.49841740	15.09086025
H	17.50958962	18.65849501	12.57384681
H	18.69304007	18.96316172	11.27844284
H	14.95148504	16.42234084	13.12327076
H	16.50351785	16.09599469	12.34286250
H	15.13147366	15.01842329	12.05673659

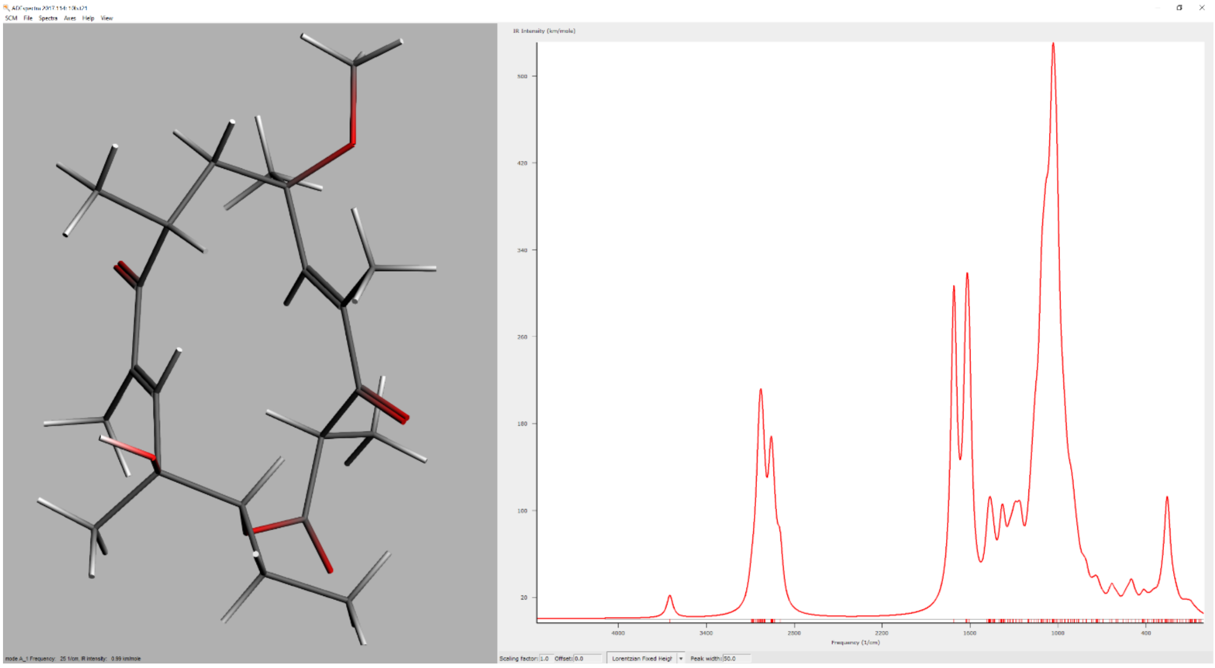

Figure S13. Calculated structure of **10b** of C(2*R*) configuration (ΔG° = -7724.69 kcal/mol; XYZ coordinates) and IR spectrum (no imaginary frequencies detected in the range 4000-400 cm^{-1}).

XYZ coordinates of **10b**:

C	0.48316878	28.44555548	27.16625652
C	2.02687911	33.25041026	29.04950613
C	2.78286509	32.22375705	23.32599748
C	1.95865179	30.94163102	30.22833888
O	4.19057463	35.85521983	26.30174070
C	2.83065178	32.05095421	29.60279038
O	1.18860345	30.16365665	29.37784142
C	3.75919700	33.76878194	27.36172372
C	4.50748642	34.42824085	26.20819711
O	1.00749019	33.61932458	29.62997133
C	1.51693972	34.97406941	27.26383554
O	-0.84607110	30.31885036	26.32720925
C	1.50771286	30.70788637	26.39448811
C	0.36407872	29.97753113	27.08295793
C	2.51518063	33.99314445	27.83380297
C	3.10337046	32.64395833	24.78372398
C	4.06106570	33.85722655	24.79822639
C	2.83379812	30.47319921	26.30988337
C	3.84724798	32.57642294	30.64924836
O	4.90964708	31.20067915	25.41302359
C	0.13557863	30.65575887	28.47934890
C	3.57431115	29.32792782	26.96842503
C	3.70173694	31.42228483	25.51574949
C	6.01953410	34.18757011	26.41910841
C	-1.22099891	30.35496309	29.13368462
C	4.93472852	36.74370256	25.45463101
H	-1.30563083	29.27278888	29.29379956

H	1.11969940	31.60378811	25.91684880
H	4.59914636	29.63335173	27.19850642
H	3.07998581	29.00633660	27.88565790
H	3.65182281	28.46697674	26.29164448
H	3.69580669	31.90018973	22.81226714
H	2.06448324	31.39610167	23.30347625
H	2.35195083	33.06605659	22.77148406
H	2.17106628	32.95249402	25.26066488
H	3.54712849	34.66895014	24.27066714
H	4.95573822	33.60221102	24.22061638
H	6.22317793	33.11223711	26.40862651
H	6.34484926	34.61543257	27.37470974
H	6.60635532	34.63907015	25.61299595
H	4.94499904	36.41912088	24.40259089
H	5.97320991	36.86874422	25.79534575
H	4.42506384	37.71007994	25.51745098
H	3.31283289	33.02045598	31.49379331
H	4.45927387	31.75311790	31.02853481
H	4.49878423	33.33491165	30.20401793
H	0.22860672	31.73545108	28.34226891
H	-2.00328350	30.64256151	28.42314145
C	-1.40117635	31.10402058	30.46748229
H	-0.77274607	29.89944498	25.45124055
H	1.34146360	35.79513367	27.96938398
H	0.54851060	34.47683099	27.12498133
H	1.86189066	35.39723311	26.32103989
H	4.35362536	32.99902676	27.84580220
O	2.06853464	30.59635818	31.38192062
H	-0.49586053	28.02604598	27.41453884
H	3.35824399	31.55747899	28.78020427
H	1.19680205	28.13328279	27.93013800
H	0.80656083	28.03860012	26.20094181
H	-1.25124646	32.18275983	30.33879869
H	-0.68391601	30.75417830	31.21685082
H	-2.41172725	30.94075888	30.86044905

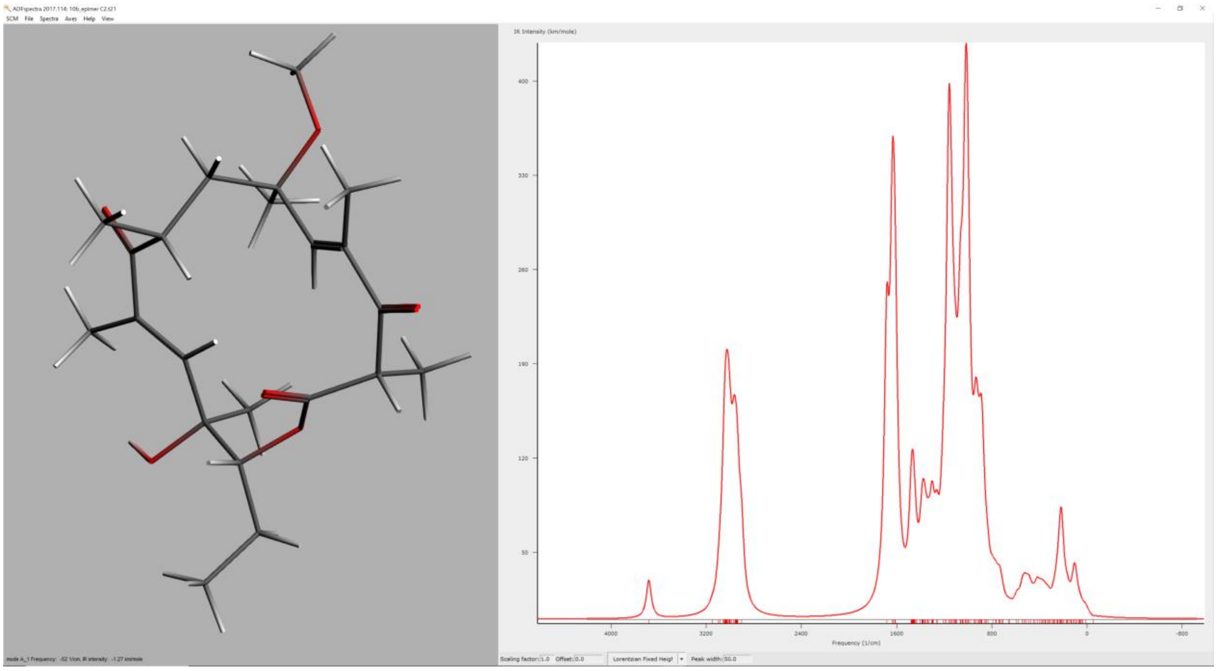

Figure S14. Calculated structure of C(2*S*)-epimer of **10b** (ΔG° = -7732.41 kcal/mol; XYZ coordinates) and IR spectrum (no imaginary frequencies detected in the range 4000-400 cm^{-1}; 1 imaginary frequency was detected at ν = -52 cm^{-1}; Intensity = -1.27 km/mole → rescanned ν = 53 cm^{-1}; Intensity = 1.26 km/mole).

XYZ coordinates of C(2*S*)-epimer of **10b**:

C	2.71698316	28.83153973	28.07040691
C	2.27162593	34.57341488	29.75536741
C	1.27161336	33.80208977	24.03504435
C	1.07818551	32.34328119	29.29911880
O	5.37464408	34.74792970	26.69162059
C	1.88990195	33.16158556	30.30295315
O	1.35405463	31.00388510	29.38953040
C	3.54243043	33.71181756	27.80534801
C	4.37496959	33.68921200	26.52256856
O	1.97437964	35.54568764	30.44196456
C	3.15038758	36.21868221	28.05622838
O	0.57834069	28.40823247	26.91685894
C	1.75592211	30.55256494	26.52957959
C	1.40951404	29.43824799	27.50779615
C	3.02606001	34.76859786	28.46632773
C	2.21323707	33.14031656	25.07635262
C	3.47932000	34.01027881	25.28168225
C	2.08520181	30.48226788	25.22437880
H	1.15723760	33.40176573	31.08620752
O	3.23918808	31.64921601	23.50764023
C	0.50845823	30.04665780	28.64020683
C	2.13939222	29.20900584	24.39903352
C	2.56372389	31.73620023	24.53295928
C	5.08921526	32.32313036	26.40413293
C	-0.07461921	29.06592237	29.67419303
C	6.33150941	34.93963357	25.63736064

H	-0.24852831	29.65191368	30.58584170
H	1.78361853	31.52992322	26.99267488
H	1.12612592	28.86291274	24.15977446
H	2.66993976	29.39585121	23.46215659
H	2.66743855	28.39899988	24.91897820
H	1.78571672	33.91224014	23.07380988
H	0.37160439	33.19562575	23.87750033
H	0.95743493	34.79069789	24.38866644
H	1.65961831	33.08198222	26.01676295
H	3.16064210	35.05350971	25.38508487
H	4.08722567	33.93687665	24.37270550
H	4.38022641	31.49600663	26.49419384
H	5.82938632	32.23248503	27.20747292
H	5.59148959	32.21679979	25.43898112
H	5.86142150	35.22558120	24.68531993
H	6.95982972	34.05236339	25.47193523
H	6.97028184	35.76216791	25.97360797
H	3.56035639	33.13938389	31.68790929
H	3.83942220	32.13462080	30.25384309
H	2.74461725	31.56759322	31.52681529
H	-0.29981460	30.60948645	28.16239009
H	0.66836311	28.30313329	29.93540584
C	-1.39740972	28.40221504	29.24440235
H	1.10339539	27.92630915	26.25693470
H	3.62781276	36.79469663	28.85754304
H	2.14857439	36.64842964	27.92651765
H	3.73196927	36.33775956	27.14465206
H	3.35578086	32.71830250	28.20555619
O	0.25859957	32.82920852	28.54188729
H	2.49028716	28.01068011	28.75799849
C	3.08562530	32.45143019	30.97976509
H	3.30090143	29.58936470	28.60144439
H	3.32842426	28.44133306	27.24919620
H	-1.25992341	27.77138606	28.36352206
H	-2.15439542	29.16050543	29.00631119
H	-1.78898576	27.78376173	30.06109379

1.4 X-Ray crystallography

Single crystals of 2'-acetylated **2** were grown from acetonitrile and those of **5** and **8** from methanol. Diffraction data were collected for crystals mounted on a plastic loop with small amount of perfluoropolyether. The diffraction measurements were carried out with a SuperNova diffractometer using hi-flux micro-focus Nova Cu-Kα radiation (λ=1.54184 Å). Data collection and reduction were performed with the CrysAlis Pro software.[3] All structures were solved by directed methods using the SHELXT program.[4] The structures were refined by full-matrix least-squares method on F^2 with SHELXL-2018.[4] All calculations were carried out within OLEX-2.[5]

CCDC 2111470; CCDC 2111471; CCDC 2111770 contain the supplementary crystallographic data for **5**, **8** and 2'-acetylated **2**.

Crystal data for **5**: $C_{32}H_{53}NO_{10}$ (M=611.75 g/mol): monoclinic, space group P2$_1$ (no. 4), a = 13.1837(2) Å, b = 8.63620(10) Å, c = 15.6798(3) Å, β = 107.884(2)°, V = 1698.99(5) Å^3, Z = 2, T = 290(1) K, μ(Cu Kα) = 0.721 mm^{-1}, D_{calc} = 1.196 g/cm^3, 17939 reflections measured (5.922° ≤ 2Θ ≤ 140.15°), 6310 unique (R_{int} = 0.0221, R_{sigma} = 0.0219) which were used in all calculations. The final R_1 was 0.0327 (I > 2σ(I)) and wR_2 was 0.0926 (all data).

Crystal data for **8**: $C_{30}H_{49}NO_8$ (M=551.70 g/mol), orthorhombic, space group P2$_1$2$_1$2$_1$ (no. 19), a = 7.70610(10) Å, b = 15.9021(3) Å, c = 24.3626(4) Å, V = 2985.47(8) Å^3, Z = 4, T = 134(3) K, μ(Cu Kα) = 0.715 mm^{-1}, D_{calc} = 1.227 g/cm^3, 22131 reflections measured (6.64° ≤ 2Θ ≤ 136.50°), 5455 unique (R_{int} = 0.0376, R_{sigma} = 0.0273) which were used in all calculations. The final R_1 was 0.0434 (I > 2σ(I)) and wR_2 was 0.1175 (all data).

Crystal data for 2'-acetylated **2**: $C_{40}H_{69}N_1O_{13}\cdot 4(C_2H_3N)$ (M=936.17 g/mol): monoclinic, space group P2$_1$ (no. 4), a = 11.11359(6) Å, b = 27.20524(13) Å, c = 18.41222(11) Å, β = 103.9742(6)°, V = 5402.14(5) Å^3, Z = 4, T = 131(1) K, μ(CuKα) = 0.680 mm^{-1}, D_{calc} = 1.151 g/cm^3, 43371 reflections measured (4.946° ≤ 2Θ ≤ 153.004°), 18813 unique (R_{int} = 0.0235, R_{sigma} = 0.0262) which were used in all calculations. The final R_1 was 0.0337 (I > 2σ(I)) and wR_2 was 0.0927 (all data).

Compound 2'-acetylated **2** crystallizes as an acetonitrile solvate with two molecules of 2'-acetylated **2** in the asymmetric unit (Fig. S2). These two molecules adopt a similar conformation (Fig. S15). Comparison of the structure of 2'-acetylated **2** with the solid state structure of clarithromycin[6] indicates that the chemical modification introduced to the macrocycle in 2'-acetylated **2** influences its C7-C12 region whereas the conformation of the remaining part of the molecule remains practically unaltered (Fig. S16). The two OH groups of 2'-acetylated **2** are involved in intermolecular hydrogen bonds (Fig. S17). The molecular structure of **8** is shown in Fig. 1. The C1=C2 bond in the 2-methylene-1,3-dioxolan fragment has Z configuration. There is a significant conjugation between π systems of the C3=O3 group and the C1=C2 vinyl group resulting in a partially double-bond between C2 and C3 (bond length of 1.464(4) Å; E-configuration). This, in turn, generates within the macrocycle a short intramolecular contact of 2.17 Å between C3-H3 and O1. The O-H group of the desosamine fragment of **8** forms an intramolecular O-H···O hydrogen bond with the C3=O3 group of the macrocycle (Fig. S18). The molecular structure of **5** is illustrated in Fig. S1 and S19. Fig. S20 shows an overlay of the molecules of **8** and **5** with the fitted fragment marked with atomic

labels. In crystals, the molecules of **5** are assembled *via* O-H···N hydrogen bond into chains along the b axis.

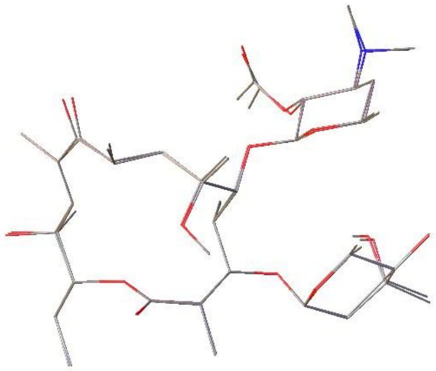

Figure S15. Superposition of molecules A and B of 2'-acetylated **2** (r.m.s. = 0.16 Å)

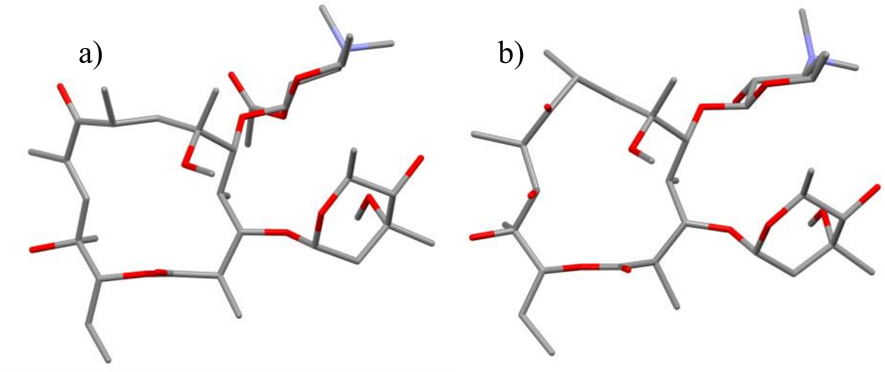

Figure S16. Comparison of the conformation of molecule B in 2'-acetylated **2** (a) with the conformation of clarithromycin molecule in its acetonitrile solvate (b) (refcode: CIWJIC)[6]

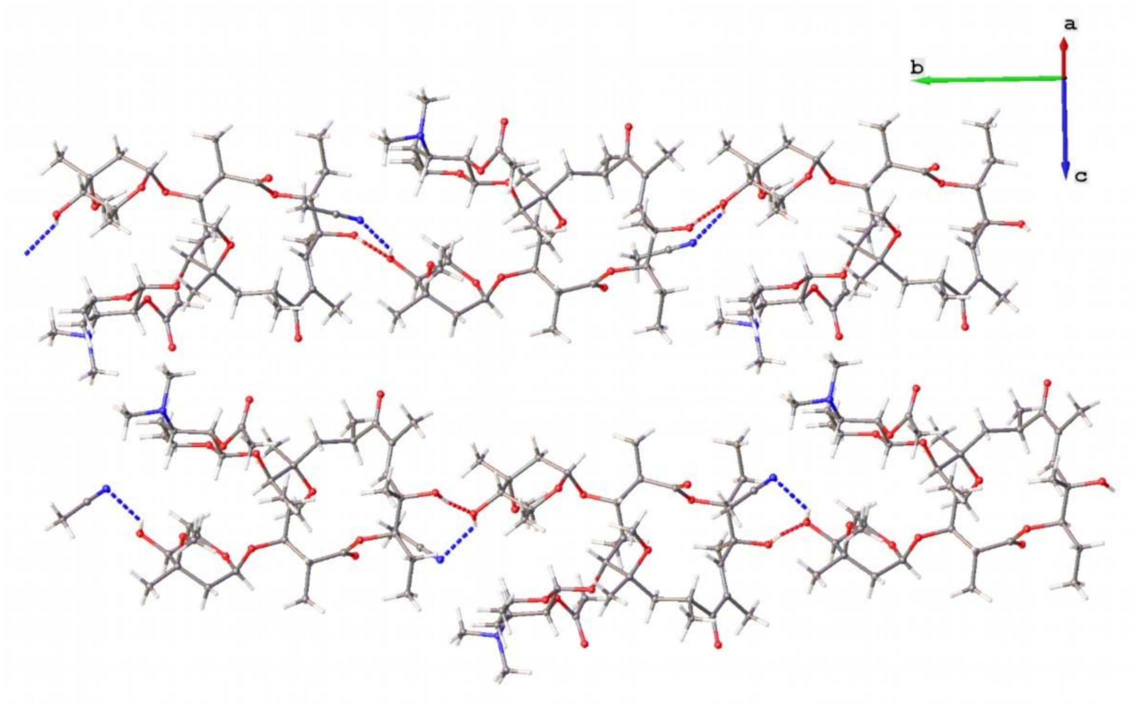

Figure S17. Two symmetry independent chains *via* O-H···O hydrogen bonds extended along [010] in 2'-acetylated **2**. The OH groups of the cladinose part are bonded to acetonitrile molecules *via* O-H···N interaction.

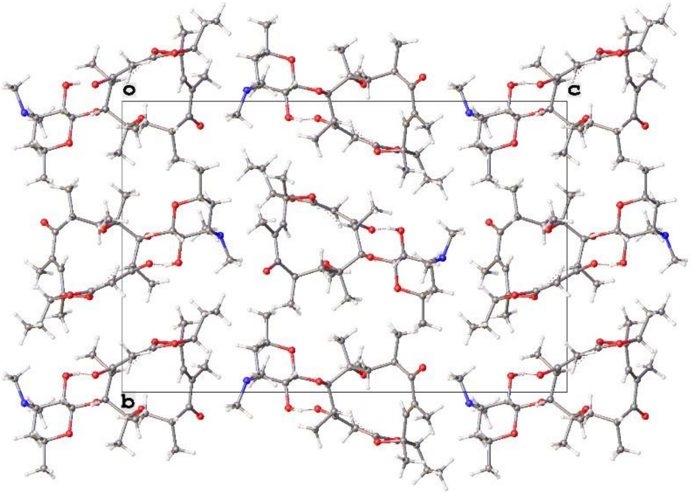

(a)

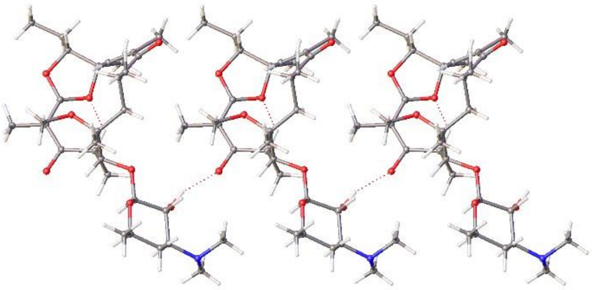

(b)

Figure S18. (a) Crystal packing in **8** and (b) hydrogen-bonded chains along [100]

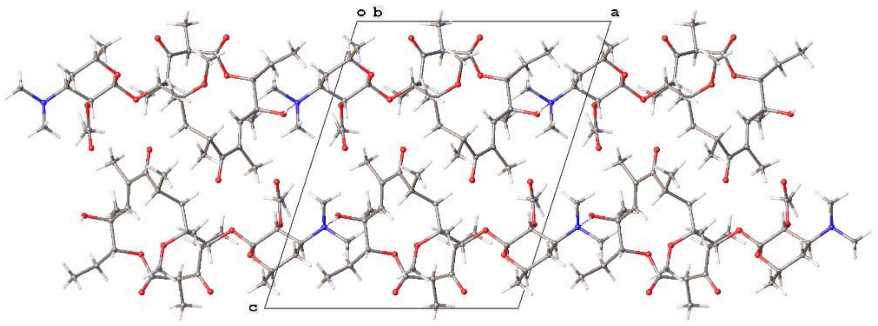

(a)

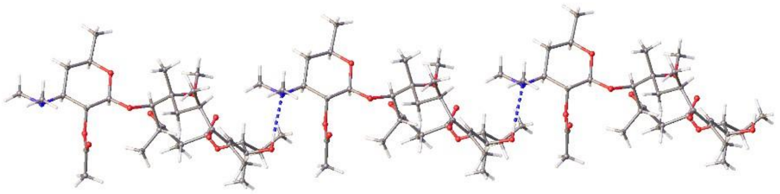

(b)

Figure S19. (a) Crystal packing in **5** and (b) hydrogen-bonded chains along [010]

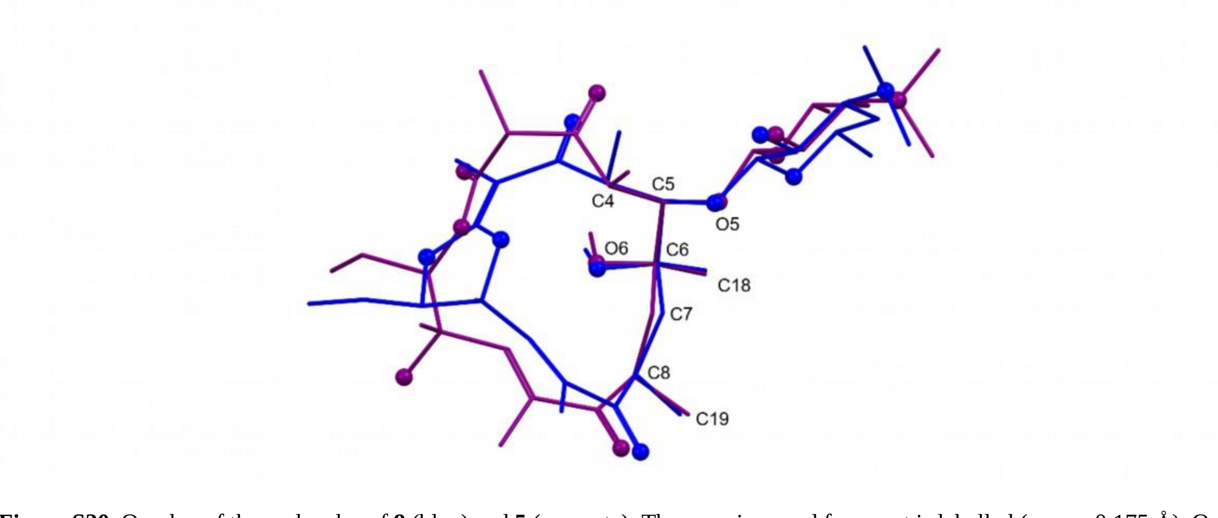

Figure S20. Overlay of the molecules of **8** (blue) and **5** (magenta). The superimposed fragment is labelled (r.m.s.=0.175 Å). O and N atoms are represented as spheres, hydrogen atoms are omitted for clarity.

2. References

(1) Velde, G. te; Bickelhaupt, F. M.; Baerends, E. J.; Guerra, C. F.; Gisbergen, S. J. A. van; Snijders, J. G.; Ziegler, T. Chemistry with ADF. *Journal of Computational Chemistry* **2001**, *22* (9), 931–967. https://doi.org/10.1002/jcc.1056.

(2) *ADF Userguide Version 2017.114*; Vrije Universiteit, Amsterdam, The Netherlands.

(3) *Rigaku Oxford Diffraction, CrysAlisPro Software System*; Rigaku Corporation: Oxford, UK, 2018.

(4) Sheldrick, G. M. Crystal Structure Refinement with SHELXL. *Acta Cryst C* **2015**, *71* (1), 3–8. https://doi.org/10.1107/S2053229614024218.

(5) Dolomanov, O. V.; Bourhis, L. J.; Gildea, R. J.; Howard, J. a. K.; Puschmann, H. OLEX2: A Complete Structure Solution, Refinement and Analysis Program. *J Appl Cryst* **2009**, *42* (2), 339–341. https://doi.org/10.1107/S0021889808042726.
(6) Liang, J.-H.; Yao, G.-W. A New Crystal Structure of Clarithromycin. *J Chem Crystallogr* **2008**, *38* (1), 61–64. https://doi.org/10.1007/s10870-007-9287-5.

3. ^{1}H and ^{13}C{^{1}H} NMR data

Table S1. ^{1}H NMR data of **2 – 10**, assigned on the basis of ^{1}H-^{13}C HSQC, ^{1}H-^{13}C HMBC, ^{1}H-^{1}H COSY and ^{1}H-^{1}H NOESY spectra. The coupling constants (*J*) are expressed in Hz.

H	**2 (CDCl₃)**	**2 (DMSO-*d₆*)**	**3 (DMSO-*d₆*)**	**3 (CDCl₃)**	**4 (DMSO-*d₆*)**	**4 (CDCl₃)**	**5 (CDCl₃)**	**6 (CDCl₃)**	**7 (CDCl₃)**	**7 (DMSO-*d₆*)**	**8 (CDCl₃)**	**9 (CDCl₃)**	**10a (CDCl₃)**	**10b (CDCl₃)**
1	---	---	---	---	---	---	---	---	---	---	---		---	---
2	2.87 (m, 1H)	2.82 (dq, 1H) $^3J_{H2-H3}$=14.1 $^3J_{H2-H16}$=7.0	2.54 (m, 1H)	2.71 (dq, 1H) $^3J_{H2-H3}$=10.5 $^3J_{H2-H16}$=6.8	2.54 (m, 1H)	2.65 (m, 1H)	3.72 (q, 1H) $^3J_{H2-H16}$=6.9	3.73 (m, 1H)	---	---	---	3.66 (m, 1H)	4.16 (q, 1H) $^3J_{H2-H16}$=7.0	4.17 (q, 1H) $^3J_{H2-H16}$=6.8
3	3.97 (d, 1H) $^3J_{H3-H2}$=8.6	3.71 (d, 1H) $^3J_{H3-H2}$=10.1	3.41 (m, 1H)	4.02 (d, 1H) $^3J_{H3-H2}$=10.5	3.41 (m, 1H)	3.79 (d, 1H) $^3J_{H3-H2}$=10.4	---	---	---	---	---	---	---	---
4	1.92 (m, 1H)	1.77 (t, 1H) $^3J_{H7-H17}$=7.5	1.78 (m, 1H)	1.68 (m, 1H)	1.78 (m, 1H)	1.82 (qd, 1H) $^3J_{H4-H5}$=3.1 $^3J_{H4-H17}$=7.2	3.04 (p, 1H) $^3J_{H4-H17}$=7.5 $^3J_{H4-H5}$=7.5	3.01 (m, 1H)	3.91 (dq, 1H) $^3J_{H4-H5}$=9.4 $^3J_{H4-H17}$=7.3	3.81 (m, 1H)	4.01 (dq, 1H) $^3J_{H4-H5}$=9.9 $^3J_{H4-H17}$=6.9	2.97 (m, 1H)	---	---
5	3.66 (d, 1H) $^3J_{H5-H4}$=7.7	3.63 (d, 1H) $^3J_{H5-H4}$=7.7	3.74 (d, 1H) $^3J_{H5-H4}$=3.8	3.92 (d, 1H) $^3J_{H5-H4}$=2.0	3.74 (d, 1H) $^3J_{H5-H4}$=3.8	3.88 (d, 1H) $^3J_{H5-H4}$=3.0	4.12 (d, 1H) $^3J_{H5-H4}$=8.2	4.11 (d, 1H) $^3J_{H5-H4}$=8.6	4.04 (d, 1H) $^3J_{H5-H4}$=9.4	3.91 (d, 1H) $^3J_{H5-H4}$=9.4	4.14 (d, 1H) $^3J_{H5-H4}$=9.9	4.06 (d, 1H) $^3J_{H5-H4}$=6.2	6.24 (q, 1H) $^4J_{H5-H4}$=1.5	6.92 (m, 1H)
6	---	---	---	---	---	---	---	---	---	---	---	---	---	---
7	2.04 (dd, 1H) ^2J=14.9	1.82 (dd, 1H) ^2J=14.7	1.69 (m, 1H)<br>1.20	1.66 m, 1H)<br>1.29	1.69 (m, 1H)<br>1.20	1.60 (dd, 1H) ^2J=15.3	1.82 (dd, 1H) ^2J=14.4	1.84 (m, 1H)<br>1.62	2.14 (m, 1H)<br>1.24	2.03 (m, 1H)<br>1.02	2.32 (dd, 1H) ^2J=14.9	1.84 (m, 2H)	2.05 (m, 1H)<br>1.88	2.22 (dd, 1H) ^2J=14.8

H	**2 (CDCl$_3$)**	**2 (DMSO-*d$_6$*)**	**3 (DMSO-*d$_6$*)**	**3 (CDCl$_3$)**	**4 (DMSO-*d$_6$*)**	**4 (CDCl$_3$)**	**5 (CDCl$_3$)**	**6 (CDCl$_3$)**	**7 (CDCl$_3$)**	**7 (DMSO-*d$_6$*)**	**8 (CDCl$_3$)**	**9 (CDCl$_3$)**	**10a (CDCl$_3$)**	**10b (CDCl$_3$)**
	$^3J_{H7a-H8}$=4.3<br>1.39 (m, 1H)	$^3J_{H7a-H8}$=3.0<br>1.06 (m, 1H)	(m, 1H)	(m, 1H)	(m, 1H)	$^3J_{H7a-H8}$=10.0<br>1.48 (dd, 1H) ^2J = 15.5 $^3J_{H7b-H8}$=4.7	$^3J_{H7a-H8}$=6.9<br>1.51 (m, 1H)	(m, 1H)	(m, 1H)	(m, 1H)	$^3J_{H7a-H8}$=2.9<br>1.29 (dd, 1H) ^2J=15.0 $^3J_{H7b-H8}$=8.8		(m, 1H)	$^3J_{H7a-H8}$=5.6<br>1.71 (ddd, 1H) ^2J=14.8 $^3J_{H7a-H8}$=4.9 $^5J_{H7a-H5}$=0.8
8	3.30 (m, 1H)	3.36 (m, 1H)	3.28 (m, 1H)	3.18 (m, 1H)	3.28 (m, 1H)	3.16 (m, 1H)	3.15 (m, 1H)	3.15 (m, 1H)	3.22 (m, 1H)	3.24 (m, 1H)	3.34 (m, 1H)	3.40 (m, 1H)	3.21 (dqd, 1H) $^3J_{H7b-H8}$=8.5 $^3J_{H8-H19}$=6.6 $^3J_{H7a-H8}$=4.7	3.37 (m, 1H)
9	---	---	---	---	---	---	---	---	---	---	---	---	---	---
10	---	---	---	---	---	---	---	---	---	---	---	---	---	---
11	6.62 (s, 1H)	6.58 (s, 1H)	6.49 (s, 1H)	6.47 (q, 1H) $^4J_{H11-H20}$=1.5	6.49 (s, 1H)	6.42 (q, 1H) $^4J_{H11-H20}$=1.5	6.59 (s, 1H)	6.79 (s, 1H)	6.33 (s, 1H)	6.33 (s, 1H)	6.40 (s, 1H)	6.76 (s, 1H)	6.54 (q, 1H) $^4J_{H11-H20}$=1.6	6.16 (q, 1H) $^4J_{H11-H20}$=1.4
12	---	---	---		---		---	---	---	---	---		---	---
13	5.00 (dd, 1H) $^3J_{H13-H14b}$=10.5	4.88 (dd, 1H) $^3J_{H13-H14b}$=10.7	4.90 (dd, 1H) $^3J_{H13-H14b}$=10.5	4.95 (dd, 1H) $^3J_{H13-H14b}$=11.0	4.90 (dd, 1H) $^3J_{H13-H14b}$=10.5	5.02 (dd, 1H) $^3J_{H13-H14b}$=10.8	4.98 (dd, 1H) $^3J_{H13-H14b}$=9.8	5.68 (dd, 1H) $^3J_{H13-H14b}$=9.9	4.29 (dd, 1H) $^3J_{H13-H14b}$=9.2	4.53 (dd, 1H) $^3J_{H13-H14b}$=10.5	4.28 (dd, 1H) $^3J_{H13-H14b}$=9.4	4.86 (m, 1H)	4.98 (dd, 1H) $^3J_{H13-H14b}$=10.9	5.02 (dd, 1H) $^3J_{H13-H14b}$=10.8

H	**2 (CDCl₃)**	**2 (DMSO-*d₆*)**	**3 (DMSO-*d₆*)**	**3 (CDCl₃)**	**4 (DMSO-*d₆*)**	**4 (CDCl₃)**	**5 (CDCl₃)**	**6 (CDCl₃)**	**7 (CDCl₃)**	**7 (DMSO-*d₆*)**	**8 (CDCl₃)**	**9 (CDCl₃)**	**10a (CDCl₃)**	**10b (CDCl₃)**
	$^3J_{H13-H14a}$=2.6	$^3J_{H13-H14a}$=2.4	$^3J_{H13-H14a}$=2.3	$^3J_{H13-H14a}$=2.2	$^3J_{H13-H14a}$=2.3	$^3J_{H13-H14a}$=2.3	$^3J_{H13-H14a}$=2.9	$^3J_{H13-H14a}$=2.9	$^3J_{H13-H14a}$=4.5	$^3J_{H13-H14a}$=3.2	$^3J_{H13-H14a}$=4.4		$^3J_{H13-H14a}$=2.2	$^3J_{H13-H14a}$=2.2
14	1.92 (m, 1H)<br><br>1.50 (ddt, 1H) ^2J =14.2 $^3J_{H14b-H15}$=7.3 $^3J_{H14b-H13}$=2.9	1.90 (m, 1H)<br><br>1.43 (ddd, 1H) ^2J =14.2 $^3J_{H14b-H13}$=10.6 $^3J_{H14b-H15}$=7.2	1.89 (m, 1H)<br><br>1.43 (ddd, 1H) ^2J =14.2 $^3J_{H14b-H13}$=10.4 $^3J_{H14b-H15}$=7.3	1.95 (dqd, 1H) ^2J =15.2 $^3J_{H14a-H15}$=7.5 $^3J_{H14a-H13}$=2.1<br><br>1.55 (m, 1H)	1.87 (m, 1H)<br><br>1.43 (ddd, 1H) ^2J =14.2 $^3J_{H14b-H13}$=10.4 $^3J_{H14b-H15}$=7.3	1.95 (m, 1H)<br><br>1.55 (m, 1H)	1.95 (ddd, 1H) ^2J =14.4 $^3J_{H14a-H15}$=7.5 $^3J_{H14a-H13}$=2.9<br><br>1.55 (ddd, 1H) ^2J =14.3 $^3J_{H14b-H13}$=9.9 $^3J_{H14b-H15}$=7.3	1.79 (m, 1H)<br><br>1.67 (m, 1H)	1.68 (m, 1H)<br><br>1.64 (m, 1H)	1.72 (m, 1H)<br><br>1.56 (m, 1H)	1.67 (m, 2H)	1.90 (m, 1H)<br><br>1.73 (m, 1H)	1.96 (m, 1H)<br><br>1.59 (m, 1H)	1.88 (m, 1H)<br><br>1.54 (m, 1H)
15	0.90 (t, 3H) $^3J_{H15-H14}$=7.3	0.79 (t, 3H) $^3J_{H15-H14}$=7.3	0.79 (t, 3H) $^3J_{H15-H14}$=7.4	0.91 (t, 3H) $^3J_{H15-H14}$=7.3	0.79 (t, 3H) $^3J_{H15-H14}$=7.4	0.91 (t, 3H) $^3J_{H15-H14}$=7.4	0.92 (t, 3H) $^3J_{H15-H14}$=7.4	0.94 (t, 3H) $^3J_{H15-H14}$=7.5	1.11 (t, 3H) $^3J_{H15-H14}$=7.3	1.01 (t, 3H) $^3J_{H15-H14}$=7.5	1.11 (t, 3H) $^3J_{H15-H14}$=7.3	0.96 (t, 3H) $^3J_{H15-H14}$=7.5	0.93 (t, 3H) $^3J_{H15-H14}$=7.4	0.90 (t, 3H) $^3J_{H15-H14}$=7.4
16	1.24 (d, 3H) $^3J_{H16-H2}$=6.9	1.18 (d, 3H) $^3J_{H16-H2}$=6.4	1.16 (d, 3H) $^3J_{H16-H2}$=6.7	1.33 (d, 3H) $^3J_{H16-H2}$=6.8	1.16 (d, 3H) $^3J_{H16-H2}$=6.7	1.31 (d, 3H) $^3J_{H16-H2}$=6.8	1.34 (d, 3H) $^3J_{H16-H2}$=6.9	1.35 (d, 3H) $^3J_{H16-H2}$=6.8	1.74 (s, 3H)	1.64 (s, 3H)	1.77 (s, 3H)	1.40 (d, 3H) $^3J_{H16-H2}$=6.8	1.46 (d, 3H) $^3J_{H16-H2}$=6.9	1.41 (d, 3H) $^3J_{H16-H2}$=6.8
17	1.09 (d, 3H) $^3J_{H17-H4}$=7.4	1.02 (d, 3H) $^3J_{H17-H4}$=7.3	0.86 (d, 3H) $^3J_{H17-H4}$=7.3	1.03 (d, 3H) $^3J_{H17-H4}$=7.0	0.86 (d, 3H) $^3J_{H17-H4}$=7.3	0.92 (d, 3H) $^3J_{H17-H4}$=7.2	1.11 (d, 3H) $^3J_{H17-H4}$=7.4	1.11 (d, 3H) $^3J_{H17-H4}$=7.4	1.07 (d, 3H) $^3J_{H17-H4}$=7.2	0.97 (d, 3H) $^3J_{H17-H4}$=7.0	1.24 (d, 3H) $^3J_{H17-H4}$=6.9	1.09 (d, 3H) $^3J_{H17-H4}$=7.1	2.04 (d, 3H) $^4J_{H17-H5}$=1.4	1.77 (d, 3H) $^4J_{H17-H5}$=1.5
18	1.39	1.35	1.21	1.26	1.21	1.29	1.28	1.29	1.32	1.26	1.36	1.22	1.17	1.36

H	**2 (CDCl$_3$)**	**2 (DMSO-*d$_6$*)**	**3 (DMSO-*d$_6$*)**	**3 (CDCl$_3$)**	**4 (DMSO-*d$_6$*)**	**4 (CDCl$_3$)**	**5 (CDCl$_3$)**	**6 (CDCl$_3$)**	**7 (CDCl$_3$)**	**7 (DMSO-*d$_6$*)**	**8 (CDCl$_3$)**	**9 (CDCl$_3$)**	**10a (CDCl$_3$)**	**10b (CDCl$_3$)**
	(s, 3H)	(s, 3H)	(s, 3H)	(s, 3H)	(s, 3H)	(s, 3H)	(s, 3H)	(s, 3H)	(s, 3H)	(s, 3H)	(s, 3H)	(s, 3H)	(s, 3H)	(s, 3H)
19	1.14 (d, 3H) $^{3}J_{H19-H8}$=6.7	0.97 (d, 3H) $^{3}J_{H19-H8}$=6.3	1.00 (d, 3H) $^{3}J_{H19-H8}$=6.6	1.19 (d, 3H) $^{3}J_{H19-H8}$=6.5	1.00 (d, 3H) $^{3}J_{H19-H8}$=6.6	1.18 (d, 3H) $^{3}J_{H19-H8}$=6.7	1.14 (d, 3H) $^{3}J_{H19-H8}$=6.8	1.21 (d, 3H) $^{3}J_{H19-H8}$=7.6	1.13 (d, 3H) $^{3}J_{H19-H8}$=6.6	1.03 (d, 3H) $^{3}J_{H19-H8}$=6.5	1.15 (d, 3H) $^{3}J_{H19-H8}$=6.5	1.10 (m, 3H)	1.05 (d, 3H) $^{3}J_{H19-H8}$=6.6	1.13 (d, 3H) $^{3}J_{H19-H8}$=6.8
20	2.00 (s, 3H)	1.90 (s, 3H)	1.91 (s, 3H)	2.03 (d, 3H) $^{4}J_{H11-H20}$=1.2	1.91 (s, 3H)	2.04 (d, 3H) $^{4}J_{H11-H20}$=1.3	2.01 (s, 3H)	1.82 (s, 3H)	1.93 (s, 3H)	1.87 (s, 3H)	1.91 (d, 3H) $^{4}J_{H11-H20}$=1.5	2.06 (d, 3H) $^{4}J_{H11-H20}$=1.4	2.05 (d, 3H) $^{4}J_{H11-H20}$=1.4	2.08 (d, 3H) $^{4}J_{H11-H20}$=1.5
21	1.40 (s, 3H)	1.25 (s, 3H)	1.25 (s, 3H)	1.36 (s, 3H)	1.25 (s, 3H)	1.39 (s, 3H)	1.46 (s, 3H)	1.85 (s, 3H)	1.75 (s, 3H)	1.76 (s, 3H)	1.76 (s, 3H)	1.37 (s, 3H)	1.42 (s, 3H)	1.26 (s, 3H)
22	3.22 (s, 3H)	3.22 (s, 3H)	3.07 (s, 3H)	3.10 (s, 3H)	3.07 (s, 3H)	3.10 (s, 3H)	2.85 (s, 3H)	2.77 (s, 3H)	2.86 (s, 3H)	2.79 (s, 3H)	2.92 (s, 3H)	3.02 (bs, 3H)	3.13 (s, 3H)	3.27 (s, 3H)
23	---	---	---	---	---	---	---	---	---	---	---	---	---	---
24	---	---	----	----	----	----	---	7.06 (d, 1H) $^{3}J_{H24-H25}$=1.7	---	---	---	---	---	---
25	---	---	---	---	---	---	---	7.36 (t, 1H) $^{3}J_{H25-H26}$=1.5	---	---	---	---	---	---
26	---	---	---	---	---	---	---	8.08 (d, 1H) $^{3}J_{H26-H25}$=1.1	---	---	---	---	---	---
1’	4.35 (d, 1H) $^{3}J_{H1'-H2'}$=7.2	4.28 (d, 1H) $^{3}J_{H1'-H2'}$=7.2	4.29 (d, 1H) $^{3}J_{H1'-H2'}$=7.5	4.50 (d, 1H) $^{3}J_{H1'-H2'}$=7.6	4.44 (d, 1H) $^{3}J_{H1'-H2'}$=7.7	4.72 (m, 1H)	4.34 (d, 1H) $^{3}J_{H1'-H2'}$=7.6	4.34 (d, 1H) $^{3}J_{H1'-H2'}$=7.6	4.45 (d, 1H) $^{3}J_{H1'-H2'}$=7.6	4.44 (d, 1H) $^{3}J_{H1'-H2'}$7.7	4.41 (d, 1H) $^{3}J_{H1''-H2b''}$=7.3	4.32 (d, 1H) $^{3}J_{H1'-H2'}$=7.6	---	---
2’	3.18	2.97	3.18	3.24	4.50	4.72	4.71	4.71	4.70	4.50	3.15	4.73	---	---

H	**2 (CDCl$_3$)**	**2 (DMSO-*d$_6$*)**	**3 (DMSO-*d$_6$*)**	**3 (CDCl$_3$)**	**4 (DMSO-*d$_6$*)**	**4 (CDCl$_3$)**	**5 (CDCl$_3$)**	**6 (CDCl$_3$)**	**7 (CDCl$_3$)**	**7 (DMSO-*d$_6$*)**	**8 (CDCl$_3$)**	**9 (CDCl$_3$)**	**10a (CDCl$_3$)**	**10b (CDCl$_3$)**
	(dd, 1H)<br>$^{3}J_{H2'-H3'}$=10.3<br>$^{3}J_{H2'-H1'}$=7.2	(ddd, 1H)<br>$^{3}J_{H2'-H3'}$=10.1<br>$^{3}J_{H2'-H1'}$=7.3<br>$^{3}J_{H2'-OH}$=2.4	(m, 1H)	(dd, 1H)<br>$^{3}J_{H2'-H3'}$=10.3<br>$^{3}J_{H2'-H1'}$=7.6	(dd, 1H)<br>$^{3}J_{H2'-H3'}$=10.5<br>$^{3}J_{H2'-H1'}$=7.6	(m, 1H)	(dd, 1H)<br>$^{3}J_{H2'-H3'}$=10.5<br>$^{3}J_{H2'-H1'}$=7.6	(dd, 1H)<br>$^{3}J_{H2'-H3'}$=10.5<br>$^{3}J_{H2'-H1'}$=7.6	(dd, 1H)<br>$^{3}J_{H2'-H3'}$=10.5<br>$^{3}J_{H2'-H1'}$=7.6	(dd, 1H)<br>$^{3}J_{H2'-H3'}$=10.5<br>$^{3}J_{H2'-H1'}$=7.6	(dd, 1H)<br>$^{3}J_{H2'-H3'}$=10.2<br>$^{3}J_{H2'-H1'}$=7.3	(dd, 1H)<br>$^{3}J_{H2'-H3'}$=10.7<br>$^{3}J_{H2'-H1'}$=7.5		
3'	2.42<br>(ddd, 1H)<br>$^{3}J_{H2'-H3'}$=10.3<br>$^{3}J_{H2'-H1'}$=7.2	2.41<br>(ddd, 1H)<br>$^{3}J_{H3'-H4'a}$=12.1<br>$^{3}J_{H3'-H2'}$=10.0<br>$^{3}J_{H3'-H4'b}$=4.0	2.44<br>(ddd, 1H)<br>$^{3}J_{H3'-H4'a}$=12.1<br>$^{3}J_{H3'-H2'}$=10.0<br>$^{3}J_{H3'-H4'b}$=4.1	2.51<br>(m, 1H)	2.75<br>(ddd, 1H)<br>$^{3}J_{H3'-H4'a}$=12.1<br>$^{3}J_{H3'-H2'}$=10.5<br>$^{3}J_{H3'-H4'b}$=4.3	2.64<br>(m, 1H)	2.64<br>(m, 1H)	2.63<br>(m, 1H)	2.65<br>(m, 1H)	2.75<br>(ddd, 1H)<br>$^{3}J_{H3'-H4'a}$=12.1<br>$^{3}J_{H3'-H2'}$=10.5<br>$^{3}J_{H3'-H4'b}$=4.3	2.48<br>(ddd, 1H)<br>$^{3}J_{H3'-H4'a}$=12.3<br>$^{3}J_{H3'-H2'}$=10.1<br>$^{3}J_{H3'-H4'b}$=4.0	2.69<br>(m, 1H)	---	---
4'	1.63<br>(m, 1H)<br><br>1.18<br>(m, 1H)	1.59<br>(m, 1H)<br><br>1.06<br>(m, 1H)	1.61<br>(m, 1H)<br><br>1.11<br>(d, 1H)<br>^{2}J=13.0	1.63<br>(m, 1H)<br><br>1.32<br>(m, 1H)	1.75<br>(m, 1H)<br><br>1.19<br>(m, 1H)	1.73<br>(ddd, 1H)<br>^{2}J=12.9<br>$^{3}J_{H3'-H4'a}$=4.4<br>$^{3}J_{H4a'-H5'}$=2.0<br><br>1.36<br>(m, 1H)	1.71<br>(m, 1H)<br><br>1.29<br>(m, 1H)	1.70<br>(m, 1H)<br><br>1.31<br>(m, 1H)	1.70<br>(m, 1H)<br><br>1.29<br>(m, 1H)	1.75<br>(m, 1H)<br><br>1.19<br>(m, 1H)	1.65<br>(m, 1H)<br><br>1.22<br>(m, 1H)	1.73<br>(m, 1H)<br><br>1.31<br>(m, 1H)	---	---
5'	3.46<br>(dqd, 1H)<br>$^{3}J_{H5'-H4'b}$=10.2	3.58<br>(m, 1H)	3.42<br>(m, 1H)	3.54<br>(dqd, 1H)<br>$^{3}J_{H5'-H4'b}$=12.5	3.59<br>(dqd, 1H)<br>$^{3}J_{H5'-H4'b}$=12.4	3.51<br>(dqd, 1H)<br>$^{3}J_{H5'-H4'b}$=11.0	3.52<br>(m, 1H)	3.48<br>(m, 1H)	3.51<br>(m, 1H)	3.59<br>(dqd, 1H)<br>$^{3}J_{H5'-H4'b}$=12.4	3.54<br>(dqd, 1H)<br>$^{3}J_{H5'-H4'b}$=12.3	3.59<br>(m, 1H)	---	---

H	**2 (CDCl$_3$)**	**2 (DMSO-*d$_6$*)**	**3 (DMSO-*d$_6$*)**	**3 (CDCl$_3$)**	**4 (DMSO-*d$_6$*)**	**4 (CDCl$_3$)**	**5 (CDCl$_3$)**	**6 (CDCl$_3$)**	**7 (CDCl$_3$)**	**7 (DMSO-*d$_6$*)**	**8 (CDCl$_3$)**	**9 (CDCl$_3$)**	**10a (CDCl$_3$)**	**10b (CDCl$_3$)**
	$^{3}J_{H5'-H6'}$=6.0<br>$^{3}J_{H5'-H4'a}$=4.8			$^{3}J_{H5'-H6'}$=6.2<br>$^{3}J_{H5'-H4'a}$=2.1	$^{3}J_{H5'-H6'}$=6.1<br>$^{3}J_{H5'-H4'a}$=1.8	$^{3}J_{H5'-H6'}$=6.2<br>$^{3}J_{H5'-H4'a}$=2.1				$^{3}J_{H5'-H6'}$=6.1<br>$^{3}J_{H5'-H4'a}$=1.8	$^{3}J_{H5'-H6'}$=6.1<br>$^{3}J_{H5'-H4'a}$=2.1			
6’	1.21<br>(d, 3H)<br>$^{3}J_{H6'-H5'}$=6.1	1.09<br>(d. 3H)<br>$^{3}J_{H6'-H5'}$=5.9	1.13<br>(d. 3H)<br>$^{3}J_{H6'-H5'}$=6.2	1.26<br>(d. 3H)<br>$^{3}J_{H6'-H5'}$=6.2	1.17<br>(d. 3H)<br>$^{3}J_{H6'-H5'}$=6.1	1.26<br>(d. 3H)<br>$^{3}J_{H6'-H5'}$=6.2	1.23<br>(d. 3H)<br>$^{3}J_{H6'-H5'}$=6.1	1.23<br>(d. 3H)<br>$^{3}J_{H6'-H5'}$=6.4	1.20<br>(d. 3H)<br>$^{3}J_{H6'-H5'}$=6.1	1.17<br>(d. 3H)<br>$^{3}J_{H6'-H5'}$=6.1	1.21<br>(d. 3H)<br>$^{3}J_{H6'-H5'}$=6.1	1.24<br>(d. 3H)<br>$^{3}J_{H6'-H5'}$=6.1	---	---
7’	2.26<br>(s, 3H)	2.19<br>(s, 3H)	2.21<br>(s, 3H)	2.26<br>(s, 3H)	2.14<br>(s, 3H)	2.24<br>(s, 3H)	2.23<br>(s, 3H)	2.22<br>(s, 3H)	2.23<br>(s, 3H)	2.14<br>(s, 3H)	2.26<br>(s, 3H)	2.26<br>(s, 3H)	---	---
8’	2.26<br>(s, 3H)	2.19<br>(s, 3H)	2.21<br>(s, 3H)	2.26<br>(s, 3H)	2.14<br>(s, 3H)	2.24<br>(s, 3H)	2.23<br>(s, 3H)	2.22<br>(s, 3H)	2.23<br>(s, 3H)	2.14<br>(s, 3H)	2.26<br>(s, 3H)	2.26<br>(s, 3H)	---	---
9’	---	---	---	---	---		---	---	---	---	---	---	---	---
10’	---	---	---	---	1.96<br>(s, 3H)	2.11<br>(s, 3H)	2.03<br>(s, 3H)	2.03<br>(s, 3H)	2.04<br>(s, 3H)	1.96<br>(s, 3H)	---	2.05<br>(s, 3H)	---	---
11’	---	---	---	---	---	---	---	---	---	---	---	---	---	---
12’	---	---	---	---	---	---	---	---	---	---	---	---	---	---
1”	4.85<br>(d, 1H)<br>$^{3}J_{H1''-H2b''}$=4.8	4.82<br>(d, 1H)<br>$^{3}J_{H1''-H2b''}$=4.8	---	---	---	---	---	---	---	---	---	---	---	---
2”	2.36<br>(d, 1H)<br>^{2}J=15.2<br><br>1.57<br>(dd, 1H)<br>^{2}J=15.2<br>$^{3}J_{H2''-H1''}$=4.9	2.30<br>(d, 1H)<br>^{2}J=15.1<br><br>1.53<br>(dd, 1H)<br>^{2}J=15.0<br>$^{3}J_{H2''-H1''}$=4.9	---	---	---	---	---	---	---	---	---	---	---	---
3”	---	---	---	---	---	---	---	---	---	---	---	---	---	---

H	2 (CDCl3)	2 (DMSO-*d6*)	3 (DMSO-*d6*)	3 (CDCl3)	4 (DMSO-*d6*)	4 (CDCl3)	5 (CDCl3)	6 (CDCl3)	7 (CDCl3)	7 (DMSO-*d6*)	8 (CDCl3)	9 (CDCl3)	10a (CDCl3)	10b (CDCl3)
4”	3.00 (d, 1H) ${}^3J_{H4”-H5”}$=9.2	2.89 (t, 1H) ${}^3J_{H4”-H5”}$=8.2	---	---	---	---	---	---	---	---	---	---	---	---
5”	4.02 (dd, 1H) ${}^3J_{H5”-H4”}$=9.3 ${}^3J_{H5”-H6”}$=6.2	4.05 (m, 1H)	---	---	---	---	---	---	---	---	---	---	---	---
6”	1.28 (d, 3H) ${}^3J_{H6”-H5”}$=6.2	1.18 (d, 3H) ${}^3J_{H6”-H5”}$=6.4	---	---	---	---	---	---	---	---	---	---	---	---
7”	1.23 (s, 3H)	1.13 (s, 3H)	---	---	---	---	---	---	---	---			---	
8”	3.30 (s, 3H)	3.20 (s, 3H)	---	---	---	---	---	---	---	---	---	---	---	---
3-OH	---	---	5.08 (d, 1H) ${}^3J_{H3-OH}$=6.5	*	5.09 (d, 1H) ${}^3J_{H3-OH}$=6.5	*	---	---	---	---	---	---	---	---
11-OH	---	---	---	---	---	---	---	---	---	---	---	---	---	---
12-OH	*	5.18 (s, 1H)	5.19 (s, 1H)	*	5.21 (s, 1H)	*	*	---	---	---	---	*	*	*
2’-OH	*	4.08 (m, 1H)	4.34 (m, 1H)	4.59 (bs, 1H)	---	---	---	---	---	---	*	---	---	---
4”-OH	*	4.35 (d, 1H)	---	---	---	---	---	---	---	---	---	---	---	---

H	2 (CDCl3)	2 (DMSO-*d6*)	3 (DMSO-*d6*)	3 (CDCl3)	4 (DMSO-*d6*)	4 (CDCl3)	5 (CDCl3)	6 (CDCl3)	7 (CDCl3)	7 (DMSO-*d6*)	8 (CDCl3)	9 (CDCl3)	10a (CDCl3)	10b (CDCl3)
		$^3J_{H4''-OH}$=8.2												

*- signal not observed

Table S2. $^{13}C\{^{1}H\}$ NMR data of all synthesized new clarithromycins **2** – **10**, assigned on the basis of ^{1}H-^{13}C HSQC, ^{1}H-^{13}C HMBC, ^{1}H-^{1}H COSY and ^{1}H-^{1}H NOESY spectra.

**C**	**2 ($CDCl_3$)**	**2 (DMSO-*d*$_6$)**	**3 (DMSO-*d*$_6$)**	**3 ($CDCl_3$)**	**4 (DMSO-*d*$_6$)**	**4 ($CDCl_3$)**	**5 ($CDCl_3$)**	**6 ($CDCl_3$)**	**7 ($CDCl_3$)**	**7 (DMSO-*d*$_6$)**	**8 ($CDCl_3$)**	**9 ($CDCl_3$)**	**10a ($CDCl_3$)**	**10b ($CDCl_3$)**
1	175.3	174.5	175.3	176.7	175.3	175.8	169.9	169.0	165.0	164.6	164.6	169.9	172.1	170.6
2	45.2	44.9	44.1	44.3	44.1	44.3	51.3	51.2	89.2	87.4	89.4	50.8	46.7	48.7
3	79.3	79.1	75.1	77.7	75.1	77.2	204.6	204.1	200.8	195.7	200.5	205.2	1961	194.8
4	40.5	39.2	38.0	38.3	38.5	37.7	47.3	47.5	42.8	41.9	42.9	48.0	140.5	137.9
5	80.0	79.3	81.7	92.0	81.8	87.8	81.1	81.0	84.5	83.6	84.7	88.5	142.9	148.9
6	78.7	77.3	77.9	80.9	77.9	80.0	78.5	78.7	78.6	77.7	78.4	79.1	78.3	78.6
7	40.5	40.6	38.9	36.9	38.9	37.5	40.3	40.4	39.9	39.8	40.6	38.6	45.5	43.1
8	37.5	34.9	35.2	36.4	35.2	37.2	38.6	39.1	37.9	36.4	37.0	34.0	35.2	35.4
9	207.5	206.1	206.4	207.7	206.4	207.6	207.2	205.1	208.4	207.0	208.0	206.3	206.9	207.0
10	138.9	136.7	136.9	138.7	136.9	139.6	139.0	138.3	136.6	134.9	135.6	139.1	139.4	138.9
11	142.5	144.8	143.8	141.5	143.8	140.8	142.1	138.6	136.9	137.5	137.7	144.5	142.4	141.8
12	73.5	71.6	71.7	73.5	71.7	73.4	73.6	84.7	87.1	87.6	87.1	75.2	73.8	73.7
13	80.3	78.8	78.7	79.2	78.8	79.4	81.5	77.2	84.1	83.8	83.8	83.3	80.6	79.5
14	22.3	21.0	21.0	20.8	21.0	21.3	22.6	22.8	23.9	22.9	23.8	21.9	21.2	21.3
15	10.8	10.5	10.4	10.5	10.5	10.6	11.1	10.6	10.3	9.8	10.1	10.7	10.6	10.5
16	15.7	16.2	15.9	15.8	15.9	15.5	14.9	15.2	11.1	11.1	11.1	15.7	14.1	13.7
17	9.7	9.3	8.4	7.6	8.4	8.0	14.1	14.1	14.9	14.5	15.2	19.1	12.3	12.9
18	22.4	23.7	22.7	20.2	22.6	20.3	21.1	21.1	22.6	22.6	22.8	22.8	24.9	23.5
19	18.8	17.1	17.2	16.2	17.2	17.1	19.1	19.0	17.6	17.1	17.2	19.9	19.5	19.9
20	13.4	12.5	12.7	12.9	12.7	13.5	13.7	13.4	13.6	12.8	13.1	12.7	12.6	12.8
21	21.0	19.6	19.7	20.4	19.7	20.8	22.1	20.3	19.9	18.8	19.7	23.4	20.4	21.8
22	50.8	50.6	49.5	48.2	49.5	48.9	50.5	50.4	51.6	51.3	51.8	50.8	50.0	49.8
23	---	---	---	---	---	---	---	146.1	---	---	---	---	---	---
24	---	---	---	---	---	---	---	131.0	---	---	---	---	---	---
25	---	---	---	---	---	---	---	117.2	---	---	---	---	---	---
26	---	---	---	---	---	---	---	137.2	---	---	---	---	---	---
1'	103.2	102.7	103.3	106.8	101.3	102.6	101.9	102.1	102.2	101.3	104.0	101.1	---	---

C	**2 (CDCl$_3$)**	**2 (DMSO-*d$_6$*)**	**3 (DMSO-*d$_6$*)**	**3 (CDCl$_3$)**	**4 (DMSO-*d$_6$*)**	**4 (CDCl$_3$)**	**5 (CDCl$_3$)**	**6 (CDCl$_3$)**	**7 (CDCl$_3$)**	**7 (DMSO-*d$_6$*)**	**8 (CDCl$_3$)**	**9 (CDCl$_3$)**	**10a (CDCl$_3$)**	**10b (CDCl$_3$)**
2’	71.0	70.8	70.5	70.5	62.2	71.9	71.7	71.7	71.9	62.2	70.6	71.5	---	---
3’	65.6	64.3	64.5	65.6	71.4	64.1	63.7	63.7	63.7	71.4	65.8	63.5	---	---
4’	28.8	29.9	30.6	28.3	29.8	30.2	30.5	30.4	30.5	29.8	28.3	30.8	---	---
5’	69.0	67.3	68.2	69.7	68.3	69.2	69.2	69.3	69.0	68.3	69.3	69.0	---	---
6’	21.6	21.4	21.2	21.4	20.8	21.2	21.1	21.1	21.1	20.8	21.2	21.2	---	---
7’	40.4	40.2	40.4	40.3	40.2	40.7	40.8	40.8	40.8	40.2	40.2	40.8	---	---
8’	40.4	40.2	40.4	40.3	40.2	40.7	40.8	40.8	40.8	40.2	40.2	40.8	---	---
9’	---	---	---	---	169.1	170.2	169.9	169.9	170.2	169.1	---	169.9	---	---
10’	---	---	---	---	20.9	21.4	21.5	21.5	21.6	20.9	---	21.5	---	---
1”	96.5	96.8	---	---	---	---	---	---	---	---	---	---	---	---
2”	35.2	35.1	---	---	---	---	---	---	---	---	---	---	---	---
3”	72.7	72.5	---	---	---	---	---	---	---	---	---	---	---	---
4”	78.1	77.4	---	---	---	---	---	---	---	---	---	---	---	---
5”	65.8	65.0	---	---	---	---	---	---	---	---	---	---	---	---
6”	18.7	18.7	---	---	---	---	---	---	---	---	---	---	---	---
7”	21.7	20.8	---	---	---	---	---	---	---	---	---	---	---	---
8”	49.6	48.9	---	---	---	---	---	---	---	---	---	---	---	---

4. **Copy of ^{1}H-NMR, ^{13}C{^{1}H}-NMR and FT-IR spectra of 2 – 10 and ^{1}H-^{1}H COSY, ^{1}H-^{13}C HSQC, ^{1}H-^{13}C HMBC for 8-10 and ^{1}H-^{1}H NOESY for 9 and 10.**

^{1}H-NMR (500 MHz, $CDCl_3$) spectrum of compound **2**.

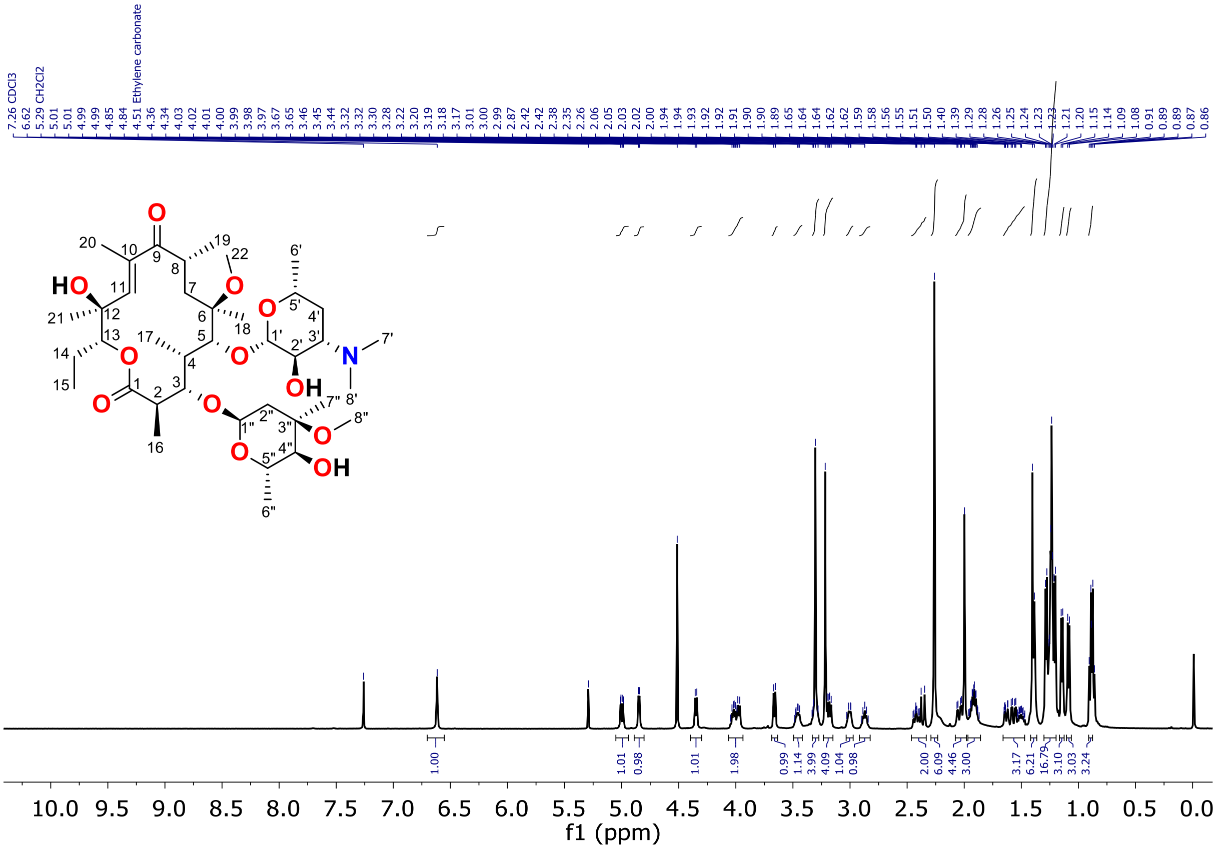

^{13}C{1H}-NMR (126 MHz, $CDCl_3$) spectrum of compound **2**.

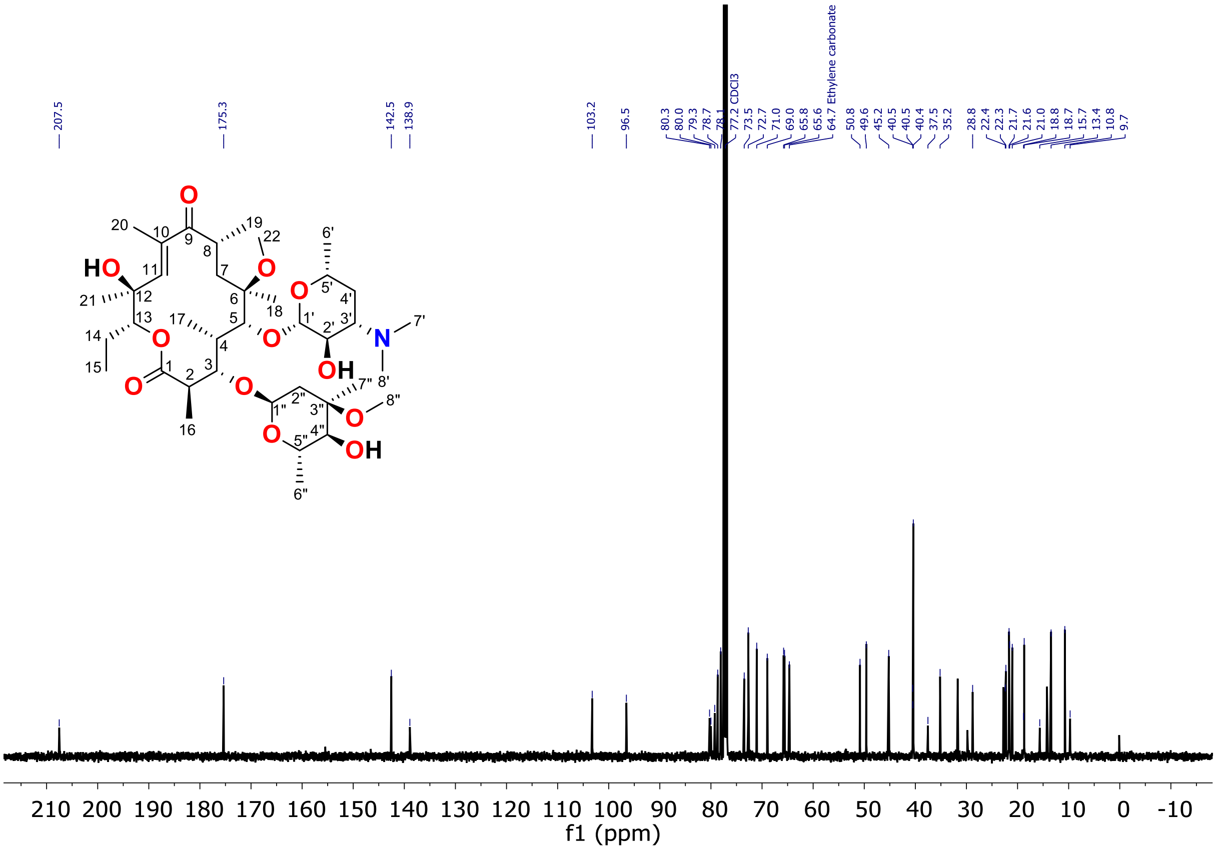

FT-IR spectrum of compound **2** in ATR technique.

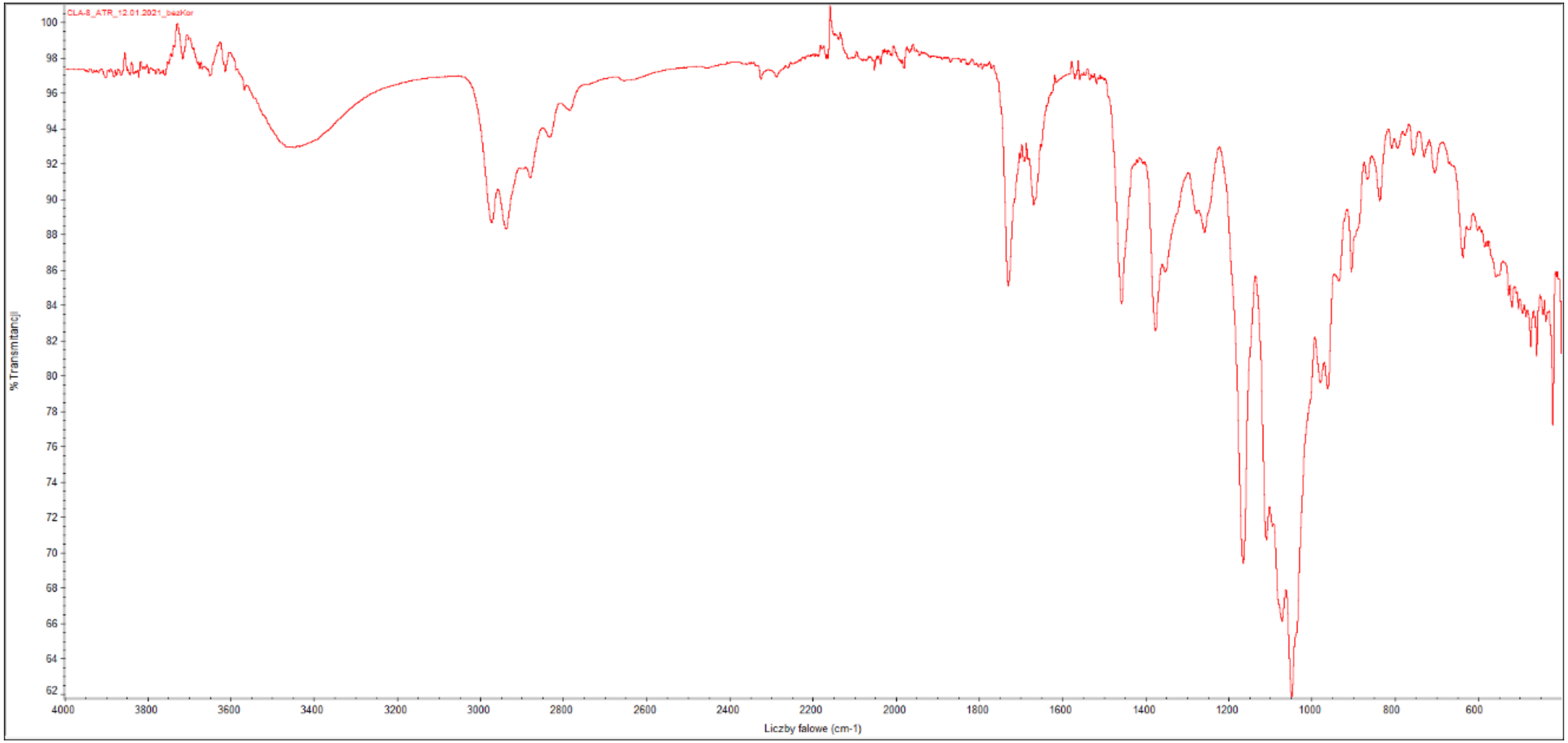

^{1}H-NMR (500 MHz, $CDCl_3$) spectrum of compound **3**.

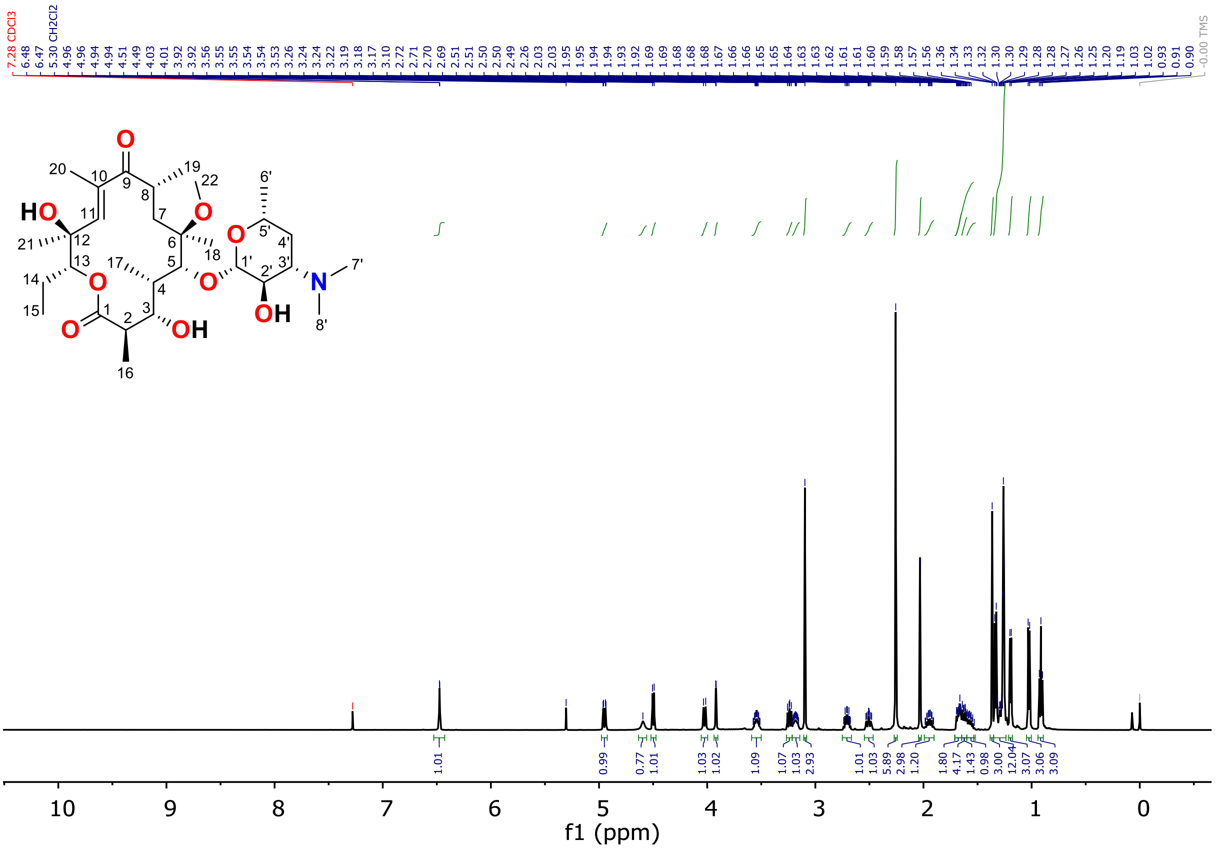

$^{13}C\{1H\}$-NMR (126 MHz, $CDCl_3$) spectrum of compound **3**.

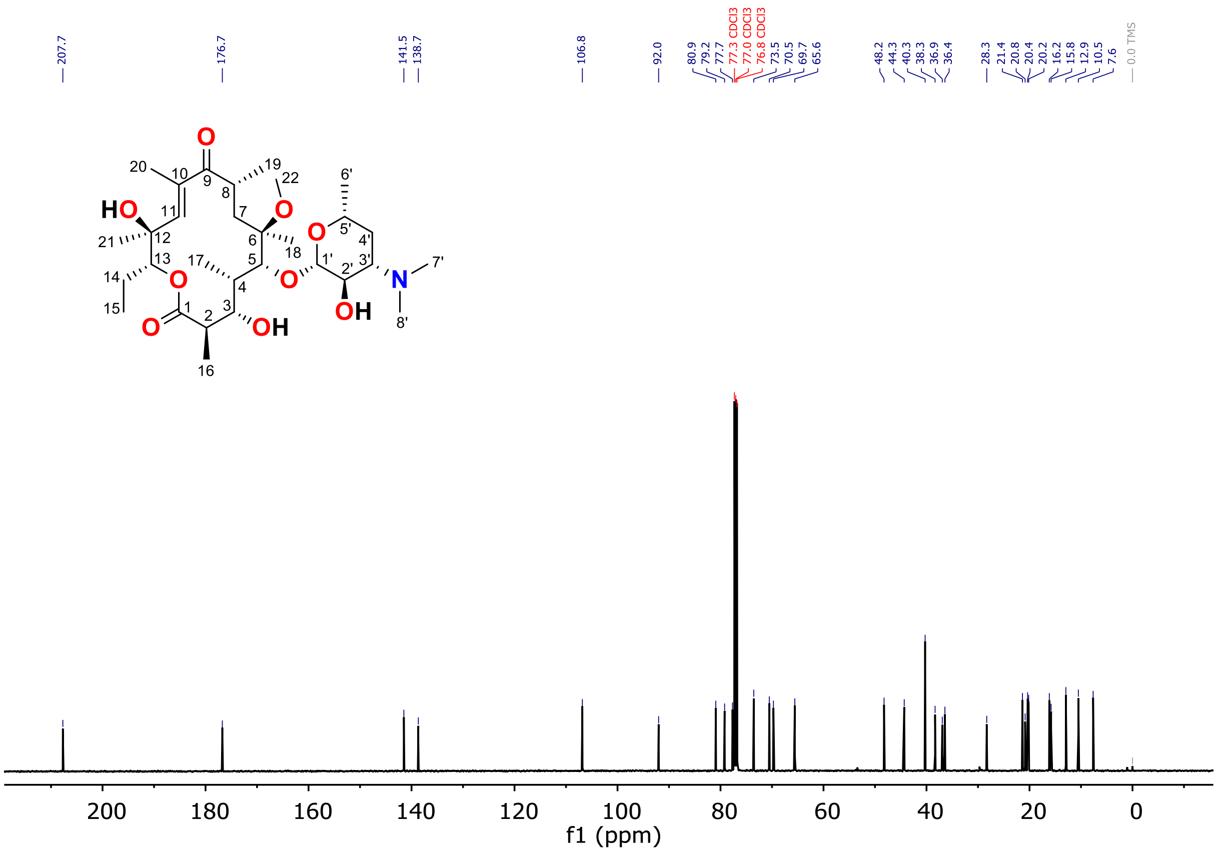

FT-IR spectrum of compound **3** in ATR technique.

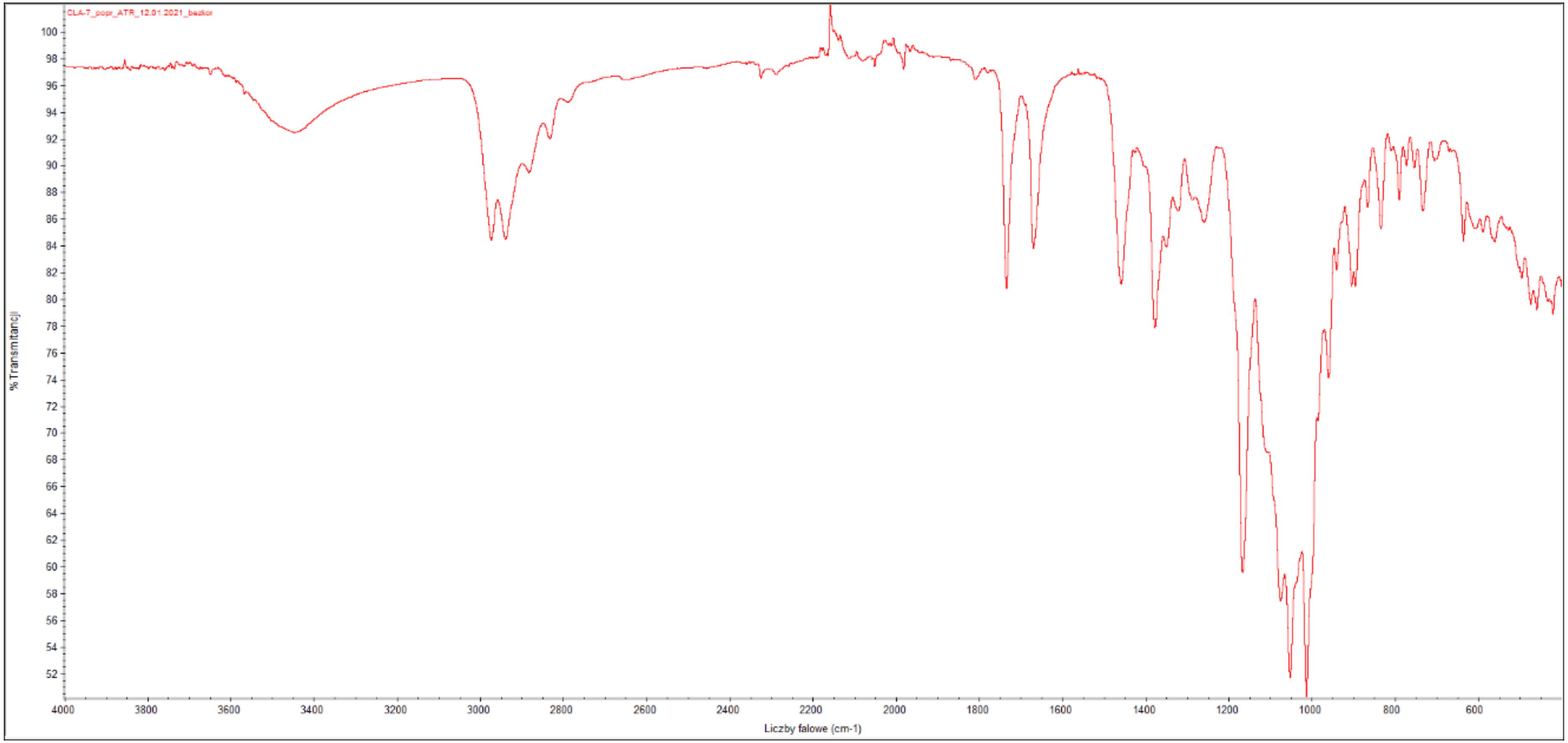

^{1}H-NMR (600 MHz, $CDCl_3$) spectrum of compound **4**.

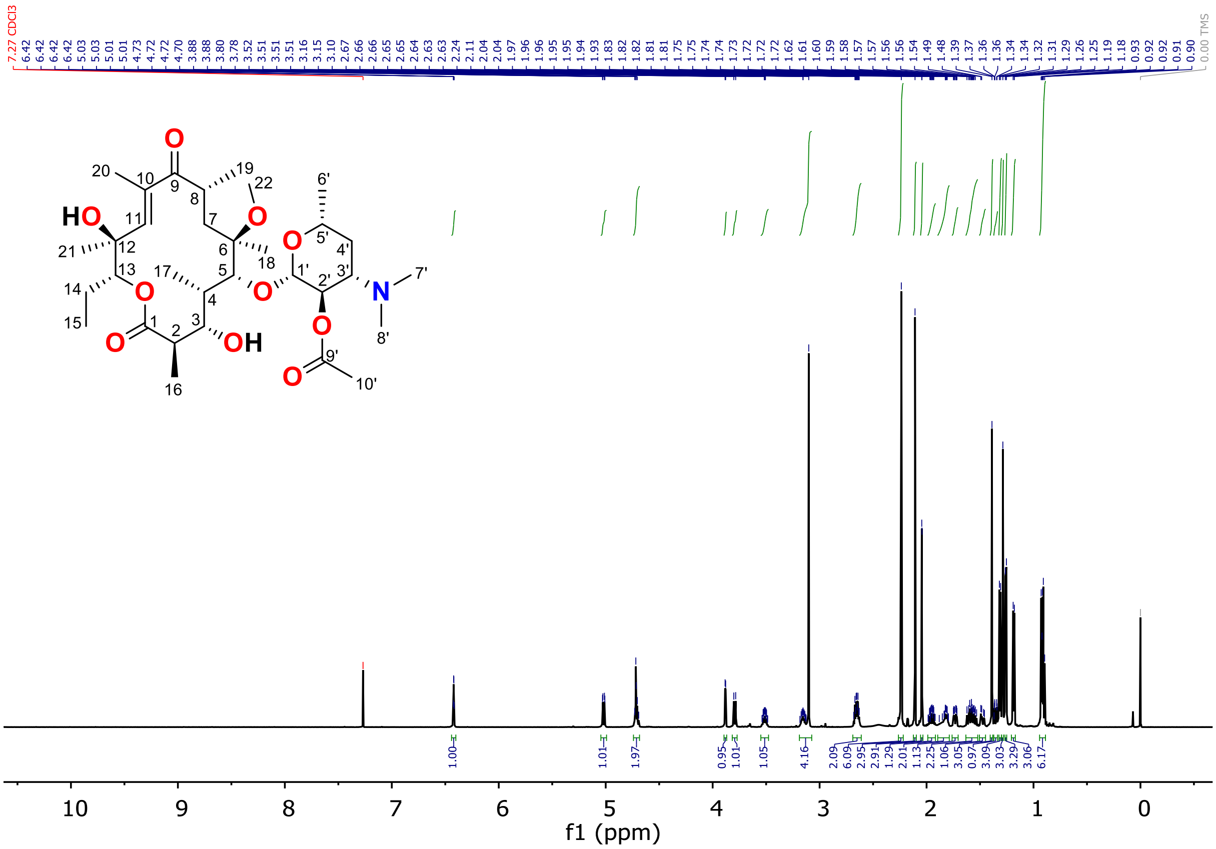

$^{13}C\{1H\}$-NMR (151 MHz, $CDCl_3$) spectrum of compound **4**.

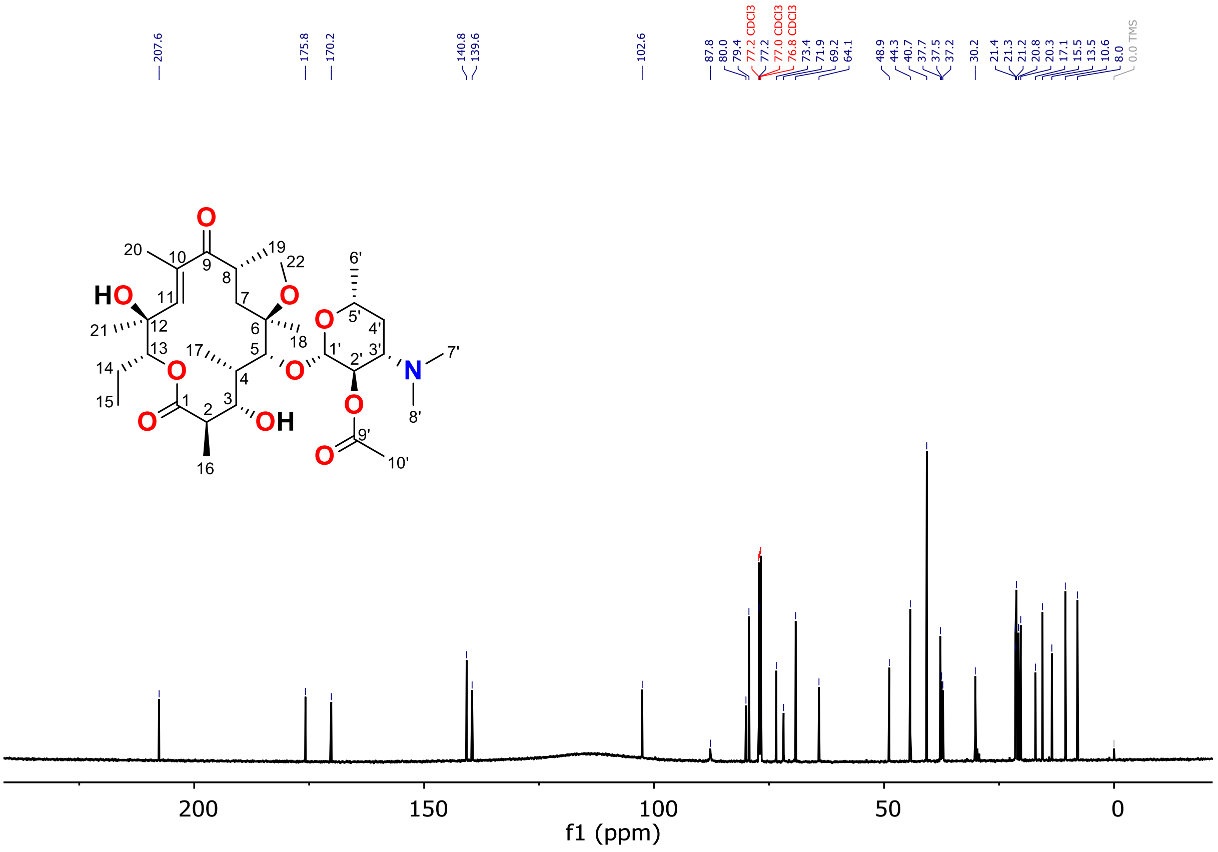

FT-IR spectrum of compound **4** in ATR technique.

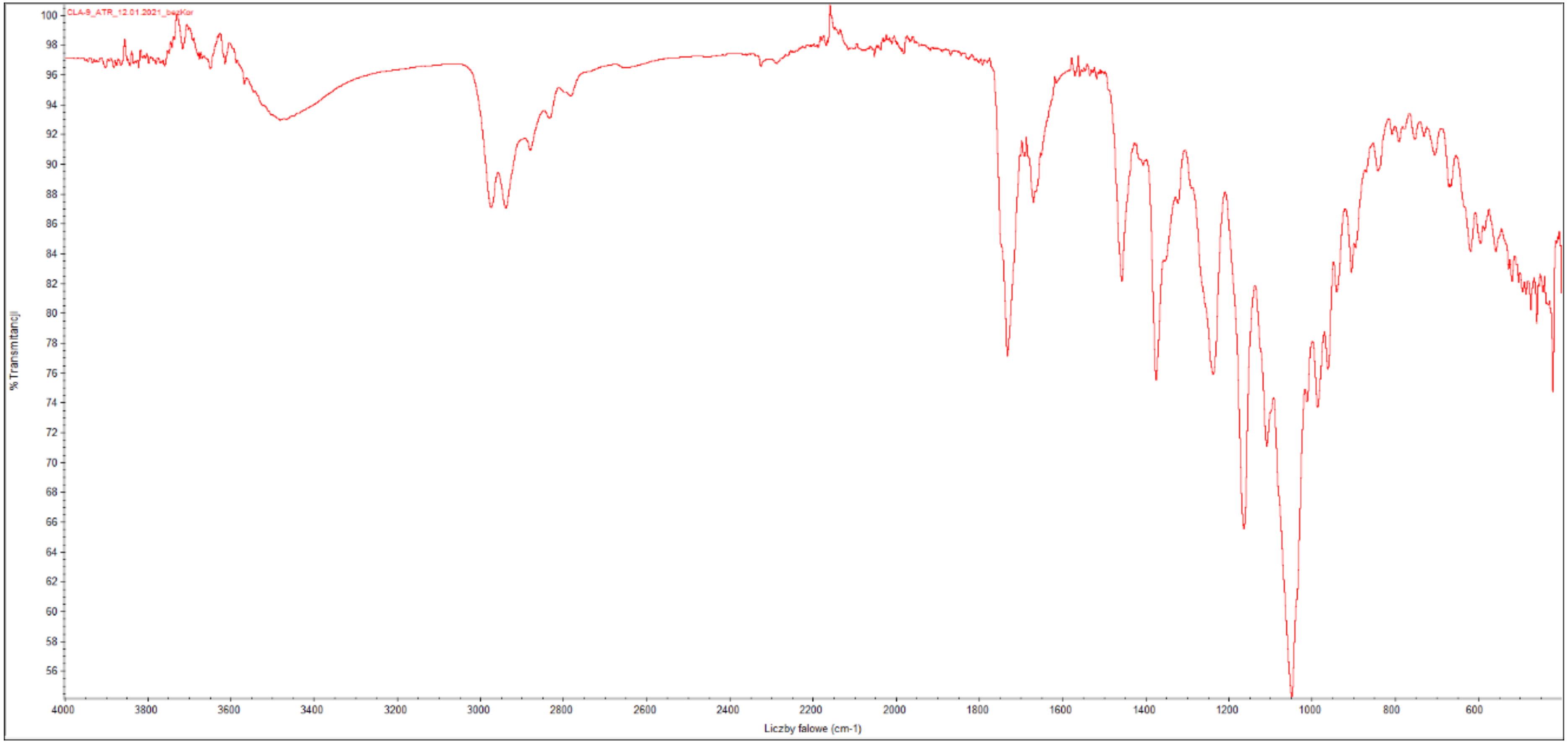

^{1}H-NMR (500 MHz, $CDCl_3$) spectrum of compound **5**.

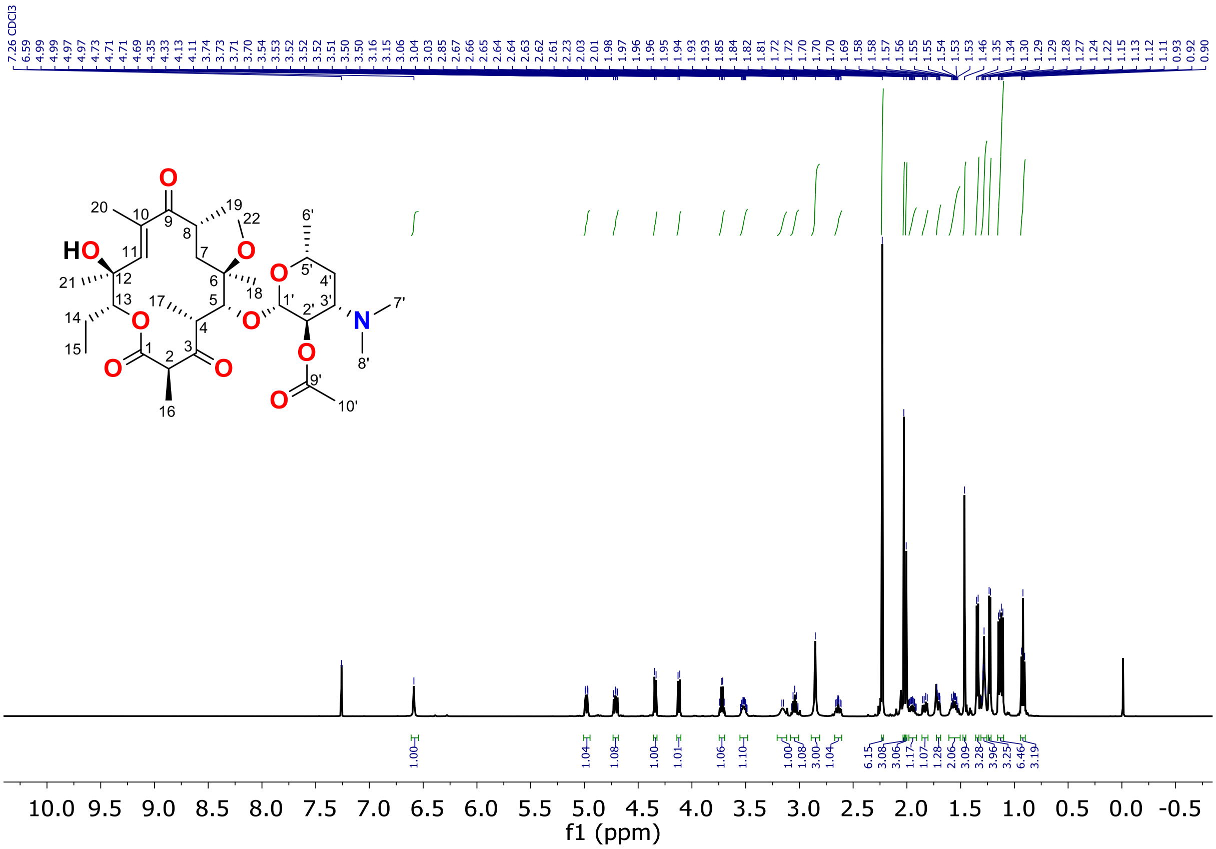

$^{13}C\{1H\}$-NMR (126 MHz, $CDCl_3$) spectrum of compound **5**.

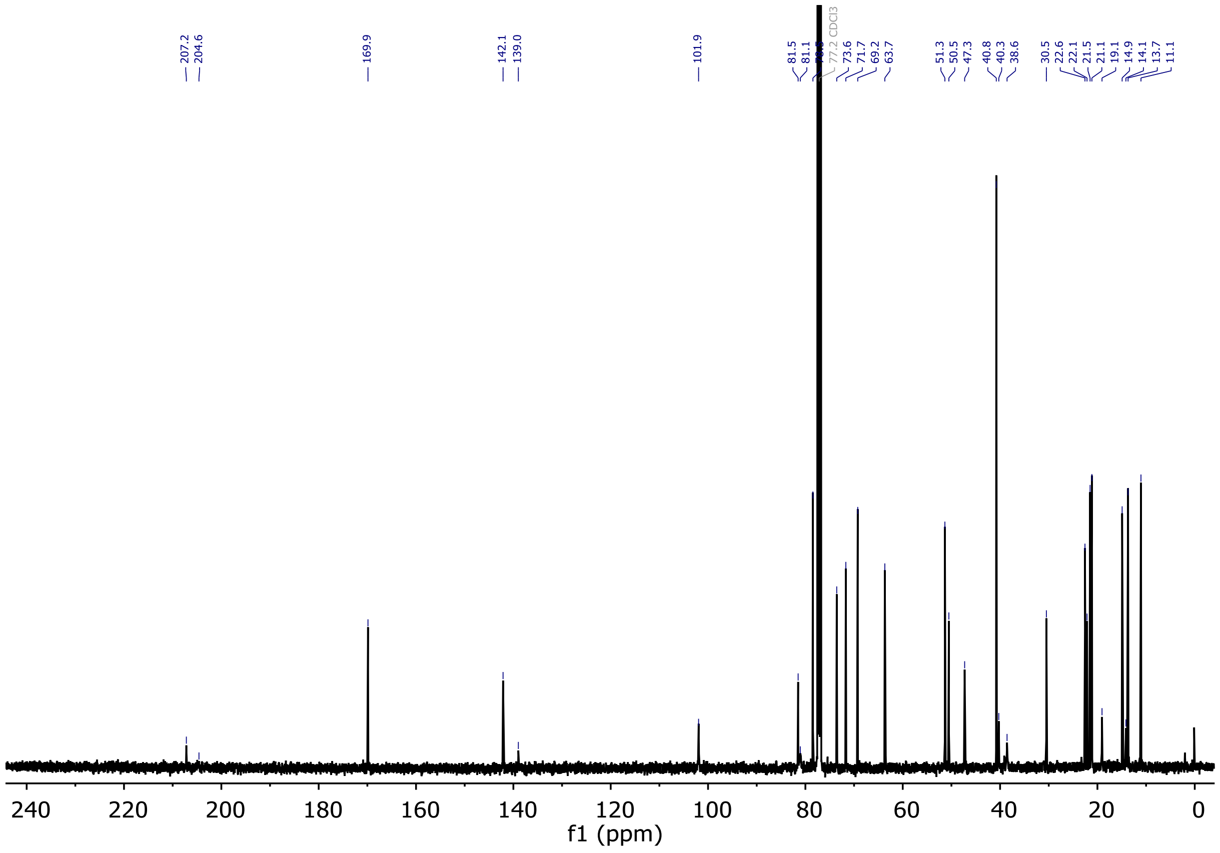

FT-IR spectrum of compound **5** in ATR technique.

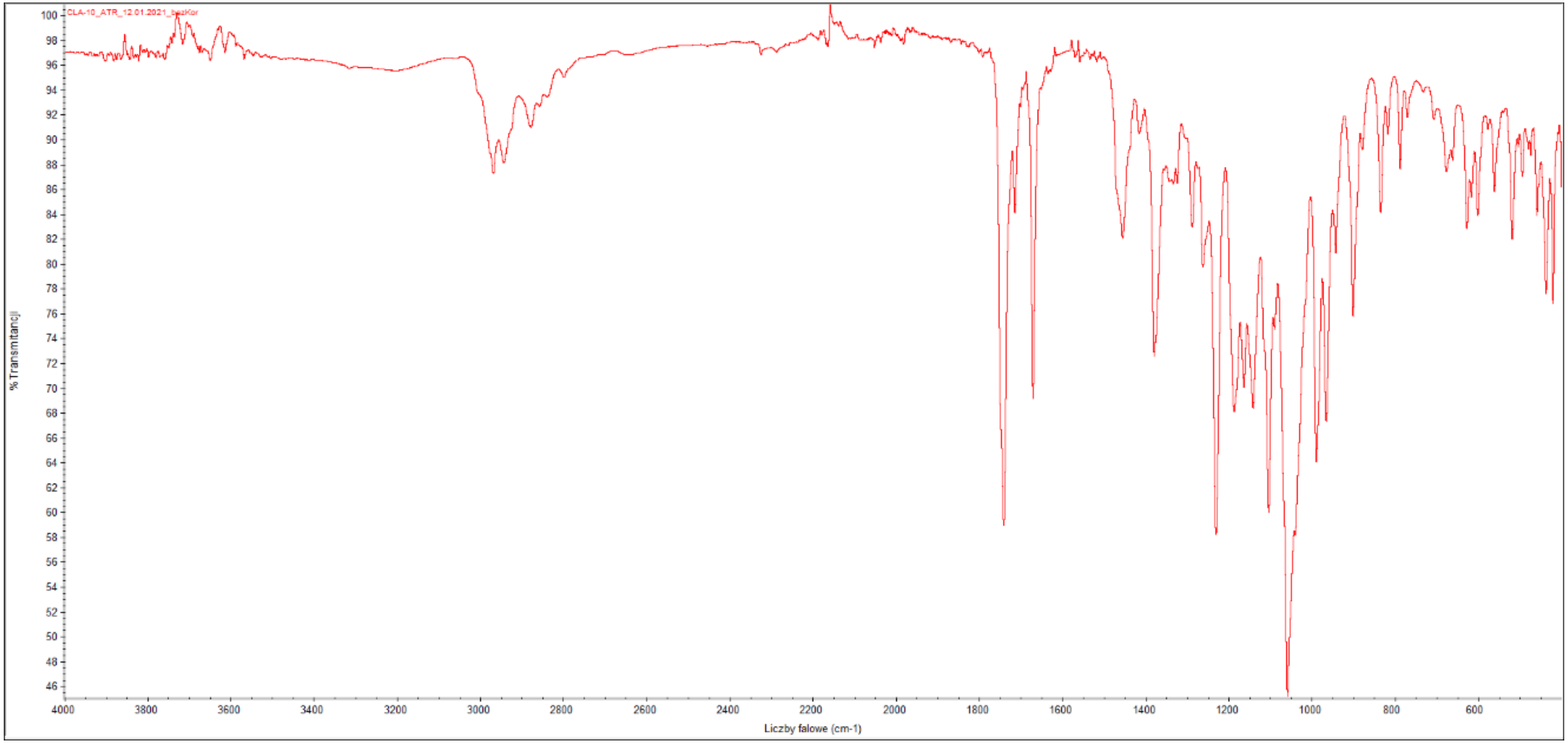

^{1}H-NMR (500 MHz, $CDCl_3$) spectrum of compound **6**.

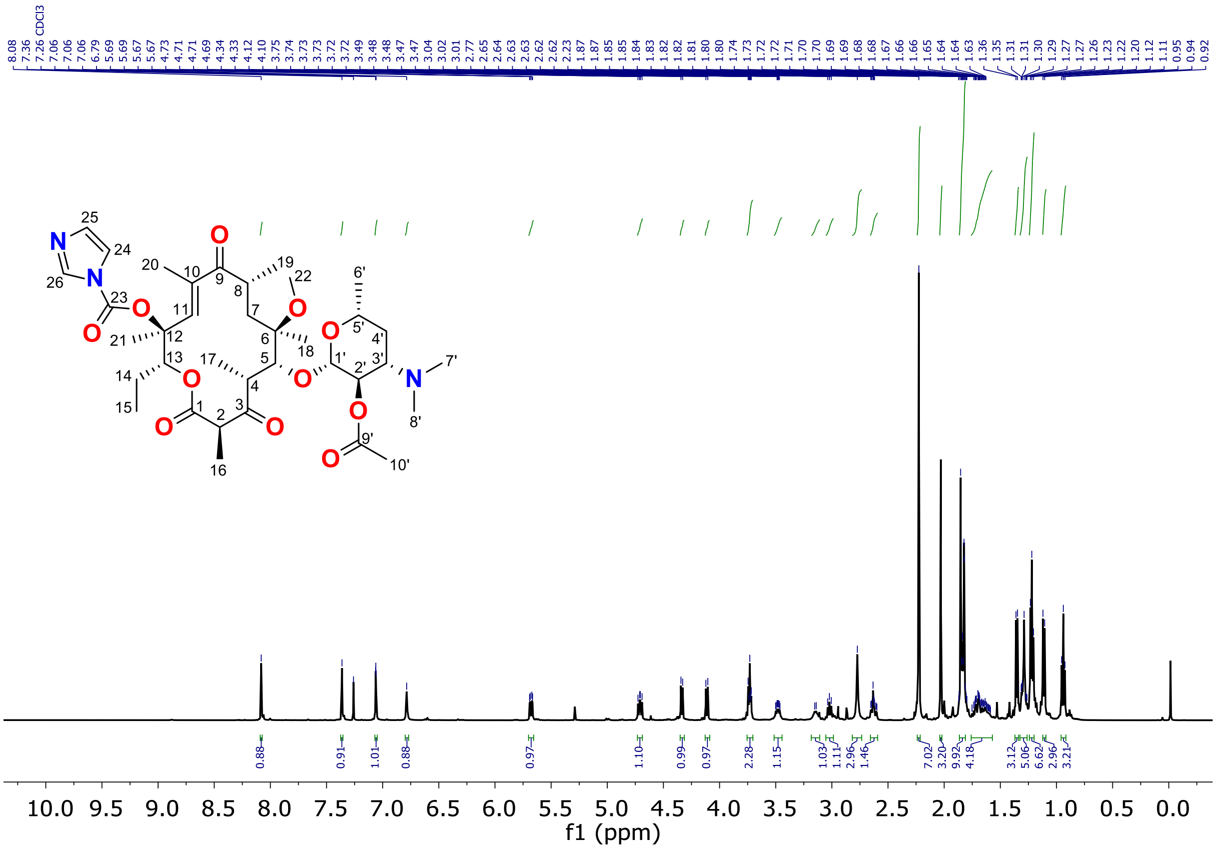

$^{13}C\{1H\}$-NMR (126 MHz, $CDCl_3$) spectrum of compound **6**.

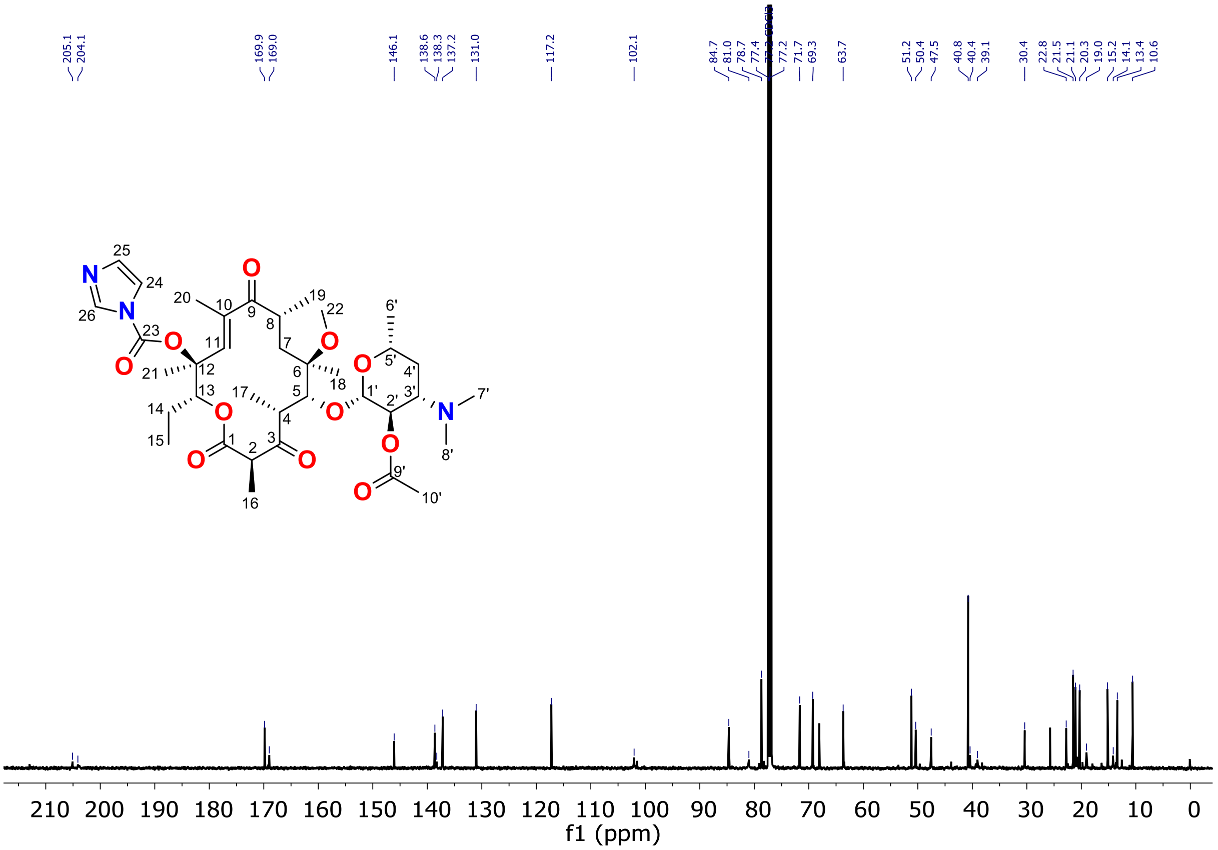

FT-IR spectrum of compound **6** in ATR technique.

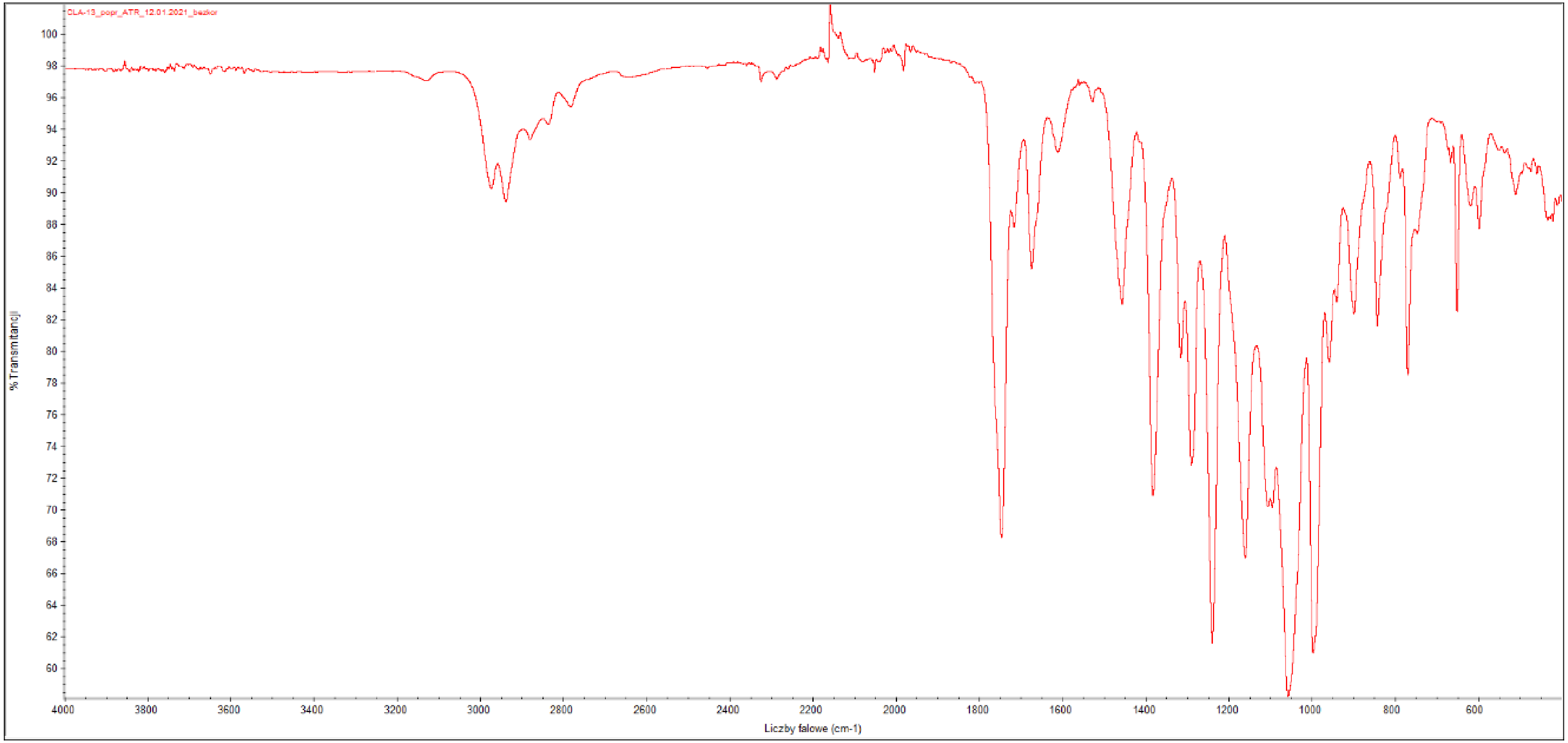

^{1}H-NMR (500 MHz, $CDCl_3$) spectrum of compound **7**.

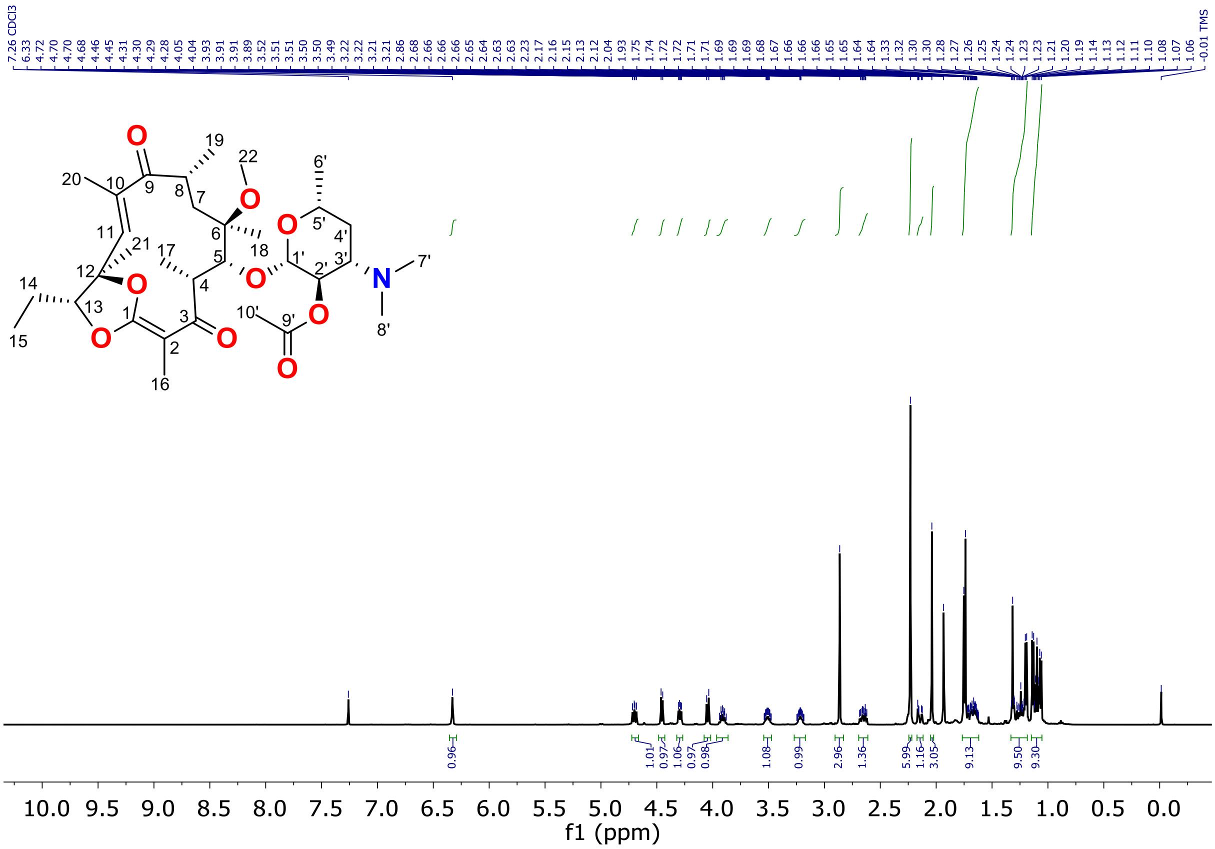

$^{13}C\{1H\}$-NMR (126 MHz, $CDCl_3$) spectrum of compound **7**.

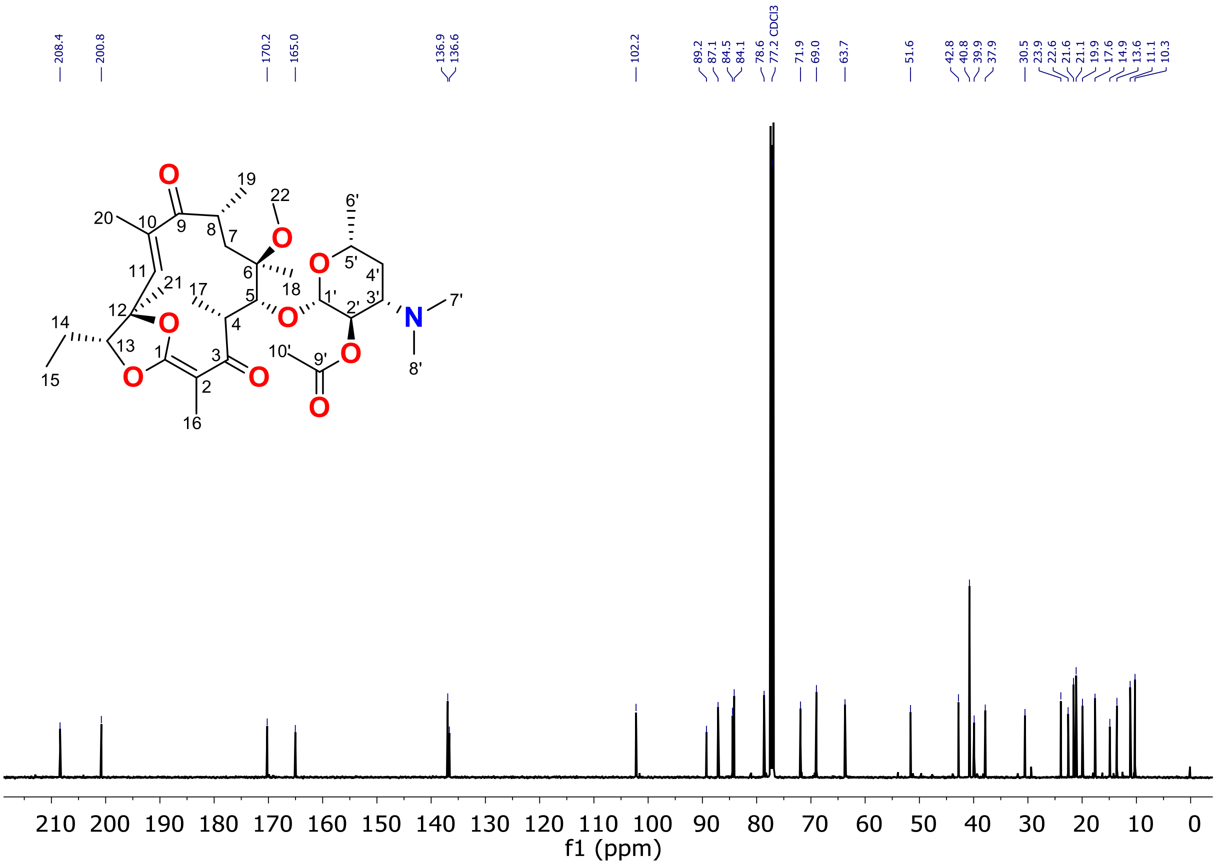

FT-IR spectrum of compound **7** in ATR technique.

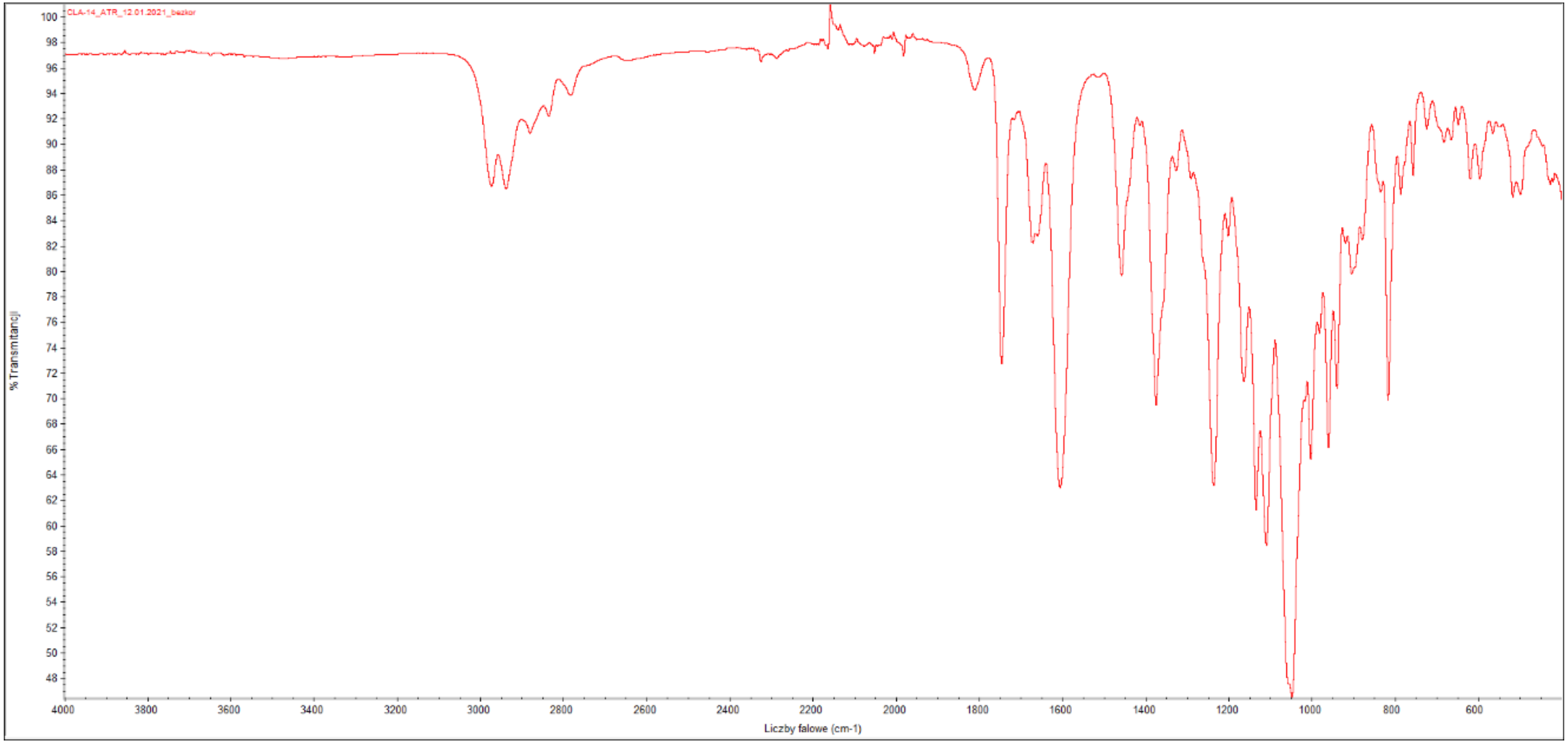

^{1}H-NMR (500 MHz, $CDCl_3$) spectrum of compound **8**.

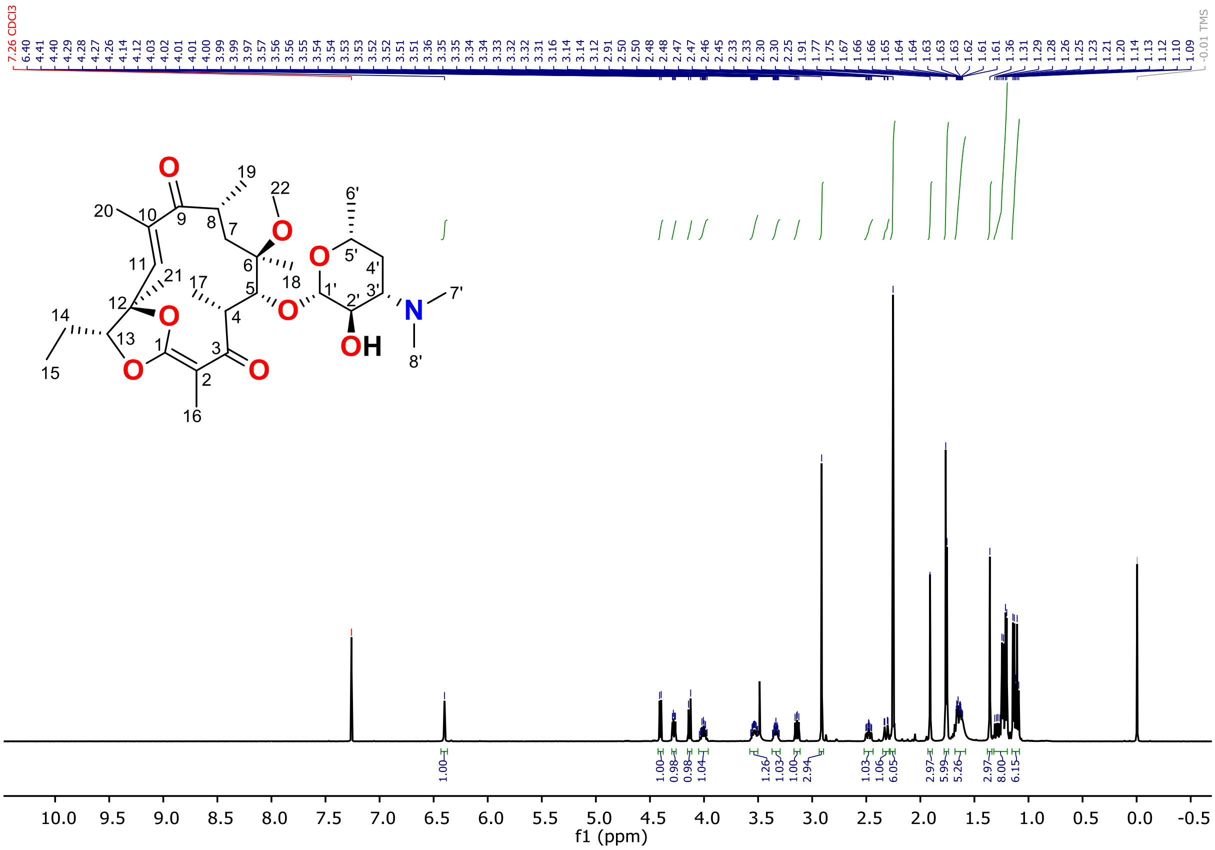

$^{13}C\{1H\}$-NMR (126 MHz, $CDCl_3$) spectrum of compound **8**.

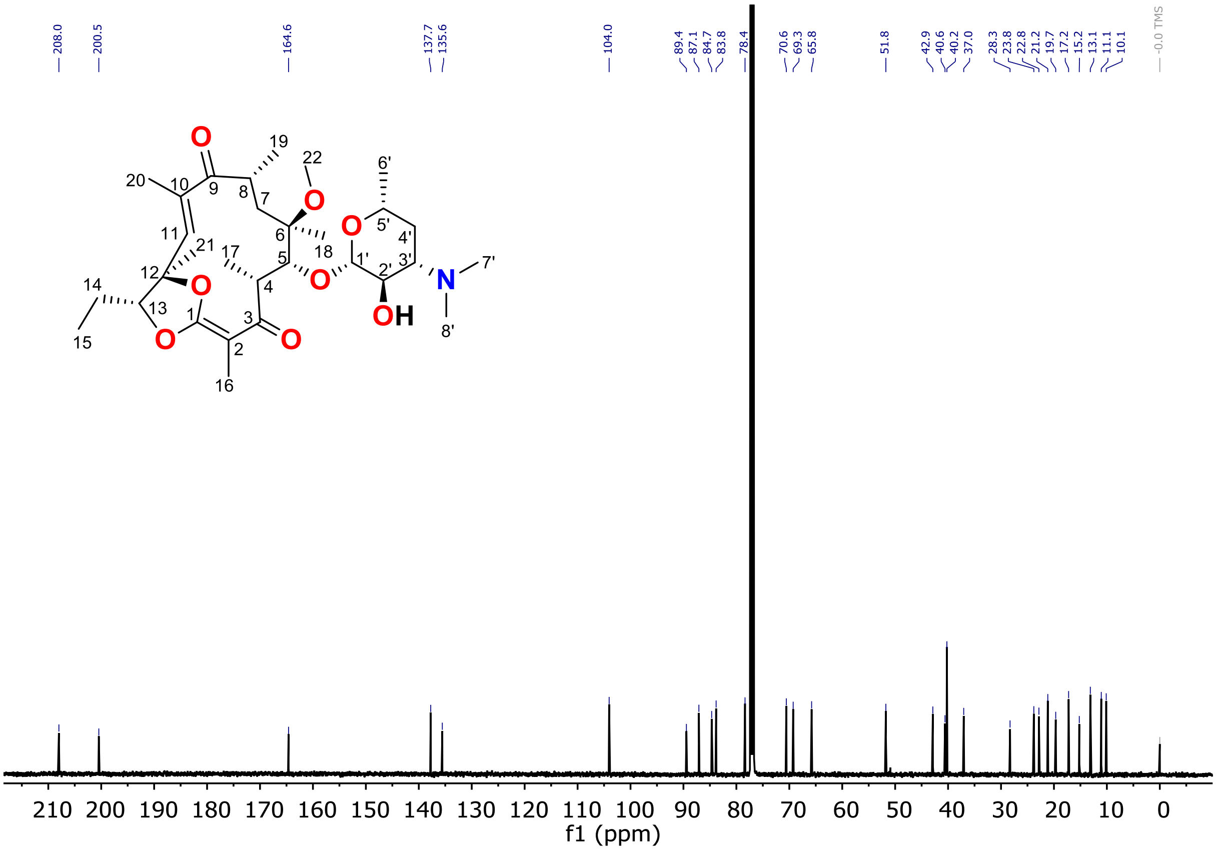

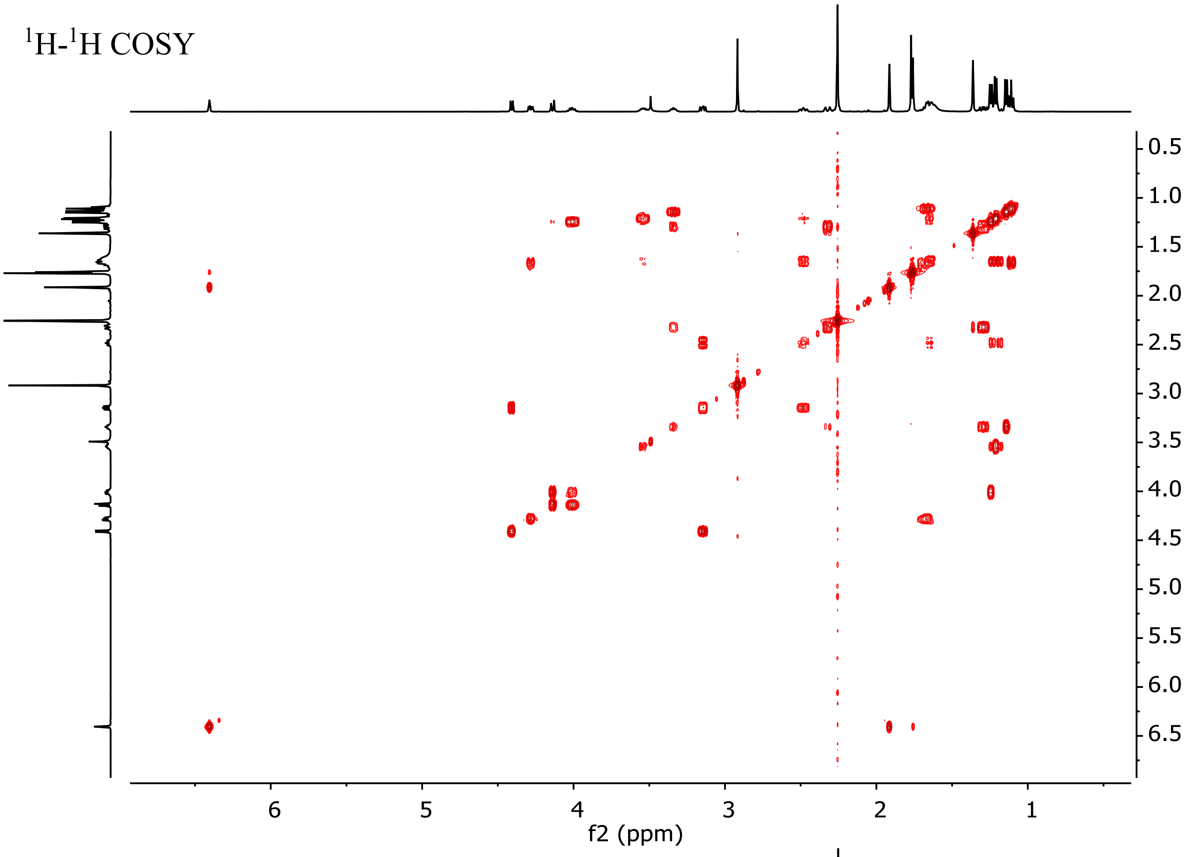

¹H-¹H COSY
f2 (ppm)

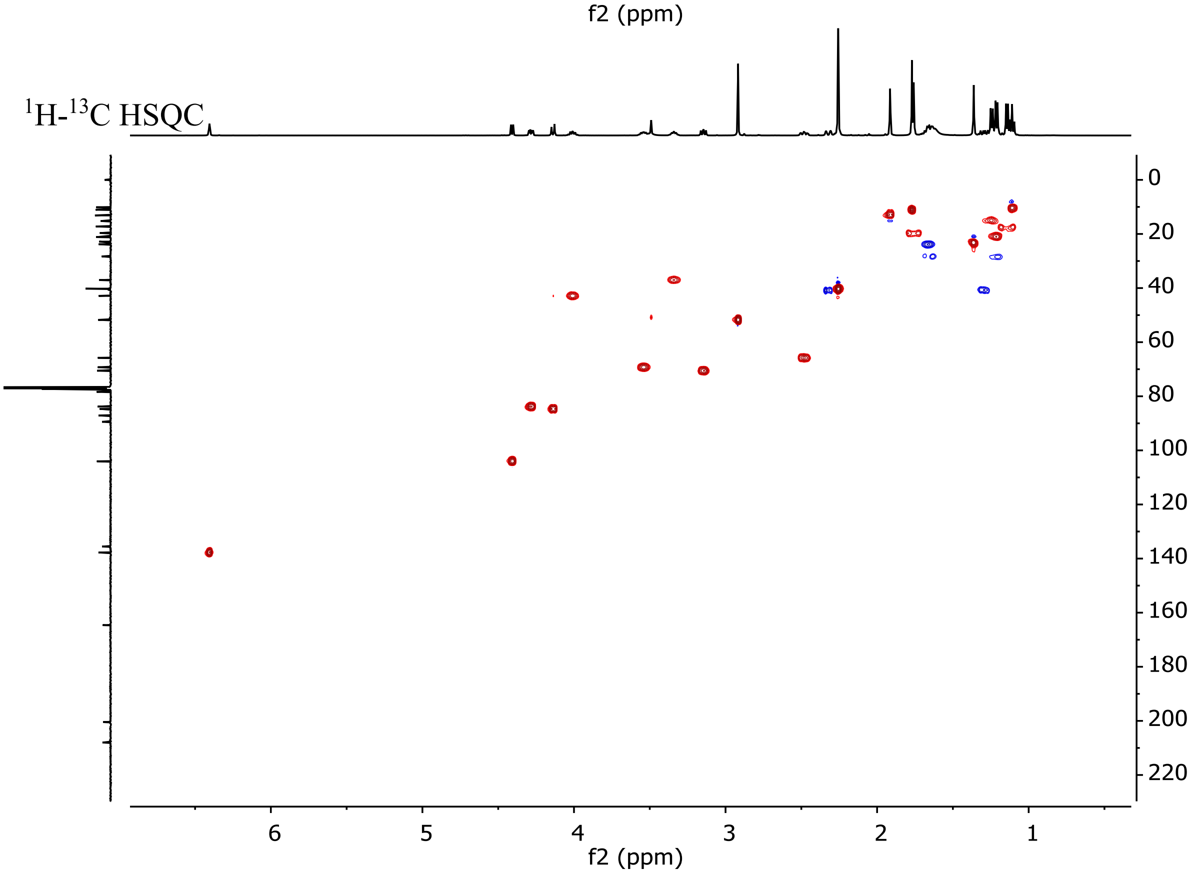

¹H-¹³C HSQC
f2 (ppm)

^1H-^{13}C HMBC

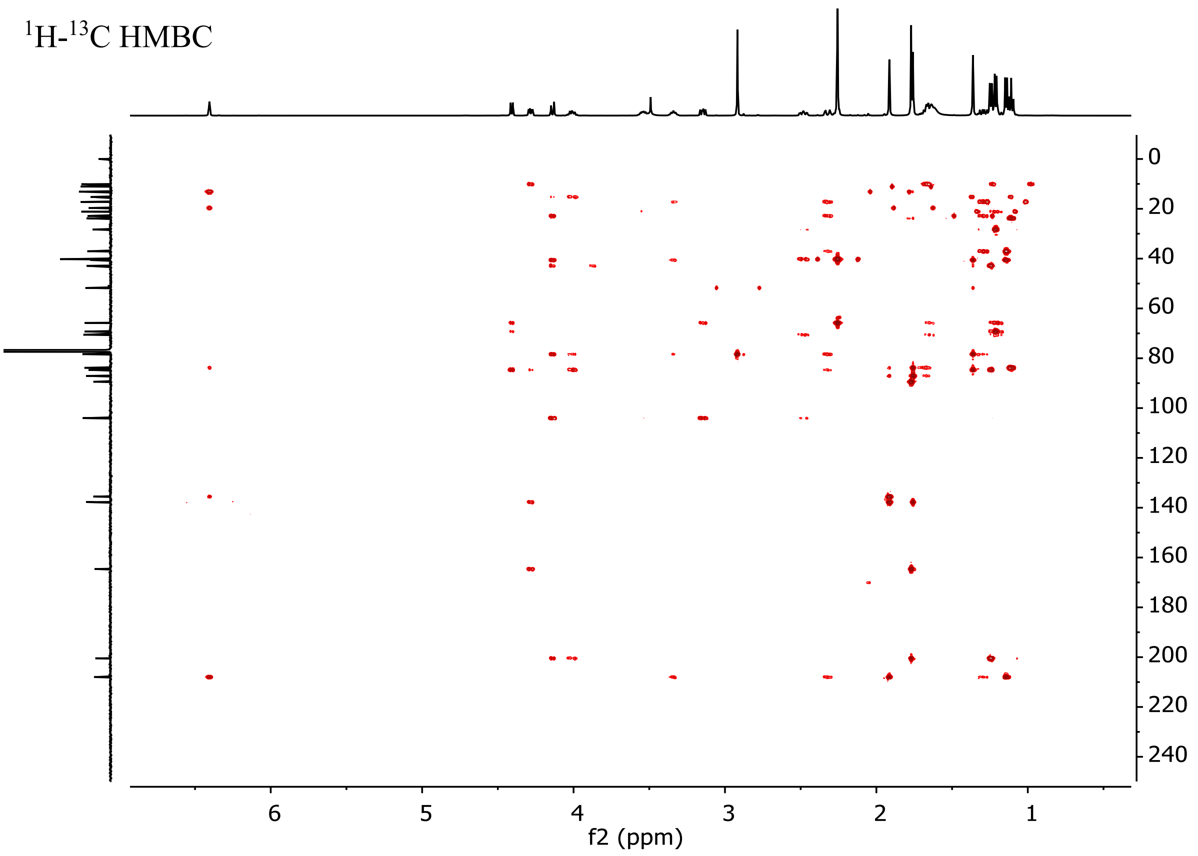

FT-IR spectrum of compound **8** in in ATR technique.

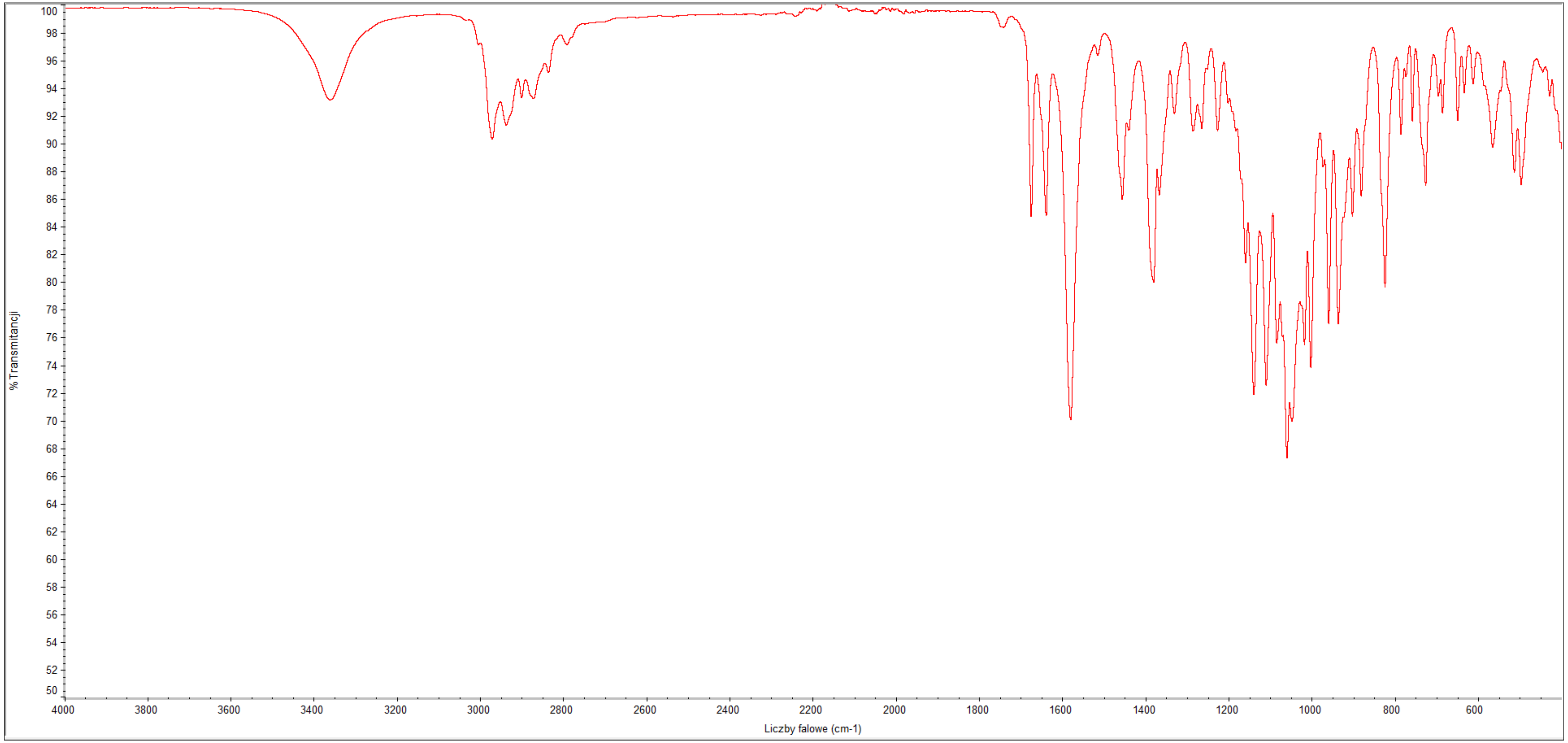

^{1}H-NMR (600 MHz, $CDCl_3$) spectrum of compound **9**.

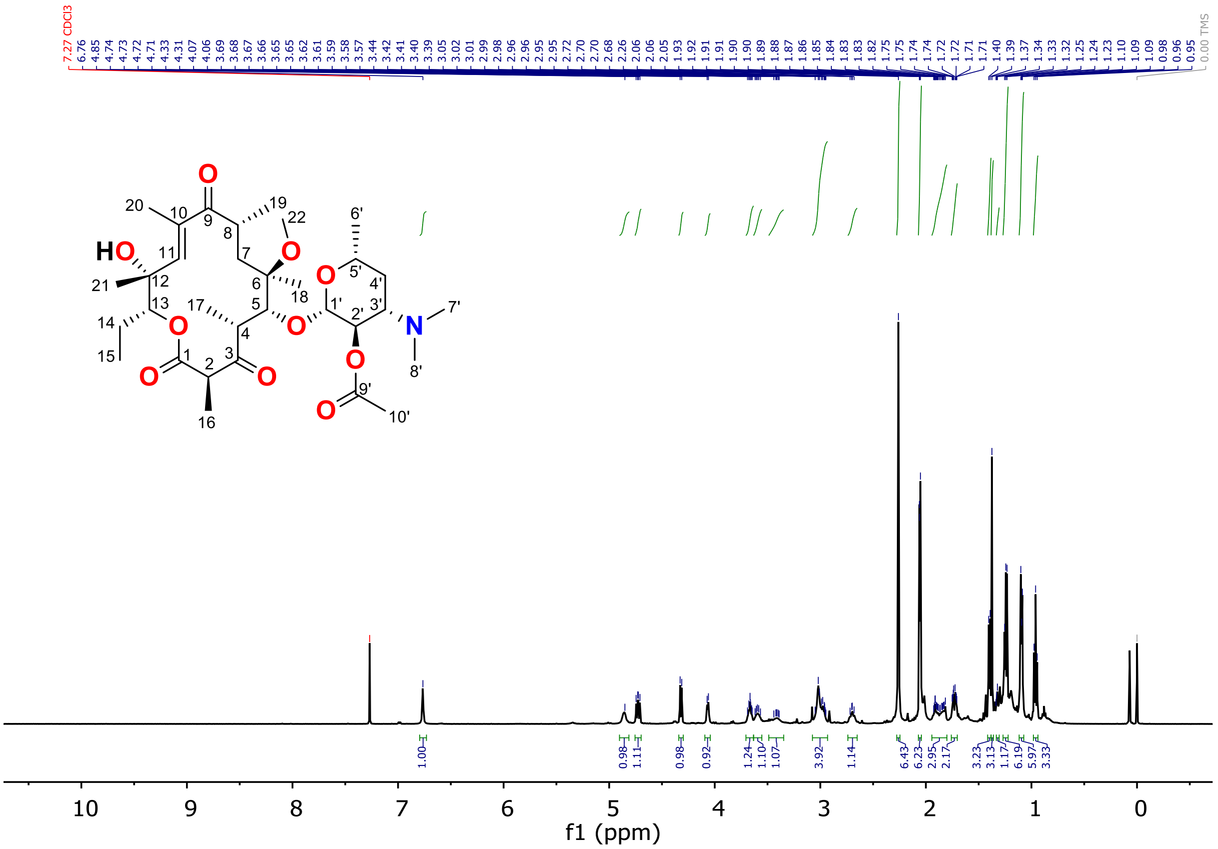

^{13}C{1H}-NMR (151 MHz, CDCl3) spectrum of compound **9**.

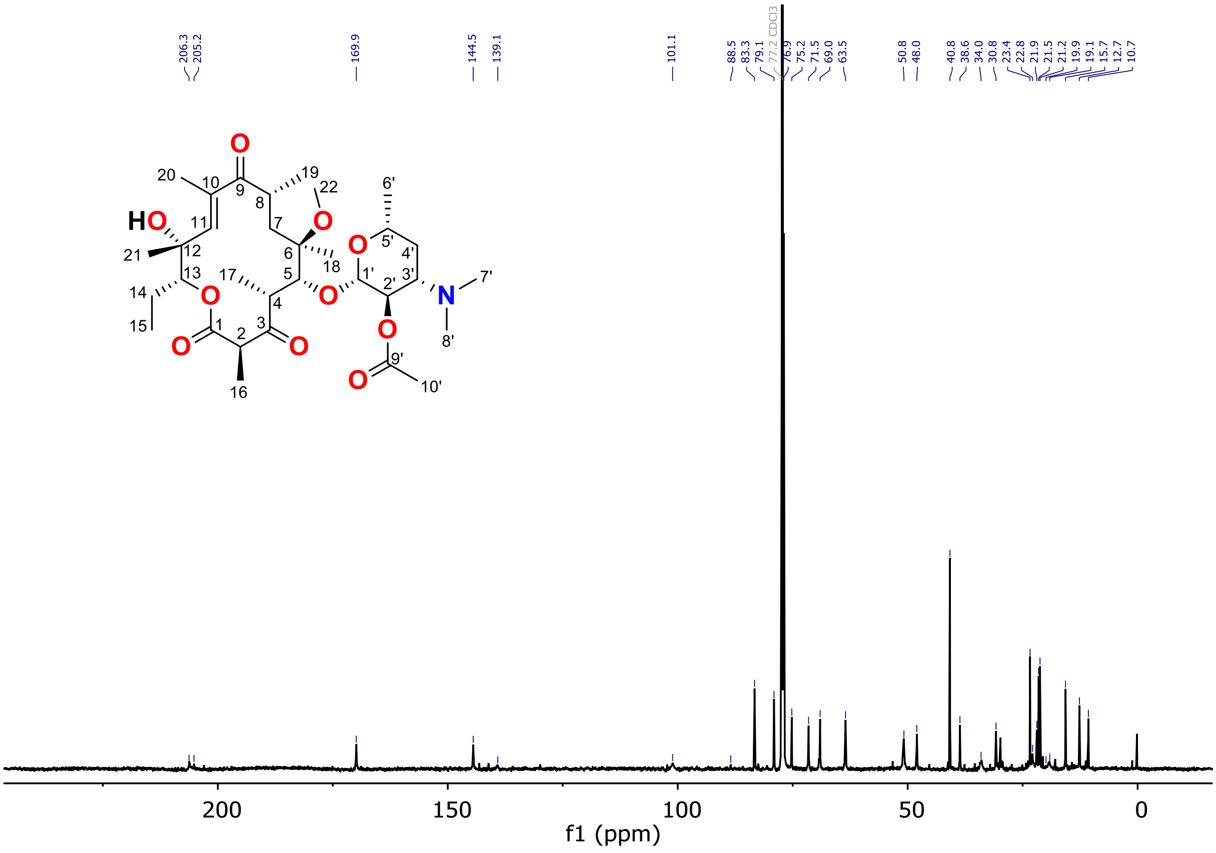

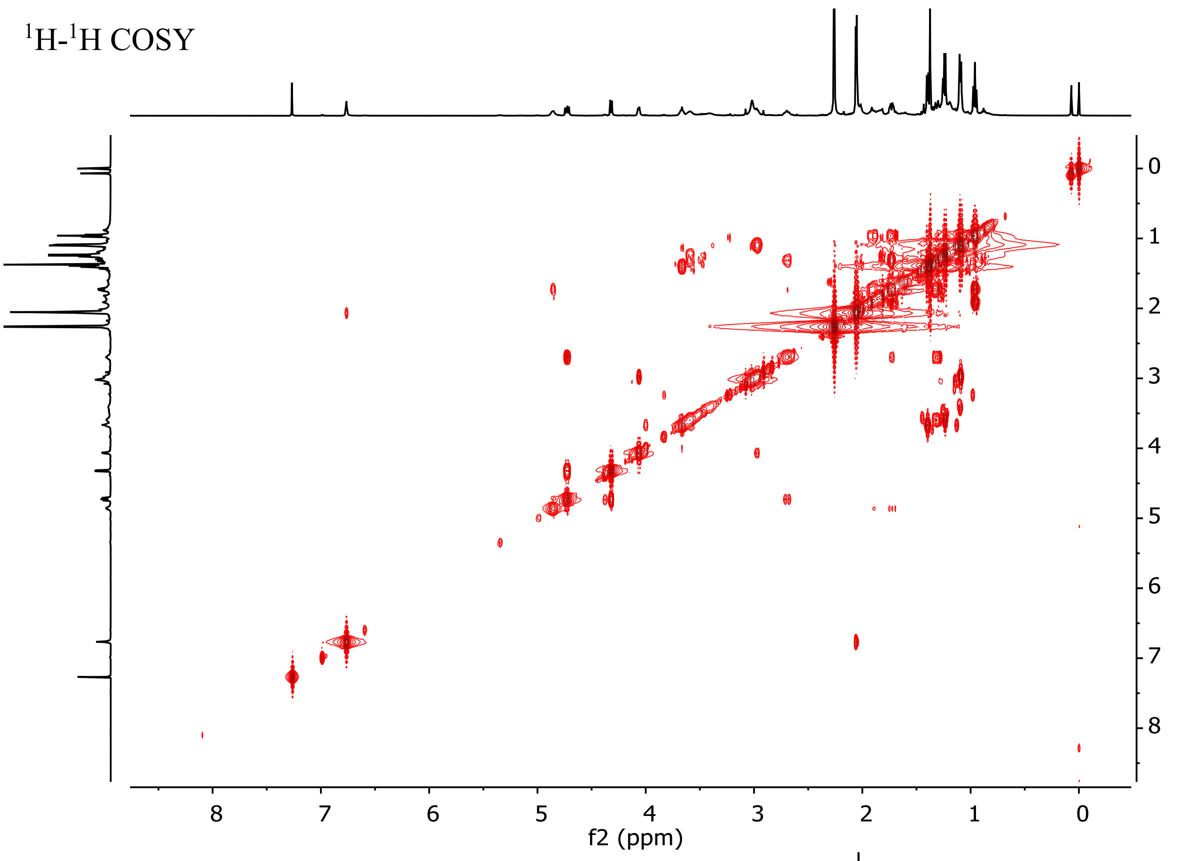

^{1}H-^{1}H COSY
f2 (ppm)

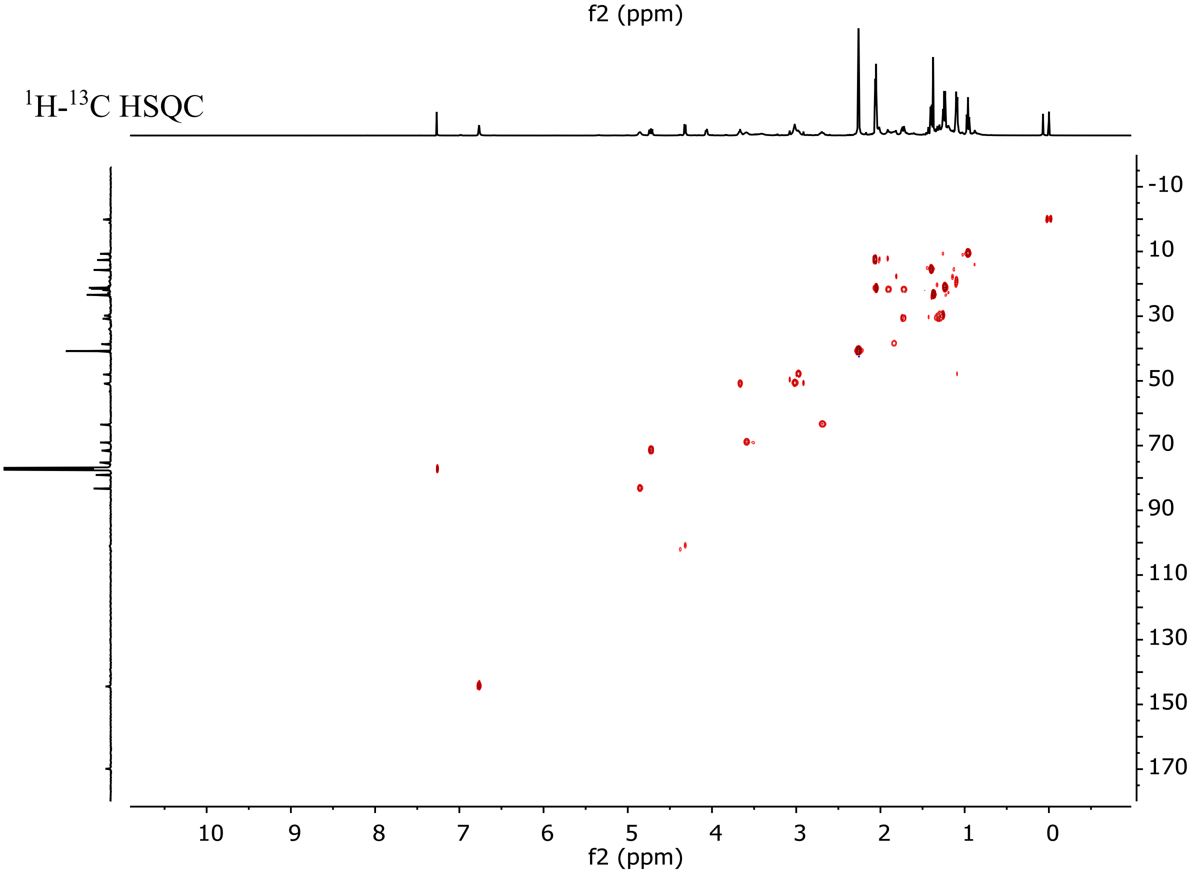

^{1}H-^{13}C HSQC
f2 (ppm)

^{1}H-^{13}C HMBC

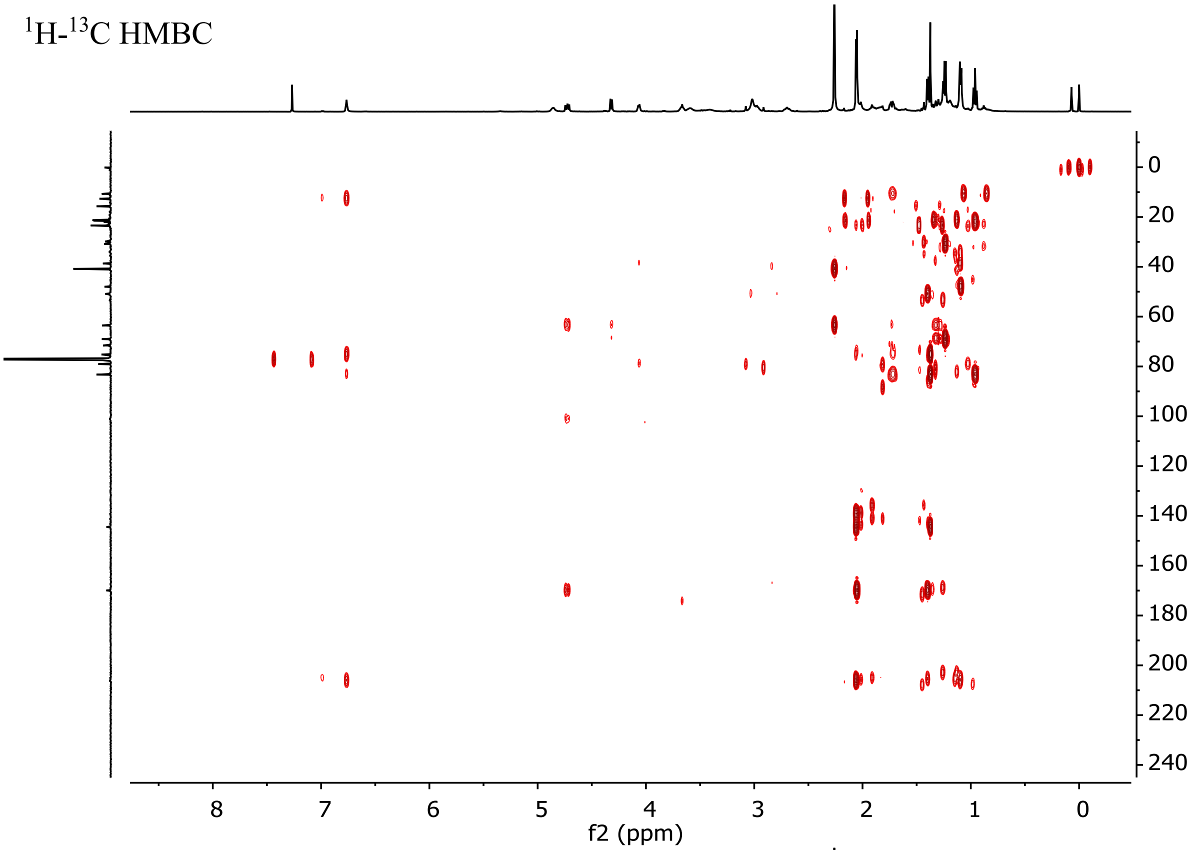

^{1}H-^{1}H NOESY

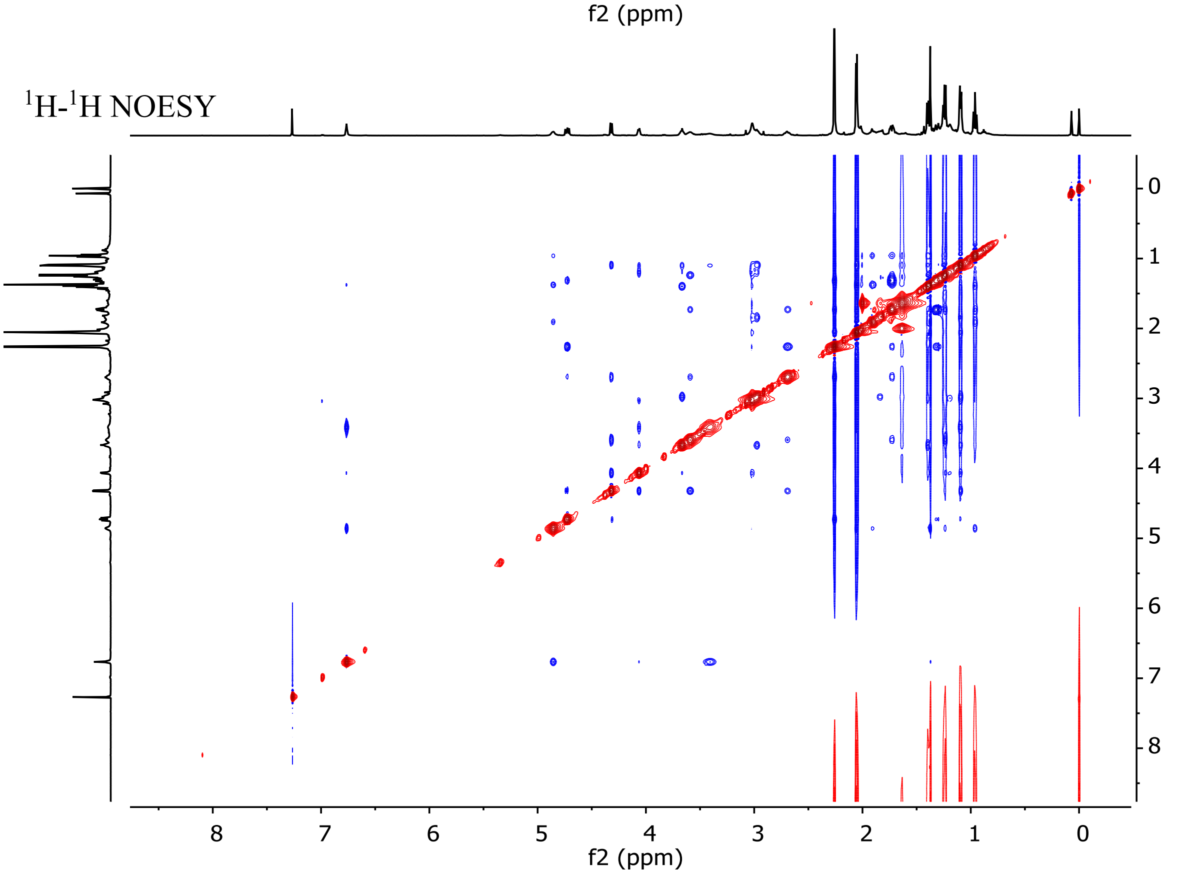

Table S3. Contacts observed in the ^{1}H-^{1}H NOESY spectra for **9**. Contacts for vicinal and geminal protons were omitted.

	H2	H4	H5	H7	H8	H11	H13	H14a	H14b	H15	H16	H17	H18	H19	H20	H21	H22	H1`	H2`	H3`	H4`a	H4`b	H5`	H6`	H7`, H8`	H10`
H2																										
H4																										
H5																										
H7																										
H8																										
H11																										
H13																										
H14a																										
H14b																										
H15																										
H16																										
H17																										
H18																										
H19																										
H20																										
H21																										
H22																										
H1`																										
H2`																										
H3`																										
H4`a																										
H4`b																										
H5`																										
H6`																										
H7` H8`																										
H10`																										

Red – strong; green – medium; blue – weak; grey – ambiguous due to overlapping signals

FT-IR spectrum of compound **9** in $CDCl_3$ solution.

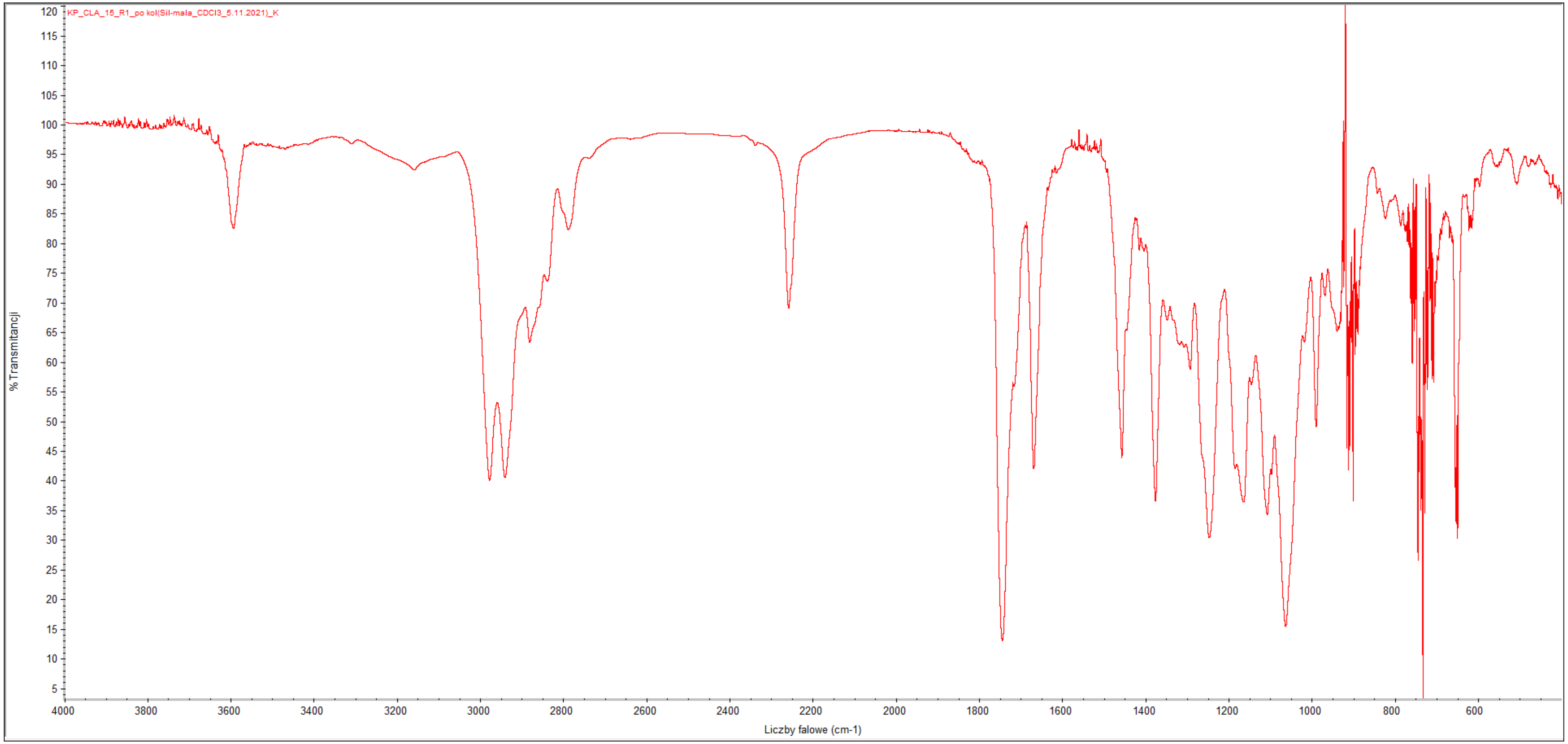

^{1}H-NMR (600 MHz, $CDCl_3$) spectrum of compounds **10a** and **10b**.

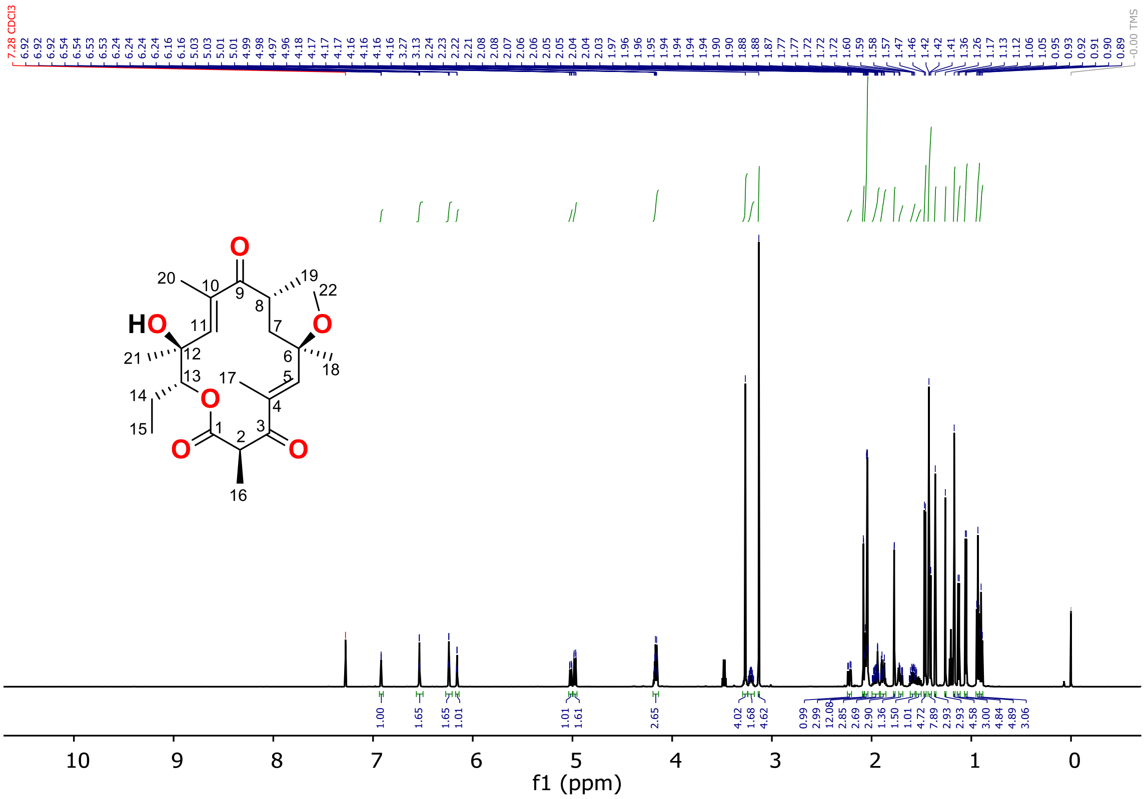

$^{13}C\{1H\}$-NMR (150 MHz, $CDCl_3$) spectrum of compounds **10a** and **10b**.

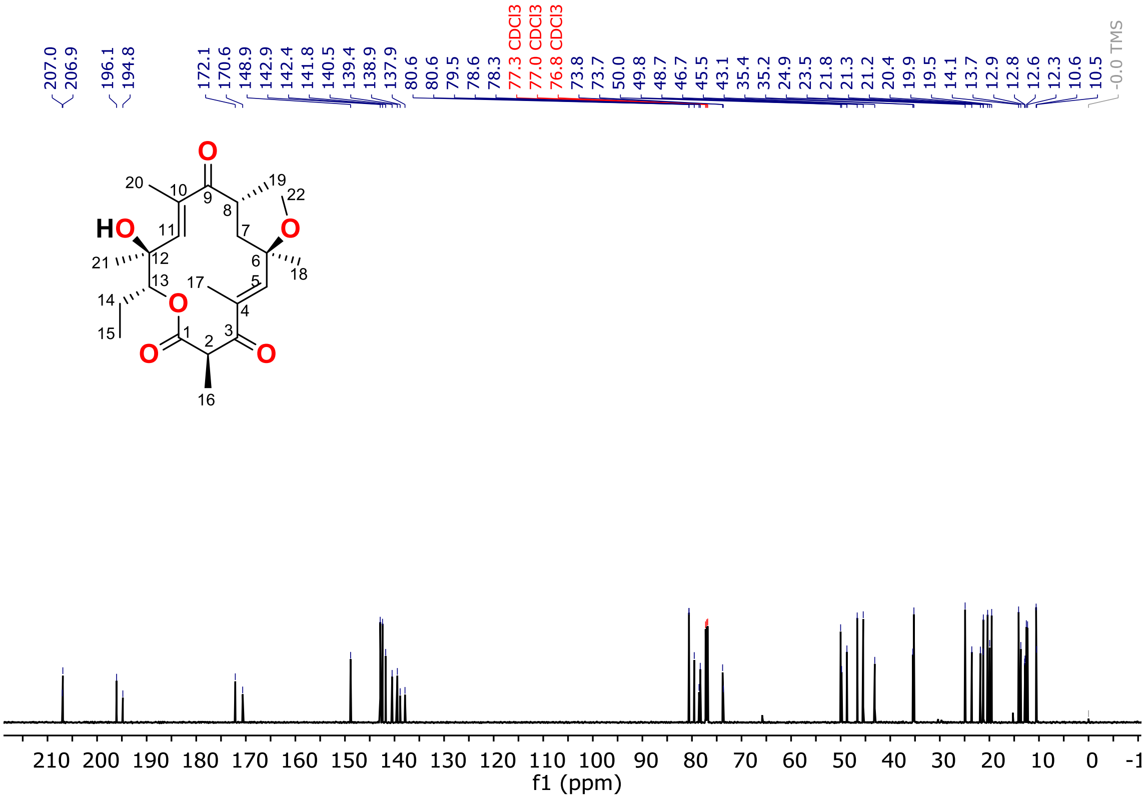

^{1}H-^{1}H COSY

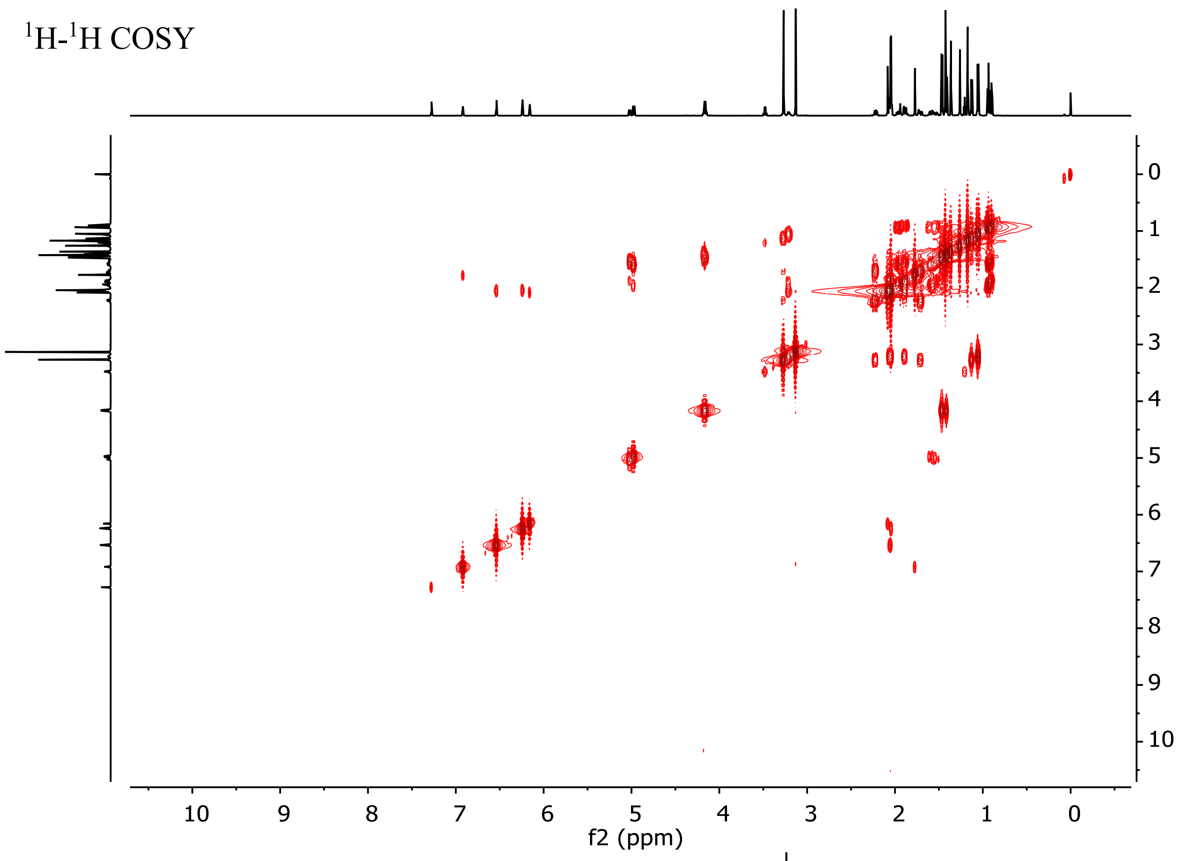

^{1}H-^{13}C HSQC

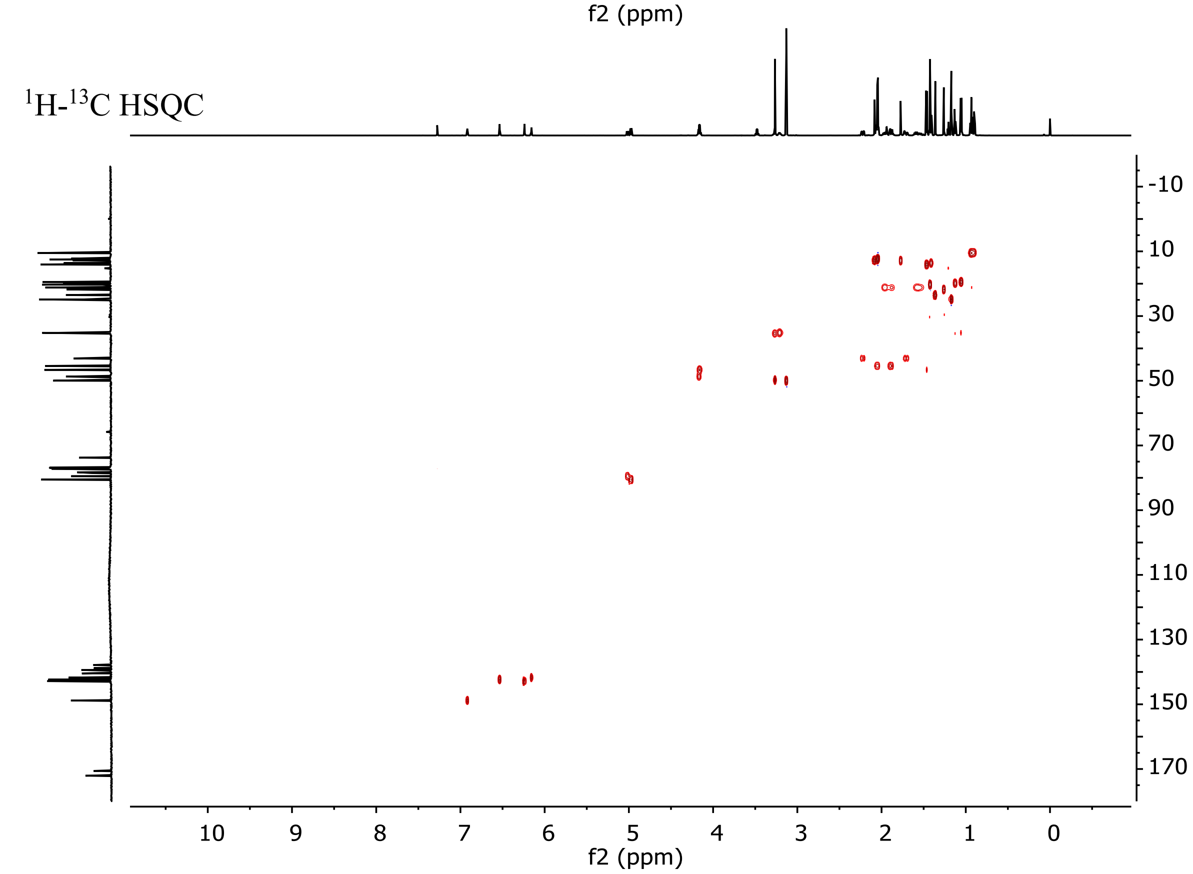

^{1}H-^{13}C HMBC

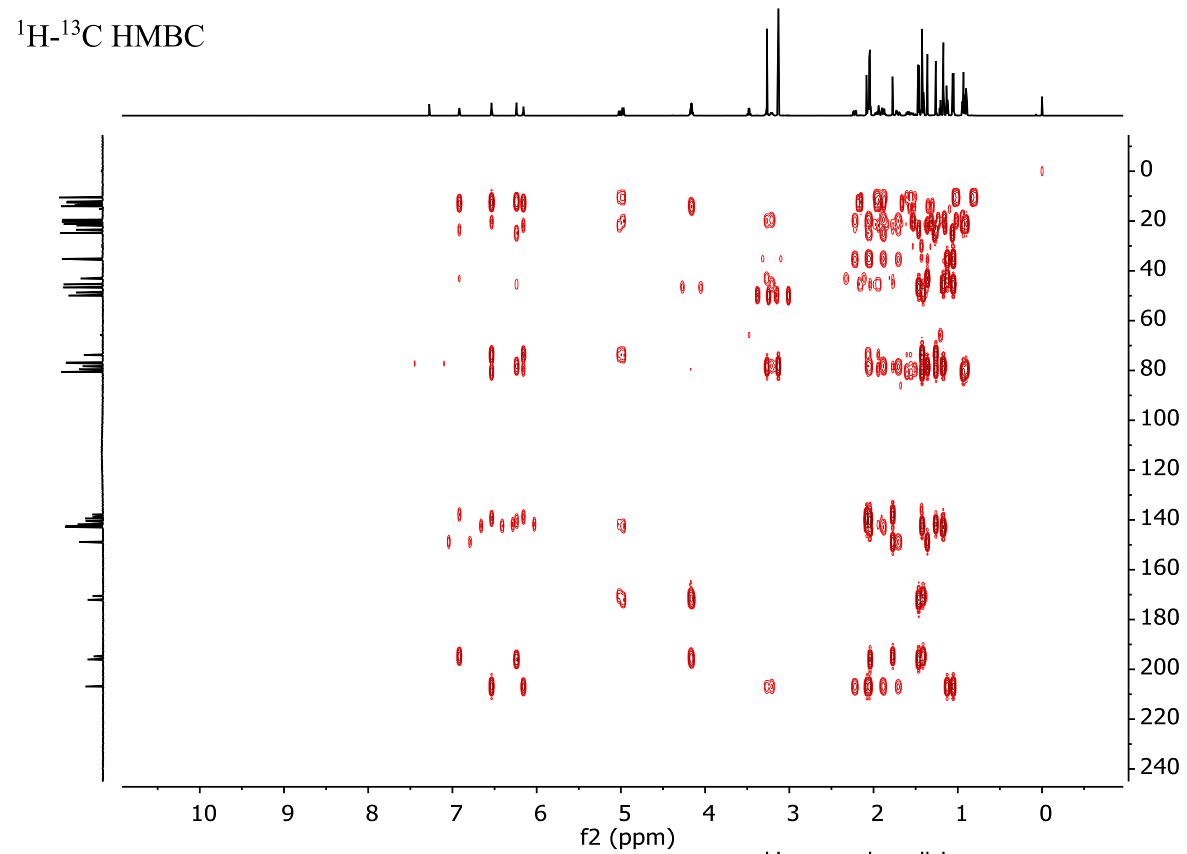

^{1}H-^{1}H NOESY

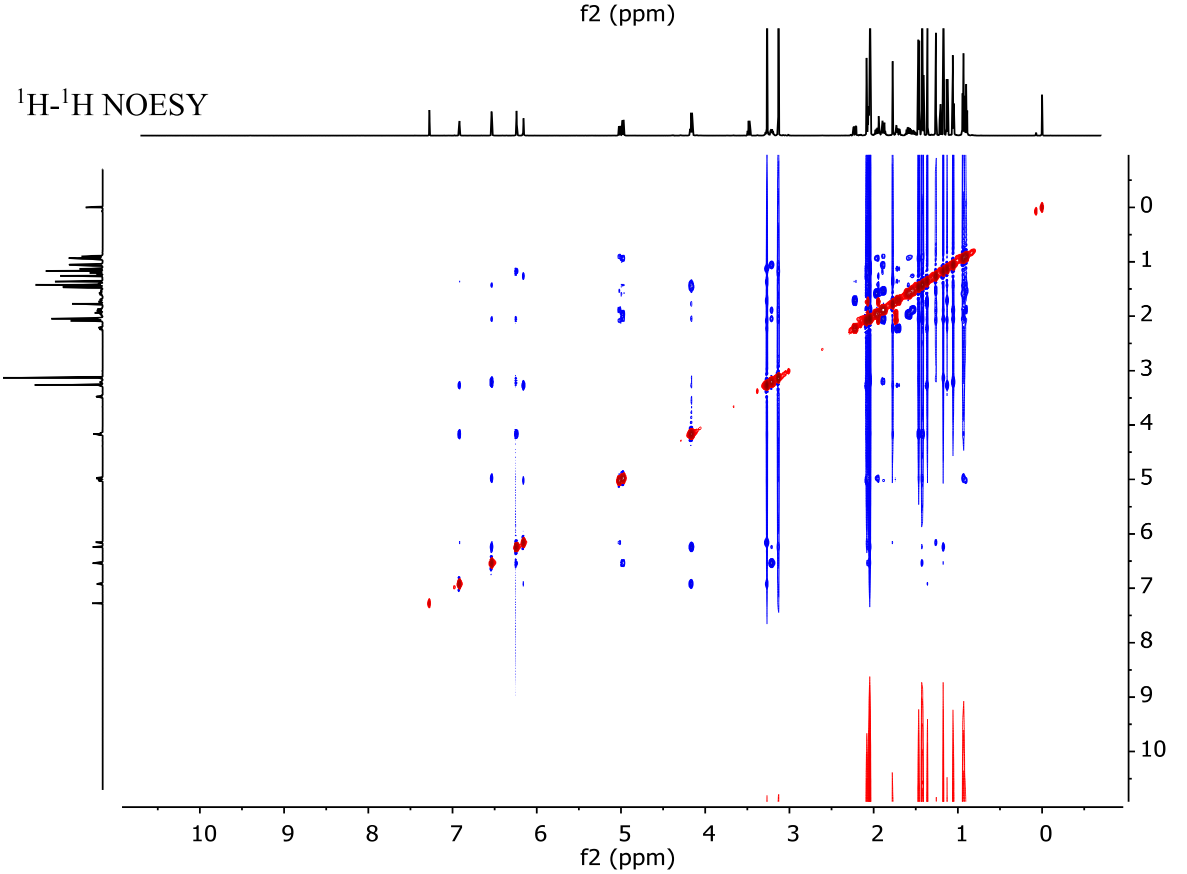

Table S4. Contacts observed in the ^{1}H-^{1}H NOESY spectra for **10a**. Contacts for vicinal and geminal protons were omitted.

	H2	H5	H7a	H7b	H8	H11	H13	H14a	H14b	H15	H16	H17	H18	H19	H20	H21	H22
H2	black	red	grey	blue		blue	blue								grey		
H5	red	black	grey	blue	green	red							red		grey		
H7a	grey	grey	black	grey	grey	grey	grey	grey	grey	grey	grey	grey	grey	grey	grey	grey	grey
H7b	blue	blue	grey	black									green	green	grey		green
H8		green	grey		black	red									grey		green
H11		red	grey		red	black	red		blue				blue		grey	green	
H13	blue	blue	grey			red	black			red	blue				grey	green	
H14a			grey				red	black							grey		
H14b			grey			blue	green		black		green				grey		
H15	blue		grey				green			black					grey		
H16			grey						green	green	black				grey		
H17	grey	grey	grey	grey	grey	grey	grey	grey	grey	grey	grey	black	grey	grey	grey	grey	grey
H18		red	grey	green		blue							black		grey		red
H19			grey	green										black	grey		
H20	grey	grey	grey		grey	grey	grey	grey	grey	grey	grey	grey	grey	grey	black	grey	grey
H21		blue	grey			green	green								grey	black	
H22			grey	green									red		grey		black

Red – strong; green – medium; blue – weak; grey – ambiguous due to overlapping signals

Table S5. Contacts observed in the ^{1}H-^{1}H NOESY spectra for **10b**. Contacts for vicinal and geminal protons were omitted.

	H2	H5	H7a	H7b	H8	H11	H13	H14a	H14b	H15	H16	H17	H18	H19	H20	H21	H22
H2	black	red			grey	blue	blue					blue					grey
H5	red	black	blue		grey	blue						blue	green		blue		grey
H7a		blue	black		grey	blue						blue	green	blue			grey
H7b				black	grey									green			grey
H8	grey	grey	grey	grey	black	red	grey	grey	grey	grey	grey	grey	grey	grey	grey	grey	grey
H11		blue	blue		grey	black	red		blue			green			blue	red	grey
H13					grey	red	black								blue	blue	grey
H14a					grey			black								blue	grey
H14b					grey	blue			black			blue				blue	grey
H15					grey		red			black	green						grey
H16					grey					green	black						grey
H17					grey	blue						black	green				grey
H18		green	green	green	grey							green	black				grey
H19			blue	green	grey									black	green		grey
H20		blue			grey	blue	blue							green	black	blue	grey
H21					grey	red	blue	blue	green						blue	black	grey
H22	grey	grey	grey	grey	grey	grey	grey	grey	grey	grey	grey	grey	grey	grey	grey	grey	black

Red – strong; green – medium; blue – weak; grey – ambiguous due to overlapping signals

^{1}H-NMR (600 MHz, DMSO-d_6) spectrum of compounds **10a** and **10b**.

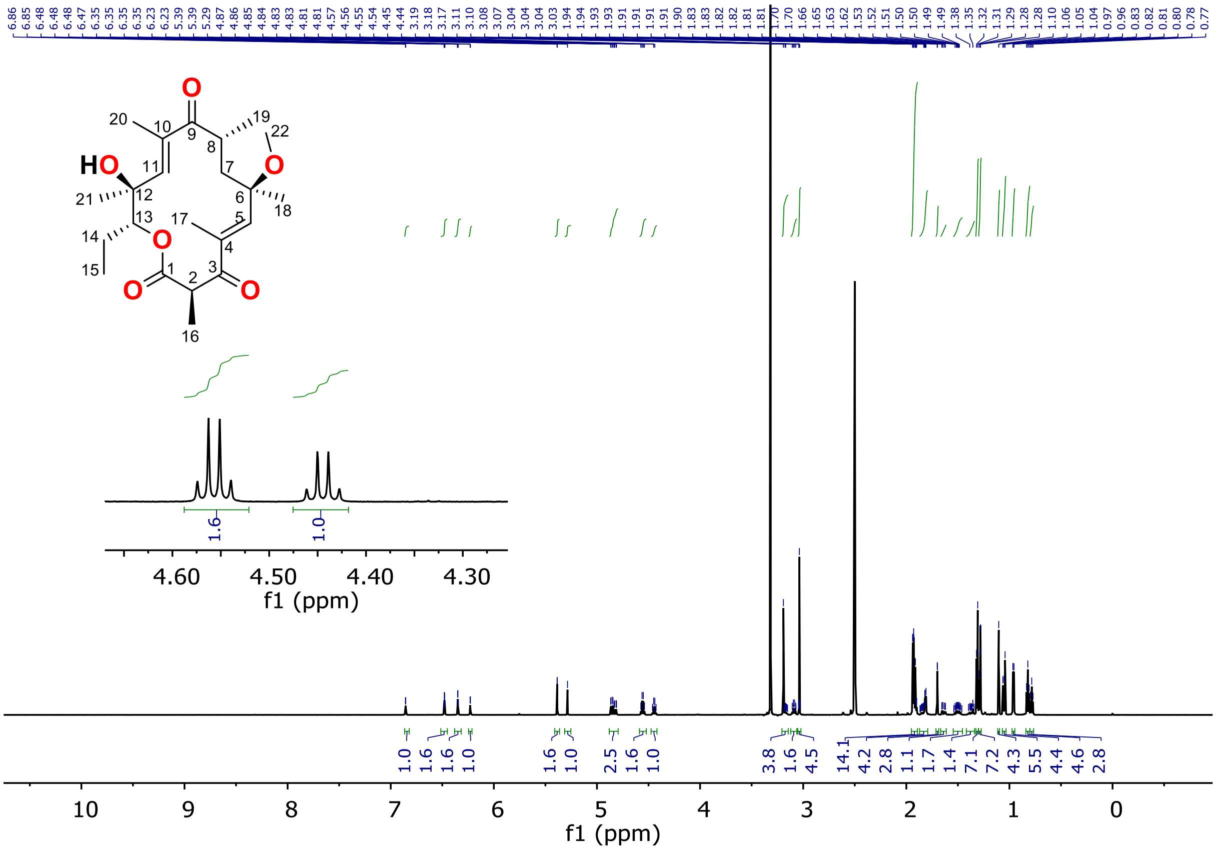

^{1}H-NMR (600 MHz, CD_3CN) spectrum of compounds **10a** and **10b**.

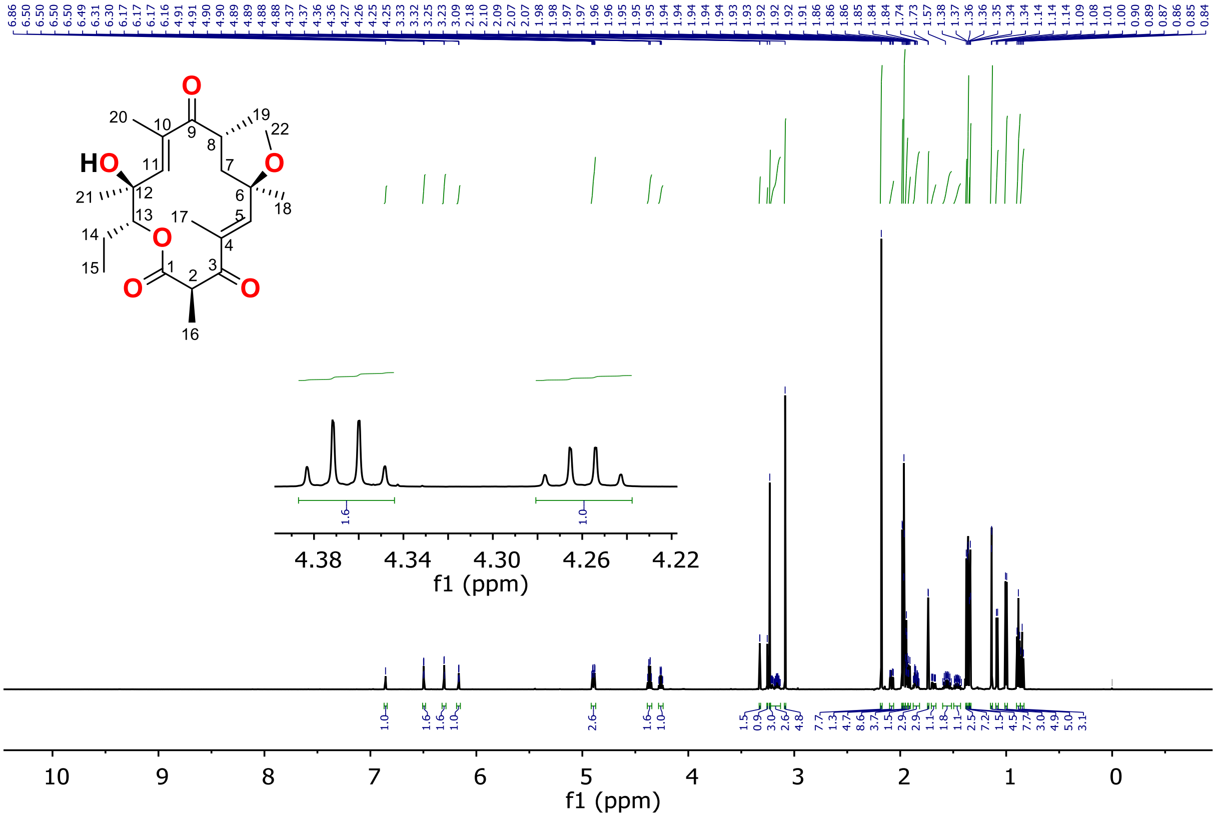

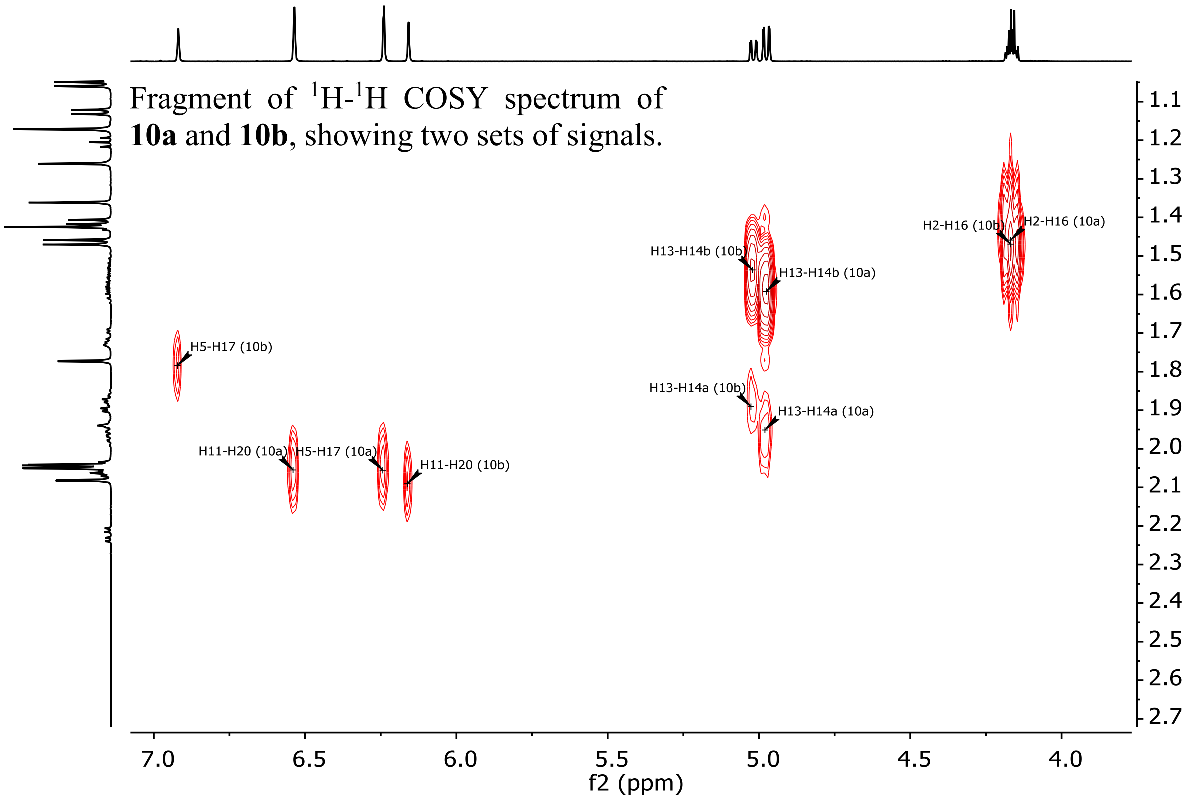
Fragment of ¹H-¹H COSY spectrum of 10a and 10b, showing two sets of signals.
H5-H17 (10b)
H11-H20 (10a)
H5-H17 (10a)
H11-H20 (10b)
H13-H14b (10b)
H13-H14b (10a)
H13-H14a (10b)
H13-H14a (10a)
H2-H16 (10b)
H2-H16 (10a)
f2 (ppm)

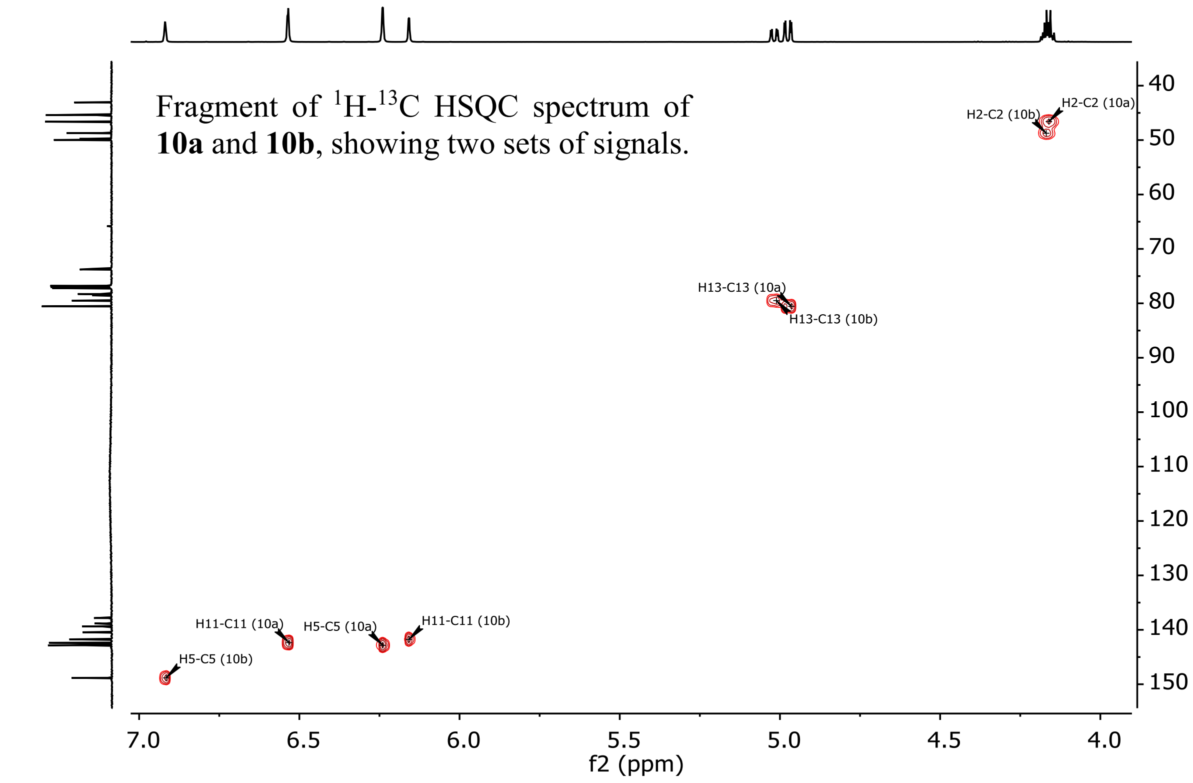
Fragment of ¹H-¹³C HSQC spectrum of 10a and 10b, showing two sets of signals.
H2-C2 (10b)
H2-C2 (10a)
H13-C13 (10a)
H13-C13 (10b)
H11-C11 (10a)
H5-C5 (10a)
H11-C11 (10b)
H5-C5 (10b)
f2 (ppm)

Fragment of ^{1}H-^{13}C HMBC spectrum of **10a** and **10b**, showing two sets of signals.

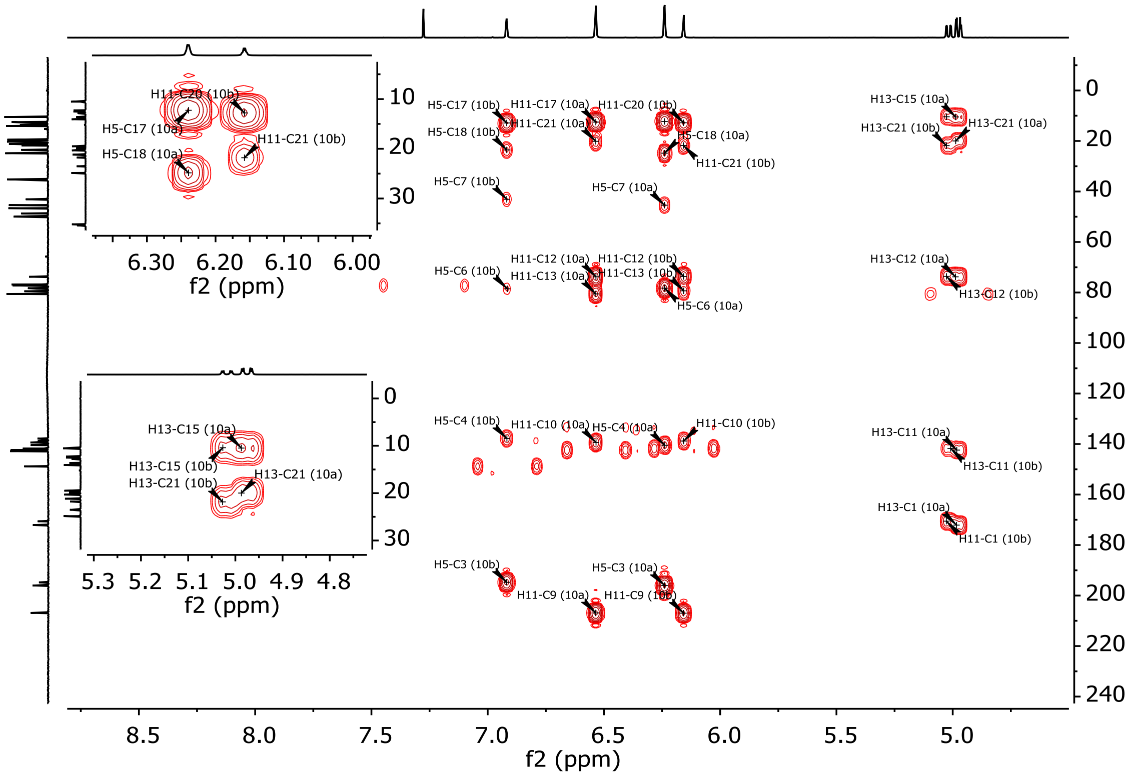

FT-IR spectrum of compounds **10a** and **10b** in $CDCl_3$ solution.

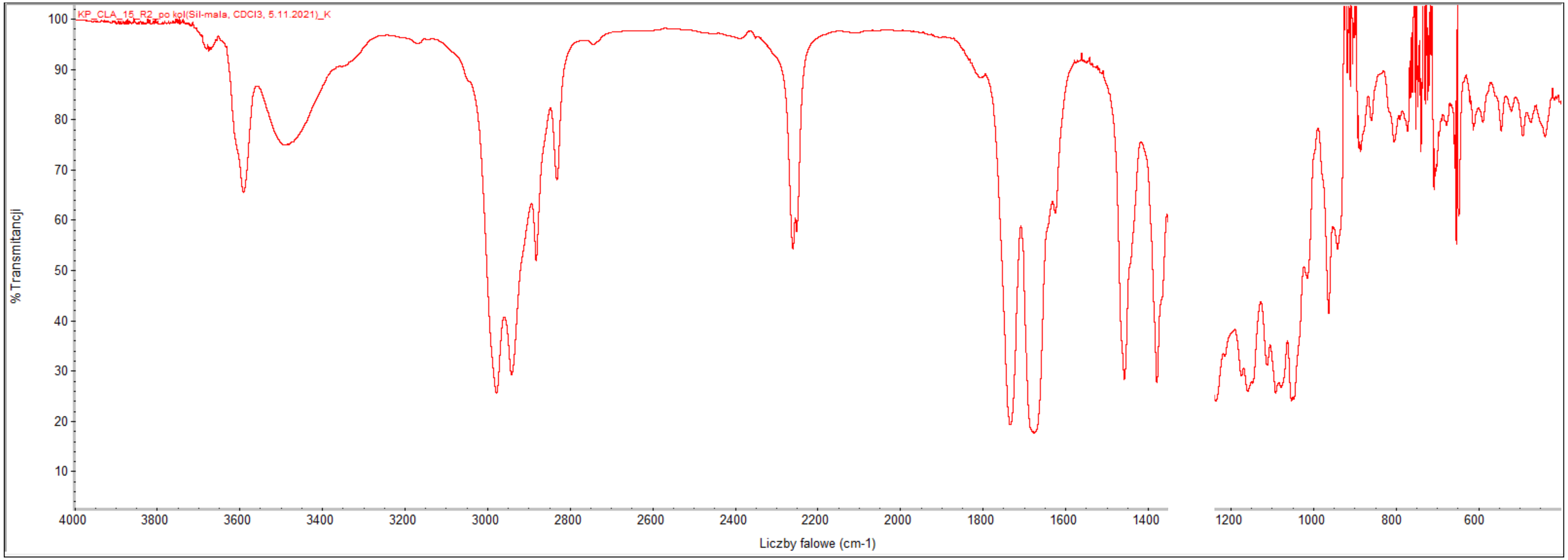

Supplement: Supplementary file 1 — jo1c02799_si_001.pdf [file jo1c02799_si_001.pdf]
