# Supplementary material for: An Approach to Modify 14-Membered Lactone Macrolide Antibiotic Scaffolds
Source: J Org Chem. 2022 Jan 12;87(5):3758–61. doi: 10.1021/acs.joc.1c02799 (PMC8902751; doi:10.1021/acs.joc.1c02799)
Supplement: Supplementary file 2 — jo1c02799_si_002.pdf [file jo1c02799_si_002.pdf]

Compound **9**

Parallel Execution: Process Information

==

Rank	Node Name	NodeID	MyNodeRank	NodeMaster
0	Biochemia	0	0	0
1	Biochemia	0	1	-1
2	Biochemia	0	2	-1
3	Biochemia	0	3	-1
4	Biochemia	0	4	-1
5	Biochemia	0	5	-1
6	Biochemia	0	6	-1
7	Biochemia	0	7	-1

==

May use up to 124843MB of RAM as shared memory on node 0

ATOMS

1 O	14.127594000000	32.500852000000	26.696554000000
2 C	14.358973000000	32.706087000000	28.074837000000
3 C	15.844981000000	32.343329000000	28.358831000000
4 C	16.221909000000	32.663456000000	29.841401000000
5 O	14.407148000000	28.055160000000	22.679247000000
6 N	17.635814000000	32.463257000000	30.238882000000
7 O	14.109206000000	34.092935000000	28.385324000000
8 C	10.791179000000	30.985837000000	25.526217000000
9 C	13.471786000000	34.688484000000	21.936285000000
10 C	11.380192000000	29.158706000000	23.707986000000
11 O	11.457872000000	33.320415000000	24.259092000000

12	C	10.294700000000	30.020141000000	24.372823000000
13	O	11.317562000000	29.165411000000	22.356088000000
14	C	12.739522000000	32.541400000000	26.226598000000
15	C	12.735862000000	33.500259000000	24.955720000000
16	O	9.912295000000	31.530428000000	26.202332000000
17	C	12.448128000000	30.116555000000	27.141537000000
18	C	14.422765000000	28.116335000000	20.287601000000
19	O	15.992551000000	30.896814000000	28.127512000000
20	C	13.814282000000	30.215583000000	21.581908000000
21	C	13.722053000000	28.674141000000	21.562273000000
22	C	12.273016000000	31.080713000000	25.921319000000
23	C	13.602349000000	33.226900000000	22.418257000000
24	C	18.655834000000	33.042962000000	29.340729000000
25	C	13.868224000000	33.055917000000	23.954164000000
26	C	15.774613000000	34.116276000000	30.176962000000
27	C	14.302560000000	34.382086000000	29.803050000000
28	C	13.867504000000	35.836576000000	30.022426000000
29	C	14.901092000000	30.989362000000	21.835226000000
30	C	9.332718000000	30.753315000000	23.400154000000
31	O	15.654011000000	33.141192000000	21.109559000000
32	C	12.195121000000	28.262619000000	21.521163000000
33	C	16.306268000000	30.513336000000	22.180196000000
34	C	14.773810000000	32.505190000000	21.707005000000
35	C	12.974688000000	34.959516000000	25.417540000000
36	C	11.915018000000	26.768042000000	21.788833000000
37	C	17.993636000000	31.096769000000	30.663409000000
38	C	10.343191000000	34.150980000000	24.666038000000
39	C	10.417328000000	26.382513000000	21.796658000000
40	H	12.881329000000	30.730846000000	21.325085000000
41	H	16.560641000000	30.803551000000	23.216870000000
42	H	16.412255000000	29.425220000000	22.100993000000

43	H	17.033265000000	31.022119000000	21.525761000000
44	H	14.346890000000	35.288377000000	22.236736000000
45	H	13.408011000000	34.732331000000	20.836007000000
46	H	12.563647000000	35.152786000000	22.352607000000
47	H	12.667092000000	32.686905000000	22.195365000000
48	H	14.787657000000	33.602982000000	24.215821000000
49	H	14.097653000000	31.995436000000	24.128067000000
50	H	13.940609000000	35.012853000000	25.936850000000
51	H	12.207906000000	35.302394000000	26.129523000000
52	H	12.999958000000	35.652376000000	24.562127000000
53	H	10.538077000000	35.218267000000	24.459908000000
54	H	10.081739000000	34.016636000000	25.731113000000
55	H	9.489867000000	33.821738000000	24.053891000000
56	H	11.812819000000	28.527710000000	20.522370000000
57	H	12.424502000000	26.212396000000	20.982297000000
58	H	12.390201000000	26.451541000000	22.728514000000
59	H	9.875900000000	26.855662000000	22.632752000000
60	H	9.909387000000	26.671382000000	20.859304000000
61	H	10.313031000000	25.290285000000	21.915126000000
62	H	14.382920000000	27.015301000000	20.270699000000
63	H	13.962297000000	28.515085000000	19.369200000000
64	H	15.480423000000	28.418239000000	20.296237000000
65	H	12.899989000000	30.685139000000	25.111764000000
66	H	11.820741000000	30.430326000000	27.993641000000
67	H	13.501976000000	30.088675000000	27.453166000000
68	H	12.159268000000	29.094040000000	26.851631000000
69	C	16.749988000000	30.483106000000	27.047064000000
70	H	14.478144000000	36.520427000000	29.408110000000
71	H	13.985899000000	36.117797000000	31.082619000000
72	H	12.808905000000	35.970918000000	29.743709000000
73	H	13.643998000000	33.706693000000	30.392693000000

74 H 16.404069000000 34.839888000000 29.629690000000

75 H 15.936177000000 34.285460000000 31.255375000000

76 H 18.793537000000 32.489197000000 28.386160000000

77 H 18.401370000000 34.086460000000 29.094051000000

78 H 19.625872000000 33.057497000000 29.869002000000

79 H 18.078099000000 30.356160000000 29.838131000000

80 H 17.246903000000 30.720531000000 31.382753000000

81 H 18.968540000000 31.131753000000 31.182817000000

82 H 13.689367000000 32.075143000000 28.697891000000

83 H 12.100352000000 32.981794000000 27.009378000000

84 H 16.481408000000 32.886006000000 27.644709000000

85 H 15.629298000000 31.975468000000 30.472486000000

86 H 13.811563000000 28.165939000000 23.462977000000

87 O 12.199467000000 28.493642000000 24.363247000000

88 H 9.696725000000 29.284034000000 24.943742000000

89 H 17.623006000000 28.591042000000 26.500740000000

90 H 16.373163000000 28.464570000000 27.804791000000

91 H 15.897677000000 28.754574000000 26.104575000000

92 C 16.660583000000 28.973769000000 26.872576000000

93 O 17.401403000000 31.231595000000 26.323793000000

94 H 9.871146000000 31.473401000000 22.765385000000

95 H 8.577603000000 31.295638000000 23.988698000000

96 H 8.817425000000 30.032839000000 22.745811000000

END

GUIBONDS

1 1 2 1.0

2 1 14 1.0

3 2 82 1.0

4 2 7 1.0

5 2 3 1.0

6 3 84 1.0

7 3 19 1.0

8 3 4 1.0

9 4 85 1.0

10 4 6 1.0

11 4 26 1.0

12 5 86 1.0

13 5 21 1.0

14 6 37 1.0

15 6 24 1.0

16 7 27 1.0

17 8 16 2.0

18 8 22 1.0

19 8 12 1.0

20 9 46 1.0

21 9 44 1.0

22 9 45 1.0

23 9 23 1.0

24 10 87 2.0

25 10 13 1.0

26 10 12 1.0

27 11 38 1.0

28 11 15 1.0

29 12 88 1.0

30 12 30 1.0

31 13 32 1.0

32 14 83 1.0

33 14 22 1.0

34 14 15 1.0

35 15 35 1.0

36 15 25 1.0

37 17 67 1.0

38 17 68 1.0

39 17 66 1.0

40 17 22 1.0

41 18 64 1.0

42 18 62 1.0

43 18 63 1.0

44 18 21 1.0

45 19 69 1.0

46 20 40 1.0

47 20 29 2.0

48 22 65 1.0

49 23 47 1.0

50 23 34 1.0

51 23 25 1.0

52 24 77 1.0

53 24 78 1.0

54 24 76 1.0

55 25 49 1.0

56 25 48 1.0

57 26 75 1.0

58 26 74 1.0

59 26 27 1.0

60 27 73 1.0

61 27 28 1.0

62 28 72 1.0

63 28 71 1.0

64 28 70 1.0

65 29 33 1.0

66 29 34 1.0

67 30 95 1.0

68 30 94 1.0

69 30 96 1.0

70 31 34 2.0

71 32 56 1.0

72 32 36 1.0

73 33 42 1.0

74 33 43 1.0

75 33 41 1.0

76 35 50 1.0

77 35 52 1.0

78 35 51 1.0

79 36 58 1.0

80 36 57 1.0

81 36 39 1.0

82 37 80 1.0

83 37 81 1.0

84 37 79 1.0

85 38 55 1.0

86 38 53 1.0

87 38 54 1.0

88 39 59 1.0

89 39 61 1.0

90 39 60 1.0

91 69 93 2.0

92 69 92 1.0

93 89 92 1.0

94 90 92 1.0

95 91 92 1.0

96 20 21 1.0

97 21 32 1.0

END

XC

GGA BLYP

DISPERSION Grimme3

END

SCANFREQ -1000 0

GEOMETRY

END

AnalyticalFreq

END

NOPRINT LOGFILE

Fragments

H t21.H

N t21.N

C t21.C

O t21.O

End

end input

```
 ***************************************************************************
 *                                              *
 *  ------------------------------------                         *
 *   Amsterdam Density Functional  (ADF)        2017                    *
 *  ------------------------------------                         *
 *                               r67691 2018-08-20           *
 *                                              *
```

*   *
*   =================   *
* | | *
* | A D F | *
* | | *
*   =================   *
*   *
*   *
*   Online information and documentation: https://www.scm.com/support/   *
*   E-mail: support@scm.com   info@scm.com   *
*   *
*   Scientific publications using ADF results must be properly referenced   *
*   See the User Manuals (or the web site) for recommended citations   *
*   The terms and conditions of the End User License Agreement apply to   *
*   the use of ADF, https://www.scm.com/license-terms/   *
*   *
******************** x86_64_windows_intel / intelmpi **********************

Licensed to: Prof. Piotr Przybylski / Biochemistry Institute, Adam Mickiewicz University / Poznan / POLAND

SCM User ID: u15220

ADF 2017 RunTime: Nov10-2021 12:43:16 Nodes: 1 Procs: 8

Temporary files are created in C:\SCMTMP\kid_0.a05124

Communication costs MPI_COMM_WORLD: 1.259 usec per message,   0.0058 usec per 8-byte item

Communication costs for intra-node: 1.255 usec per message,   0.0058 usec per 8-byte item

*** (NO TITLE) ***

A T T A C H E D   F I L E S

M O D E L   P A R A M E T E R S

DENSITY FUNCTIONAL POTENTIAL (scf)

LDA: Exchange only == Not Default ==

Gradient Corrections: Becke88 LYP == Not Default ==

SPIN (restricted / unrestr.)

Molecule: Restricted

Fragments: Restricted

OTHER ASPECTS

Relativistic Corrections: ---

Nuclear Charge Density Model: Point Charge Nuclei

Core Treatment: Frozen Orbital(s)

Hyperfine or Zeeman Interaction: ---

Settings for Grimme 3 dispersion correction

damping Zero

s6 1.000

sr,6 1.094

s8 1.682

Other (technical) parameters

rs8 1.000

alpha 14.000

version 3

Fragment File(s)

O:

file : t21.O

jobid: ADF 2017 RunTime: Nov10-2021 12:43:15 Nodes: 1 Procs: 1

title: Oxygen (TZ2P, 1s frozen)

C:

file : t21.C

jobid: ADF 2017 RunTime: Nov10-2021 12:43:14 Nodes: 1 Procs: 1

title: Carbon (TZ2P, 1s frozen)

N:

file : t21.N

jobid: ADF 2017 RunTime: Nov10-2021 12:43:13 Nodes: 1 Procs: 1

title: Nitrogen (TZ2P, 1s frozen)

H:

file : t21.H

jobid: ADF 2017 RunTime: Nov10-2021 12:43:12 Nodes: 1 Procs: 1

title: Hydrogen (TZ2P)

****

* R U N T Y P E : GEOMETRY OPTIMIZATION *

****

G E O M E T R Y *** 3D Molecule ***

ATOMS

=====	X Y Z			CHARGE		
	(Angstrom)			Nucl	+Core	At.Mass
	--------------------------			----------------		-------
1 O	14.1276	32.5009	26.6966	8.00	6.00	15.9949
2 C	14.3590	32.7061	28.0748	6.00	4.00	12.0000
3 C	15.8450	32.3433	28.3588	6.00	4.00	12.0000
4 C	16.2219	32.6635	29.8414	6.00	4.00	12.0000
5 O	14.4071	28.0552	22.6792	8.00	6.00	15.9949
6 N	17.6358	32.4633	30.2389	7.00	5.00	14.0031
7 O	14.1092	34.0929	28.3853	8.00	6.00	15.9949
8 C	10.7912	30.9858	25.5262	6.00	4.00	12.0000
9 C	13.4718	34.6885	21.9363	6.00	4.00	12.0000
10 C	11.3802	29.1587	23.7080	6.00	4.00	12.0000
11 O	11.4579	33.3204	24.2591	8.00	6.00	15.9949

12	C	10.2947	30.0201	24.3728	6.00	4.00	12.0000
13	O	11.3176	29.1654	22.3561	8.00	6.00	15.9949
14	C	12.7395	32.5414	26.2266	6.00	4.00	12.0000
15	C	12.7359	33.5003	24.9557	6.00	4.00	12.0000
16	O	9.9123	31.5304	26.2023	8.00	6.00	15.9949
17	C	12.4481	30.1166	27.1415	6.00	4.00	12.0000
18	C	14.4228	28.1163	20.2876	6.00	4.00	12.0000
19	O	15.9926	30.8968	28.1275	8.00	6.00	15.9949
20	C	13.8143	30.2156	21.5819	6.00	4.00	12.0000
21	C	13.7221	28.6741	21.5623	6.00	4.00	12.0000
22	C	12.2730	31.0807	25.9213	6.00	4.00	12.0000
23	C	13.6023	33.2269	22.4183	6.00	4.00	12.0000
24	C	18.6558	33.0430	29.3407	6.00	4.00	12.0000
25	C	13.8682	33.0559	23.9542	6.00	4.00	12.0000
26	C	15.7746	34.1163	30.1770	6.00	4.00	12.0000
27	C	14.3026	34.3821	29.8031	6.00	4.00	12.0000
28	C	13.8675	35.8366	30.0224	6.00	4.00	12.0000
29	C	14.9011	30.9894	21.8352	6.00	4.00	12.0000
30	C	9.3327	30.7533	23.4002	6.00	4.00	12.0000
31	O	15.6540	33.1412	21.1096	8.00	6.00	15.9949
32	C	12.1951	28.2626	21.5212	6.00	4.00	12.0000
33	C	16.3063	30.5133	22.1802	6.00	4.00	12.0000
34	C	14.7738	32.5052	21.7070	6.00	4.00	12.0000
35	C	12.9747	34.9595	25.4175	6.00	4.00	12.0000
36	C	11.9150	26.7680	21.7888	6.00	4.00	12.0000
37	C	17.9936	31.0968	30.6634	6.00	4.00	12.0000
38	C	10.3432	34.1510	24.6660	6.00	4.00	12.0000
39	C	10.4173	26.3825	21.7967	6.00	4.00	12.0000
40	H	12.8813	30.7308	21.3251	1.00	1.00	1.0078
41	H	16.5606	30.8036	23.2169	1.00	1.00	1.0078
42	H	16.4123	29.4252	22.1010	1.00	1.00	1.0078

43	H	17.0333	31.0221	21.5258	1.00	1.00	1.0078
44	H	14.3469	35.2884	22.2367	1.00	1.00	1.0078
45	H	13.4080	34.7323	20.8360	1.00	1.00	1.0078
46	H	12.5636	35.1528	22.3526	1.00	1.00	1.0078
47	H	12.6671	32.6869	22.1954	1.00	1.00	1.0078
48	H	14.7877	33.6030	24.2158	1.00	1.00	1.0078
49	H	14.0977	31.9954	24.1281	1.00	1.00	1.0078
50	H	13.9406	35.0129	25.9368	1.00	1.00	1.0078
51	H	12.2079	35.3024	26.1295	1.00	1.00	1.0078
52	H	13.0000	35.6524	24.5621	1.00	1.00	1.0078
53	H	10.5381	35.2183	24.4599	1.00	1.00	1.0078
54	H	10.0817	34.0166	25.7311	1.00	1.00	1.0078
55	H	9.4899	33.8217	24.0539	1.00	1.00	1.0078
56	H	11.8128	28.5277	20.5224	1.00	1.00	1.0078
57	H	12.4245	26.2124	20.9823	1.00	1.00	1.0078
58	H	12.3902	26.4515	22.7285	1.00	1.00	1.0078
59	H	9.8759	26.8557	22.6328	1.00	1.00	1.0078
60	H	9.9094	26.6714	20.8593	1.00	1.00	1.0078
61	H	10.3130	25.2903	21.9151	1.00	1.00	1.0078
62	H	14.3829	27.0153	20.2707	1.00	1.00	1.0078
63	H	13.9623	28.5151	19.3692	1.00	1.00	1.0078
64	H	15.4804	28.4182	20.2962	1.00	1.00	1.0078
65	H	12.9000	30.6851	25.1118	1.00	1.00	1.0078
66	H	11.8207	30.4303	27.9936	1.00	1.00	1.0078
67	H	13.5020	30.0887	27.4532	1.00	1.00	1.0078
68	H	12.1593	29.0940	26.8516	1.00	1.00	1.0078
69	C	16.7500	30.4831	27.0471	6.00	4.00	12.0000
70	H	14.4781	36.5204	29.4081	1.00	1.00	1.0078
71	H	13.9859	36.1178	31.0826	1.00	1.00	1.0078
72	H	12.8089	35.9709	29.7437	1.00	1.00	1.0078
73	H	13.6440	33.7067	30.3927	1.00	1.00	1.0078

74	H	16.4041	34.8399	29.6297	1.00	1.00	1.0078
75	H	15.9362	34.2855	31.2554	1.00	1.00	1.0078
76	H	18.7935	32.4892	28.3862	1.00	1.00	1.0078
77	H	18.4014	34.0865	29.0941	1.00	1.00	1.0078
78	H	19.6259	33.0575	29.8690	1.00	1.00	1.0078
79	H	18.0781	30.3562	29.8381	1.00	1.00	1.0078
80	H	17.2469	30.7205	31.3828	1.00	1.00	1.0078
81	H	18.9685	31.1318	31.1828	1.00	1.00	1.0078
82	H	13.6894	32.0751	28.6979	1.00	1.00	1.0078
83	H	12.1004	32.9818	27.0094	1.00	1.00	1.0078
84	H	16.4814	32.8860	27.6447	1.00	1.00	1.0078
85	H	15.6293	31.9755	30.4725	1.00	1.00	1.0078
86	H	13.8116	28.1659	23.4630	1.00	1.00	1.0078
87	O	12.1995	28.4936	24.3632	8.00	6.00	15.9949
88	H	9.6967	29.2840	24.9437	1.00	1.00	1.0078
89	H	17.6230	28.5910	26.5007	1.00	1.00	1.0078
90	H	16.3732	28.4646	27.8048	1.00	1.00	1.0078
91	H	15.8977	28.7546	26.1046	1.00	1.00	1.0078
92	C	16.6606	28.9738	26.8726	6.00	4.00	12.0000
93	O	17.4014	31.2316	26.3238	8.00	6.00	15.9949
94	H	9.8711	31.4734	22.7654	1.00	1.00	1.0078
95	H	8.5776	31.2956	23.9887	1.00	1.00	1.0078
96	H	8.8174	30.0328	22.7458	1.00	1.00	1.0078

FRAGMENTS

=========		Atoms in this Fragment		Cart. coord.s (Angstrom)		

1	O	1	O	14.1276	32.5009	26.6966
2	O	5	O	14.4071	28.0552	22.6792
3	O	7	O	14.1092	34.0929	28.3853

4	O	11	O	11.4579	33.3204	24.2591
5	O	13	O	11.3176	29.1654	22.3561
6	O	16	O	9.9123	31.5304	26.2023
7	O	19	O	15.9926	30.8968	28.1275
8	O	31	O	15.6540	33.1412	21.1096
9	O	87	O	12.1995	28.4936	24.3632
10	O	93	O	17.4014	31.2316	26.3238
11	C	2	C	14.3590	32.7061	28.0748
12	C	3	C	15.8450	32.3433	28.3588
13	C	4	C	16.2219	32.6635	29.8414
14	C	8	C	10.7912	30.9858	25.5262
15	C	9	C	13.4718	34.6885	21.9363
16	C	10	C	11.3802	29.1587	23.7080
17	C	12	C	10.2947	30.0201	24.3728
18	C	14	C	12.7395	32.5414	26.2266
19	C	15	C	12.7359	33.5003	24.9557
20	C	17	C	12.4481	30.1166	27.1415
21	C	18	C	14.4228	28.1163	20.2876
22	C	20	C	13.8143	30.2156	21.5819
23	C	21	C	13.7221	28.6741	21.5623
24	C	22	C	12.2730	31.0807	25.9213
25	C	23	C	13.6023	33.2269	22.4183
26	C	24	C	18.6558	33.0430	29.3407
27	C	25	C	13.8682	33.0559	23.9542
28	C	26	C	15.7746	34.1163	30.1770
29	C	27	C	14.3026	34.3821	29.8031
30	C	28	C	13.8675	35.8366	30.0224
31	C	29	C	14.9011	30.9894	21.8352
32	C	30	C	9.3327	30.7533	23.4002
33	C	32	C	12.1951	28.2626	21.5212
34	C	33	C	16.3063	30.5133	22.1802

35	C	34	C	14.7738	32.5052	21.7070
36	C	35	C	12.9747	34.9595	25.4175
37	C	36	C	11.9150	26.7680	21.7888
38	C	37	C	17.9936	31.0968	30.6634
39	C	38	C	10.3432	34.1510	24.6660
40	C	39	C	10.4173	26.3825	21.7967
41	C	69	C	16.7500	30.4831	27.0471
42	C	92	C	16.6606	28.9738	26.8726
43	N	6	N	17.6358	32.4633	30.2389
44	H	40	H	12.8813	30.7308	21.3251
45	H	41	H	16.5606	30.8036	23.2169
46	H	42	H	16.4123	29.4252	22.1010
47	H	43	H	17.0333	31.0221	21.5258
48	H	44	H	14.3469	35.2884	22.2367
49	H	45	H	13.4080	34.7323	20.8360
50	H	46	H	12.5636	35.1528	22.3526
51	H	47	H	12.6671	32.6869	22.1954
52	H	48	H	14.7877	33.6030	24.2158
53	H	49	H	14.0977	31.9954	24.1281
54	H	50	H	13.9406	35.0129	25.9368
55	H	51	H	12.2079	35.3024	26.1295
56	H	52	H	13.0000	35.6524	24.5621
57	H	53	H	10.5381	35.2183	24.4599
58	H	54	H	10.0817	34.0166	25.7311
59	H	55	H	9.4899	33.8217	24.0539
60	H	56	H	11.8128	28.5277	20.5224
61	H	57	H	12.4245	26.2124	20.9823
62	H	58	H	12.3902	26.4515	22.7285
63	H	59	H	9.8759	26.8557	22.6328
64	H	60	H	9.9094	26.6714	20.8593
65	H	61	H	10.3130	25.2903	21.9151

66	H	62	H	14.3829	27.0153	20.2707
67	H	63	H	13.9623	28.5151	19.3692
68	H	64	H	15.4804	28.4182	20.2962
69	H	65	H	12.9000	30.6851	25.1118
70	H	66	H	11.8207	30.4303	27.9936
71	H	67	H	13.5020	30.0887	27.4532
72	H	68	H	12.1593	29.0940	26.8516
73	H	70	H	14.4781	36.5204	29.4081
74	H	71	H	13.9859	36.1178	31.0826
75	H	72	H	12.8089	35.9709	29.7437
76	H	73	H	13.6440	33.7067	30.3927
77	H	74	H	16.4041	34.8399	29.6297
78	H	75	H	15.9362	34.2855	31.2554
79	H	76	H	18.7935	32.4892	28.3862
80	H	77	H	18.4014	34.0865	29.0941
81	H	78	H	19.6259	33.0575	29.8690
82	H	79	H	18.0781	30.3562	29.8381
83	H	80	H	17.2469	30.7205	31.3828
84	H	81	H	18.9685	31.1318	31.1828
85	H	82	H	13.6894	32.0751	28.6979
86	H	83	H	12.1004	32.9818	27.0094
87	H	84	H	16.4814	32.8860	27.6447
88	H	85	H	15.6293	31.9755	30.4725
89	H	86	H	13.8116	28.1659	23.4630
90	H	88	H	9.6967	29.2840	24.9437
91	H	89	H	17.6230	28.5910	26.5007
92	H	90	H	16.3732	28.4646	27.8048
93	H	91	H	15.8977	28.7546	26.1046
94	H	94	H	9.8711	31.4734	22.7654
95	H	95	H	8.5776	31.2956	23.9887
96	H	96	H	8.8174	30.0328	22.7458

S Y M M E T R Y ,   E L E C T R O N S

Symmetry: NOSYM

Irreducible Representations, including subspecies

A

Configuration of Valence Electrons

=================================

(determined in the SCF procedure)

Total: 246

Net Charge: 0 (Nuclei minus Electrons)

Aufbau principle for MO occupations will be applied through SCF cycle no.   150

Thereafter, the program will assign electrons to MOs that are spatially

similar to the occupied MOs in a "reference" cycle ("KeepOrbitals").

The reference cycle is always the PREVIOUS cycle: it will evolve with

the SCF procedure.

1

****
****

****

* B U I L D : (Fragments, Functions) *

****

S F O s *** (Symmetrized Fragment Orbitals) ***

SFOs are linear combinations of (valence) Fragment Orbitals (FOs), such that the SFOs transform as the

irreducible representations of the (molecular) symmetry group. Each SFO is therefore characterized by

an irrep of the molecule and by a few (or only one) generating FOs.

The SFOs constitute a symmetry-adapted basis for the Fock matrix. The MO eigenvector coefficients in

this basis provide a direct interpretation of the MOs in terms of Frontier Orbital Theory.

The SFOs are combined with auxiliary Core Functions (CFs) to ensure orthogonalization on the (frozen)

Core Orbitals (COs). The Core-orthogonalized SFOs (CSFOs) constitute the true Fock basis.

The FOs, and hence also the (C)SFOs are combinations of the elementary basis functions (BAS). The basis

functions that participate in the description of the SFOs depend on the irrep. The indices of the

involved functions are printed below for each irrep.

(The complete list of primitive basis functions is printed in another section)

Total nr. of (C)SFOs (summation over all irreps) : 1615

NOTE: a (C)SFO that is defined as a combination of more than one FO is usually NOT normalized.

=== A ===

Nr. of SFOs : 1615

Cartesian basis functions that participate in this irrep (total number = 1883) :

1 30 59 88 117 146 175 204 233 262

291 320 349 378 407 436 465 494 523 552

581 610 639 668 697 726 755 784 813 842

871 900 929 958 987 1016 1045 1074 1103 1132

1161 1190 1219 2 3 4 5 8 11 6

9 12 7 10 13 14 15 16 17 18

19 20 21 22 23 24 25 26 27 28

29 31 32 33 34 37 40 35 38 41

36 39 42 43 44 45 46 47 48 49

50 51 52 53 54 55 56 57 58 60

61 62 63 66 69 64 67 70 65 68

71 72 73 74 75 76 77 78 79 80

81 82 83 84 85 86 87 89 90 91

92 95 98 93 96 99 94 97 100 101

102 103 104 105 106 107 108 109 110 111

112 113 114 115 116 118 119 120 121 124

127 122 125 128 123 126 129 130 131 132

133 134 135 136 137 138 139 140 141 142

143 144 145 147 148 149 150 153 156 151

154 157 152 155 158 159 160 161 162 163

164 165 166 167 168 169 170 171 172 173

174 176 177 178 179 182 185 180 183 186

181 184 187 188 189 190 191 192 193 194

195	196	197	198	199	200	201	202	203	205
206	207	208	211	214	209	212	215	210	213
216	217	218	219	220	221	222	223	224	225
226	227	228	229	230	231	232	234	235	236
237	240	243	238	241	244	239	242	245	246
247	248	249	250	251	252	253	254	255	256
257	258	259	260	261	263	264	265	266	269
272	267	270	273	268	271	274	275	276	277
278	279	280	281	282	283	284	285	286	287
288	289	290	292	293	294	295	298	301	296
299	302	297	300	303	304	305	306	307	308
309	310	311	312	313	314	315	316	317	318
319	321	322	323	324	327	330	325	328	331
326	329	332	333	334	335	336	337	338	339
340	341	342	343	344	345	346	347	348	350
351	352	353	356	359	354	357	360	355	358
361	362	363	364	365	366	367	368	369	370
371	372	373	374	375	376	377	379	380	381
382	385	388	383	386	389	384	387	390	391
392	393	394	395	396	397	398	399	400	401
402	403	404	405	406	408	409	410	411	414
417	412	415	418	413	416	419	420	421	422
423	424	425	426	427	428	429	430	431	432
433	434	435	437	438	439	440	443	446	441
444	447	442	445	448	449	450	451	452	453
454	455	456	457	458	459	460	461	462	463
464	466	467	468	469	472	475	470	473	476
471	474	477	478	479	480	481	482	483	484
485	486	487	488	489	490	491	492	493	495
496	497	498	501	504	499	502	505	500	503
506	507	508	509	510	511	512	513	514	515

516	517	518	519	520	521	522	524	525	526
527	530	533	528	531	534	529	532	535	536
537	538	539	540	541	542	543	544	545	546
547	548	549	550	551	553	554	555	556	559
562	557	560	563	558	561	564	565	566	567
568	569	570	571	572	573	574	575	576	577
578	579	580	582	583	584	585	588	591	586
589	592	587	590	593	594	595	596	597	598
599	600	601	602	603	604	605	606	607	608
609	611	612	613	614	617	620	615	618	621
616	619	622	623	624	625	626	627	628	629
630	631	632	633	634	635	636	637	638	640
641	642	643	646	649	644	647	650	645	648
651	652	653	654	655	656	657	658	659	660
661	662	663	664	665	666	667	669	670	671
672	675	678	673	676	679	674	677	680	681
682	683	684	685	686	687	688	689	690	691
692	693	694	695	696	698	699	700	701	704
707	702	705	708	703	706	709	710	711	712
713	714	715	716	717	718	719	720	721	722
723	724	725	727	728	729	730	733	736	731
734	737	732	735	738	739	740	741	742	743
744	745	746	747	748	749	750	751	752	753
754	756	757	758	759	762	765	760	763	766
761	764	767	768	769	770	771	772	773	774
775	776	777	778	779	780	781	782	783	785
786	787	788	791	794	789	792	795	790	793
796	797	798	799	800	801	802	803	804	805
806	807	808	809	810	811	812	814	815	816
817	820	823	818	821	824	819	822	825	826
827	828	829	830	831	832	833	834	835	836

837 838 839 840 841 843 844 845 846 849

852 847 850 853 848 851 854 855 856 857

858 859 860 861 862 863 864 865 866 867

868 869 870 872 873 874 875 878 881 876

879 882 877 880 883 884 885 886 887 888

889 890 891 892 893 894 895 896 897 898

899 901 902 903 904 907 910 905 908 911

906 909 912 913 914 915 916 917 918 919

920 921 922 923 924 925 926 927 928 930

931 932 933 936 939 934 937 940 935 938

941 942 943 944 945 946 947 948 949 950

951 952 953 954 955 956 957 959 960 961

962 965 968 963 966 969 964 967 970 971

972 973 974 975 976 977 978 979 980 981

982 983 984 985 986 988 989 990 991 994

997 992 995 998 993 996 999 1000 1001 1002

1003 1004 1005 1006 1007 1008 1009 1010 1011 1012

1013 1014 1015 1017 1018 1019 1020 1023 1026 1021

1024 1027 1022 1025 1028 1029 1030 1031 1032 1033

1034 1035 1036 1037 1038 1039 1040 1041 1042 1043

1044 1046 1047 1048 1049 1052 1055 1050 1053 1056

1051 1054 1057 1058 1059 1060 1061 1062 1063 1064

1065 1066 1067 1068 1069 1070 1071 1072 1073 1075

1076 1077 1078 1081 1084 1079 1082 1085 1080 1083

1086 1087 1088 1089 1090 1091 1092 1093 1094 1095

1096 1097 1098 1099 1100 1101 1102 1104 1105 1106

1107 1110 1113 1108 1111 1114 1109 1112 1115 1116

1117 1118 1119 1120 1121 1122 1123 1124 1125 1126

1127 1128 1129 1130 1131 1133 1134 1135 1136 1139

1142 1137 1140 1143 1138 1141 1144 1145 1146 1147

1148 1149 1150 1151 1152 1153 1154 1155 1156 1157

1158 1159 1160 1162 1163 1164 1165 1168 1171 1166

1169 1172 1167 1170 1173 1174 1175 1176 1177 1178

1179 1180 1181 1182 1183 1184 1185 1186 1187 1188

1189 1191 1192 1193 1194 1197 1200 1195 1198 1201

1196 1199 1202 1203 1204 1205 1206 1207 1208 1209

1210 1211 1212 1213 1214 1215 1216 1217 1218 1220

1221 1222 1223 1226 1229 1224 1227 1230 1225 1228

1231 1232 1233 1234 1235 1236 1237 1238 1239 1240

1241 1242 1243 1244 1245 1246 1247 1248 1249 1250

1251 1252 1253 1254 1255 1256 1257 1258 1259 1260

1261 1262 1263 1264 1265 1266 1267 1268 1269 1270

1271 1272 1273 1274 1275 1276 1277 1278 1279 1280

1281 1282 1283 1284 1285 1286 1287 1288 1289 1290

1291 1292 1293 1294 1295 1296 1297 1298 1299 1300

1301 1302 1303 1304 1305 1306 1307 1308 1309 1310

1311 1312 1313 1314 1315 1316 1317 1318 1319 1320

1321 1322 1323 1324 1325 1326 1327 1328 1329 1330

1331 1332 1333 1334 1335 1336 1337 1338 1339 1340

1341 1342 1343 1344 1345 1346 1347 1348 1349 1350

1351 1352 1353 1354 1355 1356 1357 1358 1359 1360

1361 1362 1363 1364 1365 1366 1367 1368 1369 1370

1371 1372 1373 1374 1375 1376 1377 1378 1379 1380

1381 1382 1383 1384 1385 1386 1387 1388 1389 1390

1391 1392 1393 1394 1395 1396 1397 1398 1399 1400

1401 1402 1403 1404 1405 1406 1407 1408 1409 1410

1411 1412 1413 1414 1415 1416 1417 1418 1419 1420

1421 1422 1423 1424 1425 1426 1427 1428 1429 1430

1431 1432 1433 1434 1435 1436 1437 1438 1439 1440

1441 1442 1443 1444 1445 1446 1447 1448 1449 1450

1451 1452 1453 1454 1455 1456 1457 1458 1459 1460

1461 1462 1463 1464 1465 1466 1467 1468 1469 1470

1471 1472 1473 1474 1475 1476 1477 1478 1479 1480

1481 1482 1483 1484 1485 1486 1487 1488 1489 1490

1491 1492 1493 1494 1495 1496 1497 1498 1499 1500

1501 1502 1503 1504 1505 1506 1507 1508 1509 1510

1511 1512 1513 1514 1515 1516 1517 1518 1519 1520

1521 1522 1523 1524 1525 1526 1527 1528 1529 1530

1531 1532 1533 1534 1535 1536 1537 1538 1539 1540

1541 1542 1543 1544 1545 1546 1547 1548 1549 1550

1551 1552 1553 1554 1555 1556 1557 1558 1559 1560

1561 1562 1563 1564 1565 1566 1567 1568 1569 1570

1571 1572 1573 1574 1575 1576 1577 1578 1579 1580

1581 1582 1583 1584 1585 1586 1587 1588 1589 1590

1591 1592 1593 1594 1595 1596 1597 1598 1599 1600

1601 1602 1603 1604 1605 1606 1607 1608 1609 1610

1611 1612 1613 1614 1615 1616 1617 1618 1619 1620

1621 1622 1623 1624 1625 1626 1627 1628 1629 1630

1631 1632 1633 1634 1635 1636 1637 1638 1639 1640

1641 1642 1643 1644 1645 1646 1647 1648 1649 1650

1651 1652 1653 1654 1655 1656 1657 1658 1659 1660

1661 1662 1663 1664 1665 1666 1667 1668 1669 1670

1671 1672 1673 1674 1675 1676 1677 1678 1679 1680

1681 1682 1683 1684 1685 1686 1687 1688 1689 1690

1691 1692 1693 1694 1695 1696 1697 1698 1699 1700

1701 1702 1703 1704 1705 1706 1707 1708 1709 1710

1711 1712 1713 1714 1715 1716 1717 1718 1719 1720

1721 1722 1723 1724 1725 1726 1727 1728 1729 1730

1731 1732 1733 1734 1735 1736 1737 1738 1739 1740

1741 1742 1743 1744 1745 1746 1747 1748 1749 1750

1751 1752 1753 1754 1755 1756 1757 1758 1759 1760

1761 1762 1763 1764 1765 1766 1767 1768 1769 1770

1771 1772 1773 1774 1775 1776 1777 1778 1779 1780

1781 1782 1783 1784 1785 1786 1787 1788 1789 1790
1791 1792 1793 1794 1795 1796 1797 1798 1799 1800
1801 1802 1803 1804 1805 1806 1807 1808 1809 1810
1811 1812 1813 1814 1815 1816 1817 1818 1819 1820
1821 1822 1823 1824 1825 1826 1827 1828 1829 1830
1831 1832 1833 1834 1835 1836 1837 1838 1839 1840
1841 1842 1843 1844 1845 1846 1847 1848 1849 1850
1851 1852 1853 1854 1855 1856 1857 1858 1859 1860
1861 1862 1863 1864 1865 1866 1867 1868 1869 1870
1871 1872 1873 1874 1875 1876 1877 1878 1879 1880
1881 1882 1883

SFO (index Fragment Generating Expansion in Fragment Orbitals

indx incl.CFs) Occup Orb.Energy FragmentType Coeff. Orbital on Fragment

----

1 44 2.000 -0.875 au O 1.00 1 S 1

(-23.822 eV)

2 45 -- 0.903 au O 1.00 2 S 1

(24.573 eV)

3 46 -- 29.786 au O 1.00 3 S 1

(810.511 eV)

4 47 1.333 -0.333 au O 1.00 1 P:x 1

(-9.066 eV)

5 48 -- 0.346 au O 1.00 2 P:x 1

(9.420 eV)

6 49 -- 3.822 au O 1.00 3 P:x 1

(104.006 eV)

7 50 1.333 -0.333 au O 1.00 1 P:y 1

(-9.066 eV)

8 51 -- 0.346 au O 1.00 2 P:y 1

(9.420 eV)

9 52 -- 3.822 au O 1.00 3 P:y 1

(104.006 eV)

10 53 1.333 -0.333 au O 1.00 1 P:z 1

(-9.066 eV)

11 54 -- 0.346 au O 1.00 2 P:z 1

(9.420 eV)

12 55 -- 3.822 au O 1.00 3 P:z 1

(104.006 eV)

13 56 -- 1.070 au O 1.00 1 D:z2 1

(29.103 eV)

14 57 -- 1.070 au O 1.00 1 D:x2-y2 1

(29.103 eV)

15 58 -- 1.070 au O 1.00 1 D:xy 1

(29.103 eV)

16 59 -- 1.070 au O 1.00 1 D:xz 1

(29.103 eV)

17 60 -- 1.070 au O 1.00 1 D:yz 1

(29.103 eV)

18 61 -- 3.358 au O 1.00 1 F:z3 1

(91.384 eV)

19 62 -- 3.358 au O 1.00 1 F:z 1

(91.384 eV)

20 63 -- 3.358 au O 1.00 1 F:xyz 1

(91.384 eV)

21 64 -- 3.358 au O 1.00 1 F:z2x 1

(91.384 eV)

22 65 -- 3.358 au O 1.00 1 F:z2y 1

(91.384 eV)

23 66 -- 3.358 au O 1.00 1 F:x 1

(91.384 eV)

24 67 -- 3.358 au O 1.00 1 F:y 1

		(91.384 eV)					
25	68	2.000	-0.875 au	O	1.00	1 S	2
		(-23.822 eV)					
26	69	--	0.903 au	O	1.00	2 S	2
		(24.573 eV)					
27	70	--	29.786 au	O	1.00	3 S	2
		(810.511 eV)					
28	71	1.333	-0.333 au	O	1.00	1 P:x	2
		(-9.066 eV)					
29	72	--	0.346 au	O	1.00	2 P:x	2
		(9.420 eV)					
30	73	--	3.822 au	O	1.00	3 P:x	2
		(104.006 eV)					
31	74	1.333	-0.333 au	O	1.00	1 P:y	2
		(-9.066 eV)					
32	75	--	0.346 au	O	1.00	2 P:y	2
		(9.420 eV)					
33	76	--	3.822 au	O	1.00	3 P:y	2
		(104.006 eV)					
34	77	1.333	-0.333 au	O	1.00	1 P:z	2
		(-9.066 eV)					
35	78	--	0.346 au	O	1.00	2 P:z	2
		(9.420 eV)					
36	79	--	3.822 au	O	1.00	3 P:z	2
		(104.006 eV)					
37	80	--	1.070 au	O	1.00	1 D:z2	2
		(29.103 eV)					
38	81	--	1.070 au	O	1.00	1 D:x2-y2	2
		(29.103 eV)					
39	82	--	1.070 au	O	1.00	1 D:xy	2
		(29.103 eV)					

40 83 -- 1.070 au O 1.00 1 D:xz 2

(29.103 eV)

41 84 -- 1.070 au O 1.00 1 D:yz 2

(29.103 eV)

42 85 -- 3.358 au O 1.00 1 F:z3 2

(91.384 eV)

43 86 -- 3.358 au O 1.00 1 F:z 2

(91.384 eV)

44 87 -- 3.358 au O 1.00 1 F:xyz 2

(91.384 eV)

45 88 -- 3.358 au O 1.00 1 F:z2x 2

(91.384 eV)

46 89 -- 3.358 au O 1.00 1 F:z2y 2

(91.384 eV)

47 90 -- 3.358 au O 1.00 1 F:x 2

(91.384 eV)

48 91 -- 3.358 au O 1.00 1 F:y 2

(91.384 eV)

49 92 2.000 -0.875 au O 1.00 1 S 3

(-23.822 eV)

50 93 -- 0.903 au O 1.00 2 S 3

(24.573 eV)

51 94 -- 29.786 au O 1.00 3 S 3

(810.511 eV)

52 95 1.333 -0.333 au O 1.00 1 P:x 3

(-9.066 eV)

53 96 -- 0.346 au O 1.00 2 P:x 3

(9.420 eV)

54 97 -- 3.822 au O 1.00 3 P:x 3

(104.006 eV)

55 98 1.333 -0.333 au O 1.00 1 P:y 3

(-9.066 eV)

56 99 -- 0.346 au O 1.00 2 P:y 3

(9.420 eV)

57 100 -- 3.822 au O 1.00 3 P:y 3

(104.006 eV)

58 101 1.333 -0.333 au O 1.00 1 P:z 3

(-9.066 eV)

59 102 -- 0.346 au O 1.00 2 P:z 3

(9.420 eV)

60 103 -- 3.822 au O 1.00 3 P:z 3

(104.006 eV)

61 104 -- 1.070 au O 1.00 1 D:z2 3

(29.103 eV)

62 105 -- 1.070 au O 1.00 1 D:x2-y2 3

(29.103 eV)

63 106 -- 1.070 au O 1.00 1 D:xy 3

(29.103 eV)

64 107 -- 1.070 au O 1.00 1 D:xz 3

(29.103 eV)

65 108 -- 1.070 au O 1.00 1 D:yz 3

(29.103 eV)

66 109 -- 3.358 au O 1.00 1 F:z3 3

(91.384 eV)

67 110 -- 3.358 au O 1.00 1 F:z 3

(91.384 eV)

68 111 -- 3.358 au O 1.00 1 F:xyz 3

(91.384 eV)

69 112 -- 3.358 au O 1.00 1 F:z2x 3

(91.384 eV)

70 113 -- 3.358 au O 1.00 1 F:z2y 3

(91.384 eV)

71 114 -- 3.358 au O 1.00 1 F:x 3
(91.384 eV)
72 115 -- 3.358 au O 1.00 1 F:y 3
(91.384 eV)
73 116 2.000 -0.875 au O 1.00 1 S 4
(-23.822 eV)
74 117 -- 0.903 au O 1.00 2 S 4
(24.573 eV)
75 118 -- 29.786 au O 1.00 3 S 4
(810.511 eV)
76 119 1.333 -0.333 au O 1.00 1 P:x 4
(-9.066 eV)
77 120 -- 0.346 au O 1.00 2 P:x 4
(9.420 eV)
78 121 -- 3.822 au O 1.00 3 P:x 4
(104.006 eV)
79 122 1.333 -0.333 au O 1.00 1 P:y 4
(-9.066 eV)
80 123 -- 0.346 au O 1.00 2 P:y 4
(9.420 eV)
81 124 -- 3.822 au O 1.00 3 P:y 4
(104.006 eV)
82 125 1.333 -0.333 au O 1.00 1 P:z 4
(-9.066 eV)
83 126 -- 0.346 au O 1.00 2 P:z 4
(9.420 eV)
84 127 -- 3.822 au O 1.00 3 P:z 4
(104.006 eV)
85 128 -- 1.070 au O 1.00 1 D:z2 4
(29.103 eV)
86 129 -- 1.070 au O 1.00 1 D:x2-y2 4

(29.103 eV)

87 130 -- 1.070 au O 1.00 1 D:xy 4

(29.103 eV)

88 131 -- 1.070 au O 1.00 1 D:xz 4

(29.103 eV)

89 132 -- 1.070 au O 1.00 1 D:yz 4

(29.103 eV)

90 133 -- 3.358 au O 1.00 1 F:z3 4

(91.384 eV)

91 134 -- 3.358 au O 1.00 1 F:z 4

(91.384 eV)

92 135 -- 3.358 au O 1.00 1 F:xyz 4

(91.384 eV)

93 136 -- 3.358 au O 1.00 1 F:z2x 4

(91.384 eV)

94 137 -- 3.358 au O 1.00 1 F:z2y 4

(91.384 eV)

95 138 -- 3.358 au O 1.00 1 F:x 4

(91.384 eV)

96 139 -- 3.358 au O 1.00 1 F:y 4

(91.384 eV)

97 140 2.000 -0.875 au O 1.00 1 S 5

(-23.822 eV)

98 141 -- 0.903 au O 1.00 2 S 5

(24.573 eV)

99 142 -- 29.786 au O 1.00 3 S 5

(810.511 eV)

100 143 1.333 -0.333 au O 1.00 1 P:x 5

(-9.066 eV)

101 144 -- 0.346 au O 1.00 2 P:x 5

(9.420 eV)

102 145 -- 3.822 au O 1.00 3 P:x 5

(104.006 eV)

103 146 1.333 -0.333 au O 1.00 1 P:y 5

(-9.066 eV)

104 147 -- 0.346 au O 1.00 2 P:y 5

(9.420 eV)

105 148 -- 3.822 au O 1.00 3 P:y 5

(104.006 eV)

106 149 1.333 -0.333 au O 1.00 1 P:z 5

(-9.066 eV)

107 150 -- 0.346 au O 1.00 2 P:z 5

(9.420 eV)

108 151 -- 3.822 au O 1.00 3 P:z 5

(104.006 eV)

109 152 -- 1.070 au O 1.00 1 D:z2 5

(29.103 eV)

110 153 -- 1.070 au O 1.00 1 D:x2-y2 5

(29.103 eV)

111 154 -- 1.070 au O 1.00 1 D:xy 5

(29.103 eV)

112 155 -- 1.070 au O 1.00 1 D:xz 5

(29.103 eV)

113 156 -- 1.070 au O 1.00 1 D:yz 5

(29.103 eV)

114 157 -- 3.358 au O 1.00 1 F:z3 5

(91.384 eV)

115 158 -- 3.358 au O 1.00 1 F:z 5

(91.384 eV)

116 159 -- 3.358 au O 1.00 1 F:xyz 5

(91.384 eV)

117 160 -- 3.358 au O 1.00 1 F:z2x 5

(91.384 eV)

118 161 -- 3.358 au O 1.00 1 F:z2y 5

(91.384 eV)

119 162 -- 3.358 au O 1.00 1 F:x 5

(91.384 eV)

120 163 -- 3.358 au O 1.00 1 F:y 5

(91.384 eV)

121 164 2.000 -0.875 au O 1.00 1 S 6

(-23.822 eV)

122 165 -- 0.903 au O 1.00 2 S 6

(24.573 eV)

123 166 -- 29.786 au O 1.00 3 S 6

(810.511 eV)

124 167 1.333 -0.333 au O 1.00 1 P:x 6

(-9.066 eV)

125 168 -- 0.346 au O 1.00 2 P:x 6

(9.420 eV)

126 169 -- 3.822 au O 1.00 3 P:x 6

(104.006 eV)

127 170 1.333 -0.333 au O 1.00 1 P:y 6

(-9.066 eV)

128 171 -- 0.346 au O 1.00 2 P:y 6

(9.420 eV)

129 172 -- 3.822 au O 1.00 3 P:y 6

(104.006 eV)

130 173 1.333 -0.333 au O 1.00 1 P:z 6

(-9.066 eV)

131 174 -- 0.346 au O 1.00 2 P:z 6

(9.420 eV)

132 175 -- 3.822 au O 1.00 3 P:z 6

(104.006 eV)

133 176 -- 1.070 au O 1.00 1 D:z2 6

(29.103 eV)

134 177 -- 1.070 au O 1.00 1 D:x2-y2 6

(29.103 eV)

135 178 -- 1.070 au O 1.00 1 D:xy 6

(29.103 eV)

136 179 -- 1.070 au O 1.00 1 D:xz 6

(29.103 eV)

137 180 -- 1.070 au O 1.00 1 D:yz 6

(29.103 eV)

138 181 -- 3.358 au O 1.00 1 F:z3 6

(91.384 eV)

139 182 -- 3.358 au O 1.00 1 F:z 6

(91.384 eV)

140 183 -- 3.358 au O 1.00 1 F:xyz 6

(91.384 eV)

141 184 -- 3.358 au O 1.00 1 F:z2x 6

(91.384 eV)

142 185 -- 3.358 au O 1.00 1 F:z2y 6

(91.384 eV)

143 186 -- 3.358 au O 1.00 1 F:x 6

(91.384 eV)

144 187 -- 3.358 au O 1.00 1 F:y 6

(91.384 eV)

145 188 2.000 -0.875 au O 1.00 1 S 7

(-23.822 eV)

146 189 -- 0.903 au O 1.00 2 S 7

(24.573 eV)

147 190 -- 29.786 au O 1.00 3 S 7

(810.511 eV)

148 191 1.333 -0.333 au O 1.00 1 P:x 7

(-9.066 eV)

149 192 -- 0.346 au O 1.00 2 P:x 7

(9.420 eV)

150 193 -- 3.822 au O 1.00 3 P:x 7

(104.006 eV)

151 194 1.333 -0.333 au O 1.00 1 P:y 7

(-9.066 eV)

152 195 -- 0.346 au O 1.00 2 P:y 7

(9.420 eV)

153 196 -- 3.822 au O 1.00 3 P:y 7

(104.006 eV)

154 197 1.333 -0.333 au O 1.00 1 P:z 7

(-9.066 eV)

155 198 -- 0.346 au O 1.00 2 P:z 7

(9.420 eV)

156 199 -- 3.822 au O 1.00 3 P:z 7

(104.006 eV)

157 200 -- 1.070 au O 1.00 1 D:z2 7

(29.103 eV)

158 201 -- 1.070 au O 1.00 1 D:x2-y2 7

(29.103 eV)

159 202 -- 1.070 au O 1.00 1 D:xy 7

(29.103 eV)

160 203 -- 1.070 au O 1.00 1 D:xz 7

(29.103 eV)

161 204 -- 1.070 au O 1.00 1 D:yz 7

(29.103 eV)

162 205 -- 3.358 au O 1.00 1 F:z3 7

(91.384 eV)

163 206 -- 3.358 au O 1.00 1 F:z 7

(91.384 eV)

164 207 -- 3.358 au O 1.00 1 F:xyz 7

(91.384 eV)

165 208 -- 3.358 au O 1.00 1 F:z2x 7

(91.384 eV)

166 209 -- 3.358 au O 1.00 1 F:z2y 7

(91.384 eV)

167 210 -- 3.358 au O 1.00 1 F:x 7

(91.384 eV)

168 211 -- 3.358 au O 1.00 1 F:y 7

(91.384 eV)

169 212 2.000 -0.875 au O 1.00 1 S 8

(-23.822 eV)

170 213 -- 0.903 au O 1.00 2 S 8

(24.573 eV)

171 214 -- 29.786 au O 1.00 3 S 8

(810.511 eV)

172 215 1.333 -0.333 au O 1.00 1 P:x 8

(-9.066 eV)

173 216 -- 0.346 au O 1.00 2 P:x 8

(9.420 eV)

174 217 -- 3.822 au O 1.00 3 P:x 8

(104.006 eV)

175 218 1.333 -0.333 au O 1.00 1 P:y 8

(-9.066 eV)

176 219 -- 0.346 au O 1.00 2 P:y 8

(9.420 eV)

177 220 -- 3.822 au O 1.00 3 P:y 8

(104.006 eV)

178 221 1.333 -0.333 au O 1.00 1 P:z 8

(-9.066 eV)

179 222 -- 0.346 au O 1.00 2 P:z 8

(9.420 eV)

180 223 -- 3.822 au O 1.00 3 P:z 8

(104.006 eV)

181 224 -- 1.070 au O 1.00 1 D:z2 8

(29.103 eV)

182 225 -- 1.070 au O 1.00 1 D:x2-y2 8

(29.103 eV)

183 226 -- 1.070 au O 1.00 1 D:xy 8

(29.103 eV)

184 227 -- 1.070 au O 1.00 1 D:xz 8

(29.103 eV)

185 228 -- 1.070 au O 1.00 1 D:yz 8

(29.103 eV)

186 229 -- 3.358 au O 1.00 1 F:z3 8

(91.384 eV)

187 230 -- 3.358 au O 1.00 1 F:z 8

(91.384 eV)

188 231 -- 3.358 au O 1.00 1 F:xyz 8

(91.384 eV)

189 232 -- 3.358 au O 1.00 1 F:z2x 8

(91.384 eV)

190 233 -- 3.358 au O 1.00 1 F:z2y 8

(91.384 eV)

191 234 -- 3.358 au O 1.00 1 F:x 8

(91.384 eV)

192 235 -- 3.358 au O 1.00 1 F:y 8

(91.384 eV)

193 236 2.000 -0.875 au O 1.00 1 S 9

(-23.822 eV)

194 237 -- 0.903 au O 1.00 2 S 9

(24.573 eV)

195 238 -- 29.786 au O 1.00 3 S 9

(810.511 eV)

196 239 1.333 -0.333 au O 1.00 1 P:x 9

(-9.066 eV)

197 240 -- 0.346 au O 1.00 2 P:x 9

(9.420 eV)

198 241 -- 3.822 au O 1.00 3 P:x 9

(104.006 eV)

199 242 1.333 -0.333 au O 1.00 1 P:y 9

(-9.066 eV)

200 243 -- 0.346 au O 1.00 2 P:y 9

(9.420 eV)

201 244 -- 3.822 au O 1.00 3 P:y 9

(104.006 eV)

202 245 1.333 -0.333 au O 1.00 1 P:z 9

(-9.066 eV)

203 246 -- 0.346 au O 1.00 2 P:z 9

(9.420 eV)

204 247 -- 3.822 au O 1.00 3 P:z 9

(104.006 eV)

205 248 -- 1.070 au O 1.00 1 D:z2 9

(29.103 eV)

206 249 -- 1.070 au O 1.00 1 D:x2-y2 9

(29.103 eV)

207 250 -- 1.070 au O 1.00 1 D:xy 9

(29.103 eV)

208 251 -- 1.070 au O 1.00 1 D:xz 9

(29.103 eV)

209 252 -- 1.070 au O 1.00 1 D:yz 9

(29.103 eV)

210 253 -- 3.358 au O 1.00 1 F:z3 9

(91.384 eV)

211 254 -- 3.358 au O 1.00 1 F:z 9

(91.384 eV)

212 255 -- 3.358 au O 1.00 1 F:xyz 9

(91.384 eV)

213 256 -- 3.358 au O 1.00 1 F:z2x 9

(91.384 eV)

214 257 -- 3.358 au O 1.00 1 F:z2y 9

(91.384 eV)

215 258 -- 3.358 au O 1.00 1 F:x 9

(91.384 eV)

216 259 -- 3.358 au O 1.00 1 F:y 9

(91.384 eV)

217 260 2.000 -0.875 au O 1.00 1 S 10

(-23.822 eV)

218 261 -- 0.903 au O 1.00 2 S 10

(24.573 eV)

219 262 -- 29.786 au O 1.00 3 S 10

(810.511 eV)

220 263 1.333 -0.333 au O 1.00 1 P:x 10

(-9.066 eV)

221 264 -- 0.346 au O 1.00 2 P:x 10

(9.420 eV)

222 265 -- 3.822 au O 1.00 3 P:x 10

(104.006 eV)

223 266 1.333 -0.333 au O 1.00 1 P:y 10

(-9.066 eV)

224 267 -- 0.346 au O 1.00 2 P:y 10

(9.420 eV)

225 268 -- 3.822 au O 1.00 3 P:y 10

(104.006 eV)

226 269 1.333 -0.333 au O 1.00 1 P:z 10

(-9.066 eV)

227 270 -- 0.346 au O 1.00 2 P:z 10

(9.420 eV)

228 271 -- 3.822 au O 1.00 3 P:z 10

(104.006 eV)

229 272 -- 1.070 au O 1.00 1 D:z2 10

(29.103 eV)

230 273 -- 1.070 au O 1.00 1 D:x2-y2 10

(29.103 eV)

231 274 -- 1.070 au O 1.00 1 D:xy 10

(29.103 eV)

232 275 -- 1.070 au O 1.00 1 D:xz 10

(29.103 eV)

233 276 -- 1.070 au O 1.00 1 D:yz 10

(29.103 eV)

234 277 -- 3.358 au O 1.00 1 F:z3 10

(91.384 eV)

235 278 -- 3.358 au O 1.00 1 F:z 10

(91.384 eV)

236 279 -- 3.358 au O 1.00 1 F:xyz 10

(91.384 eV)

237 280 -- 3.358 au O 1.00 1 F:z2x 10

(91.384 eV)

238 281 -- 3.358 au O 1.00 1 F:z2y 10

(91.384 eV)

239 282 -- 3.358 au O 1.00 1 F:x 10

(91.384 eV)

240 283 -- 3.358 au O 1.00 1 F:y 10

(91.384 eV)

241 284 2.000 -0.501 au C 1.00 1 S 11

(-13.631 eV)

242 285 -- 0.431 au C 1.00 2 S 11

(11.741 eV)

243 286 -- 9.944 au C 1.00 3 S 11

(270.596 eV)

244 287 0.667 -0.193 au C 1.00 1 P:x 11

(-5.263 eV)

245 288 -- 0.168 au C 1.00 2 P:x 11

(4.578 eV)

246 289 -- 2.012 au C 1.00 3 P:x 11

(54.761 eV)

247 290 0.667 -0.193 au C 1.00 1 P:y 11

(-5.263 eV)

248 291 -- 0.168 au C 1.00 2 P:y 11

(4.578 eV)

249 292 -- 2.012 au C 1.00 3 P:y 11

(54.761 eV)

250 293 0.667 -0.193 au C 1.00 1 P:z 11

(-5.263 eV)

251 294 -- 0.168 au C 1.00 2 P:z 11

(4.578 eV)

252 295 -- 2.012 au C 1.00 3 P:z 11

(54.761 eV)

253 296 -- 1.242 au C 1.00 1 D:z2 11

(33.808 eV)

254 297 -- 1.242 au C 1.00 1 D:x2-y2 11

(33.808 eV)

255 298 -- 1.242 au C 1.00 1 D:xy 11

(33.808 eV)

256 299 -- 1.242 au C 1.00 1 D:xz 11

(33.808 eV)

257 300 -- 1.242 au C 1.00 1 D:yz 11

(33.808 eV)

258 301 -- 4.016 au C 1.00 1 F:z3 11

(109.273 eV)

259 302 -- 4.016 au C 1.00 1 F:z 11

(109.273 eV)

260 303 -- 4.016 au C 1.00 1 F:xyz 11

(109.273 eV)

261 304 -- 4.016 au C 1.00 1 F:z2x 11

(109.273 eV)

262 305 -- 4.016 au C 1.00 1 F:z2y 11

(109.273 eV)

263 306 -- 4.016 au C 1.00 1 F:x 11

(109.273 eV)

264 307 -- 4.016 au C 1.00 1 F:y 11

(109.273 eV)

265 308 2.000 -0.501 au C 1.00 1 S 12

(-13.631 eV)

266 309 -- 0.431 au C 1.00 2 S 12

(11.741 eV)

267 310 -- 9.944 au C 1.00 3 S 12

(270.596 eV)

268 311 0.667 -0.193 au C 1.00 1 P:x 12

(-5.263 eV)

269 312 -- 0.168 au C 1.00 2 P:x 12

(4.578 eV)

270 313 -- 2.012 au C 1.00 3 P:x 12

(54.761 eV)

271 314 0.667 -0.193 au C 1.00 1 P:y 12

(-5.263 eV)

272 315 -- 0.168 au C 1.00 2 P:y 12

(4.578 eV)

273 316 -- 2.012 au C 1.00 3 P:y 12

(54.761 eV)

274 317 0.667 -0.193 au C 1.00 1 P:z 12

(-5.263 eV)

275 318 -- 0.168 au C 1.00 2 P:z 12

(4.578 eV)

276 319 -- 2.012 au C 1.00 3 P:z 12

(54.761 eV)

277 320 -- 1.242 au C 1.00 1 D:z2 12

(33.808 eV)

278 321 -- 1.242 au C 1.00 1 D:x2-y2 12

(33.808 eV)

279 322 -- 1.242 au C 1.00 1 D:xy 12

(33.808 eV)

280 323 -- 1.242 au C 1.00 1 D:xz 12

(33.808 eV)

281 324 -- 1.242 au C 1.00 1 D:yz 12

(33.808 eV)

282 325 -- 4.016 au C 1.00 1 F:z3 12

(109.273 eV)

283 326 -- 4.016 au C 1.00 1 F:z 12

(109.273 eV)

284 327 -- 4.016 au C 1.00 1 F:xyz 12

(109.273 eV)

285 328 -- 4.016 au C 1.00 1 F:z2x 12

(109.273 eV)

286 329 -- 4.016 au C 1.00 1 F:z2y 12

(109.273 eV)

287 330 -- 4.016 au C 1.00 1 F:x 12

(109.273 eV)

288 331 -- 4.016 au C 1.00 1 F:y 12

(109.273 eV)

289 332 2.000 -0.501 au C 1.00 1 S 13

(-13.631 eV)

290 333 -- 0.431 au C 1.00 2 S 13

(11.741 eV)

291 334 -- 9.944 au C 1.00 3 S 13

(270.596 eV)

292 335 0.667 -0.193 au C 1.00 1 P:x 13

(-5.263 eV)

293 336 -- 0.168 au C 1.00 2 P:x 13

(4.578 eV)

294 337 -- 2.012 au C 1.00 3 P:x 13

(54.761 eV)

295 338 0.667 -0.193 au C 1.00 1 P:y 13

(-5.263 eV)

296 339 -- 0.168 au C 1.00 2 P:y 13

(4.578 eV)

297 340 -- 2.012 au C 1.00 3 P:y 13

(54.761 eV)

298 341 0.667 -0.193 au C 1.00 1 P:z 13

(-5.263 eV)

299 342 -- 0.168 au C 1.00 2 P:z 13

(4.578 eV)

300 343 -- 2.012 au C 1.00 3 P:z 13

(54.761 eV)

301 344 -- 1.242 au C 1.00 1 D:z2 13

(33.808 eV)

302 345 -- 1.242 au C 1.00 1 D:x2-y2 13

(33.808 eV)

303 346 -- 1.242 au C 1.00 1 D:xy 13

(33.808 eV)

304 347 -- 1.242 au C 1.00 1 D:xz 13

(33.808 eV)

305 348 -- 1.242 au C 1.00 1 D:yz 13

(33.808 eV)

306 349 -- 4.016 au C 1.00 1 F:z3 13

(109.273 eV)

307 350 -- 4.016 au C 1.00 1 F:z 13

(109.273 eV)

308 351 -- 4.016 au C 1.00 1 F:xyz 13

(109.273 eV)

309 352 -- 4.016 au C 1.00 1 F:z2x 13

(109.273 eV)

310 353 -- 4.016 au C 1.00 1 F:z2y 13

(109.273 eV)

311 354 -- 4.016 au C 1.00 1 F:x 13

(109.273 eV)

312 355 -- 4.016 au C 1.00 1 F:y 13

(109.273 eV)

313 356 2.000 -0.501 au C 1.00 1 S 14

(-13.631 eV)

314 357 -- 0.431 au C 1.00 2 S 14

(11.741 eV)

315 358 -- 9.944 au C 1.00 3 S 14

(270.596 eV)

316 359 0.667 -0.193 au C 1.00 1 P:x 14

(-5.263 eV)

317 360 -- 0.168 au C 1.00 2 P:x 14

(4.578 eV)

318 361 -- 2.012 au C 1.00 3 P:x 14

(54.761 eV)

319 362 0.667 -0.193 au C 1.00 1 P:y 14

(-5.263 eV)

320 363 -- 0.168 au C 1.00 2 P:y 14

(4.578 eV)

321 364 -- 2.012 au C 1.00 3 P:y 14

(54.761 eV)

322 365 0.667 -0.193 au C 1.00 1 P:z 14

(-5.263 eV)

323 366 -- 0.168 au C 1.00 2 P:z 14

(4.578 eV)

324 367 -- 2.012 au C 1.00 3 P:z 14

(54.761 eV)

325 368 -- 1.242 au C 1.00 1 D:z2 14

(33.808 eV)

326 369 -- 1.242 au C 1.00 1 D:x2-y2 14

(33.808 eV)

327 370 -- 1.242 au C 1.00 1 D:xy 14

(33.808 eV)

328 371 -- 1.242 au C 1.00 1 D:xz 14

(33.808 eV)

329 372 -- 1.242 au C 1.00 1 D:yz 14

(33.808 eV)

330 373 -- 4.016 au C 1.00 1 F:z3 14

(109.273 eV)

331 374 -- 4.016 au C 1.00 1 F:z 14

(109.273 eV)

332 375 -- 4.016 au C 1.00 1 F:xyz 14

(109.273 eV)

333 376 -- 4.016 au C 1.00 1 F:z2x 14

(109.273 eV)

334 377 -- 4.016 au C 1.00 1 F:z2y 14

(109.273 eV)

335 378 -- 4.016 au C 1.00 1 F:x 14

(109.273 eV)

336 379 -- 4.016 au C 1.00 1 F:y 14

(109.273 eV)

337 380 2.000 -0.501 au C 1.00 1 S 15

(-13.631 eV)

338 381 -- 0.431 au C 1.00 2 S 15

(11.741 eV)

339 382 -- 9.944 au C 1.00 3 S 15

(270.596 eV)

340 383 0.667 -0.193 au C 1.00 1 P:x 15

(-5.263 eV)

341 384 -- 0.168 au C 1.00 2 P:x 15

(4.578 eV)

342 385 -- 2.012 au C 1.00 3 P:x 15

(54.761 eV)

343 386 0.667 -0.193 au C 1.00 1 P:y 15

(-5.263 eV)

344 387 -- 0.168 au C 1.00 2 P:y 15

(4.578 eV)

345 388 -- 2.012 au C 1.00 3 P:y 15

(54.761 eV)

346 389 0.667 -0.193 au C 1.00 1 P:z 15

(-5.263 eV)

347 390 -- 0.168 au C 1.00 2 P:z 15

(4.578 eV)

348 391 -- 2.012 au C 1.00 3 P:z 15

(54.761 eV)

349 392 -- 1.242 au C 1.00 1 D:z2 15

(33.808 eV)

350 393 -- 1.242 au C 1.00 1 D:x2-y2 15

(33.808 eV)

351 394 -- 1.242 au C 1.00 1 D:xy 15

(33.808 eV)

352 395 -- 1.242 au C 1.00 1 D:xz 15

(33.808 eV)

353 396 -- 1.242 au C 1.00 1 D:yz 15

(33.808 eV)

354 397 -- 4.016 au C 1.00 1 F:z3 15

(109.273 eV)

355 398 -- 4.016 au C 1.00 1 F:z 15

(109.273 eV)

356 399 -- 4.016 au C 1.00 1 F:xyz 15

(109.273 eV)

357 400 -- 4.016 au C 1.00 1 F:z2x 15

(109.273 eV)

358 401 -- 4.016 au C 1.00 1 F:z2y 15

(109.273 eV)

359 402 -- 4.016 au C 1.00 1 F:x 15

(109.273 eV)

360 403 -- 4.016 au C 1.00 1 F:y 15

(109.273 eV)

361 404 2.000 -0.501 au C 1.00 1 S 16

(-13.631 eV)

362 405 -- 0.431 au C 1.00 2 S 16

(11.741 eV)

363 406 -- 9.944 au C 1.00 3 S 16

(270.596 eV)

364 407 0.667 -0.193 au C 1.00 1 P:x 16

(-5.263 eV)

365 408 -- 0.168 au C 1.00 2 P:x 16

(4.578 eV)

366 409 -- 2.012 au C 1.00 3 P:x 16

(54.761 eV)

367 410 0.667 -0.193 au C 1.00 1 P:y 16

(-5.263 eV)

368 411 -- 0.168 au C 1.00 2 P:y 16

(4.578 eV)

369 412 -- 2.012 au C 1.00 3 P:y 16

(54.761 eV)

370 413 0.667 -0.193 au C 1.00 1 P:z 16

(-5.263 eV)

371 414 -- 0.168 au C 1.00 2 P:z 16

(4.578 eV)

372 415 -- 2.012 au C 1.00 3 P:z 16

(54.761 eV)

373 416 -- 1.242 au C 1.00 1 D:z2 16

(33.808 eV)

374 417 -- 1.242 au C 1.00 1 D:x2-y2 16

(33.808 eV)

375 418 -- 1.242 au C 1.00 1 D:xy 16

(33.808 eV)

376 419 -- 1.242 au C 1.00 1 D:xz 16

(33.808 eV)

377 420 -- 1.242 au C 1.00 1 D:yz 16

(33.808 eV)

378 421 -- 4.016 au C 1.00 1 F:z3 16

(109.273 eV)

379 422 -- 4.016 au C 1.00 1 F:z 16

(109.273 eV)

380 423 -- 4.016 au C 1.00 1 F:xyz 16

(109.273 eV)

381 424 -- 4.016 au C 1.00 1 F:z2x 16

(109.273 eV)

382 425 -- 4.016 au C 1.00 1 F:z2y 16

(109.273 eV)

383 426 -- 4.016 au C 1.00 1 F:x 16

(109.273 eV)

384 427 -- 4.016 au C 1.00 1 F:y 16

(109.273 eV)

385 428 2.000 -0.501 au C 1.00 1 S 17

(-13.631 eV)

386 429 -- 0.431 au C 1.00 2 S 17

(11.741 eV)

387 430 -- 9.944 au C 1.00 3 S 17

(270.596 eV)

388 431 0.667 -0.193 au C 1.00 1 P:x 17

(-5.263 eV)

389 432 -- 0.168 au C 1.00 2 P:x 17

(4.578 eV)

390 433 -- 2.012 au C 1.00 3 P:x 17

(54.761 eV)

391 434 0.667 -0.193 au C 1.00 1 P:y 17

(-5.263 eV)

392 435 -- 0.168 au C 1.00 2 P:y 17

(4.578 eV)

393 436 -- 2.012 au C 1.00 3 P:y 17

(54.761 eV)

394 437 0.667 -0.193 au C 1.00 1 P:z 17

(-5.263 eV)

395 438 -- 0.168 au C 1.00 2 P:z 17

(4.578 eV)

396 439 -- 2.012 au C 1.00 3 P:z 17

(54.761 eV)

397 440 -- 1.242 au C 1.00 1 D:z2 17

(33.808 eV)

398 441 -- 1.242 au C 1.00 1 D:x2-y2 17

(33.808 eV)

399 442 -- 1.242 au C 1.00 1 D:xy 17

(33.808 eV)

400 443 -- 1.242 au C 1.00 1 D:xz 17

(33.808 eV)

401 444 -- 1.242 au C 1.00 1 D:yz 17

(33.808 eV)

402 445 -- 4.016 au C 1.00 1 F:z3 17

(109.273 eV)

403 446 -- 4.016 au C 1.00 1 F:z 17

(109.273 eV)

404 447 -- 4.016 au C 1.00 1 F:xyz 17

(109.273 eV)

405 448 -- 4.016 au C 1.00 1 F:z2x 17

(109.273 eV)

406 449 -- 4.016 au C 1.00 1 F:z2y 17

(109.273 eV)

407 450 -- 4.016 au C 1.00 1 F:x 17

(109.273 eV)

408 451 -- 4.016 au C 1.00 1 F:y 17

(109.273 eV)

409 452 2.000 -0.501 au C 1.00 1 S 18

(-13.631 eV)

410 453 -- 0.431 au C 1.00 2 S 18

(11.741 eV)

411 454 -- 9.944 au C 1.00 3 S 18

(270.596 eV)

412 455 0.667 -0.193 au C 1.00 1 P:x 18

(-5.263 eV)

413 456 -- 0.168 au C 1.00 2 P:x 18

(4.578 eV)

414 457 -- 2.012 au C 1.00 3 P:x 18

(54.761 eV)

415 458 0.667 -0.193 au C 1.00 1 P:y 18

(-5.263 eV)

416 459 -- 0.168 au C 1.00 2 P:y 18

(4.578 eV)

417 460 -- 2.012 au C 1.00 3 P:y 18

(54.761 eV)

418 461 0.667 -0.193 au C 1.00 1 P:z 18

(-5.263 eV)

419 462 -- 0.168 au C 1.00 2 P:z 18

(4.578 eV)

420 463 -- 2.012 au C 1.00 3 P:z 18

(54.761 eV)

421 464 -- 1.242 au C 1.00 1 D:z2 18

(33.808 eV)

422 465 -- 1.242 au C 1.00 1 D:x2-y2 18

(33.808 eV)

423 466 -- 1.242 au C 1.00 1 D:xy 18

(33.808 eV)

424 467 -- 1.242 au C 1.00 1 D:xz 18

(33.808 eV)

425 468 -- 1.242 au C 1.00 1 D:yz 18

(33.808 eV)

426 469 -- 4.016 au C 1.00 1 F:z3 18

(109.273 eV)

427 470 -- 4.016 au C 1.00 1 F:z 18

(109.273 eV)

428 471 -- 4.016 au C 1.00 1 F:xyz 18

(109.273 eV)

429 472 -- 4.016 au C 1.00 1 F:z2x 18

(109.273 eV)

430 473 -- 4.016 au C 1.00 1 F:z2y 18

(109.273 eV)

431 474 -- 4.016 au C 1.00 1 F:x 18

(109.273 eV)

432 475 -- 4.016 au C 1.00 1 F:y 18

(109.273 eV)

433 476 2.000 -0.501 au C 1.00 1 S 19

(-13.631 eV)

434 477 -- 0.431 au C 1.00 2 S 19

(11.741 eV)

435 478 -- 9.944 au C 1.00 3 S 19

(270.596 eV)

436 479 0.667 -0.193 au C 1.00 1 P:x 19

(-5.263 eV)

437 480 -- 0.168 au C 1.00 2 P:x 19

(4.578 eV)

438 481 -- 2.012 au C 1.00 3 P:x 19

(54.761 eV)

439 482 0.667 -0.193 au C 1.00 1 P:y 19

(-5.263 eV)

440 483 -- 0.168 au C 1.00 2 P:y 19

(4.578 eV)

441 484 -- 2.012 au C 1.00 3 P:y 19

(54.761 eV)

442 485 0.667 -0.193 au C 1.00 1 P:z 19

(-5.263 eV)

443 486 -- 0.168 au C 1.00 2 P:z 19

(4.578 eV)

444 487 -- 2.012 au C 1.00 3 P:z 19

(54.761 eV)

445 488 -- 1.242 au C 1.00 1 D:z2 19

(33.808 eV)

446 489 -- 1.242 au C 1.00 1 D:x2-y2 19

(33.808 eV)

447 490 -- 1.242 au C 1.00 1 D:xy 19

(33.808 eV)

448 491 -- 1.242 au C 1.00 1 D:xz 19

(33.808 eV)

449 492 -- 1.242 au C 1.00 1 D:yz 19

(33.808 eV)

450 493 -- 4.016 au C 1.00 1 F:z3 19

(109.273 eV)

451 494 -- 4.016 au C 1.00 1 F:z 19

(109.273 eV)

452 495 -- 4.016 au C 1.00 1 F:xyz 19

(109.273 eV)

453 496 -- 4.016 au C 1.00 1 F:z2x 19

(109.273 eV)

454 497 -- 4.016 au C 1.00 1 F:z2y 19

(109.273 eV)

455 498 -- 4.016 au C 1.00 1 F:x 19

(109.273 eV)

456 499 -- 4.016 au C 1.00 1 F:y 19

(109.273 eV)

457 500 2.000 -0.501 au C 1.00 1 S 20

(-13.631 eV)

458 501 -- 0.431 au C 1.00 2 S 20

(11.741 eV)

459 502 -- 9.944 au C 1.00 3 S 20

(270.596 eV)

460 503 0.667 -0.193 au C 1.00 1 P:x 20

(-5.263 eV)

461 504 -- 0.168 au C 1.00 2 P:x 20

(4.578 eV)

462 505 -- 2.012 au C 1.00 3 P:x 20

(54.761 eV)

463 506 0.667 -0.193 au C 1.00 1 P:y 20

(-5.263 eV)

464 507 -- 0.168 au C 1.00 2 P:y 20

(4.578 eV)

465 508 -- 2.012 au C 1.00 3 P:y 20

(54.761 eV)

466 509 0.667 -0.193 au C 1.00 1 P:z 20

(-5.263 eV)

467 510 -- 0.168 au C 1.00 2 P:z 20

(4.578 eV)

468 511 -- 2.012 au C 1.00 3 P:z 20

(54.761 eV)

469 512 -- 1.242 au C 1.00 1 D:z2 20

(33.808 eV)

470 513 -- 1.242 au C 1.00 1 D:x2-y2 20

(33.808 eV)

471 514 -- 1.242 au C 1.00 1 D:xy 20

(33.808 eV)

472 515 -- 1.242 au C 1.00 1 D:xz 20

(33.808 eV)

473 516 -- 1.242 au C 1.00 1 D:yz 20

(33.808 eV)

474 517 -- 4.016 au C 1.00 1 F:z3 20

(109.273 eV)

475 518 -- 4.016 au C 1.00 1 F:z 20

(109.273 eV)

476 519 -- 4.016 au C 1.00 1 F:xyz 20

(109.273 eV)

477 520 -- 4.016 au C 1.00 1 F:z2x 20

(109.273 eV)

478 521 -- 4.016 au C 1.00 1 F:z2y 20

(109.273 eV)

479 522 -- 4.016 au C 1.00 1 F:x 20

(109.273 eV)

480 523 -- 4.016 au C 1.00 1 F:y 20

(109.273 eV)

481 524 2.000 -0.501 au C 1.00 1 S 21

(-13.631 eV)

482 525 -- 0.431 au C 1.00 2 S 21

(11.741 eV)

483 526 -- 9.944 au C 1.00 3 S 21

(270.596 eV)

484 527 0.667 -0.193 au C 1.00 1 P:x 21

(-5.263 eV)

485 528 -- 0.168 au C 1.00 2 P:x 21

(4.578 eV)

486 529 -- 2.012 au C 1.00 3 P:x 21

(54.761 eV)

487 530 0.667 -0.193 au C 1.00 1 P:y 21

(-5.263 eV)

488 531 -- 0.168 au C 1.00 2 P:y 21

(4.578 eV)

489 532 -- 2.012 au C 1.00 3 P:y 21

(54.761 eV)

490 533 0.667 -0.193 au C 1.00 1 P:z 21

(-5.263 eV)

491 534 -- 0.168 au C 1.00 2 P:z 21

(4.578 eV)

492 535 -- 2.012 au C 1.00 3 P:z 21

(54.761 eV)

493 536 -- 1.242 au C 1.00 1 D:z2 21

(33.808 eV)

494 537 -- 1.242 au C 1.00 1 D:x2-y2 21

(33.808 eV)

495 538 -- 1.242 au C 1.00 1 D:xy 21

(33.808 eV)

496 539 -- 1.242 au C 1.00 1 D:xz 21

(33.808 eV)

497 540 -- 1.242 au C 1.00 1 D:yz 21

(33.808 eV)

498 541 -- 4.016 au C 1.00 1 F:z3 21

(109.273 eV)

499 542 -- 4.016 au C 1.00 1 F:z 21

(109.273 eV)

500 543 -- 4.016 au C 1.00 1 F:xyz 21

(109.273 eV)

501 544 -- 4.016 au C 1.00 1 F:z2x 21

(109.273 eV)

502 545 -- 4.016 au C 1.00 1 F:z2y 21

(109.273 eV)

503 546 -- 4.016 au C 1.00 1 F:x 21

(109.273 eV)

504 547 -- 4.016 au C 1.00 1 F:y 21

(109.273 eV)

505 548 2.000 -0.501 au C 1.00 1 S 22

(-13.631 eV)

506 549 -- 0.431 au C 1.00 2 S 22

(11.741 eV)

507 550 -- 9.944 au C 1.00 3 S 22

(270.596 eV)

508 551 0.667 -0.193 au C 1.00 1 P:x 22

(-5.263 eV)

509 552 -- 0.168 au C 1.00 2 P:x 22

(4.578 eV)

510 553 -- 2.012 au C 1.00 3 P:x 22

(54.761 eV)

511 554 0.667 -0.193 au C 1.00 1 P:y 22

(-5.263 eV)

512 555 -- 0.168 au C 1.00 2 P:y 22

(4.578 eV)

513 556 -- 2.012 au C 1.00 3 P:y 22

(54.761 eV)

514 557 0.667 -0.193 au C 1.00 1 P:z 22

(-5.263 eV)

515 558 -- 0.168 au C 1.00 2 P:z 22

(4.578 eV)

516 559 -- 2.012 au C 1.00 3 P:z 22

(54.761 eV)

517 560 -- 1.242 au C 1.00 1 D:z2 22

(33.808 eV)

518 561 -- 1.242 au C 1.00 1 D:x2-y2 22

(33.808 eV)

519 562 -- 1.242 au C 1.00 1 D:xy 22

(33.808 eV)

520 563 -- 1.242 au C 1.00 1 D:xz 22

(33.808 eV)

521 564 -- 1.242 au C 1.00 1 D:yz 22

(33.808 eV)

522 565 -- 4.016 au C 1.00 1 F:z3 22

(109.273 eV)

523 566 -- 4.016 au C 1.00 1 F:z 22

(109.273 eV)

524 567 -- 4.016 au C 1.00 1 F:xyz 22

(109.273 eV)

525 568 -- 4.016 au C 1.00 1 F:z2x 22

(109.273 eV)

526 569 -- 4.016 au C 1.00 1 F:z2y 22

(109.273 eV)

527 570 -- 4.016 au C 1.00 1 F:x 22

(109.273 eV)

528 571 -- 4.016 au C 1.00 1 F:y 22

(109.273 eV)

529 572 2.000 -0.501 au C 1.00 1 S 23

(-13.631 eV)

530 573 -- 0.431 au C 1.00 2 S 23

(11.741 eV)

531 574 -- 9.944 au C 1.00 3 S 23

(270.596 eV)

532 575 0.667 -0.193 au C 1.00 1 P:x 23

(-5.263 eV)

533 576 -- 0.168 au C 1.00 2 P:x 23

(4.578 eV)

534 577 -- 2.012 au C 1.00 3 P:x 23

(54.761 eV)

535 578 0.667 -0.193 au C 1.00 1 P:y 23

(-5.263 eV)

536 579 -- 0.168 au C 1.00 2 P:y 23

(4.578 eV)

537 580 -- 2.012 au C 1.00 3 P:y 23

(54.761 eV)

538 581 0.667 -0.193 au C 1.00 1 P:z 23

(-5.263 eV)

539 582 -- 0.168 au C 1.00 2 P:z 23

(4.578 eV)

540 583 -- 2.012 au C 1.00 3 P:z 23

(54.761 eV)

541 584 -- 1.242 au C 1.00 1 D:z2 23

(33.808 eV)

542 585 -- 1.242 au C 1.00 1 D:x2-y2 23

(33.808 eV)

543 586 -- 1.242 au C 1.00 1 D:xy 23

(33.808 eV)

544 587 -- 1.242 au C 1.00 1 D:xz 23

(33.808 eV)

545 588 -- 1.242 au C 1.00 1 D:yz 23

(33.808 eV)

546 589 -- 4.016 au C 1.00 1 F:z3 23

(109.273 eV)

547 590 -- 4.016 au C 1.00 1 F:z 23

(109.273 eV)

548 591 -- 4.016 au C 1.00 1 F:xyz 23

(109.273 eV)

549 592 -- 4.016 au C 1.00 1 F:z2x 23

(109.273 eV)

550 593 -- 4.016 au C 1.00 1 F:z2y 23

(109.273 eV)

551 594 -- 4.016 au C 1.00 1 F:x 23

(109.273 eV)

552 595 -- 4.016 au C 1.00 1 F:y 23

(109.273 eV)

553 596 2.000 -0.501 au C 1.00 1 S 24

(-13.631 eV)

554 597 -- 0.431 au C 1.00 2 S 24

(11.741 eV)

555 598 -- 9.944 au C 1.00 3 S 24

(270.596 eV)

556 599 0.667 -0.193 au C 1.00 1 P:x 24

(-5.263 eV)

557 600 -- 0.168 au C 1.00 2 P:x 24

(4.578 eV)

558 601 -- 2.012 au C 1.00 3 P:x 24

(54.761 eV)

559 602 0.667 -0.193 au C 1.00 1 P:y 24

(-5.263 eV)

560 603 -- 0.168 au C 1.00 2 P:y 24

(4.578 eV)

561 604 -- 2.012 au C 1.00 3 P:y 24

(54.761 eV)

562 605 0.667 -0.193 au C 1.00 1 P:z 24

(-5.263 eV)

563 606 -- 0.168 au C 1.00 2 P:z 24

(4.578 eV)

564 607 -- 2.012 au C 1.00 3 P:z 24

(54.761 eV)

565 608 -- 1.242 au C 1.00 1 D:z2 24

(33.808 eV)

566 609 -- 1.242 au C 1.00 1 D:x2-y2 24

(33.808 eV)

567 610 -- 1.242 au C 1.00 1 D:xy 24

(33.808 eV)

568 611 -- 1.242 au C 1.00 1 D:xz 24

(33.808 eV)

569 612 -- 1.242 au C 1.00 1 D:yz 24

(33.808 eV)

570 613 -- 4.016 au C 1.00 1 F:z3 24

(109.273 eV)

571 614 -- 4.016 au C 1.00 1 F:z 24

(109.273 eV)

572 615 -- 4.016 au C 1.00 1 F:xyz 24

(109.273 eV)

573 616 -- 4.016 au C 1.00 1 F:z2x 24

(109.273 eV)

574 617 -- 4.016 au C 1.00 1 F:z2y 24

(109.273 eV)

575 618 -- 4.016 au C 1.00 1 F:x 24

(109.273 eV)

576 619 -- 4.016 au C 1.00 1 F:y 24

(109.273 eV)

577 620 2.000 -0.501 au C 1.00 1 S 25

(-13.631 eV)

578 621 -- 0.431 au C 1.00 2 S 25

(11.741 eV)

579 622 -- 9.944 au C 1.00 3 S 25

(270.596 eV)

580 623 0.667 -0.193 au C 1.00 1 P:x 25

(-5.263 eV)

581 624 -- 0.168 au C 1.00 2 P:x 25

(4.578 eV)

582 625 -- 2.012 au C 1.00 3 P:x 25

(54.761 eV)

583 626 0.667 -0.193 au C 1.00 1 P:y 25

(-5.263 eV)

584 627 -- 0.168 au C 1.00 2 P:y 25

(4.578 eV)

585 628 -- 2.012 au C 1.00 3 P:y 25

(54.761 eV)

586 629 0.667 -0.193 au C 1.00 1 P:z 25

(-5.263 eV)

587 630 -- 0.168 au C 1.00 2 P:z 25

(4.578 eV)

588 631 -- 2.012 au C 1.00 3 P:z 25

(54.761 eV)

589 632 -- 1.242 au C 1.00 1 D:z2 25

(33.808 eV)

590 633 -- 1.242 au C 1.00 1 D:x2-y2 25

(33.808 eV)

591 634 -- 1.242 au C 1.00 1 D:xy 25

(33.808 eV)

592 635 -- 1.242 au C 1.00 1 D:xz 25

(33.808 eV)

593 636 -- 1.242 au C 1.00 1 D:yz 25

(33.808 eV)

594 637 -- 4.016 au C 1.00 1 F:z3 25

(109.273 eV)

595 638 -- 4.016 au C 1.00 1 F:z 25

(109.273 eV)

596 639 -- 4.016 au C 1.00 1 F:xyz 25

(109.273 eV)

597 640 -- 4.016 au C 1.00 1 F:z2x 25

(109.273 eV)

598 641 -- 4.016 au C 1.00 1 F:z2y 25

(109.273 eV)

599 642 -- 4.016 au C 1.00 1 F:x 25

(109.273 eV)

600 643 -- 4.016 au C 1.00 1 F:y 25

(109.273 eV)

601 644 2.000 -0.501 au C 1.00 1 S 26

(-13.631 eV)

602 645 -- 0.431 au C 1.00 2 S 26

(11.741 eV)

603 646 -- 9.944 au C 1.00 3 S 26

(270.596 eV)

604 647 0.667 -0.193 au C 1.00 1 P:x 26

(-5.263 eV)

605 648 -- 0.168 au C 1.00 2 P:x 26

(4.578 eV)

606 649 -- 2.012 au C 1.00 3 P:x 26

(54.761 eV)

607 650 0.667 -0.193 au C 1.00 1 P:y 26

(-5.263 eV)

608 651 -- 0.168 au C 1.00 2 P:y 26

(4.578 eV)

609 652 -- 2.012 au C 1.00 3 P:y 26

(54.761 eV)

610 653 0.667 -0.193 au C 1.00 1 P:z 26

(-5.263 eV)

611 654 -- 0.168 au C 1.00 2 P:z 26

(4.578 eV)

612 655 -- 2.012 au C 1.00 3 P:z 26

(54.761 eV)

613 656 -- 1.242 au C 1.00 1 D:z2 26

(33.808 eV)

614 657 -- 1.242 au C 1.00 1 D:x2-y2 26

(33.808 eV)

615 658 -- 1.242 au C 1.00 1 D:xy 26

(33.808 eV)

616 659 -- 1.242 au C 1.00 1 D:xz 26

(33.808 eV)

617 660 -- 1.242 au C 1.00 1 D:yz 26

(33.808 eV)

618 661 -- 4.016 au C 1.00 1 F:z3 26

(109.273 eV)

619 662 -- 4.016 au C 1.00 1 F:z 26

(109.273 eV)

620 663 -- 4.016 au C 1.00 1 F:xyz 26

(109.273 eV)

621 664 -- 4.016 au C 1.00 1 F:z2x 26

(109.273 eV)

622 665 -- 4.016 au C 1.00 1 F:z2y 26

(109.273 eV)

623 666 -- 4.016 au C 1.00 1 F:x 26

(109.273 eV)

624 667 -- 4.016 au C 1.00 1 F:y 26

(109.273 eV)

625 668 2.000 -0.501 au C 1.00 1 S 27

(-13.631 eV)

626 669 -- 0.431 au C 1.00 2 S 27

(11.741 eV)

627 670 -- 9.944 au C 1.00 3 S 27

(270.596 eV)

628 671 0.667 -0.193 au C 1.00 1 P:x 27

(-5.263 eV)

629 672 -- 0.168 au C 1.00 2 P:x 27

(4.578 eV)

630 673 -- 2.012 au C 1.00 3 P:x 27

(54.761 eV)

631 674 0.667 -0.193 au C 1.00 1 P:y 27

(-5.263 eV)

632 675 -- 0.168 au C 1.00 2 P:y 27

(4.578 eV)

633 676 -- 2.012 au C 1.00 3 P:y 27

(54.761 eV)

634 677 0.667 -0.193 au C 1.00 1 P:z 27

(-5.263 eV)

635 678 -- 0.168 au C 1.00 2 P:z 27

(4.578 eV)

636 679 -- 2.012 au C 1.00 3 P:z 27

(54.761 eV)

637 680 -- 1.242 au C 1.00 1 D:z2 27

(33.808 eV)

638 681 -- 1.242 au C 1.00 1 D:x2-y2 27

(33.808 eV)

639 682 -- 1.242 au C 1.00 1 D:xy 27

(33.808 eV)

640 683 -- 1.242 au C 1.00 1 D:xz 27

(33.808 eV)

641 684 -- 1.242 au C 1.00 1 D:yz 27

(33.808 eV)

642 685 -- 4.016 au C 1.00 1 F:z3 27

(109.273 eV)

643 686 -- 4.016 au C 1.00 1 F:z 27

(109.273 eV)

644 687 -- 4.016 au C 1.00 1 F:xyz 27

(109.273 eV)

645 688 -- 4.016 au C 1.00 1 F:z2x 27

(109.273 eV)

646 689 -- 4.016 au C 1.00 1 F:z2y 27

(109.273 eV)

647 690 -- 4.016 au C 1.00 1 F:x 27

(109.273 eV)

648 691 -- 4.016 au C 1.00 1 F:y 27

(109.273 eV)

649 692 2.000 -0.501 au C 1.00 1 S 28

(-13.631 eV)

650 693 -- 0.431 au C 1.00 2 S 28

(11.741 eV)

651 694 -- 9.944 au C 1.00 3 S 28

(270.596 eV)

652 695 0.667 -0.193 au C 1.00 1 P:x 28

(-5.263 eV)

653 696 -- 0.168 au C 1.00 2 P:x 28

(4.578 eV)

654 697 -- 2.012 au C 1.00 3 P:x 28

(54.761 eV)

655 698 0.667 -0.193 au C 1.00 1 P:y 28

(-5.263 eV)

656 699 -- 0.168 au C 1.00 2 P:y 28

(4.578 eV)

657 700 -- 2.012 au C 1.00 3 P:y 28

(54.761 eV)

658 701 0.667 -0.193 au C 1.00 1 P:z 28

(-5.263 eV)

659 702 -- 0.168 au C 1.00 2 P:z 28

(4.578 eV)

660 703 -- 2.012 au C 1.00 3 P:z 28

(54.761 eV)

661 704 -- 1.242 au C 1.00 1 D:z2 28

(33.808 eV)

662 705 -- 1.242 au C 1.00 1 D:x2-y2 28

(33.808 eV)

663 706 -- 1.242 au C 1.00 1 D:xy 28

(33.808 eV)

664 707 -- 1.242 au C 1.00 1 D:xz 28

(33.808 eV)

665 708 -- 1.242 au C 1.00 1 D:yz 28

(33.808 eV)

666 709 -- 4.016 au C 1.00 1 F:z3 28

(109.273 eV)

667 710 -- 4.016 au C 1.00 1 F:z 28

(109.273 eV)

668 711 -- 4.016 au C 1.00 1 F:xyz 28

(109.273 eV)

669 712 -- 4.016 au C 1.00 1 F:z2x 28

(109.273 eV)

670 713 -- 4.016 au C 1.00 1 F:z2y 28

(109.273 eV)

671 714 -- 4.016 au C 1.00 1 F:x 28

(109.273 eV)

672 715 -- 4.016 au C 1.00 1 F:y 28

(109.273 eV)

673 716 2.000 -0.501 au C 1.00 1 S 29

(-13.631 eV)

674 717 -- 0.431 au C 1.00 2 S 29

(11.741 eV)

675 718 -- 9.944 au C 1.00 3 S 29

(270.596 eV)

676 719 0.667 -0.193 au C 1.00 1 P:x 29

(-5.263 eV)

677 720 -- 0.168 au C 1.00 2 P:x 29

(4.578 eV)

678 721 -- 2.012 au C 1.00 3 P:x 29

(54.761 eV)

679 722 0.667 -0.193 au C 1.00 1 P:y 29

(-5.263 eV)

680 723 -- 0.168 au C 1.00 2 P:y 29

(4.578 eV)

681 724 -- 2.012 au C 1.00 3 P:y 29

(54.761 eV)

682 725 0.667 -0.193 au C 1.00 1 P:z 29

(-5.263 eV)

683 726 -- 0.168 au C 1.00 2 P:z 29

(4.578 eV)

684 727 -- 2.012 au C 1.00 3 P:z 29

(54.761 eV)

685 728 -- 1.242 au C 1.00 1 D:z2 29

(33.808 eV)

686 729 -- 1.242 au C 1.00 1 D:x2-y2 29

(33.808 eV)

687 730 -- 1.242 au C 1.00 1 D:xy 29

(33.808 eV)

688 731 -- 1.242 au C 1.00 1 D:xz 29

(33.808 eV)

689 732 -- 1.242 au C 1.00 1 D:yz 29

(33.808 eV)

690 733 -- 4.016 au C 1.00 1 F:z3 29

(109.273 eV)

691 734 -- 4.016 au C 1.00 1 F:z 29

(109.273 eV)

692 735 -- 4.016 au C 1.00 1 F:xyz 29

(109.273 eV)

693 736 -- 4.016 au C 1.00 1 F:z2x 29

(109.273 eV)

694 737 -- 4.016 au C 1.00 1 F:z2y 29

(109.273 eV)

695 738 -- 4.016 au C 1.00 1 F:x 29

(109.273 eV)

696 739 -- 4.016 au C 1.00 1 F:y 29

(109.273 eV)

697 740 2.000 -0.501 au C 1.00 1 S 30

(-13.631 eV)

698 741 -- 0.431 au C 1.00 2 S 30

(11.741 eV)

699 742 -- 9.944 au C 1.00 3 S 30

(270.596 eV)

700 743 0.667 -0.193 au C 1.00 1 P:x 30

(-5.263 eV)

701 744 -- 0.168 au C 1.00 2 P:x 30

(4.578 eV)

702 745 -- 2.012 au C 1.00 3 P:x 30

(54.761 eV)

703 746 0.667 -0.193 au C 1.00 1 P:y 30

(-5.263 eV)

704 747 -- 0.168 au C 1.00 2 P:y 30

(4.578 eV)

705 748 -- 2.012 au C 1.00 3 P:y 30

(54.761 eV)

706 749 0.667 -0.193 au C 1.00 1 P:z 30

(-5.263 eV)

707 750 -- 0.168 au C 1.00 2 P:z 30

(4.578 eV)

708 751 -- 2.012 au C 1.00 3 P:z 30

(54.761 eV)

709 752 -- 1.242 au C 1.00 1 D:z2 30

(33.808 eV)

710 753 -- 1.242 au C 1.00 1 D:x2-y2 30

(33.808 eV)

711 754 -- 1.242 au C 1.00 1 D:xy 30

(33.808 eV)

712 755 -- 1.242 au C 1.00 1 D:xz 30

(33.808 eV)

713 756 -- 1.242 au C 1.00 1 D:yz 30

(33.808 eV)

714 757 -- 4.016 au C 1.00 1 F:z3 30

(109.273 eV)

715 758 -- 4.016 au C 1.00 1 F:z 30

(109.273 eV)

716 759 -- 4.016 au C 1.00 1 F:xyz 30

(109.273 eV)

717 760 -- 4.016 au C 1.00 1 F:z2x 30

(109.273 eV)

718 761 -- 4.016 au C 1.00 1 F:z2y 30

(109.273 eV)

719 762 -- 4.016 au C 1.00 1 F:x 30

(109.273 eV)

720 763 -- 4.016 au C 1.00 1 F:y 30

(109.273 eV)

721 764 2.000 -0.501 au C 1.00 1 S 31

(-13.631 eV)

722 765 -- 0.431 au C 1.00 2 S 31

(11.741 eV)

723 766 -- 9.944 au C 1.00 3 S 31

(270.596 eV)

724 767 0.667 -0.193 au C 1.00 1 P:x 31

(-5.263 eV)

725 768 -- 0.168 au C 1.00 2 P:x 31

(4.578 eV)

726 769 -- 2.012 au C 1.00 3 P:x 31

(54.761 eV)

727 770 0.667 -0.193 au C 1.00 1 P:y 31

(-5.263 eV)

728 771 -- 0.168 au C 1.00 2 P:y 31

(4.578 eV)

729 772 -- 2.012 au C 1.00 3 P:y 31

(54.761 eV)

730 773 0.667 -0.193 au C 1.00 1 P:z 31

(-5.263 eV)

731 774 -- 0.168 au C 1.00 2 P:z 31

(4.578 eV)

732 775 -- 2.012 au C 1.00 3 P:z 31

(54.761 eV)

733 776 -- 1.242 au C 1.00 1 D:z2 31

(33.808 eV)

734 777 -- 1.242 au C 1.00 1 D:x2-y2 31

(33.808 eV)

735 778 -- 1.242 au C 1.00 1 D:xy 31

(33.808 eV)

736 779 -- 1.242 au C 1.00 1 D:xz 31

(33.808 eV)

737 780 -- 1.242 au C 1.00 1 D:yz 31

			(33.808 eV)				
738	781	--	4.016 au	C	1.00	1 F:z3	31
			(109.273 eV)				
739	782	--	4.016 au	C	1.00	1 F:z	31
			(109.273 eV)				
740	783	--	4.016 au	C	1.00	1 F:xyz	31
			(109.273 eV)				
741	784	--	4.016 au	C	1.00	1 F:z2x	31
			(109.273 eV)				
742	785	--	4.016 au	C	1.00	1 F:z2y	31
			(109.273 eV)				
743	786	--	4.016 au	C	1.00	1 F:x	31
			(109.273 eV)				
744	787	--	4.016 au	C	1.00	1 F:y	31
			(109.273 eV)				
745	788	2.000	-0.501 au	C	1.00	1 S	32
			(-13.631 eV)				
746	789	--	0.431 au	C	1.00	2 S	32
			(11.741 eV)				
747	790	--	9.944 au	C	1.00	3 S	32
			(270.596 eV)				
748	791	0.667	-0.193 au	C	1.00	1 P:x	32
			(-5.263 eV)				
749	792	--	0.168 au	C	1.00	2 P:x	32
			(4.578 eV)				
750	793	--	2.012 au	C	1.00	3 P:x	32
			(54.761 eV)				
751	794	0.667	-0.193 au	C	1.00	1 P:y	32
			(-5.263 eV)				
752	795	--	0.168 au	C	1.00	2 P:y	32
			(4.578 eV)				

753 796 -- 2.012 au C 1.00 3 P:y 32

(54.761 eV)

754 797 0.667 -0.193 au C 1.00 1 P:z 32

(-5.263 eV)

755 798 -- 0.168 au C 1.00 2 P:z 32

(4.578 eV)

756 799 -- 2.012 au C 1.00 3 P:z 32

(54.761 eV)

757 800 -- 1.242 au C 1.00 1 D:z2 32

(33.808 eV)

758 801 -- 1.242 au C 1.00 1 D:x2-y2 32

(33.808 eV)

759 802 -- 1.242 au C 1.00 1 D:xy 32

(33.808 eV)

760 803 -- 1.242 au C 1.00 1 D:xz 32

(33.808 eV)

761 804 -- 1.242 au C 1.00 1 D:yz 32

(33.808 eV)

762 805 -- 4.016 au C 1.00 1 F:z3 32

(109.273 eV)

763 806 -- 4.016 au C 1.00 1 F:z 32

(109.273 eV)

764 807 -- 4.016 au C 1.00 1 F:xyz 32

(109.273 eV)

765 808 -- 4.016 au C 1.00 1 F:z2x 32

(109.273 eV)

766 809 -- 4.016 au C 1.00 1 F:z2y 32

(109.273 eV)

767 810 -- 4.016 au C 1.00 1 F:x 32

(109.273 eV)

768 811 -- 4.016 au C 1.00 1 F:y 32

(109.273 eV)

769	812	2.000	-0.501 au	C	1.00	1 S	33
		(-13.631 eV)					
770	813	--	0.431 au	C	1.00	2 S	33
		(11.741 eV)					
771	814	--	9.944 au	C	1.00	3 S	33
		(270.596 eV)					
772	815	0.667	-0.193 au	C	1.00	1 P:x	33
		(-5.263 eV)					
773	816	--	0.168 au	C	1.00	2 P:x	33
		(4.578 eV)					
774	817	--	2.012 au	C	1.00	3 P:x	33
		(54.761 eV)					
775	818	0.667	-0.193 au	C	1.00	1 P:y	33
		(-5.263 eV)					
776	819	--	0.168 au	C	1.00	2 P:y	33
		(4.578 eV)					
777	820	--	2.012 au	C	1.00	3 P:y	33
		(54.761 eV)					
778	821	0.667	-0.193 au	C	1.00	1 P:z	33
		(-5.263 eV)					
779	822	--	0.168 au	C	1.00	2 P:z	33
		(4.578 eV)					
780	823	--	2.012 au	C	1.00	3 P:z	33
		(54.761 eV)					
781	824	--	1.242 au	C	1.00	1 D:z2	33
		(33.808 eV)					
782	825	--	1.242 au	C	1.00	1 D:x2-y2	33
		(33.808 eV)					
783	826	--	1.242 au	C	1.00	1 D:xy	33
		(33.808 eV)					

784 827 -- 1.242 au C 1.00 1 D:xz 33
(33.808 eV)
785 828 -- 1.242 au C 1.00 1 D:yz 33
(33.808 eV)
786 829 -- 4.016 au C 1.00 1 F:z3 33
(109.273 eV)
787 830 -- 4.016 au C 1.00 1 F:z 33
(109.273 eV)
788 831 -- 4.016 au C 1.00 1 F:xyz 33
(109.273 eV)
789 832 -- 4.016 au C 1.00 1 F:z2x 33
(109.273 eV)
790 833 -- 4.016 au C 1.00 1 F:z2y 33
(109.273 eV)
791 834 -- 4.016 au C 1.00 1 F:x 33
(109.273 eV)
792 835 -- 4.016 au C 1.00 1 F:y 33
(109.273 eV)
793 836 2.000 -0.501 au C 1.00 1 S 34
(-13.631 eV)
794 837 -- 0.431 au C 1.00 2 S 34
(11.741 eV)
795 838 -- 9.944 au C 1.00 3 S 34
(270.596 eV)
796 839 0.667 -0.193 au C 1.00 1 P:x 34
(-5.263 eV)
797 840 -- 0.168 au C 1.00 2 P:x 34
(4.578 eV)
798 841 -- 2.012 au C 1.00 3 P:x 34
(54.761 eV)
799 842 0.667 -0.193 au C 1.00 1 P:y 34

(-5.263 eV)

800 843 -- 0.168 au C 1.00 2 P:y 34

(4.578 eV)

801 844 -- 2.012 au C 1.00 3 P:y 34

(54.761 eV)

802 845 0.667 -0.193 au C 1.00 1 P:z 34

(-5.263 eV)

803 846 -- 0.168 au C 1.00 2 P:z 34

(4.578 eV)

804 847 -- 2.012 au C 1.00 3 P:z 34

(54.761 eV)

805 848 -- 1.242 au C 1.00 1 D:z2 34

(33.808 eV)

806 849 -- 1.242 au C 1.00 1 D:x2-y2 34

(33.808 eV)

807 850 -- 1.242 au C 1.00 1 D:xy 34

(33.808 eV)

808 851 -- 1.242 au C 1.00 1 D:xz 34

(33.808 eV)

809 852 -- 1.242 au C 1.00 1 D:yz 34

(33.808 eV)

810 853 -- 4.016 au C 1.00 1 F:z3 34

(109.273 eV)

811 854 -- 4.016 au C 1.00 1 F:z 34

(109.273 eV)

812 855 -- 4.016 au C 1.00 1 F:xyz 34

(109.273 eV)

813 856 -- 4.016 au C 1.00 1 F:z2x 34

(109.273 eV)

814 857 -- 4.016 au C 1.00 1 F:z2y 34

(109.273 eV)

815 858 -- 4.016 au C 1.00 1 F:x 34

(109.273 eV)

816 859 -- 4.016 au C 1.00 1 F:y 34

(109.273 eV)

817 860 2.000 -0.501 au C 1.00 1 S 35

(-13.631 eV)

818 861 -- 0.431 au C 1.00 2 S 35

(11.741 eV)

819 862 -- 9.944 au C 1.00 3 S 35

(270.596 eV)

820 863 0.667 -0.193 au C 1.00 1 P:x 35

(-5.263 eV)

821 864 -- 0.168 au C 1.00 2 P:x 35

(4.578 eV)

822 865 -- 2.012 au C 1.00 3 P:x 35

(54.761 eV)

823 866 0.667 -0.193 au C 1.00 1 P:y 35

(-5.263 eV)

824 867 -- 0.168 au C 1.00 2 P:y 35

(4.578 eV)

825 868 -- 2.012 au C 1.00 3 P:y 35

(54.761 eV)

826 869 0.667 -0.193 au C 1.00 1 P:z 35

(-5.263 eV)

827 870 -- 0.168 au C 1.00 2 P:z 35

(4.578 eV)

828 871 -- 2.012 au C 1.00 3 P:z 35

(54.761 eV)

829 872 -- 1.242 au C 1.00 1 D:z2 35

(33.808 eV)

830 873 -- 1.242 au C 1.00 1 D:x2-y2 35

(33.808 eV)

831 874 -- 1.242 au C 1.00 1 D:xy 35

(33.808 eV)

832 875 -- 1.242 au C 1.00 1 D:xz 35

(33.808 eV)

833 876 -- 1.242 au C 1.00 1 D:yz 35

(33.808 eV)

834 877 -- 4.016 au C 1.00 1 F:z3 35

(109.273 eV)

835 878 -- 4.016 au C 1.00 1 F:z 35

(109.273 eV)

836 879 -- 4.016 au C 1.00 1 F:xyz 35

(109.273 eV)

837 880 -- 4.016 au C 1.00 1 F:z2x 35

(109.273 eV)

838 881 -- 4.016 au C 1.00 1 F:z2y 35

(109.273 eV)

839 882 -- 4.016 au C 1.00 1 F:x 35

(109.273 eV)

840 883 -- 4.016 au C 1.00 1 F:y 35

(109.273 eV)

841 884 2.000 -0.501 au C 1.00 1 S 36

(-13.631 eV)

842 885 -- 0.431 au C 1.00 2 S 36

(11.741 eV)

843 886 -- 9.944 au C 1.00 3 S 36

(270.596 eV)

844 887 0.667 -0.193 au C 1.00 1 P:x 36

(-5.263 eV)

845 888 -- 0.168 au C 1.00 2 P:x 36

(4.578 eV)

846 889 -- 2.012 au C 1.00 3 P:x 36

(54.761 eV)

847 890 0.667 -0.193 au C 1.00 1 P:y 36

(-5.263 eV)

848 891 -- 0.168 au C 1.00 2 P:y 36

(4.578 eV)

849 892 -- 2.012 au C 1.00 3 P:y 36

(54.761 eV)

850 893 0.667 -0.193 au C 1.00 1 P:z 36

(-5.263 eV)

851 894 -- 0.168 au C 1.00 2 P:z 36

(4.578 eV)

852 895 -- 2.012 au C 1.00 3 P:z 36

(54.761 eV)

853 896 -- 1.242 au C 1.00 1 D:z2 36

(33.808 eV)

854 897 -- 1.242 au C 1.00 1 D:x2-y2 36

(33.808 eV)

855 898 -- 1.242 au C 1.00 1 D:xy 36

(33.808 eV)

856 899 -- 1.242 au C 1.00 1 D:xz 36

(33.808 eV)

857 900 -- 1.242 au C 1.00 1 D:yz 36

(33.808 eV)

858 901 -- 4.016 au C 1.00 1 F:z3 36

(109.273 eV)

859 902 -- 4.016 au C 1.00 1 F:z 36

(109.273 eV)

860 903 -- 4.016 au C 1.00 1 F:xyz 36

(109.273 eV)

861 904 -- 4.016 au C 1.00 1 F:z2x 36

(109.273 eV)

862 905 -- 4.016 au C 1.00 1 F:z2y 36

(109.273 eV)

863 906 -- 4.016 au C 1.00 1 F:x 36

(109.273 eV)

864 907 -- 4.016 au C 1.00 1 F:y 36

(109.273 eV)

865 908 2.000 -0.501 au C 1.00 1 S 37

(-13.631 eV)

866 909 -- 0.431 au C 1.00 2 S 37

(11.741 eV)

867 910 -- 9.944 au C 1.00 3 S 37

(270.596 eV)

868 911 0.667 -0.193 au C 1.00 1 P:x 37

(-5.263 eV)

869 912 -- 0.168 au C 1.00 2 P:x 37

(4.578 eV)

870 913 -- 2.012 au C 1.00 3 P:x 37

(54.761 eV)

871 914 0.667 -0.193 au C 1.00 1 P:y 37

(-5.263 eV)

872 915 -- 0.168 au C 1.00 2 P:y 37

(4.578 eV)

873 916 -- 2.012 au C 1.00 3 P:y 37

(54.761 eV)

874 917 0.667 -0.193 au C 1.00 1 P:z 37

(-5.263 eV)

875 918 -- 0.168 au C 1.00 2 P:z 37

(4.578 eV)

876 919 -- 2.012 au C 1.00 3 P:z 37

(54.761 eV)

877 920 -- 1.242 au C 1.00 1 D:z2 37

(33.808 eV)

878 921 -- 1.242 au C 1.00 1 D:x2-y2 37

(33.808 eV)

879 922 -- 1.242 au C 1.00 1 D:xy 37

(33.808 eV)

880 923 -- 1.242 au C 1.00 1 D:xz 37

(33.808 eV)

881 924 -- 1.242 au C 1.00 1 D:yz 37

(33.808 eV)

882 925 -- 4.016 au C 1.00 1 F:z3 37

(109.273 eV)

883 926 -- 4.016 au C 1.00 1 F:z 37

(109.273 eV)

884 927 -- 4.016 au C 1.00 1 F:xyz 37

(109.273 eV)

885 928 -- 4.016 au C 1.00 1 F:z2x 37

(109.273 eV)

886 929 -- 4.016 au C 1.00 1 F:z2y 37

(109.273 eV)

887 930 -- 4.016 au C 1.00 1 F:x 37

(109.273 eV)

888 931 -- 4.016 au C 1.00 1 F:y 37

(109.273 eV)

889 932 2.000 -0.501 au C 1.00 1 S 38

(-13.631 eV)

890 933 -- 0.431 au C 1.00 2 S 38

(11.741 eV)

891 934 -- 9.944 au C 1.00 3 S 38

(270.596 eV)

892 935 0.667 -0.193 au C 1.00 1 P:x 38

(-5.263 eV)

893 936 -- 0.168 au C 1.00 2 P:x 38

(4.578 eV)

894 937 -- 2.012 au C 1.00 3 P:x 38

(54.761 eV)

895 938 0.667 -0.193 au C 1.00 1 P:y 38

(-5.263 eV)

896 939 -- 0.168 au C 1.00 2 P:y 38

(4.578 eV)

897 940 -- 2.012 au C 1.00 3 P:y 38

(54.761 eV)

898 941 0.667 -0.193 au C 1.00 1 P:z 38

(-5.263 eV)

899 942 -- 0.168 au C 1.00 2 P:z 38

(4.578 eV)

900 943 -- 2.012 au C 1.00 3 P:z 38

(54.761 eV)

901 944 -- 1.242 au C 1.00 1 D:z2 38

(33.808 eV)

902 945 -- 1.242 au C 1.00 1 D:x2-y2 38

(33.808 eV)

903 946 -- 1.242 au C 1.00 1 D:xy 38

(33.808 eV)

904 947 -- 1.242 au C 1.00 1 D:xz 38

(33.808 eV)

905 948 -- 1.242 au C 1.00 1 D:yz 38

(33.808 eV)

906 949 -- 4.016 au C 1.00 1 F:z3 38

(109.273 eV)

907 950 -- 4.016 au C 1.00 1 F:z 38

(109.273 eV)

908 951 -- 4.016 au C 1.00 1 F:xyz 38

(109.273 eV)

909 952 -- 4.016 au C 1.00 1 F:z2x 38

(109.273 eV)

910 953 -- 4.016 au C 1.00 1 F:z2y 38

(109.273 eV)

911 954 -- 4.016 au C 1.00 1 F:x 38

(109.273 eV)

912 955 -- 4.016 au C 1.00 1 F:y 38

(109.273 eV)

913 956 2.000 -0.501 au C 1.00 1 S 39

(-13.631 eV)

914 957 -- 0.431 au C 1.00 2 S 39

(11.741 eV)

915 958 -- 9.944 au C 1.00 3 S 39

(270.596 eV)

916 959 0.667 -0.193 au C 1.00 1 P:x 39

(-5.263 eV)

917 960 -- 0.168 au C 1.00 2 P:x 39

(4.578 eV)

918 961 -- 2.012 au C 1.00 3 P:x 39

(54.761 eV)

919 962 0.667 -0.193 au C 1.00 1 P:y 39

(-5.263 eV)

920 963 -- 0.168 au C 1.00 2 P:y 39

(4.578 eV)

921 964 -- 2.012 au C 1.00 3 P:y 39

(54.761 eV)

922 965 0.667 -0.193 au C 1.00 1 P:z 39

(-5.263 eV)

923 966 -- 0.168 au C 1.00 2 P:z 39

(4.578 eV)

924 967 -- 2.012 au C 1.00 3 P:z 39

(54.761 eV)

925 968 -- 1.242 au C 1.00 1 D:z2 39

(33.808 eV)

926 969 -- 1.242 au C 1.00 1 D:x2-y2 39

(33.808 eV)

927 970 -- 1.242 au C 1.00 1 D:xy 39

(33.808 eV)

928 971 -- 1.242 au C 1.00 1 D:xz 39

(33.808 eV)

929 972 -- 1.242 au C 1.00 1 D:yz 39

(33.808 eV)

930 973 -- 4.016 au C 1.00 1 F:z3 39

(109.273 eV)

931 974 -- 4.016 au C 1.00 1 F:z 39

(109.273 eV)

932 975 -- 4.016 au C 1.00 1 F:xyz 39

(109.273 eV)

933 976 -- 4.016 au C 1.00 1 F:z2x 39

(109.273 eV)

934 977 -- 4.016 au C 1.00 1 F:z2y 39

(109.273 eV)

935 978 -- 4.016 au C 1.00 1 F:x 39

(109.273 eV)

936 979 -- 4.016 au C 1.00 1 F:y 39

(109.273 eV)

937 980 2.000 -0.501 au C 1.00 1 S 40

(-13.631 eV)

938 981 -- 0.431 au C 1.00 2 S 40

(11.741 eV)

939 982 -- 9.944 au C 1.00 3 S 40

(270.596 eV)

940 983 0.667 -0.193 au C 1.00 1 P:x 40

(-5.263 eV)

941 984 -- 0.168 au C 1.00 2 P:x 40

(4.578 eV)

942 985 -- 2.012 au C 1.00 3 P:x 40

(54.761 eV)

943 986 0.667 -0.193 au C 1.00 1 P:y 40

(-5.263 eV)

944 987 -- 0.168 au C 1.00 2 P:y 40

(4.578 eV)

945 988 -- 2.012 au C 1.00 3 P:y 40

(54.761 eV)

946 989 0.667 -0.193 au C 1.00 1 P:z 40

(-5.263 eV)

947 990 -- 0.168 au C 1.00 2 P:z 40

(4.578 eV)

948 991 -- 2.012 au C 1.00 3 P:z 40

(54.761 eV)

949 992 -- 1.242 au C 1.00 1 D:z2 40

(33.808 eV)

950 993 -- 1.242 au C 1.00 1 D:x2-y2 40

(33.808 eV)

951 994 -- 1.242 au C 1.00 1 D:xy 40

(33.808 eV)

952 995 -- 1.242 au C 1.00 1 D:xz 40

(33.808 eV)

953 996 -- 1.242 au C 1.00 1 D:yz 40

(33.808 eV)

954 997 -- 4.016 au C 1.00 1 F:z3 40

(109.273 eV)

955 998 -- 4.016 au C 1.00 1 F:z 40

(109.273 eV)

956 999 -- 4.016 au C 1.00 1 F:xyz 40

(109.273 eV)

957 1000 -- 4.016 au C 1.00 1 F:z2x 40

(109.273 eV)

958 1001 -- 4.016 au C 1.00 1 F:z2y 40

(109.273 eV)

959 1002 -- 4.016 au C 1.00 1 F:x 40

(109.273 eV)

960 1003 -- 4.016 au C 1.00 1 F:y 40

(109.273 eV)

961 1004 2.000 -0.501 au C 1.00 1 S 41

(-13.631 eV)

962 1005 -- 0.431 au C 1.00 2 S 41

(11.741 eV)

963 1006 -- 9.944 au C 1.00 3 S 41

(270.596 eV)

964 1007 0.667 -0.193 au C 1.00 1 P:x 41

(-5.263 eV)

965 1008 -- 0.168 au C 1.00 2 P:x 41

(4.578 eV)

966 1009 -- 2.012 au C 1.00 3 P:x 41

(54.761 eV)

967 1010 0.667 -0.193 au C 1.00 1 P:y 41

(-5.263 eV)

968 1011 -- 0.168 au C 1.00 2 P:y 41

(4.578 eV)

969 1012 -- 2.012 au C 1.00 3 P:y 41

(54.761 eV)

970 1013 0.667 -0.193 au C 1.00 1 P:z 41

(-5.263 eV)

971 1014 -- 0.168 au C 1.00 2 P:z 41

(4.578 eV)

972 1015 -- 2.012 au C 1.00 3 P:z 41

(54.761 eV)

973 1016 -- 1.242 au C 1.00 1 D:z2 41

(33.808 eV)

974 1017 -- 1.242 au C 1.00 1 D:x2-y2 41

(33.808 eV)

975 1018 -- 1.242 au C 1.00 1 D:xy 41

(33.808 eV)

976 1019 -- 1.242 au C 1.00 1 D:xz 41

(33.808 eV)

977 1020 -- 1.242 au C 1.00 1 D:yz 41

(33.808 eV)

978 1021 -- 4.016 au C 1.00 1 F:z3 41

(109.273 eV)

979 1022 -- 4.016 au C 1.00 1 F:z 41

(109.273 eV)

980 1023 -- 4.016 au C 1.00 1 F:xyz 41

(109.273 eV)

981 1024 -- 4.016 au C 1.00 1 F:z2x 41

(109.273 eV)

982 1025 -- 4.016 au C 1.00 1 F:z2y 41

(109.273 eV)

983 1026 -- 4.016 au C 1.00 1 F:x 41

(109.273 eV)

984 1027 -- 4.016 au C 1.00 1 F:y 41

(109.273 eV)

985 1028 2.000 -0.501 au C 1.00 1 S 42

(-13.631 eV)

986 1029 -- 0.431 au C 1.00 2 S 42

(11.741 eV)

987 1030 -- 9.944 au C 1.00 3 S 42

(270.596 eV)

988 1031 0.667 -0.193 au C 1.00 1 P:x 42

(-5.263 eV)

989 1032 -- 0.168 au C 1.00 2 P:x 42

(4.578 eV)

990 1033 -- 2.012 au C 1.00 3 P:x 42

(54.761 eV)

991 1034 0.667 -0.193 au C 1.00 1 P:y 42

(-5.263 eV)

992 1035 -- 0.168 au C 1.00 2 P:y 42

(4.578 eV)

993 1036 -- 2.012 au C 1.00 3 P:y 42

(54.761 eV)

994 1037 0.667 -0.193 au C 1.00 1 P:z 42

(-5.263 eV)

995 1038 -- 0.168 au C 1.00 2 P:z 42

(4.578 eV)

996 1039 -- 2.012 au C 1.00 3 P:z 42

(54.761 eV)

997 1040 -- 1.242 au C 1.00 1 D:z2 42

(33.808 eV)

998 1041 -- 1.242 au C 1.00 1 D:x2-y2 42

(33.808 eV)

999 1042 -- 1.242 au C 1.00 1 D:xy 42

(33.808 eV)

1000 1043 -- 1.242 au C 1.00 1 D:xz 42

(33.808 eV)

1001 1044 -- 1.242 au C 1.00 1 D:yz 42

(33.808 eV)

1002 1045 -- 4.016 au C 1.00 1 F:z3 42

(109.273 eV)

1003 1046 -- 4.016 au C 1.00 1 F:z 42

(109.273 eV)

1004 1047 -- 4.016 au C 1.00 1 F:xyz 42

(109.273 eV)

1005 1048 -- 4.016 au C 1.00 1 F:z2x 42

(109.273 eV)

1006 1049 -- 4.016 au C 1.00 1 F:z2y 42

(109.273 eV)

1007 1050 -- 4.016 au C 1.00 1 F:x 42

(109.273 eV)

1008 1051 -- 4.016 au C 1.00 1 F:y 42

(109.273 eV)

1009 1052 2.000 -0.678 au N 1.00 1 S 43

(-18.438 eV)

1010 1053 -- 0.592 au N 1.00 2 S 43

(16.116 eV)

1011 1054 -- 12.457 au N 1.00 3 S 43

(338.981 eV)

1012 1055 1.000 -0.260 au N 1.00 1 P:x 43

(-7.073 eV)

1013 1056 -- 0.281 au N 1.00 2 P:x 43

(7.645 eV)

1014 1057 -- 3.276 au N 1.00 3 P:x 43

(89.137 eV)

1015 1058 1.000 -0.260 au N 1.00 1 P:y 43

(-7.073 eV)

1016 1059 -- 0.281 au N 1.00 2 P:y 43

(7.645 eV)

1017 1060 -- 3.276 au N 1.00 3 P:y 43

(89.137 eV)

1018 1061 1.000 -0.260 au N 1.00 1 P:z 43

(-7.073 eV)

1019 1062 -- 0.281 au N 1.00 2 P:z 43

(7.645 eV)

1020 1063 -- 3.276 au N 1.00 3 P:z 43

(89.137 eV)

1021 1064 -- 1.250 au N 1.00 1 D:z2 43

(34.001 eV)

1022 1065 -- 1.250 au N 1.00 1 D:x2-y2 43

(34.001 eV)

1023 1066 -- 1.250 au N 1.00 1 D:xy 43

(34.001 eV)

1024 1067 -- 1.250 au N 1.00 1 D:xz 43

(34.001 eV)

1025 1068 -- 1.250 au N 1.00 1 D:yz 43

(34.001 eV)

1026 1069 -- 4.013 au N 1.00 1 F:z3 43

(109.204 eV)

1027 1070 -- 4.013 au N 1.00 1 F:z 43

(109.204 eV)

1028 1071 -- 4.013 au N 1.00 1 F:xyz 43

(109.204 eV)

1029 1072 -- 4.013 au N 1.00 1 F:z2x 43

(109.204 eV)

1030 1073 -- 4.013 au N 1.00 1 F:z2y 43

(109.204 eV)

1031 1074 -- 4.013 au N 1.00 1 F:x 43

(109.204 eV)

1032 1075 -- 4.013 au N 1.00 1 F:y 43

(109.204 eV)

1033 1076 1.000 -0.240 au H 1.00 1 S 44

(-6.532 eV)

1034 1077 -- 0.088 au H 1.00 2 S 44

(2.394 eV)

1035 1078 -- 1.502 au H 1.00 3 S 44

(40.877 eV)

1036 1079 -- 0.406 au H 1.00 1 P:x 44

(11.052 eV)

1037 1080 -- 0.406 au H 1.00 1 P:y 44

(11.052 eV)

1038 1081 -- 0.406 au H 1.00 1 P:z 44

(11.052 eV)

1039 1082 -- 2.570 au H 1.00 1 D:z2 44

(69.925 eV)

1040 1083 -- 2.570 au H 1.00 1 D:x2-y2 44

(69.925 eV)

1041 1084 -- 2.570 au H 1.00 1 D:xy 44

(69.925 eV)

1042 1085 -- 2.570 au H 1.00 1 D:xz 44

(69.925 eV)

1043 1086 -- 2.570 au H 1.00 1 D:yz 44

(69.925 eV)

1044 1087 1.000 -0.240 au H 1.00 1 S 45

(-6.532 eV)

1045 1088 -- 0.088 au H 1.00 2 S 45

(2.394 eV)

1046 1089 -- 1.502 au H 1.00 3 S 45

(40.877 eV)

1047 1090 -- 0.406 au H 1.00 1 P:x 45

		(	11.052 eV)				
1048	1091	--	0.406 au	H	1.00	1 P:y	45
		(	11.052 eV)				
1049	1092	--	0.406 au	H	1.00	1 P:z	45
		(	11.052 eV)				
1050	1093	--	2.570 au	H	1.00	1 D:z2	45
		(	69.925 eV)				
1051	1094	--	2.570 au	H	1.00	1 D:x2-y2	45
		(	69.925 eV)				
1052	1095	--	2.570 au	H	1.00	1 D:xy	45
		(	69.925 eV)				
1053	1096	--	2.570 au	H	1.00	1 D:xz	45
		(	69.925 eV)				
1054	1097	--	2.570 au	H	1.00	1 D:yz	45
		(	69.925 eV)				
1055	1098	1.000	-0.240 au	H	1.00	1 S	46
		(	-6.532 eV)				
1056	1099	--	0.088 au	H	1.00	2 S	46
		(	2.394 eV)				
1057	1100	--	1.502 au	H	1.00	3 S	46
		(	40.877 eV)				
1058	1101	--	0.406 au	H	1.00	1 P:x	46
		(	11.052 eV)				
1059	1102	--	0.406 au	H	1.00	1 P:y	46
		(	11.052 eV)				
1060	1103	--	0.406 au	H	1.00	1 P:z	46
		(	11.052 eV)				
1061	1104	--	2.570 au	H	1.00	1 D:z2	46
		(	69.925 eV)				
1062	1105	--	2.570 au	H	1.00	1 D:x2-y2	46
		(	69.925 eV)				

1063 1106 -- 2.570 au H 1.00 1 D:xy 46

(69.925 eV)

1064 1107 -- 2.570 au H 1.00 1 D:xz 46

(69.925 eV)

1065 1108 -- 2.570 au H 1.00 1 D:yz 46

(69.925 eV)

1066 1109 1.000 -0.240 au H 1.00 1 S 47

(-6.532 eV)

1067 1110 -- 0.088 au H 1.00 2 S 47

(2.394 eV)

1068 1111 -- 1.502 au H 1.00 3 S 47

(40.877 eV)

1069 1112 -- 0.406 au H 1.00 1 P:x 47

(11.052 eV)

1070 1113 -- 0.406 au H 1.00 1 P:y 47

(11.052 eV)

1071 1114 -- 0.406 au H 1.00 1 P:z 47

(11.052 eV)

1072 1115 -- 2.570 au H 1.00 1 D:z2 47

(69.925 eV)

1073 1116 -- 2.570 au H 1.00 1 D:x2-y2 47

(69.925 eV)

1074 1117 -- 2.570 au H 1.00 1 D:xy 47

(69.925 eV)

1075 1118 -- 2.570 au H 1.00 1 D:xz 47

(69.925 eV)

1076 1119 -- 2.570 au H 1.00 1 D:yz 47

(69.925 eV)

1077 1120 1.000 -0.240 au H 1.00 1 S 48

(-6.532 eV)

1078 1121 -- 0.088 au H 1.00 2 S 48

(2.394 eV)

1079 1122 -- 1.502 au H 1.00 3 S 48

(40.877 eV)

1080 1123 -- 0.406 au H 1.00 1 P:x 48

(11.052 eV)

1081 1124 -- 0.406 au H 1.00 1 P:y 48

(11.052 eV)

1082 1125 -- 0.406 au H 1.00 1 P:z 48

(11.052 eV)

1083 1126 -- 2.570 au H 1.00 1 D:z2 48

(69.925 eV)

1084 1127 -- 2.570 au H 1.00 1 D:x2-y2 48

(69.925 eV)

1085 1128 -- 2.570 au H 1.00 1 D:xy 48

(69.925 eV)

1086 1129 -- 2.570 au H 1.00 1 D:xz 48

(69.925 eV)

1087 1130 -- 2.570 au H 1.00 1 D:yz 48

(69.925 eV)

1088 1131 1.000 -0.240 au H 1.00 1 S 49

(-6.532 eV)

1089 1132 -- 0.088 au H 1.00 2 S 49

(2.394 eV)

1090 1133 -- 1.502 au H 1.00 3 S 49

(40.877 eV)

1091 1134 -- 0.406 au H 1.00 1 P:x 49

(11.052 eV)

1092 1135 -- 0.406 au H 1.00 1 P:y 49

(11.052 eV)

1093 1136 -- 0.406 au H 1.00 1 P:z 49

(11.052 eV)

1094 1137 -- 2.570 au H 1.00 1 D:z2 49

(69.925 eV)

1095 1138 -- 2.570 au H 1.00 1 D:x2-y2 49

(69.925 eV)

1096 1139 -- 2.570 au H 1.00 1 D:xy 49

(69.925 eV)

1097 1140 -- 2.570 au H 1.00 1 D:xz 49

(69.925 eV)

1098 1141 -- 2.570 au H 1.00 1 D:yz 49

(69.925 eV)

1099 1142 1.000 -0.240 au H 1.00 1 S 50

(-6.532 eV)

1100 1143 -- 0.088 au H 1.00 2 S 50

(2.394 eV)

1101 1144 -- 1.502 au H 1.00 3 S 50

(40.877 eV)

1102 1145 -- 0.406 au H 1.00 1 P:x 50

(11.052 eV)

1103 1146 -- 0.406 au H 1.00 1 P:y 50

(11.052 eV)

1104 1147 -- 0.406 au H 1.00 1 P:z 50

(11.052 eV)

1105 1148 -- 2.570 au H 1.00 1 D:z2 50

(69.925 eV)

1106 1149 -- 2.570 au H 1.00 1 D:x2-y2 50

(69.925 eV)

1107 1150 -- 2.570 au H 1.00 1 D:xy 50

(69.925 eV)

1108 1151 -- 2.570 au H 1.00 1 D:xz 50

(69.925 eV)

1109 1152 -- 2.570 au H 1.00 1 D:yz 50

(69.925 eV)

1110 1153 1.000 -0.240 au H 1.00 1 S 51

(-6.532 eV)

1111 1154 -- 0.088 au H 1.00 2 S 51

(2.394 eV)

1112 1155 -- 1.502 au H 1.00 3 S 51

(40.877 eV)

1113 1156 -- 0.406 au H 1.00 1 P:x 51

(11.052 eV)

1114 1157 -- 0.406 au H 1.00 1 P:y 51

(11.052 eV)

1115 1158 -- 0.406 au H 1.00 1 P:z 51

(11.052 eV)

1116 1159 -- 2.570 au H 1.00 1 D:z2 51

(69.925 eV)

1117 1160 -- 2.570 au H 1.00 1 D:x2-y2 51

(69.925 eV)

1118 1161 -- 2.570 au H 1.00 1 D:xy 51

(69.925 eV)

1119 1162 -- 2.570 au H 1.00 1 D:xz 51

(69.925 eV)

1120 1163 -- 2.570 au H 1.00 1 D:yz 51

(69.925 eV)

1121 1164 1.000 -0.240 au H 1.00 1 S 52

(-6.532 eV)

1122 1165 -- 0.088 au H 1.00 2 S 52

(2.394 eV)

1123 1166 -- 1.502 au H 1.00 3 S 52

(40.877 eV)

1124 1167 -- 0.406 au H 1.00 1 P:x 52

(11.052 eV)

1125 1168 -- 0.406 au H 1.00 1 P:y 52
(11.052 eV)
1126 1169 -- 0.406 au H 1.00 1 P:z 52
(11.052 eV)
1127 1170 -- 2.570 au H 1.00 1 D:z2 52
(69.925 eV)
1128 1171 -- 2.570 au H 1.00 1 D:x2-y2 52
(69.925 eV)
1129 1172 -- 2.570 au H 1.00 1 D:xy 52
(69.925 eV)
1130 1173 -- 2.570 au H 1.00 1 D:xz 52
(69.925 eV)
1131 1174 -- 2.570 au H 1.00 1 D:yz 52
(69.925 eV)
1132 1175 1.000 -0.240 au H 1.00 1 S 53
(-6.532 eV)
1133 1176 -- 0.088 au H 1.00 2 S 53
(2.394 eV)
1134 1177 -- 1.502 au H 1.00 3 S 53
(40.877 eV)
1135 1178 -- 0.406 au H 1.00 1 P:x 53
(11.052 eV)
1136 1179 -- 0.406 au H 1.00 1 P:y 53
(11.052 eV)
1137 1180 -- 0.406 au H 1.00 1 P:z 53
(11.052 eV)
1138 1181 -- 2.570 au H 1.00 1 D:z2 53
(69.925 eV)
1139 1182 -- 2.570 au H 1.00 1 D:x2-y2 53
(69.925 eV)
1140 1183 -- 2.570 au H 1.00 1 D:xy 53

(69.925 eV)

1141 1184 -- 2.570 au H 1.00 1 D:xz 53

(69.925 eV)

1142 1185 -- 2.570 au H 1.00 1 D:yz 53

(69.925 eV)

1143 1186 1.000 -0.240 au H 1.00 1 S 54

(-6.532 eV)

1144 1187 -- 0.088 au H 1.00 2 S 54

(2.394 eV)

1145 1188 -- 1.502 au H 1.00 3 S 54

(40.877 eV)

1146 1189 -- 0.406 au H 1.00 1 P:x 54

(11.052 eV)

1147 1190 -- 0.406 au H 1.00 1 P:y 54

(11.052 eV)

1148 1191 -- 0.406 au H 1.00 1 P:z 54

(11.052 eV)

1149 1192 -- 2.570 au H 1.00 1 D:z2 54

(69.925 eV)

1150 1193 -- 2.570 au H 1.00 1 D:x2-y2 54

(69.925 eV)

1151 1194 -- 2.570 au H 1.00 1 D:xy 54

(69.925 eV)

1152 1195 -- 2.570 au H 1.00 1 D:xz 54

(69.925 eV)

1153 1196 -- 2.570 au H 1.00 1 D:yz 54

(69.925 eV)

1154 1197 1.000 -0.240 au H 1.00 1 S 55

(-6.532 eV)

1155 1198 -- 0.088 au H 1.00 2 S 55

(2.394 eV)

1156 1199 -- 1.502 au H 1.00 3 S 55

(40.877 eV)

1157 1200 -- 0.406 au H 1.00 1 P:x 55

(11.052 eV)

1158 1201 -- 0.406 au H 1.00 1 P:y 55

(11.052 eV)

1159 1202 -- 0.406 au H 1.00 1 P:z 55

(11.052 eV)

1160 1203 -- 2.570 au H 1.00 1 D:z2 55

(69.925 eV)

1161 1204 -- 2.570 au H 1.00 1 D:x2-y2 55

(69.925 eV)

1162 1205 -- 2.570 au H 1.00 1 D:xy 55

(69.925 eV)

1163 1206 -- 2.570 au H 1.00 1 D:xz 55

(69.925 eV)

1164 1207 -- 2.570 au H 1.00 1 D:yz 55

(69.925 eV)

1165 1208 1.000 -0.240 au H 1.00 1 S 56

(-6.532 eV)

1166 1209 -- 0.088 au H 1.00 2 S 56

(2.394 eV)

1167 1210 -- 1.502 au H 1.00 3 S 56

(40.877 eV)

1168 1211 -- 0.406 au H 1.00 1 P:x 56

(11.052 eV)

1169 1212 -- 0.406 au H 1.00 1 P:y 56

(11.052 eV)

1170 1213 -- 0.406 au H 1.00 1 P:z 56

(11.052 eV)

1171 1214 -- 2.570 au H 1.00 1 D:z2 56

(69.925 eV)

1172 1215 -- 2.570 au H 1.00 1 D:x2-y2 56

(69.925 eV)

1173 1216 -- 2.570 au H 1.00 1 D:xy 56

(69.925 eV)

1174 1217 -- 2.570 au H 1.00 1 D:xz 56

(69.925 eV)

1175 1218 -- 2.570 au H 1.00 1 D:yz 56

(69.925 eV)

1176 1219 1.000 -0.240 au H 1.00 1 S 57

(-6.532 eV)

1177 1220 -- 0.088 au H 1.00 2 S 57

(2.394 eV)

1178 1221 -- 1.502 au H 1.00 3 S 57

(40.877 eV)

1179 1222 -- 0.406 au H 1.00 1 P:x 57

(11.052 eV)

1180 1223 -- 0.406 au H 1.00 1 P:y 57

(11.052 eV)

1181 1224 -- 0.406 au H 1.00 1 P:z 57

(11.052 eV)

1182 1225 -- 2.570 au H 1.00 1 D:z2 57

(69.925 eV)

1183 1226 -- 2.570 au H 1.00 1 D:x2-y2 57

(69.925 eV)

1184 1227 -- 2.570 au H 1.00 1 D:xy 57

(69.925 eV)

1185 1228 -- 2.570 au H 1.00 1 D:xz 57

(69.925 eV)

1186 1229 -- 2.570 au H 1.00 1 D:yz 57

(69.925 eV)

1187 1230 1.000 -0.240 au H 1.00 1 S 58

(-6.532 eV)

1188 1231 -- 0.088 au H 1.00 2 S 58

(2.394 eV)

1189 1232 -- 1.502 au H 1.00 3 S 58

(40.877 eV)

1190 1233 -- 0.406 au H 1.00 1 P:x 58

(11.052 eV)

1191 1234 -- 0.406 au H 1.00 1 P:y 58

(11.052 eV)

1192 1235 -- 0.406 au H 1.00 1 P:z 58

(11.052 eV)

1193 1236 -- 2.570 au H 1.00 1 D:z2 58

(69.925 eV)

1194 1237 -- 2.570 au H 1.00 1 D:x2-y2 58

(69.925 eV)

1195 1238 -- 2.570 au H 1.00 1 D:xy 58

(69.925 eV)

1196 1239 -- 2.570 au H 1.00 1 D:xz 58

(69.925 eV)

1197 1240 -- 2.570 au H 1.00 1 D:yz 58

(69.925 eV)

1198 1241 1.000 -0.240 au H 1.00 1 S 59

(-6.532 eV)

1199 1242 -- 0.088 au H 1.00 2 S 59

(2.394 eV)

1200 1243 -- 1.502 au H 1.00 3 S 59

(40.877 eV)

1201 1244 -- 0.406 au H 1.00 1 P:x 59

(11.052 eV)

1202 1245 -- 0.406 au H 1.00 1 P:y 59

(11.052 eV)

1203 1246 -- 0.406 au H 1.00 1 P:z 59

(11.052 eV)

1204 1247 -- 2.570 au H 1.00 1 D:z2 59

(69.925 eV)

1205 1248 -- 2.570 au H 1.00 1 D:x2-y2 59

(69.925 eV)

1206 1249 -- 2.570 au H 1.00 1 D:xy 59

(69.925 eV)

1207 1250 -- 2.570 au H 1.00 1 D:xz 59

(69.925 eV)

1208 1251 -- 2.570 au H 1.00 1 D:yz 59

(69.925 eV)

1209 1252 1.000 -0.240 au H 1.00 1 S 60

(-6.532 eV)

1210 1253 -- 0.088 au H 1.00 2 S 60

(2.394 eV)

1211 1254 -- 1.502 au H 1.00 3 S 60

(40.877 eV)

1212 1255 -- 0.406 au H 1.00 1 P:x 60

(11.052 eV)

1213 1256 -- 0.406 au H 1.00 1 P:y 60

(11.052 eV)

1214 1257 -- 0.406 au H 1.00 1 P:z 60

(11.052 eV)

1215 1258 -- 2.570 au H 1.00 1 D:z2 60

(69.925 eV)

1216 1259 -- 2.570 au H 1.00 1 D:x2-y2 60

(69.925 eV)

1217 1260 -- 2.570 au H 1.00 1 D:xy 60

(69.925 eV)

1218 1261 -- 2.570 au H 1.00 1 D:xz 60
(69.925 eV)
1219 1262 -- 2.570 au H 1.00 1 D:yz 60
(69.925 eV)
1220 1263 1.000 -0.240 au H 1.00 1 S 61
(-6.532 eV)
1221 1264 -- 0.088 au H 1.00 2 S 61
(2.394 eV)
1222 1265 -- 1.502 au H 1.00 3 S 61
(40.877 eV)
1223 1266 -- 0.406 au H 1.00 1 P:x 61
(11.052 eV)
1224 1267 -- 0.406 au H 1.00 1 P:y 61
(11.052 eV)
1225 1268 -- 0.406 au H 1.00 1 P:z 61
(11.052 eV)
1226 1269 -- 2.570 au H 1.00 1 D:z2 61
(69.925 eV)
1227 1270 -- 2.570 au H 1.00 1 D:x2-y2 61
(69.925 eV)
1228 1271 -- 2.570 au H 1.00 1 D:xy 61
(69.925 eV)
1229 1272 -- 2.570 au H 1.00 1 D:xz 61
(69.925 eV)
1230 1273 -- 2.570 au H 1.00 1 D:yz 61
(69.925 eV)
1231 1274 1.000 -0.240 au H 1.00 1 S 62
(-6.532 eV)
1232 1275 -- 0.088 au H 1.00 2 S 62
(2.394 eV)
1233 1276 -- 1.502 au H 1.00 3 S 62

(40.877 eV)

1234 1277 -- 0.406 au H 1.00 1 P:x 62

(11.052 eV)

1235 1278 -- 0.406 au H 1.00 1 P:y 62

(11.052 eV)

1236 1279 -- 0.406 au H 1.00 1 P:z 62

(11.052 eV)

1237 1280 -- 2.570 au H 1.00 1 D:z2 62

(69.925 eV)

1238 1281 -- 2.570 au H 1.00 1 D:x2-y2 62

(69.925 eV)

1239 1282 -- 2.570 au H 1.00 1 D:xy 62

(69.925 eV)

1240 1283 -- 2.570 au H 1.00 1 D:xz 62

(69.925 eV)

1241 1284 -- 2.570 au H 1.00 1 D:yz 62

(69.925 eV)

1242 1285 1.000 -0.240 au H 1.00 1 S 63

(-6.532 eV)

1243 1286 -- 0.088 au H 1.00 2 S 63

(2.394 eV)

1244 1287 -- 1.502 au H 1.00 3 S 63

(40.877 eV)

1245 1288 -- 0.406 au H 1.00 1 P:x 63

(11.052 eV)

1246 1289 -- 0.406 au H 1.00 1 P:y 63

(11.052 eV)

1247 1290 -- 0.406 au H 1.00 1 P:z 63

(11.052 eV)

1248 1291 -- 2.570 au H 1.00 1 D:z2 63

(69.925 eV)

1249 1292 -- 2.570 au H 1.00 1 D:x2-y2 63

(69.925 eV)

1250 1293 -- 2.570 au H 1.00 1 D:xy 63

(69.925 eV)

1251 1294 -- 2.570 au H 1.00 1 D:xz 63

(69.925 eV)

1252 1295 -- 2.570 au H 1.00 1 D:yz 63

(69.925 eV)

1253 1296 1.000 -0.240 au H 1.00 1 S 64

(-6.532 eV)

1254 1297 -- 0.088 au H 1.00 2 S 64

(2.394 eV)

1255 1298 -- 1.502 au H 1.00 3 S 64

(40.877 eV)

1256 1299 -- 0.406 au H 1.00 1 P:x 64

(11.052 eV)

1257 1300 -- 0.406 au H 1.00 1 P:y 64

(11.052 eV)

1258 1301 -- 0.406 au H 1.00 1 P:z 64

(11.052 eV)

1259 1302 -- 2.570 au H 1.00 1 D:z2 64

(69.925 eV)

1260 1303 -- 2.570 au H 1.00 1 D:x2-y2 64

(69.925 eV)

1261 1304 -- 2.570 au H 1.00 1 D:xy 64

(69.925 eV)

1262 1305 -- 2.570 au H 1.00 1 D:xz 64

(69.925 eV)

1263 1306 -- 2.570 au H 1.00 1 D:yz 64

(69.925 eV)

1264 1307 1.000 -0.240 au H 1.00 1 S 65

		(	-6.532 eV)				
1265	1308	--	0.088 au	H	1.00	2 S	65
		(	2.394 eV)				
1266	1309	--	1.502 au	H	1.00	3 S	65
		(	40.877 eV)				
1267	1310	--	0.406 au	H	1.00	1 P:x	65
		(	11.052 eV)				
1268	1311	--	0.406 au	H	1.00	1 P:y	65
		(	11.052 eV)				
1269	1312	--	0.406 au	H	1.00	1 P:z	65
		(	11.052 eV)				
1270	1313	--	2.570 au	H	1.00	1 D:z2	65
		(	69.925 eV)				
1271	1314	--	2.570 au	H	1.00	1 D:x2-y2	65
		(	69.925 eV)				
1272	1315	--	2.570 au	H	1.00	1 D:xy	65
		(	69.925 eV)				
1273	1316	--	2.570 au	H	1.00	1 D:xz	65
		(	69.925 eV)				
1274	1317	--	2.570 au	H	1.00	1 D:yz	65
		(	69.925 eV)				
1275	1318	1.000	-0.240 au	H	1.00	1 S	66
		(	-6.532 eV)				
1276	1319	--	0.088 au	H	1.00	2 S	66
		(	2.394 eV)				
1277	1320	--	1.502 au	H	1.00	3 S	66
		(	40.877 eV)				
1278	1321	--	0.406 au	H	1.00	1 P:x	66
		(	11.052 eV)				
1279	1322	--	0.406 au	H	1.00	1 P:y	66
		(	11.052 eV)				

1280 1323 -- 0.406 au H 1.00 1 P:z 66

(11.052 eV)

1281 1324 -- 2.570 au H 1.00 1 D:z2 66

(69.925 eV)

1282 1325 -- 2.570 au H 1.00 1 D:x2-y2 66

(69.925 eV)

1283 1326 -- 2.570 au H 1.00 1 D:xy 66

(69.925 eV)

1284 1327 -- 2.570 au H 1.00 1 D:xz 66

(69.925 eV)

1285 1328 -- 2.570 au H 1.00 1 D:yz 66

(69.925 eV)

1286 1329 1.000 -0.240 au H 1.00 1 S 67

(-6.532 eV)

1287 1330 -- 0.088 au H 1.00 2 S 67

(2.394 eV)

1288 1331 -- 1.502 au H 1.00 3 S 67

(40.877 eV)

1289 1332 -- 0.406 au H 1.00 1 P:x 67

(11.052 eV)

1290 1333 -- 0.406 au H 1.00 1 P:y 67

(11.052 eV)

1291 1334 -- 0.406 au H 1.00 1 P:z 67

(11.052 eV)

1292 1335 -- 2.570 au H 1.00 1 D:z2 67

(69.925 eV)

1293 1336 -- 2.570 au H 1.00 1 D:x2-y2 67

(69.925 eV)

1294 1337 -- 2.570 au H 1.00 1 D:xy 67

(69.925 eV)

1295 1338 -- 2.570 au H 1.00 1 D:xz 67

(69.925 eV)

1296 1339 -- 2.570 au H 1.00 1 D:yz 67

(69.925 eV)

1297 1340 1.000 -0.240 au H 1.00 1 S 68

(-6.532 eV)

1298 1341 -- 0.088 au H 1.00 2 S 68

(2.394 eV)

1299 1342 -- 1.502 au H 1.00 3 S 68

(40.877 eV)

1300 1343 -- 0.406 au H 1.00 1 P:x 68

(11.052 eV)

1301 1344 -- 0.406 au H 1.00 1 P:y 68

(11.052 eV)

1302 1345 -- 0.406 au H 1.00 1 P:z 68

(11.052 eV)

1303 1346 -- 2.570 au H 1.00 1 D:z2 68

(69.925 eV)

1304 1347 -- 2.570 au H 1.00 1 D:x2-y2 68

(69.925 eV)

1305 1348 -- 2.570 au H 1.00 1 D:xy 68

(69.925 eV)

1306 1349 -- 2.570 au H 1.00 1 D:xz 68

(69.925 eV)

1307 1350 -- 2.570 au H 1.00 1 D:yz 68

(69.925 eV)

1308 1351 1.000 -0.240 au H 1.00 1 S 69

(-6.532 eV)

1309 1352 -- 0.088 au H 1.00 2 S 69

(2.394 eV)

1310 1353 -- 1.502 au H 1.00 3 S 69

(40.877 eV)

1311 1354 -- 0.406 au H 1.00 1 P:x 69

(11.052 eV)

1312 1355 -- 0.406 au H 1.00 1 P:y 69

(11.052 eV)

1313 1356 -- 0.406 au H 1.00 1 P:z 69

(11.052 eV)

1314 1357 -- 2.570 au H 1.00 1 D:z2 69

(69.925 eV)

1315 1358 -- 2.570 au H 1.00 1 D:x2-y2 69

(69.925 eV)

1316 1359 -- 2.570 au H 1.00 1 D:xy 69

(69.925 eV)

1317 1360 -- 2.570 au H 1.00 1 D:xz 69

(69.925 eV)

1318 1361 -- 2.570 au H 1.00 1 D:yz 69

(69.925 eV)

1319 1362 1.000 -0.240 au H 1.00 1 S 70

(-6.532 eV)

1320 1363 -- 0.088 au H 1.00 2 S 70

(2.394 eV)

1321 1364 -- 1.502 au H 1.00 3 S 70

(40.877 eV)

1322 1365 -- 0.406 au H 1.00 1 P:x 70

(11.052 eV)

1323 1366 -- 0.406 au H 1.00 1 P:y 70

(11.052 eV)

1324 1367 -- 0.406 au H 1.00 1 P:z 70

(11.052 eV)

1325 1368 -- 2.570 au H 1.00 1 D:z2 70

(69.925 eV)

1326 1369 -- 2.570 au H 1.00 1 D:x2-y2 70

(69.925 eV)

1327 1370 -- 2.570 au H 1.00 1 D:xy 70

(69.925 eV)

1328 1371 -- 2.570 au H 1.00 1 D:xz 70

(69.925 eV)

1329 1372 -- 2.570 au H 1.00 1 D:yz 70

(69.925 eV)

1330 1373 1.000 -0.240 au H 1.00 1 S 71

(-6.532 eV)

1331 1374 -- 0.088 au H 1.00 2 S 71

(2.394 eV)

1332 1375 -- 1.502 au H 1.00 3 S 71

(40.877 eV)

1333 1376 -- 0.406 au H 1.00 1 P:x 71

(11.052 eV)

1334 1377 -- 0.406 au H 1.00 1 P:y 71

(11.052 eV)

1335 1378 -- 0.406 au H 1.00 1 P:z 71

(11.052 eV)

1336 1379 -- 2.570 au H 1.00 1 D:z2 71

(69.925 eV)

1337 1380 -- 2.570 au H 1.00 1 D:x2-y2 71

(69.925 eV)

1338 1381 -- 2.570 au H 1.00 1 D:xy 71

(69.925 eV)

1339 1382 -- 2.570 au H 1.00 1 D:xz 71

(69.925 eV)

1340 1383 -- 2.570 au H 1.00 1 D:yz 71

(69.925 eV)

1341 1384 1.000 -0.240 au H 1.00 1 S 72

(-6.532 eV)

1342 1385 -- 0.088 au H 1.00 2 S 72
(2.394 eV)
1343 1386 -- 1.502 au H 1.00 3 S 72
(40.877 eV)
1344 1387 -- 0.406 au H 1.00 1 P:x 72
(11.052 eV)
1345 1388 -- 0.406 au H 1.00 1 P:y 72
(11.052 eV)
1346 1389 -- 0.406 au H 1.00 1 P:z 72
(11.052 eV)
1347 1390 -- 2.570 au H 1.00 1 D:z2 72
(69.925 eV)
1348 1391 -- 2.570 au H 1.00 1 D:x2-y2 72
(69.925 eV)
1349 1392 -- 2.570 au H 1.00 1 D:xy 72
(69.925 eV)
1350 1393 -- 2.570 au H 1.00 1 D:xz 72
(69.925 eV)
1351 1394 -- 2.570 au H 1.00 1 D:yz 72
(69.925 eV)
1352 1395 1.000 -0.240 au H 1.00 1 S 73
(-6.532 eV)
1353 1396 -- 0.088 au H 1.00 2 S 73
(2.394 eV)
1354 1397 -- 1.502 au H 1.00 3 S 73
(40.877 eV)
1355 1398 -- 0.406 au H 1.00 1 P:x 73
(11.052 eV)
1356 1399 -- 0.406 au H 1.00 1 P:y 73
(11.052 eV)
1357 1400 -- 0.406 au H 1.00 1 P:z 73

(11.052 eV)

1358 1401 -- 2.570 au H 1.00 1 D:z2 73

(69.925 eV)

1359 1402 -- 2.570 au H 1.00 1 D:x2-y2 73

(69.925 eV)

1360 1403 -- 2.570 au H 1.00 1 D:xy 73

(69.925 eV)

1361 1404 -- 2.570 au H 1.00 1 D:xz 73

(69.925 eV)

1362 1405 -- 2.570 au H 1.00 1 D:yz 73

(69.925 eV)

1363 1406 1.000 -0.240 au H 1.00 1 S 74

(-6.532 eV)

1364 1407 -- 0.088 au H 1.00 2 S 74

(2.394 eV)

1365 1408 -- 1.502 au H 1.00 3 S 74

(40.877 eV)

1366 1409 -- 0.406 au H 1.00 1 P:x 74

(11.052 eV)

1367 1410 -- 0.406 au H 1.00 1 P:y 74

(11.052 eV)

1368 1411 -- 0.406 au H 1.00 1 P:z 74

(11.052 eV)

1369 1412 -- 2.570 au H 1.00 1 D:z2 74

(69.925 eV)

1370 1413 -- 2.570 au H 1.00 1 D:x2-y2 74

(69.925 eV)

1371 1414 -- 2.570 au H 1.00 1 D:xy 74

(69.925 eV)

1372 1415 -- 2.570 au H 1.00 1 D:xz 74

(69.925 eV)

1373 1416 -- 2.570 au H 1.00 1 D:yz 74

(69.925 eV)

1374 1417 1.000 -0.240 au H 1.00 1 S 75

(-6.532 eV)

1375 1418 -- 0.088 au H 1.00 2 S 75

(2.394 eV)

1376 1419 -- 1.502 au H 1.00 3 S 75

(40.877 eV)

1377 1420 -- 0.406 au H 1.00 1 P:x 75

(11.052 eV)

1378 1421 -- 0.406 au H 1.00 1 P:y 75

(11.052 eV)

1379 1422 -- 0.406 au H 1.00 1 P:z 75

(11.052 eV)

1380 1423 -- 2.570 au H 1.00 1 D:z2 75

(69.925 eV)

1381 1424 -- 2.570 au H 1.00 1 D:x2-y2 75

(69.925 eV)

1382 1425 -- 2.570 au H 1.00 1 D:xy 75

(69.925 eV)

1383 1426 -- 2.570 au H 1.00 1 D:xz 75

(69.925 eV)

1384 1427 -- 2.570 au H 1.00 1 D:yz 75

(69.925 eV)

1385 1428 1.000 -0.240 au H 1.00 1 S 76

(-6.532 eV)

1386 1429 -- 0.088 au H 1.00 2 S 76

(2.394 eV)

1387 1430 -- 1.502 au H 1.00 3 S 76

(40.877 eV)

1388 1431 -- 0.406 au H 1.00 1 P:x 76

(11.052 eV)

1389 1432 -- 0.406 au H 1.00 1 P:y 76

(11.052 eV)

1390 1433 -- 0.406 au H 1.00 1 P:z 76

(11.052 eV)

1391 1434 -- 2.570 au H 1.00 1 D:z2 76

(69.925 eV)

1392 1435 -- 2.570 au H 1.00 1 D:x2-y2 76

(69.925 eV)

1393 1436 -- 2.570 au H 1.00 1 D:xy 76

(69.925 eV)

1394 1437 -- 2.570 au H 1.00 1 D:xz 76

(69.925 eV)

1395 1438 -- 2.570 au H 1.00 1 D:yz 76

(69.925 eV)

1396 1439 1.000 -0.240 au H 1.00 1 S 77

(-6.532 eV)

1397 1440 -- 0.088 au H 1.00 2 S 77

(2.394 eV)

1398 1441 -- 1.502 au H 1.00 3 S 77

(40.877 eV)

1399 1442 -- 0.406 au H 1.00 1 P:x 77

(11.052 eV)

1400 1443 -- 0.406 au H 1.00 1 P:y 77

(11.052 eV)

1401 1444 -- 0.406 au H 1.00 1 P:z 77

(11.052 eV)

1402 1445 -- 2.570 au H 1.00 1 D:z2 77

(69.925 eV)

1403 1446 -- 2.570 au H 1.00 1 D:x2-y2 77

(69.925 eV)

1404 1447 -- 2.570 au H 1.00 1 D:xy 77

(69.925 eV)

1405 1448 -- 2.570 au H 1.00 1 D:xz 77

(69.925 eV)

1406 1449 -- 2.570 au H 1.00 1 D:yz 77

(69.925 eV)

1407 1450 1.000 -0.240 au H 1.00 1 S 78

(-6.532 eV)

1408 1451 -- 0.088 au H 1.00 2 S 78

(2.394 eV)

1409 1452 -- 1.502 au H 1.00 3 S 78

(40.877 eV)

1410 1453 -- 0.406 au H 1.00 1 P:x 78

(11.052 eV)

1411 1454 -- 0.406 au H 1.00 1 P:y 78

(11.052 eV)

1412 1455 -- 0.406 au H 1.00 1 P:z 78

(11.052 eV)

1413 1456 -- 2.570 au H 1.00 1 D:z2 78

(69.925 eV)

1414 1457 -- 2.570 au H 1.00 1 D:x2-y2 78

(69.925 eV)

1415 1458 -- 2.570 au H 1.00 1 D:xy 78

(69.925 eV)

1416 1459 -- 2.570 au H 1.00 1 D:xz 78

(69.925 eV)

1417 1460 -- 2.570 au H 1.00 1 D:yz 78

(69.925 eV)

1418 1461 1.000 -0.240 au H 1.00 1 S 79

(-6.532 eV)

1419 1462 -- 0.088 au H 1.00 2 S 79

(2.394 eV)
1420 1463 -- 1.502 au H 1.00 3 S 79
(40.877 eV)
1421 1464 -- 0.406 au H 1.00 1 P:x 79
(11.052 eV)
1422 1465 -- 0.406 au H 1.00 1 P:y 79
(11.052 eV)
1423 1466 -- 0.406 au H 1.00 1 P:z 79
(11.052 eV)
1424 1467 -- 2.570 au H 1.00 1 D:z2 79
(69.925 eV)
1425 1468 -- 2.570 au H 1.00 1 D:x2-y2 79
(69.925 eV)
1426 1469 -- 2.570 au H 1.00 1 D:xy 79
(69.925 eV)
1427 1470 -- 2.570 au H 1.00 1 D:xz 79
(69.925 eV)
1428 1471 -- 2.570 au H 1.00 1 D:yz 79
(69.925 eV)
1429 1472 1.000 -0.240 au H 1.00 1 S 80
(-6.532 eV)
1430 1473 -- 0.088 au H 1.00 2 S 80
(2.394 eV)
1431 1474 -- 1.502 au H 1.00 3 S 80
(40.877 eV)
1432 1475 -- 0.406 au H 1.00 1 P:x 80
(11.052 eV)
1433 1476 -- 0.406 au H 1.00 1 P:y 80
(11.052 eV)
1434 1477 -- 0.406 au H 1.00 1 P:z 80
(11.052 eV)

1435 1478 -- 2.570 au H 1.00 1 D:z2 80

(69.925 eV)

1436 1479 -- 2.570 au H 1.00 1 D:x2-y2 80

(69.925 eV)

1437 1480 -- 2.570 au H 1.00 1 D:xy 80

(69.925 eV)

1438 1481 -- 2.570 au H 1.00 1 D:xz 80

(69.925 eV)

1439 1482 -- 2.570 au H 1.00 1 D:yz 80

(69.925 eV)

1440 1483 1.000 -0.240 au H 1.00 1 S 81

(-6.532 eV)

1441 1484 -- 0.088 au H 1.00 2 S 81

(2.394 eV)

1442 1485 -- 1.502 au H 1.00 3 S 81

(40.877 eV)

1443 1486 -- 0.406 au H 1.00 1 P:x 81

(11.052 eV)

1444 1487 -- 0.406 au H 1.00 1 P:y 81

(11.052 eV)

1445 1488 -- 0.406 au H 1.00 1 P:z 81

(11.052 eV)

1446 1489 -- 2.570 au H 1.00 1 D:z2 81

(69.925 eV)

1447 1490 -- 2.570 au H 1.00 1 D:x2-y2 81

(69.925 eV)

1448 1491 -- 2.570 au H 1.00 1 D:xy 81

(69.925 eV)

1449 1492 -- 2.570 au H 1.00 1 D:xz 81

(69.925 eV)

1450 1493 -- 2.570 au H 1.00 1 D:yz 81

(69.925 eV)

1451 1494 1.000 -0.240 au H 1.00 1 S 82

(-6.532 eV)

1452 1495 -- 0.088 au H 1.00 2 S 82

(2.394 eV)

1453 1496 -- 1.502 au H 1.00 3 S 82

(40.877 eV)

1454 1497 -- 0.406 au H 1.00 1 P:x 82

(11.052 eV)

1455 1498 -- 0.406 au H 1.00 1 P:y 82

(11.052 eV)

1456 1499 -- 0.406 au H 1.00 1 P:z 82

(11.052 eV)

1457 1500 -- 2.570 au H 1.00 1 D:z2 82

(69.925 eV)

1458 1501 -- 2.570 au H 1.00 1 D:x2-y2 82

(69.925 eV)

1459 1502 -- 2.570 au H 1.00 1 D:xy 82

(69.925 eV)

1460 1503 -- 2.570 au H 1.00 1 D:xz 82

(69.925 eV)

1461 1504 -- 2.570 au H 1.00 1 D:yz 82

(69.925 eV)

1462 1505 1.000 -0.240 au H 1.00 1 S 83

(-6.532 eV)

1463 1506 -- 0.088 au H 1.00 2 S 83

(2.394 eV)

1464 1507 -- 1.502 au H 1.00 3 S 83

(40.877 eV)

1465 1508 -- 0.406 au H 1.00 1 P:x 83

(11.052 eV)

1466 1509 -- 0.406 au H 1.00 1 P:y 83

(11.052 eV)

1467 1510 -- 0.406 au H 1.00 1 P:z 83

(11.052 eV)

1468 1511 -- 2.570 au H 1.00 1 D:z2 83

(69.925 eV)

1469 1512 -- 2.570 au H 1.00 1 D:x2-y2 83

(69.925 eV)

1470 1513 -- 2.570 au H 1.00 1 D:xy 83

(69.925 eV)

1471 1514 -- 2.570 au H 1.00 1 D:xz 83

(69.925 eV)

1472 1515 -- 2.570 au H 1.00 1 D:yz 83

(69.925 eV)

1473 1516 1.000 -0.240 au H 1.00 1 S 84

(-6.532 eV)

1474 1517 -- 0.088 au H 1.00 2 S 84

(2.394 eV)

1475 1518 -- 1.502 au H 1.00 3 S 84

(40.877 eV)

1476 1519 -- 0.406 au H 1.00 1 P:x 84

(11.052 eV)

1477 1520 -- 0.406 au H 1.00 1 P:y 84

(11.052 eV)

1478 1521 -- 0.406 au H 1.00 1 P:z 84

(11.052 eV)

1479 1522 -- 2.570 au H 1.00 1 D:z2 84

(69.925 eV)

1480 1523 -- 2.570 au H 1.00 1 D:x2-y2 84

(69.925 eV)

1481 1524 -- 2.570 au H 1.00 1 D:xy 84

(69.925 eV)

1482 1525 -- 2.570 au H 1.00 1 D:xz 84

(69.925 eV)

1483 1526 -- 2.570 au H 1.00 1 D:yz 84

(69.925 eV)

1484 1527 1.000 -0.240 au H 1.00 1 S 85

(-6.532 eV)

1485 1528 -- 0.088 au H 1.00 2 S 85

(2.394 eV)

1486 1529 -- 1.502 au H 1.00 3 S 85

(40.877 eV)

1487 1530 -- 0.406 au H 1.00 1 P:x 85

(11.052 eV)

1488 1531 -- 0.406 au H 1.00 1 P:y 85

(11.052 eV)

1489 1532 -- 0.406 au H 1.00 1 P:z 85

(11.052 eV)

1490 1533 -- 2.570 au H 1.00 1 D:z2 85

(69.925 eV)

1491 1534 -- 2.570 au H 1.00 1 D:x2-y2 85

(69.925 eV)

1492 1535 -- 2.570 au H 1.00 1 D:xy 85

(69.925 eV)

1493 1536 -- 2.570 au H 1.00 1 D:xz 85

(69.925 eV)

1494 1537 -- 2.570 au H 1.00 1 D:yz 85

(69.925 eV)

1495 1538 1.000 -0.240 au H 1.00 1 S 86

(-6.532 eV)

1496 1539 -- 0.088 au H 1.00 2 S 86

(2.394 eV)

1497 1540 -- 1.502 au H 1.00 3 S 86

(40.877 eV)

1498 1541 -- 0.406 au H 1.00 1 P:x 86

(11.052 eV)

1499 1542 -- 0.406 au H 1.00 1 P:y 86

(11.052 eV)

1500 1543 -- 0.406 au H 1.00 1 P:z 86

(11.052 eV)

1501 1544 -- 2.570 au H 1.00 1 D:z2 86

(69.925 eV)

1502 1545 -- 2.570 au H 1.00 1 D:x2-y2 86

(69.925 eV)

1503 1546 -- 2.570 au H 1.00 1 D:xy 86

(69.925 eV)

1504 1547 -- 2.570 au H 1.00 1 D:xz 86

(69.925 eV)

1505 1548 -- 2.570 au H 1.00 1 D:yz 86

(69.925 eV)

1506 1549 1.000 -0.240 au H 1.00 1 S 87

(-6.532 eV)

1507 1550 -- 0.088 au H 1.00 2 S 87

(2.394 eV)

1508 1551 -- 1.502 au H 1.00 3 S 87

(40.877 eV)

1509 1552 -- 0.406 au H 1.00 1 P:x 87

(11.052 eV)

1510 1553 -- 0.406 au H 1.00 1 P:y 87

(11.052 eV)

1511 1554 -- 0.406 au H 1.00 1 P:z 87

(11.052 eV)

1512 1555 -- 2.570 au H 1.00 1 D:z2 87

(69.925 eV)

1513 1556 -- 2.570 au H 1.00 1 D:x2-y2 87

(69.925 eV)

1514 1557 -- 2.570 au H 1.00 1 D:xy 87

(69.925 eV)

1515 1558 -- 2.570 au H 1.00 1 D:xz 87

(69.925 eV)

1516 1559 -- 2.570 au H 1.00 1 D:yz 87

(69.925 eV)

1517 1560 1.000 -0.240 au H 1.00 1 S 88

(-6.532 eV)

1518 1561 -- 0.088 au H 1.00 2 S 88

(2.394 eV)

1519 1562 -- 1.502 au H 1.00 3 S 88

(40.877 eV)

1520 1563 -- 0.406 au H 1.00 1 P:x 88

(11.052 eV)

1521 1564 -- 0.406 au H 1.00 1 P:y 88

(11.052 eV)

1522 1565 -- 0.406 au H 1.00 1 P:z 88

(11.052 eV)

1523 1566 -- 2.570 au H 1.00 1 D:z2 88

(69.925 eV)

1524 1567 -- 2.570 au H 1.00 1 D:x2-y2 88

(69.925 eV)

1525 1568 -- 2.570 au H 1.00 1 D:xy 88

(69.925 eV)

1526 1569 -- 2.570 au H 1.00 1 D:xz 88

(69.925 eV)

1527 1570 -- 2.570 au H 1.00 1 D:yz 88

(69.925 eV)

1528 1571 1.000 -0.240 au H 1.00 1 S 89

(-6.532 eV)

1529 1572 -- 0.088 au H 1.00 2 S 89

(2.394 eV)

1530 1573 -- 1.502 au H 1.00 3 S 89

(40.877 eV)

1531 1574 -- 0.406 au H 1.00 1 P:x 89

(11.052 eV)

1532 1575 -- 0.406 au H 1.00 1 P:y 89

(11.052 eV)

1533 1576 -- 0.406 au H 1.00 1 P:z 89

(11.052 eV)

1534 1577 -- 2.570 au H 1.00 1 D:z2 89

(69.925 eV)

1535 1578 -- 2.570 au H 1.00 1 D:x2-y2 89

(69.925 eV)

1536 1579 -- 2.570 au H 1.00 1 D:xy 89

(69.925 eV)

1537 1580 -- 2.570 au H 1.00 1 D:xz 89

(69.925 eV)

1538 1581 -- 2.570 au H 1.00 1 D:yz 89

(69.925 eV)

1539 1582 1.000 -0.240 au H 1.00 1 S 90

(-6.532 eV)

1540 1583 -- 0.088 au H 1.00 2 S 90

(2.394 eV)

1541 1584 -- 1.502 au H 1.00 3 S 90

(40.877 eV)

1542 1585 -- 0.406 au H 1.00 1 P:x 90

(11.052 eV)

1543 1586 -- 0.406 au H 1.00 1 P:y 90

(11.052 eV)
1544 1587 -- 0.406 au H 1.00 1 P:z 90
(11.052 eV)
1545 1588 -- 2.570 au H 1.00 1 D:z2 90
(69.925 eV)
1546 1589 -- 2.570 au H 1.00 1 D:x2-y2 90
(69.925 eV)
1547 1590 -- 2.570 au H 1.00 1 D:xy 90
(69.925 eV)
1548 1591 -- 2.570 au H 1.00 1 D:xz 90
(69.925 eV)
1549 1592 -- 2.570 au H 1.00 1 D:yz 90
(69.925 eV)
1550 1593 1.000 -0.240 au H 1.00 1 S 91
(-6.532 eV)
1551 1594 -- 0.088 au H 1.00 2 S 91
(2.394 eV)
1552 1595 -- 1.502 au H 1.00 3 S 91
(40.877 eV)
1553 1596 -- 0.406 au H 1.00 1 P:x 91
(11.052 eV)
1554 1597 -- 0.406 au H 1.00 1 P:y 91
(11.052 eV)
1555 1598 -- 0.406 au H 1.00 1 P:z 91
(11.052 eV)
1556 1599 -- 2.570 au H 1.00 1 D:z2 91
(69.925 eV)
1557 1600 -- 2.570 au H 1.00 1 D:x2-y2 91
(69.925 eV)
1558 1601 -- 2.570 au H 1.00 1 D:xy 91
(69.925 eV)

1559 1602 -- 2.570 au H 1.00 1 D:xz 91

(69.925 eV)

1560 1603 -- 2.570 au H 1.00 1 D:yz 91

(69.925 eV)

1561 1604 1.000 -0.240 au H 1.00 1 S 92

(-6.532 eV)

1562 1605 -- 0.088 au H 1.00 2 S 92

(2.394 eV)

1563 1606 -- 1.502 au H 1.00 3 S 92

(40.877 eV)

1564 1607 -- 0.406 au H 1.00 1 P:x 92

(11.052 eV)

1565 1608 -- 0.406 au H 1.00 1 P:y 92

(11.052 eV)

1566 1609 -- 0.406 au H 1.00 1 P:z 92

(11.052 eV)

1567 1610 -- 2.570 au H 1.00 1 D:z2 92

(69.925 eV)

1568 1611 -- 2.570 au H 1.00 1 D:x2-y2 92

(69.925 eV)

1569 1612 -- 2.570 au H 1.00 1 D:xy 92

(69.925 eV)

1570 1613 -- 2.570 au H 1.00 1 D:xz 92

(69.925 eV)

1571 1614 -- 2.570 au H 1.00 1 D:yz 92

(69.925 eV)

1572 1615 1.000 -0.240 au H 1.00 1 S 93

(-6.532 eV)

1573 1616 -- 0.088 au H 1.00 2 S 93

(2.394 eV)

1574 1617 -- 1.502 au H 1.00 3 S 93

		(	40.877 eV)				
1575	1618	--	0.406 au	H	1.00	1 P:x	93
		(	11.052 eV)				
1576	1619	--	0.406 au	H	1.00	1 P:y	93
		(	11.052 eV)				
1577	1620	--	0.406 au	H	1.00	1 P:z	93
		(	11.052 eV)				
1578	1621	--	2.570 au	H	1.00	1 D:z2	93
		(	69.925 eV)				
1579	1622	--	2.570 au	H	1.00	1 D:x2-y2	93
		(	69.925 eV)				
1580	1623	--	2.570 au	H	1.00	1 D:xy	93
		(	69.925 eV)				
1581	1624	--	2.570 au	H	1.00	1 D:xz	93
		(	69.925 eV)				
1582	1625	--	2.570 au	H	1.00	1 D:yz	93
		(	69.925 eV)				
1583	1626	1.000	-0.240 au	H	1.00	1 S	94
		(	-6.532 eV)				
1584	1627	--	0.088 au	H	1.00	2 S	94
		(	2.394 eV)				
1585	1628	--	1.502 au	H	1.00	3 S	94
		(	40.877 eV)				
1586	1629	--	0.406 au	H	1.00	1 P:x	94
		(	11.052 eV)				
1587	1630	--	0.406 au	H	1.00	1 P:y	94
		(	11.052 eV)				
1588	1631	--	0.406 au	H	1.00	1 P:z	94
		(	11.052 eV)				
1589	1632	--	2.570 au	H	1.00	1 D:z2	94
		(	69.925 eV)				

1590 1633 -- 2.570 au H 1.00 1 D:x2-y2 94

(69.925 eV)

1591 1634 -- 2.570 au H 1.00 1 D:xy 94

(69.925 eV)

1592 1635 -- 2.570 au H 1.00 1 D:xz 94

(69.925 eV)

1593 1636 -- 2.570 au H 1.00 1 D:yz 94

(69.925 eV)

1594 1637 1.000 -0.240 au H 1.00 1 S 95

(-6.532 eV)

1595 1638 -- 0.088 au H 1.00 2 S 95

(2.394 eV)

1596 1639 -- 1.502 au H 1.00 3 S 95

(40.877 eV)

1597 1640 -- 0.406 au H 1.00 1 P:x 95

(11.052 eV)

1598 1641 -- 0.406 au H 1.00 1 P:y 95

(11.052 eV)

1599 1642 -- 0.406 au H 1.00 1 P:z 95

(11.052 eV)

1600 1643 -- 2.570 au H 1.00 1 D:z2 95

(69.925 eV)

1601 1644 -- 2.570 au H 1.00 1 D:x2-y2 95

(69.925 eV)

1602 1645 -- 2.570 au H 1.00 1 D:xy 95

(69.925 eV)

1603 1646 -- 2.570 au H 1.00 1 D:xz 95

(69.925 eV)

1604 1647 -- 2.570 au H 1.00 1 D:yz 95

(69.925 eV)

1605 1648 1.000 -0.240 au H 1.00 1 S 96

(-6.532 eV)

1606 1649 -- 0.088 au H 1.00 2 S 96

(2.394 eV)

1607 1650 -- 1.502 au H 1.00 3 S 96

(40.877 eV)

1608 1651 -- 0.406 au H 1.00 1 P:x 96

(11.052 eV)

1609 1652 -- 0.406 au H 1.00 1 P:y 96

(11.052 eV)

1610 1653 -- 0.406 au H 1.00 1 P:z 96

(11.052 eV)

1611 1654 -- 2.570 au H 1.00 1 D:z2 96

(69.925 eV)

1612 1655 -- 2.570 au H 1.00 1 D:x2-y2 96

(69.925 eV)

1613 1656 -- 2.570 au H 1.00 1 D:xy 96

(69.925 eV)

1614 1657 -- 2.570 au H 1.00 1 D:xz 96

(69.925 eV)

1615 1658 -- 2.570 au H 1.00 1 D:yz 96

(69.925 eV)

(Slater-type) F U N C T I O N S *** (Basis and Fit) ***

Atom Type 1 (O)

==============

Valence Basis Sets: 9

----

1 S 7.360000

2 S 1.720000

2 S 2.880000

2 S 7.580000

2 P 1.120000

2 P 2.080000

2 P 4.080000

3 D 2.000000

4 F 3.000000

Frozen Core Shells

S: 1

Atom Type 2 (C)

==============

Valence Basis Sets: 9

----

1 S 5.400000

2 S 1.280000

2 S 2.100000

2 S 4.600000

2 P 0.820000

2 P 1.480000

2 P 2.940000

3 D 2.200000

4 F 3.300000

Frozen Core Shells

S: 1

Atom Type 3 (N)

==============

Valence Basis Sets: 9

----

1 S 6.380000

2 S 1.500000

2 S 2.500000

2 S 5.150000

2 P 1.000000

2 P 1.880000

2 P 3.680000

3 D 2.200000

4 F 3.300000

Frozen Core Shells

S: 1

Atom Type 4 (H)

==============

Valence Basis Sets: 5

----

1 S 0.690000

1 S 0.920000

1 S 1.580000

2 P 1.250000

3 D 2.500000

BAS: List of all Elementary Cartesian Basis Functions

===

The numbering in the list below (to the right of the function characteristics) is referred to in print-outs of MO eigenvectors and Mulliken populations in the BAS representation (as contrasted to the SFO representation).

Notes:

1. The functions are characterized by a polynomial prefactor (powers of x,y,z and r) and an exponential decay factor alpha.
2. Since the basis sets are specific for an atom TYPE, the individual functions occur on all atoms of that type.
3. The word 'Core' in the left margin signals that it is a Core Function (CF) : not a degree of freedom in the valence set, but only used to ensure orthogonalization of the other valence basis functions on the frozen Core Orbitals.

```
(power of) X  Y  Z  R     Alpha  on Atom
           ==========     =====  ==========

O                           1    2    3    4    5    6    7    8    9   10
                        ------------------------------------------------------------------------
  Core    0  0  0  0     7.360      1   30   59   88  117  146  175  204  233  262
          0  0  0  1     1.720      2   31   60   89  118  147  176  205  234  263
          0  0  0  1     2.880      3   32   61   90  119  148  177  206  235  264
          0  0  0  1     7.580      4   33   62   91  120  149  178  207  236  265
```

```
1 0 0 0   1.120    5  34  63  92 121 150 179 208 237 266
0 1 0 0   1.120    6  35  64  93 122 151 180 209 238 267
0 0 1 0   1.120    7  36  65  94 123 152 181 210 239 268
1 0 0 0   2.080    8  37  66  95 124 153 182 211 240 269
0 1 0 0   2.080    9  38  67  96 125 154 183 212 241 270
0 0 1 0   2.080   10  39  68  97 126 155 184 213 242 271
1 0 0 0   4.080   11  40  69  98 127 156 185 214 243 272
0 1 0 0   4.080   12  41  70  99 128 157 186 215 244 273
0 0 1 0   4.080   13  42  71 100 129 158 187 216 245 274
2 0 0 0   2.000   14  43  72 101 130 159 188 217 246 275
1 1 0 0   2.000   15  44  73 102 131 160 189 218 247 276
1 0 1 0   2.000   16  45  74 103 132 161 190 219 248 277
0 2 0 0   2.000   17  46  75 104 133 162 191 220 249 278
0 1 1 0   2.000   18  47  76 105 134 163 192 221 250 279
0 0 2 0   2.000   19  48  77 106 135 164 193 222 251 280
3 0 0 0   3.000   20  49  78 107 136 165 194 223 252 281
2 1 0 0   3.000   21  50  79 108 137 166 195 224 253 282
2 0 1 0   3.000   22  51  80 109 138 167 196 225 254 283
1 2 0 0   3.000   23  52  81 110 139 168 197 226 255 284
1 1 1 0   3.000   24  53  82 111 140 169 198 227 256 285
1 0 2 0   3.000   25  54  83 112 141 170 199 228 257 286
0 3 0 0   3.000   26  55  84 113 142 171 200 229 258 287
0 2 1 0   3.000   27  56  85 114 143 172 201 230 259 288
0 1 2 0   3.000   28  57  86 115 144 173 202 231 260 289
0 0 3 0   3.000   29  58  87 116 145 174 203 232 261 290

C                  11  12  13  14  15  16  17  18  19  20  21  22  23  24  25
                   26  27  28  29  30  31  32  33  34  35  36  37  38  39  40
                   41  42
                  ---------------------------------------------------------------------------
 Core   0 0 0 0   5.400   291 320 349 378 407 436 465 494 523 552 581 610 639 668 697
```

726 755 784 813 842 871 900 929 958 987 1016 1045 1074 1103 1132
1161 1190
0 0 0 1 1.280 292 321 350 379 408 437 466 495 524 553 582 611 640 669 698
727 756 785 814 843 872 901 930 959 988 1017 1046 1075 1104 1133
1162 1191
0 0 0 1 2.100 293 322 351 380 409 438 467 496 525 554 583 612 641 670 699
728 757 786 815 844 873 902 931 960 989 1018 1047 1076 1105 1134
1163 1192
0 0 0 1 4.600 294 323 352 381 410 439 468 497 526 555 584 613 642 671 700
729 758 787 816 845 874 903 932 961 990 1019 1048 1077 1106 1135
1164 1193
1 0 0 0 0.820 295 324 353 382 411 440 469 498 527 556 585 614 643 672 701
730 759 788 817 846 875 904 933 962 991 1020 1049 1078 1107 1136
1165 1194
0 1 0 0 0.820 296 325 354 383 412 441 470 499 528 557 586 615 644 673 702
731 760 789 818 847 876 905 934 963 992 1021 1050 1079 1108 1137
1166 1195
0 0 1 0 0.820 297 326 355 384 413 442 471 500 529 558 587 616 645 674 703
732 761 790 819 848 877 906 935 964 993 1022 1051 1080 1109 1138
1167 1196
1 0 0 0 1.480 298 327 356 385 414 443 472 501 530 559 588 617 646 675 704
733 762 791 820 849 878 907 936 965 994 1023 1052 1081 1110 1139
1168 1197
0 1 0 0 1.480 299 328 357 386 415 444 473 502 531 560 589 618 647 676 705
734 763 792 821 850 879 908 937 966 995 1024 1053 1082 1111 1140
1169 1198
0 0 1 0 1.480 300 329 358 387 416 445 474 503 532 561 590 619 648 677 706
735 764 793 822 851 880 909 938 967 996 1025 1054 1083 1112 1141
1170 1199
1 0 0 0 2.940 301 330 359 388 417 446 475 504 533 562 591 620 649 678 707
736 765 794 823 852 881 910 939 968 997 1026 1055 1084 1113 1142

1171 1200

0 1 0 0 2.940 302 331 360 389 418 447 476 505 534 563 592 621 650 679 708

737 766 795 824 853 882 911 940 969 998 1027 1056 1085 1114 1143

1172 1201

0 0 1 0 2.940 303 332 361 390 419 448 477 506 535 564 593 622 651 680 709

738 767 796 825 854 883 912 941 970 999 1028 1057 1086 1115 1144

1173 1202

2 0 0 0 2.200 304 333 362 391 420 449 478 507 536 565 594 623 652 681 710

739 768 797 826 855 884 913 942 971 1000 1029 1058 1087 1116 1145

1174 1203

1 1 0 0 2.200 305 334 363 392 421 450 479 508 537 566 595 624 653 682 711

740 769 798 827 856 885 914 943 972 1001 1030 1059 1088 1117 1146

1175 1204

1 0 1 0 2.200 306 335 364 393 422 451 480 509 538 567 596 625 654 683 712

741 770 799 828 857 886 915 944 973 1002 1031 1060 1089 1118 1147

1176 1205

0 2 0 0 2.200 307 336 365 394 423 452 481 510 539 568 597 626 655 684 713

742 771 800 829 858 887 916 945 974 1003 1032 1061 1090 1119 1148

1177 1206

0 1 1 0 2.200 308 337 366 395 424 453 482 511 540 569 598 627 656 685 714

743 772 801 830 859 888 917 946 975 1004 1033 1062 1091 1120 1149

1178 1207

0 0 2 0 2.200 309 338 367 396 425 454 483 512 541 570 599 628 657 686 715

744 773 802 831 860 889 918 947 976 1005 1034 1063 1092 1121 1150

1179 1208

3 0 0 0 3.300 310 339 368 397 426 455 484 513 542 571 600 629 658 687 716

745 774 803 832 861 890 919 948 977 1006 1035 1064 1093 1122 1151

1180 1209

2 1 0 0 3.300 311 340 369 398 427 456 485 514 543 572 601 630 659 688 717

746 775 804 833 862 891 920 949 978 1007 1036 1065 1094 1123 1152

1181 1210

2 0 1 0 3.300 312 341 370 399 428 457 486 515 544 573 602 631 660 689 718
747 776 805 834 863 892 921 950 979 1008 1037 1066 1095 1124 1153
1182 1211
1 2 0 0 3.300 313 342 371 400 429 458 487 516 545 574 603 632 661 690 719
748 777 806 835 864 893 922 951 980 1009 1038 1067 1096 1125 1154
1183 1212
1 1 1 0 3.300 314 343 372 401 430 459 488 517 546 575 604 633 662 691 720
749 778 807 836 865 894 923 952 981 1010 1039 1068 1097 1126 1155
1184 1213
1 0 2 0 3.300 315 344 373 402 431 460 489 518 547 576 605 634 663 692 721
750 779 808 837 866 895 924 953 982 1011 1040 1069 1098 1127 1156
1185 1214
0 3 0 0 3.300 316 345 374 403 432 461 490 519 548 577 606 635 664 693 722
751 780 809 838 867 896 925 954 983 1012 1041 1070 1099 1128 1157
1186 1215
0 2 1 0 3.300 317 346 375 404 433 462 491 520 549 578 607 636 665 694 723
752 781 810 839 868 897 926 955 984 1013 1042 1071 1100 1129 1158
1187 1216
0 1 2 0 3.300 318 347 376 405 434 463 492 521 550 579 608 637 666 695 724
753 782 811 840 869 898 927 956 985 1014 1043 1072 1101 1130 1159
1188 1217
0 0 3 0 3.300 319 348 377 406 435 464 493 522 551 580 609 638 667 696 725
754 783 812 841 870 899 928 957 986 1015 1044 1073 1102 1131 1160
1189 1218

N 43

Core 0 0 0 0 6.380 1219
0 0 0 1 1.500 1220
0 0 0 1 2.500 1221
0 0 0 1 5.150 1222

1 0 0 0 1.000 1223

0 1 0 0 1.000 1224

0 0 1 0 1.000 1225

1 0 0 0 1.880 1226

0 1 0 0 1.880 1227

0 0 1 0 1.880 1228

1 0 0 0 3.680 1229

0 1 0 0 3.680 1230

0 0 1 0 3.680 1231

2 0 0 0 2.200 1232

1 1 0 0 2.200 1233

1 0 1 0 2.200 1234

0 2 0 0 2.200 1235

0 1 1 0 2.200 1236

0 0 2 0 2.200 1237

3 0 0 0 3.300 1238

2 1 0 0 3.300 1239

2 0 1 0 3.300 1240

1 2 0 0 3.300 1241

1 1 1 0 3.300 1242

1 0 2 0 3.300 1243

0 3 0 0 3.300 1244

0 2 1 0 3.300 1245

0 1 2 0 3.300 1246

0 0 3 0 3.300 1247

H 44 45 46 47 48 49 50 51 52 53 54 55 56 57 58

59 60 61 62 63 64 65 66 67 68 69 70 71 72 73

74 75 76 77 78 79 80 81 82 83 84 85 86 87 88

89 90 91 92 93 94 95 96

----

0 0 0 0 0.690 1248 1260 1272 1284 1296 1308 1320 1332 1344 1356 1368 1380 1392
1404 1416

1428 1440 1452 1464 1476 1488 1500 1512 1524 1536 1548 1560 1572 1584
1596

1608 1620 1632 1644 1656 1668 1680 1692 1704 1716 1728 1740 1752 1764
1776

1788 1800 1812 1824 1836 1848 1860 1872

0 0 0 0 0.920 1249 1261 1273 1285 1297 1309 1321 1333 1345 1357 1369 1381 1393
1405 1417

1429 1441 1453 1465 1477 1489 1501 1513 1525 1537 1549 1561 1573 1585
1597

1609 1621 1633 1645 1657 1669 1681 1693 1705 1717 1729 1741 1753 1765
1777

1789 1801 1813 1825 1837 1849 1861 1873

0 0 0 0 1.580 1250 1262 1274 1286 1298 1310 1322 1334 1346 1358 1370 1382 1394
1406 1418

1430 1442 1454 1466 1478 1490 1502 1514 1526 1538 1550 1562 1574 1586
1598

1610 1622 1634 1646 1658 1670 1682 1694 1706 1718 1730 1742 1754 1766
1778

1790 1802 1814 1826 1838 1850 1862 1874

1 0 0 0 1.250 1251 1263 1275 1287 1299 1311 1323 1335 1347 1359 1371 1383 1395
1407 1419

1431 1443 1455 1467 1479 1491 1503 1515 1527 1539 1551 1563 1575 1587
1599

1611 1623 1635 1647 1659 1671 1683 1695 1707 1719 1731 1743 1755 1767
1779

1791 1803 1815 1827 1839 1851 1863 1875

0 1 0 0 1.250 1252 1264 1276 1288 1300 1312 1324 1336 1348 1360 1372 1384 1396
1408 1420

1432 1444 1456 1468 1480 1492 1504 1516 1528 1540 1552 1564 1576 1588
1600

1612 1624 1636 1648 1660 1672 1684 1696 1708 1720 1732 1744 1756 1768
1780

1792 1804 1816 1828 1840 1852 1864 1876

0 0 1 0 1.250 1253 1265 1277 1289 1301 1313 1325 1337 1349 1361 1373 1385 1397
1409 1421

1433 1445 1457 1469 1481 1493 1505 1517 1529 1541 1553 1565 1577 1589 1601

1613 1625 1637 1649 1661 1673 1685 1697 1709 1721 1733 1745 1757 1769 1781

1793 1805 1817 1829 1841 1853 1865 1877

2 0 0 0 2.500 1254 1266 1278 1290 1302 1314 1326 1338 1350 1362 1374 1386 1398 1410 1422

1434 1446 1458 1470 1482 1494 1506 1518 1530 1542 1554 1566 1578 1590 1602

1614 1626 1638 1650 1662 1674 1686 1698 1710 1722 1734 1746 1758 1770 1782

1794 1806 1818 1830 1842 1854 1866 1878

1 1 0 0 2.500 1255 1267 1279 1291 1303 1315 1327 1339 1351 1363 1375 1387 1399 1411 1423

1435 1447 1459 1471 1483 1495 1507 1519 1531 1543 1555 1567 1579 1591 1603

1615 1627 1639 1651 1663 1675 1687 1699 1711 1723 1735 1747 1759 1771 1783

1795 1807 1819 1831 1843 1855 1867 1879

1 0 1 0 2.500 1256 1268 1280 1292 1304 1316 1328 1340 1352 1364 1376 1388 1400 1412 1424

1436 1448 1460 1472 1484 1496 1508 1520 1532 1544 1556 1568 1580 1592 1604

1616 1628 1640 1652 1664 1676 1688 1700 1712 1724 1736 1748 1760 1772 1784

1796 1808 1820 1832 1844 1856 1868 1880

0 2 0 0 2.500 1257 1269 1281 1293 1305 1317 1329 1341 1353 1365 1377 1389 1401 1413 1425

1437 1449 1461 1473 1485 1497 1509 1521 1533 1545 1557 1569 1581 1593 1605

1617 1629 1641 1653 1665 1677 1689 1701 1713 1725 1737 1749 1761 1773 1785

1797 1809 1821 1833 1845 1857 1869 1881

0 1 1 0 2.500 1258 1270 1282 1294 1306 1318 1330 1342 1354 1366 1378 1390 1402 1414 1426

1438 1450 1462 1474 1486 1498 1510 1522 1534 1546 1558 1570 1582 1594 1606

1618 1630 1642 1654 1666 1678 1690 1702 1714 1726 1738 1750 1762 1774
1786

1798 1810 1822 1834 1846 1858 1870 1882

0 0 2 0 2.500 1259 1271 1283 1295 1307 1319 1331 1343 1355 1367 1379 1391 1403
1415 1427

1439 1451 1463 1475 1487 1499 1511 1523 1535 1547 1559 1571 1583 1595
1607

1619 1631 1643 1655 1667 1679 1691 1703 1715 1727 1739 1751 1763 1775
1787

1799 1811 1823 1835 1847 1859 1871 1883

Total number of charge fitting functions (nprimf) 6176

Total number of Cartesian basis functions (naos) 1883

Total number of Cartesian core functions (ncos) 43

Coordinates (Cartesian)

======================

Atom	bohr			angstrom			Geometric Variables		
	X	Y	Z	X	Y	Z	(0:frozen, *:LT par.)		
1 O	26.697283	61.417709	50.449176	14.127594	32.500852	26.696554	1	2	3
2 C	27.134526	61.805547	53.053753	14.358973	32.706087	28.074837	4	5	6
3 C	29.942675	61.120034	53.590424	15.844981	32.343329	28.358831	7	8	9
4 C	30.654965	61.724986	56.392075	16.221909	32.663456	29.841401	10	11	12
5 O	27.225564	53.016569	42.857566	14.407148	28.055160	22.679247	13	14	15
6 N	33.326858	61.346665	57.143205	17.635814	32.463257	30.238882	16	17	18
7 O	26.662535	64.426310	53.640488	14.109206	34.092935	28.385324	19	20	21
8 C	20.392373	58.554746	48.237559	10.791179	30.985837	25.526217	22	23	24
9 C	25.457986	65.551734	41.453571	13.471786	34.688484	21.936285	25	26	27

10 C	21.505446	55.101968	44.801601	11.380192	29.158706	23.707986	28	29	30
11 O	21.652240	62.966459	45.843040	11.457872	33.320415	24.259092	31	32	33
12 C	19.454164	56.729845	46.057960	10.294700	30.020141	24.372823	34	35	36
13 O	21.387093	55.114639	42.246884	11.317562	29.165411	22.356088	37	38	39
14 C	24.074208	61.494334	49.561087	12.739522	32.541400	26.226598	40	41	42
15 C	24.067291	63.306315	47.159476	12.735862	33.500259	24.955720	43	44	45
16 O	18.731523	59.583874	49.515231	9.912295	31.530428	26.202332	46	47	48
17 C	23.523553	56.912041	51.290072	12.448128	30.116555	27.141537	49	50	51
18 C	27.255076	53.132173	38.338010	14.422765	28.116335	20.287601	52	53	54
19 O	30.221541	58.386517	53.153294	15.992551	30.896814	28.127512	55	56	57
20 C	26.105210	57.099177	40.783895	13.814282	30.215583	21.581908	58	59	60
21 C	25.930922	54.186273	40.746791	13.722053	28.674141	21.562273	61	62	63
22 C	23.192639	58.734035	48.984194	12.273016	31.080713	25.921319	64	65	66
23 C	25.704714	62.789741	42.364366	13.602349	33.226900	22.418257	67	68	69
24 C	35.254417	62.442149	55.445942	18.655834	33.042962	29.340729	70	71	72
25 C	26.207145	62.466630	45.266810	13.868224	33.055917	23.954164	73	74	75
26 C	29.809698	64.470418	57.026193	15.774613	34.116276	30.176962	76	77	78
27 C	27.027921	64.972726	56.319602	14.302560	34.382086	29.803050	79	80	81
28 C	26.205785	67.721314	56.734163	13.867504	35.836576	30.022426	82	83	84
29 C	28.158983	58.561407	41.262597	14.901092	30.989362	21.835226	85	86	87
30 C	17.636281	58.115343	44.219882	9.332718	30.753315	23.400154	88	89	90
31 O	29.581794	62.627776	39.891285	15.654011	33.141192	21.109559	91	92	93
32 C	23.045439	53.408609	40.669104	12.195121	28.262619	21.521163	94	95	96
33 C	30.814381	57.661848	41.914496	16.306268	30.513336	22.180196	97	98	99
34 C	27.918455	61.425907	41.020294	14.773810	32.505190	21.707005	100	101	102
35 C	24.518607	66.063911	48.032189	12.974688	34.959516	25.417540	103	104	105
36 C	22.516121	50.584268	41.174927	11.915018	26.768042	21.788833	106	107	108
37 C	34.003044	58.764377	57.945445	17.993636	31.096769	30.663409	109	110	111

38 C	19.545798	64.535999	46.612056	10.343191	34.150980	24.666038	112	113	114
39 C	19.685897	49.855724	41.189714	10.417328	26.382513	21.796658	115	116	117
40 H	24.342184	58.072883	40.298570	12.881329	30.730846	21.325085	118	119	120
41 H	31.295076	58.210275	43.873526	16.560641	30.803551	23.216870	121	122	123
42 H	31.014667	55.605607	41.764824	16.412255	29.425220	22.100993	124	125	126
43 H	32.188206	58.623309	40.677793	17.033265	31.022119	21.525761	127	128	129
44 H	27.111693	66.685368	42.021341	14.346890	35.288377	22.236736	130	131	132
45 H	25.337469	65.634593	39.374347	13.408011	34.732331	20.836007	133	134	135
46 H	23.741852	66.429138	42.240305	12.563647	35.152786	22.352607	136	137	138
47 H	23.937335	61.769298	41.943161	12.667092	32.686905	22.195365	139	140	141
48 H	27.944622	63.500433	45.761270	14.787657	33.602982	24.215821	142	143	144
49 H	26.640703	60.462611	45.595439	14.097653	31.995436	24.128067	145	146	147
50 H	26.343933	66.164703	49.013543	13.940609	35.012853	25.936850	148	149	150
51 H	23.069599	66.711856	49.377642	12.207906	35.302394	26.129523	151	152	153
52 H	24.566360	67.373226	46.415693	12.999958	35.652376	24.562127	154	155	156
53 H	19.914079	66.552879	46.222527	10.538077	35.218267	24.459908	157	158	159
54 H	19.051726	64.282126	48.624756	10.081739	34.016636	25.731113	160	161	162
55 H	17.933250	63.913822	45.455266	9.489867	33.821738	24.053891	163	164	165
56 H	22.322993	53.909559	38.781659	11.812819	28.527710	20.522370	166	167	168

57 H	23.478906	49.534250	39.650795	12.424502	26.212396	20.982297	169	170	171
58 H	23.414087	49.986168	42.950667	12.390201	26.451541	22.728514	172	173	174
59 H	18.662746	50.749846	42.769703	9.875900	26.855662	22.632752	175	176	177
60 H	18.726027	50.401607	39.418372	9.909387	26.671382	20.859304	178	179	180
61 H	19.488804	47.791712	41.413586	10.313031	25.290285	21.915126	181	182	183
62 H	27.179780	51.051520	38.306069	14.382920	27.015301	20.270699	184	185	186
63 H	26.384917	53.885701	36.602483	13.962297	28.515085	19.369200	187	188	189
64 H	29.253760	53.702689	38.354329	15.480423	28.418239	20.296237	190	191	192
65 H	24.377446	57.986509	47.454356	12.899989	30.685139	25.111764	193	194	195
66 H	22.337963	57.504982	52.900315	11.820741	30.430326	27.993641	196	197	198
67 H	25.515037	56.859355	51.878965	13.501976	30.088675	27.453166	199	200	201
68 H	22.977686	54.979767	50.742229	12.159268	29.094040	26.851631	202	203	204
69 C	31.652890	57.604722	51.111543	16.749988	30.483106	27.047064	205	206	207
70 H	27.359727	69.013605	55.573274	14.478144	36.520427	29.408110	208	209	210
71 H	26.429519	68.252745	58.737637	13.985899	36.117797	31.082619	211	212	213
72 H	24.205322	67.975183	56.207464	12.808905	35.970918	29.743709	214	215	216
73 H	25.783419	63.696418	57.433866	13.643998	33.706693	30.392693	217	218	219
74 H	30.999198	65.837847	55.991999	16.404069	34.839888	29.629690	220	221	222
75 H	30.115010	64.790129	59.064099	15.936177	34.285460	31.255375	223	224	225

76 H	35.514638	61.395684	53.642068	18.793537	32.489197	28.386160	226	227	228
77 H	34.773550	64.414074	54.979788	18.401370	34.086460	29.094051	229	230	231
78 H	37.087523	62.469616	56.444233	19.625872	33.057497	29.869002	232	233	234
79 H	34.162656	57.364829	56.385896	18.078099	30.356160	29.838131	235	236	237
80 H	32.591923	58.053390	59.304808	17.246903	30.720531	31.382753	238	239	240
81 H	35.845346	58.830487	58.926984	18.968540	31.131753	31.182817	241	242	243
82 H	25.869154	60.613236	54.231154	13.689367	32.075143	28.697891	244	245	246
83 H	22.866351	62.326558	51.040327	12.100352	32.981794	27.009378	247	248	249
84 H	31.145347	62.145545	52.240929	16.481408	32.886006	27.644709	250	251	252
85 H	29.535093	60.424877	57.584653	15.629298	31.975468	30.472486	253	254	255
86 H	26.100071	53.225911	44.338601	13.811563	28.165939	23.462977	256	257	258
87 O	23.053652	53.845180	46.039864	12.199467	28.493642	24.363247	259	260	261
88 H	18.324155	55.338804	47.136841	9.696725	29.284034	24.943742	262	263	264
89 H	33.302655	54.029239	50.079141	17.623006	28.591042	26.500740	265	266	267
90 H	30.940794	53.790242	52.543440	16.373163	28.464570	27.804791	268	269	270
91 H	30.042256	54.338270	49.330497	15.897677	28.754574	26.104575	271	272	273
92 C	31.483939	54.752488	50.781809	16.660583	28.973769	26.872576	274	275	276
93 O	32.883886	59.019161	49.744759	17.401403	31.231595	26.323793	277	278	279
94 H	18.653762	59.476108	43.020343	9.871146	31.473401	22.765385	280	281	282

95 H 16.209320 59.140185 45.332069 8.577603 31.295638 23.988698 283 284
285

96 H 16.662518 56.753840 42.983353 8.817425 30.032839 22.745811 286 287
288

1

****

****

* T E C H N I C A L *

****

P A R A L L E L I Z A T I O N and V E C T O R I Z A T I O N

Nr of parallel processes: 8

Maximum vector length in NumInt loops: 128

IO buffersize (Mb): 64.000000

G E O M E T R Y U P D A T E S

Optimization coordinates: Delocalized

Optimization code branch: NEW

Hessian update method: BFGS (Broyden-Fletcher-Goldfarb-Shanno)

Iterative Geometry Improvements:

Max. nr. of iterations: 576

Convergence criteria:

Change in energy (hartree): 0.0010000000

Gradients (au/Angstrom): 0.0010000000

Cart/bondlength changes (Angstrom): 0.0100000000

Max. step to take:

Trust Radius: 0.2000000000

GDIIS (Geometry Direct Inversion in Iteration Space):

First geometry cycle: 0

Max. number expansion vectors: 5

S C F U P D A T E S

Max. nr. of cycles: 300

Convergence criterion: 0.0000010000

secondary criterion: 0.0010000000

Mix parameter (when DIIS does not apply): 0.2000000000

Mixed ADIIS+DIIS will be used

Max number of expansion vectors: 10

Pure ADIIS when Max([F,P]) is above 0.1000000000

Pure DIIS when Max([F,P]) is below 0.0010000000

P R E C I S I O N *** (General: NumInt, NeglectFunctionTails, ...) ***

NumInt: Target precision: 4.0000000000

------- Initial precision: 4.0000000000

Min. precision (optimization): 4.0000000000

Neglect Functions: Basis functions: 0.1000000000E-05

------------------ Fit functions: 0.1000000000E-05

L I N E A R S C A L I N G

Cut-off radii density fit: 0.1000000000E-07

Overlap cut-off criterion AO matrix elements: 0.1000000000E-05

Cut-offs for Coulomb potential and fitted density:0.1000000000E-07

Cut-off criterion for Coulomb multipole terms: 0.1000000000E-07

Progressive Convergence parameter: 0.000000000

****

****

* C O M P U T A T I O N *

****

Number of elements of the density matrix on this node (used, total): 222269 1773786

Numerical Integration : Fuzzy Cells (Becke)

Becke grid quality: NORMAL

Lebedev angular grid order: min: 11 max: 23

Nr. of radial points: min: 46 max: 52

Total nr. of points: 600016

Nr. of blocks: 4688

Block length: 128

Nr. of dummy points: 48

Test of Precision of the Numerical Integration Grid

==

Integral of the Total Core Density: 85.99994382725670

Relative Error: -6.532E-07

Density Fitting (zlmFit)

ZlmFit Fit Quality: NORMAL

Max L-expansion: min: 6 max: 7

Nr. of radial interpolation points: min: 39 max: 48

S C F *** ScaSCF ***

Initial density matrix is calculated as sum of fragments

CYCLE 1

SCF Error: norm([F,P])= 5.591976581110, max([F,P])= 0.223027878582

orbitals (Q,E):

A :114...133 (2.00 -0.4132) (2.00 -0.4117) (2.00 -0.4079) (2.00 -0.4032)

(2.00 -0.3901) (2.00 -0.3895) (2.00 -0.3875) (2.00 -0.3867)

(2.00 -0.3809) (2.00 -0.3575) (0.00 -0.2263) (0.00 -0.2012)

(0.00 -0.1670) (0.00 -0.1650) (0.00 -0.1571) (0.00 -0.1154)

(0.00 -0.1021) (0.00 -0.0999) (0.00 -0.0898) (0.00 -0.0810)

CYCLE 2

SDIIS (wt 0.000): 0.2688 0.7312

A-DIIS (wt 1.000): 0.0000 1.0000

DIIS coefficients: 0.0000 1.0000

SCF Error: norm([F,P])= 2.866205919024, max([F,P])= 0.215392472325

orbitals (Q,E):

A :114...124 (2.00 -0.0984) (2.00 -0.0967) (2.00 -0.0899) (2.00 -0.0756)

(2.00 -0.0641) (2.00 -0.0559) (2.00 -0.0549) (2.00 -0.0489)

(2.00 -0.0183) (2.00 -0.0085) (0.00 -0.0023)

CYCLE 3

SDIIS (wt 0.000): 0.0727 0.6263 0.3011

A-DIIS (wt 1.000): 0.0000 0.7665 0.2335

DIIS coefficients: 0.0000 0.7665 0.2335

SCF Error: norm([F,P])= 5.342520203299, max([F,P])= 0.516062407081

orbitals (Q,E):

A :114...124 (2.00 -0.1910) (2.00 -0.1873) (2.00 -0.1678) (2.00 -0.1519)

(2.00 -0.1505) (2.00 -0.1397) (2.00 -0.1338) (2.00 -0.1245)

(2.00 -0.1218) (2.00 -0.0876) (0.00 -0.0453)

CYCLE 4

SDIIS (wt 0.000): 0.4533 0.1526 0.3940

A-DIIS (wt 1.000): 0.2878 0.0000 0.7122

DIIS coefficients: 0.2878 0.0000 0.7122

SCF Error: norm([F,P])= 1.790369297991, max([F,P])= 0.166945123089

orbitals (Q,E):

A :114...124 (2.00 -0.2670) (2.00 -0.2606) (2.00 -0.2568) (2.00 -0.2400)
(2.00 -0.2374) (2.00 -0.2317) (2.00 -0.2157) (2.00 -0.2067)
(2.00 -0.1996) (2.00 -0.1721) (0.00 -0.1199)

CYCLE 5

SDIIS (wt 0.000): 0.2712 -0.0013 0.5443 0.1858

A-DIIS (wt 1.000): 0.1933 0.0000 0.6483 0.1584

DIIS coefficients: 0.1933 0.0000 0.6483 0.1584

SCF Error: norm([F,P])= 3.181173461216, max([F,P])= 0.335107252830

orbitals (Q,E):

A :114...124 (2.00 -0.2720) (2.00 -0.2563) (2.00 -0.2540) (2.00 -0.2502)
(2.00 -0.2474) (2.00 -0.2387) (2.00 -0.2313) (2.00 -0.2242)
(2.00 -0.2166) (2.00 -0.2017) (0.00 -0.0807)

CYCLE 6

SDIIS (wt 0.424): 0.0892 0.0037 0.3588 0.0848 0.4636

A-DIIS (wt 0.576): 0.0000 0.0000 0.1250 0.0000 0.8750

DIIS coefficients: 0.0378 0.0016 0.2242 0.0360 0.7005

SCF Error: norm([F,P])= 0.727800880720, max([F,P])= 0.058017684782

orbitals (Q,E):

A :114...124 (2.00 -0.2450) (2.00 -0.2427) (2.00 -0.2361) (2.00 -0.2241)
(2.00 -0.2207) (2.00 -0.2140) (2.00 -0.2092) (2.00 -0.2042)
(2.00 -0.1995) (2.00 -0.1676) (0.00 -0.0760)

CYCLE 7

SDIIS (wt 0.424): 0.0004 0.0069 0.1252 0.0173 0.5183 0.3319

A-DIIS (wt 0.576): 0.0000 0.0000 0.0000 0.0000 0.4104 0.5896

DIIS coefficients: 0.0002 0.0029 0.0531 0.0073 0.4561 0.4803

SCF Error: norm([F,P])= 0.613665252467, max([F,P])= 0.079944472399

orbitals (Q,E):

A :114...124 (2.00 -0.2485) (2.00 -0.2436) (2.00 -0.2361) (2.00 -0.2271)

(2.00 -0.2204) (2.00 -0.2184) (2.00 -0.2176) (2.00 -0.2094)

(2.00 -0.1877) (2.00 -0.1837) (0.00 -0.0683)

CYCLE 8

SDIIS (wt 0.424): -0.0061 -0.0013 0.0318 0.0031 0.3171 0.3737 0.2816

A-DIIS (wt 0.576): 0.0000 0.0000 0.0000 0.0000 0.0000 0.2723 0.7277

DIIS coefficients: -0.0026 -0.0005 0.0135 0.0013 0.1345 0.3153 0.5385

SCF Error: norm([F,P])= 0.369695159040, max([F,P])= 0.068418351257

orbitals (Q,E):

A :114...124 (2.00 -0.2521) (2.00 -0.2501) (2.00 -0.2484) (2.00 -0.2385)

(2.00 -0.2301) (2.00 -0.2234) (2.00 -0.2228) (2.00 -0.2171)

(2.00 -0.2042) (2.00 -0.1717) (0.00 -0.0772)

CYCLE 9

SDIIS (wt 0.424): -0.0029 0.0013 0.0037 -0.0020 0.1387 0.2344 0.3837 0.2429

A-DIIS (wt 0.576): 0.0000 0.0000 0.0000 0.0000 0.0000 0.0000 0.4442 0.5558

DIIS coefficients: -0.0012 0.0006 0.0016 -0.0009 0.0588 0.0994 0.4186 0.4231

SCF Error: norm([F,P])= 0.311058604054, max([F,P])= 0.061550366266

orbitals (Q,E):

A :114...124 (2.00 -0.2470) (2.00 -0.2458) (2.00 -0.2347) (2.00 -0.2335)

(2.00 -0.2273) (2.00 -0.2202) (2.00 -0.2189) (2.00 -0.2144)

(2.00 -0.1991) (2.00 -0.1768) (0.00 -0.0744)

CYCLE 10

SDIIS (wt 0.907): -0.0013 -0.0006 0.0002 0.0013 0.0135 0.0478 0.2950 0.3131 0.3311

A-DIIS (wt 0.093): 0.0000 0.0000 0.0000 0.0000 0.0000 0.0000 0.0000 0.0117 0.9883

DIIS coefficients: -0.0012 -0.0005 0.0002 0.0012 0.0123 0.0433 0.2676 0.2852 0.3921

SCF Error: norm([F,P])= 0.094878032858, max([F,P])= 0.010188705855

orbitals (Q,E):

A :114...124 (2.00 -0.2490) (2.00 -0.2465) (2.00 -0.2365) (2.00 -0.2358)

(2.00 -0.2282) (2.00 -0.2207) (2.00 -0.2197) (2.00 -0.2135)

(2.00 -0.1970) (2.00 -0.1782) (0.00 -0.0734)

CYCLE 11

SDIIS (wt 0.966): -0.0011 -0.0007 -0.0015 0.0004 -0.0115 -0.0109 0.1141 0.1525 0.3208 0.4379

A-DIIS (wt 0.034): 0.0000 0.0000 0.0000 0.0000 0.0000 0.0000 0.0000 0.0000 0.0820 0.9180

DIIS coefficients: -0.0010 -0.0007 -0.0015 0.0004 -0.0111 -0.0105 0.1102 0.1473 0.3127 0.4541

SCF Error: norm([F,P])= 0.027870365608, max([F,P])= 0.004336027968

orbitals (Q,E):

A :114...124 (2.00 -0.2485) (2.00 -0.2461) (2.00 -0.2358) (2.00 -0.2350)

(2.00 -0.2280) (2.00 -0.2203) (2.00 -0.2194) (2.00 -0.2141)

(2.00 -0.1975) (2.00 -0.1769) (0.00 -0.0733)

CYCLE 12

SDIIS (wt 0.998): -0.0001 0.0000 0.0002 -0.0078 -0.0153 0.0274 0.0526 0.1729 0.3570 0.4130

A-DIIS (wt 0.002): 0.0000 0.0000 0.0000 0.0000 0.0000 0.0000 0.0000 0.0000 0.0788 0.9212

DIIS coefficients: -0.0001 0.0000 0.0002 -0.0078 -0.0153 0.0274 0.0525 0.1726 0.3565 0.4140

SCF Error: norm([F,P])= 0.008228529949, max([F,P])= 0.001188394015

orbitals (Q,E):

A :114...124 (2.00 -0.2486) (2.00 -0.2461) (2.00 -0.2360) (2.00 -0.2351)

(2.00 -0.2281) (2.00 -0.2204) (2.00 -0.2195) (2.00 -0.2141)

(2.00 -0.1974) (2.00 -0.1774) (0.00 -0.0734)

CYCLE 13

SDIIS (wt 1.000): -0.0000 0.0000 -0.0029 -0.0069 0.0082 0.0193 0.0788 0.1882 0.2714 0.4440

DIIS coefficients: -0.0000 0.0000 -0.0029 -0.0069 0.0082 0.0193 0.0788 0.1882 0.2714 0.4440

SCF Error: norm([F,P])= 0.001827864714, max([F,P])= 0.000245365610

orbitals (Q,E):

A :114...124 (2.00 -0.2486) (2.00 -0.2462) (2.00 -0.2360) (2.00 -0.2351)

(2.00 -0.2280) (2.00 -0.2204) (2.00 -0.2195) (2.00 -0.2141)

(2.00 -0.1975) (2.00 -0.1773) (0.00 -0.0735)

CYCLE 14

SDIIS (wt 1.000): -0.0000 -0.0009 -0.0026 0.0023 0.0067 0.0319 0.0840 0.1356 0.2917 0.4513

DIIS coefficients: -0.0000 -0.0009 -0.0026 0.0023 0.0067 0.0319 0.0840 0.1356 0.2917 0.4513

SCF Error: norm([F,P])= 0.000572708389, max([F,P])= 0.000074348410

orbitals (Q,E):

A :114...124 (2.00 -0.2486) (2.00 -0.2462) (2.00 -0.2360) (2.00 -0.2351)

(2.00 -0.2281) (2.00 -0.2205) (2.00 -0.2195) (2.00 -0.2141)

(2.00 -0.1975) (2.00 -0.1773) (0.00 -0.0735)

CYCLE 15

SDIIS (wt 1.000): -0.0004 -0.0012 0.0009 0.0029 0.0145 0.0398 0.0674 0.1600 0.3228 0.3933

DIIS coefficients: -0.0004 -0.0012 0.0009 0.0029 0.0145 0.0398 0.0674 0.1600 0.3228 0.3933

SCF Error: norm([F,P])= 0.000239578657, max([F,P])= 0.000035119495

orbitals (Q,E):

A :114...124 (2.00 -0.2486) (2.00 -0.2462) (2.00 -0.2360) (2.00 -0.2351)

(2.00 -0.2280) (2.00 -0.2204) (2.00 -0.2195) (2.00 -0.2141)

(2.00 -0.1975) (2.00 -0.1773) (0.00 -0.0735)

CYCLE 16

SDIIS (wt 1.000): -0.0001 0.0000 0.0003 0.0022 0.0081 0.0166 0.0547 0.1506 0.3111 0.4565

DIIS coefficients: -0.0001 0.0000 0.0003 0.0022 0.0081 0.0166 0.0547 0.1506 0.3111 0.4565

SCF Error: norm([F,P])= 0.000102960458, max([F,P])= 0.000019096561

orbitals (Q,E):

A :114...124 (2.00 -0.2486) (2.00 -0.2462) (2.00 -0.2360) (2.00 -0.2351)

(2.00 -0.2280) (2.00 -0.2204) (2.00 -0.2195) (2.00 -0.2141)

(2.00 -0.1975) (2.00 -0.1773) (0.00 -0.0735)

CYCLE 17

SDIIS (wt 1.000): -0.0000 0.0000 0.0004 0.0020 0.0047 0.0187 0.0573 0.1595 0.3214 0.4359

DIIS coefficients: -0.0000 0.0000 0.0004 0.0020 0.0047 0.0187 0.0573 0.1595 0.3214 0.4359

SCF Error: norm([F,P])= 0.000041791619, max([F,P])= 0.000005132879

orbitals (Q,E):

A :114...124 (2.00 -0.2486) (2.00 -0.2462) (2.00 -0.2360) (2.00 -0.2351)

(2.00 -0.2280) (2.00 -0.2204) (2.00 -0.2195) (2.00 -0.2141)

(2.00 -0.1975) (2.00 -0.1773) (0.00 -0.0735)

CYCLE 18

SDIIS (wt 1.000): 0.0000 0.0003 0.0012 0.0026 0.0099 0.0290 0.0825 0.1718 0.2846 0.4181

DIIS coefficients: 0.0000 0.0003 0.0012 0.0026 0.0099 0.0290 0.0825 0.1718 0.2846 0.4181

SCF Error: norm([F,P])= 0.000013884057, max([F,P])= 0.000002028411

orbitals (Q,E):

A :114...124 (2.00 -0.2486) (2.00 -0.2462) (2.00 -0.2360) (2.00 -0.2351)

(2.00 -0.2280) (2.00 -0.2204) (2.00 -0.2195) (2.00 -0.2141)

(2.00 -0.1975) (2.00 -0.1773) (0.00 -0.0735)

CYCLE 19

SDIIS (wt 1.000): 0.0000 0.0001 0.0000 0.0002 0.0000 0.0056 0.0220 0.0860 0.2861 0.6001

DIIS coefficients: 0.0000 0.0001 0.0000 0.0002 0.0000 0.0056 0.0220 0.0860 0.2861 0.6001

SCF Error: norm([F,P])= 0.000005625275, max([F,P])= 0.000000925529

orbitals (Q,E):

```
A :114...124      ( 2.00   -0.2486)  ( 2.00   -0.2462)  ( 2.00   -0.2360)  ( 2.00   -0.2351)
                  ( 2.00   -0.2280)  ( 2.00   -0.2204)  ( 2.00   -0.2195)  ( 2.00   -0.2141)
                  ( 2.00   -0.1975)  ( 2.00   -0.1773)  ( 0.00   -0.0735)
```

SCF CONVERGED

CYCLE 20

*** Setting up for NEW gradients in Focky

*** Using FIT density in Focky

*** Setting up for NEW Hessian in Focky

orbitals (Q,E):

```
A :114...133      ( 2.00   -0.2486)  ( 2.00   -0.2462)  ( 2.00   -0.2360)  ( 2.00   -0.2351)
                  ( 2.00   -0.2280)  ( 2.00   -0.2204)  ( 2.00   -0.2195)  ( 2.00   -0.2141)
                  ( 2.00   -0.1975)  ( 2.00   -0.1773)  ( 0.00   -0.0735)  ( 0.00   -0.0713)
                  ( 0.00   -0.0647)  ( 0.00   -0.0422)  ( 0.00   -0.0204)  ( 0.00   -0.0157)
                  ( 0.00   -0.0137)  ( 0.00   -0.0105)  ( 0.00   -0.0060)  ( 0.00   -0.0011)
```

Fit test: (difference of exact and fit density, squared integrated, result summed over spins)

SCF: 0.00098908897263

Gross Charges per Atom (Z minus electrons)

==

```
-0.6529   0.9022   0.5178   0.3273  -0.5783  -0.3832  -0.6315   0.6595   0.6139   0.8454
-0.6526  -0.0016  -0.5731   0.5810   0.3205  -0.5873   0.5779   0.5835  -0.5850   0.2423
 0.3348   0.1095   0.1623   0.7231   0.3943   0.3996   0.5580   0.5551   0.0297   0.6209
-0.5613   0.5295   0.5358   0.5633   0.6293   0.3711   0.7300   0.9608   0.5625  -0.2005
```

-0.1718	-0.2047	-0.1843	-0.2059	-0.2344	-0.2243	-0.1469	-0.1848	-0.1355	-0.2032
-0.2309	-0.2137	-0.2140	-0.1809	-0.2382	-0.1647	-0.1858	-0.1475	-0.1935	-0.2076
-0.2135	-0.2097	-0.2220	-0.1870	-0.1170	-0.2123	-0.1727	-0.2022	0.8191	-0.1907
-0.2109	-0.2101	-0.2080	-0.1819	-0.2043	-0.1656	-0.2199	-0.2248	-0.1962	-0.2337
-0.2029	-0.2376	-0.1918	-0.1532	-0.1564	0.2425	-0.6707	-0.1178	-0.1691	-0.1866
-0.1053	0.4652	-0.6123	-0.1701	-0.2058	-0.2259				

Net Total: 0.00000000

M U L T I P O L E D E R I V E D C H A R G E A N A L Y S I S

See: M. Swart, P.Th. van Duijnen, J.G. Snijders, J.Comput.Chem., (2001), p. 79-88.

Multipole derived atomic charges (a.u.)

Atom	Level:	MDC-m	MDC-d	MDC-q
1	O	-0.358201	-0.350792	-0.375584
2	C	0.365373	0.375851	0.399908
3	C	0.243985	0.249794	0.254184
4	C	0.180525	0.162333	0.208072
5	O	-0.311234	-0.546553	-0.616827
6	N	-0.216249	-0.262877	-0.443872
7	O	-0.380761	-0.369485	-0.438442
8	C	0.253203	0.305887	0.304713
9	C	0.226812	0.190365	0.174453

10	C	0.341376	0.406590	0.412586
11	O	-0.385858	-0.357197	-0.391355
12	C	0.124913	0.098473	0.094699
13	O	-0.315100	-0.304098	-0.346500
14	C	0.246575	0.244047	0.249463
15	C	0.195963	0.158063	0.174981
16	O	-0.377603	-0.480637	-0.486030
17	C	0.227937	0.192243	0.190895
18	C	0.228125	0.188939	0.180193
19	O	-0.322581	-0.329807	-0.398579
20	C	0.055993	0.043482	-0.073426
21	C	0.195398	0.219141	0.287071
22	C	0.119908	0.102317	0.107062
23	C	0.120872	0.099236	0.108873
24	C	0.289033	0.262603	0.282695
25	C	0.170749	0.132618	0.134731
26	C	0.163694	0.121423	0.161451
27	C	0.248214	0.234104	0.259523
28	C	0.232819	0.190472	0.275472
29	C	0.034398	0.025239	-0.057471
30	C	0.235657	0.187920	0.171373
31	O	-0.375976	-0.474172	-0.522551
32	C	0.254081	0.242450	0.264824
33	C	0.225112	0.184629	0.154753
34	C	0.229185	0.291881	0.400859
35	C	0.220661	0.189237	0.198416
36	C	0.179787	0.142305	0.150197
37	C	0.293038	0.266663	0.306344
38	C	0.369088	0.338206	0.385861
39	C	0.228094	0.187080	0.147576
40	H	-0.072280	-0.059350	-0.017801

41	H	-0.056406	-0.028983	-0.013209
42	H	-0.061375	-0.050664	-0.014255
43	H	-0.055011	-0.033606	0.001927
44	H	-0.068258	-0.050556	-0.045546
45	H	-0.068768	-0.054020	-0.044894
46	H	-0.075867	-0.070747	-0.067426
47	H	-0.070353	-0.058382	-0.063285
48	H	-0.066357	-0.044902	-0.027219
49	H	-0.081871	-0.064080	-0.071273
50	H	-0.055699	-0.047547	-0.046720
51	H	-0.071479	-0.052719	-0.058139
52	H	-0.070300	-0.057514	-0.061960
53	H	-0.066780	-0.062888	-0.074919
54	H	-0.057098	-0.044225	-0.052289
55	H	-0.051731	-0.040282	-0.043351
56	H	-0.056487	-0.041154	-0.030044
57	H	-0.067456	-0.048995	-0.052797
58	H	-0.060886	-0.034684	-0.034352
59	H	-0.074190	-0.058877	-0.046130
60	H	-0.074148	-0.065680	-0.054213
61	H	-0.068319	-0.051803	-0.039341
62	H	-0.065619	-0.044963	-0.030294
63	H	-0.071910	-0.074437	-0.077002
64	H	-0.058174	-0.035769	-0.035084
65	H	-0.071203	-0.043535	-0.050307
66	H	-0.071075	-0.063032	-0.056071
67	H	-0.065934	-0.050644	-0.059177
68	H	-0.064279	-0.052779	-0.049530
69	C	0.353798	0.443461	0.475962
70	H	-0.063700	-0.052374	-0.073693
71	H	-0.067374	-0.065208	-0.089914

72	H	-0.063489	-0.047684	-0.065880
73	H	-0.079638	-0.062291	-0.072968
74	H	-0.072051	-0.059437	-0.065300
75	H	-0.069381	-0.068712	-0.071598
76	H	-0.072789	-0.043417	-0.033688
77	H	-0.066401	-0.053903	-0.043068
78	H	-0.064887	-0.060042	-0.033687
79	H	-0.083227	-0.063454	-0.047803
80	H	-0.066199	-0.057236	-0.053010
81	H	-0.066897	-0.063320	-0.054090
82	H	-0.069212	-0.041828	-0.036078
83	H	-0.061170	-0.021461	-0.018132
84	H	-0.043780	0.004288	-0.009012
85	H	-0.071504	-0.057086	-0.039488
86	H	0.066456	0.263386	0.310072
87	O	-0.369876	-0.441059	-0.458551
88	H	-0.043463	0.007050	0.028027
89	H	-0.045279	-0.028748	-0.032371
90	H	-0.052015	-0.051104	-0.060815
91	H	-0.040330	-0.009586	-0.025069
92	C	0.239059	0.194000	0.242079
93	O	-0.375697	-0.475152	-0.507004
94	H	-0.061693	-0.054293	-0.048501
95	H	-0.060046	-0.043952	-0.034904
96	H	-0.066911	-0.061993	-0.057409

*** Using NEW gradient routines ***

Geometry CYCLE 1

==============

Energy gradients wrt nuclear displacements

===

Atom	Cartesian (a.u./angstrom)		
	X	Y	Z
1 O	0.003764	-0.001480	-0.007105
2 C	-0.001695	0.000049	0.004561
3 C	0.001209	-0.001860	-0.000380
4 C	0.001425	-0.000123	-0.001253
5 O	0.010751	-0.006279	-0.005073
6 N	-0.001198	0.005920	0.007916
7 O	-0.001111	0.006071	-0.001577
8 C	0.021150	-0.011456	-0.018177
9 C	0.001839	-0.002207	-0.001080
10 C	-0.016592	0.015779	-0.007512
11 O	-0.001661	-0.004595	-0.002814
12 C	0.000225	0.000688	0.002419
13 O	-0.001974	0.002851	-0.012044
14 C	0.001519	0.000335	0.000083
15 C	-0.001687	-0.001022	-0.000260
16 O	-0.026270	0.012881	0.019222
17 C	-0.001895	0.002013	-0.001637
18 C	-0.000079	-0.000503	0.001093
19 O	-0.003881	-0.003178	0.005001
20 C	-0.009117	-0.009755	-0.003400
21 C	-0.001634	-0.002609	0.001839
22 C	-0.000917	0.002580	0.001968
23 C	0.002536	0.000126	-0.001441
24 C	-0.001529	-0.003594	0.000263
25 C	-0.003915	0.002835	-0.000492

26	C	-0.001644	-0.001262	-0.001036
27	C	-0.000714	-0.001632	-0.000428
28	C	0.001244	-0.001788	0.000014
29	C	0.008736	0.008813	0.001127
30	C	0.001350	-0.002356	0.000790
31	O	0.023168	0.016886	-0.016160
32	C	0.001149	0.001972	0.005056
33	C	0.002390	0.000426	-0.000590
34	C	-0.019793	-0.012158	0.011135
35	C	-0.000773	-0.001694	-0.001144
36	C	-0.000354	0.001722	-0.000484
37	C	-0.000276	0.002184	-0.003484
38	C	0.001985	0.000813	0.001262
39	C	0.002205	-0.000079	-0.000191
40	H	-0.007741	0.004920	-0.001773
41	H	0.002882	0.001362	0.006512
42	H	0.001058	-0.007237	-0.001068
43	H	0.005692	0.003945	-0.003214
44	H	0.005951	0.005620	0.001938
45	H	-0.000629	0.001131	-0.008391
46	H	-0.006307	0.004888	0.001998
47	H	-0.005229	-0.002638	-0.000850
48	H	0.006126	0.003795	0.000753
49	H	0.001663	-0.006154	0.000856
50	H	0.005818	0.001649	0.002713
51	H	-0.005650	0.003913	0.004491
52	H	0.000818	0.007318	-0.005582
53	H	0.001356	0.008237	-0.001022
54	H	-0.003009	0.000931	0.007776
55	H	-0.007299	-0.000605	-0.004758
56	H	-0.002464	0.001500	-0.006360

57	H	0.003327	-0.004295	-0.005651
58	H	0.003333	-0.004558	0.006369
59	H	-0.004922	0.001711	0.005965
60	H	-0.004593	0.001130	-0.006447
61	H	-0.000824	-0.008237	0.001354
62	H	0.000696	-0.008364	-0.000667
63	H	-0.002361	0.002339	-0.006973
64	H	0.008130	0.001637	-0.000669
65	H	0.003744	-0.004471	-0.004239
66	H	-0.005160	-0.000124	0.008040
67	H	0.005770	-0.001337	0.002312
68	H	-0.001534	-0.008471	-0.001368
69	C	-0.013978	-0.016942	0.015410
70	H	0.003940	0.006215	-0.004329
71	H	0.000235	0.002677	0.007318
72	H	-0.007969	0.001672	-0.001467
73	H	-0.004164	-0.003984	0.003545
74	H	0.004746	0.004768	-0.003609
75	H	0.001291	0.001529	0.007757
76	H	0.002970	-0.001930	-0.006858
77	H	-0.001263	0.008157	-0.001829
78	H	0.008120	0.000469	0.003803
79	H	0.001866	-0.006653	-0.002673
80	H	-0.004491	-0.003245	0.005760
81	H	0.007342	-0.000074	0.004237
82	H	-0.003795	-0.003354	0.003609
83	H	-0.002571	0.002816	0.006263
84	H	0.005518	0.002520	-0.005324
85	H	-0.003566	-0.004881	0.004554
86	H	-0.008029	0.000453	0.008772
87	O	0.021060	-0.017977	0.016440

88 H -0.004539 -0.002966 0.003040

89 H 0.006655 -0.004407 -0.003289

90 H -0.002186 -0.004882 0.006736

91 H -0.004283 -0.003256 -0.005550

92 C 0.000660 0.002578 0.000234

93 O 0.019824 0.021056 -0.022174

94 H 0.001196 0.005527 -0.006620

95 H -0.006116 0.003720 0.003228

96 H -0.005057 -0.004454 -0.005014

Max Cartesian displacement 1.94811111950084

Limit Cartesian step to 0.566917837497000

Geometry Convergence after Step 1 (Hartree/Angstrom,Angstrom)

----

current energy	-19.66623345 Hartree		
energy change	-19.66623345	0.00100000	F
constrained gradient max	0.02626993	0.00100000	F
constrained gradient rms	0.00650519	0.00066667	F
gradient max	0.02626993		
gradient rms	0.00650519		
cart. step max	0.29480461	0.01000000	F
cart. step rms	0.07962579	0.00666667	F

Coordinates (Cartesian)

======================

Atom bohr angstrom Geometric Variables

	X	Y	Z	X	Y	Z	(0:frozen, *:LT par.)		
1 O	26.722782	61.415601	50.475286	14.141087	32.499737	26.710371	1	2	3
2 C	27.144206	61.911813	53.057876	14.364095	32.762320	28.077019	4	5	6
3 C	29.926392	61.184926	53.631837	15.836365	32.377668	28.380746	7	8	9
4 C	30.644186	61.880187	56.403952	16.216205	32.745585	29.847686	10	11	12
5 O	27.167654	52.992762	42.656052	14.376503	28.042562	22.572611	13	14	15
6 N	33.299594	61.432996	57.157845	17.621386	32.508941	30.246629	16	17	18
7 O	26.736115	64.564008	53.525889	14.148143	34.165802	28.324681	19	20	21
8 C	20.496762	58.378891	48.370742	10.846419	30.892779	25.596695	22	23	24
9 C	25.485526	65.523178	41.553706	13.486360	34.673372	21.989274	25	26	27
10 C	21.563659	55.084356	44.772658	11.410997	29.149386	23.692670	28	29	30
11 O	21.679079	62.704859	45.825281	11.472075	33.181982	24.249694	31	32	33
12 C	19.532095	56.634611	46.143455	10.335940	29.969746	24.418065	34	35	36
13 O	21.385347	55.225460	42.230417	11.316638	29.224055	22.347374	37	38	39
14 C	24.102712	61.400323	49.592908	12.754606	32.491652	26.243437	40	41	42
15 C	24.045247	63.184893	47.180926	12.724197	33.436006	24.967071	43	44	45
16 O	18.863577	59.364408	49.697013	9.982175	31.414292	26.298527	46	47	48
17 C	23.767345	56.808228	51.319784	12.577138	30.061620	27.157260	49	50	51
18 C	27.133674	53.286439	38.143362	14.358522	28.197969	20.184598	52	53	54
19 O	30.132325	58.433373	53.304616	15.945340	30.921609	28.207588	55	56	57
20 C	26.112995	57.166794	40.759718	13.818402	30.251365	21.569114	58	59	60
21 C	25.872557	54.268002	40.610897	13.691167	28.717390	21.490361	61	62	63
22 C	23.309545	58.617539	49.030843	12.334880	31.019066	25.946005	64	65	66
23 C	25.764197	62.758482	42.433479	13.633826	33.210358	22.454830	67	68	69
24 C	35.244239	62.389058	55.402799	18.650448	33.014868	29.317898	70	71	72
25 C	26.243569	62.420836	45.332797	13.887498	33.031684	23.989083	73	74	75
26 C	29.882732	64.672171	56.914895	15.813261	34.223039	30.118065	76	77	78
27 C	27.119880	65.220517	56.176411	14.351222	34.513211	29.727277	79	80	81
28 C	26.378711	68.004581	56.462034	13.959013	35.986474	29.878421	82	83	84
29 C	28.193871	58.558626	41.280962	14.919554	30.987890	21.844944	85	86	87

30 C	17.680370	58.094469	44.406467	9.356049	30.742269	23.498890	88	89	90
31 O	29.675900	62.634858	40.022368	15.703810	33.144939	21.178925	91	92	93
32 C	22.971225	53.565355	40.539576	12.155849	28.345565	21.452620	94	95	96
33 C	30.815704	57.582457	41.938429	16.306968	30.471324	22.192861	97	98	99
34 C	28.003482	61.427165	41.108910	14.818804	32.505856	21.753899	100	101	102
35 C	24.359458	65.960571	48.042473	12.890470	34.904831	25.422982	103	104	105
36 C	22.367094	50.742305	40.926601	11.836156	26.851672	21.657424	106	107	108
37 C	33.872298	58.848309	58.018579	17.924448	31.141184	30.702110	109	110	111
38 C	19.450029	64.088105	46.590916	10.292512	33.913965	24.654851	112	113	114
39 C	19.515663	50.118569	40.950664	10.327244	26.521605	21.670158	115	116	117
40 H	24.375380	58.195262	40.312594	12.898896	30.795606	21.332506	118	119	120
41 H	31.291730	58.090068	43.906611	16.558870	30.739940	23.234378	121	122	123
42 H	30.963344	55.526832	41.761098	16.385096	29.383534	22.099021	124	125	126
43 H	32.220755	58.524219	40.726810	17.050489	30.969683	21.551700	127	128	129
44 H	27.116486	66.670097	42.148100	14.349427	35.280296	22.303814	130	131	132
45 H	25.380473	65.627720	39.477922	13.430768	34.728694	20.890817	133	134	135
46 H	23.752923	66.362655	42.337475	12.569506	35.117605	22.404027	136	137	138
47 H	24.017168	61.719206	41.985622	12.709338	32.660397	22.217835	139	140	141
48 H	27.938787	63.501694	45.860462	14.784569	33.603650	24.268311	142	143	144
49 H	26.728350	60.430273	45.655847	14.144034	31.978323	24.160034	145	146	147

50 H 26.162578 66.146321 49.046894 13.844640 35.003125 25.954498 148 149
150

51 H 22.866431 66.546690 49.363081 12.100394 35.214992 26.121817 151 152
153

52 H 24.369815 67.257295 46.419649 12.895950 35.591028 24.564220 154 155
156

53 H 19.659491 66.131059 46.238801 10.403355 34.995049 24.468520 157 158
159

54 H 18.957807 63.758127 48.589708 10.032039 33.739348 25.712566 160 161
162

55 H 17.909659 63.356722 45.405924 9.477383 33.526933 24.027780 163 164
165

56 H 22.232670 54.174026 38.693478 11.765022 28.667660 20.475707 166 167
168

57 H 23.273448 49.732289 39.346050 12.315778 26.317194 20.821033 169 170
171

58 H 23.266619 50.043355 42.660505 12.312165 26.481803 22.574967 172 173
174

59 H 18.550854 50.982618 42.579396 9.816689 26.978840 22.532046 175 176
177

60 H 18.558289 50.777813 39.220634 9.820624 26.870461 20.754666 178 179
180

61 H 19.242672 48.058902 41.087589 10.182783 25.431676 21.742616 181 182
183

62 H 27.007327 51.214497 38.027665 14.291662 27.101545 20.123374 184 185
186

63 H 26.255203 54.131115 36.458292 13.893655 28.644952 19.292897 187 188
189

64 H 29.141946 53.809821 38.152196 15.421253 28.474931 20.189273 190 191
192

65 H 24.471538 57.918566 47.464598 12.949780 30.649185 25.117184 193 194
195

66 H 22.618537 57.371373 52.962464 11.969214 30.359623 28.026529 196 197
198

67 H 25.775141 56.816424 51.843464 13.639617 30.065957 27.434380 199 200
201

68 H 23.260149 54.864232 50.789262 12.308741 29.032901 26.876520 202 203
204

69 C	31.530716	57.545103	51.289281	16.685336	30.451557	27.141118	205	206	207
70 H	27.571327	69.203778	55.247479	14.590118	36.621062	29.235707	208	209	210
71 H	26.615852	68.618978	58.436900	14.084502	36.311599	30.923476	211	212	213
72 H	24.390559	68.291203	55.918468	12.906928	36.138148	29.590779	214	215	216
73 H	25.839681	64.034978	57.344208	13.673770	33.885851	30.345248	217	218	219
74 H	31.111542	65.955577	55.826396	16.463519	34.902188	29.542056	220	221	222
75 H	30.192214	65.071309	58.934894	15.977031	34.434254	31.187003	223	224	225
76 H	35.432588	61.268726	53.638079	18.750118	32.422013	28.384049	226	227	228
77 H	34.836374	64.357808	54.872663	18.434615	34.056685	29.037363	229	230	231
78 H	37.087463	62.375518	56.375106	19.625840	33.007702	29.832421	232	233	234
79 H	33.953546	57.412481	56.489728	17.967443	30.381377	29.893077	235	236	237
80 H	32.445342	58.232946	59.403111	17.169336	30.815548	31.434773	238	239	240
81 H	35.722807	58.857540	58.979621	18.903695	31.146069	31.210671	241	242	243
82 H	25.849266	60.800108	54.276913	13.678843	32.174031	28.722105	244	245	246
83 H	22.868335	62.203065	51.062647	12.101402	32.916445	27.021189	247	248	249
84 H	31.154864	62.124720	52.248313	16.486444	32.874986	27.648616	250	251	252
85 H	29.483303	60.666664	57.642516	15.601892	32.103416	30.503106	253	254	255
86 H	26.079428	53.186033	44.161494	13.800639	28.144836	23.369256	256	257	258
87 O	23.136730	53.782858	45.909508	12.243430	28.460663	24.294266	259	260	261

88 H 18.434846 55.190159 47.180615 9.755300 29.205374 24.966906 262 263 264

89 H 33.030122 53.889457 50.341058 17.478788 28.517073 26.639341 265 266 267

90 H 30.680769 53.806146 52.821533 16.235564 28.472986 27.951952 268 269 270

91 H 29.781663 54.308068 49.605544 15.759777 28.738592 26.250124 271 272 273

92 C 31.247956 54.698054 51.034558 16.535706 28.944964 27.006325 274 275 276

93 O 32.792874 58.871606 49.883113 17.353242 31.153513 26.397007 277 278 279

94 H 18.677343 59.512544 43.262791 9.883624 31.492682 22.893683 280 281 282

95 H 16.271160 59.057796 45.588446 8.610327 31.252040 24.124367 283 284 285

96 H 16.695812 56.790391 43.122890 8.835043 30.052181 22.819650 286 287 288

Number of elements of the density matrix on this node (used, total): 222269 1773786

Numerical Integration : Fuzzy Cells (Becke)

Becke grid quality: NORMAL

Lebedev angular grid order: min: 11 max: 23

Nr. of radial points: min: 46 max: 52

Total nr. of points: 600089

Nr. of blocks: 4689

Block length: 128

Nr. of dummy points: 103

Test of Precision of the Numerical Integration Grid

==

Integral of the Total Core Density: 85.99994613187907

Relative Error: -6.264E-07

Density Fitting (zlmFit)

ZlmFit Fit Quality: NORMAL

Max L-expansion: min: 6 max: 7

Nr. of radial interpolation points: min: 39 max: 48

S C F *** ScaSCF ***

Initial density matrix is taken from previous geometry

CYCLE 1

SCF Error: norm([F,P])=   0.278500915674, max([F,P])=   0.021043353771

orbitals (Q,E):

A :114...133   (2.00   -0.2560) (2.00   -0.2512) (2.00   -0.2398) (2.00   -0.2387)

(2.00   -0.2354) (2.00   -0.2289) (2.00   -0.2233) (2.00   -0.2210)

(2.00   -0.2000) (2.00   -0.1826) (0.00   -0.0761) (0.00   -0.0747)

(0.00   -0.0676) (0.00   -0.0440) (0.00   -0.0235) (0.00   -0.0186)

(0.00   -0.0176) (0.00   -0.0131) (0.00   -0.0098) (0.00   -0.0052)

CYCLE   2

SDIIS (wt 0.000): 0.0442 0.9558

A-DIIS (wt 1.000): 0.0000 1.0000

DIIS coefficients: 0.0000 1.0000

SCF Error: norm([F,P])=   0.052902267759, max([F,P])=   0.004119946190

orbitals (Q,E):

A :114...124   (2.00   -0.2470) (2.00   -0.2443) (2.00   -0.2374) (2.00   -0.2332)

(2.00   -0.2241) (2.00   -0.2177) (2.00   -0.2174) (2.00   -0.2122)

(2.00   -0.1978) (2.00   -0.1747) (0.00   -0.0715)

CYCLE   3

SDIIS (wt 0.000): 0.0021 0.6348 0.3631

A-DIIS (wt 1.000): 0.0000 0.7024 0.2976

DIIS coefficients: 0.0000 0.7024 0.2976

SCF Error: norm([F,P])=   0.089609379740, max([F,P])=   0.009207016378

orbitals (Q,E):

A :114...124   (2.00   -0.2489) (2.00   -0.2460) (2.00   -0.2364) (2.00   -0.2350)

(2.00   -0.2278) (2.00   -0.2212) (2.00   -0.2189) (2.00   -0.2144)

(2.00   -0.1960) (2.00   -0.1769) (0.00   -0.0711)

CYCLE 4

SDIIS (wt 0.987): 0.3761 0.1698 0.4541

A-DIIS (wt 0.013): 0.0043 0.0000 0.9957

DIIS coefficients: 0.3713 0.1676 0.4611

SCF Error: norm([F,P])= 0.020338352978, max([F,P])= 0.002267741280

orbitals (Q,E):

A :114...124 (2.00 -0.2499) (2.00 -0.2463) (2.00 -0.2364) (2.00 -0.2349)

(2.00 -0.2288) (2.00 -0.2220) (2.00 -0.2190) (2.00 -0.2154)

(2.00 -0.1970) (2.00 -0.1774) (0.00 -0.0714)

CYCLE 5

SDIIS (wt 0.993): 0.1557 0.0518 0.3551 0.4374

A-DIIS (wt 0.007): 0.0000 0.0000 0.2285 0.7715

DIIS coefficients: 0.1546 0.0514 0.3542 0.4397

SCF Error: norm([F,P])= 0.011092955871, max([F,P])= 0.001696925053

orbitals (Q,E):

A :114...124 (2.00 -0.2498) (2.00 -0.2463) (2.00 -0.2364) (2.00 -0.2350)

(2.00 -0.2286) (2.00 -0.2219) (2.00 -0.2190) (2.00 -0.2153)

(2.00 -0.1966) (2.00 -0.1773) (0.00 -0.0714)

CYCLE 6

SDIIS (wt 1.000): 0.0738 0.0197 0.2066 0.3113 0.3887

DIIS coefficients: 0.0738 0.0197 0.2066 0.3113 0.3887

SCF Error: norm([F,P])= 0.003675763572, max([F,P])= 0.000415974617

orbitals (Q,E):

A :114...124 (2.00 -0.2497) (2.00 -0.2463) (2.00 -0.2364) (2.00 -0.2350)

(2.00 -0.2287) (2.00 -0.2219) (2.00 -0.2190) (2.00 -0.2152)

(2.00 -0.1966) (2.00 -0.1773) (0.00 -0.0713)

CYCLE 7

SDIIS (wt 1.000): 0.0207 0.0030 0.0763 0.1356 0.2801 0.4842

DIIS coefficients: 0.0207 0.0030 0.0763 0.1356 0.2801 0.4842

SCF Error: norm([F,P])= 0.001332044706, max([F,P])= 0.000160594535

orbitals (Q,E):

A :114...124 (2.00 -0.2498) (2.00 -0.2463) (2.00 -0.2363) (2.00 -0.2350)

(2.00 -0.2287) (2.00 -0.2219) (2.00 -0.2190) (2.00 -0.2152)

(2.00 -0.1966) (2.00 -0.1773) (0.00 -0.0713)

CYCLE 8

SDIIS (wt 1.000): 0.0069 0.0001 0.0320 0.0628 0.1574 0.3448 0.3960

DIIS coefficients: 0.0069 0.0001 0.0320 0.0628 0.1574 0.3448 0.3960

SCF Error: norm([F,P])= 0.000543883901, max([F,P])= 0.000087237879

orbitals (Q,E):

A :114...124 (2.00 -0.2498) (2.00 -0.2463) (2.00 -0.2363) (2.00 -0.2350)

(2.00 -0.2287) (2.00 -0.2219) (2.00 -0.2190) (2.00 -0.2152)

(2.00 -0.1966) (2.00 -0.1773) (0.00 -0.0713)

CYCLE 9

SDIIS (wt 1.000): 0.0013 -0.0003 0.0091 0.0207 0.0615 0.1567 0.2642 0.4870

DIIS coefficients: 0.0013 -0.0003 0.0091 0.0207 0.0615 0.1567 0.2642 0.4870

SCF Error: norm([F,P])= 0.000167121147, max([F,P])= 0.000021928319

orbitals (Q,E):

A :114...124 (2.00 -0.2498) (2.00 -0.2463) (2.00 -0.2364) (2.00 -0.2350)

(2.00 -0.2287) (2.00 -0.2219) (2.00 -0.2190) (2.00 -0.2152)

(2.00 -0.1966) (2.00 -0.1773) (0.00 -0.0713)

CYCLE 10

SDIIS (wt 1.000): 0.0000 -0.0002 0.0022 0.0064 0.0223 0.0640 0.1305 0.3387 0.4362

DIIS coefficients: 0.0000 -0.0002 0.0022 0.0064 0.0223 0.0640 0.1305 0.3387 0.4362

SCF Error: norm([F,P])= 0.000068977088, max([F,P])= 0.000009432262

orbitals (Q,E):

A :114...124 (2.00 -0.2498) (2.00 -0.2463) (2.00 -0.2364) (2.00 -0.2350)

(2.00 -0.2287) (2.00 -0.2219) (2.00 -0.2190) (2.00 -0.2152)

(2.00 -0.1966) (2.00 -0.1773) (0.00 -0.0713)

CYCLE 11

SDIIS (wt 1.000): -0.0001 -0.0001 0.0004 0.0019 0.0081 0.0257 0.0593 0.1806 0.3111 0.4131

DIIS coefficients: -0.0001 -0.0001 0.0004 0.0019 0.0081 0.0257 0.0593 0.1806 0.3111 0.4131

SCF Error: norm([F,P])= 0.000026186212, max([F,P])= 0.000004043222

orbitals (Q,E):

A :114...124 (2.00 -0.2498) (2.00 -0.2463) (2.00 -0.2363) (2.00 -0.2350)

(2.00 -0.2287) (2.00 -0.2219) (2.00 -0.2190) (2.00 -0.2152)

(2.00 -0.1966) (2.00 -0.1773) (0.00 -0.0713)

CYCLE 12

SDIIS (wt 1.000): -0.0000 0.0003 0.0008 0.0032 0.0099 0.0235 0.0783 0.1555 0.3152 0.4133

DIIS coefficients: -0.0000 0.0003 0.0008 0.0032 0.0099 0.0235 0.0783 0.1555 0.3152 0.4133

SCF Error: norm([F,P])= 0.000011763284, max([F,P])= 0.000001458681

orbitals (Q,E):

A :114...124 (2.00 -0.2498) (2.00 -0.2463) (2.00 -0.2363) (2.00 -0.2350)

(2.00 -0.2287) (2.00 -0.2219) (2.00 -0.2190) (2.00 -0.2152)

(2.00 -0.1966) (2.00 -0.1773) (0.00 -0.0713)

CYCLE 13

SDIIS (wt 1.000): 0.0000 0.0002 0.0008 0.0026 0.0072 0.0282 0.0635 0.1570 0.2639 0.4765

DIIS coefficients: 0.0000 0.0002 0.0008 0.0026 0.0072 0.0282 0.0635 0.1570 0.2639 0.4765

SCF Error: norm([F,P])=   0.000003085914, max([F,P])=   0.000000344606

orbitals (Q,E):

A :114...124   (2.00   -0.2498) (2.00   -0.2463) (2.00   -0.2363) (2.00   -0.2350)

(2.00   -0.2287) (2.00   -0.2219) (2.00   -0.2190) (2.00   -0.2152)

(2.00   -0.1966) (2.00   -0.1773) (0.00   -0.0713)

SCF CONVERGED

CYCLE   14

*** Setting up for NEW gradients in Focky

*** Using FIT density in Focky

*** Setting up for NEW Hessian in Focky

orbitals (Q,E):

A :114...133   (2.00   -0.2498) (2.00   -0.2463) (2.00   -0.2363) (2.00   -0.2350)

(2.00   -0.2287) (2.00   -0.2219) (2.00   -0.2190) (2.00   -0.2152)

(2.00   -0.1966) (2.00   -0.1773) (0.00   -0.0713) (0.00   -0.0700)

(0.00   -0.0635) (0.00   -0.0414) (0.00   -0.0197) (0.00   -0.0144)

(0.00   -0.0135) (0.00   -0.0098) (0.00   -0.0058) (0.00   -0.0007)

Fit test: (difference of exact and fit density, squared integrated, result summed over spins)

SCF: 0.00099464983918

Gross Charges per Atom (Z minus electrons)

==

-0.6548   0.9115   0.5207   0.3289   -0.5799   -0.3832   -0.6333   0.6664   0.6170   0.8528

-0.6558   -0.0029   -0.5749   0.5814   0.3215   -0.5910   0.5831   0.5868   -0.5890   0.2473

0.3364	0.1032	0.1616	0.7295	0.4003	0.4007	0.5588	0.5598	0.0307	0.6287
-0.5631	0.5351	0.5372	0.5680	0.6332	0.3690	0.7360	0.9646	0.5698	-0.2035
-0.1765	-0.2034	-0.1868	-0.2082	-0.2368	-0.2266	-0.1439	-0.1867	-0.1404	-0.1977
-0.2369	-0.2145	-0.2226	-0.1775	-0.2334	-0.1663	-0.1883	-0.1499	-0.1943	-0.2093
-0.2162	-0.2113	-0.2234	-0.1897	-0.1198	-0.2178	-0.1510	-0.2109	0.8278	-0.1924
-0.2124	-0.2116	-0.2084	-0.1842	-0.2065	-0.1654	-0.2227	-0.2287	-0.1961	-0.2362
-0.2065	-0.2442	-0.1945	-0.1534	-0.1572	0.2446	-0.6712	-0.1210	-0.1755	-0.1878
-0.1111	0.4707	-0.6130	-0.1674	-0.2101	-0.2338				

Net Total: -0.00000000

M U L T I P O L E   D E R I V E D   C H A R G E   A N A L Y S I S

See: M. Swart, P.Th. van Duijnen, J.G. Snijders, J.Comput.Chem., (2001), p. 79-88.

Multipole derived atomic charges (a.u.)

Atom		Level: MDC-m	MDC-d	MDC-q
1	O	-0.355617	-0.347469	-0.371888
2	C	0.364724	0.375286	0.399571
3	C	0.243327	0.249358	0.255239
4	C	0.180154	0.162610	0.208278
5	O	-0.311152	-0.547881	-0.618307
6	N	-0.215974	-0.268674	-0.449599
7	O	-0.379468	-0.365691	-0.436050

8	C	0.251268	0.306554	0.305192
9	C	0.227032	0.190916	0.177152
10	C	0.341044	0.408909	0.415514
11	O	-0.384068	-0.350164	-0.389757
12	C	0.124922	0.099448	0.098341
13	O	-0.314032	-0.302002	-0.347690
14	C	0.245582	0.244293	0.249375
15	C	0.195476	0.156514	0.174566
16	O	-0.374332	-0.479290	-0.484481
17	C	0.227579	0.191556	0.191730
18	C	0.228492	0.189865	0.182280
19	O	-0.320435	-0.325059	-0.397678
20	C	0.056860	0.045089	-0.069586
21	C	0.195368	0.219615	0.285388
22	C	0.119762	0.102983	0.107707
23	C	0.119779	0.098050	0.107027
24	C	0.289140	0.263755	0.283661
25	C	0.171765	0.133763	0.136907
26	C	0.163930	0.121801	0.162723
27	C	0.248428	0.233998	0.257451
28	C	0.233205	0.191211	0.280685
29	C	0.033898	0.025564	-0.057174
30	C	0.235966	0.188796	0.173927
31	O	-0.374609	-0.475621	-0.523968
32	C	0.253871	0.242427	0.264441
33	C	0.225090	0.185170	0.157247
34	C	0.228692	0.294325	0.403664
35	C	0.220183	0.188624	0.198333
36	C	0.179886	0.143357	0.151465
37	C	0.293027	0.267771	0.309758
38	C	0.366675	0.336238	0.381410

39	C	0.228482	0.188024	0.150129
40	H	-0.072425	-0.060069	-0.019673
41	H	-0.056841	-0.029868	-0.015101
42	H	-0.061052	-0.050462	-0.014790
43	H	-0.055969	-0.035335	0.000594
44	H	-0.068538	-0.051207	-0.047386
45	H	-0.069249	-0.055156	-0.046511
46	H	-0.075845	-0.070739	-0.067665
47	H	-0.070168	-0.057979	-0.061308
48	H	-0.066062	-0.045645	-0.028435
49	H	-0.081243	-0.063400	-0.070323
50	H	-0.054933	-0.047712	-0.045992
51	H	-0.072254	-0.054315	-0.058334
52	H	-0.070843	-0.058890	-0.064506
53	H	-0.067335	-0.063554	-0.073316
54	H	-0.056645	-0.043523	-0.051443
55	H	-0.053594	-0.042091	-0.042972
56	H	-0.056753	-0.041440	-0.030014
57	H	-0.067401	-0.049384	-0.052555
58	H	-0.061101	-0.035384	-0.034681
59	H	-0.074602	-0.059355	-0.047583
60	H	-0.074227	-0.065457	-0.054152
61	H	-0.068626	-0.053014	-0.040802
62	H	-0.065841	-0.045510	-0.030303
63	H	-0.072237	-0.074909	-0.078497
64	H	-0.058372	-0.036232	-0.036414
65	H	-0.071154	-0.044462	-0.050469
66	H	-0.071154	-0.062950	-0.057096
67	H	-0.066772	-0.051882	-0.060344
68	H	-0.065510	-0.054183	-0.051981
69	C	0.352362	0.444161	0.478581

70	H	-0.063797	-0.052515	-0.074508
71	H	-0.067419	-0.065514	-0.091506
72	H	-0.063517	-0.047870	-0.067481
73	H	-0.079794	-0.062703	-0.073304
74	H	-0.072040	-0.059518	-0.066540
75	H	-0.069273	-0.068511	-0.071157
76	H	-0.072927	-0.042638	-0.032972
77	H	-0.066555	-0.054164	-0.043539
78	H	-0.064988	-0.060427	-0.032881
79	H	-0.083508	-0.063546	-0.049298
80	H	-0.066322	-0.057517	-0.053273
81	H	-0.067175	-0.064090	-0.054975
82	H	-0.069040	-0.041900	-0.035825
83	H	-0.061885	-0.022085	-0.019273
84	H	-0.043960	0.003964	-0.010479
85	H	-0.071766	-0.057623	-0.039028
86	H	0.064408	0.263716	0.310871
87	O	-0.365805	-0.442634	-0.460987
88	H	-0.044280	0.004052	0.025302
89	H	-0.045585	-0.029855	-0.030603
90	H	-0.052165	-0.051307	-0.058617
91	H	-0.040584	-0.009872	-0.023138
92	C	0.239204	0.194735	0.234248
93	O	-0.372194	-0.473730	-0.505351
94	H	-0.060424	-0.053028	-0.047486
95	H	-0.060631	-0.044976	-0.037818
96	H	-0.067513	-0.064519	-0.059895

*** Using NEW gradient routines ***

Geometry CYCLE 2

==============

Energy gradients wrt nuclear displacements

==

Atom	Cartesian (a.u./angstrom) X	Y	Z
1 O	0.005620	-0.001513	-0.007437
2 C	-0.004451	0.001321	0.004552
3 C	0.002685	-0.001583	-0.002599
4 C	0.001299	0.000210	0.001180
5 O	0.007331	-0.004380	-0.000710
6 N	-0.000363	0.004828	0.005035
7 O	-0.000799	0.005673	-0.002219
8 C	0.010150	-0.007398	-0.005087
9 C	0.000959	-0.000954	-0.000368
10 C	-0.008191	0.008176	-0.003934
11 O	0.000058	-0.004210	-0.003083
12 C	-0.000176	0.000416	0.000792
13 O	-0.001848	0.003243	-0.007649
14 C	-0.001244	-0.000232	0.001800
15 C	-0.001855	0.001386	-0.001638
16 O	-0.014942	0.009149	0.008770
17 C	-0.002942	0.000905	0.000107
18 C	-0.000088	-0.000699	0.000234
19 O	-0.004000	-0.003465	0.002718
20 C	-0.004798	-0.004729	-0.000960
21 C	-0.001267	-0.001899	0.000490
22 C	-0.000542	0.004275	-0.000904
23 C	0.001369	-0.000532	-0.002134

24	C	-0.002475	-0.003131	0.000474
25	C	-0.001914	0.001095	0.000288
26	C	-0.002062	-0.000135	-0.000406
27	C	-0.000596	-0.001505	-0.000579
28	C	0.001188	-0.001351	0.000015
29	C	0.004016	0.005375	-0.002549
30	C	0.002333	-0.000224	0.001539
31	O	0.012763	0.010082	-0.010971
32	C	-0.000186	0.000984	0.003166
33	C	0.001955	0.000139	0.000949
34	C	-0.008484	-0.006661	0.009264
35	C	0.000915	-0.001964	0.001288
36	C	0.000118	0.000674	0.000076
37	C	-0.000863	0.002288	-0.003206
38	C	0.000945	-0.003262	0.000553
39	C	0.001381	0.000725	-0.000374
40	H	-0.006079	0.004718	-0.000151
41	H	0.002351	0.000672	0.005261
42	H	0.000717	-0.005543	-0.000684
43	H	0.004098	0.002869	-0.002389
44	H	0.004457	0.004171	0.001554
45	H	-0.000366	0.000900	-0.006270
46	H	-0.005072	0.003629	0.002135
47	H	-0.004734	-0.002943	-0.001187
48	H	0.004854	0.003206	0.000402
49	H	0.001648	-0.004578	0.000775
50	H	0.004744	0.001464	0.002497
51	H	-0.003908	0.002791	0.003389
52	H	0.000857	0.005510	-0.004908
53	H	-0.000235	0.006089	-0.000621
54	H	-0.002396	-0.001049	0.006839

55 H -0.006113 -0.003467 -0.005026

56 H -0.002038 0.001269 -0.004863

57 H 0.002320 -0.003015 -0.004713

58 H 0.002158 -0.004057 0.004529

59 H -0.003579 0.001296 0.004875

60 H -0.003511 0.001199 -0.004854

61 H -0.000696 -0.006380 0.000787

62 H 0.000454 -0.006276 -0.000693

63 H -0.001907 0.002044 -0.005226

64 H 0.005955 0.001006 -0.000753

65 H 0.003649 -0.003013 -0.002682

66 H -0.003892 0.000074 0.006280

67 H 0.006271 -0.001602 0.000827

68 H -0.000400 -0.006152 -0.000675

69 C -0.006216 -0.008416 0.010911

70 H 0.003212 0.004441 -0.003514

71 H 0.000254 0.002201 0.005561

72 H -0.006137 0.001443 -0.001125

73 H -0.003335 -0.002839 0.002900

74 H 0.003849 0.003519 -0.003051

75 H 0.001191 0.001239 0.005863

76 H 0.001850 -0.001836 -0.005334

77 H -0.000942 0.006182 -0.001612

78 H 0.006407 0.000128 0.002946

79 H 0.001116 -0.005471 -0.002479

80 H -0.003589 -0.002172 0.004573

81 H 0.005813 -0.000209 0.003173

82 H -0.002235 -0.001982 0.003072

83 H -0.001927 0.002569 0.004571

84 H 0.004645 0.001351 -0.004246

85 H -0.002694 -0.003806 0.003547

86 H -0.005407 0.000555 0.006329

87 O 0.013659 -0.012050 0.005449

88 H -0.003256 -0.002171 0.002665

89 H 0.004975 -0.003427 -0.002598

90 H -0.001709 -0.003366 0.005318

91 H -0.003374 -0.002692 -0.004588

92 C -0.000261 0.002686 -0.000276

93 O 0.010856 0.011268 -0.013246

94 H 0.000779 0.006544 -0.004543

95 H -0.004303 0.003645 0.002974

96 H -0.003823 -0.003281 -0.004179

----

dE(predicted): -0.10921E-01, dE(actual): -0.40952E-02, Trust radius: 1.959592, all in a.u.

Max Cartesian displacement 1.12145004318810

Limit Cartesian step to 0.566917837497000

Geometry Convergence after Step 2 (Hartree/Angstrom,Angstrom)

----

current energy -19.67032862 Hartree

energy change -0.00409517 0.00100000 F

constrained gradient max 0.01494169 0.00100000 F

constrained gradient rms 0.00422113 0.00066667 F

gradient max 0.01494169

gradient rms 0.00422113

cart. step max 0.29619613 0.01000000 F

cart. step rms 0.09075228 0.00666667 F

Coordinates (Cartesian)

======================

Atom	bohr X	bohr Y	bohr Z	angstrom X	angstrom Y	angstrom Z	Geometric Variables (0:frozen, *:LT par.)		
1 O	26.727275	61.465928	50.458631	14.143465	32.526368	26.701558	1	2	3
2 C	27.092792	62.014968	53.023678	14.336888	32.816908	28.058922	4	5	6
3 C	29.834510	61.253303	53.649415	15.787743	32.413852	28.390048	7	8	9
4 C	30.521550	61.955357	56.409409	16.151309	32.785363	29.850573	10	11	12
5 O	27.184834	52.990532	42.375925	14.385595	28.041382	22.424374	13	14	15
6 N	33.137932	61.381643	57.173799	17.535839	32.481767	30.255072	16	17	18
7 O	26.740793	64.676995	53.428048	14.150618	34.225592	28.272905	19	20	21
8 C	20.650288	58.154783	48.538553	10.927662	30.774186	25.685496	22	23	24
9 C	25.603544	65.531889	41.664632	13.548812	34.677982	22.047974	25	26	27
10 C	21.723766	54.978617	44.883748	11.495722	29.093431	23.751457	28	29	30
11 O	21.751846	62.731253	45.757925	11.510581	33.195950	24.214051	31	32	33
12 C	19.673255	56.340250	46.389183	10.410638	29.813976	24.548098	34	35	36
13 O	21.400891	55.225674	42.375402	11.324864	29.224168	22.424097	37	38	39
14 C	24.130242	61.367968	49.555693	12.769174	32.474530	26.223744	40	41	42
15 C	24.092524	63.147470	47.158580	12.749214	33.416202	24.955246	43	44	45
16 O	19.075401	59.000065	49.983691	10.094268	31.221490	26.450230	46	47	48
17 C	24.184403	56.805099	51.256355	12.797835	30.059964	27.123695	49	50	51
18 C	26.902347	53.523006	37.903884	14.236109	28.323155	20.057872	52	53	54
19 O	29.988515	58.504992	53.352604	15.869238	30.959508	28.232982	55	56	57
20 C	26.064374	57.235711	40.759486	13.792673	30.287834	21.568991	58	59	60
21 C	25.788523	54.364913	40.477884	13.646699	28.768673	21.419974	61	62	63
22 C	23.471135	58.560975	49.006867	12.420390	30.989133	25.933317	64	65	66
23 C	25.838291	62.753339	42.473967	13.673035	33.207637	22.476255	67	68	69
24 C	35.108762	62.223891	55.403336	18.578757	32.927465	29.318183	70	71	72
25 C	26.298348	62.351855	45.355004	13.916486	32.995181	24.000835	73	74	75

26 C	29.856392	64.774024	56.853193	15.799322	34.276937	30.085414	76	77	78
27 C	27.127802	65.386553	56.060711	14.355414	34.601074	29.666051	79	80	81
28 C	26.472957	68.189766	56.262128	14.008886	36.084470	29.772636	82	83	84
29 C	28.167485	58.546248	41.297926	14.905591	30.981340	21.853921	85	86	87
30 C	17.626209	57.648542	44.775378	9.327388	30.506295	23.694109	88	89	90
31 O	29.777690	62.582367	40.154690	15.757675	33.117162	21.248947	91	92	93
32 C	22.892264	53.679015	40.513928	12.114065	28.405712	21.439047	94	95	96
33 C	30.740657	57.478725	41.950182	16.267255	30.416431	22.199080	97	98	99
34 C	28.058674	61.410000	41.151603	14.848011	32.496772	21.776491	100	101	102
35 C	24.426051	65.904268	48.035054	12.925709	34.875037	25.419056	103	104	105
36 C	22.273023	50.860415	40.796645	11.786376	26.914173	21.588655	106	107	108
37 C	33.547347	58.756866	57.976388	17.752491	31.092795	30.679783	109	110	111
38 C	19.630429	64.323634	46.376379	10.387976	34.038601	24.541323	112	113	114
39 C	19.417023	50.318698	40.918147	10.275046	26.627508	21.652951	115	116	117
40 H	24.351023	58.304421	40.362176	12.886006	30.853371	21.358744	118	119	120
41 H	31.234743	57.985820	43.905873	16.528714	30.684775	23.233987	121	122	123
42 H	30.806135	55.424958	41.792144	16.301904	29.329625	22.115450	124	125	126
43 H	32.169222	58.351176	40.727240	17.023219	30.878112	21.551928	127	128	129
44 H	27.242689	66.631995	42.293938	14.416210	35.260133	22.380988	130	131	132
45 H	25.509888	65.686044	39.600153	13.499251	34.759557	20.955498	133	134	135
46 H	23.884686	66.365760	42.461443	12.639231	35.119248	22.469628	136	137	138
47 H	24.083384	61.755753	41.996809	12.744378	32.679737	22.223754	139	140	141

48	H	28.002448	63.383437	45.920915	14.818257	33.541070	24.300302	142	143 144
49	H	26.728080	60.351804	45.653525	14.143891	31.936799	24.158805	145	146 147
50	H	26.217124	66.061660	49.050915	13.873504	34.958325	25.956626	148	149 150
51	H	22.932921	66.487851	49.343975	12.135579	35.183856	26.111707	151	152 153
52	H	24.456488	67.198151	46.422720	12.941816	35.559730	24.565846	154	155 156
53	H	20.000298	66.304358	45.880165	10.583702	35.086755	24.278738	157	158 159
54	H	19.098192	64.178767	48.377007	10.106328	33.961941	25.600010	160	161 162
55	H	18.061597	63.625555	45.228138	9.557786	33.669194	23.933700	163	164 165
56	H	22.065454	54.387572	38.751053	11.676535	28.780664	20.506174	166	167 168
57	H	23.088658	49.908554	39.143140	12.217991	26.410470	20.713658	169	170 171
58	H	23.228252	50.086568	42.457375	12.291862	26.504670	22.467475	172	173 174
59	H	18.557980	51.149563	42.611653	9.820460	27.067183	22.549116	175	176 177
60	H	18.420025	51.080313	39.264147	9.747458	27.030537	20.777692	178	179 180
61	H	19.081695	48.273665	40.983073	10.097598	25.545324	21.687308	181	182 183
62	H	26.748794	51.470467	37.680303	14.154852	27.236998	19.939557	184	185 186
63	H	25.941195	54.466383	36.329859	13.727489	28.822368	19.224934	187	188 189
64	H	28.903872	54.034864	37.837168	15.295270	28.594019	20.022567	190	191 192
65	H	24.574653	57.944731	47.374270	13.004346	30.663031	25.069384	193	194 195
66	H	23.111120	57.321048	52.952489	12.229878	30.332992	28.021250	196	197 198

67 H 26.208110 56.947916 51.652717 13.868735 30.135539 27.333441 199 200
201

68 H 23.760752 54.839272 50.767886 12.573648 29.019693 26.865209 202 203
204

69 C 31.340636 57.590536 51.330075 16.584750 30.475599 27.162706 205 206
207

70 H 27.716922 69.307072 55.036171 14.667163 36.675723 29.123887 208 209
210

71 H 26.705452 68.845150 58.215686 14.131916 36.431284 30.806414 211 212
213

72 H 24.510906 68.519991 55.682799 12.970613 36.259217 29.466068 214 215
216

73 H 25.802907 64.277042 57.240342 13.654311 34.013946 30.290284 217 218
219

74 H 31.135325 65.988863 55.759393 16.476105 34.919803 29.506600 220 221
222

75 H 30.146792 65.203604 58.861128 15.952995 34.504261 31.147968 223 224
225

76 H 35.199632 61.090222 53.648628 18.626843 32.327553 28.389631 226 227
228

77 H 34.808276 64.203243 54.875498 18.419747 33.974893 29.038863 229 230
231

78 H 36.950949 62.101651 56.349182 19.553600 32.862779 29.818703 232 233
234

79 H 33.527572 57.372766 56.408529 17.742027 30.360360 29.850108 235 236
237

80 H 32.087146 58.204650 59.340161 16.979786 30.800574 31.401461 238 239
240

81 H 35.389470 58.623721 58.924739 18.727301 31.022337 31.181629 241 242
243

82 H 25.759539 60.952563 54.234933 13.631361 32.254707 28.699890 244 245
246

83 H 22.861012 62.129383 51.007219 12.097526 32.877453 26.991858 247 248
249

84 H 31.091792 62.155744 52.279532 16.453068 32.891403 27.665137 250 251
252

85 H 29.298747 60.808844 57.638791 15.504229 32.178655 30.501135 253 254
255

86 H 26.209599 53.138944 43.948848 13.869523 28.119918 23.256729 256 257
258

87 O 23.438139 53.797925 45.893096 12.402929 28.468636 24.285580 259 260
261

88 H 18.768165 54.811553 47.477247 9.931685 29.005025 25.123877 262 263
264

89 H 32.709798 53.910557 50.357356 17.309280 28.528238 26.647965 265 266
267

90 H 30.399954 53.892305 52.860017 16.086963 28.518580 27.972316 268 269
270

91 H 29.472707 54.435100 49.671442 15.596285 28.805814 26.284995 271 272
273

92 C 30.968162 54.763753 51.073580 16.387646 28.979730 27.026975 274 275
276

93 O 32.613664 58.873412 49.935514 17.258408 31.154468 26.424736 277 278
279

94 H 18.462319 59.126037 43.595777 9.769838 31.288151 23.069892 280 281
282

95 H 16.222977 58.498067 46.034413 8.584830 30.955844 24.360362 283 284
285

96 H 16.684737 56.277027 43.545027 8.829182 29.780520 23.043036 286 287
288

Number of elements of the density matrix on this node (used, total): 222269 1773786

Numerical Integration : Fuzzy Cells (Becke)

Becke grid quality: NORMAL

Lebedev angular grid order: min: 11 max: 23

Nr. of radial points: min: 46 max: 52

Total nr. of points: 600207

Nr. of blocks: 4690

Block length: 128

Nr. of dummy points: 113

Test of Precision of the Numerical Integration Grid

==

Integral of the Total Core Density: 85.99993016803045

Relative Error: -8.120E-07

Density Fitting (zlmFit)

ZlmFit Fit Quality: NORMAL

Max L-expansion: min: 6 max: 7

Nr. of radial interpolation points: min: 39 max: 48

S C F *** ScaSCF ***

Initial density matrix is taken from previous geometry

CYCLE 1

SCF Error: norm([F,P])= 0.374131579055, max([F,P])= 0.031592321143

orbitals (Q,E):

A :114...133 (2.00 -0.2559) (2.00 -0.2533) (2.00 -0.2393) (2.00 -0.2335)

(2.00 -0.2304) (2.00 -0.2273) (2.00 -0.2230) (2.00 -0.2210)

(2.00 -0.1962) (2.00 -0.1775) (0.00 -0.0790) (0.00 -0.0691)

(0.00 -0.0675) (0.00 -0.0342) (0.00 -0.0231) (0.00 -0.0156)

(0.00 -0.0127) (0.00 -0.0097) (0.00 -0.0071) (0.00 -0.0037)

CYCLE 2

SDIIS (wt 0.000): 0.0845 0.9155

A-DIIS (wt 1.000): 0.0000 1.0000

DIIS coefficients: 0.0000 1.0000

SCF Error: norm([F,P])= 0.097722184703, max([F,P])= 0.009324693382

orbitals (Q,E):

A :114...124 (2.00 -0.2479) (2.00 -0.2434) (2.00 -0.2380) (2.00 -0.2332)

(2.00 -0.2291) (2.00 -0.2159) (2.00 -0.2116) (2.00 -0.2067)

(2.00 -0.1966) (2.00 -0.1773) (0.00 -0.0689)

CYCLE 3

SDIIS (wt 0.000): 0.0098 0.6693 0.3210

A-DIIS (wt 1.000): 0.0000 0.7434 0.2566

DIIS coefficients: 0.0000 0.7434 0.2566

SCF Error: norm([F,P])= 0.195664986353, max([F,P])= 0.021074895773

orbitals (Q,E):

A :114...124 (2.00 -0.2487) (2.00 -0.2443) (2.00 -0.2403) (2.00 -0.2346)

(2.00 -0.2288) (2.00 -0.2184) (2.00 -0.2170) (2.00 -0.2121)

(2.00 -0.1950) (2.00 -0.1773) (0.00 -0.0677)

CYCLE 4

SDIIS (wt 0.958): 0.4130 0.1383 0.4487

A-DIIS (wt 0.042): 0.1376 0.0000 0.8624

DIIS coefficients: 0.4012 0.1325 0.4663

SCF Error: norm([F,P])= 0.043693040389, max([F,P])= 0.005207197292

orbitals (Q,E):

A :114...124 (2.00 -0.2496) (2.00 -0.2470) (2.00 -0.2373) (2.00 -0.2347)

(2.00 -0.2289) (2.00 -0.2199) (2.00 -0.2185) (2.00 -0.2156)

(2.00 -0.1948) (2.00 -0.1771) (0.00 -0.0704)

CYCLE 5

SDIIS (wt 0.979): 0.1839 0.0453 0.3133 0.4575

A-DIIS (wt 0.021): 0.0000 0.0000 0.0008 0.9992

DIIS coefficients: 0.1801 0.0444 0.3067 0.4688

SCF Error: norm([F,P])= 0.015160521295, max([F,P])= 0.003076333268

orbitals (Q,E):

A :114...124 (2.00 -0.2496) (2.00 -0.2467) (2.00 -0.2375) (2.00 -0.2348)

(2.00 -0.2288) (2.00 -0.2192) (2.00 -0.2186) (2.00 -0.2150)

(2.00 -0.1947) (2.00 -0.1769) (0.00 -0.0698)

CYCLE 6

SDIIS (wt 1.000): 0.0830 0.0165 0.1680 0.3443 0.3882

DIIS coefficients: 0.0830 0.0165 0.1680 0.3443 0.3882

SCF Error: norm([F,P])= 0.006521459980, max([F,P])= 0.000898801403

orbitals (Q,E):

A :114...124   (2.00   -0.2495) (2.00   -0.2467) (2.00   -0.2376) (2.00   -0.2348)
(2.00   -0.2288) (2.00   -0.2194) (2.00   -0.2186) (2.00   -0.2150)
(2.00   -0.1948) (2.00   -0.1770) (0.00   -0.0698)

CYCLE   7

SDIIS (wt 1.000): 0.0278 0.0040 0.0651 0.1609 0.2543 0.4879

DIIS coefficients: 0.0278 0.0040 0.0651 0.1609 0.2543 0.4879

SCF Error: norm([F,P])=   0.001751640648, max([F,P])=   0.000248623410

orbitals (Q,E):

A :114...124   (2.00   -0.2495) (2.00   -0.2467) (2.00   -0.2376) (2.00   -0.2348)
(2.00   -0.2288) (2.00   -0.2194) (2.00   -0.2186) (2.00   -0.2151)
(2.00   -0.1947) (2.00   -0.1770) (0.00   -0.0698)

CYCLE   8

SDIIS (wt 1.000): 0.0091 0.0006 0.0255 0.0741 0.1399 0.3765 0.3743

DIIS coefficients: 0.0091 0.0006 0.0255 0.0741 0.1399 0.3765 0.3743

SCF Error: norm([F,P])=   0.000981378604, max([F,P])=   0.000139859348

orbitals (Q,E):

A :114...124   (2.00   -0.2496) (2.00   -0.2467) (2.00   -0.2376) (2.00   -0.2348)
(2.00   -0.2288) (2.00   -0.2194) (2.00   -0.2186) (2.00   -0.2151)
(2.00   -0.1948) (2.00   -0.1770) (0.00   -0.0698)

CYCLE   9

SDIIS (wt 1.000): 0.0019 -0.0001 0.0069 0.0244 0.0522 0.1694 0.2477 0.4977

DIIS coefficients: 0.0019 -0.0001 0.0069 0.0244 0.0522 0.1694 0.2477 0.4977

SCF Error: norm([F,P])=   0.000256016358, max([F,P])=   0.000023877073

orbitals (Q,E):

A :114...124   (2.00   -0.2496) (2.00   -0.2467) (2.00   -0.2376) (2.00   -0.2348)

(2.00 -0.2288) (2.00 -0.2194) (2.00 -0.2186) (2.00 -0.2151)

(2.00 -0.1948) (2.00 -0.1770) (0.00 -0.0698)

CYCLE 10

SDIIS (wt 1.000): 0.0002 -0.0001 0.0017 0.0081 0.0196 0.0731 0.1276 0.3430 0.4267

DIIS coefficients: 0.0002 -0.0001 0.0017 0.0081 0.0196 0.0731 0.1276 0.3430 0.4267

SCF Error: norm([F,P])= 0.000105192567, max([F,P])= 0.000014544509

orbitals (Q,E):

A :114...124 (2.00 -0.2496) (2.00 -0.2467) (2.00 -0.2376) (2.00 -0.2348)

(2.00 -0.2288) (2.00 -0.2194) (2.00 -0.2186) (2.00 -0.2151)

(2.00 -0.1948) (2.00 -0.1770) (0.00 -0.0698)

CYCLE 11

SDIIS (wt 1.000): -0.0001 -0.0001 0.0002 0.0021 0.0063 0.0270 0.0546 0.1722 0.3030 0.4348

DIIS coefficients: -0.0001 -0.0001 0.0002 0.0021 0.0063 0.0270 0.0546 0.1722 0.3030 0.4348

SCF Error: norm([F,P])= 0.000040197799, max([F,P])= 0.000008469889

orbitals (Q,E):

A :114...124 (2.00 -0.2496) (2.00 -0.2467) (2.00 -0.2376) (2.00 -0.2348)

(2.00 -0.2288) (2.00 -0.2194) (2.00 -0.2186) (2.00 -0.2151)

(2.00 -0.1948) (2.00 -0.1770) (0.00 -0.0698)

CYCLE 12

SDIIS (wt 1.000): -0.0000 0.0001 0.0009 0.0025 0.0102 0.0213 0.0724 0.1480 0.2988 0.4458

DIIS coefficients: -0.0000 0.0001 0.0009 0.0025 0.0102 0.0213 0.0724 0.1480 0.2988 0.4458

SCF Error: norm([F,P])= 0.000014556587, max([F,P])= 0.000003035601

orbitals (Q,E):

A :114...124 (2.00 -0.2496) (2.00 -0.2467) (2.00 -0.2376) (2.00 -0.2348)

(2.00 -0.2288) (2.00 -0.2194) (2.00 -0.2186) (2.00 -0.2151)

(2.00   -0.1948) (2.00   -0.1770) (0.00   -0.0698)

CYCLE   13

SDIIS (wt 1.000): 0.0000 0.0002 0.0007 0.0030 0.0069 0.0266 0.0625 0.1480 0.2939 0.4582

DIIS coefficients: 0.0000 0.0002 0.0007 0.0030 0.0069 0.0266 0.0625 0.1480 0.2939 0.4582

SCF Error: norm([F,P])=   0.000004664163, max([F,P])=   0.000000583302

orbitals (Q,E):

A :114...124   (2.00   -0.2496) (2.00   -0.2467) (2.00   -0.2376) (2.00   -0.2348)

(2.00   -0.2288) (2.00   -0.2194) (2.00   -0.2186) (2.00   -0.2151)

(2.00   -0.1948) (2.00   -0.1770) (0.00   -0.0698)

SCF CONVERGED

CYCLE   14

*** Setting up for NEW gradients in Focky

*** Using FIT density in Focky

*** Setting up for NEW Hessian in Focky

orbitals (Q,E):

A :114...133   (2.00   -0.2496) (2.00   -0.2467) (2.00   -0.2376) (2.00   -0.2348)

(2.00   -0.2288) (2.00   -0.2194) (2.00   -0.2186) (2.00   -0.2151)

(2.00   -0.1948) (2.00   -0.1770) (0.00   -0.0698) (0.00   -0.0669)

(0.00   -0.0603) (0.00   -0.0387) (0.00   -0.0187) (0.00   -0.0134)

(0.00   -0.0112) (0.00   -0.0081) (0.00   -0.0050) (0.00   -0.0006)

Fit test: (difference of exact and fit density, squared integrated, result summed over spins)

SCF: 0.00100851009696

Gross Charges per Atom (Z minus electrons)

==

-0.6595	0.9311	0.5336	0.3339	-0.5824	-0.3839	-0.6366	0.6692	0.6306	0.8714
-0.6572	-0.0083	-0.5782	0.5871	0.3248	-0.5824	0.5898	0.5991	-0.5989	0.2576
0.3424	0.0863	0.1621	0.7435	0.4057	0.4039	0.5624	0.5724	0.0300	0.6368
-0.5669	0.5453	0.5450	0.5771	0.6486	0.3711	0.7514	0.9794	0.5883	-0.2130
-0.1876	-0.1992	-0.1934	-0.2135	-0.2463	-0.2277	-0.1510	-0.1899	-0.1371	-0.2057
-0.2403	-0.2215	-0.2087	-0.1946	-0.2541	-0.1701	-0.1945	-0.1584	-0.1957	-0.2131
-0.2238	-0.2169	-0.2277	-0.1954	-0.1214	-0.2315	-0.0990	-0.2285	0.8429	-0.1966
-0.2175	-0.2166	-0.2106	-0.1904	-0.2123	-0.1667	-0.2290	-0.2370	-0.1947	-0.2428
-0.2155	-0.2528	-0.1985	-0.1556	-0.1615	0.2448	-0.6745	-0.1262	-0.1923	-0.1942
-0.1183	0.4863	-0.6141	-0.1751	-0.2159	-0.2313				

Net Total: 0.00000000

M U L T I P O L E D E R I V E D C H A R G E A N A L Y S I S

See: M. Swart, P.Th. van Duijnen, J.G. Snijders, J.Comput.Chem., (2001), p. 79-88.

Multipole derived atomic charges (a.u.)

Atom		Level: MDC-m	MDC-d	MDC-q
1	O	-0.350797	-0.338866	-0.363449
2	C	0.362909	0.374372	0.398492
3	C	0.241910	0.248938	0.255405
4	C	0.179713	0.163908	0.209736

5	O	-0.306234	-0.547546	-0.617023
6	N	-0.214971	-0.280280	-0.468064
7	O	-0.377103	-0.360101	-0.432637
8	C	0.249620	0.309947	0.312415
9	C	0.228071	0.193402	0.187040
10	C	0.338406	0.410635	0.414405
11	O	-0.384254	-0.359891	-0.396356
12	C	0.123683	0.101074	0.100335
13	O	-0.313085	-0.298881	-0.347426
14	C	0.244147	0.242863	0.245927
15	C	0.194476	0.159780	0.173936
16	O	-0.367010	-0.477192	-0.486221
17	C	0.227784	0.193473	0.198309
18	C	0.229723	0.192580	0.193094
19	O	-0.314221	-0.312317	-0.389853
20	C	0.058742	0.048970	-0.061271
21	C	0.195201	0.220209	0.282051
22	C	0.118916	0.103101	0.107144
23	C	0.118957	0.098910	0.106806
24	C	0.289485	0.266495	0.287203
25	C	0.171812	0.136294	0.139280
26	C	0.164835	0.123512	0.165617
27	C	0.249070	0.234296	0.256512
28	C	0.234370	0.193313	0.289042
29	C	0.033917	0.027898	-0.054190
30	C	0.236463	0.190885	0.180688
31	O	-0.370562	-0.477186	-0.524097
32	C	0.253509	0.241394	0.261976
33	C	0.224279	0.187345	0.170939
34	C	0.226480	0.296274	0.400313
35	C	0.220146	0.191315	0.200572

36	C	0.180675	0.146154	0.154827
37	C	0.293230	0.270350	0.314771
38	C	0.369629	0.340633	0.391704
39	C	0.229577	0.190296	0.155221
40	H	-0.073185	-0.062083	-0.022097
41	H	-0.058488	-0.032561	-0.020176
42	H	-0.060873	-0.050710	-0.018485
43	H	-0.057766	-0.038809	-0.005390
44	H	-0.069015	-0.052637	-0.049841
45	H	-0.070346	-0.058060	-0.051879
46	H	-0.076434	-0.070310	-0.068935
47	H	-0.071034	-0.059312	-0.060007
48	H	-0.066153	-0.047298	-0.029241
49	H	-0.081997	-0.063702	-0.071550
50	H	-0.055282	-0.051239	-0.048308
51	H	-0.073333	-0.055380	-0.059071
52	H	-0.072013	-0.060203	-0.065836
53	H	-0.066749	-0.060546	-0.075439
54	H	-0.061235	-0.051055	-0.056142
55	H	-0.053570	-0.044860	-0.048354
56	H	-0.057358	-0.041944	-0.029829
57	H	-0.066961	-0.049740	-0.051860
58	H	-0.062416	-0.038462	-0.037961
59	H	-0.075344	-0.060132	-0.049840
60	H	-0.074123	-0.064225	-0.054105
61	H	-0.069325	-0.056013	-0.043894
62	H	-0.066474	-0.047236	-0.034110
63	H	-0.072794	-0.075822	-0.082944
64	H	-0.058550	-0.036771	-0.038515
65	H	-0.071717	-0.044962	-0.050935
66	H	-0.071776	-0.063058	-0.057028

67	H	-0.068425	-0.052847	-0.062523
68	H	-0.067672	-0.059025	-0.057006
69	C	0.349562	0.446052	0.481871
70	H	-0.064331	-0.053393	-0.076804
71	H	-0.067814	-0.066745	-0.094538
72	H	-0.063701	-0.048640	-0.069901
73	H	-0.080355	-0.064013	-0.073967
74	H	-0.072446	-0.060195	-0.067613
75	H	-0.069205	-0.068380	-0.070671
76	H	-0.073568	-0.041499	-0.032711
77	H	-0.066903	-0.054759	-0.042805
78	H	-0.065337	-0.061322	-0.030832
79	H	-0.084105	-0.063949	-0.050389
80	H	-0.066651	-0.058192	-0.052488
81	H	-0.068035	-0.066422	-0.056546
82	H	-0.069519	-0.042527	-0.036165
83	H	-0.062388	-0.024033	-0.021397
84	H	-0.044949	0.002538	-0.013369
85	H	-0.072629	-0.058946	-0.038562
86	H	0.062288	0.263454	0.309159
87	O	-0.359269	-0.439580	-0.460082
88	H	-0.045076	0.001997	0.024110
89	H	-0.046494	-0.032878	-0.029157
90	H	-0.052396	-0.051437	-0.055634
91	H	-0.041166	-0.011268	-0.022481
92	C	0.239767	0.196866	0.225320
93	O	-0.364405	-0.471435	-0.502948
94	H	-0.062062	-0.055513	-0.050445
95	H	-0.061732	-0.049181	-0.042080
96	H	-0.068172	-0.063957	-0.060744

*** Using NEW gradient routines ***

Geometry CYCLE 3

==============

Energy gradients wrt nuclear displacements

==

Atom	Cartesian (a.u./angstrom)		
	X	Y	Z
1 O	0.003675	-0.003966	-0.005692
2 C	-0.007955	0.001143	0.002538
3 C	0.005358	-0.000265	-0.005753
4 C	0.001360	0.000388	0.004971
5 O	0.003476	0.003614	0.004342
6 N	0.000199	0.000528	-0.000684
7 O	-0.000052	0.002494	-0.002861
8 C	-0.008049	0.009483	0.006674
9 C	-0.000101	0.001132	0.000570
10 C	0.006633	-0.012500	0.003142
11 O	0.002402	0.002421	-0.006550
12 C	0.000267	-0.000134	0.000985
13 O	-0.001960	0.004205	0.003787
14 C	-0.002086	-0.000799	0.005352
15 C	-0.003110	0.001667	-0.000183
16 O	0.009807	-0.012728	-0.004750
17 C	-0.002032	0.002987	0.002243
18 C	-0.000436	-0.000472	-0.000126
19 O	-0.002527	-0.004578	-0.002610
20 C	0.005708	0.005311	0.004355

21	C	0.001154	-0.001091	-0.002570
22	C	-0.000174	-0.001237	0.000143
23	C	0.000080	0.000320	-0.000168
24	C	-0.002806	-0.001165	0.000110
25	C	-0.000140	-0.002660	0.002771
26	C	-0.001788	0.002039	0.000574
27	C	0.000414	-0.000989	-0.000296
28	C	0.000547	-0.000430	-0.000240
29	C	-0.005795	-0.001154	-0.004163
30	C	0.001337	-0.001885	-0.002185
31	O	-0.010502	-0.007240	0.006768
32	C	-0.002544	-0.002168	-0.001345
33	C	-0.001053	-0.000079	0.003275
34	C	0.009729	0.005244	-0.010158
35	C	-0.002344	-0.001443	0.000545
36	C	0.001240	-0.001821	0.001037
37	C	-0.002623	0.002940	-0.002227
38	C	0.000608	0.006620	0.001636
39	C	0.000453	0.002408	-0.000127
40	H	-0.000414	0.001812	0.002720
41	H	0.000477	-0.000925	0.001161
42	H	-0.000381	-0.002450	0.000379
43	H	-0.000006	0.000270	-0.000737
44	H	0.000747	0.000475	0.000312
45	H	0.000026	0.000068	-0.000852
46	H	-0.001416	0.000620	0.001613
47	H	-0.001376	-0.001730	-0.000924
48	H	0.000734	0.001015	0.000273
49	H	0.000241	-0.002026	0.001005
50	H	0.001981	-0.000774	0.001718
51	H	-0.001567	0.000426	0.000196

52	H	-0.000022	0.000983	-0.002233
53	H	0.001828	0.000790	-0.000987
54	H	0.000620	0.000631	-0.000914
55	H	0.000694	0.000733	0.000274
56	H	-0.000803	0.000024	-0.000895
57	H	0.000061	0.000080	-0.001740
58	H	-0.000388	-0.000993	-0.000115
59	H	-0.000315	0.000269	0.001398
60	H	-0.000755	0.000091	-0.000995
61	H	0.000348	-0.001238	0.000205
62	H	0.000324	-0.000846	0.000001
63	H	-0.000492	0.000462	-0.000661
64	H	0.000623	0.000101	-0.000533
65	H	0.002501	-0.001130	-0.000729
66	H	-0.000875	0.000283	0.001150
67	H	0.009808	-0.000257	-0.000314
68	H	0.000948	-0.001055	-0.000461
69	C	0.007390	0.010945	-0.005108
70	H	0.000593	0.000719	-0.000606
71	H	0.000010	0.000262	0.001023
72	H	-0.001260	0.000321	-0.000101
73	H	-0.000722	-0.000278	0.000438
74	H	0.000728	0.000368	-0.000637
75	H	0.000614	-0.000246	0.000915
76	H	-0.000585	-0.000721	-0.001535
77	H	-0.000378	0.000889	-0.000267
78	H	0.001222	-0.000143	0.000722
79	H	-0.000343	-0.001647	-0.002179
80	H	-0.000884	-0.000185	0.000878
81	H	0.001205	-0.000183	0.000392
82	H	0.000399	-0.000896	0.000394

83 H 0.001808 0.001218 0.001821

84 H 0.001789 -0.000835 -0.001043

85 H 0.000017 -0.001709 0.000104

86 H -0.000231 -0.001210 -0.001074

87 O -0.006230 0.010944 -0.006236

88 H -0.001174 -0.000893 -0.000325

89 H 0.000856 -0.000493 -0.000613

90 H -0.000125 -0.000356 0.001082

91 H -0.000964 -0.000194 -0.001253

92 C -0.001866 0.002729 0.000571

93 O -0.010138 -0.009044 0.011023

94 H -0.000249 0.000291 -0.000923

95 H -0.000417 -0.000433 0.000509

96 H -0.000584 -0.001067 -0.000419

----

dE(predicted): -0.86425E-02, dE(actual): 0.30890E-03, Trust radius: 0.195959, all in a.u.

Interpolation step length 0.195959179422654

Max Cartesian displacement 0.762803986470342

Limit Cartesian step to 0.566917837497000

Geometry Convergence after Step 3 (Hartree/Angstrom,Angstrom)

----

current energy -19.67001972 Hartree

energy change 0.00030890 0.00100000 T

constrained gradient max 0.01272801 0.00100000 F

constrained gradient rms 0.00314584 0.00066667 F

gradient max 0.01272801

gradient rms 0.00314584

cart. step max 0.30037625 0.01000000 F

cart. step rms 0.07423254 0.00666667 F

Coordinates (Cartesian)

======================

Atom	bohr X	bohr Y	bohr Z	angstrom X	angstrom Y	angstrom Z	Geometric Variables (0:frozen, *:LT par.)		
1 O	26.748739	61.403803	50.501927	14.154823	32.493493	26.724469	1	2	3
2 C	27.120187	61.917681	53.077159	14.351385	32.765426	28.087223	4	5	6
3 C	29.902244	61.281899	53.697758	15.823586	32.428984	28.415630	7	8	9
4 C	30.556282	61.977700	56.470524	16.169688	32.797186	29.882915	10	11	12
5 O	27.046874	52.862188	42.362448	14.312589	27.973465	22.417242	13	14	15
6 N	33.199274	61.549656	57.257257	17.568299	32.570675	30.299235	16	17	18
7 O	26.650070	64.554003	53.524725	14.102610	34.160507	28.324064	19	20	21
8 C	20.597683	58.246906	48.467077	10.899824	30.822935	25.647672	22	23	24
9 C	25.659371	65.433451	41.629454	13.578354	34.625891	22.029358	25	26	27
10 C	21.587908	55.043444	44.814602	11.423829	29.127736	23.714866	28	29	30
11 O	21.777768	62.607865	45.793966	11.524298	33.130655	24.233123	31	32	33
12 C	19.574583	56.501449	46.284033	10.358423	29.899279	24.492455	34	35	36
13 O	21.340220	55.307371	42.295965	11.292758	29.267400	22.382061	37	38	39
14 C	24.153673	61.299477	49.569005	12.781573	32.438286	26.230788	40	41	42
15 C	24.131441	63.065575	47.164324	12.769808	33.372865	24.958286	43	44	45
16 O	19.032566	59.169300	49.893543	10.071600	31.311045	26.402526	46	47	48
17 C	24.022106	56.694388	51.214002	12.711951	30.001378	27.101283	49	50	51
18 C	26.842136	53.415494	37.894669	14.204246	28.266262	20.052995	52	53	54
19 O	30.230767	58.553070	53.360346	15.997433	30.984950	28.237079	55	56	57
20 C	26.075716	57.153227	40.737403	13.798675	30.244185	21.557305	58	59	60
21 C	25.722630	54.290778	40.454874	13.611830	28.729443	21.407797	61	62	63

22 C	23.426165	58.516717	48.984801	12.396593	30.965713	25.921640	64	65	66
23 C	25.883377	62.661101	42.461516	13.696893	33.158827	22.469667	67	68	69
24 C	35.140937	62.528432	55.522138	18.595783	33.088621	29.381050	70	71	72
25 C	26.332469	62.274007	45.347772	13.934543	32.953985	23.997008	73	74	75
26 C	29.750746	64.751581	56.953349	15.743417	34.265061	30.138415	76	77	78
27 C	26.998691	65.244954	56.166180	14.287092	34.526143	29.721862	79	80	81
28 C	26.211336	68.011051	56.405369	13.870442	35.989898	29.848436	82	83	84
29 C	28.197571	58.440828	41.295947	14.921512	30.925554	21.852874	85	86	87
30 C	17.629120	57.933215	44.648270	9.328929	30.656937	23.626847	88	89	90
31 O	29.842126	62.476658	40.155269	15.791773	33.061224	21.249253	91	92	93
32 C	22.804611	53.708603	40.456138	12.067680	28.421369	21.408466	94	95	96
33 C	30.764132	57.363368	41.958110	16.279677	30.355387	22.203276	97	98	99
34 C	28.106111	61.307255	41.151769	14.873114	32.442402	21.776578	100	101	102
35 C	24.468368	65.828941	48.023395	12.948103	34.835175	25.412886	103	104	105
36 C	22.080472	50.913722	40.728596	11.684483	26.942381	21.552645	106	107	108
37 C	33.767209	58.947776	58.045414	17.868838	31.193820	30.716310	109	110	111
38 C	19.614818	64.142985	46.412762	10.379715	33.943006	24.560576	112	113	114
39 C	19.206390	50.463027	40.805085	10.163584	26.703884	21.593121	115	116	117
40 H	24.387897	58.252924	40.315140	12.905519	30.826120	21.333853	118	119	120
41 H	31.249753	57.869126	43.916157	16.536657	30.623022	23.239430	121	122	123
42 H	30.825009	55.310606	41.797962	16.311893	29.269112	22.118529	124	125	126
43 H	32.202435	58.232603	40.743861	17.040795	30.815367	21.560722	127	128	129
44 H	27.298660	66.534759	42.256479	14.445829	35.208678	22.361166	130	131	132

45	H	25.575080	65.570799	39.563250	13.533750	34.698572	20.935970	133	134	135
46	H	23.939745	66.279426	42.409729	12.668368	35.073562	22.442262	136	137	138
47	H	24.129218	61.665517	41.982354	12.768632	32.631986	22.216105	139	140	141
48	H	28.036181	63.309940	45.905992	14.836108	33.502177	24.292405	142	143	144
49	H	26.764976	60.276548	45.653349	14.163415	31.896975	24.158712	145	146	147
50	H	26.265056	65.996135	49.025265	13.898869	34.923650	25.943053	148	149	150
51	H	22.984355	66.418374	49.339718	12.162797	35.147090	26.109454	151	152	153
52	H	24.486549	67.115218	46.405933	12.957724	35.515844	24.556962	154	155	156
53	H	19.922138	66.132466	45.909560	10.542341	34.995794	24.294293	157	158	159
54	H	19.092003	63.989887	48.416521	10.103053	33.861990	25.620920	160	161	162
55	H	18.061568	63.395478	45.272672	9.557770	33.547442	23.957266	163	164	165
56	H	22.031929	54.449768	38.682346	11.658795	28.813576	20.469816	166	167	168
57	H	22.888886	49.932620	39.089054	12.112277	26.423205	20.685036	169	170	171
58	H	22.984785	50.111025	42.404825	12.163025	26.517612	22.439667	172	173	174
59	H	18.346470	51.322961	42.483183	9.708534	27.158941	22.481132	175	176	177
60	H	18.261735	51.255239	39.135001	9.663694	27.123104	20.709351	178	179	180
61	H	18.802921	48.430301	40.865194	9.950077	25.628212	21.624930	181	182	183
62	H	26.624023	51.369251	37.665745	14.088826	27.183437	19.931854	184	185	186
63	H	25.934197	54.391398	36.308939	13.723786	28.782688	19.213863	187	188	189

64	H	28.860347	53.861672	37.856739	15.272238	28.502369	20.032924	190	191	192
65	H	24.524657	57.880836	47.357494	12.977890	30.629219	25.060507	193	194	195
66	H	22.937391	57.225866	52.897711	12.137945	30.282624	27.992263	196	197	198
67	H	26.034795	56.744790	51.655530	13.777020	30.028050	27.334929	199	200	201
68	H	23.524377	54.757068	50.682702	12.448564	28.976193	26.820131	202	203	204
69	C	31.644990	57.748813	51.325553	16.745808	30.559356	27.160313	205	206	207
70	H	27.402505	69.202877	55.197370	14.500781	36.620585	29.209190	208	209	210
71	H	26.409819	68.650025	58.368014	13.975474	36.328029	30.887023	211	212	213
72	H	24.236894	68.255708	55.826349	12.825612	36.119365	29.542032	214	215	216
73	H	25.726358	64.057477	57.327572	13.613802	33.897757	30.336444	217	218	219
74	H	30.970563	66.043185	55.880317	16.388916	34.948548	29.570590	220	221	222
75	H	30.015284	65.166604	58.967947	15.883404	34.484682	31.204494	223	224	225
76	H	35.327795	61.420887	53.758992	18.694664	32.502534	28.448033	226	227	228
77	H	34.728911	64.491327	55.008176	18.377748	34.127340	29.109073	229	230	231
78	H	36.973257	62.508985	56.493957	19.565405	33.078330	29.895315	232	233	234
79	H	33.855703	57.577680	56.469450	17.915666	30.468796	29.882346	235	236	237
80	H	32.330188	58.291552	59.387244	17.108399	30.846561	31.426376	238	239	240
81	H	35.602519	58.925879	59.015448	18.840042	31.182232	31.229630	241	242	243
82	H	25.828501	60.785304	54.270418	13.667854	32.166198	28.718668	244	245	246

83 H 22.871214 62.068964 51.004797 12.102925 32.845481 26.990576 247 248
249

84 H 31.107290 62.274516 52.343875 16.461269 32.954255 27.699186 250 251
252

85 H 29.392748 60.751789 57.680572 15.553972 32.148462 30.523244 253 254
255

86 H 26.060392 53.042789 43.926781 13.790566 28.069035 23.245051 256 257
258

87 O 23.212386 53.757797 45.866879 12.283466 28.447401 24.271707 259 260
261

88 H 18.571895 55.012865 47.341395 9.827824 29.111554 25.051987 262 263
264

89 H 33.274922 54.181549 50.327649 17.608330 28.671641 26.632245 265 266
267

90 H 30.967583 53.978627 52.823869 16.387339 28.564259 27.953187 268 269
270

91 H 30.010172 54.479170 49.638486 15.880699 28.829135 26.267555 271 272
273

92 C 31.475656 54.903024 51.046007 16.656200 29.053429 27.012384 274 275
276

93 O 32.838495 59.134119 49.937207 17.377383 31.292428 26.425632 277 278
279

94 H 18.565810 59.375737 43.501301 9.824604 31.420287 23.019897 280 281
282

95 H 16.249195 58.844557 45.890210 8.598704 31.139198 24.284054 283 284
285

96 H 16.639215 56.627738 43.384806 8.805093 29.966108 22.958251 286 287
288

Number of elements of the density matrix on this node (used, total): 222269 1773786

Numerical Integration : Fuzzy Cells (Becke)

Becke grid quality: NORMAL

Lebedev angular grid order: min: 11 max: 23

Nr. of radial points: min: 46 max: 52

Total nr. of points: 600219

Nr. of blocks: 4690

Block length: 128

Nr. of dummy points: 101

Test of Precision of the Numerical Integration Grid

===

Integral of the Total Core Density: 85.99999854299764

Relative Error: -1.694E-08

Density Fitting (zlmFit)

ZlmFit Fit Quality: NORMAL

Max L-expansion: min: 6 max: 7

Nr. of radial interpolation points: min: 39 max: 48

S C F *** ScaSCF ***

Initial density matrix is taken from previous geometry

CYCLE   1

SCF Error: norm([F,P])=   0.260724923305, max([F,P])=   0.021069801681

orbitals (Q,E):

A :114...133   (2.00   -0.2614) (2.00   -0.2603) (2.00   -0.2551) (2.00   -0.2467)

(2.00   -0.2396) (2.00   -0.2309) (2.00   -0.2306) (2.00   -0.2264)

(2.00   -0.2053) (2.00   -0.1917) (0.00   -0.0812) (0.00   -0.0797)

(0.00   -0.0763) (0.00   -0.0591) (0.00   -0.0290) (0.00   -0.0227)

(0.00   -0.0213) (0.00   -0.0199) (0.00   -0.0143) (0.00   -0.0093)

CYCLE   2

SDIIS (wt 0.000): 0.1083 0.8917

A-DIIS (wt 1.000): 0.0000 1.0000

DIIS coefficients: 0.0000 1.0000

SCF Error: norm([F,P])=   0.079222351883, max([F,P])=   0.005710826430

orbitals (Q,E):

A :114...124   (2.00   -0.2444) (2.00   -0.2377) (2.00   -0.2313) (2.00   -0.2282)

(2.00   -0.2247) (2.00   -0.2188) (2.00   -0.2150) (2.00   -0.2115)

(2.00   -0.1960) (2.00   -0.1689) (0.00   -0.0660)

CYCLE   3

SDIIS (wt 0.000): 0.0109 0.6641 0.3250

A-DIIS (wt 1.000): 0.0000 0.7340 0.2660

DIIS coefficients: 0.0000 0.7340 0.2660

SCF Error: norm([F,P])=   0.155574910208, max([F,P])=   0.012265580701

orbitals (Q,E):

A :114...124   (2.00   -0.2479) (2.00   -0.2448) (2.00   -0.2347) (2.00   -0.2317)

(2.00   -0.2267) (2.00   -0.2199) (2.00   -0.2184) (2.00   -0.2136)

(2.00   -0.1941) (2.00   -0.1751) (0.00   -0.0681)

CYCLE   4

SDIIS (wt 0.954): 0.4362 0.1732 0.3906

A-DIIS (wt 0.046): 0.1307 0.0000 0.8693

DIIS coefficients: 0.4220 0.1651 0.4128

SCF Error: norm([F,P])=   0.038671887692, max([F,P])=   0.005596055626

orbitals (Q,E):

A :114...124   (2.00   -0.2492) (2.00   -0.2468) (2.00   -0.2371) (2.00   -0.2356)

(2.00   -0.2280) (2.00   -0.2204) (2.00   -0.2197) (2.00   -0.2151)

(2.00   -0.1963) (2.00   -0.1774) (0.00   -0.0695)

CYCLE   5

SDIIS (wt 0.995): 0.1921 0.0641 0.2587 0.4851

A-DIIS (wt 0.005): 0.0000 0.0000 0.0000 1.0000

DIIS coefficients: 0.1911 0.0637 0.2573 0.4879

SCF Error: norm([F,P])=   0.010954547880, max([F,P])=   0.001533903715

orbitals (Q,E):

A :114...124   (2.00   -0.2491) (2.00   -0.2468) (2.00   -0.2368) (2.00   -0.2357)

(2.00   -0.2281) (2.00   -0.2206) (2.00   -0.2197) (2.00   -0.2152)

(2.00   -0.1958) (2.00   -0.1770) (0.00   -0.0696)

CYCLE   6

SDIIS (wt 1.000): 0.0795 0.0241 0.1232 0.3378 0.4353

DIIS coefficients: 0.0795 0.0241 0.1232 0.3378 0.4353

SCF Error: norm([F,P])= 0.004346459554, max([F,P])= 0.000464660497

orbitals (Q,E):

A :114...124 (2.00 -0.2492) (2.00 -0.2468) (2.00 -0.2368) (2.00 -0.2357)

(2.00 -0.2280) (2.00 -0.2205) (2.00 -0.2197) (2.00 -0.2151)

(2.00 -0.1959) (2.00 -0.1772) (0.00 -0.0695)

CYCLE 7

SDIIS (wt 1.000): 0.0294 0.0085 0.0478 0.1635 0.2847 0.4660

DIIS coefficients: 0.0294 0.0085 0.0478 0.1635 0.2847 0.4660

SCF Error: norm([F,P])= 0.001320515418, max([F,P])= 0.000221108849

orbitals (Q,E):

A :114...124 (2.00 -0.2492) (2.00 -0.2468) (2.00 -0.2368) (2.00 -0.2357)

(2.00 -0.2281) (2.00 -0.2205) (2.00 -0.2197) (2.00 -0.2151)

(2.00 -0.1959) (2.00 -0.1772) (0.00 -0.0695)

CYCLE 8

SDIIS (wt 1.000): 0.0089 0.0024 0.0158 0.0693 0.1452 0.3402 0.4183

DIIS coefficients: 0.0089 0.0024 0.0158 0.0693 0.1452 0.3402 0.4183

SCF Error: norm([F,P])= 0.000599828865, max([F,P])= 0.000097550021

orbitals (Q,E):

A :114...124 (2.00 -0.2492) (2.00 -0.2468) (2.00 -0.2369) (2.00 -0.2357)

(2.00 -0.2281) (2.00 -0.2205) (2.00 -0.2197) (2.00 -0.2151)

(2.00 -0.1959) (2.00 -0.1772) (0.00 -0.0695)

CYCLE 9

SDIIS (wt 1.000): 0.0021 0.0005 0.0044 0.0243 0.0592 0.1672 0.2951 0.4472

DIIS coefficients: 0.0021 0.0005 0.0044 0.0243 0.0592 0.1672 0.2951 0.4472

SCF Error: norm([F,P])= 0.000210452370, max([F,P])= 0.000039339907

orbitals (Q,E):

A :114...124 (2.00 -0.2492) (2.00 -0.2468) (2.00 -0.2369) (2.00 -0.2357)

(2.00 -0.2281) (2.00 -0.2205) (2.00 -0.2197) (2.00 -0.2151)

(2.00 -0.1959) (2.00 -0.1772) (0.00 -0.0695)

CYCLE 10

SDIIS (wt 1.000): 0.0002 -0.0000 0.0009 0.0071 0.0207 0.0687 0.1504 0.3123 0.4396

DIIS coefficients: 0.0002 -0.0000 0.0009 0.0071 0.0207 0.0687 0.1504 0.3123 0.4396

SCF Error: norm([F,P])= 0.000079850982, max([F,P])= 0.000013017745

orbitals (Q,E):

A :114...124 (2.00 -0.2492) (2.00 -0.2468) (2.00 -0.2369) (2.00 -0.2357)

(2.00 -0.2281) (2.00 -0.2205) (2.00 -0.2197) (2.00 -0.2151)

(2.00 -0.1959) (2.00 -0.1772) (0.00 -0.0695)

CYCLE 11

SDIIS (wt 1.000): -0.0001 -0.0001 0.0002 0.0020 0.0075 0.0288 0.0735 0.1711 0.3309 0.3861

DIIS coefficients: -0.0001 -0.0001 0.0002 0.0020 0.0075 0.0288 0.0735 0.1711 0.3309 0.3861

SCF Error: norm([F,P])= 0.000035761842, max([F,P])= 0.000006215616

orbitals (Q,E):

A :114...124 (2.00 -0.2492) (2.00 -0.2468) (2.00 -0.2369) (2.00 -0.2357)

(2.00 -0.2281) (2.00 -0.2205) (2.00 -0.2197) (2.00 -0.2151)

(2.00 -0.1959) (2.00 -0.1772) (0.00 -0.0695)

CYCLE 12

SDIIS (wt 1.000): -0.0000 0.0001 0.0007 0.0025 0.0099 0.0279 0.0683 0.1584 0.2682 0.4640

DIIS coefficients: -0.0000 0.0001 0.0007 0.0025 0.0099 0.0279 0.0683 0.1584 0.2682 0.4640

SCF Error: norm([F,P])= 0.000011659372, max([F,P])= 0.000002010238

orbitals (Q,E):

```
A :114...124    ( 2.00   -0.2492)  ( 2.00   -0.2468)  ( 2.00   -0.2369)  ( 2.00   -0.2357)
                ( 2.00   -0.2281)  ( 2.00   -0.2205)  ( 2.00   -0.2197)  ( 2.00   -0.2151)
                ( 2.00   -0.1959)  ( 2.00   -0.1772)  ( 0.00   -0.0695)
```

CYCLE 13

SDIIS (wt 1.000): 0.0000 0.0000 0.0005 0.0025 0.0088 0.0237 0.0653 0.1333 0.3055 0.4604

DIIS coefficients: 0.0000 0.0000 0.0005 0.0025 0.0088 0.0237 0.0653 0.1333 0.3055 0.4604

SCF Error: norm([F,P])= 0.000003789375, max([F,P])= 0.000000474595

orbitals (Q,E):

```
A :114...124    ( 2.00   -0.2492)  ( 2.00   -0.2468)  ( 2.00   -0.2369)  ( 2.00   -0.2357)
                ( 2.00   -0.2281)  ( 2.00   -0.2205)  ( 2.00   -0.2197)  ( 2.00   -0.2151)
                ( 2.00   -0.1959)  ( 2.00   -0.1772)  ( 0.00   -0.0695)
```

SCF CONVERGED

CYCLE 14

*** Setting up for NEW gradients in Focky

*** Using FIT density in Focky

*** Setting up for NEW Hessian in Focky

orbitals (Q,E):

```
A :114...133    ( 2.00   -0.2492)  ( 2.00   -0.2468)  ( 2.00   -0.2369)  ( 2.00   -0.2357)
                ( 2.00   -0.2281)  ( 2.00   -0.2205)  ( 2.00   -0.2197)  ( 2.00   -0.2151)
                ( 2.00   -0.1959)  ( 2.00   -0.1772)  ( 0.00   -0.0695)  ( 0.00   -0.0687)
                ( 0.00   -0.0612)  ( 0.00   -0.0398)  ( 0.00   -0.0192)  ( 0.00   -0.0134)
                ( 0.00   -0.0122)  ( 0.00   -0.0091)  ( 0.00   -0.0052)  ( 0.00   -0.0005)
```

Fit test: (difference of exact and fit density, squared integrated, result summed over spins)

SCF: 0.00100500832241

Gross Charges per Atom (Z minus electrons)

==

-0.6600	0.9236	0.5321	0.3310	-0.5796	-0.3839	-0.6354	0.6718	0.6304	0.8630
-0.6601	-0.0029	-0.5777	0.5849	0.3266	-0.5873	0.6011	0.5993	-0.5926	0.2582
0.3402	0.0976	0.1642	0.7443	0.4075	0.4064	0.5631	0.5717	0.0304	0.6393
-0.5656	0.5453	0.5463	0.5718	0.6515	0.3708	0.7514	0.9825	0.5880	-0.2102
-0.1881	-0.2034	-0.1911	-0.2127	-0.2445	-0.2303	-0.1479	-0.1872	-0.1441	-0.2065
-0.2403	-0.2217	-0.2183	-0.1908	-0.2449	-0.1698	-0.1949	-0.1561	-0.1962	-0.2134
-0.2237	-0.2160	-0.2272	-0.1955	-0.1190	-0.2253	-0.1521	-0.2181	0.8350	-0.1963
-0.2173	-0.2163	-0.2110	-0.1893	-0.2115	-0.1685	-0.2290	-0.2353	-0.1954	-0.2427
-0.2144	-0.2516	-0.1953	-0.1566	-0.1610	0.2436	-0.6729	-0.1251	-0.1817	-0.1910
-0.1240	0.4844	-0.6122	-0.1718	-0.2154	-0.2372				

Net Total: -0.00000000

M U L T I P O L E   D E R I V E D   C H A R G E   A N A L Y S I S

See: M. Swart, P.Th. van Duijnen, J.G. Snijders, J.Comput.Chem., (2001), p. 79-88.

Multipole derived atomic charges (a.u.)

Atom Level: MDC-m MDC-d MDC-q

1	O	-0.354537	-0.346339	-0.370382
2	C	0.364142	0.375584	0.399459
3	C	0.242508	0.250319	0.255609
4	C	0.180139	0.164838	0.210497
5	O	-0.305680	-0.548665	-0.620054
6	N	-0.214780	-0.277094	-0.464963
7	O	-0.377959	-0.361833	-0.433660
8	C	0.251221	0.310139	0.310572
9	C	0.228009	0.193121	0.185065
10	C	0.339682	0.410092	0.415071
11	O	-0.383701	-0.352864	-0.390986
12	C	0.124607	0.102077	0.102326
13	O	-0.313784	-0.299353	-0.348132
14	C	0.245557	0.245190	0.249572
15	C	0.195427	0.158102	0.174538
16	O	-0.371480	-0.479933	-0.487778
17	C	0.229524	0.194815	0.195766
18	C	0.229929	0.192733	0.193589
19	O	-0.318698	-0.322467	-0.396574
20	C	0.059026	0.047692	-0.060188
21	C	0.195439	0.221108	0.282800
22	C	0.119525	0.104564	0.109112
23	C	0.119365	0.099179	0.108132
24	C	0.289651	0.266747	0.287368
25	C	0.172105	0.135586	0.138696
26	C	0.164745	0.123624	0.165408
27	C	0.248875	0.234838	0.257609
28	C	0.234292	0.193312	0.285840
29	C	0.033878	0.026793	-0.054972
30	C	0.236647	0.190780	0.179901

31	O	-0.372468	-0.476695	-0.521579
32	C	0.253973	0.242101	0.261849
33	C	0.225266	0.187545	0.165817
34	C	0.227309	0.295184	0.395864
35	C	0.221009	0.191020	0.200767
36	C	0.180576	0.145857	0.154640
37	C	0.293490	0.270411	0.313401
38	C	0.369137	0.339810	0.387494
39	C	0.229714	0.190349	0.155182
40	H	-0.073145	-0.060697	-0.021998
41	H	-0.058809	-0.032823	-0.019082
42	H	-0.061477	-0.052057	-0.018492
43	H	-0.056291	-0.035875	-0.001487
44	H	-0.068667	-0.051990	-0.049081
45	H	-0.070186	-0.057474	-0.051010
46	H	-0.076508	-0.070985	-0.069278
47	H	-0.071270	-0.059224	-0.059874
48	H	-0.065642	-0.046017	-0.027954
49	H	-0.082285	-0.064194	-0.071954
50	H	-0.055054	-0.048638	-0.046466
51	H	-0.072700	-0.055224	-0.059616
52	H	-0.072224	-0.061270	-0.067034
53	H	-0.066929	-0.061917	-0.074193
54	H	-0.059557	-0.047980	-0.053977
55	H	-0.054017	-0.044471	-0.046733
56	H	-0.057401	-0.042125	-0.029730
57	H	-0.067277	-0.050152	-0.052031
58	H	-0.062159	-0.037653	-0.036623
59	H	-0.075278	-0.060088	-0.049991
60	H	-0.074312	-0.064676	-0.054520
61	H	-0.069131	-0.055529	-0.043523

62	H	-0.066095	-0.046680	-0.033246
63	H	-0.072616	-0.075542	-0.082781
64	H	-0.058414	-0.036540	-0.038414
65	H	-0.071957	-0.045882	-0.052511
66	H	-0.071612	-0.062940	-0.055925
67	H	-0.067783	-0.053769	-0.063213
68	H	-0.065839	-0.056498	-0.054035
69	C	0.351113	0.446563	0.478201
70	H	-0.064531	-0.053780	-0.076445
71	H	-0.067880	-0.066785	-0.093824
72	H	-0.063798	-0.048804	-0.069141
73	H	-0.080439	-0.063903	-0.073950
74	H	-0.072599	-0.060206	-0.066857
75	H	-0.069503	-0.069081	-0.071054
76	H	-0.073868	-0.042005	-0.032845
77	H	-0.066848	-0.054548	-0.042819
78	H	-0.065388	-0.061153	-0.031812
79	H	-0.084055	-0.063429	-0.049739
80	H	-0.066689	-0.058157	-0.052611
81	H	-0.067866	-0.066051	-0.055508
82	H	-0.069869	-0.043602	-0.036504
83	H	-0.061692	-0.022143	-0.019187
84	H	-0.045533	0.002948	-0.011530
85	H	-0.072620	-0.058872	-0.039481
86	H	0.064278	0.265854	0.312881
87	O	-0.364093	-0.442677	-0.461440
88	H	-0.044959	0.001612	0.023356
89	H	-0.046306	-0.031680	-0.032461
90	H	-0.052333	-0.051349	-0.059182
91	H	-0.041938	-0.012901	-0.027099
92	C	0.239941	0.197055	0.240043

93	O	-0.368349	-0.473798	-0.505215
94	H	-0.061360	-0.054800	-0.050314
95	H	-0.061401	-0.047974	-0.041182
96	H	-0.068459	-0.065688	-0.062184

*** Using NEW gradient routines ***

Geometry CYCLE 4

==============

Energy gradients wrt nuclear displacements

==

Atom		Cartesian (a.u./angstrom)		
		X	Y	Z
1	O	0.001586	0.003749	-0.002211
2	C	-0.002186	0.001746	0.002452
3	C	0.002676	0.001521	-0.002268
4	C	0.001010	-0.001500	0.002510
5	O	0.003453	0.000256	0.000534
6	N	0.000879	0.001980	0.002685
7	O	-0.000115	0.000656	-0.001648
8	C	0.001343	0.003910	-0.000122
9	C	-0.000238	0.000600	0.000252
10	C	-0.002093	-0.003385	-0.000597
11	O	0.000539	-0.000451	-0.000656
12	C	0.000041	-0.000849	0.000361
13	O	-0.001522	0.002145	0.000195
14	C	-0.005369	-0.002813	0.001903
15	C	-0.002179	-0.000237	-0.001523

16	O	-0.000512	-0.004120	0.003216
17	C	0.000755	0.000551	0.001303
18	C	-0.000271	-0.000274	0.000138
19	O	0.000386	-0.001725	0.002617
20	C	-0.000397	0.001441	0.002407
21	C	0.000416	-0.002126	-0.001833
22	C	-0.001853	-0.000543	-0.002955
23	C	0.000175	-0.000759	-0.000386
24	C	-0.001208	-0.001852	0.000035
25	C	0.000074	-0.001276	0.000860
26	C	-0.001236	0.001559	-0.000053
27	C	0.000956	-0.000826	-0.000112
28	C	0.000205	-0.000057	-0.000260
29	C	-0.000127	0.000708	-0.001617
30	C	0.000808	0.000111	-0.000663
31	O	0.001204	0.000690	-0.000678
32	C	-0.000612	-0.000064	-0.001246
33	C	0.001089	0.000414	0.002211
34	C	-0.000185	-0.000292	-0.003290
35	C	0.001623	-0.000994	0.001596
36	C	-0.000005	-0.001245	0.000846
37	C	-0.000921	0.001901	-0.002116
38	C	-0.000043	0.003069	-0.000649
39	C	0.000574	0.001512	-0.000028
40	H	-0.001085	0.001272	0.001915
41	H	0.000186	-0.000848	0.001082
42	H	0.000447	-0.000461	0.000575
43	H	0.000454	0.000516	-0.000632
44	H	0.000838	0.000518	0.000281
45	H	-0.000010	0.000081	-0.000929
46	H	-0.000771	0.000419	0.000889

47	H	-0.000692	-0.001252	-0.000985
48	H	0.000777	0.001041	-0.000550
49	H	-0.000043	-0.000997	0.000647
50	H	0.001028	0.000069	0.000811
51	H	-0.000736	0.000549	0.000287
52	H	0.000266	0.000707	-0.001267
53	H	0.000123	0.000540	-0.000730
54	H	0.000042	0.000612	0.000260
55	H	-0.000739	-0.000829	-0.000988
56	H	-0.000218	0.000364	-0.000927
57	H	-0.000110	0.000115	-0.001499
58	H	-0.000063	-0.000980	0.000259
59	H	-0.000369	0.000213	0.001138
60	H	-0.000641	0.000141	-0.000886
61	H	0.000006	-0.001260	0.000218
62	H	0.000283	-0.001077	-0.000082
63	H	-0.000524	0.000427	-0.000663
64	H	0.000783	0.000153	-0.000617
65	H	0.001916	-0.000933	-0.000178
66	H	-0.000775	-0.000100	0.000802
67	H	-0.001080	-0.001356	-0.000911
68	H	0.000105	-0.001371	-0.000443
69	C	0.000293	0.000193	0.001742
70	H	0.000431	0.000653	-0.000535
71	H	0.000007	0.000300	0.000933
72	H	-0.001024	0.000183	-0.000068
73	H	-0.000710	-0.000401	0.000340
74	H	0.000512	0.000482	-0.000639
75	H	0.000335	-0.000086	0.000947
76	H	0.000100	-0.000435	-0.001106
77	H	-0.000273	0.001198	-0.000273

78 H 0.001110 -0.000059 0.000535

79 H 0.000103 -0.001194 -0.000932

80 H -0.000588 -0.000311 0.000826

81 H 0.001147 -0.000235 0.000403

82 H -0.001007 0.000224 -0.000285

83 H 0.000734 0.000360 0.001738

84 H 0.001313 -0.001285 -0.000855

85 H 0.000037 -0.001567 -0.000037

86 H -0.001088 -0.000510 -0.000309

87 O 0.001971 0.002698 0.002433

88 H -0.000981 -0.000741 -0.000053

89 H 0.000914 -0.000630 -0.000454

90 H -0.000188 -0.000628 0.000857

91 H -0.000125 -0.000570 -0.000936

92 C -0.000704 0.000421 -0.000329

93 O 0.000902 0.001311 -0.001270

94 H -0.000078 0.001682 -0.000655

95 H -0.000723 0.000305 0.000449

96 H -0.000540 -0.000761 -0.000550

----

dE(predicted): -0.34639E-02, dE(actual): -0.31180E-02, Trust radius: 0.195959, all in a.u.

Geometry Convergence after Step 4 (Hartree/Angstrom,Angstrom)

----

current energy -19.67313773 Hartree

energy change -0.00311802 0.00100000 F

constrained gradient max 0.00536903 0.00100000 F

constrained gradient rms 0.00122270 0.00066667 F

gradient max 0.00536903

gradient rms 0.00122270

cart. step max 0.21569618 0.01000000 F

cart. step rms 0.05852781 0.00666667 F

Coordinates (Cartesian)

======================

Atom	bohr X	Y	Z	angstrom X	Y	Z	Geometric Variables (0:frozen, *:LT par.)		
1 O	26.757858	61.464604	50.463552	14.159649	32.525668	26.704162	1	2	3
2 C	27.103893	61.880265	53.055261	14.342762	32.745626	28.075635	4	5	6
3 C	29.886896	61.264691	53.657159	15.815465	32.419879	28.394146	7	8	9
4 C	30.514460	61.835706	56.459395	16.147557	32.722046	29.877025	10	11	12
5 O	27.082089	52.907628	42.557563	14.331224	27.997511	22.520492	13	14	15
6 N	33.152754	61.389886	57.243638	17.543682	32.486129	30.292029	16	17	18
7 O	26.591665	64.485313	53.613945	14.071703	34.124158	28.371278	19	20	21
8 C	20.598773	58.370552	48.422426	10.900401	30.888366	25.624044	22	23	24
9 C	25.660830	65.428785	41.531640	13.579127	34.623422	21.977598	25	26	27
10 C	21.573547	55.046689	44.884071	11.416229	29.129453	23.751627	28	29	30
11 O	21.796437	62.685990	45.756400	11.534178	33.171998	24.213244	31	32	33
12 C	19.570255	56.575012	46.286969	10.356133	29.938207	24.494009	34	35	36
13 O	21.362794	55.262430	42.360354	11.304704	29.243618	22.416134	37	38	39
14 C	24.172532	61.394229	49.516028	12.791553	32.488427	26.202754	40	41	42
15 C	24.159030	63.143879	47.105503	12.784408	33.414302	24.927159	43	44	45
16 O	19.039778	59.387574	49.780976	10.075417	31.426551	26.342958	46	47	48
17 C	23.991617	56.835692	51.224448	12.695817	30.076153	27.106810	49	50	51
18 C	26.920519	53.297023	38.073953	14.245725	28.203570	20.147868	52	53	54
19 O	30.255540	58.564726	53.180643	16.010542	30.991118	28.141985	55	56	57

20 C	26.089791	57.120415	40.762169	13.806123	30.226822	21.570411	58	59	60
21 C	25.765557	54.250127	40.586657	13.634545	28.707931	21.477534	61	62	63
22 C	23.424067	58.616242	48.958804	12.395482	31.018379	25.907883	64	65	66
23 C	25.883249	62.672805	42.407096	13.696826	33.165020	22.440869	67	68	69
24 C	35.101120	62.467314	55.578580	18.574713	33.056279	29.410918	70	71	72
25 C	26.348651	62.326833	45.292177	13.943106	32.981940	23.967588	73	74	75
26 C	29.678140	64.574335	57.060302	15.704995	34.171266	30.195011	76	77	78
27 C	26.924802	65.068476	56.282186	14.247991	34.432755	29.783250	79	80	81
28 C	26.104258	67.810354	56.632895	13.813779	35.883694	29.968837	82	83	84
29 C	28.195603	58.432708	41.300926	14.920471	30.921257	21.855509	85	86	87
30 C	17.669967	57.981645	44.583790	9.350544	30.682565	23.592726	88	89	90
31 O	29.830611	62.446207	40.104290	15.785680	33.045109	21.222276	91	92	93
32 C	22.858773	53.631463	40.580547	12.096342	28.380548	21.474300	94	95	96
33 C	30.767911	57.375970	41.956810	16.281677	30.362056	22.202588	97	98	99
34 C	28.096005	61.294666	41.110657	14.867766	32.435741	21.754823	100	101	102
35 C	24.492643	65.911392	47.942500	12.960948	34.878806	25.370079	103	104	105
36 C	22.167479	50.842471	40.945877	11.730525	26.904677	21.667625	106	107	108
37 C	33.733123	58.761394	57.921613	17.850800	31.095191	30.650798	109	110	111
38 C	19.634795	64.182439	46.457997	10.390286	33.963884	24.584513	112	113	114
39 C	19.299613	50.362416	40.989265	10.212915	26.650643	21.690585	115	116	117
40 H	24.406044	58.189231	40.264115	12.915122	30.792415	21.306852	118	119	120
41 H	31.267705	57.919069	43.899105	16.546157	30.649452	23.230406	121	122	123
42 H	30.832540	55.322635	41.831168	16.315878	29.275478	22.136101	124	125	126
43 H	32.190862	58.227138	40.715353	17.034670	30.812475	21.545637	127	128	129

44	H	27.302262	66.535955	42.134879	14.447735	35.209311	22.296818	130	131	132
45	H	25.570174	65.531407	39.465920	13.531153	34.677727	20.884465	133	134	135
46	H	23.946096	66.287880	42.302096	12.671728	35.078035	22.385305	136	137	138
47	H	24.125457	61.675928	41.954305	12.766642	32.637495	22.201262	139	140	141
48	H	28.056000	63.362358	45.832029	14.846596	33.529916	24.253265	142	143	144
49	H	26.776300	60.333635	45.619292	14.169408	31.927185	24.140689	145	146	147
50	H	26.294026	66.090090	48.929536	13.914199	34.973370	25.892395	148	149	150
51	H	23.017042	66.501667	49.264728	12.180094	35.191167	26.069771	151	152	153
52	H	24.493524	67.184736	46.318145	12.961415	35.552631	24.510507	154	155	156
53	H	19.925634	66.189030	46.026390	10.544191	35.025726	24.356117	157	158	159
54	H	19.141779	63.951475	48.459576	10.129393	33.841663	25.643703	160	161	162
55	H	18.071511	63.470821	45.314358	9.563032	33.587312	23.979325	163	164	165
56	H	22.092837	54.303669	38.778901	11.691026	28.736264	20.520910	166	167	168
57	H	23.017085	49.809126	39.363029	12.180117	26.357855	20.830018	169	170	171
58	H	23.052079	50.121891	42.666880	12.198635	26.523362	22.578340	172	173	174
59	H	18.402284	51.279845	42.613715	9.738069	27.136126	22.550207	175	176	177
60	H	18.380625	51.079317	39.274166	9.726608	27.030011	20.782994	178	179	180
61	H	18.914456	48.331998	41.121405	10.009099	25.576192	21.760510	181	182	183
62	H	26.720190	51.244677	37.914393	14.139716	27.117515	20.063433	184	185	186

63 H	26.024351	54.209536	36.447051	13.771493	28.686451	19.286949	187	188	189
64 H	28.933301	53.758574	38.046121	15.310843	28.447812	20.133140	190	191	192
65 H	24.525059	57.947079	47.349261	12.978102	30.664274	25.056150	193	194	195
66 H	22.896795	57.409587	52.884476	12.116462	30.379845	27.985259	196	197	198
67 H	25.999424	56.889092	51.683849	13.758303	30.104411	27.349915	199	200	201
68 H	23.486553	54.893404	50.728581	12.428548	29.048338	26.844409	202	203	204
69 C	31.646855	57.883467	51.089186	16.746795	30.630612	27.035233	205	206	207
70 H	27.286670	69.062571	55.482632	14.439484	36.546339	29.360144	208	209	210
71 H	26.286551	68.368091	58.619476	13.910244	36.178836	31.020091	211	212	213
72 H	24.132240	68.055401	56.053867	12.770232	36.013367	29.662429	214	215	216
73 H	25.664244	63.819280	57.386877	13.580933	33.771709	30.367828	217	218	219
74 H	30.886564	65.924783	56.052967	16.344466	34.885893	29.661953	220	221	222
75 H	29.927069	64.902779	59.090572	15.836723	34.345072	31.269384	223	224	225
76 H	35.315783	61.443140	53.771286	18.688308	32.514309	28.454539	226	227	228
77 H	34.674366	64.445166	55.150963	18.348884	34.102913	29.184633	229	230	231
78 H	36.920944	62.420621	56.567346	19.537722	33.031570	29.934150	232	233	234
79 H	33.850360	57.469290	56.285763	17.912839	30.411439	29.785143	235	236	237
80 H	32.285901	58.034780	59.211797	17.084963	30.710683	31.333534	238	239	240
81 H	35.554774	58.709948	58.910878	18.814776	31.067967	31.174294	241	242	243

82 H 25.829655 60.675975 54.193200 13.668465 32.108343 28.677806 244 245
246

83 H 22.889143 62.179614 50.939533 12.112413 32.904035 26.956040 247 248
249

84 H 31.075408 62.345914 52.360872 16.444398 32.992037 27.708180 250 251
252

85 H 29.352891 60.549346 57.603237 15.532881 32.041334 30.482320 253 254
255

86 H 26.094958 53.157279 44.109137 13.808857 28.129621 23.341550 256 257
258

87 O 23.135918 53.733550 45.983782 12.243000 28.434570 24.333569 259 260
261

88 H 18.534406 55.136141 47.377241 9.807985 29.176789 25.070956 262 263
264

89 H 33.341420 54.404704 49.920042 17.643520 28.789729 26.416549 265 266
267

90 H 31.091561 54.046196 52.447030 16.452946 28.600015 27.753773 268 269
270

91 H 30.058309 54.649410 49.307167 15.906172 28.919222 26.092229 271 272
273

92 C 31.541555 55.050266 50.699692 16.691072 29.131346 26.829122 274 275
276

93 O 32.785052 59.349255 49.748148 17.349102 31.406273 26.325586 277 278
279

94 H 18.649929 59.361815 43.401808 9.869117 31.412920 22.967248 280 281
282

95 H 16.294673 58.958895 45.776096 8.622770 31.199704 24.223667 283 284
285

96 H 16.673806 56.652081 43.354614 8.823398 29.978990 22.942274 286 287
288

Number of elements of the density matrix on this node (used, total): 222269 1773786

Numerical Integration : Fuzzy Cells (Becke)

Becke grid quality: NORMAL

Lebedev angular grid order: min: 11 max: 23

Nr. of radial points: min: 46 max: 52

Total nr. of points: 600201

Nr. of blocks: 4690

Block length: 128

Nr. of dummy points: 119

Test of Precision of the Numerical Integration Grid

==

Integral of the Total Core Density: 85.99998313761812

Relative Error: -1.961E-07

Density Fitting (zlmFit)

ZlmFit Fit Quality: NORMAL

Max L-expansion: min: 6 max: 7

Nr. of radial interpolation points: min: 39 max: 48

S C F *** ScaSCF ***

Initial density matrix is taken from previous geometry

CYCLE   1

SCF Error: norm([F,P])=   0.224240584245, max([F,P])=   0.016312648947

orbitals (Q,E):

A :114...133   (2.00   -0.2514) (2.00   -0.2503) (2.00   -0.2417) (2.00   -0.2382)

(2.00   -0.2317) (2.00   -0.2229) (2.00   -0.2223) (2.00   -0.2177)

(2.00   -0.1965) (2.00   -0.1810) (0.00   -0.0714) (0.00   -0.0700)

(0.00   -0.0642) (0.00   -0.0431) (0.00   -0.0211) (0.00   -0.0156)

(0.00   -0.0138) (0.00   -0.0111) (0.00   -0.0075) (0.00   -0.0022)

CYCLE   2

SDIIS (wt 0.000): 0.0389 0.9611

A-DIIS (wt 1.000): 0.0000 1.0000

DIIS coefficients: 0.0000 1.0000

SCF Error: norm([F,P])=   0.038563046411, max([F,P])=   0.003003106992

orbitals (Q,E):

A :114...124   (2.00   -0.2475) (2.00   -0.2454) (2.00   -0.2356) (2.00   -0.2340)

(2.00   -0.2246) (2.00   -0.2218) (2.00   -0.2201) (2.00   -0.2130)

(2.00   -0.1981) (2.00   -0.1745) (0.00   -0.0705)

CYCLE   3

SDIIS (wt 0.000): 0.0033 0.6328 0.3639

A-DIIS (wt 1.000): 0.0000 0.6937 0.3063

DIIS coefficients: 0.0000 0.6937 0.3063

SCF Error: norm([F,P])= 0.064316405770, max([F,P])= 0.007900053132

orbitals (Q,E):

A :114...124 (2.00 -0.2482) (2.00 -0.2466) (2.00 -0.2363) (2.00 -0.2350)

(2.00 -0.2270) (2.00 -0.2212) (2.00 -0.2203) (2.00 -0.2147)

(2.00 -0.1963) (2.00 -0.1765) (0.00 -0.0696)

CYCLE 4

SDIIS (wt 0.982): 0.3687 0.1960 0.4353

A-DIIS (wt 0.018): 0.0000 0.0000 1.0000

DIIS coefficients: 0.3622 0.1926 0.4452

SCF Error: norm([F,P])= 0.016433263930, max([F,P])= 0.002736263036

orbitals (Q,E):

A :114...124 (2.00 -0.2486) (2.00 -0.2473) (2.00 -0.2373) (2.00 -0.2361)

(2.00 -0.2277) (2.00 -0.2212) (2.00 -0.2204) (2.00 -0.2149)

(2.00 -0.1962) (2.00 -0.1771) (0.00 -0.0695)

CYCLE 5

SDIIS (wt 0.994): 0.1544 0.0744 0.3235 0.4476

A-DIIS (wt 0.006): 0.0000 0.0000 0.2029 0.7971

DIIS coefficients: 0.1535 0.0740 0.3228 0.4497

SCF Error: norm([F,P])= 0.008167561370, max([F,P])= 0.001589533476

orbitals (Q,E):

A :114...124 (2.00 -0.2486) (2.00 -0.2471) (2.00 -0.2368) (2.00 -0.2359)

(2.00 -0.2275) (2.00 -0.2213) (2.00 -0.2204) (2.00 -0.2150)

(2.00 -0.1961) (2.00 -0.1769) (0.00 -0.0695)

CYCLE 6

SDIIS (wt 1.000): 0.0639 0.0287 0.1669 0.3008 0.4397

DIIS coefficients: 0.0639 0.0287 0.1669 0.3008 0.4397

SCF Error: norm([F,P])= 0.002507817215, max([F,P])= 0.000364112663

orbitals (Q,E):

A :114...124 (2.00 -0.2485) (2.00 -0.2471) (2.00 -0.2368) (2.00 -0.2359)

(2.00 -0.2275) (2.00 -0.2212) (2.00 -0.2204) (2.00 -0.2149)

(2.00 -0.1961) (2.00 -0.1769) (0.00 -0.0695)

CYCLE 7

SDIIS (wt 1.000): 0.0211 0.0089 0.0670 0.1431 0.3088 0.4512

DIIS coefficients: 0.0211 0.0089 0.0670 0.1431 0.3088 0.4512

SCF Error: norm([F,P])= 0.000977096171, max([F,P])= 0.000151034458

orbitals (Q,E):

A :114...124 (2.00 -0.2485) (2.00 -0.2471) (2.00 -0.2368) (2.00 -0.2359)

(2.00 -0.2275) (2.00 -0.2212) (2.00 -0.2204) (2.00 -0.2149)

(2.00 -0.1961) (2.00 -0.1770) (0.00 -0.0695)

CYCLE 8

SDIIS (wt 1.000): 0.0066 0.0025 0.0261 0.0636 0.1664 0.3251 0.4096

DIIS coefficients: 0.0066 0.0025 0.0261 0.0636 0.1664 0.3251 0.4096

SCF Error: norm([F,P])= 0.000393467282, max([F,P])= 0.000054183979

orbitals (Q,E):

A :114...124 (2.00 -0.2485) (2.00 -0.2471) (2.00 -0.2368) (2.00 -0.2359)

(2.00 -0.2275) (2.00 -0.2213) (2.00 -0.2204) (2.00 -0.2149)

(2.00 -0.1961) (2.00 -0.1770) (0.00 -0.0695)

CYCLE 9

SDIIS (wt 1.000): 0.0014 0.0004 0.0080 0.0219 0.0669 0.1537 0.2833 0.4644

DIIS coefficients: 0.0014 0.0004 0.0080 0.0219 0.0669 0.1537 0.2833 0.4644

SCF Error: norm([F,P])= 0.000131542301, max([F,P])= 0.000021634851

orbitals (Q,E):

A :114...124 (2.00 -0.2485) (2.00 -0.2471) (2.00 -0.2368) (2.00 -0.2359)

(2.00 -0.2275) (2.00 -0.2212) (2.00 -0.2204) (2.00 -0.2149)

(2.00 -0.1961) (2.00 -0.1770) (0.00 -0.0695)

CYCLE 10

SDIIS (wt 1.000): 0.0001 -0.0000 0.0020 0.0066 0.0243 0.0638 0.1452 0.3274 0.4306

DIIS coefficients: 0.0001 -0.0000 0.0020 0.0066 0.0243 0.0638 0.1452 0.3274 0.4306

SCF Error: norm([F,P])= 0.000052787874, max([F,P])= 0.000007432727

orbitals (Q,E):

A :114...124 (2.00 -0.2485) (2.00 -0.2471) (2.00 -0.2368) (2.00 -0.2359)

(2.00 -0.2275) (2.00 -0.2212) (2.00 -0.2204) (2.00 -0.2149)

(2.00 -0.1961) (2.00 -0.1770) (0.00 -0.0695)

CYCLE 11

SDIIS (wt 1.000): -0.0001 -0.0001 0.0004 0.0018 0.0085 0.0249 0.0665 0.1706 0.3044 0.4230

DIIS coefficients: -0.0001 -0.0001 0.0004 0.0018 0.0085 0.0249 0.0665 0.1706 0.3044 0.4230

SCF Error: norm([F,P])= 0.000019740108, max([F,P])= 0.000003404069

orbitals (Q,E):

A :114...124 (2.00 -0.2485) (2.00 -0.2471) (2.00 -0.2368) (2.00 -0.2359)

(2.00 -0.2275) (2.00 -0.2212) (2.00 -0.2204) (2.00 -0.2149)

(2.00 -0.1961) (2.00 -0.1770) (0.00 -0.0695)

CYCLE 12

SDIIS (wt 1.000): -0.0000 0.0002 0.0007 0.0031 0.0089 0.0258 0.0710 0.1519 0.3159 0.4225

DIIS coefficients: -0.0000 0.0002 0.0007 0.0031 0.0089 0.0258 0.0710 0.1519 0.3159 0.4225

SCF Error: norm([F,P])= 0.000008742525, max([F,P])= 0.000001602797

orbitals (Q,E):

A :114...124 (2.00 -0.2485) (2.00 -0.2471) (2.00 -0.2368) (2.00 -0.2359)

(2.00 -0.2275) (2.00 -0.2212) (2.00 -0.2204) (2.00 -0.2149)

(2.00 -0.1961) (2.00 -0.1770) (0.00 -0.0695)

CYCLE 13

SDIIS (wt 1.000): 0.0001 0.0002 0.0010 0.0029 0.0094 0.0273 0.0651 0.1513 0.2501 0.4926

DIIS coefficients: 0.0001 0.0002 0.0010 0.0029 0.0094 0.0273 0.0651 0.1513 0.2501 0.4926

SCF Error: norm([F,P])= 0.000001857595, max([F,P])= 0.000000231537

orbitals (Q,E):

A :114...124 (2.00 -0.2485) (2.00 -0.2471) (2.00 -0.2368) (2.00 -0.2359)

(2.00 -0.2275) (2.00 -0.2212) (2.00 -0.2204) (2.00 -0.2149)

(2.00 -0.1961) (2.00 -0.1770) (0.00 -0.0695)

SCF CONVERGED

CYCLE 14

*** Setting up for NEW gradients in Focky

*** Using FIT density in Focky

*** Setting up for NEW Hessian in Focky

orbitals (Q,E):

A :114...133 (2.00 -0.2485) (2.00 -0.2471) (2.00 -0.2368) (2.00 -0.2359)

(2.00 -0.2275) (2.00 -0.2212) (2.00 -0.2204) (2.00 -0.2149)

(2.00 -0.1961) (2.00 -0.1770) (0.00 -0.0695) (0.00 -0.0681)

(0.00 -0.0605) (0.00 -0.0383) (0.00 -0.0189) (0.00 -0.0132)

(0.00 -0.0118) (0.00 -0.0088) (0.00 -0.0050) (0.00 -0.0001)

Fit test: (difference of exact and fit density, squared integrated, result summed over spins)

SCF: 0.00100995565614

Gross Charges per Atom (Z minus electrons)

==

-0.6612	0.9259	0.5380	0.3332	-0.5790	-0.3841	-0.6353	0.6776	0.6319	0.8640
-0.6602	-0.0038	-0.5784	0.5913	0.3279	-0.5909	0.6034	0.6016	-0.5960	0.2589
0.3404	0.0998	0.1671	0.7479	0.4068	0.4070	0.5649	0.5739	0.0303	0.6449
-0.5658	0.5503	0.5510	0.5741	0.6525	0.3723	0.7544	0.9877	0.5907	-0.2137
-0.1871	-0.2057	-0.1926	-0.2134	-0.2453	-0.2321	-0.1485	-0.1875	-0.1438	-0.2125
-0.2385	-0.2222	-0.2227	-0.1868	-0.2482	-0.1714	-0.1971	-0.1538	-0.1973	-0.2148
-0.2256	-0.2172	-0.2282	-0.1958	-0.1258	-0.2254	-0.1560	-0.2201	0.8356	-0.1972
-0.2187	-0.2175	-0.2124	-0.1908	-0.2123	-0.1732	-0.2291	-0.2359	-0.1965	-0.2433
-0.2154	-0.2501	-0.1951	-0.1603	-0.1639	0.2410	-0.6730	-0.1262	-0.1827	-0.1944
-0.1200	0.4888	-0.6124	-0.1737	-0.2184	-0.2389				

Net Total: -0.00000000

M U L T I P O L E D E R I V E D C H A R G E A N A L Y S I S

See: M. Swart, P.Th. van Duijnen, J.G. Snijders, J.Comput.Chem., (2001), p. 79-88.

Multipole derived atomic charges (a.u.)

Atom	Level:	MDC-m	MDC-d	MDC-q
1	O	-0.353107	-0.344861	-0.368974
2	C	0.363236	0.376601	0.399990
3	C	0.242424	0.250841	0.254378
4	C	0.180361	0.165174	0.211259
5	O	-0.303787	-0.547354	-0.619507
6	N	-0.214554	-0.276325	-0.468997
7	O	-0.379255	-0.371285	-0.439504
8	C	0.250402	0.310126	0.310111
9	C	0.228299	0.193715	0.186088
10	C	0.338549	0.411298	0.417096
11	O	-0.382119	-0.352932	-0.391765
12	C	0.124226	0.102377	0.101901
13	O	-0.313331	-0.298736	-0.347200
14	C	0.244935	0.245231	0.250171
15	C	0.195381	0.158467	0.175031
16	O	-0.369645	-0.479242	-0.486020
17	C	0.229102	0.194982	0.195646
18	C	0.230048	0.193494	0.195715
19	O	-0.317913	-0.319551	-0.389989
20	C	0.058840	0.046976	-0.061411
21	C	0.195092	0.221088	0.283936
22	C	0.119328	0.105700	0.109978
23	C	0.119748	0.099693	0.108799
24	C	0.290189	0.267475	0.288748
25	C	0.172394	0.136006	0.138644
26	C	0.165056	0.124600	0.165815
27	C	0.249138	0.237061	0.260333
28	C	0.234594	0.193886	0.289594

29	C	0.033804	0.027264	-0.055758
30	C	0.237047	0.191841	0.180519
31	O	-0.370898	-0.476140	-0.521177
32	C	0.253922	0.242357	0.261901
33	C	0.225369	0.188259	0.170691
34	C	0.226993	0.295628	0.396646
35	C	0.222009	0.192219	0.202599
36	C	0.180495	0.146500	0.154902
37	C	0.293881	0.270808	0.312679
38	C	0.368555	0.339948	0.386531
39	C	0.229930	0.191047	0.158451
40	H	-0.072990	-0.060139	-0.021151
41	H	-0.059335	-0.033916	-0.020231
42	H	-0.061983	-0.052981	-0.020532
43	H	-0.055911	-0.035325	-0.003002
44	H	-0.068813	-0.052488	-0.050134
45	H	-0.070325	-0.057567	-0.051572
46	H	-0.076318	-0.071135	-0.069236
47	H	-0.071600	-0.060549	-0.061258
48	H	-0.065076	-0.045039	-0.026019
49	H	-0.082730	-0.064566	-0.072546
50	H	-0.055889	-0.048803	-0.048275
51	H	-0.071949	-0.054130	-0.059175
52	H	-0.071536	-0.060273	-0.066353
53	H	-0.067514	-0.063291	-0.074090
54	H	-0.058574	-0.046144	-0.053049
55	H	-0.054171	-0.044365	-0.045906
56	H	-0.057625	-0.042514	-0.029751
57	H	-0.068042	-0.051799	-0.054060
58	H	-0.061579	-0.036924	-0.035433
59	H	-0.075422	-0.060041	-0.050034

60	H	-0.074778	-0.065458	-0.056221
61	H	-0.069206	-0.055896	-0.044462
62	H	-0.066286	-0.047200	-0.035522
63	H	-0.072400	-0.075074	-0.082535
64	H	-0.058633	-0.036870	-0.038119
65	H	-0.073099	-0.048055	-0.055185
66	H	-0.072091	-0.062963	-0.056888
67	H	-0.067305	-0.053461	-0.062837
68	H	-0.065721	-0.057740	-0.055164
69	C	0.350546	0.446799	0.475824
70	H	-0.064731	-0.054306	-0.078341
71	H	-0.068108	-0.067246	-0.094994
72	H	-0.063750	-0.048907	-0.070084
73	H	-0.080653	-0.063264	-0.074048
74	H	-0.072829	-0.060525	-0.065868
75	H	-0.069556	-0.069164	-0.071469
76	H	-0.074722	-0.043621	-0.034611
77	H	-0.066698	-0.054438	-0.041416
78	H	-0.065391	-0.061242	-0.031561
79	H	-0.084130	-0.063916	-0.048980
80	H	-0.066629	-0.058097	-0.051886
81	H	-0.068008	-0.066600	-0.055594
82	H	-0.070683	-0.043272	-0.036862
83	H	-0.061869	-0.021897	-0.017743
84	H	-0.045909	0.001820	-0.012441
85	H	-0.073021	-0.059272	-0.040086
86	H	0.064341	0.266840	0.313704
87	O	-0.364441	-0.446040	-0.464203
88	H	-0.045221	0.000872	0.022411
89	H	-0.046373	-0.031776	-0.032130
90	H	-0.052550	-0.051701	-0.058812

91	H	-0.042459	-0.013671	-0.028481
92	C	0.240041	0.197479	0.239400
93	O	-0.367234	-0.474522	-0.505396
94	H	-0.061585	-0.055131	-0.051241
95	H	-0.061670	-0.048816	-0.041785
96	H	-0.068544	-0.065915	-0.062414

*** Using NEW gradient routines ***

Geometry CYCLE 5

==============

Energy gradients wrt nuclear displacements

==

Atom	Cartesian (a.u./angstrom)		
	X	Y	Z
1 O	0.001575	-0.000863	0.001166
2 C	-0.002343	-0.000081	0.000330
3 C	0.002454	0.002128	-0.001537
4 C	0.000145	-0.000760	0.002122
5 O	0.001529	-0.000178	-0.000161
6 N	0.000624	0.000291	0.001146
7 O	0.001064	-0.001159	0.000891
8 C	-0.003059	0.003820	0.005320
9 C	-0.000365	0.000606	-0.000089
10 C	0.004689	-0.003546	0.000177
11 O	-0.000163	-0.000919	0.001618
12 C	0.000260	-0.000733	-0.000616
13 O	-0.003965	-0.001240	0.002695

14	C	-0.004211	-0.000730	0.000571
15	C	-0.001811	0.001658	-0.002785
16	O	0.005633	-0.003948	-0.003816
17	C	0.001048	0.002336	0.002394
18	C	-0.000039	0.000106	-0.000182
19	O	0.000303	-0.000989	-0.000787
20	C	0.002070	0.002933	0.002064
21	C	0.001294	-0.001610	0.000719
22	C	0.000017	0.000248	-0.003020
23	C	0.000065	0.000379	0.000347
24	C	0.000122	-0.000605	-0.000779
25	C	0.001585	-0.001512	0.000555
26	C	-0.000809	0.001550	0.000356
27	C	0.001235	-0.000844	0.000176
28	C	-0.000192	0.000282	-0.000264
29	C	-0.001730	-0.001008	-0.000086
30	C	0.000486	-0.000286	-0.001137
31	O	-0.004909	-0.003897	0.003718
32	C	-0.000143	0.000819	-0.002639
33	C	0.000800	0.000365	-0.000505
34	C	0.002917	0.002236	-0.005600
35	C	0.000232	0.000426	0.000128
36	C	-0.000190	-0.000339	0.001700
37	C	-0.000750	0.000913	-0.000958
38	C	0.000346	0.001208	-0.000087
39	C	-0.000342	0.001414	0.000012
40	H	0.001328	0.000003	-0.000021
41	H	-0.000971	-0.000725	-0.000138
42	H	-0.000167	0.000152	0.000101
43	H	-0.000129	-0.000204	0.000146
44	H	-0.000191	-0.000346	-0.000084

45	H	0.000055	-0.000138	0.000487
46	H	0.000439	-0.000231	0.000108
47	H	0.000531	0.000127	-0.000530
48	H	-0.000381	0.000165	-0.000580
49	H	-0.000252	0.000041	-0.000055
50	H	-0.001031	0.000050	-0.000549
51	H	-0.000253	0.000084	-0.000439
52	H	-0.000075	-0.000518	0.000187
53	H	0.000440	-0.000199	0.000122
54	H	0.000486	0.000151	-0.000337
55	H	0.000635	0.000257	0.000254
56	H	0.000399	-0.000208	0.000261
57	H	-0.000350	0.000867	-0.000223
58	H	-0.000080	-0.000251	-0.000152
59	H	0.000337	-0.000016	-0.000123
60	H	0.000188	-0.000177	0.000348
61	H	0.000215	0.000129	0.000066
62	H	0.000118	0.000482	0.000412
63	H	0.000020	-0.000086	0.000281
64	H	-0.000284	0.000142	0.000008
65	H	0.000622	0.000025	0.001319
66	H	0.000474	0.000155	-0.000534
67	H	-0.001514	0.000552	-0.000266
68	H	0.000101	-0.000067	-0.000421
69	C	0.001786	0.005609	-0.003304
70	H	-0.000280	-0.000247	0.000186
71	H	0.000005	-0.000146	-0.000316
72	H	0.000307	-0.000055	0.000195
73	H	0.000016	0.000237	-0.000336
74	H	-0.000393	-0.000492	0.000156
75	H	0.000152	-0.000268	-0.000323

76 H -0.000216 0.000235 0.000479

77 H 0.000066 -0.000061 0.000109

78 H -0.000380 -0.000009 -0.000055

79 H -0.000033 0.000117 0.000081

80 H 0.000178 0.000147 -0.000063

81 H -0.000200 -0.000103 -0.000316

82 H -0.000783 -0.001026 -0.001109

83 H 0.000608 -0.000427 0.000517

84 H 0.000103 -0.001323 -0.000135

85 H 0.000685 -0.000564 -0.000850

86 H 0.000077 0.001635 -0.001109

87 O -0.005270 0.003319 -0.001373

88 H -0.000486 0.000043 -0.000753

89 H -0.000212 0.000060 0.000340

90 H 0.000076 0.000005 -0.000251

91 H 0.000647 0.000241 0.000115

92 C 0.000376 -0.000005 0.000179

93 O -0.003471 -0.004910 0.004635

94 H -0.000086 -0.000424 0.000218

95 H 0.000275 -0.000320 -0.000040

96 H 0.000271 0.000046 0.000315

----

dE(predicted): -0.12227E-02, dE(actual): -0.70904E-03, Trust radius: 0.195959, all in a.u.

Geometry Convergence after Step 5 (Hartree/Angstrom,Angstrom)

----

current energy -19.67384677 Hartree

energy change -0.00070904 0.00100000 T

constrained gradient max 0.00563312 0.00100000 F

constrained gradient rms 0.00144356 0.00066667 F

gradient max 0.00563312

gradient rms 0.00144356

cart. step max 0.19301992 0.01000000 F

cart. step rms 0.05783210 0.00666667 F

Coordinates (Cartesian)

=======================

Atom	bohr X	bohr Y	bohr Z	angstrom X	angstrom Y	angstrom Z	Geometric Variables (0:frozen, *:LT par.)		
1 O	26.765286	61.374700	50.492831	14.163580	32.478093	26.719655	1	2	3
2 C	27.129921	61.905731	53.058894	14.356536	32.759102	28.077558	4	5	6
3 C	29.899043	61.263858	53.672633	15.821892	32.419437	28.402334	7	8	9
4 C	30.550639	61.951254	56.440060	16.166702	32.783192	29.866793	10	11	12
5 O	27.085839	52.928828	42.482252	14.333209	28.008729	22.480640	13	14	15
6 N	33.176594	61.475675	57.245767	17.556297	32.531526	30.293155	16	17	18
7 O	26.669226	64.544917	53.489045	14.112747	34.155699	28.305184	19	20	21
8 C	20.597126	58.306513	48.411298	10.899530	30.854478	25.618156	22	23	24
9 C	25.631324	65.468314	41.630369	13.563513	34.644340	22.029843	25	26	27
10 C	21.600457	55.033640	44.841658	11.430470	29.122548	23.729184	28	29	30
11 O	21.787576	62.678863	45.836476	11.529489	33.168226	24.255619	31	32	33
12 C	19.579703	56.514727	46.270297	10.361132	29.906306	24.485187	34	35	36
13 O	21.376857	55.269657	42.320352	11.312146	29.247443	22.394966	37	38	39
14 C	24.178884	61.309649	49.560547	12.794915	32.443669	26.226312	40	41	42
15 C	24.152423	63.108819	47.191283	12.780912	33.395749	24.972551	43	44	45
16 O	19.033097	59.311274	49.777892	10.071881	31.386175	26.341326	46	47	48
17 C	23.993394	56.699123	51.165886	12.696757	30.003884	27.075821	49	50	51

18 C	26.930056	53.389265	38.005987	14.250772	28.252382	20.111902	52	53	54
19 O	30.206784	58.540249	53.305484	15.984742	30.978166	28.208047	55	56	57
20 C	26.078706	57.169065	40.744020	13.800257	30.252566	21.560807	58	59	60
21 C	25.770465	54.300731	40.531272	13.637143	28.734709	21.448226	61	62	63
22 C	23.422692	58.542707	48.952742	12.394755	30.979466	25.904676	64	65	66
23 C	25.864390	62.707034	42.483029	13.686846	33.183133	22.481051	67	68	69
24 C	35.157279	62.420962	55.539905	18.604431	33.031751	29.390452	70	71	72
25 C	26.338946	62.332118	45.360954	13.937970	32.984736	24.003983	73	74	75
26 C	29.784984	64.735334	56.905589	15.761535	34.256463	30.113141	76	77	78
27 C	27.037543	65.256221	56.121238	14.307652	34.532105	29.698080	79	80	81
28 C	26.285928	68.030760	56.336553	13.909914	36.000328	29.812020	82	83	84
29 C	28.179993	58.482936	41.302456	14.912210	30.947837	21.856318	85	86	87
30 C	17.639739	57.900507	44.596386	9.334548	30.639629	23.599391	88	89	90
31 O	29.805709	62.520388	40.168137	15.772502	33.084365	21.256062	91	92	93
32 C	22.871327	53.662252	40.519879	12.102985	28.396841	21.442196	94	95	96
33 C	30.762675	57.422826	41.904472	16.278907	30.386851	22.174892	97	98	99
34 C	28.079379	61.349000	41.170812	14.858967	32.464493	21.786656	100	101	102
35 C	24.480847	65.855704	48.092177	12.954706	34.849338	25.449284	103	104	105
36 C	22.209691	50.861908	40.849506	11.752862	26.914963	21.616628	106	107	108
37 C	33.694412	58.880279	58.084617	17.830315	31.158102	30.737056	109	110	111
38 C	19.605750	64.097858	46.630527	10.374916	33.919126	24.675812	112	113	114
39 C	19.349876	50.336422	40.851355	10.239513	26.636887	21.617606	115	116	117
40 H	24.388532	58.233720	40.259683	12.905855	30.815958	21.304507	118	119	120
41 H	31.292711	57.939153	43.845722	16.559389	30.660080	23.202157	121	122	123
42 H	30.831538	55.372461	41.744337	16.315347	29.301844	22.090152	124	125	126

43 H 32.163362 58.297905 40.655238 17.020118 30.849923 21.513826 127 128
129

44 H 27.267048 66.577604 42.243272 14.429100 35.231351 22.354177 130 131
132

45 H 25.541350 65.587095 39.565918 13.515901 34.707196 20.937382 133 134
135

46 H 23.912259 66.313398 42.405344 12.653823 35.091539 22.439942 136 137
138

47 H 24.108547 61.709140 42.027214 12.757694 32.655071 22.239844 139 140
141

48 H 28.043355 63.364325 45.914488 14.839904 33.530957 24.296901 142 143
144

49 H 26.776146 60.336932 45.658841 14.169326 31.928929 24.161618 145 146
147

50 H 26.288895 66.015396 49.070835 13.911484 34.933843 25.967167 148 149
150

51 H 23.013919 66.405739 49.440153 12.178441 35.140404 26.162602 151 152
153

52 H 24.465200 67.169268 46.500985 12.946427 35.544446 24.607262 154 155
156

53 H 19.860562 66.129203 46.308817 10.509757 34.994067 24.505571 157 158
159

54 H 19.127730 63.748909 48.617822 10.121959 33.734470 25.727444 160 161
162

55 H 18.047224 63.425880 45.458482 9.550179 33.563530 24.055593 163 164
165

56 H 22.098636 54.349378 38.727193 11.694094 28.760453 20.493548 166 167
168

57 H 23.093863 49.852280 39.270906 12.220746 26.380690 20.781268 169 170
171

58 H 23.084151 50.140150 42.574268 12.215607 26.533025 22.529332 172 173
174

59 H 18.416875 51.225143 42.470876 9.745791 27.107178 22.474620 175 176
177

60 H 18.443440 51.055638 39.131097 9.759848 27.017480 20.707285 178 179
180

61 H 18.993608 48.300079 40.959060 10.050985 25.559301 21.674601 181 182
183

62 H	26.731038	51.340219	37.811666	14.145456	27.168074	20.009072	184	185	186
63 H	26.036390	54.327467	36.393114	13.777864	28.748857	19.258406	187	188	189
64 H	28.942020	53.852284	37.989674	15.315457	28.497401	20.103270	190	191	192
65 H	24.512606	57.910459	47.320951	12.971513	30.644895	25.041169	193	194	195
66 H	22.906117	57.231085	52.844238	12.121395	30.285386	27.963967	196	197	198
67 H	26.004152	56.732912	51.620413	13.760804	30.021764	27.316346	199	200	201
68 H	23.478617	54.773812	50.620294	12.424349	28.985053	26.787106	202	203	204
69 C	31.508922	57.733527	51.201060	16.673803	30.551267	27.094434	205	206	207
70 H	27.490836	69.192727	55.117877	14.547524	36.615214	29.167124	208	209	210
71 H	26.494763	68.684064	58.290581	14.020425	36.346042	30.846047	211	212	213
72 H	24.317380	68.293979	55.755668	12.868203	36.139618	29.504629	214	215	216
73 H	25.757862	64.095679	57.297469	13.630474	33.917973	30.320515	217	218	219
74 H	31.018489	66.004362	55.826000	16.414277	34.928004	29.541847	220	221	222
75 H	30.054050	65.159806	58.914822	15.903919	34.481084	31.176381	223	224	225
76 H	35.360959	61.295820	53.792707	18.712214	32.436351	28.465874	226	227	228
77 H	34.775006	64.380274	55.002913	18.402141	34.068574	29.106288	229	230	231
78 H	36.969207	62.389910	56.542301	19.563262	33.015318	29.920897	232	233	234
79 H	33.793957	57.491840	56.529312	17.882992	30.423371	29.914024	235	236	237
80 H	32.222612	58.263663	59.402905	17.051472	30.831803	31.434664	238	239	240

81 H 35.506853 58.849178 59.090157 18.789417 31.141644 31.269165 241 242
243

82 H 25.843904 60.784288 54.264555 13.676005 32.165660 28.715566 244 245
246

83 H 22.899337 62.072605 50.998758 12.117807 32.847408 26.987381 247 248
249

84 H 31.096238 62.269047 52.324676 16.455420 32.951361 27.689026 250 251
252

85 H 29.357024 60.759056 57.650345 15.535068 32.152308 30.507249 253 254
255

86 H 26.085296 53.121493 44.032822 13.803744 28.110683 23.301166 256 257
258

87 O 23.190859 53.732476 45.918683 12.272074 28.434002 24.299121 259 260
261

88 H 18.579702 55.050715 47.359660 9.831955 29.131584 25.061653 262 263
264

89 H 33.032173 54.157848 50.093400 17.479873 28.659099 26.508286 265 266
267

90 H 30.895649 53.974389 52.734523 16.349273 28.562017 27.905908 268 269
270

91 H 29.732499 54.491751 49.626401 15.733761 28.835793 26.261160 271 272
273

92 C 31.294102 54.893570 50.929708 16.560126 29.048426 26.950841 274 275
276

93 O 32.624718 59.111599 49.749162 17.264257 31.280511 26.326123 277 278
279

94 H 18.586785 59.303101 43.415389 9.835703 31.381849 22.974434 280 281
282

95 H 16.260078 58.846519 45.807616 8.604463 31.140237 24.240346 283 284
285

96 H 16.654694 56.563660 43.367137 8.813284 29.932200 22.948901 286 287
288

Number of elements of the density matrix on this node (used, total): 222269 1773786

Numerical Integration : Fuzzy Cells (Becke)

Becke grid quality: NORMAL

Lebedev angular grid order: min: 11 max: 23

Nr. of radial points: min: 46 max: 52

Total nr. of points: 600212

Nr. of blocks: 4690

Block length: 128

Nr. of dummy points: 108

Test of Precision of the Numerical Integration Grid

===

Integral of the Total Core Density: 86.00002202574530

Relative Error: 2.561E-07

Density Fitting (zlmFit)

ZlmFit Fit Quality: NORMAL

Max L-expansion: min: 6 max: 7

Nr. of radial interpolation points: min: 39 max: 48

S C F *** ScaSCF ***

Initial density matrix is taken from previous geometry

CYCLE   1

SCF Error: norm([F,P])=   0.224891704552, max([F,P])=   0.020781460504

orbitals (Q,E):

A :114...133   (2.00   -0.2566) (2.00   -0.2510) (2.00   -0.2456) (2.00   -0.2390)

(2.00   -0.2378) (2.00   -0.2264) (2.00   -0.2240) (2.00   -0.2205)

(2.00   -0.1995) (2.00   -0.1868) (0.00   -0.0739) (0.00   -0.0729)

(0.00   -0.0649) (0.00   -0.0480) (0.00   -0.0225) (0.00   -0.0193)

(0.00   -0.0160) (0.00   -0.0135) (0.00   -0.0108) (0.00   -0.0044)

CYCLE   2

SDIIS (wt 0.000): 0.0486 0.9514

A-DIIS (wt 1.000): 0.0000 1.0000

DIIS coefficients: 0.0000 1.0000

SCF Error: norm([F,P])=   0.044992273016, max([F,P])=   0.004418962643

orbitals (Q,E):

A :114...124   (2.00   -0.2470) (2.00   -0.2460) (2.00   -0.2348) (2.00   -0.2316)

(2.00   -0.2220) (2.00   -0.2198) (2.00   -0.2190) (2.00   -0.2133)

(2.00   -0.1950) (2.00   -0.1700) (0.00   -0.0689)

CYCLE 3

SDIIS (wt 0.000): 0.0017 0.6257 0.3726

A-DIIS (wt 1.000): 0.0000 0.6847 0.3153

DIIS coefficients: 0.0000 0.6847 0.3153

SCF Error: norm([F,P])= 0.073575447856, max([F,P])= 0.007224767978

orbitals (Q,E):

A :114...124 (2.00 -0.2486) (2.00 -0.2468) (2.00 -0.2351) (2.00 -0.2340)

(2.00 -0.2271) (2.00 -0.2203) (2.00 -0.2195) (2.00 -0.2143)

(2.00 -0.1955) (2.00 -0.1759) (0.00 -0.0690)

CYCLE 4

SDIIS (wt 0.984): 0.3686 0.1847 0.4467

A-DIIS (wt 0.016): 0.0000 0.0000 1.0000

DIIS coefficients: 0.3628 0.1818 0.4555

SCF Error: norm([F,P])= 0.016065537886, max([F,P])= 0.002560554978

orbitals (Q,E):

A :114...124 (2.00 -0.2494) (2.00 -0.2468) (2.00 -0.2365) (2.00 -0.2349)

(2.00 -0.2282) (2.00 -0.2208) (2.00 -0.2198) (2.00 -0.2149)

(2.00 -0.1952) (2.00 -0.1768) (0.00 -0.0691)

CYCLE 5

SDIIS (wt 0.996): 0.1603 0.0658 0.3429 0.4309

A-DIIS (wt 0.004): 0.0000 0.0000 0.2092 0.7908

DIIS coefficients: 0.1597 0.0656 0.3424 0.4323

SCF Error: norm([F,P])= 0.008602841453, max([F,P])= 0.001390513510

orbitals (Q,E):

A :114...124 (2.00 -0.2493) (2.00 -0.2468) (2.00 -0.2362) (2.00 -0.2348)

(2.00 -0.2281) (2.00 -0.2207) (2.00 -0.2197) (2.00 -0.2148)

(2.00 -0.1954) (2.00 -0.1765) (0.00 -0.0691)

CYCLE 6

SDIIS (wt 1.000): 0.0672 0.0224 0.1889 0.3138 0.4077

DIIS coefficients: 0.0672 0.0224 0.1889 0.3138 0.4077

SCF Error: norm([F,P])= 0.003162394638, max([F,P])= 0.000344767079

orbitals (Q,E):

A :114...124 (2.00 -0.2493) (2.00 -0.2468) (2.00 -0.2362) (2.00 -0.2348)

(2.00 -0.2281) (2.00 -0.2207) (2.00 -0.2197) (2.00 -0.2148)

(2.00 -0.1953) (2.00 -0.1767) (0.00 -0.0691)

CYCLE 7

SDIIS (wt 1.000): 0.0217 0.0051 0.0773 0.1499 0.2772 0.4688

DIIS coefficients: 0.0217 0.0051 0.0773 0.1499 0.2772 0.4688

SCF Error: norm([F,P])= 0.001003495109, max([F,P])= 0.000124880439

orbitals (Q,E):

A :114...124 (2.00 -0.2493) (2.00 -0.2468) (2.00 -0.2362) (2.00 -0.2348)

(2.00 -0.2281) (2.00 -0.2207) (2.00 -0.2197) (2.00 -0.2148)

(2.00 -0.1953) (2.00 -0.1767) (0.00 -0.0691)

CYCLE 8

SDIIS (wt 1.000): 0.0072 0.0009 0.0321 0.0690 0.1500 0.3443 0.3966

DIIS coefficients: 0.0072 0.0009 0.0321 0.0690 0.1500 0.3443 0.3966

SCF Error: norm([F,P])= 0.000454817226, max([F,P])= 0.000079137787

orbitals (Q,E):

A :114...124 (2.00 -0.2493) (2.00 -0.2468) (2.00 -0.2362) (2.00 -0.2348)

(2.00 -0.2281) (2.00 -0.2207) (2.00 -0.2197) (2.00 -0.2148)

(2.00 -0.1953) (2.00 -0.1767) (0.00 -0.0691)

CYCLE 9

SDIIS (wt 1.000): 0.0014 -0.0001 0.0090 0.0223 0.0562 0.1533 0.2534 0.5045

DIIS coefficients: 0.0014 -0.0001 0.0090 0.0223 0.0562 0.1533 0.2534 0.5045

SCF Error: norm([F,P])= 0.000123889213, max([F,P])= 0.000016924840

orbitals (Q,E):

A :114...124 (2.00 -0.2493) (2.00 -0.2468) (2.00 -0.2362) (2.00 -0.2348)

(2.00 -0.2281) (2.00 -0.2207) (2.00 -0.2197) (2.00 -0.2148)

(2.00 -0.1953) (2.00 -0.1767) (0.00 -0.0691)

CYCLE 10

SDIIS (wt 1.000): 0.0001 -0.0002 0.0022 0.0066 0.0192 0.0602 0.1215 0.3388 0.4516

DIIS coefficients: 0.0001 -0.0002 0.0022 0.0066 0.0192 0.0602 0.1215 0.3388 0.4516

SCF Error: norm([F,P])= 0.000049924945, max([F,P])= 0.000007082870

orbitals (Q,E):

A :114...124 (2.00 -0.2493) (2.00 -0.2468) (2.00 -0.2362) (2.00 -0.2348)

(2.00 -0.2281) (2.00 -0.2207) (2.00 -0.2197) (2.00 -0.2148)

(2.00 -0.1953) (2.00 -0.1767) (0.00 -0.0691)

CYCLE 11

SDIIS (wt 1.000): -0.0001 -0.0001 0.0004 0.0018 0.0066 0.0234 0.0548 0.1777 0.3252 0.4102

DIIS coefficients: -0.0001 -0.0001 0.0004 0.0018 0.0066 0.0234 0.0548 0.1777 0.3252 0.4102

SCF Error: norm([F,P])= 0.000020793937, max([F,P])= 0.000002471467

orbitals (Q,E):

A :114...124 (2.00 -0.2493) (2.00 -0.2468) (2.00 -0.2362) (2.00 -0.2348)

(2.00 -0.2281) (2.00 -0.2207) (2.00 -0.2197) (2.00 -0.2148)

(2.00 -0.1953) (2.00 -0.1767) (0.00 -0.0691)

CYCLE 12

SDIIS (wt 1.000): -0.0000 0.0003 0.0008 0.0028 0.0096 0.0234 0.0801 0.1698 0.3134 0.4000

DIIS coefficients: -0.0000 0.0003 0.0008 0.0028 0.0096 0.0234 0.0801 0.1698 0.3134 0.4000

SCF Error: norm([F,P])= 0.000009211858, max([F,P])= 0.000001187559

orbitals (Q,E):

A :114...124 (2.00 -0.2493) (2.00 -0.2468) (2.00 -0.2362) (2.00 -0.2348)

(2.00 -0.2281) (2.00 -0.2207) (2.00 -0.2197) (2.00 -0.2148)

(2.00 -0.1953) (2.00 -0.1767) (0.00 -0.0691)

CYCLE 13

SDIIS (wt 1.000): 0.0000 0.0001 0.0007 0.0027 0.0077 0.0301 0.0728 0.1627 0.2699 0.4532

DIIS coefficients: 0.0000 0.0001 0.0007 0.0027 0.0077 0.0301 0.0728 0.1627 0.2699 0.4532

SCF Error: norm([F,P])= 0.000002658953, max([F,P])= 0.000000364113

orbitals (Q,E):

A :114...124 (2.00 -0.2493) (2.00 -0.2468) (2.00 -0.2362) (2.00 -0.2348)

(2.00 -0.2281) (2.00 -0.2207) (2.00 -0.2197) (2.00 -0.2148)

(2.00 -0.1953) (2.00 -0.1767) (0.00 -0.0691)

SCF CONVERGED

CYCLE 14

*** Setting up for NEW gradients in Focky

*** Using FIT density in Focky

*** Setting up for NEW Hessian in Focky

orbitals (Q,E):

A :114...133 (2.00 -0.2493) (2.00 -0.2468) (2.00 -0.2362) (2.00 -0.2348)

(2.00 -0.2281) (2.00 -0.2207) (2.00 -0.2197) (2.00 -0.2148)

(2.00 -0.1953) (2.00 -0.1767) (0.00 -0.0691) (0.00 -0.0683)

(0.00 -0.0611) (0.00 -0.0383) (0.00 -0.0184) (0.00 -0.0132)

(0.00 -0.0112) (0.00 -0.0084) (0.00 -0.0050) (0.00 0.0000)

Fit test: (difference of exact and fit density, squared integrated, result summed over spins)

SCF: 0.00100894660258

Gross Charges per Atom (Z minus electrons)

===

-0.6615	0.9295	0.5394	0.3339	-0.5798	-0.3835	-0.6371	0.6745	0.6318	0.8651
-0.6595	-0.0018	-0.5783	0.5925	0.3278	-0.5921	0.6035	0.6033	-0.5948	0.2573
0.3418	0.0967	0.1676	0.7489	0.4085	0.4091	0.5644	0.5766	0.0315	0.6444
-0.5662	0.5501	0.5516	0.5745	0.6571	0.3741	0.7532	0.9868	0.5903	-0.2150
-0.1855	-0.2065	-0.1926	-0.2142	-0.2445	-0.2328	-0.1491	-0.1893	-0.1417	-0.2079
-0.2402	-0.2277	-0.2255	-0.1833	-0.2488	-0.1723	-0.1961	-0.1549	-0.1987	-0.2150
-0.2250	-0.2183	-0.2290	-0.1954	-0.1230	-0.2279	-0.1498	-0.2181	0.8330	-0.1977
-0.2192	-0.2175	-0.2120	-0.1918	-0.2127	-0.1770	-0.2281	-0.2352	-0.1983	-0.2425
-0.2140	-0.2556	-0.1983	-0.1600	-0.1632	0.2410	-0.6737	-0.1265	-0.1866	-0.1929
-0.1193	0.4890	-0.6122	-0.1756	-0.2177	-0.2385				

Net Total: -0.00000000

M U L T I P O L E D E R I V E D C H A R G E A N A L Y S I S

See: M. Swart, P.Th. van Duijnen, J.G. Snijders, J.Comput.Chem., (2001), p. 79-88.

Multipole derived atomic charges (a.u.)

Atom		Level: MDC-m	MDC-d	MDC-q
1	O	-0.351272	-0.341643	-0.363592
2	C	0.363409	0.375625	0.398882
3	C	0.242815	0.251266	0.256974
4	C	0.180640	0.164657	0.209774
5	O	-0.305602	-0.548782	-0.619733
6	N	-0.214642	-0.273005	-0.457977
7	O	-0.377156	-0.357398	-0.430501
8	C	0.250178	0.309425	0.308690
9	C	0.228285	0.193903	0.188471
10	C	0.339200	0.409941	0.415712
11	O	-0.382398	-0.355262	-0.395869
12	C	0.124425	0.102484	0.102621
13	O	-0.313046	-0.299161	-0.347560
14	C	0.244557	0.245251	0.249569
15	C	0.195005	0.159297	0.176030
16	O	-0.369586	-0.478693	-0.484165
17	C	0.229217	0.194524	0.196978
18	C	0.229932	0.193630	0.196685
19	O	-0.318930	-0.323526	-0.395617
20	C	0.059049	0.047102	-0.060644
21	C	0.195118	0.221798	0.283603
22	C	0.119362	0.105339	0.109258
23	C	0.119964	0.099620	0.108625
24	C	0.290840	0.267808	0.288182
25	C	0.172642	0.136187	0.138826
26	C	0.165017	0.124216	0.165587
27	C	0.249013	0.234318	0.256343

28	C	0.234669	0.193898	0.285763
29	C	0.033909	0.027137	-0.056162
30	C	0.237178	0.192057	0.180212
31	O	-0.371745	-0.476620	-0.521431
32	C	0.254173	0.243238	0.263478
33	C	0.225313	0.188205	0.165942
34	C	0.227323	0.295801	0.397015
35	C	0.221550	0.192123	0.202018
36	C	0.180605	0.146102	0.153523
37	C	0.294196	0.270754	0.313195
38	C	0.367801	0.339558	0.386753
39	C	0.229805	0.190660	0.155261
40	H	-0.072939	-0.059876	-0.020004
41	H	-0.058399	-0.033008	-0.017646
42	H	-0.062007	-0.053177	-0.019326
43	H	-0.056212	-0.035518	-0.001964
44	H	-0.069170	-0.053328	-0.051850
45	H	-0.070529	-0.057575	-0.052226
46	H	-0.076350	-0.071549	-0.070081
47	H	-0.071532	-0.060909	-0.061416
48	H	-0.065239	-0.045426	-0.026791
49	H	-0.082305	-0.063793	-0.071636
50	H	-0.055784	-0.050718	-0.048563
51	H	-0.072460	-0.054485	-0.058927
52	H	-0.072080	-0.061805	-0.067233
53	H	-0.067885	-0.063994	-0.074425
54	H	-0.058289	-0.045782	-0.053271
55	H	-0.054426	-0.044349	-0.045766
56	H	-0.057754	-0.043112	-0.030468
57	H	-0.067477	-0.050501	-0.051285
58	H	-0.061953	-0.037121	-0.036739

59	H	-0.075517	-0.060697	-0.049779
60	H	-0.074834	-0.065417	-0.055450
61	H	-0.069102	-0.055599	-0.043435
62	H	-0.066678	-0.047888	-0.036180
63	H	-0.072744	-0.075529	-0.083742
64	H	-0.058671	-0.036964	-0.038867
65	H	-0.072984	-0.047075	-0.054227
66	H	-0.072560	-0.064716	-0.058954
67	H	-0.067906	-0.053346	-0.063526
68	H	-0.065684	-0.057136	-0.054788
69	C	0.351025	0.446784	0.475747
70	H	-0.064684	-0.053886	-0.076461
71	H	-0.067935	-0.067167	-0.093918
72	H	-0.063737	-0.048887	-0.069031
73	H	-0.080686	-0.064663	-0.073891
74	H	-0.072864	-0.060814	-0.067280
75	H	-0.069634	-0.069361	-0.071575
76	H	-0.075447	-0.045192	-0.035518
77	H	-0.066516	-0.054275	-0.042957
78	H	-0.065280	-0.061203	-0.032487
79	H	-0.083938	-0.064260	-0.050032
80	H	-0.066522	-0.058003	-0.053002
81	H	-0.067946	-0.066657	-0.056960
82	H	-0.070447	-0.045201	-0.038375
83	H	-0.062982	-0.023149	-0.019715
84	H	-0.046129	0.001241	-0.013382
85	H	-0.073128	-0.059777	-0.039999
86	H	0.063413	0.265254	0.311884
87	O	-0.362247	-0.440767	-0.459183
88	H	-0.045057	0.000825	0.021829
89	H	-0.046498	-0.032613	-0.031279

90	H	-0.052737	-0.052285	-0.057553
91	H	-0.042119	-0.012735	-0.025181
92	C	0.240267	0.197871	0.233816
93	O	-0.368059	-0.473936	-0.504031
94	H	-0.061648	-0.054729	-0.050560
95	H	-0.061348	-0.048368	-0.041288
96	H	-0.068462	-0.065487	-0.061774

*** Using NEW gradient routines ***

Geometry CYCLE 6

==============

Energy gradients wrt nuclear displacements

==

Atom		Cartesian (a.u./angstrom)		
		X	Y	Z
1	O	0.002403	0.002691	-0.000631
2	C	-0.002086	-0.000441	0.001563
3	C	0.000524	0.001980	-0.000624
4	C	-0.000786	0.000869	0.001082
5	O	-0.001268	-0.000268	0.001046
6	N	0.000487	-0.000035	-0.000091
7	O	0.000191	-0.000237	-0.002332
8	C	-0.002040	0.002226	0.001586
9	C	-0.000122	0.000299	-0.000472
10	C	0.003704	-0.001377	0.001485
11	O	0.000531	-0.000263	0.001981
12	C	-0.000335	-0.000873	-0.000781

13	O	-0.000935	0.000300	0.001522
14	C	-0.001639	-0.002050	0.000707
15	C	0.000387	0.001951	-0.002056
16	O	0.003159	-0.001101	-0.002004
17	C	-0.000155	-0.000616	0.000670
18	C	-0.000160	0.000291	0.000112
19	O	-0.001419	-0.001285	-0.003467
20	C	0.000768	0.001188	0.000515
21	C	0.000932	0.000566	0.000748
22	C	-0.001272	-0.000428	-0.001121
23	C	-0.000933	0.000496	0.000023
24	C	0.001332	0.000505	-0.000969
25	C	0.001693	-0.001046	0.000870
26	C	-0.000336	0.000559	0.000251
27	C	0.000369	-0.000112	0.000371
28	C	-0.000114	0.000264	-0.000100
29	C	-0.001821	-0.001074	0.000562
30	C	-0.000111	-0.000008	-0.001124
31	O	-0.003605	-0.002399	0.001338
32	C	-0.000878	-0.000544	-0.000955
33	C	0.000616	0.000522	-0.001925
34	C	0.003563	0.001402	-0.000436
35	C	-0.000886	-0.000112	0.001539
36	C	0.000805	-0.000583	0.000687
37	C	0.000153	-0.000514	0.000410
38	C	-0.000291	-0.000390	0.000140
39	C	-0.000611	0.000175	-0.000068
40	H	0.001382	-0.000461	-0.000735
41	H	-0.000862	-0.000240	-0.000369
42	H	-0.000156	0.000456	-0.000274
43	H	-0.000295	-0.000266	0.000639

44	H	-0.000472	-0.000512	-0.000220
45	H	-0.000024	-0.000144	0.000778
46	H	0.000656	-0.000387	-0.000427
47	H	0.000589	0.000657	-0.000101
48	H	-0.000459	-0.000322	-0.000019
49	H	-0.000595	0.000540	-0.000265
50	H	0.000140	-0.000734	0.000464
51	H	-0.000162	-0.000149	-0.000618
52	H	0.000289	-0.000525	0.001242
53	H	0.000201	-0.000524	0.000395
54	H	0.000348	-0.000512	-0.000113
55	H	0.001018	0.000827	0.000843
56	H	0.000793	-0.000280	0.000384
57	H	-0.000597	0.000471	-0.000064
58	H	0.000042	0.000403	-0.000561
59	H	0.000464	-0.000415	-0.000632
60	H	0.000389	-0.000192	0.000630
61	H	0.000186	0.000582	-0.000036
62	H	-0.000054	0.000627	0.000427
63	H	0.000157	-0.000312	0.000601
64	H	-0.000581	0.000132	0.000118
65	H	-0.000023	-0.000270	0.000914
66	H	0.000036	-0.000484	-0.001219
67	H	-0.000103	-0.000999	-0.000941
68	H	-0.000104	0.000292	-0.000285
69	C	0.003301	0.001644	0.000401
70	H	-0.000360	-0.000541	0.000402
71	H	-0.000007	-0.000290	-0.000551
72	H	0.000623	-0.000171	0.000251
73	H	0.000257	0.000335	-0.000426
74	H	-0.000280	-0.000506	0.000260

```
  75 H    -0.000040 -0.000280 -0.000712
  76 H    -0.000136  0.000492  0.001005
  77 H     0.000141 -0.000622  0.000324
  78 H    -0.000784  0.000066 -0.000188
  79 H     0.000145  0.000518  0.000765
  80 H     0.000419  0.000198 -0.000344
  81 H    -0.000580 -0.000056 -0.000446
  82 H     0.001182  0.000965 -0.000436
  83 H     0.000697  0.000462 -0.000678
  84 H    -0.000947 -0.001147  0.000353
  85 H     0.000512  0.000198 -0.000742
  86 H     0.000309 -0.000280 -0.001285
  87 O    -0.002520  0.003240 -0.000967
  88 H    -0.000438  0.000593 -0.000677
  89 H    -0.000313  0.000377  0.000435
  90 H     0.000117  0.000516 -0.000579
  91 H     0.000431  0.000122  0.000232
  92 C    -0.001246  0.000436  0.000290
  93 O    -0.003135 -0.002482  0.001764
  94 H    -0.000166 -0.001308  0.000624
  95 H     0.000398 -0.000536 -0.000285
  96 H     0.000402  0.000269  0.000603
 ----------------------------------------

 dE(predicted):    -0.55969E-03, dE(actual):     0.97020E-04, Trust radius:    0.195959, all in a.u.
 Interpolation step length   9.756715567837265E-002

---------------------------------------------------------------------
Geometry Convergence after Step   6       (Hartree/Angstrom,Angstrom)
---------------------------------------------------------------------
current energy                           -19.67374975 Hartree
```

energy change	0.00009702	0.00100000	T
constrained gradient max	0.00370402	0.00100000	F
constrained gradient rms	0.00100475	0.00066667	F
gradient max	0.00370402		
gradient rms	0.00100475		
cart. step max	0.19425655	0.01000000	F
cart. step rms	0.04831947	0.00666667	F

Coordinates (Cartesian)

=======================

Atom	bohr			angstrom			Geometric Variables		
	X	Y	Z	X	Y	Z	(0:frozen, *:LT par.)		
1 O	26.759982	61.490516	50.479133	14.160773	32.539380	26.712407	1	2	3
2 C	27.117823	61.933929	53.065048	14.350134	32.774024	28.080814	4	5	6
3 C	29.889863	61.283906	53.665336	15.817034	32.430047	28.398473	7	8	9
4 C	30.516541	61.854483	56.465176	16.148658	32.731983	29.880084	10	11	12
5 O	27.070298	52.870531	42.516298	14.324985	27.977880	22.498656	13	14	15
6 N	33.138816	61.358075	57.273281	17.536306	32.469295	30.307715	16	17	18
7 O	26.636341	64.551525	53.601469	14.095344	34.159196	28.364676	19	20	21
8 C	20.618738	58.344123	48.453078	10.910966	30.874380	25.640265	22	23	24
9 C	25.648573	65.413200	41.519044	13.572641	34.615175	21.970932	25	26	27
10 C	21.626096	55.057427	44.881979	11.444037	29.135136	23.750520	28	29	30
11 O	21.793330	62.654628	45.754884	11.532533	33.155401	24.212442	31	32	33
12 C	19.606921	56.534269	46.324041	10.375536	29.916647	24.513627	34	35	36
13 O	21.390779	55.278855	42.358049	11.319513	29.252310	22.414914	37	38	39
14 C	24.177453	61.404731	49.534871	12.794157	32.493984	26.212725	40	41	42
15 C	24.140596	63.151472	47.123712	12.774653	33.418320	24.936794	43	44	45

16	O	19.043685	59.347931	49.818873	10.077484	31.405572	26.363012	46	47	48
17	C	24.049244	56.820595	51.221237	12.726312	30.068164	27.105112	49	50	51
18	C	26.913140	53.315487	38.037407	14.241821	28.213340	20.128529	52	53	54
19	O	30.230046	58.582434	53.178352	15.997052	31.000489	28.140772	55	56	57
20	C	26.098121	57.117611	40.765635	13.810531	30.225338	21.572245	58	59	60
21	C	25.765505	54.249841	40.562071	13.634518	28.707780	21.464524	61	62	63
22	C	23.445443	58.619516	48.978625	12.406794	31.020112	25.918372	64	65	66
23	C	25.889362	62.670465	42.431553	13.700060	33.163782	22.453811	67	68	69
24	C	35.128812	62.379783	55.622007	18.589367	33.009960	29.433899	70	71	72
25	C	26.346035	62.360943	45.321106	13.941721	32.999990	23.982896	73	74	75
26	C	29.734548	64.614087	57.040811	15.734845	34.192302	30.184697	76	77	78
27	C	26.988319	65.157442	56.262743	14.281603	34.479833	29.772962	79	80	81
28	C	26.225283	67.919418	56.587509	13.877822	35.941408	29.944820	82	83	84
29	C	28.207234	58.427735	41.326723	14.926625	30.918626	21.869160	85	86	87
30	C	17.638150	57.905142	44.668590	9.333707	30.642082	23.637600	88	89	90
31	O	29.857344	62.453678	40.150561	15.799826	33.049063	21.246762	91	92	93
32	C	22.859378	53.642168	40.561531	12.096662	28.386213	21.464238	94	95	96
33	C	30.778834	57.364457	41.976615	16.287457	30.355963	22.213068	97	98	99
34	C	28.119294	61.294367	41.162342	14.880090	32.435582	21.782173	100	101	102
35	C	24.435865	65.928243	47.945632	12.930903	34.887724	25.371736	103	104	105
36	C	22.170645	50.847680	40.900943	11.732200	26.907434	21.643847	106	107	108
37	C	33.665515	58.733641	58.015202	17.815023	31.080504	30.700323	109	110	111
38	C	19.576441	64.071926	46.454149	10.359407	33.905403	24.582477	112	113	114
39	C	19.306698	50.337950	40.895427	10.216664	26.637696	21.640928	115	116	117
40	H	24.409096	58.190767	40.288509	12.916737	30.793228	21.319761	118	119	120
41	H	31.298162	57.919111	43.911370	16.562274	30.649473	23.236896	121	122	123

42	H	30.838090	55.310029	41.859252	16.318815	29.268807	22.150962	124	125	126
43	H	32.196018	58.205964	40.721159	17.037399	30.801270	21.548709	127	128	129
44	H	27.274680	66.545098	42.120071	14.433139	35.214149	22.288982	130	131	132
45	H	25.572642	65.488823	39.450714	13.532459	34.655193	20.876419	133	134	135
46	H	23.918897	66.266678	42.263926	12.657335	35.066816	22.365107	136	137	138
47	H	24.140598	61.653459	41.986246	12.774655	32.625606	22.218164	139	140	141
48	H	28.037011	63.423032	45.862937	14.836547	33.562023	24.269621	142	143	144
49	H	26.803726	60.376879	45.664965	14.183921	31.950068	24.164859	145	146	147
50	H	26.242510	66.140062	48.918460	13.886938	34.999813	25.886534	148	149	150
51	H	22.962636	66.500477	49.278693	12.151304	35.190537	26.077161	151	152	153
52	H	24.404460	67.195946	46.315931	12.914284	35.558563	24.509335	154	155	156
53	H	19.800986	66.091753	46.043140	10.478230	34.974249	24.364981	157	158	159
54	H	19.071842	63.805839	48.448771	10.092384	33.764596	25.637985	160	161	162
55	H	18.045841	63.320023	45.290536	9.549448	33.507513	23.966719	163	164	165
56	H	22.088638	54.330883	38.767351	11.688804	28.750665	20.514798	166	167	168
57	H	23.053621	49.822064	39.330695	12.199451	26.364701	20.812908	169	170	171
58	H	23.034099	50.125942	42.632508	12.189120	26.525506	22.560152	172	173	174
59	H	18.371259	51.239465	42.507948	9.721652	27.114757	22.494237	175	176	177
60	H	18.409355	51.053594	39.167498	9.741811	27.016398	20.726547	178	179	180

61 H	18.938412	48.303146	41.011726	10.021776	25.560924	21.702471	181	182	183
62 H	26.689883	51.267248	37.848111	14.123678	27.129459	20.028358	184	185	186
63 H	26.030144	54.259513	36.420674	13.774559	28.712898	19.272991	187	188	189
64 H	28.931695	53.753729	38.017943	15.309994	28.445248	20.118229	190	191	192
65 H	24.533484	57.972054	47.350216	12.982561	30.677490	25.056655	193	194	195
66 H	22.963348	57.367800	52.897520	12.151681	30.357733	27.992162	196	197	198
67 H	26.062152	56.889170	51.667318	13.791497	30.104452	27.341167	199	200	201
68 H	23.557572	54.878184	50.713581	12.466130	29.040284	26.836471	202	203	204
69 C	31.546381	57.886251	51.039775	16.693626	30.632085	27.009086	205	206	207
70 H	27.433348	69.135898	55.424316	14.517103	36.585142	29.329285	208	209	210
71 H	26.420092	68.493441	58.568833	13.980910	36.245168	30.993292	211	212	213
72 H	24.258159	68.200139	56.005875	12.836865	36.089959	29.637033	214	215	216
73 H	25.706667	63.944810	57.384791	13.603383	33.838136	30.366724	217	218	219
74 H	30.969833	65.932907	56.022416	16.388530	34.890192	29.645786	220	221	222
75 H	29.989235	64.956862	59.068645	15.869620	34.373691	31.257781	223	224	225
76 H	35.353366	61.326181	53.831675	18.708195	32.452418	28.486495	226	227	228
77 H	34.740624	64.357988	55.159505	18.383947	34.056781	29.189153	229	230	231
78 H	36.934299	62.317957	56.637138	19.544789	32.977243	29.971083	232	233	234
79 H	33.787625	57.405798	56.408093	17.879641	30.377840	29.849877	235	236	237

80 H 32.185565 58.057078 59.296039 17.031867 30.722483 31.378112 238 239 240

81 H 35.469906 58.674477 59.036282 18.769866 31.049196 31.240655 241 242 243

82 H 25.836285 60.757894 54.224617 13.671973 32.151693 28.694432 244 245 246

83 H 22.886279 62.180751 50.957107 12.110897 32.904636 26.965340 247 248 249

84 H 31.087489 62.361006 52.372033 16.450791 33.000023 27.714086 250 251 252

85 H 29.318423 60.607665 57.616684 15.514642 32.072195 30.489436 253 254 255

86 H 26.069824 53.067463 44.068618 13.795557 28.082092 23.320108 256 257 258

87 O 23.229873 53.756137 45.951989 12.292719 28.446523 24.316745 259 260 261

88 H 18.628208 55.065790 47.428216 9.857623 29.139561 25.097931 262 263 264

89 H 33.078430 54.374034 49.745272 17.504351 28.773500 26.324064 265 266 267

90 H 30.932886 54.046665 52.367431 16.368979 28.600263 27.711651 268 269 270

91 H 29.778715 54.729110 49.286721 15.758217 28.961398 26.081409 271 272 273

92 C 31.336452 55.063033 50.615287 16.582536 29.138102 26.784456 274 275 276

93 O 32.677480 59.343244 49.668256 17.292178 31.403092 26.283309 277 278 279

94 H 18.560266 59.317172 43.476434 9.821670 31.389296 23.006738 280 281 282

95 H 16.263151 58.841869 45.894226 8.606089 31.137776 24.286179 283 284 285

96 H 16.648946 56.560829 43.448879 8.810243 29.930702 22.992156 286 287 288

Number of elements of the density matrix on this node (used, total): 222269 1773786

Numerical Integration : Fuzzy Cells (Becke)

Becke grid quality: NORMAL

Lebedev angular grid order: min: 11 max: 23

Nr. of radial points: min: 46 max: 52

Total nr. of points: 600198

Nr. of blocks: 4690

Block length: 128

Nr. of dummy points: 122

Test of Precision of the Numerical Integration Grid

===

Integral of the Total Core Density: 85.99998731868342

Relative Error: -1.475E-07

Density Fitting (zlmFit)

ZlmFit Fit Quality: NORMAL

Max L-expansion: min: 6 max: 7

Nr. of radial interpolation points: min: 39 max: 48

S C F *** ScaSCF ***

Initial density matrix is taken from previous geometry

CYCLE 1

SCF Error: norm([F,P])= 0.178988243001, max([F,P])= 0.018952349011

orbitals (Q,E):

```
A :114...133   ( 2.00  -0.2575) ( 2.00  -0.2531) ( 2.00  -0.2464) ( 2.00  -0.2414)
               ( 2.00  -0.2360) ( 2.00  -0.2296) ( 2.00  -0.2263) ( 2.00  -0.2229)
               ( 2.00  -0.2024) ( 2.00  -0.1840) ( 0.00  -0.0772) ( 0.00  -0.0763)
               ( 0.00  -0.0688) ( 0.00  -0.0496) ( 0.00  -0.0241) ( 0.00  -0.0189)
               ( 0.00  -0.0179) ( 0.00  -0.0149) ( 0.00  -0.0111) ( 0.00  -0.0056)
```

CYCLE 2

SDIIS (wt 0.000): 0.0637 0.9363

A-DIIS (wt 1.000): 0.0000 1.0000

DIIS coefficients: 0.0000 1.0000

SCF Error: norm([F,P])= 0.041226701692, max([F,P])= 0.003000837249

orbitals (Q,E):

```
A :114...124   ( 2.00  -0.2455) ( 2.00  -0.2439) ( 2.00  -0.2331) ( 2.00  -0.2308)
               ( 2.00  -0.2240) ( 2.00  -0.2182) ( 2.00  -0.2175) ( 2.00  -0.2124)
```

(2.00 -0.1943) (2.00 -0.1739) (0.00 -0.0672)

CYCLE 3

SDIIS (wt 0.000): 0.0048 0.6559 0.3393

A-DIIS (wt 1.000): 0.0000 0.7180 0.2820

DIIS coefficients: 0.0000 0.7180 0.2820

SCF Error: norm([F,P])= 0.076719177918, max([F,P])= 0.007031678682

orbitals (Q,E):

A :114...124 (2.00 -0.2479) (2.00 -0.2466) (2.00 -0.2349) (2.00 -0.2337)

(2.00 -0.2264) (2.00 -0.2208) (2.00 -0.2193) (2.00 -0.2144)

(2.00 -0.1944) (2.00 -0.1758) (0.00 -0.0691)

CYCLE 4

SDIIS (wt 0.983): 0.4225 0.1908 0.3867

A-DIIS (wt 0.017): 0.0833 0.0000 0.9167

DIIS coefficients: 0.4166 0.1875 0.3959

SCF Error: norm([F,P])= 0.019776946436, max([F,P])= 0.002711376468

orbitals (Q,E):

A :114...124 (2.00 -0.2490) (2.00 -0.2469) (2.00 -0.2363) (2.00 -0.2349)

(2.00 -0.2277) (2.00 -0.2218) (2.00 -0.2198) (2.00 -0.2156)

(2.00 -0.1959) (2.00 -0.1766) (0.00 -0.0700)

CYCLE 5

SDIIS (wt 1.000): 0.1549 0.0604 0.2394 0.5453

DIIS coefficients: 0.1549 0.0604 0.2394 0.5453

SCF Error: norm([F,P])= 0.005598523122, max([F,P])= 0.000880357129

orbitals (Q,E):

A :114...124 (2.00 -0.2489) (2.00 -0.2470) (2.00 -0.2363) (2.00 -0.2349)

(2.00 -0.2277) (2.00 -0.2217) (2.00 -0.2198) (2.00 -0.2155)

(2.00 -0.1956) (2.00 -0.1766) (0.00 -0.0699)

CYCLE 6

SDIIS (wt 1.000): 0.0690 0.0249 0.1251 0.3714 0.4096

DIIS coefficients: 0.0690 0.0249 0.1251 0.3714 0.4096

SCF Error: norm([F,P])= 0.002419606586, max([F,P])= 0.000312425258

orbitals (Q,E):

A :114...124 (2.00 -0.2490) (2.00 -0.2469) (2.00 -0.2362) (2.00 -0.2349)

(2.00 -0.2276) (2.00 -0.2217) (2.00 -0.2198) (2.00 -0.2155)

(2.00 -0.1957) (2.00 -0.1766) (0.00 -0.0699)

CYCLE 7

SDIIS (wt 1.000): 0.0248 0.0084 0.0497 0.1732 0.2759 0.4679

DIIS coefficients: 0.0248 0.0084 0.0497 0.1732 0.2759 0.4679

SCF Error: norm([F,P])= 0.000786002128, max([F,P])= 0.000128080436

orbitals (Q,E):

A :114...124 (2.00 -0.2490) (2.00 -0.2469) (2.00 -0.2362) (2.00 -0.2349)

(2.00 -0.2276) (2.00 -0.2217) (2.00 -0.2198) (2.00 -0.2155)

(2.00 -0.1957) (2.00 -0.1766) (0.00 -0.0699)

CYCLE 8

SDIIS (wt 1.000): 0.0073 0.0021 0.0173 0.0725 0.1471 0.3594 0.3942

DIIS coefficients: 0.0073 0.0021 0.0173 0.0725 0.1471 0.3594 0.3942

SCF Error: norm([F,P])= 0.000406571335, max([F,P])= 0.000059780650

orbitals (Q,E):

A :114...124 (2.00 -0.2490) (2.00 -0.2469) (2.00 -0.2362) (2.00 -0.2349)

(2.00 -0.2276) (2.00 -0.2217) (2.00 -0.2198) (2.00 -0.2155)

(2.00 -0.1957) (2.00 -0.1766) (0.00 -0.0699)

CYCLE 9

SDIIS (wt 1.000): 0.0018 0.0004 0.0056 0.0272 0.0662 0.1887 0.2799 0.4304

DIIS coefficients: 0.0018 0.0004 0.0056 0.0272 0.0662 0.1887 0.2799 0.4304

SCF Error: norm([F,P])= 0.000129693398, max([F,P])= 0.000017652872

orbitals (Q,E):

A :114...124 (2.00 -0.2490) (2.00 -0.2469) (2.00 -0.2362) (2.00 -0.2349)

(2.00 -0.2276) (2.00 -0.2217) (2.00 -0.2198) (2.00 -0.2155)

(2.00 -0.1957) (2.00 -0.1766) (0.00 -0.0699)

CYCLE 10

SDIIS (wt 1.000): 0.0001 -0.0001 0.0010 0.0062 0.0198 0.0663 0.1260 0.3005 0.4803

DIIS coefficients: 0.0001 -0.0001 0.0010 0.0062 0.0198 0.0663 0.1260 0.3005 0.4803

SCF Error: norm([F,P])= 0.000047176485, max([F,P])= 0.000005485969

orbitals (Q,E):

A :114...124 (2.00 -0.2490) (2.00 -0.2469) (2.00 -0.2362) (2.00 -0.2349)

(2.00 -0.2276) (2.00 -0.2217) (2.00 -0.2198) (2.00 -0.2155)

(2.00 -0.1957) (2.00 -0.1766) (0.00 -0.0699)

CYCLE 11

SDIIS (wt 1.000): -0.0001 -0.0001 0.0002 0.0018 0.0078 0.0298 0.0643 0.1760 0.3451 0.3752

DIIS coefficients: -0.0001 -0.0001 0.0002 0.0018 0.0078 0.0298 0.0643 0.1760 0.3451 0.3752

SCF Error: norm([F,P])= 0.000019355732, max([F,P])= 0.000003589303

orbitals (Q,E):

A :114...124 (2.00 -0.2490) (2.00 -0.2469) (2.00 -0.2362) (2.00 -0.2349)

(2.00 -0.2276) (2.00 -0.2217) (2.00 -0.2198) (2.00 -0.2155)

(2.00 -0.1957) (2.00 -0.1766) (0.00 -0.0699)

CYCLE 12

SDIIS (wt 1.000): -0.0000 0.0001 0.0007 0.0026 0.0100 0.0228 0.0666 0.1479 0.2568 0.4925

DIIS coefficients: -0.0000 0.0001 0.0007 0.0026 0.0100 0.0228 0.0666 0.1479 0.2568 0.4925

SCF Error: norm([F,P])= 0.000006070168, max([F,P])= 0.000000846135

orbitals (Q,E):

A :114...124 (2.00 -0.2490) (2.00 -0.2469) (2.00 -0.2362) (2.00 -0.2349)

(2.00 -0.2276) (2.00 -0.2217) (2.00 -0.2198) (2.00 -0.2155)

(2.00 -0.1957) (2.00 -0.1766) (0.00 -0.0699)

SCF CONVERGED

CYCLE 13

*** Setting up for NEW gradients in Focky

*** Using FIT density in Focky

*** Setting up for NEW Hessian in Focky

orbitals (Q,E):

A :114...133 (2.00 -0.2490) (2.00 -0.2469) (2.00 -0.2362) (2.00 -0.2349)

(2.00 -0.2276) (2.00 -0.2217) (2.00 -0.2198) (2.00 -0.2155)

(2.00 -0.1957) (2.00 -0.1766) (0.00 -0.0699) (0.00 -0.0692)

(0.00 -0.0625) (0.00 -0.0394) (0.00 -0.0189) (0.00 -0.0132)

(0.00 -0.0117) (0.00 -0.0086) (0.00 -0.0051) (0.00 -0.0003)

Fit test: (difference of exact and fit density, squared integrated, result summed over spins)

SCF: 0.00100620733010

Gross Charges per Atom (Z minus electrons)

==

-0.6615	0.9265	0.5397	0.3341	-0.5808	-0.3839	-0.6348	0.6706	0.6302	0.8622
-0.6597	-0.0007	-0.5777	0.5894	0.3267	-0.5907	0.6006	0.6016	-0.5967	0.2563
0.3413	0.0975	0.1665	0.7473	0.4083	0.4075	0.5641	0.5730	0.0299	0.6419
-0.5654	0.5487	0.5502	0.5714	0.6517	0.3733	0.7514	0.9843	0.5882	-0.2128
-0.1841	-0.2065	-0.1919	-0.2133	-0.2436	-0.2328	-0.1471	-0.1870	-0.1425	-0.2103
-0.2402	-0.2220	-0.2255	-0.1845	-0.2436	-0.1717	-0.1949	-0.1551	-0.1985	-0.2145
-0.2236	-0.2175	-0.2282	-0.1954	-0.1203	-0.2262	-0.1443	-0.2212	0.8273	-0.1968
-0.2182	-0.2169	-0.2120	-0.1909	-0.2116	-0.1759	-0.2278	-0.2346	-0.1986	-0.2417
-0.2127	-0.2526	-0.1967	-0.1602	-0.1642	0.2420	-0.6729	-0.1256	-0.1866	-0.1946
-0.1139	0.4911	-0.6108	-0.1741	-0.2150	-0.2392				

Net Total: 0.00000000

M U L T I P O L E D E R I V E D C H A R G E A N A L Y S I S

See: M. Swart, P.Th. van Duijnen, J.G. Snijders, J.Comput.Chem., (2001), p. 79-88.

Multipole derived atomic charges (a.u.)

Atom	Level:	MDC-m	MDC-d	MDC-q
1	O	-0.352114	-0.341199	-0.364959
2	C	0.363610	0.377053	0.399889
3	C	0.242832	0.251040	0.255202
4	C	0.180722	0.164968	0.210354
5	O	-0.307417	-0.550097	-0.620966

6	N	-0.214780	-0.272780	-0.461163
7	O	-0.379465	-0.372112	-0.440213
8	C	0.250645	0.308497	0.307805
9	C	0.228157	0.193491	0.185627
10	C	0.340342	0.409570	0.416101
11	O	-0.383057	-0.353416	-0.394784
12	C	0.124852	0.102341	0.102622
13	O	-0.313142	-0.299654	-0.348499
14	C	0.245086	0.245280	0.250350
15	C	0.195792	0.158646	0.175431
16	O	-0.371354	-0.479083	-0.484813
17	C	0.228715	0.193994	0.195984
18	C	0.229887	0.193096	0.194914
19	O	-0.318072	-0.319513	-0.387815
20	C	0.059108	0.047278	-0.061516
21	C	0.195275	0.222116	0.283981
22	C	0.119207	0.105110	0.109194
23	C	0.119881	0.099341	0.109150
24	C	0.290610	0.267378	0.287932
25	C	0.172524	0.135857	0.138811
26	C	0.164920	0.124570	0.165007
27	C	0.249179	0.237160	0.259494
28	C	0.234561	0.193714	0.292111
29	C	0.033579	0.026343	-0.054550
30	C	0.236944	0.191551	0.179591
31	O	-0.372999	-0.476463	-0.520049
32	C	0.254353	0.243875	0.264222
33	C	0.224946	0.187659	0.170803
34	C	0.227844	0.295081	0.393380
35	C	0.222122	0.192042	0.202266
36	C	0.180577	0.145578	0.152165

37	C	0.294170	0.270442	0.312083
38	C	0.367958	0.339089	0.386234
39	C	0.229619	0.190136	0.152595
40	H	-0.072773	-0.060066	-0.020419
41	H	-0.058930	-0.033521	-0.019119
42	H	-0.062078	-0.053448	-0.021125
43	H	-0.056138	-0.034904	-0.003789
44	H	-0.068865	-0.052656	-0.050747
45	H	-0.070387	-0.057366	-0.051343
46	H	-0.076488	-0.071547	-0.069274
47	H	-0.071323	-0.060402	-0.060697
48	H	-0.064855	-0.044905	-0.025872
49	H	-0.082257	-0.063631	-0.071306
50	H	-0.055648	-0.048020	-0.047740
51	H	-0.072230	-0.054850	-0.059571
52	H	-0.071013	-0.059510	-0.065896
53	H	-0.067702	-0.063459	-0.073596
54	H	-0.058316	-0.045956	-0.052799
55	H	-0.054367	-0.044108	-0.045615
56	H	-0.057632	-0.043104	-0.030560
57	H	-0.067092	-0.049593	-0.050040
58	H	-0.062066	-0.036853	-0.035552
59	H	-0.075421	-0.060757	-0.049155
60	H	-0.074754	-0.065261	-0.054837
61	H	-0.068906	-0.055041	-0.042271
62	H	-0.066530	-0.047524	-0.035565
63	H	-0.072777	-0.075631	-0.083438
64	H	-0.058480	-0.036622	-0.037553
65	H	-0.073203	-0.047697	-0.054758
66	H	-0.072345	-0.063563	-0.058529
67	H	-0.067514	-0.053165	-0.062787

68	H	-0.065886	-0.057633	-0.055622
69	C	0.351834	0.444376	0.472452
70	H	-0.064652	-0.054116	-0.078728
71	H	-0.067965	-0.067063	-0.095444
72	H	-0.063592	-0.048739	-0.070674
73	H	-0.080621	-0.063021	-0.073686
74	H	-0.072817	-0.060594	-0.066031
75	H	-0.069621	-0.069258	-0.071741
76	H	-0.075332	-0.045068	-0.035498
77	H	-0.066490	-0.054187	-0.041699
78	H	-0.065342	-0.061297	-0.032265
79	H	-0.083822	-0.064561	-0.049072
80	H	-0.066548	-0.057998	-0.052697
81	H	-0.067840	-0.066458	-0.056652
82	H	-0.070891	-0.043588	-0.037215
83	H	-0.062373	-0.023279	-0.019255
84	H	-0.046058	0.000744	-0.013459
85	H	-0.073160	-0.059555	-0.040319
86	H	0.063124	0.264822	0.310423
87	O	-0.362953	-0.440925	-0.459297
88	H	-0.044695	0.001453	0.022625
89	H	-0.046192	-0.031899	-0.028372
90	H	-0.052434	-0.051387	-0.054038
91	H	-0.041861	-0.012138	-0.023842
92	C	0.240454	0.197634	0.225411
93	O	-0.369112	-0.474230	-0.503444
94	H	-0.061332	-0.054108	-0.049672
95	H	-0.060779	-0.046962	-0.040184
96	H	-0.068573	-0.065780	-0.062025

*** Using NEW gradient routines ***

Geometry CYCLE 7

==============

Energy gradients wrt nuclear displacements

==

Atom	Cartesian (a.u./angstrom)		
	X	Y	Z
1 O	0.001457	-0.002279	0.000749
2 C	0.000171	0.000677	0.000251
3 C	-0.000592	0.000634	0.000148
4 C	-0.000681	0.000880	0.000234
5 O	-0.001643	-0.000070	0.001254
6 N	-0.000031	0.000173	0.000458
7 O	0.000590	-0.000395	0.001837
8 C	0.002562	-0.001937	-0.002329
9 C	-0.000115	0.000005	-0.000087
10 C	-0.001186	0.002925	0.000131
11 O	0.000504	0.000099	0.000046
12 C	-0.000611	-0.000969	-0.000843
13 O	0.001382	0.000823	-0.001318
14 C	-0.000643	-0.000108	-0.001394
15 C	-0.001044	0.000907	-0.000141
16 O	-0.003919	0.002161	0.003354
17 C	-0.000002	0.000080	0.000778
18 C	-0.000140	-0.000153	0.000124
19 O	-0.001726	-0.000561	-0.001249
20 C	-0.002083	-0.001278	-0.000660
21 C	-0.000057	0.000900	-0.000468

22	C	0.000826	0.001001	-0.000510
23	C	-0.001110	0.000005	-0.000261
24	C	0.000888	0.000299	-0.000774
25	C	-0.000091	-0.000101	0.000511
26	C	-0.000425	-0.000042	-0.000055
27	C	0.000304	-0.000092	0.000079
28	C	-0.000028	0.000099	0.000154
29	C	0.000806	0.000448	-0.000189
30	C	-0.000035	0.000767	0.000297
31	O	0.003148	0.002911	-0.003470
32	C	-0.000934	-0.000652	0.000431
33	C	0.000470	0.000582	-0.000089
34	C	-0.000650	-0.001916	0.004006
35	C	0.000973	0.000784	-0.000580
36	C	0.000732	-0.000615	-0.000228
37	C	0.000411	-0.000503	0.000143
38	C	-0.000795	-0.000663	-0.000629
39	C	-0.000307	-0.000417	-0.000012
40	H	-0.000422	-0.000018	0.000494
41	H	-0.000233	-0.000495	0.000143
42	H	0.000246	0.000141	0.000100
43	H	-0.000166	0.000071	0.000175
44	H	0.000092	-0.000017	-0.000021
45	H	-0.000016	-0.000002	0.000018
46	H	0.000174	-0.000047	-0.000116
47	H	-0.000246	0.000002	-0.000240
48	H	-0.000055	0.000196	0.000012
49	H	-0.000448	0.000128	-0.000012
50	H	-0.000563	0.000106	-0.000456
51	H	0.000473	0.000038	-0.000167
52	H	0.000102	0.000167	0.000141

53	H	-0.000379	-0.000195	-0.000043
54	H	-0.000112	-0.000134	-0.000065
55	H	-0.000231	-0.000379	-0.000216
56	H	0.000564	-0.000011	-0.000144
57	H	-0.000429	-0.000168	-0.000427
58	H	0.000244	0.000151	-0.000174
59	H	0.000032	-0.000334	-0.000196
60	H	0.000033	0.000023	0.000063
61	H	-0.000067	-0.000056	0.000001
62	H	-0.000012	-0.000192	0.000104
63	H	-0.000042	-0.000095	0.000054
64	H	-0.000057	0.000068	-0.000226
65	H	0.000378	0.000068	0.000466
66	H	0.000041	0.000082	-0.000114
67	H	0.001012	0.000153	-0.000068
68	H	0.000030	-0.000108	0.000040
69	C	-0.001131	-0.003438	0.004597
70	H	0.000011	-0.000019	-0.000023
71	H	0.000036	0.000053	0.000004
72	H	-0.000054	0.000021	0.000029
73	H	-0.000069	-0.000038	-0.000111
74	H	0.000027	-0.000101	0.000033
75	H	0.000019	-0.000003	0.000022
76	H	0.000133	0.000157	0.000344
77	H	0.000032	0.000086	0.000083
78	H	-0.000079	0.000047	0.000067
79	H	0.000191	-0.000010	0.000581
80	H	0.000014	-0.000099	0.000070
81	H	0.000029	-0.000097	-0.000041
82	H	0.000246	-0.000382	-0.000157
83	H	0.000143	-0.000319	-0.000036

```
  84 H    -0.000407 -0.000382 -0.000330
  85 H     0.000118 -0.000109 -0.000126
  86 H     0.000039 -0.001120  0.000081
  87 O     0.003648 -0.002015  0.000097
  88 H    -0.000534  0.000496 -0.000076
  89 H     0.000180 -0.000017  0.000149
  90 H    -0.000043 -0.000116 -0.000037
  91 H     0.000210 -0.000124 -0.000127
  92 C    -0.001121 -0.000350  0.000067
  93 O     0.002463  0.003789 -0.004474
  94 H    -0.000119  0.000331  0.000232
  95 H    -0.000238  0.000185  0.000048
  96 H    -0.000060  0.000019  0.000207
 ----------------------------------------

 dE(predicted):    -0.55729E-03, dE(actual):    -0.37645E-03, Trust radius:    0.195959, all in a.u.

----------------------------------------------------------------------
Geometry Convergence after Step   7       (Hartree/Angstrom,Angstrom)
----------------------------------------------------------------------
current energy                             -19.67412620 Hartree
energy change                       -0.00037645     0.00100000    T
constrained gradient max             0.00459723     0.00100000    F
constrained gradient rms             0.00096232     0.00066667    F
gradient max                         0.00459723
gradient rms                         0.00096232
cart. step max                       0.04546806     0.01000000    F
cart. step rms                       0.01478888     0.00666667    F
```

Coordinates (Cartesian)

======================

Atom	bohr			angstrom			Geometric Variables		
	X	Y	Z	X	Y	Z	(0:frozen, *:LT par.)		
1 O	26.747663	61.452589	50.480780	14.154254	32.519310	26.713278	1	2	3
2 C	27.113421	61.913481	53.060687	14.347805	32.763203	28.078506	4	5	6
3 C	29.890817	61.284133	53.653234	15.817539	32.430167	28.392068	7	8	9
4 C	30.515540	61.860878	56.449294	16.148129	32.735367	29.871680	10	11	12
5 O	27.098244	52.889947	42.521545	14.339773	27.988154	22.501432	13	14	15
6 N	33.138380	61.361214	57.246061	17.536075	32.470956	30.293311	16	17	18
7 O	26.621769	64.532465	53.580247	14.087634	34.149110	28.353445	19	20	21
8 C	20.589894	58.341826	48.442683	10.895703	30.873165	25.634764	22	23	24
9 C	25.671438	65.426489	41.570706	13.584740	34.622207	21.998270	25	26	27
10 C	21.580126	55.024267	44.894825	11.419711	29.117588	23.757318	28	29	30
11 O	21.800182	62.671567	45.762201	11.536160	33.164365	24.216314	31	32	33
12 C	19.572379	56.535302	46.314846	10.357257	29.917194	24.508761	34	35	36
13 O	21.387469	55.269515	42.370353	11.317761	29.247368	22.421425	37	38	39
14 C	24.164953	61.374647	49.543741	12.787543	32.478065	26.217419	40	41	42
15 C	24.148418	63.130230	47.141426	12.778792	33.407079	24.946168	43	44	45
16 O	19.019343	59.369274	49.789502	10.064603	31.416867	26.347470	46	47	48
17 C	23.991028	56.788643	51.225090	12.695505	30.051256	27.107150	49	50	51
18 C	26.919601	53.329823	38.044396	14.245239	28.220927	20.132228	52	53	54
19 O	30.260644	58.587721	53.166388	16.013243	31.003287	28.134441	55	56	57
20 C	26.104610	57.126586	40.773979	13.813965	30.230087	21.576661	58	59	60
21 C	25.776384	54.259264	40.572599	13.640275	28.712766	21.470095	61	62	63
22 C	23.415007	58.594850	48.982013	12.390688	31.007059	25.920165	64	65	66
23 C	25.900449	62.673737	42.454100	13.705928	33.165513	22.465742	67	68	69
24 C	35.116761	62.373444	55.577378	18.582990	33.006605	29.410282	70	71	72
25 C	26.351945	62.335235	45.339904	13.944849	32.986386	23.992844	73	74	75

26 C	29.730767	64.621850	57.013567	15.732845	34.196410	30.170280	76	77	78
27 C	26.981755	65.156322	56.236649	14.278130	34.479241	29.759153	79	80	81
28 C	26.215442	67.918735	56.544912	13.872614	35.941047	29.922279	82	83	84
29 C	28.211348	58.433953	41.339061	14.928802	30.921916	21.875689	85	86	87
30 C	17.638990	57.924529	44.634463	9.334151	30.652341	23.619541	88	89	90
31 O	29.877804	62.457987	40.197411	15.810653	33.051343	21.271554	91	92	93
32 C	22.871635	53.642231	40.578784	12.103148	28.386246	21.473368	94	95	96
33 C	30.776337	57.366515	42.005492	16.286136	30.357052	22.228349	97	98	99
34 C	28.125183	61.299571	41.176489	14.883206	32.438336	21.789659	100	101	102
35 C	24.465170	65.896811	47.985577	12.946410	34.871091	25.392874	103	104	105
36 C	22.186522	50.849014	40.925347	11.740602	26.908139	21.656761	106	107	108
37 C	33.657430	58.732459	57.973895	17.810745	31.079879	30.678464	109	110	111
38 C	19.610413	64.130590	46.458846	10.377384	33.936447	24.584963	112	113	114
39 C	19.322880	50.341965	40.907183	10.225228	26.639821	21.647149	115	116	117
40 H	24.416338	58.198796	40.293591	12.920570	30.797477	21.322450	118	119	120
41 H	31.295097	57.938746	43.935051	16.560652	30.659864	23.249428	121	122	123
42 H	30.827038	55.311056	41.907716	16.312966	29.269350	22.176608	124	125	126
43 H	32.199001	58.189748	40.744446	17.038977	30.792689	21.561032	127	128	129
44 H	27.300484	66.545634	42.186210	14.446794	35.214433	22.323981	130	131	132
45 H	25.598630	65.524630	39.503517	13.546211	34.674141	20.904361	133	134	135
46 H	23.943900	66.277701	42.322805	12.670566	35.072649	22.396264	136	137	138
47 H	24.147841	61.668644	41.998186	12.778487	32.633641	22.224483	139	140	141

48	H	28.049038	63.380609	45.893798	14.842912	33.539574	24.285952	142	143 144
49	H	26.795953	60.345545	45.668433	14.179808	31.933487	24.166694	145	146 147
50	H	26.272009	66.085848	48.963194	13.902548	34.971125	25.910207	148	149 150
51	H	22.994341	66.469900	49.320696	12.168081	35.174356	26.099388	151	152 153
52	H	24.446352	67.176247	46.365132	12.936453	35.548139	24.535371	154	155 156
53	H	19.873666	66.144838	46.044718	10.516691	35.002341	24.365816	157	158 159
54	H	19.100816	63.877268	48.453377	10.107717	33.802395	25.640423	160	161 162
55	H	18.066655	63.405945	45.296129	9.560462	33.552981	23.969679	163	164 165
56	H	22.097500	54.324967	38.783978	11.693493	28.747534	20.523597	166	167 168
57	H	23.079798	49.816927	39.365817	12.213303	26.361982	20.831493	169	170 171
58	H	23.039668	50.136276	42.665301	12.192067	26.530975	22.577505	172	173 174
59	H	18.380290	51.254726	42.509096	9.726431	27.122833	22.494845	175	176 177
60	H	18.436090	51.048663	39.170436	9.755958	27.013789	20.728102	178	179 180
61	H	18.951652	48.308698	41.033233	10.028782	25.563862	21.713852	181	182 183
62	H	26.699485	51.281942	37.852376	14.128759	27.137235	20.030615	184	185 186
63	H	26.031678	54.274658	36.431178	13.775371	28.720912	19.278549	187	188 189
64	H	28.937030	53.771349	38.021587	15.312817	28.454573	20.120157	190	191 192
65	H	24.503411	57.940942	47.356469	12.966647	30.661026	25.059964	193	194 195
66	H	22.899961	57.343307	52.895304	12.118138	30.344771	27.990989	196	197 198

67 H 26.001086 56.840252 51.683928 13.759182 30.078566 27.349957 199 200
201

68 H 23.486173 54.851481 50.712648 12.428347 29.026154 26.835978 202 203
204

69 C 31.574948 57.916613 51.019075 16.708743 30.648152 26.998132 205 206
207

70 H 27.419739 69.129096 55.372056 14.509901 36.581542 29.301630 208 209
210

71 H 26.412728 68.505482 58.521919 13.977014 36.251540 30.968466 211 212
213

72 H 24.247336 68.192705 55.964632 12.831138 36.086025 29.615208 214 215
216

73 H 25.705124 63.949537 57.370168 13.602566 33.840638 30.358985 217 218
219

74 H 30.961772 65.937876 55.986867 16.384264 34.892821 29.626974 220 221
222

75 H 29.987866 64.975024 59.038978 15.868895 34.383302 31.242082 223 224
225

76 H 35.318657 61.317767 53.785470 18.689828 32.447965 28.462045 226 227
228

77 H 34.732506 64.352526 55.117181 18.379651 34.053890 29.166756 229 230
231

78 H 36.931408 62.303847 56.574863 19.543260 32.969776 29.938128 232 233
234

79 H 33.761161 57.412591 56.358784 17.865637 30.381435 29.823784 235 236
237

80 H 32.182432 58.057909 59.260877 17.030209 30.722922 31.359506 238 239
240

81 H 35.468033 58.660167 58.982371 18.768875 31.041624 31.212127 241 242
243

82 H 25.842386 60.739707 54.233677 13.675202 32.142069 28.699226 244 245
246

83 H 22.877963 62.157911 50.965596 12.106496 32.892550 26.969832 247 248
249

84 H 31.077721 62.373000 52.359931 16.445622 33.006370 27.707682 250 251
252

85 H 29.319835 60.616682 57.605781 15.515389 32.076967 30.483667 253 254
255

86 H 26.114973 53.101126 44.082457 13.819449 28.099906 23.327432 256 257
258

87 O 23.146314 53.696450 45.981689 12.248502 28.414938 24.332462 259 260
261

88 H 18.566465 55.084114 47.417165 9.824950 29.149258 25.092083 262 263
264

89 H 33.142106 54.430739 49.697671 17.538047 28.803507 26.298875 265 266
267

90 H 31.010015 54.063788 52.325416 16.409793 28.609324 27.689417 268 269
270

91 H 29.837067 54.752055 49.253745 15.789096 28.973540 26.063959 271 272
273

92 C 31.396136 55.094558 50.578200 16.614120 29.154784 26.764831 274 275
276

93 O 32.697623 59.389780 49.665182 17.302837 31.427718 26.281682 277 278
279

94 H 18.592907 59.316827 43.444486 9.838943 31.389113 22.989832 280 281
282

95 H 16.266446 58.886711 45.842185 8.607833 31.161506 24.258640 283 284
285

96 H 16.643206 56.588356 43.411592 8.807205 29.945268 22.972425 286 287
288

Number of elements of the density matrix on this node (used, total): 222269 1773786

Numerical Integration : Fuzzy Cells (Becke)

Becke grid quality: NORMAL

Lebedev angular grid order: min: 11 max: 23

Nr. of radial points: min: 46 max: 52

Total nr. of points: 600196

Nr. of blocks: 4690

Block length: 128

Nr. of dummy points: 124

Test of Precision of the Numerical Integration Grid

===

Integral of the Total Core Density: 85.99999002571171

Relative Error: -1.160E-07

Density Fitting (zlmFit)

ZlmFit Fit Quality: NORMAL

Max L-expansion: min: 6 max: 7

Nr. of radial interpolation points: min: 39 max: 48

S C F *** ScaSCF ***

Initial density matrix is taken from previous geometry

CYCLE 1

SCF Error: norm([F,P])= 0.073737341535, max([F,P])= 0.006786209886

orbitals (Q,E):

A :114...133 (2.00 -0.2492) (2.00 -0.2480) (2.00 -0.2365) (2.00 -0.2356)
(2.00 -0.2279) (2.00 -0.2212) (2.00 -0.2203) (2.00 -0.2152)
(2.00 -0.1955) (2.00 -0.1764) (0.00 -0.0695) (0.00 -0.0687)
(0.00 -0.0625) (0.00 -0.0390) (0.00 -0.0191) (0.00 -0.0133)
(0.00 -0.0117) (0.00 -0.0088) (0.00 -0.0051) (0.00 -0.0002)

CYCLE 2

SDIIS (wt 0.000): 0.0527 0.9473

A-DIIS (wt 1.000): 0.0000 1.0000

DIIS coefficients: 0.0000 1.0000

SCF Error: norm([F,P])= 0.014677161327, max([F,P])= 0.001434918182

orbitals (Q,E):

A :114...124 (2.00 -0.2489) (2.00 -0.2457) (2.00 -0.2371) (2.00 -0.2361)
(2.00 -0.2279) (2.00 -0.2216) (2.00 -0.2205) (2.00 -0.2158)
(2.00 -0.1958) (2.00 -0.1769) (0.00 -0.0702)

CYCLE 3

SDIIS (wt 0.000): 0.0054 0.6365 0.3581

A-DIIS (wt 1.000): 0.0000 0.6968 0.3032

DIIS coefficients: 0.0000 0.6968 0.3032

SCF Error: norm([F,P])= 0.025186272113, max([F,P])= 0.002822972385

orbitals (Q,E):

A :114...124 (2.00 -0.2492) (2.00 -0.2469) (2.00 -0.2367) (2.00 -0.2356)

(2.00 -0.2279) (2.00 -0.2213) (2.00 -0.2203) (2.00 -0.2154)

(2.00 -0.1958) (2.00 -0.1766) (0.00 -0.0698)

CYCLE 4

SDIIS (wt 1.000): 0.3700 0.1732 0.4568

DIIS coefficients: 0.3700 0.1732 0.4568

SCF Error: norm([F,P])= 0.005464742424, max([F,P])= 0.000635434026

orbitals (Q,E):

A :114...124 (2.00 -0.2491) (2.00 -0.2470) (2.00 -0.2368) (2.00 -0.2357)

(2.00 -0.2279) (2.00 -0.2213) (2.00 -0.2203) (2.00 -0.2153)

(2.00 -0.1957) (2.00 -0.1766) (0.00 -0.0697)

CYCLE 5

SDIIS (wt 1.000): 0.1798 0.0724 0.3516 0.3963

DIIS coefficients: 0.1798 0.0724 0.3516 0.3963

SCF Error: norm([F,P])= 0.003002337528, max([F,P])= 0.000464409916

orbitals (Q,E):

A :114...124 (2.00 -0.2491) (2.00 -0.2471) (2.00 -0.2367) (2.00 -0.2357)

(2.00 -0.2279) (2.00 -0.2213) (2.00 -0.2203) (2.00 -0.2154)

(2.00 -0.1957) (2.00 -0.1766) (0.00 -0.0697)

CYCLE 6

SDIIS (wt 1.000): 0.0713 0.0246 0.1719 0.2665 0.4657

DIIS coefficients: 0.0713 0.0246 0.1719 0.2665 0.4657

SCF Error: norm([F,P])= 0.000828183575, max([F,P])= 0.000102047081

orbitals (Q,E):

A :114...124 (2.00 -0.2491) (2.00 -0.2470) (2.00 -0.2367) (2.00 -0.2357)

(2.00 -0.2279) (2.00 -0.2213) (2.00 -0.2203) (2.00 -0.2154)

(2.00 -0.1957) (2.00 -0.1766) (0.00 -0.0697)

CYCLE 7

SDIIS (wt 1.000): 0.0204 0.0053 0.0622 0.1200 0.3049 0.4872

DIIS coefficients: 0.0204 0.0053 0.0622 0.1200 0.3049 0.4872

SCF Error: norm([F,P])= 0.000292196172, max([F,P])= 0.000028295766

orbitals (Q,E):

A :114...124 (2.00 -0.2491) (2.00 -0.2470) (2.00 -0.2367) (2.00 -0.2357)

(2.00 -0.2279) (2.00 -0.2213) (2.00 -0.2203) (2.00 -0.2154)

(2.00 -0.1957) (2.00 -0.1766) (0.00 -0.0697)

CYCLE 8

SDIIS (wt 1.000): 0.0067 0.0013 0.0243 0.0525 0.1515 0.3037 0.4599

DIIS coefficients: 0.0067 0.0013 0.0243 0.0525 0.1515 0.3037 0.4599

SCF Error: norm([F,P])= 0.000088526836, max([F,P])= 0.000010720671

orbitals (Q,E):

A :114...124 (2.00 -0.2491) (2.00 -0.2470) (2.00 -0.2367) (2.00 -0.2357)

(2.00 -0.2279) (2.00 -0.2213) (2.00 -0.2203) (2.00 -0.2154)

(2.00 -0.1957) (2.00 -0.1766) (0.00 -0.0697)

CYCLE 9

SDIIS (wt 1.000): 0.0019 0.0002 0.0085 0.0207 0.0671 0.1543 0.3624 0.3849

DIIS coefficients: 0.0019 0.0002 0.0085 0.0207 0.0671 0.1543 0.3624 0.3849

SCF Error: norm([F,P])= 0.000050996889, max([F,P])= 0.000008963275

orbitals (Q,E):

A :114...124 (2.00 -0.2491) (2.00 -0.2470) (2.00 -0.2367) (2.00 -0.2357)

(2.00 -0.2279) (2.00 -0.2213) (2.00 -0.2203) (2.00 -0.2154)

(2.00 -0.1957) (2.00 -0.1766) (0.00 -0.0697)

CYCLE 10

SDIIS (wt 1.000): 0.0002 -0.0001 0.0021 0.0063 0.0235 0.0599 0.1792 0.2623 0.4666

DIIS coefficients: 0.0002 -0.0001 0.0021 0.0063 0.0235 0.0599 0.1792 0.2623 0.4666

SCF Error: norm([F,P])= 0.000014798900, max([F,P])= 0.000002182013

orbitals (Q,E):

A :114...124 (2.00 -0.2491) (2.00 -0.2470) (2.00 -0.2367) (2.00 -0.2357)

(2.00 -0.2279) (2.00 -0.2213) (2.00 -0.2203) (2.00 -0.2154)

(2.00 -0.1957) (2.00 -0.1766) (0.00 -0.0697)

CYCLE 11

SDIIS (wt 1.000): -0.0001 -0.0001 0.0003 0.0015 0.0068 0.0199 0.0750 0.1308 0.3232 0.4427

DIIS coefficients: -0.0001 -0.0001 0.0003 0.0015 0.0068 0.0199 0.0750 0.1308 0.3232 0.4427

SCF Error: norm([F,P])= 0.000005867912, max([F,P])= 0.000000786966

orbitals (Q,E):

A :114...124 (2.00 -0.2491) (2.00 -0.2470) (2.00 -0.2367) (2.00 -0.2357)

(2.00 -0.2279) (2.00 -0.2213) (2.00 -0.2203) (2.00 -0.2154)

(2.00 -0.1957) (2.00 -0.1766) (0.00 -0.0697)

SCF CONVERGED

CYCLE 12

*** Setting up for NEW gradients in Focky

*** Using FIT density in Focky

*** Setting up for NEW Hessian in Focky

orbitals (Q,E):

A :114...133 (2.00 -0.2491) (2.00 -0.2470) (2.00 -0.2367) (2.00 -0.2357)

(2.00 -0.2279) (2.00 -0.2213) (2.00 -0.2203) (2.00 -0.2154)

(2.00 -0.1957) (2.00 -0.1766) (0.00 -0.0697) (0.00 -0.0690)

(0.00 -0.0614) (0.00 -0.0391) (0.00 -0.0189) (0.00 -0.0132)

(0.00 -0.0117) (0.00 -0.0087) (0.00 -0.0051) (0.00 -0.0002)

Fit test: (difference of exact and fit density, squared integrated, result summed over spins)

SCF: 0.00100801068657

Gross Charges per Atom (Z minus electrons)

==

-0.6623	0.9276	0.5413	0.3342	-0.5789	-0.3838	-0.6354	0.6741	0.6305	0.8606
-0.6601	-0.0019	-0.5780	0.5917	0.3270	-0.5905	0.6014	0.6020	-0.5970	0.2578
0.3409	0.0980	0.1679	0.7485	0.4089	0.4071	0.5642	0.5738	0.0298	0.6431
-0.5656	0.5501	0.5503	0.5716	0.6539	0.3732	0.7525	0.9867	0.5892	-0.2132
-0.1848	-0.2053	-0.1925	-0.2134	-0.2449	-0.2319	-0.1481	-0.1870	-0.1438	-0.2109
-0.2399	-0.2231	-0.2242	-0.1858	-0.2466	-0.1716	-0.1963	-0.1534	-0.1982	-0.2148
-0.2245	-0.2174	-0.2277	-0.1953	-0.1233	-0.2251	-0.1531	-0.2185	0.8314	-0.1969
-0.2184	-0.2171	-0.2117	-0.1914	-0.2121	-0.1747	-0.2289	-0.2361	-0.1974	-0.2425
-0.2141	-0.2533	-0.1967	-0.1597	-0.1648	0.2395	-0.6727	-0.1259	-0.1852	-0.1949
-0.1151	0.4899	-0.6115	-0.1739	-0.2160	-0.2393				

Net Total: 0.00000000

M U L T I P O L E D E R I V E D C H A R G E A N A L Y S I S

See: M. Swart, P.Th. van Duijnen, J.G. Snijders, J.Comput.Chem., (2001), p. 79-88.

Multipole derived atomic charges (a.u.)

Atom	Level:	MDC-m	MDC-d	MDC-q
1	O	-0.351761	-0.340386	-0.363108
2	C	0.363310	0.376124	0.398874
3	C	0.242448	0.251271	0.255268
4	C	0.180511	0.165403	0.210754
5	O	-0.303677	-0.547074	-0.618622
6	N	-0.214627	-0.275325	-0.465189
7	O	-0.378775	-0.367708	-0.437213
8	C	0.250675	0.309475	0.309162
9	C	0.228219	0.193577	0.186885
10	C	0.339344	0.410805	0.417731
11	O	-0.382968	-0.353937	-0.394042
12	C	0.124546	0.102704	0.102207
13	O	-0.313620	-0.299332	-0.348304
14	C	0.244942	0.244903	0.249734
15	C	0.195604	0.158992	0.175291
16	O	-0.370216	-0.478697	-0.484668
17	C	0.228899	0.194129	0.195728
18	C	0.230089	0.193172	0.195073
19	O	-0.318169	-0.320063	-0.388305
20	C	0.058917	0.047065	-0.061854
21	C	0.195330	0.221477	0.283756
22	C	0.119305	0.105178	0.109444
23	C	0.119924	0.099704	0.109681
24	C	0.290449	0.267611	0.287994
25	C	0.172664	0.136521	0.139096
26	C	0.164921	0.124532	0.165213

27	C	0.249182	0.236304	0.258759
28	C	0.234611	0.193751	0.290529
29	C	0.033593	0.026627	-0.054084
30	C	0.236907	0.191665	0.179446
31	O	-0.372295	-0.476513	-0.518932
32	C	0.254291	0.243317	0.263142
33	C	0.224754	0.187781	0.172855
34	C	0.227402	0.294779	0.390581
35	C	0.221906	0.192249	0.202431
36	C	0.180448	0.145934	0.152878
37	C	0.294025	0.270780	0.313209
38	C	0.368310	0.339633	0.386849
39	C	0.229709	0.190538	0.156214
40	H	-0.072600	-0.060324	-0.020568
41	H	-0.059122	-0.033650	-0.019500
42	H	-0.062110	-0.053618	-0.021838
43	H	-0.056242	-0.035047	-0.004610
44	H	-0.068864	-0.052690	-0.050678
45	H	-0.070542	-0.057850	-0.052332
46	H	-0.076324	-0.071114	-0.069210
47	H	-0.071511	-0.060786	-0.060883
48	H	-0.064812	-0.044664	-0.025431
49	H	-0.082738	-0.064342	-0.072374
50	H	-0.055792	-0.048876	-0.048267
51	H	-0.072280	-0.054831	-0.059591
52	H	-0.071352	-0.060188	-0.066391
53	H	-0.067766	-0.063621	-0.074303
54	H	-0.058464	-0.046188	-0.053082
55	H	-0.054319	-0.044216	-0.045807
56	H	-0.057714	-0.043056	-0.030281
57	H	-0.067612	-0.050826	-0.051569

58	H	-0.061793	-0.036684	-0.035587
59	H	-0.075453	-0.060485	-0.049603
60	H	-0.074894	-0.065541	-0.056029
61	H	-0.068973	-0.055287	-0.043333
62	H	-0.066420	-0.047405	-0.035787
63	H	-0.072391	-0.075095	-0.083044
64	H	-0.058363	-0.036468	-0.036746
65	H	-0.073615	-0.048649	-0.055970
66	H	-0.072303	-0.063483	-0.058056
67	H	-0.067408	-0.053584	-0.063146
68	H	-0.065398	-0.056729	-0.054630
69	C	0.351498	0.445361	0.472703
70	H	-0.064677	-0.054084	-0.078194
71	H	-0.067923	-0.067047	-0.095042
72	H	-0.063567	-0.048732	-0.070252
73	H	-0.080621	-0.063483	-0.073683
74	H	-0.072837	-0.060547	-0.066105
75	H	-0.069549	-0.069106	-0.071452
76	H	-0.075275	-0.044282	-0.034843
77	H	-0.066571	-0.054235	-0.041446
78	H	-0.065408	-0.061505	-0.031707
79	H	-0.083748	-0.064350	-0.049317
80	H	-0.066634	-0.058041	-0.052601
81	H	-0.067992	-0.066859	-0.056818
82	H	-0.071018	-0.044562	-0.037914
83	H	-0.062245	-0.023179	-0.019051
84	H	-0.046296	0.000922	-0.013218
85	H	-0.073323	-0.059725	-0.040211
86	H	0.065396	0.267575	0.312922
87	O	-0.366801	-0.446831	-0.464767
88	H	-0.045065	0.001071	0.022298

89 H -0.046141 -0.031694 -0.028515

90 H -0.052354 -0.051217 -0.054119

91 H -0.042312 -0.013124 -0.025244

92 C 0.240407 0.197684 0.226852

93 O -0.367651 -0.473696 -0.503255

94 H -0.061452 -0.054434 -0.050084

95 H -0.061074 -0.047596 -0.040598

96 H -0.068717 -0.065950 -0.062158

*** Using NEW gradient routines ***

Geometry CYCLE 8

==============

Energy gradients wrt nuclear displacements

==

Atom Cartesian (a.u./angstrom)

X Y Z

----

1 O 0.000896 0.000004 -0.000109

2 C -0.000153 0.001068 0.000509

3 C -0.000009 0.000382 -0.000204

4 C -0.000203 0.000745 0.000809

5 O 0.001387 0.000511 -0.001872

6 N -0.000069 -0.000454 -0.000552

7 O 0.000027 -0.000243 0.000410

8 C 0.000287 -0.001438 0.000808

9 C -0.000128 0.000082 0.000177

10 C -0.001558 -0.000138 -0.000607

11 O 0.000307 0.000440 -0.000440

12 C	0.000555	0.000469	-0.000433
13 O	-0.000499	-0.000852	0.000470
14 C	-0.000193	-0.000425	0.000141
15 C	-0.000015	0.000141	0.000264
16 O	-0.000385	0.000171	0.000010
17 C	-0.000195	-0.000385	0.000353
18 C	-0.000011	0.000100	0.000028
19 O	0.000790	-0.000654	-0.000254
20 C	-0.000024	0.000096	-0.000172
21 C	0.000942	-0.000740	-0.000012
22 C	0.000244	0.000694	-0.000214
23 C	-0.000037	0.000301	0.000754
24 C	-0.000017	0.000184	-0.000411
25 C	-0.000183	-0.000053	0.000264
26 C	-0.000658	0.000071	-0.000100
27 C	0.000351	-0.000021	-0.000114
28 C	-0.000111	0.000110	0.000129
29 C	0.000425	0.000035	-0.000369
30 C	0.000092	0.000217	0.000022
31 O	0.000328	0.000319	0.000145
32 C	0.000491	0.000563	-0.000263
33 C	-0.000249	0.000253	0.000541
34 C	-0.000350	-0.000434	-0.001323
35 C	0.000008	-0.000163	-0.000331
36 C	-0.000162	0.000057	0.000393
37 C	-0.000237	0.000214	-0.000048
38 C	-0.000290	0.000507	-0.000074
39 C	-0.000300	-0.000069	0.000021
40 H	-0.000095	-0.000040	0.000764
41 H	-0.000213	-0.000638	0.000018
42 H	0.000047	-0.000116	0.000223

43	H	-0.000443	-0.000052	0.000109
44	H	-0.000118	-0.000122	-0.000077
45	H	-0.000002	-0.000064	0.000222
46	H	0.000124	-0.000076	0.000054
47	H	0.000081	-0.000068	-0.000138
48	H	-0.000228	0.000102	0.000137
49	H	-0.000153	0.000266	-0.000131
50	H	-0.000108	-0.000220	-0.000064
51	H	0.000216	-0.000119	-0.000193
52	H	-0.000010	-0.000051	-0.000045
53	H	-0.000057	-0.000319	-0.000031
54	H	0.000049	0.000119	-0.000424
55	H	0.000214	0.000099	0.000139
56	H	0.000231	-0.000066	0.000158
57	H	-0.000277	0.000167	-0.000032
58	H	0.000155	0.000130	-0.000164
59	H	0.000111	-0.000120	-0.000170
60	H	0.000141	-0.000007	0.000216
61	H	0.000017	0.000165	-0.000044
62	H	0.000067	0.000134	0.000160
63	H	0.000011	-0.000114	0.000077
64	H	-0.000339	-0.000066	-0.000247
65	H	-0.000070	0.000250	0.000680
66	H	0.000095	-0.000077	-0.000291
67	H	-0.000290	-0.000035	-0.000141
68	H	-0.000023	0.000198	0.000077
69	C	-0.000438	0.000557	0.000472
70	H	-0.000096	-0.000177	0.000082
71	H	0.000035	-0.000051	-0.000181
72	H	0.000165	-0.000023	0.000067
73	H	0.000052	0.000055	-0.000142

74	H	-0.000155	-0.000133	0.000070
75	H	0.000092	-0.000124	-0.000207
76	H	-0.000169	-0.000042	0.000240
77	H	-0.000040	-0.000215	0.000068
78	H	-0.000215	-0.000006	-0.000036
79	H	-0.000009	0.000016	-0.000022
80	H	0.000074	0.000064	-0.000127
81	H	-0.000132	-0.000052	-0.000171
82	H	0.000492	-0.000042	-0.000195
83	H	0.000213	0.000020	-0.000201
84	H	-0.000046	-0.000419	-0.000289
85	H	0.000281	-0.000144	-0.000237
86	H	0.000021	0.000374	0.000461
87	O	-0.000513	-0.000387	0.000577
88	H	-0.000118	0.000166	-0.000272
89	H	-0.000070	0.000050	0.000227
90	H	0.000023	0.000054	-0.000248
91	H	0.000356	0.000089	-0.000063
92	C	-0.000164	-0.000454	0.000181
93	O	0.000070	-0.000037	0.000353
94	H	-0.000093	-0.000137	0.000157
95	H	0.000092	-0.000064	0.000017
96	H	0.000067	0.000138	0.000232

----

dE(predicted): -0.29384E-03, dE(actual): -0.11430E-03, Trust radius: 0.195959, all in a.u.

Geometry Convergence after Step 8 (Hartree/Angstrom,Angstrom)

----

current energy -19.67424050 Hartree

energy change -0.00011430 0.00100000 T

constrained gradient max 0.00187171 0.00100000 F

constrained gradient rms 0.00035372 0.00066667 T

gradient max 0.00187171

gradient rms 0.00035372

cart. step max 0.09632440 0.01000000 F

cart. step rms 0.01700960 0.00666667 F

Coordinates (Cartesian)

======================

Atom	bohr			angstrom			Geometric Variables		
	X	Y	Z	X	Y	Z	(0:frozen, *:LT par.)		
1 O	26.737756	61.461264	50.475156	14.149011	32.523900	26.710302	1	2	3
2 C	27.109916	61.905387	53.056439	14.345950	32.758920	28.076258	4	5	6
3 C	29.891685	61.284029	53.636106	15.817999	32.430112	28.383005	7	8	9
4 C	30.517150	61.832067	56.436992	16.148980	32.720121	29.865170	10	11	12
5 O	27.073567	52.886568	42.611666	14.326715	27.986367	22.549122	13	14	15
6 N	33.139840	61.328215	57.230709	17.536848	32.453494	30.285187	16	17	18
7 O	26.611521	64.519253	53.595161	14.082210	34.142118	28.361338	19	20	21
8 C	20.577835	58.361207	48.429805	10.889322	30.883421	25.627949	22	23	24
9 C	25.686494	65.416298	41.557564	13.592707	34.616814	21.991316	25	26	27
10 C	21.588804	55.040270	44.899086	11.424303	29.126057	23.759573	28	29	30
11 O	21.798553	62.682675	45.757241	11.535298	33.170243	24.213689	31	32	33
12 C	19.568070	56.536790	46.314791	10.354977	29.917981	24.508732	34	35	36
13 O	21.394545	55.275556	42.374118	11.321506	29.250565	22.423418	37	38	39
14 C	24.155524	61.387232	49.540533	12.782553	32.484724	26.215721	40	41	42
15 C	24.145205	63.141592	47.138852	12.777092	33.413092	24.944806	43	44	45

16 O	19.006307	59.383483	49.777186	10.057705	31.424386	26.340952	46	47	48
17 C	23.972499	56.799403	51.214381	12.685700	30.056950	27.101483	49	50	51
18 C	26.970861	53.291489	38.126486	14.272365	28.200641	20.175668	52	53	54
19 O	30.276693	58.597412	53.112396	16.021736	31.008415	28.105870	55	56	57
20 C	26.124553	57.112890	40.809442	13.824518	30.222840	21.595427	58	59	60
21 C	25.790732	54.244785	40.628782	13.647867	28.705104	21.499826	61	62	63
22 C	23.402030	58.609581	48.973556	12.383821	31.014855	25.915690	64	65	66
23 C	25.911932	62.668127	42.455978	13.712004	33.162545	22.466736	67	68	69
24 C	35.118400	62.358956	55.574340	18.583857	32.998938	29.408674	70	71	72
25 C	26.353640	62.342725	45.344516	13.945746	32.990349	23.995284	73	74	75
26 C	29.728756	64.587249	57.024993	15.731780	34.178100	30.176327	76	77	78
27 C	26.977346	65.124347	56.255443	14.275796	34.462320	29.769098	79	80	81
28 C	26.208375	67.883506	56.584406	13.868875	35.922405	29.943178	82	83	84
29 C	28.228472	58.422906	41.377422	14.937864	30.916070	21.895989	85	86	87
30 C	17.619322	57.898248	44.629522	9.323744	30.638434	23.616926	88	89	90
31 O	29.900057	62.439019	40.222243	15.822429	33.041306	21.284695	91	92	93
32 C	22.884979	53.637234	40.599535	12.110209	28.383602	21.484349	94	95	96
33 C	30.793710	57.362420	42.054228	16.295330	30.354885	22.254139	97	98	99
34 C	28.142005	61.287098	41.196469	14.892107	32.431736	21.800233	100	101	102
35 C	24.459332	65.907778	47.985371	12.943321	34.876894	25.392765	103	104	105
36 C	22.185799	50.848571	40.952109	11.740219	26.907905	21.670923	106	107	108
37 C	33.661510	58.693357	57.933419	17.812904	31.059187	30.657045	109	110	111
38 C	19.608512	64.137925	46.459964	10.376377	33.940328	24.585554	112	113	114
39 C	19.320982	50.353193	40.893563	10.224223	26.645762	21.639941	115	116	117
40 H	24.446078	58.183603	40.292712	12.936307	30.789437	21.321985	118	119	120
41 H	31.315165	57.957979	43.976076	16.571272	30.670042	23.271137	121	122	123

42	H	30.844312	55.306199	41.978863	16.322107	29.266780	22.214258	124	125 126
43	H	32.215622	58.171330	40.782770	17.047773	30.782942	21.581313	127	128 129
44	H	27.312089	66.539153	42.175416	14.452935	35.211003	22.318269	130	131 132
45	H	25.623636	65.504199	39.489532	13.559444	34.663329	20.896961	133	134 135
46	H	23.954944	66.270567	42.297026	12.676410	35.068874	22.382622	136	137 138
47	H	24.161220	61.660257	41.999193	12.785567	32.629203	22.225016	139	140 141
48	H	28.049178	63.390024	45.899382	14.842986	33.544556	24.288907	142	143 144
49	H	26.798724	60.354542	45.681832	14.181274	31.938248	24.173785	145	146 147
50	H	26.265079	66.098201	48.965169	13.898881	34.977662	25.911251	148	149 150
51	H	22.986491	66.478781	49.319376	12.163927	35.179056	26.098690	151	152 153
52	H	24.440951	67.187801	46.365423	12.933594	35.554253	24.535525	154	155 156
53	H	19.869492	66.153339	46.049589	10.514482	35.006840	24.368393	157	158 159
54	H	19.102121	63.880086	48.454782	10.108407	33.803886	25.641166	160	161 162
55	H	18.063769	63.414113	45.297945	9.558935	33.557303	23.970640	163	164 165
56	H	22.131185	54.315822	38.794381	11.711319	28.742695	20.529102	166	167 168
57	H	23.100037	49.802906	39.413988	12.224013	26.354563	20.856984	169	170 171
58	H	23.008548	50.143992	42.709838	12.175599	26.535058	22.601073	172	173 174
59	H	18.357871	51.282496	42.473815	9.714567	27.137528	22.476175	175	176 177
60	H	18.463967	51.050154	39.137921	9.770710	27.014578	20.710896	178	179 180

61 H 18.939205 48.322552 41.029362 10.022195 25.571193 21.711803 181 182
183

62 H 26.742922 51.243662 37.943718 14.151745 27.116978 20.078951 184 185
186

63 H 26.115770 54.230519 36.492296 13.819871 28.697555 19.310891 187 188
189

64 H 28.990843 53.722097 38.135710 15.341293 28.428509 20.180548 190 191
192

65 H 24.490065 57.956724 47.346729 12.959584 30.669378 25.054810 193 194
195

66 H 22.879213 57.352767 52.883781 12.107158 30.349777 27.984892 196 197
198

67 H 25.981614 56.848467 51.677461 13.748878 30.082913 27.346535 199 200
201

68 H 23.466687 54.863567 50.698306 12.418036 29.032549 26.828388 202 203
204

69 C 31.555085 57.965822 50.930947 16.698232 30.674192 26.951497 205 206
207

70 H 27.410050 69.103855 55.419192 14.504774 36.568185 29.326574 208 209
210

71 H 26.406818 68.456100 58.565451 13.973886 36.225408 30.991502 211 212
213

72 H 24.239403 68.159294 56.007955 12.826940 36.068345 29.638134 214 215
216

73 H 25.704326 63.908239 57.383052 13.602144 33.818783 30.365803 217 218
219

74 H 30.956859 65.913791 56.008247 16.381664 34.880076 29.638288 220 221
222

75 H 29.986754 64.924426 59.053048 15.868307 34.356527 31.249527 223 224
225

76 H 35.322499 61.322002 53.771514 18.691861 32.450206 28.454660 226 227
228

77 H 34.732817 64.342258 55.133880 18.379815 34.048456 29.175593 229 230
231

78 H 36.932618 62.280692 56.572023 19.543900 32.957523 29.936625 232 233
234

79 H 33.769002 57.390297 56.304860 17.869786 30.369637 29.795249 235 236
237

80 H 32.185447 58.004143 59.211310 17.031805 30.694471 31.333276 238 239
240

81 H 35.470655 58.613748 58.943994 18.770262 31.017060 31.191818 241 242
243

82 H 25.845862 60.719842 54.225487 13.677041 32.131557 28.694892 244 245
246

83 H 22.868330 62.171196 50.962016 12.101399 32.899580 26.967938 247 248
249

84 H 31.069544 62.397176 52.355133 16.441295 33.019164 27.705143 250 251
252

85 H 29.322034 60.577018 57.582475 15.516552 32.055977 30.471333 253 254
255

86 H 26.062287 53.110885 44.152226 13.791568 28.105070 23.364352 256 257
258

87 O 23.172002 53.737416 45.989675 12.262095 28.436616 24.336688 259 260
261

88 H 18.579994 55.081127 47.427484 9.832109 29.147677 25.097544 262 263
264

89 H 33.114098 54.513175 49.515645 17.523226 28.847130 26.202551 265 266
267

90 H 31.055892 54.090404 52.193962 16.434070 28.623409 27.619855 268 269
270

91 H 29.795549 54.814058 49.165676 15.767126 29.006350 26.017355 271 272
273

92 C 31.387401 55.149602 50.452512 16.609497 29.183913 26.698320 274 275
276

93 O 32.634698 59.466607 49.574306 17.269538 31.468373 26.233593 277 278
279

94 H 18.558746 59.293616 43.431434 9.820865 31.376830 22.982925 280 281
282

95 H 16.238633 58.853289 45.833400 8.593114 31.143819 24.253991 283 284
285

96 H 16.635836 56.546238 43.413976 8.803305 29.922981 22.973687 286 287
288

Number of elements of the density matrix on this node (used, total): 222269 1773786

Numerical Integration : Fuzzy Cells (Becke)

Becke grid quality: NORMAL

Lebedev angular grid order: min: 11 max: 23

Nr. of radial points: min: 46 max: 52

Total nr. of points: 600214

Nr. of blocks: 4690

Block length: 128

Nr. of dummy points: 106

Test of Precision of the Numerical Integration Grid

===

Integral of the Total Core Density: 85.99998258264128

Relative Error: -2.025E-07

Density Fitting (zlmFit)

ZlmFit Fit Quality: NORMAL

Max L-expansion: min: 6 max: 7

Nr. of radial interpolation points: min: 39 max: 48

S C F *** ScaSCF ***

Initial density matrix is taken from previous geometry

CYCLE 1

SCF Error: norm([F,P])= 0.074588741980, max([F,P])= 0.006751913624

orbitals (Q,E):

A :114...133 (2.00 -0.2490) (2.00 -0.2466) (2.00 -0.2371) (2.00 -0.2353)

(2.00 -0.2281) (2.00 -0.2213) (2.00 -0.2200) (2.00 -0.2152)

(2.00 -0.1953) (2.00 -0.1769) (0.00 -0.0694) (0.00 -0.0687)

(0.00 -0.0614) (0.00 -0.0394) (0.00 -0.0188) (0.00 -0.0133)

(0.00 -0.0115) (0.00 -0.0088) (0.00 -0.0053) (0.00 -0.0003)

CYCLE 2

SDIIS (wt 0.000): 0.0454 0.9546

A-DIIS (wt 1.000): 0.7258 0.2742

DIIS coefficients: 0.7258 0.2742

SCF Error: norm([F,P])= 0.013754607445, max([F,P])= 0.001469766119

orbitals (Q,E):

A :114...124 (2.00 -0.2490) (2.00 -0.2469) (2.00 -0.2369) (2.00 -0.2350)

(2.00 -0.2280) (2.00 -0.2213) (2.00 -0.2200) (2.00 -0.2152)

(2.00 -0.1954) (2.00 -0.1769) (0.00 -0.0695)

CYCLE 3

SDIIS (wt 0.000): 0.0054 -0.2651 1.2597

A-DIIS (wt 1.000): 0.5310 0.0000 0.4690

DIIS coefficients: 0.5310 0.0000 0.4690

SCF Error: norm([F,P])= 0.005476332316, max([F,P])= 0.000574734317

orbitals (Q,E):

A :114...124 (2.00 -0.2489) (2.00 -0.2469) (2.00 -0.2367) (2.00 -0.2351)

(2.00 -0.2279) (2.00 -0.2213) (2.00 -0.2200) (2.00 -0.2153)

(2.00 -0.1953) (2.00 -0.1768) (0.00 -0.0695)

CYCLE 4

SDIIS (wt 1.000): -0.5573 0.5675 0.9898

DIIS coefficients: -0.5573 0.5675 0.9898

SCF Error: norm([F,P])= 0.005970588045, max([F,P])= 0.000693048209

orbitals (Q,E):

A :114...124 (2.00 -0.2489) (2.00 -0.2471) (2.00 -0.2366) (2.00 -0.2350)

(2.00 -0.2278) (2.00 -0.2212) (2.00 -0.2200) (2.00 -0.2152)

(2.00 -0.1954) (2.00 -0.1766) (0.00 -0.0695)

CYCLE 5

SDIIS (wt 1.000): -0.3509 0.2049 0.7023 0.4438

DIIS coefficients: -0.3509 0.2049 0.7023 0.4438

SCF Error: norm([F,P])= 0.002665959306, max([F,P])= 0.000328601249

orbitals (Q,E):

A :114...124 (2.00 -0.2489) (2.00 -0.2471) (2.00 -0.2365) (2.00 -0.2349)

(2.00 -0.2278) (2.00 -0.2213) (2.00 -0.2199) (2.00 -0.2153)

(2.00 -0.1954) (2.00 -0.1767) (0.00 -0.0695)

CYCLE 6

SDIIS (wt 1.000): -0.1815 0.0838 0.3771 0.3068 0.4137

DIIS coefficients: -0.1815 0.0838 0.3771 0.3068 0.4137

SCF Error: norm([F,P])= 0.000900327108, max([F,P])= 0.000113695187

orbitals (Q,E):

A :114...124 (2.00 -0.2489) (2.00 -0.2471) (2.00 -0.2365) (2.00 -0.2350)

(2.00 -0.2277) (2.00 -0.2213) (2.00 -0.2200) (2.00 -0.2153)

(2.00 -0.1954) (2.00 -0.1767) (0.00 -0.0695)

CYCLE 7

SDIIS (wt 1.000): -0.0678 0.0227 0.1456 0.1464 0.2993 0.4538

DIIS coefficients: -0.0678 0.0227 0.1456 0.1464 0.2993 0.4538

SCF Error: norm([F,P])= 0.000358295270, max([F,P])= 0.000064787562

orbitals (Q,E):

A :114...124 (2.00 -0.2489) (2.00 -0.2471) (2.00 -0.2365) (2.00 -0.2350)

(2.00 -0.2278) (2.00 -0.2213) (2.00 -0.2200) (2.00 -0.2153)

(2.00 -0.1954) (2.00 -0.1767) (0.00 -0.0695)

CYCLE 8

SDIIS (wt 1.000): -0.0238 0.0056 0.0524 0.0619 0.1549 0.3207 0.4283

DIIS coefficients: -0.0238 0.0056 0.0524 0.0619 0.1549 0.3207 0.4283

SCF Error: norm([F,P])= 0.000137344228, max([F,P])= 0.000016628273

orbitals (Q,E):

A :114...124 (2.00 -0.2489) (2.00 -0.2471) (2.00 -0.2365) (2.00 -0.2350)

(2.00 -0.2278) (2.00 -0.2213) (2.00 -0.2200) (2.00 -0.2153)

(2.00 -0.1954) (2.00 -0.1767) (0.00 -0.0695)

CYCLE 9

SDIIS (wt 1.000): -0.0057 0.0003 0.0130 0.0195 0.0595 0.1487 0.2869 0.4778

DIIS coefficients: -0.0057 0.0003 0.0130 0.0195 0.0595 0.1487 0.2869 0.4778

SCF Error: norm([F,P])= 0.000045774841, max([F,P])= 0.000005259128

orbitals (Q,E):

A :114...124 (2.00 -0.2489) (2.00 -0.2471) (2.00 -0.2365) (2.00 -0.2350)

(2.00 -0.2278) (2.00 -0.2213) (2.00 -0.2200) (2.00 -0.2153)

(2.00 -0.1954) (2.00 -0.1767) (0.00 -0.0695)

CYCLE 10

SDIIS (wt 1.000): -0.0014 -0.0003 0.0032 0.0066 0.0240 0.0680 0.1517 0.3376 0.4106

DIIS coefficients: -0.0014 -0.0003 0.0032 0.0066 0.0240 0.0680 0.1517 0.3376 0.4106

SCF Error: norm([F,P])= 0.000019071681, max([F,P])= 0.000002393176

orbitals (Q,E):

A :114...124 (2.00 -0.2489) (2.00 -0.2471) (2.00 -0.2365) (2.00 -0.2350)

(2.00 -0.2278) (2.00 -0.2213) (2.00 -0.2200) (2.00 -0.2153)

(2.00 -0.1954) (2.00 -0.1767) (0.00 -0.0695)

CYCLE 11

SDIIS (wt 1.000): -0.0000 -0.0003 0.0001 0.0016 0.0082 0.0264 0.0671 0.1796 0.3215 0.3959

DIIS coefficients: -0.0000 -0.0003 0.0001 0.0016 0.0082 0.0264 0.0671 0.1796 0.3215 0.3959

SCF Error: norm([F,P])= 0.000008979414, max([F,P])= 0.000001165867

orbitals (Q,E):

A :114...124 (2.00 -0.2489) (2.00 -0.2471) (2.00 -0.2365) (2.00 -0.2350)

(2.00 -0.2278) (2.00 -0.2213) (2.00 -0.2200) (2.00 -0.2153)

(2.00 -0.1954) (2.00 -0.1767) (0.00 -0.0695)

CYCLE 12

SDIIS (wt 1.000): -0.0001 0.0000 0.0005 0.0030 0.0096 0.0257 0.0740 0.1498 0.2394 0.4982

DIIS coefficients: -0.0001 0.0000 0.0005 0.0030 0.0096 0.0257 0.0740 0.1498 0.2394 0.4982

SCF Error: norm([F,P])= 0.000001979921, max([F,P])= 0.000000226600

orbitals (Q,E):

A :114...124 (2.00 -0.2489) (2.00 -0.2471) (2.00 -0.2365) (2.00 -0.2350)

(2.00 -0.2278) (2.00 -0.2213) (2.00 -0.2200) (2.00 -0.2153)

(2.00 -0.1954) (2.00 -0.1767) (0.00 -0.0695)

SCF CONVERGED

CYCLE 13

*** Setting up for NEW gradients in Focky

*** Using FIT density in Focky

*** Setting up for NEW Hessian in Focky

orbitals (Q,E):

A :114...133 (2.00 -0.2489) (2.00 -0.2471) (2.00 -0.2365) (2.00 -0.2350)

(2.00 -0.2278) (2.00 -0.2213) (2.00 -0.2200) (2.00 -0.2153)

(2.00 -0.1954) (2.00 -0.1767) (0.00 -0.0695) (0.00 -0.0688)

(0.00 -0.0620) (0.00 -0.0388) (0.00 -0.0187) (0.00 -0.0131)

(0.00 -0.0114) (0.00 -0.0085) (0.00 -0.0051) (0.00 -0.0002)

Fit test: (difference of exact and fit density, squared integrated, result summed over spins)

SCF: 0.00100791264592

Gross Charges per Atom (Z minus electrons)

==

-0.6627 0.9274 0.5428 0.3343 -0.5807 -0.3834 -0.6353 0.6740 0.6296 0.8647

-0.6598	-0.0017	-0.5778	0.5914	0.3269	-0.5906	0.6013	0.6015	-0.5977	0.2579
0.3404	0.0979	0.1679	0.7486	0.4091	0.4068	0.5640	0.5738	0.0289	0.6424
-0.5658	0.5511	0.5517	0.5720	0.6533	0.3722	0.7524	0.9865	0.5896	-0.2140
-0.1832	-0.2072	-0.1932	-0.2134	-0.2447	-0.2320	-0.1483	-0.1864	-0.1422	-0.2111
-0.2395	-0.2238	-0.2243	-0.1854	-0.2469	-0.1721	-0.1962	-0.1542	-0.1982	-0.2148
-0.2245	-0.2181	-0.2282	-0.1949	-0.1204	-0.2247	-0.1556	-0.2186	0.8309	-0.1969
-0.2185	-0.2171	-0.2116	-0.1914	-0.2120	-0.1767	-0.2283	-0.2357	-0.1974	-0.2423
-0.2140	-0.2534	-0.1969	-0.1601	-0.1652	0.2413	-0.6734	-0.1261	-0.1852	-0.1960
-0.1128	0.4908	-0.6117	-0.1748	-0.2159	-0.2385				

Net Total: 0.00000000

M U L T I P O L E D E R I V E D C H A R G E A N A L Y S I S

See: M. Swart, P.Th. van Duijnen, J.G. Snijders, J.Comput.Chem., (2001), p. 79-88.

Multipole derived atomic charges (a.u.)

Atom	Level:	MDC-m	MDC-d	MDC-q
1	O	-0.351388	-0.338053	-0.360530
2	C	0.363173	0.375844	0.398327
3	C	0.242374	0.251361	0.255090
4	C	0.180557	0.165614	0.210979
5	O	-0.306862	-0.550054	-0.620934
6	N	-0.214448	-0.275386	-0.465777

7	O	-0.378927	-0.368624	-0.437739
8	C	0.250469	0.309654	0.308968
9	C	0.228174	0.193512	0.187314
10	C	0.339656	0.409752	0.416295
11	O	-0.382927	-0.355145	-0.395700
12	C	0.124466	0.102512	0.102436
13	O	-0.312880	-0.299477	-0.348080
14	C	0.244820	0.244591	0.249479
15	C	0.195590	0.159250	0.175482
16	O	-0.369855	-0.478597	-0.484416
17	C	0.228931	0.194217	0.196414
18	C	0.229912	0.193232	0.195377
19	O	-0.318486	-0.320708	-0.387317
20	C	0.059125	0.047095	-0.059396
21	C	0.195112	0.221752	0.283917
22	C	0.119049	0.104972	0.109017
23	C	0.119917	0.099761	0.110020
24	C	0.290550	0.267864	0.288205
25	C	0.172687	0.136255	0.138768
26	C	0.164899	0.124562	0.165032
27	C	0.249156	0.236395	0.258966
28	C	0.234611	0.193761	0.291070
29	C	0.033644	0.026762	-0.053770
30	C	0.236921	0.191550	0.179077
31	O	-0.372153	-0.476632	-0.518460
32	C	0.254152	0.243540	0.263796
33	C	0.224967	0.187969	0.174774
34	C	0.227305	0.294954	0.389377
35	C	0.221999	0.192369	0.202431
36	C	0.180413	0.145899	0.151918
37	C	0.294027	0.270853	0.313009

38	C	0.368213	0.339607	0.387169
39	C	0.229580	0.190334	0.155653
40	H	-0.072831	-0.060051	-0.021593
41	H	-0.059077	-0.033707	-0.019305
42	H	-0.062349	-0.054172	-0.022984
43	H	-0.056056	-0.034576	-0.005328
44	H	-0.068893	-0.052775	-0.050948
45	H	-0.070593	-0.057873	-0.052663
46	H	-0.076384	-0.071286	-0.069407
47	H	-0.071668	-0.061228	-0.061010
48	H	-0.064551	-0.044075	-0.024714
49	H	-0.082582	-0.063833	-0.071860
50	H	-0.055749	-0.048696	-0.048462
51	H	-0.072236	-0.054709	-0.059638
52	H	-0.071331	-0.060201	-0.066389
53	H	-0.067736	-0.063520	-0.074020
54	H	-0.058448	-0.046089	-0.053050
55	H	-0.054314	-0.044270	-0.046006
56	H	-0.057613	-0.043039	-0.030454
57	H	-0.067323	-0.050350	-0.050805
58	H	-0.061882	-0.036652	-0.035060
59	H	-0.075546	-0.060809	-0.049979
60	H	-0.074824	-0.065307	-0.055694
61	H	-0.069018	-0.055423	-0.043311
62	H	-0.066658	-0.047820	-0.036668
63	H	-0.072673	-0.075410	-0.083530
64	H	-0.058435	-0.036398	-0.036889
65	H	-0.073540	-0.048078	-0.055707
66	H	-0.072372	-0.063695	-0.058453
67	H	-0.067306	-0.053440	-0.063508
68	H	-0.065438	-0.057096	-0.055181

69	C	0.351500	0.445074	0.470618
70	H	-0.064704	-0.054140	-0.078466
71	H	-0.067979	-0.067148	-0.095235
72	H	-0.063537	-0.048699	-0.070418
73	H	-0.080648	-0.063432	-0.073588
74	H	-0.072884	-0.060534	-0.065769
75	H	-0.069538	-0.069087	-0.071505
76	H	-0.075636	-0.044945	-0.035766
77	H	-0.066522	-0.054160	-0.041116
78	H	-0.065310	-0.061333	-0.031460
79	H	-0.083593	-0.064351	-0.049006
80	H	-0.066623	-0.057976	-0.052456
81	H	-0.068026	-0.066998	-0.056920
82	H	-0.071137	-0.044805	-0.038089
83	H	-0.062238	-0.023521	-0.018974
84	H	-0.046460	0.000651	-0.013274
85	H	-0.073416	-0.059825	-0.040383
86	H	0.062912	0.264936	0.309934
87	O	-0.361859	-0.440369	-0.458603
88	H	-0.044917	0.000871	0.021934
89	H	-0.046069	-0.031632	-0.027567
90	H	-0.052467	-0.051423	-0.052899
91	H	-0.042179	-0.012759	-0.023777
92	C	0.240437	0.197710	0.223130
93	O	-0.367143	-0.473178	-0.502043
94	H	-0.061389	-0.054125	-0.049708
95	H	-0.061014	-0.047650	-0.040383
96	H	-0.068655	-0.065688	-0.061843

*** Using NEW gradient routines ***

Geometry CYCLE 9

==============

Energy gradients wrt nuclear displacements

===

Atom	Cartesian (a.u./angstrom)		
	X	Y	Z
1 O	0.000453	0.000064	-0.000495
2 C	0.000199	0.000558	0.000494
3 C	-0.000107	0.000533	0.000227
4 C	0.000091	0.000665	0.000545
5 O	-0.001768	0.000128	0.001534
6 N	-0.000142	-0.000563	-0.000521
7 O	0.000054	-0.000386	0.000137
8 C	-0.001082	0.001009	-0.000292
9 C	-0.000095	0.000077	0.000046
10 C	0.000277	-0.002311	0.000790
11 O	0.000173	0.000282	-0.000049
12 C	0.000301	0.000366	0.000384
13 O	0.000433	0.000251	-0.000222
14 C	-0.000099	0.000164	0.000298
15 C	0.000074	0.000260	-0.000054
16 O	0.001081	-0.001257	-0.000311
17 C	0.000025	-0.000397	0.000062
18 C	0.000211	0.000057	-0.000163
19 O	0.000364	-0.000264	-0.000571
20 C	-0.000094	0.000390	0.000666
21 C	-0.000093	0.000452	-0.000168
22 C	-0.000098	-0.000052	0.000094

23	C	0.000295	0.000292	0.000591
24	C	0.000082	0.000111	-0.000270
25	C	-0.000196	-0.000145	0.000102
26	C	-0.000410	-0.000107	-0.000053
27	C	0.000369	-0.000082	-0.000106
28	C	-0.000173	0.000063	0.000016
29	C	0.000229	-0.000017	0.000132
30	C	-0.000075	0.000199	-0.000158
31	O	-0.000829	-0.000815	0.000983
32	C	-0.000727	-0.000027	0.000072
33	C	0.000226	0.000217	-0.000409
34	C	0.000140	0.000314	-0.001671
35	C	-0.000177	-0.000216	-0.000096
36	C	0.000212	-0.000193	0.000376
37	C	-0.000236	0.000337	0.000005
38	C	-0.000007	0.000329	0.000136
39	C	-0.000141	0.000127	-0.000019
40	H	0.000227	-0.000218	-0.000167
41	H	-0.000300	-0.000488	-0.000187
42	H	0.000237	0.000273	0.000019
43	H	-0.000216	0.000017	0.000175
44	H	-0.000141	-0.000112	-0.000088
45	H	-0.000012	-0.000049	0.000197
46	H	0.000147	-0.000110	-0.000073
47	H	0.000170	-0.000043	-0.000041
48	H	-0.000172	0.000002	0.000128
49	H	-0.000110	-0.000001	-0.000171
50	H	-0.000086	-0.000100	-0.000012
51	H	0.000143	-0.000125	-0.000089
52	H	-0.000066	-0.000195	0.000102
53	H	0.000016	-0.000227	-0.000004

54	H	0.000084	0.000034	-0.000301
55	H	0.000289	0.000223	0.000161
56	H	0.000156	-0.000118	0.000128
57	H	-0.000205	0.000101	-0.000011
58	H	-0.000039	0.000161	-0.000188
59	H	0.000099	-0.000112	-0.000107
60	H	0.000152	0.000034	0.000160
61	H	0.000082	0.000192	-0.000048
62	H	0.000020	0.000178	0.000117
63	H	0.000070	-0.000119	0.000115
64	H	-0.000178	0.000024	-0.000123
65	H	-0.000183	-0.000199	-0.000017
66	H	0.000097	-0.000079	-0.000211
67	H	-0.000468	0.000083	0.000008
68	H	-0.000133	0.000153	-0.000063
69	C	0.000999	0.001255	0.000289
70	H	-0.000103	-0.000148	0.000064
71	H	0.000030	-0.000057	-0.000183
72	H	0.000162	-0.000017	0.000043
73	H	0.000054	0.000063	-0.000121
74	H	-0.000167	-0.000097	0.000090
75	H	0.000107	-0.000096	-0.000180
76	H	-0.000171	0.000016	0.000218
77	H	-0.000053	-0.000209	0.000066
78	H	-0.000196	0.000032	-0.000009
79	H	0.000007	0.000060	-0.000013
80	H	0.000069	0.000082	-0.000151
81	H	-0.000139	-0.000009	-0.000155
82	H	0.000299	-0.000118	-0.000201
83	H	0.000027	0.000006	-0.000248
84	H	0.000006	-0.000352	-0.000231

```
  85 H     0.000190 -0.000083 -0.000203
  86 H     0.000572  0.000043 -0.000277
  87 O     0.000545  0.001381 -0.001578
  88 H    -0.000052  0.000053 -0.000269
  89 H    -0.000070  0.000096  0.000214
  90 H     0.000022  0.000163 -0.000246
  91 H     0.000284  0.000073  0.000032
  92 C    -0.000083 -0.000391  0.000044
  93 O    -0.000980 -0.001017  0.001232
  94 H    -0.000045 -0.000248  0.000146
  95 H     0.000172 -0.000165 -0.000065
  96 H     0.000092  0.000097  0.000220
 ----------------------------------------

 dE(predicted):    -0.11888E-03, dE(actual):    -0.13082E-03, Trust radius:    0.195959, all in a.u.

---------------------------------------------------------------------
Geometry Convergence after Step   9       (Hartree/Angstrom,Angstrom)
---------------------------------------------------------------------
current energy                          -19.67437132 Hartree
energy change                     -0.00013082     0.00100000    T
constrained gradient max           0.00231149     0.00100000    F
constrained gradient rms           0.00039603     0.00066667    T
gradient max                      0.00231149
gradient rms                      0.00039603
cart. step max                    0.10049242     0.01000000    F
cart. step rms                    0.02198750     0.00666667    F

 Coordinates (Cartesian)
```

======================

Atom	bohr			angstrom			Geometric Variables		
	X	Y	Z	X	Y	Z	(0:frozen, *:LT par.)		
1 O	26.718706	61.490162	50.469226	14.138931	32.539192	26.707164	1	2	3
2 C	27.103697	61.917328	53.051178	14.342659	32.765239	28.073474	4	5	6
3 C	29.884734	61.274388	53.612535	15.814320	32.425010	28.370532	7	8	9
4 C	30.524415	61.791715	56.416065	16.152825	32.698767	29.854096	10	11	12
5 O	27.109882	52.897771	42.688865	14.345932	27.992295	22.589975	13	14	15
6 N	33.144653	61.261551	57.201988	17.539395	32.418217	30.269988	16	17	18
7 O	26.626593	64.531564	53.608070	14.090186	34.148633	28.368169	19	20	21
8 C	20.562994	58.386345	48.418912	10.881468	30.896723	25.622185	22	23	24
9 C	25.668901	65.415255	41.538189	13.583397	34.616262	21.981063	25	26	27
10 C	21.600653	55.044722	44.920586	11.430574	29.128413	23.770950	28	29	30
11 O	21.779555	62.690439	45.753801	11.525244	33.174352	24.211869	31	32	33
12 C	19.566404	56.544733	46.312798	10.354095	29.922184	24.507677	34	35	36
13 O	21.425334	55.264060	42.392524	11.337799	29.244481	22.433158	37	38	39
14 C	24.135519	61.414248	49.539407	12.771967	32.499020	26.215125	40	41	42
15 C	24.124202	63.163055	47.134544	12.765978	33.424449	24.942527	43	44	45
16 O	18.985002	59.414873	49.753717	10.046430	31.440997	26.328533	46	47	48
17 C	23.949193	56.829114	51.219027	12.673367	30.072672	27.103942	49	50	51
18 C	27.042365	53.271742	38.200563	14.310203	28.190192	20.214868	52	53	54
19 O	30.250762	58.592270	53.054846	16.008014	31.005694	28.075415	55	56	57
20 C	26.158201	57.108616	40.847748	13.842324	30.220578	21.615697	58	59	60
21 C	25.837626	54.237522	40.686500	13.672683	28.701260	21.530369	61	62	63
22 C	23.384766	58.635585	48.973782	12.374685	31.028615	25.915810	64	65	66
23 C	25.905474	62.673739	42.454608	13.708586	33.165514	22.466011	67	68	69
24 C	35.129644	62.289890	55.551918	18.589807	32.962390	29.396809	70	71	72
25 C	26.339856	62.367385	45.346468	13.938451	33.003399	23.996317	73	74	75
26 C	29.760460	64.549335	57.026578	15.748557	34.158037	30.177165	76	77	78

27 C	27.010288	65.115868	56.270883	14.293229	34.457833	29.777269	79	80	81
28 C	26.267255	67.879633	56.621068	13.900033	35.920355	29.962579	82	83	84
29 C	28.252194	58.435030	41.415369	14.950417	30.922486	21.916069	85	86	87
30 C	17.623793	57.887344	44.605100	9.326109	30.632663	23.604003	88	89	90
31 O	29.903785	62.452779	40.238720	15.824402	33.048587	21.293413	91	92	93
32 C	22.934396	53.618859	40.639922	12.136360	28.373878	21.505720	94	95	96
33 C	30.822622	57.396830	42.108039	16.310629	30.373095	22.282615	97	98	99
34 C	28.148416	61.297296	41.213475	14.895500	32.437132	21.809232	100	101	102
35 C	24.426042	65.932170	47.977322	12.925705	34.889802	25.388505	103	104	105
36 C	22.242095	50.830413	41.007852	11.770010	26.898296	21.700421	106	107	108
37 C	33.647653	58.617486	57.884113	17.805571	31.019038	30.630954	109	110	111
38 C	19.581876	64.132620	46.459588	10.362283	33.937521	24.585355	112	113	114
39 C	19.379818	50.324124	40.923749	10.255358	26.630379	21.655915	115	116	117
40 H	24.478991	58.166983	40.307662	12.953724	30.780642	21.329896	118	119	120
41 H	31.340183	58.021085	44.022123	16.584511	30.703436	23.295504	121	122	123
42 H	30.884075	55.340061	42.058406	16.343149	29.284699	22.256350	124	125	126
43 H	32.241676	58.197019	40.827354	17.061560	30.796536	21.604905	127	128	129
44 H	27.285706	66.550996	42.156175	14.438974	35.217270	22.308087	130	131	132
45 H	25.614444	65.490356	39.469189	13.554580	34.656004	20.886195	133	134	135
46 H	23.929386	66.264666	42.265041	12.662886	35.065751	22.365697	136	137	138
47 H	24.161870	61.653303	41.998263	12.785911	32.625523	22.224524	139	140	141

48	H	28.029396	63.425652	45.899234	14.832517	33.563410	24.288829	142	143	144
49	H	26.795265	60.383326	45.695487	14.179444	31.953480	24.181011	145	146	147
50	H	26.229816	66.132291	48.959353	13.880221	34.995701	25.908174	148	149	150
51	H	22.948782	66.499379	49.308506	12.143972	35.189956	26.092938	151	152	153
52	H	24.404446	67.209505	46.355093	12.914277	35.565738	24.530059	154	155	156
53	H	19.830276	66.149803	46.048742	10.493730	35.004968	24.367945	157	158	159
54	H	19.079994	63.872010	48.455507	10.096698	33.799612	25.641550	160	161	162
55	H	18.039514	63.399276	45.299831	9.546100	33.549452	23.971638	163	164	165
56	H	22.190803	54.282001	38.824538	11.742867	28.724798	20.545060	166	167	168
57	H	23.177233	49.774944	39.488958	12.264863	26.339766	20.896656	169	170	171
58	H	23.048132	50.143458	42.780440	12.196546	26.534775	22.638434	172	173	174
59	H	18.396681	51.265401	42.484837	9.735105	27.128482	22.482007	175	176	177
60	H	18.538656	51.000782	39.152289	9.810234	26.988452	20.718499	178	179	180
61	H	19.003758	48.293360	41.075347	10.056356	25.555746	21.736138	181	182	183
62	H	26.821208	51.222189	38.027334	14.193172	27.105615	20.123199	184	185	186
63	H	26.200110	54.199308	36.553002	13.864501	28.681039	19.343016	187	188	189
64	H	29.061364	53.707432	38.226264	15.378612	28.420749	20.228468	190	191	192
65	H	24.477695	57.980889	47.350478	12.953038	30.682165	25.056794	193	194	195
66	H	22.850031	57.384006	52.884444	12.091715	30.366308	27.985242	196	197	198

67 H	25.956647	56.880280	51.689021	13.735666	30.099748	27.352652	199	200	201
68 H	23.446868	54.891898	50.704284	12.407548	29.047541	26.831552	202	203	204
69 C	31.472647	57.980216	50.834593	16.654608	30.681809	26.900508	205	206	207
70 H	27.475574	69.097438	55.459714	14.539448	36.564789	29.348017	208	209	210
71 H	26.477674	68.437113	58.605376	14.011382	36.215361	31.012630	211	212	213
72 H	24.298601	68.176741	56.053577	12.858266	36.077578	29.662276	214	215	216
73 H	25.731252	63.903839	57.396352	13.616392	33.816455	30.372841	217	218	219
74 H	30.996473	65.873059	56.015293	16.402627	34.858522	29.642017	220	221	222
75 H	30.027687	64.870082	59.056306	15.889967	34.327769	31.251251	223	224	225
76 H	35.323593	61.266770	53.739723	18.692440	32.420978	28.437837	226	227	228
77 H	34.759932	64.280098	55.128411	18.394164	34.015563	29.172699	229	230	231
78 H	36.944666	62.188463	56.546489	19.550275	32.908717	29.923113	232	233	234
79 H	33.743743	57.326163	56.245328	17.856420	30.335699	29.763746	235	236	237
80 H	32.167796	57.929389	59.158450	17.022464	30.654913	31.305303	238	239	240
81 H	35.457251	58.517070	58.892441	18.763169	30.965900	31.164538	241	242	243
82 H	25.836305	60.733473	54.218861	13.671984	32.138770	28.691386	244	245	246
83 H	22.847704	62.198320	50.960661	12.090484	32.913933	26.967220	247	248	249
84 H	31.062647	62.395579	52.338361	16.437645	33.018319	27.696268	250	251	252
85 H	29.321167	60.538437	57.555367	15.516094	32.035561	30.456989	253	254	255

86 H 26.082136 53.124893 44.218181 13.802072 28.112483 23.399254 256 257
258

87 O 23.175858 53.749854 46.032327 12.264136 28.443198 24.359258 259 260
261

88 H 18.577632 55.093979 47.431660 9.830860 29.154478 25.099754 262 263
264

89 H 32.968435 54.538589 49.325741 17.446145 28.860578 26.102058 265 266
267

90 H 30.992289 54.091198 52.061993 16.400413 28.623829 27.550020 268 269
270

91 H 29.641861 54.860962 49.084322 15.685797 29.031171 25.974305 271 272
273

92 C 31.275409 55.171542 50.324713 16.550234 29.195523 26.630691 274 275
276

93 O 32.521596 59.492653 49.466664 17.209687 31.482156 26.176632 277 278
279

94 H 18.566390 59.276926 43.402281 9.824911 31.367998 22.967498 280 281
282

95 H 16.232341 58.847174 45.792861 8.589785 31.140583 24.232538 283 284
285

96 H 16.652356 56.523200 43.392982 8.812047 29.910789 22.962577 286 287
288

Number of elements of the density matrix on this node (used, total): 222269 1773786

Numerical Integration : Fuzzy Cells (Becke)

Becke grid quality: NORMAL

Lebedev angular grid order: min: 11 max: 23

Nr. of radial points: min: 46 max: 52

Total nr. of points: 600218

Nr. of blocks: 4690

Block length: 128

Nr. of dummy points: 102

Test of Precision of the Numerical Integration Grid

==

Integral of the Total Core Density: 85.99996872428679

Relative Error: -3.637E-07

Density Fitting (zlmFit)

ZlmFit Fit Quality: NORMAL

Max L-expansion: min: 6 max: 7

Nr. of radial interpolation points: min: 39 max: 48

S C F *** ScaSCF ***

Initial density matrix is taken from previous geometry

CYCLE 1

SCF Error: norm([F,P])= 0.071816671678, max([F,P])= 0.007837613124

orbitals (Q,E):

A :114...133 (2.00 -0.2495) (2.00 -0.2478) (2.00 -0.2372) (2.00 -0.2354)

(2.00 -0.2286) (2.00 -0.2219) (2.00 -0.2205) (2.00 -0.2158)

(2.00 -0.1955) (2.00 -0.1774) (0.00 -0.0699) (0.00 -0.0692)

(0.00 -0.0630) (0.00 -0.0395) (0.00 -0.0191) (0.00 -0.0137)

(0.00 -0.0118) (0.00 -0.0089) (0.00 -0.0056) (0.00 -0.0007)

CYCLE 2

SDIIS (wt 0.000): 0.0281 0.9719

A-DIIS (wt 1.000): 0.0000 1.0000

DIIS coefficients: 0.0000 1.0000

SCF Error: norm([F,P])= 0.010661843490, max([F,P])= 0.000687350226

orbitals (Q,E):

A :114...124 (2.00 -0.2484) (2.00 -0.2470) (2.00 -0.2364) (2.00 -0.2347)

(2.00 -0.2271) (2.00 -0.2212) (2.00 -0.2198) (2.00 -0.2149)

(2.00 -0.1958) (2.00 -0.1769) (0.00 -0.0694)

CYCLE 3

SDIIS (wt 0.000): 0.0027 0.6487 0.3485

A-DIIS (wt 1.000): 0.0000 0.7111 0.2889

DIIS coefficients: 0.0000 0.7111 0.2889

SCF Error: norm([F,P])= 0.018800455995, max([F,P])= 0.002017288254

orbitals (Q,E):

A :114...124 (2.00 -0.2486) (2.00 -0.2471) (2.00 -0.2362) (2.00 -0.2348)

(2.00 -0.2274) (2.00 -0.2213) (2.00 -0.2199) (2.00 -0.2151)

(2.00 -0.1952) (2.00 -0.1768) (0.00 -0.0694)

CYCLE 4

SDIIS (wt 1.000): 0.3665 0.1836 0.4499

DIIS coefficients: 0.3665 0.1836 0.4499

SCF Error: norm([F,P])= 0.004794749058, max([F,P])= 0.000636275442

orbitals (Q,E):

A :114...124 (2.00 -0.2487) (2.00 -0.2472) (2.00 -0.2365) (2.00 -0.2348)

(2.00 -0.2276) (2.00 -0.2213) (2.00 -0.2200) (2.00 -0.2151)

(2.00 -0.1953) (2.00 -0.1768) (0.00 -0.0694)

CYCLE 5

SDIIS (wt 1.000): 0.1589 0.0749 0.3162 0.4499

DIIS coefficients: 0.1589 0.0749 0.3162 0.4499

SCF Error: norm([F,P])= 0.002223708591, max([F,P])= 0.000314932027

orbitals (Q,E):

A :114...124 (2.00 -0.2487) (2.00 -0.2472) (2.00 -0.2364) (2.00 -0.2348)

(2.00 -0.2276) (2.00 -0.2213) (2.00 -0.2200) (2.00 -0.2151)

(2.00 -0.1952) (2.00 -0.1768) (0.00 -0.0694)

CYCLE 6

SDIIS (wt 1.000): 0.0652 0.0296 0.1569 0.2978 0.4505

DIIS coefficients: 0.0652 0.0296 0.1569 0.2978 0.4505

SCF Error: norm([F,P])= 0.000706739387, max([F,P])= 0.000062121177

orbitals (Q,E):

A :114...124 (2.00 -0.2487) (2.00 -0.2472) (2.00 -0.2364) (2.00 -0.2348)

(2.00 -0.2276) (2.00 -0.2213) (2.00 -0.2200) (2.00 -0.2151)

(2.00 -0.1953) (2.00 -0.1768) (0.00 -0.0694)

CYCLE 7

SDIIS (wt 1.000): 0.0192 0.0084 0.0579 0.1388 0.3287 0.4469

DIIS coefficients: 0.0192 0.0084 0.0579 0.1388 0.3287 0.4469

SCF Error: norm([F,P])= 0.000315440228, max([F,P])= 0.000056457518

orbitals (Q,E):

A :114...124 (2.00 -0.2487) (2.00 -0.2472) (2.00 -0.2364) (2.00 -0.2348)

(2.00 -0.2276) (2.00 -0.2213) (2.00 -0.2200) (2.00 -0.2151)

(2.00 -0.1953) (2.00 -0.1768) (0.00 -0.0694)

CYCLE 8

SDIIS (wt 1.000): 0.0054 0.0022 0.0213 0.0607 0.1765 0.3163 0.4176

DIIS coefficients: 0.0054 0.0022 0.0213 0.0607 0.1765 0.3163 0.4176

SCF Error: norm([F,P])= 0.000117425609, max([F,P])= 0.000017071931

orbitals (Q,E):

A :114...124 (2.00 -0.2487) (2.00 -0.2472) (2.00 -0.2364) (2.00 -0.2348)

(2.00 -0.2276) (2.00 -0.2213) (2.00 -0.2200) (2.00 -0.2151)

(2.00 -0.1953) (2.00 -0.1768) (0.00 -0.0694)

CYCLE 9

SDIIS (wt 1.000): 0.0009 0.0003 0.0063 0.0214 0.0733 0.1516 0.2835 0.4627

DIIS coefficients: 0.0009 0.0003 0.0063 0.0214 0.0733 0.1516 0.2835 0.4627

SCF Error: norm([F,P])= 0.000038797482, max([F,P])= 0.000005173331

orbitals (Q,E):

A :114...124 (2.00 -0.2487) (2.00 -0.2472) (2.00 -0.2364) (2.00 -0.2348)

(2.00 -0.2276) (2.00 -0.2213) (2.00 -0.2200) (2.00 -0.2151)

(2.00 -0.1953) (2.00 -0.1768) (0.00 -0.0694)

CYCLE 10

SDIIS (wt 1.000): -0.0000 -0.0000 0.0016 0.0070 0.0281 0.0651 0.1441 0.3260 0.4281

DIIS coefficients: -0.0000 -0.0000 0.0016 0.0070 0.0281 0.0651 0.1441 0.3260 0.4281

SCF Error: norm([F,P])= 0.000015692261, max([F,P])= 0.000002397661

orbitals (Q,E):

A :114...124 (2.00 -0.2487) (2.00 -0.2472) (2.00 -0.2364) (2.00 -0.2348)

(2.00 -0.2276) (2.00 -0.2213) (2.00 -0.2200) (2.00 -0.2151)

(2.00 -0.1953) (2.00 -0.1768) (0.00 -0.0694)

CYCLE 11

SDIIS (wt 1.000): -0.0002 -0.0001 0.0002 0.0017 0.0093 0.0244 0.0628 0.1713 0.3317 0.3989

DIIS coefficients: -0.0002 -0.0001 0.0002 0.0017 0.0093 0.0244 0.0628 0.1713 0.3317 0.3989

SCF Error: norm([F,P])= 0.000007384056, max([F,P])= 0.000001104480

orbitals (Q,E):

A :114...124 (2.00 -0.2487) (2.00 -0.2472) (2.00 -0.2364) (2.00 -0.2348)

(2.00 -0.2276) (2.00 -0.2213) (2.00 -0.2200) (2.00 -0.2151)

(2.00 -0.1953) (2.00 -0.1768) (0.00 -0.0694)

CYCLE 12

SDIIS (wt 1.000): 0.0000 0.0002 0.0009 0.0038 0.0096 0.0252 0.0721 0.1614 0.2541 0.4728

DIIS coefficients: 0.0000 0.0002 0.0009 0.0038 0.0096 0.0252 0.0721 0.1614 0.2541 0.4728

SCF Error: norm([F,P])= 0.000001900793, max([F,P])= 0.000000261713

orbitals (Q,E):

A :114...124 (2.00 -0.2487) (2.00 -0.2472) (2.00 -0.2364) (2.00 -0.2348)

(2.00 -0.2276) (2.00 -0.2213) (2.00 -0.2200) (2.00 -0.2151)

(2.00 -0.1953) (2.00 -0.1768) (0.00 -0.0694)

SCF CONVERGED

CYCLE 13

*** Setting up for NEW gradients in Focky

*** Using FIT density in Focky

*** Setting up for NEW Hessian in Focky

orbitals (Q,E):

A :114...133 (2.00 -0.2487) (2.00 -0.2472) (2.00 -0.2364) (2.00 -0.2348)

(2.00 -0.2276) (2.00 -0.2213) (2.00 -0.2200) (2.00 -0.2151)

(2.00 -0.1953) (2.00 -0.1768) (0.00 -0.0694) (0.00 -0.0687)

(0.00 -0.0622) (0.00 -0.0387) (0.00 -0.0186) (0.00 -0.0131)

(0.00 -0.0113) (0.00 -0.0084) (0.00 -0.0050) (0.00 -0.0002)

Fit test: (difference of exact and fit density, squared integrated, result summed over spins)

SCF: 0.00100774163075

Gross Charges per Atom (Z minus electrons)

==

-0.6629	0.9270	0.5437	0.3344	-0.5808	-0.3830	-0.6349	0.6739	0.6288	0.8651
-0.6597	-0.0013	-0.5777	0.5910	0.3268	-0.5912	0.6001	0.6010	-0.5987	0.2578
0.3402	0.0977	0.1679	0.7484	0.4103	0.4067	0.5636	0.5737	0.0282	0.6420
-0.5657	0.5511	0.5519	0.5720	0.6516	0.3720	0.7520	0.9863	0.5891	-0.2139
-0.1795	-0.2084	-0.1939	-0.2133	-0.2444	-0.2321	-0.1485	-0.1861	-0.1413	-0.2107
-0.2395	-0.2237	-0.2252	-0.1845	-0.2464	-0.1720	-0.1962	-0.1541	-0.1980	-0.2147
-0.2244	-0.2183	-0.2282	-0.1944	-0.1196	-0.2249	-0.1543	-0.2182	0.8296	-0.1969
-0.2185	-0.2170	-0.2115	-0.1912	-0.2119	-0.1789	-0.2275	-0.2350	-0.1976	-0.2421
-0.2136	-0.2535	-0.1974	-0.1605	-0.1655	0.2403	-0.6738	-0.1261	-0.1868	-0.1974
-0.1079	0.4927	-0.6123	-0.1749	-0.2160	-0.2384				

Net Total: 0.00000000

M U L T I P O L E   D E R I V E D   C H A R G E   A N A L Y S I S

See: M. Swart, P.Th. van Duijnen, J.G. Snijders, J.Comput.Chem., (2001), p. 79-88.

Multipole derived atomic charges (a.u.)

Atom		Level: MDC-m	MDC-d	MDC-q
1	O	-0.351166	-0.335390	-0.357862
2	C	0.363092	0.375740	0.397995
3	C	0.242373	0.251179	0.254950
4	C	0.180629	0.165733	0.211060
5	O	-0.307105	-0.550315	-0.620080
6	N	-0.214339	-0.275359	-0.465570
7	O	-0.379207	-0.370872	-0.439365
8	C	0.250397	0.309676	0.308660
9	C	0.228131	0.193409	0.187251
10	C	0.339656	0.409481	0.415807
11	O	-0.382848	-0.355431	-0.396255
12	C	0.124416	0.102506	0.102552
13	O	-0.312857	-0.299564	-0.347540
14	C	0.244836	0.244393	0.249311
15	C	0.195645	0.159220	0.175529
16	O	-0.369865	-0.478660	-0.484268

17	C	0.228588	0.193866	0.196920
18	C	0.229863	0.193236	0.195522
19	O	-0.318618	-0.320617	-0.385278
20	C	0.058928	0.046664	-0.058748
21	C	0.195065	0.221719	0.283942
22	C	0.118911	0.105011	0.108733
23	C	0.119940	0.099768	0.110166
24	C	0.290649	0.268066	0.288256
25	C	0.172751	0.135992	0.138370
26	C	0.164867	0.124577	0.164817
27	C	0.249129	0.236741	0.259171
28	C	0.234592	0.193744	0.293262
29	C	0.033788	0.026875	-0.052929
30	C	0.236918	0.191425	0.178681
31	O	-0.372144	-0.476618	-0.518145
32	C	0.254151	0.243491	0.263733
33	C	0.224852	0.187761	0.176702
34	C	0.227402	0.295081	0.388641
35	C	0.222024	0.192312	0.202356
36	C	0.180385	0.145856	0.151499
37	C	0.294022	0.270852	0.312626
38	C	0.368070	0.339525	0.387003
39	C	0.229527	0.190262	0.155958
40	H	-0.072931	-0.059891	-0.022302
41	H	-0.058676	-0.033245	-0.018789
42	H	-0.062604	-0.054650	-0.024170
43	H	-0.056023	-0.034415	-0.006434
44	H	-0.068908	-0.052796	-0.051043
45	H	-0.070592	-0.057821	-0.052681
46	H	-0.076399	-0.071419	-0.069495
47	H	-0.071743	-0.061440	-0.061279

48	H	-0.064325	-0.043458	-0.024101
49	H	-0.082462	-0.063417	-0.071337
50	H	-0.055643	-0.048413	-0.048590
51	H	-0.072260	-0.054691	-0.059629
52	H	-0.071240	-0.060054	-0.066297
53	H	-0.067800	-0.063731	-0.073852
54	H	-0.058236	-0.045635	-0.052822
55	H	-0.054308	-0.044162	-0.045822
56	H	-0.057536	-0.042952	-0.030438
57	H	-0.067293	-0.050366	-0.050819
58	H	-0.061867	-0.036548	-0.034772
59	H	-0.075524	-0.060789	-0.050007
60	H	-0.074819	-0.065314	-0.055824
61	H	-0.069010	-0.055402	-0.043369
62	H	-0.066742	-0.047989	-0.037295
63	H	-0.072674	-0.075357	-0.083562
64	H	-0.058368	-0.036217	-0.036475
65	H	-0.073704	-0.048053	-0.055857
66	H	-0.072488	-0.063988	-0.059175
67	H	-0.067511	-0.053565	-0.064124
68	H	-0.065372	-0.057167	-0.055470
69	C	0.351670	0.444052	0.468259
70	H	-0.064693	-0.054149	-0.079104
71	H	-0.068038	-0.067241	-0.095814
72	H	-0.063480	-0.048625	-0.071013
73	H	-0.080682	-0.063254	-0.073483
74	H	-0.072893	-0.060463	-0.065450
75	H	-0.069506	-0.069038	-0.071656
76	H	-0.075986	-0.045743	-0.036992
77	H	-0.066468	-0.054091	-0.040851
78	H	-0.065190	-0.061098	-0.031159

79	H	-0.083417	-0.064363	-0.048666
80	H	-0.066607	-0.057908	-0.052327
81	H	-0.068016	-0.067020	-0.057069
82	H	-0.071122	-0.044683	-0.038086
83	H	-0.062214	-0.023772	-0.018768
84	H	-0.046557	0.000163	-0.013661
85	H	-0.073438	-0.059827	-0.040515
86	H	0.062652	0.264387	0.307907
87	O	-0.361750	-0.439586	-0.457483
88	H	-0.044870	0.000790	0.021761
89	H	-0.045963	-0.031490	-0.025911
90	H	-0.052491	-0.051485	-0.051001
91	H	-0.042075	-0.012193	-0.021524
92	C	0.240509	0.197680	0.216953
93	O	-0.366792	-0.472125	-0.500323
94	H	-0.061324	-0.053976	-0.049668
95	H	-0.061024	-0.047688	-0.040218
96	H	-0.068627	-0.065643	-0.061743

*** Using NEW gradient routines ***

Geometry CYCLE 10

==============

Energy gradients wrt nuclear displacements

==

Atom Cartesian (a.u./angstrom)

X Y Z

----

1 O 0.000518 -0.000318 -0.000792

2	C	0.000465	0.000214	0.000684
3	C	-0.000305	0.000623	0.000462
4	C	0.000303	0.000711	0.000320
5	O	-0.001785	-0.000255	0.001315
6	N	-0.000273	-0.000402	-0.000223
7	O	0.000243	-0.000337	0.000295
8	C	-0.001266	0.001641	-0.000683
9	C	-0.000012	0.000058	-0.000036
10	C	0.000892	-0.001726	0.000985
11	O	-0.000168	-0.000228	0.000633
12	C	0.000284	0.000204	0.000713
13	O	0.000236	0.000138	-0.000716
14	C	-0.000575	0.000685	0.000272
15	C	0.000346	0.000224	-0.000395
16	O	0.001336	-0.000893	-0.000562
17	C	0.000256	-0.000242	-0.000130
18	C	0.000353	-0.000024	-0.000157
19	O	-0.000742	0.000087	-0.000944
20	C	-0.000317	0.000321	0.000886
21	C	-0.000319	0.000389	0.000100
22	C	-0.000034	-0.000258	-0.000053
23	C	0.000567	0.000139	0.000285
24	C	0.000216	0.000009	-0.000270
25	C	-0.000172	-0.000070	-0.000106
26	C	-0.000153	-0.000285	0.000021
27	C	0.000363	-0.000170	-0.000149
28	C	-0.000090	0.000035	-0.000034
29	C	0.000263	0.000174	0.000313
30	C	-0.000183	0.000153	-0.000211
31	O	-0.000788	-0.000956	0.001014
32	C	-0.000746	0.000080	0.000177

33	C	0.000384	0.000086	-0.000936
34	C	-0.000108	0.000427	-0.001405
35	C	-0.000167	-0.000174	0.000035
36	C	0.000261	-0.000113	0.000402
37	C	-0.000192	0.000347	0.000003
38	C	0.000210	-0.000210	0.000242
39	C	-0.000144	0.000236	-0.000072
40	H	0.000284	-0.000191	-0.000576
41	H	-0.000247	-0.000355	-0.000273
42	H	0.000267	0.000343	-0.000077
43	H	0.000001	0.000118	0.000182
44	H	-0.000079	-0.000030	-0.000047
45	H	-0.000023	-0.000018	0.000071
46	H	0.000106	-0.000072	-0.000155
47	H	0.000195	-0.000031	0.000035
48	H	-0.000033	0.000017	0.000075
49	H	-0.000121	-0.000218	-0.000217
50	H	-0.000105	0.000080	0.000009
51	H	0.000094	-0.000090	0.000082
52	H	-0.000073	-0.000224	0.000169
53	H	-0.000021	-0.000037	0.000063
54	H	0.000036	-0.000142	0.000115
55	H	0.000110	0.000149	0.000014
56	H	0.000048	-0.000087	0.000073
57	H	-0.000064	0.000029	0.000005
58	H	-0.000023	0.000137	-0.000073
59	H	0.000003	-0.000025	0.000006
60	H	0.000083	0.000055	0.000096
61	H	0.000071	0.000076	-0.000028
62	H	0.000028	0.000126	0.000083
63	H	0.000046	-0.000070	0.000010

64	H	0.000007	0.000040	-0.000071
65	H	-0.000299	-0.000468	-0.000281
66	H	0.000052	-0.000084	-0.000057
67	H	-0.000197	0.000152	0.000029
68	H	-0.000180	-0.000055	-0.000172
69	C	0.001761	0.000806	0.000862
70	H	-0.000024	-0.000051	-0.000003
71	H	0.000025	-0.000023	-0.000092
72	H	0.000012	0.000021	-0.000013
73	H	-0.000009	0.000019	-0.000066
74	H	-0.000064	-0.000004	0.000056
75	H	0.000127	-0.000032	-0.000014
76	H	-0.000131	0.000086	0.000123
77	H	-0.000082	-0.000116	0.000058
78	H	-0.000064	0.000078	0.000085
79	H	0.000055	0.000006	0.000070
80	H	0.000011	0.000039	-0.000099
81	H	-0.000040	0.000013	-0.000059
82	H	0.000027	-0.000300	-0.000137
83	H	-0.000202	-0.000035	-0.000228
84	H	0.000011	-0.000300	-0.000231
85	H	0.000023	-0.000087	-0.000101
86	H	0.000435	0.000076	-0.000315
87	O	0.000277	0.001430	-0.000992
88	H	-0.000023	-0.000125	-0.000150
89	H	0.000096	0.000066	0.000166
90	H	-0.000019	0.000171	-0.000104
91	H	0.000100	0.000015	-0.000038
92	C	-0.000283	-0.000206	-0.000133
93	O	-0.001149	-0.000740	0.000884
94	H	0.000019	-0.000212	0.000020

95 H 0.000163 -0.000088 -0.000029

96 H 0.000023 0.000050 0.000099

----

dE(predicted): -0.98290E-04, dE(actual): -0.77135E-04, Trust radius: 0.195959, all in a.u.

Max Cartesian displacement 0.726062785346130

Limit Cartesian step to 0.566917837497000

Geometry Convergence after Step 10 (Hartree/Angstrom,Angstrom)

----

current energy	-19.67444846 Hartree		
energy change	-0.00007713	0.00100000	T
constrained gradient max	0.00178494	0.00100000	F
constrained gradient rms	0.00041109	0.00066667	T
gradient max	0.00178494		
gradient rms	0.00041109		
cart. step max	0.30225871	0.01000000	F
cart. step rms	0.07049354	0.00666667	F

Coordinates (Cartesian)

======================

Atom	bohr X	bohr Y	bohr Z	angstrom X	angstrom Y	angstrom Z	Geometric Variables (0:frozen, *:LT par.)		
1 O	26.627365	61.564139	50.465356	14.090595	32.578339	26.705117	1	2	3
2 C	27.066908	61.940190	53.044264	14.323191	32.777337	28.069816	4	5	6
3 C	29.855320	61.250287	53.511875	15.798755	32.412256	28.317265	7	8	9

4 C	30.575888	61.681301	56.310492	16.180063	32.640339	29.798229	10	11	12
5 O	27.202189	52.913973	42.937769	14.394779	28.000869	22.721689	13	14	15
6 N	33.205132	61.097797	57.031163	17.571399	32.331562	30.179592	16	17	18
7 O	26.641164	64.549711	53.664114	14.097897	34.158236	28.397826	19	20	21
8 C	20.491498	58.441949	48.390801	10.843634	30.926147	25.607309	22	23	24
9 C	25.682669	65.407342	41.515538	13.590683	34.612075	21.969076	25	26	27
10 C	21.624119	55.058225	44.973799	11.442991	29.135558	23.799109	28	29	30
11 O	21.720955	62.721694	45.733716	11.494234	33.190891	24.201240	31	32	33
12 C	19.546016	56.565675	46.291258	10.343306	29.933266	24.496279	34	35	36
13 O	21.520003	55.240896	42.438336	11.387895	29.232223	22.457400	37	38	39
14 C	24.042338	61.486845	49.549235	12.722657	32.537437	26.220326	40	41	42
15 C	24.046063	63.223821	47.138042	12.724629	33.456605	24.944378	43	44	45
16 O	18.886288	59.483711	49.682232	9.994193	31.477424	26.290705	46	47	48
17 C	23.847315	56.911871	51.247915	12.619456	30.116465	27.119229	49	50	51
18 C	27.274024	53.224378	38.445309	14.432792	28.165128	20.344381	52	53	54
19 O	30.165969	58.585753	52.848889	15.963143	31.002245	27.966428	55	56	57
20 C	26.278955	57.093393	40.999566	13.906224	30.212522	21.696036	58	59	60
21 C	25.983982	54.216884	40.877515	13.750131	28.690340	21.631449	61	62	63
22 C	23.303454	58.704135	48.986470	12.331657	31.064890	25.922523	64	65	66
23 C	25.923227	62.684989	42.488093	13.717981	33.171468	22.483730	67	68	69
24 C	35.174683	62.137168	55.369777	18.613641	32.881573	29.300424	70	71	72
25 C	26.298400	62.429970	45.393013	13.916514	33.036517	24.020948	73	74	75
26 C	29.868826	64.437043	57.000945	15.805902	34.098615	30.163601	76	77	78
27 C	27.108140	65.067010	56.328757	14.345010	34.431979	29.807895	79	80	81
28 C	26.422882	67.834404	56.760477	13.982387	35.896421	30.036351	82	83	84
29 C	28.341893	58.453068	41.602237	14.997884	30.932032	22.014956	85	86	87
30 C	17.638693	57.868487	44.513598	9.333994	30.622685	23.555582	88	89	90
31 O	29.979908	62.463726	40.380838	15.864684	33.054381	21.368619	91	92	93
32 C	23.087503	53.577914	40.753282	12.217381	28.352211	21.565708	94	95	96
33 C	30.902961	57.461746	42.393939	16.353143	30.407446	22.433906	97	98	99

34 C	28.213222	61.309418	41.336590	14.929794	32.443547	21.874381	100	101	102
35 C	24.315921	65.999911	47.973148	12.867431	34.925649	25.386297	103	104	105
36 C	22.401989	50.790048	41.140994	11.854622	26.876936	21.770876	106	107	108
37 C	33.687044	58.432305	57.644849	17.826416	30.921044	30.504340	109	110	111
38 C	19.502390	64.144698	46.412702	10.320220	33.943912	24.560544	112	113	114
39 C	19.548179	50.259909	40.966735	10.344451	26.596399	21.678663	115	116	117
40 H	24.609924	58.126464	40.382234	13.023011	30.759200	21.369358	118	119	120
41 H	31.370149	58.159286	44.296788	16.600368	30.776569	23.440851	121	122	123
42 H	30.980912	55.404698	42.413977	16.394393	29.318904	22.444510	124	125	126
43 H	32.348158	58.230395	41.121675	17.117908	30.814198	21.760653	127	128	129
44 H	27.272272	66.571140	42.153286	14.431865	35.227930	22.306558	130	131	132
45 H	25.679602	65.445250	39.444201	13.589060	34.632135	20.872972	133	134	135
46 H	23.916672	66.252084	42.183427	12.656158	35.059093	22.322508	136	137	138
47 H	24.201301	61.637353	42.010533	12.806777	32.617082	22.231017	139	140	141
48 H	27.968913	63.510781	45.960708	14.800511	33.608458	24.321359	142	143	144
49 H	26.769312	60.456059	45.779906	14.165710	31.991969	24.225683	145	146	147
50 H	26.104446	66.218860	48.980448	13.813878	35.041512	25.919337	148	149	150
51 H	22.814605	66.562666	49.280360	12.072969	35.223446	26.078044	151	152	153
52 H	24.308434	67.270234	46.344634	12.863469	35.597875	24.524524	154	155	156

53 H	19.733962	66.162825	45.993012	10.442763	35.011859	24.338454	157	158	159
54 H	18.986035	63.890122	48.406454	10.046977	33.809197	25.615592	160	161	162
55 H	17.977012	63.388475	45.243794	9.513025	33.543737	23.941985	163	164	165
56 H	22.392487	54.210031	38.907020	11.849594	28.686713	20.588708	166	167	168
57 H	23.399208	49.716277	39.674890	12.382328	26.308721	20.995048	169	170	171
58 H	23.148794	50.136945	42.952449	12.249814	26.531329	22.729457	172	173	174
59 H	18.504340	51.222983	42.475161	9.792075	27.106035	22.476887	175	176	177
60 H	18.763426	50.896181	39.154099	9.929178	26.933099	20.719457	178	179	180
61 H	19.181508	48.229028	41.143905	10.150417	25.521703	21.772417	181	182	183
62 H	27.068334	51.171397	38.288915	14.323945	27.078737	20.261621	184	185	186
63 H	26.483596	54.128372	36.758898	14.014515	28.643501	19.451971	187	188	189
64 H	29.289558	53.670509	38.535126	15.499367	28.401210	20.391911	190	191	192
65 H	24.417798	58.046377	47.377746	12.921342	30.716820	25.071224	193	194	195
66 H	22.725713	57.471410	52.897804	12.025929	30.412561	27.992313	196	197	198
67 H	25.848624	56.972494	51.743014	13.678503	30.148545	27.381224	199	200	201
68 H	23.359781	54.969341	50.738433	12.361464	29.088522	26.849622	202	203	204
69 C	31.202600	58.040117	50.516031	16.511705	30.713507	26.731932	205	206	207
70 H	27.622124	69.059391	55.596356	14.616999	36.544656	29.420324	208	209	210
71 H	26.692816	68.342207	58.751446	14.125230	36.165138	31.089926	211	212	213

72 H	24.445432	68.177298	56.250893	12.935965	36.077873	29.766691	214	215	216
73 H	25.838685	63.851549	57.462071	13.673243	33.788784	30.407618	217	218	219
74 H	31.101808	65.764475	55.989544	16.458368	34.801061	29.628391	220	221	222
75 H	30.189270	64.708400	59.030652	15.975473	34.242211	31.237676	223	224	225
76 H	35.321939	61.156380	53.528954	18.691565	32.362562	28.326303	226	227	228
77 H	34.827969	64.142862	55.000610	18.430167	33.942941	29.105069	229	230	231
78 H	37.006738	61.985087	56.327048	19.583123	32.801096	29.806990	232	233	234
79 H	33.734826	57.177345	55.975470	17.851701	30.256948	29.620943	235	236	237
80 H	32.222898	57.734056	58.932412	17.051623	30.551547	31.185689	238	239	240
81 H	35.514206	58.287251	58.616501	18.793308	30.844285	31.018517	241	242	243
82 H	25.811574	60.749334	54.218964	13.658897	32.147163	28.691440	244	245	246
83 H	22.749811	62.271648	50.966853	12.038682	32.952737	26.970497	247	248	249
84 H	31.008299	62.400445	52.240060	16.408885	33.020893	27.644249	250	251	252
85 H	29.378560	60.421712	57.450079	15.546465	31.973793	30.401273	253	254	255
86 H	26.116940	53.144991	44.426529	13.820490	28.123118	23.509507	256	257	258
87 O	23.161455	53.773272	46.149090	12.256514	28.455590	24.421047	259	260	261
88 H	18.541975	55.125390	47.410965	9.811991	29.171100	25.088802	262	263	264
89 H	32.520804	54.644379	48.754555	17.209269	28.916560	25.799800	265	266	267
90 H	30.802380	54.113558	51.647505	16.299918	28.635662	27.330683	268	269	270

91 H 29.188690 54.996307 48.837009 15.445989 29.102792 25.843432 271 272 273

92 C 30.934426 55.252907 49.929118 16.369793 29.238579 26.421351 274 275 276

93 O 32.146255 59.591302 49.114291 17.011066 31.534359 25.990164 277 278 279

94 H 18.602948 59.246341 43.313077 9.844256 31.351813 22.920293 280 281 282

95 H 16.210334 58.837728 45.649370 8.578139 31.135585 24.156606 283 284 285

96 H 16.707868 56.479132 43.296998 8.841423 29.887469 22.911785 286 287 288

Number of elements of the density matrix on this node (used, total): 222269 1773786

Numerical Integration : Fuzzy Cells (Becke)

Becke grid quality: NORMAL

Lebedev angular grid order: min: 11 max: 23

Nr. of radial points: min: 46 max: 52

Total nr. of points: 600131

Nr. of blocks: 4689

Block length: 128

Nr. of dummy points: 61

Test of Precision of the Numerical Integration Grid

==

Integral of the Total Core Density: 85.99993955619357

Relative Error: -7.028E-07

Density Fitting (zlmFit)

ZlmFit Fit Quality: NORMAL

Max L-expansion: min: 6 max: 7

Nr. of radial interpolation points: min: 39 max: 48

S C F *** ScaSCF ***

Initial density matrix is taken from previous geometry

CYCLE 1

SCF Error: norm([F,P])= 0.225985513570, max([F,P])= 0.023065049320

orbitals (Q,E):

A :114...133 (2.00 -0.2553) (2.00 -0.2539) (2.00 -0.2454) (2.00 -0.2410)

(2.00 -0.2345) (2.00 -0.2272) (2.00 -0.2261) (2.00 -0.2209)

(2.00   -0.1999) (2.00   -0.1831) (0.00   -0.0749) (0.00   -0.0741)

(0.00   -0.0696) (0.00   -0.0483) (0.00   -0.0234) (0.00   -0.0183)

(0.00   -0.0165) (0.00   -0.0135) (0.00   -0.0100) (0.00   -0.0050)

CYCLE   2

SDIIS (wt 0.000): 0.0542 0.9458

A-DIIS (wt 1.000): 0.0000 1.0000

DIIS coefficients: 0.0000 1.0000

SCF Error: norm([F,P])=   0.047039959526, max([F,P])=   0.004679271318

orbitals (Q,E):

A :114...124   (2.00   -0.2451) (2.00   -0.2442) (2.00   -0.2342) (2.00   -0.2313)

(2.00   -0.2244) (2.00   -0.2195) (2.00   -0.2163) (2.00   -0.2130)

(2.00   -0.1954) (2.00   -0.1764) (0.00   -0.0671)

CYCLE   3

SDIIS (wt 0.000): 0.0070 0.6888 0.3043

A-DIIS (wt 1.000): 0.0000 0.7574 0.2426

DIIS coefficients: 0.0000 0.7574 0.2426

SCF Error: norm([F,P])=   0.100547200185, max([F,P])=   0.012477602778

orbitals (Q,E):

A :114...124   (2.00   -0.2473) (2.00   -0.2462) (2.00   -0.2349) (2.00   -0.2328)

(2.00   -0.2260) (2.00   -0.2205) (2.00   -0.2184) (2.00   -0.2139)

(2.00   -0.1937) (2.00   -0.1767) (0.00   -0.0681)

CYCLE   4

SDIIS (wt 0.979): 0.4133 0.1348 0.4520

A-DIIS (wt 0.021): 0.1371 0.0000 0.8629

DIIS coefficients: 0.4074 0.1319 0.4606

SCF Error: norm([F,P])=   0.022590069064, max([F,P])=   0.003094011882

orbitals (Q,E):

A :114...124 (2.00 -0.2484) (2.00 -0.2473) (2.00 -0.2371) (2.00 -0.2342)

(2.00 -0.2274) (2.00 -0.2211) (2.00 -0.2195) (2.00 -0.2148)

(2.00 -0.1947) (2.00 -0.1771) (0.00 -0.0688)

CYCLE 5

SDIIS (wt 1.000): 0.1632 0.0408 0.2724 0.5235

DIIS coefficients: 0.1632 0.0408 0.2724 0.5235

SCF Error: norm([F,P])= 0.006573775313, max([F,P])= 0.000942037202

orbitals (Q,E):

A :114...124 (2.00 -0.2482) (2.00 -0.2471) (2.00 -0.2369) (2.00 -0.2341)

(2.00 -0.2273) (2.00 -0.2211) (2.00 -0.2194) (2.00 -0.2148)

(2.00 -0.1945) (2.00 -0.1773) (0.00 -0.0688)

CYCLE 6

SDIIS (wt 1.000): 0.0704 0.0151 0.1348 0.3488 0.4309

DIIS coefficients: 0.0704 0.0151 0.1348 0.3488 0.4309

SCF Error: norm([F,P])= 0.002620902749, max([F,P])= 0.000251054717

orbitals (Q,E):

A :114...124 (2.00 -0.2483) (2.00 -0.2472) (2.00 -0.2368) (2.00 -0.2341)

(2.00 -0.2272) (2.00 -0.2211) (2.00 -0.2194) (2.00 -0.2147)

(2.00 -0.1945) (2.00 -0.1772) (0.00 -0.0688)

CYCLE 7

SDIIS (wt 1.000): 0.0227 0.0041 0.0491 0.1602 0.2987 0.4652

DIIS coefficients: 0.0227 0.0041 0.0491 0.1602 0.2987 0.4652

SCF Error: norm([F,P])= 0.000976836728, max([F,P])= 0.000143580716

orbitals (Q,E):

A :114...124 (2.00 -0.2483) (2.00 -0.2472) (2.00 -0.2369) (2.00 -0.2341)

(2.00 -0.2273) (2.00 -0.2211) (2.00 -0.2194) (2.00 -0.2147)

(2.00 -0.1945) (2.00 -0.1772) (0.00 -0.0688)

CYCLE 8

SDIIS (wt 1.000): 0.0068 0.0009 0.0172 0.0693 0.1608 0.3454 0.3995

DIIS coefficients: 0.0068 0.0009 0.0172 0.0693 0.1608 0.3454 0.3995

SCF Error: norm([F,P])= 0.000446431085, max([F,P])= 0.000064909039

orbitals (Q,E):

A :114...124 (2.00 -0.2483) (2.00 -0.2472) (2.00 -0.2369) (2.00 -0.2342)

(2.00 -0.2273) (2.00 -0.2211) (2.00 -0.2194) (2.00 -0.2147)

(2.00 -0.1946) (2.00 -0.1772) (0.00 -0.0688)

CYCLE 9

SDIIS (wt 1.000): 0.0015 0.0001 0.0052 0.0262 0.0721 0.1805 0.2807 0.4336

DIIS coefficients: 0.0015 0.0001 0.0052 0.0262 0.0721 0.1805 0.2807 0.4336

SCF Error: norm([F,P])= 0.000148619937, max([F,P])= 0.000016646179

orbitals (Q,E):

A :114...124 (2.00 -0.2483) (2.00 -0.2472) (2.00 -0.2369) (2.00 -0.2342)

(2.00 -0.2273) (2.00 -0.2211) (2.00 -0.2194) (2.00 -0.2147)

(2.00 -0.1945) (2.00 -0.1772) (0.00 -0.0688)

CYCLE 10

SDIIS (wt 1.000): 0.0001 -0.0001 0.0011 0.0075 0.0249 0.0720 0.1334 0.3095 0.4515

DIIS coefficients: 0.0001 -0.0001 0.0011 0.0075 0.0249 0.0720 0.1334 0.3095 0.4515

SCF Error: norm([F,P])= 0.000057536165, max([F,P])= 0.000008432908

orbitals (Q,E):

A :114...124 (2.00 -0.2483) (2.00 -0.2472) (2.00 -0.2369) (2.00 -0.2342)

(2.00 -0.2273) (2.00 -0.2211) (2.00 -0.2194) (2.00 -0.2147)

(2.00 -0.1945) (2.00 -0.1772) (0.00 -0.0688)

CYCLE 11

SDIIS (wt 1.000): -0.0001 -0.0001 0.0001 0.0019 0.0085 0.0285 0.0605 0.1688 0.3361 0.3958

DIIS coefficients: -0.0001 -0.0001 0.0001 0.0019 0.0085 0.0285 0.0605 0.1688 0.3361 0.3958

SCF Error: norm([F,P])= 0.000025184570, max([F,P])= 0.000004795840

orbitals (Q,E):

A :114...124 (2.00 -0.2483) (2.00 -0.2472) (2.00 -0.2369) (2.00 -0.2342)

(2.00 -0.2273) (2.00 -0.2211) (2.00 -0.2194) (2.00 -0.2147)

(2.00 -0.1945) (2.00 -0.1772) (0.00 -0.0688)

CYCLE 12

SDIIS (wt 1.000): -0.0000 0.0001 0.0008 0.0033 0.0110 0.0242 0.0704 0.1606 0.2603 0.4692

DIIS coefficients: -0.0000 0.0001 0.0008 0.0033 0.0110 0.0242 0.0704 0.1606 0.2603 0.4692

SCF Error: norm([F,P])= 0.000007302046, max([F,P])= 0.000001057761

orbitals (Q,E):

A :114...124 (2.00 -0.2483) (2.00 -0.2472) (2.00 -0.2369) (2.00 -0.2342)

(2.00 -0.2273) (2.00 -0.2211) (2.00 -0.2194) (2.00 -0.2147)

(2.00 -0.1945) (2.00 -0.1772) (0.00 -0.0688)

CYCLE 13

SDIIS (wt 1.000): 0.0000 0.0001 0.0007 0.0027 0.0074 0.0240 0.0637 0.1220 0.3027 0.4767

DIIS coefficients: 0.0000 0.0001 0.0007 0.0027 0.0074 0.0240 0.0637 0.1220 0.3027 0.4767

SCF Error: norm([F,P])= 0.000002429918, max([F,P])= 0.000000334917

orbitals (Q,E):

A :114...124 (2.00 -0.2483) (2.00 -0.2472) (2.00 -0.2369) (2.00 -0.2342)

(2.00 -0.2273) (2.00 -0.2211) (2.00 -0.2194) (2.00 -0.2147)

(2.00 -0.1945) (2.00 -0.1772) (0.00 -0.0688)

SCF CONVERGED

CYCLE 14

*** Setting up for NEW gradients in Focky

*** Using FIT density in Focky

*** Setting up for NEW Hessian in Focky

orbitals (Q,E):

A :114...133 (2.00 -0.2483) (2.00 -0.2472) (2.00 -0.2369) (2.00 -0.2342)

(2.00 -0.2273) (2.00 -0.2211) (2.00 -0.2194) (2.00 -0.2147)

(2.00 -0.1945) (2.00 -0.1772) (0.00 -0.0688) (0.00 -0.0681)

(0.00 -0.0624) (0.00 -0.0389) (0.00 -0.0183) (0.00 -0.0130)

(0.00 -0.0107) (0.00 -0.0078) (0.00 -0.0049) (0.00 -0.0000)

Fit test: (difference of exact and fit density, squared integrated, result summed over spins)

SCF: 0.00100808808378

Gross Charges per Atom (Z minus electrons)

==

-0.6639	0.9242	0.5467	0.3331	-0.5808	-0.3816	-0.6334	0.6735	0.6256	0.8661
-0.6593	-0.0005	-0.5773	0.5891	0.3254	-0.5917	0.5975	0.6003	-0.6006	0.2575
0.3390	0.0974	0.1675	0.7470	0.4129	0.4072	0.5617	0.5731	0.0227	0.6409
-0.5656	0.5507	0.5496	0.5719	0.6469	0.3716	0.7509	0.9850	0.5877	-0.2138
-0.1571	-0.2127	-0.1987	-0.2132	-0.2436	-0.2322	-0.1502	-0.1859	-0.1331	-0.2093
-0.2399	-0.2235	-0.2260	-0.1833	-0.2457	-0.1715	-0.1960	-0.1540	-0.1977	-0.2144
-0.2241	-0.2190	-0.2281	-0.1937	-0.1167	-0.2248	-0.1548	-0.2170	0.8280	-0.1967
-0.2186	-0.2171	-0.2108	-0.1907	-0.2116	-0.1840	-0.2255	-0.2329	-0.1980	-0.2411

-0.2125 -0.2529 -0.1993 -0.1609 -0.1660 0.2366 -0.6747 -0.1260 -0.1905 -0.2030

-0.0931 0.4989 -0.6165 -0.1748 -0.2165 -0.2380

Net Total: 0.00000000

M U L T I P O L E   D E R I V E D   C H A R G E   A N A L Y S I S

See: M. Swart, P.Th. van Duijnen, J.G. Snijders, J.Comput.Chem., (2001), p. 79-88.

Multipole derived atomic charges (a.u.)

Atom	Level:	MDC-m	MDC-d	MDC-q
1	O	-0.350485	-0.324152	-0.347176
2	C	0.362805	0.374742	0.396437
3	C	0.242446	0.250264	0.254282
4	C	0.180827	0.165894	0.211172
5	O	-0.307692	-0.550812	-0.616074
6	N	-0.214145	-0.275168	-0.464828
7	O	-0.379818	-0.376688	-0.443731
8	C	0.250347	0.309722	0.308562
9	C	0.227984	0.193083	0.188244
10	C	0.339691	0.408954	0.414760
11	O	-0.383048	-0.356801	-0.398446
12	C	0.124301	0.102446	0.102653
13	O	-0.312776	-0.299859	-0.345949

14	C	0.244999	0.243355	0.248389
15	C	0.195872	0.159171	0.175448
16	O	-0.369783	-0.478600	-0.483918
17	C	0.227727	0.193063	0.198078
18	C	0.229743	0.193331	0.195989
19	O	-0.319068	-0.321379	-0.378667
20	C	0.057819	0.045212	-0.055747
21	C	0.194993	0.221772	0.283576
22	C	0.118624	0.105006	0.107973
23	C	0.119894	0.099713	0.110938
24	C	0.290874	0.268540	0.287659
25	C	0.172836	0.134992	0.136986
26	C	0.164814	0.124611	0.164628
27	C	0.249081	0.237509	0.259862
28	C	0.234541	0.193713	0.299109
29	C	0.034311	0.027325	-0.048288
30	C	0.236925	0.191108	0.177409
31	O	-0.372102	-0.476554	-0.516251
32	C	0.254245	0.243538	0.263657
33	C	0.223311	0.186162	0.182765
34	C	0.227962	0.295614	0.384184
35	C	0.222068	0.192172	0.201932
36	C	0.180325	0.145690	0.150406
37	C	0.294042	0.270894	0.311216
38	C	0.367924	0.339565	0.387396
39	C	0.229399	0.190088	0.156890
40	H	-0.073367	-0.060006	-0.025778
41	H	-0.057525	-0.031665	-0.017593
42	H	-0.063804	-0.056615	-0.028806
43	H	-0.056463	-0.034969	-0.010736
44	H	-0.068971	-0.052897	-0.051540

45	H	-0.070609	-0.057710	-0.053339
46	H	-0.076537	-0.071928	-0.070118
47	H	-0.071954	-0.062025	-0.061768
48	H	-0.063664	-0.041673	-0.022651
49	H	-0.082117	-0.061682	-0.069356
50	H	-0.055465	-0.047802	-0.049166
51	H	-0.072455	-0.054928	-0.059957
52	H	-0.070931	-0.059518	-0.065770
53	H	-0.067911	-0.064104	-0.073710
54	H	-0.057987	-0.044949	-0.052448
55	H	-0.054258	-0.043957	-0.045686
56	H	-0.057320	-0.042689	-0.030588
57	H	-0.067173	-0.050281	-0.050924
58	H	-0.061877	-0.036387	-0.033494
59	H	-0.075427	-0.060718	-0.050232
60	H	-0.074803	-0.065361	-0.056095
61	H	-0.068992	-0.055286	-0.043529
62	H	-0.066991	-0.048491	-0.039120
63	H	-0.072709	-0.075301	-0.083809
64	H	-0.058211	-0.035863	-0.035372
65	H	-0.074226	-0.048185	-0.056622
66	H	-0.072665	-0.064494	-0.060717
67	H	-0.068021	-0.053980	-0.066251
68	H	-0.065151	-0.057140	-0.056120
69	C	0.352441	0.440371	0.460377
70	H	-0.064655	-0.054154	-0.080804
71	H	-0.068126	-0.067355	-0.097107
72	H	-0.063289	-0.048408	-0.072802
73	H	-0.080728	-0.062958	-0.073004
74	H	-0.072926	-0.060247	-0.064611
75	H	-0.069422	-0.068816	-0.072417

76	H	-0.076959	-0.047938	-0.040887
77	H	-0.066354	-0.054003	-0.040143
78	H	-0.064859	-0.060371	-0.030078
79	H	-0.082993	-0.064275	-0.047802
80	H	-0.066553	-0.057709	-0.052002
81	H	-0.067878	-0.066840	-0.056893
82	H	-0.070917	-0.044797	-0.038073
83	H	-0.062007	-0.025193	-0.018890
84	H	-0.046902	-0.001856	-0.014678
85	H	-0.073492	-0.059858	-0.041100
86	H	0.061636	0.262568	0.299518
87	O	-0.361595	-0.438065	-0.454703
88	H	-0.044773	0.000646	0.021329
89	H	-0.045409	-0.030487	-0.020777
90	H	-0.052401	-0.051331	-0.045223
91	H	-0.042009	-0.011011	-0.015054
92	C	0.240654	0.197661	0.198805
93	O	-0.364054	-0.465435	-0.492766
94	H	-0.061113	-0.053596	-0.049533
95	H	-0.061033	-0.047746	-0.039580
96	H	-0.068515	-0.065431	-0.061334

*** Using NEW gradient routines ***

Geometry CYCLE 11

==============

Energy gradients wrt nuclear displacements

==

Atom Cartesian (a.u./angstrom)

	X	Y	Z
1 O	0.000844	-0.001812	-0.001758
2 C	0.000960	-0.000850	0.001237
3 C	-0.000863	0.000805	0.000644
4 C	0.000778	0.000915	-0.000215
5 O	-0.001853	-0.000937	0.000619
6 N	-0.000466	0.000054	0.000481
7 O	0.000454	0.000395	0.000335
8 C	-0.001103	0.001493	-0.000436
9 C	-0.000117	0.000139	-0.000358
10 C	0.003011	0.000873	0.001668
11 O	-0.001423	-0.001227	0.001779
12 C	0.000039	-0.000092	0.001007
13 O	-0.000001	0.000013	-0.001908
14 C	-0.001167	0.001761	-0.000186
15 C	0.001054	0.000371	-0.000505
16 O	0.001097	0.000186	-0.000948
17 C	0.000817	0.000171	-0.000702
18 C	0.000411	-0.000111	-0.000025
19 O	-0.003605	0.000564	-0.001814
20 C	-0.000308	-0.000089	0.001002
21 C	-0.000879	0.000202	0.000589
22 C	0.000632	-0.000558	-0.000499
23 C	0.000884	-0.000279	-0.000725
24 C	0.000582	-0.000397	-0.000193
25 C	-0.000071	-0.000229	-0.000181
26 C	0.000584	-0.000708	0.000271
27 C	0.000005	-0.000005	-0.000129
28 C	0.000102	-0.000183	-0.000338
29 C	-0.000532	0.000793	0.000191

30	C	-0.000461	-0.000148	-0.000272
31	O	-0.000550	-0.000712	0.000595
32	C	-0.000863	0.000189	0.000530
33	C	0.000363	-0.000499	-0.001879
34	C	0.000084	0.000450	0.000522
35	C	0.000019	0.000106	0.000188
36	C	0.000117	0.000155	-0.000078
37	C	0.000085	0.000104	-0.000044
38	C	0.000581	-0.000998	0.000508
39	C	0.000199	0.000187	-0.000012
40	H	0.000221	-0.000017	-0.001003
41	H	0.000173	0.000168	0.000388
42	H	0.000340	0.000404	-0.000111
43	H	0.000534	0.000397	0.000181
44	H	0.000150	0.000205	0.000038
45	H	-0.000102	0.000098	-0.000299
46	H	0.000059	-0.000030	-0.000419
47	H	0.000196	0.000050	0.000267
48	H	0.000251	0.000199	0.000075
49	H	-0.000233	-0.001083	-0.000112
50	H	-0.000023	0.000458	0.000208
51	H	0.000007	-0.000029	0.000526
52	H	-0.000076	-0.000171	0.000393
53	H	0.000014	0.000403	0.000136
54	H	-0.000102	-0.000355	0.000784
55	H	-0.000294	-0.000043	-0.000296
56	H	-0.000200	-0.000054	-0.000226
57	H	0.000216	-0.000252	-0.000000
58	H	-0.000023	0.000070	0.000273
59	H	-0.000226	0.000164	0.000268
60	H	-0.000109	0.000081	-0.000228

61	H	0.000035	-0.000224	0.000005
62	H	0.000062	-0.000093	-0.000017
63	H	-0.000029	0.000001	-0.000186
64	H	0.000407	0.000064	0.000075
65	H	-0.000482	-0.000938	-0.000848
66	H	-0.000049	-0.000100	0.000363
67	H	0.000411	0.000513	0.000203
68	H	-0.000196	-0.000489	-0.000375
69	C	0.002610	0.000191	0.001154
70	H	0.000123	0.000212	-0.000176
71	H	0.000063	0.000066	0.000244
72	H	-0.000393	0.000105	-0.000139
73	H	-0.000148	-0.000049	0.000096
74	H	0.000206	0.000250	-0.000063
75	H	0.000071	0.000200	0.000367
76	H	0.000143	0.000347	0.000028
77	H	-0.000044	0.000138	0.000021
78	H	0.000320	0.000147	0.000276
79	H	0.000138	-0.000005	0.000331
80	H	-0.000104	-0.000088	0.000075
81	H	0.000270	0.000078	0.000248
82	H	-0.000556	-0.000929	-0.000085
83	H	-0.000884	-0.000113	-0.000378
84	H	-0.000175	-0.000253	-0.000243
85	H	-0.000295	-0.000029	0.000149
86	H	0.000121	0.000111	-0.000392
87	O	-0.000560	0.001096	-0.000046
88	H	0.000048	-0.000458	0.000268
89	H	0.000626	-0.000044	0.000137
90	H	-0.000122	0.000091	0.000190
91	H	-0.000346	0.000015	0.000136

92 C -0.000437 -0.000215 -0.000033

93 O -0.001088 -0.000274 -0.000598

94 H 0.000186 -0.000083 -0.000360

95 H 0.000044 0.000113 0.000035

96 H -0.000192 -0.000108 -0.000264

----

dE(predicted): -0.31884E-03, dE(actual): -0.28093E-03, Trust radius: 0.195959, all in a.u.

Max Cartesian displacement 0.948039517142561

Limit Cartesian step to 0.566917837497000

Geometry Convergence after Step 11 (Hartree/Angstrom,Angstrom)

----

current energy -19.67472939 Hartree

energy change -0.00028093 0.00100000 T

constrained gradient max 0.00360484 0.00100000 F

constrained gradient rms 0.00063192 0.00066667 T

gradient max 0.00360484

gradient rms 0.00063192

cart. step max 0.30532292 0.01000000 F

cart. step rms 0.07763886 0.00666667 F

Coordinates (Cartesian)

======================

Atom bohr angstrom Geometric Variables

X Y Z X Y Z (0:frozen, *:LT par.)

----

1 O	26.515175	61.643377	50.475114	14.031227	32.620270	26.710280	1	2	3
2 C	27.012946	61.959756	53.048259	14.294635	32.787691	28.071930	4	5	6
3 C	29.809953	61.227329	53.396587	15.774748	32.400107	28.256257	7	8	9
4 C	30.632662	61.556872	56.180990	16.210107	32.574494	29.729700	10	11	12
5 O	27.301155	52.922478	43.192070	14.447149	28.005369	22.856259	13	14	15
6 N	33.279013	60.932226	56.806140	17.610495	32.243946	30.060515	16	17	18
7 O	26.635382	64.556155	53.750660	14.094837	34.161646	28.443624	19	20	21
8 C	20.407264	58.500313	48.361467	10.799059	30.957032	25.591786	22	23	24
9 C	25.734812	65.396093	41.504527	13.618276	34.606122	21.963250	25	26	27
10 C	21.635175	55.067931	45.032846	11.448842	29.140694	23.830356	28	29	30
11 O	21.672471	62.759687	45.696061	11.468578	33.210996	24.181314	31	32	33
12 C	19.516651	56.593793	46.263245	10.327767	29.948145	24.481455	34	35	36
13 O	21.616025	55.210766	42.492370	11.438708	29.216279	22.485994	37	38	39
14 C	23.933501	61.566145	49.558763	12.665064	32.579401	26.225368	40	41	42
15 C	23.969545	63.287449	47.137705	12.684137	33.490275	24.944199	43	44	45
16 O	18.771948	59.556566	49.604145	9.933687	31.515977	26.249383	46	47	48
17 C	23.725264	57.005655	51.286671	12.554869	30.166093	27.139737	49	50	51
18 C	27.502075	53.177479	38.701582	14.553472	28.140310	20.479995	52	53	54
19 O	30.054091	58.589281	52.611985	15.903940	31.004112	27.841064	55	56	57
20 C	26.405346	57.072504	41.166336	13.973107	30.201468	21.784287	58	59	60
21 C	26.133028	54.191578	41.081058	13.829003	28.676948	21.739160	61	62	63
22 C	23.207529	58.777966	49.003205	12.280895	31.103960	25.931380	64	65	66
23 C	25.962296	62.691982	42.531037	13.738656	33.175168	22.506455	67	68	69
24 C	35.209619	62.004126	55.119869	18.632128	32.811170	29.168179	70	71	72
25 C	26.263248	62.485302	45.448255	13.897913	33.065798	24.050181	73	74	75
26 C	29.975233	64.299242	56.975286	15.862210	34.025694	30.150023	76	77	78
27 C	27.199574	64.982596	56.414204	14.393395	34.387309	29.853111	79	80	81
28 C	26.565152	67.742792	56.957598	14.057673	35.847942	30.140663	82	83	84
29 C	28.437579	58.460999	41.807699	15.048519	30.936229	22.123682	85	86	87
30 C	17.661663	57.866533	44.409372	9.346149	30.621651	23.500428	88	89	90
31 O	30.080475	62.459089	40.546272	15.917902	33.051927	21.456163	91	92	93

32 C	23.245384	53.530549	40.880641	12.300928	28.327146	21.633104	94	95	96
33 C	30.979961	57.509219	42.703294	16.393889	30.432568	22.597610	97	98	99
34 C	28.296292	61.311036	41.478917	14.973753	32.444403	21.949698	100	101	102
35 C	24.214094	66.072241	47.956968	12.813547	34.963924	25.377735	103	104	105
36 C	22.568555	50.741995	41.284622	11.942765	26.851507	21.846881	106	107	108
37 C	33.758077	58.245190	57.326736	17.864005	30.822027	30.336002	109	110	111
38 C	19.431273	64.170157	46.325619	10.282587	33.957385	24.514462	112	113	114
39 C	19.725662	50.184235	41.032471	10.438371	26.556354	21.713448	115	116	117
40 H	24.748261	58.082593	40.480373	13.096216	30.735984	21.421291	118	119	120
41 H	31.385816	58.262496	44.600010	16.608659	30.831185	23.601309	121	122	123
42 H	31.068106	55.453115	42.775941	16.440534	29.344525	22.636053	124	125	126
43 H	32.459921	58.254939	41.455987	17.177050	30.827186	21.937564	127	128	129
44 H	27.299643	66.580176	42.167571	14.446349	35.232712	22.314118	130	131	132
45 H	25.790839	65.399205	39.432953	13.647924	34.607769	20.867020	133	134	135
46 H	23.945546	66.242424	42.108092	12.671437	35.053981	22.282643	136	137	138
47 H	24.260112	61.624988	42.024634	12.837898	32.610539	22.238478	139	140	141
48 H	27.916618	63.580836	46.038301	14.772838	33.645529	24.362420	142	143	144
49 H	26.737001	60.520153	45.874925	14.148612	32.025885	24.275965	145	146	147
50 H	25.981783	66.304969	48.999203	13.748968	35.087078	25.929261	148	149	150

51 H	22.683356	66.641595	49.228151	12.003515	35.265213	26.050416	151	152	153
52 H	24.236978	67.330582	46.318355	12.825656	35.629810	24.510618	154	155	156
53 H	19.654673	66.187781	45.895172	10.400805	35.025065	24.286679	157	158	159
54 H	18.882282	63.926326	48.312875	9.992073	33.828355	25.566072	160	161	162
55 H	17.931626	63.392376	45.136174	9.489008	33.545801	23.885035	163	164	165
56 H	22.602119	54.131761	39.004686	11.960526	28.645294	20.640391	166	167	168
57 H	23.619799	49.656392	39.865132	12.499059	26.277031	21.095719	169	170	171
58 H	23.264094	50.117620	43.127018	12.310828	26.521103	22.821835	172	173	174
59 H	18.628911	51.159539	42.495733	9.857995	27.072462	22.487774	175	176	177
60 H	18.988858	50.787254	39.187806	10.048471	26.875457	20.737294	178	179	180
61 H	19.370742	48.152380	41.228151	10.250555	25.481142	21.816998	181	182	183
62 H	27.316342	51.120724	38.562875	14.455186	27.051922	20.406595	184	185	186
63 H	26.757075	54.056065	36.980792	14.159235	28.605238	19.569392	187	188	189
64 H	29.511097	53.640455	38.849437	15.616600	28.385306	20.558237	190	191	192
65 H	24.347804	58.113710	47.414316	12.884303	30.752451	25.090576	193	194	195
66 H	22.579040	57.575404	52.917167	11.948313	30.467592	28.002559	196	197	198
67 H	25.719756	57.073660	51.809159	13.610309	30.202080	27.416226	199	200	201
68 H	23.251282	55.056969	50.787508	12.304049	29.134893	26.875592	202	203	204
69 C	30.934898	58.136976	50.192379	16.370043	30.764763	26.560663	205	206	207

70 H	27.740560	68.992144	55.794052	14.679672	36.509070	29.524941	208	209 210
71 H	26.907146	68.182091	58.954375	14.238649	36.080409	31.197312	211	212 213
72 H	24.575910	68.125063	56.526393	13.005012	36.050231	29.912479	214	215 216
73 H	25.953618	63.746130	57.551711	13.734063	33.732999	30.455054	217	218 219
74 H	31.191315	65.644041	55.965003	16.505733	34.737331	29.615404	220	221 222
75 H	30.361958	64.506443	59.001492	16.066856	34.135340	31.222245	223	224 225
76 H	35.296582	61.077777	53.246065	18.678147	32.320968	28.176604	226	227 228
77 H	34.872524	64.023642	54.819603	18.453745	33.879852	29.009284	229	230 231
78 H	37.067791	61.807453	56.017990	19.615430	32.707096	29.643444	232	233 234
79 H	33.745754	57.040005	55.619825	17.857484	30.184271	29.432744	235	236 237
80 H	32.327509	57.519104	58.637296	17.106981	30.437799	31.029521	238	239 240
81 H	35.612415	58.058865	58.239264	18.845279	30.723428	30.818891	241	242 243
82 H	25.780298	60.749533	54.227849	13.642346	32.147269	28.696142	244	245 246
83 H	22.628346	62.353924	50.964246	11.974405	32.996276	26.969118	247	248 249
84 H	30.927024	62.413129	52.124734	16.365876	33.027606	27.583221	250	251 252
85 H	29.459450	60.275190	57.322098	15.589270	31.896257	30.333548	253	254 255
86 H	26.165175	53.156849	44.642628	13.846014	28.129393	23.623862	256	257 258
87 O	23.125989	53.793697	46.279919	12.237746	28.466399	24.490279	259	260 261
88 H	18.483369	55.168879	47.376808	9.780978	29.194113	25.070727	262	263 264

89 H 32.054633 54.809634 48.177579 16.962581 29.004009 25.494477 265 266
267

90 H 30.528456 54.166704 51.156531 16.154963 28.663785 27.070870 268 269
270

91 H 28.744824 55.218937 48.511511 15.211106 29.220603 25.671186 271 272
273

92 C 30.567826 55.388635 49.489948 16.175797 29.310404 26.188953 274 275
276

93 O 31.815014 59.738768 48.803932 16.835780 31.612395 25.825929 277 278
279

94 H 18.659745 59.228255 43.216658 9.874312 31.342243 22.869271 280 281
282

95 H 16.197967 58.851578 45.485958 8.571595 31.142914 24.070133 283 284
285

96 H 16.769442 56.457022 43.185695 8.874007 29.875769 22.852885 286 287
288

Number of elements of the density matrix on this node (used, total): 222269 1773786

Numerical Integration : Fuzzy Cells (Becke)

Becke grid quality: NORMAL

Lebedev angular grid order: min: 11 max: 23

Nr. of radial points: min: 46 max: 52

Total nr. of points: 600097

Nr. of blocks: 4689

Block length: 128

Nr. of dummy points: 95

Test of Precision of the Numerical Integration Grid

==

Integral of the Total Core Density: 85.99995046540747

Relative Error: -5.760E-07

Density Fitting (zlmFit)

ZlmFit Fit Quality: NORMAL

Max L-expansion: min: 6 max: 7

Nr. of radial interpolation points: min: 39 max: 48

S C F *** ScaSCF ***

Initial density matrix is taken from previous geometry

CYCLE 1

SCF Error: norm([F,P])= 0.231987905965, max([F,P])= 0.016982686873

orbitals (Q,E):

A :114...133 (2.00 -0.2557) (2.00 -0.2547) (2.00 -0.2455) (2.00 -0.2406)

(2.00 -0.2348) (2.00 -0.2277) (2.00 -0.2256) (2.00 -0.2213)

(2.00 -0.1997) (2.00 -0.1839) (0.00 -0.0755) (0.00 -0.0738)

(0.00 -0.0705) (0.00 -0.0478) (0.00 -0.0233) (0.00 -0.0184)

(0.00 -0.0164) (0.00 -0.0127) (0.00 -0.0101) (0.00 -0.0051)

CYCLE 2

SDIIS (wt 0.000): 0.0581 0.9419

A-DIIS (wt 1.000): 0.0000 1.0000

DIIS coefficients: 0.0000 1.0000

SCF Error: norm([F,P])= 0.050733923686, max([F,P])= 0.004710797171

orbitals (Q,E):

A :114...124 (2.00 -0.2438) (2.00 -0.2437) (2.00 -0.2365) (2.00 -0.2317)

(2.00 -0.2232) (2.00 -0.2187) (2.00 -0.2156) (2.00 -0.2122)

(2.00 -0.1947) (2.00 -0.1758) (0.00 -0.0663)

CYCLE 3

SDIIS (wt 0.000): 0.0070 0.6906 0.3024

A-DIIS (wt 1.000): 0.0000 0.7603 0.2397

DIIS coefficients: 0.0000 0.7603 0.2397

SCF Error: norm([F,P])= 0.109544639197, max([F,P])= 0.013160166320

orbitals (Q,E):

A :114...124 (2.00 -0.2466) (2.00 -0.2460) (2.00 -0.2365) (2.00 -0.2329)

(2.00 -0.2254) (2.00 -0.2201) (2.00 -0.2176) (2.00 -0.2134)

(2.00 -0.1930) (2.00 -0.1767) (0.00 -0.0677)

CYCLE 4

SDIIS (wt 0.983): 0.4160 0.1290 0.4550

A-DIIS (wt 0.017): 0.1513 0.0000 0.8487

DIIS coefficients: 0.4115 0.1268 0.4617

SCF Error: norm([F,P])= 0.024567983173, max([F,P])= 0.002671125385

orbitals (Q,E):

A :114...124 (2.00 -0.2481) (2.00 -0.2474) (2.00 -0.2383) (2.00 -0.2339)

(2.00 -0.2271) (2.00 -0.2210) (2.00 -0.2186) (2.00 -0.2146)

(2.00 -0.1942) (2.00 -0.1774) (0.00 -0.0688)

CYCLE 5

SDIIS (wt 1.000): 0.1702 0.0392 0.2834 0.5072

DIIS coefficients: 0.1702 0.0392 0.2834 0.5072

SCF Error: norm([F,P])= 0.007508974486, max([F,P])= 0.000926114854

orbitals (Q,E):

A :114...124 (2.00 -0.2479) (2.00 -0.2473) (2.00 -0.2381) (2.00 -0.2339)

(2.00 -0.2270) (2.00 -0.2210) (2.00 -0.2186) (2.00 -0.2145)

(2.00 -0.1939) (2.00 -0.1775) (0.00 -0.0688)

CYCLE 6

SDIIS (wt 1.000): 0.0695 0.0132 0.1339 0.3293 0.4541

DIIS coefficients: 0.0695 0.0132 0.1339 0.3293 0.4541

SCF Error: norm([F,P])= 0.002719767946, max([F,P])= 0.000258553210

orbitals (Q,E):

A :114...124 (2.00 -0.2479) (2.00 -0.2473) (2.00 -0.2381) (2.00 -0.2339)

(2.00 -0.2269) (2.00 -0.2210) (2.00 -0.2186) (2.00 -0.2145)

(2.00 -0.1939) (2.00 -0.1775) (0.00 -0.0687)

CYCLE 7

SDIIS (wt 1.000): 0.0233 0.0036 0.0506 0.1546 0.3105 0.4574

DIIS coefficients: 0.0233 0.0036 0.0506 0.1546 0.3105 0.4574

SCF Error: norm([F,P])=   0.001041819750, max([F,P])=   0.000116712506

orbitals (Q,E):

A :114...124   (2.00   -0.2479) (2.00   -0.2473) (2.00   -0.2381) (2.00   -0.2339)

(2.00   -0.2270) (2.00   -0.2210) (2.00   -0.2186) (2.00   -0.2145)

(2.00   -0.1939) (2.00   -0.1775) (0.00   -0.0687)

CYCLE   8

SDIIS (wt 1.000): 0.0071 0.0008 0.0179 0.0668 0.1658 0.3391 0.4026

DIIS coefficients: 0.0071 0.0008 0.0179 0.0668 0.1658 0.3391 0.4026

SCF Error: norm([F,P])=   0.000473192181, max([F,P])=   0.000074419762

orbitals (Q,E):

A :114...124   (2.00   -0.2479) (2.00   -0.2473) (2.00   -0.2381) (2.00   -0.2339)

(2.00   -0.2270) (2.00   -0.2210) (2.00   -0.2186) (2.00   -0.2145)

(2.00   -0.1940) (2.00   -0.1775) (0.00   -0.0687)

CYCLE   9

SDIIS (wt 1.000): 0.0016 0.0000 0.0054 0.0251 0.0738 0.1769 0.2887 0.4285

DIIS coefficients: 0.0016 0.0000 0.0054 0.0251 0.0738 0.1769 0.2887 0.4285

SCF Error: norm([F,P])=   0.000166019287, max([F,P])=   0.000019752843

orbitals (Q,E):

A :114...124   (2.00   -0.2479) (2.00   -0.2473) (2.00   -0.2381) (2.00   -0.2339)

(2.00   -0.2269) (2.00   -0.2210) (2.00   -0.2186) (2.00   -0.2145)

(2.00   -0.1940) (2.00   -0.1775) (0.00   -0.0687)

CYCLE   10

SDIIS (wt 1.000): 0.0001 -0.0001 0.0010 0.0068 0.0244 0.0678 0.1345 0.2983 0.4671

DIIS coefficients: 0.0001 -0.0001 0.0010 0.0068 0.0244 0.0678 0.1345 0.2983 0.4671

SCF Error: norm([F,P])=   0.000058929261, max([F,P])=   0.000008070713

orbitals (Q,E):

A :114...124   (2.00   -0.2479) (2.00   -0.2473) (2.00   -0.2381) (2.00   -0.2339)

(2.00   -0.2270) (2.00   -0.2210) (2.00   -0.2186) (2.00   -0.2145)

(2.00   -0.1940) (2.00   -0.1775) (0.00   -0.0687)

CYCLE   11

SDIIS (wt 1.000): -0.0001 -0.0001 0.0001 0.0018 0.0087 0.0278 0.0632 0.1649 0.3418 0.3920

DIIS coefficients: -0.0001 -0.0001 0.0001 0.0018 0.0087 0.0278 0.0632 0.1649 0.3418 0.3920

SCF Error: norm([F,P])=   0.000025691259, max([F,P])=   0.000004731615

orbitals (Q,E):

A :114...124   (2.00   -0.2479) (2.00   -0.2473) (2.00   -0.2381) (2.00   -0.2339)

(2.00   -0.2269) (2.00   -0.2210) (2.00   -0.2186) (2.00   -0.2145)

(2.00   -0.1940) (2.00   -0.1775) (0.00   -0.0687)

CYCLE   12

SDIIS (wt 1.000): -0.0000 0.0001 0.0008 0.0034 0.0109 0.0257 0.0707 0.1670 0.2718 0.4495

DIIS coefficients: -0.0000 0.0001 0.0008 0.0034 0.0109 0.0257 0.0707 0.1670 0.2718 0.4495

SCF Error: norm([F,P])=   0.000008582259, max([F,P])=   0.000001361626

orbitals (Q,E):

A :114...124   (2.00   -0.2479) (2.00   -0.2473) (2.00   -0.2381) (2.00   -0.2339)

(2.00   -0.2269) (2.00   -0.2210) (2.00   -0.2186) (2.00   -0.2145)

(2.00   -0.1940) (2.00   -0.1775) (0.00   -0.0687)

CYCLE   13

SDIIS (wt 1.000): 0.0000 0.0001 0.0008 0.0029 0.0084 0.0256 0.0691 0.1346 0.2999 0.4586

DIIS coefficients: 0.0000 0.0001 0.0008 0.0029 0.0084 0.0256 0.0691 0.1346 0.2999 0.4586

SCF Error: norm([F,P])=   0.000002855247, max([F,P])=   0.000000362553

orbitals (Q,E):

```
A :114...124       ( 2.00    -0.2479)  ( 2.00    -0.2473)  ( 2.00    -0.2381)  ( 2.00    -0.2339)
                   ( 2.00    -0.2269)  ( 2.00    -0.2210)  ( 2.00    -0.2186)  ( 2.00    -0.2145)
                   ( 2.00    -0.1940)  ( 2.00    -0.1775)  ( 0.00    -0.0687)
```

SCF CONVERGED

CYCLE   14

*** Setting up for NEW gradients in Focky

*** Using FIT density in Focky

*** Setting up for NEW Hessian in Focky

orbitals (Q,E):

```
A :114...133       ( 2.00    -0.2479)  ( 2.00    -0.2473)  ( 2.00    -0.2381)  ( 2.00    -0.2339)
                   ( 2.00    -0.2269)  ( 2.00    -0.2210)  ( 2.00    -0.2186)  ( 2.00    -0.2145)
                   ( 2.00    -0.1940)  ( 2.00    -0.1775)  ( 0.00    -0.0687)  ( 0.00    -0.0674)
                   ( 0.00    -0.0626)  ( 0.00    -0.0398)  ( 0.00    -0.0181)  ( 0.00    -0.0128)
                   ( 0.00    -0.0102)  ( 0.00    -0.0071)  ( 0.00    -0.0047)  ( 0.00     0.0002)
```

Fit test: (difference of exact and fit density, squared integrated, result summed over spins)

SCF: 0.00100833463775

Gross Charges per Atom (Z minus electrons)

==

```
-0.6654   0.9193   0.5475   0.3305  -0.5803  -0.3803  -0.6306   0.6733   0.6225   0.8655
-0.6588   0.0005  -0.5766   0.5857   0.3236  -0.5916   0.5939   0.6003  -0.6021   0.2566
 0.3372   0.0970   0.1664   0.7456   0.4173   0.4093   0.5588   0.5712   0.0139   0.6399
-0.5653   0.5486   0.5449   0.5714   0.6418   0.3722   0.7487   0.9826   0.5860  -0.2136
-0.1252  -0.2172  -0.2055  -0.2132  -0.2430  -0.2320  -0.1525  -0.1869  -0.1236  -0.2083
```

-0.2404	-0.2225	-0.2263	-0.1823	-0.2447	-0.1710	-0.1954	-0.1542	-0.1975	-0.2141
-0.2237	-0.2198	-0.2279	-0.1933	-0.1123	-0.2246	-0.1570	-0.2154	0.8280	-0.1963
-0.2183	-0.2168	-0.2098	-0.1902	-0.2119	-0.1861	-0.2245	-0.2313	-0.1982	-0.2399
-0.2113	-0.2508	-0.2009	-0.1596	-0.1668	0.2315	-0.6758	-0.1258	-0.1948	-0.2128
-0.0739	0.5090	-0.6218	-0.1743	-0.2169	-0.2377				

Net Total: 0.00000000

M U L T I P O L E D E R I V E D C H A R G E A N A L Y S I S

See: M. Swart, P.Th. van Duijnen, J.G. Snijders, J.Comput.Chem., (2001), p. 79-88.

Multipole derived atomic charges (a.u.)

Atom	Level:	MDC-m	MDC-d	MDC-q
1	O	-0.350422	-0.312759	-0.337233
2	C	0.362628	0.373880	0.395262
3	C	0.242264	0.248787	0.252804
4	C	0.180889	0.165909	0.211233
5	O	-0.307099	-0.550194	-0.610284
6	N	-0.214246	-0.275581	-0.465317
7	O	-0.380517	-0.384215	-0.449586
8	C	0.250479	0.309691	0.308743
9	C	0.227838	0.192769	0.189098
10	C	0.339601	0.408657	0.413912

11	O	-0.383434	-0.357921	-0.400310
12	C	0.124193	0.102399	0.102533
13	O	-0.313037	-0.300399	-0.344472
14	C	0.245291	0.242021	0.247471
15	C	0.196152	0.158924	0.175142
16	O	-0.369635	-0.478178	-0.483335
17	C	0.226848	0.192220	0.198468
18	C	0.229679	0.193480	0.196414
19	O	-0.319041	-0.322620	-0.371678
20	C	0.056283	0.043508	-0.053117
21	C	0.194998	0.221615	0.283046
22	C	0.118407	0.104994	0.107437
23	C	0.119859	0.099646	0.111839
24	C	0.290919	0.268745	0.286348
25	C	0.172917	0.134093	0.135886
26	C	0.164798	0.124677	0.164427
27	C	0.249144	0.238621	0.261126
28	C	0.234419	0.193637	0.304677
29	C	0.034744	0.027694	-0.042831
30	C	0.236871	0.190772	0.176141
31	O	-0.372020	-0.476243	-0.514017
32	C	0.254454	0.243498	0.263363
33	C	0.220209	0.183370	0.188452
34	C	0.228663	0.296024	0.378952
35	C	0.222123	0.191964	0.201382
36	C	0.180337	0.145501	0.149752
37	C	0.294004	0.270936	0.309503
38	C	0.367806	0.339517	0.387663
39	C	0.229357	0.189999	0.157228
40	H	-0.073733	-0.060322	-0.029303
41	H	-0.057111	-0.030362	-0.017061

42	H	-0.065487	-0.058506	-0.033824
43	H	-0.057316	-0.036826	-0.016097
44	H	-0.069005	-0.052896	-0.051796
45	H	-0.070556	-0.057584	-0.053985
46	H	-0.076635	-0.072360	-0.070678
47	H	-0.072039	-0.062460	-0.062213
48	H	-0.063023	-0.039946	-0.021230
49	H	-0.082225	-0.060152	-0.067774
50	H	-0.055455	-0.047191	-0.049748
51	H	-0.072590	-0.055227	-0.060413
52	H	-0.070487	-0.058639	-0.064873
53	H	-0.067982	-0.064346	-0.073582
54	H	-0.057853	-0.044540	-0.052049
55	H	-0.054181	-0.043752	-0.045666
56	H	-0.057111	-0.042283	-0.030645
57	H	-0.067089	-0.050178	-0.051352
58	H	-0.061932	-0.036453	-0.032023
59	H	-0.075292	-0.060512	-0.050221
60	H	-0.074770	-0.065443	-0.056184
61	H	-0.068928	-0.055037	-0.043407
62	H	-0.067135	-0.048836	-0.040993
63	H	-0.072597	-0.075020	-0.083717
64	H	-0.058118	-0.035799	-0.034273
65	H	-0.074934	-0.048616	-0.057982
66	H	-0.072783	-0.064781	-0.061673
67	H	-0.068111	-0.054111	-0.067786
68	H	-0.064882	-0.056998	-0.056651
69	C	0.353477	0.435930	0.453189
70	H	-0.064602	-0.054131	-0.082475
71	H	-0.068096	-0.067240	-0.098051
72	H	-0.063091	-0.048193	-0.074530

73	H	-0.080693	-0.062496	-0.072284
74	H	-0.072940	-0.060014	-0.063637
75	H	-0.069429	-0.068627	-0.073503
76	H	-0.077512	-0.049210	-0.044111
77	H	-0.066305	-0.053985	-0.039162
78	H	-0.064705	-0.059906	-0.029071
79	H	-0.082695	-0.064050	-0.046958
80	H	-0.066521	-0.057543	-0.051598
81	H	-0.067638	-0.066330	-0.056058
82	H	-0.070698	-0.044836	-0.037743
83	H	-0.061662	-0.026609	-0.019042
84	H	-0.047170	-0.004191	-0.015652
85	H	-0.073550	-0.059822	-0.041756
86	H	0.061664	0.261696	0.290899
87	O	-0.363683	-0.439088	-0.454220
88	H	-0.044764	0.000652	0.021087
89	H	-0.044863	-0.029516	-0.016659
90	H	-0.052148	-0.050608	-0.038552
91	H	-0.042021	-0.009332	-0.009229
92	C	0.240720	0.197659	0.181350
93	O	-0.358103	-0.454234	-0.481842
94	H	-0.060970	-0.053377	-0.049509
95	H	-0.061015	-0.047686	-0.038897
96	H	-0.068352	-0.065173	-0.060905

*** Using NEW gradient routines ***

Geometry CYCLE 12

=============

Energy gradients wrt nuclear displacements

===

Atom Cartesian (a.u./angstrom)

X Y Z

1	O	0.001674	-0.004949	-0.001745
2	C	0.001048	-0.000824	0.000971
3	C	-0.001136	0.000383	-0.000203
4	C	0.001013	0.001194	-0.000490
5	O	-0.000401	-0.001669	-0.001141
6	N	-0.000165	0.000587	0.001199
7	O	0.000076	0.001619	0.000578
8	C	-0.000307	0.000727	-0.000068
9	C	-0.000273	0.000252	-0.000712
10	C	0.002820	0.003442	0.001547
11	O	-0.002341	-0.001922	0.002444
12	C	-0.000018	-0.000067	0.000917
13	O	-0.000127	-0.000073	-0.002567
14	C	-0.001354	0.002731	-0.001419
15	C	0.001661	0.000625	-0.000002
16	O	-0.000252	0.001677	-0.000395
17	C	0.001459	0.000677	-0.001281
18	C	0.000207	-0.000014	0.000356
19	O	-0.007090	0.000861	-0.001124
20	C	0.000262	-0.000765	0.000471
21	C	-0.001051	-0.000314	0.001348
22	C	0.001435	-0.000703	-0.001013
23	C	0.001038	-0.000661	-0.001751
24	C	0.000788	-0.000865	0.000433
25	C	0.000241	-0.000490	-0.000082
26	C	0.001283	-0.001179	0.000268

27 C	-0.000655	0.000391	0.000151
28 C	0.000254	-0.000518	-0.000754
29 C	-0.001862	0.001469	-0.000044
30 C	-0.000411	-0.000513	-0.000091
31 O	0.000526	0.000229	-0.000352
32 C	-0.000477	0.000277	0.000607
33 C	0.000274	-0.001103	-0.001495
34 C	-0.000122	-0.000014	0.002919
35 C	0.000531	0.000620	0.000165
36 C	-0.000106	0.000217	-0.000929
37 C	0.000513	-0.000390	0.000011
38 C	0.000741	-0.001662	0.000430
39 C	0.000691	-0.000055	0.000335
40 H	-0.000072	0.000378	-0.001021
41 H	0.000909	0.000797	0.004264
42 H	0.000303	0.000260	0.000000
43 H	0.001116	0.000705	0.000321
44 H	0.000425	0.000480	0.000183
45 H	-0.000138	0.000217	-0.000720
46 H	-0.000124	0.000096	-0.000543
47 H	0.000013	0.000140	0.000457
48 H	0.000578	0.000444	0.000072
49 H	-0.000322	-0.001863	0.000215
50 H	0.000073	0.000763	0.000278
51 H	-0.000077	0.000101	0.000873
52 H	-0.000063	0.000104	0.000380
53 H	0.000046	0.000833	0.000158
54 H	-0.000271	-0.000550	0.001345
55 H	-0.000712	-0.000327	-0.000601
56 H	-0.000364	0.000005	-0.000672
57 H	0.000390	-0.000494	-0.000024

58 H 0.000023 -0.000135 0.000508

59 H -0.000397 0.000277 0.000510

60 H -0.000332 0.000115 -0.000625

61 H -0.000048 -0.000561 0.000018

62 H 0.000120 -0.000417 -0.000101

63 H -0.000248 0.000128 -0.000451

64 H 0.000737 0.000126 0.000263

65 H -0.000435 -0.001273 -0.001261

66 H -0.000191 -0.000097 0.000817

67 H 0.001137 0.000911 0.000546

68 H -0.000054 -0.000924 -0.000478

69 C 0.003338 0.000007 0.000222

70 H 0.000286 0.000519 -0.000360

71 H 0.000176 0.000181 0.000816

72 H -0.000801 0.000170 -0.000226

73 H -0.000282 -0.000147 0.000334

74 H 0.000478 0.000482 -0.000214

75 H 0.000063 0.000210 0.000700

76 H 0.000553 0.000320 -0.000286

77 H 0.000076 0.000523 -0.000100

78 H 0.000652 0.000137 0.000320

79 H 0.000228 -0.000122 0.000435

80 H -0.000191 -0.000254 0.000251

81 H 0.000623 0.000082 0.000556

82 H -0.000983 -0.001723 -0.000193

83 H -0.001624 -0.000446 -0.000446

84 H -0.000267 -0.000339 -0.000644

85 H -0.000511 -0.000131 0.000379

86 H -0.000258 0.000375 0.000025

87 O -0.001348 0.000717 0.002214

88 H 0.000155 -0.000753 0.000586

```
  89 H     0.001672  0.000024 -0.000582
  90 H    -0.000049 -0.000318  0.000706
  91 H    -0.001531 -0.000012  0.000078
  92 C    -0.000836  0.000503 -0.000314
  93 O    -0.001849  0.000329 -0.005234
  94 H     0.000395  0.000158 -0.000750
  95 H    -0.000199  0.000439  0.000204
  96 H    -0.000378 -0.000396 -0.000680
 ----------------------------------------

 dE(predicted):    -0.50781E-03, dE(actual):     0.45500E-03, Trust radius:    0.195959, all in a.u.
 Interpolation step length   9.298249096585232E-002

----------------------------------------------------------------------
Geometry Convergence after Step  12       (Hartree/Angstrom,Angstrom)
----------------------------------------------------------------------
current energy                          -19.67427438 Hartree
energy change                       0.00045500     0.00100000     T
constrained gradient max            0.00708950     0.00100000     F
constrained gradient rms            0.00106852     0.00066667     F
gradient max                        0.00708950
gradient rms                        0.00106852
cart. step max                      0.28689321     0.01000000     F
cart. step rms                      0.07802924     0.00666667     F


 Coordinates (Cartesian)
 ======================

   Atom                 bohr                      angstrom               Geometric Variables
```

	X	Y	Z	X	Y	Z	(0:frozen, *:LT par.)		
1 O	26.665079	61.661527	50.436883	14.110552	32.629875	26.690049	1	2	3
2 C	27.096505	61.978621	53.026235	14.338853	32.797674	28.060275	4	5	6
3 C	29.872539	61.244754	53.536804	15.807867	32.409328	28.330457	7	8	9
4 C	30.533390	61.604018	56.359552	16.157574	32.599443	29.824191	10	11	12
5 O	27.260989	52.906438	42.919263	14.425894	27.996881	22.711896	13	14	15
6 N	33.138387	60.982543	57.137121	17.536079	32.270572	30.235663	16	17	18
7 O	26.693125	64.585659	53.672035	14.125394	34.177259	28.402018	19	20	21
8 C	20.531389	58.472627	48.412722	10.864743	30.942382	25.618909	22	23	24
9 C	25.598977	65.388877	41.464719	13.546396	34.602304	21.942184	25	26	27
10 C	21.667133	55.029788	45.035934	11.465753	29.120510	23.831990	28	29	30
11 O	21.743128	62.716253	45.702504	11.505968	33.188012	24.184723	31	32	33
12 C	19.595677	56.565547	46.333981	10.369586	29.933198	24.518887	34	35	36
13 O	21.553339	55.202028	42.500419	11.405536	29.211655	22.490253	37	38	39
14 C	24.071811	61.559084	49.546921	12.738254	32.575664	26.219102	40	41	42
15 C	24.059537	63.258944	47.106416	12.731759	33.475192	24.927642	43	44	45
16 O	18.915004	59.532702	49.675790	10.009389	31.503349	26.287296	46	47	48
17 C	23.869653	56.998756	51.305509	12.631276	30.162443	27.149706	49	50	51
18 C	27.253104	53.223438	38.424035	14.421722	28.164630	20.333124	52	53	54
19 O	30.183290	58.595706	52.814155	15.972309	31.007512	27.948047	55	56	57
20 C	26.270944	57.082564	40.990430	13.901985	30.206792	21.691201	58	59	60
21 C	25.998225	54.202709	40.880099	13.757668	28.682838	21.632817	61	62	63
22 C	23.337968	58.765122	49.020729	12.349921	31.097163	25.940653	64	65	66
23 C	25.885609	62.677488	42.455451	13.698074	33.167498	22.466457	67	68	69
24 C	35.160088	62.030383	55.545270	18.605917	32.825065	29.393291	70	71	72
25 C	26.314590	62.469329	45.359251	13.925082	33.057346	24.003082	73	74	75
26 C	29.848762	64.359388	57.079278	15.795285	34.057521	30.205053	76	77	78
27 C	27.115310	65.048721	56.354886	14.348804	34.422301	29.821721	79	80	81
28 C	26.466542	67.818627	56.826941	14.005491	35.888072	30.071522	82	83	84
29 C	28.334343	58.466792	41.535617	14.993888	30.939294	21.979702	85	86	87

30	C	17.710749	57.850491	44.519219	9.372125	30.613161	23.558556	88	89	90
31	O	29.879963	62.488020	40.226874	15.811796	33.067236	21.287145	91	92	93
32	C	23.107094	53.538622	40.803112	12.227748	28.331418	21.592077	94	95	96
33	C	30.927884	57.508598	42.255219	16.366331	30.432240	22.360499	97	98	99
34	C	28.166965	61.321198	41.262741	14.905316	32.449781	21.835302	100	101	102
35	C	24.299654	66.045432	47.918110	12.858823	34.949737	25.357172	103	104	105
36	C	22.448803	50.745132	41.201432	11.879395	26.853167	21.802859	106	107	108
37	C	33.579791	58.301423	57.715027	17.769660	30.851784	30.541477	109	110	111
38	C	19.512552	64.130699	46.361654	10.325598	33.936504	24.533531	112	113	114
39	C	19.598233	50.187606	41.062042	10.370938	26.558137	21.729097	115	116	117
40	H	24.578745	58.097904	40.406295	13.006512	30.744087	21.382091	118	119	120
41	H	31.461112	58.255871	44.119494	16.648503	30.827679	23.347031	121	122	123
42	H	31.019991	55.452312	42.311314	16.415072	29.344100	22.390183	124	125	126
43	H	32.320073	58.259302	40.913838	17.103046	30.829495	21.650670	127	128	129
44	H	27.189754	66.573346	42.061353	14.388198	35.229097	22.257910	130	131	132
45	H	25.554435	65.409794	39.393174	13.522825	34.613372	20.845970	133	134	135
46	H	23.838550	66.222903	42.162122	12.614817	35.043651	22.311234	136	137	138
47	H	24.166786	61.604401	42.024034	12.788513	32.599645	22.238161	139	140	141
48	H	27.980370	63.576802	45.888296	14.806574	33.643395	24.283040	142	143	144
49	H	26.819935	60.509693	45.773733	14.192498	32.020351	24.222416	145	146	147

50	H	26.080809	66.289098	48.934633	13.801370	35.078680	25.895093	148	149	150
51	H	22.783495	66.607829	49.209405	12.056506	35.247345	26.040496	151	152	153
52	H	24.292185	67.301289	46.277678	12.854870	35.614308	24.489093	154	155	156
53	H	19.727548	66.144161	45.909200	10.439369	35.001983	24.294102	157	158	159
54	H	19.000428	63.905352	48.360253	10.054594	33.817256	25.591144	160	161	162
55	H	17.991625	63.342793	45.207309	9.520758	33.519563	23.922678	163	164	165
56	H	22.382162	54.161310	38.964604	11.844130	28.660931	20.619180	166	167	168
57	H	23.440515	49.676468	39.727369	12.404187	26.287655	21.022818	169	170	171
58	H	23.220968	50.099421	43.005377	12.288007	26.511472	22.757465	172	173	174
59	H	18.562679	51.147205	42.579019	9.822947	27.065935	22.531847	175	176	177
60	H	18.785529	50.808003	39.255632	9.940874	26.886437	20.773186	178	179	180
61	H	19.252773	48.153819	41.252215	10.188129	25.481903	21.829732	181	182	183
62	H	27.057524	51.169392	38.262641	14.318225	27.077676	20.247718	184	185	186
63	H	26.431141	54.129346	36.753528	13.986757	28.644016	19.449130	187	188	189
64	H	29.267449	53.681987	38.485262	15.487667	28.407284	20.365523	190	191	192
65	H	24.466333	58.096652	47.425355	12.947026	30.743424	25.096417	193	194	195
66	H	22.740239	57.578247	52.943949	12.033616	30.469096	28.016731	196	197	198
67	H	25.868347	57.059129	51.809720	13.688940	30.194391	27.416523	199	200	201
68	H	23.383357	55.050656	50.816918	12.373939	29.131553	26.891155	202	203	204

69 C	31.146554	58.096735	50.439842	16.482046	30.743468	26.691615	205	206	207
70 H	27.711252	69.047301	55.714986	14.664163	36.538258	29.483101	208	209	210
71 H	26.698907	68.282021	58.833647	14.128453	36.133289	31.133425	211	212	213
72 H	24.506530	68.204310	56.281170	12.968297	36.092166	29.782713	214	215	216
73 H	25.802414	63.834243	57.439240	13.654049	33.779627	30.395537	217	218	219
74 H	31.127976	65.683265	56.121105	16.472216	34.758087	29.698010	220	221	222
75 H	30.121908	64.594542	59.120693	15.939827	34.181960	31.285323	223	224	225
76 H	35.343364	61.083458	53.689208	18.702903	32.323974	28.411105	226	227	228
77 H	34.846656	64.047272	55.205978	18.440056	33.892357	29.213745	229	230	231
78 H	36.966879	61.837560	56.543221	19.562030	32.723028	29.921384	232	233	234
79 H	33.653802	57.075850	56.024378	17.808825	30.203239	29.646824	235	236	237
80 H	32.079004	57.595768	58.956190	16.975478	30.478368	31.198272	238	239	240
81 H	35.382003	58.121061	58.727230	18.723350	30.756341	31.077112	241	242	243
82 H	25.805074	60.794037	54.167641	13.655457	32.170819	28.664281	244	245	246
83 H	22.796776	62.367341	50.968017	12.063535	33.003375	26.971113	247	248	249
84 H	31.063081	62.422732	52.326040	16.437874	33.032687	27.689748	250	251	252
85 H	29.293015	60.342342	57.450400	15.501196	31.931792	30.401443	253	254	255
86 H	26.180510	53.098757	44.416926	13.854129	28.098652	23.504425	256	257	258
87 O	23.179175	53.714570	46.212303	12.265891	28.424526	24.454497	259	260	261

88 H 18.577462 55.147213 47.469291 9.830769 29.182649 25.119667 262 263
264

89 H 32.282119 54.725796 48.504413 17.082962 28.959644 25.667430 265 266
267

90 H 30.661518 54.145586 51.445018 16.225376 28.652610 27.223531 268 269
270

91 H 28.971898 55.181957 48.735293 15.331268 29.201034 25.789607 271 272
273

92 C 30.766560 55.342921 49.764136 16.280962 29.286212 26.334047 274 275
276

93 O 32.101464 59.669360 49.069803 16.987363 31.575666 25.966621 277 278
279

94 H 18.691337 59.210135 43.310340 9.891029 31.332654 22.918845 280 281
282

95 H 16.272447 58.839290 45.625620 8.611008 31.136411 24.144038 283 284
285

96 H 16.789146 56.447674 43.310243 8.884433 29.870823 22.918794 286 287
288

Number of elements of the density matrix on this node (used, total): 222269 1773786

Numerical Integration : Fuzzy Cells (Becke)

Becke grid quality: NORMAL

Lebedev angular grid order: min: 11 max: 23

Nr. of radial points: min: 46 max: 52

Total nr. of points: 600146

Nr. of blocks: 4689

Block length: 128

Nr. of dummy points: 46

Test of Precision of the Numerical Integration Grid

==

Integral of the Total Core Density: 85.99994883833736

Relative Error: -5.949E-07

Density Fitting (zlmFit)

ZlmFit Fit Quality: NORMAL

Max L-expansion: min: 6 max: 7

Nr. of radial interpolation points: min: 39 max: 48

S C F *** ScaSCF ***

Initial density matrix is taken from previous geometry

CYCLE 1

SCF Error: norm([F,P])= 0.253403357543, max([F,P])= 0.019460832641

orbitals (Q,E):

A :114...133 (2.00 -0.2574) (2.00 -0.2562) (2.00 -0.2490) (2.00 -0.2448)

(2.00 -0.2394) (2.00 -0.2303) (2.00 -0.2288) (2.00 -0.2229)

(2.00 -0.2048) (2.00 -0.1896) (0.00 -0.0786) (0.00 -0.0762)

(0.00 -0.0707) (0.00 -0.0518) (0.00 -0.0257) (0.00 -0.0214)

(0.00 -0.0202) (0.00 -0.0160) (0.00 -0.0132) (0.00 -0.0071)

CYCLE 2

SDIIS (wt 0.000): 0.0752 0.9248

A-DIIS (wt 1.000): 0.0000 1.0000

DIIS coefficients: 0.0000 1.0000

SCF Error: norm([F,P])= 0.061449202241, max([F,P])= 0.005749983936

orbitals (Q,E):

A :114...124 (2.00 -0.2455) (2.00 -0.2449) (2.00 -0.2325) (2.00 -0.2295)

(2.00 -0.2217) (2.00 -0.2205) (2.00 -0.2166) (2.00 -0.2141)

(2.00 -0.1903) (2.00 -0.1703) (0.00 -0.0685)

CYCLE 3

SDIIS (wt 0.000): 0.0056 0.6280 0.3664

A-DIIS (wt 1.000): 0.0000 0.6944 0.3056

DIIS coefficients: 0.0000 0.6944 0.3056

SCF Error: norm([F,P])= 0.102349484927, max([F,P])= 0.013873704615

orbitals (Q,E):

A :114...124 (2.00 -0.2472) (2.00 -0.2466) (2.00 -0.2351) (2.00 -0.2333)

(2.00 -0.2259) (2.00 -0.2212) (2.00 -0.2192) (2.00 -0.2144)

(2.00 -0.1939) (2.00 -0.1767) (0.00 -0.0684)

CYCLE 4

SDIIS (wt 0.981): 0.3797 0.1790 0.4412

A-DIIS (wt 0.019): 0.0000 0.0000 1.0000

DIIS coefficients: 0.3726 0.1756 0.4518

SCF Error: norm([F,P])= 0.022710336525, max([F,P])= 0.002874402172

orbitals (Q,E):

A :114...124 (2.00 -0.2481) (2.00 -0.2474) (2.00 -0.2368) (2.00 -0.2350)

(2.00 -0.2274) (2.00 -0.2218) (2.00 -0.2203) (2.00 -0.2151)

(2.00 -0.1951) (2.00 -0.1778) (0.00 -0.0689)

CYCLE 5

SDIIS (wt 0.992): 0.1601 0.0613 0.3143 0.4643

A-DIIS (wt 0.008): 0.0000 0.0000 0.1153 0.8847

DIIS coefficients: 0.1589 0.0608 0.3128 0.4674

SCF Error: norm([F,P])= 0.009501857653, max([F,P])= 0.001744655419

orbitals (Q,E):

A :114...124 (2.00 -0.2482) (2.00 -0.2474) (2.00 -0.2367) (2.00 -0.2348)

(2.00 -0.2273) (2.00 -0.2218) (2.00 -0.2202) (2.00 -0.2151)

(2.00 -0.1949) (2.00 -0.1775) (0.00 -0.0690)

CYCLE 6

SDIIS (wt 1.000): 0.0746 0.0253 0.1702 0.3198 0.4100

DIIS coefficients: 0.0746 0.0253 0.1702 0.3198 0.4100

SCF Error: norm([F,P])= 0.003199846063, max([F,P])= 0.000340913961

orbitals (Q,E):

A :114...124 (2.00 -0.2481) (2.00 -0.2473) (2.00 -0.2366) (2.00 -0.2348)

(2.00 -0.2273) (2.00 -0.2217) (2.00 -0.2202) (2.00 -0.2150)

(2.00 -0.1949) (2.00 -0.1776) (0.00 -0.0689)

CYCLE 7

SDIIS (wt 1.000): 0.0276 0.0080 0.0721 0.1592 0.2995 0.4336

DIIS coefficients: 0.0276 0.0080 0.0721 0.1592 0.2995 0.4336

SCF Error: norm([F,P])= 0.001280216260, max([F,P])= 0.000219340005

orbitals (Q,E):

A :114...124 (2.00 -0.2481) (2.00 -0.2474) (2.00 -0.2366) (2.00 -0.2348)

(2.00 -0.2273) (2.00 -0.2217) (2.00 -0.2202) (2.00 -0.2150)

(2.00 -0.1949) (2.00 -0.1777) (0.00 -0.0689)

CYCLE 8

SDIIS (wt 1.000): 0.0071 0.0014 0.0227 0.0612 0.1508 0.3321 0.4247

DIIS coefficients: 0.0071 0.0014 0.0227 0.0612 0.1508 0.3321 0.4247

SCF Error: norm([F,P])= 0.000575599178, max([F,P])= 0.000089726632

orbitals (Q,E):

A :114...124 (2.00 -0.2481) (2.00 -0.2473) (2.00 -0.2366) (2.00 -0.2348)

(2.00 -0.2273) (2.00 -0.2218) (2.00 -0.2202) (2.00 -0.2150)

(2.00 -0.1949) (2.00 -0.1776) (0.00 -0.0689)

CYCLE 9

SDIIS (wt 1.000): 0.0012 -0.0000 0.0057 0.0196 0.0594 0.1576 0.2659 0.4906

DIIS coefficients: 0.0012 -0.0000 0.0057 0.0196 0.0594 0.1576 0.2659 0.4906

SCF Error: norm([F,P])= 0.000147909030, max([F,P])= 0.000013778705

orbitals (Q,E):

A :114...124 (2.00 -0.2481) (2.00 -0.2473) (2.00 -0.2366) (2.00 -0.2348)

(2.00 -0.2273) (2.00 -0.2218) (2.00 -0.2202) (2.00 -0.2150)

(2.00 -0.1949) (2.00 -0.1776) (0.00 -0.0689)

CYCLE 10

SDIIS (wt 1.000): 0.0000 -0.0001 0.0012 0.0057 0.0210 0.0633 0.1236 0.3198 0.4656

DIIS coefficients: 0.0000 -0.0001 0.0012 0.0057 0.0210 0.0633 0.1236 0.3198 0.4656

SCF Error: norm([F,P])= 0.000055270732, max([F,P])= 0.000005835327

orbitals (Q,E):

A :114...124 (2.00 -0.2481) (2.00 -0.2473) (2.00 -0.2366) (2.00 -0.2348)

(2.00 -0.2273) (2.00 -0.2218) (2.00 -0.2202) (2.00 -0.2150)

(2.00 -0.1949) (2.00 -0.1776) (0.00 -0.0689)

CYCLE 11

SDIIS (wt 1.000): -0.0002 -0.0001 0.0001 0.0016 0.0079 0.0260 0.0568 0.1683 0.3318 0.4077

DIIS coefficients: -0.0002 -0.0001 0.0001 0.0016 0.0079 0.0260 0.0568 0.1683 0.3318 0.4077

SCF Error: norm([F,P])= 0.000024204596, max([F,P])= 0.000003406440

orbitals (Q,E):

A :114...124 (2.00 -0.2481) (2.00 -0.2473) (2.00 -0.2366) (2.00 -0.2348)

(2.00 -0.2273) (2.00 -0.2218) (2.00 -0.2202) (2.00 -0.2150)

(2.00 -0.1949) (2.00 -0.1776) (0.00 -0.0689)

CYCLE 12

SDIIS (wt 1.000): -0.0000 0.0002 0.0008 0.0034 0.0105 0.0242 0.0751 0.1741 0.3177 0.3941

DIIS coefficients: -0.0000 0.0002 0.0008 0.0034 0.0105 0.0242 0.0751 0.1741 0.3177 0.3941

SCF Error: norm([F,P])= 0.000011392737, max([F,P])= 0.000001748983

orbitals (Q,E):

A :114...124 (2.00 -0.2481) (2.00 -0.2473) (2.00 -0.2366) (2.00 -0.2348)

(2.00 -0.2273) (2.00 -0.2218) (2.00 -0.2202) (2.00 -0.2150)

(2.00 -0.1949) (2.00 -0.1776) (0.00 -0.0689)

CYCLE 13

SDIIS (wt 1.000): 0.0000 0.0001 0.0008 0.0027 0.0073 0.0251 0.0683 0.1509 0.2542 0.4906

DIIS coefficients: 0.0000 0.0001 0.0008 0.0027 0.0073 0.0251 0.0683 0.1509 0.2542 0.4906

SCF Error: norm([F,P])= 0.000002819212, max([F,P])= 0.000000270336

orbitals (Q,E):

A :114...124 (2.00 -0.2481) (2.00 -0.2473) (2.00 -0.2366) (2.00 -0.2348)

(2.00 -0.2273) (2.00 -0.2218) (2.00 -0.2202) (2.00 -0.2150)

(2.00 -0.1949) (2.00 -0.1776) (0.00 -0.0689)

SCF CONVERGED

CYCLE 14

*** Setting up for NEW gradients in Focky

*** Using FIT density in Focky

*** Setting up for NEW Hessian in Focky

orbitals (Q,E):

A :114...133 (2.00 -0.2481) (2.00 -0.2473) (2.00 -0.2366) (2.00 -0.2348)

(2.00 -0.2273) (2.00 -0.2218) (2.00 -0.2202) (2.00 -0.2150)

(2.00 -0.1949) (2.00 -0.1776) (0.00 -0.0689) (0.00 -0.0684)

(0.00 -0.0623) (0.00 -0.0391) (0.00 -0.0185) (0.00 -0.0132)

(0.00 -0.0111) (0.00 -0.0077) (0.00 -0.0050) (0.00 -0.0002)

Fit test: (difference of exact and fit density, squared integrated, result summed over spins)

SCF: 0.00100758043758

Gross Charges per Atom (Z minus electrons)

===

-0.6631 0.9253 0.5423 0.3339 -0.5789 -0.3807 -0.6316 0.6739 0.6283 0.8618

-0.6597 -0.0020 -0.5776 0.5898 0.3257 -0.5918 0.5955 0.5996 -0.6027 0.2568

0.3388	0.0957	0.1656	0.7462	0.4116	0.4076	0.5603	0.5723	0.0254	0.6435
-0.5657	0.5502	0.5495	0.5741	0.6431	0.3730	0.7495	0.9864	0.5862	-0.2130
-0.1635	-0.2118	-0.1973	-0.2134	-0.2440	-0.2315	-0.1473	-0.1869	-0.1362	-0.2076
-0.2395	-0.2224	-0.2263	-0.1832	-0.2454	-0.1710	-0.1955	-0.1538	-0.1977	-0.2141
-0.2236	-0.2190	-0.2273	-0.1926	-0.1172	-0.2237	-0.1528	-0.2177	0.8228	-0.1969
-0.2184	-0.2170	-0.2105	-0.1897	-0.2112	-0.1861	-0.2243	-0.2318	-0.1978	-0.2403
-0.2119	-0.2502	-0.1993	-0.1618	-0.1663	0.2325	-0.6735	-0.1255	-0.1925	-0.2045
-0.0860	0.5018	-0.6149	-0.1730	-0.2187	-0.2379				

Net Total: 0.00000000

M U L T I P O L E D E R I V E D C H A R G E A N A L Y S I S

See: M. Swart, P.Th. van Duijnen, J.G. Snijders, J.Comput.Chem., (2001), p. 79-88.

Multipole derived atomic charges (a.u.)

Atom	Level:	MDC-m	MDC-d	MDC-q
1	O	-0.349695	-0.324341	-0.349006
2	C	0.362109	0.374194	0.395666
3	C	0.242793	0.250491	0.254252
4	C	0.181123	0.166509	0.211879
5	O	-0.306222	-0.549392	-0.612879
6	N	-0.213838	-0.275271	-0.466267
7	O	-0.379575	-0.380875	-0.445562

8	C	0.250177	0.309076	0.307573
9	C	0.227894	0.193180	0.184882
10	C	0.339203	0.409525	0.416538
11	O	-0.382036	-0.352929	-0.392651
12	C	0.124344	0.102546	0.102330
13	O	-0.312980	-0.296219	-0.342603
14	C	0.244994	0.243313	0.248362
15	C	0.195854	0.158735	0.174905
16	O	-0.370232	-0.478894	-0.484276
17	C	0.227253	0.192490	0.197974
18	C	0.229584	0.193298	0.196710
19	O	-0.320038	-0.320620	-0.375436
20	C	0.057577	0.044073	-0.060435
21	C	0.195168	0.221518	0.283321
22	C	0.118453	0.104585	0.107168
23	C	0.119688	0.098866	0.108881
24	C	0.290999	0.268921	0.287860
25	C	0.173277	0.135174	0.137139
26	C	0.164636	0.124363	0.162650
27	C	0.248701	0.238211	0.260896
28	C	0.234467	0.193755	0.300560
29	C	0.034174	0.027206	-0.048885
30	C	0.237131	0.191363	0.177790
31	O	-0.372206	-0.476532	-0.519681
32	C	0.254271	0.242720	0.262466
33	C	0.223691	0.186296	0.186126
34	C	0.228200	0.296487	0.392784
35	C	0.221852	0.191789	0.201807
36	C	0.180408	0.145394	0.150145
37	C	0.293994	0.270847	0.311256
38	C	0.367787	0.338949	0.385678

39	C	0.229426	0.190011	0.156772
40	H	-0.073134	-0.059040	-0.023414
41	H	-0.057411	-0.032167	-0.018118
42	H	-0.063484	-0.056450	-0.029835
43	H	-0.056357	-0.034340	-0.012396
44	H	-0.068948	-0.052652	-0.050820
45	H	-0.070571	-0.057756	-0.051514
46	H	-0.076627	-0.072009	-0.069830
47	H	-0.071215	-0.061256	-0.062541
48	H	-0.063204	-0.041271	-0.022271
49	H	-0.081749	-0.061760	-0.069061
50	H	-0.055011	-0.046905	-0.049036
51	H	-0.072470	-0.054842	-0.059530
52	H	-0.070956	-0.059474	-0.066070
53	H	-0.067974	-0.064299	-0.073549
54	H	-0.057758	-0.044769	-0.052274
55	H	-0.054284	-0.043959	-0.045582
56	H	-0.057441	-0.042891	-0.030849
57	H	-0.067133	-0.050242	-0.050688
58	H	-0.061810	-0.035881	-0.033038
59	H	-0.075165	-0.060235	-0.049483
60	H	-0.074864	-0.065693	-0.056544
61	H	-0.068798	-0.054782	-0.043019
62	H	-0.067026	-0.048676	-0.039751
63	H	-0.072423	-0.074524	-0.083412
64	H	-0.058278	-0.035865	-0.035105
65	H	-0.074654	-0.049649	-0.057644
66	H	-0.072308	-0.063778	-0.060512
67	H	-0.068330	-0.054130	-0.066881
68	H	-0.065059	-0.056229	-0.055840
69	C	0.352655	0.439183	0.460674

70	H	-0.064660	-0.054298	-0.081694
71	H	-0.068171	-0.067239	-0.097231
72	H	-0.063309	-0.048332	-0.072848
73	H	-0.081098	-0.062548	-0.073498
74	H	-0.072960	-0.060199	-0.063704
75	H	-0.069440	-0.068745	-0.071796
76	H	-0.077261	-0.048240	-0.041100
77	H	-0.066318	-0.053995	-0.039597
78	H	-0.064710	-0.060005	-0.029822
79	H	-0.082480	-0.064110	-0.047275
80	H	-0.066598	-0.057787	-0.051938
81	H	-0.067778	-0.066678	-0.057134
82	H	-0.071261	-0.043559	-0.037610
83	H	-0.061741	-0.024870	-0.017994
84	H	-0.047267	-0.002479	-0.015881
85	H	-0.073278	-0.059691	-0.040674
86	H	0.063048	0.263781	0.298756
87	O	-0.365853	-0.445095	-0.461349
88	H	-0.044773	0.000811	0.021796
89	H	-0.045364	-0.030517	-0.018275
90	H	-0.052142	-0.050502	-0.041586
91	H	-0.042403	-0.011115	-0.012171
92	C	0.240778	0.197569	0.187865
93	O	-0.364772	-0.467209	-0.492396
94	H	-0.061033	-0.053359	-0.049494
95	H	-0.061393	-0.048631	-0.040512
96	H	-0.068380	-0.065427	-0.061597

*** Using NEW gradient routines ***

Geometry CYCLE 13

==============

Energy gradients wrt nuclear displacements

==

Atom	Cartesian (a.u./angstrom)		
	X	Y	Z
1 O	-0.000618	0.001391	-0.003860
2 C	0.001921	-0.002351	0.000548
3 C	-0.000881	0.000437	0.003470
4 C	0.001026	-0.001076	-0.000887
5 O	0.000039	-0.002166	-0.001269
6 N	-0.000378	0.000936	0.001163
7 O	-0.001380	0.000476	-0.002561
8 C	0.000102	-0.001405	0.000707
9 C	0.000959	-0.000186	0.000303
10 C	0.003960	0.004365	0.000386
11 O	-0.000372	-0.002358	-0.000637
12 C	-0.001238	-0.000430	0.000141
13 O	-0.002085	0.000577	-0.000506
14 C	-0.001737	0.003420	0.002853
15 C	0.000994	-0.000225	-0.001653
16 O	0.000736	0.001175	-0.001655
17 C	0.000509	0.000291	-0.000701
18 C	-0.000427	0.000194	-0.000223
19 O	-0.000427	0.000678	-0.003585
20 C	-0.000404	-0.000726	0.000346
21 C	-0.000396	0.000470	0.002027
22 C	0.000598	0.001025	-0.000213
23 C	-0.001901	-0.001073	-0.002434

24	C	0.000468	-0.000416	0.000316
25	C	0.000714	0.001043	0.000385
26	C	0.000845	-0.001410	0.000088
27	C	0.000481	-0.000142	-0.000365
28	C	0.000155	-0.000404	-0.000246
29	C	-0.000248	0.000358	-0.000713
30	C	0.000834	-0.001005	-0.000663
31	O	-0.001296	-0.000211	-0.000990
32	C	-0.000025	-0.000400	0.001270
33	C	0.000104	-0.000611	-0.000987
34	C	0.002416	0.000738	0.003320
35	C	-0.000333	-0.000567	0.001263
36	C	-0.000189	0.000423	-0.001020
37	C	0.000431	0.000192	0.000131
38	C	0.001033	-0.001387	0.000810
39	C	0.000355	-0.000184	0.000115
40	H	-0.000022	0.000250	-0.001499
41	H	0.000257	0.000717	-0.001008
42	H	0.000127	0.000233	-0.000798
43	H	0.000751	0.000714	0.000261
44	H	0.000304	0.000421	0.000209
45	H	-0.000002	0.000070	-0.000620
46	H	-0.000401	0.000243	0.000013
47	H	-0.000502	-0.000293	0.000386
48	H	0.000985	-0.000716	-0.000074
49	H	0.000122	-0.000308	0.000188
50	H	0.000205	0.000727	0.000427
51	H	-0.000377	0.000180	0.000767
52	H	-0.000138	-0.000168	-0.000079
53	H	0.000059	0.000727	0.000232
54	H	-0.000205	-0.000280	0.001198

55	H	-0.000566	-0.000288	-0.000459
56	H	-0.000217	0.000149	-0.000384
57	H	0.000480	-0.000602	0.000109
58	H	0.000247	-0.000095	0.000405
59	H	-0.000440	0.000258	0.000436
60	H	-0.000327	0.000076	-0.000441
61	H	-0.000053	-0.000442	0.000023
62	H	0.000055	-0.000354	-0.000133
63	H	-0.000168	0.000090	-0.000379
64	H	0.000606	0.000300	0.000411
65	H	-0.000365	0.000194	-0.000431
66	H	-0.000086	0.000207	0.000854
67	H	0.000716	0.000676	0.000076
68	H	-0.000035	-0.000171	-0.000097
69	C	0.002198	-0.001665	0.002907
70	H	0.000299	0.000401	-0.000299
71	H	0.000008	0.000121	0.000496
72	H	-0.000508	0.000085	-0.000117
73	H	-0.000053	-0.000097	0.000147
74	H	0.000506	0.000312	-0.000109
75	H	-0.000114	0.000398	0.000600
76	H	0.000380	0.000397	-0.000179
77	H	-0.000094	0.000431	-0.000094
78	H	0.000605	0.000120	0.000299
79	H	0.000144	-0.000140	0.000151
80	H	-0.000198	-0.000217	0.000169
81	H	0.000444	0.000066	0.000464
82	H	-0.001857	-0.001095	-0.000141
83	H	-0.000061	0.000593	0.000774
84	H	-0.000304	0.000233	0.000374
85	H	-0.000624	0.000057	0.000635

86 H -0.000836 -0.000179 -0.000661

87 O -0.002379 -0.002497 0.000812

88 H -0.000101 -0.000478 0.000761

89 H 0.000426 -0.000151 -0.000125

90 H -0.000170 0.000094 0.000407

91 H -0.000292 -0.000088 0.000057

92 C -0.001510 0.000107 0.000169

93 O -0.001292 0.000984 -0.000237

94 H 0.000387 0.000255 -0.000715

95 H -0.000074 0.000335 0.000083

96 H -0.000287 -0.000357 -0.000696

----

dE(predicted): -0.39432E-03, dE(actual): -0.10737E-03, Trust radius: 0.195959, all in a.u.

Interpolation step length 0.141749446635075

Geometry Convergence after Step 13 (Hartree/Angstrom,Angstrom)

----

current energy -19.67438175 Hartree

energy change -0.00010737 0.00100000 T

constrained gradient max 0.00436511 0.00100000 F

constrained gradient rms 0.00098538 0.00066667 F

gradient max 0.00436511

gradient rms 0.00098538

cart. step max 0.14579168 0.01000000 F

cart. step rms 0.04853958 0.00666667 F

Coordinates (Cartesian)

======================

Atom	bohr			angstrom			Geometric Variables		
	X	Y	Z	X	Y	Z	(0:frozen, *:LT par.)		
1 O	26.590867	61.654742	50.436467	14.071281	32.626285	26.689829	1	2	3
2 C	27.063348	61.993044	53.014555	14.321307	32.805306	28.054095	4	5	6
3 C	29.835425	61.220895	53.454492	15.788227	32.396703	28.286899	7	8	9
4 C	30.558203	61.567317	56.262560	16.170704	32.580021	29.772865	10	11	12
5 O	27.307208	53.004361	43.140281	14.450352	28.048700	22.828853	13	14	15
6 N	33.161709	60.870355	56.974625	17.548421	32.211205	30.149673	16	17	18
7 O	26.727434	64.608902	53.667298	14.143549	34.189558	28.399511	19	20	21
8 C	20.463133	58.483862	48.379830	10.828624	30.948327	25.601503	22	23	24
9 C	25.583447	65.414574	41.452512	13.538177	34.615902	21.935725	25	26	27
10 C	21.668977	55.069412	45.031370	11.466729	29.141478	23.829575	28	29	30
11 O	21.664370	62.726823	45.711193	11.464291	33.193605	24.189322	31	32	33
12 C	19.553488	56.565257	46.304615	10.347260	29.933045	24.503347	34	35	36
13 O	21.601852	55.211254	42.491807	11.431208	29.216538	22.485696	37	38	39
14 C	24.001402	61.544402	49.536416	12.700995	32.567895	26.213542	40	41	42
15 C	23.982550	63.267113	47.114111	12.691019	33.479514	24.931714	43	44	45
16 O	18.840682	59.534936	49.643355	9.970059	31.504531	26.270132	46	47	48
17 C	23.781578	56.976583	51.278678	12.584669	30.150710	27.135508	49	50	51
18 C	27.450947	53.194966	38.639543	14.526416	28.149564	20.447166	52	53	54
19 O	30.097859	58.569334	52.725265	15.927101	30.993557	27.901009	55	56	57
20 C	26.343486	57.109809	41.077533	13.940372	30.221210	21.737295	58	59	60
21 C	26.101512	54.226882	41.023123	13.812325	28.695630	21.708502	61	62	63
22 C	23.268457	58.754085	48.998567	12.313137	31.091323	25.928925	64	65	66
23 C	25.878937	62.712467	42.466095	13.694544	33.186008	22.472090	67	68	69
24 C	35.169101	61.856396	55.326932	18.610687	32.732995	29.277752	70	71	72
25 C	26.251903	62.503450	45.375246	13.891909	33.075402	24.011546	73	74	75
26 C	29.950970	64.336149	57.000008	15.849371	34.045224	30.163105	76	77	78

27	C	27.213921	65.075506	56.338248	14.400987	34.436475	29.812917	79	80	81
28	C	26.629110	67.859515	56.810034	14.091518	35.909709	30.062576	82	83	84
29	C	28.381402	58.518444	41.649114	15.018791	30.966627	22.039762	85	86	87
30	C	17.636475	57.814483	44.498135	9.332821	30.594107	23.547399	88	89	90
31	O	29.933453	62.538396	40.345198	15.840101	33.093894	21.349759	91	92	93
32	C	23.219647	53.535486	40.869635	12.287308	28.329759	21.627280	94	95	96
33	C	30.964441	57.586964	42.440187	16.385677	30.473709	22.458380	97	98	99
34	C	28.196978	61.366873	41.336869	14.921198	32.473951	21.874529	100	101	102
35	C	24.214073	66.052188	47.932969	12.813536	34.953313	25.365035	103	104	105
36	C	22.571365	50.748227	41.318165	11.944252	26.854805	21.864632	106	107	108
37	C	33.534632	58.175760	57.536675	17.745763	30.785286	30.447097	109	110	111
38	C	19.429401	64.141233	46.354628	10.281596	33.942079	24.529813	112	113	114
39	C	19.731674	50.166945	41.089829	10.441552	26.547204	21.743801	115	116	117
40	H	24.657386	58.097866	40.433357	13.048127	30.744066	21.396411	118	119	120
41	H	31.468216	58.394156	44.289072	16.652263	30.900856	23.436767	121	122	123
42	H	31.056284	55.534153	42.569571	16.434278	29.387408	22.526847	124	125	126
43	H	32.376949	58.292075	41.095714	17.133143	30.846838	21.746915	127	128	129
44	H	27.147712	66.620341	42.074822	14.365950	35.253966	22.265037	130	131	132
45	H	25.582696	65.422078	39.380764	13.537780	34.619873	20.839403	133	134	135
46	H	23.799453	66.232522	42.105644	12.594128	35.048741	22.281347	136	137	138
47	H	24.179792	61.623854	42.000604	12.795395	32.609939	22.225762	139	140	141

48 H	27.896447	63.629832	45.929735	14.762164	33.671457	24.304969	142	143	144
49 H	26.771030	60.546670	45.788532	14.166619	32.039918	24.230248	145	146	147
50 H	25.991500	66.296240	48.954642	13.754109	35.082459	25.905681	148	149	150
51 H	22.694495	66.607243	49.222386	12.009409	35.247035	26.047365	151	152	153
52 H	24.208592	67.312917	46.296780	12.810635	35.620461	24.499201	154	155	156
53 H	19.637917	66.150548	45.882976	10.391938	35.005363	24.280225	157	158	159
54 H	18.915292	63.932378	48.354177	10.009541	33.831557	25.587928	160	161	162
55 H	17.912668	63.337680	45.205757	9.478976	33.516857	23.921856	163	164	165
56 H	22.542053	54.109078	38.997689	11.928741	28.633291	20.636688	166	167	168
57 H	23.623240	49.650677	39.909010	12.500880	26.274007	21.118938	169	170	171
58 H	23.281912	50.157628	43.165463	12.320257	26.542274	22.842179	172	173	174
59 H	18.635311	51.163187	42.538528	9.861382	27.074393	22.510419	175	176	177
60 H	18.983908	50.729995	39.237584	10.045852	26.845157	20.763635	178	179	180
61 H	19.390985	48.137469	41.325810	10.261267	25.473252	21.868677	181	182	183
62 H	27.270225	51.136932	38.522523	14.430782	27.060499	20.385241	184	185	186
63 H	26.686227	54.054441	36.918391	14.121743	28.604378	19.536371	187	188	189
64 H	29.459631	53.664498	38.760768	15.589365	28.398029	20.511315	190	191	192
65 H	24.397386	58.081440	47.405008	12.910541	30.735374	25.085650	193	194	195
66 H	22.649639	57.557262	52.914321	11.985673	30.457991	28.001053	196	197	198

67 H 25.778470 57.022862 51.791228 13.641379 30.175199 27.406737 199 200 201

68 H 23.283680 55.033475 50.781054 12.321193 29.122461 26.872177 202 203 204

69 C 31.030279 58.069013 50.335527 16.420517 30.728798 26.636414 205 206 207

70 H 27.874312 69.057755 55.666481 14.750451 36.543790 29.457433 208 209 210

71 H 26.912636 68.327441 58.808792 14.241554 36.157324 31.120272 211 212 213

72 H 24.666429 68.281532 56.302610 13.052912 36.133031 29.794058 214 215 216

73 H 25.902369 63.890718 57.456421 13.706943 33.809512 30.404628 217 218 219

74 H 31.232246 65.634794 56.011158 16.527393 34.732437 29.639828 220 221 222

75 H 30.277635 64.563921 59.034137 16.022234 34.165755 31.239520 223 224 225

76 H 35.267371 60.907325 53.465736 18.662689 32.230768 28.292849 226 227 228

77 H 34.910344 63.882491 54.999092 18.473759 33.805159 29.104266 229 230 231

78 H 36.996251 61.604105 56.272488 19.577573 32.599488 29.778118 232 233 234

79 H 33.522141 56.952316 55.843317 17.739153 30.137868 29.551011 235 236 237

80 H 32.047800 57.515450 58.818453 16.958965 30.435865 31.125385 238 239 240

81 H 35.357920 57.936600 58.496979 18.710605 30.658728 30.955268 241 242 243

82 H 25.784335 60.834238 54.194625 13.644483 32.192092 28.678560 244 245 246

83 H 22.713353 62.336286 50.954149 12.019389 32.986942 26.963775 247 248 249

84 H 31.017666 62.382640 52.219924 16.413842 33.011471 27.633593 250 251 252

85 H 29.311608 60.333676 57.377386 15.511035 31.927206 30.362805 253 254 255

86 H 26.174547 53.222289 44.595507 13.850974 28.164022 23.598926 256 257
258

87 O 23.206028 53.819079 46.244615 12.280101 28.479830 24.471597 259 260
261

88 H 18.564451 55.129248 47.442877 9.823884 29.173142 25.105690 262 263
264

89 H 32.154900 54.695351 48.400931 17.015640 28.943534 25.612670 265 266
267

90 H 30.538161 54.119406 51.343758 16.160099 28.638756 27.169946 268 269
270

91 H 28.844805 55.159023 48.637377 15.264013 29.188898 25.737791 271 272
273

92 C 30.642121 55.315839 49.662388 16.215112 29.271881 26.280204 274 275
276

93 O 32.032025 59.623747 48.977642 16.950618 31.551528 25.917852 277 278
279

94 H 18.584194 59.199232 43.292252 9.834332 31.326884 22.909273 280 281
282

95 H 16.180026 58.767601 45.611693 8.562101 31.098475 24.136668 283 284
285

96 H 16.742514 56.396252 43.287237 8.859757 29.843611 22.906619 286 287
288

Number of elements of the density matrix on this node (used, total): 222269 1773786

Numerical Integration : Fuzzy Cells (Becke)

Becke grid quality: NORMAL

Lebedev angular grid order: min: 11 max: 23

Nr. of radial points: min: 46 max: 52

Total nr. of points: 600099

Nr. of blocks: 4689

Block length: 128

Nr. of dummy points: 93

Test of Precision of the Numerical Integration Grid

==

Integral of the Total Core Density: 85.99993245325885

Relative Error: -7.854E-07

Density Fitting (zlmFit)

ZlmFit Fit Quality: NORMAL

Max L-expansion: min: 6 max: 7

Nr. of radial interpolation points: min: 39 max: 48

S C F *** ScaSCF ***

Initial density matrix is taken from previous geometry

CYCLE 1

SCF Error: norm([F,P])= 0.163126082078, max([F,P])= 0.010175958604

orbitals (Q,E):

A :114...133 (2.00 -0.2513) (2.00 -0.2502) (2.00 -0.2392) (2.00 -0.2368)

(2.00 -0.2297) (2.00 -0.2239) (2.00 -0.2227) (2.00 -0.2170)

(2.00 -0.1976) (2.00 -0.1793) (0.00 -0.0716) (0.00 -0.0705)

(0.00 -0.0660) (0.00 -0.0401) (0.00 -0.0207) (0.00 -0.0152)

(0.00 -0.0135) (0.00 -0.0089) (0.00 -0.0065) (0.00 -0.0023)

CYCLE 2

SDIIS (wt 0.000): 0.0492 0.9508

A-DIIS (wt 1.000): 0.0000 1.0000

DIIS coefficients: 0.0000 1.0000

SCF Error: norm([F,P])= 0.031695477782, max([F,P])= 0.002181298516

orbitals (Q,E):

A :114...124 (2.00 -0.2469) (2.00 -0.2464) (2.00 -0.2366) (2.00 -0.2316)

(2.00 -0.2256) (2.00 -0.2208) (2.00 -0.2171) (2.00 -0.2149)

(2.00 -0.1939) (2.00 -0.1758) (0.00 -0.0693)

CYCLE 3

SDIIS (wt 0.000): 0.0040 0.6191 0.3769

A-DIIS (wt 1.000): 0.0000 0.6796 0.3204

DIIS coefficients: 0.0000 0.6796 0.3204

SCF Error: norm([F,P])= 0.050262917603, max([F,P])= 0.004283712915

orbitals (Q,E):

A :114...124 (2.00 -0.2486) (2.00 -0.2475) (2.00 -0.2369) (2.00 -0.2340)

(2.00 -0.2268) (2.00 -0.2214) (2.00 -0.2191) (2.00 -0.2150)

(2.00 -0.1937) (2.00 -0.1767) (0.00 -0.0688)

CYCLE 4

SDIIS (wt 0.990): 0.3649 0.2022 0.4328

A-DIIS (wt 0.010): 0.0000 0.0000 1.0000

DIIS coefficients: 0.3613 0.2002 0.4385

SCF Error: norm([F,P])= 0.012867689564, max([F,P])= 0.001983133714

orbitals (Q,E):

A :114...124 (2.00 -0.2486) (2.00 -0.2477) (2.00 -0.2367) (2.00 -0.2341)

(2.00 -0.2271) (2.00 -0.2214) (2.00 -0.2194) (2.00 -0.2151)

(2.00 -0.1948) (2.00 -0.1769) (0.00 -0.0689)

CYCLE 5

SDIIS (wt 0.998): 0.1639 0.0828 0.3366 0.4167

A-DIIS (wt 0.002): 0.0000 0.0000 0.2510 0.7490

DIIS coefficients: 0.1635 0.0826 0.3364 0.4175

SCF Error: norm([F,P])= 0.007116328261, max([F,P])= 0.001232388784

orbitals (Q,E):

A :114...124 (2.00 -0.2485) (2.00 -0.2476) (2.00 -0.2368) (2.00 -0.2340)

(2.00 -0.2270) (2.00 -0.2214) (2.00 -0.2193) (2.00 -0.2151)

(2.00 -0.1945) (2.00 -0.1768) (0.00 -0.0689)

CYCLE 6

SDIIS (wt 1.000): 0.0703 0.0333 0.1725 0.2742 0.4498

DIIS coefficients: 0.0703 0.0333 0.1725 0.2742 0.4498

SCF Error: norm([F,P])= 0.001842741490, max([F,P])= 0.000230119257

orbitals (Q,E):

A :114...124 (2.00 -0.2486) (2.00 -0.2476) (2.00 -0.2368) (2.00 -0.2341)

(2.00 -0.2270) (2.00 -0.2214) (2.00 -0.2193) (2.00 -0.2151)

(2.00 -0.1945) (2.00 -0.1768) (0.00 -0.0689)

CYCLE 7

SDIIS (wt 1.000): 0.0241 0.0107 0.0698 0.1314 0.3209 0.4430

DIIS coefficients: 0.0241 0.0107 0.0698 0.1314 0.3209 0.4430

SCF Error: norm([F,P])= 0.000776149854, max([F,P])= 0.000126180951

orbitals (Q,E):

A :114...124 (2.00 -0.2486) (2.00 -0.2476) (2.00 -0.2368) (2.00 -0.2341)

(2.00 -0.2270) (2.00 -0.2214) (2.00 -0.2193) (2.00 -0.2151)

(2.00 -0.1945) (2.00 -0.1768) (0.00 -0.0689)

CYCLE 8

SDIIS (wt 1.000): 0.0064 0.0026 0.0228 0.0508 0.1619 0.3162 0.4393

DIIS coefficients: 0.0064 0.0026 0.0228 0.0508 0.1619 0.3162 0.4393

SCF Error: norm([F,P])= 0.000304447895, max([F,P])= 0.000050929537

orbitals (Q,E):

A :114...124 (2.00 -0.2486) (2.00 -0.2476) (2.00 -0.2368) (2.00 -0.2341)

(2.00 -0.2270) (2.00 -0.2214) (2.00 -0.2193) (2.00 -0.2151)

(2.00 -0.1945) (2.00 -0.1768) (0.00 -0.0689)

CYCLE 9

SDIIS (wt 1.000): 0.0013 0.0004 0.0068 0.0183 0.0713 0.1632 0.3106 0.4282

DIIS coefficients: 0.0013 0.0004 0.0068 0.0183 0.0713 0.1632 0.3106 0.4282

SCF Error: norm([F,P])= 0.000115072217, max([F,P])= 0.000013710566

orbitals (Q,E):

A :114...124 (2.00 -0.2486) (2.00 -0.2476) (2.00 -0.2368) (2.00 -0.2341)

(2.00   -0.2270) (2.00   -0.2214) (2.00   -0.2193) (2.00   -0.2151)

(2.00   -0.1945) (2.00   -0.1768) (0.00   -0.0689)

CYCLE   10

SDIIS (wt 1.000): 0.0000 -0.0001 0.0015 0.0055 0.0262 0.0676 0.1492 0.2811 0.4689

DIIS coefficients: 0.0000 -0.0001 0.0015 0.0055 0.0262 0.0676 0.1492 0.2811 0.4689

SCF Error: norm([F,P])=   0.000035566210, max([F,P])=   0.000003813328

orbitals (Q,E):

A :114...124   (2.00   -0.2486) (2.00   -0.2476) (2.00   -0.2368) (2.00   -0.2341)

(2.00   -0.2270) (2.00   -0.2214) (2.00   -0.2193) (2.00   -0.2151)

(2.00   -0.1945) (2.00   -0.1768) (0.00   -0.0689)

CYCLE   11

SDIIS (wt 1.000): -0.0002 -0.0001 0.0002 0.0014 0.0092 0.0269 0.0679 0.1514 0.3652 0.3782

DIIS coefficients: -0.0002 -0.0001 0.0002 0.0014 0.0092 0.0269 0.0679 0.1514 0.3652 0.3782

SCF Error: norm([F,P])=   0.000019876480, max([F,P])=   0.000002941096

orbitals (Q,E):

A :114...124   (2.00   -0.2486) (2.00   -0.2476) (2.00   -0.2368) (2.00   -0.2341)

(2.00   -0.2270) (2.00   -0.2214) (2.00   -0.2193) (2.00   -0.2151)

(2.00   -0.1945) (2.00   -0.1768) (0.00   -0.0689)

CYCLE   12

SDIIS (wt 1.000): -0.0000 0.0002 0.0007 0.0038 0.0104 0.0273 0.0640 0.1820 0.2520 0.4597

DIIS coefficients: -0.0000 0.0002 0.0007 0.0038 0.0104 0.0273 0.0640 0.1820 0.2520 0.4597

SCF Error: norm([F,P])=   0.000005500070, max([F,P])=   0.000000672967

orbitals (Q,E):

A :114...124   (2.00   -0.2486) (2.00   -0.2476) (2.00   -0.2368) (2.00   -0.2341)

(2.00   -0.2270) (2.00   -0.2214) (2.00   -0.2193) (2.00   -0.2151)

(2.00 -0.1945) (2.00 -0.1768) (0.00 -0.0689)

SCF CONVERGED

CYCLE 13

*** Setting up for NEW gradients in Focky

*** Using FIT density in Focky

*** Setting up for NEW Hessian in Focky

orbitals (Q,E):

A :114...133 (2.00 -0.2486) (2.00 -0.2476) (2.00 -0.2368) (2.00 -0.2341)

(2.00 -0.2270) (2.00 -0.2214) (2.00 -0.2193) (2.00 -0.2151)

(2.00 -0.1945) (2.00 -0.1768) (0.00 -0.0689) (0.00 -0.0682)

(0.00 -0.0636) (0.00 -0.0388) (0.00 -0.0183) (0.00 -0.0129)

(0.00 -0.0106) (0.00 -0.0072) (0.00 -0.0046) (0.00 -0.0000)

Fit test: (difference of exact and fit density, squared integrated, result summed over spins)

SCF: 0.00100853122420

Gross Charges per Atom (Z minus electrons)

==

-0.6650	0.9235	0.5461	0.3333	-0.5817	-0.3813	-0.6314	0.6735	0.6255	0.8686
-0.6596	0.0007	-0.5769	0.5875	0.3248	-0.5920	0.5961	0.6009	-0.6038	0.2577
0.3379	0.0956	0.1676	0.7467	0.4135	0.4081	0.5606	0.5727	0.0218	0.6394
-0.5657	0.5510	0.5490	0.5723	0.6434	0.3709	0.7503	0.9868	0.5875	-0.2139
-0.1515	-0.2129	-0.2016	-0.2136	-0.2432	-0.2319	-0.1505	-0.1879	-0.1307	-0.2068
-0.2411	-0.2225	-0.2270	-0.1839	-0.2446	-0.1718	-0.1961	-0.1544	-0.1974	-0.2143
-0.2241	-0.2200	-0.2284	-0.1926	-0.1132	-0.2243	-0.1551	-0.2164	0.8271	-0.1968
-0.2184	-0.2171	-0.2103	-0.1908	-0.2122	-0.1839	-0.2256	-0.2333	-0.1965	-0.2413
-0.2131	-0.2532	-0.2012	-0.1604	-0.1664	0.2341	-0.6760	-0.1261	-0.1944	-0.2088

-0.0759 0.5035 -0.6187 -0.1752 -0.2154 -0.2381

Net Total: -0.00000000

M U L T I P O L E   D E R I V E D   C H A R G E   A N A L Y S I S

See: M. Swart, P.Th. van Duijnen, J.G. Snijders, J.Comput.Chem., (2001), p. 79-88.

Multipole derived atomic charges (a.u.)

Atom	Level:	MDC-m	MDC-d	MDC-q
1	O	-0.350812	-0.322490	-0.346409
2	C	0.362891	0.374611	0.396415
3	C	0.242474	0.249781	0.254134
4	C	0.180863	0.166515	0.211579
5	O	-0.309287	-0.551128	-0.612615
6	N	-0.214220	-0.277151	-0.468520
7	O	-0.380438	-0.382528	-0.447867
8	C	0.250531	0.310019	0.308152
9	C	0.228001	0.193092	0.187162
10	C	0.339988	0.408087	0.413616
11	O	-0.383298	-0.357016	-0.398499
12	C	0.124287	0.102242	0.102719
13	O	-0.312057	-0.300983	-0.346014
14	C	0.245287	0.243085	0.248299

15	C	0.196209	0.159191	0.175378
16	O	-0.370240	-0.478898	-0.483952
17	C	0.227249	0.192549	0.198707
18	C	0.229558	0.193122	0.194479
19	O	-0.319364	-0.318522	-0.371661
20	C	0.057177	0.044826	-0.056471
21	C	0.194826	0.221513	0.284324
22	C	0.118289	0.104677	0.107177
23	C	0.119875	0.099612	0.110413
24	C	0.290874	0.268768	0.287309
25	C	0.172736	0.134513	0.136223
26	C	0.164787	0.124816	0.164012
27	C	0.249196	0.238833	0.260614
28	C	0.234514	0.193770	0.304407
29	C	0.034742	0.027762	-0.046595
30	C	0.236984	0.190882	0.176929
31	O	-0.372537	-0.477009	-0.517912
32	C	0.254209	0.243970	0.264476
33	C	0.222838	0.185520	0.189622
34	C	0.228391	0.296214	0.387268
35	C	0.222020	0.192196	0.201885
36	C	0.180177	0.145639	0.150060
37	C	0.293880	0.270940	0.311690
38	C	0.368457	0.340078	0.388028
39	C	0.229271	0.189983	0.158926
40	H	-0.073507	-0.060206	-0.026917
41	H	-0.056474	-0.030711	-0.017554
42	H	-0.063942	-0.056509	-0.031175
43	H	-0.056891	-0.035915	-0.015572
44	H	-0.069047	-0.053056	-0.051534
45	H	-0.070422	-0.057264	-0.052264

46	H	-0.076634	-0.072110	-0.070234
47	H	-0.071995	-0.061909	-0.062416
48	H	-0.063903	-0.041512	-0.022584
49	H	-0.082256	-0.062022	-0.069360
50	H	-0.055108	-0.046887	-0.049060
51	H	-0.072803	-0.055330	-0.060146
52	H	-0.070623	-0.058758	-0.065082
53	H	-0.067847	-0.064187	-0.073415
54	H	-0.057714	-0.044417	-0.052159
55	H	-0.054127	-0.043689	-0.045529
56	H	-0.057039	-0.042223	-0.030497
57	H	-0.067081	-0.050362	-0.051339
58	H	-0.061845	-0.036367	-0.033221
59	H	-0.075513	-0.060994	-0.050814
60	H	-0.074726	-0.065248	-0.056659
61	H	-0.068925	-0.055134	-0.043954
62	H	-0.067346	-0.049157	-0.040847
63	H	-0.072732	-0.075148	-0.083241
64	H	-0.058128	-0.035715	-0.033696
65	H	-0.074623	-0.048088	-0.056803
66	H	-0.072721	-0.064863	-0.061741
67	H	-0.068222	-0.054754	-0.068099
68	H	-0.065027	-0.056726	-0.056051
69	C	0.353409	0.438303	0.459305
70	H	-0.064616	-0.054163	-0.082275
71	H	-0.068087	-0.067204	-0.098125
72	H	-0.063209	-0.048341	-0.074132
73	H	-0.080903	-0.062417	-0.072887
74	H	-0.072898	-0.060153	-0.064023
75	H	-0.069528	-0.068870	-0.072733
76	H	-0.076901	-0.047530	-0.041048

77	H	-0.066455	-0.054284	-0.039425
78	H	-0.064906	-0.060379	-0.029249
79	H	-0.082441	-0.064114	-0.047546
80	H	-0.066840	-0.058210	-0.052173
81	H	-0.067958	-0.066997	-0.057445
82	H	-0.070871	-0.044009	-0.037652
83	H	-0.062546	-0.025957	-0.019252
84	H	-0.047210	-0.003568	-0.017075
85	H	-0.073742	-0.059853	-0.040996
86	H	0.060294	0.260008	0.290804
87	O	-0.359760	-0.434159	-0.449968
88	H	-0.044648	0.000660	0.021052
89	H	-0.044815	-0.029255	-0.016719
90	H	-0.052149	-0.050367	-0.041190
91	H	-0.042331	-0.010894	-0.011282
92	C	0.240757	0.197429	0.186589
93	O	-0.362692	-0.462017	-0.489131
94	H	-0.061024	-0.053267	-0.049564
95	H	-0.060601	-0.046834	-0.038182
96	H	-0.068435	-0.065378	-0.061202

*** Using NEW gradient routines ***

Geometry CYCLE 14

==============

Energy gradients wrt nuclear displacements

==

Atom Cartesian (a.u./angstrom)

X Y Z

1	O	0.000845	-0.000482	-0.002007
2	C	0.000849	-0.000460	0.001246
3	C	-0.001755	0.000515	-0.000024
4	C	-0.000151	0.000399	-0.000429
5	O	-0.002652	-0.000105	0.001177
6	N	-0.000657	0.000294	-0.000354
7	O	0.001052	0.000208	0.001263
8	C	-0.000354	0.001125	-0.001615
9	C	-0.000250	0.000193	-0.000764
10	C	0.004471	0.001766	0.002949
11	O	-0.002008	-0.000858	0.002481
12	C	-0.000081	-0.000258	0.001537
13	O	0.001218	-0.000950	-0.003511
14	C	-0.000826	0.000346	-0.001420
15	C	0.001518	-0.000903	0.000444
16	O	0.000054	0.001006	-0.000277
17	C	0.000614	-0.000526	-0.001567
18	C	0.000597	-0.000044	-0.000153
19	O	0.000706	-0.000434	0.000404
20	C	0.000284	-0.000250	0.001402
21	C	-0.001401	0.000238	0.000963
22	C	-0.000501	-0.002035	-0.000180
23	C	0.001101	-0.000785	-0.001681
24	C	0.000234	-0.000300	0.000296
25	C	-0.000253	0.000622	-0.000457
26	C	0.000848	-0.000844	0.000111
27	C	-0.000562	0.000764	0.000017
28	C	0.000441	-0.000343	-0.000058
29	C	-0.001143	0.000560	0.000055
30	C	-0.001191	0.000539	0.000202

31	O	0.000163	-0.000274	-0.000781
32	C	-0.001001	0.000629	0.000856
33	C	0.000166	-0.001718	-0.002605
34	C	-0.000228	0.000746	0.003166
35	C	0.000092	0.000482	-0.000408
36	C	-0.000206	0.000846	-0.000081
37	C	-0.000028	0.000141	-0.000154
38	C	0.000374	-0.001166	0.000405
39	C	0.000525	0.000094	0.000362
40	H	0.000190	-0.000106	-0.001717
41	H	0.000396	0.000994	0.000734
42	H	0.000427	0.000272	-0.000285
43	H	0.000597	0.000527	0.000277
44	H	0.000182	0.000217	0.000100
45	H	-0.000133	0.000257	-0.000356
46	H	0.000152	-0.000077	-0.000624
47	H	0.000441	0.000415	0.000500
48	H	0.000158	0.000241	-0.000079
49	H	0.000015	-0.001020	-0.000089
50	H	-0.000151	0.000473	0.000118
51	H	0.000130	-0.000140	0.000555
52	H	-0.000010	0.000000	0.000587
53	H	-0.000214	0.000344	0.000110
54	H	-0.000150	-0.000252	0.000984
55	H	-0.000435	-0.000082	-0.000411
56	H	-0.000420	-0.000211	-0.000308
57	H	0.000364	-0.000189	0.000088
58	H	-0.000026	0.000235	0.000651
59	H	-0.000154	0.000189	0.000313
60	H	-0.000138	0.000023	-0.000356
61	H	0.000089	-0.000244	0.000043

62	H	0.000044	-0.000138	-0.000055
63	H	0.000058	0.000012	-0.000213
64	H	0.000588	0.000064	0.000149
65	H	-0.000612	-0.001880	-0.001383
66	H	-0.000100	-0.000250	0.000419
67	H	0.000442	0.000237	0.000040
68	H	-0.000374	-0.000821	-0.000452
69	C	-0.005559	0.001517	-0.001584
70	H	0.000174	0.000224	-0.000167
71	H	0.000042	0.000112	0.000316
72	H	-0.000546	0.000141	-0.000194
73	H	-0.000196	-0.000063	0.000098
74	H	0.000378	0.000247	-0.000012
75	H	-0.000059	0.000340	0.000378
76	H	0.000148	0.000150	-0.000232
77	H	-0.000003	0.000054	-0.000040
78	H	0.000434	0.000105	0.000248
79	H	0.000024	-0.000077	0.000134
80	H	-0.000161	-0.000089	0.000050
81	H	0.000384	0.000098	0.000303
82	H	-0.000348	-0.000335	0.000643
83	H	-0.000378	0.000031	-0.000586
84	H	0.000277	0.000090	0.000239
85	H	-0.000371	0.000164	0.000435
86	H	0.000092	-0.000156	-0.000081
87	O	0.000116	0.001118	-0.000634
88	H	0.000052	-0.000176	0.000511
89	H	0.000859	-0.000417	0.000059
90	H	0.000011	-0.000187	0.000119
91	H	-0.000761	0.000600	0.000250
92	C	0.001149	-0.000862	0.001038

93 O 0.002223 -0.000593 -0.000853

94 H 0.000064 -0.000041 -0.000267

95 H -0.000097 0.000189 -0.000035

96 H -0.000208 -0.000049 -0.000289

----

dE(predicted): -0.37132E-03, dE(actual): -0.29676E-03, Trust radius: 0.195959, all in a.u.

Interpolation step length 0.120168861555098

Geometry Convergence after Step 14 (Hartree/Angstrom,Angstrom)

----

current energy	-19.67467851 Hartree		
energy change	-0.00029676	0.00100000	T
constrained gradient max	0.00555917	0.00100000	F
constrained gradient rms	0.00087215	0.00066667	F
gradient max	0.00555917		
gradient rms	0.00087215		
cart. step max	0.15945123	0.01000000	F
cart. step rms	0.05967435	0.00666667	F

Coordinates (Cartesian)

======================

Atom	bohr			angstrom			Geometric Variables		
	X	Y	Z	X	Y	Z	(0:frozen, *:LT par.)		
1 O	26.527578	61.629895	50.523114	14.037790	32.613136	26.735681	1	2	3
2 C	27.002861	61.985355	53.092469	14.289299	32.801237	28.095325	4	5	6

3 C	29.804953	61.287805	53.457617	15.772102	32.432110	28.288553	7	8	9
4 C	30.594367	61.634144	56.249842	16.189842	32.615385	29.766134	10	11	12
5 O	27.259303	52.828052	42.938876	14.425002	27.955401	22.722275	13	14	15
6 N	33.237764	61.023442	56.905328	17.588667	32.292215	30.113003	16	17	18
7 O	26.602540	64.584334	53.773523	14.077458	34.176558	28.455723	19	20	21
8 C	20.443153	58.441922	48.417996	10.818051	30.926133	25.621700	22	23	24
9 C	25.795874	65.355364	41.532031	13.650589	34.584569	21.977805	25	26	27
10 C	21.628314	55.039621	45.020798	11.445211	29.125713	23.823980	28	29	30
11 O	21.715357	62.729650	45.688712	11.491272	33.195101	24.177425	31	32	33
12 C	19.532982	56.546187	46.314735	10.336409	29.922953	24.508702	34	35	36
13 O	21.558586	55.215622	42.483209	11.408312	29.218849	22.481146	37	38	39
14 C	23.960974	61.528685	49.568779	12.679601	32.559578	26.230668	40	41	42
15 C	24.009846	63.241460	47.139902	12.705464	33.465939	24.945362	43	44	45
16 O	18.812307	59.499359	49.668688	9.955044	31.485705	26.283538	46	47	48
17 C	23.767622	57.001356	51.360509	12.577284	30.163819	27.178811	49	50	51
18 C	27.295946	53.219705	38.457150	14.444392	28.162655	20.350647	52	53	54
19 O	30.091079	58.653455	52.677480	15.923513	31.038072	27.875722	55	56	57
20 C	26.329292	57.045983	41.088600	13.932861	30.187434	21.743151	58	59	60
21 C	26.024966	54.173028	40.913799	13.771819	28.667132	21.650650	61	62	63
22 C	23.246364	58.732987	49.046452	12.301446	31.080159	25.954265	64	65	66
23 C	25.998465	62.642733	42.539031	13.757795	33.149107	22.510686	67	68	69
24 C	35.178412	62.086553	55.224569	18.615614	32.854789	29.223583	70	71	72
25 C	26.297325	62.415917	45.454076	13.915945	33.029081	24.053261	73	74	75
26 C	29.908037	64.374742	57.029869	15.826652	34.065647	30.178907	76	77	78
27 C	27.131393	65.030188	56.440873	14.357315	34.412493	29.867224	79	80	81
28 C	26.467189	67.787376	56.963170	14.005833	35.871534	30.143612	82	83	84
29 C	28.398466	58.382822	41.717274	15.027821	30.894859	22.075831	85	86	87
30 C	17.626279	57.827699	44.521365	9.327425	30.601101	23.559692	88	89	90
31 O	30.101898	62.378704	40.525189	15.929239	33.009388	21.445007	91	92	93
32 C	23.124308	53.552283	40.787815	12.236857	28.338648	21.583982	94	95	96
33 C	30.939536	57.353082	42.521040	16.372497	30.349944	22.501165	97	98	99

34 C	28.309088	61.242647	41.459477	14.980524	32.408213	21.939410	100	101	102
35 C	24.280944	66.029978	47.938910	12.848922	34.941560	25.368179	103	104	105
36 C	22.419680	50.760889	41.122652	11.863984	26.861506	21.761170	106	107	108
37 C	33.719728	58.341363	57.453896	17.843711	30.872920	30.403293	109	110	111
38 C	19.487403	64.184322	46.261445	10.312289	33.964881	24.480503	112	113	114
39 C	19.564589	50.248012	40.913273	10.353135	26.590103	21.650372	115	116	117
40 H	24.653729	58.098516	40.524049	13.046192	30.744410	21.444403	118	119	120
41 H	31.437134	58.092836	44.399915	16.635815	30.741405	23.495423	121	122	123
42 H	30.960894	55.294848	42.595043	16.383800	29.260773	22.540326	124	125	126
43 H	32.395328	58.047120	41.217198	17.142869	30.717213	21.811202	127	128	129
44 H	27.378075	66.516330	42.193301	14.487854	35.198926	22.327733	130	131	132
45 H	25.840176	65.370796	39.460766	13.674032	34.592735	20.881738	133	134	135
46 H	24.020278	66.217829	42.151664	12.710984	35.040966	22.305700	136	137	138
47 H	24.281242	61.603438	42.026675	12.849080	32.599135	22.239559	139	140	141
48 H	27.957253	63.498225	46.048865	14.794341	33.601814	24.368010	142	143	144
49 H	26.751179	60.445362	45.875096	14.156114	31.986308	24.276055	145	146	147
50 H	26.041630	66.244956	48.996174	13.780637	35.055321	25.927658	148	149	150
51 H	22.745402	66.629860	49.189276	12.036348	35.259003	26.029844	151	152	153
52 H	24.337999	67.275948	46.291860	12.879114	35.600899	24.496598	154	155	156

53	H	19.741997	66.187400	45.784521	10.447015	35.024864	24.228125	157	158	159
54	H	18.915937	63.995210	48.247602	10.009883	33.864807	25.531531	160	161	162
55	H	17.989429	63.397236	45.076917	9.519596	33.548373	23.853677	163	164	165
56	H	22.438702	54.214257	38.948129	11.874050	28.688949	20.610462	166	167	168
57	H	23.423193	49.708417	39.645263	12.395020	26.304561	20.979370	169	170	171
58	H	23.147693	50.068919	42.927430	12.249232	26.495331	22.716217	172	173	174
59	H	18.511600	51.194398	42.425943	9.795917	27.090909	22.450842	175	176	177
60	H	18.799340	50.915112	39.103181	9.948182	26.943117	20.692512	178	179	180
61	H	19.184653	48.216632	41.056684	10.152081	25.515143	21.726261	181	182	183
62	H	27.073120	51.171915	38.257644	14.326478	27.079011	20.245073	184	185	186
63	H	26.505164	54.165773	36.794029	14.025929	28.663293	19.470562	187	188	189
64	H	29.315296	53.648210	38.548902	15.512987	28.389410	20.399201	190	191	192
65	H	24.392393	58.052390	47.469608	12.907898	30.720002	25.119835	193	194	195
66	H	22.623879	57.602477	52.980618	11.972041	30.481918	28.036135	196	197	198
67	H	25.763979	57.073828	51.878176	13.633711	30.202169	27.452749	199	200	201
68	H	23.290645	55.044859	50.898206	12.324879	29.128485	26.934171	202	203	204
69	C	31.154585	58.222000	50.325946	16.486296	30.809755	26.631344	205	206	207
70	H	27.644823	69.040652	55.807010	14.629010	36.534739	29.531798	208	209	210
71	H	26.782975	68.239139	58.960916	14.172940	36.110597	31.200773	211	212	213

72	H	24.480298	68.150385	56.507904	12.954416	36.063631	29.902695	214	215	216
73	H	25.885266	63.787729	57.571350	13.697893	33.755013	30.465447	217	218	219
74	H	31.120933	65.726305	56.025660	16.468488	34.780863	29.647502	220	221	222
75	H	30.274201	64.591063	59.058466	16.020417	34.180118	31.252394	223	224	225
76	H	35.278305	61.147571	53.358231	18.668475	32.357901	28.235960	226	227	228
77	H	34.842938	64.103757	54.909319	18.438089	33.922248	29.056760	229	230	231
78	H	37.030023	61.896203	56.136086	19.595444	32.754060	29.705937	232	233	234
79	H	33.717872	57.120123	55.759239	17.842729	30.226668	29.506519	235	236	237
80	H	32.284912	57.625235	58.764977	17.084440	30.493961	31.097087	238	239	240
81	H	35.569581	58.167104	58.376485	18.822611	30.780706	30.891505	241	242	243
82	H	25.780697	60.777714	54.282821	13.642557	32.162181	28.725232	244	245	246
83	H	22.636915	62.322024	50.952781	11.978940	32.979395	26.963050	247	248	249
84	H	30.919210	62.486840	52.196296	16.361741	33.066612	27.621091	250	251	252
85	H	29.417692	60.348395	57.381488	15.567172	31.934995	30.364976	253	254	255
86	H	26.170961	53.006442	44.433472	13.849076	28.049801	23.513181	256	257	258
87	O	23.148267	53.755816	46.223953	12.249535	28.446353	24.460662	259	260	261
88	H	18.529113	55.104479	47.432430	9.805185	29.160035	25.100161	262	263	264
89	H	32.366792	54.915937	48.332551	17.127768	29.060262	25.576485	265	266	267
90	H	30.619239	54.242818	51.179446	16.203003	28.704063	27.082997	268	269	270

91 H 29.050285 55.370002 48.432718 15.372749 29.300543 25.629491 271 272
273

92 C 30.798221 55.494437 49.544577 16.297717 29.366391 26.217861 274 275
276

93 O 32.167609 59.833450 49.040816 17.022366 31.662498 25.951282 277 278
279

94 H 18.584568 59.217518 43.329473 9.834530 31.336561 22.928970 280 281
282

95 H 16.176528 58.780728 45.644313 8.560250 31.105421 24.153930 283 284
285

96 H 16.721707 56.427723 43.296810 8.848746 29.860265 22.911685 286 287
288

Number of elements of the density matrix on this node (used, total): 222269 1773786

Numerical Integration : Fuzzy Cells (Becke)

Becke grid quality: NORMAL

Lebedev angular grid order: min: 11 max: 23

Nr. of radial points: min: 46 max: 52

Total nr. of points: 600129

Nr. of blocks: 4689

Block length: 128

Nr. of dummy points: 63

Test of Precision of the Numerical Integration Grid

===

Integral of the Total Core Density: 85.99995648899211

Relative Error: -5.059E-07

Density Fitting (zlmFit)

ZlmFit Fit Quality: NORMAL

Max L-expansion: min: 6 max: 7

Nr. of radial interpolation points: min: 39 max: 48

S C F *** ScaSCF ***

Initial density matrix is taken from previous geometry

CYCLE 1

SCF Error: norm([F,P])= 0.223137344726, max([F,P])= 0.014340100366

orbitals (Q,E):

A :114...133 (2.00 -0.2570) (2.00 -0.2561) (2.00 -0.2476) (2.00 -0.2457)

(2.00 -0.2327) (2.00 -0.2306) (2.00 -0.2281) (2.00 -0.2218)

(2.00   -0.2042) (2.00   -0.1841) (0.00   -0.0786) (0.00   -0.0762)

(0.00   -0.0710) (0.00   -0.0505) (0.00   -0.0255) (0.00   -0.0200)

(0.00   -0.0172) (0.00   -0.0142) (0.00   -0.0104) (0.00   -0.0063)

CYCLE   2

SDIIS (wt 0.000): 0.0765 0.9235

A-DIIS (wt 1.000): 0.0000 1.0000

DIIS coefficients: 0.0000 1.0000

SCF Error: norm([F,P])=   0.056005934326, max([F,P])=   0.003940410332

orbitals (Q,E):

A :114...124   (2.00   -0.2446) (2.00   -0.2420) (2.00   -0.2331) (2.00   -0.2309)

(2.00   -0.2263) (2.00   -0.2194) (2.00   -0.2157) (2.00   -0.2131)

(2.00   -0.1921) (2.00   -0.1728) (0.00   -0.0685)

CYCLE   3

SDIIS (wt 0.000): 0.0087 0.6804 0.3109

A-DIIS (wt 1.000): 0.0000 0.7487 0.2513

DIIS coefficients: 0.0000 0.7487 0.2513

SCF Error: norm([F,P])=   0.116969050967, max([F,P])=   0.012211543742

orbitals (Q,E):

A :114...124   (2.00   -0.2477) (2.00   -0.2461) (2.00   -0.2357) (2.00   -0.2338)

(2.00   -0.2263) (2.00   -0.2202) (2.00   -0.2189) (2.00   -0.2144)

(2.00   -0.1926) (2.00   -0.1753) (0.00   -0.0690)

CYCLE   4

SDIIS (wt 0.977): 0.4236 0.1460 0.4304

A-DIIS (wt 0.023): 0.1418 0.0000 0.8582

DIIS coefficients: 0.4170 0.1426 0.4404

SCF Error: norm([F,P])=   0.026501440221, max([F,P])=   0.003310855548

orbitals (Q,E):

A :114...124 (2.00 -0.2485) (2.00 -0.2476) (2.00 -0.2377) (2.00 -0.2358)

(2.00 -0.2266) (2.00 -0.2212) (2.00 -0.2204) (2.00 -0.2152)

(2.00 -0.1953) (2.00 -0.1764) (0.00 -0.0696)

CYCLE 5

SDIIS (wt 0.997): 0.1858 0.0515 0.2818 0.4810

A-DIIS (wt 0.003): 0.0000 0.0000 0.0000 1.0000

DIIS coefficients: 0.1851 0.0513 0.2808 0.4828

SCF Error: norm([F,P])= 0.008410298838, max([F,P])= 0.001345148092

orbitals (Q,E):

A :114...124 (2.00 -0.2483) (2.00 -0.2475) (2.00 -0.2377) (2.00 -0.2358)

(2.00 -0.2267) (2.00 -0.2211) (2.00 -0.2204) (2.00 -0.2151)

(2.00 -0.1949) (2.00 -0.1763) (0.00 -0.0697)

CYCLE 6

SDIIS (wt 1.000): 0.0793 0.0193 0.1393 0.3276 0.4345

DIIS coefficients: 0.0793 0.0193 0.1393 0.3276 0.4345

SCF Error: norm([F,P])= 0.003158906097, max([F,P])= 0.000474288761

orbitals (Q,E):

A :114...124 (2.00 -0.2484) (2.00 -0.2475) (2.00 -0.2376) (2.00 -0.2358)

(2.00 -0.2266) (2.00 -0.2211) (2.00 -0.2203) (2.00 -0.2151)

(2.00 -0.1950) (2.00 -0.1763) (0.00 -0.0696)

CYCLE 7

SDIIS (wt 1.000): 0.0269 0.0058 0.0518 0.1483 0.2859 0.4812

DIIS coefficients: 0.0269 0.0058 0.0518 0.1483 0.2859 0.4812

SCF Error: norm([F,P])= 0.001023501071, max([F,P])= 0.000100826036

orbitals (Q,E):

A :114...124 (2.00 -0.2484) (2.00 -0.2475) (2.00 -0.2376) (2.00 -0.2358)

(2.00 -0.2266) (2.00 -0.2212) (2.00 -0.2204) (2.00 -0.2151)

(2.00 -0.1949) (2.00 -0.1763) (0.00 -0.0697)

CYCLE 8

SDIIS (wt 1.000): 0.0087 0.0015 0.0194 0.0674 0.1598 0.3764 0.3668

DIIS coefficients: 0.0087 0.0015 0.0194 0.0674 0.1598 0.3764 0.3668

SCF Error: norm([F,P])= 0.000589385275, max([F,P])= 0.000068129651

orbitals (Q,E):

A :114...124 (2.00 -0.2484) (2.00 -0.2475) (2.00 -0.2376) (2.00 -0.2358)

(2.00 -0.2266) (2.00 -0.2212) (2.00 -0.2204) (2.00 -0.2151)

(2.00 -0.1950) (2.00 -0.1763) (0.00 -0.0697)

CYCLE 9

SDIIS (wt 1.000): 0.0011 0.0000 0.0038 0.0191 0.0587 0.1756 0.2557 0.4860

DIIS coefficients: 0.0011 0.0000 0.0038 0.0191 0.0587 0.1756 0.2557 0.4860

SCF Error: norm([F,P])= 0.000173319053, max([F,P])= 0.000020532391

orbitals (Q,E):

A :114...124 (2.00 -0.2484) (2.00 -0.2475) (2.00 -0.2376) (2.00 -0.2358)

(2.00 -0.2266) (2.00 -0.2212) (2.00 -0.2204) (2.00 -0.2151)

(2.00 -0.1950) (2.00 -0.1763) (0.00 -0.0697)

CYCLE 10

SDIIS (wt 1.000): -0.0001 -0.0001 0.0006 0.0053 0.0205 0.0705 0.1231 0.3076 0.4727

DIIS coefficients: -0.0001 -0.0001 0.0006 0.0053 0.0205 0.0705 0.1231 0.3076 0.4727

SCF Error: norm([F,P])= 0.000053546550, max([F,P])= 0.000006562899

orbitals (Q,E):

A :114...124 (2.00 -0.2484) (2.00 -0.2475) (2.00 -0.2376) (2.00 -0.2358)

(2.00 -0.2266) (2.00 -0.2212) (2.00 -0.2204) (2.00 -0.2151)

(2.00 -0.1950) (2.00 -0.1763) (0.00 -0.0697)

CYCLE 11

SDIIS (wt 1.000): -0.0002 -0.0001 0.0000 0.0015 0.0079 0.0304 0.0588 0.1613 0.3202 0.4202

DIIS coefficients: -0.0002 -0.0001 0.0000 0.0015 0.0079 0.0304 0.0588 0.1613 0.3202 0.4202

SCF Error: norm([F,P])= 0.000020852319, max([F,P])= 0.000002534291

orbitals (Q,E):

A :114...124 (2.00 -0.2484) (2.00 -0.2475) (2.00 -0.2376) (2.00 -0.2358)

(2.00 -0.2266) (2.00 -0.2212) (2.00 -0.2204) (2.00 -0.2151)

(2.00 -0.1950) (2.00 -0.1763) (0.00 -0.0697)

CYCLE 12

SDIIS (wt 1.000): -0.0000 0.0001 0.0008 0.0032 0.0117 0.0236 0.0674 0.1547 0.3245 0.4140

DIIS coefficients: -0.0000 0.0001 0.0008 0.0032 0.0117 0.0236 0.0674 0.1547 0.3245 0.4140

SCF Error: norm([F,P])= 0.000010202376, max([F,P])= 0.000001629040

orbitals (Q,E):

A :114...124 (2.00 -0.2484) (2.00 -0.2475) (2.00 -0.2376) (2.00 -0.2358)

(2.00 -0.2266) (2.00 -0.2212) (2.00 -0.2204) (2.00 -0.2151)

(2.00 -0.1950) (2.00 -0.1763) (0.00 -0.0697)

CYCLE 13

SDIIS (wt 1.000): 0.0000 0.0001 0.0006 0.0027 0.0068 0.0222 0.0615 0.1708 0.2875 0.4478

DIIS coefficients: 0.0000 0.0001 0.0006 0.0027 0.0068 0.0222 0.0615 0.1708 0.2875 0.4478

SCF Error: norm([F,P])= 0.000003229974, max([F,P])= 0.000000443608

orbitals (Q,E):

A :114...124 (2.00 -0.2484) (2.00 -0.2475) (2.00 -0.2376) (2.00 -0.2358)
(2.00 -0.2266) (2.00 -0.2212) (2.00 -0.2204) (2.00 -0.2151)
(2.00 -0.1950) (2.00 -0.1763) (0.00 -0.0697)

SCF CONVERGED

CYCLE 14

*** Setting up for NEW gradients in Focky

*** Using FIT density in Focky

*** Setting up for NEW Hessian in Focky

orbitals (Q,E):

A :114...133 (2.00 -0.2484) (2.00 -0.2475) (2.00 -0.2376) (2.00 -0.2358)
(2.00 -0.2266) (2.00 -0.2212) (2.00 -0.2204) (2.00 -0.2151)
(2.00 -0.1950) (2.00 -0.1763) (0.00 -0.0697) (0.00 -0.0687)
(0.00 -0.0623) (0.00 -0.0399) (0.00 -0.0189) (0.00 -0.0127)
(0.00 -0.0108) (0.00 -0.0074) (0.00 -0.0046) (0.00 -0.0002)

Fit test: (difference of exact and fit density, squared integrated, result summed over spins)

SCF: 0.00100792875724

Gross Charges per Atom (Z minus electrons)

==

-0.6656	0.9209	0.5496	0.3301	-0.5791	-0.3819	-0.6314	0.6719	0.6257	0.8609
-0.6593	0.0007	-0.5775	0.5875	0.3243	-0.5893	0.5960	0.6004	-0.6022	0.2580
0.3399	0.0980	0.1652	0.7462	0.4173	0.4091	0.5604	0.5714	0.0177	0.6401
-0.5649	0.5475	0.5496	0.5733	0.6426	0.3732	0.7496	0.9834	0.5874	-0.2109
-0.1550	-0.2089	-0.2012	-0.2129	-0.2438	-0.2316	-0.1526	-0.1844	-0.1360	-0.2072
-0.2409	-0.2211	-0.2237	-0.1857	-0.2452	-0.1695	-0.1946	-0.1570	-0.1971	-0.2142
-0.2236	-0.2189	-0.2274	-0.1933	-0.1133	-0.2224	-0.1567	-0.2194	0.8243	-0.1964

-0.2183 -0.2173 -0.2101 -0.1906 -0.2122 -0.1793 -0.2270 -0.2335 -0.1993 -0.2402

-0.2118 -0.2513 -0.1998 -0.1572 -0.1677 0.2331 -0.6734 -0.1259 -0.1921 -0.2110

-0.0841 0.5059 -0.6160 -0.1749 -0.2149 -0.2372

Net Total: -0.00000000

M U L T I P O L E   D E R I V E D   C H A R G E   A N A L Y S I S

See: M. Swart, P.Th. van Duijnen, J.G. Snijders, J.Comput.Chem., (2001), p. 79-88.

Multipole derived atomic charges (a.u.)

Atom	Level:	MDC-m	MDC-d	MDC-q
1	O	-0.351518	-0.318147	-0.341574
2	C	0.363135	0.375594	0.396905
3	C	0.241992	0.249539	0.252226
4	C	0.180497	0.165802	0.210910
5	O	-0.303940	-0.546644	-0.610938
6	N	-0.214900	-0.276327	-0.466499
7	O	-0.380737	-0.384090	-0.450527
8	C	0.250921	0.309418	0.309128
9	C	0.228100	0.193156	0.188930
10	C	0.339572	0.410600	0.416235
11	O	-0.384414	-0.358503	-0.400588
12	C	0.124427	0.102334	0.101481

13	O	-0.314338	-0.299965	-0.345452
14	C	0.245564	0.242380	0.247832
15	C	0.196370	0.159118	0.174853
16	O	-0.369610	-0.477517	-0.482991
17	C	0.227789	0.193196	0.198593
18	C	0.229913	0.193061	0.195448
19	O	-0.318823	-0.321359	-0.373680
20	C	0.057833	0.046015	-0.057617
21	C	0.195296	0.221286	0.282190
22	C	0.118957	0.105193	0.108366
23	C	0.119836	0.099521	0.111880
24	C	0.290601	0.268053	0.285700
25	C	0.172656	0.134744	0.136995
26	C	0.164805	0.124971	0.164757
27	C	0.249308	0.238828	0.261689
28	C	0.234507	0.193761	0.303721
29	C	0.033795	0.027108	-0.043096
30	C	0.236658	0.191022	0.177275
31	O	-0.371842	-0.475579	-0.513071
32	C	0.254925	0.243294	0.263198
33	C	0.222052	0.185448	0.193238
34	C	0.228210	0.295681	0.378667
35	C	0.222057	0.192001	0.201289
36	C	0.180808	0.145456	0.149785
37	C	0.294034	0.271086	0.310753
38	C	0.368693	0.340226	0.389073
39	C	0.229555	0.189968	0.153185
40	H	-0.072791	-0.060374	-0.025971
41	H	-0.058882	-0.033229	-0.021195
42	H	-0.063971	-0.056497	-0.032846
43	H	-0.056669	-0.035437	-0.015435

44	H	-0.068806	-0.052516	-0.051207
45	H	-0.070484	-0.057632	-0.053760
46	H	-0.076524	-0.071881	-0.070141
47	H	-0.071802	-0.061543	-0.060921
48	H	-0.063548	-0.041429	-0.022021
49	H	-0.083305	-0.063496	-0.071840
50	H	-0.055417	-0.047002	-0.048881
51	H	-0.072510	-0.055242	-0.060272
52	H	-0.070461	-0.058235	-0.064486
53	H	-0.067697	-0.063681	-0.073896
54	H	-0.058439	-0.045723	-0.052456
55	H	-0.053992	-0.043894	-0.046376
56	H	-0.057079	-0.042132	-0.030402
57	H	-0.066673	-0.049105	-0.049170
58	H	-0.062276	-0.036946	-0.033128
59	H	-0.075001	-0.060149	-0.048961
60	H	-0.074547	-0.064910	-0.054635
61	H	-0.068797	-0.054910	-0.042197
62	H	-0.066709	-0.048223	-0.039124
63	H	-0.072424	-0.074960	-0.083553
64	H	-0.057887	-0.035678	-0.033919
65	H	-0.074308	-0.049156	-0.057383
66	H	-0.072335	-0.063472	-0.060351
67	H	-0.066818	-0.053553	-0.066103
68	H	-0.065406	-0.056952	-0.056668
69	C	0.353168	0.439052	0.459960
70	H	-0.064606	-0.054085	-0.082011
71	H	-0.068086	-0.067190	-0.097850
72	H	-0.063231	-0.048469	-0.074526
73	H	-0.080577	-0.062459	-0.072272
74	H	-0.072894	-0.059994	-0.063947

75	H	-0.069657	-0.069039	-0.073561
76	H	-0.076384	-0.046713	-0.039815
77	H	-0.066443	-0.054223	-0.039281
78	H	-0.065302	-0.060903	-0.029999
79	H	-0.083119	-0.064500	-0.047631
80	H	-0.066770	-0.058040	-0.052598
81	H	-0.067631	-0.066023	-0.055984
82	H	-0.070868	-0.044534	-0.037701
83	H	-0.062053	-0.026371	-0.019581
84	H	-0.046933	-0.001969	-0.013796
85	H	-0.074183	-0.060154	-0.041946
86	H	0.064542	0.264547	0.298550
87	O	-0.368680	-0.447430	-0.462546
88	H	-0.044840	0.001383	0.022770
89	H	-0.045384	-0.030379	-0.017204
90	H	-0.051888	-0.050107	-0.040119
91	H	-0.042295	-0.011035	-0.013109
92	C	0.241036	0.198013	0.186071
93	O	-0.363165	-0.464343	-0.492519
94	H	-0.061413	-0.053992	-0.049893
95	H	-0.060782	-0.047093	-0.038655
96	H	-0.068744	-0.065722	-0.061775

*** Using NEW gradient routines ***

Geometry CYCLE 15

==============

Energy gradients wrt nuclear displacements

==

Atom	Cartesian (a.u./angstrom)		
	X	Y	Z
1 O	0.000639	-0.005082	0.003371
2 C	-0.000383	0.000356	0.000395
3 C	-0.002037	0.000378	-0.003540
4 C	-0.000207	0.001460	-0.001008
5 O	0.004159	0.000555	-0.003131
6 N	0.000390	0.000249	0.000821
7 O	0.001663	0.001353	0.002112
8 C	0.001729	-0.001676	0.000950
9 C	-0.000630	-0.000004	-0.000554
10 C	-0.001417	0.004546	-0.000952
11 O	-0.002057	0.000139	0.001552
12 C	0.000476	-0.000743	0.000132
13 O	0.001627	0.000872	-0.000752
14 C	0.000988	-0.001199	-0.004569
15 C	0.000853	0.000983	0.001906
16 O	-0.002662	0.001611	0.001415
17 C	0.000481	0.001312	-0.000150
18 C	0.000081	-0.000422	0.000540
19 O	-0.007523	0.000944	0.001246
20 C	0.000568	-0.000438	-0.000810
21 C	0.000457	-0.000831	0.000158
22 C	0.001089	-0.000443	-0.000451
23 C	0.000931	-0.000228	-0.001364
24 C	0.000264	-0.000113	0.000812
25 C	-0.000663	-0.001511	0.000014
26 C	0.001166	-0.000354	0.000127
27 C	-0.001497	0.000315	0.000431
28 C	0.000135	-0.000118	-0.000487

29 C -0.002387 -0.000499 -0.001113

30 C -0.000447 -0.000353 0.000287

31 O 0.001252 0.001988 -0.002137

32 C -0.000426 -0.000435 0.000189

33 C -0.000943 -0.000022 -0.000411

34 C 0.001396 -0.000833 0.004692

35 C 0.000630 0.001323 -0.000793

36 C 0.000426 -0.001181 -0.001263

37 C 0.000835 -0.001253 0.000144

38 C -0.000157 0.000155 -0.000616

39 C 0.000413 -0.000039 -0.000319

40 H -0.000999 0.000509 0.001411

41 H 0.000400 -0.000456 0.001779

42 H -0.000265 -0.000726 0.000037

43 H 0.000422 0.000299 -0.000546

44 H 0.000254 0.000123 0.000107

45 H -0.000108 0.000151 -0.000408

46 H -0.000056 0.000084 -0.000259

47 H -0.000143 0.000180 0.000129

48 H -0.000145 0.000684 -0.000055

49 H -0.000353 -0.000824 0.000238

50 H 0.000176 0.000263 0.000077

51 H 0.000089 0.000228 0.000416

52 H 0.000120 0.000441 0.000118

53 H 0.000083 0.000330 -0.000175

54 H -0.000060 0.000013 0.000231

55 H -0.000416 -0.000209 -0.000261

56 H -0.000193 -0.000073 -0.000590

57 H 0.000060 -0.000508 -0.000177

58 H -0.000038 -0.000312 -0.000453

59 H -0.000318 0.000084 0.000216

60	H	-0.000399	0.000089	-0.000320
61	H	-0.000172	-0.000332	0.000008
62	H	0.000012	-0.000499	-0.000215
63	H	-0.000308	0.000097	-0.000113
64	H	0.000240	-0.000012	0.000109
65	H	0.000221	0.000038	-0.000817
66	H	-0.000014	0.000166	0.000563
67	H	0.000781	0.000739	0.001121
68	H	0.000319	0.000041	0.000182
69	C	0.004271	-0.002962	0.000871
70	H	0.000130	0.000355	-0.000201
71	H	0.000090	0.000142	0.000417
72	H	-0.000389	0.000106	-0.000155
73	H	-0.000268	-0.000148	0.000329
74	H	0.000204	0.000375	-0.000117
75	H	-0.000112	0.000178	0.000402
76	H	0.000558	-0.000164	-0.000405
77	H	0.000324	0.000286	-0.000090
78	H	0.000295	-0.000028	-0.000003
79	H	0.000155	0.000145	0.000696
80	H	-0.000064	-0.000214	0.000212
81	H	0.000321	-0.000000	0.000424
82	H	0.000050	-0.001154	-0.000056
83	H	-0.001170	-0.000900	-0.000679
84	H	0.000026	-0.000196	-0.000655
85	H	-0.000175	0.000081	0.000092
86	H	-0.000736	-0.001177	-0.000251
87	O	-0.001558	0.000551	0.004354
88	H	-0.000077	-0.000221	0.000142
89	H	0.000584	0.000224	-0.000121
90	H	-0.000151	-0.000112	0.000722

91 H -0.000243 -0.000477 -0.000181

92 C -0.000541 0.000634 -0.001735

93 O 0.000503 0.003098 -0.002914

94 H 0.000149 0.000230 -0.000248

95 H -0.000245 0.000144 0.000173

96 H -0.000335 -0.000166 -0.000256

----

dE(predicted): -0.67960E-03, dE(actual): 0.19871E-03, Trust radius: 0.195959, all in a.u.

Interpolation step length 0.131497216396013

Geometry Convergence after Step 15 (Hartree/Angstrom,Angstrom)

----

current energy -19.67447980 Hartree

energy change 0.00019871 0.00100000 T

constrained gradient max 0.00752265 0.00100000 F

constrained gradient rms 0.00118683 0.00066667 F

gradient max 0.00752265

gradient rms 0.00118683

cart. step max 0.13071726 0.01000000 F

cart. step rms 0.03953860 0.00666667 F

Coordinates (Cartesian)

======================

Atom bohr angstrom Geometric Variables

X Y Z X Y Z (0:frozen, *:LT par.)

----

1 O	26.599693	61.625071	50.450063	14.075951	32.610583	26.697024	1	2	3
2 C	27.061028	61.968834	53.028979	14.320079	32.792495	28.061727	4	5	6
3 C	29.849324	61.255186	53.479791	15.795582	32.414849	28.300287	7	8	9
4 C	30.565165	61.621953	56.286729	16.174389	32.608933	29.785654	10	11	12
5 O	27.214918	52.931110	43.035497	14.401515	28.009937	22.773404	13	14	15
6 N	33.184436	60.996493	57.005447	17.560447	32.277954	30.165983	16	17	18
7 O	26.665227	64.576856	53.670326	14.110630	34.172601	28.401113	19	20	21
8 C	20.479430	58.454828	48.389640	10.837248	30.932963	25.606695	22	23	24
9 C	25.651563	65.403558	41.482256	13.574223	34.610073	21.951465	25	26	27
10 C	21.625878	55.046661	44.993008	11.443922	29.129438	23.809274	28	29	30
11 O	21.693678	62.702103	45.708780	11.479800	33.180524	24.188045	31	32	33
12 C	19.545943	56.561993	46.298095	10.343267	29.931318	24.499897	34	35	36
13 O	21.533517	55.233012	42.459684	11.395046	29.228051	22.468697	37	38	39
14 C	24.013372	61.521156	49.543519	12.707329	32.555594	26.217301	40	41	42
15 C	24.008454	63.235682	47.116417	12.704727	33.462882	24.932934	43	44	45
16 O	18.866670	59.517290	49.653249	9.983812	31.495193	26.275368	46	47	48
17 C	23.817534	56.951008	51.273852	12.603696	30.137176	27.132954	49	50	51
18 C	27.327796	53.209923	38.535401	14.461247	28.157479	20.392056	52	53	54
19 O	30.151827	58.607037	52.750727	15.955660	31.013508	27.914483	55	56	57
20 C	26.303919	57.092821	41.052015	13.919435	30.212220	21.723791	58	59	60
21 C	26.016032	54.214404	40.950893	13.767091	28.689027	21.670279	61	62	63
22 C	23.287325	58.729872	48.999003	12.323122	31.078510	25.929156	64	65	66
23 C	25.906895	62.689897	42.474074	13.709338	33.174065	22.476312	67	68	69
24 C	35.164913	62.027202	55.351772	18.608471	32.823382	29.290897	70	71	72
25 C	26.273749	62.454129	45.381621	13.903469	33.049302	24.014919	73	74	75
26 C	29.886456	64.376001	57.015594	15.815231	34.066313	30.171353	76	77	78
27 C	27.134879	65.048925	56.344055	14.359159	34.422409	29.815990	79	80	81
28 C	26.481666	67.817196	56.817590	14.013494	35.887315	30.066574	82	83	84
29 C	28.358611	58.470531	41.648592	15.006731	30.941273	22.039486	85	86	87
30 C	17.635861	57.840385	44.506061	9.332496	30.607813	23.551593	88	89	90
31 O	29.982739	62.476411	40.398809	15.866182	33.061093	21.378129	91	92	93

32 C	23.124485	53.562817	40.808245	12.236951	28.344222	21.594793	94	95	96
33 C	30.925707	57.511226	42.459217	16.365179	30.433630	22.468450	97	98	99
34 C	28.213815	61.321748	41.349086	14.930108	32.450071	21.880994	100	101	102
35 C	24.253503	66.018171	47.935549	12.834401	34.935312	25.366400	103	104	105
36 C	22.442525	50.778910	41.223154	11.876073	26.871042	21.814354	106	107	108
37 C	33.626241	58.316206	57.583087	17.794240	30.859607	30.471658	109	110	111
38 C	19.470752	64.131424	46.358764	10.303478	33.936888	24.532002	112	113	114
39 C	19.594993	50.233330	41.001228	10.369224	26.582333	21.696915	115	116	117
40 H	24.620849	58.114734	40.455914	13.028792	30.752993	21.408348	118	119	120
41 H	31.436434	58.339856	44.295837	16.635444	30.872122	23.440348	121	122	123
42 H	30.985747	55.460078	42.620378	16.396951	29.348210	22.553733	124	125	126
43 H	32.352826	58.173759	41.109100	17.120378	30.784227	21.753999	127	128	129
44 H	27.233506	66.581409	42.113405	14.411351	35.233364	22.285454	130	131	132
45 H	25.650985	65.426468	39.410689	13.573917	34.622196	20.855239	133	134	135
46 H	23.879865	66.242634	42.142968	12.636680	35.054092	22.301098	136	137	138
47 H	24.194656	61.626623	41.996073	12.803260	32.611405	22.223365	139	140	141
48 H	27.932573	63.553372	45.948295	14.781281	33.630996	24.314790	142	143	144
49 H	26.765039	60.487919	45.781445	14.163449	32.008828	24.226497	145	146	147
50 H	26.021314	66.249623	48.976713	13.769887	35.057791	25.917360	148	149	150

51	H	22.722930	66.586821	49.206246	12.024457	35.236228	26.038824	151	152	153
52	H	24.275861	67.275397	46.296932	12.846233	35.600607	24.499281	154	155	156
53	H	19.695692	66.141327	45.896834	10.422511	35.000483	24.287559	157	158	159
54	H	18.956611	63.917364	48.358046	10.031406	33.823612	25.589976	160	161	162
55	H	17.946698	63.345397	45.207995	9.496983	33.520940	23.923041	163	164	165
56	H	22.446242	54.170860	38.947797	11.878040	28.665985	20.610287	166	167	168
57	H	23.473370	49.688821	39.792964	12.421572	26.294192	21.057530	169	170	171
58	H	23.156550	50.154764	43.058479	12.253918	26.540758	22.785566	172	173	174
59	H	18.516968	51.223517	42.467567	9.798757	27.106318	22.472868	175	176	177
60	H	18.845859	50.829117	39.160040	9.972799	26.897610	20.722601	178	179	180
61	H	19.231719	48.205082	41.212533	10.176988	25.509031	21.808733	181	182	183
62	H	27.114387	51.157760	38.380384	14.348316	27.071521	20.310025	184	185	186
63	H	26.561249	54.114436	36.838258	14.055607	28.636126	19.493967	187	188	189
64	H	29.344314	53.646905	38.647742	15.528342	28.388719	20.451504	190	191	192
65	H	24.413424	58.063365	47.402474	12.919028	30.725809	25.084309	193	194	195
66	H	22.699931	57.531173	52.919334	12.012286	30.444185	28.003705	196	197	198
67	H	25.819660	56.994436	51.765782	13.663176	30.160156	27.393272	199	200	201
68	H	23.313676	55.008848	50.777969	12.337066	29.109429	26.870544	202	203	204
69	C	31.240653	58.094217	50.434658	16.531842	30.742136	26.688872	205	206	207

70 H 27.701150 69.046414 55.679335 14.658817 36.537789 29.464235 208 209
210

71 H 26.748058 68.288787 58.817759 14.154463 36.136870 31.125018 211 212
213

72 H 24.510959 68.192337 56.305275 12.970641 36.085831 29.795468 214 215
216

73 H 25.849340 63.831420 57.456739 13.678882 33.778133 30.404797 217 218
219

74 H 31.137902 65.703579 56.027258 16.477468 34.768836 29.648348 220 221
222

75 H 30.202857 64.614212 59.050093 15.982664 34.192369 31.247963 223 224
225

76 H 35.284811 61.075981 53.493675 18.671918 32.320017 28.307633 226 227
228

77 H 34.853405 64.045117 55.018552 18.443628 33.891216 29.114564 229 230
231

78 H 36.998736 61.824805 56.296369 19.578888 32.716278 29.790756 232 233
234

79 H 33.639864 57.081849 55.897340 17.801449 30.206414 29.579599 235 236
237

80 H 32.160320 57.627015 58.873656 17.018509 30.494903 31.154597 238 239
240

81 H 35.457748 58.128763 58.538839 18.763432 30.760417 30.977419 241 242
243

82 H 25.801085 60.786080 54.206120 13.653346 32.166608 28.684644 244 245
246

83 H 22.716083 62.311440 50.954658 12.020834 32.973794 26.964044 247 248
249

84 H 31.002357 62.440572 52.241068 16.405741 33.042128 27.644783 250 251
252

85 H 29.347446 60.359077 57.400991 15.530000 31.940648 30.375296 253 254
255

86 H 26.096967 53.147993 44.502571 13.809920 28.124707 23.549746 256 257
258

87 O 23.151695 53.746205 46.168428 12.251349 28.441267 24.431280 259 260
261

88 H 18.546074 55.126271 47.427469 9.814160 29.171566 25.097536 262 263
264

89 H 32.393645 54.707249 48.540641 17.141978 28.949830 25.686601 265 266
267

90 H 30.629307 54.150952 51.401157 16.208331 28.655450 27.200321 268 269
270

91 H 29.085760 55.230944 48.622401 15.391521 29.226957 25.729867 271 272
273

92 C 30.834845 55.351230 49.732269 16.317097 29.290609 26.317183 274 275
276

93 O 32.292394 59.647248 49.115277 17.088399 31.563964 25.990686 277 278
279

94 H 18.592983 59.225268 43.307787 9.838983 31.340662 22.917494 280 281
282

95 H 16.189867 58.797410 45.629721 8.567309 31.114250 24.146208 283 284
285

96 H 16.728041 56.438065 43.286871 8.852098 29.865738 22.906426 286 287
288

Number of elements of the density matrix on this node (used, total): 222269 1773786

Numerical Integration : Fuzzy Cells (Becke)

Becke grid quality: NORMAL

Lebedev angular grid order: min: 11 max: 23

Nr. of radial points: min: 46 max: 52

Total nr. of points: 600116

Nr. of blocks: 4689

Block length: 128

Nr. of dummy points: 76

Test of Precision of the Numerical Integration Grid

===

Integral of the Total Core Density: 85.99993816330027

Relative Error: -7.190E-07

Density Fitting (zlmFit)

ZlmFit Fit Quality: NORMAL

Max L-expansion: min: 6 max: 7

Nr. of radial interpolation points: min: 39 max: 48

S C F *** ScaSCF ***

Initial density matrix is taken from previous geometry

CYCLE 1

SCF Error: norm([F,P])= 0.154598075914, max([F,P])= 0.012069016103

orbitals (Q,E):

A :114...133 (2.00 -0.2504) (2.00 -0.2482) (2.00 -0.2397) (2.00 -0.2370)

(2.00 -0.2307) (2.00 -0.2231) (2.00 -0.2221) (2.00 -0.2169)

(2.00 -0.1967) (2.00 -0.1794) (0.00 -0.0708) (0.00 -0.0699)

(0.00 -0.0638) (0.00 -0.0417) (0.00 -0.0200) (0.00 -0.0153)

(0.00 -0.0132) (0.00 -0.0092) (0.00 -0.0067) (0.00 -0.0018)

CYCLE 2

SDIIS (wt 0.000): 0.0547 0.9453

A-DIIS (wt 1.000): 0.0000 1.0000

DIIS coefficients: 0.0000 1.0000

SCF Error: norm([F,P])= 0.032644967433, max([F,P])= 0.002524113544

orbitals (Q,E):

A :114...124 (2.00 -0.2489) (2.00 -0.2489) (2.00 -0.2359) (2.00 -0.2331)

(2.00 -0.2255) (2.00 -0.2222) (2.00 -0.2184) (2.00 -0.2151)

(2.00 -0.1948) (2.00 -0.1757) (0.00 -0.0695)

CYCLE 3

SDIIS (wt 0.000): 0.0038 0.6459 0.3502

A-DIIS (wt 1.000): 0.0000 0.7059 0.2941

DIIS coefficients: 0.0000 0.7059 0.2941

SCF Error: norm([F,P])= 0.058258784257, max([F,P])= 0.005757431689

orbitals (Q,E):

A :114...124 (2.00 -0.2483) (2.00 -0.2476) (2.00 -0.2366) (2.00 -0.2343)

(2.00 -0.2271) (2.00 -0.2216) (2.00 -0.2195) (2.00 -0.2155)

(2.00 -0.1946) (2.00 -0.1768) (0.00 -0.0691)

CYCLE 4

SDIIS (wt 0.993): 0.3761 0.1664 0.4575

A-DIIS (wt 0.007): 0.0103 0.0000 0.9897

DIIS coefficients: 0.3737 0.1653 0.4610

SCF Error: norm([F,P])= 0.012019251772, max([F,P])= 0.001650230207

orbitals (Q,E):

A :114...124 (2.00 -0.2486) (2.00 -0.2475) (2.00 -0.2371) (2.00 -0.2347)

(2.00 -0.2275) (2.00 -0.2216) (2.00 -0.2199) (2.00 -0.2151)

(2.00 -0.1949) (2.00 -0.1770) (0.00 -0.0690)

CYCLE 5

SDIIS (wt 0.996): 0.1809 0.0666 0.3453 0.4073

A-DIIS (wt 0.004): 0.0000 0.0000 0.1730 0.8270

DIIS coefficients: 0.1802 0.0663 0.3446 0.4090

SCF Error: norm([F,P])= 0.006350107439, max([F,P])= 0.001402151760

orbitals (Q,E):

A :114...124 (2.00 -0.2486) (2.00 -0.2475) (2.00 -0.2370) (2.00 -0.2345)

(2.00 -0.2275) (2.00 -0.2217) (2.00 -0.2198) (2.00 -0.2153)

(2.00 -0.1949) (2.00 -0.1769) (0.00 -0.0690)

CYCLE 6

SDIIS (wt 1.000): 0.0712 0.0214 0.1727 0.2730 0.4617

DIIS coefficients: 0.0712 0.0214 0.1727 0.2730 0.4617

SCF Error: norm([F,P])= 0.001833412590, max([F,P])= 0.000228325089

orbitals (Q,E):

A :114...124 (2.00 -0.2485) (2.00 -0.2475) (2.00 -0.2370) (2.00 -0.2345)

(2.00 -0.2275) (2.00 -0.2216) (2.00 -0.2198) (2.00 -0.2153)

(2.00 -0.1948) (2.00 -0.1769) (0.00 -0.0690)

CYCLE 7

SDIIS (wt 1.000): 0.0198 0.0039 0.0616 0.1169 0.3079 0.4899

DIIS coefficients: 0.0198 0.0039 0.0616 0.1169 0.3079 0.4899

SCF Error: norm([F,P])= 0.000662362088, max([F,P])= 0.000064316910

orbitals (Q,E):

A :114...124 (2.00 -0.2485) (2.00 -0.2475) (2.00 -0.2370) (2.00 -0.2345)

(2.00 -0.2275) (2.00 -0.2216) (2.00 -0.2198) (2.00 -0.2153)

(2.00 -0.1949) (2.00 -0.1769) (0.00 -0.0690)

CYCLE 8

SDIIS (wt 1.000): 0.0072 0.0008 0.0270 0.0557 0.1702 0.3368 0.4023

DIIS coefficients: 0.0072 0.0008 0.0270 0.0557 0.1702 0.3368 0.4023

SCF Error: norm([F,P])= 0.000256220659, max([F,P])= 0.000035838867

orbitals (Q,E):

A :114...124 (2.00 -0.2485) (2.00 -0.2475) (2.00 -0.2370) (2.00 -0.2345)

(2.00 -0.2275) (2.00 -0.2216) (2.00 -0.2198) (2.00 -0.2153)

(2.00 -0.1948) (2.00 -0.1769) (0.00 -0.0690)

CYCLE 9

SDIIS (wt 1.000): 0.0009 -0.0003 0.0067 0.0167 0.0654 0.1587 0.3316 0.4203

DIIS coefficients: 0.0009 -0.0003 0.0067 0.0167 0.0654 0.1587 0.3316 0.4203

SCF Error: norm([F,P])= 0.000135274182, max([F,P])= 0.000021148202

orbitals (Q,E):

A :114...124 (2.00 -0.2485) (2.00 -0.2475) (2.00 -0.2370) (2.00 -0.2345)

(2.00 -0.2275) (2.00 -0.2216) (2.00 -0.2198) (2.00 -0.2153)

(2.00 -0.1949) (2.00 -0.1769) (0.00 -0.0690)

CYCLE 10

SDIIS (wt 1.000): -0.0001 -0.0002 0.0015 0.0047 0.0233 0.0636 0.1661 0.2644 0.4768

DIIS coefficients: -0.0001 -0.0002 0.0015 0.0047 0.0233 0.0636 0.1661 0.2644 0.4768

SCF Error: norm([F,P])= 0.000031864591, max([F,P])= 0.000003405836

orbitals (Q,E):

A :114...124 (2.00 -0.2485) (2.00 -0.2475) (2.00 -0.2370) (2.00 -0.2345)

(2.00 -0.2275) (2.00 -0.2216) (2.00 -0.2198) (2.00 -0.2153)

(2.00 -0.1949) (2.00 -0.1769) (0.00 -0.0690)

CYCLE 11

SDIIS (wt 1.000): -0.0002 -0.0001 0.0001 0.0009 0.0067 0.0205 0.0630 0.1131 0.2833 0.5126

DIIS coefficients: -0.0002 -0.0001 0.0001 0.0009 0.0067 0.0205 0.0630 0.1131 0.2833 0.5126

SCF Error: norm([F,P])= 0.000009479031, max([F,P])= 0.000001045770

orbitals (Q,E):

A :114...124 (2.00 -0.2485) (2.00 -0.2475) (2.00 -0.2370) (2.00 -0.2345)

(2.00 -0.2275) (2.00 -0.2216) (2.00 -0.2198) (2.00 -0.2153)

(2.00 -0.1949) (2.00 -0.1769) (0.00 -0.0690)

CYCLE 12

SDIIS (wt 1.000): -0.0000 0.0002 0.0007 0.0033 0.0094 0.0288 0.0540 0.1506 0.3720 0.3810

DIIS coefficients: -0.0000 0.0002 0.0007 0.0033 0.0094 0.0288 0.0540 0.1506 0.3720 0.3810

SCF Error: norm([F,P])= 0.000005046167, max([F,P])= 0.000000545629

orbitals (Q,E):

A :114...124 (2.00 -0.2485) (2.00 -0.2475) (2.00 -0.2370) (2.00 -0.2345)

(2.00 -0.2275) (2.00 -0.2216) (2.00 -0.2198) (2.00 -0.2153)

(2.00 -0.1949) (2.00 -0.1769) (0.00 -0.0690)

SCF CONVERGED

CYCLE 13

*** Setting up for NEW gradients in Focky

*** Using FIT density in Focky

*** Setting up for NEW Hessian in Focky

orbitals (Q,E):

A :114...133 (2.00 -0.2485) (2.00 -0.2475) (2.00 -0.2370) (2.00 -0.2345)

(2.00 -0.2275) (2.00 -0.2216) (2.00 -0.2198) (2.00 -0.2153)

(2.00 -0.1949) (2.00 -0.1769) (0.00 -0.0690) (0.00 -0.0684)

(0.00 -0.0632) (0.00 -0.0392) (0.00 -0.0183) (0.00 -0.0131)

(0.00 -0.0110) (0.00 -0.0075) (0.00 -0.0047) (0.00 -0.0001)

Fit test: (difference of exact and fit density, squared integrated, result summed over spins)

SCF: 0.00100910613715

Gross Charges per Atom (Z minus electrons)

==

-0.6651	0.9256	0.5451	0.3333	-0.5817	-0.3819	-0.6320	0.6748	0.6251	0.8679
-0.6596	-0.0010	-0.5769	0.5874	0.3258	-0.5917	0.5970	0.6007	-0.6021	0.2578
0.3380	0.0973	0.1683	0.7467	0.4133	0.4080	0.5611	0.5726	0.0209	0.6413
-0.5654	0.5509	0.5516	0.5713	0.6438	0.3712	0.7504	0.9867	0.5878	-0.2117
-0.1601	-0.2116	-0.2006	-0.2129	-0.2437	-0.2319	-0.1506	-0.1866	-0.1338	-0.2067
-0.2407	-0.2224	-0.2260	-0.1835	-0.2464	-0.1725	-0.1964	-0.1529	-0.1978	-0.2145
-0.2239	-0.2197	-0.2275	-0.1933	-0.1166	-0.2240	-0.1555	-0.2173	0.8231	-0.1969
-0.2185	-0.2173	-0.2103	-0.1903	-0.2117	-0.1793	-0.2265	-0.2344	-0.1980	-0.2409
-0.2128	-0.2525	-0.1994	-0.1604	-0.1666	0.2366	-0.6748	-0.1263	-0.1944	-0.2073
-0.0835	0.5048	-0.6166	-0.1746	-0.2168	-0.2373				

Net Total: -0.00000000

M U L T I P O L E   D E R I V E D   C H A R G E   A N A L Y S I S

See: M. Swart, P.Th. van Duijnen, J.G. Snijders, J.Comput.Chem., (2001), p. 79-88.

Multipole derived atomic charges (a.u.)

Atom		Level: MDC-m	MDC-d	MDC-q
1	O	-0.351280	-0.325768	-0.349200
2	C	0.362809	0.374853	0.396475
3	C	0.242910	0.250517	0.254146
4	C	0.180680	0.166370	0.211531
5	O	-0.309168	-0.551607	-0.615028
6	N	-0.214194	-0.276914	-0.468041
7	O	-0.379450	-0.379779	-0.445627
8	C	0.250388	0.309602	0.308533
9	C	0.228038	0.192973	0.187716
10	C	0.339443	0.408622	0.414877
11	O	-0.382523	-0.356160	-0.397448
12	C	0.124170	0.102086	0.102214
13	O	-0.310620	-0.298324	-0.344552
14	C	0.245363	0.243927	0.249013
15	C	0.195816	0.159205	0.175298
16	O	-0.369580	-0.478411	-0.483584
17	C	0.227560	0.192726	0.197970
18	C	0.229675	0.193078	0.194732
19	O	-0.319636	-0.320087	-0.375802

20	C	0.057655	0.045355	-0.057513
21	C	0.195002	0.221635	0.283923
22	C	0.118573	0.104810	0.107644
23	C	0.119810	0.099698	0.111085
24	C	0.290605	0.268159	0.286420
25	C	0.172692	0.135050	0.137128
26	C	0.164664	0.124603	0.163896
27	C	0.248864	0.238074	0.260771
28	C	0.234493	0.193772	0.301385
29	C	0.034187	0.026944	-0.044816
30	C	0.237188	0.191363	0.177859
31	O	-0.372103	-0.476587	-0.515116
32	C	0.253801	0.243965	0.264154
33	C	0.223493	0.186082	0.189992
34	C	0.227651	0.295306	0.380879
35	C	0.221782	0.192032	0.201749
36	C	0.180109	0.145701	0.150738
37	C	0.293893	0.271002	0.312151
38	C	0.368143	0.339820	0.387297
39	C	0.229329	0.190023	0.159611
40	H	-0.073552	-0.060909	-0.027206
41	H	-0.057302	-0.030920	-0.018058
42	H	-0.063665	-0.056548	-0.031328
43	H	-0.056360	-0.034619	-0.014192
44	H	-0.068782	-0.052605	-0.051089
45	H	-0.070479	-0.057526	-0.053071
46	H	-0.076496	-0.071835	-0.069922
47	H	-0.071786	-0.061616	-0.061313
48	H	-0.063953	-0.042090	-0.022551
49	H	-0.082901	-0.062820	-0.070899
50	H	-0.055118	-0.047304	-0.049000

51	H	-0.072612	-0.055005	-0.059975
52	H	-0.070984	-0.059413	-0.065829
53	H	-0.067789	-0.064034	-0.073255
54	H	-0.057778	-0.044340	-0.051892
55	H	-0.054260	-0.044058	-0.045872
56	H	-0.057515	-0.042955	-0.031289
57	H	-0.067321	-0.050637	-0.051371
58	H	-0.061578	-0.035894	-0.033084
59	H	-0.075565	-0.061018	-0.051014
60	H	-0.074836	-0.065398	-0.056905
61	H	-0.068860	-0.055077	-0.044134
62	H	-0.067127	-0.048704	-0.040016
63	H	-0.072605	-0.075148	-0.083360
64	H	-0.058132	-0.035863	-0.033892
65	H	-0.074606	-0.049199	-0.057375
66	H	-0.072592	-0.064438	-0.061078
67	H	-0.067462	-0.053949	-0.066170
68	H	-0.065284	-0.056850	-0.056436
69	C	0.352612	0.440018	0.464178
70	H	-0.064649	-0.054186	-0.081566
71	H	-0.068151	-0.067283	-0.097454
72	H	-0.063306	-0.048461	-0.073580
73	H	-0.080860	-0.062641	-0.073071
74	H	-0.072875	-0.060096	-0.064142
75	H	-0.069427	-0.068783	-0.072388
76	H	-0.076135	-0.046189	-0.038792
77	H	-0.066525	-0.054352	-0.039715
78	H	-0.065015	-0.060592	-0.029535
79	H	-0.082950	-0.064386	-0.047754
80	H	-0.066789	-0.058198	-0.052730
81	H	-0.067700	-0.066296	-0.056591

82	H	-0.070930	-0.044093	-0.037803
83	H	-0.061710	-0.024676	-0.018347
84	H	-0.047011	-0.002027	-0.015167
85	H	-0.073681	-0.059890	-0.041147
86	H	0.060560	0.261641	0.295891
87	O	-0.360181	-0.436874	-0.453296
88	H	-0.044960	0.000448	0.020808
89	H	-0.045640	-0.030922	-0.018691
90	H	-0.052297	-0.050844	-0.042563
91	H	-0.041997	-0.010326	-0.012987
92	C	0.240962	0.197812	0.189609
93	O	-0.364118	-0.465625	-0.494244
94	H	-0.060959	-0.053242	-0.049358
95	H	-0.060860	-0.047627	-0.039305
96	H	-0.068343	-0.065254	-0.061149

*** Using NEW gradient routines ***

Geometry CYCLE 16

==============

Energy gradients wrt nuclear displacements

==

Atom		Cartesian (a.u./angstrom) X	Y	Z
1	O	-0.000947	0.000487	-0.001661
2	C	0.001525	-0.000905	0.001660
3	C	-0.000677	0.001509	0.002455
4	C	0.001243	-0.000330	-0.000591

5	O	-0.003824	-0.000958	0.001728
6	N	-0.000252	0.000057	0.000422
7	O	-0.000077	0.000479	-0.001707
8	C	-0.001248	-0.000958	0.002052
9	C	-0.000140	0.000149	-0.000541
10	C	0.003951	0.000044	0.000275
11	O	-0.001420	-0.002188	0.001607
12	C	-0.000135	0.001589	-0.000488
13	O	-0.001705	-0.000186	-0.001635
14	C	-0.001429	0.002802	0.000364
15	C	0.000414	-0.000307	-0.000342
16	O	0.001833	0.001132	-0.002251
17	C	0.000592	0.000265	-0.000519
18	C	-0.000011	0.000068	-0.000556
19	O	-0.002596	0.000631	-0.002663
20	C	-0.000370	-0.000611	-0.001609
21	C	-0.001307	0.000364	-0.000107
22	C	0.001143	0.000247	-0.001177
23	C	0.000857	-0.000408	-0.000934
24	C	-0.000289	-0.000320	0.000312
25	C	-0.000166	-0.000341	-0.001133
26	C	0.000661	-0.000851	0.000100
27	C	0.000040	-0.000702	-0.000405
28	C	0.000091	-0.000265	-0.000432
29	C	0.000552	0.001862	0.001287
30	C	-0.000499	-0.000486	-0.000321
31	O	-0.000501	-0.000999	0.000899
32	C	0.000156	0.000138	0.002522
33	C	0.000770	-0.000735	-0.000967
34	C	0.000668	0.000806	-0.000044
35	C	0.000196	-0.000487	0.000539

36	C	-0.000855	0.001656	0.000328
37	C	-0.000015	0.000357	0.000184
38	C	0.001377	-0.000871	0.000662
39	C	0.000282	-0.000148	0.000178
40	H	-0.000387	-0.000171	0.000621
41	H	0.000044	0.000407	0.000107
42	H	0.000738	0.000948	0.000150
43	H	0.000201	0.000441	0.000082
44	H	0.000171	0.000266	0.000031
45	H	-0.000024	0.000113	-0.000289
46	H	-0.000005	-0.000036	-0.000209
47	H	0.000093	-0.000180	0.000262
48	H	0.000340	-0.000118	0.000038
49	H	0.000257	-0.000799	-0.000109
50	H	-0.000026	0.000503	0.000343
51	H	-0.000394	-0.000090	0.000453
52	H	0.000049	-0.000387	0.000199
53	H	0.000039	0.000570	0.000217
54	H	-0.000033	-0.000284	0.001097
55	H	-0.000301	-0.000152	-0.000318
56	H	-0.000034	-0.000022	-0.000059
57	H	0.000316	0.000036	0.000008
58	H	0.000231	0.000259	0.001167
59	H	-0.000133	0.000223	0.000250
60	H	-0.000032	0.000014	-0.000222
61	H	-0.000010	-0.000259	0.000012
62	H	-0.000016	-0.000005	-0.000012
63	H	0.000049	0.000004	-0.000278
64	H	0.000289	0.000008	-0.000059
65	H	-0.000603	-0.000687	-0.000130
66	H	-0.000121	-0.000012	0.000359

67	H	-0.000102	0.000147	-0.000038
68	H	-0.000220	-0.000375	-0.000362
69	C	0.006861	-0.000302	0.001802
70	H	0.000179	0.000212	-0.000194
71	H	0.000037	0.000008	0.000258
72	H	-0.000344	0.000077	-0.000100
73	H	0.000014	-0.000062	0.000180
74	H	0.000105	0.000281	-0.000120
75	H	0.000141	0.000064	0.000392
76	H	0.000121	0.000127	-0.000256
77	H	-0.000085	0.000126	-0.000028
78	H	0.000481	0.000025	0.000157
79	H	-0.000008	0.000002	0.000023
80	H	-0.000094	-0.000073	0.000078
81	H	0.000333	0.000104	0.000260
82	H	-0.000588	-0.000305	0.000617
83	H	-0.001259	0.000044	-0.000302
84	H	-0.000758	0.000597	0.000474
85	H	-0.000473	0.000030	0.000308
86	H	-0.000161	0.000193	-0.000220
87	O	0.000829	-0.001850	-0.002782
88	H	0.000314	-0.000370	0.000239
89	H	0.000547	0.000076	0.000132
90	H	-0.000157	0.000126	0.000296
91	H	-0.000264	-0.000286	0.000069
92	C	-0.002082	0.000597	-0.000410
93	O	-0.001981	-0.001371	-0.000900
94	H	0.000223	0.000028	-0.000338
95	H	-0.000052	0.000090	0.000005
96	H	-0.000142	-0.000134	-0.000438

----

dE(predicted): -0.36023E-03, dE(actual): -0.27159E-03, Trust radius: 0.195959, all in a.u.

Geometry Convergence after Step 16 (Hartree/Angstrom,Angstrom)

----

current energy	-19.67475139 Hartree		
energy change	-0.00027159	0.00100000	T
constrained gradient max	0.00686056	0.00100000	F
constrained gradient rms	0.00092597	0.00066667	F
gradient max	0.00686056		
gradient rms	0.00092597		
cart. step max	0.10494221	0.01000000	F
cart. step rms	0.02158310	0.00666667	F

Coordinates (Cartesian)

======================

Atom	bohr X	bohr Y	bohr Z	angstrom X	angstrom Y	angstrom Z	Geometric Variables (0:frozen, *:LT par.)		
1 O	26.575792	61.645313	50.468236	14.063304	32.621295	26.706641	1	2	3
2 C	27.044350	61.975766	53.045509	14.311254	32.796163	28.070475	4	5	6
3 C	29.832348	61.238800	53.467104	15.786599	32.406178	28.293573	7	8	9
4 C	30.582664	61.593789	56.267951	16.183649	32.594029	29.775718	10	11	12
5 O	27.284443	52.938874	43.061862	14.438306	28.014046	22.787356	13	14	15
6 N	33.210868	60.959934	56.953442	17.574435	32.258608	30.138464	16	17	18
7 O	26.665239	64.580914	53.713031	14.110637	34.174748	28.423712	19	20	21
8 C	20.459016	58.481069	48.387549	10.826445	30.946849	25.605588	22	23	24

9 C	25.672571	65.416424	41.504426	13.585339	34.616881	21.963196	25	26	27
10 C	21.621385	55.025833	45.037006	11.441544	29.118417	23.832557	28	29	30
11 O	21.712588	62.730933	45.680139	11.489807	33.195780	24.172889	31	32	33
12 C	19.536917	56.566923	46.307421	10.338491	29.933927	24.504832	34	35	36
13 O	21.564871	55.193135	42.499409	11.411638	29.206949	22.489719	37	38	39
14 C	23.993929	61.550948	49.548972	12.697040	32.571359	26.220187	40	41	42
15 C	24.010503	63.263793	47.117544	12.705811	33.477757	24.933530	43	44	45
16 O	18.835050	59.542471	49.641919	9.967079	31.508519	26.269372	46	47	48
17 C	23.775153	56.998654	51.310311	12.581269	30.162389	27.152247	49	50	51
18 C	27.372714	53.206894	38.566280	14.485017	28.155876	20.408397	52	53	54
19 O	30.104948	58.590792	52.727783	15.930852	31.004912	27.902341	55	56	57
20 C	26.322518	57.088237	41.076110	13.929277	30.209794	21.736541	58	59	60
21 C	26.059248	54.207112	40.982152	13.789960	28.685168	21.686821	61	62	63
22 C	23.263098	58.759480	49.016385	12.310302	31.094178	25.938354	64	65	66
23 C	25.921758	62.700782	42.492233	13.717203	33.179825	22.485921	67	68	69
24 C	35.174476	62.004547	55.287819	18.613531	32.811393	29.257054	70	71	72
25 C	26.286163	62.470742	45.400766	13.910038	33.058093	24.025051	73	74	75
26 C	29.919006	64.346556	57.021218	15.832456	34.050731	30.174329	76	77	78
27 C	27.161641	65.031875	56.385659	14.373321	34.413386	29.838006	79	80	81
28 C	26.521664	67.798418	56.888859	14.034660	35.877378	30.104288	82	83	84
29 C	28.373026	58.476242	41.657688	15.014359	30.944294	22.044299	85	86	87
30 C	17.652662	57.843970	44.487166	9.341387	30.609711	23.541594	88	89	90
31 O	29.983640	62.484467	40.390251	15.866659	33.065356	21.373600	91	92	93
32 C	23.170946	53.535325	40.841448	12.261537	28.329674	21.612364	94	95	96
33 C	30.941409	57.519125	42.464374	16.373489	30.437810	22.471179	97	98	99
34 C	28.219473	61.327815	41.351642	14.933102	32.453282	21.882347	100	101	102
35 C	24.258155	66.050680	47.925985	12.836863	34.952515	25.361339	103	104	105
36 C	22.508709	50.740786	41.227665	11.911096	26.850868	21.816741	106	107	108

37	C	33.657422	58.271695	57.496091	17.810741	30.836053	30.425621	109	110	111
38	C	19.471773	64.154099	46.282245	10.304018	33.948887	24.491509	112	113	114
39	C	19.663772	50.179045	41.006703	10.405620	26.553607	21.699813	115	116	117
40	H	24.639284	58.097835	40.458829	13.038547	30.744050	21.409890	118	119	120
41	H	31.445044	58.328629	44.311163	16.640001	30.866181	23.448457	121	122	123
42	H	31.004805	55.465366	42.601785	16.407036	29.351007	22.543894	124	125	126
43	H	32.369576	58.199945	41.123827	17.129242	30.798084	21.761792	127	128	129
44	H	27.259249	66.588327	42.135150	14.424973	35.237025	22.296961	130	131	132
45	H	25.669086	65.442367	39.432767	13.583495	34.630609	20.866922	133	134	135
46	H	23.904316	66.260079	42.169586	12.649619	35.063324	22.315184	136	137	138
47	H	24.204272	61.644705	42.015806	12.808349	32.620973	22.233807	139	140	141
48	H	27.944428	63.570249	45.968577	14.787554	33.639927	24.325523	142	143	144
49	H	26.771262	60.505155	45.809814	14.166742	32.017949	24.241509	145	146	147
50	H	26.023694	66.280714	48.971847	13.771146	35.074244	25.914785	148	149	150
51	H	22.724645	66.629485	49.188699	12.025365	35.258805	26.029539	151	152	153
52	H	24.290145	67.302532	46.282679	12.853791	35.614966	24.491739	154	155	156
53	H	19.697125	66.163040	45.815659	10.423270	35.011973	24.244602	157	158	159
54	H	18.919974	63.946034	48.271972	10.012019	33.838784	25.544427	160	161	162
55	H	17.973279	63.355982	45.105823	9.511049	33.526542	23.868974	163	164	165

56 H 22.488950 54.152218 38.984936 11.900640 28.656120 20.629940 166 167
168

57 H 23.541823 49.674094 39.781178 12.457796 26.286398 21.051293 169 170
171

58 H 23.231762 50.098141 43.052742 12.293719 26.510794 22.782530 172 173
174

59 H 18.583461 51.141934 42.489537 9.833944 27.063146 22.484495 175 176
177

60 H 18.904117 50.793957 39.175910 10.003628 26.879005 20.730999 178 179
180

61 H 19.314517 48.145562 41.192156 10.220802 25.477534 21.797950 181 182
183

62 H 27.180847 51.151622 38.419240 14.383485 27.068272 20.330586 184 185
186

63 H 26.589851 54.097212 36.868948 14.070743 28.627012 19.510207 187 188
189

64 H 29.384853 53.666092 38.670042 15.549795 28.398873 20.463305 190 191
192

65 H 24.403081 58.084238 47.432999 12.913554 30.736855 25.100462 193 194
195

66 H 22.641224 57.591040 52.940455 11.981220 30.475866 28.014882 196 197
198

67 H 25.773021 57.047249 51.821218 13.638495 30.188104 27.422607 199 200
201

68 H 23.279444 55.051561 50.826777 12.318951 29.132032 26.896372 202 203
204

69 C 31.106053 58.101403 50.364605 16.460614 30.745938 26.651801 205 206
207

70 H 27.731513 69.033997 55.747005 14.674885 36.531218 29.500045 208 209
210

71 H 26.811759 68.253142 58.889752 14.188172 36.118007 31.163115 211 212
213

72 H 24.546290 68.182995 56.402090 12.989337 36.080887 29.846701 214 215
216

73 H 25.884139 63.808930 57.502154 13.697296 33.766232 30.428829 217 218
219

74 H 31.163657 65.676590 56.027242 16.491097 34.754555 29.648340 220 221
222

75 H	30.257717	64.571407	59.053707	16.011694	34.169717	31.249876	223	224	225
76 H	35.275799	61.068562	53.420448	18.667149	32.316091	28.268884	226	227	228
77 H	34.861561	64.025736	54.974453	18.447944	33.880961	29.091228	229	230	231
78 H	37.017862	61.793103	56.211614	19.589009	32.699502	29.745905	232	233	234
79 H	33.654696	57.056420	55.796577	17.809298	30.192957	29.526277	235	236	237
80 H	32.203828	57.567868	58.793149	17.041532	30.463604	31.111995	238	239	240
81 H	35.497865	58.072641	58.432342	18.784661	30.730718	30.921064	241	242	243
82 H	25.784594	60.790316	54.219079	13.644620	32.168850	28.691501	244	245	246
83 H	22.695201	62.348436	50.954702	12.009783	32.993371	26.964067	247	248	249
84 H	30.984730	62.413678	52.217256	16.396413	33.027896	27.632182	250	251	252
85 H	29.377391	60.327023	57.391137	15.545846	31.923686	30.370082	253	254	255
86 H	26.180631	53.158963	44.539436	13.854193	28.130512	23.569254	256	257	258
87 O	23.135935	53.744619	46.249568	12.243010	28.440427	24.474217	259	260	261
88 H	18.516049	55.149484	47.441006	9.798271	29.183850	25.104699	262	263	264
89 H	32.272053	54.741986	48.428992	17.077635	28.968211	25.627519	265	266	267
90 H	30.606095	54.144362	51.340171	16.196048	28.651962	27.168049	268	269	270
91 H	28.959427	55.195259	48.609695	15.324669	29.208073	25.723143	271	272	273
92 C	30.737199	55.351774	49.668352	16.265425	29.290897	26.283360	274	275	276
93 O	32.094082	59.677889	49.020087	16.983457	31.580179	25.940313	277	278	279

94 H 18.630516 59.209392 43.282884 9.858845 31.332261 22.904316 280 281 282

95 H 16.206035 58.825060 45.589674 8.575864 31.128881 24.125016 283 284 285

96 H 16.741677 56.437190 43.275277 8.859314 29.865275 22.900290 286 287 288

Number of elements of the density matrix on this node (used, total): 222269 1773786

Numerical Integration : Fuzzy Cells (Becke)

Becke grid quality: NORMAL

Lebedev angular grid order: min: 11 max: 23

Nr. of radial points: min: 46 max: 52

Total nr. of points: 600112

Nr. of blocks: 4689

Block length: 128

Nr. of dummy points: 80

Test of Precision of the Numerical Integration Grid

===

Integral of the Total Core Density: 85.99993812998208

Relative Error: -7.194E-07

Density Fitting (zlmFit)

ZlmFit Fit Quality: NORMAL

Max L-expansion: min: 6 max: 7

Nr. of radial interpolation points: min: 39 max: 48

S C F *** ScaSCF ***

Initial density matrix is taken from previous geometry

CYCLE 1

SCF Error: norm([F,P])= 0.097975693935, max([F,P])= 0.011763995563

orbitals (Q,E):

```
A :114...133       ( 2.00    -0.2503)  ( 2.00    -0.2502)  ( 2.00    -0.2394)  ( 2.00    -0.2365)
                   ( 2.00    -0.2285)  ( 2.00    -0.2234)  ( 2.00    -0.2214)  ( 2.00    -0.2171)
                   ( 2.00    -0.1958)  ( 2.00    -0.1783)  ( 0.00    -0.0714)  ( 0.00    -0.0698)
                   ( 0.00    -0.0656)  ( 0.00    -0.0420)  ( 0.00    -0.0200)  ( 0.00    -0.0141)
                   ( 0.00    -0.0122)  ( 0.00    -0.0088)  ( 0.00    -0.0058)  ( 0.00    -0.0014)
```

CYCLE 2

SDIIS (wt 0.000): 0.0452 0.9548

A-DIIS (wt 1.000): 0.0000 1.0000

DIIS coefficients: 0.0000 1.0000

SCF Error: norm([F,P])=   0.018760505806, max([F,P])=   0.001641184775

orbitals (Q,E):

A :114...124   (2.00   -0.2474) (2.00   -0.2455) (2.00   -0.2365) (2.00   -0.2345)

(2.00   -0.2267) (2.00   -0.2206) (2.00   -0.2197) (2.00   -0.2145)

(2.00   -0.1946) (2.00   -0.1772) (0.00   -0.0686)

CYCLE   3

SDIIS (wt 0.000): 0.0036 0.6617 0.3347

A-DIIS (wt 1.000): 0.0000 0.7241 0.2759

DIIS coefficients: 0.0000 0.7241 0.2759

SCF Error: norm([F,P])=   0.035766484437, max([F,P])=   0.003743426791

orbitals (Q,E):

A :114...124   (2.00   -0.2481) (2.00   -0.2469) (2.00   -0.2369) (2.00   -0.2348)

(2.00   -0.2268) (2.00   -0.2213) (2.00   -0.2199) (2.00   -0.2148)

(2.00   -0.1946) (2.00   -0.1771) (0.00   -0.0690)

CYCLE   4

SDIIS (wt 1.000): 0.3989 0.1576 0.4435

A-DIIS (wt 0.000): 0.0720 0.0000 0.9280

DIIS coefficients: 0.3989 0.1576 0.4435

SCF Error: norm([F,P])=   0.007706441015, max([F,P])=   0.001007498365

orbitals (Q,E):

A :114...124   (2.00   -0.2483) (2.00   -0.2475) (2.00   -0.2373) (2.00   -0.2349)

(2.00   -0.2270) (2.00   -0.2215) (2.00   -0.2199) (2.00   -0.2152)

(2.00   -0.1946) (2.00   -0.1771) (0.00   -0.0693)

CYCLE 5

SDIIS (wt 1.000): 0.1828 0.0570 0.3203 0.4398

DIIS coefficients: 0.1828 0.0570 0.3203 0.4398

SCF Error: norm([F,P])= 0.003454564469, max([F,P])= 0.000638276484

orbitals (Q,E):

A :114...124 (2.00 -0.2483) (2.00 -0.2475) (2.00 -0.2373) (2.00 -0.2350)

(2.00 -0.2270) (2.00 -0.2215) (2.00 -0.2200) (2.00 -0.2152)

(2.00 -0.1946) (2.00 -0.1772) (0.00 -0.0693)

CYCLE 6

SDIIS (wt 1.000): 0.0724 0.0170 0.1616 0.3079 0.4412

DIIS coefficients: 0.0724 0.0170 0.1616 0.3079 0.4412

SCF Error: norm([F,P])= 0.001240393613, max([F,P])= 0.000157451709

orbitals (Q,E):

A :114...124 (2.00 -0.2483) (2.00 -0.2475) (2.00 -0.2372) (2.00 -0.2350)

(2.00 -0.2270) (2.00 -0.2215) (2.00 -0.2200) (2.00 -0.2151)

(2.00 -0.1946) (2.00 -0.1771) (0.00 -0.0693)

CYCLE 7

SDIIS (wt 1.000): 0.0226 0.0031 0.0622 0.1399 0.2836 0.4885

DIIS coefficients: 0.0226 0.0031 0.0622 0.1399 0.2836 0.4885

SCF Error: norm([F,P])= 0.000376092666, max([F,P])= 0.000048154326

orbitals (Q,E):

A :114...124 (2.00 -0.2483) (2.00 -0.2475) (2.00 -0.2373) (2.00 -0.2350)

(2.00 -0.2270) (2.00 -0.2215) (2.00 -0.2200) (2.00 -0.2152)

(2.00 -0.1946) (2.00 -0.1771) (0.00 -0.0693)

CYCLE 8

SDIIS (wt 1.000): 0.0089 0.0005 0.0289 0.0712 0.1660 0.3718 0.3527

DIIS coefficients: 0.0089 0.0005 0.0289 0.0712 0.1660 0.3718 0.3527

SCF Error: norm([F,P])= 0.000200892126, max([F,P])= 0.000026688138

orbitals (Q,E):

A :114...124 (2.00 -0.2483) (2.00 -0.2475) (2.00 -0.2373) (2.00 -0.2350)

(2.00 -0.2270) (2.00 -0.2215) (2.00 -0.2200) (2.00 -0.2152)

(2.00 -0.1946) (2.00 -0.1771) (0.00 -0.0693)

CYCLE 9

SDIIS (wt 1.000): 0.0017 -0.0002 0.0079 0.0220 0.0607 0.1638 0.2472 0.4969

DIIS coefficients: 0.0017 -0.0002 0.0079 0.0220 0.0607 0.1638 0.2472 0.4969

SCF Error: norm([F,P])= 0.000058694889, max([F,P])= 0.000006890033

orbitals (Q,E):

A :114...124 (2.00 -0.2483) (2.00 -0.2475) (2.00 -0.2373) (2.00 -0.2350)

(2.00 -0.2270) (2.00 -0.2215) (2.00 -0.2200) (2.00 -0.2152)

(2.00 -0.1946) (2.00 -0.1771) (0.00 -0.0693)

CYCLE 10

SDIIS (wt 1.000): 0.0001 -0.0002 0.0017 0.0055 0.0189 0.0595 0.1152 0.3154 0.4839

DIIS coefficients: 0.0001 -0.0002 0.0017 0.0055 0.0189 0.0595 0.1152 0.3154 0.4839

SCF Error: norm([F,P])= 0.000018938234, max([F,P])= 0.000002476805

orbitals (Q,E):

A :114...124 (2.00 -0.2483) (2.00 -0.2475) (2.00 -0.2373) (2.00 -0.2350)

(2.00 -0.2270) (2.00 -0.2215) (2.00 -0.2200) (2.00 -0.2152)

(2.00 -0.1946) (2.00 -0.1771) (0.00 -0.0693)

CYCLE 11

SDIIS (wt 1.000): -0.0001 -0.0001 0.0002 0.0011 0.0055 0.0203 0.0472 0.1494 0.3081 0.4683

DIIS coefficients: -0.0001 -0.0001 0.0002 0.0011 0.0055 0.0203 0.0472 0.1494 0.3081 0.4683

SCF Error: norm([F,P])= 0.000006413988, max([F,P])= 0.000000847139

orbitals (Q,E):

A :114...124 (2.00 -0.2483) (2.00 -0.2475) (2.00 -0.2373) (2.00 -0.2350)

(2.00 -0.2270) (2.00 -0.2215) (2.00 -0.2200) (2.00 -0.2152)

(2.00 -0.1946) (2.00 -0.1771) (0.00 -0.0693)

SCF CONVERGED

CYCLE 12

*** Setting up for NEW gradients in Focky

*** Using FIT density in Focky

*** Setting up for NEW Hessian in Focky

orbitals (Q,E):

A :114...133 (2.00 -0.2483) (2.00 -0.2475) (2.00 -0.2373) (2.00 -0.2350)

(2.00 -0.2270) (2.00 -0.2215) (2.00 -0.2200) (2.00 -0.2152)

(2.00 -0.1946) (2.00 -0.1771) (0.00 -0.0693) (0.00 -0.0684)

(0.00 -0.0624) (0.00 -0.0395) (0.00 -0.0185) (0.00 -0.0129)

(0.00 -0.0110) (0.00 -0.0075) (0.00 -0.0047) (0.00 -0.0001)

Fit test: (difference of exact and fit density, squared integrated, result summed over spins)

SCF: 0.00100834308860

Gross Charges per Atom (Z minus electrons)

==

-0.6647 0.9227 0.5465 0.3319 -0.5798 -0.3815 -0.6314 0.6730 0.6267 0.8623

-0.6595 -0.0002 -0.5769 0.5874 0.3249 -0.5908 0.5958 0.6003 -0.6025 0.2585

0.3384	0.0982	0.1675	0.7464	0.4141	0.4082	0.5605	0.5721	0.0211	0.6412
-0.5653	0.5505	0.5491	0.5704	0.6425	0.3719	0.7502	0.9849	0.5867	-0.2130
-0.1545	-0.2106	-0.2014	-0.2129	-0.2445	-0.2316	-0.1501	-0.1868	-0.1337	-0.2067
-0.2413	-0.2206	-0.2260	-0.1840	-0.2441	-0.1708	-0.1956	-0.1541	-0.1977	-0.2140
-0.2239	-0.2191	-0.2275	-0.1928	-0.1169	-0.2235	-0.1560	-0.2166	0.8255	-0.1967
-0.2183	-0.2173	-0.2103	-0.1902	-0.2119	-0.1823	-0.2256	-0.2335	-0.1976	-0.2407
-0.2127	-0.2516	-0.1997	-0.1595	-0.1666	0.2329	-0.6743	-0.1256	-0.1937	-0.2080
-0.0813	0.5050	-0.6180	-0.1743	-0.2163	-0.2384				

Net Total: 0.00000000

M U L T I P O L E D E R I V E D C H A R G E A N A L Y S I S

See: M. Swart, P.Th. van Duijnen, J.G. Snijders, J.Comput.Chem., (2001), p. 79-88.

Multipole derived atomic charges (a.u.)

Atom	Level:	MDC-m	MDC-d	MDC-q
1	O	-0.350949	-0.321572	-0.345628
2	C	0.362786	0.374863	0.396451
3	C	0.242384	0.249793	0.253567
4	C	0.180666	0.166260	0.211622
5	O	-0.305787	-0.548518	-0.611352
6	N	-0.214409	-0.277712	-0.469418
7	O	-0.380272	-0.382851	-0.448426

8	C	0.250720	0.309438	0.307920
9	C	0.227986	0.193229	0.187557
10	C	0.339452	0.409542	0.416482
11	O	-0.383008	-0.354940	-0.395869
12	C	0.124292	0.102383	0.101782
13	O	-0.313163	-0.299319	-0.344823
14	C	0.245482	0.242947	0.248364
15	C	0.196172	0.158998	0.174767
16	O	-0.369799	-0.477964	-0.483115
17	C	0.227430	0.192763	0.198451
18	C	0.229751	0.193288	0.195619
19	O	-0.319059	-0.320080	-0.373805
20	C	0.057258	0.044598	-0.057757
21	C	0.195138	0.221353	0.283456
22	C	0.118817	0.105078	0.107843
23	C	0.119859	0.099748	0.111156
24	C	0.290671	0.268496	0.286676
25	C	0.173077	0.135629	0.137748
26	C	0.164785	0.124740	0.164449
27	C	0.249078	0.238670	0.261350
28	C	0.234480	0.193760	0.303121
29	C	0.034490	0.027574	-0.045426
30	C	0.236888	0.191101	0.177246
31	O	-0.372593	-0.476726	-0.515874
32	C	0.254493	0.243279	0.263256
33	C	0.222670	0.185705	0.190680
34	C	0.227912	0.294867	0.382114
35	C	0.221844	0.191887	0.201431
36	C	0.180384	0.145505	0.150232
37	C	0.293886	0.271013	0.311159
38	C	0.368216	0.339498	0.387169

39	C	0.229430	0.190102	0.156406
40	H	-0.073368	-0.060224	-0.026243
41	H	-0.057040	-0.031073	-0.017989
42	H	-0.063986	-0.056766	-0.031818
43	H	-0.056727	-0.035505	-0.015166
44	H	-0.068897	-0.052637	-0.050864
45	H	-0.070627	-0.057957	-0.053352
46	H	-0.076541	-0.071746	-0.069933
47	H	-0.071738	-0.062095	-0.062298
48	H	-0.063580	-0.041566	-0.022580
49	H	-0.082534	-0.062520	-0.070240
50	H	-0.055191	-0.046887	-0.048930
51	H	-0.072667	-0.055435	-0.060181
52	H	-0.070671	-0.058654	-0.065222
53	H	-0.067856	-0.064078	-0.073627
54	H	-0.057962	-0.045130	-0.052239
55	H	-0.054189	-0.043894	-0.045920
56	H	-0.057296	-0.042363	-0.030659
57	H	-0.067237	-0.050289	-0.051126
58	H	-0.061805	-0.036467	-0.032841
59	H	-0.075274	-0.060493	-0.049713
60	H	-0.074778	-0.065380	-0.055970
61	H	-0.068875	-0.054985	-0.043140
62	H	-0.066949	-0.048508	-0.039878
63	H	-0.072429	-0.074730	-0.083260
64	H	-0.058052	-0.035798	-0.034080
65	H	-0.074630	-0.049216	-0.057762
66	H	-0.072452	-0.064062	-0.060813
67	H	-0.067684	-0.054083	-0.066983
68	H	-0.065053	-0.056478	-0.055921
69	C	0.353027	0.438432	0.459388

70	H	-0.064631	-0.054193	-0.082085
71	H	-0.068127	-0.067197	-0.097728
72	H	-0.063251	-0.048392	-0.074049
73	H	-0.080801	-0.062466	-0.072829
74	H	-0.072857	-0.059988	-0.063887
75	H	-0.069497	-0.068787	-0.072905
76	H	-0.076644	-0.047178	-0.040718
77	H	-0.066417	-0.054151	-0.039223
78	H	-0.064937	-0.060347	-0.029166
79	H	-0.082787	-0.064120	-0.047302
80	H	-0.066688	-0.057918	-0.051892
81	H	-0.067763	-0.066406	-0.056270
82	H	-0.070981	-0.044107	-0.037583
83	H	-0.061943	-0.025814	-0.018949
84	H	-0.047006	-0.002430	-0.015374
85	H	-0.073668	-0.059885	-0.041294
86	H	0.062911	0.263366	0.295749
87	O	-0.365834	-0.443811	-0.459704
88	H	-0.044994	0.000779	0.021668
89	H	-0.045152	-0.029946	-0.017552
90	H	-0.052030	-0.050305	-0.040991
91	H	-0.042249	-0.010461	-0.011533
92	C	0.240962	0.197658	0.187329
93	O	-0.363349	-0.463155	-0.490646
94	H	-0.061216	-0.053629	-0.049681
95	H	-0.060974	-0.047526	-0.039241
96	H	-0.068477	-0.065427	-0.061365

*** Using NEW gradient routines ***

Geometry CYCLE 17

==============

Energy gradients wrt nuclear displacements

==

Atom	Cartesian (a.u./angstrom)		
	X	Y	Z
1 O	-0.000285	-0.001635	0.000266
2 C	0.000728	-0.000853	0.000493
3 C	-0.000144	0.000035	0.000087
4 C	0.000971	0.000153	-0.000409
5 O	0.001244	-0.000956	-0.001573
6 N	-0.000091	0.000442	0.000802
7 O	0.000103	0.000519	-0.000115
8 C	0.000284	0.000419	-0.001075
9 C	-0.000015	-0.000070	0.000037
10 C	-0.001479	0.000450	-0.000390
11 O	-0.000545	-0.000921	0.000167
12 C	0.000580	0.000795	0.000515
13 O	-0.000362	0.000236	-0.000934
14 C	-0.000327	0.001324	-0.001105
15 C	0.000759	0.000100	0.000664
16 O	-0.000900	0.001131	0.000557
17 C	0.000769	0.000617	-0.000479
18 C	-0.000085	-0.000094	0.000016
19 O	-0.003247	0.000639	-0.000707
20 C	-0.000166	-0.000371	0.000349
21 C	-0.000120	-0.000502	0.000706
22 C	0.000848	-0.000291	0.000039
23 C	0.000664	0.000096	0.000094

24	C	-0.000056	-0.000496	0.000230
25	C	-0.000347	0.000148	-0.000196
26	C	0.000773	-0.000324	0.000274
27	C	-0.000489	0.000067	0.000068
28	C	0.000192	-0.000176	-0.000275
29	C	0.000306	0.000588	0.000646
30	C	-0.000107	-0.000352	0.000054
31	O	0.001423	0.000685	0.000488
32	C	0.000068	0.000545	0.000347
33	C	-0.000054	-0.000650	-0.000903
34	C	-0.002267	-0.000659	-0.001461
35	C	0.000729	0.000396	0.000136
36	C	-0.000275	0.000188	-0.000451
37	C	0.000114	-0.000088	-0.000105
38	C	0.000195	-0.000962	-0.000129
39	C	0.000267	0.000044	-0.000042
40	H	0.000088	0.000061	-0.000536
41	H	0.000189	0.000436	0.000301
42	H	0.000124	-0.000081	-0.000184
43	H	0.000513	0.000445	0.000029
44	H	0.000221	0.000260	0.000125
45	H	-0.000014	0.000010	-0.000388
46	H	-0.000272	0.000169	0.000063
47	H	-0.000184	0.000072	0.000233
48	H	0.000307	0.000229	0.000041
49	H	-0.000100	-0.000734	-0.000036
50	H	0.000055	0.000472	0.000097
51	H	0.000061	0.000147	0.000457
52	H	0.000029	0.000163	-0.000184
53	H	-0.000149	0.000400	0.000024
54	H	-0.000193	-0.000268	0.000683

55	H	-0.000523	-0.000393	-0.000344
56	H	-0.000167	-0.000085	-0.000359
57	H	0.000412	-0.000278	-0.000084
58	H	-0.000062	-0.000326	0.000191
59	H	-0.000264	0.000177	0.000241
60	H	-0.000229	0.000029	-0.000301
61	H	-0.000073	-0.000378	0.000030
62	H	-0.000045	-0.000307	-0.000060
63	H	-0.000112	0.000099	-0.000274
64	H	0.000437	0.000035	0.000166
65	H	-0.000259	-0.000230	-0.000169
66	H	-0.000184	0.000016	0.000472
67	H	0.000566	0.000337	0.000324
68	H	0.000055	-0.000438	-0.000154
69	C	0.001562	-0.001805	0.001279
70	H	0.000178	0.000312	-0.000201
71	H	0.000055	0.000103	0.000357
72	H	-0.000421	0.000085	-0.000091
73	H	-0.000150	-0.000108	0.000239
74	H	0.000328	0.000320	-0.000147
75	H	-0.000060	0.000187	0.000426
76	H	0.000219	0.000197	-0.000264
77	H	0.000004	0.000387	-0.000092
78	H	0.000451	0.000073	0.000177
79	H	0.000090	-0.000101	0.000164
80	H	-0.000135	-0.000198	0.000241
81	H	0.000350	0.000013	0.000313
82	H	-0.000712	-0.000517	0.000046
83	H	-0.000845	-0.000346	-0.000073
84	H	-0.000060	0.000200	-0.000030
85	H	-0.000344	-0.000046	0.000205

86 H -0.000361 0.000648 0.000242

87 O 0.000137 -0.000469 0.002102

88 H 0.000198 -0.000656 0.000157

89 H 0.000434 -0.000187 0.000046

90 H -0.000161 -0.000127 0.000337

91 H -0.000289 -0.000122 -0.000052

92 C -0.000321 -0.000059 -0.000086

93 O 0.000139 0.001594 -0.001854

94 H 0.000208 0.000286 -0.000374

95 H -0.000160 0.000315 0.000159

96 H -0.000216 -0.000236 -0.000316

----

dE(predicted): -0.39207E-03, dE(actual): -0.29697E-03, Trust radius: 0.195959, all in a.u.

Geometry Convergence after Step 17 (Hartree/Angstrom,Angstrom)

----

current energy -19.67504836 Hartree

energy change -0.00029697 0.00100000 T

constrained gradient max 0.00324691 0.00100000 F

constrained gradient rms 0.00055339 0.00066667 T

gradient max 0.00324691

gradient rms 0.00055339

cart. step max 0.09169747 0.01000000 F

cart. step rms 0.01874967 0.00666667 F

Coordinates (Cartesian)

======================

Atom	bohr			angstrom			Geometric Variables		
	X	Y	Z	X	Y	Z	(0:frozen, *:LT par.)		
1 O	26.554691	61.656407	50.474769	14.052137	32.627165	26.710098	1	2	3
2 C	27.032121	61.975494	53.050869	14.304783	32.796019	28.073311	4	5	6
3 C	29.824131	61.244365	53.444827	15.782250	32.409122	28.281785	7	8	9
4 C	30.589697	61.573005	56.244245	16.187371	32.583031	29.763173	10	11	12
5 O	27.288623	52.928206	43.109976	14.440517	28.008400	22.812817	13	14	15
6 N	33.223626	60.934224	56.903461	17.581186	32.245003	30.112015	16	17	18
7 O	26.651861	64.573962	53.745236	14.103558	34.171069	28.440754	19	20	21
8 C	20.442658	58.486957	48.383822	10.817789	30.949965	25.603616	22	23	24
9 C	25.704158	65.394467	41.497059	13.602054	34.605262	21.959298	25	26	27
10 C	21.632325	55.043162	45.039749	11.447333	29.127587	23.834009	28	29	30
11 O	21.700901	62.746496	45.680999	11.483622	33.204016	24.173344	31	32	33
12 C	19.532652	56.573259	46.298765	10.336234	29.937280	24.500252	34	35	36
13 O	21.584980	55.195619	42.501036	11.422280	29.208264	22.490580	37	38	39
14 C	23.974765	61.555598	49.552038	12.686900	32.573820	26.221809	40	41	42
15 C	23.998510	63.269951	47.122177	12.699465	33.481016	24.935982	43	44	45
16 O	18.813634	59.550341	49.629867	9.955746	31.512683	26.262995	46	47	48
17 C	23.746915	57.003591	51.316049	12.566326	30.165001	27.155284	49	50	51
18 C	27.413667	53.183104	38.614747	14.506688	28.143287	20.434044	52	53	54
19 O	30.104378	58.606655	52.674606	15.930551	31.013306	27.874201	55	56	57
20 C	26.346143	57.072553	41.109524	13.941778	30.201495	21.754223	58	59	60
21 C	26.081722	54.191986	41.016798	13.801853	28.677164	21.705155	61	62	63
22 C	23.244681	58.764241	49.019849	12.300556	31.096697	25.940187	64	65	66
23 C	25.945529	62.685799	42.505877	13.729783	33.171896	22.493141	67	68	69
24 C	35.173888	61.998126	55.234768	18.613220	32.807996	29.228980	70	71	72
25 C	26.282744	62.470962	45.418499	13.908229	33.058209	24.034435	73	74	75
26 C	29.925999	64.317001	57.029701	15.836157	34.035091	30.178818	76	77	78
27 C	27.162922	65.002357	56.418547	14.373999	34.397766	29.855409	79	80	81

28 C	26.520519	67.762767	56.951004	14.034054	35.858512	30.137173	82	83	84
29 C	28.395729	58.454762	41.703833	15.026372	30.932928	22.068718	85	86	87
30 C	17.652296	57.848897	44.473359	9.341193	30.612318	23.534288	88	89	90
31 O	30.020447	62.456280	40.429520	15.886137	33.050440	21.394380	91	92	93
32 C	23.193711	53.525851	40.857389	12.273583	28.324660	21.620799	94	95	96
33 C	30.953764	57.485900	42.528068	16.380026	30.420228	22.504885	97	98	99
34 C	28.254109	61.306768	41.395621	14.951431	32.442145	21.905619	100	101	102
35 C	24.247179	66.056726	47.930844	12.831054	34.955714	25.363910	103	104	105
36 C	22.522237	50.735343	41.255069	11.918255	26.847987	21.831242	106	107	108
37 C	33.675497	58.239689	57.410542	17.820305	30.819116	30.380351	109	110	111
38 C	19.468405	64.187335	46.271661	10.302236	33.966475	24.485908	112	113	114
39 C	19.676478	50.182304	41.024945	10.412344	26.555332	21.709466	115	116	117
40 H	24.668946	58.084490	40.479643	13.054244	30.736988	21.420905	118	119	120
41 H	31.446471	58.287173	44.381432	16.640756	30.844244	23.485642	121	122	123
42 H	31.007425	55.431384	42.659893	16.408423	29.333025	22.574643	124	125	126
43 H	32.394439	58.164486	41.199839	17.142399	30.779320	21.802016	127	128	129
44 H	27.282944	66.571946	42.136578	14.437512	35.228357	22.297717	130	131	132
45 H	25.722534	65.406669	39.425506	13.611779	34.611719	20.863079	133	134	135
46 H	23.928278	66.240634	42.137301	12.662299	35.053034	22.298099	136	137	138
47 H	24.233530	61.626231	42.018625	12.823832	32.611197	22.235299	139	140	141
48 H	27.936240	63.573546	45.993454	14.783222	33.641672	24.338687	142	143	144

49 H	26.765898	60.507493	45.839740	14.163903	32.019186	24.257346	145	146	147
50 H	26.011099	66.283985	48.979973	13.764481	35.075974	25.919086	148	149	150
51 H	22.711957	66.636511	49.190824	12.018650	35.262523	26.030663	151	152	153
52 H	24.283561	67.309313	46.288430	12.850307	35.618554	24.494782	154	155	156
53 H	19.710125	66.192958	45.799476	10.430149	35.027805	24.236039	157	158	159
54 H	18.908892	63.989612	48.260044	10.006155	33.861844	25.538115	160	161	162
55 H	17.968165	63.396632	45.092724	9.508344	33.548053	23.862042	163	164	165
56 H	22.523137	54.136772	38.994807	11.918731	28.647946	20.635163	166	167	168
57 H	23.558279	49.657731	39.819074	12.466505	26.277740	21.071347	169	170	171
58 H	23.235116	50.101088	43.086900	12.295494	26.512354	22.800606	172	173	174
59 H	18.593641	51.156596	42.498297	9.839331	27.070905	22.489130	175	176	177
60 H	18.926661	50.790670	39.188051	10.015558	26.877265	20.737424	178	179	180
61 H	19.319120	48.151196	41.219168	10.223238	25.480516	21.812244	181	182	183
62 H	27.221665	51.127628	38.472337	14.405085	27.055576	20.358684	184	185	186
63 H	26.644579	54.068653	36.908830	14.099704	28.611899	19.531312	187	188	189
64 H	29.425082	53.641125	38.732432	15.571083	28.385661	20.496320	190	191	192
65 H	24.388557	58.087669	47.439402	12.905869	30.738671	25.103850	193	194	195
66 H	22.607890	57.598357	52.941558	11.963580	30.479738	28.015466	196	197	198
67 H	25.742963	57.049220	51.834654	13.622590	30.189147	27.429717	199	200	201

68 H	23.250292	55.057117	50.831917	12.303524	29.134971	26.899092	202	203	204
69 C	31.065124	58.162142	50.284131	16.438956	30.778080	26.609216	205	206	207
70 H	27.720956	69.011050	55.813324	14.669298	36.519075	29.535139	208	209	210
71 H	26.821853	68.199682	58.954034	14.193513	36.089718	31.197131	211	212	213
72 H	24.541654	68.147824	56.479607	12.986884	36.062275	29.887721	214	215	216
73 H	25.894868	63.766457	57.531503	13.702974	33.743756	30.444360	217	218	219
74 H	31.161434	65.659291	56.040995	16.489921	34.745400	29.655617	220	221	222
75 H	30.277171	64.522553	59.061978	16.021989	34.143865	31.254253	223	224	225
76 H	35.261969	61.081250	53.357131	18.659830	32.322805	28.235378	226	227	228
77 H	34.857648	64.022049	54.944724	18.445873	33.879009	29.075496	229	230	231
78 H	37.024025	61.778078	56.142462	19.592270	32.691551	29.709312	232	233	234
79 H	33.661927	57.045608	55.696383	17.813125	30.187236	29.473257	235	236	237
80 H	32.230823	57.519381	58.708315	17.055817	30.437946	31.067102	238	239	240
81 H	35.522037	58.030430	58.332115	18.797452	30.708381	30.868026	241	242	243
82 H	25.785372	60.776737	54.224060	13.645031	32.161664	28.694137	244	245	246
83 H	22.673417	62.353501	50.954720	11.998255	32.996052	26.964077	247	248	249
84 H	30.966084	62.435331	52.200640	16.386546	33.039354	27.623389	250	251	252
85 H	29.394789	60.292766	57.362846	15.555052	31.905558	30.355111	253	254	255
86 H	26.169085	53.143352	44.576170	13.848083	28.122251	23.588693	256	257	258

87 O 23.135739 53.760335 46.263282 12.242906 28.448744 24.481475 259 260
261

88 H 18.510441 55.152216 47.426197 9.795303 29.185296 25.096863 262 263
264

89 H 32.191225 54.845142 48.255709 17.034863 29.022799 25.535822 265 266
267

90 H 30.577656 54.186188 51.183200 16.180999 28.674096 27.084983 268 269
270

91 H 28.883159 55.296714 48.506225 15.284310 29.261761 25.668389 271 272
273

92 C 30.679948 55.429047 49.535521 16.235129 29.331789 26.213069 274 275
276

93 O 32.032047 59.764705 48.955431 16.950629 31.626120 25.906099 277 278
279

94 H 18.631550 59.218396 43.275223 9.859392 31.337026 22.900262 280 281
282

95 H 16.199315 58.824782 45.571859 8.572309 31.128734 24.115589 283 284
285

96 H 16.749011 56.442243 43.255896 8.863195 29.867949 22.890034 286 287
288

Number of elements of the density matrix on this node (used, total): 222269 1773786

Numerical Integration : Fuzzy Cells (Becke)

Becke grid quality: NORMAL

Lebedev angular grid order: min: 11 max: 23

Nr. of radial points: min: 46 max: 52

Total nr. of points: 600103

Nr. of blocks: 4689

Block length: 128

Nr. of dummy points: 89

Test of Precision of the Numerical Integration Grid

==

Integral of the Total Core Density: 85.99994476537188

Relative Error: -6.423E-07

Density Fitting (zlmFit)

ZlmFit Fit Quality: NORMAL

Max L-expansion: min: 6 max: 7

Nr. of radial interpolation points: min: 39 max: 48

S C F *** ScaSCF ***

Initial density matrix is taken from previous geometry

```
CYCLE    1
SCF Error: norm([F,P])=      0.064823144579, max([F,P])=      0.006304678521
orbitals (Q,E):
---------------
A :114...133       ( 2.00    -0.2488)  ( 2.00    -0.2474)  ( 2.00    -0.2378)  ( 2.00    -0.2352)
                   ( 2.00    -0.2270)  ( 2.00    -0.2214)  ( 2.00    -0.2200)  ( 2.00    -0.2152)
                   ( 2.00    -0.1948)  ( 2.00    -0.1771)  ( 0.00    -0.0693)  ( 0.00    -0.0685)
                   ( 0.00    -0.0626)  ( 0.00    -0.0400)  ( 0.00    -0.0184)  ( 0.00    -0.0129)
                   ( 0.00    -0.0110)  ( 0.00    -0.0073)  ( 0.00    -0.0047)  ( 0.00    -0.0000)

CYCLE    2
  MIX coefficients:  0.8000  0.2000
SCF Error: norm([F,P])=      0.010336478674, max([F,P])=      0.000830858088
orbitals (Q,E):
---------------
A :114...124       ( 2.00    -0.2486)  ( 2.00    -0.2475)  ( 2.00    -0.2377)  ( 2.00    -0.2351)
                   ( 2.00    -0.2269)  ( 2.00    -0.2213)  ( 2.00    -0.2199)  ( 2.00    -0.2152)
                   ( 2.00    -0.1947)  ( 2.00    -0.1770)  ( 0.00    -0.0693)

CYCLE    3
 SDIIS (wt  0.000): -0.7552  1.7552
A-DIIS (wt  1.000):  0.0000  1.0000
 DIIS coefficients:  0.0000  1.0000
SCF Error: norm([F,P])=      0.005552032422, max([F,P])=      0.000538597517
orbitals (Q,E):
---------------
A :114...124       ( 2.00    -0.2482)  ( 2.00    -0.2477)  ( 2.00    -0.2372)  ( 2.00    -0.2348)
                   ( 2.00    -0.2267)  ( 2.00    -0.2211)  ( 2.00    -0.2198)  ( 2.00    -0.2151)
                   ( 2.00    -0.1945)  ( 2.00    -0.1768)  ( 0.00    -0.0693)

CYCLE    4
```

SDIIS (wt 1.000): 0.4726 -0.1320 0.6594

DIIS coefficients: 0.4726 -0.1320 0.6594

SCF Error: norm([F,P])= 0.006503696219, max([F,P])= 0.000454518480

orbitals (Q,E):

A :114...124 (2.00 -0.2485) (2.00 -0.2476) (2.00 -0.2375) (2.00 -0.2350)

(2.00 -0.2268) (2.00 -0.2212) (2.00 -0.2199) (2.00 -0.2152)

(2.00 -0.1947) (2.00 -0.1769) (0.00 -0.0693)

CYCLE 5

SDIIS (wt 1.000): 0.4850 -0.4006 0.4915 0.4241

DIIS coefficients: 0.4850 -0.4006 0.4915 0.4241

SCF Error: norm([F,P])= 0.002371634634, max([F,P])= 0.000299883936

orbitals (Q,E):

A :114...124 (2.00 -0.2484) (2.00 -0.2476) (2.00 -0.2374) (2.00 -0.2349)

(2.00 -0.2268) (2.00 -0.2212) (2.00 -0.2199) (2.00 -0.2151)

(2.00 -0.1946) (2.00 -0.1769) (0.00 -0.0693)

CYCLE 6

SDIIS (wt 1.000): 0.2500 -0.2349 0.2321 0.2647 0.4881

DIIS coefficients: 0.2500 -0.2349 0.2321 0.2647 0.4881

SCF Error: norm([F,P])= 0.000603652093, max([F,P])= 0.000081129190

orbitals (Q,E):

A :114...124 (2.00 -0.2484) (2.00 -0.2476) (2.00 -0.2374) (2.00 -0.2350)

(2.00 -0.2268) (2.00 -0.2212) (2.00 -0.2199) (2.00 -0.2151)

(2.00 -0.1946) (2.00 -0.1769) (0.00 -0.0693)

CYCLE 7

SDIIS (wt 1.000): 0.0972 -0.0998 0.0842 0.1169 0.3322 0.4692

```
 DIIS coefficients:  0.0972 -0.0998  0.0842  0.1169  0.3322  0.4692
SCF Error: norm([F,P])=     0.000255391693, max([F,P])=     0.000032045639
orbitals (Q,E):
---------------
A :114...124       ( 2.00    -0.2484)  ( 2.00    -0.2476)  ( 2.00    -0.2374)  ( 2.00    -0.2350)
                   ( 2.00    -0.2268)  ( 2.00    -0.2212)  ( 2.00    -0.2199)  ( 2.00    -0.2151)
                   ( 2.00    -0.1946)  ( 2.00    -0.1769)  ( 0.00    -0.0693)

CYCLE    8
 SDIIS (wt  1.000):  0.0416 -0.0445  0.0347  0.0535  0.1787  0.3150  0.4209
 DIIS coefficients:  0.0416 -0.0445  0.0347  0.0535  0.1787  0.3150  0.4209
SCF Error: norm([F,P])=     0.000087809390, max([F,P])=     0.000013021105
orbitals (Q,E):
---------------
A :114...124       ( 2.00    -0.2484)  ( 2.00    -0.2476)  ( 2.00    -0.2374)  ( 2.00    -0.2350)
                   ( 2.00    -0.2268)  ( 2.00    -0.2212)  ( 2.00    -0.2199)  ( 2.00    -0.2151)
                   ( 2.00    -0.1946)  ( 2.00    -0.1769)  ( 0.00    -0.0693)

CYCLE    9
 SDIIS (wt  1.000):  0.0129 -0.0148  0.0103  0.0186  0.0738  0.1530  0.3278  0.4184
 DIIS coefficients:  0.0129 -0.0148  0.0103  0.0186  0.0738  0.1530  0.3278  0.4184
SCF Error: norm([F,P])=     0.000043421196, max([F,P])=     0.000005220845
orbitals (Q,E):
---------------
A :114...124       ( 2.00    -0.2484)  ( 2.00    -0.2476)  ( 2.00    -0.2374)  ( 2.00    -0.2350)
                   ( 2.00    -0.2268)  ( 2.00    -0.2212)  ( 2.00    -0.2199)  ( 2.00    -0.2151)
                   ( 2.00    -0.1946)  ( 2.00    -0.1769)  ( 0.00    -0.0693)

CYCLE   10
 SDIIS (wt  1.000):  0.0032 -0.0040  0.0024  0.0055  0.0261  0.0619  0.1659  0.2852  0.4538
 DIIS coefficients:  0.0032 -0.0040  0.0024  0.0055  0.0261  0.0619  0.1659  0.2852  0.4538
```

SCF Error: norm([F,P])=   0.000013279944, max([F,P])=   0.000001722792

orbitals (Q,E):

A :114...124   (2.00   -0.2484) (2.00   -0.2476) (2.00   -0.2374) (2.00   -0.2350)

(2.00   -0.2268) (2.00   -0.2212) (2.00   -0.2199) (2.00   -0.2151)

(2.00   -0.1946) (2.00   -0.1769) (0.00   -0.0693)

CYCLE   11

SDIIS (wt 1.000): 0.0006 -0.0009 0.0003 0.0015 0.0089 0.0240 0.0751 0.1471 0.3225 0.4209

DIIS coefficients: 0.0006 -0.0009 0.0003 0.0015 0.0089 0.0240 0.0751 0.1471 0.3225 0.4209

SCF Error: norm([F,P])=   0.000005381014, max([F,P])=   0.000000807178

orbitals (Q,E):

A :114...124   (2.00   -0.2484) (2.00   -0.2476) (2.00   -0.2374) (2.00   -0.2350)

(2.00   -0.2268) (2.00   -0.2212) (2.00   -0.2199) (2.00   -0.2151)

(2.00   -0.1946) (2.00   -0.1769) (0.00   -0.0693)

SCF CONVERGED

CYCLE   12

*** Setting up for NEW gradients in Focky

*** Using FIT density in Focky

*** Setting up for NEW Hessian in Focky

orbitals (Q,E):

A :114...133   (2.00   -0.2484) (2.00   -0.2476) (2.00   -0.2374) (2.00   -0.2350)

(2.00   -0.2268) (2.00   -0.2212) (2.00   -0.2199) (2.00   -0.2151)

(2.00   -0.1946) (2.00   -0.1769) (0.00   -0.0693) (0.00   -0.0683)

(0.00   -0.0627) (0.00   -0.0396) (0.00   -0.0185) (0.00   -0.0128)

(0.00   -0.0109) (0.00   -0.0072) (0.00   -0.0046) (0.00   -0.0000)

Fit test: (difference of exact and fit density, squared integrated, result summed over spins)

SCF: 0.00100855800314

Gross Charges per Atom (Z minus electrons)

==

-0.6652	0.9214	0.5478	0.3313	-0.5804	-0.3813	-0.6310	0.6733	0.6262	0.8637
-0.6595	-0.0001	-0.5771	0.5869	0.3247	-0.5905	0.5958	0.6006	-0.6030	0.2580
0.3382	0.0975	0.1666	0.7467	0.4160	0.4086	0.5604	0.5718	0.0192	0.6411
-0.5652	0.5501	0.5489	0.5719	0.6424	0.3719	0.7503	0.9853	0.5872	-0.2132
-0.1495	-0.2110	-0.2029	-0.2132	-0.2440	-0.2317	-0.1512	-0.1864	-0.1329	-0.2075
-0.2409	-0.2214	-0.2249	-0.1848	-0.2453	-0.1706	-0.1957	-0.1546	-0.1975	-0.2141
-0.2241	-0.2193	-0.2278	-0.1931	-0.1142	-0.2229	-0.1593	-0.2162	0.8255	-0.1966
-0.2183	-0.2174	-0.2102	-0.1904	-0.2124	-0.1827	-0.2258	-0.2336	-0.1974	-0.2407
-0.2130	-0.2514	-0.2002	-0.1589	-0.1670	0.2331	-0.6747	-0.1256	-0.1942	-0.2112
-0.0778	0.5079	-0.6184	-0.1746	-0.2159	-0.2381				

Net Total: -0.00000000

M U L T I P O L E D E R I V E D C H A R G E A N A L Y S I S

See: M. Swart, P.Th. van Duijnen, J.G. Snijders, J.Comput.Chem., (2001), p. 79-88.

Multipole derived atomic charges (a.u.)

Atom Level: MDC-m MDC-d MDC-q

1	O	-0.350923	-0.318686	-0.342823
2	C	0.362799	0.374702	0.396217
3	C	0.242083	0.249702	0.253107
4	C	0.180636	0.166363	0.211904
5	O	-0.306272	-0.548494	-0.610629
6	N	-0.214575	-0.278448	-0.471283
7	O	-0.380629	-0.384545	-0.449928
8	C	0.250730	0.309593	0.308415
9	C	0.228006	0.193220	0.188032
10	C	0.339532	0.409380	0.415513
11	O	-0.383512	-0.356462	-0.397601
12	C	0.124338	0.102337	0.101938
13	O	-0.313207	-0.299979	-0.345037
14	C	0.245450	0.242420	0.247902
15	C	0.196296	0.159089	0.174874
16	O	-0.369802	-0.478027	-0.483300
17	C	0.227525	0.192819	0.198763
18	C	0.229740	0.193248	0.194873
19	O	-0.319218	-0.320574	-0.372684
20	C	0.057018	0.044625	-0.057123
21	C	0.195033	0.221195	0.283304
22	C	0.118673	0.104942	0.107664
23	C	0.119851	0.099635	0.111250
24	C	0.290706	0.268683	0.286653
25	C	0.172935	0.134885	0.136917
26	C	0.164842	0.124901	0.164647
27	C	0.249241	0.239059	0.261898
28	C	0.234510	0.193794	0.303974
29	C	0.034503	0.027685	-0.044392
30	C	0.236875	0.191055	0.176955

31	O	-0.372285	-0.476420	-0.515681
32	C	0.254612	0.243481	0.263571
33	C	0.222101	0.185349	0.192814
34	C	0.228329	0.295594	0.382787
35	C	0.222021	0.192126	0.201596
36	C	0.180432	0.145570	0.150222
37	C	0.293873	0.271154	0.310684
38	C	0.368548	0.339967	0.388111
39	C	0.229449	0.190117	0.156765
40	H	-0.073269	-0.060207	-0.026826
41	H	-0.057471	-0.031664	-0.019176
42	H	-0.064194	-0.056729	-0.032871
43	H	-0.056913	-0.036088	-0.016446
44	H	-0.068941	-0.052748	-0.051205
45	H	-0.070534	-0.057649	-0.053264
46	H	-0.076627	-0.072012	-0.070296
47	H	-0.071866	-0.061977	-0.062187
48	H	-0.063379	-0.041000	-0.021990
49	H	-0.082742	-0.062465	-0.070342
50	H	-0.055338	-0.047040	-0.049375
51	H	-0.072615	-0.055284	-0.060219
52	H	-0.070524	-0.058409	-0.064779
53	H	-0.067778	-0.063943	-0.073764
54	H	-0.058059	-0.045178	-0.052263
55	H	-0.054081	-0.043861	-0.046004
56	H	-0.057055	-0.042120	-0.030451
57	H	-0.067140	-0.050273	-0.051280
58	H	-0.061811	-0.036427	-0.032544
59	H	-0.075241	-0.060434	-0.049869
60	H	-0.074736	-0.065307	-0.055979
61	H	-0.068886	-0.055049	-0.043271

62	H	-0.066980	-0.048602	-0.040075
63	H	-0.072491	-0.074806	-0.083116
64	H	-0.058084	-0.035803	-0.033751
65	H	-0.074812	-0.049326	-0.058039
66	H	-0.072368	-0.063932	-0.060794
67	H	-0.067584	-0.054086	-0.067294
68	H	-0.064935	-0.056338	-0.056012
69	C	0.353192	0.437820	0.457876
70	H	-0.064631	-0.054190	-0.082337
71	H	-0.068092	-0.067148	-0.097820
72	H	-0.063223	-0.048399	-0.074348
73	H	-0.080770	-0.062389	-0.072685
74	H	-0.072870	-0.059955	-0.063631
75	H	-0.069555	-0.068847	-0.073238
76	H	-0.076744	-0.047244	-0.041222
77	H	-0.066446	-0.054212	-0.038826
78	H	-0.064988	-0.060424	-0.028939
79	H	-0.082660	-0.064021	-0.046894
80	H	-0.066750	-0.057968	-0.051658
81	H	-0.067865	-0.066605	-0.056123
82	H	-0.071055	-0.044422	-0.037735
83	H	-0.062030	-0.026297	-0.019170
84	H	-0.047124	-0.002720	-0.015390
85	H	-0.073792	-0.059948	-0.041366
86	H	0.062435	0.262451	0.293517
87	O	-0.364878	-0.442209	-0.457481
88	H	-0.044786	0.001027	0.021828
89	H	-0.045117	-0.029855	-0.016596
90	H	-0.052011	-0.050106	-0.039160
91	H	-0.042329	-0.010433	-0.010694
92	C	0.240958	0.197739	0.183301

93	O	-0.362109	-0.461584	-0.488740
94	H	-0.061231	-0.053706	-0.049842
95	H	-0.060817	-0.047177	-0.038621
96	H	-0.068532	-0.065475	-0.061394

*** Using NEW gradient routines ***

Geometry CYCLE 18

==============

Energy gradients wrt nuclear displacements

==

Atom	Cartesian (a.u./angstrom)		
	X	Y	Z
1 O	0.000429	-0.002164	0.000503
2 C	0.000358	-0.000225	0.000039
3 C	-0.000847	-0.000210	-0.001578
4 C	0.000332	0.000420	-0.000109
5 O	0.000870	-0.000288	-0.001162
6 N	-0.000104	0.000324	0.000374
7 O	0.000673	0.000546	0.001278
8 C	0.000865	-0.000178	-0.000417
9 C	-0.000251	0.000076	-0.000352
10 C	0.001539	0.003302	0.000595
11 O	-0.001488	-0.000202	0.001040
12 C	-0.000055	-0.000575	0.000542
13 O	0.000363	-0.000759	-0.001377
14 C	0.000187	0.000196	-0.002137
15 C	0.001124	-0.000189	0.001027

16	O	-0.001193	0.001071	0.000482
17	C	0.000552	0.000349	-0.000686
18	C	0.000203	-0.000296	0.000072
19	O	-0.003060	0.000550	0.000634
20	C	0.000027	-0.000349	0.000980
21	C	-0.000099	-0.000312	0.000641
22	C	0.000477	-0.000695	-0.000137
23	C	0.000792	-0.000346	-0.000779
24	C	0.000030	-0.000380	0.000445
25	C	-0.000252	-0.000207	0.000116
26	C	0.000785	-0.000171	0.000160
27	C	-0.000754	0.000535	0.000199
28	C	0.000202	-0.000133	-0.000168
29	C	-0.000788	0.000089	-0.000532
30	C	-0.000450	-0.000127	0.000236
31	O	0.001103	0.000993	-0.000716
32	C	-0.000339	0.000525	-0.000346
33	C	-0.000389	-0.000641	-0.000927
34	C	-0.000866	-0.000357	0.001572
35	C	0.000096	0.000749	-0.000412
36	C	-0.000037	-0.000039	-0.000416
37	C	0.000125	-0.000245	-0.000127
38	C	0.000117	-0.000451	0.000020
39	C	0.000366	0.000139	-0.000009
40	H	-0.000067	0.000142	-0.000567
41	H	0.000274	0.000108	0.000996
42	H	-0.000012	-0.000306	-0.000171
43	H	0.000451	0.000356	-0.000005
44	H	0.000176	0.000150	0.000080
45	H	-0.000080	0.000097	-0.000307
46	H	0.000006	0.000004	-0.000291

47 H	0.000049	0.000256	0.000263
48 H	0.000113	0.000397	-0.000019
49 H	-0.000299	-0.000838	0.000055
50 H	0.000030	0.000264	0.000030
51 H	0.000040	0.000081	0.000404
52 H	-0.000051	0.000193	0.000204
53 H	0.000053	0.000358	-0.000016
54 H	-0.000131	-0.000062	0.000404
55 H	-0.000405	-0.000172	-0.000244
56 H	-0.000260	-0.000075	-0.000345
57 H	0.000266	-0.000251	0.000039
58 H	-0.000059	-0.000170	0.000186
59 H	-0.000216	0.000157	0.000220
60 H	-0.000206	0.000016	-0.000248
61 H	-0.000051	-0.000295	0.000024
62 H	0.000058	-0.000235	-0.000122
63 H	-0.000092	0.000038	-0.000155
64 H	0.000345	0.000008	0.000182
65 H	-0.000140	-0.000396	-0.000513
66 H	-0.000152	-0.000052	0.000377
67 H	0.000334	0.000322	0.000430
68 H	0.000027	-0.000381	-0.000118
69 C	0.001981	-0.001371	0.000782
70 H	0.000107	0.000263	-0.000153
71 H	0.000053	0.000114	0.000281
72 H	-0.000363	0.000077	-0.000107
73 H	-0.000173	-0.000082	0.000201
74 H	0.000254	0.000261	-0.000078
75 H	-0.000093	0.000192	0.000334
76 H	0.000187	0.000099	-0.000289
77 H	0.000083	0.000244	-0.000093

78 H 0.000305 0.000066 0.000111
79 H 0.000072 -0.000085 0.000187
80 H -0.000089 -0.000162 0.000159
81 H 0.000244 0.000013 0.000248
82 H -0.000380 -0.000533 -0.000028
83 H -0.000620 -0.000353 -0.000246
84 H 0.000288 -0.000157 -0.000269
85 H -0.000175 0.000003 0.000152
86 H -0.000196 0.000210 0.000077
87 O -0.001117 -0.000383 0.001550
88 H -0.000038 -0.000321 0.000297
89 H 0.000650 0.000022 -0.000105
90 H -0.000107 -0.000062 0.000352
91 H -0.000500 -0.000142 0.000019
92 C -0.000580 0.000224 -0.000498
93 O -0.000193 0.001616 -0.001777
94 H 0.000130 0.000081 -0.000307
95 H -0.000163 0.000229 0.000081
96 H -0.000210 -0.000100 -0.000221
----

dE(predicted): -0.12917E-03, dE(actual): -0.59767E-04, Trust radius: 0.195959, all in a.u.

Geometry Convergence after Step 18 (Hartree/Angstrom,Angstrom)
----
current energy -19.67510813 Hartree
energy change -0.00005977 0.00100000 T
constrained gradient max 0.00330182 0.00100000 F
constrained gradient rms 0.00058665 0.00066667 T
gradient max 0.00330182

gradient rms 0.00058665

cart. step max 0.03266930 0.01000000 F

cart. step rms 0.00857285 0.00666667 F

Coordinates (Cartesian)

======================

Atom	bohr X	bohr Y	bohr Z	angstrom X	angstrom Y	angstrom Z	Geometric Variables (0:frozen, *:LT par.)		
1 O	26.561427	61.669794	50.464904	14.055702	32.634249	26.704877	1	2	3
2 C	27.037120	61.970276	53.042929	14.307428	32.793258	28.069109	4	5	6
3 C	29.830936	61.247438	53.442879	15.785852	32.410748	28.280754	7	8	9
4 C	30.583317	61.556957	56.246916	16.183994	32.574539	29.764586	10	11	12
5 O	27.280038	52.911604	43.116215	14.435974	27.999615	22.816118	13	14	15
6 N	33.216880	60.923694	56.913114	17.577616	32.239430	30.117123	16	17	18
7 O	26.644794	64.563038	53.748687	14.099818	34.165288	28.442580	19	20	21
8 C	20.445240	58.500456	48.382055	10.819155	30.957108	25.602681	22	23	24
9 C	25.710838	65.387635	41.492771	13.605589	34.601646	21.957029	25	26	27
10 C	21.627995	55.030083	45.057627	11.445042	29.120666	23.843469	28	29	30
11 O	21.714675	62.747293	45.667561	11.490911	33.204437	24.166232	31	32	33
12 C	19.533824	56.570951	46.311531	10.336854	29.936058	24.507007	34	35	36
13 O	21.581343	55.189341	42.520508	11.420355	29.204942	22.500884	37	38	39
14 C	23.979243	61.570621	49.550798	12.689269	32.581769	26.221153	40	41	42
15 C	24.005859	63.276427	47.115041	12.703354	33.484443	24.932206	43	44	45
16 O	18.816574	59.574441	49.616970	9.957302	31.525436	26.256170	46	47	48
17 C	23.739863	57.025175	51.327354	12.562595	30.176423	27.161266	49	50	51
18 C	27.401462	53.187316	38.620703	14.500229	28.145515	20.437196	52	53	54
19 O	30.128469	58.617390	52.652797	15.943299	31.018987	27.862660	55	56	57

20 C	26.343346	57.067677	41.129434	13.940298	30.198914	21.764759	58	59	60
21 C	26.075145	54.187589	41.028948	13.798372	28.674837	21.711584	61	62	63
22 C	23.245508	58.779314	49.024687	12.300993	31.104674	25.942747	64	65	66
23 C	25.952823	62.681244	42.506444	13.733643	33.169486	22.493442	67	68	69
24 C	35.169691	62.006740	55.259796	18.610999	32.812554	29.242225	70	71	72
25 C	26.295117	62.476529	45.418740	13.914777	33.061155	24.034562	73	74	75
26 C	29.907108	64.293399	57.046109	15.826160	34.022601	30.187501	76	77	78
27 C	27.145190	64.974193	56.426536	14.364616	34.382862	29.859637	79	80	81
28 C	26.492373	67.728729	56.975087	14.019160	35.840500	30.149917	82	83	84
29 C	28.397871	58.448259	41.711864	15.027506	30.929487	22.072968	85	86	87
30 C	17.657553	57.835073	44.474624	9.343975	30.605003	23.534958	88	89	90
31 O	30.019649	62.442403	40.416079	15.885714	33.043097	21.387268	91	92	93
32 C	23.187292	53.523862	40.870160	12.270186	28.323608	21.627558	94	95	96
33 C	30.959991	57.482858	42.525869	16.383321	30.418618	22.503721	97	98	99
34 C	28.257957	61.298629	41.394263	14.953467	32.437837	21.904901	100	101	102
35 C	24.246733	66.063329	47.922591	12.830819	34.959208	25.359543	103	104	105
36 C	22.513402	50.732717	41.257220	11.913580	26.846598	21.832381	106	107	108
37 C	33.677073	58.227579	57.403122	17.821140	30.812708	30.376424	109	110	111
38 C	19.479933	64.184311	46.257792	10.308336	33.964875	24.478569	112	113	114
39 C	19.667548	50.183013	41.020875	10.407618	26.555707	21.707312	115	116	117
40 H	24.662983	58.082301	40.513000	13.051089	30.735830	21.438556	118	119	120
41 H	31.475065	58.312858	44.359378	16.655887	30.857836	23.473972	121	122	123
42 H	31.007470	55.430862	42.690142	16.408447	29.332749	22.590650	124	125	126
43 H	32.388853	58.133945	41.171632	17.139443	30.763159	21.787089	127	128	129

44 H	27.290946	66.565696	42.127344	14.441747	35.225050	22.292831	130	131	132
45 H	25.725897	65.395670	39.421372	13.613558	34.605898	20.860892	133	134	135
46 H	23.936397	66.235513	42.134635	12.666596	35.050324	22.296689	136	137	138
47 H	24.240135	61.619796	42.025201	12.827327	32.607792	22.238779	139	140	141
48 H	27.946469	63.583816	45.989914	14.788635	33.647106	24.336815	142	143	144
49 H	26.783542	60.516400	45.847478	14.173240	32.023900	24.261441	145	146	147
50 H	26.005867	66.293798	48.979200	13.761712	35.081167	25.918676	148	149	150
51 H	22.704629	66.641515	49.174742	12.014772	35.265171	26.022153	151	152	153
52 H	24.288236	67.313906	46.278958	12.852781	35.620985	24.489770	154	155	156
53 H	19.720681	66.191045	45.790656	10.435735	35.026792	24.231372	157	158	159
54 H	18.917657	63.981421	48.244638	10.010793	33.857510	25.529963	160	161	162
55 H	17.982748	63.395062	45.074685	9.516060	33.547222	23.852496	163	164	165
56 H	22.515553	54.143685	39.011194	11.914717	28.651604	20.643835	166	167	168
57 H	23.550616	49.658986	39.819448	12.462449	26.278404	21.071544	169	170	171
58 H	23.223171	50.092614	43.087958	12.289173	26.507870	22.801165	172	173	174
59 H	18.583326	51.154975	42.494480	9.833872	27.070047	22.487110	175	176	177
60 H	18.921973	50.796866	39.184300	10.013077	26.880544	20.735439	178	179	180
61 H	19.307532	48.152096	41.209391	10.217106	25.480992	21.807071	181	182	183
62 H	27.203878	51.133300	38.468141	14.395672	27.058577	20.356463	184	185	186

63 H	26.632452	54.083516	36.920517	14.093287	28.619764	19.537496	187	188	189
64 H	29.413927	53.639637	38.737771	15.565180	28.384873	20.499146	190	191	192
65 H	24.392891	58.098134	47.448861	12.908162	30.744209	25.108856	193	194	195
66 H	22.601656	57.629981	52.949425	11.960281	30.496473	28.019629	196	197	198
67 H	25.735872	57.065568	51.846661	13.618837	30.197798	27.436072	199	200	201
68 H	23.237165	55.079019	50.849835	12.296578	29.146562	26.908574	202	203	204
69 C	31.071256	58.191954	50.252372	16.442200	30.793856	26.592410	205	206	207
70 H	27.695462	68.987957	55.852785	14.655808	36.506855	29.556021	208	209	210
71 H	26.781945	68.151894	58.982544	14.172395	36.064429	31.212218	211	212	213
72 H	24.515260	68.111786	56.496023	12.972917	36.043205	29.896408	214	215	216
73 H	25.876281	63.727250	57.525737	13.693138	33.723009	30.441309	217	218	219
74 H	31.142483	65.645539	56.071353	16.479893	34.738123	29.671682	220	221	222
75 H	30.248694	64.487285	59.080962	16.006920	34.125202	31.264299	223	224	225
76 H	35.268376	61.103363	53.376422	18.663221	32.334507	28.245586	226	227	228
77 H	34.847005	64.031278	54.983152	18.440241	33.883893	29.095831	229	230	231
78 H	37.016789	61.786673	56.173124	19.588441	32.696099	29.725537	232	233	234
79 H	33.675486	57.046418	55.680191	17.820300	30.187664	29.464688	235	236	237
80 H	32.229246	57.492824	58.688910	17.054983	30.423892	31.056834	238	239	240
81 H	35.520059	58.018751	58.331351	18.796406	30.702201	30.867621	241	242	243

82 H 25.791269 60.761789 54.206541 13.648152 32.153754 28.684866 244 245 246

83 H 22.683374 62.376045 50.954282 12.003524 33.007982 26.963845 247 248 249

84 H 30.971350 62.455583 52.214486 16.389333 33.050071 27.630716 250 251 252

85 H 29.387619 60.265952 57.351975 15.551258 31.891368 30.349358 253 254 255

86 H 26.153227 53.106027 44.579293 13.839692 28.102499 23.590346 256 257 258

87 O 23.140595 53.758361 46.280576 12.245476 28.447699 24.490626 259 260 261

88 H 18.509950 55.159600 47.449122 9.795044 29.189203 25.108994 262 263 264

89 H 32.195811 54.892748 48.193973 17.037289 29.047991 25.503152 265 266 267

90 H 30.612792 54.208630 51.131926 16.199592 28.685971 27.057850 268 269 270

91 H 28.889473 55.326205 48.477127 15.287651 29.277367 25.652991 271 272 273

92 C 30.694277 55.461625 49.491064 16.242712 29.349028 26.189543 274 275 276

93 O 32.033477 59.803092 48.932943 16.951386 31.646433 25.894198 277 278 279

94 H 18.639831 59.195670 43.269212 9.863774 31.325000 22.897081 280 281 282

95 H 16.203406 58.818867 45.563912 8.574473 31.125604 24.111384 283 284 285

96 H 16.755960 56.420687 43.265180 8.866872 29.856542 22.894947 286 287 288

Number of elements of the density matrix on this node (used, total): 222269 1773786

Numerical Integration : Fuzzy Cells (Becke)

Becke grid quality: NORMAL

Lebedev angular grid order: min: 11 max: 23

Nr. of radial points: min: 46 max: 52

Total nr. of points: 600120

Nr. of blocks: 4689

Block length: 128

Nr. of dummy points: 72

Test of Precision of the Numerical Integration Grid

==

Integral of the Total Core Density: 85.99994161018185

Relative Error: -6.790E-07

Density Fitting (zlmFit)

ZlmFit Fit Quality: NORMAL

Max L-expansion: min: 6 max: 7

Nr. of radial interpolation points: min: 39 max: 48

S C F *** ScaSCF ***

Initial density matrix is taken from previous geometry

CYCLE   1

SCF Error: norm([F,P])=   0.042474681424, max([F,P])=   0.004230318835

orbitals (Q,E):

A :114...133   (2.00   -0.2481) (2.00   -0.2473) (2.00   -0.2372) (2.00   -0.2348)

(2.00   -0.2269) (2.00   -0.2211) (2.00   -0.2196) (2.00   -0.2150)

(2.00   -0.1941) (2.00   -0.1770) (0.00   -0.0690) (0.00   -0.0677)

(0.00   -0.0623) (0.00   -0.0391) (0.00   -0.0185) (0.00   -0.0128)

(0.00   -0.0106) (0.00   -0.0071) (0.00   -0.0046) (0.00   -0.0001)

CYCLE   2

MIX coefficients: 0.8000 0.2000

SCF Error: norm([F,P])=   0.007700366058, max([F,P])=   0.000702850282

orbitals (Q,E):

A :114...124   (2.00   -0.2482) (2.00   -0.2474) (2.00   -0.2373) (2.00   -0.2349)

(2.00   -0.2269) (2.00   -0.2212) (2.00   -0.2197) (2.00   -0.2150)

(2.00   -0.1943) (2.00   -0.1769) (0.00   -0.0691)

CYCLE   3

SDIIS (wt 0.000): -0.6474 1.6474

A-DIIS (wt 1.000): 0.0000 1.0000

DIIS coefficients: 0.0000 1.0000

SCF Error: norm([F,P])=   0.003934162471, max([F,P])=   0.000315246931

orbitals (Q,E):

A :114...124   (2.00   -0.2483) (2.00   -0.2476) (2.00   -0.2373) (2.00   -0.2350)

(2.00   -0.2268) (2.00   -0.2215) (2.00   -0.2201) (2.00   -0.2153)

(2.00   -0.1947) (2.00   -0.1769) (0.00   -0.0694)

CYCLE   4

SDIIS (wt 1.000): 0.4559 -0.1089 0.6531

DIIS coefficients: 0.4559 -0.1089 0.6531

SCF Error: norm([F,P])=   0.004867416981, max([F,P])=   0.000475329570

orbitals (Q,E):

A :114...124   (2.00   -0.2482) (2.00   -0.2475) (2.00   -0.2373) (2.00   -0.2350)

(2.00   -0.2268) (2.00   -0.2213) (2.00   -0.2199) (2.00   -0.2151)

(2.00   -0.1944) (2.00   -0.1770) (0.00   -0.0692)

CYCLE   5

SDIIS (wt 1.000): 0.4409 -0.3485 0.4767 0.4308

DIIS coefficients: 0.4409 -0.3485 0.4767 0.4308

SCF Error: norm([F,P])=   0.001690952432, max([F,P])=   0.000268543533

orbitals (Q,E):

A :114...124   (2.00   -0.2483) (2.00   -0.2475) (2.00   -0.2373) (2.00   -0.2350)

(2.00   -0.2268) (2.00   -0.2214) (2.00   -0.2199) (2.00   -0.2152)

(2.00   -0.1945) (2.00   -0.1770) (0.00   -0.0693)

CYCLE   6

SDIIS (wt 1.000): 0.2304 -0.2088 0.2314 0.2735 0.4735

DIIS coefficients: 0.2304 -0.2088 0.2314 0.2735 0.4735

SCF Error: norm([F,P])= 0.000452493415, max([F,P])= 0.000064893626

orbitals (Q,E):

A :114...124 (2.00 -0.2483) (2.00 -0.2475) (2.00 -0.2373) (2.00 -0.2350)

(2.00 -0.2268) (2.00 -0.2213) (2.00 -0.2199) (2.00 -0.2152)

(2.00 -0.1945) (2.00 -0.1769) (0.00 -0.0693)

CYCLE 7

SDIIS (wt 1.000): 0.0875 -0.0860 0.0836 0.1160 0.3036 0.4952

DIIS coefficients: 0.0875 -0.0860 0.0836 0.1160 0.3036 0.4952

SCF Error: norm([F,P])= 0.000158545080, max([F,P])= 0.000021237785

orbitals (Q,E):

A :114...124 (2.00 -0.2483) (2.00 -0.2475) (2.00 -0.2373) (2.00 -0.2350)

(2.00 -0.2268) (2.00 -0.2213) (2.00 -0.2199) (2.00 -0.2152)

(2.00 -0.1945) (2.00 -0.1769) (0.00 -0.0693)

CYCLE 8

SDIIS (wt 1.000): 0.0373 -0.0384 0.0346 0.0529 0.1656 0.3482 0.3998

DIIS coefficients: 0.0373 -0.0384 0.0346 0.0529 0.1656 0.3482 0.3998

SCF Error: norm([F,P])= 0.000068016673, max([F,P])= 0.000009028551

orbitals (Q,E):

A :114...124 (2.00 -0.2483) (2.00 -0.2475) (2.00 -0.2373) (2.00 -0.2350)

(2.00 -0.2268) (2.00 -0.2213) (2.00 -0.2199) (2.00 -0.2152)

(2.00 -0.1945) (2.00 -0.1770) (0.00 -0.0693)

CYCLE 9

SDIIS (wt 1.000): 0.0124 -0.0137 0.0111 0.0191 0.0712 0.1762 0.3113 0.4124

DIIS coefficients: 0.0124 -0.0137 0.0111 0.0191 0.0712 0.1762 0.3113 0.4124

SCF Error: norm([F,P])= 0.000030740439, max([F,P])= 0.000003634309

orbitals (Q,E):

A :114...124 (2.00 -0.2483) (2.00 -0.2475) (2.00 -0.2373) (2.00 -0.2350)

(2.00 -0.2268) (2.00 -0.2213) (2.00 -0.2199) (2.00 -0.2152)

(2.00 -0.1945) (2.00 -0.1770) (0.00 -0.0693)

CYCLE 10

SDIIS (wt 1.000): 0.0028 -0.0035 0.0023 0.0049 0.0231 0.0656 0.1460 0.2700 0.4888

DIIS coefficients: 0.0028 -0.0035 0.0023 0.0049 0.0231 0.0656 0.1460 0.2700 0.4888

SCF Error: norm([F,P])= 0.000008563760, max([F,P])= 0.000000903100

orbitals (Q,E):

A :114...124 (2.00 -0.2483) (2.00 -0.2475) (2.00 -0.2373) (2.00 -0.2350)

(2.00 -0.2268) (2.00 -0.2213) (2.00 -0.2199) (2.00 -0.2152)

(2.00 -0.1945) (2.00 -0.1770) (0.00 -0.0693)

SCF CONVERGED

CYCLE 11

*** Setting up for NEW gradients in Focky

*** Using FIT density in Focky

*** Setting up for NEW Hessian in Focky

orbitals (Q,E):

A :114...133 (2.00 -0.2483) (2.00 -0.2475) (2.00 -0.2373) (2.00 -0.2350)

(2.00 -0.2268) (2.00 -0.2213) (2.00 -0.2199) (2.00 -0.2152)

(2.00 -0.1945) (2.00 -0.1770) (0.00 -0.0693) (0.00 -0.0681)

(0.00 -0.0625) (0.00 -0.0391) (0.00 -0.0186) (0.00 -0.0128)

(0.00 -0.0108) (0.00 -0.0071) (0.00 -0.0046) (0.00 -0.0001)

Fit test: (difference of exact and fit density, squared integrated, result summed over spins)

SCF: 0.00100901993062

Gross Charges per Atom (Z minus electrons)

===

-0.6654	0.9226	0.5471	0.3315	-0.5806	-0.3812	-0.6309	0.6741	0.6266	0.8640
-0.6595	-0.0006	-0.5770	0.5875	0.3250	-0.5908	0.5963	0.6010	-0.6034	0.2581
0.3384	0.0980	0.1664	0.7470	0.4165	0.4089	0.5603	0.5719	0.0191	0.6423
-0.5655	0.5501	0.5499	0.5729	0.6422	0.3721	0.7505	0.9859	0.5876	-0.2130
-0.1512	-0.2110	-0.2035	-0.2135	-0.2443	-0.2316	-0.1508	-0.1867	-0.1338	-0.2076
-0.2408	-0.2216	-0.2252	-0.1842	-0.2461	-0.1707	-0.1956	-0.1555	-0.1977	-0.2141
-0.2241	-0.2196	-0.2278	-0.1930	-0.1134	-0.2226	-0.1617	-0.2168	0.8262	-0.1967
-0.2184	-0.2175	-0.2103	-0.1904	-0.2124	-0.1833	-0.2257	-0.2336	-0.1972	-0.2407
-0.2131	-0.2506	-0.1999	-0.1597	-0.1672	0.2331	-0.6748	-0.1258	-0.1939	-0.2115
-0.0786	0.5088	-0.6185	-0.1747	-0.2167	-0.2381				

Net Total: -0.00000000

M U L T I P O L E D E R I V E D C H A R G E A N A L Y S I S

See: M. Swart, P.Th. van Duijnen, J.G. Snijders, J.Comput.Chem., (2001), p. 79-88.

Multipole derived atomic charges (a.u.)

Atom Level: MDC-m MDC-d MDC-q

1	O	-0.350481	-0.318211	-0.342674
2	C	0.362382	0.374207	0.395718
3	C	0.242233	0.249981	0.253158
4	C	0.180752	0.166607	0.212285
5	O	-0.306748	-0.549248	-0.610959
6	N	-0.214516	-0.278608	-0.472315
7	O	-0.380292	-0.384772	-0.449706
8	C	0.250582	0.309614	0.308113
9	C	0.227987	0.193252	0.187909
10	C	0.339350	0.409122	0.415753
11	O	-0.382964	-0.355905	-0.396896
12	C	0.124255	0.102419	0.102084
13	O	-0.312913	-0.298416	-0.343473
14	C	0.245279	0.242297	0.247796
15	C	0.196160	0.159242	0.174838
16	O	-0.369299	-0.477717	-0.482762
17	C	0.227673	0.193031	0.198895
18	C	0.229705	0.193299	0.195004
19	O	-0.319759	-0.321046	-0.372728
20	C	0.057122	0.044537	-0.057751
21	C	0.195108	0.221297	0.283208
22	C	0.118628	0.104894	0.107565
23	C	0.119722	0.099393	0.110963
24	C	0.290777	0.268850	0.286801
25	C	0.173098	0.135076	0.137130
26	C	0.164839	0.124922	0.164388
27	C	0.249099	0.239081	0.262257
28	C	0.234523	0.193871	0.303493
29	C	0.034558	0.027748	-0.043859
30	C	0.236948	0.191255	0.177329
31	O	-0.372101	-0.476527	-0.516194

32	C	0.254557	0.243257	0.263233
33	C	0.222251	0.185502	0.194555
34	C	0.228190	0.295852	0.383863
35	C	0.221986	0.192244	0.201759
36	C	0.180498	0.145525	0.150124
37	C	0.293900	0.271223	0.310555
38	C	0.368366	0.339798	0.387757
39	C	0.229470	0.190069	0.155843
40	H	-0.073284	-0.060057	-0.026679
41	H	-0.057387	-0.031554	-0.019029
42	H	-0.064218	-0.056834	-0.033737
43	H	-0.056935	-0.036055	-0.017247
44	H	-0.068981	-0.052795	-0.051256
45	H	-0.070599	-0.057803	-0.053351
46	H	-0.076656	-0.072078	-0.070355
47	H	-0.071695	-0.061847	-0.062194
48	H	-0.063239	-0.040888	-0.021957
49	H	-0.082631	-0.062417	-0.070169
50	H	-0.055325	-0.047070	-0.049630
51	H	-0.072612	-0.055250	-0.060190
52	H	-0.070619	-0.058597	-0.065076
53	H	-0.067833	-0.064000	-0.073625
54	H	-0.057956	-0.045075	-0.052153
55	H	-0.054166	-0.044012	-0.046177
56	H	-0.057138	-0.042319	-0.030711
57	H	-0.066982	-0.049978	-0.050682
58	H	-0.061967	-0.036558	-0.032642
59	H	-0.075261	-0.060553	-0.049806
60	H	-0.074668	-0.065139	-0.055632
61	H	-0.068858	-0.055044	-0.043038
62	H	-0.067074	-0.048783	-0.040264

63	H	-0.072531	-0.074765	-0.083242
64	H	-0.058117	-0.035868	-0.033689
65	H	-0.074649	-0.049180	-0.058044
66	H	-0.072353	-0.063888	-0.060813
67	H	-0.067507	-0.053947	-0.067270
68	H	-0.064960	-0.056421	-0.056111
69	C	0.353182	0.438247	0.458298
70	H	-0.064688	-0.054283	-0.082411
71	H	-0.068162	-0.067248	-0.097745
72	H	-0.063228	-0.048409	-0.074188
73	H	-0.080864	-0.062421	-0.072837
74	H	-0.072894	-0.059968	-0.063374
75	H	-0.069520	-0.068837	-0.073144
76	H	-0.076762	-0.047188	-0.041204
77	H	-0.066465	-0.054290	-0.038705
78	H	-0.064957	-0.060361	-0.028823
79	H	-0.082525	-0.063847	-0.046619
80	H	-0.066770	-0.058022	-0.051546
81	H	-0.067861	-0.066650	-0.056013
82	H	-0.071247	-0.044440	-0.037810
83	H	-0.061928	-0.026315	-0.018940
84	H	-0.047297	-0.002901	-0.015649
85	H	-0.073746	-0.059951	-0.041261
86	H	0.062321	0.262504	0.293242
87	O	-0.364678	-0.442505	-0.457852
88	H	-0.045011	0.000454	0.021255
89	H	-0.045182	-0.029838	-0.016114
90	H	-0.051993	-0.050072	-0.038625
91	H	-0.042532	-0.010888	-0.010782
92	C	0.240885	0.197744	0.181609
93	O	-0.362036	-0.462048	-0.488707

94	H	-0.061222	-0.053585	-0.049702
95	H	-0.060991	-0.047628	-0.039113
96	H	-0.068552	-0.065489	-0.061525

*** Using NEW gradient routines ***

Geometry CYCLE 19

==============

Energy gradients wrt nuclear displacements

==

Atom		Cartesian (a.u./angstrom) X	Y	Z
1	O	-0.000091	-0.001271	-0.000270
2	C	0.000661	-0.000845	-0.000174
3	C	-0.000765	0.000253	-0.000270
4	C	0.000264	0.000163	-0.000133
5	O	0.000056	-0.000397	-0.000673
6	N	-0.000079	0.000190	0.000375
7	O	0.000071	0.000448	0.000131
8	C	-0.000510	0.000486	-0.000237
9	C	-0.000023	0.000135	-0.000138
10	C	-0.000256	-0.000130	0.000198
11	O	-0.001060	-0.000482	0.000444
12	C	0.000081	0.000271	0.000594
13	O	0.000393	0.000745	-0.000272
14	C	0.000186	0.001095	-0.000749
15	C	0.000971	0.000010	0.000258
16	O	0.000453	0.000426	-0.000551

17	C	0.000479	0.000456	-0.000378
18	C	0.000050	-0.000208	0.000042
19	O	-0.001411	0.000635	0.000067
20	C	-0.000298	-0.000164	0.000587
21	C	0.000138	0.000125	0.000276
22	C	0.000499	-0.000261	0.000191
23	C	0.000224	-0.000450	-0.000802
24	C	0.000155	-0.000242	0.000348
25	C	0.000152	-0.000198	0.000329
26	C	0.000735	-0.000265	0.000085
27	C	-0.000293	0.000024	0.000020
28	C	0.000057	-0.000132	-0.000262
29	C	-0.000553	0.000246	-0.000530
30	C	0.000069	-0.000373	-0.000138
31	O	-0.000355	0.000170	-0.000040
32	C	-0.000196	-0.000147	0.000021
33	C	-0.000170	-0.000365	-0.000360
34	C	0.000864	0.000138	0.000801
35	C	-0.000203	0.000319	0.000084
36	C	0.000104	-0.000231	-0.000393
37	C	0.000224	-0.000251	0.000002
38	C	0.000386	-0.000649	0.000330
39	C	0.000286	0.000081	-0.000053
40	H	-0.000129	0.000133	-0.000282
41	H	0.000306	-0.000075	0.000612
42	H	0.000152	0.000144	-0.000038
43	H	0.000201	0.000394	0.000046
44	H	0.000077	0.000099	0.000047
45	H	-0.000046	0.000043	-0.000174
46	H	-0.000002	-0.000035	-0.000216
47	H	-0.000068	-0.000061	0.000209

48	H	0.000129	0.000117	0.000016
49	H	-0.000167	-0.000551	0.000047
50	H	0.000029	0.000244	0.000070
51	H	-0.000040	0.000017	0.000332
52	H	-0.000120	-0.000072	0.000180
53	H	0.000123	0.000316	0.000086
54	H	-0.000075	-0.000172	0.000403
55	H	-0.000176	-0.000062	-0.000117
56	H	-0.000220	0.000092	-0.000135
57	H	0.000193	-0.000214	0.000027
58	H	-0.000052	0.000015	-0.000006
59	H	-0.000135	0.000107	0.000125
60	H	-0.000164	0.000000	-0.000159
61	H	-0.000043	-0.000154	0.000009
62	H	0.000005	-0.000122	-0.000111
63	H	-0.000070	0.000004	-0.000050
64	H	0.000144	-0.000016	0.000114
65	H	-0.000254	-0.000176	-0.000420
66	H	-0.000051	0.000009	0.000253
67	H	0.000137	0.000526	0.000495
68	H	0.000024	-0.000088	-0.000103
69	C	0.000293	-0.000100	-0.000791
70	H	0.000045	0.000158	-0.000088
71	H	0.000035	0.000014	0.000144
72	H	-0.000190	0.000042	-0.000066
73	H	-0.000069	-0.000002	0.000126
74	H	0.000104	0.000157	-0.000032
75	H	-0.000040	0.000108	0.000222
76	H	0.000183	0.000168	-0.000120
77	H	0.000070	0.000105	-0.000057
78	H	0.000186	0.000053	0.000066

79 H   0.000069 0.000014 0.000227

80 H   -0.000018 -0.000081 0.000045

81 H   0.000137 0.000023 0.000182

82 H   -0.000596 -0.000603 -0.000170

83 H   -0.000578 -0.000213 -0.000106

84 H   -0.000021 -0.000126 0.000054

85 H   -0.000191 0.000115 0.000123

86 H   -0.000172 -0.000562 -0.000414

87 O   -0.000559 0.001118 0.001205

88 H   0.000163 -0.000371 0.000089

89 H   0.000396 -0.000004 -0.000051

90 H   -0.000072 -0.000025 0.000219

91 H   -0.000156 0.000047 0.000169

92 C   0.000156 0.000157 -0.000327

93 O   -0.000227 0.000028 -0.000290

94 H   0.000167 -0.000037 -0.000268

95 H   0.000001 0.000107 0.000076

96 H   -0.000116 -0.000108 -0.000180

----

dE(predicted):   -0.62217E-04, dE(actual):   -0.21964E-04, Trust radius: 0.195959, all in a.u.

Geometry Convergence after Step 19   (Hartree/Angstrom,Angstrom)

----

current energy   -19.67513009 Hartree

energy change   -0.00002196   0.00100000   T

constrained gradient max   0.00141139   0.00100000   F

constrained gradient rms   0.00033208   0.00066667   T

gradient max   0.00141139

gradient rms   0.00033208

cart. step max 0.07556783 0.01000000 F

cart. step rms 0.01598329 0.00666667 F

Coordinates (Cartesian)

=======================

Atom	bohr X	Y	Z	angstrom X	Y	Z	Geometric Variables (0:frozen, *:LT par.)		
1 O	26.540284	61.684957	50.462459	14.044513	32.642274	26.703583	1	2	3
2 C	27.026708	61.980506	53.039292	14.301918	32.798671	28.067185	4	5	6
3 C	29.816864	61.230763	53.427143	15.778405	32.401924	28.272427	7	8	9
4 C	30.591057	61.540562	56.224915	16.188090	32.565863	29.752943	10	11	12
5 O	27.309687	52.948324	43.162922	14.451664	28.019046	22.840834	13	14	15
6 N	33.228347	60.898056	56.869074	17.583684	32.225863	30.093818	16	17	18
7 O	26.657183	64.575536	53.747568	14.106374	34.171902	28.441988	19	20	21
8 C	20.427844	58.502200	48.380511	10.809949	30.958031	25.601864	22	23	24
9 C	25.689243	65.407231	41.492724	13.594162	34.612016	21.957004	25	26	27
10 C	21.628451	55.032172	45.061626	11.445284	29.121771	23.845586	28	29	30
11 O	21.698532	62.748594	45.656316	11.482369	33.205126	24.160282	31	32	33
12 C	19.527306	56.577695	46.299718	10.333406	29.939627	24.500755	34	35	36
13 O	21.596320	55.184289	42.524729	11.428281	29.202268	22.503117	37	38	39
14 C	23.956874	61.582573	49.550271	12.677432	32.588094	26.220874	40	41	42
15 C	23.983411	63.285496	47.110128	12.691475	33.489242	24.929606	43	44	45
16 O	18.793062	59.574966	49.609532	9.944860	31.525714	26.252234	46	47	48
17 C	23.717949	57.034057	51.329979	12.550998	30.181123	27.162655	49	50	51
18 C	27.446347	53.195966	38.664332	14.523982	28.150093	20.460283	52	53	54
19 O	30.082188	58.593115	52.647981	15.918808	31.006141	27.860112	55	56	57
20 C	26.358699	57.085195	41.143528	13.948423	30.208184	21.772218	58	59	60

21 C	26.105523	54.202955	41.061588	13.814448	28.682968	21.728857	61	62	63
22 C	23.226239	58.789096	49.026950	12.290797	31.109850	25.943945	64	65	66
23 C	25.939556	62.700717	42.504132	13.726622	33.179790	22.492218	67	68	69
24 C	35.169426	61.969527	55.193931	18.610858	32.792862	29.207370	70	71	72
25 C	26.279163	62.494822	45.416984	13.906334	33.070836	24.033633	73	74	75
26 C	29.933261	64.280626	57.027556	15.840000	34.015843	30.177683	76	77	78
27 C	27.172169	64.977055	56.424094	14.378893	34.384377	29.858345	79	80	81
28 C	26.537194	67.733487	56.984760	14.042878	35.843018	30.155036	82	83	84
29 C	28.406627	58.479158	41.719449	15.032140	30.945838	22.076982	85	86	87
30 C	17.669119	57.850657	44.450201	9.350095	30.613249	23.522033	88	89	90
31 O	30.003114	62.476650	40.402038	15.876964	33.061220	21.379838	91	92	93
32 C	23.222882	53.518749	40.894778	12.289020	28.320902	21.640585	94	95	96
33 C	30.974667	57.531520	42.535302	16.391088	30.444369	22.508712	97	98	99
34 C	28.251503	61.327704	41.393300	14.950052	32.453223	21.904391	100	101	102
35 C	24.216001	66.073943	47.914376	12.814556	34.964825	25.355196	103	104	105
36 C	22.567085	50.725723	41.300009	11.941987	26.842897	21.855023	106	107	108
37 C	33.681452	58.200057	57.356644	17.823457	30.798144	30.351829	109	110	111
38 C	19.460332	64.188467	46.227492	10.297964	33.967074	24.462535	112	113	114
39 C	19.724495	50.154323	41.072784	10.437753	26.540525	21.734781	115	116	117
40 H	24.669930	58.088530	40.531277	13.054765	30.739126	21.448228	118	119	120
41 H	31.486820	58.374640	44.363101	16.662108	30.890529	23.475942	121	122	123
42 H	31.032345	55.480447	42.709858	16.421610	29.358988	22.601084	124	125	126
43 H	32.398674	58.182347	41.175743	17.144640	30.788772	21.789265	127	128	129
44 H	27.265767	66.589490	42.128784	14.428422	35.237640	22.293592	130	131	132

45 H	25.704890	65.417355	39.421272	13.602442	34.617373	20.860839	133	134	135
46 H	23.912158	66.249411	42.135435	12.653769	35.057679	22.297112	136	137	138
47 H	24.231666	61.632791	42.019470	12.822845	32.614668	22.235746	139	140	141
48 H	27.925944	63.607486	45.990756	14.777773	33.659632	24.337260	142	143	144
49 H	26.775064	60.536877	45.845353	14.168754	32.034736	24.260316	145	146	147
50 H	25.969465	66.307863	48.979770	13.742449	35.088610	25.918978	148	149	150
51 H	22.665880	66.652408	49.156646	11.994267	35.270935	26.012577	151	152	153
52 H	24.265077	67.322228	46.268933	12.840526	35.625389	24.484465	154	155	156
53 H	19.703115	66.192911	45.751495	10.426440	35.027780	24.210648	157	158	159
54 H	18.887455	63.995668	48.212430	9.994811	33.865049	25.512919	160	161	162
55 H	17.969604	63.393032	45.040278	9.509105	33.546148	23.834289	163	164	165
56 H	22.556264	54.120749	39.028131	11.936261	28.639467	20.652797	166	167	168
57 H	23.608302	49.650847	39.865906	12.492975	26.274097	21.096129	169	170	171
58 H	23.285209	50.101009	43.133074	12.322002	26.512312	22.825040	172	173	174
59 H	18.636631	51.125064	42.544504	9.862080	27.054219	22.513582	175	176	177
60 H	18.970266	50.754165	39.235058	10.038632	26.857948	20.762298	178	179	180
61 H	19.379676	48.121667	41.271689	10.255283	25.464889	21.840038	181	182	183
62 H	27.266712	51.139106	38.527049	14.428923	27.061650	20.387637	184	185	186
63 H	26.673865	54.072781	36.955489	14.115201	28.614084	19.556002	187	188	189

64	H	29.454638	53.666592	38.782519	15.586723	28.399138	20.522825	190	191	192
65	H	24.377591	58.108369	47.453719	12.900065	30.749624	25.111427	193	194	195
66	H	22.582993	57.642075	52.953126	11.950405	30.502872	28.021587	196	197	198
67	H	25.714958	57.067071	51.845714	13.607770	30.198593	27.435571	199	200	201
68	H	23.209346	55.089157	50.853098	12.281857	29.151927	26.910301	202	203	204
69	C	31.037580	58.138652	50.257989	16.424380	30.765650	26.595382	205	206	207
70	H	27.741286	68.989711	55.860070	14.680056	36.507783	29.559876	208	209	210
71	H	26.839656	68.148686	58.992033	14.202934	36.062731	31.217239	211	212	213
72	H	24.559632	68.128869	56.517644	12.996397	36.052245	29.907849	214	215	216
73	H	25.902088	63.733426	57.525806	13.706795	33.726277	30.441346	217	218	219
74	H	31.170302	65.625844	56.045352	16.494614	34.727701	29.657923	220	221	222
75	H	30.288042	64.472449	59.060377	16.027741	34.117351	31.253406	223	224	225
76	H	35.246270	61.060832	53.312037	18.651523	32.312001	28.211515	226	227	228
77	H	34.852466	63.994878	54.915723	18.443131	33.864631	29.060149	229	230	231
78	H	37.024402	61.743644	56.089832	19.592470	32.673329	29.681461	232	233	234
79	H	33.656349	57.017227	55.634880	17.810173	30.172217	29.440711	235	236	237
80	H	32.243779	57.473175	58.658425	17.062673	30.413495	31.040702	238	239	240
81	H	35.533205	57.983424	58.265569	18.803362	30.683507	30.832811	241	242	243
82	H	25.776164	60.782189	54.207868	13.640159	32.164549	28.685568	244	245	246

83 H 22.661593 62.389317 50.953650 11.991999 33.015005 26.963510 247 248
249

84 H 30.961859 62.422597 52.187096 16.384310 33.032616 27.616222 250 251
252

85 H 29.399455 60.254876 57.340701 15.557522 31.885507 30.343392 253 254
255

86 H 26.174969 53.145189 44.619989 13.851197 28.123223 23.611881 256 257
258

87 O 23.131596 53.756788 46.293813 12.240713 28.446867 24.497631 259 260
261

88 H 18.489337 55.168787 47.427339 9.784136 29.194065 25.097467 262 263
264

89 H 32.124349 54.800590 48.240272 16.999473 28.999223 25.527653 265 266
267

90 H 30.469989 54.171348 51.150869 16.124024 28.666243 27.067874 268 269
270

91 H 28.825601 55.313207 48.456403 15.253851 29.270489 25.642024 271 272
273

92 C 30.612694 55.412811 49.505266 16.199540 29.323197 26.197059 274 275
276

93 O 32.070434 59.717221 48.951619 16.970943 31.600992 25.904081 277 278
279

94 H 18.663763 59.212122 43.255914 9.876438 31.333706 22.890044 280 281
282

95 H 16.208133 58.835263 45.529766 8.576975 31.134280 24.093314 283 284
285

96 H 16.773932 56.440963 43.230462 8.876383 29.867272 22.876575 286 287
288

Number of elements of the density matrix on this node (used, total): 222269 1773786

Numerical Integration : Fuzzy Cells (Becke)

Becke grid quality: NORMAL

Lebedev angular grid order: min: 11 max: 23

Nr. of radial points: min: 46 max: 52

Total nr. of points: 600102

Nr. of blocks: 4689

Block length: 128

Nr. of dummy points: 90

Test of Precision of the Numerical Integration Grid

===

Integral of the Total Core Density: 85.99994108388654

Relative Error: -6.851E-07

Density Fitting (zlmFit)

ZlmFit Fit Quality: NORMAL

Max L-expansion: min: 6 max: 7

Nr. of radial interpolation points: min: 39 max: 48

S C F *** ScaSCF ***

Initial density matrix is taken from previous geometry

CYCLE   1

SCF Error: norm([F,P])=   0.071152346137, max([F,P])=   0.010784630477

orbitals (Q,E):

A :114...133   (2.00   -0.2494) (2.00   -0.2482) (2.00   -0.2384) (2.00   -0.2358)

(2.00   -0.2276) (2.00   -0.2222) (2.00   -0.2206) (2.00   -0.2160)

(2.00   -0.1952) (2.00   -0.1771) (0.00   -0.0701) (0.00   -0.0689)

(0.00   -0.0634) (0.00   -0.0403) (0.00   -0.0191) (0.00   -0.0133)

(0.00   -0.0116) (0.00   -0.0074) (0.00   -0.0050) (0.00   -0.0005)

CYCLE   2

SDIIS (wt 0.000): 0.0367 0.9633

A-DIIS (wt 1.000): 0.0000 1.0000

DIIS coefficients: 0.0000 1.0000

SCF Error: norm([F,P])=   0.011265536546, max([F,P])=   0.001867053837

orbitals (Q,E):

A :114...124   (2.00   -0.2475) (2.00   -0.2472) (2.00   -0.2365) (2.00   -0.2345)

(2.00   -0.2267) (2.00   -0.2211) (2.00   -0.2196) (2.00   -0.2149)

(2.00   -0.1946) (2.00   -0.1769) (0.00   -0.0689)

CYCLE   3

SDIIS (wt 0.000): 0.0055 0.6589 0.3356

A-DIIS (wt 1.000): 0.0000 0.7238 0.2762

DIIS coefficients: 0.0000 0.7238 0.2762

SCF Error: norm([F,P])=   0.020964301814, max([F,P])=   0.003257142174

orbitals (Q,E):

A :114...124   (2.00   -0.2483) (2.00   -0.2475) (2.00   -0.2374) (2.00   -0.2351)

(2.00   -0.2268) (2.00   -0.2213) (2.00   -0.2199) (2.00   -0.2151)

(2.00   -0.1946) (2.00   -0.1768) (0.00   -0.0692)

CYCLE   4

SDIIS (wt 1.000): 0.3799 0.1717 0.4484

DIIS coefficients: 0.3799 0.1717 0.4484

SCF Error: norm([F,P])=   0.004951164400, max([F,P])=   0.000788527159

orbitals (Q,E):

A :114...124   (2.00   -0.2485) (2.00   -0.2475) (2.00   -0.2375) (2.00   -0.2352)

(2.00   -0.2270) (2.00   -0.2215) (2.00   -0.2200) (2.00   -0.2154)

(2.00   -0.1947) (2.00   -0.1768) (0.00   -0.0694)

CYCLE   5

SDIIS (wt 1.000): 0.1748 0.0730 0.2992 0.4530

DIIS coefficients: 0.1748 0.0730 0.2992 0.4530

SCF Error: norm([F,P])=   0.001919849153, max([F,P])=   0.000190712229

orbitals (Q,E):

A :114...124   (2.00   -0.2484) (2.00   -0.2476) (2.00   -0.2375) (2.00   -0.2352)

(2.00   -0.2270) (2.00   -0.2215) (2.00   -0.2200) (2.00   -0.2153)

(2.00   -0.1947) (2.00   -0.1768) (0.00   -0.0693)

CYCLE   6

SDIIS (wt 1.000): 0.0710 0.0277 0.1454 0.2966 0.4593

DIIS coefficients: 0.0710 0.0277 0.1454 0.2966 0.4593

SCF Error: norm([F,P])=   0.000639806825, max([F,P])=   0.000107151059

orbitals (Q,E):

A :114...124   (2.00   -0.2485) (2.00   -0.2476) (2.00   -0.2375) (2.00   -0.2352)

(2.00   -0.2270) (2.00   -0.2215) (2.00   -0.2200) (2.00   -0.2153)

(2.00   -0.1947) (2.00   -0.1768) (0.00   -0.0693)

CYCLE   7

SDIIS (wt 1.000): 0.0182 0.0062 0.0473 0.1236 0.3071 0.4975

DIIS coefficients: 0.0182 0.0062 0.0473 0.1236 0.3071 0.4975

SCF Error: norm([F,P])=   0.000240565444, max([F,P])=   0.000031695865

orbitals (Q,E):

A :114...124   (2.00   -0.2485) (2.00   -0.2476) (2.00   -0.2375) (2.00   -0.2352)

(2.00   -0.2270) (2.00   -0.2215) (2.00   -0.2200) (2.00   -0.2153)

(2.00   -0.1947) (2.00   -0.1768) (0.00   -0.0693)

CYCLE   8

SDIIS (wt 1.000): 0.0054 0.0015 0.0177 0.0547 0.1652 0.3477 0.4078

DIIS coefficients: 0.0054 0.0015 0.0177 0.0547 0.1652 0.3477 0.4078

SCF Error: norm([F,P])=   0.000098717774, max([F,P])=   0.000012993976

orbitals (Q,E):

A :114...124   (2.00   -0.2485) (2.00   -0.2476) (2.00   -0.2375) (2.00   -0.2352)

(2.00   -0.2270) (2.00   -0.2215) (2.00   -0.2200) (2.00   -0.2153)

(2.00   -0.1947) (2.00   -0.1768) (0.00   -0.0693)

CYCLE   9

SDIIS (wt 1.000): 0.0008 0.0001 0.0048 0.0194 0.0704 0.1749 0.3095 0.4200

DIIS coefficients: 0.0008 0.0001 0.0048 0.0194 0.0704 0.1749 0.3095 0.4200

SCF Error: norm([F,P])=   0.000042343147, max([F,P])=   0.000005791872

orbitals (Q,E):

A :114...124 (2.00 -0.2485) (2.00 -0.2476) (2.00 -0.2375) (2.00 -0.2352)

(2.00 -0.2270) (2.00 -0.2215) (2.00 -0.2200) (2.00 -0.2153)

(2.00 -0.1947) (2.00 -0.1768) (0.00 -0.0693)

CYCLE 10

SDIIS (wt 1.000): -0.0001 -0.0001 0.0012 0.0065 0.0274 0.0755 0.1570 0.2816 0.4510

DIIS coefficients: -0.0001 -0.0001 0.0012 0.0065 0.0274 0.0755 0.1570 0.2816 0.4510

SCF Error: norm([F,P])= 0.000012351433, max([F,P])= 0.000001399777

orbitals (Q,E):

A :114...124 (2.00 -0.2485) (2.00 -0.2476) (2.00 -0.2375) (2.00 -0.2352)

(2.00 -0.2270) (2.00 -0.2215) (2.00 -0.2200) (2.00 -0.2153)

(2.00 -0.1947) (2.00 -0.1768) (0.00 -0.0693)

CYCLE 11

SDIIS (wt 1.000): -0.0002 -0.0001 0.0000 0.0015 0.0085 0.0266 0.0641 0.1390 0.3282 0.4324

DIIS coefficients: -0.0002 -0.0001 0.0000 0.0015 0.0085 0.0266 0.0641 0.1390 0.3282 0.4324

SCF Error: norm([F,P])= 0.000005457528, max([F,P])= 0.000000896686

orbitals (Q,E):

A :114...124 (2.00 -0.2485) (2.00 -0.2476) (2.00 -0.2375) (2.00 -0.2352)

(2.00 -0.2270) (2.00 -0.2215) (2.00 -0.2200) (2.00 -0.2153)

(2.00 -0.1947) (2.00 -0.1768) (0.00 -0.0693)

SCF CONVERGED

CYCLE 12

*** Setting up for NEW gradients in Focky

*** Using FIT density in Focky

*** Setting up for NEW Hessian in Focky

orbitals (Q,E):

A :114...133 (2.00 -0.2485) (2.00 -0.2476) (2.00 -0.2375) (2.00 -0.2352)

(2.00 -0.2270) (2.00 -0.2215) (2.00 -0.2200) (2.00 -0.2153)

(2.00 -0.1947) (2.00 -0.1768) (0.00 -0.0693) (0.00 -0.0683)

(0.00 -0.0627) (0.00 -0.0394) (0.00 -0.0186) (0.00 -0.0128)

(0.00 -0.0110) (0.00 -0.0071) (0.00 -0.0046) (0.00 -0.0001)

Fit test: (difference of exact and fit density, squared integrated, result summed over spins)

SCF: 0.00100907910675

Gross Charges per Atom (Z minus electrons)

==

-0.6656	0.9220	0.5459	0.3312	-0.5803	-0.3815	-0.6305	0.6735	0.6271	0.8636
-0.6597	-0.0008	-0.5768	0.5864	0.3252	-0.5905	0.5959	0.6013	-0.6035	0.2578
0.3380	0.0978	0.1663	0.7469	0.4162	0.4094	0.5603	0.5716	0.0190	0.6423
-0.5654	0.5496	0.5498	0.5730	0.6413	0.3723	0.7505	0.9858	0.5871	-0.2126
-0.1505	-0.2113	-0.2036	-0.2136	-0.2442	-0.2315	-0.1507	-0.1879	-0.1339	-0.2069
-0.2411	-0.2208	-0.2246	-0.1846	-0.2460	-0.1709	-0.1958	-0.1545	-0.1978	-0.2140
-0.2240	-0.2197	-0.2276	-0.1928	-0.1141	-0.2230	-0.1590	-0.2165	0.8261	-0.1967
-0.2182	-0.2176	-0.2103	-0.1902	-0.2126	-0.1809	-0.2264	-0.2342	-0.1976	-0.2407
-0.2131	-0.2504	-0.2002	-0.1591	-0.1670	0.2317	-0.6750	-0.1257	-0.1948	-0.2125
-0.0745	0.5092	-0.6195	-0.1741	-0.2168	-0.2382				

Net Total: -0.00000000

M U L T I P O L E D E R I V E D C H A R G E A N A L Y S I S

See: M. Swart, P.Th. van Duijnen, J.G. Snijders, J.Comput.Chem., (2001), p. 79-88.

Multipole derived atomic charges (a.u.)

Atom		Level: MDC-m	MDC-d	MDC-q
1	O	-0.351241	-0.319574	-0.344327
2	C	0.362480	0.374327	0.396022
3	C	0.242356	0.249670	0.253219
4	C	0.180716	0.166549	0.212203
5	O	-0.306682	-0.548820	-0.609602
6	N	-0.214699	-0.279113	-0.472685
7	O	-0.380134	-0.385750	-0.450431
8	C	0.250680	0.309599	0.308476
9	C	0.228009	0.193234	0.187342
10	C	0.339244	0.409037	0.415613
11	O	-0.382935	-0.355065	-0.395647
12	C	0.124248	0.102399	0.102056
13	O	-0.312478	-0.298476	-0.343220
14	C	0.245538	0.242462	0.247943
15	C	0.196176	0.159038	0.174631
16	O	-0.369575	-0.477881	-0.483157
17	C	0.227475	0.192794	0.198714
18	C	0.229647	0.193235	0.194360
19	O	-0.319575	-0.320190	-0.372549
20	C	0.056772	0.044369	-0.058927
21	C	0.195129	0.221195	0.283631

22	C	0.118624	0.104780	0.107408
23	C	0.119705	0.099326	0.110533
24	C	0.290642	0.268563	0.286230
25	C	0.172993	0.135120	0.137211
26	C	0.164804	0.124869	0.164558
27	C	0.249079	0.239325	0.262526
28	C	0.234482	0.193850	0.304457
29	C	0.034666	0.027747	-0.043944
30	C	0.237017	0.191248	0.177256
31	O	-0.372190	-0.476531	-0.517186
32	C	0.254525	0.243431	0.263339
33	C	0.222275	0.185359	0.194553
34	C	0.228261	0.295940	0.386163
35	C	0.221854	0.192141	0.201684
36	C	0.180419	0.145521	0.150697
37	C	0.293869	0.271215	0.310801
38	C	0.368475	0.339788	0.387532
39	C	0.229516	0.190148	0.156538
40	H	-0.073312	-0.060349	-0.026971
41	H	-0.056923	-0.031203	-0.019188
42	H	-0.064152	-0.056757	-0.033518
43	H	-0.056944	-0.036093	-0.017423
44	H	-0.068989	-0.052763	-0.051027
45	H	-0.070430	-0.057557	-0.052793
46	H	-0.076644	-0.072015	-0.070266
47	H	-0.071451	-0.061389	-0.062157
48	H	-0.063737	-0.041706	-0.022860
49	H	-0.082847	-0.063024	-0.070573
50	H	-0.055335	-0.047091	-0.049649
51	H	-0.072700	-0.055391	-0.060235
52	H	-0.070578	-0.058391	-0.064879

53	H	-0.067774	-0.063876	-0.073595
54	H	-0.057971	-0.045130	-0.052116
55	H	-0.054148	-0.043990	-0.046116
56	H	-0.057162	-0.042319	-0.030795
57	H	-0.067183	-0.050359	-0.051601
58	H	-0.061816	-0.036439	-0.032621
59	H	-0.075311	-0.060520	-0.049839
60	H	-0.074712	-0.065305	-0.055887
61	H	-0.068797	-0.054897	-0.043082
62	H	-0.067069	-0.048751	-0.040180
63	H	-0.072427	-0.074616	-0.082799
64	H	-0.058195	-0.036054	-0.033611
65	H	-0.074855	-0.049405	-0.058202
66	H	-0.072369	-0.063979	-0.060938
67	H	-0.067576	-0.054163	-0.067299
68	H	-0.064973	-0.056252	-0.055935
69	C	0.353441	0.438369	0.461009
70	H	-0.064665	-0.054255	-0.082676
71	H	-0.068108	-0.067114	-0.097792
72	H	-0.063247	-0.048441	-0.074529
73	H	-0.080861	-0.062321	-0.072878
74	H	-0.072841	-0.059931	-0.063528
75	H	-0.069551	-0.068831	-0.073351
76	H	-0.076201	-0.046325	-0.040287
77	H	-0.066519	-0.054416	-0.038752
78	H	-0.065061	-0.060552	-0.028774
79	H	-0.082573	-0.063736	-0.046466
80	H	-0.066824	-0.058141	-0.051719
81	H	-0.067811	-0.066476	-0.055849
82	H	-0.071141	-0.044106	-0.037592
83	H	-0.061803	-0.026107	-0.018929

84	H	-0.047199	-0.002888	-0.016285
85	H	-0.073588	-0.059823	-0.041216
86	H	0.062440	0.262493	0.292049
87	O	-0.365324	-0.442969	-0.458343
88	H	-0.045067	0.000465	0.021121
89	H	-0.044938	-0.029285	-0.015455
90	H	-0.051773	-0.049656	-0.038917
91	H	-0.042714	-0.011047	-0.011141
92	C	0.240984	0.197689	0.181822
93	O	-0.362288	-0.461203	-0.489251
94	H	-0.061182	-0.053612	-0.049794
95	H	-0.060944	-0.047447	-0.038916
96	H	-0.068430	-0.065430	-0.061429

*** Using NEW gradient routines ***

Geometry CYCLE 20

==============

Energy gradients wrt nuclear displacements

==

Atom Cartesian (a.u./angstrom)

X Y Z

1	O	-0.000483	-0.001042	0.000122
2	C	0.000542	0.000059	-0.000021
3	C	-0.000560	0.000201	-0.000386
4	C	0.000230	-0.000336	-0.000113
5	O	0.000113	-0.000734	-0.000960
6	N	0.000104	0.000412	0.000429

7	O	-0.000266	0.000682	0.000184
8	C	0.000089	-0.001032	0.000643
9	C	0.000087	0.000122	-0.000117
10	C	-0.000169	0.000802	-0.000373
11	O	-0.000825	-0.000485	0.000112
12	C	-0.000141	0.000369	-0.000164
13	O	-0.000189	0.000062	-0.000071
14	C	-0.000197	0.001314	-0.000435
15	C	0.000737	-0.000390	0.000278
16	O	-0.000358	0.001011	-0.000211
17	C	0.000170	0.000277	-0.000468
18	C	-0.000143	0.000067	-0.000002
19	O	-0.000097	-0.000136	0.000899
20	C	-0.000065	-0.000486	-0.000480
21	C	0.000068	-0.000044	0.000590
22	C	0.000560	0.000024	-0.000023
23	C	-0.000289	-0.000515	-0.001136
24	C	-0.000096	-0.000142	0.000529
25	C	0.000432	0.000087	0.000074
26	C	0.000526	-0.000104	-0.000005
27	C	-0.000360	-0.000143	-0.000158
28	C	0.000191	-0.000135	-0.000186
29	C	-0.000335	0.000464	-0.000339
30	C	0.000052	-0.000250	0.000040
31	O	0.000110	0.000350	-0.000731
32	C	0.000348	-0.000048	0.000161
33	C	0.000035	-0.000247	0.000051
34	C	0.001024	0.000083	0.001973
35	C	-0.000001	0.000242	-0.000033
36	C	-0.000031	0.000231	-0.000239
37	C	0.000313	-0.000246	-0.000016

38	C	0.000342	-0.000447	0.000204
39	C	0.000094	-0.000091	0.000037
40	H	-0.000293	0.000239	-0.000073
41	H	0.000122	0.000325	0.000265
42	H	0.000043	0.000018	-0.000227
43	H	0.000228	0.000436	0.000060
44	H	0.000123	0.000134	0.000076
45	H	-0.000037	0.000065	-0.000249
46	H	-0.000117	0.000054	-0.000080
47	H	-0.000178	-0.000101	0.000206
48	H	0.000198	-0.000107	-0.000027
49	H	-0.000007	-0.000167	0.000013
50	H	0.000045	0.000211	-0.000013
51	H	-0.000101	0.000013	0.000267
52	H	-0.000104	0.000026	-0.000008
53	H	0.000096	0.000325	0.000065
54	H	-0.000100	-0.000051	0.000389
55	H	-0.000231	-0.000148	-0.000170
56	H	-0.000159	0.000168	-0.000121
57	H	0.000217	-0.000153	0.000038
58	H	0.000123	0.000034	0.000325
59	H	-0.000118	0.000142	0.000146
60	H	-0.000151	-0.000014	-0.000177
61	H	-0.000085	-0.000212	0.000016
62	H	0.000016	-0.000153	-0.000049
63	H	-0.000100	0.000030	-0.000169
64	H	0.000206	0.000056	0.000158
65	H	-0.000200	-0.000238	-0.000288
66	H	-0.000119	-0.000020	0.000255
67	H	0.000400	0.000288	0.000255
68	H	0.000094	-0.000173	-0.000088

69	C	-0.003345	0.000489	-0.002272
70	H	0.000079	0.000168	-0.000105
71	H	0.000031	0.000043	0.000213
72	H	-0.000240	0.000041	-0.000050
73	H	-0.000064	-0.000052	0.000120
74	H	0.000142	0.000144	-0.000085
75	H	-0.000036	0.000055	0.000243
76	H	0.000218	-0.000002	-0.000300
77	H	0.000052	0.000187	-0.000076
78	H	0.000198	-0.000012	-0.000017
79	H	0.000026	-0.000049	0.000118
80	H	-0.000068	-0.000087	0.000110
81	H	0.000166	-0.000002	0.000193
82	H	-0.000397	-0.000359	-0.000013
83	H	-0.000477	-0.000209	0.000034
84	H	-0.000138	0.000025	0.000089
85	H	-0.000171	0.000052	0.000243
86	H	-0.000306	-0.000181	0.000123
87	O	-0.000058	-0.000249	0.000954
88	H	0.000160	-0.000202	0.000161
89	H	0.000219	-0.000202	0.000086
90	H	-0.000024	-0.000306	0.000292
91	H	-0.000097	0.000245	0.000216
92	C	0.001114	-0.000091	0.000157
93	O	0.001746	-0.000453	-0.000444
94	H	0.000128	0.000106	-0.000298
95	H	-0.000087	0.000191	0.000061
96	H	-0.000147	-0.000118	-0.000210

----

dE(predicted): -0.88848E-04, dE(actual): -0.79520E-04, Trust radius: 0.195959, all in a.u.

Geometry Convergence after Step 20 (Hartree/Angstrom,Angstrom)

----

current energy	-19.67520961 Hartree		
energy change	-0.00007952	0.00100000	T
constrained gradient max	0.00334509	0.00100000	F
constrained gradient rms	0.00042203	0.00066667	T
gradient max	0.00334509		
gradient rms	0.00042203		
cart. step max	0.02417615	0.01000000	F
cart. step rms	0.00657042	0.00666667	T

Coordinates (Cartesian)

======================

Atom	bohr			angstrom			Geometric Variables		
	X	Y	Z	X	Y	Z	(0:frozen, *:LT par.)		
1 O	26.548615	61.680898	50.458098	14.048922	32.640125	26.701276	1	2	3
2 C	27.030870	61.970869	53.036723	14.304121	32.793571	28.065825	4	5	6
3 C	29.821570	61.226363	53.429951	15.780895	32.399596	28.273912	7	8	9
4 C	30.590351	61.534193	56.228193	16.187717	32.562493	29.754678	10	11	12
5 O	27.309293	52.969934	43.190263	14.451455	28.030482	22.855303	13	14	15
6 N	33.228797	60.896328	56.870456	17.583922	32.224949	30.094549	16	17	18
7 O	26.656073	64.563798	53.747907	14.105786	34.165690	28.442167	19	20	21
8 C	20.427445	58.508210	48.378321	10.809739	30.961212	25.600705	22	23	24
9 C	25.697049	65.421027	41.501178	13.598293	34.619317	21.961478	25	26	27
10 C	21.622370	55.030811	45.066790	11.442065	29.121051	23.848318	28	29	30

11 O	21.712050	62.751600	45.645629	11.489522	33.206716	24.154626	31	32	33
12 C	19.522854	56.580341	46.302525	10.331050	29.941027	24.502241	34	35	36
13 O	21.590570	55.178809	42.530102	11.425238	29.199368	22.505961	37	38	39
14 C	23.964305	61.581341	49.547301	12.681364	32.587442	26.219302	40	41	42
15 C	23.992254	63.286046	47.106820	12.696154	33.489533	24.927856	43	44	45
16 O	18.795074	59.576693	49.613779	9.945925	31.526628	26.254481	46	47	48
17 C	23.714675	57.028114	51.321484	12.549265	30.177978	27.158160	49	50	51
18 C	27.452449	53.189632	38.689198	14.527210	28.146741	20.473442	52	53	54
19 O	30.093017	58.590206	52.648725	15.924539	31.004602	27.860505	55	56	57
20 C	26.351471	57.088234	41.145379	13.944598	30.209793	21.773197	58	59	60
21 C	26.103967	54.205593	41.078035	13.813624	28.684364	21.737560	61	62	63
22 C	23.226646	58.789821	49.022611	12.291012	31.110234	25.941649	64	65	66
23 C	25.941115	62.710389	42.501752	13.727447	33.184909	22.490959	67	68	69
24 C	35.166141	61.975070	55.195323	18.609120	32.795795	29.208107	70	71	72
25 C	26.287031	62.495540	45.412915	13.910498	33.071215	24.031480	73	74	75
26 C	29.924106	64.270660	57.035030	15.835155	34.010568	30.181638	76	77	78
27 C	27.163274	64.961172	56.426589	14.374186	34.375972	29.859665	79	80	81
28 C	26.519847	67.714491	56.992430	14.033699	35.832966	30.159095	82	83	84
29 C	28.399924	58.484943	41.709912	15.028592	30.948899	22.071935	85	86	87
30 C	17.664906	57.853518	44.453124	9.347866	30.614763	23.523580	88	89	90
31 O	29.996496	62.477780	40.384990	15.873462	33.061817	21.370816	91	92	93
32 C	23.223572	53.513274	40.907119	12.289385	28.318005	21.647115	94	95	96
33 C	30.970852	57.538837	42.517202	16.389069	30.448241	22.499135	97	98	99
34 C	28.242871	61.332208	41.375737	14.945484	32.455607	21.895097	100	101	102
35 C	24.231042	66.072696	47.913634	12.822515	34.964165	25.354803	103	104	105
36 C	22.571623	50.719593	41.313697	11.944388	26.839653	21.862267	106	107	108
37 C	33.686021	58.197898	57.349795	17.825875	30.797001	30.348205	109	110	111
38 C	19.471916	64.195851	46.198674	10.304094	33.970982	24.447286	112	113	114

39 C 19.730699 50.143637 41.078911 10.441036 26.534870 21.738024 115 116
117

40 H 24.660926 58.086742 40.531353 13.050000 30.738180 21.448269 118 119
120

41 H 31.504133 58.409775 44.325118 16.671269 30.909122 23.455843 121 122
123

42 H 31.019573 55.490732 42.725648 16.414851 29.364431 22.609439 124 125
126

43 H 32.385217 58.158281 41.133418 17.137519 30.776037 21.766867 127 128
129

44 H 27.280590 66.594809 42.135010 14.436266 35.240455 22.296887 130 131
132

45 H 25.704595 65.438322 39.429941 13.602286 34.628469 20.865426 133 134
135

46 H 23.925976 66.267666 42.154292 12.661081 35.067339 22.307091 136 137
138

47 H 24.228031 61.650447 42.018450 12.820922 32.624011 22.235206 139 140
141

48 H 27.935386 63.605268 45.987454 14.782770 33.658458 24.335513 142 143
144

49 H 26.781303 60.536380 45.836537 14.172055 32.034473 24.255651 145 146
147

50 H 25.981435 66.299181 48.985530 13.748783 35.084016 25.922026 148 149
150

51 H 22.678466 66.655855 49.150213 12.000927 35.272759 26.009173 151 152
153

52 H 24.290968 67.321875 46.269292 12.854227 35.625202 24.484655 154 155
156

53 H 19.716872 66.197034 45.711635 10.433719 35.029962 24.189555 157 158
159

54 H 18.889996 64.014702 48.181747 9.996155 33.875122 25.496683 160 161
162

55 H 17.986562 63.393930 45.009533 9.518079 33.546623 23.818019 163 164
165

56 H 22.560210 54.110309 39.037955 11.938349 28.633942 20.657996 166 167
168

57 H 23.617540 49.645609 39.882590 12.497864 26.271325 21.104958 169 170
171

58 H	23.286143	50.096992	43.148561	12.322496	26.510187	22.833235	172	173	174
59 H	18.637108	51.113447	42.546686	9.862333	27.048071	22.514736	175	176	177
60 H	18.981387	50.741420	39.238729	10.044517	26.851203	20.764241	178	179	180
61 H	19.388605	48.110770	41.277954	10.260008	25.459123	21.843353	181	182	183
62 H	27.277621	51.131862	38.562199	14.434695	27.057816	20.406237	184	185	186
63 H	26.681689	54.056049	36.974472	14.119342	28.605229	19.566048	187	188	189
64 H	29.459279	53.665334	38.808645	15.589179	28.398472	20.536650	190	191	192
65 H	24.375907	58.109028	47.448016	12.899174	30.749973	25.108409	193	194	195
66 H	22.590168	57.641702	52.949434	11.954202	30.502675	28.019634	196	197	198
67 H	25.714013	57.045680	51.828303	13.607270	30.187274	27.426357	199	200	201
68 H	23.190707	55.087633	50.843669	12.271993	29.151120	26.905311	202	203	204
69 C	31.050024	58.141519	50.260132	16.430965	30.767167	26.596517	205	206	207
70 H	27.724778	68.976064	55.875032	14.671321	36.500561	29.567794	208	209	210
71 H	26.814839	68.125080	59.001550	14.189802	36.050240	31.222275	211	212	213
72 H	24.543140	68.106552	56.519923	12.987671	36.040435	29.909055	214	215	216
73 H	25.893699	63.711666	57.521912	13.702355	33.714762	30.439285	217	218	219
74 H	31.159507	65.620608	56.057713	16.488901	34.724930	29.664464	220	221	222
75 H	30.274699	64.459500	59.068643	16.020681	34.110498	31.257780	223	224	225
76 H	35.241954	61.070470	53.311919	18.649239	32.317101	28.211453	226	227	228

77 H	34.844926	64.000283	54.922421	18.439141	33.867491	29.063693	229	230	231
78 H	37.022189	61.750408	56.088928	19.591298	32.676909	29.680982	232	233	234
79 H	33.660123	57.020347	55.624527	17.812170	30.173868	29.435232	235	236	237
80 H	32.250898	57.465683	58.651187	17.066440	30.409530	31.036872	238	239	240
81 H	35.539155	57.980970	58.255340	18.806511	30.682208	30.827398	241	242	243
82 H	25.781202	60.769376	54.202225	13.642824	32.157769	28.682582	244	245	246
83 H	22.673188	62.391645	50.952173	11.998135	33.016237	26.962729	247	248	249
84 H	30.965644	62.422700	52.193467	16.386313	33.032670	27.619593	250	251	252
85 H	29.401244	60.243002	57.339976	15.558468	31.879224	30.343009	253	254	255
86 H	26.174571	53.178647	44.645563	13.850986	28.140928	23.625414	256	257	258
87 O	23.123136	53.753544	46.299520	12.236237	28.445150	24.500651	259	260	261
88 H	18.483539	55.175390	47.433580	9.781068	29.197559	25.100770	262	263	264
89 H	32.083600	54.815680	48.194586	16.977910	29.007209	25.503477	265	266	267
90 H	30.465140	54.169677	51.121170	16.121458	28.665359	27.052158	268	269	270
91 H	28.793203	55.349699	48.460816	15.236707	29.289799	25.644359	271	272	273
92 C	30.594704	55.426317	49.486534	16.190020	29.330344	26.187146	274	275	276
93 O	32.058273	59.731743	48.948893	16.964507	31.608677	25.902639	277	278	279
94 H	18.659259	59.215907	43.260111	9.874055	31.335709	22.892265	280	281	282
95 H	16.203486	58.836941	45.532779	8.574516	31.135168	24.094909	283	284	285

96 H 16.770532 56.444064 43.232912 8.874583 29.868912 22.877872 286 287 288

Number of elements of the density matrix on this node (used, total): 222269 1773786

Numerical Integration : Fuzzy Cells (Becke)

Becke grid quality: NORMAL

Lebedev angular grid order: min: 11 max: 23

Nr. of radial points: min: 46 max: 52

Total nr. of points: 600096

Nr. of blocks: 4689

Block length: 128

Nr. of dummy points: 96

Test of Precision of the Numerical Integration Grid

===

Integral of the Total Core Density: 85.99993455881896

Relative Error: -7.609E-07

Density Fitting (zlmFit)

ZlmFit Fit Quality: NORMAL

Max L-expansion: min: 6 max: 7

Nr. of radial interpolation points: min: 39 max: 48

S C F *** ScaSCF ***

Initial density matrix is taken from previous geometry

CYCLE 1

SCF Error: norm([F,P])= 0.036358179306, max([F,P])= 0.004378160469

orbitals (Q,E):

```
A :114...133       ( 2.00    -0.2478)  ( 2.00    -0.2470)  ( 2.00    -0.2371)  ( 2.00    -0.2346)
                   ( 2.00    -0.2265)  ( 2.00    -0.2211)  ( 2.00    -0.2194)  ( 2.00    -0.2149)
                   ( 2.00    -0.1942)  ( 2.00    -0.1766)  ( 0.00    -0.0689)  ( 0.00    -0.0679)
                   ( 0.00    -0.0622)  ( 0.00    -0.0392)  ( 0.00    -0.0182)  ( 0.00    -0.0125)
                   ( 0.00    -0.0106)  ( 0.00    -0.0067)  ( 0.00    -0.0042)  ( 0.00     0.0003)
```

CYCLE 2

MIX coefficients: 0.8000 0.2000

SCF Error: norm([F,P])= 0.006964709812, max([F,P])= 0.000815216040

orbitals (Q,E):

A :114...124 (2.00 -0.2481) (2.00 -0.2473) (2.00 -0.2371) (2.00 -0.2348)

(2.00 -0.2267) (2.00 -0.2212) (2.00 -0.2196) (2.00 -0.2150)

(2.00 -0.1943) (2.00 -0.1767) (0.00 -0.0691)

CYCLE 3

SDIIS (wt 0.000): -0.7363 1.7363

A-DIIS (wt 1.000): 0.0000 1.0000

DIIS coefficients: 0.0000 1.0000

SCF Error: norm([F,P])= 0.003630741764, max([F,P])= 0.000454016557

orbitals (Q,E):

A :114...124 (2.00 -0.2487) (2.00 -0.2479) (2.00 -0.2373) (2.00 -0.2352)

(2.00 -0.2271) (2.00 -0.2216) (2.00 -0.2201) (2.00 -0.2155)

(2.00 -0.1946) (2.00 -0.1768) (0.00 -0.0696)

CYCLE 4

SDIIS (wt 1.000): 0.4285 -0.0546 0.6261

DIIS coefficients: 0.4285 -0.0546 0.6261

SCF Error: norm([F,P])= 0.004520062541, max([F,P])= 0.000479026166

orbitals (Q,E):

A :114...124 (2.00 -0.2484) (2.00 -0.2476) (2.00 -0.2373) (2.00 -0.2351)

(2.00 -0.2269) (2.00 -0.2215) (2.00 -0.2199) (2.00 -0.2153)

(2.00 -0.1945) (2.00 -0.1768) (0.00 -0.0694)

CYCLE 5

SDIIS (wt 1.000): 0.3998 -0.2560 0.4461 0.4101

DIIS coefficients: 0.3998 -0.2560 0.4461 0.4101

SCF Error: norm([F,P])= 0.001319560608, max([F,P])= 0.000192511310

orbitals (Q,E):

A :114...124 (2.00 -0.2485) (2.00 -0.2476) (2.00 -0.2373) (2.00 -0.2351)

(2.00 -0.2270) (2.00 -0.2215) (2.00 -0.2199) (2.00 -0.2153)

(2.00 -0.1946) (2.00 -0.1768) (0.00 -0.0694)

CYCLE 6

SDIIS (wt 1.000): 0.2036 -0.1653 0.2024 0.2747 0.4847

DIIS coefficients: 0.2036 -0.1653 0.2024 0.2747 0.4847

SCF Error: norm([F,P])= 0.000421798234, max([F,P])= 0.000060176934

orbitals (Q,E):

A :114...124 (2.00 -0.2484) (2.00 -0.2476) (2.00 -0.2373) (2.00 -0.2351)

(2.00 -0.2270) (2.00 -0.2215) (2.00 -0.2199) (2.00 -0.2154)

(2.00 -0.1946) (2.00 -0.1768) (0.00 -0.0694)

CYCLE 7

SDIIS (wt 1.000): 0.0755 -0.0711 0.0679 0.1190 0.3110 0.4977

DIIS coefficients: 0.0755 -0.0711 0.0679 0.1190 0.3110 0.4977

SCF Error: norm([F,P])= 0.000145253555, max([F,P])= 0.000016553286

orbitals (Q,E):

A :114...124 (2.00 -0.2484) (2.00 -0.2476) (2.00 -0.2373) (2.00 -0.2351)

(2.00 -0.2270) (2.00 -0.2215) (2.00 -0.2199) (2.00 -0.2154)

(2.00 -0.1946) (2.00 -0.1768) (0.00 -0.0694)

CYCLE 8

SDIIS (wt 1.000): 0.0275 -0.0279 0.0235 0.0482 0.1452 0.3075 0.4760

DIIS coefficients: 0.0275 -0.0279 0.0235 0.0482 0.1452 0.3075 0.4760

SCF Error: norm([F,P])= 0.000045119048, max([F,P])= 0.000003770744

orbitals (Q,E):

A :114...124 (2.00 -0.2484) (2.00 -0.2476) (2.00 -0.2373) (2.00 -0.2351)

(2.00 -0.2270) (2.00 -0.2215) (2.00 -0.2199) (2.00 -0.2154)

(2.00 -0.1946) (2.00 -0.1768) (0.00 -0.0694)

CYCLE 9

SDIIS (wt 1.000): 0.0102 -0.0112 0.0082 0.0206 0.0696 0.1713 0.3723 0.3589

DIIS coefficients: 0.0102 -0.0112 0.0082 0.0206 0.0696 0.1713 0.3723 0.3589

SCF Error: norm([F,P])= 0.000027355267, max([F,P])= 0.000004696001

orbitals (Q,E):

A :114...124 (2.00 -0.2484) (2.00 -0.2476) (2.00 -0.2373) (2.00 -0.2351)

(2.00 -0.2270) (2.00 -0.2215) (2.00 -0.2199) (2.00 -0.2154)

(2.00 -0.1946) (2.00 -0.1768) (0.00 -0.0694)

CYCLE 10

SDIIS (wt 1.000): 0.0018 -0.0024 0.0013 0.0053 0.0208 0.0611 0.1709 0.2506 0.4905

DIIS coefficients: 0.0018 -0.0024 0.0013 0.0053 0.0208 0.0611 0.1709 0.2506 0.4905

SCF Error: norm([F,P])= 0.000007918429, max([F,P])= 0.000001272337

orbitals (Q,E):

A :114...124 (2.00 -0.2484) (2.00 -0.2476) (2.00 -0.2373) (2.00 -0.2351)

(2.00 -0.2270) (2.00 -0.2215) (2.00 -0.2199) (2.00 -0.2154)

(2.00 -0.1946) (2.00 -0.1768) (0.00 -0.0694)

CYCLE 11

SDIIS (wt 1.000): 0.0001 -0.0003 -0.0000 0.0013 0.0064 0.0225 0.0747 0.1303 0.3329 0.4323

DIIS coefficients: 0.0001 -0.0003 -0.0000 0.0013 0.0064 0.0225 0.0747 0.1303 0.3329 0.4323

SCF Error: norm([F,P])= 0.000002918097, max([F,P])= 0.000000420821

orbitals (Q,E):

A :114...124 (2.00 -0.2484) (2.00 -0.2476) (2.00 -0.2373) (2.00 -0.2351)

(2.00 -0.2270) (2.00 -0.2215) (2.00 -0.2199) (2.00 -0.2154)

(2.00 -0.1946) (2.00 -0.1768) (0.00 -0.0694)

SCF CONVERGED

CYCLE 12

*** Setting up for NEW gradients in Focky

*** Using FIT density in Focky

*** Setting up for NEW Hessian in Focky

orbitals (Q,E):

A :114...133 (2.00 -0.2484) (2.00 -0.2476) (2.00 -0.2373) (2.00 -0.2351)

(2.00 -0.2270) (2.00 -0.2215) (2.00 -0.2199) (2.00 -0.2154)

(2.00 -0.1946) (2.00 -0.1768) (0.00 -0.0694) (0.00 -0.0683)

(0.00 -0.0627) (0.00 -0.0395) (0.00 -0.0186) (0.00 -0.0128)

(0.00 -0.0110) (0.00 -0.0069) (0.00 -0.0045) (0.00 -0.0000)

Fit test: (difference of exact and fit density, squared integrated, result summed over spins)

SCF: 0.00100962833481

Gross Charges per Atom (Z minus electrons)

==

-0.6654	0.9222	0.5465	0.3316	-0.5805	-0.3816	-0.6306	0.6731	0.6279	0.8640
-0.6598	-0.0009	-0.5768	0.5857	0.3249	-0.5901	0.5963	0.6016	-0.6034	0.2584
0.3378	0.0980	0.1666	0.7473	0.4163	0.4093	0.5605	0.5717	0.0196	0.6422
-0.5655	0.5506	0.5508	0.5723	0.6414	0.3722	0.7508	0.9859	0.5874	-0.2130
-0.1534	-0.2101	-0.2042	-0.2135	-0.2448	-0.2315	-0.1506	-0.1881	-0.1338	-0.2062
-0.2417	-0.2205	-0.2247	-0.1852	-0.2454	-0.1710	-0.1959	-0.1545	-0.1980	-0.2140
-0.2242	-0.2200	-0.2277	-0.1926	-0.1143	-0.2229	-0.1598	-0.2163	0.8242	-0.1968
-0.2182	-0.2177	-0.2104	-0.1903	-0.2128	-0.1812	-0.2264	-0.2344	-0.1975	-0.2407

-0.2134 -0.2507 -0.2000 -0.1591 -0.1671 0.2321 -0.6751 -0.1255 -0.1958 -0.2129

-0.0735 0.5113 -0.6193 -0.1742 -0.2166 -0.2384

Net Total: -0.00000000

M U L T I P O L E   D E R I V E D   C H A R G E   A N A L Y S I S

See: M. Swart, P.Th. van Duijnen, J.G. Snijders, J.Comput.Chem., (2001), p. 79-88.

Multipole derived atomic charges (a.u.)

Atom		Level: MDC-m	MDC-d	MDC-q
1	O	-0.350975	-0.319189	-0.343862
2	C	0.362448	0.374397	0.395977
3	C	0.242337	0.250061	0.253478
4	C	0.180684	0.166637	0.212400
5	O	-0.306677	-0.548580	-0.609489
6	N	-0.214744	-0.279722	-0.473892
7	O	-0.380000	-0.385514	-0.450226
8	C	0.250657	0.309641	0.308443
9	C	0.228040	0.193378	0.187433
10	C	0.339203	0.409026	0.415782
11	O	-0.382850	-0.354278	-0.394519
12	C	0.124222	0.102379	0.101960
13	O	-0.312228	-0.298707	-0.343473

14	C	0.245469	0.242146	0.247699
15	C	0.196191	0.159061	0.174494
16	O	-0.369603	-0.477944	-0.483347
17	C	0.227668	0.192972	0.199144
18	C	0.229619	0.193259	0.194198
19	O	-0.319523	-0.320572	-0.373112
20	C	0.056767	0.044521	-0.059151
21	C	0.195069	0.221062	0.283816
22	C	0.118625	0.104686	0.107254
23	C	0.119713	0.099449	0.110644
24	C	0.290685	0.268707	0.286453
25	C	0.173082	0.135455	0.137618
26	C	0.164836	0.124919	0.164670
27	C	0.249091	0.239330	0.262780
28	C	0.234512	0.193889	0.303932
29	C	0.034812	0.028035	-0.043702
30	C	0.237040	0.191252	0.177269
31	O	-0.372338	-0.476821	-0.517418
32	C	0.254489	0.243422	0.263466
33	C	0.222466	0.185704	0.196305
34	C	0.228053	0.295523	0.385719
35	C	0.221788	0.192106	0.201573
36	C	0.180430	0.145581	0.150874
37	C	0.293833	0.271325	0.310919
38	C	0.368624	0.339766	0.387688
39	C	0.229558	0.190264	0.156786
40	H	-0.073361	-0.060530	-0.026934
41	H	-0.056770	-0.031092	-0.019024
42	H	-0.064050	-0.056475	-0.033674
43	H	-0.057020	-0.036353	-0.018381
44	H	-0.068984	-0.052723	-0.050833

45	H	-0.070483	-0.057741	-0.053010
46	H	-0.076678	-0.071941	-0.070264
47	H	-0.071513	-0.061721	-0.062562
48	H	-0.063670	-0.041627	-0.022934
49	H	-0.082848	-0.063086	-0.070740
50	H	-0.055213	-0.046830	-0.049377
51	H	-0.072803	-0.055691	-0.060426
52	H	-0.070660	-0.058516	-0.065092
53	H	-0.067711	-0.063714	-0.073624
54	H	-0.058057	-0.045472	-0.052323
55	H	-0.054173	-0.044030	-0.046312
56	H	-0.057225	-0.042257	-0.030826
57	H	-0.067271	-0.050476	-0.051915
58	H	-0.061764	-0.036604	-0.032615
59	H	-0.075307	-0.060544	-0.049905
60	H	-0.074746	-0.065361	-0.055962
61	H	-0.068806	-0.054910	-0.043122
62	H	-0.067114	-0.048859	-0.040428
63	H	-0.072418	-0.074539	-0.082620
64	H	-0.058252	-0.036164	-0.033532
65	H	-0.075009	-0.049557	-0.058442
66	H	-0.072276	-0.063917	-0.060841
67	H	-0.067507	-0.053973	-0.067321
68	H	-0.064917	-0.056109	-0.055777
69	C	0.353111	0.437595	0.460105
70	H	-0.064700	-0.054298	-0.082613
71	H	-0.068098	-0.067084	-0.097609
72	H	-0.063247	-0.048454	-0.074390
73	H	-0.080883	-0.062376	-0.072999
74	H	-0.072864	-0.059973	-0.063551
75	H	-0.069546	-0.068851	-0.073338

76	H	-0.076290	-0.046427	-0.040544
77	H	-0.066506	-0.054410	-0.038637
78	H	-0.065034	-0.060518	-0.028648
79	H	-0.082613	-0.063801	-0.046394
80	H	-0.066823	-0.058136	-0.051662
81	H	-0.067876	-0.066569	-0.055803
82	H	-0.071197	-0.044182	-0.037691
83	H	-0.061850	-0.026349	-0.019150
84	H	-0.047181	-0.002646	-0.015939
85	H	-0.073601	-0.059857	-0.041133
86	H	0.062208	0.262233	0.291388
87	O	-0.364840	-0.442414	-0.457929
88	H	-0.045006	0.000628	0.021337
89	H	-0.045183	-0.029872	-0.015919
90	H	-0.051987	-0.049899	-0.038239
91	H	-0.042520	-0.010145	-0.009440
92	C	0.241152	0.197820	0.180105
93	O	-0.362555	-0.461452	-0.489055
94	H	-0.061251	-0.053664	-0.049770
95	H	-0.060922	-0.047359	-0.038879
96	H	-0.068366	-0.065358	-0.061373

*** Using NEW gradient routines ***

Geometry CYCLE 21

==============

Energy gradients wrt nuclear displacements

==

Atom Cartesian (a.u./angstrom)

		X	Y	Z
1	O	-0.000172	-0.000344	-0.000098
2	C	0.000194	-0.000211	0.000081
3	C	-0.000170	-0.000048	-0.000021
4	C	0.000391	-0.000275	0.000027
5	O	0.000265	-0.000280	-0.000547
6	N	-0.000031	0.000189	0.000231
7	O	-0.000207	0.000298	-0.000175
8	C	-0.000029	0.000104	-0.000235
9	C	0.000123	0.000056	0.000098
10	C	0.000508	0.000931	-0.000132
11	O	-0.000057	-0.000285	-0.000658
12	C	-0.000249	0.000068	-0.000115
13	O	-0.000322	-0.000143	0.000056
14	C	0.000086	0.001117	0.000007
15	C	0.000152	-0.000174	0.000453
16	O	-0.000211	-0.000056	0.000211
17	C	0.000068	0.000024	-0.000437
18	C	-0.000196	0.000271	-0.000041
19	O	-0.001060	0.000445	-0.000421
20	C	0.000335	-0.000194	-0.000248
21	C	-0.000157	-0.000192	0.000613
22	C	0.000281	-0.000377	0.000266
23	C	-0.000233	0.000031	-0.000257
24	C	-0.000014	-0.000077	0.000174
25	C	0.000044	0.000279	0.000117
26	C	0.000237	-0.000024	0.000022
27	C	-0.000118	-0.000162	-0.000174
28	C	0.000105	-0.000046	-0.000077
29	C	0.000024	0.000178	0.000304

30	C	-0.000063	-0.000031	0.000081
31	O	0.000034	-0.000111	0.000382
32	C	0.000189	-0.000021	0.000162
33	C	-0.000096	-0.000319	-0.000045
34	C	-0.000172	0.000025	-0.000622
35	C	0.000495	0.000067	0.000140
36	C	-0.000146	0.000202	-0.000261
37	C	0.000119	-0.000089	0.000011
38	C	-0.000036	-0.000145	-0.000150
39	C	0.000004	-0.000146	0.000024
40	H	-0.000016	0.000005	-0.000219
41	H	0.000040	0.000262	-0.000094
42	H	0.000035	-0.000062	-0.000132
43	H	0.000146	0.000293	0.000136
44	H	0.000030	0.000066	0.000040
45	H	0.000010	-0.000017	-0.000118
46	H	-0.000137	0.000049	0.000073
47	H	-0.000073	-0.000036	0.000202
48	H	0.000100	-0.000087	0.000012
49	H	0.000002	-0.000017	-0.000016
50	H	0.000050	0.000178	-0.000036
51	H	0.000145	-0.000025	0.000136
52	H	-0.000047	-0.000011	-0.000142
53	H	-0.000136	-0.000007	-0.000023
54	H	-0.000075	0.000017	0.000104
55	H	-0.000091	-0.000120	-0.000070
56	H	-0.000148	0.000067	0.000024
57	H	0.000156	-0.000091	0.000104
58	H	-0.000022	-0.000029	0.000130
59	H	-0.000052	0.000066	0.000037
60	H	-0.000063	-0.000019	-0.000074

61	H	-0.000047	-0.000076	0.000015
62	H	-0.000042	-0.000066	0.000013
63	H	-0.000032	0.000039	-0.000106
64	H	0.000134	0.000050	0.000122
65	H	-0.000190	-0.000236	-0.000109
66	H	-0.000108	-0.000040	0.000133
67	H	0.000292	0.000030	-0.000020
68	H	0.000058	-0.000136	-0.000108
69	C	0.002724	-0.001333	0.000997
70	H	0.000024	0.000052	-0.000037
71	H	0.000005	0.000019	0.000097
72	H	-0.000101	0.000018	-0.000014
73	H	0.000004	-0.000019	0.000037
74	H	0.000013	0.000038	-0.000048
75	H	-0.000026	0.000025	0.000105
76	H	0.000100	0.000046	-0.000099
77	H	0.000033	0.000076	-0.000026
78	H	0.000097	-0.000011	-0.000015
79	H	-0.000004	-0.000002	0.000019
80	H	-0.000038	-0.000021	0.000045
81	H	0.000057	0.000014	0.000080
82	H	-0.000167	-0.000118	0.000079
83	H	-0.000181	-0.000123	0.000063
84	H	-0.000146	0.000149	0.000116
85	H	-0.000136	0.000075	0.000085
86	H	-0.000081	0.000385	0.000316
87	O	-0.000043	-0.000533	0.000305
88	H	0.000099	-0.000171	0.000128
89	H	0.000052	0.000088	0.000086
90	H	-0.000051	0.000011	0.000140
91	H	-0.000160	-0.000260	0.000037

92 C -0.001010 0.000414 -0.000288

93 O -0.000853 0.000478 -0.000749

94 H 0.000041 0.000094 -0.000109

95 H -0.000012 0.000090 -0.000029

96 H -0.000077 -0.000063 -0.000081

----

dE(predicted): -0.84186E-04, dE(actual): -0.38652E-04, Trust radius: 0.195959, all in a.u.

Geometry Convergence after Step 21 (Hartree/Angstrom,Angstrom)

----

current energy -19.67524827 Hartree

energy change -0.00003865 0.00100000 T

constrained gradient max 0.00272432 0.00100000 F

constrained gradient rms 0.00029115 0.00066667 T

gradient max 0.00272432

gradient rms 0.00029115

cart. step max 0.03907125 0.01000000 F

cart. step rms 0.01047159 0.00666667 F

Coordinates (Cartesian)

=======================

Atom	bohr X	bohr Y	bohr Z	angstrom X	angstrom Y	angstrom Z	Geometric Variables (0:frozen, *:LT par.)		
1 O	26.544604	61.692219	50.460514	14.046800	32.646116	26.702554	1	2	3
2 C	27.025900	61.971634	53.040701	14.301490	32.793977	28.067930	4	5	6

3 C	29.818949	61.232771	53.426368	15.779508	32.402987	28.272016	7	8	9
4 C	30.588816	61.524257	56.225242	16.186904	32.557235	29.753117	10	11	12
5 O	27.294703	52.961788	43.214019	14.443735	28.026171	22.867874	13	14	15
6 N	33.231897	60.891558	56.853218	17.585563	32.222425	30.085427	16	17	18
7 O	26.646979	64.559151	53.768786	14.100974	34.163232	28.453216	19	20	21
8 C	20.425572	58.515332	48.370603	10.808747	30.964980	25.596621	22	23	24
9 C	25.723173	65.415370	41.498279	13.612117	34.616323	21.959943	25	26	27
10 C	21.611754	55.024833	45.063433	11.436448	29.117888	23.846542	28	29	30
11 O	21.709630	62.759879	45.647013	11.488242	33.211098	24.155359	31	32	33
12 C	19.518503	56.586441	46.295685	10.328747	29.944255	24.498621	34	35	36
13 O	21.584342	55.170271	42.526280	11.421942	29.194850	22.503938	37	38	39
14 C	23.960761	61.584472	49.547337	12.679489	32.589099	26.219321	40	41	42
15 C	23.991189	63.289148	47.106094	12.695590	33.491175	24.927471	43	44	45
16 O	18.792739	59.591982	49.598972	9.944689	31.534719	26.246646	46	47	48
17 C	23.700960	57.033380	51.325586	12.542008	30.180765	27.160330	49	50	51
18 C	27.461567	53.171893	38.712520	14.532036	28.137354	20.485784	52	53	54
19 O	30.097753	58.603534	52.625013	15.927045	31.011654	27.847958	55	56	57
20 C	26.348014	57.075675	41.161367	13.942769	30.203146	21.781657	58	59	60
21 C	26.100629	54.193029	41.092308	13.811858	28.677716	21.745113	61	62	63
22 C	23.223323	58.793384	49.022498	12.289253	31.112119	25.941589	64	65	66
23 C	25.952979	62.705264	42.503380	13.733725	33.182197	22.491820	67	68	69
24 C	35.156605	61.989347	55.175545	18.604074	32.803350	29.197641	70	71	72
25 C	26.287783	62.492584	45.415961	13.910895	33.069652	24.033091	73	74	75
26 C	29.913630	64.252045	57.054179	15.829611	34.000718	30.191771	76	77	78
27 C	27.151292	64.939193	56.450696	14.367845	34.364341	29.872422	79	80	81
28 C	26.499860	67.686385	57.037321	14.023122	35.818093	30.182850	82	83	84
29 C	28.398899	58.468523	41.725383	15.028050	30.940210	22.080122	85	86	87
30 C	17.668254	57.866183	44.442971	9.349637	30.621465	23.518208	88	89	90
31 O	30.009506	62.451830	40.388851	15.880347	33.048085	21.372860	91	92	93
32 C	23.220679	53.503300	40.907793	12.287854	28.312727	21.647472	94	95	96
33 C	30.964992	57.513714	42.537578	16.385968	30.434947	22.509917	97	98	99

34 C	28.250495	61.315412	41.382044	14.949518	32.446719	21.898435	100	101	102
35 C	24.233268	66.074723	47.915204	12.823693	34.965237	25.355634	103	104	105
36 C	22.563164	50.710042	41.308363	11.939912	26.834599	21.859444	106	107	108
37 C	33.699047	58.190089	57.305170	17.832768	30.792869	30.324590	109	110	111
38 C	19.473409	64.209640	46.201928	10.304884	33.978278	24.449007	112	113	114
39 C	19.720937	50.141686	41.071898	10.435870	26.533838	21.734312	115	116	117
40 H	24.658878	58.076207	40.547392	13.048916	30.732605	21.456756	118	119	120
41 H	31.507741	58.398557	44.335636	16.673178	30.903185	23.461408	121	122	123
42 H	30.998577	55.467498	42.767545	16.403741	29.352136	22.631610	124	125	126
43 H	32.381010	58.106565	41.143880	17.135293	30.748670	21.772404	127	128	129
44 H	27.310680	66.582784	42.133938	14.452190	35.234092	22.296320	130	131	132
45 H	25.735677	65.429679	39.427142	13.618734	34.623895	20.863945	133	134	135
46 H	23.954676	66.271186	42.146055	12.676269	35.069201	22.302732	136	137	138
47 H	24.237206	61.652417	42.014483	12.825777	32.625054	22.233107	139	140	141
48 H	27.936595	63.600071	45.993591	14.783409	33.655708	24.338760	142	143	144
49 H	26.777514	60.533007	45.843245	14.170050	32.032688	24.259201	145	146	147
50 H	25.978436	66.294256	48.997128	13.747196	35.081409	25.928163	148	149	150
51 H	22.675256	66.661880	49.143116	11.999229	35.275948	26.005417	151	152	153
52 H	24.306624	67.325507	46.272630	12.862512	35.627124	24.486421	154	155	156

53 H	19.725300	66.211371	45.720672	10.438179	35.037548	24.194338	157	158	159
54 H	18.889555	64.024546	48.184043	9.995922	33.880331	25.497897	160	161	162
55 H	17.986563	63.415882	45.009256	9.518079	33.558239	23.817872	163	164	165
56 H	22.566295	54.102719	39.036270	11.941569	28.629926	20.657104	166	167	168
57 H	23.605586	49.638213	39.873098	12.491538	26.267411	21.099935	169	170	171
58 H	23.276719	50.080414	43.141130	12.317509	26.501414	22.829303	172	173	174
59 H	18.629724	51.107939	42.543674	9.858426	27.045157	22.513143	175	176	177
60 H	18.973045	50.748505	39.234168	10.040103	26.854952	20.761827	178	179	180
61 H	19.374506	48.108833	41.262998	10.252547	25.458098	21.835438	181	182	183
62 H	27.288090	51.113781	38.589461	14.440236	27.048248	20.420664	184	185	186
63 H	26.698611	54.034050	36.992248	14.128297	28.593588	19.575455	187	188	189
64 H	29.467652	53.648328	38.839326	15.593610	28.389472	20.552886	190	191	192
65 H	24.378298	58.110745	47.452866	12.900440	30.750882	25.110975	193	194	195
66 H	22.578865	57.656451	52.951377	11.948221	30.510480	28.020662	196	197	198
67 H	25.700248	57.040019	51.832735	13.599986	30.184278	27.428702	199	200	201
68 H	23.166749	55.094735	50.851678	12.259316	29.154878	26.909549	202	203	204
69 C	31.064766	58.185678	50.235246	16.438766	30.790535	26.583347	205	206	207
70 H	27.702340	68.959640	55.930624	14.659447	36.491870	29.597212	208	209	210
71 H	26.792367	68.082250	59.049680	14.177910	36.027575	31.247745	211	212	213

72 H	24.522520	68.076885	56.566461	12.976759	36.024736	29.933682	214	215	216
73 H	25.884662	63.677759	57.535785	13.697573	33.696819	30.446626	217	218	219
74 H	31.144809	65.613062	56.086939	16.481123	34.720937	29.679930	220	221	222
75 H	30.264474	64.426402	59.088977	16.015270	34.092984	31.268540	223	224	225
76 H	35.225325	61.098031	53.285766	18.640439	32.331686	28.197613	226	227	228
77 H	34.826343	64.015218	54.919008	18.429307	33.875395	29.061888	229	230	231
78 H	37.017947	61.765095	56.058196	19.589054	32.684681	29.664720	232	233	234
79 H	33.667383	57.028431	55.569272	17.816012	30.178146	29.405992	235	236	237
80 H	32.272853	57.441887	58.607334	17.078058	30.396937	31.013665	238	239	240
81 H	35.557613	57.969936	58.198664	18.816278	30.676369	30.797407	241	242	243
82 H	25.781451	60.760709	54.201192	13.642956	32.153182	28.682035	244	245	246
83 H	22.669259	62.396761	50.950526	11.996055	33.018944	26.961857	247	248	249
84 H	30.959261	62.440113	52.196892	16.382935	33.041885	27.621406	250	251	252
85 H	29.408356	60.218496	57.329099	15.562232	31.866256	30.337253	253	254	255
86 H	26.149172	53.168294	44.661016	13.837546	28.135449	23.633592	256	257	258
87 O	23.108986	53.745899	46.298802	12.228749	28.441105	24.500271	259	260	261
88 H	18.469543	55.188750	47.426565	9.773661	29.204629	25.097057	262	263	264
89 H	32.148448	54.886684	48.150766	17.012226	29.044782	25.480288	265	266	267
90 H	30.505620	54.200514	51.054441	16.142879	28.681677	27.016847	268	269	270

91 H 28.852220 55.391124 48.386982 15.267938 29.311721 25.605288 271 272 273

92 C 30.641706 55.473322 49.433070 16.214892 29.355218 26.158854 274 275 276

93 O 32.083395 59.793257 48.952738 16.977801 31.641229 25.904673 277 278 279

94 H 18.668990 59.225512 43.252087 9.879204 31.340791 22.888019 280 281 282

95 H 16.208187 58.854084 45.520396 8.577003 31.144240 24.088356 283 284 285

96 H 16.771196 56.459841 43.221321 8.874935 29.877261 22.871738 286 287 288

Number of elements of the density matrix on this node (used, total): 222269 1773786

Numerical Integration : Fuzzy Cells (Becke)

Becke grid quality: NORMAL

Lebedev angular grid order: min: 11 max: 23

Nr. of radial points: min: 46 max: 52

Total nr. of points: 600116

Nr. of blocks: 4689

Block length: 128

Nr. of dummy points: 76

Test of Precision of the Numerical Integration Grid

==

Integral of the Total Core Density: 85.99993564694756

Relative Error: -7.483E-07

Density Fitting (zlmFit)

ZlmFit Fit Quality: NORMAL

Max L-expansion: min: 6 max: 7

Nr. of radial interpolation points: min: 39 max: 48

S C F *** ScaSCF ***

Initial density matrix is taken from previous geometry

CYCLE 1

SCF Error: norm([F,P])= 0.043998367417, max([F,P])= 0.004948666453

orbitals (Q,E):

A :114...133 (2.00 -0.2491) (2.00 -0.2479) (2.00 -0.2381) (2.00 -0.2359)

(2.00 -0.2272) (2.00 -0.2218) (2.00 -0.2205) (2.00 -0.2158)

(2.00 -0.1952) (2.00 -0.1771) (0.00 -0.0698) (0.00 -0.0690)

(0.00 -0.0632) (0.00 -0.0403) (0.00 -0.0189) (0.00 -0.0132)

(0.00 -0.0116) (0.00 -0.0073) (0.00 -0.0048) (0.00 -0.0003)

CYCLE 2

SDIIS (wt 0.000): 0.0391 0.9609

A-DIIS (wt 1.000): 0.0000 1.0000

DIIS coefficients: 0.0000 1.0000

SCF Error: norm([F,P])= 0.007787163017, max([F,P])= 0.000723484066

orbitals (Q,E):

A :114...124 (2.00 -0.2479) (2.00 -0.2476) (2.00 -0.2366) (2.00 -0.2347)

(2.00 -0.2267) (2.00 -0.2214) (2.00 -0.2198) (2.00 -0.2153)

(2.00 -0.1945) (2.00 -0.1766) (0.00 -0.0696)

CYCLE 3

SDIIS (wt 0.000): 0.0019 0.6324 0.3657

A-DIIS (wt 1.000): 0.0000 0.6916 0.3084

DIIS coefficients: 0.0000 0.6916 0.3084

SCF Error: norm([F,P])= 0.013036886693, max([F,P])= 0.001065454245

orbitals (Q,E):

A :114...124 (2.00 -0.2485) (2.00 -0.2476) (2.00 -0.2372) (2.00 -0.2352)

(2.00 -0.2268) (2.00 -0.2214) (2.00 -0.2200) (2.00 -0.2154)

(2.00 -0.1946) (2.00 -0.1766) (0.00 -0.0695)

CYCLE 4

SDIIS (wt 1.000): 0.3643 0.1809 0.4549

DIIS coefficients: 0.3643 0.1809 0.4549

SCF Error: norm([F,P])= 0.002881715447, max([F,P])= 0.000435027132

orbitals (Q,E):

A :114...124 (2.00 -0.2485) (2.00 -0.2477) (2.00 -0.2373) (2.00 -0.2353)

(2.00 -0.2269) (2.00 -0.2215) (2.00 -0.2200) (2.00 -0.2155)

(2.00 -0.1948) (2.00 -0.1767) (0.00 -0.0695)

CYCLE 5

SDIIS (wt 1.000): 0.1675 0.0736 0.3294 0.4295

DIIS coefficients: 0.1675 0.0736 0.3294 0.4295

SCF Error: norm([F,P])= 0.001422729678, max([F,P])= 0.000306566155

orbitals (Q,E):

A :114...124 (2.00 -0.2485) (2.00 -0.2477) (2.00 -0.2373) (2.00 -0.2353)

(2.00 -0.2268) (2.00 -0.2215) (2.00 -0.2200) (2.00 -0.2155)

(2.00 -0.1947) (2.00 -0.1767) (0.00 -0.0696)

CYCLE 6

SDIIS (wt 1.000): 0.0711 0.0281 0.1762 0.3142 0.4104

DIIS coefficients: 0.0711 0.0281 0.1762 0.3142 0.4104

SCF Error: norm([F,P])= 0.000571009514, max([F,P])= 0.000085892798

orbitals (Q,E):

A :114...124 (2.00 -0.2485) (2.00 -0.2477) (2.00 -0.2373) (2.00 -0.2352)

(2.00 -0.2268) (2.00 -0.2215) (2.00 -0.2200) (2.00 -0.2155)

(2.00 -0.1947) (2.00 -0.1767) (0.00 -0.0695)

CYCLE 7

SDIIS (wt 1.000): 0.0204 0.0067 0.0643 0.1408 0.2727 0.4951

DIIS coefficients: 0.0204 0.0067 0.0643 0.1408 0.2727 0.4951

SCF Error: norm([F,P])= 0.000175112571, max([F,P])= 0.000021310412

orbitals (Q,E):

A :114...124 (2.00 -0.2485) (2.00 -0.2477) (2.00 -0.2373) (2.00 -0.2353)
(2.00 -0.2268) (2.00 -0.2215) (2.00 -0.2200) (2.00 -0.2155)
(2.00 -0.1947) (2.00 -0.1767) (0.00 -0.0695)

CYCLE 8

SDIIS (wt 1.000): 0.0065 0.0016 0.0259 0.0653 0.1476 0.3507 0.4024

DIIS coefficients: 0.0065 0.0016 0.0259 0.0653 0.1476 0.3507 0.4024

SCF Error: norm([F,P])= 0.000075527674, max([F,P])= 0.000014882773

orbitals (Q,E):

A :114...124 (2.00 -0.2485) (2.00 -0.2477) (2.00 -0.2373) (2.00 -0.2353)
(2.00 -0.2268) (2.00 -0.2215) (2.00 -0.2200) (2.00 -0.2155)
(2.00 -0.1947) (2.00 -0.1767) (0.00 -0.0695)

CYCLE 9

SDIIS (wt 1.000): 0.0011 0.0001 0.0076 0.0230 0.0603 0.1700 0.2971 0.4408

DIIS coefficients: 0.0011 0.0001 0.0076 0.0230 0.0603 0.1700 0.2971 0.4408

SCF Error: norm([F,P])= 0.000029575964, max([F,P])= 0.000005537617

orbitals (Q,E):

A :114...124 (2.00 -0.2485) (2.00 -0.2477) (2.00 -0.2373) (2.00 -0.2353)
(2.00 -0.2268) (2.00 -0.2215) (2.00 -0.2200) (2.00 -0.2155)
(2.00 -0.1947) (2.00 -0.1767) (0.00 -0.0695)

CYCLE 10

SDIIS (wt 1.000): -0.0000 -0.0002 0.0019 0.0072 0.0219 0.0703 0.1510 0.3027 0.4451

DIIS coefficients: -0.0000 -0.0002 0.0019 0.0072 0.0219 0.0703 0.1510 0.3027 0.4451

SCF Error: norm([F,P])= 0.000009899070, max([F,P])= 0.000001086949

orbitals (Q,E):

A :114...124 (2.00 -0.2485) (2.00 -0.2477) (2.00 -0.2373) (2.00 -0.2353)

(2.00 -0.2268) (2.00 -0.2215) (2.00 -0.2200) (2.00 -0.2155)

(2.00 -0.1947) (2.00 -0.1767) (0.00 -0.0695)

CYCLE 11

SDIIS (wt 1.000): -0.0002 -0.0001 0.0004 0.0024 0.0083 0.0294 0.0716 0.1640 0.3266 0.3976

DIIS coefficients: -0.0002 -0.0001 0.0004 0.0024 0.0083 0.0294 0.0716 0.1640 0.3266 0.3976

SCF Error: norm([F,P])= 0.000004216474, max([F,P])= 0.000000467199

orbitals (Q,E):

A :114...124 (2.00 -0.2485) (2.00 -0.2477) (2.00 -0.2373) (2.00 -0.2353)

(2.00 -0.2268) (2.00 -0.2215) (2.00 -0.2200) (2.00 -0.2155)

(2.00 -0.1947) (2.00 -0.1767) (0.00 -0.0695)

SCF CONVERGED

CYCLE 12

*** Setting up for NEW gradients in Focky

*** Using FIT density in Focky

*** Setting up for NEW Hessian in Focky

orbitals (Q,E):

A :114...133 (2.00 -0.2485) (2.00 -0.2477) (2.00 -0.2373) (2.00 -0.2353)

(2.00 -0.2268) (2.00 -0.2215) (2.00 -0.2200) (2.00 -0.2155)

(2.00 -0.1947) (2.00 -0.1767) (0.00 -0.0695) (0.00 -0.0685)

(0.00 -0.0629) (0.00 -0.0393) (0.00 -0.0187) (0.00 -0.0129)

(0.00 -0.0112) (0.00 -0.0069) (0.00 -0.0044) (0.00 -0.0001)

Fit test: (difference of exact and fit density, squared integrated, result summed over spins)

SCF: 0.00100955585737

Gross Charges per Atom (Z minus electrons)

==

-0.6655	0.9217	0.5465	0.3311	-0.5812	-0.3818	-0.6305	0.6739	0.6278	0.8636
-0.6597	-0.0013	-0.5767	0.5856	0.3252	-0.5902	0.5971	0.6015	-0.6033	0.2586
0.3379	0.0983	0.1663	0.7475	0.4169	0.4095	0.5605	0.5713	0.0185	0.6428
-0.5652	0.5506	0.5517	0.5721	0.6408	0.3722	0.7510	0.9858	0.5876	-0.2128
-0.1551	-0.2088	-0.2049	-0.2134	-0.2445	-0.2318	-0.1512	-0.1877	-0.1340	-0.2056
-0.2414	-0.2209	-0.2244	-0.1849	-0.2460	-0.1709	-0.1959	-0.1549	-0.1981	-0.2139
-0.2243	-0.2198	-0.2279	-0.1926	-0.1143	-0.2224	-0.1627	-0.2162	0.8256	-0.1967
-0.2180	-0.2177	-0.2105	-0.1902	-0.2132	-0.1801	-0.2267	-0.2350	-0.1972	-0.2409
-0.2137	-0.2503	-0.1998	-0.1583	-0.1673	0.2332	-0.6746	-0.1253	-0.1954	-0.2140
-0.0739	0.5116	-0.6194	-0.1742	-0.2165	-0.2385				

Net Total: -0.00000000

M U L T I P O L E   D E R I V E D   C H A R G E   A N A L Y S I S

See: M. Swart, P.Th. van Duijnen, J.G. Snijders, J.Comput.Chem., (2001), p. 79-88.

Multipole derived atomic charges (a.u.)

Atom		Level: MDC-m	MDC-d	MDC-q
1	O	-0.351193	-0.318949	-0.343654
2	C	0.362424	0.374416	0.396069
3	C	0.242025	0.250031	0.253104

4	C	0.180618	0.166804	0.212843
5	O	-0.307005	-0.548417	-0.609516
6	N	-0.214948	-0.280734	-0.476221
7	O	-0.380161	-0.386492	-0.451126
8	C	0.250769	0.309568	0.308242
9	C	0.228050	0.193393	0.187437
10	C	0.339240	0.408992	0.416052
11	O	-0.382874	-0.354698	-0.395102
12	C	0.124263	0.102253	0.101830
13	O	-0.312233	-0.298666	-0.343369
14	C	0.245399	0.242043	0.247636
15	C	0.196217	0.159177	0.174732
16	O	-0.369521	-0.477705	-0.482935
17	C	0.227863	0.193064	0.198756
18	C	0.229622	0.193186	0.193179
19	O	-0.319179	-0.320472	-0.372916
20	C	0.056777	0.044741	-0.059302
21	C	0.195018	0.220848	0.283699
22	C	0.118701	0.104738	0.107452
23	C	0.119756	0.099488	0.110971
24	C	0.290616	0.268674	0.286464
25	C	0.173024	0.135121	0.137361
26	C	0.164901	0.124998	0.164640
27	C	0.249191	0.239569	0.263352
28	C	0.234531	0.193898	0.303815
29	C	0.034829	0.028139	-0.042869
30	C	0.237032	0.191340	0.177237
31	O	-0.372297	-0.476609	-0.517141
32	C	0.254506	0.243407	0.263593
33	C	0.222467	0.185848	0.198260
34	C	0.228102	0.295414	0.385372

35	C	0.221779	0.192137	0.201559
36	C	0.180467	0.145620	0.151174
37	C	0.293768	0.271406	0.310799
38	C	0.368723	0.339927	0.387944
39	C	0.229611	0.190342	0.156447
40	H	-0.073342	-0.060551	-0.027071
41	H	-0.057125	-0.031657	-0.019876
42	H	-0.063961	-0.056052	-0.034280
43	H	-0.057013	-0.036475	-0.019099
44	H	-0.068953	-0.052632	-0.050733
45	H	-0.070427	-0.057574	-0.053024
46	H	-0.076689	-0.072039	-0.070349
47	H	-0.071606	-0.061755	-0.062659
48	H	-0.063563	-0.041454	-0.022774
49	H	-0.083100	-0.063400	-0.071231
50	H	-0.055232	-0.046882	-0.049543
51	H	-0.072734	-0.055493	-0.060300
52	H	-0.070676	-0.058567	-0.064989
53	H	-0.067655	-0.063692	-0.073626
54	H	-0.057962	-0.045259	-0.052151
55	H	-0.054125	-0.043989	-0.046291
56	H	-0.057165	-0.042146	-0.030863
57	H	-0.067295	-0.050499	-0.052235
58	H	-0.061757	-0.036724	-0.032501
59	H	-0.075272	-0.060501	-0.049869
60	H	-0.074704	-0.065300	-0.055720
61	H	-0.068817	-0.054945	-0.043058
62	H	-0.067019	-0.048700	-0.039946
63	H	-0.072457	-0.074543	-0.082282
64	H	-0.058296	-0.036215	-0.033365
65	H	-0.074925	-0.049707	-0.058678

66	H	-0.072188	-0.063697	-0.060630
67	H	-0.067298	-0.053884	-0.066999
68	H	-0.064901	-0.056018	-0.055630
69	C	0.353055	0.438014	0.460106
70	H	-0.064702	-0.054291	-0.082615
71	H	-0.068057	-0.066988	-0.097435
72	H	-0.063234	-0.048459	-0.074377
73	H	-0.080899	-0.062376	-0.073119
74	H	-0.072833	-0.059874	-0.063332
75	H	-0.069544	-0.068848	-0.073360
76	H	-0.076040	-0.045883	-0.040041
77	H	-0.066522	-0.054397	-0.038255
78	H	-0.065114	-0.060712	-0.028586
79	H	-0.082588	-0.063637	-0.046148
80	H	-0.066875	-0.058196	-0.051480
81	H	-0.067924	-0.066596	-0.055477
82	H	-0.071266	-0.044246	-0.037748
83	H	-0.061860	-0.026287	-0.018964
84	H	-0.047156	-0.002319	-0.015555
85	H	-0.073644	-0.059869	-0.041084
86	H	0.061816	0.261820	0.290817
87	O	-0.364166	-0.442177	-0.457562
88	H	-0.045049	0.000611	0.021236
89	H	-0.045084	-0.029547	-0.015243
90	H	-0.051895	-0.049710	-0.037813
91	H	-0.042797	-0.010735	-0.009996
92	C	0.241094	0.197757	0.179664
93	O	-0.362773	-0.462155	-0.489768
94	H	-0.061290	-0.053757	-0.049857
95	H	-0.060885	-0.047210	-0.038698
96	H	-0.068384	-0.065420	-0.061403

*** Using NEW gradient routines ***

Geometry CYCLE 22

==============

Energy gradients wrt nuclear displacements

==

Atom		Cartesian (a.u./angstrom)		
		X	Y	Z
1	O	0.000416	0.000047	-0.000217
2	C	-0.000468	0.000160	-0.000019
3	C	0.000576	-0.000416	-0.000381
4	C	0.000115	0.000067	0.000285
5	O	0.000041	0.000278	0.000022
6	N	-0.000126	0.000130	0.000021
7	O	0.000214	0.000032	0.000301
8	C	0.000340	-0.000378	-0.000241
9	C	0.000023	0.000018	-0.000025
10	C	-0.000075	0.000095	-0.000165
11	O	-0.000330	-0.000189	0.000177
12	C	-0.000017	0.000133	-0.000080
13	O	0.000039	-0.000069	-0.000109
14	C	-0.000148	-0.000001	-0.000095
15	C	0.000128	-0.000008	0.000097
16	O	-0.000453	0.000589	0.000220
17	C	0.000008	0.000105	-0.000039
18	C	-0.000036	0.000034	-0.000045
19	O	-0.000315	0.000135	0.000306

20	C	0.000173	-0.000072	0.000406
21	C	-0.000145	-0.000161	0.000069
22	C	-0.000037	-0.000129	-0.000104
23	C	0.000168	0.000112	0.000009
24	C	-0.000203	-0.000001	0.000076
25	C	-0.000081	-0.000018	0.000265
26	C	0.000002	0.000057	0.000066
27	C	0.000006	0.000104	-0.000099
28	C	0.000043	0.000053	0.000115
29	C	0.000075	-0.000147	0.000311
30	C	0.000006	-0.000002	0.000141
31	O	0.000822	0.000442	0.000125
32	C	0.000037	0.000072	-0.000101
33	C	-0.000220	-0.000204	0.000187
34	C	-0.001172	-0.000314	-0.001182
35	C	0.000101	0.000015	0.000156
36	C	-0.000073	0.000035	-0.000124
37	C	-0.000069	0.000003	-0.000067
38	C	-0.000063	-0.000160	-0.000017
39	C	0.000015	0.000031	-0.000029
40	H	-0.000016	-0.000064	-0.000052
41	H	-0.000062	-0.000107	0.000103
42	H	0.000008	-0.000234	0.000022
43	H	0.000050	0.000101	-0.000008
44	H	0.000023	0.000035	0.000008
45	H	0.000016	-0.000008	-0.000072
46	H	-0.000025	0.000013	-0.000028
47	H	0.000191	0.000053	0.000102
48	H	0.000078	-0.000030	-0.000061
49	H	0.000030	-0.000133	0.000084
50	H	0.000075	0.000001	-0.000002

51	H	-0.000010	0.000035	0.000069
52	H	0.000010	0.000031	-0.000004
53	H	-0.000048	0.000077	0.000013
54	H	-0.000061	-0.000015	0.000111
55	H	-0.000103	-0.000027	-0.000047
56	H	-0.000072	-0.000003	-0.000008
57	H	0.000094	-0.000045	0.000058
58	H	-0.000072	-0.000108	0.000042
59	H	-0.000049	0.000047	0.000029
60	H	-0.000024	-0.000005	-0.000069
61	H	-0.000018	-0.000053	0.000031
62	H	0.000007	-0.000118	-0.000058
63	H	-0.000004	0.000027	-0.000026
64	H	0.000095	0.000003	0.000022
65	H	0.000029	0.000078	-0.000005
66	H	-0.000070	-0.000032	0.000061
67	H	-0.000165	-0.000081	0.000045
68	H	0.000080	-0.000065	-0.000050
69	C	-0.000721	-0.000334	-0.000254
70	H	0.000014	0.000027	-0.000010
71	H	-0.000009	0.000043	0.000061
72	H	-0.000063	0.000020	-0.000002
73	H	-0.000005	-0.000037	0.000007
74	H	-0.000013	0.000009	-0.000068
75	H	-0.000008	0.000055	0.000048
76	H	-0.000004	-0.000048	-0.000119
77	H	-0.000006	0.000046	0.000015
78	H	0.000070	-0.000038	-0.000012
79	H	-0.000003	-0.000040	-0.000070
80	H	-0.000033	-0.000004	0.000012
81	H	0.000034	0.000013	0.000023

```
 82 H     0.000049 -0.000062  0.000078
 83 H     0.000071  0.000052  0.000104
 84 H     0.000189  0.000087 -0.000109
 85 H    -0.000025 -0.000051 -0.000050
 86 H     0.000096  0.000389  0.000080
 87 O     0.000506 -0.000565 -0.000207
 88 H    -0.000014 -0.000030  0.000017
 89 H     0.000044 -0.000060 -0.000094
 90 H    -0.000029 -0.000124  0.000018
 91 H     0.000006  0.000037  0.000146
 92 C     0.000264 -0.000099 -0.000162
 93 O     0.000409  0.000792  0.000080
 94 H     0.000003  0.000006 -0.000060
 95 H    -0.000066  0.000082  0.000014
 96 H    -0.000063 -0.000021 -0.000014
----------------------------------------

dE(predicted):    -0.35760E-04, dE(actual):    -0.36340E-04, Trust radius:    0.195959, all in a.u.

----------------------------------------------------------------------
Geometry Convergence after Step  22       (Hartree/Angstrom,Angstrom)
----------------------------------------------------------------------
current energy                          -19.67528461 Hartree
energy change                     -0.00003634     0.00100000    T
constrained gradient max           0.00118173     0.00100000    F
constrained gradient rms           0.00019151     0.00066667    T
gradient max                       0.00118173
gradient rms                       0.00019151
cart. step max                     0.02315611     0.01000000    F
cart. step rms                     0.00520532     0.00666667    T
```

Coordinates (Cartesian)

=======================

Atom	bohr X	bohr Y	bohr Z	angstrom X	angstrom Y	angstrom Z	Geometric Variables (0:frozen, *:LT par.)		
1 O	26.539748	61.690590	50.459716	14.044230	32.645255	26.702132	1	2	3
2 C	27.022800	61.968379	53.039552	14.299850	32.792254	28.067322	4	5	6
3 C	29.815167	61.227300	53.423289	15.777507	32.400092	28.270387	7	8	9
4 C	30.589261	61.520643	56.219979	16.187140	32.555323	29.750332	10	11	12
5 O	27.304886	52.966489	43.220096	14.449123	28.028659	22.871090	13	14	15
6 N	33.233336	60.886603	56.841916	17.586324	32.219803	30.079447	16	17	18
7 O	26.646403	64.556068	53.766932	14.100669	34.161600	28.452235	19	20	21
8 C	20.418963	58.517487	48.373246	10.805250	30.966121	25.598019	22	23	24
9 C	25.716392	65.415277	41.500997	13.608529	34.616274	21.961382	25	26	27
10 C	21.613348	55.028373	45.071610	11.437291	29.119761	23.850869	28	29	30
11 O	21.711011	62.758686	45.640607	11.488972	33.210466	24.151969	31	32	33
12 C	19.515456	56.587291	46.298579	10.327134	29.944705	24.500153	34	35	36
13 O	21.591198	55.173292	42.534459	11.425570	29.196449	22.508266	37	38	39
14 C	23.955703	61.585420	49.547377	12.676812	32.589601	26.219343	40	41	42
15 C	23.988010	63.289039	47.104829	12.693908	33.491117	24.926802	43	44	45
16 O	18.785555	59.592428	49.600777	9.940888	31.534955	26.247601	46	47	48
17 C	23.690002	57.037629	51.332471	12.536209	30.183013	27.163974	49	50	51
18 C	27.469158	53.180333	38.718798	14.536053	28.141820	20.489105	52	53	54
19 O	30.091944	58.596356	52.626490	15.923971	31.007856	27.848739	55	56	57
20 C	26.356013	57.083014	41.169799	13.947001	30.207030	21.786119	58	59	60
21 C	26.109957	54.200191	41.099988	13.816794	28.681506	21.749177	61	62	63
22 C	23.216037	58.794693	49.026344	12.285398	31.112812	25.943624	64	65	66
23 C	25.954515	62.706393	42.506748	13.734538	33.182794	22.493602	67	68	69

24	C	35.154606	61.980750	55.158008	18.603017	32.798800	29.188361	70	71	72
25	C	26.288523	62.495893	45.418989	13.911287	33.071402	24.034694	73	74	75
26	C	29.916135	64.248737	57.048796	15.830937	33.998967	30.188923	76	77	78
27	C	27.153562	64.936604	56.448206	14.369046	34.362971	29.871104	79	80	81
28	C	26.503709	67.684094	57.034043	14.025159	35.816880	30.181116	82	83	84
29	C	28.409757	58.476408	41.721717	15.033796	30.944382	22.078182	85	86	87
30	C	17.668287	57.865425	44.441926	9.349655	30.621064	23.517654	88	89	90
31	O	30.003781	62.462780	40.377887	15.877317	33.053880	21.367058	91	92	93
32	C	23.230696	53.507817	40.917825	12.293155	28.315118	21.652780	94	95	96
33	C	30.983819	57.523510	42.510156	16.395931	30.440131	22.495406	97	98	99
34	C	28.255990	61.323422	41.385707	14.952426	32.450957	21.900373	100	101	102
35	C	24.223526	66.075159	47.912634	12.818538	34.965468	25.354274	103	104	105
36	C	22.577774	50.714259	41.321430	11.947643	26.836830	21.866359	106	107	108
37	C	33.699018	58.185437	57.295871	17.832752	30.790408	30.319669	109	110	111
38	C	19.476594	64.214600	46.185516	10.306570	33.980903	24.440323	112	113	114
39	C	19.735656	50.143130	41.093930	10.443660	26.534602	21.745971	115	116	117
40	H	24.663213	58.083175	40.566021	13.051210	30.736293	21.466614	118	119	120
41	H	31.545267	58.412859	44.300075	16.693036	30.910754	23.442590	121	122	123
42	H	31.019153	55.478184	42.745186	16.414629	29.357791	22.619778	124	125	126
43	H	32.385400	58.111399	41.100121	17.137616	30.751228	21.749247	127	128	129
44	H	27.300495	66.587361	42.136080	14.446800	35.236514	22.297453	130	131	132
45	H	25.728481	65.429190	39.430066	13.614926	34.623636	20.865492	133	134	135
46	H	23.945656	66.265879	42.148977	12.671495	35.066393	22.304278	136	137	138

47	H	24.242094	61.648296	42.017206	12.828364	32.622873	22.234548	139	140 141
48	H	27.933668	63.608042	45.997753	14.781861	33.659926	24.340963	142	143 144
49	H	26.782649	60.537942	45.847890	14.172768	32.035300	24.261658	145	146 147
50	H	25.963825	66.297104	49.001639	13.739465	35.082917	25.930550	148	149 150
51	H	22.659677	66.661165	49.133417	11.990985	35.275569	26.000285	151	152 153
52	H	24.302218	67.324909	46.269696	12.860180	35.626807	24.484869	154	155 156
53	H	19.734455	66.214225	45.699416	10.443024	35.039059	24.183090	157	158 159
54	H	18.887305	64.036108	48.166362	9.994731	33.886449	25.488541	160	161 162
55	H	17.991496	63.420930	44.991029	9.520690	33.560911	23.808227	163	164 165
56	H	22.575220	54.104379	39.045912	11.946292	28.630804	20.662207	166	167 168
57	H	23.616501	49.643633	39.882790	12.497314	26.270279	21.105064	169	170 171
58	H	23.297923	50.086153	43.151954	12.328730	26.504451	22.835031	172	173 174
59	H	18.648461	51.107058	42.569941	9.868341	27.044691	22.527043	175	176 177
60	H	18.981388	50.750662	39.259250	10.044518	26.856094	20.775100	178	179 180
61	H	19.392130	48.109923	41.284553	10.261873	25.458675	21.846845	181	182 183
62	H	27.296856	51.122336	38.595942	14.444874	27.052775	20.424093	184	185 186
63	H	26.703677	54.042023	36.999607	14.130978	28.597807	19.579349	187	188 189
64	H	29.474876	53.658023	38.843003	15.597432	28.394603	20.554832	190	191 192
65	H	24.371621	58.108863	47.458602	12.896906	30.749886	25.114011	193	194 195

66	H	22.571406	57.667985	52.957556	11.944274	30.516583	28.023932	196	197	198
67	H	25.689773	57.038942	51.837564	13.594442	30.183708	27.431258	199	200	201
68	H	23.148840	55.099915	50.863246	12.249839	29.157619	26.915670	202	203	204
69	C	31.051841	58.170014	50.235958	16.431926	30.782246	26.583724	205	206	207
70	H	27.705868	68.956110	55.925845	14.661314	36.490002	29.594683	208	209	210
71	H	26.798079	68.080597	59.045813	14.180933	36.026701	31.245699	211	212	213
72	H	24.526343	68.075163	56.564573	12.978782	36.023825	29.932683	214	215	216
73	H	25.887507	63.676094	57.534840	13.699079	33.695938	30.446126	217	218	219
74	H	31.146755	65.608921	56.079953	16.482153	34.718746	29.676233	220	221	222
75	H	30.269579	64.423373	59.082943	16.017972	34.091381	31.265347	223	224	225
76	H	35.218735	61.085490	53.270222	18.636952	32.325049	28.189387	226	227	228
77	H	34.824025	64.005987	54.898202	18.428080	33.870510	29.050878	229	230	231
78	H	37.017548	61.757939	56.037263	19.588843	32.680894	29.653642	232	233	234
79	H	33.660899	57.021973	55.561442	17.812580	30.174728	29.401849	235	236	237
80	H	32.275986	57.440350	58.603071	17.079716	30.396124	31.011410	238	239	240
81	H	35.559931	57.964336	58.183861	18.817505	30.673405	30.789573	241	242	243
82	H	25.778287	60.759044	54.201135	13.641282	32.152301	28.682005	244	245	246
83	H	22.665339	62.400808	50.949539	11.993981	33.021085	26.961335	247	248	249
84	H	30.954902	62.432093	52.191120	16.380629	33.037641	27.618351	250	251	252

85 H 29.410720 60.215876 57.326824 15.563483 31.864869 30.336049 253 254 255

86 H 26.154427 53.159721 44.664679 13.840326 28.130913 23.635530 256 257 258

87 O 23.112018 53.756636 46.311172 12.230353 28.446787 24.506817 259 260 261

88 H 18.465389 55.189590 47.428141 9.771463 29.205074 25.097891 262 263 264

89 H 32.121766 54.862022 48.159474 16.998106 29.031732 25.484896 265 266 267

90 H 30.483190 54.189362 51.068394 16.131009 28.675775 27.024230 268 269 270

91 H 28.827910 55.376499 48.400310 15.255073 29.303981 25.612341 271 272 273

92 C 30.619452 55.456631 49.443038 16.203116 29.346385 26.164129 274 275 276

93 O 32.069243 59.770360 48.944936 16.970313 31.629113 25.900545 277 278 279

94 H 18.670154 59.227220 43.255175 9.879820 31.341695 22.889653 280 281 282

95 H 16.203937 58.849925 45.516294 8.574754 31.142039 24.086185 283 284 285

96 H 16.776986 56.458622 43.216932 8.877999 29.876616 22.869415 286 287 288

Number of elements of the density matrix on this node (used, total): 222269 1773786

Numerical Integration : Fuzzy Cells (Becke)

Becke grid quality: NORMAL

Lebedev angular grid order: min: 11 max: 23

Nr. of radial points: min: 46 max: 52

Total nr. of points: 600105

Nr. of blocks: 4689

Block length: 128

Nr. of dummy points: 87

Test of Precision of the Numerical Integration Grid

===

Integral of the Total Core Density: 85.99993323084675

Relative Error: -7.764E-07

Density Fitting (zlmFit)

ZlmFit Fit Quality: NORMAL

Max L-expansion: min: 6 max: 7

Nr. of radial interpolation points: min: 39 max: 48

S C F *** ScaSCF ***

Initial density matrix is taken from previous geometry

CYCLE 1

SCF Error: norm([F,P])= 0.023562885022, max([F,P])= 0.002899755681

orbitals (Q,E):

A :114...133 (2.00 -0.2480) (2.00 -0.2473) (2.00 -0.2368) (2.00 -0.2349)

(2.00 -0.2265) (2.00 -0.2210) (2.00 -0.2196) (2.00 -0.2150)

(2.00 -0.1941) (2.00 -0.1763) (0.00 -0.0690) (0.00 -0.0679)

(0.00 -0.0623) (0.00 -0.0386) (0.00 -0.0184) (0.00 -0.0125)

(0.00 -0.0105) (0.00 -0.0066) (0.00 -0.0042) (0.00 0.0002)

CYCLE 2

MIX coefficients: 0.8000 0.2000

SCF Error: norm([F,P])= 0.004408237107, max([F,P])= 0.000465395395

orbitals (Q,E):

A :114...124 (2.00 -0.2482) (2.00 -0.2474) (2.00 -0.2369) (2.00 -0.2350)

(2.00 -0.2266) (2.00 -0.2211) (2.00 -0.2197) (2.00 -0.2151)

(2.00 -0.1943) (2.00 -0.1764) (0.00 -0.0691)

CYCLE 3

SDIIS (wt 0.000): -0.8397 1.8397

A-DIIS (wt 1.000): 0.0000 1.0000

DIIS coefficients: 0.0000 1.0000

SCF Error: norm([F,P])= 0.002419590216, max([F,P])= 0.000299181012

orbitals (Q,E):

A :114...124 (2.00 -0.2485) (2.00 -0.2477) (2.00 -0.2373) (2.00 -0.2354)

(2.00 -0.2269) (2.00 -0.2215) (2.00 -0.2203) (2.00 -0.2155)

(2.00 -0.1948) (2.00 -0.1767) (0.00 -0.0696)

CYCLE 4

SDIIS (wt 1.000): 0.3829 -0.0060 0.6231

DIIS coefficients: 0.3829 -0.0060 0.6231

SCF Error: norm([F,P])= 0.002726853686, max([F,P])= 0.000227176728

orbitals (Q,E):

A :114...124 (2.00 -0.2484) (2.00 -0.2476) (2.00 -0.2372) (2.00 -0.2353)

(2.00 -0.2268) (2.00 -0.2214) (2.00 -0.2201) (2.00 -0.2154)

(2.00 -0.1946) (2.00 -0.1766) (0.00 -0.0694)

CYCLE 5

SDIIS (wt 1.000): 0.3504 -0.2239 0.4131 0.4604

DIIS coefficients: 0.3504 -0.2239 0.4131 0.4604

SCF Error: norm([F,P])= 0.000771448077, max([F,P])= 0.000139434809

orbitals (Q,E):

A :114...124 (2.00 -0.2484) (2.00 -0.2477) (2.00 -0.2372) (2.00 -0.2353)

(2.00 -0.2268) (2.00 -0.2214) (2.00 -0.2201) (2.00 -0.2154)

(2.00 -0.1947) (2.00 -0.1766) (0.00 -0.0694)

CYCLE 6

SDIIS (wt 1.000): 0.1772 -0.1368 0.1927 0.3029 0.4640

DIIS coefficients: 0.1772 -0.1368 0.1927 0.3029 0.4640

SCF Error: norm([F,P])= 0.000263110196, max([F,P])= 0.000038186818

orbitals (Q,E):

A :114...124 (2.00 -0.2484) (2.00 -0.2477) (2.00 -0.2372) (2.00 -0.2353)

(2.00 -0.2269) (2.00 -0.2214) (2.00 -0.2201) (2.00 -0.2154)

(2.00 -0.1947) (2.00 -0.1766) (0.00 -0.0694)

CYCLE 7

SDIIS (wt 1.000): 0.0729 -0.0632 0.0738 0.1389 0.3068 0.4709

DIIS coefficients: 0.0729 -0.0632 0.0738 0.1389 0.3068 0.4709

SCF Error: norm([F,P])= 0.000092700243, max([F,P])= 0.000014273566

orbitals (Q,E):

A :114...124 (2.00 -0.2484) (2.00 -0.2477) (2.00 -0.2372) (2.00 -0.2353)

(2.00 -0.2268) (2.00 -0.2214) (2.00 -0.2201) (2.00 -0.2154)

(2.00 -0.1947) (2.00 -0.1766) (0.00 -0.0694)

CYCLE 8

SDIIS (wt 1.000): 0.0294 -0.0281 0.0276 0.0596 0.1604 0.3359 0.4152

DIIS coefficients: 0.0294 -0.0281 0.0276 0.0596 0.1604 0.3359 0.4152

SCF Error: norm([F,P])= 0.000040005299, max([F,P])= 0.000007339672

orbitals (Q,E):

A :114...124 (2.00 -0.2484) (2.00 -0.2477) (2.00 -0.2372) (2.00 -0.2353)

(2.00 -0.2268) (2.00 -0.2214) (2.00 -0.2201) (2.00 -0.2154)

(2.00 -0.1947) (2.00 -0.1766) (0.00 -0.0694)

CYCLE 9

SDIIS (wt 1.000): 0.0108 -0.0112 0.0093 0.0227 0.0719 0.1781 0.3161 0.4023

DIIS coefficients: 0.0108 -0.0112 0.0093 0.0227 0.0719 0.1781 0.3161 0.4023

SCF Error: norm([F,P])= 0.000017163405, max([F,P])= 0.000003016601

orbitals (Q,E):

A :114...124 (2.00 -0.2484) (2.00 -0.2477) (2.00 -0.2372) (2.00 -0.2353)

(2.00 -0.2268) (2.00 -0.2214) (2.00 -0.2201) (2.00 -0.2154)

(2.00 -0.1947) (2.00 -0.1766) (0.00 -0.0694)

CYCLE 10

SDIIS (wt 1.000): 0.0031 -0.0035 0.0024 0.0067 0.0256 0.0716 0.1513 0.2611 0.4818

DIIS coefficients: 0.0031 -0.0035 0.0024 0.0067 0.0256 0.0716 0.1513 0.2611 0.4818

SCF Error: norm([F,P])= 0.000004669572, max([F,P])= 0.000000794418

orbitals (Q,E):

A :114...124 (2.00 -0.2484) (2.00 -0.2477) (2.00 -0.2372) (2.00 -0.2353)

(2.00 -0.2268) (2.00 -0.2214) (2.00 -0.2201) (2.00 -0.2154)

(2.00 -0.1947) (2.00 -0.1766) (0.00 -0.0694)

SCF CONVERGED

CYCLE 11

*** Setting up for NEW gradients in Focky

*** Using FIT density in Focky

*** Setting up for NEW Hessian in Focky

orbitals (Q,E):

A :114...133 (2.00 -0.2484) (2.00 -0.2477) (2.00 -0.2372) (2.00 -0.2353)

(2.00 -0.2268) (2.00 -0.2214) (2.00 -0.2201) (2.00 -0.2154)

(2.00 -0.1947) (2.00 -0.1766) (0.00 -0.0694) (0.00 -0.0685)

(0.00 -0.0627) (0.00 -0.0391) (0.00 -0.0187) (0.00 -0.0128)

(0.00 -0.0110) (0.00 -0.0069) (0.00 -0.0044) (0.00 -0.0001)

Fit test: (difference of exact and fit density, squared integrated, result summed over spins)

SCF: 0.00100986634752

Gross Charges per Atom (Z minus electrons)

==

-0.6655	0.9220	0.5468	0.3313	-0.5809	-0.3818	-0.6306	0.6738	0.6287	0.8640
-0.6599	-0.0011	-0.5769	0.5857	0.3255	-0.5901	0.5976	0.6020	-0.6034	0.2585
0.3380	0.0983	0.1659	0.7478	0.4171	0.4096	0.5606	0.5716	0.0194	0.6429
-0.5656	0.5500	0.5519	0.5738	0.6409	0.3727	0.7513	0.9866	0.5879	-0.2128
-0.1564	-0.2092	-0.2047	-0.2138	-0.2447	-0.2316	-0.1511	-0.1883	-0.1345	-0.2059
-0.2415	-0.2207	-0.2240	-0.1853	-0.2468	-0.1709	-0.1959	-0.1553	-0.1981	-0.2139
-0.2245	-0.2200	-0.2281	-0.1924	-0.1139	-0.2224	-0.1627	-0.2167	0.8259	-0.1968
-0.2182	-0.2178	-0.2105	-0.1903	-0.2133	-0.1801	-0.2269	-0.2352	-0.1971	-0.2410
-0.2139	-0.2504	-0.2000	-0.1586	-0.1674	0.2324	-0.6748	-0.1254	-0.1956	-0.2139
-0.0735	0.5116	-0.6198	-0.1742	-0.2167	-0.2386				

Net Total: 0.00000000

M U L T I P O L E D E R I V E D C H A R G E A N A L Y S I S

See: M. Swart, P.Th. van Duijnen, J.G. Snijders, J.Comput.Chem., (2001), p. 79-88.

Multipole derived atomic charges (a.u.)

Atom	Level:	MDC-m	MDC-d	MDC-q
1	O	-0.351148	-0.318844	-0.343621
2	C	0.362375	0.374306	0.396022
3	C	0.242123	0.250092	0.253287
4	C	0.180638	0.166804	0.212832
5	O	-0.307081	-0.548471	-0.609271

6	N	-0.214958	-0.280793	-0.476240
7	O	-0.380084	-0.386446	-0.451052
8	C	0.250718	0.309787	0.308554
9	C	0.228089	0.193479	0.187441
10	C	0.339162	0.409040	0.415855
11	O	-0.382687	-0.354413	-0.394592
12	C	0.124223	0.102359	0.101953
13	O	-0.312281	-0.298664	-0.343190
14	C	0.245378	0.241915	0.247542
15	C	0.196132	0.159164	0.174543
16	O	-0.369403	-0.477793	-0.483148
17	C	0.228006	0.193267	0.199090
18	C	0.229602	0.193218	0.192931
19	O	-0.319223	-0.320298	-0.372818
20	C	0.056831	0.044656	-0.059973
21	C	0.195037	0.220817	0.283688
22	C	0.118668	0.104719	0.107393
23	C	0.119691	0.099311	0.110449
24	C	0.290655	0.268725	0.286537
25	C	0.172980	0.135146	0.137361
26	C	0.164908	0.125009	0.164698
27	C	0.249183	0.239586	0.263339
28	C	0.234550	0.193947	0.303996
29	C	0.034972	0.028283	-0.043320
30	C	0.237036	0.191341	0.177401
31	O	-0.372265	-0.476832	-0.518278
32	C	0.254514	0.243446	0.263536
33	C	0.222684	0.185981	0.198776
34	C	0.228132	0.295965	0.388124
35	C	0.221745	0.192230	0.201637
36	C	0.180492	0.145715	0.151484

37	C	0.293782	0.271448	0.310903
38	C	0.368759	0.339958	0.387871
39	C	0.229657	0.190406	0.156530
40	H	-0.073264	-0.060304	-0.026756
41	H	-0.056812	-0.031558	-0.019597
42	H	-0.063991	-0.056149	-0.034444
43	H	-0.057040	-0.036498	-0.019463
44	H	-0.069028	-0.052775	-0.050907
45	H	-0.070399	-0.057550	-0.052778
46	H	-0.076721	-0.072061	-0.070433
47	H	-0.071441	-0.061417	-0.062528
48	H	-0.063717	-0.041751	-0.023287
49	H	-0.082979	-0.063515	-0.071100
50	H	-0.055321	-0.047111	-0.049775
51	H	-0.072792	-0.055526	-0.060301
52	H	-0.070667	-0.058489	-0.064904
53	H	-0.067634	-0.063574	-0.073584
54	H	-0.058031	-0.045391	-0.052162
55	H	-0.054164	-0.044152	-0.046455
56	H	-0.057172	-0.042199	-0.030923
57	H	-0.067279	-0.050471	-0.052250
58	H	-0.061844	-0.036802	-0.032801
59	H	-0.075304	-0.060536	-0.049936
60	H	-0.074671	-0.065248	-0.055659
61	H	-0.068821	-0.054996	-0.043127
62	H	-0.067079	-0.048788	-0.039864
63	H	-0.072485	-0.074539	-0.082188
64	H	-0.058345	-0.036291	-0.033389
65	H	-0.074856	-0.049455	-0.058411
66	H	-0.072194	-0.063717	-0.060687
67	H	-0.067310	-0.053883	-0.067117

68	H	-0.064908	-0.056051	-0.055582
69	C	0.352986	0.438035	0.460253
70	H	-0.064712	-0.054315	-0.082658
71	H	-0.068080	-0.067041	-0.097520
72	H	-0.063230	-0.048462	-0.074431
73	H	-0.080894	-0.062369	-0.073107
74	H	-0.072847	-0.059930	-0.063455
75	H	-0.069567	-0.068862	-0.073391
76	H	-0.076046	-0.045888	-0.040108
77	H	-0.066520	-0.054406	-0.038278
78	H	-0.065119	-0.060758	-0.028614
79	H	-0.082597	-0.063645	-0.046187
80	H	-0.066888	-0.058233	-0.051549
81	H	-0.067954	-0.066648	-0.055497
82	H	-0.071264	-0.044254	-0.037728
83	H	-0.061953	-0.026500	-0.019218
84	H	-0.047206	-0.002444	-0.015779
85	H	-0.073620	-0.059866	-0.041064
86	H	0.061940	0.261788	0.290594
87	O	-0.364318	-0.442342	-0.457670
88	H	-0.045106	0.000434	0.021141
89	H	-0.045150	-0.029635	-0.015371
90	H	-0.052014	-0.049923	-0.038178
91	H	-0.042731	-0.010632	-0.009750
92	C	0.241041	0.197766	0.179769
93	O	-0.362832	-0.462053	-0.489798
94	H	-0.061311	-0.053790	-0.049930
95	H	-0.060935	-0.047301	-0.038783
96	H	-0.068395	-0.065497	-0.061560

*** Using NEW gradient routines ***

Geometry CYCLE 23

==============

Energy gradients wrt nuclear displacements

==

Atom Cartesian (a.u./angstrom)

X Y Z

----

1 O 0.000318 0.000120 -0.000176

2 C -0.000427 0.000050 0.000121

3 C 0.000437 -0.000257 -0.000149

4 C 0.000095 0.000048 0.000244

5 O -0.000195 0.000044 -0.000099

6 N -0.000115 -0.000021 -0.000170

7 O 0.000140 -0.000104 0.000109

8 C -0.000319 0.000369 0.000148

9 C -0.000016 0.000025 0.000020

10 C 0.000101 -0.000660 0.000234

11 O 0.000096 0.000135 -0.000075

12 C 0.000027 0.000086 0.000076

13 O 0.000042 -0.000045 0.000115

14 C -0.000065 -0.000082 -0.000033

15 C -0.000112 -0.000075 0.000079

16 O 0.000333 -0.000332 -0.000156

17 C -0.000071 -0.000022 0.000041

18 C 0.000044 0.000030 -0.000022

19 O 0.000049 -0.000001 -0.000013

20 C 0.000084 0.000228 0.000266

21 C 0.000017 0.000002 0.000178

22 C -0.000175 -0.000370 0.000090

23 C -0.000281 -0.000030 -0.000510

24 C -0.000108 0.000045 -0.000062

25 C 0.000107 -0.000021 0.000175

26 C -0.000120 0.000083 0.000038

27 C 0.000047 0.000051 -0.000013

28 C -0.000005 0.000059 0.000059

29 C -0.000237 -0.000349 -0.000267

30 C 0.000012 0.000088 0.000047

31 O -0.000463 -0.000238 -0.000190

32 C 0.000101 -0.000090 0.000020

33 C -0.000039 -0.000122 -0.000128

34 C 0.000719 0.000300 0.000708

35 C -0.000194 0.000043 -0.000093

36 C 0.000053 0.000032 0.000048

37 C -0.000103 0.000024 -0.000006

38 C 0.000067 0.000116 0.000124

39 C -0.000025 0.000093 -0.000060

40 H 0.000096 0.000010 -0.000266

41 H 0.000011 0.000012 -0.000072

42 H 0.000016 0.000094 -0.000077

43 H -0.000004 0.000078 0.000096

44 H -0.000040 -0.000051 -0.000002

45 H 0.000000 -0.000006 0.000045

46 H 0.000037 -0.000021 -0.000026

47 H 0.000063 0.000016 0.000082

48 H -0.000026 -0.000036 -0.000042

49 H 0.000017 0.000021 0.000021

50 H -0.000050 -0.000057 -0.000067

51 H -0.000038 -0.000027 -0.000044

52 H -0.000035 -0.000010 0.000008

53	H	0.000072	-0.000008	0.000021
54	H	0.000050	0.000043	-0.000069
55	H	0.000053	0.000031	0.000038
56	H	-0.000003	-0.000013	0.000047
57	H	0.000032	0.000042	0.000048
58	H	-0.000016	0.000036	-0.000024
59	H	0.000030	0.000039	-0.000025
60	H	0.000036	-0.000031	0.000018
61	H	0.000026	0.000051	0.000018
62	H	0.000001	0.000019	-0.000027
63	H	0.000027	-0.000026	0.000066
64	H	0.000002	0.000006	0.000016
65	H	-0.000034	-0.000064	-0.000066
66	H	0.000013	-0.000039	-0.000069
67	H	-0.000145	-0.000037	0.000001
68	H	0.000049	-0.000005	-0.000027
69	C	-0.000049	0.000452	-0.000250
70	H	-0.000026	-0.000042	0.000030
71	H	-0.000014	-0.000008	-0.000049
72	H	0.000037	-0.000002	0.000014
73	H	0.000019	0.000002	-0.000033
74	H	-0.000061	-0.000047	-0.000015
75	H	-0.000003	0.000017	-0.000069
76	H	-0.000062	-0.000020	0.000018
77	H	-0.000003	-0.000055	0.000023
78	H	-0.000043	-0.000024	-0.000024
79	H	-0.000026	0.000015	-0.000062
80	H	0.000009	0.000033	-0.000040
81	H	-0.000059	0.000017	-0.000048
82	H	0.000122	0.000035	0.000053
83	H	0.000125	0.000034	-0.000063

```
  84 H     0.000083  0.000029  0.000036
  85 H     0.000037 -0.000002 -0.000062
  86 H     0.000034 -0.000133 -0.000203
  87 O    -0.000206  0.000700 -0.000164
  88 H     0.000017  0.000029 -0.000018
  89 H    -0.000069 -0.000007  0.000016
  90 H     0.000007  0.000026 -0.000070
  91 H     0.000023  0.000024  0.000099
  92 C     0.000205 -0.000041  0.000014
  93 O    -0.000169 -0.000315  0.000453
  94 H    -0.000042 -0.000048  0.000037
  95 H     0.000034 -0.000021 -0.000023
  96 H     0.000019  0.000035  0.000059
 ----------------------------------------

 dE(predicted):    -0.21255E-04, dE(actual):    -0.82387E-05, Trust radius:    0.195959, all in a.u.

 ----------------------------------------------------------------------
 Geometry Convergence after Step  23       (Hartree/Angstrom,Angstrom)
 ----------------------------------------------------------------------
 current energy                          -19.67529284 Hartree
 energy change                     -0.00000824     0.00100000    T
 constrained gradient max           0.00071854     0.00100000    T
 constrained gradient rms           0.00014678     0.00066667    T
 gradient max                      0.00071854
 gradient rms                      0.00014678
 cart. step max                    0.01383801     0.01000000    F
 cart. step rms                    0.00249410     0.00666667    T
```

Coordinates (Cartesian)

======================

Atom	bohr X	bohr Y	bohr Z	angstrom X	angstrom Y	angstrom Z	Geometric Variables (0:frozen, *:LT par.)		
1 O	26.536868	61.690344	50.461060	14.042706	32.645124	26.702843	1	2	3
2 C	27.022085	61.967423	53.040199	14.299472	32.791748	28.067665	4	5	6
3 C	29.815252	61.227331	53.421489	15.777552	32.400108	28.269435	7	8	9
4 C	30.593291	61.521744	56.217214	16.189272	32.555905	29.748868	10	11	12
5 O	27.303390	52.965181	43.220229	14.448332	28.027967	22.871160	13	14	15
6 N	33.239094	60.892581	56.839489	17.589371	32.222966	30.078162	16	17	18
7 O	26.644199	64.554717	53.767743	14.099503	34.160885	28.452664	19	20	21
8 C	20.418326	58.515712	48.372503	10.804913	30.965181	25.597626	22	23	24
9 C	25.723248	65.419886	41.505659	13.612157	34.618713	21.963849	25	26	27
10 C	21.612205	55.024805	45.070813	11.436686	29.117873	23.850447	28	29	30
11 O	21.711903	62.760285	45.639261	11.489444	33.211313	24.151257	31	32	33
12 C	19.515415	56.585521	46.297063	10.327113	29.943768	24.499350	34	35	36
13 O	21.591024	55.172108	42.533929	11.425478	29.195822	22.507986	37	38	39
14 C	23.953299	61.586352	49.548009	12.675540	32.590094	26.219677	40	41	42
15 C	23.987752	63.290225	47.105593	12.693772	33.491745	24.927206	43	44	45
16 O	18.783833	59.591479	49.598159	9.939976	31.534453	26.246216	46	47	48
17 C	23.689900	57.041034	51.334815	12.536155	30.184815	27.165214	49	50	51
18 C	27.468913	53.181729	38.718542	14.535923	28.142559	20.488970	52	53	54
19 O	30.091600	58.595908	52.625288	15.923789	31.007619	27.848103	55	56	57
20 C	26.354971	57.083320	41.171967	13.946450	30.207192	21.787267	58	59	60
21 C	26.109317	54.200296	41.100018	13.816455	28.681562	21.749193	61	62	63
22 C	23.215022	58.795561	49.026894	12.284861	31.113271	25.943915	64	65	66
23 C	25.956514	62.709576	42.508778	13.735596	33.184479	22.494677	67	68	69
24 C	35.159993	61.989615	55.156801	18.605867	32.803491	29.187722	70	71	72
25 C	26.288776	62.496260	45.420812	13.911421	33.071596	24.035659	73	74	75

26 C	29.917770	64.249146	57.046424	15.831802	33.999184	30.187668	76	77	78
27 C	27.153857	64.934586	56.448637	14.369202	34.361903	29.871333	79	80	81
28 C	26.502203	67.681412	57.035428	14.024362	35.815461	30.181849	82	83	84
29 C	28.408472	58.477192	41.725381	15.033116	30.944797	22.080120	85	86	87
30 C	17.671509	57.864919	44.438011	9.351360	30.620796	23.515583	88	89	90
31 O	30.008126	62.462426	40.385242	15.879617	33.053692	21.370950	91	92	93
32 C	23.230163	53.507542	40.915989	12.292873	28.314972	21.651809	94	95	96
33 C	30.981333	57.524602	42.518316	16.394615	30.440708	22.499724	97	98	99
34 C	28.256114	61.324028	41.387043	14.952491	32.451278	21.901080	100	101	102
35 C	24.224845	66.075877	47.914772	12.819236	34.965848	25.355405	103	104	105
36 C	22.577839	50.713120	41.316409	11.947678	26.836227	21.863702	106	107	108
37 C	33.711311	58.193230	57.298474	17.839257	30.794531	30.321047	109	110	111
38 C	19.475795	64.214598	46.181611	10.306147	33.980902	24.438256	112	113	114
39 C	19.735583	50.140724	41.092696	10.443621	26.533328	21.745318	115	116	117
40 H	24.660450	58.083729	40.573366	13.049748	30.736586	21.470501	118	119	120
41 H	31.541992	58.417913	44.306505	16.691303	30.913428	23.445993	121	122	123
42 H	31.014666	55.479784	42.758587	16.412254	29.358637	22.626870	124	125	126
43 H	32.384402	58.107453	41.107612	17.137087	30.749140	21.753211	127	128	129
44 H	27.309208	66.588716	42.142280	14.451410	35.237231	22.300734	130	131	132
45 H	25.735849	65.435893	39.434713	13.618825	34.627183	20.867951	133	134	135
46 H	23.953770	66.272859	42.154182	12.675789	35.070087	22.307033	136	137	138
47 H	24.242479	61.654796	42.017061	12.828567	32.626313	22.234471	139	140	141

48	H	27.934559	63.606491	46.001560	14.782332	33.659106	24.342977	142	143	144
49	H	26.780741	60.537549	45.848364	14.171758	32.035091	24.261910	145	146	147
50	H	25.964306	66.295958	49.005666	13.739719	35.082310	25.932681	148	149	150
51	H	22.660285	66.662871	49.134232	11.991306	35.276472	26.000716	151	152	153
52	H	24.306307	67.326226	46.272382	12.862344	35.627504	24.486290	154	155	156
53	H	19.732938	66.214660	45.696836	10.442221	35.039289	24.181724	157	158	159
54	H	18.883610	64.034613	48.161525	9.992776	33.885658	25.485982	160	161	162
55	H	17.992973	63.420517	44.984516	9.521471	33.560692	23.804780	163	164	165
56	H	22.575033	54.106290	39.044605	11.946193	28.631815	20.661515	166	167	168
57	H	23.614421	49.645062	39.874266	12.496214	26.271035	21.100553	169	170	171
58	H	23.301172	50.082183	43.144746	12.330449	26.502350	22.831216	172	173	174
59	H	18.650397	51.100263	42.573048	9.869365	27.041095	22.528687	175	176	177
60	H	18.977463	50.752207	39.260886	10.042441	26.856911	20.775966	178	179	180
61	H	19.393640	48.106802	41.279021	10.262672	25.457023	21.843917	181	182	183
62	H	27.297057	51.123727	38.595022	14.444980	27.053511	20.423606	184	185	186
63	H	26.703272	54.043875	36.999567	14.130763	28.598787	19.579327	187	188	189
64	H	29.474500	53.659828	38.843005	15.597233	28.395558	20.554833	190	191	192
65	H	24.372115	58.109854	47.460254	12.897168	30.750411	25.114885	193	194	195
66	H	22.571772	57.673280	52.959564	11.944467	30.519386	28.024994	196	197	198

67 H 25.689986 57.043303 51.839149 13.594555 30.186016 27.432096 199 200
201

68 H 23.148717 55.102729 50.868175 12.249774 29.159108 26.918279 202 203
204

69 C 31.048576 58.166539 50.233829 16.430199 30.780407 26.582597 205 206
207

70 H 27.702118 68.954688 55.926148 14.659329 36.489249 29.594843 208 209
210

71 H 26.798292 68.077895 59.046984 14.181045 36.025271 31.246318 211 212
213

72 H 24.523997 68.070759 56.568015 12.977540 36.021494 29.934504 214 215
216

73 H 25.889892 63.672814 57.536301 13.700341 33.694202 30.446899 217 218
219

74 H 31.146348 65.610730 56.076851 16.481938 34.719703 29.674592 220 221
222

75 H 30.272796 64.423717 59.080353 16.019674 34.091563 31.263976 223 224
225

76 H 35.229408 61.092249 53.270180 18.642600 32.328626 28.189365 226 227
228

77 H 34.824656 64.013741 54.893955 18.428414 33.874613 29.048630 229 230
231

78 H 37.022241 61.772782 56.039081 19.591326 32.688748 29.654605 232 233
234

79 H 33.677167 57.026408 55.566246 17.821189 30.177075 29.404391 235 236
237

80 H 32.289520 57.446835 58.606354 17.086878 30.399556 31.013147 238 239
240

81 H 35.572318 57.978358 58.187934 18.824060 30.680826 30.791729 241 242
243

82 H 25.778418 60.757524 54.202129 13.641351 32.151497 28.682531 244 245
246

83 H 22.661960 62.401130 50.949800 11.992193 33.021256 26.961473 247 248
249

84 H 30.952711 62.431846 52.187064 16.379469 33.037510 27.616205 250 251
252

85 H 29.417300 60.216062 57.325788 15.566965 31.864968 30.335501 253 254
255

86 H 26.152351 53.157470 44.664659 13.839228 28.129722 23.635520 256 257
258

87 O 23.105754 53.745960 46.309902 12.227039 28.441137 24.506145 259 260
261

88 H 18.462899 55.188570 47.425302 9.770145 29.204533 25.096389 262 263
264

89 H 32.100292 54.854487 48.155001 16.986743 29.027745 25.482529 265 266
267

90 H 30.457040 54.188597 51.062972 16.117171 28.675370 27.021361 268 269
270

91 H 28.809393 55.387037 48.394789 15.245274 29.309558 25.609419 271 272
273

92 C 30.600865 55.456389 49.438585 16.193280 29.346257 26.161772 274 275
276

93 O 32.072551 59.762641 48.942402 16.972063 31.625028 25.899204 277 278
279

94 H 18.675864 59.226224 43.252690 9.882842 31.341168 22.888338 280 281
282

95 H 16.206290 58.850232 45.510513 8.575999 31.142202 24.083126 283 284
285

96 H 16.781015 56.458704 43.211651 8.880131 29.876660 22.866621 286 287
288

Number of elements of the density matrix on this node (used, total): 222269 1773786

Numerical Integration : Fuzzy Cells (Becke)

Becke grid quality: NORMAL

Lebedev angular grid order: min: 11 max: 23

Nr. of radial points: min: 46 max: 52

Total nr. of points: 600117

Nr. of blocks: 4689

Block length: 128

Nr. of dummy points: 75

Test of Precision of the Numerical Integration Grid

==

Integral of the Total Core Density: 85.99993151145561

Relative Error: -7.964E-07

Density Fitting (zlmFit)

ZlmFit Fit Quality: NORMAL

Max L-expansion: min: 6 max: 7

Nr. of radial interpolation points: min: 39 max: 48

S C F *** ScaSCF ***

Initial density matrix is taken from previous geometry

CYCLE 1

SCF Error: norm([F,P])= 0.013102211587, max([F,P])= 0.001456402339

orbitals (Q,E):

A :114...133 (2.00 -0.2484) (2.00 -0.2477) (2.00 -0.2372) (2.00 -0.2353)

(2.00 -0.2268) (2.00 -0.2215) (2.00 -0.2202) (2.00 -0.2154)

(2.00 -0.1947) (2.00 -0.1766) (0.00 -0.0696) (0.00 -0.0686)

(0.00 -0.0628) (0.00 -0.0391) (0.00 -0.0187) (0.00 -0.0128)

(0.00 -0.0111) (0.00 -0.0068) (0.00 -0.0044) (0.00 -0.0001)

CYCLE 2

SDIIS (wt 0.000): 0.0427 0.9573

A-DIIS (wt 1.000): 0.0000 1.0000

DIIS coefficients: 0.0000 1.0000

SCF Error: norm([F,P])= 0.002275685693, max([F,P])= 0.000211357183

orbitals (Q,E):

A :114...124 (2.00 -0.2485) (2.00 -0.2476) (2.00 -0.2373) (2.00 -0.2353)

(2.00 -0.2270) (2.00 -0.2214) (2.00 -0.2200) (2.00 -0.2154)

(2.00 -0.1946) (2.00 -0.1766) (0.00 -0.0695)

CYCLE 3

SDIIS (wt 0.000): 0.0052 0.6291 0.3657

A-DIIS (wt 1.000): 0.0000 0.6845 0.3155

DIIS coefficients: 0.0000 0.6845 0.3155

SCF Error: norm([F,P])= 0.003766387504, max([F,P])= 0.000267558015

orbitals (Q,E):

A :114...124 (2.00 -0.2485) (2.00 -0.2476) (2.00 -0.2373) (2.00 -0.2353)

(2.00   -0.2269) (2.00   -0.2214) (2.00   -0.2201) (2.00   -0.2154)

(2.00   -0.1946) (2.00   -0.1766) (0.00   -0.0695)

CYCLE   4

SDIIS (wt 1.000): 0.3617 0.2002 0.4381

DIIS coefficients: 0.3617 0.2002 0.4381

SCF Error: norm([F,P])=   0.000907785848, max([F,P])=   0.000122260554

orbitals (Q,E):

A :114...124   (2.00   -0.2485) (2.00   -0.2476) (2.00   -0.2373) (2.00   -0.2353)

(2.00   -0.2269) (2.00   -0.2214) (2.00   -0.2201) (2.00   -0.2155)

(2.00   -0.1947) (2.00   -0.1766) (0.00   -0.0695)

CYCLE   5

SDIIS (wt 1.000): 0.1821 0.0970 0.3264 0.3945

DIIS coefficients: 0.1821 0.0970 0.3264 0.3945

SCF Error: norm([F,P])=   0.000476050571, max([F,P])=   0.000078728003

orbitals (Q,E):

A :114...124   (2.00   -0.2485) (2.00   -0.2476) (2.00   -0.2373) (2.00   -0.2353)

(2.00   -0.2269) (2.00   -0.2214) (2.00   -0.2201) (2.00   -0.2154)

(2.00   -0.1946) (2.00   -0.1766) (0.00   -0.0695)

CYCLE   6

SDIIS (wt 1.000): 0.0735 0.0379 0.1597 0.2657 0.4631

DIIS coefficients: 0.0735 0.0379 0.1597 0.2657 0.4631

SCF Error: norm([F,P])=   0.000137506204, max([F,P])=   0.000012838748

orbitals (Q,E):

A :114...124   (2.00   -0.2485) (2.00   -0.2476) (2.00   -0.2373) (2.00   -0.2353)

(2.00   -0.2269) (2.00   -0.2214) (2.00   -0.2201) (2.00   -0.2154)

(2.00   -0.1946) (2.00   -0.1766) (0.00   -0.0695)

CYCLE   7

SDIIS (wt 1.000): 0.0214 0.0103 0.0566 0.1156 0.3090 0.4870

DIIS coefficients: 0.0214 0.0103 0.0566 0.1156 0.3090 0.4870

SCF Error: norm([F,P])=   0.000050328036, max([F,P])=   0.000005503404

orbitals (Q,E):

A :114...124   (2.00   -0.2485) (2.00   -0.2476) (2.00   -0.2373) (2.00   -0.2353)

(2.00   -0.2269) (2.00   -0.2214) (2.00   -0.2201) (2.00   -0.2154)

(2.00   -0.1946) (2.00   -0.1766) (0.00   -0.0695)

CYCLE   8

SDIIS (wt 1.000): 0.0062 0.0026 0.0214 0.0509 0.1688 0.3493 0.4009

DIIS coefficients: 0.0062 0.0026 0.0214 0.0509 0.1688 0.3493 0.4009

SCF Error: norm([F,P])=   0.000022523626, max([F,P])=   0.000002841687

orbitals (Q,E):

A :114...124   (2.00   -0.2485) (2.00   -0.2476) (2.00   -0.2373) (2.00   -0.2353)

(2.00   -0.2269) (2.00   -0.2214) (2.00   -0.2201) (2.00   -0.2154)

(2.00   -0.1946) (2.00   -0.1766) (0.00   -0.0695)

CYCLE   9

SDIIS (wt 1.000): 0.0011 0.0003 0.0063 0.0178 0.0726 0.1750 0.3028 0.4241

DIIS coefficients: 0.0011 0.0003 0.0063 0.0178 0.0726 0.1750 0.3028 0.4241

SCF Error: norm([F,P])=   0.000009080854, max([F,P])=   0.000001170578

orbitals (Q,E):

A :114...124   (2.00   -0.2485) (2.00   -0.2476) (2.00   -0.2373) (2.00   -0.2353)

(2.00   -0.2269) (2.00   -0.2214) (2.00   -0.2201) (2.00   -0.2154)

(2.00   -0.1946) (2.00   -0.1766) (0.00   -0.0695)

CYCLE 10

SDIIS (wt 1.000): -0.0001 -0.0001 0.0014 0.0052 0.0261 0.0693 0.1485 0.2758 0.4740

DIIS coefficients: -0.0001 -0.0001 0.0014 0.0052 0.0261 0.0693 0.1485 0.2758 0.4740

SCF Error: norm([F,P])= 0.000002563524, max([F,P])= 0.000000279592

orbitals (Q,E):

A :114...124 (2.00 -0.2485) (2.00 -0.2476) (2.00 -0.2373) (2.00 -0.2353)

(2.00 -0.2269) (2.00 -0.2214) (2.00 -0.2201) (2.00 -0.2154)

(2.00 -0.1946) (2.00 -0.1766) (0.00 -0.0695)

SCF CONVERGED

CYCLE 11

*** Setting up for NEW gradients in Focky

*** Using FIT density in Focky

*** Setting up for NEW Hessian in Focky

orbitals (Q,E):

A :114...133 (2.00 -0.2485) (2.00 -0.2476) (2.00 -0.2373) (2.00 -0.2353)

(2.00 -0.2269) (2.00 -0.2214) (2.00 -0.2201) (2.00 -0.2154)

(2.00 -0.1946) (2.00 -0.1766) (0.00 -0.0695) (0.00 -0.0685)

(0.00 -0.0627) (0.00 -0.0392) (0.00 -0.0187) (0.00 -0.0128)

(0.00 -0.0111) (0.00 -0.0068) (0.00 -0.0045) (0.00 -0.0001)

Fit test: (difference of exact and fit density, squared integrated, result summed over spins)

SCF: 0.00100986375129

Gross Charges per Atom (Z minus electrons)

==

-0.6656	0.9222	0.5465	0.3312	-0.5808	-0.3818	-0.6306	0.6737	0.6286	0.8636
-0.6599	-0.0013	-0.5769	0.5858	0.3253	-0.5900	0.5975	0.6020	-0.6033	0.2586
0.3379	0.0982	0.1660	0.7476	0.4171	0.4097	0.5605	0.5716	0.0189	0.6430
-0.5655	0.5499	0.5519	0.5733	0.6408	0.3728	0.7510	0.9862	0.5877	-0.2125
-0.1560	-0.2090	-0.2049	-0.2137	-0.2447	-0.2317	-0.1512	-0.1883	-0.1342	-0.2056
-0.2416	-0.2207	-0.2240	-0.1853	-0.2465	-0.1709	-0.1959	-0.1551	-0.1981	-0.2140
-0.2244	-0.2199	-0.2280	-0.1925	-0.1143	-0.2224	-0.1621	-0.2169	0.8253	-0.1968
-0.2182	-0.2178	-0.2105	-0.1902	-0.2131	-0.1801	-0.2268	-0.2349	-0.1975	-0.2408
-0.2136	-0.2503	-0.1999	-0.1587	-0.1673	0.2323	-0.6746	-0.1253	-0.1957	-0.2141
-0.0729	0.5120	-0.6198	-0.1741	-0.2168	-0.2385				

Net Total: 0.00000000

M U L T I P O L E D E R I V E D C H A R G E A N A L Y S I S

See: M. Swart, P.Th. van Duijnen, J.G. Snijders, J.Comput.Chem., (2001), p. 79-88.

Multipole derived atomic charges (a.u.)

Atom		Level: MDC-m	MDC-d	MDC-q
1	O	-0.351221	-0.318787	-0.343579
2	C	0.362361	0.374277	0.395937
3	C	0.242230	0.250087	0.253311
4	C	0.180699	0.166741	0.212756
5	O	-0.307027	-0.548281	-0.608958

6	N	-0.214965	-0.280199	-0.475214
7	O	-0.380006	-0.386253	-0.450905
8	C	0.250708	0.309646	0.308481
9	C	0.228081	0.193471	0.187537
10	C	0.339131	0.409118	0.416009
11	O	-0.382748	-0.354344	-0.394510
12	C	0.124229	0.102353	0.101921
13	O	-0.312205	-0.298283	-0.342824
14	C	0.245437	0.241975	0.247570
15	C	0.196163	0.159144	0.174529
16	O	-0.369375	-0.477706	-0.483048
17	C	0.227990	0.193250	0.199096
18	C	0.229598	0.193172	0.192669
19	O	-0.319439	-0.320803	-0.373277
20	C	0.056769	0.044662	-0.060344
21	C	0.195051	0.220770	0.283679
22	C	0.118704	0.104747	0.107445
23	C	0.119701	0.099381	0.110637
24	C	0.290673	0.268672	0.286438
25	C	0.173015	0.135246	0.137490
26	C	0.164881	0.124951	0.164575
27	C	0.249139	0.239501	0.263287
28	C	0.234541	0.193945	0.303922
29	C	0.034972	0.028233	-0.042940
30	C	0.237043	0.191367	0.177417
31	O	-0.372268	-0.476778	-0.517917
32	C	0.254510	0.243402	0.263481
33	C	0.222620	0.185936	0.199032
34	C	0.228059	0.295731	0.387140
35	C	0.221719	0.192174	0.201558
36	C	0.180486	0.145669	0.151569

37	C	0.293826	0.271413	0.310750
38	C	0.368767	0.339938	0.387906
39	C	0.229676	0.190416	0.156426
40	H	-0.073300	-0.060523	-0.026880
41	H	-0.056727	-0.031399	-0.019557
42	H	-0.064014	-0.056157	-0.034497
43	H	-0.057074	-0.036570	-0.019630
44	H	-0.069012	-0.052736	-0.050835
45	H	-0.070409	-0.057580	-0.052915
46	H	-0.076726	-0.072053	-0.070439
47	H	-0.071452	-0.061492	-0.062561
48	H	-0.063714	-0.041763	-0.023326
49	H	-0.083038	-0.063546	-0.071183
50	H	-0.055296	-0.047059	-0.049704
51	H	-0.072794	-0.055554	-0.060307
52	H	-0.070681	-0.058519	-0.064932
53	H	-0.067632	-0.063587	-0.073631
54	H	-0.058037	-0.045445	-0.052215
55	H	-0.054148	-0.044102	-0.046443
56	H	-0.057170	-0.042211	-0.030962
57	H	-0.067313	-0.050509	-0.052406
58	H	-0.061809	-0.036742	-0.032681
59	H	-0.075291	-0.060479	-0.049845
60	H	-0.074692	-0.065308	-0.055661
61	H	-0.068793	-0.054922	-0.043022
62	H	-0.067045	-0.048708	-0.039671
63	H	-0.072464	-0.074542	-0.082117
64	H	-0.058351	-0.036346	-0.033378
65	H	-0.074900	-0.049658	-0.058557
66	H	-0.072152	-0.063589	-0.060602
67	H	-0.067290	-0.053814	-0.067023

68	H	-0.064926	-0.056041	-0.055635
69	C	0.353080	0.438038	0.460820
70	H	-0.064711	-0.054312	-0.082644
71	H	-0.068092	-0.067066	-0.097517
72	H	-0.063236	-0.048470	-0.074432
73	H	-0.080883	-0.062377	-0.073055
74	H	-0.072848	-0.059944	-0.063456
75	H	-0.069581	-0.068877	-0.073419
76	H	-0.076018	-0.045938	-0.040115
77	H	-0.066528	-0.054425	-0.038400
78	H	-0.065122	-0.060757	-0.028759
79	H	-0.082602	-0.063650	-0.046093
80	H	-0.066885	-0.058252	-0.051671
81	H	-0.067907	-0.066552	-0.055455
82	H	-0.071274	-0.044255	-0.037734
83	H	-0.061877	-0.026424	-0.019143
84	H	-0.047216	-0.002492	-0.015870
85	H	-0.073590	-0.059859	-0.041116
86	H	0.061992	0.261915	0.290541
87	O	-0.364620	-0.443022	-0.458362
88	H	-0.045074	0.000503	0.021197
89	H	-0.045152	-0.029602	-0.015273
90	H	-0.051989	-0.049911	-0.038138
91	H	-0.042750	-0.010628	-0.009800
92	C	0.241062	0.197803	0.179439
93	O	-0.362812	-0.461838	-0.489675
94	H	-0.061314	-0.053788	-0.049895
95	H	-0.060967	-0.047378	-0.038883
96	H	-0.068363	-0.065445	-0.061524

*** Using NEW gradient routines ***

Geometry CYCLE 24

==============

Energy gradients wrt nuclear displacements

==

Atom	Cartesian (a.u./angstrom)		
	X	Y	Z
1 O	-0.000075	0.000146	0.000105
2 C	-0.000071	0.000004	-0.000006
3 C	-0.000067	-0.000029	-0.000062
4 C	-0.000014	-0.000072	0.000003
5 O	-0.000085	-0.000035	-0.000086
6 N	0.000032	0.000012	0.000048
7 O	-0.000042	0.000004	-0.000077
8 C	-0.000065	0.000091	0.000220
9 C	0.000013	-0.000014	0.000047
10 C	0.000178	0.000022	-0.000088
11 O	0.000069	0.000109	-0.000129
12 C	-0.000089	-0.000022	-0.000041
13 O	-0.000180	-0.000022	0.000185
14 C	-0.000056	-0.000030	0.000006
15 C	-0.000035	-0.000029	0.000047
16 O	0.000147	-0.000171	-0.000118
17 C	-0.000056	0.000088	0.000100
18 C	-0.000041	-0.000032	-0.000072
19 O	0.000074	0.000020	0.000018
20 C	-0.000051	0.000052	-0.000032
21 C	0.000108	0.000021	0.000084

22	C	-0.000010	-0.000076	0.000060
23	C	-0.000047	0.000062	-0.000083
24	C	0.000049	0.000053	-0.000050
25	C	0.000053	-0.000022	0.000124
26	C	-0.000008	0.000021	-0.000027
27	C	0.000014	-0.000109	-0.000011
28	C	-0.000022	0.000027	-0.000025
29	C	0.000062	-0.000082	0.000049
30	C	0.000115	-0.000044	-0.000001
31	O	-0.000171	-0.000085	0.000106
32	C	0.000138	-0.000093	-0.000057
33	C	-0.000066	-0.000031	0.000149
34	C	0.000180	0.000098	-0.000311
35	C	-0.000063	-0.000011	0.000025
36	C	0.000040	0.000041	0.000028
37	C	0.000059	-0.000120	0.000052
38	C	-0.000035	0.000081	-0.000006
39	C	-0.000052	-0.000006	-0.000047
40	H	-0.000045	-0.000000	0.000046
41	H	0.000030	-0.000007	0.000018
42	H	0.000027	0.000072	-0.000018
43	H	-0.000039	0.000083	0.000021
44	H	-0.000022	-0.000022	-0.000004
45	H	0.000010	-0.000015	0.000024
46	H	0.000002	-0.000006	0.000017
47	H	0.000025	-0.000037	0.000020
48	H	-0.000018	-0.000015	-0.000014
49	H	0.000016	0.000036	0.000002
50	H	0.000009	-0.000036	-0.000022
51	H	-0.000001	-0.000002	-0.000027
52	H	-0.000012	-0.000002	-0.000030

53 H	0.000026	-0.000017	-0.000004
54 H	0.000021	0.000022	-0.000054
55 H	0.000037	0.000019	0.000034
56 H	-0.000016	0.000028	0.000027
57 H	0.000038	0.000017	0.000043
58 H	-0.000003	0.000007	0.000009
59 H	0.000010	0.000029	-0.000031
60 H	0.000026	-0.000025	0.000012
61 H	-0.000006	0.000022	0.000017
62 H	-0.000005	0.000002	-0.000028
63 H	0.000011	-0.000014	0.000020
64 H	-0.000036	-0.000008	0.000008
65 H	-0.000001	0.000057	0.000045
66 H	0.000018	0.000006	-0.000025
67 H	0.000021	0.000009	0.000018
68 H	0.000054	0.000058	-0.000001
69 C	0.000161	0.000162	-0.000053
70 H	-0.000012	-0.000014	0.000014
71 H	-0.000004	-0.000021	-0.000029
72 H	0.000031	-0.000002	0.000008
73 H	0.000000	0.000003	-0.000009
74 H	-0.000027	-0.000024	-0.000013
75 H	0.000011	-0.000008	-0.000032
76 H	0.000025	0.000006	0.000031
77 H	0.000017	-0.000018	0.000016
78 H	-0.000020	-0.000022	-0.000018
79 H	0.000006	0.000022	0.000043
80 H	0.000016	0.000018	-0.000019
81 H	-0.000024	-0.000003	-0.000012
82 H	0.000007	0.000032	-0.000025
83 H	0.000050	0.000006	-0.000003

```
  84 H    -0.000073 -0.000013  0.000043
  85 H    -0.000002  0.000031 -0.000010
  86 H    -0.000018 -0.000045 -0.000067
  87 O    -0.000167  0.000000 -0.000103
  88 H    -0.000024 -0.000007 -0.000034
  89 H    -0.000055  0.000040 -0.000004
  90 H     0.000034  0.000014  0.000007
  91 H    -0.000047 -0.000019  0.000020
  92 C     0.000006  0.000113 -0.000056
  93 O    -0.000025 -0.000289  0.000030
  94 H     0.000000 -0.000023  0.000009
  95 H     0.000022 -0.000018 -0.000002
  96 H     0.000002  0.000005  0.000015
 ----------------------------------------

 dE(predicted):    -0.78757E-05, dE(actual):    -0.34617E-05, Trust radius:    0.195959, all in a.u.

----------------------------------------------------------------------
Geometry Convergence after Step  24       (Hartree/Angstrom,Angstrom)
----------------------------------------------------------------------
current energy                            -19.67529631 Hartree
energy change                     -0.00000346     0.00100000    T
constrained gradient max           0.00031061     0.00100000    T
constrained gradient rms           0.00006224     0.00066667    T
gradient max                       0.00031061
gradient rms                       0.00006224
cart. step max                     0.01233907     0.01000000    F
cart. step rms                     0.00259181     0.00666667    T
```

Coordinates (Cartesian)

======================

Atom	bohr X	bohr Y	bohr Z	angstrom X	angstrom Y	angstrom Z	Geometric Variables (0:frozen, *:LT par.)		
1 O	26.539608	61.686191	50.459927	14.044156	32.642927	26.702243	1	2	3
2 C	27.022973	61.964638	53.039439	14.299941	32.790274	28.067262	4	5	6
3 C	29.816266	61.227218	53.423290	15.778088	32.400048	28.270388	7	8	9
4 C	30.592457	61.524529	56.219189	16.188831	32.557378	29.749914	10	11	12
5 O	27.299985	52.969130	43.218238	14.446530	28.030057	22.870107	13	14	15
6 N	33.238826	60.899151	56.842308	17.589229	32.226443	30.079654	16	17	18
7 O	26.642231	64.551882	53.765945	14.098462	34.159385	28.451713	19	20	21
8 C	20.418999	58.515396	48.371549	10.805269	30.965014	25.597121	22	23	24
9 C	25.728940	65.422683	41.506013	13.615169	34.620193	21.964036	25	26	27
10 C	21.609006	55.025489	45.068980	11.434994	29.118235	23.849477	28	29	30
11 O	21.713172	62.759541	45.640069	11.490116	33.210919	24.151685	31	32	33
12 C	19.513164	56.586055	46.296858	10.325922	29.944051	24.499242	34	35	36
13 O	21.587107	55.174024	42.532117	11.423405	29.196836	22.507027	37	38	39
14 C	23.956094	61.583495	49.546411	12.677019	32.588582	26.218832	40	41	42
15 C	23.989906	63.289162	47.105283	12.694912	33.491182	24.927042	43	44	45
16 O	18.786326	59.590591	49.600541	9.941296	31.533983	26.247476	46	47	48
17 C	23.694148	57.035313	51.326932	12.538403	30.181788	27.161043	49	50	51
18 C	27.464133	53.182268	38.716244	14.533393	28.142844	20.487754	52	53	54
19 O	30.094854	58.595082	52.629880	15.925511	31.007182	27.850533	55	56	57
20 C	26.351673	57.084997	41.168178	13.944705	30.208080	21.785261	58	59	60
21 C	26.104730	54.202135	41.097222	13.814028	28.682535	21.747713	61	62	63
22 C	23.216740	58.793615	49.022384	12.285770	31.112241	25.941529	64	65	66
23 C	25.958824	62.711418	42.507561	13.736818	33.185453	22.494033	67	68	69
24 C	35.158537	61.998571	55.159875	18.605096	32.808231	29.189349	70	71	72
25 C	26.290058	62.495370	45.419379	13.912100	33.071126	24.034900	73	74	75

26	C	29.913405	64.251336	57.047330	15.829492	34.000343	30.188147	76	77	78
27	C	27.149319	64.933750	56.447100	14.366801	34.361460	29.870519	79	80	81
28	C	26.494369	67.680170	57.032044	14.020216	35.814804	30.180058	82	83	84
29	C	28.406620	58.476800	41.721277	15.032136	30.944590	22.077949	85	86	87
30	C	17.666825	57.865596	44.440129	9.348881	30.621155	23.516704	88	89	90
31	O	30.011336	62.461595	40.385864	15.881315	33.053253	21.371279	91	92	93
32	C	23.225369	53.509380	40.913795	12.290336	28.315944	21.650648	94	95	96
33	C	30.978320	57.519571	42.512649	16.393021	30.438046	22.496725	97	98	99
34	C	28.257362	61.324122	41.385699	14.953152	32.451328	21.900369	100	101	102
35	C	24.228461	66.074189	47.916268	12.821149	34.964955	25.356197	103	104	105
36	C	22.573147	50.714677	41.313456	11.945195	26.837051	21.862139	106	107	108
37	C	33.714572	58.200619	57.301695	17.840983	30.798441	30.322751	109	110	111
38	C	19.475889	64.211004	46.185315	10.306196	33.979000	24.440216	112	113	114
39	C	19.730336	50.142372	41.097021	10.440844	26.534201	21.747607	115	116	117
40	H	24.657114	58.086690	40.571983	13.047983	30.738153	21.469769	118	119	120
41	H	31.541705	58.411993	44.300448	16.691151	30.910296	23.442787	121	122	123
42	H	31.007491	55.474690	42.753689	16.408457	29.355942	22.624278	124	125	126
43	H	32.381766	58.098686	41.100807	17.135693	30.744501	21.749611	127	128	129
44	H	27.316212	66.589299	42.143527	14.455117	35.237539	22.301394	130	131	132
45	H	25.741809	65.439976	39.435067	13.621979	34.629344	20.868139	133	134	135
46	H	23.960394	66.277393	42.154753	12.679294	35.072486	22.307335	136	137	138
47	H	24.243720	61.659042	42.014258	12.829224	32.628560	22.232988	139	140	141

48 H	27.936605	63.603834	46.001478	14.783415	33.657699	24.342934	142	143	144
49 H	26.780354	60.535817	45.845052	14.171553	32.034175	24.260157	145	146	147
50 H	25.968011	66.292852	49.007292	13.741680	35.080666	25.933542	148	149	150
51 H	22.664354	66.661091	49.136337	11.993460	35.275530	26.001830	151	152	153
52 H	24.310413	67.325672	46.274811	12.864516	35.627211	24.487576	154	155	156
53 H	19.730799	66.212024	45.703378	10.441089	35.037894	24.185186	157	158	159
54 H	18.884445	64.027459	48.165156	9.993218	33.881872	25.487903	160	161	162
55 H	17.993413	63.417297	44.987510	9.521704	33.558988	23.806365	163	164	165
56 H	22.569806	54.108284	39.042608	11.943427	28.632871	20.660459	166	167	168
57 H	23.605491	49.647893	39.867309	12.491488	26.272534	21.096872	169	170	171
58 H	23.301311	50.081688	43.139150	12.330523	26.502088	22.828255	172	173	174
59 H	18.649377	51.098453	42.582683	9.868825	27.040137	22.533785	175	176	177
60 H	18.966570	50.757560	39.268784	10.036677	26.859744	20.780146	178	179	180
61 H	19.389356	48.107979	41.280097	10.260405	25.457646	21.844487	181	182	183
62 H	27.292955	51.124115	38.594690	14.442810	27.053716	20.423430	184	185	186
63 H	26.697707	54.042677	36.996708	14.127818	28.598153	19.577815	187	188	189
64 H	29.469605	53.661151	38.839678	15.594643	28.396258	20.553073	190	191	192
65 H	24.371603	58.109402	47.453444	12.896897	30.750171	25.111281	193	194	195
66 H	22.579519	57.666249	52.954613	11.948567	30.515665	28.022375	196	197	198

67 H 25.695163 57.035239 51.827592 13.597295 30.181749 27.425980 199 200
201

68 H 23.150154 55.098143 50.858557 12.250534 29.156681 26.913189 202 203
204

69 C 31.055066 58.163978 50.240276 16.433633 30.779052 26.586009 205 206
207

70 H 27.693636 68.954126 55.922799 14.654841 36.488952 29.593071 208 209
210

71 H 26.788764 68.078127 59.043589 14.176003 36.025394 31.244522 211 212
213

72 H 24.516024 68.067131 56.563120 12.973321 36.019575 29.931914 214 215
216

73 H 25.885796 63.671221 57.534395 13.698173 33.693359 30.445891 217 218
219

74 H 31.141413 65.614050 56.078582 16.479326 34.721460 29.675508 220 221
222

75 H 30.266637 64.426644 59.081537 16.016414 34.093112 31.264603 223 224
225

76 H 35.230165 61.100356 53.273750 18.643001 32.332916 28.191255 226 227
228

77 H 34.819647 64.021959 54.895753 18.425764 33.878962 29.049581 229 230
231

78 H 37.020805 61.785631 56.043102 19.590566 32.695548 29.656733 232 233
234

79 H 33.682129 57.033440 55.569619 17.823815 30.180796 29.406176 235 236
237

80 H 32.293734 57.452433 58.609627 17.089108 30.402518 31.014879 238 239
240

81 H 35.575856 57.988361 58.191272 18.825932 30.686119 30.793495 241 242
243

82 H 25.779433 60.753971 54.200798 13.641889 32.149617 28.681827 244 245
246

83 H 22.664837 62.397158 50.948970 11.993715 33.019154 26.961034 247 248
249

84 H 30.953593 62.431259 52.188197 16.379936 33.037199 27.616804 250 251
252

85 H 29.417837 60.217639 57.327837 15.567249 31.865802 30.336585 253 254
255

86 H 26.150870 53.164654 44.663868 13.838445 28.133523 23.635101 256 257
258

87 O 23.103706 53.746589 46.306933 12.225954 28.441470 24.504574 259 260
261

88 H 18.461903 55.189033 47.426182 9.769618 29.204778 25.096855 262 263
264

89 H 32.123610 54.849694 48.173543 16.999082 29.025208 25.492341 265 266
267

90 H 30.472331 54.186176 51.077497 16.125263 28.674089 27.029047 268 269
270

91 H 28.830317 55.373219 48.400593 15.256347 29.302246 25.612491 271 272
273

92 C 30.617907 55.450364 49.450475 16.202299 29.343069 26.168064 274 275
276

93 O 32.076232 59.760817 48.947224 16.974011 31.624063 25.901755 277 278
279

94 H 18.669550 59.227485 43.254087 9.879500 31.341835 22.889077 280 281
282

95 H 16.202519 58.850239 45.514535 8.574004 31.142205 24.085255 283 284
285

96 H 16.775337 56.459509 43.214338 8.877126 29.877085 22.868043 286 287
288

Number of elements of the density matrix on this node (used, total): 222269 1773786

Numerical Integration : Fuzzy Cells (Becke)

Becke grid quality: NORMAL

Lebedev angular grid order: min: 11 max: 23

Nr. of radial points: min: 46 max: 52

Total nr. of points: 600118

Nr. of blocks: 4689

Block length: 128

Nr. of dummy points: 74

Test of Precision of the Numerical Integration Grid

==

Integral of the Total Core Density: 85.99993132620131

Relative Error: -7.985E-07

Density Fitting (zlmFit)

ZlmFit Fit Quality: NORMAL

Max L-expansion: min: 6 max: 7

Nr. of radial interpolation points: min: 39 max: 48

S C F *** ScaSCF ***

```
Initial density matrix is taken from previous geometry

CYCLE    1
SCF Error: norm([F,P])=     0.009981971222, max([F,P])=     0.000859920893
orbitals (Q,E):
---------------
A :114...133       ( 2.00    -0.2486)  ( 2.00    -0.2478)  ( 2.00    -0.2374)  ( 2.00    -0.2355)
                   ( 2.00    -0.2270)  ( 2.00    -0.2216)  ( 2.00    -0.2202)  ( 2.00    -0.2156)
                   ( 2.00    -0.1948)  ( 2.00    -0.1767)  ( 0.00    -0.0697)  ( 0.00    -0.0687)
                   ( 0.00    -0.0629)  ( 0.00    -0.0394)  ( 0.00    -0.0188)  ( 0.00    -0.0129)
                   ( 0.00    -0.0112)  ( 0.00    -0.0070)  ( 0.00    -0.0046)  ( 0.00    -0.0001)

CYCLE    2
 SDIIS (wt  0.000):  0.0352  0.9648
A-DIIS (wt  1.000):  0.0000  1.0000
 DIIS coefficients:  0.0000  1.0000
SCF Error: norm([F,P])=     0.001687250313, max([F,P])=     0.000138683702
orbitals (Q,E):
---------------
A :114...124       ( 2.00    -0.2484)  ( 2.00    -0.2476)  ( 2.00    -0.2371)  ( 2.00    -0.2352)
                   ( 2.00    -0.2269)  ( 2.00    -0.2214)  ( 2.00    -0.2200)  ( 2.00    -0.2154)
                   ( 2.00    -0.1945)  ( 2.00    -0.1766)  ( 0.00    -0.0695)

CYCLE    3
 SDIIS (wt  0.000):  0.0041  0.6630  0.3328
A-DIIS (wt  1.000):  0.0000  0.7231  0.2769
 DIIS coefficients:  0.0000  0.7231  0.2769
SCF Error: norm([F,P])=     0.003182821159, max([F,P])=     0.000347312011
orbitals (Q,E):
---------------
A :114...124       ( 2.00    -0.2485)  ( 2.00    -0.2476)  ( 2.00    -0.2372)  ( 2.00    -0.2353)
```

(2.00 -0.2269) (2.00 -0.2214) (2.00 -0.2201) (2.00 -0.2154)

(2.00 -0.1946) (2.00 -0.1766) (0.00 -0.0695)

CYCLE 4

SDIIS (wt 1.000): 0.3681 0.1575 0.4744

DIIS coefficients: 0.3681 0.1575 0.4744

SCF Error: norm([F,P])= 0.000697456887, max([F,P])= 0.000062359381

orbitals (Q,E):

A :114...124 (2.00 -0.2485) (2.00 -0.2476) (2.00 -0.2372) (2.00 -0.2353)

(2.00 -0.2269) (2.00 -0.2215) (2.00 -0.2201) (2.00 -0.2154)

(2.00 -0.1946) (2.00 -0.1766) (0.00 -0.0695)

CYCLE 5

SDIIS (wt 1.000): 0.1689 0.0613 0.3514 0.4184

DIIS coefficients: 0.1689 0.0613 0.3514 0.4184

SCF Error: norm([F,P])= 0.000387343343, max([F,P])= 0.000055739276

orbitals (Q,E):

A :114...124 (2.00 -0.2485) (2.00 -0.2476) (2.00 -0.2372) (2.00 -0.2353)

(2.00 -0.2269) (2.00 -0.2214) (2.00 -0.2201) (2.00 -0.2154)

(2.00 -0.1946) (2.00 -0.1766) (0.00 -0.0695)

CYCLE 6

SDIIS (wt 1.000): 0.0729 0.0234 0.1813 0.2754 0.4471

DIIS coefficients: 0.0729 0.0234 0.1813 0.2754 0.4471

SCF Error: norm([F,P])= 0.000110565416, max([F,P])= 0.000015585273

orbitals (Q,E):

A :114...124 (2.00 -0.2485) (2.00 -0.2476) (2.00 -0.2372) (2.00 -0.2353)

(2.00 -0.2269) (2.00 -0.2214) (2.00 -0.2201) (2.00 -0.2154)

(2.00   -0.1946) (2.00   -0.1766) (0.00   -0.0695)

CYCLE   7

SDIIS (wt 1.000): 0.0182 0.0044 0.0589 0.1145 0.3189 0.4851

DIIS coefficients: 0.0182 0.0044 0.0589 0.1145 0.3189 0.4851

SCF Error: norm([F,P])=   0.000047039470, max([F,P])=   0.000006573088

orbitals (Q,E):

A :114...124   (2.00   -0.2485) (2.00   -0.2476) (2.00   -0.2372) (2.00   -0.2353)

(2.00   -0.2269) (2.00   -0.2215) (2.00   -0.2201) (2.00   -0.2154)

(2.00   -0.1946) (2.00   -0.1766) (0.00   -0.0695)

CYCLE   8

SDIIS (wt 1.000): 0.0056 0.0008 0.0239 0.0547 0.1858 0.3571 0.3720

DIIS coefficients: 0.0056 0.0008 0.0239 0.0547 0.1858 0.3571 0.3720

SCF Error: norm([F,P])=   0.000020663169, max([F,P])=   0.000002665666

orbitals (Q,E):

A :114...124   (2.00   -0.2485) (2.00   -0.2476) (2.00   -0.2372) (2.00   -0.2353)

(2.00   -0.2269) (2.00   -0.2215) (2.00   -0.2201) (2.00   -0.2154)

(2.00   -0.1946) (2.00   -0.1766) (0.00   -0.0695)

CYCLE   9

SDIIS (wt 1.000): 0.0008 -0.0001 0.0065 0.0181 0.0731 0.1607 0.2425 0.4984

DIIS coefficients: 0.0008 -0.0001 0.0065 0.0181 0.0731 0.1607 0.2425 0.4984

SCF Error: norm([F,P])=   0.000005540462, max([F,P])=   0.000000650160

orbitals (Q,E):

A :114...124   (2.00   -0.2485) (2.00   -0.2476) (2.00   -0.2372) (2.00   -0.2353)

(2.00   -0.2269) (2.00   -0.2215) (2.00   -0.2201) (2.00   -0.2154)

(2.00   -0.1946) (2.00   -0.1766) (0.00   -0.0695)

SCF CONVERGED

CYCLE 10

*** Setting up for NEW gradients in Focky

*** Using FIT density in Focky

*** Setting up for NEW Hessian in Focky

orbitals (Q,E):

```
A :114...133      ( 2.00   -0.2485)  ( 2.00   -0.2476)  ( 2.00   -0.2372)  ( 2.00   -0.2353)
                  ( 2.00   -0.2269)  ( 2.00   -0.2215)  ( 2.00   -0.2201)  ( 2.00   -0.2154)
                  ( 2.00   -0.1946)  ( 2.00   -0.1766)  ( 0.00   -0.0695)  ( 0.00   -0.0686)
                  ( 0.00   -0.0628)  ( 0.00   -0.0392)  ( 0.00   -0.0187)  ( 0.00   -0.0128)
                  ( 0.00   -0.0110)  ( 0.00   -0.0069)  ( 0.00   -0.0045)  ( 0.00   -0.0000)
```

Fit test: (difference of exact and fit density, squared integrated, result summed over spins)

SCF: 0.00100982851780

Gross Charges per Atom (Z minus electrons)

==

```
-0.6656   0.9222   0.5465   0.3312  -0.5809  -0.3818  -0.6307   0.6735   0.6285   0.8636
-0.6599  -0.0010  -0.5769   0.5858   0.3252  -0.5901   0.5977   0.6019  -0.6031   0.2586
 0.3380   0.0982   0.1659   0.7476   0.4173   0.4096   0.5605   0.5716   0.0191   0.6427
-0.5655   0.5500   0.5519   0.5732   0.6409   0.3728   0.7511   0.9860   0.5877  -0.2126
-0.1566  -0.2085  -0.2048  -0.2136  -0.2447  -0.2318  -0.1513  -0.1882  -0.1343  -0.2055
-0.2417  -0.2208  -0.2242  -0.1851  -0.2463  -0.1710  -0.1959  -0.1551  -0.1982  -0.2140
-0.2243  -0.2199  -0.2280  -0.1924  -0.1145  -0.2225  -0.1621  -0.2167   0.8254  -0.1968
-0.2182  -0.2178  -0.2105  -0.1902  -0.2131  -0.1802  -0.2268  -0.2350  -0.1974  -0.2408
-0.2136  -0.2504  -0.1998  -0.1587  -0.1673   0.2326  -0.6746  -0.1253  -0.1957  -0.2138
-0.0734   0.5115  -0.6197  -0.1742  -0.2167  -0.2385
```

Net Total: 0.00000000

M U L T I P O L E   D E R I V E D   C H A R G E   A N A L Y S I S

See: M. Swart, P.Th. van Duijnen, J.G. Snijders, J.Comput.Chem., (2001), p. 79-88.

Multipole derived atomic charges (a.u.)

Atom		Level: MDC-m	MDC-d	MDC-q
1	O	-0.351202	-0.318937	-0.343667
2	C	0.362383	0.374299	0.395953
3	C	0.242233	0.250118	0.253355
4	C	0.180703	0.166739	0.212811
5	O	-0.306983	-0.548080	-0.609024
6	N	-0.214949	-0.280177	-0.475290
7	O	-0.380029	-0.386031	-0.450762
8	C	0.250709	0.309626	0.308431
9	C	0.228082	0.193461	0.187589
10	C	0.339167	0.409066	0.415940
11	O	-0.382794	-0.354486	-0.394825
12	C	0.124236	0.102364	0.101978
13	O	-0.312168	-0.298403	-0.342923
14	C	0.245412	0.242013	0.247619
15	C	0.196168	0.159174	0.174613

16	O	-0.369428	-0.477723	-0.483036
17	C	0.227992	0.193255	0.199046
18	C	0.229585	0.193142	0.192548
19	O	-0.319384	-0.320804	-0.373519
20	C	0.056812	0.044823	-0.060428
21	C	0.195047	0.220726	0.283722
22	C	0.118708	0.104784	0.107476
23	C	0.119711	0.099398	0.110717
24	C	0.290666	0.268667	0.286483
25	C	0.173007	0.135238	0.137495
26	C	0.164890	0.124936	0.164550
27	C	0.249148	0.239454	0.263262
28	C	0.234544	0.193940	0.303723
29	C	0.034980	0.028270	-0.043038
30	C	0.237055	0.191349	0.177373
31	O	-0.372315	-0.476802	-0.517817
32	C	0.254504	0.243389	0.263507
33	C	0.222617	0.185994	0.199147
34	C	0.228060	0.295672	0.386933
35	C	0.221719	0.192155	0.201515
36	C	0.180483	0.145658	0.151658
37	C	0.293815	0.271404	0.310761
38	C	0.368732	0.339916	0.387940
39	C	0.229699	0.190448	0.156321
40	H	-0.073289	-0.060629	-0.026855
41	H	-0.056771	-0.031497	-0.019607
42	H	-0.063986	-0.056104	-0.034454
43	H	-0.057109	-0.036634	-0.019665
44	H	-0.069002	-0.052720	-0.050834
45	H	-0.070407	-0.057557	-0.052966
46	H	-0.076733	-0.072073	-0.070446

47	H	-0.071456	-0.061509	-0.062496
48	H	-0.063700	-0.041760	-0.023360
49	H	-0.083038	-0.063517	-0.071147
50	H	-0.055273	-0.047008	-0.049619
51	H	-0.072797	-0.055559	-0.060316
52	H	-0.070704	-0.058579	-0.064983
53	H	-0.067637	-0.063591	-0.073602
54	H	-0.058019	-0.045413	-0.052201
55	H	-0.054166	-0.044104	-0.046449
56	H	-0.057174	-0.042199	-0.030960
57	H	-0.067339	-0.050537	-0.052543
58	H	-0.061822	-0.036820	-0.032763
59	H	-0.075303	-0.060469	-0.049813
60	H	-0.074691	-0.065321	-0.055617
61	H	-0.068778	-0.054883	-0.042938
62	H	-0.067049	-0.048711	-0.039560
63	H	-0.072458	-0.074559	-0.082054
64	H	-0.058364	-0.036374	-0.033439
65	H	-0.074901	-0.049608	-0.058536
66	H	-0.072186	-0.063641	-0.060618
67	H	-0.067313	-0.053825	-0.066984
68	H	-0.064917	-0.056072	-0.055580
69	C	0.353050	0.438124	0.460716
70	H	-0.064712	-0.054313	-0.082585
71	H	-0.068083	-0.067057	-0.097466
72	H	-0.063237	-0.048468	-0.074374
73	H	-0.080881	-0.062394	-0.073079
74	H	-0.072847	-0.059951	-0.063482
75	H	-0.069572	-0.068853	-0.073350
76	H	-0.076017	-0.045943	-0.040099
77	H	-0.066525	-0.054398	-0.038387

78	H	-0.065117	-0.060768	-0.028782
79	H	-0.082619	-0.063656	-0.046111
80	H	-0.066881	-0.058245	-0.051651
81	H	-0.067914	-0.066568	-0.055429
82	H	-0.071281	-0.044301	-0.037762
83	H	-0.061877	-0.026392	-0.019140
84	H	-0.047187	-0.002410	-0.015770
85	H	-0.073569	-0.059849	-0.041089
86	H	0.062000	0.261882	0.290730
87	O	-0.364515	-0.442751	-0.458203
88	H	-0.045070	0.000499	0.021173
89	H	-0.045139	-0.029575	-0.015338
90	H	-0.051992	-0.049947	-0.038372
91	H	-0.042726	-0.010633	-0.009928
92	C	0.241065	0.197785	0.180088
93	O	-0.362980	-0.462071	-0.489882
94	H	-0.061310	-0.053771	-0.049822
95	H	-0.060948	-0.047334	-0.038867
96	H	-0.068343	-0.065405	-0.061473

*** Using NEW gradient routines ***

Geometry CYCLE 25

==============

Energy gradients wrt nuclear displacements

==

Atom Cartesian (a.u./angstrom)

X Y Z

1	O	-0.000038	0.000134	0.000025
2	C	-0.000015	-0.000010	-0.000018
3	C	0.000043	-0.000047	0.000032
4	C	-0.000008	-0.000021	0.000017
5	O	0.000084	0.000124	-0.000041
6	N	-0.000010	-0.000003	0.000013
7	O	-0.000016	-0.000049	-0.000060
8	C	0.000035	0.000050	-0.000040
9	C	-0.000012	-0.000015	0.000012
10	C	-0.000005	0.000024	-0.000083
11	O	0.000030	0.000043	0.000004
12	C	-0.000031	0.000040	-0.000069
13	O	-0.000104	0.000019	0.000071
14	C	-0.000022	-0.000030	-0.000017
15	C	-0.000067	0.000052	-0.000038
16	O	-0.000032	-0.000028	0.000074
17	C	-0.000023	0.000066	0.000032
18	C	-0.000034	0.000030	-0.000066
19	O	0.000086	0.000031	-0.000037
20	C	0.000118	0.000011	-0.000099
21	C	-0.000027	-0.000018	0.000061
22	C	0.000014	-0.000038	-0.000008
23	C	0.000007	0.000062	0.000006
24	C	0.000026	0.000019	-0.000070
25	C	0.000010	-0.000045	0.000061
26	C	-0.000002	-0.000001	0.000006
27	C	0.000021	-0.000031	0.000002
28	C	-0.000023	0.000011	0.000000
29	C	0.000061	-0.000087	-0.000001
30	C	0.000007	-0.000006	-0.000016
31	O	0.000006	0.000038	-0.000004

32	C	0.000055	-0.000094	-0.000034
33	C	-0.000125	-0.000001	0.000099
34	C	-0.000022	0.000046	-0.000141
35	C	0.000075	-0.000011	0.000073
36	C	-0.000006	0.000040	-0.000007
37	C	-0.000010	-0.000047	0.000018
38	C	-0.000089	-0.000043	-0.000041
39	C	-0.000058	-0.000043	-0.000030
40	H	-0.000033	-0.000011	0.000102
41	H	-0.000001	-0.000046	0.000003
42	H	-0.000014	-0.000048	-0.000013
43	H	-0.000047	0.000020	-0.000006
44	H	-0.000010	-0.000008	-0.000010
45	H	0.000006	-0.000006	0.000016
46	H	0.000018	-0.000009	-0.000001
47	H	0.000016	-0.000026	-0.000004
48	H	-0.000008	0.000010	-0.000003
49	H	-0.000012	0.000019	-0.000005
50	H	0.000008	-0.000006	-0.000002
51	H	0.000033	-0.000000	-0.000022
52	H	0.000013	-0.000011	0.000005
53	H	-0.000022	-0.000027	-0.000008
54	H	-0.000003	-0.000015	-0.000032
55	H	0.000019	0.000006	0.000021
56	H	-0.000039	0.000007	0.000031
57	H	0.000031	0.000002	0.000043
58	H	-0.000037	-0.000019	-0.000023
59	H	0.000005	0.000022	-0.000028
60	H	0.000025	-0.000022	0.000007
61	H	-0.000014	0.000013	0.000015
62	H	-0.000009	0.000014	-0.000004

63	H	0.000016	-0.000000	-0.000003
64	H	-0.000015	-0.000001	0.000014
65	H	0.000015	0.000015	0.000060
66	H	-0.000004	0.000003	-0.000021
67	H	0.000015	-0.000023	-0.000018
68	H	0.000010	-0.000006	-0.000025
69	C	-0.000217	0.000003	-0.000026
70	H	-0.000006	-0.000008	0.000007
71	H	-0.000001	-0.000008	-0.000013
72	H	0.000022	-0.000006	0.000004
73	H	0.000004	0.000004	-0.000006
74	H	-0.000014	-0.000014	-0.000009
75	H	0.000013	0.000000	-0.000014
76	H	0.000002	0.000015	0.000031
77	H	0.000001	-0.000008	0.000014
78	H	-0.000005	-0.000011	0.000002
79	H	0.000000	0.000014	0.000017
80	H	0.000012	0.000013	-0.000011
81	H	-0.000007	-0.000002	-0.000008
82	H	0.000010	0.000050	-0.000019
83	H	0.000025	0.000009	0.000012
84	H	-0.000052	0.000009	0.000007
85	H	-0.000001	0.000009	-0.000017
86	H	0.000034	0.000056	0.000079
87	O	0.000144	-0.000068	0.000051
88	H	-0.000017	-0.000011	-0.000035
89	H	-0.000049	0.000005	0.000013
90	H	0.000027	-0.000001	-0.000005
91	H	-0.000021	-0.000011	0.000046
92	C	0.000096	-0.000010	0.000016
93	O	0.000144	-0.000031	0.000062

94 H -0.000003 -0.000010 0.000020

95 H 0.000006 -0.000013 -0.000010

96 H -0.000006 0.000008 0.000013

----

dE(predicted): -0.27861E-05, dE(actual): 0.46495E-05, Trust radius: 0.195959, all in a.u.

Interpolation step length 1.160307778023889E-003

Geometry Convergence after Step 25 ** CONVERGED **

----

current energy -19.67529166 Hartree

energy change 0.00000465 0.00100000 T

constrained gradient max 0.00021703 0.00100000 T

constrained gradient rms 0.00004076 0.00066667 T

gradient max 0.00021703

gradient rms 0.00004076

cart. step max 0.00939447 0.01000000 T

cart. step rms 0.00259084 0.00666667 T

Number of elements of the density matrix on this node (used, total): 222269 1773786

Numerical Integration : Fuzzy Cells (Becke)

Becke grid quality: NORMAL

Lebedev angular grid order: min: 11 max: 23

Nr. of radial points: min: 46 max: 52

Total nr. of points: 600118

Nr. of blocks: 4689

Block length: 128

Nr. of dummy points: 74

Test of Precision of the Numerical Integration Grid

==

Integral of the Total Core Density: 85.99993132620131

Relative Error: -7.985E-07

Density Fitting (zlmFit)

ZlmFit Fit Quality: NORMAL

Max L-expansion: min: 6 max: 7

Nr. of radial interpolation points: min: 39 max: 48

S C F *** ScaSCF ***

Initial density matrix is taken from previous geometry

CYCLE 1

SCF Error: norm([F,P])= 0.000001018742, max([F,P])= 0.000000280839

orbitals (Q,E):

A :114...133 (2.00 -0.2485) (2.00 -0.2476) (2.00 -0.2372) (2.00 -0.2353)

(2.00 -0.2269) (2.00 -0.2215) (2.00 -0.2201) (2.00 -0.2154)

(2.00 -0.1946) (2.00 -0.1766) (0.00 -0.0695) (0.00 -0.0686)

(0.00 -0.0628) (0.00 -0.0392) (0.00 -0.0187) (0.00 -0.0128)

(0.00 -0.0110) (0.00 -0.0069) (0.00 -0.0045) (0.00 -0.0000)

CYCLE 2

SDIIS (wt 0.000): 0.7495 0.2505

A-DIIS (wt 1.000): 0.0000 1.0000

DIIS coefficients: 0.0000 1.0000

SCF Error: norm([F,P])= 0.000003003831, max([F,P])= 0.000000834005

orbitals (Q,E):

A :114...124 (2.00 -0.2485) (2.00 -0.2476) (2.00 -0.2372) (2.00 -0.2353)

(2.00 -0.2269) (2.00 -0.2215) (2.00 -0.2201) (2.00 -0.2154)

(2.00 -0.1946) (2.00 -0.1766) (0.00 -0.0695)

SCF CONVERGED

CYCLE 3

1

****
****

****

* R E S U L T S *

****

*** Setting up for NEW gradients in Focky

*** Using FIT density in Focky

*** Setting up for NEW Hessian in Focky

Orbital Energies, per Irrep and Spin:

=====================================

	Occup	E (au)	E (eV)	Diff (eV) with prev. cycle
	-----	--------------------	------	--------------------------
A				
114	2.000	-0.24846074238363E+00	-6.761	7.96E-07
115	2.000	-0.24763434844781E+00	-6.738	2.90E-06
116	2.000	-0.23724018108047E+00	-6.456	3.07E-06
117	2.000	-0.23530957852514E+00	-6.403	3.28E-06
118	2.000	-0.22689330625408E+00	-6.174	-2.15E-07
119	2.000	-0.22145010580557E+00	-6.026	1.47E-06
120	2.000	-0.22008417404069E+00	-5.989	2.22E-06
121	2.000	-0.21542780374130E+00	-5.862	-8.49E-07
122	2.000	-0.19463567534156E+00	-5.296	-6.56E-06
123	2.000	-0.17662912161557E+00	-4.806	9.13E-08
124	0.000	-0.69530653162084E-01	-1.892	
125	0.000	-0.68553988381685E-01	-1.865	
126	0.000	-0.62778000265323E-01	-1.708	
127	0.000	-0.39181550097696E-01	-1.066	
128	0.000	-0.18677075437214E-01	-0.508	
129	0.000	-0.12831793637008E-01	-0.349	

130	0.000	-0.11048587504148E-01	-0.301
131	0.000	-0.68751972180050E-02	-0.187
132	0.000	-0.44637841194654E-02	-0.121
133	0.000	-0.47881598838889E-04	-0.001

HOMO : 123 A -0.17662912161557E+00

LUMO : 124 A -0.69530653162084E-01

Orbital Energies of the Core Orbitals:

=====================================

(Note that the atoms are grouped by atomtype, see the labels, and may hence NOT be in input order)

AtomType	Orbital	Atom	E (au)	E (eV)
--------	-------	----	--------------------	----------------
O	1S	1	-0.18805497401246E+02	-511.724
		5	-0.18778475463332E+02	-510.988
		7	-0.18799729299246E+02	-511.567
		11	-0.18797369524127E+02	-511.502
		13	-0.18847826732247E+02	-512.875
		16	-0.18785247453381E+02	-511.173
		19	-0.18831873707460E+02	-512.441
		31	-0.18765429286072E+02	-510.633
		87	-0.18796138840321E+02	-511.469
		93	-0.18777216320385E+02	-510.954
C	1S	2	-0.10033931615639E+02	-273.037
		3	-0.99862615906967E+01	-271.740
		4	-0.99687488436508E+01	-271.263
		8	-0.10025612811320E+02	-272.811

		9	-0.99037626629450E+01	-269.495
		10	-0.10071871563681E+02	-274.070
		12	-0.99658618879381E+01	-271.185
		14	-0.99878745611939E+01	-271.784
		15	-0.99935129260193E+01	-271.937
		17	-0.99219100695681E+01	-269.989
		18	-0.99238133905015E+01	-270.041
		20	-0.99289805260687E+01	-270.181
		21	-0.10003144179586E+02	-272.199
		22	-0.99458361710751E+01	-270.640
		23	-0.99295514767850E+01	-270.197
		24	-0.99394487849953E+01	-270.466
		25	-0.99212297513658E+01	-269.970
		26	-0.99309124061420E+01	-270.234
		27	-0.99889559354888E+01	-271.813
		28	-0.99232342090629E+01	-270.025
		29	-0.99374435458548E+01	-270.412
		30	-0.99272254019142E+01	-270.134
		32	-0.10005426034497E+02	-272.261
		33	-0.99043141626314E+01	-269.510
		34	-0.99978848759623E+01	-272.056
		35	-0.99119146289470E+01	-269.717
		36	-0.99379375846857E+01	-270.425
		37	-0.99400385816282E+01	-270.482
		38	-0.99648831980980E+01	-271.158
		39	-0.99274066927020E+01	-270.138
		69	-0.10053129112211E+02	-273.560
		92	-0.99336469418280E+01	-270.308
N	1S	6	-0.14018563924975E+02	-381.465

Fit test: (difference of exact and fit density, squared integrated, result summed over spins)

Sum-of-Fragments: 0.00000000006389

Orthogonalized Fragments: 0.00037481790745

SCF: 0.00100982851934

Electron Density at Nuclei

The electron density is calculated at points on a small sphere around the center of a nucleus.

The printed electron density is the average electron density on these points.

The radius of the sphere is the printed approximate finite nuclear radius.

	Atom	Nuclear Radius (Angstrom)	Electron Density (a.u.)
	----	-------------------------	-----------------------
1)	O	0.0000345546	306.98773
2)	C	0.0000320678	124.12617
3)	C	0.0000320678	124.19152
4)	C	0.0000320678	124.11030
5)	O	0.0000345546	306.99810
6)	N	0.0000333706	201.57567
7)	O	0.0000345546	307.16166
8)	C	0.0000320678	124.42787
9)	C	0.0000320678	124.09919
10)	C	0.0000320678	124.31891
11)	O	0.0000345546	307.02382
12)	C	0.0000320678	124.15132
13)	O	0.0000345546	307.16322
14)	C	0.0000320678	124.17978

15)	C	0.0000320678	124.13929
16)	O	0.0000345546	307.31348
17)	C	0.0000320678	124.11139
18)	C	0.0000320678	124.13728
19)	O	0.0000345546	307.15786
20)	C	0.0000320678	124.32460
21)	C	0.0000320678	124.09390
22)	C	0.0000320678	124.14743
23)	C	0.0000320678	124.12184
24)	C	0.0000320678	124.08861
25)	C	0.0000320678	124.14984
26)	C	0.0000320678	124.15346
27)	C	0.0000320678	124.11374
28)	C	0.0000320678	124.11181
29)	C	0.0000320678	124.31755
30)	C	0.0000320678	124.09548
31)	O	0.0000345546	307.33898
32)	C	0.0000320678	124.22643
33)	C	0.0000320678	124.08700
34)	C	0.0000320678	124.41523
35)	C	0.0000320678	124.12516
36)	C	0.0000320678	124.08997
37)	C	0.0000320678	124.10029
38)	C	0.0000320678	124.09735
39)	C	0.0000320678	124.08578
40)	H	0.0000181514	0.51356
41)	H	0.0000181514	0.50324
42)	H	0.0000181514	0.51082
43)	H	0.0000181514	0.50428
44)	H	0.0000181514	0.51053
45)	H	0.0000181514	0.50967

46)	H	0.0000181514	0.51347
47)	H	0.0000181514	0.51537
48)	H	0.0000181514	0.51370
49)	H	0.0000181514	0.52009
50)	H	0.0000181514	0.50848
51)	H	0.0000181514	0.51264
52)	H	0.0000181514	0.51197
53)	H	0.0000181514	0.51598
54)	H	0.0000181514	0.51416
55)	H	0.0000181514	0.51645
56)	H	0.0000181514	0.52098
57)	H	0.0000181514	0.51267
58)	H	0.0000181514	0.51438
59)	H	0.0000181514	0.51342
60)	H	0.0000181514	0.51075
61)	H	0.0000181514	0.51011
62)	H	0.0000181514	0.51134
63)	H	0.0000181514	0.51021
64)	H	0.0000181514	0.51029
65)	H	0.0000181514	0.51954
66)	H	0.0000181514	0.51078
67)	H	0.0000181514	0.51183
68)	H	0.0000181514	0.50967
69)	C	0.0000320678	124.32197
70)	H	0.0000181514	0.50987
71)	H	0.0000181514	0.50880
72)	H	0.0000181514	0.50937
73)	H	0.0000181514	0.51736
74)	H	0.0000181514	0.51366
75)	H	0.0000181514	0.51034
76)	H	0.0000181514	0.51126

77)	H	0.0000181514	0.51522
78)	H	0.0000181514	0.51269
79)	H	0.0000181514	0.51319
80)	H	0.0000181514	0.51405
81)	H	0.0000181514	0.51387
82)	H	0.0000181514	0.51967
83)	H	0.0000181514	0.51684
84)	H	0.0000181514	0.51257
85)	H	0.0000181514	0.51968
86)	H	0.0000181514	0.46597
87)	O	0.0000345546	307.23938
88)	H	0.0000181514	0.50520
89)	H	0.0000181514	0.50618
90)	H	0.0000181514	0.50672
91)	H	0.0000181514	0.49914
92)	C	0.0000320678	124.11059
93)	O	0.0000345546	307.29497
94)	H	0.0000181514	0.51266
95)	H	0.0000181514	0.50931
96)	H	0.0000181514	0.51088

M U L L I K E N   P O P U L A T I O N S

The survey below gives for each atom:

a) the total charge (Z minus electrons)

b) the net spin polarization (nr of electrons spin-A minus spin-B)

c) for each spin the atomic electron valence density (integrated) per L-value.

Atom	Charge	Spin density	S	P	D	F
----	------	------------	------	------	------	------
1 O	-0.6656		1.8667	4.7177	0.0738	0.0074
2 C	0.9222		0.9623	1.9775	0.1296	0.0084
3 C	0.5465		1.1320	2.2260	0.0896	0.0059
4 C	0.3312		1.1761	2.3962	0.0911	0.0054
5 O	-0.5809		1.8363	4.6901	0.0497	0.0048
6 N	-0.3818		1.5402	3.7575	0.0783	0.0057
7 O	-0.6307		1.8713	4.6846	0.0683	0.0066
8 C	0.6735		1.1484	2.0466	0.1232	0.0083
9 C	0.6285		0.9394	2.3892	0.0406	0.0024
10 C	0.8636		0.9588	2.0080	0.1589	0.0107
11 O	-0.6599		1.8714	4.7121	0.0699	0.0065
12 C	-0.0010		1.2654	2.6647	0.0668	0.0041
13 O	-0.5769		1.8610	4.6363	0.0724	0.0071
14 C	0.5858		1.1338	2.1825	0.0919	0.0059
15 C	0.3252		1.2785	2.2792	0.1101	0.0070
16 O	-0.5901		1.8747	4.6518	0.0574	0.0062
17 C	0.5977		0.9529	2.4070	0.0401	0.0023
18 C	0.6019		0.9440	2.4125	0.0393	0.0023
19 O	-0.6031		1.8633	4.6615	0.0712	0.0071
20 C	0.2586		1.1882	2.4934	0.0552	0.0046
21 C	0.3380		1.2550	2.2849	0.1151	0.0071
22 C	0.0982		1.2591	2.5690	0.0695	0.0042
23 C	0.1659		1.2274	2.5302	0.0722	0.0042
24 C	0.7476		0.8779	2.3139	0.0572	0.0034
25 C	0.4173		1.1144	2.4110	0.0541	0.0033
26 C	0.4096		1.0886	2.4438	0.0547	0.0033
27 C	0.5605		1.0974	2.2368	0.0992	0.0061
28 C	0.5716		0.9486	2.4377	0.0398	0.0024

29 C	0.0191	1.3053	2.5949	0.0750	0.0057
30 C	0.6427	0.9353	2.3797	0.0400	0.0023
31 O	-0.5655	1.8710	4.6323	0.0561	0.0061
32 C	0.5500	1.1638	2.1933	0.0874	0.0056
33 C	0.5519	0.9574	2.4489	0.0396	0.0023
34 C	0.5732	1.1189	2.1759	0.1237	0.0083
35 C	0.6409	0.9423	2.3752	0.0394	0.0023
36 C	0.3728	1.0862	2.4808	0.0569	0.0034
37 C	0.7511	0.8782	2.3094	0.0579	0.0034
38 C	0.9860	0.8096	2.1373	0.0632	0.0039
39 C	0.5877	0.9390	2.4296	0.0413	0.0024
40 H	-0.2126	1.1153	0.0908	0.0065	0.0000
41 H	-0.1566	1.0581	0.0921	0.0065	0.0000
42 H	-0.2085	1.1104	0.0915	0.0066	0.0000
43 H	-0.2048	1.1116	0.0867	0.0065	0.0000
44 H	-0.2136	1.1219	0.0852	0.0064	0.0000
45 H	-0.2447	1.1528	0.0855	0.0064	0.0000
46 H	-0.2318	1.1382	0.0870	0.0066	0.0000
47 H	-0.1513	1.0605	0.0844	0.0065	0.0000
48 H	-0.1882	1.1006	0.0812	0.0064	0.0000
49 H	-0.1343	1.0323	0.0950	0.0070	0.0000
50 H	-0.2055	1.1087	0.0901	0.0067	0.0000
51 H	-0.2417	1.1460	0.0890	0.0067	0.0000
52 H	-0.2208	1.1252	0.0891	0.0066	0.0000
53 H	-0.2242	1.1336	0.0840	0.0066	0.0000
54 H	-0.1851	1.0894	0.0890	0.0067	0.0000
55 H	-0.2463	1.1550	0.0848	0.0066	0.0000
56 H	-0.1710	1.0900	0.0746	0.0064	0.0000
57 H	-0.1959	1.1071	0.0824	0.0064	0.0000
58 H	-0.1551	1.0655	0.0832	0.0064	0.0000
59 H	-0.1982	1.1071	0.0846	0.0064	0.0000

60 H	-0.2140	1.1251	0.0826	0.0063	0.0000
61 H	-0.2243	1.1343	0.0838	0.0063	0.0000
62 H	-0.2199	1.1271	0.0863	0.0065	0.0000
63 H	-0.2280	1.1383	0.0834	0.0064	0.0000
64 H	-0.1924	1.0999	0.0859	0.0065	0.0000
65 H	-0.1145	1.0214	0.0865	0.0065	0.0000
66 H	-0.2225	1.1319	0.0842	0.0065	0.0000
67 H	-0.1621	1.0593	0.0960	0.0068	0.0000
68 H	-0.2167	1.1210	0.0892	0.0065	0.0000
69 C	0.8254	0.9481	2.0581	0.1577	0.0106
70 H	-0.1968	1.1078	0.0826	0.0064	0.0000
71 H	-0.2182	1.1294	0.0824	0.0063	0.0000
72 H	-0.2178	1.1290	0.0825	0.0063	0.0000
73 H	-0.2105	1.1310	0.0733	0.0061	0.0000
74 H	-0.1902	1.1025	0.0813	0.0064	0.0000
75 H	-0.2131	1.1278	0.0791	0.0062	0.0000
76 H	-0.1802	1.0917	0.0820	0.0064	0.0000
77 H	-0.2268	1.1349	0.0854	0.0066	0.0000
78 H	-0.2350	1.1473	0.0812	0.0064	0.0000
79 H	-0.1974	1.1096	0.0814	0.0064	0.0000
80 H	-0.2408	1.1520	0.0823	0.0065	0.0000
81 H	-0.2136	1.1275	0.0797	0.0064	0.0000
82 H	-0.2504	1.1619	0.0819	0.0066	0.0000
83 H	-0.1998	1.1111	0.0823	0.0065	0.0000
84 H	-0.1587	1.0645	0.0876	0.0067	0.0000
85 H	-0.1673	1.0864	0.0744	0.0064	0.0000
86 H	0.2326	0.6114	0.1446	0.0115	0.0000
87 O	-0.6746	1.8799	4.7313	0.0573	0.0061
88 H	-0.1253	1.0401	0.0789	0.0063	0.0000
89 H	-0.1957	1.1086	0.0808	0.0063	0.0000
90 H	-0.2138	1.1250	0.0825	0.0063	0.0000

91 H -0.0734 0.9768 0.0899 0.0067 0.0000

92 C 0.5115 0.9533 2.4965 0.0364 0.0023

93 O -0.6197 1.8761 4.6790 0.0582 0.0063

94 H -0.1742 1.0809 0.0867 0.0066 0.0000

95 H -0.2167 1.1239 0.0863 0.0065 0.0000

96 H -0.2385 1.1469 0.0852 0.0064 0.0000

Populations of individual BAS functions

----

1 O 0.0004 0.9594 0.9224 -0.0156 0.3692 0.5864 0.3355 0.8198 0.9485 0.8028
0.2620 0.3388 0.2548 0.0204 0.0088 0.0177 -0.0139 0.0102 0.0306 0.0082
0.0058 -0.0009 -0.0080 0.0004 0.0023 -0.0126 -0.0092 0.0087 0.0127

5 O 0.0004 0.9146 0.9375 -0.0162 0.4698 0.5164 0.2349 0.8805 0.9269 0.8048
0.2985 0.3172 0.2410 -0.0009 0.0067 0.0193 -0.0093 0.0078 0.0261 -0.0032
0.0012 0.0019 -0.0044 0.0007 0.0091 -0.0074 -0.0055 0.0072 0.0051

7 O 0.0004 0.9477 0.9392 -0.0160 0.5843 0.3600 0.3391 0.9442 0.8079 0.8020
0.3370 0.2588 0.2514 -0.0125 0.0099 0.0100 0.0219 0.0148 0.0242 -0.0114
-0.0077 -0.0077 0.0065 0.0003 0.0068 0.0095 0.0003 0.0005 0.0095

11 O 0.0004 0.9576 0.9291 -0.0157 0.2735 0.5250 0.5167 0.7694 0.8854 0.8896
0.2328 0.3086 0.3111 0.0352 0.0173 0.0196 -0.0039 0.0081 -0.0063 0.0096
0.0099 0.0096 -0.0033 0.0011 -0.0037 -0.0063 -0.0018 -0.0021 -0.0064

13 O 0.0004 0.9392 0.9374 -0.0160 0.4724 0.4703 0.2519 0.8952 0.8856 0.8152
0.3004 0.2957 0.2496 -0.0042 0.0093 0.0158 -0.0044 0.0162 0.0397 -0.0067
-0.0010 -0.0069 -0.0007 0.0007 0.0095 -0.0066 -0.0063 0.0098 0.0153

16 O 0.0004 0.9309 0.9600 -0.0166 0.4352 0.4123 0.4160 0.8951 0.8134 0.8295
0.3058 0.2695 0.2750 0.0113 0.0125 0.0136 0.0056 0.0079 0.0065 0.0021
0.0036 0.0036 -0.0003 0.0010 0.0007 -0.0025 -0.0007 0.0001 -0.0015

19 O 0.0004 0.9413 0.9376 -0.0160 0.5254 0.3535 0.3517 0.9033 0.8390 0.8422
0.3119 0.2658 0.2688 -0.0078 0.0093 0.0121 0.0201 0.0154 0.0222 -0.0088
-0.0065 -0.0055 0.0038 0.0007 0.0068 0.0080 -0.0008 0.0005 0.0088

31 O 0.0004 0.9220 0.9653 -0.0168 0.3767 0.4963 0.3710 0.8524 0.9226 0.7663

0.2816 0.3221 0.2433 0.0169 0.0135 0.0124 0.0002 0.0073 0.0058 0.0059
0.0060 0.0047 -0.0021 0.0011 -0.0014 -0.0051 -0.0013 0.0007 -0.0024
87 O 0.0004 0.9486 0.9472 -0.0162 0.4130 0.4097 0.5073 0.8295 0.8146 0.9029
0.2747 0.2680 0.3115 0.0142 0.0102 0.0132 0.0071 0.0110 0.0016 0.0024
0.0028 0.0039 -0.0000 0.0010 -0.0006 -0.0012 0.0013 -0.0002 -0.0035
93 O 0.0004 0.9356 0.9566 -0.0165 0.3927 0.4241 0.4607 0.7949 0.8831 0.8716
0.2565 0.2998 0.2955 0.0045 0.0119 0.0087 0.0118 0.0149 0.0064 -0.0030
-0.0010 -0.0006 0.0033 0.0011 0.0008 0.0023 0.0037 0.0011 -0.0013
2 C 0.0004 0.1910 0.8037 -0.0328 -0.3203 -0.2231 -0.2025 0.8696 0.6805 0.6513
0.1933 0.1633 0.1653 0.0195 0.0243 0.0217 0.0136 0.0327 0.0179 0.0031
0.0003 -0.0003 -0.0005 0.0006 0.0001 0.0016 -0.0005 0.0010 0.0032
3 C 0.0004 0.3871 0.7742 -0.0298 -0.2062 -0.1395 -0.2553 0.8370 0.6221 0.8426
0.1874 0.1469 0.1911 0.0146 0.0190 0.0123 0.0090 0.0196 0.0152 0.0025
0.0000 0.0001 -0.0007 0.0004 0.0001 0.0012 -0.0005 0.0006 0.0022
4 C 0.0004 0.4512 0.7528 -0.0283 -0.0920 -0.1827 -0.1660 0.6958 0.8133 0.7939
0.1690 0.1850 0.1799 0.0130 0.0182 0.0228 0.0120 0.0123 0.0127 0.0022
0.0003 0.0004 -0.0002 0.0003 -0.0004 0.0013 -0.0002 -0.0000 0.0017
8 C 0.0004 0.3969 0.7819 -0.0308 -0.2785 -0.1048 -0.1640 0.7715 0.6357 0.6667
0.2052 0.1520 0.1629 0.0411 0.0213 0.0237 0.0064 0.0194 0.0113 0.0040
0.0036 0.0039 -0.0012 0.0014 -0.0001 -0.0028 0.0003 0.0012 -0.0020
9 C 0.0004 0.1712 0.8014 -0.0337 -0.2238 -0.1521 -0.1915 0.8145 0.7570 0.8073
0.1970 0.1855 0.1954 0.0026 0.0111 0.0034 0.0110 0.0066 0.0058 -0.0004
-0.0008 -0.0009 0.0018 0.0002 -0.0009 0.0029 0.0009 -0.0012 0.0008
10 C 0.0004 0.2097 0.7810 -0.0324 -0.1563 -0.1116 -0.2353 0.6873 0.6552 0.6299
0.1839 0.1673 0.1877 0.0227 0.0177 0.0384 0.0099 0.0336 0.0365 -0.0002
0.0024 -0.0002 0.0006 0.0011 0.0034 -0.0027 -0.0017 0.0034 0.0045
12 C 0.0004 0.5496 0.7427 -0.0273 -0.0845 -0.0397 -0.0934 0.7849 0.7693 0.7899
0.1775 0.1809 0.1797 0.0065 0.0155 0.0145 0.0057 0.0174 0.0072 -0.0014
0.0009 0.0014 0.0009 0.0010 0.0013 -0.0015 0.0013 0.0016 -0.0015
14 C 0.0004 0.3892 0.7737 -0.0295 -0.1552 -0.2724 -0.1818 0.6293 0.8395 0.8028
0.1523 0.1808 0.1871 0.0097 0.0204 0.0187 0.0162 0.0172 0.0097 0.0012

-0.0010 0.0003 -0.0003 0.0006 0.0008 0.0026 0.0018 0.0003 -0.0005

15 C 0.0004 0.5586 0.7461 -0.0266 -0.1355 -0.2081 -0.1160 0.6629 0.8267 0.7482

0.1532 0.1805 0.1674 0.0128 0.0198 0.0238 0.0172 0.0225 0.0139 0.0004

-0.0011 0.0015 -0.0005 0.0010 0.0023 0.0022 0.0016 0.0007 -0.0011

17 C 0.0004 0.1912 0.7946 -0.0333 -0.1779 -0.1835 -0.1768 0.8104 0.7972 0.7612

0.2005 0.1920 0.1839 0.0044 0.0036 0.0086 0.0069 0.0089 0.0077 0.0004

-0.0014 -0.0007 -0.0007 0.0003 0.0009 0.0006 0.0009 0.0015 0.0005

18 C 0.0004 0.1745 0.8029 -0.0338 -0.1772 -0.1972 -0.1496 0.7971 0.8019 0.7604

0.1946 0.1960 0.1865 0.0062 0.0034 0.0079 0.0062 0.0073 0.0084 0.0007

-0.0007 -0.0002 -0.0011 0.0003 0.0011 0.0007 -0.0009 0.0007 0.0017

20 C 0.0004 0.4522 0.7648 -0.0292 -0.1962 -0.3071 0.1590 0.8109 0.8173 0.6593

0.2044 0.1885 0.1572 0.0160 0.0171 0.0063 0.0222 0.0087 -0.0151 0.0023

0.0008 0.0030 0.0028 0.0006 -0.0036 0.0047 0.0032 -0.0040 -0.0052

21 C 0.0004 0.5387 0.7423 -0.0264 -0.1715 -0.1516 -0.1673 0.7748 0.7825 0.7124

0.1685 0.1754 0.1617 0.0182 0.0158 0.0216 0.0220 0.0235 0.0140 0.0015

-0.0005 0.0013 -0.0002 0.0008 0.0006 0.0019 0.0011 0.0003 0.0001

22 C 0.0004 0.5508 0.7346 -0.0267 -0.1292 -0.1309 -0.0656 0.7843 0.7899 0.7806

0.1784 0.1754 0.1862 0.0118 0.0109 0.0134 0.0142 0.0140 0.0053 0.0021

-0.0005 0.0008 -0.0002 0.0005 -0.0008 0.0018 0.0018 -0.0001 -0.0012

23 C 0.0004 0.5105 0.7441 -0.0276 -0.0869 -0.1745 -0.0983 0.7846 0.8005 0.7645

0.1871 0.1801 0.1731 0.0054 0.0172 0.0116 0.0107 0.0155 0.0119 -0.0000

-0.0001 -0.0011 0.0011 0.0003 0.0001 0.0014 -0.0001 0.0001 0.0024

24 C 0.0004 0.0930 0.8198 -0.0353 -0.1882 -0.2161 -0.1723 0.7562 0.7997 0.7615

0.1845 0.1972 0.1913 0.0108 0.0067 0.0126 0.0081 0.0080 0.0110 0.0010

0.0000 0.0016 -0.0008 0.0005 0.0008 0.0007 -0.0002 0.0002 -0.0005

25 C 0.0004 0.3795 0.7644 -0.0299 -0.1650 -0.0915 -0.2621 0.7913 0.7901 0.7955

0.1840 0.1992 0.1695 0.0054 0.0086 0.0142 0.0027 0.0087 0.0145 -0.0001

0.0009 -0.0004 -0.0008 0.0003 0.0019 -0.0010 -0.0024 0.0008 0.0039

26 C 0.0004 0.3461 0.7726 -0.0305 -0.1969 -0.1728 -0.1174 0.7894 0.7882 0.7969

0.1806 0.1811 0.1947 0.0108 0.0132 0.0072 0.0107 0.0085 0.0044 0.0024

0.0005 0.0007 0.0003 0.0002 -0.0017 0.0019 0.0007 -0.0014 -0.0005

27 C 0.0004 0.3414 0.7864 -0.0309 -0.2059 -0.2366 -0.1109 0.8222 0.8449 0.5965
0.1880 0.1898 0.1486 0.0142 0.0165 0.0232 0.0161 0.0192 0.0100 0.0019
0.0000 -0.0001 0.0007 0.0003 -0.0006 0.0029 0.0002 -0.0010 0.0018
28 C 0.0004 0.1783 0.8038 -0.0339 -0.1642 -0.1408 -0.1946 0.8012 0.7508 0.8042
0.1985 0.1851 0.1974 0.0039 0.0089 0.0038 0.0127 0.0064 0.0041 0.0000
-0.0014 -0.0007 0.0014 0.0002 -0.0007 0.0038 0.0013 -0.0016 0.0000
29 C 0.0004 0.5796 0.7529 -0.0276 -0.2559 -0.1249 0.1701 0.8308 0.7934 0.6478
0.1948 0.1901 0.1486 0.0264 0.0190 0.0149 0.0226 0.0075 -0.0154 0.0049
0.0019 0.0044 0.0016 0.0006 -0.0043 0.0038 0.0029 -0.0041 -0.0060
30 C 0.0004 0.1658 0.8031 -0.0340 -0.1905 -0.1949 -0.1924 0.7880 0.8014 0.7904
0.1904 0.1959 0.1913 0.0033 0.0095 0.0118 0.0011 0.0117 0.0026 -0.0016
0.0011 0.0015 0.0006 0.0008 0.0015 -0.0022 0.0009 0.0015 -0.0018
32 C 0.0004 0.4119 0.7810 -0.0296 -0.2142 -0.2649 -0.1117 0.7678 0.7853 0.7173
0.1702 0.1705 0.1731 0.0166 0.0185 0.0143 0.0167 0.0164 0.0049 0.0023
0.0006 0.0014 0.0007 0.0007 -0.0013 0.0024 0.0017 -0.0013 -0.0016
33 C 0.0004 0.1982 0.7918 -0.0331 -0.1434 -0.2072 -0.1516 0.7520 0.8202 0.7947
0.1850 0.2012 0.1979 0.0106 0.0069 0.0101 0.0053 0.0048 0.0018 0.0025
0.0007 0.0020 -0.0014 0.0002 -0.0004 0.0006 -0.0005 -0.0004 -0.0011
34 C 0.0004 0.3683 0.7810 -0.0309 -0.1224 -0.2533 -0.0110 0.6651 0.7897 0.5769
0.1938 0.1955 0.1416 0.0277 0.0289 0.0176 0.0331 0.0191 -0.0026 0.0021
0.0016 0.0034 0.0028 0.0011 -0.0016 0.0025 0.0024 -0.0023 -0.0039
35 C 0.0004 0.1804 0.7948 -0.0334 -0.1837 -0.1873 -0.2298 0.8121 0.7630 0.8235
0.1993 0.1822 0.1957 0.0021 0.0048 0.0087 0.0117 0.0109 0.0011 -0.0007
-0.0023 0.0006 0.0004 0.0002 0.0007 0.0036 0.0023 -0.0006 -0.0019
36 C 0.0004 0.3438 0.7728 -0.0309 -0.2268 -0.1509 -0.0758 0.8169 0.7670 0.7899
0.1802 0.1778 0.2026 0.0118 0.0124 0.0116 0.0113 0.0087 0.0011 0.0030
-0.0001 0.0011 -0.0001 0.0002 -0.0017 0.0029 0.0012 -0.0016 -0.0014
37 C 0.0004 0.0899 0.8234 -0.0355 -0.2140 -0.1399 -0.2136 0.8206 0.6715 0.8122
0.2017 0.1710 0.2001 0.0054 0.0083 0.0079 0.0152 0.0199 0.0012 0.0000
-0.0019 0.0005 0.0004 0.0003 0.0002 0.0040 0.0021 -0.0009 -0.0015
38 C 0.0004 0.0056 0.8406 -0.0371 -0.1936 -0.2651 -0.2317 0.6524 0.7792 0.8265

0.1677 0.1929 0.2092 0.0092 0.0131 0.0156 0.0125 0.0052 0.0078 0.0008
0.0015 0.0008 0.0009 0.0007 -0.0004 0.0007 -0.0008 -0.0012 0.0009

39 C 0.0004 0.1691 0.8033 -0.0338 -0.1492 -0.1711 -0.1771 0.7536 0.8012 0.7927
0.1843 0.1973 0.1980 0.0124 0.0072 0.0110 0.0045 0.0040 0.0021 0.0041
0.0009 0.0015 -0.0018 0.0002 -0.0014 0.0004 -0.0006 -0.0005 -0.0004

69 C 0.0004 0.1919 0.7890 -0.0333 -0.0417 -0.2492 -0.1448 0.5950 0.7649 0.5937
0.1531 0.2089 0.1781 0.0011 0.0200 0.0288 0.0451 0.0320 0.0307 -0.0041
-0.0023 -0.0016 0.0036 0.0014 0.0031 0.0042 0.0028 0.0016 0.0019

92 C 0.0004 0.1841 0.8026 -0.0339 -0.1305 -0.1468 -0.1854 0.7993 0.7586 0.8185
0.1997 0.1834 0.1996 0.0018 0.0043 0.0076 0.0131 0.0090 0.0006 -0.0010
-0.0023 0.0006 0.0006 0.0002 0.0007 0.0039 0.0025 -0.0009 -0.0022

6 N 0.0004 0.6802 0.9011 -0.0414 0.1130 0.2068 0.4687 0.7602 0.7897 0.8665
0.1576 0.1734 0.2216 0.0250 0.0170 0.0162 0.0185 0.0108 -0.0090 0.0053
0.0017 0.0060 -0.0006 0.0006 -0.0028 0.0046 0.0024 -0.0049 -0.0068

40 H 0.2488 0.5377 0.3288 0.0336 0.0309 0.0263 0.0080 0.0018 0.0014 -0.0006
0.0005 -0.0047

41 H 0.0736 0.6703 0.3141 0.0242 0.0288 0.0391 -0.0043 0.0005 0.0015 -0.0025
0.0017 0.0095

42 H 0.2260 0.5586 0.3259 0.0259 0.0405 0.0251 -0.0063 0.0021 0.0001 0.0147
0.0021 -0.0062

43 H 0.2372 0.5504 0.3240 0.0269 0.0284 0.0314 0.0044 0.0011 0.0015 -0.0054
0.0011 0.0038

44 H 0.2373 0.5569 0.3278 0.0333 0.0249 0.0270 0.0063 0.0015 0.0014 0.0010
0.0008 -0.0046

45 H 0.2673 0.5655 0.3200 0.0263 0.0236 0.0355 -0.0074 0.0001 0.0021 -0.0060
0.0020 0.0156

46 H 0.2725 0.5387 0.3270 0.0328 0.0244 0.0299 0.0096 0.0016 0.0016 -0.0026
0.0006 -0.0042

47 H 0.1919 0.5340 0.3346 0.0313 0.0290 0.0241 0.0085 0.0017 0.0015 -0.0006
0.0005 -0.0052

48 H 0.2304 0.5414 0.3288 0.0277 0.0274 0.0261 0.0078 0.0016 0.0013 -0.0006

0.0007 -0.0045

49 H -0.0125 0.7342 0.3107 0.0271 0.0376 0.0303 -0.0055 0.0020 0.0004 0.0131
0.0019 -0.0049

50 H 0.2414 0.5390 0.3283 0.0343 0.0247 0.0311 0.0091 0.0014 0.0018 -0.0056
0.0007 -0.0008

51 H 0.2779 0.5434 0.3247 0.0350 0.0246 0.0295 0.0060 0.0012 0.0017 -0.0041
0.0009 0.0010

52 H 0.2406 0.5598 0.3247 0.0262 0.0251 0.0378 -0.0068 0.0008 0.0015 0.0021
0.0016 0.0075

53 H 0.2431 0.5678 0.3227 0.0213 0.0364 0.0264 -0.0060 0.0017 0.0003 0.0142
0.0021 -0.0056

54 H 0.1656 0.5971 0.3266 0.0225 0.0272 0.0393 -0.0042 0.0003 0.0018 -0.0070
0.0019 0.0139

55 H 0.2458 0.5936 0.3156 0.0259 0.0287 0.0301 0.0048 0.0014 0.0016 -0.0031
0.0008 0.0010

56 H 0.2150 0.5450 0.3300 0.0228 0.0228 0.0289 -0.0042 0.0004 0.0017 -0.0048
0.0016 0.0116

57 H 0.2040 0.5811 0.3220 0.0280 0.0244 0.0300 -0.0010 0.0009 0.0013 0.0002
0.0012 0.0037

58 H 0.1714 0.5609 0.3332 0.0256 0.0227 0.0348 -0.0040 0.0005 0.0016 -0.0037
0.0016 0.0105

59 H 0.1817 0.5997 0.3257 0.0241 0.0302 0.0303 0.0001 0.0009 0.0013 -0.0020
0.0013 0.0048

60 H 0.2178 0.5839 0.3235 0.0235 0.0266 0.0324 -0.0031 0.0004 0.0016 -0.0049
0.0017 0.0106

61 H 0.2373 0.5752 0.3218 0.0236 0.0352 0.0250 -0.0056 0.0019 0.0001 0.0149
0.0020 -0.0069

62 H 0.2331 0.5687 0.3254 0.0272 0.0355 0.0236 -0.0061 0.0021 0.0001 0.0153
0.0020 -0.0068

63 H 0.2457 0.5707 0.3219 0.0275 0.0273 0.0286 -0.0034 0.0006 0.0016 -0.0031
0.0016 0.0092

64 H 0.1848 0.5901 0.3251 0.0346 0.0268 0.0245 0.0145 0.0020 0.0020 -0.0057 0.0002 -0.0063

65 H 0.0003 0.7040 0.3171 0.0271 0.0287 0.0307 0.0012 0.0009 0.0014 -0.0043 0.0014 0.0060

66 H 0.2455 0.5627 0.3237 0.0290 0.0279 0.0273 -0.0003 0.0008 0.0016 -0.0045 0.0015 0.0074

67 H 0.0769 0.6641 0.3183 0.0431 0.0274 0.0255 0.0139 0.0021 0.0020 -0.0064 0.0002 -0.0050

68 H 0.2319 0.5661 0.3230 0.0264 0.0337 0.0291 -0.0050 0.0019 0.0003 0.0131 0.0019 -0.0056

70 H 0.2059 0.5740 0.3279 0.0297 0.0244 0.0285 0.0009 0.0012 0.0011 0.0025 0.0012 -0.0005

71 H 0.2331 0.5740 0.3223 0.0257 0.0228 0.0338 -0.0067 0.0002 0.0020 -0.0053 0.0019 0.0143

72 H 0.2334 0.5724 0.3232 0.0345 0.0221 0.0260 0.0138 0.0018 0.0019 -0.0053 0.0002 -0.0060

73 H 0.2681 0.5367 0.3263 0.0240 0.0279 0.0215 0.0014 0.0012 0.0012 0.0021 0.0011 -0.0009

74 H 0.2055 0.5690 0.3281 0.0274 0.0259 0.0280 0.0015 0.0013 0.0010 0.0036 0.0011 -0.0022

75 H 0.2494 0.5538 0.3246 0.0231 0.0228 0.0332 -0.0059 0.0002 0.0018 -0.0064 0.0018 0.0146

76 H 0.1707 0.5926 0.3284 0.0231 0.0257 0.0332 -0.0064 0.0004 0.0017 -0.0024 0.0018 0.0113

77 H 0.2266 0.5871 0.3212 0.0262 0.0356 0.0236 -0.0053 0.0019 0.0002 0.0149 0.0020 -0.0071

78 H 0.2498 0.5801 0.3174 0.0310 0.0256 0.0246 0.0115 0.0016 0.0018 -0.0060 0.0005 -0.0030

79 H 0.2199 0.5566 0.3331 0.0260 0.0220 0.0334 -0.0070 0.0006 0.0016 0.0008 0.0017 0.0087

80 H 0.2590 0.5726 0.3205 0.0332 0.0193 0.0297 0.0043 0.0011 0.0015 -0.0036

0.0010 0.0022

81 H 0.2252 0.5806 0.3217 0.0353 0.0178 0.0266 0.0116 0.0014 0.0018 -0.0063

0.0005 -0.0026

82 H 0.2947 0.5465 0.3207 0.0268 0.0279 0.0271 0.0017 0.0013 0.0012 0.0003

0.0015 0.0007

83 H 0.2402 0.5428 0.3281 0.0288 0.0268 0.0267 0.0018 0.0012 0.0016 -0.0030

0.0011 0.0038

84 H 0.1819 0.5527 0.3298 0.0297 0.0248 0.0331 0.0003 0.0013 0.0011 0.0012

0.0016 0.0012

85 H 0.2166 0.5368 0.3331 0.0230 0.0271 0.0243 0.0008 0.0014 0.0011 0.0023

0.0011 -0.0003

86 H -0.0008 0.2198 0.3924 0.0616 0.0459 0.0370 0.0031 0.0016 0.0027 -0.0058

0.0024 0.0074

88 H 0.1817 0.5272 0.3311 0.0263 0.0262 0.0265 -0.0011 0.0015 0.0013 0.0032

0.0014 0.0001

89 H 0.1969 0.5912 0.3204 0.0299 0.0213 0.0296 0.0053 0.0011 0.0015 -0.0045

0.0010 0.0020

90 H 0.2407 0.5608 0.3236 0.0240 0.0245 0.0340 -0.0068 0.0008 0.0013 0.0022

0.0017 0.0071

91 H -0.2152 0.9026 0.2894 0.0346 0.0255 0.0298 0.0098 0.0015 0.0019 -0.0058

0.0007 -0.0012

94 H 0.1561 0.5961 0.3287 0.0299 0.0301 0.0267 -0.0013 0.0013 0.0012 0.0030

0.0014 0.0009

95 H 0.2359 0.5656 0.3224 0.0269 0.0278 0.0316 0.0049 0.0013 0.0015 -0.0015

0.0011 -0.0008

96 H 0.2678 0.5575 0.3215 0.0256 0.0314 0.0282 -0.0025 0.0012 0.0011 0.0039

0.0015 0.0013

Gross Charges per Atom (Z minus electrons)

==

-0.6656 0.9222 0.5465 0.3312 -0.5809 -0.3818 -0.6307 0.6735 0.6285 0.8636

-0.6599 -0.0010 -0.5769 0.5858 0.3252 -0.5901 0.5977 0.6019 -0.6031 0.2586

0.3380 0.0982 0.1659 0.7476 0.4173 0.4096 0.5605 0.5716 0.0191 0.6427

-0.5655 0.5500 0.5519 0.5732 0.6409 0.3728 0.7511 0.9860 0.5877 -0.2126

-0.1566 -0.2085 -0.2048 -0.2136 -0.2447 -0.2318 -0.1513 -0.1882 -0.1343 -0.2055

-0.2417 -0.2208 -0.2242 -0.1851 -0.2463 -0.1710 -0.1959 -0.1551 -0.1982 -0.2140

-0.2243 -0.2199 -0.2280 -0.1924 -0.1145 -0.2225 -0.1621 -0.2167 0.8254 -0.1968

-0.2182 -0.2178 -0.2105 -0.1902 -0.2131 -0.1802 -0.2268 -0.2350 -0.1974 -0.2408

-0.2136 -0.2504 -0.1998 -0.1587 -0.1673 0.2326 -0.6746 -0.1253 -0.1957 -0.2138

-0.0734 0.5115 -0.6197 -0.1742 -0.2167 -0.2385

Net Total: -0.00000000

Atom-Atom Population Matrix (off-diagonal elements not doubled)

==

1 : 6.5071

2 : 0.2458 2.0727

3 : -0.0209 0.3450 2.6457

4 : 0.0032 -0.0283 0.3116 2.7068

5 : -0.0000 -0.0000 0.0000 -0.0000 6.1441

6 : -0.0012 0.0075 -0.0458 0.3500 0.0000 4.7822

7 : -0.0475 0.2703 -0.0356 -0.0123 0.0000 -0.0006 6.4413

8 : 0.0050 -0.0005 -0.0002 -0.0000 -0.0001 -0.0000 0.0004 2.5303

9 : 0.0003 0.0000 -0.0000 0.0000 -0.0001 0.0000 0.0000 0.0000 2.0333

10 : 0.0000 -0.0001 0.0000 0.0000 -0.0033 -0.0000 0.0000 -0.0321 -0.0000 2.1771

11 : 0.0069 0.0006 0.0001 0.0000 0.0000 0.0000 -0.0000 0.0283 -0.0059 -0.0001

6.4796

12 : -0.0014 -0.0005 -0.0000 0.0000 -0.0001 -0.0000 0.0000 0.2876 -0.0000 0.3487

-0.0032 2.9964

13 : -0.0000 -0.0000 -0.0000 -0.0000 0.0028 -0.0000 0.0000 0.0024 0.0000 0.3075

-0.0001 -0.0845 6.4059

14 : 0.2086 -0.0119 -0.0061 -0.0004 0.0001 -0.0002 -0.0005 -0.0319 -0.0011 -0.0023
-0.0533 0.0014 -0.0004 2.5752

15 : -0.0539 -0.0042 -0.0017 -0.0002 0.0000 -0.0000 -0.0025 0.0024 0.0066 -0.0005
0.2133 -0.0002 0.0001 0.3207 2.9193

16 : -0.0001 -0.0001 -0.0000 0.0000 -0.0000 0.0000 -0.0000 0.4878 -0.0000 0.0060
-0.0134 -0.0380 -0.0006 -0.0046 -0.0033 6.2121

17 : -0.0046 -0.0005 -0.0009 -0.0000 -0.0001 0.0000 -0.0017 -0.0300 -0.0000 -0.0015
0.0042 -0.0007 0.0001 -0.0243 0.0028 -0.0044 2.0444

18 : 0.0000 0.0000 0.0000 -0.0000 -0.0517 -0.0000 0.0000 -0.0000 0.0000 -0.0008
0.0000 -0.0001 0.0032 -0.0000 -0.0000 0.0000 0.0000 2.0777

19 : -0.0075 -0.0247 0.1846 -0.0250 0.0000 0.0001 0.0070 0.0000 0.0000 -0.0000
0.0000 0.0000 -0.0000 -0.0021 0.0002 -0.0000 -0.0043 0.0000 6.4316

20 : 0.0002 0.0000 -0.0000 0.0000 -0.0477 -0.0000 -0.0000 0.0003 -0.0002 0.0022
-0.0007 -0.0008 -0.0045 -0.0000 -0.0001 0.0000 0.0000 -0.0553 0.0000 2.8467

21 : 0.0000 0.0000 0.0000 -0.0000 0.2236 -0.0000 0.0000 -0.0001 0.0002 0.0001
0.0001 -0.0020 -0.0293 0.0001 -0.0004 0.0000 0.0000 0.3860 -0.0000 0.3224
2.8744

22 : -0.0386 -0.0032 -0.0014 0.0002 0.0001 -0.0000 0.0003 0.3056 -0.0002 0.0069
0.0021 -0.1163 -0.0022 0.3031 -0.0414 -0.0479 0.3747 -0.0000 0.0012 -0.0012
0.0000 3.0046

23 : 0.0023 -0.0003 -0.0001 0.0000 -0.0008 -0.0000 0.0000 0.0003 0.3534 0.0001
-0.0014 -0.0004 -0.0002 0.0058 -0.0307 -0.0000 0.0003 0.0002 -0.0000 0.0038
-0.0041 0.0022 2.8958

24 : 0.0001 -0.0026 -0.0020 -0.0393 -0.0000 0.3765 -0.0005 -0.0000 -0.0000 -0.0000
0.0000 -0.0000 -0.0000 -0.0001 0.0000 -0.0000 0.0000 -0.0000 -0.0005 -0.0000
-0.0000 -0.0000 0.0000 1.8820

25 : 0.0055 -0.0021 -0.0006 0.0000 0.0003 -0.0000 0.0002 -0.0002 -0.0365 0.0001
-0.0588 0.0011 0.0003 -0.0557 0.3271 -0.0004 -0.0000 -0.0001 0.0001 -0.0064
-0.0001 -0.0060 0.3352 -0.0000 2.4913

26 : -0.0018 0.0034 -0.0758 0.3167 0.0000 -0.0359 -0.0386 0.0000 -0.0000 0.0000
-0.0000 -0.0000 0.0000 0.0003 -0.0001 -0.0000 -0.0000 0.0000 0.0051 -0.0000

0.0000 -0.0000 -0.0000 -0.0010 0.0000 2.5034

27 : 0.0018 -0.0224 0.0049 -0.0248 -0.0000 0.0052 0.2357 -0.0000 0.0000 0.0000
0.0000 -0.0000 -0.0000 -0.0002 -0.0001 -0.0000 -0.0003 0.0000 0.0009 0.0000
0.0000 0.0000 0.0000 -0.0005 -0.0001 0.3580 2.4054

28 : -0.0012 -0.0058 0.0011 -0.0014 -0.0000 -0.0009 -0.0437 -0.0000 -0.0000 0.0000
0.0000 -0.0000 -0.0000 -0.0006 -0.0001 -0.0000 -0.0000 0.0000 0.0001 0.0000
0.0000 -0.0000 0.0000 -0.0001 -0.0000 -0.0288 0.3746 2.0694

29 : 0.0001 0.0000 -0.0000 -0.0000 -0.0127 0.0000 -0.0000 -0.0001 0.0059 0.0004
-0.0001 -0.0002 -0.0009 0.0001 -0.0047 0.0000 -0.0000 -0.0078 0.0001 0.5528
-0.0507 -0.0002 -0.1227 -0.0000 -0.0105 -0.0000 0.0000 0.0000 3.1882

30 : 0.0001 -0.0000 0.0000 -0.0000 0.0001 -0.0000 0.0000 -0.0325 -0.0000 -0.0471
-0.0007 0.3504 0.0064 -0.0004 0.0008 -0.0110 -0.0004 -0.0000 0.0000 0.0000
0.0002 -0.0015 0.0000 -0.0000 -0.0001 0.0000 0.0000 -0.0000 0.0001 2.0057

31 : 0.0000 0.0000 -0.0000 -0.0000 -0.0000 0.0000 0.0000 -0.0000 0.0131 0.0000
-0.0001 0.0000 -0.0000 -0.0001 -0.0008 -0.0000 -0.0000 -0.0000 -0.0000 -0.0053
-0.0016 0.0000 -0.0682 0.0000 -0.0018 0.0000 0.0000 -0.0000 -0.0524 -0.0000
6.1735

32 : 0.0000 0.0000 0.0000 0.0000 -0.0588 -0.0000 0.0000 0.0006 -0.0001 -0.0130
-0.0001 -0.0047 0.1689 -0.0001 0.0000 -0.0001 0.0001 -0.0401 -0.0000 -0.0700
0.2975 -0.0002 -0.0004 0.0000 0.0002 0.0000 0.0000 0.0000 0.0011 -0.0014
0.0000 2.6468

33 : 0.0000 0.0000 0.0000 0.0000 -0.0039 0.0000 -0.0000 0.0000 -0.0013 -0.0005
0.0001 -0.0001 -0.0001 -0.0001 0.0007 -0.0000 -0.0000 0.0006 0.0000 -0.0597
-0.0181 -0.0001 -0.0015 0.0000 0.0004 0.0000 -0.0000 -0.0000 0.3630 0.0000
0.0049 -0.0010 2.0495

34 : 0.0001 0.0000 0.0000 0.0000 -0.0011 -0.0000 -0.0000 -0.0000 -0.0453 0.0001
0.0020 0.0000 0.0003 -0.0004 0.0090 0.0000 -0.0000 -0.0025 0.0000 -0.0333
0.0047 0.0002 0.3237 0.0000 -0.0139 0.0000 -0.0000 -0.0000 0.3420 0.0000
0.4961 0.0001 -0.0477 2.5657

35 : -0.0026 0.0002 0.0001 0.0000 0.0000 0.0000 0.0008 0.0011 0.0019 -0.0000
-0.0365 0.0003 -0.0000 -0.0525 0.3754 -0.0009 -0.0007 0.0000 -0.0001 -0.0001

0.0000 0.0037 -0.0016 -0.0000 -0.0486 0.0000 -0.0000 -0.0002 0.0001 -0.0001
-0.0001 -0.0000 0.0000 -0.0002 2.0670
36 : 0.0000 0.0000 0.0000 0.0000 0.0004 -0.0000 0.0000 0.0002 0.0000 -0.0033
-0.0000 -0.0020 -0.0397 -0.0000 -0.0000 -0.0000 -0.0000 0.0020 -0.0000 -0.0004
-0.0358 -0.0000 -0.0001 -0.0000 -0.0000 0.0000 -0.0000 0.0000 -0.0012 -0.0002
-0.0000 0.3461 -0.0002 0.0000 -0.0000 2.3998
37 : -0.0000 -0.0000 0.0013 -0.0382 -0.0000 0.3921 -0.0000 0.0000 0.0000 -0.0000
0.0000 -0.0000 0.0000 -0.0000 -0.0000 -0.0000 -0.0000 -0.0000 -0.0013 -0.0000
-0.0000 0.0000 0.0000 -0.0326 0.0000 0.0007 -0.0005 -0.0001 0.0000 0.0000
-0.0000 -0.0000 -0.0000 0.0000 -0.0000 -0.0000 1.8836
38 : 0.0003 -0.0002 -0.0001 -0.0000 -0.0000 0.0000 0.0000 -0.0027 0.0002 0.0004
0.2584 0.0017 0.0000 -0.0006 -0.0200 0.0037 -0.0001 -0.0000 0.0000 0.0000
-0.0000 0.0007 -0.0008 -0.0000 -0.0005 -0.0000 -0.0000 0.0000 -0.0001 -0.0006
-0.0000 0.0000 0.0000 0.0001 0.0004 -0.0000 -0.0000 1.6877
39 : -0.0000 0.0000 -0.0000 0.0000 0.0012 -0.0000 -0.0000 -0.0000 0.0000 -0.0008
0.0000 0.0001 -0.0031 -0.0000 0.0000 -0.0000 0.0000 0.0000 0.0000 -0.0013
0.0016 -0.0000 -0.0000 0.0000 0.0000 -0.0000 0.0000 0.0000 0.0001 0.0000
-0.0000 -0.0323 -0.0000 0.0000 0.0000 0.3666 0.0000 0.0000 2.0662
40 : 0.0000 -0.0000 -0.0000 0.0000 0.0100 -0.0000 -0.0000 0.0004 -0.0003 -0.0041
0.0012 -0.0012 -0.0034 -0.0004 0.0000 0.0000 -0.0000 0.0024 0.0000 0.3906
-0.0752 -0.0009 -0.0089 -0.0000 -0.0036 0.0000 -0.0000 0.0000 -0.0595 -0.0002
0.0019 -0.0252 0.0101 -0.0303 0.0002 -0.0021 -0.0000 0.0000 -0.0011 1.0508
41 : 0.0004 -0.0001 -0.0001 -0.0000 -0.0005 0.0000 0.0000 0.0000 -0.0008 -0.0002
0.0000 -0.0000 0.0001 -0.0000 -0.0007 -0.0000 0.0000 -0.0005 -0.0014 -0.0053
-0.0009 -0.0003 -0.0047 -0.0000 0.0030 0.0000 -0.0000 0.0000 -0.0469 0.0000
-0.0017 0.0002 0.3892 -0.0101 0.0001 -0.0001 -0.0000 -0.0000 0.0000 -0.0022
0.9400
42 : -0.0000 0.0000 -0.0000 0.0000 -0.0033 0.0000 -0.0000 0.0000 0.0001 -0.0001
-0.0000 -0.0000 -0.0003 -0.0000 0.0002 0.0000 0.0000 -0.0076 0.0002 -0.0306
0.0056 0.0002 -0.0021 0.0000 0.0005 -0.0000 0.0000 -0.0000 -0.0547 -0.0000
0.0014 -0.0025 0.3770 0.0068 -0.0000 0.0003 0.0000 -0.0000 0.0000 -0.0022

-0.0627 1.0598

43 : 0.0000 0.0000 0.0000 0.0000 0.0018 -0.0000 -0.0000 0.0000 0.0003 0.0000

0.0000 -0.0000 -0.0000 -0.0000 -0.0001 -0.0000 0.0000 0.0011 0.0000 -0.0055

-0.0023 -0.0000 -0.0025 0.0000 -0.0016 0.0000 0.0000 -0.0000 -0.0389 0.0000

0.0044 -0.0002 0.3921 -0.0275 0.0000 -0.0002 -0.0000 -0.0000 -0.0000 0.0000

-0.0403 -0.0653 0.9928

44 : 0.0001 -0.0001 -0.0000 0.0000 -0.0000 -0.0000 0.0000 -0.0000 0.3937 0.0000

0.0005 0.0000 -0.0000 -0.0006 -0.0075 0.0000 -0.0000 0.0000 -0.0000 0.0003

0.0000 -0.0001 -0.0451 0.0000 -0.0216 -0.0000 0.0000 0.0000 -0.0013 -0.0000

-0.0002 -0.0000 -0.0001 -0.0122 -0.0002 -0.0000 -0.0000 -0.0002 0.0000 -0.0001

-0.0003 0.0001 -0.0002 1.0188

45 : 0.0001 0.0000 -0.0000 0.0000 -0.0000 -0.0000 0.0000 -0.0000 0.3607 0.0000

-0.0001 0.0000 0.0000 0.0000 0.0017 -0.0000 -0.0000 0.0000 0.0000 0.0000

-0.0001 0.0001 -0.0246 0.0000 0.0045 -0.0000 0.0000 -0.0000 -0.0028 -0.0000

-0.0004 -0.0000 -0.0004 -0.0161 -0.0002 0.0000 0.0000 0.0004 -0.0000 -0.0012

-0.0004 0.0000 0.0001 -0.0455 1.0620

46 : -0.0001 -0.0000 -0.0000 -0.0000 -0.0000 -0.0000 -0.0000 0.0002 0.3501 0.0000

-0.0001 -0.0001 0.0000 -0.0002 0.0088 -0.0000 0.0000 -0.0000 0.0000 -0.0008

-0.0001 0.0003 -0.0608 -0.0000 -0.0060 -0.0000 -0.0000 -0.0000 -0.0008 0.0000

0.0036 -0.0000 0.0001 0.0069 -0.0023 0.0000 -0.0000 -0.0009 -0.0000 0.0006

0.0001 -0.0001 0.0002 -0.0805 -0.0722 1.1390

47 : 0.0001 -0.0001 -0.0000 -0.0000 -0.0002 -0.0000 0.0000 -0.0000 -0.0446 0.0011

0.0001 0.0017 -0.0008 0.0018 -0.0295 -0.0001 -0.0000 -0.0004 0.0000 -0.0018

-0.0014 0.0015 0.4210 0.0000 -0.0545 0.0000 0.0000 0.0000 -0.0171 0.0006

0.0030 0.0015 -0.0013 -0.0538 -0.0028 0.0001 0.0000 0.0004 0.0001 -0.0110

0.0003 0.0004 -0.0021 0.0126 -0.0175 -0.0348 0.9883

48 : -0.0097 0.0019 0.0001 0.0000 0.0001 0.0000 -0.0003 0.0001 -0.0205 -0.0000

0.0074 0.0001 0.0000 0.0011 -0.0420 0.0001 -0.0005 -0.0000 0.0002 0.0006

0.0002 -0.0021 -0.0635 0.0000 0.4094 -0.0000 -0.0000 -0.0000 -0.0041 -0.0000

0.0008 0.0000 -0.0001 -0.0053 -0.0206 0.0000 0.0000 0.0003 -0.0000 -0.0007

-0.0003 -0.0003 -0.0001 0.0001 0.0025 -0.0080 0.0165 0.9991

49 : -0.0086 -0.0032 0.0005 -0.0001 -0.0015 -0.0001 0.0001 0.0006 0.0070 0.0006
-0.0031 -0.0008 0.0005 -0.0176 -0.0585 -0.0003 0.0010 -0.0001 -0.0011 -0.0098
-0.0020 -0.0139 -0.0586 -0.0000 0.3562 0.0000 -0.0001 -0.0000 0.0271 0.0000
-0.0047 0.0002 -0.0034 -0.0298 0.0076 -0.0001 -0.0000 -0.0008 0.0000 -0.0036
-0.0021 -0.0012 -0.0013 0.0035 -0.0069 -0.0019 -0.0176 -0.0543 1.0312
50 : 0.0006 -0.0015 -0.0000 -0.0003 -0.0000 0.0000 -0.0027 0.0008 -0.0007 0.0000
0.0113 0.0001 0.0000 -0.0093 -0.0389 -0.0000 -0.0003 -0.0000 0.0001 -0.0001
-0.0000 0.0018 -0.0013 0.0000 -0.0215 0.0003 0.0013 0.0005 -0.0000 -0.0000
-0.0000 0.0000 0.0000 0.0001 0.3750 -0.0000 -0.0000 -0.0040 0.0000 0.0000
-0.0000 0.0000 0.0000 -0.0003 -0.0004 0.0024 -0.0016 -0.0155 0.0047 1.0281
51 : -0.0007 -0.0016 -0.0002 -0.0000 -0.0000 -0.0000 -0.0010 0.0001 -0.0005 0.0000
-0.0069 -0.0000 -0.0000 -0.0238 -0.0340 -0.0008 -0.0003 0.0000 -0.0000 0.0001
0.0000 0.0009 0.0005 -0.0000 0.0080 -0.0001 -0.0001 0.0005 0.0001 -0.0000
-0.0000 -0.0000 0.0000 0.0004 0.3628 0.0000 0.0000 0.0016 0.0000 0.0001
0.0000 0.0000 0.0000 -0.0006 -0.0004 0.0028 -0.0004 0.0045 -0.0056 -0.0492
1.0883
52 : 0.0006 0.0015 0.0002 0.0000 0.0000 0.0000 0.0007 -0.0003 0.0012 -0.0000
-0.0085 -0.0002 -0.0000 0.0056 -0.0534 -0.0000 -0.0000 -0.0000 -0.0000 -0.0001
-0.0000 -0.0055 -0.0014 -0.0000 -0.0159 -0.0000 -0.0001 -0.0001 -0.0003 0.0001
-0.0002 0.0000 -0.0000 -0.0010 0.3528 -0.0000 -0.0000 -0.0034 -0.0000 -0.0001
-0.0000 -0.0000 -0.0000 0.0001 0.0007 -0.0080 0.0014 -0.0090 0.0011 -0.0586
-0.0818 1.1070
53 : 0.0002 -0.0003 0.0000 0.0000 0.0000 0.0000 0.0000 -0.0005 0.0020 -0.0001
-0.0619 0.0018 -0.0000 -0.0072 -0.0179 0.0006 0.0002 0.0000 -0.0000 0.0000
-0.0000 0.0022 -0.0020 0.0000 0.0020 0.0000 0.0000 -0.0000 -0.0001 -0.0022
-0.0000 0.0000 -0.0000 -0.0004 0.0097 -0.0000 0.0000 0.3623 0.0000 -0.0002
-0.0000 0.0000 -0.0000 0.0009 0.0008 -0.0061 0.0007 -0.0001 0.0002 0.0015
-0.0050 -0.0121 1.1722
54 : -0.0008 0.0003 -0.0000 -0.0000 0.0000 -0.0000 -0.0001 -0.0067 -0.0001 0.0001
-0.0456 -0.0006 -0.0000 0.0094 -0.0028 0.0025 -0.0004 -0.0000 0.0000 -0.0001
0.0000 -0.0008 0.0009 0.0000 -0.0038 0.0000 0.0000 -0.0000 -0.0000 0.0016

-0.0000 -0.0000 0.0000 0.0001 -0.0020 -0.0000 -0.0000 0.3842 -0.0000 0.0003
0.0000 -0.0000 0.0000 -0.0001 0.0001 -0.0019 -0.0009 0.0006 -0.0003 0.0013
-0.0118 0.0067 -0.1257 1.0781

55 : 0.0001 -0.0000 -0.0000 0.0000 -0.0000 0.0000 -0.0000 -0.0002 -0.0004 0.0000
-0.0532 -0.0027 0.0002 0.0005 -0.0032 -0.0021 -0.0001 -0.0000 0.0000 -0.0000
-0.0000 -0.0003 -0.0007 -0.0000 -0.0029 -0.0000 -0.0000 0.0000 -0.0001 0.0032
-0.0000 0.0001 -0.0000 0.0000 -0.0043 0.0000 -0.0000 0.3522 -0.0000 0.0001
-0.0000 0.0000 -0.0000 -0.0001 -0.0002 0.0018 -0.0013 0.0002 -0.0004 -0.0018
-0.0007 -0.0002 -0.0904 -0.0896 1.1413

56 : 0.0000 -0.0000 -0.0000 -0.0000 0.0086 -0.0000 0.0000 -0.0001 -0.0001 -0.0098
-0.0000 -0.0018 -0.0538 -0.0000 0.0000 -0.0000 0.0000 -0.0173 -0.0000 -0.0078
-0.0677 -0.0001 -0.0000 0.0000 0.0001 0.0000 -0.0000 -0.0000 -0.0002 -0.0008
-0.0001 0.3899 -0.0002 0.0003 -0.0000 -0.0624 0.0000 0.0000 -0.0135 -0.0135
0.0001 -0.0002 -0.0001 0.0000 -0.0000 -0.0000 0.0009 0.0000 -0.0002 0.0000
-0.0000 0.0000 0.0000 -0.0000 0.0000 1.0367

57 : 0.0000 -0.0000 0.0000 0.0000 -0.0033 -0.0000 0.0000 0.0001 -0.0000 -0.0014
-0.0000 0.0005 0.0108 0.0000 -0.0000 -0.0000 0.0000 -0.0027 0.0000 -0.0014
-0.0070 -0.0000 -0.0000 0.0000 -0.0000 0.0000 -0.0000 -0.0000 -0.0003 -0.0001
-0.0000 -0.0458 0.0000 -0.0000 -0.0000 0.3826 -0.0000 -0.0000 -0.0464 0.0004
-0.0000 -0.0001 -0.0000 -0.0000 -0.0000 0.0000 -0.0000 -0.0000 -0.0000 -0.0000
-0.0000 -0.0000 -0.0000 0.0000 -0.0000 -0.0199 1.0059

58 : 0.0000 0.0000 0.0000 -0.0000 -0.0030 -0.0000 0.0000 -0.0001 0.0000 -0.0072
-0.0000 -0.0028 -0.0100 -0.0000 -0.0000 -0.0000 -0.0000 -0.0023 -0.0000 0.0009
-0.0192 0.0000 -0.0000 -0.0000 0.0000 0.0000 0.0000 0.0000 -0.0000 -0.0003
-0.0000 -0.0512 0.0000 0.0001 -0.0000 0.4138 -0.0000 -0.0000 -0.0525 -0.0007
-0.0000 0.0003 0.0000 0.0000 0.0000 0.0000 0.0000 0.0000 -0.0000 -0.0000
0.0000 -0.0000 -0.0000 0.0000 0.0000 0.0147 -0.0359 0.9486

59 : 0.0000 -0.0000 0.0000 0.0000 0.0000 -0.0000 0.0000 -0.0000 0.0000 0.0126
-0.0000 0.0035 -0.0003 -0.0000 -0.0000 0.0000 -0.0000 0.0002 -0.0000 -0.0004
0.0008 0.0002 0.0000 -0.0000 -0.0000 0.0000 -0.0000 -0.0000 -0.0000 -0.0002
0.0000 -0.0133 -0.0000 -0.0000 -0.0000 -0.0555 -0.0000 0.0000 0.3788 -0.0003

0.0000 -0.0000 -0.0000 0.0000 0.0000 0.0000 0.0000 0.0000 -0.0000 0.0000
0.0000 -0.0000 0.0000 0.0000 -0.0000 -0.0079 0.0123 -0.0318 1.0326

60 : 0.0000 0.0000 -0.0000 -0.0000 0.0001 0.0000 -0.0000 -0.0000 -0.0000 -0.0036
0.0000 -0.0004 -0.0030 -0.0000 0.0000 -0.0000 0.0000 0.0002 0.0000 -0.0004
0.0008 -0.0000 -0.0000 0.0000 0.0000 -0.0000 0.0000 0.0000 0.0000 -0.0005
-0.0000 -0.0123 -0.0000 0.0000 0.0000 -0.0443 0.0000 0.0000 0.3829 0.0000
0.0000 0.0000 -0.0000 -0.0000 -0.0000 -0.0000 0.0000 -0.0000 0.0000 0.0000
0.0000 0.0000 -0.0000 0.0000 0.0000 0.0012 -0.0264 0.0147 -0.0724 1.0311

61 : -0.0000 0.0000 -0.0000 0.0000 0.0003 0.0000 -0.0000 -0.0000 0.0000 -0.0007
0.0000 0.0000 0.0012 -0.0000 0.0000 -0.0000 0.0000 -0.0001 0.0000 -0.0001
-0.0041 -0.0000 -0.0000 0.0000 -0.0000 -0.0000 0.0000 0.0000 -0.0001 0.0003
-0.0000 0.0008 -0.0000 -0.0000 0.0000 -0.0268 0.0000 0.0000 0.3667 0.0000
-0.0000 -0.0000 -0.0000 0.0000 -0.0000 -0.0000 -0.0000 -0.0000 0.0000 0.0000
0.0000 -0.0000 0.0000 -0.0000 -0.0000 0.0043 -0.0242 -0.0189 -0.0569 -0.0545
1.0374

62 : 0.0000 0.0000 0.0000 -0.0000 -0.0112 0.0000 -0.0000 -0.0000 -0.0000 0.0001
0.0000 -0.0000 0.0007 -0.0000 -0.0000 0.0000 -0.0000 0.3671 -0.0000 0.0117
-0.0445 0.0000 -0.0002 0.0000 0.0001 0.0000 -0.0000 0.0000 0.0008 0.0000
-0.0002 -0.0073 -0.0008 0.0001 0.0000 -0.0051 0.0000 0.0000 -0.0010 -0.0040
0.0002 -0.0005 -0.0007 0.0000 -0.0000 -0.0000 0.0001 0.0000 -0.0000 0.0000
-0.0000 0.0000 0.0000 -0.0000 -0.0000 -0.0026 -0.0036 0.0024 0.0003 -0.0003
0.0001 1.0563

63 : 0.0000 0.0000 0.0000 0.0000 0.0117 -0.0000 -0.0000 -0.0000 0.0001 0.0004
0.0000 -0.0000 0.0005 0.0000 -0.0000 0.0000 0.0000 0.3645 0.0000 -0.0134
-0.0249 -0.0000 -0.0001 -0.0000 -0.0001 0.0000 0.0000 0.0000 -0.0087 -0.0000
0.0003 -0.0175 -0.0002 -0.0005 0.0000 -0.0008 -0.0000 -0.0000 0.0002 -0.0014
-0.0005 -0.0013 0.0014 -0.0000 0.0000 0.0000 -0.0001 -0.0000 -0.0001 -0.0000
0.0000 -0.0000 -0.0000 0.0000 -0.0000 -0.0074 0.0022 -0.0012 0.0001 0.0000
-0.0002 -0.0838 1.0648

64 : -0.0000 -0.0000 -0.0000 -0.0000 -0.0153 -0.0000 0.0000 0.0000 0.0000 -0.0015
-0.0000 -0.0002 -0.0020 0.0000 -0.0000 -0.0000 0.0000 0.3790 -0.0000 -0.0286

-0.0354 -0.0000 0.0002 -0.0000 -0.0001 -0.0000 0.0000 0.0000 0.0033 -0.0000

-0.0001 0.0031 0.0033 -0.0009 -0.0000 0.0025 -0.0000 0.0000 0.0002 -0.0020

-0.0005 -0.0078 -0.0013 -0.0000 0.0000 -0.0000 -0.0000 0.0000 0.0001 -0.0000

0.0000 -0.0000 0.0000 -0.0000 0.0000 0.0009 -0.0008 -0.0001 0.0001 0.0001

-0.0001 -0.0501 -0.0579 1.0078

65 : -0.0100 -0.0008 -0.0002 0.0002 -0.0008 0.0000 -0.0002 -0.0761 0.0001 0.0076

-0.0014 -0.0257 -0.0021 -0.0655 -0.0227 0.0088 -0.0513 -0.0000 0.0014 0.0059

-0.0001 0.4031 0.0001 0.0000 0.0001 0.0000 -0.0001 -0.0000 -0.0006 -0.0031

0.0003 0.0004 -0.0002 0.0009 -0.0012 0.0004 0.0000 -0.0001 -0.0001 0.0019

0.0014 -0.0005 0.0002 -0.0002 0.0001 0.0004 -0.0042 0.0013 0.0006 -0.0012

0.0002 -0.0013 -0.0002 0.0003 -0.0000 0.0000 0.0001 0.0001 0.0006 -0.0001

-0.0001 -0.0000 0.0000 -0.0001 0.9896

66 : -0.0009 -0.0036 0.0006 0.0002 -0.0000 -0.0000 0.0005 -0.0165 -0.0000 0.0014

0.0003 -0.0071 0.0001 -0.0168 0.0004 0.0035 0.3740 -0.0000 0.0002 0.0000

-0.0000 -0.0478 -0.0000 -0.0000 -0.0003 0.0002 -0.0003 -0.0000 -0.0000 -0.0006

-0.0000 -0.0000 0.0000 0.0000 -0.0003 0.0000 -0.0000 0.0002 0.0000 0.0000

0.0000 0.0000 0.0000 -0.0000 0.0000 -0.0000 -0.0000 -0.0000 0.0008 -0.0002

0.0003 -0.0001 0.0002 0.0000 -0.0001 -0.0000 0.0000 -0.0000 0.0000 0.0000

0.0000 -0.0000 -0.0000 0.0000 0.0169 1.0521

67 : 0.0048 0.0045 0.0018 -0.0000 0.0001 -0.0000 -0.0007 0.0055 0.0000 0.0003

0.0009 -0.0029 0.0000 -0.0153 0.0015 0.0005 0.3683 0.0000 -0.0011 0.0001

0.0000 -0.0489 0.0002 -0.0001 0.0002 -0.0005 0.0012 0.0000 -0.0000 -0.0001

-0.0000 0.0001 0.0001 0.0000 -0.0001 0.0000 -0.0000 -0.0002 0.0000 -0.0000

-0.0003 0.0001 0.0000 -0.0000 0.0000 0.0000 -0.0000 -0.0003 -0.0013 0.0002

-0.0002 -0.0000 -0.0001 0.0002 0.0000 -0.0000 0.0000 -0.0000 0.0000 0.0000

0.0000 -0.0000 0.0000 -0.0000 -0.0360 -0.0679 1.0337

68 : 0.0008 -0.0011 0.0008 -0.0000 -0.0002 0.0000 -0.0004 -0.0071 -0.0000 -0.0032

0.0005 0.0067 -0.0012 0.0060 -0.0036 -0.0025 0.3668 0.0000 0.0003 -0.0001

0.0000 -0.0392 -0.0000 0.0000 0.0000 -0.0000 -0.0001 -0.0000 0.0001 0.0001

0.0000 -0.0001 -0.0000 -0.0000 0.0001 -0.0000 -0.0000 -0.0001 -0.0001 -0.0001

-0.0001 0.0000 -0.0000 0.0000 -0.0000 0.0000 -0.0001 -0.0002 0.0006 -0.0000

-0.0000 -0.0001 0.0000 -0.0003 0.0000 0.0000 0.0000 -0.0003 0.0000 -0.0000
-0.0000 0.0000 -0.0000 -0.0000 -0.0330 -0.0588 -0.0555 1.0416

69 : 0.0063 -0.0083 -0.0251 -0.0058 0.0001 -0.0014 0.0008 -0.0001 0.0000 0.0001
0.0000 0.0000 -0.0000 -0.0003 0.0001 0.0000 -0.0007 0.0000 0.2810 0.0002
0.0001 0.0009 -0.0001 0.0008 -0.0002 0.0000 0.0002 0.0001 -0.0005 -0.0000
-0.0000 -0.0000 0.0002 0.0000 -0.0001 0.0000 -0.0002 -0.0000 -0.0000 0.0000
-0.0005 0.0006 0.0010 0.0000 0.0000 0.0000 -0.0001 0.0003 0.0026 0.0003
-0.0000 -0.0000 0.0000 -0.0000 -0.0000 -0.0000 0.0000 0.0000 0.0000 -0.0000
0.0000 0.0000 0.0000 -0.0001 0.0009 0.0003 0.0045 -0.0004 2.0961

70 : -0.0004 -0.0040 -0.0000 0.0021 0.0000 -0.0002 -0.0102 -0.0000 0.0000 0.0000
-0.0000 -0.0000 -0.0000 -0.0005 0.0001 -0.0000 -0.0000 0.0000 0.0001 0.0000
0.0000 -0.0000 0.0000 -0.0001 0.0000 -0.0210 -0.0552 0.3954 0.0000 0.0000
0.0000 0.0000 0.0000 0.0000 0.0002 0.0000 0.0000 -0.0000 -0.0000 0.0000
0.0000 0.0000 0.0000 -0.0000 0.0000 0.0000 0.0000 0.0001 -0.0000 -0.0012
0.0008 0.0000 -0.0000 -0.0000 0.0000 -0.0000 0.0000 0.0000 0.0000 0.0000
-0.0000 -0.0000 0.0000 0.0000 -0.0000 -0.0000 -0.0000 -0.0000 -0.0000 1.0056

71 : 0.0001 -0.0002 0.0007 0.0014 -0.0000 -0.0003 0.0094 -0.0000 -0.0000 -0.0000
0.0000 0.0000 0.0000 0.0001 -0.0000 0.0000 0.0000 0.0000 -0.0000 0.0000
-0.0000 0.0000 0.0000 -0.0000 -0.0000 -0.0054 -0.0383 0.3657 0.0000 -0.0000
0.0000 0.0000 -0.0000 -0.0000 -0.0001 -0.0000 0.0000 0.0000 0.0000 0.0000
0.0000 0.0000 -0.0000 0.0000 -0.0000 -0.0000 -0.0000 0.0000 -0.0000 -0.0002
0.0000 0.0000 -0.0000 0.0000 0.0000 -0.0000 -0.0000 0.0000 -0.0000 0.0000
0.0000 0.0000 0.0000 0.0000 0.0000 -0.0000 0.0000 0.0000 0.0000 -0.0645
1.0416

72 : -0.0004 -0.0027 0.0007 -0.0045 -0.0000 0.0002 -0.0068 0.0000 0.0000 0.0000
0.0000 -0.0000 0.0000 0.0001 -0.0001 0.0000 -0.0000 0.0000 -0.0001 0.0000
-0.0000 0.0000 -0.0000 0.0000 -0.0000 -0.0008 -0.0307 0.3723 -0.0000 0.0000
-0.0000 0.0000 -0.0000 -0.0000 0.0004 -0.0000 -0.0001 0.0000 0.0000 -0.0000
-0.0000 -0.0000 0.0000 0.0000 0.0000 -0.0000 0.0000 -0.0000 0.0000 0.0012
-0.0018 0.0000 0.0000 -0.0001 0.0000 0.0000 -0.0000 0.0000 -0.0000 -0.0000
0.0000 0.0000 0.0000 0.0000 -0.0000 0.0000 0.0000 -0.0000 -0.0000 -0.0553

-0.0617 1.0376

73 : 0.0002 -0.0188 -0.0094 -0.0115 0.0000 0.0005 -0.0630 0.0000 0.0000 -0.0000
-0.0000 0.0000 -0.0000 -0.0005 0.0002 0.0000 0.0003 -0.0000 0.0008 -0.0000
0.0000 -0.0003 -0.0000 -0.0003 -0.0000 -0.0980 0.3756 -0.0451 -0.0000 0.0000
-0.0000 -0.0000 0.0000 0.0000 0.0001 0.0000 -0.0002 -0.0000 -0.0000 -0.0000
-0.0000 -0.0000 -0.0000 -0.0000 0.0000 -0.0000 0.0000 0.0001 0.0000 -0.0002
0.0002 0.0000 0.0000 -0.0000 -0.0000 -0.0000 0.0000 0.0000 0.0000 -0.0000
0.0000 -0.0000 0.0000 0.0000 -0.0001 -0.0010 -0.0000 0.0001 -0.0000 0.0166
-0.0196 -0.0300 1.1057

74 : 0.0001 -0.0048 -0.0216 -0.0678 -0.0000 -0.0136 -0.0041 0.0000 -0.0000 0.0000
-0.0000 0.0000 0.0000 -0.0001 -0.0000 -0.0000 -0.0000 -0.0000 0.0002 -0.0000
0.0000 0.0000 0.0000 0.0002 -0.0000 0.4005 -0.0589 -0.0167 -0.0000 0.0000
-0.0000 -0.0000 -0.0000 -0.0000 0.0001 -0.0000 -0.0016 -0.0000 -0.0000 -0.0000
-0.0000 -0.0000 -0.0000 0.0000 -0.0000 -0.0000 0.0000 0.0001 -0.0000 -0.0003
-0.0001 0.0001 -0.0000 0.0000 -0.0000 0.0000 0.0000 0.0000 -0.0000 -0.0000
-0.0000 0.0000 -0.0000 0.0000 0.0000 0.0000 -0.0002 -0.0000 -0.0001 -0.0063
-0.0055 0.0014 0.0118 1.0128

75 : 0.0001 0.0003 0.0037 -0.0377 -0.0000 -0.0039 0.0117 0.0000 -0.0000 0.0000
-0.0000 0.0000 0.0000 -0.0000 0.0000 -0.0000 -0.0000 0.0000 -0.0016 0.0000
0.0000 -0.0000 0.0000 -0.0007 0.0000 0.3869 -0.0434 -0.0053 -0.0000 -0.0000
-0.0000 0.0000 0.0000 0.0000 -0.0000 0.0000 -0.0004 -0.0000 0.0000 0.0000
-0.0000 0.0000 0.0000 -0.0000 -0.0000 0.0000 0.0000 0.0000 -0.0000 0.0002
-0.0000 -0.0000 -0.0000 0.0000 -0.0000 0.0000 0.0000 -0.0000 0.0000 0.0000
0.0000 -0.0000 0.0000 -0.0000 0.0000 0.0000 -0.0002 0.0000 -0.0008 -0.0053
-0.0064 0.0028 -0.0225 -0.0411 1.0083

76 : 0.0007 -0.0004 0.0095 -0.0171 0.0000 -0.0763 -0.0000 0.0000 0.0000 0.0000
-0.0000 0.0000 -0.0000 0.0002 -0.0001 0.0000 -0.0000 -0.0000 -0.0001 -0.0000
0.0000 0.0000 0.0000 0.3771 0.0001 -0.0052 0.0000 -0.0002 -0.0000 0.0000
0.0000 0.0000 -0.0000 0.0000 0.0000 0.0000 -0.0104 -0.0000 -0.0000 0.0000
0.0001 -0.0000 -0.0000 -0.0000 -0.0000 0.0000 0.0000 -0.0001 0.0002 -0.0000
-0.0000 0.0000 -0.0000 0.0000 -0.0000 -0.0000 0.0000 0.0000 0.0000 -0.0000

-0.0000 -0.0000 0.0000 0.0000 -0.0000 0.0000 0.0000 -0.0000 0.0027 0.0000
-0.0000 0.0000 -0.0001 -0.0028 0.0002 1.0823

77 : -0.0002 -0.0006 -0.0086 -0.0012 -0.0000 -0.0521 -0.0003 -0.0000 0.0000 -0.0000
0.0000 -0.0000 -0.0000 -0.0001 0.0000 -0.0000 0.0000 0.0000 0.0009 0.0000
-0.0000 -0.0000 -0.0000 0.3488 -0.0000 -0.0032 -0.0010 0.0002 -0.0000 -0.0000
0.0000 -0.0000 0.0000 0.0000 -0.0000 -0.0000 -0.0018 0.0000 0.0000 0.0000
-0.0000 0.0000 0.0000 -0.0000 0.0000 -0.0000 -0.0000 0.0000 -0.0000 0.0000
-0.0000 -0.0000 0.0000 -0.0000 0.0000 -0.0000 -0.0000 -0.0000 -0.0000 0.0000
0.0000 0.0000 -0.0000 -0.0000 0.0000 0.0000 0.0000 0.0000 -0.0003 0.0002
0.0000 -0.0000 -0.0002 -0.0041 0.0015 -0.1014 1.1084

78 : -0.0000 -0.0009 -0.0060 0.0029 0.0000 -0.0527 -0.0000 -0.0000 -0.0000 -0.0000
0.0000 -0.0000 -0.0000 -0.0001 0.0000 -0.0000 0.0000 0.0000 0.0000 -0.0000
-0.0000 -0.0000 0.0000 0.3564 -0.0000 0.0040 -0.0002 -0.0000 -0.0000 -0.0000
0.0000 0.0000 -0.0000 0.0000 -0.0000 -0.0000 -0.0072 -0.0000 0.0000 -0.0000
0.0000 -0.0000 0.0000 -0.0000 0.0000 -0.0000 0.0000 0.0000 -0.0000 0.0000
-0.0000 0.0000 0.0000 0.0000 0.0000 0.0000 -0.0000 -0.0000 -0.0000 0.0000
0.0000 0.0000 -0.0000 -0.0000 0.0000 -0.0000 0.0000 0.0000 0.0003 -0.0001
0.0000 -0.0000 0.0001 0.0012 0.0005 -0.1024 -0.0484 1.1024

79 : 0.0003 -0.0022 0.0099 -0.0074 0.0000 -0.0755 -0.0001 0.0000 0.0000 0.0000
-0.0000 0.0000 -0.0000 0.0001 0.0000 0.0000 -0.0002 0.0000 -0.0059 0.0000
0.0000 -0.0000 0.0000 -0.0091 -0.0000 -0.0032 0.0011 -0.0000 -0.0000 0.0000
-0.0000 0.0000 0.0000 0.0000 -0.0000 0.0000 0.3676 -0.0000 -0.0000 0.0000
-0.0001 0.0000 0.0000 0.0000 0.0000 0.0000 -0.0000 -0.0000 0.0001 -0.0000
-0.0000 -0.0000 -0.0000 0.0000 -0.0000 -0.0000 0.0000 0.0000 0.0000 -0.0000
-0.0000 0.0000 0.0000 0.0000 -0.0000 -0.0000 0.0009 -0.0000 0.0043 0.0000
0.0000 0.0000 0.0003 -0.0025 -0.0000 0.0391 -0.0024 -0.0174 1.1455

80 : -0.0001 0.0005 -0.0078 -0.0184 -0.0000 -0.0450 0.0001 -0.0000 0.0000 -0.0000
0.0000 -0.0000 0.0000 -0.0000 -0.0000 -0.0000 0.0001 -0.0000 0.0007 -0.0000
-0.0000 0.0000 -0.0000 -0.0027 0.0000 0.0047 -0.0009 -0.0000 0.0000 -0.0000
0.0000 -0.0000 -0.0000 -0.0000 0.0000 -0.0000 0.3551 -0.0000 0.0000 -0.0000
0.0000 0.0000 -0.0000 -0.0000 0.0000 -0.0000 0.0000 0.0000 -0.0000 0.0000

-0.0000 0.0000 0.0000 -0.0000 0.0000 0.0000 -0.0000 -0.0000 -0.0000 0.0000
0.0000 0.0000 -0.0000 -0.0000 0.0000 -0.0000 -0.0003 0.0000 0.0002 -0.0000
-0.0000 -0.0000 0.0001 0.0018 0.0000 -0.0018 -0.0109 0.0071 -0.1112 1.1154
81 : -0.0000 0.0002 -0.0010 -0.0044 -0.0000 -0.0583 -0.0000 -0.0000 0.0000 -0.0000
0.0000 -0.0000 0.0000 -0.0000 -0.0000 -0.0000 0.0000 0.0000 0.0010 -0.0000
-0.0000 0.0000 -0.0000 -0.0048 -0.0000 -0.0034 -0.0005 -0.0001 0.0000 -0.0000
-0.0000 -0.0000 0.0000 0.0000 0.0000 -0.0000 0.3618 -0.0000 0.0000 -0.0000
-0.0000 0.0000 0.0000 -0.0000 0.0000 -0.0000 -0.0000 0.0000 -0.0000 -0.0000
0.0000 0.0000 0.0000 -0.0000 0.0000 0.0000 -0.0000 -0.0000 -0.0000 0.0000
0.0000 0.0000 -0.0000 -0.0000 0.0000 -0.0000 -0.0001 -0.0000 -0.0007 -0.0000
-0.0000 -0.0000 -0.0001 0.0003 -0.0025 -0.0190 0.0070 0.0029 -0.1247 -0.0447
1.0984
82 : -0.0619 0.3602 -0.1172 -0.0162 -0.0000 -0.0002 -0.0711 -0.0008 0.0000 -0.0001
0.0001 0.0001 0.0000 -0.0103 -0.0014 -0.0001 0.0044 0.0000 -0.0025 0.0000
0.0000 0.0030 0.0002 0.0003 0.0013 -0.0104 -0.0274 -0.0008 0.0000 -0.0000
0.0000 -0.0000 -0.0000 0.0000 -0.0007 -0.0000 -0.0001 0.0001 0.0000 0.0000
0.0000 0.0000 0.0000 0.0000 0.0000 -0.0000 0.0000 -0.0002 -0.0018 -0.0021
0.0001 0.0001 -0.0001 0.0001 0.0001 0.0000 -0.0000 0.0000 -0.0000 0.0000
0.0000 0.0000 0.0000 -0.0000 0.0024 -0.0079 -0.0143 0.0031 -0.0059 0.0004
0.0011 -0.0026 0.0179 -0.0010 0.0029 0.0001 -0.0004 0.0001 -0.0008 0.0004
0.0001 1.2184
83 : -0.0448 -0.0041 -0.0001 0.0006 0.0000 0.0000 0.0069 -0.0177 0.0000 -0.0002
-0.0052 -0.0014 -0.0001 0.3944 -0.0369 0.0091 -0.0104 -0.0000 -0.0002 -0.0000
-0.0000 -0.0913 -0.0017 0.0000 0.0084 -0.0002 -0.0006 0.0005 -0.0001 -0.0002
-0.0000 -0.0000 0.0000 0.0001 -0.0224 0.0000 0.0000 -0.0003 -0.0000 0.0000
-0.0000 0.0000 -0.0000 -0.0002 -0.0001 0.0003 -0.0010 0.0006 0.0039 -0.0103
-0.0030 0.0023 0.0000 -0.0074 0.0009 -0.0000 0.0000 -0.0000 0.0000 -0.0000
-0.0000 -0.0000 -0.0000 -0.0000 0.0088 0.0002 -0.0113 0.0013 0.0002 0.0003
-0.0001 -0.0002 -0.0005 -0.0001 -0.0001 0.0000 0.0000 -0.0000 -0.0000 -0.0000
0.0000 0.0010 1.0335
84 : -0.0128 -0.0505 0.4325 -0.0687 -0.0000 -0.0174 -0.0090 -0.0002 0.0000 0.0000

0.0000 -0.0000 -0.0000 -0.0033 0.0003 0.0000 0.0002 0.0000 -0.0493 0.0000
0.0000 -0.0006 -0.0001 0.0022 -0.0001 -0.0175 -0.0021 -0.0003 0.0000 0.0000
0.0000 -0.0000 -0.0001 -0.0001 0.0004 -0.0000 -0.0021 0.0000 -0.0000 -0.0000
0.0005 -0.0000 -0.0000 0.0000 0.0000 -0.0000 -0.0000 -0.0011 -0.0002 -0.0023
0.0001 0.0004 -0.0000 -0.0000 0.0000 -0.0000 -0.0000 -0.0000 -0.0000 -0.0000
-0.0000 -0.0000 0.0000 0.0000 -0.0001 -0.0001 -0.0015 0.0002 -0.0167 -0.0007
0.0004 -0.0003 -0.0015 -0.0034 -0.0008 0.0071 -0.0076 -0.0041 0.0038 -0.0018
-0.0003 0.0012 -0.0010 0.9611

85 : 0.0010 -0.0171 -0.0865 0.4044 0.0000 -0.0507 -0.0019 0.0000 -0.0000 -0.0000
-0.0000 -0.0000 -0.0000 -0.0003 0.0001 0.0000 0.0004 0.0000 -0.0103 0.0000
-0.0000 -0.0002 -0.0000 0.0065 -0.0000 -0.0438 -0.0176 -0.0003 0.0000 0.0000
-0.0000 -0.0000 -0.0000 0.0000 0.0000 0.0000 -0.0168 -0.0000 -0.0000 0.0000
0.0000 -0.0000 0.0000 0.0000 0.0000 -0.0000 0.0000 0.0000 -0.0000 -0.0002
-0.0000 0.0000 0.0000 0.0000 -0.0000 -0.0000 0.0000 -0.0000 0.0000 -0.0000
-0.0000 -0.0000 0.0000 -0.0000 0.0000 0.0007 -0.0023 0.0002 -0.0043 0.0002
-0.0001 -0.0010 0.0029 0.0160 -0.0334 -0.0035 0.0045 -0.0041 -0.0086 -0.0004
0.0066 -0.0090 0.0003 0.0123 1.0246

86 : 0.0000 0.0000 -0.0001 0.0000 0.4198 0.0000 -0.0000 0.0000 0.0000 0.0016
0.0000 -0.0012 -0.0004 0.0000 -0.0000 0.0000 -0.0000 0.0027 0.0002 -0.0010
-0.0325 -0.0015 -0.0003 0.0000 -0.0000 -0.0000 0.0000 -0.0000 -0.0056 0.0001
-0.0001 -0.0144 -0.0012 -0.0006 0.0000 -0.0056 0.0000 -0.0000 0.0003 0.0026
0.0002 -0.0014 0.0002 -0.0000 -0.0000 -0.0000 -0.0003 -0.0001 -0.0013 0.0000
0.0000 -0.0000 0.0000 0.0000 -0.0000 0.0007 -0.0012 -0.0026 -0.0005 0.0005
-0.0001 -0.0043 0.0017 -0.0038 0.0008 0.0002 -0.0002 0.0003 -0.0008 0.0000
-0.0000 0.0000 -0.0000 -0.0000 0.0000 -0.0000 0.0000 0.0000 -0.0000 0.0000
0.0000 0.0000 -0.0000 -0.0000 0.0000 0.3649

87 : 0.0000 0.0001 0.0000 0.0000 -0.0231 0.0000 0.0000 -0.0102 -0.0000 0.4763
0.0000 -0.0330 -0.0808 0.0019 0.0003 -0.0004 -0.0076 0.0003 -0.0000 -0.0022
0.0044 0.0069 -0.0001 0.0000 -0.0000 -0.0000 0.0000 0.0000 -0.0000 0.0046
0.0000 0.0085 0.0001 -0.0000 0.0000 -0.0013 -0.0000 -0.0000 -0.0012 -0.0000
0.0000 0.0004 -0.0000 -0.0000 -0.0000 0.0000 -0.0001 -0.0000 0.0001 0.0000

0.0000 0.0000 -0.0000 -0.0000 0.0000 0.0008 0.0020 -0.0006 -0.0004 -0.0000
-0.0002 -0.0000 0.0002 0.0001 -0.0009 0.0007 0.0005 -0.0030 0.0001 0.0000
0.0000 0.0000 -0.0000 -0.0000 0.0000 0.0000 0.0000 0.0000 0.0000 0.0000
0.0000 0.0000 -0.0000 0.0000 0.0000 0.0405 6.3066

88 : -0.0000 -0.0000 -0.0000 0.0000 -0.0001 -0.0000 0.0000 -0.0337 0.0000 -0.0557
0.0012 0.4601 -0.0046 -0.0004 0.0002 -0.0180 -0.0008 -0.0000 -0.0000 -0.0001
-0.0003 -0.0104 -0.0001 -0.0000 -0.0000 0.0000 0.0000 -0.0000 0.0000 -0.0349
-0.0000 -0.0009 -0.0000 -0.0000 0.0001 -0.0005 0.0000 0.0003 0.0009 -0.0003
-0.0000 0.0000 -0.0000 0.0000 0.0000 -0.0000 0.0001 -0.0000 0.0004 0.0000
0.0000 -0.0000 0.0004 -0.0007 -0.0004 -0.0001 -0.0004 0.0001 -0.0027 0.0001
0.0005 0.0000 0.0000 -0.0000 -0.0045 0.0009 -0.0001 -0.0036 -0.0000 -0.0000
-0.0000 0.0000 -0.0000 0.0000 -0.0000 0.0000 -0.0000 -0.0000 0.0000 0.0000
0.0000 0.0000 -0.0007 -0.0000 -0.0000 -0.0006 -0.0124 0.8798

89 : -0.0001 0.0000 -0.0014 0.0003 -0.0002 -0.0002 0.0001 0.0000 0.0000 -0.0001
-0.0000 -0.0000 0.0000 -0.0001 0.0000 -0.0000 0.0004 0.0000 0.0025 0.0005
-0.0003 0.0002 0.0000 0.0001 -0.0000 -0.0000 0.0000 0.0000 -0.0002 -0.0000
-0.0000 -0.0000 0.0017 0.0000 -0.0000 -0.0000 0.0001 -0.0000 -0.0000 -0.0000
-0.0024 -0.0002 0.0025 0.0000 0.0000 -0.0000 -0.0000 -0.0001 -0.0005 0.0000
-0.0000 0.0000 -0.0000 0.0000 0.0000 0.0000 0.0000 -0.0001 -0.0000 -0.0000
0.0000 -0.0000 0.0000 0.0000 0.0013 -0.0001 -0.0027 0.0003 -0.0399 0.0000
-0.0000 0.0000 0.0000 -0.0000 0.0000 -0.0004 -0.0000 0.0001 0.0005 -0.0001
-0.0001 0.0001 0.0000 0.0005 0.0001 0.0024 0.0001 -0.0000 0.9874

90 : 0.0003 -0.0001 -0.0051 0.0004 0.0000 -0.0000 0.0000 0.0001 -0.0000 -0.0001
-0.0000 -0.0000 0.0000 0.0001 -0.0000 0.0000 0.0026 -0.0000 -0.0089 -0.0001
-0.0001 -0.0007 -0.0000 0.0001 0.0001 0.0001 0.0000 0.0000 0.0003 -0.0000
0.0000 -0.0000 -0.0013 0.0000 0.0000 0.0000 0.0001 0.0000 0.0000 -0.0000
0.0005 0.0007 -0.0005 -0.0000 0.0000 -0.0000 0.0000 0.0000 -0.0006 0.0000
0.0000 0.0000 0.0000 0.0000 -0.0000 -0.0000 0.0000 -0.0001 -0.0000 0.0000
0.0000 -0.0000 0.0000 -0.0000 0.0015 0.0012 -0.0079 0.0009 -0.0453 0.0000
0.0000 -0.0000 0.0000 0.0000 -0.0000 0.0003 -0.0000 -0.0000 -0.0029 0.0009
-0.0002 -0.0002 0.0000 -0.0011 0.0001 0.0022 0.0001 -0.0000 -0.0641 1.0322

91 : 0.0007 0.0003 -0.0016 -0.0011 -0.0009 -0.0002 -0.0001 -0.0001 -0.0000 -0.0002
0.0000 0.0002 -0.0000 0.0001 0.0002 0.0000 0.0021 0.0001 -0.0109 -0.0012
-0.0007 0.0048 -0.0001 -0.0000 0.0008 0.0001 0.0000 0.0000 0.0015 0.0000
0.0001 -0.0000 0.0015 -0.0002 -0.0000 -0.0000 0.0002 -0.0000 0.0000 0.0004
-0.0011 0.0006 -0.0011 -0.0000 0.0000 -0.0000 0.0002 0.0008 -0.0016 0.0000
-0.0000 0.0000 0.0000 -0.0000 0.0000 -0.0000 -0.0000 0.0001 -0.0000 -0.0000
-0.0000 0.0002 -0.0002 0.0003 0.0026 -0.0020 0.0033 -0.0001 -0.0304 -0.0000
0.0000 0.0000 -0.0000 0.0000 -0.0000 0.0001 -0.0000 -0.0000 -0.0005 0.0000
-0.0000 0.0000 0.0002 -0.0006 -0.0006 0.0042 -0.0023 0.0001 -0.0602 -0.0643
0.8612
92 : -0.0024 -0.0011 -0.0131 -0.0008 -0.0006 -0.0004 0.0003 0.0001 -0.0000 -0.0002
-0.0000 -0.0000 0.0000 -0.0004 0.0001 -0.0000 0.0020 -0.0000 -0.0821 -0.0001
-0.0003 0.0001 0.0000 0.0002 -0.0000 0.0001 -0.0000 0.0000 0.0009 -0.0000
0.0001 0.0001 0.0018 0.0001 -0.0000 -0.0000 -0.0003 -0.0000 0.0000 -0.0001
0.0028 -0.0007 -0.0018 -0.0000 0.0000 -0.0000 0.0000 -0.0000 -0.0004 0.0000
-0.0000 0.0000 -0.0000 0.0000 0.0000 -0.0000 0.0000 -0.0001 0.0000 -0.0000
0.0000 -0.0000 0.0000 0.0001 0.0026 -0.0003 0.0033 -0.0002 0.4122 0.0000
-0.0000 -0.0000 0.0000 0.0000 -0.0001 -0.0007 0.0001 0.0001 0.0008 0.0000
-0.0001 -0.0003 -0.0001 -0.0026 -0.0008 0.0028 0.0003 -0.0000 0.3778 0.3648
0.3772 2.0948
93 : -0.0044 -0.0021 -0.0057 0.0006 -0.0000 0.0004 -0.0001 0.0001 0.0000 -0.0000
0.0000 -0.0000 0.0000 0.0004 -0.0003 -0.0000 0.0006 -0.0000 -0.0766 0.0000
0.0000 0.0000 0.0000 -0.0019 0.0004 0.0006 -0.0003 -0.0000 0.0001 0.0000
-0.0000 -0.0000 -0.0047 -0.0001 0.0001 -0.0000 0.0003 -0.0000 -0.0000 0.0000
0.0057 -0.0011 0.0002 0.0000 0.0000 -0.0000 -0.0000 -0.0020 0.0041 -0.0001
0.0000 0.0000 -0.0000 -0.0000 -0.0000 -0.0000 0.0000 -0.0000 -0.0000 -0.0000
-0.0000 -0.0000 0.0000 0.0000 -0.0007 0.0000 -0.0023 0.0001 0.5461 -0.0000
-0.0000 -0.0000 -0.0000 0.0000 0.0000 0.0024 -0.0001 0.0002 -0.0017 0.0000
0.0000 -0.0009 -0.0001 0.0174 -0.0003 0.0001 0.0000 0.0000 -0.0098 0.0078
-0.0083 -0.0464 6.2017
94 : -0.0000 0.0000 0.0000 -0.0000 0.0001 -0.0000 0.0000 -0.0091 0.0001 -0.0196

0.0003 -0.0552 -0.0032 0.0003 0.0009 -0.0010 -0.0000 0.0001 -0.0000 -0.0003
0.0003 -0.0009 0.0002 -0.0000 0.0008 0.0000 0.0000 -0.0000 0.0002 0.3932
0.0000 -0.0001 0.0000 -0.0000 0.0000 -0.0008 0.0000 -0.0036 0.0004 -0.0016
0.0000 0.0000 0.0000 0.0001 0.0002 0.0002 -0.0025 0.0001 0.0005 0.0001
-0.0001 0.0000 -0.0004 -0.0006 -0.0014 -0.0002 -0.0002 -0.0003 0.0002 -0.0002
0.0001 0.0000 0.0000 -0.0000 -0.0007 -0.0002 0.0001 -0.0001 0.0000 -0.0000
-0.0000 0.0000 -0.0000 0.0000 0.0000 0.0000 -0.0000 -0.0000 0.0000 -0.0000
-0.0000 0.0000 -0.0002 -0.0000 -0.0000 0.0001 -0.0000 0.0093 0.0000 -0.0000
-0.0000 -0.0000 0.0000 0.9992

95 : 0.0000 -0.0000 0.0000 -0.0000 -0.0000 -0.0000 0.0000 -0.0219 -0.0000 0.0118
0.0001 -0.0289 0.0017 -0.0004 -0.0003 0.0063 -0.0001 -0.0000 -0.0000 0.0002
0.0000 -0.0019 -0.0001 -0.0000 0.0000 0.0000 0.0000 -0.0000 0.0000 0.3674
-0.0000 -0.0008 0.0000 -0.0000 -0.0000 0.0002 0.0000 -0.0014 -0.0001 -0.0001
0.0000 0.0000 0.0000 -0.0000 -0.0000 0.0000 0.0005 0.0000 -0.0000 0.0000
0.0000 -0.0000 -0.0004 0.0014 0.0005 -0.0004 0.0000 -0.0001 0.0009 -0.0002
0.0000 0.0000 0.0000 -0.0000 -0.0007 -0.0004 0.0002 -0.0004 -0.0000 0.0000
-0.0000 -0.0000 0.0000 0.0000 -0.0000 0.0000 -0.0000 -0.0000 0.0000 -0.0000
-0.0000 -0.0000 0.0002 -0.0000 0.0000 -0.0000 -0.0015 -0.0172 0.0000 -0.0000
-0.0000 0.0000 0.0000 -0.0678 1.0345

96 : -0.0000 -0.0000 0.0000 0.0000 0.0000 -0.0000 -0.0000 -0.0018 -0.0000 -0.0136
0.0007 -0.0401 -0.0055 0.0002 -0.0001 -0.0008 -0.0003 -0.0000 0.0000 -0.0003
-0.0002 -0.0018 0.0000 0.0000 -0.0000 -0.0000 0.0000 -0.0000 -0.0001 0.3615
-0.0000 -0.0018 -0.0000 0.0000 -0.0000 0.0009 -0.0000 -0.0009 -0.0003 0.0001
-0.0000 -0.0000 0.0000 -0.0000 -0.0000 0.0000 0.0003 -0.0000 0.0000 -0.0000
-0.0000 0.0000 -0.0005 0.0003 0.0021 -0.0000 0.0003 0.0007 -0.0023 0.0008
-0.0003 -0.0000 -0.0000 -0.0000 -0.0028 0.0000 -0.0003 0.0006 0.0000 -0.0000
0.0000 0.0000 0.0000 -0.0000 -0.0000 0.0000 -0.0000 -0.0000 0.0000 -0.0000
-0.0000 -0.0000 -0.0001 0.0000 0.0000 -0.0001 0.0004 -0.0247 -0.0000 0.0000
0.0000 -0.0000 -0.0000 -0.0624 -0.0639 1.0947

H I R S H F E L D C H A R G E A N A L Y S I S

For each fragment: the (numerical) integral of rho(scf) * rho(fragment)/rho(sum-of-fragments)

(nuclear charges are included, electrons are counted negative)

The fragments and their ordering are defined in the early G E O M E T R Y output section.

If you use single-atom fragments, this usually implies that all atoms of the same

chemical type are grouped together. This may not be the order in which you listed them

in the input file!

1	O	-0.1090
2	O	-0.2100
3	O	-0.1422
4	O	-0.1218
5	O	-0.0808
6	O	-0.2109
7	O	-0.0954
8	O	-0.2246
9	O	-0.2001
10	O	-0.2238
11	C	0.1018
12	C	0.0285
13	C	0.0134
14	C	0.1687
15	C	-0.1081
16	C	0.2127
17	C	-0.0291
18	C	0.0451

19	C	0.0896
20	C	-0.1072
21	C	-0.1032
22	C	-0.0537
23	C	0.0825
24	C	-0.0397
25	C	-0.0295
26	C	-0.0607
27	C	-0.0674
28	C	-0.0701
29	C	0.0495
30	C	-0.1030
31	C	0.0088
32	C	-0.1070
33	C	0.0569
34	C	-0.1113
35	C	0.1543
36	C	-0.1120
37	C	-0.0646
38	C	-0.0632
39	C	-0.0280
40	C	-0.1018
41	C	0.2116
42	C	-0.1006
43	N	-0.0994
44	H	0.0273
45	H	0.0343
46	H	0.0292
47	H	0.0401
48	H	0.0337
49	H	0.0335

50	H	0.0297
51	H	0.0284
52	H	0.0379
53	H	0.0202
54	H	0.0364
55	H	0.0339
56	H	0.0368
57	H	0.0350
58	H	0.0302
59	H	0.0415
60	H	0.0444
61	H	0.0431
62	H	0.0323
63	H	0.0296
64	H	0.0350
65	H	0.0407
66	H	0.0366
67	H	0.0384
68	H	0.0452
69	H	0.0247
70	H	0.0323
71	H	0.0264
72	H	0.0307
73	H	0.0397
74	H	0.0419
75	H	0.0403
76	H	0.0289
77	H	0.0354
78	H	0.0401
79	H	0.0235
80	H	0.0381

81	H	0.0391
82	H	0.0174
83	H	0.0365
84	H	0.0364
85	H	0.0269
86	H	0.0325
87	H	0.0344
88	H	0.0339
89	H	0.1045
90	H	0.0557
91	H	0.0544
92	H	0.0471
93	H	0.0541
94	H	0.0334
95	H	0.0374
96	H	0.0351

Sum of these charges (accuracy NumInt/Tails) = -0.00007652

V O R O N O I   C H A R G E S

For each atom: the (numerical) integral of the total electronic charge density in its Voronoi cell,

i.e. the region of space that is closer to that atom than to any other atom.

(cf. Wigner-Seitz cells in crystals)

Within the Voronoi cell the subintegrals over the atomic sphere and the remaining part are evaluated

separately to give the numbers of electrons (negative charge) in these regions.

The net total charge in the cell (including the nuclear charge) is also given.

Values are provided for

a) the Initial (sum-of-fragments) density

b) the Orthogonalized-Fragments density

c) the SCF density

d) the Voronoi Deformation Density (VDD): the difference SCF-Initial for the complete atomic cell

Atom	Initial			OrthFrag			SCF			
	Sphere	RestCell	NetTotal	Sphere	RestCell	NetTotal	Sphere	RestCell	NetTotal	VDD
-----	-----------------------			-----------------------			-----------------------			-----
1 O	-2.834	-5.379	-0.214	-3.043	-5.059	-0.102	-2.857	-5.493	-0.350	-0.137
2 C	-2.273	-2.974	0.753	-2.469	-3.016	0.515	-2.290	-2.859	0.851	0.099
3 C	-2.271	-3.043	0.686	-2.475	-3.080	0.445	-2.298	-3.010	0.691	0.006
4 C	-2.273	-3.084	0.643	-2.481	-3.099	0.420	-2.299	-3.066	0.635	-0.008
5 O	-2.834	-4.763	0.404	-3.013	-4.665	0.323	-2.856	-4.964	0.180	-0.223
6 N	-3.124	-3.924	-0.049	-3.549	-3.476	-0.025	-3.179	-4.030	-0.209	-0.160
7 O	-2.833	-5.395	-0.228	-3.023	-5.098	-0.120	-2.853	-5.545	-0.398	-0.170
8 C	-2.275	-3.532	0.193	-2.451	-3.373	0.176	-2.294	-3.348	0.357	0.165
9 C	-2.273	-2.259	1.467	-2.468	-2.500	1.032	-2.308	-2.309	1.383	-0.084
10 C	-2.278	-3.431	0.291	-2.452	-3.303	0.244	-2.294	-3.226	0.480	0.190
11 O	-2.833	-5.395	-0.228	-3.027	-5.076	-0.102	-2.853	-5.525	-0.378	-0.150
12 C	-2.271	-3.110	0.619	-2.486	-3.139	0.375	-2.303	-3.157	0.540	-0.079
13 O	-2.835	-5.367	-0.202	-3.045	-5.069	-0.114	-2.856	-5.454	-0.310	-0.108
14 C	-2.271	-3.067	0.663	-2.471	-3.093	0.436	-2.294	-3.013	0.693	0.031
15 C	-2.270	-3.497	0.233	-2.474	-3.398	0.128	-2.288	-3.425	0.286	0.053
16 O	-2.834	-5.287	-0.121	-2.997	-5.096	-0.093	-2.855	-5.510	-0.365	-0.244
17 C	-2.273	-2.275	1.452	-2.468	-2.517	1.015	-2.308	-2.322	1.370	-0.082
18 C	-2.273	-2.259	1.468	-2.468	-2.500	1.032	-2.309	-2.311	1.381	-0.087

19 O	-2.834	-5.368	-0.202	-3.042	-5.082	-0.124	-2.855	-5.474	-0.329	-0.127
20 C	-2.273	-3.163	0.564	-2.472	-3.138	0.390	-2.309	-3.201	0.490	-0.073
21 C	-2.271	-3.472	0.257	-2.480	-3.387	0.133	-2.289	-3.413	0.298	0.040
22 C	-2.272	-3.072	0.656	-2.489	-3.113	0.399	-2.304	-3.124	0.571	-0.085
23 C	-2.272	-3.087	0.640	-2.486	-3.116	0.398	-2.303	-3.129	0.568	-0.072
24 C	-2.273	-2.253	1.474	-2.463	-2.485	1.052	-2.304	-2.252	1.443	-0.030
25 C	-2.272	-2.702	1.026	-2.477	-2.834	0.689	-2.305	-2.737	0.958	-0.068
26 C	-2.273	-2.685	1.042	-2.477	-2.812	0.711	-2.307	-2.735	0.958	-0.084
27 C	-2.272	-3.038	0.691	-2.472	-3.061	0.468	-2.294	-2.986	0.720	0.029
28 C	-2.274	-2.244	1.483	-2.467	-2.487	1.046	-2.309	-2.303	1.388	-0.095
29 C	-2.273	-3.592	0.135	-2.485	-3.452	0.063	-2.306	-3.606	0.088	-0.047
30 C	-2.273	-2.248	1.478	-2.468	-2.494	1.039	-2.308	-2.296	1.396	-0.082
31 O	-2.834	-5.306	-0.140	-2.992	-5.120	-0.112	-2.854	-5.528	-0.381	-0.242
32 C	-2.270	-3.046	0.684	-2.468	-3.080	0.452	-2.293	-2.991	0.716	0.032
33 C	-2.274	-2.244	1.482	-2.472	-2.497	1.031	-2.310	-2.302	1.388	-0.094
34 C	-2.276	-3.553	0.171	-2.455	-3.382	0.163	-2.297	-3.386	0.317	0.146
35 C	-2.274	-2.257	1.469	-2.472	-2.504	1.024	-2.310	-2.316	1.374	-0.095
36 C	-2.273	-2.654	1.073	-2.478	-2.792	0.730	-2.306	-2.702	0.992	-0.081
37 C	-2.273	-2.247	1.480	-2.461	-2.476	1.062	-2.304	-2.243	1.453	-0.027
38 C	-2.273	-2.201	1.526	-2.456	-2.450	1.094	-2.301	-2.164	1.535	0.009
39 C	-2.273	-2.260	1.467	-2.465	-2.493	1.042	-2.307	-2.307	1.386	-0.081
40 H	-0.076	-1.510	-0.586	-0.107	-1.297	-0.404	-0.111	-1.431	-0.542	0.044
41 H	-0.076	-1.459	-0.534	-0.103	-1.260	-0.363	-0.109	-1.372	-0.481	0.054
42 H	-0.076	-1.452	-0.527	-0.104	-1.261	-0.366	-0.110	-1.382	-0.492	0.036
43 H	-0.075	-1.446	-0.521	-0.099	-1.270	-0.369	-0.108	-1.366	-0.474	0.047
44 H	-0.076	-1.436	-0.511	-0.100	-1.258	-0.359	-0.111	-1.365	-0.476	0.036
45 H	-0.076	-1.437	-0.513	-0.099	-1.266	-0.366	-0.111	-1.364	-0.475	0.038
46 H	-0.076	-1.416	-0.492	-0.104	-1.240	-0.344	-0.111	-1.353	-0.464	0.028
47 H	-0.076	-1.511	-0.586	-0.107	-1.285	-0.392	-0.111	-1.419	-0.530	0.057
48 H	-0.076	-1.486	-0.562	-0.103	-1.276	-0.379	-0.111	-1.386	-0.497	0.065
49 H	-0.076	-1.523	-0.599	-0.111	-1.271	-0.381	-0.112	-1.428	-0.540	0.058

50 H	-0.076	-1.447	-0.522	-0.102	-1.260	-0.362	-0.109	-1.365	-0.474	0.048
51 H	-0.076	-1.407	-0.483	-0.103	-1.240	-0.343	-0.111	-1.346	-0.457	0.026
52 H	-0.076	-1.449	-0.524	-0.104	-1.257	-0.361	-0.111	-1.369	-0.480	0.044
53 H	-0.076	-1.413	-0.488	-0.101	-1.253	-0.354	-0.112	-1.346	-0.458	0.031
54 H	-0.075	-1.410	-0.486	-0.102	-1.249	-0.351	-0.111	-1.348	-0.459	0.027
55 H	-0.076	-1.416	-0.492	-0.102	-1.257	-0.359	-0.112	-1.338	-0.449	0.043
56 H	-0.075	-1.482	-0.557	-0.102	-1.284	-0.385	-0.112	-1.373	-0.485	0.072
57 H	-0.076	-1.442	-0.518	-0.103	-1.251	-0.355	-0.111	-1.351	-0.461	0.056
58 H	-0.076	-1.493	-0.568	-0.103	-1.289	-0.392	-0.111	-1.407	-0.518	0.050
59 H	-0.076	-1.445	-0.520	-0.101	-1.260	-0.361	-0.111	-1.374	-0.485	0.035
60 H	-0.075	-1.426	-0.501	-0.099	-1.257	-0.356	-0.110	-1.359	-0.469	0.032
61 H	-0.075	-1.408	-0.484	-0.099	-1.250	-0.348	-0.110	-1.339	-0.449	0.035
62 H	-0.076	-1.440	-0.516	-0.102	-1.261	-0.364	-0.110	-1.365	-0.475	0.041
63 H	-0.076	-1.439	-0.515	-0.100	-1.260	-0.359	-0.110	-1.361	-0.471	0.044
64 H	-0.076	-1.440	-0.516	-0.102	-1.254	-0.356	-0.110	-1.348	-0.458	0.059
65 H	-0.076	-1.555	-0.632	-0.111	-1.305	-0.416	-0.113	-1.446	-0.559	0.073
66 H	-0.076	-1.437	-0.513	-0.101	-1.261	-0.362	-0.111	-1.368	-0.479	0.034
67 H	-0.076	-1.461	-0.537	-0.107	-1.249	-0.355	-0.111	-1.384	-0.495	0.042
68 H	-0.076	-1.437	-0.512	-0.101	-1.255	-0.356	-0.110	-1.363	-0.472	0.040
69 C	-2.278	-3.406	0.316	-2.446	-3.280	0.273	-2.293	-3.188	0.519	0.203
70 H	-0.075	-1.428	-0.503	-0.099	-1.259	-0.358	-0.110	-1.353	-0.462	0.041
71 H	-0.076	-1.443	-0.518	-0.099	-1.266	-0.365	-0.110	-1.360	-0.470	0.048
72 H	-0.075	-1.406	-0.482	-0.099	-1.249	-0.347	-0.110	-1.336	-0.446	0.036
73 H	-0.075	-1.463	-0.538	-0.100	-1.273	-0.374	-0.112	-1.382	-0.493	0.045
74 H	-0.076	-1.456	-0.532	-0.105	-1.259	-0.363	-0.111	-1.374	-0.485	0.047
75 H	-0.075	-1.470	-0.546	-0.100	-1.278	-0.378	-0.110	-1.379	-0.489	0.056
76 H	-0.075	-1.439	-0.514	-0.101	-1.254	-0.355	-0.110	-1.372	-0.482	0.032
77 H	-0.075	-1.429	-0.504	-0.103	-1.257	-0.360	-0.111	-1.353	-0.464	0.040
78 H	-0.075	-1.402	-0.477	-0.099	-1.250	-0.349	-0.111	-1.332	-0.442	0.035
79 H	-0.075	-1.448	-0.524	-0.101	-1.268	-0.368	-0.111	-1.395	-0.506	0.017
80 H	-0.075	-1.408	-0.484	-0.100	-1.252	-0.352	-0.111	-1.339	-0.450	0.034

81 H	-0.075	-1.401	-0.476	-0.099	-1.248	-0.347	-0.111	-1.335	-0.446	0.031
82 H	-0.075	-1.448	-0.523	-0.105	-1.260	-0.364	-0.112	-1.369	-0.481	0.042
83 H	-0.075	-1.506	-0.581	-0.104	-1.292	-0.396	-0.111	-1.404	-0.516	0.066
84 H	-0.075	-1.516	-0.591	-0.104	-1.297	-0.401	-0.110	-1.407	-0.517	0.074
85 H	-0.075	-1.493	-0.568	-0.103	-1.281	-0.384	-0.112	-1.394	-0.506	0.062
86 H	-0.081	-1.562	-0.643	-0.099	-1.341	-0.440	-0.101	-1.397	-0.498	0.144
87 O	-2.834	-5.191	-0.025	-3.012	-5.031	-0.043	-2.857	-5.423	-0.280	-0.255
88 H	-0.076	-1.508	-0.583	-0.100	-1.301	-0.400	-0.109	-1.382	-0.491	0.092
89 H	-0.076	-1.422	-0.498	-0.100	-1.260	-0.360	-0.109	-1.327	-0.436	0.061
90 H	-0.076	-1.440	-0.515	-0.099	-1.278	-0.377	-0.109	-1.355	-0.465	0.050
91 H	-0.076	-1.436	-0.511	-0.104	-1.252	-0.356	-0.108	-1.336	-0.444	0.068
92 C	-2.274	-2.237	1.489	-2.472	-2.491	1.037	-2.311	-2.300	1.389	-0.100
93 O	-2.834	-5.263	-0.097	-3.002	-5.101	-0.103	-2.856	-5.506	-0.361	-0.264
94 H	-0.076	-1.444	-0.520	-0.103	-1.260	-0.363	-0.111	-1.365	-0.476	0.044
95 H	-0.076	-1.428	-0.503	-0.101	-1.254	-0.355	-0.110	-1.355	-0.465	0.038
96 H	-0.076	-1.439	-0.515	-0.100	-1.268	-0.368	-0.111	-1.369	-0.480	0.035

Total NetCharge: -0.001 -0.000 -0.000 0.001

(accuracy NumInt/Tails)

Remark: the 'NetTotal' Voronoi charges often do not match the Mulliken and/or Hirshfeld charges very

well. This is caused by the fact that chemically different atoms are not treated in accordance with

their relative sizes. (Voronoi cells are defined by boundary planes halfway between the atoms.)

However, the CHANGES in charge, comparing 'Initial' to 'SCF' for instance, do give a fair indication

of the flow of charge caused by the relaxation from sum-of-fragments to self-consistency.

Warning: the absolute accuracy of the VDD charges obtained using the Fuzzy Cells (Becke) integration

scheme is much poorer than the one obtained with an 'equivalent' Voronoi integration grid.

M U L T I P O L E   D E R I V E D   C H A R G E   A N A L Y S I S

This charge analysis uses the atomic multipoles (obtained from the fitted density) up to some level X,

and reconstructs these multipoles exactly (up to level X) by distributing charges over all atoms.

This is achieved by using Lagrange multipliers and a weight function to keep the multipoles local.

Dummy atoms can be included (by setting INCDUM in MDC-block to 1) to obtain a fractional charge.

This is generally useful and necessary only for small symmetrical molecules, when there are not

enough degrees of freedom to reconstruct the multipoles.

Since the atomic multipoles are reconstructed up to level X,

the molecular multipoles are represented also up to level X.

The recommended level is to reconstruct up to quadrupole : -> MDC-q charges.

See: M. Swart, P.Th. van Duijnen, J.G. Snijders, J.Comput.Chem., (2001), p. 79-88.

Atomic electronic multipole moments from SCF equations (a.u.)

----

atom	charge	dip-x	dip-y	dip-z	quad-xx	quad-xy	quad-xz	quad-yy	quad-yz	quad-zz
1 O	-0.351202	-0.212777	0.037796	0.207016	0.169521	0.012764	0.289963	-0.545615	0.061089	0.376095
2 C	0.362383	-0.061411	0.046416	-0.059449	-0.054049	0.001389	-0.011568	0.003513	0.017301	0.050536
3 C	0.242233	0.032647	-0.067043	-0.092394	-0.062806	-0.011348	-0.010877	0.057638	0.016568	0.005167

4 C 0.180703 0.003690 -0.052435 0.042103 0.025349 -0.003914 0.011107 0.023337 -0.032682 -0.048686

5 O -0.306983 -0.335859 0.181253 0.075259 -0.067404 -0.094002 -0.126707 -0.562549 -0.190940 0.629953

6 N -0.214949 -0.044507 -0.172306 -0.335934 0.505590 -0.084456 -0.146660 0.219394 -0.382035 -0.724984

7 O -0.380029 0.088822 -0.245918 0.187843 -0.633885 -0.082673 0.106336 0.254574 0.273909 0.379312

8 C 0.250708 -0.056493 0.028347 0.040826 0.130981 -0.023807 0.023075 -0.051011 0.095766 -0.079970

9 C 0.228081 0.006413 0.045107 -0.022040 0.021613 -0.005505 -0.024400 -0.025887 0.004932 0.004275

10 C 0.339166 0.035019 -0.031102 -0.069628 -0.017935 -0.128525 0.026999 -0.138876 -0.022625 0.156812

11 O -0.382794 -0.017084 0.217294 0.210969 0.618553 -0.163747 0.134346 -0.323820 0.119715 -0.294732

12 C 0.124236 -0.053028 -0.062526 0.042261 -0.026364 -0.068420 -0.023494 -0.008098 0.006435 0.034462

13 O -0.312168 0.160953 -0.174012 0.154653 -0.286965 -0.152669 -0.186754 -0.275696 0.130012 0.562661

14 C 0.245412 0.066795 0.024815 0.082936 0.102230 -0.002581 -0.008741 -0.051199 0.020834 -0.051031

15 C 0.196168 -0.063256 -0.019361 -0.045188 0.053919 0.013769 0.060056 -0.042068 0.028351 -0.011851

16 O -0.369428 0.256383 -0.140945 -0.206207 0.030141 -0.229231 -0.244132 0.008200 0.058734 -0.038341

17 C 0.227992 0.015256 -0.030006 0.033530 0.010380 0.001812 0.003076 -0.001121 0.023493 -0.009259

18 C 0.229585 0.044821 -0.030529 -0.024932 -0.003663 -0.000357 0.017759 -0.030319 -0.012182 0.033982

19 O -0.319383 0.080544 0.233455 -0.198901 -0.368917 -0.097358 -0.165957 0.165020 0.259847 0.203897

20 C 0.056811 -0.067102 -0.000316 -0.027721 0.322399 0.044117 0.203752 0.378952 0.013566 -0.701351

21 C 0.195047 0.056045 -0.052576 0.068992 0.033724 -0.033561 0.023923 -0.026049 -0.029227 -0.007675

22 C 0.118708 0.036418 -0.027129 -0.035973 0.044784 0.008931 -0.033160 0.001486 0.009809 -0.046270

23 C 0.119711 -0.048112 -0.024487 -0.002886 0.054659 0.005234 0.000141 0.007078 -0.031114 -0.061737

24 C 0.290666 0.016285 0.012101 -0.004833 0.026524 -0.002098 0.001340 0.017760 -0.040030 -0.044284

25 C 0.173007 0.073419 -0.035587 0.046358 -0.012012 0.002544 -0.002202 -0.036577 0.004885 0.048589

26 C 0.164890 0.051133 0.045264 0.022891 -0.010117 -0.004696 0.002368 -0.011697 -0.001381 0.021813

27 C 0.249148 -0.062283 -0.033423 -0.071097 -0.042119 -0.004340 0.005203 0.012910 0.003839 0.029209

28 C 0.234544 -0.014106 0.042738 -0.009699 -0.017828 -0.003623 0.008498 -0.014236 0.013907 0.032063

29 C 0.034980 0.043421 0.025275 0.008154 0.328274 0.052147 0.163239 0.286550 -0.021314 -0.614824

30 C 0.237055 -0.021588 0.025299 -0.028706 -0.028891 -0.005512 -0.013705 0.008810 0.022184 0.020082

31 O -0.372314 -0.262968 -0.167812 0.166071 0.229705 0.251925 -0.160109 -0.187631 -0.154966 -0.042074

32 C 0.254504 -0.101064 0.085777 -0.006265 -0.016504 -0.044101 -0.016867 0.015579 0.003455 0.000924

33 C 0.222617 0.053460 0.004789 0.016324 -0.027578 0.009820 0.017412 0.077843 -0.019642 -0.050265

34 C 0.228060 0.060633 0.035940 -0.001264 0.087319 -0.000592 -0.112931 0.111668 -0.049545 -0.198988

35 C 0.221719 0.009914 0.048321 0.024487 0.008639 -0.018539 0.007540 -0.034284 -0.015459 0.025645

36 C 0.180483 0.057472 -0.040747 0.011078 0.009098 0.008360 -0.007569 -0.005116 0.005753 -0.003982

37 C 0.293815 0.002401 -0.012106 0.009987 0.045034 0.006106 -0.003116 0.009397 -0.037091 -0.054431

38 C 0.368732 0.024224 -0.039687 -0.019851 0.015019 -0.007621 -0.006965 0.013954 0.014316 -0.028973

39 C 0.229699 -0.054433 -0.011940 -0.000311 -0.001462 -0.001210 0.004167 0.019415 0.000444 -0.017953

40 H -0.073289 -0.016101 0.002609 0.011341 0.105302 -0.058341 0.056199 0.042887 -0.011557 -0.148189

41 H -0.056771 0.004784 0.004113 0.003115 -0.082165 0.012872 0.042854 -0.037655 0.066850 0.119820

42 H -0.063986 -0.007841 -0.004347 0.000475 -0.086834 0.007937 0.015343 0.172881 -0.015912 -0.086047

43 H -0.057109 0.001373 0.010009 0.002331 0.039687 0.029602 -0.086426 -0.071862 -0.038166 0.032175

44 H -0.069002 0.001895 -0.009806 -0.003371 0.063627 0.074736 0.045755 -0.001907 0.026698 -0.061721

45 H -0.070407 0.009417 -0.003269 0.005790 -0.104430 -0.004132 -0.002198 -0.079059 -0.000818 0.183489

46 H -0.076733 0.004293 -0.007606 0.009308 0.102379 -0.062120 -0.060373 -0.056366 0.026022 -0.046014

47 H -0.071456 -0.006889 0.000753 0.006674 0.079207 0.068310 0.030244 -0.003653 0.033225 -0.075553

48 H -0.063700 0.003415 -0.002935 0.007788 0.068784 0.068900 0.031166 -0.019598 0.029115 -0.049186

49 H -0.083038 -0.000494 0.011706 0.018067 -0.070982 -0.031698 -0.009917 0.122151 -0.048948 -0.051169

50 H -0.055273 0.002233 -0.011052 0.006903 0.091720 0.008382 0.079164 -0.076251 0.009292 -0.015469

51 H -0.072797 0.003711 -0.006869 -0.002097 0.068924 -0.029218 -0.075562 -0.063734 0.028655 -0.005190

52 H -0.070704 -0.002551 -0.008709 -0.003463 -0.099197 0.004170 -0.002423 -0.002357 -0.079299 0.101554

53 H -0.067637 0.008730 -0.009338 -0.000382 -0.049978 0.024036 -0.011477 0.136910 -0.037613 -0.086932

54 H -0.058019 0.003906 -0.014046 0.000462 -0.057204 -0.002220 -0.038357 -0.078443 -0.023982 0.135647

55 H -0.054166 0.007300 -0.011352 -0.001095 0.052081 0.037377 0.066192 -0.037072 0.052094 -0.015009

56 H -0.057174 -0.008405 0.008706 0.003162 -0.050331 -0.019324 0.035798 -0.057048 -0.032501 0.107380

57 H -0.067339 0.004562 -0.001037 0.001166 -0.007469 -0.031774 -0.068991 -0.015897 0.064746 0.023366

58 H -0.061822 0.003568 0.004246 0.007912 -0.064263 -0.005143 0.059161 -0.054990 -0.042884 0.119253

59 H -0.075303 0.006568 0.002458 -0.002556 -0.016820 -0.038913 -0.066030 -0.027029 0.073965 0.043849

60 H -0.074691 0.004739 -0.000472 0.005849 -0.047781 -0.011325 0.056485 -0.072080 -0.052727 0.119861

61 H -0.068778 0.004288 0.001423 0.000841 -0.073378 0.027175 -0.000667 0.178958 -0.017554 -0.105580

62 H -0.067049 -0.001279 0.001050 0.011125 -0.078365 0.027973 -0.013196 0.175511 0.009946 -0.097146

63 H -0.072458 0.002168 -0.004172 0.002726 -0.036726 -0.038950 0.049702 -0.071626 -0.063242 0.108352

64 H -0.058364 0.003426 -0.001943 0.011864 0.166640 0.047385 0.011802 -0.098334 0.015221 -0.068306

65 H -0.074901 0.009194 0.001283 0.005326 0.003393 -0.014118 -0.068004 -0.035116 0.038386 0.031724

66 H -0.072186 0.002300 0.002866 -0.006528 -0.012072 -0.029185 -0.070065 -0.059585 0.041575 0.071657

67 H -0.067313 0.001747 0.006222 -0.004022 0.164203 0.004473 0.037685 -0.080586 0.006191 -0.083617

68 H -0.064917 0.005418 -0.001493 -0.007859 -0.082802 0.042262 0.016162 0.145352 0.040114 -0.062550

69 C 0.353050 -0.027757 0.049129 0.064293 -0.203444 0.075945 -0.109337 0.113965 0.004582 0.089478

70 H -0.064712 -0.002674 -0.007763 -0.005171 -0.001325 0.058756 -0.062437 0.024344 -0.056980 -0.023020

71 H -0.068083 0.000431 -0.001286 -0.002631 -0.099039 -0.003915 0.029129 -0.070814 0.035669 0.169854

72 H -0.063237 0.002433 -0.005575 -0.006795 0.159301 -0.033451 0.041525 -0.070372 -0.001652 -0.088929

73 H -0.080881 0.002519 0.010491 -0.015158 0.006702 0.051158 -0.041891 0.002674 -0.034586 -0.009377

74 H -0.072847 0.000047 -0.005647 -0.001177 0.031784 0.050713 -0.057609 0.022441 -0.050578 -0.054225

75 H -0.069572 -0.005465 -0.001008 -0.005321 -0.096798 0.000732 0.019909 -0.074948 0.019909 0.171746

76 H -0.076017 -0.012027 0.005884 0.008939 -0.072724 0.016866 0.005725 -0.045543 0.065698 0.118266

77 H -0.066525 -0.005378 -0.002808 0.001499 -0.054150 -0.036226 -0.009043 0.159468 -0.021437 -0.105318

78 H -0.065117 -0.006873 -0.002018 0.003045 0.123434 -0.007305 0.066731 -0.088149 -0.026151 -0.035286

79 H -0.082619 -0.010418 0.018204 0.008842 -0.105264 0.002857 0.020030 -0.006707 0.067370 0.111971

80 H -0.066881 0.000187 0.008405 -0.005253 0.043478 0.024176 -0.079634 -0.041787 -0.048375 -0.001691

81 H -0.067914 -0.005164 0.008413 -0.002580 0.120197 -0.005422 0.073660 -0.060774 -0.017573 -0.059423

82 H -0.071281 0.001588 0.006394 -0.014120 0.001718 0.050702 -0.037893 0.009238 -0.028640 -0.010955

83 H -0.061877 0.001668 0.003454 -0.001524 0.022930 -0.037873 -0.047337 -0.044223 0.038147 0.021293

84 H -0.047187 -0.000410 -0.002977 -0.013170 -0.002009 0.046681 -0.045724 0.003191 -0.043855 -0.001182

85 H -0.073569 0.014702 -0.001664 -0.002093 0.000994 0.037754 -0.036318 0.012899 -0.055637 -0.013893

86 H 0.062000 -0.011390 -0.010212 0.046999 0.002184 -0.016216 -0.038189 -0.088945 -0.005792 0.086760

87 O -0.364515 -0.132073 0.176632 -0.220103 0.193967 -0.227775 0.181211 -0.076185 -0.126009 -0.117782

88 H -0.045070 -0.010398 -0.003468 0.012255 -0.014231 0.054699 -0.050820 0.030184 -0.059113 -0.015953

89 H -0.045139 0.006507 0.002758 -0.014346 0.048880 -0.037210 -0.090220 -0.058449 0.034687 0.009568

90 H -0.051992 -0.005159 0.001505 0.008215 -0.110331 0.007094 -0.011854 0.025540 -0.081712 0.084791

91 H -0.042726 -0.011241 -0.002445 -0.009506 0.116474 0.006995 0.083995 -0.087399 0.011956 -0.029076

92 C 0.241065 -0.023618 -0.029213 -0.027012 -0.045951 0.004769 -0.035322 0.004721 -0.003697 0.041229

93 O -0.362980 -0.177468 -0.221987 0.199682 -0.121816 0.182483 -0.123809 0.070815 -0.249205 0.051001

94 H -0.061310 0.006003 0.006297 0.001948 -0.029983 0.057002 -0.052358 0.047502 -0.063914 -0.017518

95 H -0.060948 0.005247 0.005834 0.005969 0.041855 -0.062506 -0.059502 -0.023325 0.043780 -0.018531

96 H -0.068343 0.006829 0.000344 -0.001791 -0.043912 0.047035 0.050059 0.046462 0.074523 -0.002549

Multipole derived atomic charges (a.u.)

The MDC-m charges are just the Monopole terms in the multipole expansion, while for the MDC-d charges

also the Dipoles are reconstructed. The usually preferred charges are the MDC-q charges.

These reconstruct the Monopoles, Dipoles and Quadrupoles (both atomic AND molecular).

Atom		Level: MDC-m	MDC-d	MDC-q
1	O	-0.351202	-0.318937	-0.343667
2	C	0.362383	0.374299	0.395953
3	C	0.242233	0.250118	0.253355
4	C	0.180703	0.166739	0.212811
5	O	-0.306983	-0.548080	-0.609024
6	N	-0.214949	-0.280178	-0.475290
7	O	-0.380029	-0.386031	-0.450762
8	C	0.250708	0.309625	0.308431
9	C	0.228081	0.193461	0.187589
10	C	0.339166	0.409066	0.415940
11	O	-0.382794	-0.354486	-0.394825
12	C	0.124236	0.102364	0.101978
13	O	-0.312168	-0.298403	-0.342923
14	C	0.245412	0.242013	0.247619
15	C	0.196168	0.159174	0.174613
16	O	-0.369428	-0.477722	-0.483035
17	C	0.227992	0.193255	0.199046
18	C	0.229585	0.193142	0.192548
19	O	-0.319383	-0.320803	-0.373519
20	C	0.056811	0.044822	-0.060429
21	C	0.195047	0.220726	0.283721
22	C	0.118708	0.104784	0.107476

23	C	0.119711	0.099398	0.110717
24	C	0.290666	0.268667	0.286483
25	C	0.173007	0.135238	0.137495
26	C	0.164890	0.124936	0.164550
27	C	0.249148	0.239454	0.263262
28	C	0.234544	0.193940	0.303723
29	C	0.034980	0.028271	-0.043037
30	C	0.237055	0.191349	0.177373
31	O	-0.372314	-0.476801	-0.517817
32	C	0.254504	0.243389	0.263506
33	C	0.222617	0.185994	0.199147
34	C	0.228060	0.295672	0.386933
35	C	0.221719	0.192155	0.201515
36	C	0.180483	0.145658	0.151658
37	C	0.293815	0.271404	0.310761
38	C	0.368732	0.339916	0.387940
39	C	0.229699	0.190448	0.156321
40	H	-0.073289	-0.060629	-0.026855
41	H	-0.056771	-0.031497	-0.019607
42	H	-0.063986	-0.056104	-0.034454
43	H	-0.057109	-0.036634	-0.019665
44	H	-0.069002	-0.052720	-0.050834
45	H	-0.070407	-0.057557	-0.052966
46	H	-0.076733	-0.072073	-0.070446
47	H	-0.071456	-0.061509	-0.062497
48	H	-0.063700	-0.041760	-0.023360
49	H	-0.083038	-0.063517	-0.071147
50	H	-0.055273	-0.047008	-0.049619
51	H	-0.072797	-0.055559	-0.060316
52	H	-0.070704	-0.058579	-0.064983
53	H	-0.067637	-0.063591	-0.073602

54	H	-0.058019	-0.045413	-0.052201
55	H	-0.054166	-0.044104	-0.046449
56	H	-0.057174	-0.042199	-0.030960
57	H	-0.067339	-0.050537	-0.052543
58	H	-0.061822	-0.036820	-0.032763
59	H	-0.075303	-0.060469	-0.049813
60	H	-0.074691	-0.065321	-0.055617
61	H	-0.068778	-0.054883	-0.042938
62	H	-0.067049	-0.048711	-0.039560
63	H	-0.072458	-0.074559	-0.082054
64	H	-0.058364	-0.036374	-0.033439
65	H	-0.074901	-0.049608	-0.058536
66	H	-0.072186	-0.063641	-0.060618
67	H	-0.067313	-0.053825	-0.066984
68	H	-0.064917	-0.056072	-0.055580
69	C	0.353050	0.438124	0.460716
70	H	-0.064712	-0.054313	-0.082585
71	H	-0.068083	-0.067056	-0.097466
72	H	-0.063237	-0.048468	-0.074374
73	H	-0.080881	-0.062394	-0.073079
74	H	-0.072847	-0.059951	-0.063482
75	H	-0.069572	-0.068853	-0.073350
76	H	-0.076017	-0.045943	-0.040099
77	H	-0.066525	-0.054398	-0.038387
78	H	-0.065117	-0.060768	-0.028782
79	H	-0.082619	-0.063656	-0.046111
80	H	-0.066881	-0.058245	-0.051651
81	H	-0.067914	-0.066568	-0.055429
82	H	-0.071281	-0.044301	-0.037762
83	H	-0.061877	-0.026392	-0.019140
84	H	-0.047187	-0.002410	-0.015770

85	H	-0.073569	-0.059849	-0.041089
86	H	0.062000	0.261882	0.290730
87	O	-0.364515	-0.442751	-0.458203
88	H	-0.045070	0.000499	0.021173
89	H	-0.045139	-0.029575	-0.015338
90	H	-0.051992	-0.049947	-0.038372
91	H	-0.042726	-0.010633	-0.009928
92	C	0.241065	0.197785	0.180088
93	O	-0.362980	-0.462072	-0.489882
94	H	-0.061310	-0.053771	-0.049822
95	H	-0.060948	-0.047334	-0.038866
96	H	-0.068343	-0.065405	-0.061473

Average absolute deviations in atomic multipoles

Stated here are the average differences between the atomic multipoles
and the reconstructed atomic multipoles (from the distributed charges).
If these values are not zero, this means there are not enough degrees of freedom,
to be able to reconstruct the atomic multipoles. (This usually happens only
for small and/or highly symmetric molecules). If this is the case, one could add
dummy atoms as extra point charges (and setting INCDUM in MDC-block to 1).

Level:	MDC-d	MDC-q
Charge (a.u.)	0.0000	0.0000
Dipole (Debye)	0.0000	0.0000
Quad. (a.u.)	0.0784	0.0000

Represented molecular multipole moments

Given here are the Molecular multipole moments from the atomic charges, and from the Fit Density.

Note that the atomic charges represent the latter, NOT the ones from the Exact density.

	Q (a.u.)	Dipole moment (Debye)			Quadrupole moment (a.u.)					
		x	y	z	xx	xy	xz	yy	yz	zz
MDC-m	0.0000	-2.1303	-1.0483	1.2654	-57.6161	-92.5855	-18.5681	-51.1990	35.8327	108.8151
MDC-d	0.0000	-3.5125	-1.9820	2.0107	-77.6477	-154.4832	-44.3983	-89.0717	33.9824	166.7194
MDC-q	0.0000	-3.5125	-1.9820	2.0107	-75.8272	-154.8439	-43.7457	-89.8013	35.0447	165.6285
Fit.Dens.	0.0000	-3.5125	-1.9820	2.0107	-75.8272	-155.0628	-44.0222	-89.8013	34.8883	165.6285

Dipole Moment *** (Debye) ***

Vector : -3.50815438 -1.98321470 2.01013280

Magnitude: 4.50343442

This molecular dipole moment is calculated with analytic integration

Quadrupole Moment (Buckingham convention) *** (a.u.) ***

quad-xx	quad-xy	quad-xz	quad-yy	quad-yz	quad-zz
-75.73654108	-155.15442810	-44.12833096	-89.88877862	34.71769748	165.62531970

This molecular quadrupole moment is calculated with analytic integration

1

S F O P O P U L A T I O N S , M O A N A L Y S I S

A Mulliken population analysis is performed on (input-)selected MOs. All populations refer to SFOs.

BAS populations may have been printed directly after the SCF part.

=== A ===

SFO contributions (%) per orbital

(multiplication by the orbital occupation yields the SFO Gross Populations)

Orb.:	114	115	116	117	118	119	120	121	122	123	124	125	126	127
occup:	2.00	2.00	2.00	2.00	2.00	2.00	2.00	2.00	2.00	2.00	0.00	0.00	0.00	0.00
CF+SFO	----	----	----	----	----	----	----	----	----	----	----	----	----	----
------														
50:	45.64	0.60	0.03	0.06	0.21	1.85	0.00	8.57	0.00	0.04	0.15	0.01	0.02	-0.00

71:	0.13	3.58	4.72	9.70	0.00	0.69	3.76	0.15	0.24	0.00	0.00	0.11	0.00	0.00
74:	0.06	7.99	7.34	20.90	0.02	2.99	14.31	0.62	1.10	0.00	0.07	0.66	0.19	0.02
77:	0.01	1.44	1.27	2.31	0.00	0.12	0.21	0.03	0.08	-0.00	0.01	0.23	0.10	0.00
95:	0.94	0.37	0.48	0.50	52.87	0.64	0.03	0.44	0.01	0.09	0.00	0.00	0.00	0.14
98:	3.05	0.03	0.02	0.02	1.39	0.12	0.00	0.66	0.00	0.00	-0.00	0.00	0.00	0.28
101:	0.24	0.02	0.02	0.01	1.75	-0.00	0.00	-0.00	-0.00	0.02	0.00	0.00	0.00	0.00
119:	1.20	0.05	0.06	0.00	0.00	2.88	0.15	0.74	0.00	-0.00	0.09	0.00	0.00	0.00
122:	2.75	0.14	0.23	0.00	0.00	15.86	1.00	5.33	0.01	0.02	0.79	0.03	0.02	0.00
125:	5.02	0.31	0.24	0.00	0.04	20.89	0.83	5.76	0.04	0.08	0.40	0.04	0.00	0.00
143:	0.06	3.05	0.23	0.48	0.01	0.19	0.10	0.41	0.01	0.00	0.04	0.27	5.04	0.02
146:	0.16	0.97	0.07	0.07	0.01	0.00	0.20	0.03	0.02	0.00	0.04	0.31	5.17	0.01
149:	0.09	5.48	0.12	0.22	0.03	0.00	0.19	0.02	0.01	-0.00	0.01	0.00	0.00	0.00
167:	2.47	0.01	0.04	0.07	1.09	7.41	2.86	19.61	0.01	0.00	-0.00	0.00	0.04	0.00
170:	1.19	0.03	0.03	0.05	0.58	6.23	1.71	6.08	0.00	0.00	14.09	0.68	0.72	0.00
171:	-0.01	0.00	0.00	-0.00	-0.01	-0.03	-0.01	-0.05	-0.00	0.00	-1.07	-0.07	0.04	0.00
173:	0.44	0.08	0.01	0.01	0.71	1.30	1.19	14.41	0.01	0.00	12.18	0.75	0.18	0.00
174:	0.00	-0.00	-0.00	0.00	-0.01	0.00	-0.00	-0.11	-0.00	-0.00	-1.14	-0.06	-0.01	-0.00
191:	0.25	0.02	0.74	0.66	3.21	0.17	0.01	0.34	0.00	0.19	0.01	0.00	0.03	6.84
197:	0.02	0.00	4.21	2.62	0.93	0.02	0.14	0.04	0.00	0.40	0.00	0.00	0.00	1.59
215:	0.01	0.02	1.43	1.43	0.00	0.00	0.29	0.02	15.74	0.00	0.25	4.05	0.09	0.01
218:	0.02	0.10	0.07	0.42	0.01	0.07	2.33	0.04	47.26	0.00	0.04	0.93	0.06	0.00
221:	0.09	0.03	0.40	0.09	0.00	0.01	3.21	0.16	1.07	0.00	0.75	15.29	0.39	0.03
222:	-0.00	-0.00	-0.00	0.00	0.00	0.00	-0.03	-0.00	-0.00	-0.00	-0.06	-1.25	-0.03	-0.01
239:	0.01	10.79	0.23	0.40	0.07	0.05	0.57	0.23	0.02	0.00	0.13	0.39	7.90	0.07
242:	0.48	4.18	-0.00	-0.00	0.00	0.06	0.01	0.26	0.01	0.00	0.43	0.50	13.45	0.06
243:	-0.01	-0.05	0.00	0.00	-0.00	-0.00	-0.00	-0.00	0.00	-0.00	-0.06	-0.08	-1.30	-0.01
245:	0.92	37.47	0.37	0.67	0.11	0.01	0.71	0.01	0.00	0.00	0.10	-0.00	0.08	-0.00
263:	0.02	0.00	1.32	0.48	1.76	0.01	0.05	0.04	0.00	0.08	-0.00	0.00	0.06	20.27
264:	-0.00	0.00	0.00	0.00	-0.02	-0.00	-0.00	0.00	-0.00	0.01	-0.00	-0.00	-0.00	-1.89
266:	0.21	0.00	22.18	13.42	0.67	0.13	0.60	0.00	0.01	0.19	0.00	0.00	0.01	1.28
269:	0.23	0.00	19.97	14.52	0.00	0.34	0.74	0.04	0.01	0.08	0.00	0.00	0.01	4.44

287:	0.04	0.00	-0.01	-0.01	1.71	0.07	0.01	0.08	0.00	0.42	0.00	0.00	0.01	0.37
288:	0.03	0.00	-0.02	-0.01	1.04	0.06	0.00	-0.00	0.00	0.11	0.00	0.01	0.01	-0.09
311:	0.18	0.01	0.00	-0.00	4.19	0.28	0.03	0.00	0.00	0.39	-0.00	0.00	0.00	0.03
317:	0.00	0.00	0.02	0.02	0.28	0.00	-0.00	0.01	0.00	3.64	-0.00	-0.00	-0.00	0.06
356:	0.02	0.03	0.00	0.00	0.01	-0.01	0.00	0.05	0.00	0.00	0.13	0.05	1.46	0.01
362:	0.00	-0.00	0.00	-0.00	0.00	0.33	0.06	0.05	0.00	0.00	27.22	1.26	1.69	-0.00
363:	-0.01	-0.03	0.00	-0.00	0.00	0.02	0.01	0.18	0.00	0.00	-1.53	-0.04	-0.38	-0.00
365:	0.00	-0.00	0.00	0.00	0.01	0.01	0.01	0.55	-0.00	0.00	22.05	1.33	0.03	0.00
407:	0.00	0.24	0.02	0.05	0.02	0.06	0.16	0.28	0.01	-0.00	0.39	0.92	19.44	0.15
408:	0.01	0.09	0.04	0.04	0.00	0.00	-0.01	0.01	0.00	-0.00	0.04	0.10	-1.67	0.03
410:	0.02	0.17	0.01	0.03	0.00	0.02	-0.00	-0.01	0.01	0.00	0.48	0.80	30.91	0.06
411:	0.02	0.04	-0.00	-0.01	0.00	-0.00	0.01	-0.00	-0.00	-0.00	-0.13	-0.03	-1.59	-0.01
416:	0.02	1.03	0.01	0.02	0.01	0.01	0.03	0.02	0.00	0.00	0.01	0.00	0.00	0.00
431:	0.04	2.52	0.03	0.05	0.02	0.33	0.06	1.58	-0.00	0.00	-0.03	0.02	0.07	0.00
434:	0.00	0.67	0.02	0.02	0.12	0.69	0.48	2.79	0.00	0.00	0.27	-0.00	0.90	0.02
437:	0.00	0.36	0.02	0.03	0.12	0.59	0.36	2.48	0.00	0.00	0.46	0.03	-0.01	-0.00
458:	1.70	0.02	0.00	0.00	0.00	0.88	0.01	1.77	0.02	0.00	1.24	0.00	0.14	-0.00
459:	1.08	0.05	0.00	0.00	0.03	0.14	0.00	0.25	-0.00	-0.00	-0.22	-0.01	-0.07	-0.01
461:	0.58	0.03	-0.00	0.02	0.00	4.18	0.38	0.40	0.09	0.05	-0.11	0.05	-0.02	-0.00
482:	1.06	0.02	-0.00	0.00	0.00	0.82	0.14	0.44	0.04	0.02	0.00	0.03	0.02	0.00
485:	0.11	0.00	-0.00	0.00	-0.00	1.59	0.03	0.29	0.21	0.02	-0.00	0.14	-0.00	0.01
509:	-0.00	0.12	0.01	0.01	0.02	0.05	0.04	0.35	0.00	0.00	2.54	0.07	-0.01	0.01
551:	-0.00	0.01	0.73	0.64	0.00	0.21	2.24	0.05	0.04	0.00	0.18	3.76	0.01	-0.00
554:	0.01	0.62	1.35	2.84	0.00	0.17	0.48	0.02	0.56	0.00	0.00	0.05	0.09	-0.00
557:	0.01	0.15	6.91	4.21	0.01	1.85	22.36	0.58	0.09	0.00	1.11	18.44	0.62	0.00
599:	0.33	0.09	0.00	0.01	0.18	1.16	0.50	6.00	0.00	0.00	-0.00	0.04	-0.04	0.03
602:	5.58	0.12	0.00	0.02	-0.00	0.50	0.01	0.83	0.00	0.00	0.39	-0.00	0.03	-0.01
623:	0.01	-0.00	0.09	0.05	0.00	0.03	0.01	-0.00	6.31	0.00	0.00	0.20	-0.00	-0.01
626:	0.14	0.01	0.06	0.06	0.00	0.03	0.00	0.02	1.39	-0.00	0.00	0.01	-0.00	-0.00
629:	0.00	0.01	0.14	0.20	0.00	0.19	0.02	0.00	1.93	0.00	0.00	0.08	-0.00	0.00
671:	0.54	0.03	0.03	0.00	0.03	1.25	0.05	0.18	0.01	0.00	-0.00	0.38	0.00	-0.00

674:	1.00	-0.00	0.10	0.04	0.00	-0.02	0.38	0.00	-0.00	0.00	0.02	-0.00	0.00	0.01
695:	0.16	0.04	0.08	0.07	3.12	0.04	0.00	0.01	0.00	-0.00	-0.00	-0.00	-0.00	-0.01
698:	0.01	0.00	0.10	0.06	0.36	0.02	-0.00	0.00	0.00	1.50	0.00	-0.00	0.00	0.01
719:	0.11	0.03	0.01	0.02	1.34	0.03	0.00	0.00	0.00	0.19	-0.00	-0.00	-0.00	-0.00
764:	-0.00	-0.00	0.04	0.14	0.00	0.00	0.01	0.00	1.26	0.00	0.00	0.01	-0.00	-0.01
767:	-0.00	0.04	0.78	0.21	0.00	0.10	1.30	0.03	0.14	0.00	0.02	0.32	0.02	0.00
770:	0.01	0.01	0.12	0.02	0.00	0.01	0.13	0.01	7.22	-0.00	0.01	0.11	-0.00	-0.00
773:	0.01	0.02	5.32	2.48	0.01	1.62	18.83	0.48	0.01	0.00	0.66	10.60	0.24	0.02
774:	-0.00	0.00	0.02	-0.01	0.00	0.01	0.15	0.01	0.03	-0.00	-0.07	-1.46	-0.03	-0.05
791:	0.03	1.13	0.00	0.01	0.01	0.05	0.01	0.55	0.00	0.00	1.03	0.08	0.02	-0.00
815:	0.03	0.30	0.39	1.17	0.00	0.34	1.35	0.16	0.29	0.00	-0.00	0.38	0.20	0.00
845:	0.00	0.00	0.16	1.02	0.00	0.12	1.15	0.03	0.02	0.00	0.00	0.02	0.00	0.01
863:	0.01	0.00	-0.00	0.00	0.00	0.00	0.01	0.00	0.51	-0.00	0.29	4.78	0.13	0.01
866:	0.00	0.02	0.02	0.07	-0.00	0.01	0.06	0.00	1.18	0.00	0.01	0.38	0.02	0.00
867:	0.00	-0.00	-0.00	-0.01	0.00	-0.00	0.01	0.01	1.11	-0.00	0.01	-0.03	0.02	-0.01
869:	0.00	-0.00	-0.01	-0.00	0.00	0.01	0.09	0.01	0.00	0.00	1.15	23.20	0.61	0.05
873:	0.00	0.00	0.01	0.03	0.00	0.00	0.01	0.00	1.43	0.00	0.00	0.01	0.00	0.00
890:	0.42	-0.00	0.00	-0.00	-0.00	1.03	0.23	0.60	0.01	0.00	0.07	0.04	0.01	-0.00
1007:	-0.00	0.00	0.11	0.05	0.03	0.00	0.00	0.00	0.00	0.06	-0.00	0.00	0.09	41.15
1008:	0.02	0.00	-0.00	0.00	-0.00	0.00	-0.00	0.00	-0.00	0.02	-0.01	-0.00	-0.01	-2.65
1010:	0.00	-0.00	0.06	-0.00	0.00	-0.00	0.00	-0.00	-0.00	0.05	0.00	0.00	0.01	3.62
1013:	-0.00	0.00	0.09	0.10	0.05	0.00	0.01	0.00	0.00	0.00	0.00	0.00	0.02	9.13
1034:	0.01	0.00	3.12	2.09	0.02	0.03	0.10	0.00	0.00	0.04	0.00	0.00	0.00	0.18
1052:	0.02	0.00	0.02	0.01	0.23	0.04	0.01	0.00	0.00	3.89	0.00	0.00	-0.00	0.00
1055:	0.11	0.01	0.01	0.01	0.31	0.03	0.00	0.00	0.00	1.53	0.00	0.00	0.00	-0.00
1058:	0.02	0.00	0.04	0.02	0.38	0.06	0.02	-0.00	0.00	11.97	0.00	0.00	-0.00	0.00
1061:	0.00	0.00	0.18	0.10	0.27	0.08	0.04	0.00	0.00	49.37	0.00	0.00	0.00	0.00
1087:	0.00	0.01	0.43	0.31	0.00	0.21	2.06	0.05	0.05	-0.00	0.03	0.35	0.03	0.06
1109:	0.00	0.03	0.98	1.18	0.00	0.11	1.60	0.04	-0.03	0.00	0.06	0.91	0.01	0.01
1219:	1.27	0.04	0.05	0.00	0.00	2.93	0.17	0.92	0.00	0.00	0.44	0.01	0.03	0.00
1230:	1.33	0.08	0.05	0.00	0.00	3.61	0.15	1.00	0.01	0.01	-0.00	0.01	0.01	-0.00

1351:	1.21	-0.02	-0.01	-0.00	0.00	0.01	0.06	0.35	0.01	0.00	0.09	0.06	-0.16	-0.09
1373:	0.14	0.11	0.00	-0.00	-0.04	0.12	0.05	0.63	-0.00	-0.00	1.23	0.09	0.02	0.00
1428:	0.02	0.03	0.01	0.02	4.24	0.01	0.00	0.05	0.00	0.04	-0.00	0.00	0.00	-0.00
1450:	-0.00	0.00	0.00	0.00	1.28	0.02	0.00	0.03	0.00	0.15	-0.00	0.00	0.00	0.00
1461:	0.00	0.00	0.04	0.02	0.10	0.02	0.01	-0.00	0.00	7.11	0.00	0.00	-0.00	0.07
1483:	0.01	0.00	0.00	0.00	0.02	0.00	0.00	0.00	0.00	1.12	0.00	0.00	0.00	-0.00
1494:	0.01	0.00	0.03	0.01	0.08	0.02	0.01	-0.00	0.00	7.76	-0.00	-0.00	0.00	0.16
1516:	0.00	0.00	0.01	0.00	0.09	-0.00	0.00	0.00	-0.00	1.39	-0.00	0.00	-0.00	0.08
1527:	1.44	0.00	0.02	0.01	6.26	0.04	0.01	0.88	0.00	0.00	0.15	-0.00	0.03	-0.00
1538:	2.75	0.02	0.01	0.01	0.05	0.17	0.01	-0.05	0.11	-0.00	0.01	0.09	0.12	0.02
1582:	0.02	0.02	0.00	0.02	-0.00	0.11	0.02	0.02	0.00	0.00	5.50	-0.00	9.69	0.09
1593:	0.00	-0.00	-0.03	-0.03	0.00	0.00	-0.00	0.00	0.00	0.03	0.01	-0.00	-0.02	2.14
1594:	-0.00	0.00	-0.02	-0.01	0.00	0.00	-0.00	-0.00	0.00	0.01	0.01	-0.00	-0.03	-1.08
1604:	0.03	0.00	1.03	0.71	0.03	0.00	0.01	-0.00	0.00	0.01	0.01	0.01	-0.02	1.55
1615:	0.01	0.00	0.11	0.04	0.02	0.00	-0.00	0.00	0.00	-0.00	-0.01	-0.02	0.32	9.80
1616:	0.00	0.00	0.03	-0.06	-0.01	0.00	-0.00	-0.00	0.00	0.01	-0.04	-0.03	0.19	-1.21
1637:	0.02	1.53	0.03	0.05	0.01	-0.00	0.07	0.01	0.00	-0.00	-0.30	-0.01	-0.02	-0.00

Summation over all MOs, multiplied by occupation: Total SFO Gross Populations in this Irrep

===
=========

0.00	0.00	0.00	0.00	0.00	0.00	0.00	0.00	0.00	0.00	0.00	0.00
0.00	0.00	0.00	0.00	0.00	0.00	0.00	0.00	0.00	0.00	0.00	0.00
0.00	0.00	0.00	0.00	0.00	0.00	0.00	0.00	0.00	0.00	0.00	0.00
0.00	0.00	0.00	0.00	0.00	0.00	0.00	1.88	-0.01	-0.00	1.47	-0.02
0.00	1.88	-0.01	0.00	1.41	-0.02	0.00	0.02	0.01	0.01	0.02	0.01
0.00	0.00	0.00	0.00	0.00	0.00	0.00	1.84	-0.01	-0.00	1.65	-0.01
0.00	1.77	-0.01	0.00	1.30	-0.02	-0.00	0.01	0.01	0.01	0.02	0.01
0.00	0.00	0.00	0.00	0.00	0.00	0.00	1.88	-0.01	-0.00	1.87	-0.01

0.00	1.44	-0.02	0.00	1.41	-0.02	0.00	0.02	0.01	0.01	0.01	0.01
0.00	0.00	0.00	0.00	0.00	0.00	0.00	1.88	-0.01	-0.00	1.30	-0.02
0.00	1.72	-0.01	0.00	1.73	-0.01	0.00	0.01	0.02	0.02	0.02	0.01
0.00	0.00	0.00	0.00	0.00	0.00	0.00	1.87	-0.01	-0.00	1.68	-0.01
0.00	1.66	-0.01	0.00	1.35	-0.03	0.00	0.02	0.01	0.01	0.02	0.02
0.00	0.00	0.00	0.00	0.00	0.00	0.00	1.88	-0.00	-0.00	1.64	-0.00
-0.00	1.50	-0.00	0.00	1.52	-0.00	-0.00	0.01	0.01	0.01	0.01	0.01
0.00	0.00	0.00	0.00	0.00	0.00	0.00	1.88	-0.01	-0.00	1.75	-0.01
0.00	1.48	-0.02	0.00	1.48	-0.02	0.00	0.02	0.01	0.01	0.01	0.02
0.00	0.00	0.00	0.00	0.00	0.00	0.00	1.88	-0.00	-0.00	1.51	-0.00
-0.00	1.74	-0.00	-0.00	1.38	-0.00	-0.00	0.01	0.01	0.01	0.01	0.01
0.00	0.00	0.00	0.00	0.00	0.00	0.00	1.89	-0.01	-0.00	1.52	-0.00
-0.00	1.50	-0.00	0.00	1.72	0.00	-0.00	0.01	0.01	0.01	0.01	0.01
0.00	0.00	0.00	0.00	0.00	0.00	0.00	1.88	-0.00	0.00	1.45	-0.00
0.00	1.61	-0.00	-0.00	1.62	0.00	-0.00	0.01	0.01	0.01	0.01	0.01
0.00	0.00	0.00	0.00	0.00	0.00	0.00	0.97	-0.01	-0.00	0.72	0.03
-0.01	0.56	0.08	-0.01	0.53	0.10	-0.01	0.03	0.02	0.02	0.02	0.03
0.00	0.00	0.00	0.00	0.00	0.00	0.00	1.15	-0.02	0.00	0.80	0.03
-0.01	0.57	0.07	-0.01	0.79	0.00	-0.01	0.02	0.02	0.02	0.01	0.02
0.00	0.00	0.00	0.00	0.00	0.00	0.00	1.19	-0.02	0.00	0.75	0.04
-0.01	0.82	-0.00	-0.01	0.79	0.03	-0.01	0.02	0.02	0.02	0.02	0.01
0.00	0.00	0.00	0.00	0.00	0.00	0.00	1.15	-0.00	0.00	0.64	0.07
-0.01	0.66	0.03	-0.01	0.64	0.03	-0.01	0.02	0.03	0.02	0.02	0.02
0.00	0.00	0.00	0.00	0.00	0.00	0.00	0.96	-0.02	0.00	0.80	0.00
-0.01	0.79	0.01	-0.01	0.81	0.01	-0.01	0.01	0.01	0.01	0.00	0.01
0.00	0.00	0.00	0.00	0.00	0.00	0.00	0.95	0.00	-0.00	0.66	0.06
-0.01	0.68	0.04	-0.01	0.47	0.12	-0.02	0.05	0.02	0.02	0.04	0.03
0.00	0.00	0.00	0.00	0.00	0.00	0.00	1.28	-0.02	0.00	0.85	0.03
-0.01	0.89	0.03	-0.01	0.87	0.01	-0.01	0.01	0.01	0.02	0.01	0.02
0.00	0.00	0.00	0.00	0.00	0.00	0.00	1.15	-0.02	0.00	0.58	0.06
-0.01	0.74	0.01	-0.01	0.80	0.02	-0.01	0.01	0.02	0.02	0.02	0.02

0.00	0.00	0.00	0.00	0.00	0.00	0.00	1.29	-0.01	0.00	0.65	0.04
-0.01	0.80	0.01	-0.01	0.75	0.06	-0.01	0.02	0.02	0.02	0.02	0.02
0.00	0.00	0.00	0.00	0.00	0.00	0.00	0.97	-0.02	0.00	0.83	0.01
-0.01	0.80	0.02	-0.01	0.77	0.01	-0.01	0.01	0.01	0.00	0.01	0.01
0.00	0.00	0.00	0.00	0.00	0.00	0.00	0.97	-0.02	0.00	0.82	0.00
-0.01	0.80	0.01	-0.01	0.79	0.02	-0.01	0.01	0.01	0.00	0.01	0.01
0.00	0.00	0.00	0.00	0.00	0.00	0.00	1.21	-0.02	0.00	0.79	0.04
-0.01	0.71	0.00	-0.01	0.98	-0.00	-0.00	0.00	0.02	0.02	0.01	0.01
0.00	0.00	0.00	0.00	0.00	0.00	0.00	1.27	-0.02	0.00	0.74	0.04
-0.01	0.78	0.04	-0.01	0.67	0.04	-0.01	0.02	0.03	0.02	0.02	0.02
0.00	0.00	0.00	0.00	0.00	0.00	0.00	1.28	-0.02	0.00	0.81	0.03
-0.01	0.83	0.01	-0.01	0.88	0.02	-0.01	0.01	0.02	0.01	0.01	0.01
0.00	0.00	0.00	0.00	0.00	0.00	0.00	1.24	-0.02	0.00	0.86	0.03
-0.01	0.81	0.00	-0.01	0.84	0.00	-0.01	0.02	0.01	0.02	0.01	0.02
0.00	0.00	0.00	0.00	0.00	0.00	0.00	0.89	-0.02	0.00	0.74	0.02
-0.01	0.79	0.00	-0.01	0.78	0.01	-0.01	0.02	0.01	0.01	0.01	0.01
0.00	0.00	0.00	0.00	0.00	0.00	0.00	1.13	-0.02	0.00	0.81	0.01
-0.01	0.88	0.02	-0.01	0.71	0.01	-0.01	0.01	0.01	0.01	0.01	0.01
0.00	0.00	0.00	0.00	0.00	0.00	0.00	1.11	-0.02	0.00	0.77	0.01
-0.01	0.79	0.02	-0.01	0.87	0.01	-0.01	0.01	0.01	0.01	0.01	0.01
0.00	0.00	0.00	0.00	0.00	0.00	0.00	1.11	-0.01	0.00	0.79	0.02
-0.01	0.80	0.01	-0.01	0.58	0.07	-0.01	0.02	0.02	0.02	0.02	0.02
0.00	0.00	0.00	0.00	0.00	0.00	0.00	0.97	-0.02	0.00	0.84	0.00
-0.01	0.79	0.01	-0.01	0.82	0.00	-0.01	0.01	0.01	0.01	0.00	0.01
0.00	0.00	0.00	0.00	0.00	0.00	0.00	1.32	-0.01	0.00	0.77	0.01
-0.01	0.84	0.03	-0.01	0.97	-0.00	-0.00	0.01	0.03	0.02	0.01	0.01
0.00	0.00	0.00	0.00	0.00	0.00	0.00	0.95	-0.02	0.00	0.79	0.01
-0.01	0.81	0.00	-0.01	0.79	0.01	-0.01	0.00	0.00	0.01	0.01	0.01
0.00	0.00	0.00	0.00	0.00	0.00	0.00	1.19	-0.02	0.00	0.71	0.02
-0.01	0.68	0.02	-0.01	0.76	0.03	-0.01	0.02	0.02	0.02	0.01	0.02
0.00	0.00	0.00	0.00	0.00	0.00	0.00	0.97	-0.02	0.00	0.79	0.01

-0.01	0.81	0.02	-0.01	0.85	-0.00	-0.01	0.01	0.01	0.01	0.01	0.00
0.00	0.00	0.00	0.00	0.00	0.00	0.00	1.13	-0.01	0.00	0.67	0.08
-0.01	0.69	0.05	-0.01	0.69	0.02	-0.00	0.01	0.04	0.03	0.02	0.02
0.00	0.00	0.00	0.00	0.00	0.00	0.00	0.96	-0.02	0.00	0.83	0.01
-0.01	0.76	0.01	-0.01	0.79	0.01	-0.01	0.00	0.01	0.00	0.01	0.01
0.00	0.00	0.00	0.00	0.00	0.00	0.00	1.10	-0.01	0.00	0.76	0.02
-0.01	0.78	0.02	-0.01	0.91	0.01	-0.01	0.01	0.02	0.01	0.01	0.01
0.00	0.00	0.00	0.00	0.00	0.00	0.00	0.89	-0.02	0.00	0.82	0.00
-0.01	0.68	0.04	-0.01	0.81	-0.01	-0.01	0.01	0.02	0.01	0.01	0.02
0.00	0.00	0.00	0.00	0.00	0.00	0.00	0.82	-0.01	0.00	0.60	0.04
-0.01	0.70	0.02	-0.01	0.81	0.00	-0.01	0.01	0.02	0.01	0.02	0.01
0.00	0.00	0.00	0.00	0.00	0.00	0.00	0.96	-0.02	0.00	0.79	0.01
-0.01	0.83	0.00	-0.01	0.83	-0.01	-0.01	0.01	0.01	0.01	0.01	0.00
0.00	0.00	0.00	0.00	0.00	0.00	0.00	0.94	0.01	-0.00	0.68	0.03
-0.01	0.64	0.10	-0.01	0.53	0.11	-0.01	0.05	0.03	0.02	0.03	0.03
0.00	0.00	0.00	0.00	0.00	0.00	0.00	0.97	-0.02	0.00	0.87	0.00
-0.01	0.79	0.02	-0.01	0.84	0.01	-0.01	0.00	0.01	0.00	0.01	0.01
0.00	0.00	0.00	0.00	0.00	0.00	0.00	1.55	-0.01	0.00	1.05	-0.02
-0.00	1.18	-0.01	-0.00	1.58	-0.02	0.00	0.01	0.02	0.02	0.02	0.01
0.00	0.00	0.00	0.00	0.00	0.00	0.00	1.12	-0.00	-0.00	0.03	0.03
0.03	0.00	0.00	0.00	0.00	0.00	1.05	0.01	-0.00	0.02	0.03	0.04
0.00	0.00	0.00	0.00	0.00	1.12	-0.01	-0.00	0.03	0.04	0.03	0.00
0.00	0.00	0.00	0.00	1.10	0.01	-0.00	0.03	0.03	0.03	0.00	0.00
0.00	0.00	0.00	1.12	0.00	-0.00	0.03	0.02	0.03	0.00	0.00	0.00
0.00	0.00	1.15	0.01	-0.00	0.03	0.02	0.04	0.00	0.00	0.00	0.00
0.00	1.16	-0.02	-0.01	0.03	0.02	0.03	0.00	0.00	0.00	0.00	0.00
1.06	0.00	-0.00	0.03	0.03	0.02	0.00	0.00	0.00	0.00	0.00	1.09
0.01	-0.00	0.03	0.03	0.03	0.00	0.00	0.00	0.00	0.00	1.04	-0.01
-0.00	0.03	0.04	0.03	0.00	0.00	0.00	0.00	0.00	1.11	0.00	-0.00
0.03	0.02	0.03	0.00	0.00	0.00	0.00	0.00	1.15	-0.00	-0.00	0.04
0.02	0.03	0.00	0.00	0.00	0.00	0.00	1.14	-0.02	-0.00	0.03	0.03

0.04	0.00	0.00	0.00	0.00	0.00	1.16	-0.02	-0.01	0.02	0.04	0.03
0.00	0.00	0.00	0.00	0.00	1.10	-0.01	-0.01	0.02	0.03	0.04	0.00
0.00	0.00	0.00	0.00	1.17	-0.01	-0.00	0.03	0.03	0.03	0.00	0.00
0.00	0.00	0.00	1.09	0.01	-0.00	0.02	0.02	0.03	0.00	0.00	0.00
0.00	0.00	1.10	0.01	-0.00	0.03	0.02	0.03	0.00	0.00	0.00	0.00
0.00	1.06	0.01	-0.00	0.03	0.02	0.03	0.00	0.00	0.00	0.00	0.00
1.12	-0.00	-0.00	0.02	0.03	0.03	0.00	0.00	0.00	0.00	0.00	1.13
-0.00	-0.00	0.02	0.03	0.03	0.00	0.00	0.00	0.00	0.00	1.13	0.00
-0.00	0.02	0.04	0.02	0.00	0.00	0.00	0.00	0.00	1.13	-0.00	-0.00
0.03	0.04	0.02	0.00	0.00	0.00	0.00	0.00	1.14	-0.00	-0.00	0.03
0.03	0.03	0.00	0.00	0.00	0.00	0.00	1.10	0.00	-0.00	0.03	0.03
0.02	0.00	0.00	0.00	0.00	0.00	1.02	0.00	-0.00	0.03	0.03	0.03
0.00	0.00	0.00	0.00	0.00	1.13	0.00	-0.00	0.03	0.03	0.03	0.00
0.00	0.00	0.00	0.00	1.07	-0.01	-0.00	0.04	0.03	0.03	0.00	0.00
0.00	0.00	0.00	1.12	0.01	-0.00	0.03	0.03	0.03	0.00	0.00	0.00
0.00	0.00	1.11	-0.00	-0.00	0.03	0.02	0.03	0.00	0.00	0.00	0.00
0.00	1.13	-0.00	-0.00	0.03	0.02	0.03	0.00	0.00	0.00	0.00	0.00
1.13	0.00	-0.00	0.03	0.02	0.03	0.00	0.00	0.00	0.00	0.00	1.14
-0.00	-0.01	0.02	0.03	0.02	0.00	0.00	0.00	0.00	0.00	1.10	0.00
-0.00	0.03	0.03	0.03	0.00	0.00	0.00	0.00	0.00	1.12	0.01	-0.00
0.02	0.02	0.03	0.00	0.00	0.00	0.00	0.00	1.10	0.00	-0.01	0.02
0.03	0.03	0.00	0.00	0.00	0.00	0.00	1.15	-0.01	-0.00	0.03	0.04
0.02	0.00	0.00	0.00	0.00	0.00	1.16	-0.00	-0.00	0.03	0.03	0.02
0.00	0.00	0.00	0.00	0.00	1.12	-0.01	-0.01	0.03	0.02	0.03	0.00
0.00	0.00	0.00	0.00	1.16	-0.01	-0.00	0.03	0.02	0.03	0.00	0.00
0.00	0.00	0.00	1.14	-0.01	-0.00	0.04	0.02	0.03	0.00	0.00	0.00
0.00	0.00	1.18	-0.01	-0.01	0.03	0.03	0.03	0.00	0.00	0.00	0.00
0.00	1.10	0.01	-0.00	0.03	0.03	0.03	0.00	0.00	0.00	0.00	0.00
1.06	0.01	-0.00	0.03	0.02	0.03	0.00	0.00	0.00	0.00	0.00	1.09
0.00	-0.00	0.02	0.03	0.02	0.00	0.00	0.00	0.00	0.00	0.52	0.08
0.01	0.06	0.05	0.04	0.00	0.00	0.00	0.00	0.00	1.01	0.03	-0.00

0.03	0.03	0.03	0.00	0.00	0.00	0.00	0.00	1.10	0.01	-0.00	0.03
0.02	0.03	0.00	0.00	0.00	0.00	0.00	1.13	0.00	-0.00	0.02	0.02
0.03	0.00	0.00	0.00	0.00	0.00	0.97	0.02	-0.00	0.03	0.03	0.03
0.00	0.00	0.00	0.00	0.00	1.09	-0.00	-0.00	0.03	0.03	0.03	0.00
0.00	0.00	0.00	0.00	1.12	0.01	-0.00	0.03	0.03	0.03	0.00	0.00
0.00	0.00	0.00	1.15	-0.00	-0.00	0.03	0.03	0.03	0.00	0.00	0.00
0.00	0.00										

List of all MOs, ordered by energy, with the most significant SFO gross populations

==
=

Each percentage contribution in the table below corresponds to the indicated SFO.

In general, a SFO may be a linear combination of several Fragment Orbitals on the same,

or on symmetry-related Fragments. Only the first 'member' of such a combination is

specified here. A full definition of all SFOs is given in an earlier part of the output.

The numbering of the SFOs in this table does NOT include the Core Orbitals, and starts

from one for each symmetry representation, as in the SFO definition list earlier.

E(eV)	Occ	MO	%	SFO (first member)	E(eV)	Occ	Fragment
-22.749	2.00	11 A	54.25%	1 S	-18.438	2.00	43 N
			14.58%	1 S	-13.631	2.00	13 C
			7.83%	1 S	-13.631	2.00	26 C
			7.55%	1 S	-13.631	2.00	38 C
			3.13%	1 P:y	-5.263	0.67	38 C
			-2.02%	2 P:x	4.578	0.00	13 C
			1.89%	1 P:x	-5.263	0.67	26 C

			1.68%	1 S	-6.532	1.00	88 H	
			1.60%	1 S	-13.631	2.00	28 C	
			-1.43%	2 P:z	4.578	0.00	12 C	
			1.24%	1 S	-13.631	2.00	12 C	
			1.19%	1 P:z	-7.073	1.00	43 N	
			1.16%	1 S	-6.532	1.00	79 H	
			1.15%	1 S	-6.532	1.00	81 H	
			1.15%	1 S	-6.532	1.00	82 H	
			1.14%	1 P:x	-5.263	0.67	13 C	
			1.12%	1 S	-6.532	1.00	83 H	
			1.03%	1 S	-6.532	1.00	80 H	
-20.470	2.00	12 A	16.82%	1 S	-13.631	2.00	33 C	
			11.07%	1 S	-13.631	2.00	17 C	
			8.89%	1 S	-13.631	2.00	37 C	
			8.62%	1 S	-13.631	2.00	23 C	
			6.37%	1 S	-13.631	2.00	22 C	
			3.98%	1 P:z	-9.066	1.33	5 O	
			3.61%	1 S	-23.822	2.00	2 O	
			3.42%	1 S	-13.631	2.00	31 C	
			3.39%	1 S	-13.631	2.00	16 C	
			3.32%	1 S	-6.532	1.00	60 H	
			3.06%	1 S	-13.631	2.00	32 C	
			2.61%	1 S	-13.631	2.00	21 C	
			2.25%	1 S	-13.631	2.00	40 C	
			2.22%	1 S	-13.631	2.00	24 C	
			1.84%	1 S	-6.532	1.00	61 H	
			1.70%	1 S	-6.532	1.00	90 H	
			1.60%	1 S	-13.631	2.00	14 C	
			1.45%	1 P:x	-5.263	0.67	16 C	
			1.40%	1 P:y	-5.263	0.67	16 C	
			1.33%	1 P:x	-9.066	1.33	5 O	

			1.27%	1 S	-23.822	2.00	9 O
			1.22%	1 S	-6.532	1.00	62 H
			1.08%	1 P:y	-9.066	1.33	5 O
-20.393	2.00	13 A	13.07%	1 S	-13.631	2.00	24 C
			10.93%	1 S	-13.631	2.00	18 C
			10.64%	1 S	-13.631	2.00	19 C
			8.23%	1 S	-13.631	2.00	27 C
			5.66%	1 S	-13.631	2.00	25 C
			3.54%	1 S	-13.631	2.00	31 C
			3.37%	1 S	-13.631	2.00	11 C
			2.95%	1 S	-13.631	2.00	20 C
			2.88%	1 P:z	-9.066	1.33	1 O
			2.67%	1 S	-13.631	2.00	22 C
			2.01%	1 S	-13.631	2.00	12 C
			1.97%	1 S	-13.631	2.00	36 C
			1.91%	1 P:x	-9.066	1.33	4 O
			1.91%	1 S	-6.532	1.00	69 H
			1.69%	1 S	-23.822	2.00	4 O
			1.69%	1 P:x	-9.066	1.33	1 O
			1.57%	1 S	-13.631	2.00	14 C
			1.48%	1 S	-6.532	1.00	53 H
			1.39%	1 S	-6.532	1.00	52 H
			1.33%	1 S	-13.631	2.00	39 C
			1.31%	1 S	-13.631	2.00	29 C
			1.27%	1 S	-13.631	2.00	35 C
			-1.27%	2 P:x	4.578	0.00	14 C
			1.24%	1 S	-6.532	1.00	86 H
			1.24%	1 S	-13.631	2.00	23 C
			1.09%	1 P:x	-5.263	0.67	14 C
-19.943	2.00	14 A	18.62%	1 S	-13.631	2.00	31 C
			11.95%	1 S	-13.631	2.00	22 C

			8.73%	1 S	-13.631	2.00	17 C
			3.60%	1 S	-13.631	2.00	34 C
			3.27%	1 S	-13.631	2.00	25 C
			3.14%	1 S	-13.631	2.00	32 C
			3.13%	1 S	-13.631	2.00	18 C
			2.84%	1 S	-13.631	2.00	35 C
			2.74%	1 S	-13.631	2.00	37 C
			2.55%	1 S	-13.631	2.00	12 C
			2.27%	1 S	-13.631	2.00	24 C
			2.01%	1 S	-6.532	1.00	44 H
			1.75%	1 S	-13.631	2.00	11 C
			1.66%	1 S	-23.822	2.00	8 O
			1.63%	1 S	-13.631	2.00	23 C
			1.61%	1 S	-13.631	2.00	33 C
			1.52%	1 S	-6.532	1.00	90 H
			1.49%	1 S	-13.631	2.00	16 C
			1.32%	1 P:y	-5.263	0.67	35 C
			-1.21%	2 P:y	4.578	0.00	23 C
			1.04%	1 S	-13.631	2.00	40 C
-19.807	2.00	15 A	12.96%	1 S	-13.631	2.00	17 C
			8.63%	1 S	-13.631	2.00	25 C
			7.27%	1 S	-13.631	2.00	27 C
			7.22%	1 S	-13.631	2.00	24 C
			5.56%	1 S	-13.631	2.00	37 C
			4.48%	1 S	-13.631	2.00	33 C
			4.34%	1 S	-13.631	2.00	14 C
			4.11%	1 S	-13.631	2.00	32 C
			3.22%	1 S	-13.631	2.00	23 C
			3.18%	1 S	-13.631	2.00	19 C
			2.89%	1 S	-13.631	2.00	20 C
			2.58%	1 S	-6.532	1.00	90 H

			2.09%	1 S	-13.631	2.00	15	C
			2.08%	1 S	-13.631	2.00	40	C
			2.07%	1 S	-23.822	2.00	6	O
			1.50%	1 S	-13.631	2.00	21	C
			1.37%	1 S	-6.532	1.00	52	H
			-1.23%	2 P:y	4.578	0.00	18	C
			1.13%	1 P:x	-5.263	0.67	14	C
			1.13%	1 S	-13.631	2.00	36	C
			1.12%	1 S	-13.631	2.00	35	C
			1.11%	1 S	-6.532	1.00	61	H
			-1.05%	2 P:y	4.578	0.00	22	C
-19.784	2.00	16 A	18.27%	1 S	-13.631	2.00	28	C
			13.90%	1 S	-13.631	2.00	13	C
			9.17%	1 S	-13.631	2.00	12	C
			8.52%	1 S	-13.631	2.00	29	C
			3.99%	1 S	-23.822	2.00	3	O
			3.68%	1 S	-18.438	2.00	43	N
			3.25%	1 S	-6.532	1.00	78	H
			3.13%	1 S	-13.631	2.00	30	C
			2.82%	1 S	-13.631	2.00	38	C
			2.47%	1 S	-6.532	1.00	77	H
			2.10%	1 S	-6.532	1.00	88	H
			1.94%	1 S	-13.631	2.00	26	C
			1.83%	1 P:x	-7.073	1.00	43	N
			1.56%	1 P:y	-9.066	1.33	7	O
			1.35%	1 S	-13.631	2.00	42	C
			1.35%	1 P:x	-5.263	0.67	13	C
			1.31%	1 P:z	-9.066	1.33	7	O
			1.27%	1 S	-13.631	2.00	41	C
			1.27%	1 P:x	-5.263	0.67	29	C
			-1.11%	2 P:y	4.578	0.00	13	C

			1.06%	1 S	-6.532	1.00	87 H
			1.02%	1 S	-6.532	1.00	76 H
-19.450	2.00	17 A	7.39%	1 S	-13.631	2.00	37 C
			6.72%	1 S	-13.631	2.00	25 C
			6.21%	1 S	-13.631	2.00	29 C
			5.94%	1 S	-13.631	2.00	31 C
			5.76%	1 S	-13.631	2.00	27 C
			4.29%	1 S	-13.631	2.00	22 C
			4.16%	1 S	-13.631	2.00	30 C
			3.98%	1 S	-13.631	2.00	17 C
			3.85%	1 S	-13.631	2.00	40 C
			3.45%	1 S	-13.631	2.00	12 C
			2.46%	1 S	-13.631	2.00	11 C
			2.41%	1 S	-13.631	2.00	19 C
			2.30%	1 S	-13.631	2.00	15 C
			2.07%	1 S	-13.631	2.00	34 C
			2.06%	1 S	-13.631	2.00	28 C
			1.94%	1 S	-13.631	2.00	33 C
			1.65%	1 S	-13.631	2.00	32 C
			-1.30%	2 P:y	4.578	0.00	35 C
			1.26%	1 S	-6.532	1.00	61 H
			1.22%	1 S	-13.631	2.00	42 C
			1.21%	1 S	-6.532	1.00	62 H
			1.21%	1 S	-6.532	1.00	52 H
-19.288	2.00	18 A	14.70%	1 S	-13.631	2.00	24 C
			7.37%	1 S	-13.631	2.00	29 C
			7.26%	1 S	-13.631	2.00	20 C
			6.38%	1 S	-13.631	2.00	17 C
			5.60%	1 S	-13.631	2.00	30 C
			4.71%	1 S	-13.631	2.00	12 C
			3.81%	1 S	-13.631	2.00	32 C

			2.64%	1 S	-13.631	2.00	37 C
			2.50%	1 S	-13.631	2.00	11 C
			2.16%	1 S	-13.631	2.00	28 C
			-2.07%	2 P:x	4.578	0.00	14 C
			1.93%	1 S	-23.822	2.00	1 O
			1.89%	1 S	-6.532	1.00	69 H
			1.82%	1 S	-13.631	2.00	42 C
			1.72%	1 S	-6.532	1.00	70 H
			1.54%	1 S	-13.631	2.00	40 C
			1.48%	1 S	-6.532	1.00	72 H
			1.36%	1 S	-6.532	1.00	90 H
			1.30%	1 P:y	-9.066	1.33	7 O
			1.24%	1 S	-13.631	2.00	41 C
			1.21%	1 S	-6.532	1.00	71 H
			1.20%	1 S	-6.532	1.00	74 H
			1.19%	1 S	-6.532	1.00	75 H
			1.14%	1 S	-6.532	1.00	73 H
			1.11%	1 P:y	-9.066	1.33	3 O
			1.05%	1 S	-6.532	1.00	96 H
			1.05%	1 S	-13.631	2.00	18 C
			1.01%	1 S	-6.532	1.00	76 H
			-1.01%	2 P:z	4.578	0.00	14 C
-18.808	2.00	19 A	9.67%	1 S	-13.631	2.00	19 C
			7.37%	1 S	-13.631	2.00	25 C
			7.05%	1 S	-13.631	2.00	23 C
			6.53%	1 S	-13.631	2.00	36 C
			6.12%	1 S	-13.631	2.00	21 C
			3.89%	1 S	-13.631	2.00	40 C
			3.73%	1 S	-13.631	2.00	37 C
			3.23%	1 S	-13.631	2.00	20 C
			3.09%	1 S	-13.631	2.00	24 C

			2.85%	1 S	-13.631	2.00	15 C
			2.38%	1 S	-13.631	2.00	35 C
			2.26%	1 S	-13.631	2.00	31 C
			1.73%	1 S	-6.532	1.00	67 H
			1.69%	1 S	-6.532	1.00	54 H
			1.62%	1 S	-6.532	1.00	51 H
			1.60%	1 P:x	-9.066	1.33	4 O
			1.50%	1 S	-6.532	1.00	55 H
			-1.36%	2 P:z	4.578	0.00	27 C
			-1.32%	2 P:y	4.578	0.00	18 C
			1.25%	1 S	-6.532	1.00	66 H
			1.21%	1 S	-13.631	2.00	42 C
			1.16%	1 S	-6.532	1.00	68 H
			1.11%	1 S	-23.822	2.00	8 O
			1.08%	1 S	-6.532	1.00	65 H
			1.05%	1 S	-13.631	2.00	39 C
			1.04%	1 S	-6.532	1.00	56 H
			1.02%	1 S	-13.631	2.00	18 C
			-1.00%	2 P:y	4.578	0.00	22 C
-18.585	2.00	20 A	10.44%	1 S	-13.631	2.00	31 C
			8.55%	1 S	-13.631	2.00	25 C
			6.82%	1 S	-13.631	2.00	34 C
			6.32%	1 S	-13.631	2.00	23 C
			6.28%	1 S	-13.631	2.00	21 C
			6.02%	1 S	-13.631	2.00	40 C
			5.41%	1 S	-13.631	2.00	15 C
			4.49%	1 S	-13.631	2.00	37 C
			3.43%	1 S	-13.631	2.00	19 C
			2.70%	1 S	-13.631	2.00	36 C
			1.79%	1 S	-6.532	1.00	67 H
			1.71%	1 S	-6.532	1.00	65 H

			1.69%	1 S	-6.532	1.00	47 H
			-1.48%	2 P:y	4.578	0.00	35 C
			1.43%	1 S	-6.532	1.00	64 H
			1.34%	1 S	-6.532	1.00	51 H
			1.32%	1 S	-6.532	1.00	48 H
			1.30%	1 S	-6.532	1.00	49 H
			1.30%	1 S	-6.532	1.00	66 H
			1.26%	1 S	-6.532	1.00	63 H
			1.26%	1 P:y	-5.263	0.67	35 C
			1.20%	1 S	-6.532	1.00	45 H
			1.17%	1 S	-6.532	1.00	46 H
			1.14%	1 S	-13.631	2.00	20 C
			1.06%	1 S	-6.532	1.00	50 H
			1.01%	1 P:z	-5.263	0.67	23 C
			1.01%	1 S	-6.532	1.00	68 H
-18.412	2.00	21 A	28.59%	1 S	-13.631	2.00	42 C
			7.15%	1 P:y	-5.263	0.67	41 C
			7.02%	1 S	-6.532	1.00	92 H
			6.79%	1 S	-6.532	1.00	91 H
			5.93%	1 S	-13.631	2.00	41 C
			5.66%	1 S	-6.532	1.00	93 H
			5.14%	1 S	-13.631	2.00	13 C
			4.98%	1 S	-13.631	2.00	30 C
			2.02%	1 S	-13.631	2.00	28 C
			1.83%	1 S	-13.631	2.00	29 C
			-1.78%	2 P:y	4.578	0.00	41 C
			1.62%	1 P:y	-9.066	1.33	7 O
			1.46%	1 S	-6.532	1.00	75 H
			1.46%	1 S	-23.822	2.00	10 O
			1.33%	1 P:y	-5.263	0.67	12 C
			1.11%	1 S	-6.532	1.00	74 H

			1.10%	1 S	-6.532	1.00	73 H	
			1.09%	1 S	-23.822	2.00	7 O	
-17.515	2.00	22 A	8.04%	1 S	-13.631	2.00	21 C	
			7.05%	1 P:z	-9.066	1.33	5 O	
			6.75%	1 S	-13.631	2.00	33 C	
			6.17%	1 S	-13.631	2.00	40 C	
			5.01%	1 S	-13.631	2.00	30 C	
			2.68%	1 S	-6.532	1.00	68 H	
			2.17%	1 S	-13.631	2.00	11 C	
			2.14%	1 S	-13.631	2.00	36 C	
			2.05%	1 S	-6.532	1.00	66 H	
			2.02%	1 S	-6.532	1.00	65 H	
			2.00%	1 S	-6.532	1.00	67 H	
			1.94%	1 S	-13.631	2.00	18 C	
			1.87%	1 S	-6.532	1.00	60 H	
			1.86%	1 S	-13.631	2.00	16 C	
			1.73%	1 S	-6.532	1.00	63 H	
			1.64%	1 P:x	-5.263	0.67	23 C	
			1.58%	1 S	-13.631	2.00	32 C	
			1.57%	1 S	-6.532	1.00	64 H	
			1.54%	1 S	-6.532	1.00	74 H	
			1.52%	1 P:x	-9.066	1.33	1 O	
			1.47%	1 S	-6.532	1.00	75 H	
			1.38%	1 S	-13.631	2.00	13 C	
			1.36%	1 P:z	-9.066	1.33	1 O	
			1.28%	1 S	-6.532	1.00	73 H	
			1.26%	1 P:x	-9.066	1.33	5 O	
			1.20%	1 S	-13.631	2.00	28 C	
			1.19%	1 S	-13.631	2.00	42 C	
			1.18%	1 S	-13.631	2.00	12 C	
-17.507	2.00	23 A	7.35%	1 S	-13.631	2.00	30 C	

			5.31%	1 S	-13.631	2.00	21 C
			4.47%	1 P:z	-9.066	1.33	5 O
			4.35%	1 S	-13.631	2.00	33 C
			3.97%	1 S	-13.631	2.00	40 C
			3.31%	1 S	-13.631	2.00	11 C
			2.84%	1 S	-13.631	2.00	32 C
			2.59%	1 S	-13.631	2.00	18 C
			2.25%	1 S	-6.532	1.00	74 H
			2.15%	1 S	-6.532	1.00	75 H
			2.12%	1 P:x	-9.066	1.33	1 O
			2.08%	1 S	-13.631	2.00	36 C
			2.05%	1 P:z	-9.066	1.33	1 O
			2.01%	1 S	-13.631	2.00	13 C
			1.96%	1 S	-13.631	2.00	20 C
			1.88%	1 S	-6.532	1.00	73 H
			1.84%	1 S	-13.631	2.00	42 C
			1.82%	1 S	-6.532	1.00	68 H
			1.81%	1 S	-13.631	2.00	28 C
			1.79%	1 S	-13.631	2.00	12 C
			1.37%	1 S	-6.532	1.00	66 H
			1.33%	1 S	-6.532	1.00	67 H
			1.31%	1 S	-6.532	1.00	65 H
			1.29%	1 S	-13.631	2.00	16 C
			1.20%	1 S	-6.532	1.00	60 H
			1.12%	1 P:x	-5.263	0.67	23 C
			1.07%	1 S	-6.532	1.00	63 H
			1.04%	1 S	-6.532	1.00	95 H
			1.03%	1 S	-6.532	1.00	64 H
-17.289	2.00	24 A	24.10%	1 S	-13.631	2.00	26 C
			18.83%	1 S	-13.631	2.00	38 C
			9.64%	1 S	-6.532	1.00	80 H

			9.51%	1 P:y	-7.073	1.00	43 N
			8.37%	1 S	-6.532	1.00	83 H
			6.48%	1 S	-6.532	1.00	81 H
			4.71%	1 S	-6.532	1.00	79 H
			4.30%	1 S	-6.532	1.00	84 H
			3.63%	1 S	-6.532	1.00	82 H
			3.17%	1 P:z	-7.073	1.00	43 N
			2.62%	1 P:x	-7.073	1.00	43 N
			-1.52%	2 P:y	7.645	0.00	43 N
			1.19%	1 P:y	11.052	0.00	80 H
			1.10%	1 S	-6.532	1.00	88 H
-17.177	2.00	25 A	19.54%	1 S	-13.631	2.00	36 C
			13.83%	1 S	-13.631	2.00	39 C
			7.01%	1 S	-6.532	1.00	55 H
			6.51%	1 P:x	-9.066	1.33	4 O
			6.30%	1 S	-6.532	1.00	56 H
			5.77%	1 S	-6.532	1.00	57 H
			5.57%	1 S	-6.532	1.00	54 H
			4.99%	1 S	-6.532	1.00	59 H
			4.56%	1 P:y	-5.263	0.67	19 C
			4.39%	1 S	-6.532	1.00	58 H
			2.78%	1 S	-13.631	2.00	15 C
			-2.75%	2 P:y	4.578	0.00	19 C
			2.65%	1 S	-23.822	2.00	4 O
			1.89%	1 S	-13.631	2.00	27 C
			1.87%	1 P:y	-9.066	1.33	4 O
			1.63%	1 P:z	-5.263	0.67	19 C
			1.62%	1 S	-13.631	2.00	20 C
			1.09%	1 S	-6.532	1.00	50 H
-16.876	2.00	26 A	24.87%	1 S	-13.631	2.00	34 C
			17.08%	1 S	-13.631	2.00	22 C

			7.97%	1 P:x	-5.263	0.67	31 C
			7.85%	1 S	-6.532	1.00	47 H
			7.37%	1 S	-6.532	1.00	45 H
			6.72%	1 S	-6.532	1.00	46 H
			5.79%	1 S	-6.532	1.00	44 H
			4.58%	1 S	-13.631	2.00	21 C
			-3.79%	2 P:x	4.578	0.00	31 C
			3.37%	1 P:y	-5.263	0.67	23 C
			1.99%	1 S	-6.532	1.00	66 H
			1.81%	1 S	-6.532	1.00	68 H
			1.75%	1 S	-13.631	2.00	40 C
			1.13%	1 S	-6.532	1.00	67 H
-16.808	2.00	27 A	8.92%	1 S	-13.631	2.00	18 C
			8.49%	1 S	-13.631	2.00	28 C
			7.46%	1 S	-13.631	2.00	12 C
			6.00%	1 S	-13.631	2.00	20 C
			3.99%	1 S	-13.631	2.00	30 C
			3.89%	1 S	-13.631	2.00	42 C
			3.18%	1 S	-6.532	1.00	78 H
			3.13%	1 S	-13.631	2.00	39 C
			2.63%	1 P:z	-9.066	1.33	7 O
			2.31%	1 S	-6.532	1.00	72 H
			2.20%	1 S	-6.532	1.00	86 H
			2.14%	1 P:y	-9.066	1.33	7 O
			2.12%	1 S	-6.532	1.00	77 H
			2.01%	1 P:y	-5.263	0.67	24 C
			1.81%	1 P:z	-9.066	1.33	1 O
			1.78%	1 S	-6.532	1.00	71 H
			1.77%	1 S	-6.532	1.00	70 H
			1.64%	1 S	-6.532	1.00	91 H
			1.62%	1 P:x	-9.066	1.33	4 O

			1.61%	1 S	-6.532	1.00	75	H
			1.58%	1 P:x	-9.066	1.33	1	O
			1.52%	1 S	-6.532	1.00	59	H
			1.45%	1 P:z	-5.263	0.67	13	C
			1.40%	1 S	-6.532	1.00	87	H
			1.27%	1 P:z	-5.263	0.67	19	C
			1.19%	1 S	-6.532	1.00	58	H
			1.18%	1 S	-6.532	1.00	73	H
			1.18%	1 S	-6.532	1.00	93	H
			1.09%	1 P:x	-5.263	0.67	29	C
			1.05%	1 S	-6.532	1.00	74	H
			1.03%	1 P:y	-5.263	0.67	13	C
			1.00%	1 S	-6.532	1.00	92	H
-16.631	2.00	28 A	16.16%	1 S	-13.631	2.00	15	C
			13.15%	1 S	-13.631	2.00	27	C
			5.77%	1 S	-6.532	1.00	49	H
			5.12%	1 S	-6.532	1.00	53	H
			5.07%	1 S	-13.631	2.00	39	C
			4.91%	1 S	-6.532	1.00	50	H
			4.44%	1 S	-6.532	1.00	48	H
			3.86%	1 S	-6.532	1.00	52	H
			3.50%	1 S	-13.631	2.00	32	C
			2.89%	1 S	-6.532	1.00	59	H
			2.42%	1 S	-13.631	2.00	20	C
			2.34%	1 P:x	-5.263	0.67	19	C
			1.99%	1 P:z	-5.263	0.67	25	C
			1.94%	1 P:y	-5.263	0.67	25	C
			1.67%	1 P:x	-9.066	1.33	4	O
			1.49%	1 S	-6.532	1.00	58	H
			1.46%	1 S	-6.532	1.00	57	H
			1.44%	1 S	-6.532	1.00	95	H

			-1.44%	2 P:y	4.578	0.00	25 C
			1.36%	1 S	-13.631	2.00	14 C
			1.31%	1 S	-6.532	1.00	94 H
			1.30%	1 S	-6.532	1.00	96 H
			-1.23%	2 P:z	4.578	0.00	25 C
-16.546	2.00	29 A	18.00%	1 S	-13.631	2.00	32 C
			6.90%	1 S	-6.532	1.00	96 H
			6.23%	1 S	-6.532	1.00	95 H
			5.69%	1 S	-6.532	1.00	94 H
			5.16%	1 S	-13.631	2.00	14 C
			4.72%	1 S	-13.631	2.00	20 C
			3.36%	1 P:x	-5.263	0.67	17 C
			2.97%	1 S	-13.631	2.00	39 C
			2.85%	1 S	-13.631	2.00	15 C
			2.74%	1 S	-13.631	2.00	17 C
			2.43%	1 S	-13.631	2.00	27 C
			1.81%	1 S	-13.631	2.00	16 C
			1.78%	1 S	-6.532	1.00	71 H
			1.77%	1 P:z	-5.263	0.67	17 C
			1.74%	1 S	-6.532	1.00	70 H
			1.32%	1 S	-6.532	1.00	90 H
			1.28%	1 P:y	-5.263	0.67	14 C
			1.26%	1 P:z	-5.263	0.67	24 C
			1.21%	1 S	-6.532	1.00	57 H
			1.12%	1 S	-6.532	1.00	72 H
			1.07%	1 P:x	-9.066	1.33	4 O
			1.04%	1 S	-6.532	1.00	53 H
			1.04%	1 S	-6.532	1.00	49 H
			1.04%	1 S	-13.631	2.00	40 C
-16.169	2.00	30 A	8.13%	1 S	-13.631	2.00	38 C
			7.04%	1 P:x	-7.073	1.00	43 N

			6.25%	1 S	-13.631	2.00	13 C
			6.06%	1 S	-13.631	2.00	28 C
			4.76%	1 S	-6.532	1.00	84 H
			3.48%	1 S	-13.631	2.00	26 C
			3.46%	1 S	-13.631	2.00	30 C
			2.65%	1 S	-6.532	1.00	81 H
			2.59%	1 S	-13.631	2.00	20 C
			2.57%	1 S	-6.532	1.00	82 H
			2.40%	1 P:x	-5.263	0.67	13 C
			2.15%	1 S	-6.532	1.00	83 H
			2.07%	1 S	-13.631	2.00	11 C
			1.97%	1 S	-18.438	2.00	43 N
			1.90%	1 S	-6.532	1.00	75 H
			1.88%	1 S	-6.532	1.00	77 H
			1.70%	1 S	-6.532	1.00	78 H
			1.63%	1 P:x	-5.263	0.67	29 C
			1.58%	1 S	-6.532	1.00	88 H
			1.47%	1 S	-13.631	2.00	29 C
			1.39%	1 S	-13.631	2.00	18 C
			1.30%	1 S	-6.532	1.00	73 H
			1.23%	1 P:y	-5.263	0.67	13 C
			1.20%	1 S	-23.822	2.00	3 O
			1.19%	1 S	-6.532	1.00	79 H
			1.15%	1 P:y	-7.073	1.00	43 N
			1.14%	1 S	-6.532	1.00	72 H
-15.813	2.00	31 A	8.92%	1 S	-13.631	2.00	35 C
			5.97%	1 S	-13.631	2.00	40 C
			4.55%	1 S	-13.631	2.00	27 C
			3.87%	1 S	-13.631	2.00	37 C
			3.22%	1 S	-13.631	2.00	34 C
			3.20%	1 P:y	-5.263	0.67	33 C

			3.06%	1 P:y	-5.263	0.67	31 C	
			2.86%	1 P:y	-5.263	0.67	22 C	
			2.79%	1 S	-6.532	1.00	65 H	
			2.70%	1 P:y	-5.263	0.67	23 C	
			2.69%	1 S	-6.532	1.00	63 H	
			2.66%	1 S	-6.532	1.00	64 H	
			2.49%	1 S	-6.532	1.00	61 H	
			2.24%	1 S	-13.631	2.00	23 C	
			2.04%	1 S	-13.631	2.00	21 C	
			1.99%	1 S	-23.822	2.00	8 O	
			1.94%	1 P:x	-5.263	0.67	35 C	
			1.85%	1 S	-6.532	1.00	62 H	
			1.80%	1 S	-6.532	1.00	66 H	
			1.75%	1 S	-13.631	2.00	31 C	
			1.74%	1 S	-6.532	1.00	52 H	
			1.69%	1 S	-13.631	2.00	22 C	
			1.67%	1 P:y	-5.263	0.67	25 C	
			1.65%	1 S	-6.532	1.00	46 H	
			1.64%	1 S	-13.631	2.00	15 C	
			1.42%	1 P:y	-5.263	0.67	35 C	
			1.32%	1 P:x	-5.263	0.67	37 C	
			1.14%	1 P:x	-5.263	0.67	19 C	
			1.11%	1 S	-6.532	1.00	47 H	
			1.08%	1 P:z	-9.066	1.33	5 O	
			-1.04%	2 P:x	4.578	0.00	37 C	
-15.795	2.00	32 A	5.43%	1 S	-13.631	2.00	20 C	
			4.09%	1 S	-13.631	2.00	14 C	
			3.63%	1 P:z	-9.066	1.33	5 O	
			3.09%	1 S	-13.631	2.00	24 C	
			2.86%	1 S	-13.631	2.00	16 C	
			2.68%	1 S	-6.532	1.00	71 H	

			2.51%	1 S	-13.631	2.00	40 C		
			2.22%	1 P:z	-9.066	1.33	1 O		
			2.19%	1 S	-13.631	2.00	37 C		
			2.15%	1 S	-6.532	1.00	70 H		
			2.11%	1 S	-13.631	2.00	21 C		
			2.10%	1 S	-6.532	1.00	72 H		
			1.67%	1 P:z	-9.066	1.33	7 O		
			1.57%	1 P:x	-5.263	0.67	14 C		
			1.55%	1 S	-13.631	2.00	30 C		
			1.51%	1 P:z	-9.066	1.33	3 O		
			1.46%	1 S	-13.631	2.00	12 C		
			1.39%	1 S	-13.631	2.00	11 C		
			1.28%	1 P:y	-5.263	0.67	17 C		
			1.23%	1 P:y	-5.263	0.67	33 C		
			1.22%	1 S	-6.532	1.00	64 H		
			1.22%	1 P:y	-9.066	1.33	7 O		
			1.19%	1 S	-6.532	1.00	61 H		
			1.17%	1 P:z	-5.263	0.67	24 C		
			1.17%	1 S	-6.532	1.00	63 H		
			1.16%	1 S	-6.532	1.00	65 H		
			1.11%	1 S	-23.822	2.00	6 O		
			1.09%	1 P:x	-5.263	0.67	24 C		
			1.05%	1 P:x	-5.263	0.67	33 C		
			1.01%	1 S	-13.631	2.00	32 C		
-15.511	2.00	33 A	6.56%	1 P:z	-9.066	1.33	5 O		
			4.32%	1 S	-13.631	2.00	11 C		
			3.41%	1 S	-13.631	2.00	37 C		
			2.63%	1 P:y	-9.066	1.33	3 O		
			2.44%	1 S	-13.631	2.00	40 C		
			2.42%	1 P:z	-9.066	1.33	7 O		
			2.38%	1 P:z	-9.066	1.33	3 O		

			1.98%	1 S	-6.532	1.00	61	H
			1.97%	1 S	-13.631	2.00	30	C
			1.93%	1 S	-6.532	1.00	62	H
			1.82%	1 S	-13.631	2.00	35	C
			1.80%	1 P:y	-9.066	1.33	7	O
			1.78%	1 S	-13.631	2.00	21	C
			1.73%	1 P:z	-9.066	1.33	1	O
			1.71%	1 P:y	-5.263	0.67	33	C
			1.59%	1 S	-6.532	1.00	85	H
			1.45%	1 S	-6.532	1.00	64	H
			1.44%	1 P:x	-5.263	0.67	33	C
			1.39%	1 S	-6.532	1.00	74	H
			1.38%	1 S	-23.822	2.00	1	O
			1.37%	1 S	-6.532	1.00	63	H
			1.29%	1 P:z	-5.263	0.67	17	C
			1.23%	1 S	-13.631	2.00	17	C
			1.14%	1 S	-13.631	2.00	12	C
			1.12%	1 S	-6.532	1.00	65	H
			1.11%	1 S	-6.532	1.00	90	H
			1.01%	1 P:z	-5.263	0.67	16	C
-15.106	2.00	34 A	5.60%	1 S	-13.631	2.00	18	C
			4.65%	1 S	-6.532	1.00	86	H
			3.54%	1 S	-6.532	1.00	85	H
			3.45%	1 S	-13.631	2.00	24	C
			3.24%	1 S	-13.631	2.00	11	C
			3.22%	1 P:y	-9.066	1.33	3	O
			2.56%	1 S	-6.532	1.00	69	H
			2.49%	1 S	-23.822	2.00	1	O
			2.43%	1 S	-13.631	2.00	14	C
			2.33%	1 S	-13.631	2.00	17	C
			2.25%	1 S	-6.532	1.00	90	H

			2.17%	1 S	-13.631	2.00	37 C
			2.13%	1 P:x	-9.066	1.33	1 O
			2.00%	1 P:x	-5.263	0.67	14 C
			1.82%	1 S	-13.631	2.00	20 C
			1.71%	1 P:z	-5.263	0.67	11 C
			1.70%	1 P:z	-9.066	1.33	3 O
			1.67%	1 P:z	-9.066	1.33	5 O
			1.58%	1 S	-6.532	1.00	70 H
			1.49%	1 S	-6.532	1.00	62 H
			1.48%	1 P:y	-5.263	0.67	17 C
			1.42%	1 S	-13.631	2.00	30 C
			1.34%	1 S	-6.532	1.00	61 H
			1.34%	1 P:z	-5.263	0.67	19 C
			1.34%	1 S	-6.532	1.00	71 H
			1.26%	1 S	-13.631	2.00	32 C
			1.23%	1 S	-6.532	1.00	74 H
			1.13%	1 P:z	-5.263	0.67	24 C
			1.12%	1 S	-13.631	2.00	40 C
			1.03%	1 S	-6.532	1.00	94 H
-14.848	2.00	35 A	7.45%	1 S	-13.631	2.00	23 C
			4.38%	1 S	-13.631	2.00	25 C
			3.75%	1 S	-13.631	2.00	15 C
			3.17%	1 P:x	-5.263	0.67	33 C
			3.17%	1 S	-13.631	2.00	21 C
			2.89%	1 P:z	-9.066	1.33	2 O
			2.46%	1 P:z	-9.066	1.33	5 O
			2.37%	1 S	-13.631	2.00	31 C
			2.34%	1 S	-6.532	1.00	68 H
			2.17%	1 S	-6.532	1.00	50 H
			2.15%	1 P:y	-5.263	0.67	22 C
			2.12%	1 S	-6.532	1.00	66 H

			1.90%	1 S	-6.532	1.00	48 H
			1.85%	1 S	-6.532	1.00	67 H
			1.84%	1 S	-13.631	2.00	35 C
			1.75%	1 S	-13.631	2.00	19 C
			1.64%	1 P:z	-5.263	0.67	27 C
			1.60%	1 S	-6.532	1.00	49 H
			1.59%	1 S	-13.631	2.00	34 C
			1.52%	1 P:x	-5.263	0.67	22 C
			1.52%	1 S	-13.631	2.00	36 C
			1.51%	1 S	-6.532	1.00	51 H
			1.14%	1 S	-6.532	1.00	55 H
			1.10%	1 P:y	-5.263	0.67	25 C
			1.06%	1 P:x	-5.263	0.67	31 C
			1.05%	1 S	-13.631	2.00	22 C
-14.589	2.00	36 A	9.06%	1 S	-6.532	1.00	76 H
			8.37%	1 S	-6.532	1.00	85 H
			8.18%	1 S	-13.631	2.00	29 C
			5.01%	1 P:z	-9.066	1.33	3 O
			4.30%	1 P:y	-5.263	0.67	11 C
			3.72%	1 S	-6.532	1.00	87 H
			3.32%	1 S	-13.631	2.00	11 C
			3.30%	1 S	-18.438	2.00	43 N
			2.99%	1 S	-6.532	1.00	73 H
			2.81%	1 S	-6.532	1.00	77 H
			2.51%	1 S	-13.631	2.00	30 C
			2.37%	1 S	-23.822	2.00	3 O
			2.35%	1 P:y	-5.263	0.67	12 C
			2.29%	1 S	-13.631	2.00	38 C
			2.07%	1 P:y	-5.263	0.67	30 C
			1.86%	1 S	-6.532	1.00	84 H
			1.82%	1 P:x	-5.263	0.67	28 C

			1.79%	1 P:y	-5.263	0.67	29 C
			1.64%	1 P:x	-5.263	0.67	12 C
			1.62%	1 S	-6.532	1.00	82 H
			1.52%	1 S	-6.532	1.00	83 H
			1.51%	1 S	-6.532	1.00	74 H
			1.51%	1 P:y	-5.263	0.67	38 C
			1.45%	1 P:z	-9.066	1.33	1 O
			1.40%	1 S	-13.631	2.00	12 C
			1.38%	1 P:y	-5.263	0.67	13 C
			1.33%	1 S	-6.532	1.00	75 H
			1.33%	1 S	-13.631	2.00	28 C
			1.31%	1 P:x	-9.066	1.33	7 O
			-1.28%	2 P:x	4.578	0.00	12 C
			1.27%	1 P:z	-5.263	0.67	11 C
			-1.04%	2 P:x	4.578	0.00	11 C
-14.194	2.00	37 A	6.23%	1 S	-13.631	2.00	13 C
			5.75%	1 S	-6.532	1.00	88 H
			5.02%	1 S	-6.532	1.00	77 H
			4.89%	1 P:x	-5.263	0.67	13 C
			4.82%	1 P:y	-9.066	1.33	7 O
			4.32%	1 S	-13.631	2.00	28 C
			3.77%	1 S	-18.438	2.00	43 N
			3.64%	1 S	-6.532	1.00	78 H
			3.62%	1 P:z	-5.263	0.67	12 C
			3.18%	1 P:z	-9.066	1.33	7 O
			2.75%	1 P:y	-5.263	0.67	28 C
			2.51%	1 S	-13.631	2.00	41 C
			2.21%	1 S	-13.631	2.00	26 C
			2.15%	1 S	-6.532	1.00	76 H
			2.04%	1 P:z	-9.066	1.33	3 O
			2.02%	1 S	-6.532	1.00	81 H

			1.99%	1 S	-6.532	1.00	79	H
			1.69%	1 P:z	-5.263	0.67	11	C
			1.67%	1 S	-6.532	1.00	91	H
			1.55%	1 P:x	-5.263	0.67	29	C
			1.42%	1 S	-6.532	1.00	80	H
			1.30%	1 P:y	-5.263	0.67	38	C
			1.25%	1 S	-13.631	2.00	42	C
			1.23%	1 S	-6.532	1.00	85	H
			1.16%	1 S	-6.532	1.00	83	H
			1.15%	1 P:x	-9.066	1.33	7	O
			1.15%	1 P:y	-5.263	0.67	29	C
			1.09%	1 S	-6.532	1.00	93	H
			1.08%	1 S	-6.532	1.00	82	H
-14.093	2.00	38 A	11.43%	1 P:z	-9.066	1.33	7	O
			7.90%	1 S	-6.532	1.00	87	H
			7.08%	1 P:y	-5.263	0.67	12	C
			4.59%	1 P:x	-5.263	0.67	11	C
			3.73%	1 S	-13.631	2.00	12	C
			3.13%	1 S	-13.631	2.00	41	C
			2.91%	1 S	-23.822	2.00	7	O
			2.63%	1 P:x	-9.066	1.33	1	O
			2.04%	1 S	-6.532	1.00	88	H
			1.87%	1 S	-6.532	1.00	85	H
			1.44%	1 P:z	-5.263	0.67	12	C
			1.35%	1 S	-13.631	2.00	29	C
			1.29%	1 P:x	-9.066	1.33	3	O
			1.28%	1 P:z	-9.066	1.33	3	O
			1.20%	1 S	-6.532	1.00	91	H
			1.18%	1 S	-18.438	2.00	43	N
			1.18%	1 S	-6.532	1.00	78	H
			1.15%	1 S	-23.822	2.00	10	O

			1.08%	1 P:x	-9.066	1.33	7 O		
			1.06%	1 S	-6.532	1.00	81 H		
			1.03%	1 P:z	-5.263	0.67	41 C		
-13.825	2.00	39 A	4.41%	1 S	-13.631	2.00	25 C		
			3.95%	1 P:y	-5.263	0.67	35 C		
			3.34%	1 S	-13.631	2.00	31 C		
			3.26%	1 P:y	-5.263	0.67	31 C		
			2.76%	1 S	-6.532	1.00	51 H		
			2.45%	1 S	-6.532	1.00	62 H		
			2.45%	1 S	-13.631	2.00	22 C		
			2.45%	1 S	-13.631	2.00	33 C		
			2.29%	1 S	-6.532	1.00	60 H		
			2.07%	1 S	-6.532	1.00	46 H		
			1.98%	1 S	-13.631	2.00	37 C		
			1.81%	1 S	-6.532	1.00	87 H		
			1.72%	1 S	-13.631	2.00	16 C		
			1.72%	1 P:y	-5.263	0.67	23 C		
			1.68%	1 S	-13.631	2.00	15 C		
			1.64%	1 P:z	-5.263	0.67	17 C		
			1.62%	1 P:z	-5.263	0.67	33 C		
			1.53%	1 P:z	-9.066	1.33	7 O		
			1.47%	1 S	-6.532	1.00	50 H		
			1.42%	1 S	-6.532	1.00	48 H		
			1.39%	1 P:y	-5.263	0.67	15 C		
			1.28%	1 S	-13.631	2.00	35 C		
			1.27%	1 S	-13.631	2.00	34 C		
			1.24%	1 P:y	-5.263	0.67	12 C		
			1.24%	1 S	-6.532	1.00	69 H		
			1.16%	1 P:y	-9.066	1.33	8 O		
			1.11%	1 S	-6.532	1.00	86 H		
			1.08%	1 P:x	-5.263	0.67	23 C		

			1.07%	1 S	-13.631	2.00	14 C	
-13.679	2.00	40 A	8.20%	1 P:z	-9.066	1.33	2 O	
			5.09%	1 S	-13.631	2.00	14 C	
			4.09%	1 S	-13.631	2.00	19 C	
			3.64%	1 P:y	-5.263	0.67	17 C	
			3.28%	1 S	-6.532	1.00	60 H	
			2.74%	1 S	-13.631	2.00	23 C	
			2.57%	1 P:y	-5.263	0.67	18 C	
			2.35%	1 P:z	-5.263	0.67	17 C	
			2.29%	1 S	-13.631	2.00	35 C	
			2.06%	1 S	-23.822	2.00	6 O	
			2.02%	1 P:x	-5.263	0.67	16 C	
			1.99%	1 P:z	-5.263	0.67	33 C	
			1.74%	1 S	-6.532	1.00	44 H	
			1.69%	1 S	-13.631	2.00	22 C	
			1.46%	1 S	-6.532	1.00	67 H	
			1.37%	1 P:x	-9.066	1.33	4 O	
			1.33%	1 S	-13.631	2.00	24 C	
			1.32%	1 S	-13.631	2.00	16 C	
			1.32%	1 P:z	-5.263	0.67	21 C	
			1.31%	1 S	-6.532	1.00	56 H	
			1.23%	1 P:y	-5.263	0.67	31 C	
			1.22%	1 P:x	-5.263	0.67	24 C	
			1.21%	1 S	-6.532	1.00	89 H	
			1.20%	1 P:y	-5.263	0.67	23 C	
			1.20%	1 P:y	-5.263	0.67	36 C	
			1.10%	1 S	-6.532	1.00	69 H	
			1.06%	1 P:z	-9.066	1.33	6 O	
-13.481	2.00	41 A	10.09%	1 P:z	-9.066	1.33	2 O	
			5.12%	1 P:x	-9.066	1.33	4 O	
			4.15%	1 S	-13.631	2.00	19 C	

			3.09%	1 P:x	-5.263	0.67	23 C	
			2.37%	1 S	-6.532	1.00	89 H	
			2.35%	1 S	-6.532	1.00	58 H	
			2.33%	1 S	-6.532	1.00	44 H	
			2.04%	1 S	-6.532	1.00	52 H	
			1.92%	1 S	-6.532	1.00	60 H	
			1.88%	1 S	-6.532	1.00	57 H	
			1.73%	1 P:x	-9.066	1.33	1 O	
			1.70%	1 P:x	-5.263	0.67	27 C	
			1.58%	1 P:x	-5.263	0.67	22 C	
			1.49%	1 S	-6.532	1.00	51 H	
			1.43%	1 P:z	-5.263	0.67	33 C	
			1.43%	1 P:y	-9.066	1.33	5 O	
			1.42%	1 S	-23.822	2.00	4 O	
			1.40%	1 S	-6.532	1.00	67 H	
			1.32%	1 P:y	-5.263	0.67	39 C	
			1.25%	1 S	-6.532	1.00	88 H	
			1.24%	1 P:z	-9.066	1.33	4 O	
			1.20%	1 P:z	-5.263	0.67	21 C	
			1.15%	1 S	-13.631	2.00	39 C	
			1.14%	1 S	-6.532	1.00	54 H	
			1.12%	1 S	-13.631	2.00	27 C	
			1.10%	1 P:x	-5.263	0.67	39 C	
			1.10%	1 S	-6.532	1.00	53 H	
			1.07%	1 P:z	-9.066	1.33	1 O	
-13.288	2.00	42 A	9.26%	1 P:z	-9.066	1.33	2 O	
			6.92%	1 P:x	-5.263	0.67	23 C	
			5.16%	1 P:z	-9.066	1.33	5 O	
			4.02%	1 P:x	-9.066	1.33	9 O	
			3.98%	1 P:y	-5.263	0.67	17 C	
			3.77%	1 P:x	-5.263	0.67	33 C	

			3.31%	1 S	-6.532	1.00	89 H
			2.75%	1 P:x	-9.066	1.33	4 O
			2.11%	1 P:x	-5.263	0.67	16 C
			1.96%	1 P:z	-5.263	0.67	16 C
			1.91%	1 S	-13.631	2.00	14 C
			1.89%	1 P:x	-9.066	1.33	2 O
			1.60%	1 P:x	-5.263	0.67	22 C
			1.59%	1 P:z	-9.066	1.33	1 O
			1.49%	1 P:y	-9.066	1.33	5 O
			1.49%	1 P:x	-9.066	1.33	1 O
			1.36%	1 P:x	-9.066	1.33	5 O
			1.36%	1 P:y	-5.263	0.67	31 C
			1.11%	1 S	-6.532	1.00	67 H
			1.10%	1 P:z	-5.263	0.67	11 C
			1.06%	1 P:y	-9.066	1.33	3 O
-12.985	2.00	43 A	9.20%	1 P:x	-5.263	0.67	24 C
			8.86%	1 S	-6.532	1.00	86 H
			7.35%	1 S	-6.532	1.00	69 H
			5.47%	1 P:x	-5.263	0.67	18 C
			4.82%	1 P:z	-9.066	1.33	1 O
			3.11%	1 P:x	-9.066	1.33	6 O
			2.85%	1 P:z	-5.263	0.67	18 C
			2.65%	1 P:y	-9.066	1.33	1 O
			2.26%	1 P:z	-5.263	0.67	11 C
			2.10%	1 P:z	-5.263	0.67	24 C
			2.09%	1 P:y	-5.263	0.67	18 C
			2.07%	1 S	-6.532	1.00	52 H
			1.89%	1 P:x	-5.263	0.67	20 C
			1.81%	1 P:x	-5.263	0.67	12 C
			1.69%	1 S	-6.532	1.00	70 H
			1.59%	1 S	-13.631	2.00	14 C

			1.49%	1 P:x	-5.263	0.67	14	C
			1.37%	1 S	-13.631	2.00	27	C
			1.30%	1 P:x	-9.066	1.33	7	O
			1.20%	1 P:x	-9.066	1.33	4	O
			1.17%	1 S	-23.822	2.00	6	O
			1.07%	1 P:z	-5.263	0.67	25	C
-12.763	2.00	44 A	8.97%	1 P:y	-9.066	1.33	3	O
			5.65%	1 P:z	-5.263	0.67	29	C
			4.18%	1 P:y	-5.263	0.67	11	C
			4.17%	1 S	-6.532	1.00	76	H
			4.12%	1 P:z	-9.066	1.33	1	O
			3.41%	1 P:z	-9.066	1.33	3	O
			2.81%	1 P:y	-9.066	1.33	1	O
			2.74%	1 P:x	-5.263	0.67	17	C
			2.47%	1 P:x	-5.263	0.67	18	C
			2.32%	1 S	-6.532	1.00	90	H
			1.93%	1 P:y	-5.263	0.67	24	C
			1.90%	1 P:z	-5.263	0.67	30	C
			1.80%	1 P:y	-5.263	0.67	29	C
			1.43%	1 P:z	-5.263	0.67	33	C
			1.43%	1 S	-6.532	1.00	86	H
			1.42%	1 P:x	-5.263	0.67	28	C
			1.39%	1 P:y	-5.263	0.67	18	C
			1.24%	1 P:y	-5.263	0.67	16	C
			1.20%	1 P:y	-9.066	1.33	9	O
			1.15%	1 S	-6.532	1.00	94	H
			1.13%	1 S	-6.532	1.00	74	H
			1.08%	1 P:z	-9.066	1.33	2	O
			1.06%	1 S	-6.532	1.00	60	H
			1.05%	1 S	-6.532	1.00	77	H
			1.00%	1 P:z	-5.263	0.67	24	C

-12.558	2.00	45 A	4.61%	1 S	-6.532	1.00	76 H
			4.59%	1 P:z	-5.263	0.67	29 C
			3.73%	1 S	-6.532	1.00	90 H
			3.06%	1 P:z	-9.066	1.33	3 O
			2.90%	1 P:y	-5.263	0.67	11 C
			2.46%	1 S	-13.631	2.00	14 C
			2.35%	1 S	-6.532	1.00	85 H
			2.32%	1 S	-6.532	1.00	60 H
			2.27%	1 P:x	-5.263	0.67	17 C
			2.17%	1 P:z	-5.263	0.67	33 C
			2.07%	1 P:y	-5.263	0.67	17 C
			2.03%	1 P:y	-9.066	1.33	3 O
			1.81%	1 S	-6.532	1.00	62 H
			1.67%	1 P:x	-5.263	0.67	12 C
			1.59%	1 S	-6.532	1.00	69 H
			1.57%	1 P:z	-5.263	0.67	37 C
			1.52%	1 P:z	-5.263	0.67	28 C
			1.50%	1 P:x	-5.263	0.67	24 C
			1.44%	1 P:z	-5.263	0.67	11 C
			1.33%	1 S	-6.532	1.00	77 H
			1.27%	1 P:y	-9.066	1.33	9 O
			1.25%	1 S	-6.532	1.00	94 H
			1.22%	1 P:x	-9.066	1.33	7 O
			1.18%	1 S	-6.532	1.00	73 H
			1.18%	1 P:x	-9.066	1.33	6 O
			1.10%	1 S	-23.822	2.00	6 O
			1.10%	1 S	-13.631	2.00	24 C
			1.02%	1 P:z	-5.263	0.67	30 C
			1.01%	1 P:y	-5.263	0.67	32 C
-12.504	2.00	46 A	7.14%	1 P:y	-9.066	1.33	5 O
			4.73%	1 P:z	-9.066	1.33	9 O

			4.55%	1 P:z	-9.066	1.33	5 O	
			3.45%	1 P:y	-5.263	0.67	37 C	
			3.29%	1 P:y	-5.263	0.67	16 C	
			3.09%	1 P:x	-5.263	0.67	25 C	
			2.83%	1 P:z	-5.263	0.67	19 C	
			2.61%	1 S	-6.532	1.00	61 H	
			2.43%	1 P:x	-9.066	1.33	4 O	
			2.04%	1 S	-23.822	2.00	5 O	
			1.99%	1 S	-6.532	1.00	51 H	
			1.82%	1 P:x	-5.263	0.67	27 C	
			1.77%	1 S	-13.631	2.00	35 C	
			1.72%	1 P:x	-5.263	0.67	17 C	
			1.68%	1 S	-23.822	2.00	9 O	
			1.66%	1 P:y	-9.066	1.33	3 O	
			1.65%	1 P:x	-9.066	1.33	1 O	
			1.57%	1 P:x	-5.263	0.67	12 C	
			1.56%	1 S	-13.631	2.00	33 C	
			1.45%	1 P:x	-9.066	1.33	7 O	
			1.30%	1 S	-6.532	1.00	57 H	
			1.20%	1 S	-6.532	1.00	63 H	
			1.20%	1 P:y	-5.263	0.67	33 C	
			1.06%	1 P:y	-5.263	0.67	39 C	
-12.319	2.00	47 A	6.89%	1 P:y	-9.066	1.33	5 O	
			3.68%	1 P:x	-9.066	1.33	4 O	
			3.62%	1 P:z	-5.263	0.67	19 C	
			3.55%	1 P:z	-9.066	1.33	9 O	
			3.46%	1 P:x	-9.066	1.33	1 O	
			3.35%	1 P:x	-5.263	0.67	12 C	
			3.09%	1 P:x	-5.263	0.67	11 C	
			2.73%	1 P:x	-9.066	1.33	9 O	
			2.70%	1 S	-23.822	2.00	9 O	

			2.69%	1 P:x	-9.066	1.33	7 O
			2.51%	1 P:z	-5.263	0.67	18 C
			2.47%	1 P:y	-5.263	0.67	16 C
			2.12%	1 P:y	-5.263	0.67	37 C
			2.04%	1 P:z	-5.263	0.67	13 C
			1.89%	1 S	-6.532	1.00	61 H
			1.89%	1 S	-6.532	1.00	88 H
			1.88%	1 S	-6.532	1.00	85 H
			1.76%	1 S	-6.532	1.00	57 H
			1.50%	1 P:y	-5.263	0.67	39 C
			1.43%	1 S	-23.822	2.00	5 O
			1.36%	1 P:x	-5.263	0.67	25 C
			1.34%	1 P:y	-5.263	0.67	35 C
			1.31%	1 P:x	-5.263	0.67	13 C
			1.29%	1 P:z	-9.066	1.33	5 O
			1.05%	1 P:z	-7.073	1.00	43 N
-12.188	2.00	48 A	5.82%	1 P:y	-5.263	0.67	17 C
			5.23%	1 P:z	-5.263	0.67	14 C
			3.79%	1 P:x	-5.263	0.67	24 C
			2.39%	1 P:z	-5.263	0.67	12 C
			2.39%	1 P:x	-7.073	1.00	43 N
			2.31%	1 P:x	-5.263	0.67	20 C
			2.27%	1 S	-6.532	1.00	96 H
			2.23%	1 P:x	-9.066	1.33	6 O
			1.99%	1 P:x	-5.263	0.67	14 C
			1.82%	1 P:z	-9.066	1.33	6 O
			1.81%	1 S	-6.532	1.00	71 H
			1.79%	1 P:x	-5.263	0.67	16 C
			1.58%	1 P:z	-9.066	1.33	7 O
			1.58%	1 S	-6.532	1.00	69 H
			1.33%	1 P:z	-5.263	0.67	37 C

			1.32%	1 S	-6.532	1.00	62 H	
			1.25%	1 P:y	-5.263	0.67	31 C	
			1.24%	1 P:y	-9.066	1.33	4 O	
			1.16%	1 P:y	-9.066	1.33	9 O	
			1.16%	1 S	-6.532	1.00	83 H	
			1.15%	1 P:z	-5.263	0.67	13 C	
			1.14%	1 P:y	-5.263	0.67	32 C	
			1.13%	1 P:z	-5.263	0.67	24 C	
			1.13%	1 P:y	-9.066	1.33	10 O	
			1.10%	1 S	-6.532	1.00	87 H	
			1.09%	1 P:y	-5.263	0.67	16 C	
			1.09%	1 P:x	-5.263	0.67	22 C	
			1.08%	1 S	-6.532	1.00	80 H	
			1.05%	1 P:x	-5.263	0.67	29 C	
			1.00%	1 P:x	-5.263	0.67	13 C	
-12.094	2.00	49 A	12.06%	1 P:y	-9.066	1.33	9 O	
			9.79%	1 S	-23.822	2.00	9 O	
			8.80%	1 P:x	-9.066	1.33	5 O	
			8.48%	1 P:x	-9.066	1.33	9 O	
			4.10%	1 P:z	-9.066	1.33	9 O	
			2.71%	1 P:z	-5.263	0.67	33 C	
			2.63%	1 P:z	-5.263	0.67	16 C	
			2.43%	1 S	-13.631	2.00	16 C	
			2.28%	1 P:x	-5.263	0.67	22 C	
			2.12%	1 P:x	-5.263	0.67	17 C	
			1.92%	1 P:x	-5.263	0.67	16 C	
			1.79%	1 S	-6.532	1.00	44 H	
			1.64%	1 S	-6.532	1.00	60 H	
			1.63%	2 P:z	4.578	0.00	16 C	
			1.45%	1 P:z	-9.066	1.33	5 O	
			1.39%	1 P:y	-5.263	0.67	33 C	

			1.19%	1 P:y	-5.263	0.67	31 C
-11.938	2.00	50 A	6.14%	1 P:x	-5.263	0.67	25 C
			5.37%	1 P:y	-9.066	1.33	10 O
			4.63%	1 P:y	-5.263	0.67	42 C
			3.32%	1 S	-6.532	1.00	51 H
			3.15%	1 S	-6.532	1.00	85 H
			3.11%	1 S	-6.532	1.00	92 H
			2.34%	1 S	-23.822	2.00	10 O
			2.31%	1 P:x	-9.066	1.33	8 O
			2.23%	1 P:y	-5.263	0.67	35 C
			2.12%	1 S	-13.631	2.00	41 C
			2.10%	1 P:z	-5.263	0.67	41 C
			1.75%	1 P:z	-9.066	1.33	3 O
			1.52%	1 S	-13.631	2.00	35 C
			1.49%	1 P:x	-9.066	1.33	5 O
			1.47%	1 P:x	-5.263	0.67	15 C
			1.25%	1 P:y	-5.263	0.67	13 C
			1.16%	1 S	-23.822	2.00	8 O
			1.10%	1 P:z	-5.263	0.67	11 C
			1.09%	1 P:z	-9.066	1.33	4 O
			1.08%	1 S	-6.532	1.00	86 H
			1.07%	1 S	-6.532	1.00	88 H
			1.03%	1 P:x	-5.263	0.67	27 C
			1.03%	1 P:y	-5.263	0.67	41 C
			1.03%	1 P:y	-7.073	1.00	43 N
-11.885	2.00	51 A	7.32%	1 P:y	-9.066	1.33	10 O
			5.00%	1 P:y	-5.263	0.67	42 C
			4.62%	1 P:z	-9.066	1.33	7 O
			4.54%	1 P:x	-5.263	0.67	25 C
			2.83%	1 P:y	-5.263	0.67	41 C
			2.83%	1 P:z	-5.263	0.67	13 C

			2.75%	1 S	-6.532	1.00	92 H
			2.74%	1 S	-6.532	1.00	51 H
			2.27%	1 S	-13.631	2.00	41 C
			2.27%	1 P:z	-9.066	1.33	4 O
			2.05%	1 S	-23.822	2.00	10 O
			2.00%	1 P:x	-5.263	0.67	42 C
			1.81%	1 P:x	-5.263	0.67	41 C
			1.76%	1 P:z	-7.073	1.00	43 N
			1.68%	1 P:z	-5.263	0.67	12 C
			1.55%	1 P:y	-5.263	0.67	35 C
			1.53%	1 P:x	-9.066	1.33	6 O
			1.48%	1 S	-6.532	1.00	93 H
			1.31%	1 P:z	-5.263	0.67	39 C
			1.29%	1 S	-6.532	1.00	69 H
			1.21%	1 P:x	-5.263	0.67	19 C
			1.19%	1 P:y	-5.263	0.67	31 C
			1.15%	1 P:y	-5.263	0.67	28 C
			1.14%	1 S	-6.532	1.00	44 H
			1.13%	1 P:z	-9.066	1.33	1 O
			1.09%	1 P:x	-5.263	0.67	24 C
			1.09%	1 P:x	-5.263	0.67	15 C
			1.02%	1 P:x	-5.263	0.67	12 C
-11.809	2.00	52 A	6.96%	1 P:y	-9.066	1.33	7 O
			6.17%	1 S	-23.822	2.00	10 O
			6.04%	1 P:y	-9.066	1.33	10 O
			4.11%	1 S	-6.532	1.00	92 H
			3.85%	1 P:x	-9.066	1.33	10 O
			3.05%	1 P:x	-5.263	0.67	13 C
			2.98%	1 P:y	-5.263	0.67	42 C
			2.75%	1 S	-13.631	2.00	41 C
			2.63%	1 P:x	-7.073	1.00	43 N

			2.63%	1 P:z	-5.263	0.67	41 C
			2.48%	1 P:x	-5.263	0.67	29 C
			2.45%	1 P:x	-9.066	1.33	3 O
			2.07%	1 P:y	-5.263	0.67	26 C
			2.02%	1 P:x	-5.263	0.67	28 C
			1.97%	1 S	-6.532	1.00	80 H
			1.78%	1 P:z	-5.263	0.67	12 C
			1.78%	1 S	-6.532	1.00	83 H
			1.71%	1 P:y	-7.073	1.00	43 N
			1.71%	1 S	-6.532	1.00	78 H
			1.67%	1 P:z	-5.263	0.67	28 C
			1.47%	1 S	-6.532	1.00	85 H
			1.41%	1 P:z	-9.066	1.33	10 O
			1.38%	1 P:y	-5.263	0.67	38 C
			1.33%	1 P:x	-5.263	0.67	19 C
			1.26%	1 P:z	-5.263	0.67	42 C
-11.722	2.00	53 A	4.29%	1 P:x	-9.066	1.33	4 O
			3.42%	1 P:y	-5.263	0.67	39 C
			2.96%	1 S	-6.532	1.00	57 H
			2.87%	1 P:y	-9.066	1.33	7 O
			2.15%	1 P:x	-5.263	0.67	25 C
			2.13%	1 S	-6.532	1.00	51 H
			2.01%	1 P:x	-9.066	1.33	10 O
			1.88%	1 P:z	-5.263	0.67	37 C
			1.80%	1 P:y	-9.066	1.33	4 O
			1.80%	1 P:y	-9.066	1.33	9 O
			1.80%	1 P:x	-5.263	0.67	19 C
			1.63%	1 P:z	-5.263	0.67	13 C
			1.56%	1 P:z	-7.073	1.00	43 N
			1.49%	1 P:z	-5.263	0.67	19 C
			1.47%	1 S	-6.532	1.00	82 H

			1.44%	1 P:z	-9.066	1.33	7 O	
			1.24%	1 P:z	-5.263	0.67	28 C	
			1.22%	1 S	-6.532	1.00	61 H	
			1.21%	1 P:x	-5.263	0.67	36 C	
			1.21%	1 P:z	-5.263	0.67	33 C	
			1.16%	1 P:x	-5.263	0.67	13 C	
			1.16%	1 S	-6.532	1.00	59 H	
			1.14%	1 P:z	-5.263	0.67	24 C	
			1.13%	1 P:z	-5.263	0.67	18 C	
			1.12%	1 S	-6.532	1.00	63 H	
			1.08%	1 P:x	-5.263	0.67	41 C	
			1.03%	1 P:z	-9.066	1.33	9 O	
			1.02%	1 P:y	-5.263	0.67	38 C	
			1.02%	1 P:z	-9.066	1.33	1 O	
-11.599	2.00	54 A	7.96%	1 S	-6.532	1.00	44 H	
			4.97%	1 P:z	-5.263	0.67	37 C	
			4.48%	1 P:x	-5.263	0.67	22 C	
			4.27%	1 P:z	-5.263	0.67	33 C	
			3.85%	1 P:x	-5.263	0.67	17 C	
			3.14%	1 P:x	-5.263	0.67	34 C	
			2.61%	1 S	-6.532	1.00	61 H	
			2.46%	1 P:y	-5.263	0.67	31 C	
			2.05%	1 P:z	-9.066	1.33	2 O	
			2.00%	1 P:x	-9.066	1.33	2 O	
			1.99%	1 P:z	-5.263	0.67	40 C	
			1.95%	1 S	-13.631	2.00	31 C	
			1.94%	1 S	-6.532	1.00	60 H	
			1.90%	1 P:y	-5.263	0.67	16 C	
			1.78%	1 S	-6.532	1.00	62 H	
			1.50%	1 P:z	-5.263	0.67	17 C	
			1.38%	1 S	-6.532	1.00	90 H	

			1.33%	1 P:x	-9.066	1.33	9 O
			1.32%	1 P:y	-5.263	0.67	22 C
			1.28%	1 S	-6.532	1.00	45 H
			1.28%	1 P:y	-9.066	1.33	6 O
			1.16%	1 S	-6.532	1.00	47 H
			1.07%	1 S	-6.532	1.00	63 H
			1.03%	1 S	-6.532	1.00	89 H
			1.02%	1 S	-23.822	2.00	6 O
			1.01%	1 S	-23.822	2.00	2 O
-11.493	2.00	55 A	4.87%	1 P:y	-9.066	1.33	4 O
			4.65%	1 P:y	-5.263	0.67	36 C
			4.13%	1 P:y	-5.263	0.67	19 C
			3.47%	1 P:y	-5.263	0.67	27 C
			2.61%	1 S	-6.532	1.00	55 H
			2.49%	1 P:x	-9.066	1.33	4 O
			2.17%	1 P:x	-9.066	1.33	5 O
			2.12%	1 P:z	-5.263	0.67	37 C
			2.10%	1 P:y	-5.263	0.67	14 C
			2.07%	1 P:y	-5.263	0.67	24 C
			2.06%	1 S	-6.532	1.00	58 H
			2.03%	1 S	-6.532	1.00	61 H
			1.96%	1 P:z	-9.066	1.33	6 O
			1.91%	1 P:z	-5.263	0.67	20 C
			1.88%	1 S	-6.532	1.00	53 H
			1.69%	1 P:x	-9.066	1.33	6 O
			1.66%	1 P:x	-5.263	0.67	39 C
			1.58%	1 S	-6.532	1.00	90 H
			1.57%	1 P:x	-5.263	0.67	16 C
			1.51%	1 P:y	-9.066	1.33	9 O
			1.51%	1 P:z	-5.263	0.67	17 C
			1.38%	1 S	-6.532	1.00	56 H

			1.34%	1	S	-13.631	2.00	19	C
			1.30%	1	P:z	-5.263	0.67	40	C
			1.20%	1	P:x	-5.263	0.67	32	C
			1.13%	1	P:z	-5.263	0.67	39	C
			1.11%	1	P:y	-9.066	1.33	5	O
			1.07%	1	S	-6.532	1.00	63	H
			1.04%	1	P:y	-5.263	0.67	40	C
-11.427	2.00	56 A	5.86%	1	P:y	-5.263	0.67	23	C
			5.26%	1	P:y	-5.263	0.67	22	C
			4.49%	1	P:y	-5.263	0.67	21	C
			3.75%	1	S	-6.532	1.00	66	H
			2.67%	1	P:x	-5.263	0.67	31	C
			2.42%	1	P:z	-9.066	1.33	2	O
			2.33%	1	S	-6.532	1.00	59	H
			2.22%	1	P:y	-5.263	0.67	13	C
			2.15%	1	S	-6.532	1.00	44	H
			1.90%	1	P:z	-9.066	1.33	4	O
			1.79%	1	P:y	-7.073	1.00	43	N
			1.75%	1	P:y	-9.066	1.33	8	O
			1.58%	1	P:z	-5.263	0.67	39	C
			1.57%	1	S	-23.822	2.00	8	O
			1.56%	1	S	-6.532	1.00	53	H
			1.51%	1	P:x	-5.263	0.67	30	C
			1.46%	1	P:x	-5.263	0.67	19	C
			1.45%	1	S	-6.532	1.00	84	H
			1.45%	1	P:x	-5.263	0.67	29	C
			1.30%	1	P:x	-5.263	0.67	38	C
			1.22%	1	S	-6.532	1.00	79	H
			1.20%	1	P:x	-5.263	0.67	36	C
			1.18%	1	S	-6.532	1.00	55	H
			1.18%	1	P:x	-5.263	0.67	33	C

			1.17%	1 P:y	-5.263	0.67	28 C	
			1.11%	1 P:z	-5.263	0.67	26 C	
			1.07%	1 S	-6.532	1.00	75 H	
			1.05%	1 P:y	-5.263	0.67	27 C	
			1.04%	1 P:x	-7.073	1.00	43 N	
			1.02%	1 P:x	-9.066	1.33	8 O	
-11.338	2.00	57 A	5.89%	1 P:z	-9.066	1.33	10 O	
			5.05%	1 P:y	-7.073	1.00	43 N	
			4.35%	1 P:x	-9.066	1.33	7 O	
			2.99%	1 P:y	-9.066	1.33	7 O	
			2.43%	1 P:y	-5.263	0.67	38 C	
			2.34%	1 P:y	-5.263	0.67	13 C	
			2.30%	1 S	-6.532	1.00	44 H	
			1.96%	1 P:y	-5.263	0.67	22 C	
			1.89%	1 P:y	-5.263	0.67	42 C	
			1.78%	1 S	-23.822	2.00	10 O	
			1.75%	1 S	-6.532	1.00	88 H	
			1.73%	1 P:x	-5.263	0.67	26 C	
			1.70%	1 P:y	-5.263	0.67	23 C	
			1.64%	1 S	-6.532	1.00	79 H	
			1.59%	1 P:x	-5.263	0.67	29 C	
			1.58%	1 S	-6.532	1.00	76 H	
			1.49%	1 P:y	-5.263	0.67	41 C	
			1.48%	1 P:x	-5.263	0.67	41 C	
			1.34%	1 S	-6.532	1.00	91 H	
			1.32%	1 P:x	-5.263	0.67	31 C	
			1.27%	1 P:x	-5.263	0.67	30 C	
			1.23%	1 P:x	-5.263	0.67	34 C	
			1.20%	1 P:z	-9.066	1.33	2 O	
			1.14%	1 S	-6.532	1.00	84 H	
			1.14%	1 S	-6.532	1.00	61 H	

			1.13%	1 S	-6.532	1.00	85	H
			1.13%	1 P:z	-9.066	1.33	7	O
			1.12%	1 P:y	-5.263	0.67	37	C
			1.09%	1 S	-6.532	1.00	63	H
			1.06%	1 P:z	-5.263	0.67	37	C
			1.04%	1 S	-23.822	2.00	7	O
			1.01%	1 P:y	-5.263	0.67	21	C
-11.260	2.00	58 A	8.59%	1 P:x	-7.073	1.00	43	N
			6.60%	1 P:x	-5.263	0.67	38	C
			4.40%	1 P:y	-5.263	0.67	28	C
			3.61%	1 P:z	-5.263	0.67	26	C
			3.50%	1 S	-6.532	1.00	84	H
			3.27%	1 P:x	-5.263	0.67	30	C
			3.00%	1 S	-6.532	1.00	80	H
			2.88%	1 S	-6.532	1.00	83	H
			2.85%	1 P:z	-9.066	1.33	1	O
			2.80%	1 S	-6.532	1.00	75	H
			2.68%	1 P:y	-5.263	0.67	13	C
			2.38%	1 P:x	-5.263	0.67	13	C
			2.36%	1 P:x	-5.263	0.67	29	C
			2.35%	1 P:y	-5.263	0.67	26	C
			2.14%	1 P:x	-9.066	1.33	3	O
			1.69%	1 P:x	-5.263	0.67	41	C
			1.57%	1 P:z	-9.066	1.33	10	O
			1.49%	1 P:x	-9.066	1.33	7	O
			1.40%	1 P:y	-9.066	1.33	3	O
			1.27%	1 P:x	-9.066	1.33	6	O
			1.24%	1 S	-6.532	1.00	79	H
			1.09%	1 P:y	-5.263	0.67	39	C
			1.04%	1 P:x	-5.263	0.67	42	C
-11.206	2.00	59 A	4.12%	1 P:y	-5.263	0.67	27	C

			3.48%	1 P:y	-5.263	0.67	19	C
			3.20%	1 P:z	-9.066	1.33	10	O
			2.61%	1 P:y	-9.066	1.33	4	O
			2.51%	1 P:z	-5.263	0.67	36	C
			2.49%	1 P:y	-5.263	0.67	17	C
			2.48%	1 S	-6.532	1.00	55	H
			2.33%	1 P:x	-9.066	1.33	7	O
			2.26%	1 P:y	-5.263	0.67	37	C
			2.25%	1 P:x	-5.263	0.67	41	C
			2.23%	1 S	-6.532	1.00	61	H
			2.18%	1 S	-6.532	1.00	53	H
			1.97%	1 S	-6.532	1.00	44	H
			1.92%	1 S	-6.532	1.00	90	H
			1.84%	1 P:z	-5.263	0.67	18	C
			1.70%	1 P:x	-5.263	0.67	32	C
			1.59%	1 P:y	-5.263	0.67	40	C
			1.48%	1 P:x	-5.263	0.67	42	C
			1.28%	1 P:y	-5.263	0.67	42	C
			1.23%	1 P:y	-5.263	0.67	36	C
			1.23%	1 P:x	-5.263	0.67	34	C
			1.21%	1 S	-6.532	1.00	85	H
			1.20%	1 S	-6.532	1.00	58	H
			1.17%	1 S	-6.532	1.00	96	H
			1.16%	1 P:z	-5.263	0.67	37	C
			1.13%	1 S	-6.532	1.00	63	H
			1.10%	1 S	-6.532	1.00	91	H
			1.10%	1 P:x	-5.263	0.67	22	C
			1.09%	1 P:z	-5.263	0.67	14	C
			1.06%	1 P:x	-5.263	0.67	20	C
-11.131	2.00	60 A	4.50%	1 P:y	-5.263	0.67	24	C
			3.40%	1 P:y	-5.263	0.67	18	C

2.66%	1 P:y	-5.263	0.67	35	C
2.31%	1 P:y	-5.263	0.67	31	C
2.27%	1 P:x	-5.263	0.67	25	C
2.23%	1 P:x	-9.066	1.33	8	O
2.02%	1 S	-6.532	1.00	69	H
1.76%	1 P:y	-5.263	0.67	27	C
1.72%	1 P:y	-5.263	0.67	14	C
1.70%	1 P:y	-5.263	0.67	40	C
1.68%	1 S	-6.532	1.00	65	H
1.67%	1 S	-6.532	1.00	59	H
1.65%	1 P:z	-5.263	0.67	20	C
1.63%	1 P:x	-5.263	0.67	42	C
1.61%	1 S	-6.532	1.00	53	H
1.51%	1 S	-6.532	1.00	70	H
1.49%	1 S	-6.532	1.00	66	H
1.45%	1 P:y	-5.263	0.67	21	C
1.45%	1 S	-6.532	1.00	86	H
1.42%	1 P:x	-5.263	0.67	15	C
1.40%	1 P:x	-5.263	0.67	36	C
1.38%	1 P:x	-9.066	1.33	6	O
1.37%	1 P:x	-5.263	0.67	33	C
1.37%	1 P:y	-5.263	0.67	20	C
1.35%	1 S	-6.532	1.00	93	H
1.32%	1 S	-6.532	1.00	50	H
1.29%	1 P:x	-5.263	0.67	39	C
1.26%	1 S	-6.532	1.00	55	H
1.26%	1 P:z	-5.263	0.67	19	C
1.26%	1 S	-6.532	1.00	72	H
1.24%	1 P:y	-5.263	0.67	23	C
1.21%	1 P:z	-5.263	0.67	24	C
1.15%	1 P:y	-9.066	1.33	4	O

1.14% 1 P:x -5.263 0.67 22 C

1.12% 1 S -6.532 1.00 51 H

1.06% 1 P:x -5.263 0.67 41 C

1.03% 1 P:y -5.263 0.67 34 C

1.02% 1 P:y -9.066 1.33 8 O

-10.939 2.00 61 A 11.92% 1 P:x -5.263 0.67 42 C

9.52% 1 S -6.532 1.00 93 H

7.84% 1 P:x -5.263 0.67 41 C

6.15% 1 P:x -9.066 1.33 10 O

2.86% 1 P:y -9.066 1.33 4 O

2.71% 1 S -6.532 1.00 59 H

2.55% 1 P:x -5.263 0.67 39 C

2.53% 1 P:x -5.263 0.67 11 C

2.33% 1 P:z -7.073 1.00 43 N

2.32% 1 P:z -5.263 0.67 42 C

2.15% 1 P:y -5.263 0.67 41 C

2.11% 1 S -6.532 1.00 91 H

1.79% 1 P:z -5.263 0.67 13 C

1.68% 1 P:y -5.263 0.67 26 C

1.61% 1 P:z -5.263 0.67 38 C

1.45% 1 P:x -5.263 0.67 12 C

1.12% 1 S -6.532 1.00 78 H

1.11% 1 S -6.532 1.00 80 H

1.05% 1 S -6.532 1.00 83 H

1.04% 1 P:x -5.263 0.67 36 C

1.02% 1 P:z -9.066 1.33 7 O

-10.814 2.00 62 A 6.37% 1 P:z -9.066 1.33 6 O

3.46% 1 S -6.532 1.00 59 H

3.18% 1 P:x -9.066 1.33 9 O

3.12% 1 P:x -5.263 0.67 20 C

2.98% 1 P:x -9.066 1.33 4 O

			2.86%	1 P:x	-9.066	1.33	6 O
			2.82%	1 P:z	-9.066	1.33	4 O
			2.70%	1 P:x	-5.263	0.67	39 C
			2.49%	1 S	-6.532	1.00	71 H
			2.10%	1 S	-23.822	2.00	6 O
			2.08%	1 P:y	-9.066	1.33	4 O
			1.87%	1 P:y	-5.263	0.67	14 C
			1.85%	1 P:z	-5.263	0.67	38 C
			1.64%	1 P:y	-5.263	0.67	36 C
			1.61%	1 P:z	-5.263	0.67	39 C
			1.51%	1 S	-6.532	1.00	64 H
			1.48%	1 P:y	-5.263	0.67	37 C
			1.48%	1 P:z	-5.263	0.67	40 C
			1.41%	1 P:y	-5.263	0.67	26 C
			1.41%	1 P:x	-9.066	1.33	8 O
			1.34%	1 P:x	-9.066	1.33	5 O
			1.34%	1 P:y	-5.263	0.67	19 C
			1.33%	1 P:z	-7.073	1.00	43 N
			1.32%	1 P:x	-5.263	0.67	24 C
			1.25%	1 P:y	-5.263	0.67	16 C
			1.25%	1 P:z	-5.263	0.67	23 C
			1.17%	1 P:z	-5.263	0.67	37 C
			1.15%	1 P:x	-5.263	0.67	21 C
			1.15%	1 P:y	-5.263	0.67	40 C
			1.13%	1 S	-6.532	1.00	62 H
			1.11%	1 S	-6.532	1.00	82 H
			1.09%	1 S	-6.532	1.00	68 H
			1.04%	1 S	-6.532	1.00	65 H
-10.768	2.00	63 A	8.34%	1 S	-6.532	1.00	58 H
			7.37%	1 P:z	-5.263	0.67	39 C
			6.08%	1 S	-6.532	1.00	57 H

			5.11%	1 P:y	-9.066	1.33	6 O		
			4.68%	1 P:y	-5.263	0.67	27 C		
			3.62%	1 P:y	-5.263	0.67	39 C		
			3.27%	1 S	-6.532	1.00	53 H		
			3.26%	1 P:z	-9.066	1.33	4 O		
			2.59%	1 P:z	-5.263	0.67	15 C		
			2.15%	1 S	-6.532	1.00	49 H		
			1.69%	1 P:y	-9.066	1.33	4 O		
			1.66%	1 P:x	-9.066	1.33	6 O		
			1.64%	1 P:y	-5.263	0.67	26 C		
			1.62%	1 P:y	-5.263	0.67	25 C		
			1.53%	1 P:y	-9.066	1.33	3 O		
			1.35%	1 P:x	-9.066	1.33	3 O		
			1.34%	1 P:z	-5.263	0.67	25 C		
			1.32%	1 S	-6.532	1.00	78 H		
			1.14%	1 S	-6.532	1.00	80 H		
			1.07%	1 P:z	-5.263	0.67	38 C		
			1.05%	1 P:z	-5.263	0.67	32 C		
			1.03%	1 S	-6.532	1.00	83 H		
			1.01%	1 P:z	-5.263	0.67	28 C		
			1.01%	1 P:z	-9.066	1.33	1 O		
-10.686	2.00	64 A	5.93%	1 S	-6.532	1.00	78 H		
			4.16%	1 P:y	-7.073	1.00	43 N		
			4.08%	1 P:z	-9.066	1.33	10 O		
			3.59%	1 P:z	-5.263	0.67	28 C		
			3.03%	1 S	-6.532	1.00	87 H		
			3.00%	1 P:x	-9.066	1.33	3 O		
			2.77%	1 P:y	-5.263	0.67	41 C		
			2.58%	1 P:y	-5.263	0.67	42 C		
			2.46%	1 P:x	-5.263	0.67	29 C		
			2.37%	1 S	-6.532	1.00	91 H		

			2.31%	1 P:z	-5.263	0.67	42 C	
			2.26%	1 P:y	-5.263	0.67	38 C	
			2.23%	1 P:y	-9.066	1.33	10 O	
			2.11%	1 S	-23.822	2.00	10 O	
			2.06%	2 P:y	4.578	0.00	41 C	
			1.81%	1 S	-6.532	1.00	58 H	
			1.80%	1 S	-6.532	1.00	79 H	
			1.56%	1 P:x	-9.066	1.33	10 O	
			1.44%	1 P:x	-5.263	0.67	28 C	
			1.44%	1 S	-13.631	2.00	12 C	
			1.43%	1 P:y	-9.066	1.33	4 O	
			1.31%	1 P:x	-5.263	0.67	39 C	
			1.27%	1 S	-6.532	1.00	57 H	
			1.26%	1 P:y	-9.066	1.33	3 O	
			1.25%	1 P:x	-5.263	0.67	26 C	
			1.22%	1 P:x	-9.066	1.33	1 O	
			1.15%	1 P:z	-5.263	0.67	26 C	
			1.15%	1 P:x	-5.263	0.67	30 C	
			1.02%	1 P:z	-5.263	0.67	39 C	
-10.652	2.00	65 A	4.27%	1 P:z	-5.263	0.67	40 C	
			3.43%	1 P:z	-5.263	0.67	23 C	
			3.24%	1 P:x	-5.263	0.67	21 C	
			3.17%	1 S	-6.532	1.00	64 H	
			2.96%	1 P:z	-5.263	0.67	38 C	
			2.89%	1 P:z	-5.263	0.67	37 C	
			2.88%	1 P:x	-9.066	1.33	5 O	
			2.83%	1 P:x	-9.066	1.33	9 O	
			2.68%	1 S	-6.532	1.00	68 H	
			2.43%	1 S	-6.532	1.00	79 H	
			1.99%	1 P:y	-9.066	1.33	2 O	
			1.92%	1 S	-6.532	1.00	62 H	

			1.87%	1 S	-6.532	1.00	59 H
			1.71%	1 P:z	-5.263	0.67	17 C
			1.70%	1 P:y	-5.263	0.67	26 C
			1.68%	1 S	-6.532	1.00	82 H
			1.65%	1 P:z	-5.263	0.67	26 C
			1.62%	1 P:z	-5.263	0.67	33 C
			1.48%	1 P:y	-5.263	0.67	32 C
			1.44%	1 P:z	-7.073	1.00	43 N
			1.43%	1 S	-6.532	1.00	63 H
			1.34%	1 P:z	-5.263	0.67	39 C
			1.31%	1 P:y	-9.066	1.33	6 O
			1.29%	1 S	-6.532	1.00	78 H
			1.21%	1 S	-6.532	1.00	65 H
			1.13%	1 P:y	-5.263	0.67	16 C
			1.07%	1 P:y	-5.263	0.67	14 C
			1.06%	1 P:x	-9.066	1.33	4 O
			1.00%	1 P:y	-5.263	0.67	40 C
-10.468	2.00	66 A	6.35%	1 P:x	-9.066	1.33	6 O
			5.48%	1 P:z	-9.066	1.33	6 O
			3.20%	1 P:x	-5.263	0.67	20 C
			2.84%	1 P:z	-5.263	0.67	23 C
			2.71%	1 S	-23.822	2.00	6 O
			2.57%	1 P:z	-5.263	0.67	40 C
			2.49%	1 P:z	-5.263	0.67	38 C
			2.47%	1 S	-6.532	1.00	70 H
			2.28%	1 S	-6.532	1.00	64 H
			2.25%	1 S	-6.532	1.00	82 H
			2.14%	1 S	-6.532	1.00	71 H
			1.80%	1 S	-6.532	1.00	66 H
			1.78%	1 P:z	-5.263	0.67	37 C
			1.69%	1 S	-6.532	1.00	96 H

			1.52%	1 P:y	-5.263	0.67	21 C
			1.50%	1 P:z	-7.073	1.00	43 N
			1.49%	1 P:x	-9.066	1.33	2 O
			1.44%	1 P:y	-5.263	0.67	17 C
			1.41%	1 S	-6.532	1.00	79 H
			1.40%	1 P:x	-5.263	0.67	24 C
			1.32%	1 S	-6.532	1.00	60 H
			1.25%	1 P:y	-5.263	0.67	14 C
			1.18%	1 P:y	-9.066	1.33	6 O
			1.17%	1 P:z	-5.263	0.67	26 C
			1.14%	1 P:z	-5.263	0.67	33 C
			1.03%	1 P:z	-5.263	0.67	32 C
			1.03%	1 P:z	-5.263	0.67	24 C
			1.02%	1 P:y	-5.263	0.67	20 C
			1.00%	1 P:z	-5.263	0.67	15 C
-10.420	2.00	67 A	6.23%	1 S	-6.532	1.00	82 H
			5.63%	1 P:z	-5.263	0.67	38 C
			3.58%	1 P:z	-7.073	1.00	43 N
			2.97%	1 P:z	-5.263	0.67	15 C
			2.61%	1 S	-6.532	1.00	49 H
			2.59%	1 S	-6.532	1.00	59 H
			2.59%	1 P:z	-5.263	0.67	39 C
			2.51%	1 P:z	-5.263	0.67	25 C
			2.27%	1 S	-6.532	1.00	81 H
			2.25%	1 S	-6.532	1.00	79 H
			2.00%	1 P:x	-5.263	0.67	11 C
			1.98%	1 P:z	-5.263	0.67	35 C
			1.79%	1 P:z	-5.263	0.67	27 C
			1.76%	1 P:z	-5.263	0.67	26 C
			1.73%	1 P:x	-5.263	0.67	12 C
			1.47%	1 S	-6.532	1.00	58 H

			1.34%	1 P:y	-5.263	0.67	38 C
			1.31%	1 S	-6.532	1.00	84 H
			1.30%	1 P:z	-9.066	1.33	8 O
			1.30%	1 P:y	-5.263	0.67	26 C
			1.23%	1 S	-6.532	1.00	72 H
			1.22%	1 S	-6.532	1.00	48 H
			1.17%	1 P:x	-5.263	0.67	26 C
			1.15%	1 P:y	-5.263	0.67	20 C
			1.05%	1 P:z	-5.263	0.67	17 C
			1.01%	1 P:y	-5.263	0.67	29 C
-10.258	2.00	68 A	3.72%	1 P:z	-5.263	0.67	34 C
			3.43%	1 P:z	-5.263	0.67	42 C
			3.38%	1 S	-6.532	1.00	92 H
			3.26%	1 P:z	-5.263	0.67	31 C
			2.99%	1 P:y	-9.066	1.33	9 O
			2.88%	1 S	-6.532	1.00	45 H
			2.70%	1 P:y	-9.066	1.33	5 O
			2.65%	1 S	-6.532	1.00	96 H
			2.48%	1 P:x	-9.066	1.33	8 O
			2.44%	1 P:y	-5.263	0.67	16 C
			2.28%	1 P:x	-9.066	1.33	9 O
			1.99%	1 P:x	-5.263	0.67	32 C
			1.88%	1 P:y	-9.066	1.33	8 O
			1.75%	1 S	-6.532	1.00	91 H
			1.70%	1 P:z	-5.263	0.67	35 C
			1.69%	1 S	-6.532	1.00	47 H
			1.45%	1 P:z	-5.263	0.67	40 C
			1.41%	1 P:y	-5.263	0.67	32 C
			1.37%	1 P:x	-5.263	0.67	16 C
			1.34%	1 P:z	-9.066	1.33	10 O
			1.19%	1 P:y	-9.066	1.33	6 O

			1.12%	1 S	-23.822	2.00	8 O
			1.12%	1 P:z	-5.263	0.67	28 C
			1.12%	1 P:x	-9.066	1.33	5 O
			1.11%	1 S	-6.532	1.00	94 H
			1.09%	1 S	-6.532	1.00	63 H
			1.08%	1 P:y	-5.263	0.67	29 C
			1.07%	1 P:x	-9.066	1.33	6 O
-10.204	2.00	69 A	4.42%	1 S	-6.532	1.00	92 H
			4.39%	1 P:z	-5.263	0.67	42 C
			3.20%	1 P:y	-9.066	1.33	9 O
			3.15%	1 P:y	-9.066	1.33	5 O
			2.95%	1 P:z	-5.263	0.67	40 C
			2.94%	1 P:y	-5.263	0.67	29 C
			2.40%	1 P:x	-9.066	1.33	6 O
			2.40%	1 S	-6.532	1.00	75 H
			2.36%	1 S	-6.532	1.00	91 H
			2.29%	1 P:x	-9.066	1.33	9 O
			2.23%	1 P:y	-5.263	0.67	30 C
			2.22%	1 P:y	-5.263	0.67	16 C
			2.13%	1 P:z	-5.263	0.67	28 C
			1.84%	1 P:y	-7.073	1.00	43 N
			1.67%	1 S	-6.532	1.00	64 H
			1.62%	1 P:x	-5.263	0.67	30 C
			1.60%	1 P:z	-9.066	1.33	3 O
			1.55%	1 P:y	-5.263	0.67	32 C
			1.48%	1 P:x	-5.263	0.67	16 C
			1.37%	1 S	-6.532	1.00	94 H
			1.37%	1 S	-6.532	1.00	63 H
			1.35%	1 S	-6.532	1.00	61 H
			1.21%	1 P:z	-5.263	0.67	37 C
			1.21%	1 P:y	-9.066	1.33	6 O

			1.17%	1 S	-6.532	1.00	57 H
			1.16%	1 S	-6.532	1.00	96 H
			1.16%	1 P:z	-5.263	0.67	26 C
			1.13%	1 P:y	-5.263	0.67	38 C
			1.12%	1 P:y	-9.066	1.33	3 O
			1.10%	1 S	-6.532	1.00	78 H
			1.08%	1 S	-6.532	1.00	79 H
			1.06%	1 P:z	-9.066	1.33	10 O
			1.05%	1 S	-23.822	2.00	6 O
-10.162	2.00	70 A	8.65%	1 P:x	-9.066	1.33	8 O
			4.44%	1 P:y	-5.263	0.67	20 C
			4.39%	1 P:y	-9.066	1.33	8 O
			3.22%	1 S	-23.822	2.00	8 O
			3.20%	1 P:z	-5.263	0.67	35 C
			3.18%	1 S	-6.532	1.00	72 H
			2.59%	1 S	-6.532	1.00	75 H
			2.53%	1 P:z	-5.263	0.67	24 C
			2.48%	1 P:z	-5.263	0.67	31 C
			2.11%	1 P:x	-5.263	0.67	30 C
			1.78%	1 P:y	-9.066	1.33	5 O
			1.66%	1 P:y	-5.263	0.67	40 C
			1.63%	1 S	-6.532	1.00	57 H
			1.59%	1 P:y	-5.263	0.67	34 C
			1.48%	1 S	-6.532	1.00	65 H
			1.47%	1 P:y	-7.073	1.00	43 N
			1.36%	1 P:z	-5.263	0.67	28 C
			1.36%	1 P:y	-9.066	1.33	6 O
			1.25%	1 S	-6.532	1.00	45 H
			1.20%	1 P:y	-5.263	0.67	29 C
			1.12%	1 S	-6.532	1.00	46 H
			1.12%	1 P:z	-5.263	0.67	34 C

			1.06%	1 P:y	-5.263	0.67	39 C
-10.108	2.00	71 A	3.26%	1 P:z	-5.263	0.67	39 C
			3.22%	1 P:y	-9.066	1.33	6 O
			2.93%	1 P:z	-5.263	0.67	32 C
			2.85%	1 P:z	-5.263	0.67	17 C
			2.83%	1 S	-6.532	1.00	58 H
			2.81%	1 P:y	-5.263	0.67	32 C
			2.72%	1 P:x	-5.263	0.67	17 C
			2.67%	1 S	-6.532	1.00	94 H
			2.42%	1 S	-6.532	1.00	57 H
			2.41%	1 P:x	-9.066	1.33	8 O
			2.30%	1 S	-6.532	1.00	60 H
			2.21%	1 P:y	-5.263	0.67	14 C
			2.14%	1 P:z	-5.263	0.67	35 C
			1.96%	1 S	-6.532	1.00	64 H
			1.96%	1 P:z	-5.263	0.67	24 C
			1.70%	1 P:z	-5.263	0.67	15 C
			1.50%	1 P:x	-5.263	0.67	14 C
			1.50%	1 P:y	-5.263	0.67	20 C
			1.49%	1 S	-6.532	1.00	49 H
			1.39%	1 P:z	-5.263	0.67	18 C
			1.38%	1 P:x	-9.066	1.33	2 O
			1.33%	1 P:z	-9.066	1.33	9 O
			1.21%	1 P:x	-9.066	1.33	5 O
			1.17%	1 S	-6.532	1.00	48 H
			1.16%	1 P:y	-5.263	0.67	39 C
			1.14%	1 P:z	-5.263	0.67	40 C
			1.11%	1 P:z	-5.263	0.67	25 C
			1.10%	1 P:x	-5.263	0.67	24 C
-9.998	2.00	72 A	4.74%	1 P:x	-5.263	0.67	30 C
			4.37%	1 S	-6.532	1.00	92 H

			3.97%	1 S	-6.532	1.00	75 H
			3.61%	1 S	-6.532	1.00	91 H
			3.25%	1 P:x	-9.066	1.33	8 O
			3.24%	1 P:z	-5.263	0.67	42 C
			2.92%	1 P:z	-5.263	0.67	34 C
			2.88%	1 P:z	-5.263	0.67	28 C
			2.74%	1 S	-6.532	1.00	77 H
			2.49%	1 S	-6.532	1.00	47 H
			2.31%	1 P:z	-9.066	1.33	10 O
			2.02%	1 P:z	-9.066	1.33	8 O
			1.98%	1 P:y	-7.073	1.00	43 N
			1.94%	1 P:x	-5.263	0.67	42 C
			1.80%	1 P:y	-9.066	1.33	10 O
			1.76%	1 P:y	-9.066	1.33	8 O
			1.56%	1 S	-23.822	2.00	8 O
			1.53%	1 S	-6.532	1.00	73 H
			1.41%	1 P:x	-5.263	0.67	38 C
			1.38%	1 P:z	-9.066	1.33	7 O
			1.35%	1 P:x	-9.066	1.33	10 O
			1.33%	1 S	-6.532	1.00	84 H
			1.30%	1 P:x	-5.263	0.67	15 C
			1.24%	1 S	-6.532	1.00	50 H
			1.07%	1 P:y	-5.263	0.67	38 C
			1.03%	1 P:y	-5.263	0.67	21 C
-9.932	2.00	73 A	4.67%	1 P:y	-5.263	0.67	40 C
			3.97%	1 S	-6.532	1.00	70 H
			3.92%	1 S	-6.532	1.00	65 H
			3.36%	1 P:z	-5.263	0.67	20 C
			3.11%	1 S	-6.532	1.00	68 H
			3.10%	1 S	-6.532	1.00	66 H
			2.87%	1 S	-6.532	1.00	63 H

			2.69%	1 P:y	-5.263	0.67	21	C
			2.22%	1 S	-6.532	1.00	77	H
			2.11%	1 P:x	-5.263	0.67	21	C
			1.96%	1 P:z	-9.066	1.33	8	O
			1.87%	1 P:y	-5.263	0.67	24	C
			1.85%	1 P:y	-9.066	1.33	5	O
			1.84%	1 P:x	-9.066	1.33	8	O
			1.81%	1 P:z	-5.263	0.67	17	C
			1.71%	1 P:x	-5.263	0.67	32	C
			1.60%	1 S	-6.532	1.00	95	H
			1.55%	1 S	-6.532	1.00	73	H
			1.54%	1 P:z	-5.263	0.67	18	C
			1.50%	1 P:z	-5.263	0.67	28	C
			1.41%	1 P:x	-9.066	1.33	6	O
			1.12%	1 S	-6.532	1.00	47	H
			1.10%	1 P:x	-5.263	0.67	34	C
			1.05%	1 P:x	-9.066	1.33	5	O
			1.04%	1 P:y	-5.263	0.67	30	C
			1.00%	1 S	-23.822	2.00	8	O
-9.912	2.00	74 A	4.46%	1 P:x	-5.263	0.67	32	C
			3.59%	1 P:z	-5.263	0.67	20	C
			3.05%	1 P:z	-5.263	0.67	24	C
			3.00%	1 P:y	-5.263	0.67	21	C
			2.89%	1 S	-6.532	1.00	56	H
			2.72%	1 P:z	-5.263	0.67	36	C
			2.66%	1 P:x	-9.066	1.33	8	O
			2.59%	1 S	-6.532	1.00	66	H
			2.40%	1 P:z	-5.263	0.67	23	C
			2.22%	1 P:z	-9.066	1.33	8	O
			2.21%	1 P:y	-5.263	0.67	24	C
			2.12%	1 S	-6.532	1.00	60	H

			1.83%	1 S	-6.532	1.00	96 H
			1.80%	1 P:y	-9.066	1.33	8 O
			1.59%	1 P:y	-5.263	0.67	20 C
			1.55%	1 S	-6.532	1.00	64 H
			1.54%	1 P:y	-9.066	1.33	6 O
			1.48%	1 S	-6.532	1.00	95 H
			1.48%	1 S	-23.822	2.00	8 O
			1.45%	1 S	-6.532	1.00	94 H
			1.39%	1 S	-6.532	1.00	65 H
			1.31%	1 P:y	-5.263	0.67	40 C
			1.30%	1 P:y	-9.066	1.33	9 O
			1.26%	1 S	-6.532	1.00	55 H
			1.23%	1 P:x	-5.263	0.67	35 C
			1.21%	1 P:x	-5.263	0.67	21 C
			1.16%	1 S	-6.532	1.00	47 H
			1.07%	1 P:z	-5.263	0.67	40 C
-9.857	2.00	75 A	7.97%	1 P:z	-5.263	0.67	36 C
			5.45%	1 S	-6.532	1.00	56 H
			4.97%	1 P:z	-5.263	0.67	42 C
			4.65%	1 S	-6.532	1.00	92 H
			3.55%	1 S	-6.532	1.00	55 H
			3.20%	1 S	-6.532	1.00	91 H
			3.14%	1 S	-6.532	1.00	95 H
			2.68%	1 P:x	-5.263	0.67	32 C
			2.57%	1 P:y	-5.263	0.67	25 C
			2.19%	1 P:z	-5.263	0.67	34 C
			1.92%	1 P:z	-5.263	0.67	31 C
			1.91%	1 S	-6.532	1.00	47 H
			1.80%	1 S	-6.532	1.00	54 H
			1.80%	1 P:y	-5.263	0.67	20 C
			1.69%	1 S	-6.532	1.00	50 H

			1.56%	1 S	-6.532	1.00	72 H
			1.31%	1 P:y	-9.066	1.33	6 O
			1.29%	1 P:y	-9.066	1.33	2 O
			1.23%	1 P:x	-5.263	0.67	15 C
			1.15%	1 S	-6.532	1.00	57 H
			1.14%	1 P:z	-5.263	0.67	22 C
			1.11%	1 P:z	-5.263	0.67	21 C
			1.05%	1 P:y	-5.263	0.67	39 C
			1.05%	1 P:y	-5.263	0.67	19 C
			1.03%	1 S	-6.532	1.00	70 H
-9.801	2.00	76 A	8.41%	1 P:y	-5.263	0.67	30 C
			6.57%	1 P:z	-5.263	0.67	42 C
			5.56%	1 S	-6.532	1.00	91 H
			5.51%	1 P:y	-5.263	0.67	29 C
			4.95%	1 S	-6.532	1.00	73 H
			2.50%	1 S	-6.532	1.00	92 H
			2.26%	1 P:x	-5.263	0.67	12 C
			2.21%	1 P:z	-9.066	1.33	3 O
			2.01%	1 S	-6.532	1.00	56 H
			1.95%	1 P:z	-5.263	0.67	36 C
			1.73%	1 S	-6.532	1.00	77 H
			1.69%	1 S	-6.532	1.00	46 H
			1.54%	1 P:x	-5.263	0.67	28 C
			1.46%	1 S	-6.532	1.00	66 H
			1.43%	1 P:x	-5.263	0.67	30 C
			1.34%	1 P:x	-5.263	0.67	29 C
			1.33%	1 P:y	-9.066	1.33	7 O
			1.26%	1 P:y	-5.263	0.67	21 C
			1.19%	1 P:x	-5.263	0.67	11 C
			1.18%	1 S	-6.532	1.00	50 H
			1.13%	1 P:y	-5.263	0.67	34 C

			1.13%	1 S	-6.532	1.00	74 H
			1.10%	1 P:z	-5.263	0.67	12 C
			1.09%	1 P:y	-5.263	0.67	28 C
-9.758	2.00	77 A	7.09%	1 S	-6.532	1.00	68 H
			6.80%	1 P:x	-5.263	0.67	21 C
			3.32%	1 P:y	-5.263	0.67	32 C
			2.68%	1 S	-6.532	1.00	74 H
			2.58%	1 S	-6.532	1.00	65 H
			2.46%	1 S	-6.532	1.00	67 H
			2.42%	1 S	-6.532	1.00	54 H
			2.37%	1 P:x	-5.263	0.67	36 C
			2.30%	1 P:y	-5.263	0.67	40 C
			2.13%	1 P:z	-5.263	0.67	18 C
			2.02%	1 P:z	-5.263	0.67	30 C
			1.69%	1 P:z	-5.263	0.67	15 C
			1.66%	1 P:y	-9.066	1.33	9 O
			1.58%	1 S	-6.532	1.00	49 H
			1.51%	1 S	-6.532	1.00	95 H
			1.45%	1 P:x	-5.263	0.67	12 C
			1.36%	1 S	-6.532	1.00	70 H
			1.35%	1 S	-6.532	1.00	94 H
			1.30%	1 P:x	-9.066	1.33	4 O
			1.28%	1 P:z	-5.263	0.67	17 C
			1.28%	1 P:z	-5.263	0.67	25 C
			1.16%	1 P:z	-5.263	0.67	20 C
			1.14%	1 S	-6.532	1.00	86 H
			1.12%	1 S	-6.532	1.00	52 H
			1.10%	1 P:y	-9.066	1.33	1 O
			1.04%	1 S	-6.532	1.00	59 H
-9.645	2.00	78 A	7.44%	1 S	-6.532	1.00	95 H
			5.23%	1 P:z	-5.263	0.67	23 C

			4.01%	1 P:x	-5.263	0.67	32 C
			3.80%	1 P:z	-5.263	0.67	21 C
			2.90%	1 S	-6.532	1.00	50 H
			2.89%	1 P:y	-9.066	1.33	2 O
			2.26%	1 P:y	-5.263	0.67	32 C
			2.23%	1 S	-6.532	1.00	52 H
			2.10%	1 P:x	-5.263	0.67	21 C
			1.99%	1 P:z	-5.263	0.67	34 C
			1.93%	1 P:z	-9.066	1.33	9 O
			1.91%	1 P:y	-5.263	0.67	15 C
			1.85%	1 S	-6.532	1.00	60 H
			1.82%	1 S	-6.532	1.00	46 H
			1.79%	1 S	-6.532	1.00	47 H
			1.73%	1 S	-6.532	1.00	64 H
			1.67%	1 P:z	-9.066	1.33	2 O
			1.52%	1 P:z	-9.066	1.33	8 O
			1.51%	1 P:z	-5.263	0.67	25 C
			1.50%	2 P:z	4.578	0.00	23 C
			1.44%	1 S	-6.532	1.00	68 H
			1.40%	1 S	-6.532	1.00	57 H
			1.31%	1 P:x	-9.066	1.33	2 O
			1.31%	1 P:y	-5.263	0.67	39 C
			1.28%	1 P:y	-5.263	0.67	34 C
			1.17%	1 P:y	-9.066	1.33	9 O
			1.07%	1 S	-6.532	1.00	54 H
-9.609	2.00	79 A	24.81%	1 P:z	-5.263	0.67	30 C
			23.94%	1 S	-6.532	1.00	74 H
			8.97%	1 S	-6.532	1.00	73 H
			2.67%	1 S	-6.532	1.00	75 H
			2.24%	1 S	-6.532	1.00	91 H
			1.73%	1 S	-6.532	1.00	56 H

			1.69%	1 P:x	-5.263	0.67	42 C
			1.38%	1 P:y	-9.066	1.33	3 O
			1.19%	1 P:z	-5.263	0.67	36 C
			1.15%	1 P:x	-5.263	0.67	20 C
			1.02%	1 P:y	-9.066	1.33	1 O
-9.559	2.00	80 A	18.00%	1 S	-6.532	1.00	67 H
			11.74%	1 P:z	-5.263	0.67	21 C
			4.68%	1 S	-6.532	1.00	95 H
			3.94%	1 P:z	-9.066	1.33	2 O
			3.42%	1 P:x	-5.263	0.67	21 C
			3.03%	1 P:y	-9.066	1.33	2 O
			2.89%	1 S	-6.532	1.00	68 H
			2.33%	1 P:z	-5.263	0.67	32 C
			2.08%	1 P:z	-5.263	0.67	23 C
			1.93%	1 P:x	-5.263	0.67	32 C
			1.78%	1 P:y	-9.066	1.33	6 O
			1.75%	1 P:z	-5.263	0.67	15 C
			1.67%	1 P:x	-5.263	0.67	34 C
			1.61%	1 S	-6.532	1.00	49 H
			1.57%	2 P:z	4.578	0.00	23 C
			1.55%	1 P:y	-5.263	0.67	21 C
			1.44%	1 S	-6.532	1.00	66 H
			1.23%	1 S	-6.532	1.00	47 H
			1.22%	1 P:y	-5.263	0.67	14 C
			1.08%	1 S	-6.532	1.00	94 H
			1.03%	1 P:x	-9.066	1.33	2 O
			1.00%	1 P:x	-5.263	0.67	31 C
-9.547	2.00	81 A	11.49%	1 S	-6.532	1.00	96 H
			9.70%	1 P:z	-5.263	0.67	32 C
			4.81%	1 S	-6.532	1.00	95 H
			4.70%	1 S	-6.532	1.00	71 H

			3.38%	1 P:x	-5.263	0.67	20 C	
			3.31%	1 P:x	-5.263	0.67	36 C	
			3.24%	1 P:y	-5.263	0.67	32 C	
			3.19%	1 P:x	-9.066	1.33	6 O	
			2.58%	1 S	-6.532	1.00	54 H	
			2.43%	1 P:z	-5.263	0.67	20 C	
			2.35%	1 P:y	-5.263	0.67	14 C	
			2.20%	1 S	-6.532	1.00	72 H	
			1.63%	1 P:x	-5.263	0.67	21 C	
			1.61%	1 S	-6.532	1.00	67 H	
			1.56%	1 P:z	-5.263	0.67	17 C	
			1.53%	1 S	-6.532	1.00	55 H	
			1.45%	1 P:z	-9.066	1.33	6 O	
			1.43%	1 S	-13.631	2.00	14 C	
			1.38%	1 P:z	-9.066	1.33	9 O	
			1.38%	1 S	-6.532	1.00	68 H	
			1.33%	1 S	-6.532	1.00	59 H	
			1.18%	1 S	-6.532	1.00	65 H	
			1.07%	1 P:y	-5.263	0.67	40 C	
			1.06%	1 P:z	-5.263	0.67	14 C	
-9.485	2.00	82 A	6.75%	1 S	-6.532	1.00	72 H	
			6.20%	1 S	-6.532	1.00	56 H	
			5.16%	1 S	-6.532	1.00	67 H	
			4.84%	1 P:z	-5.263	0.67	36 C	
			4.56%	1 P:y	-5.263	0.67	20 C	
			3.78%	1 S	-6.532	1.00	95 H	
			3.41%	1 P:x	-5.263	0.67	32 C	
			3.10%	1 S	-6.532	1.00	66 H	
			3.04%	1 P:y	-5.263	0.67	21 C	
			2.95%	1 S	-6.532	1.00	71 H	
			2.50%	1 P:x	-5.263	0.67	20 C	

			1.98%	1 S	-6.532	1.00	94 H
			1.92%	1 P:z	-5.263	0.67	21 C
			1.80%	1 P:x	-5.263	0.67	31 C
			1.74%	1 P:z	-5.263	0.67	34 C
			1.69%	1 S	-6.532	1.00	46 H
			1.55%	1 P:y	-5.263	0.67	36 C
			1.41%	1 P:z	-5.263	0.67	30 C
			1.36%	1 P:z	-5.263	0.67	25 C
			1.35%	1 S	-6.532	1.00	74 H
			1.31%	1 P:y	-5.263	0.67	34 C
			1.31%	1 P:y	-9.066	1.33	6 O
			1.24%	1 S	-6.532	1.00	50 H
			1.22%	1 S	-6.532	1.00	55 H
			1.08%	1 P:z	-5.263	0.67	32 C
			1.02%	1 P:z	-9.066	1.33	9 O
-9.412	2.00	83 A	6.70%	1 S	-6.532	1.00	79 H
			6.52%	1 P:z	-5.263	0.67	26 C
			5.82%	1 P:x	-9.066	1.33	7 O
			4.42%	1 S	-6.532	1.00	84 H
			3.66%	1 P:z	-5.263	0.67	34 C
			3.22%	1 P:x	-5.263	0.67	38 C
			2.82%	1 S	-6.532	1.00	93 H
			2.45%	1 S	-6.532	1.00	45 H
			2.29%	1 P:x	-5.263	0.67	42 C
			2.19%	1 S	-6.532	1.00	82 H
			2.18%	1 S	-6.532	1.00	47 H
			1.88%	1 P:z	-5.263	0.67	38 C
			1.82%	1 S	-6.532	1.00	81 H
			1.68%	1 P:y	-5.263	0.67	26 C
			1.61%	1 S	-6.532	1.00	52 H
			1.46%	1 P:y	-5.263	0.67	15 C

			1.44%	1 S	-6.532	1.00	66 H
			1.29%	1 S	-6.532	1.00	78 H
			1.25%	1 P:z	-9.066	1.33	3 O
			1.18%	1 P:y	-5.263	0.67	21 C
			1.11%	1 S	-6.532	1.00	74 H
			1.02%	1 S	-6.532	1.00	63 H
			1.02%	1 P:y	-5.263	0.67	25 C
-9.399	2.00	84 A	4.39%	1 P:z	-5.263	0.67	34 C
			3.89%	1 S	-6.532	1.00	47 H
			3.49%	1 S	-6.532	1.00	79 H
			3.44%	1 P:z	-5.263	0.67	26 C
			3.41%	1 S	-6.532	1.00	84 H
			2.79%	1 S	-6.532	1.00	66 H
			2.59%	1 S	-6.532	1.00	45 H
			2.44%	1 P:x	-5.263	0.67	38 C
			2.44%	1 P:y	-5.263	0.67	21 C
			2.43%	1 S	-6.532	1.00	92 H
			2.42%	1 P:z	-5.263	0.67	42 C
			1.95%	1 S	-6.532	1.00	93 H
			1.75%	1 S	-6.532	1.00	82 H
			1.64%	1 P:y	-9.066	1.33	2 O
			1.47%	1 P:z	-5.263	0.67	38 C
			1.42%	1 S	-6.532	1.00	72 H
			1.40%	1 S	-6.532	1.00	96 H
			1.39%	1 P:x	-9.066	1.33	2 O
			1.37%	1 S	-6.532	1.00	54 H
			1.35%	1 P:x	-9.066	1.33	7 O
			1.32%	1 P:x	-5.263	0.67	36 C
			1.32%	1 P:x	-9.066	1.33	5 O
			1.23%	1 P:y	-5.263	0.67	32 C
			1.23%	1 P:x	-5.263	0.67	34 C

			1.21%	1 P:z	-5.263	0.67	31 C		
			1.21%	1 S	-6.532	1.00	78 H		
			1.08%	1 P:y	-5.263	0.67	15 C		
			1.06%	1 S	-6.532	1.00	59 H		
-9.305	2.00	85 A	10.82%	1 P:x	-5.263	0.67	40 C		
			10.54%	1 P:x	-5.263	0.67	37 C		
			3.58%	1 P:x	-9.066	1.33	5 O		
			3.47%	1 S	-6.532	1.00	68 H		
			2.93%	1 S	-6.532	1.00	63 H		
			2.80%	1 S	-6.532	1.00	81 H		
			2.77%	1 P:z	-5.263	0.67	38 C		
			2.59%	1 P:x	-5.263	0.67	21 C		
			2.48%	1 P:y	-9.066	1.33	5 O		
			2.46%	1 S	-6.532	1.00	82 H		
			2.00%	1 P:y	-5.263	0.67	21 C		
			1.95%	1 P:x	-9.066	1.33	9 O		
			1.91%	2 P:x	4.578	0.00	37 C		
			1.87%	1 S	-6.532	1.00	66 H		
			1.74%	1 P:z	-5.263	0.67	26 C		
			1.63%	1 S	-6.532	1.00	71 H		
			1.57%	1 P:x	-5.263	0.67	26 C		
			1.52%	1 S	-6.532	1.00	79 H		
			1.50%	1 P:x	-5.263	0.67	20 C		
			1.34%	1 S	-6.532	1.00	64 H		
			1.33%	1 S	-6.532	1.00	84 H		
			1.31%	1 S	-6.532	1.00	61 H		
			1.24%	1 S	-6.532	1.00	93 H		
			1.23%	1 S	-6.532	1.00	62 H		
			1.23%	1 S	-6.532	1.00	94 H		
-9.278	2.00	86 A	7.53%	1 S	-6.532	1.00	81 H		
			5.19%	1 P:x	-5.263	0.67	26 C		

			4.83%	1 S	-6.532	1.00	93 H
			4.57%	1 P:z	-5.263	0.67	38 C
			4.06%	1 S	-6.532	1.00	82 H
			3.72%	1 P:x	-5.263	0.67	42 C
			3.69%	1 S	-6.532	1.00	54 H
			3.57%	1 P:x	-5.263	0.67	36 C
			2.93%	1 P:x	-9.066	1.33	10 O
			2.13%	1 P:z	-5.263	0.67	26 C
			2.06%	1 P:x	-5.263	0.67	40 C
			1.98%	1 P:x	-9.066	1.33	7 O
			1.94%	1 P:x	-5.263	0.67	37 C
			1.58%	1 S	-6.532	1.00	78 H
			1.47%	1 S	-6.532	1.00	79 H
			1.46%	1 P:y	-9.066	1.33	3 O
			1.40%	1 P:z	-5.263	0.67	28 C
			1.29%	1 P:z	-9.066	1.33	10 O
			1.29%	1 S	-6.532	1.00	59 H
			1.22%	1 P:x	-5.263	0.67	41 C
			1.18%	1 S	-6.532	1.00	63 H
			1.12%	1 S	-6.532	1.00	92 H
			1.09%	1 S	-6.532	1.00	55 H
			1.09%	1 P:z	-5.263	0.67	42 C
			1.03%	1 S	-6.532	1.00	83 H
-9.234	2.00	87 A	9.05%	1 S	-6.532	1.00	54 H
			7.93%	1 P:x	-5.263	0.67	36 C
			2.94%	1 S	-6.532	1.00	59 H
			2.69%	1 P:x	-5.263	0.67	40 C
			2.48%	1 P:x	-5.263	0.67	37 C
			2.34%	1 S	-6.532	1.00	67 H
			2.11%	1 S	-6.532	1.00	50 H
			2.04%	1 S	-6.532	1.00	49 H

			2.02%	1 P:z	-5.263	0.67	15 C
			1.96%	1 S	-6.532	1.00	55 H
			1.89%	1 S	-6.532	1.00	66 H
			1.88%	1 P:y	-5.263	0.67	21 C
			1.73%	1 S	-6.532	1.00	81 H
			1.65%	1 P:z	-9.066	1.33	2 O
			1.64%	1 S	-6.532	1.00	44 H
			1.59%	1 P:y	-5.263	0.67	15 C
			1.54%	1 P:x	-5.263	0.67	34 C
			1.50%	1 P:x	-5.263	0.67	31 C
			1.37%	1 S	-6.532	1.00	46 H
			1.36%	1 P:y	-5.263	0.67	39 C
			1.34%	1 P:z	-5.263	0.67	21 C
			1.28%	1 P:y	-5.263	0.67	34 C
			1.25%	1 P:z	-5.263	0.67	36 C
			1.22%	1 P:x	-5.263	0.67	26 C
			1.11%	1 S	-6.532	1.00	57 H
			1.02%	1 S	-6.532	1.00	96 H
-9.183	2.00	88 A	5.22%	1 S	-6.532	1.00	81 H
			4.85%	1 P:x	-5.263	0.67	26 C
			3.80%	1 P:x	-5.263	0.67	20 C
			3.56%	1 S	-6.532	1.00	71 H
			3.09%	1 S	-6.532	1.00	46 H
			3.00%	1 P:y	-5.263	0.67	34 C
			2.94%	1 S	-6.532	1.00	65 H
			2.85%	1 P:y	-5.263	0.67	40 C
			2.79%	1 S	-6.532	1.00	70 H
			2.58%	1 P:x	-5.263	0.67	13 C
			2.31%	1 P:y	-9.066	1.33	5 O
			2.30%	1 P:x	-7.073	1.00	43 N
			2.03%	1 P:x	-5.263	0.67	30 C

			1.79%	1 P:x	-5.263	0.67	38 C	
			1.72%	1 S	-6.532	1.00	75 H	
			1.52%	1 S	-6.532	1.00	94 H	
			1.48%	1 S	-6.532	1.00	45 H	
			1.37%	1 S	-6.532	1.00	80 H	
			1.34%	1 S	-6.532	1.00	84 H	
			1.31%	1 P:z	-9.066	1.33	6 O	
			1.22%	1 S	-6.532	1.00	83 H	
			1.21%	1 P:y	-9.066	1.33	3 O	
			1.19%	1 S	-6.532	1.00	73 H	
			1.17%	1 P:z	-9.066	1.33	1 O	
			1.02%	1 S	-6.532	1.00	63 H	
-9.143	2.00	89 A	3.97%	1 P:x	-5.263	0.67	26 C	
			3.83%	1 P:x	-5.263	0.67	42 C	
			3.76%	1 S	-6.532	1.00	93 H	
			3.49%	1 S	-6.532	1.00	81 H	
			3.18%	1 P:x	-5.263	0.67	13 C	
			3.06%	1 P:x	-7.073	1.00	43 N	
			2.70%	1 S	-6.532	1.00	80 H	
			2.66%	1 S	-6.532	1.00	65 H	
			2.39%	1 P:x	-9.066	1.33	3 O	
			2.32%	1 P:x	-9.066	1.33	10 O	
			2.31%	1 P:y	-5.263	0.67	40 C	
			2.17%	1 S	-6.532	1.00	94 H	
			1.91%	1 S	-6.532	1.00	91 H	
			1.80%	1 P:x	-5.263	0.67	38 C	
			1.75%	1 P:x	-5.263	0.67	41 C	
			1.75%	1 P:z	-9.066	1.33	7 O	
			1.71%	1 P:y	-9.066	1.33	5 O	
			1.70%	1 P:y	-9.066	1.33	10 O	
			1.70%	1 S	-6.532	1.00	88 H	

			1.66%	1 S	-6.532	1.00	84 H
			1.66%	1 S	-6.532	1.00	46 H
			1.65%	1 P:y	-5.263	0.67	26 C
			1.57%	1 P:y	-5.263	0.67	34 C
			1.31%	1 P:z	-5.263	0.67	13 C
			1.23%	1 S	-6.532	1.00	90 H
			1.05%	1 S	-6.532	1.00	79 H
-9.116	2.00	90 A	5.33%	1 S	-6.532	1.00	45 H
			4.86%	1 P:x	-9.066	1.33	2 O
			4.69%	1 S	-6.532	1.00	52 H
			4.24%	1 P:x	-5.263	0.67	27 C
			3.85%	1 P:x	-5.263	0.67	34 C
			3.82%	1 P:x	-5.263	0.67	15 C
			3.71%	1 P:x	-9.066	1.33	5 O
			3.46%	1 S	-6.532	1.00	48 H
			2.89%	1 P:z	-5.263	0.67	34 C
			2.56%	1 P:x	-5.263	0.67	31 C
			2.18%	1 P:y	-9.066	1.33	9 O
			1.96%	1 S	-6.532	1.00	50 H
			1.95%	1 S	-6.532	1.00	59 H
			1.73%	1 P:x	-9.066	1.33	1 O
			1.61%	1 P:x	-5.263	0.67	22 C
			1.54%	1 S	-6.532	1.00	44 H
			1.42%	1 S	-6.532	1.00	84 H
			1.33%	1 S	-6.532	1.00	67 H
			1.32%	1 P:y	-5.263	0.67	27 C
			1.30%	1 S	-23.822	2.00	2 O
			1.29%	1 P:y	-9.066	1.33	2 O
			1.18%	1 P:x	-5.263	0.67	38 C
			1.12%	1 P:x	-5.263	0.67	39 C
			1.02%	2 P:x	4.578	0.00	31 C

-9.012	2.00	91 A	9.57%	1 P:z	-9.066	1.33	8 O
			6.31%	1 P:z	-5.263	0.67	34 C
			5.31%	1 S	-6.532	1.00	47 H
			4.70%	1 P:z	-5.263	0.67	35 C
			4.70%	1 S	-6.532	1.00	55 H
			4.50%	1 P:x	-5.263	0.67	15 C
			3.48%	1 S	-6.532	1.00	50 H
			3.23%	1 P:x	-5.263	0.67	36 C
			2.57%	1 S	-6.532	1.00	48 H
			2.02%	1 S	-6.532	1.00	45 H
			1.87%	1 S	-6.532	1.00	46 H
			1.75%	1 S	-6.532	1.00	68 H
			1.71%	1 P:y	-5.263	0.67	35 C
			1.62%	1 S	-6.532	1.00	65 H
			1.51%	1 P:x	-5.263	0.67	21 C
			1.18%	1 S	-6.532	1.00	54 H
			1.09%	1 P:y	-5.263	0.67	40 C
			1.09%	1 P:x	-5.263	0.67	27 C
			1.06%	1 P:y	-5.263	0.67	34 C
-8.953	2.00	92 A	6.51%	1 S	-6.532	1.00	83 H
			4.18%	1 S	-6.532	1.00	96 H
			4.18%	1 S	-6.532	1.00	80 H
			4.08%	1 P:x	-5.263	0.67	38 C
			3.47%	1 P:z	-9.066	1.33	6 O
			3.46%	1 S	-6.532	1.00	46 H
			3.24%	1 P:y	-5.263	0.67	34 C
			2.86%	1 S	-6.532	1.00	45 H
			2.72%	1 S	-6.532	1.00	71 H
			2.39%	1 P:y	-5.263	0.67	26 C
			2.38%	1 P:x	-5.263	0.67	20 C
			2.19%	1 P:x	-9.066	1.33	2 O

			2.17%	1 P:y	-5.263	0.67	32 C	
			2.13%	1 S	-6.532	1.00	84 H	
			1.91%	1 S	-6.532	1.00	70 H	
			1.78%	1 P:x	-5.263	0.67	34 C	
			1.52%	1 S	-6.532	1.00	94 H	
			1.41%	1 S	-6.532	1.00	77 H	
			1.36%	1 P:z	-5.263	0.67	14 C	
			1.27%	1 S	-6.532	1.00	90 H	
			1.09%	1 P:x	-5.263	0.67	27 C	
			1.07%	1 P:y	-5.263	0.67	13 C	
			1.05%	1 P:x	-9.066	1.33	6 O	
			1.00%	1 S	-6.532	1.00	52 H	
-8.923	2.00	93 A	11.25%	1 S	-6.532	1.00	83 H	
			9.40%	1 S	-6.532	1.00	80 H	
			5.84%	1 P:y	-5.263	0.67	26 C	
			4.99%	1 P:x	-5.263	0.67	38 C	
			4.37%	1 P:x	-5.263	0.67	15 C	
			3.91%	1 P:x	-5.263	0.67	27 C	
			3.75%	1 S	-6.532	1.00	48 H	
			2.82%	1 S	-6.532	1.00	52 H	
			2.62%	1 P:z	-5.263	0.67	38 C	
			2.31%	1 S	-6.532	1.00	50 H	
			2.12%	1 P:x	-5.263	0.67	20 C	
			1.89%	1 S	-6.532	1.00	84 H	
			1.86%	1 S	-6.532	1.00	71 H	
			1.48%	1 S	-6.532	1.00	77 H	
			1.44%	1 S	-6.532	1.00	70 H	
			1.38%	1 P:y	-5.263	0.67	38 C	
			1.28%	1 P:y	-7.073	1.00	43 N	
			1.26%	1 S	-6.532	1.00	59 H	
			1.25%	1 S	-6.532	1.00	46 H	

			1.24%	1 S	-6.532	1.00	81	H
			1.22%	1 P:y	-5.263	0.67	34	C
			1.22%	1 S	-6.532	1.00	45	H
			1.08%	1 P:x	-9.066	1.33	8	O
			1.04%	1 S	-6.532	1.00	88	H
-8.860	2.00	94 A	6.17%	1 S	-6.532	1.00	73	H
			5.52%	1 P:y	-9.066	1.33	7	O
			5.23%	1 P:x	-5.263	0.67	30	C
			3.86%	1 S	-6.532	1.00	75	H
			3.39%	1 P:x	-9.066	1.33	10	O
			3.14%	1 P:x	-9.066	1.33	3	O
			2.69%	1 P:x	-5.263	0.67	29	C
			2.57%	1 P:x	-5.263	0.67	15	C
			2.45%	1 S	-6.532	1.00	76	H
			2.39%	1 P:z	-5.263	0.67	13	C
			2.04%	1 S	-6.532	1.00	48	H
			2.00%	1 S	-6.532	1.00	78	H
			1.79%	1 P:z	-9.066	1.33	8	O
			1.78%	1 P:z	-5.263	0.67	12	C
			1.77%	1 P:y	-9.066	1.33	5	O
			1.74%	1 S	-6.532	1.00	50	H
			1.73%	1 P:x	-9.066	1.33	2	O
			1.59%	1 P:x	-9.066	1.33	1	O
			1.46%	1 P:z	-5.263	0.67	28	C
			1.44%	1 P:x	-5.263	0.67	28	C
			1.40%	1 S	-6.532	1.00	91	H
			1.39%	1 P:y	-5.263	0.67	30	C
			1.26%	1 P:y	-5.263	0.67	42	C
-8.836	2.00	95 A	5.52%	1 S	-6.532	1.00	63	H
			5.47%	1 P:x	-9.066	1.33	2	O
			5.12%	1 P:x	-5.263	0.67	40	C

			5.02%	1 S	-6.532	1.00	61 H
			4.20%	1 P:x	-5.263	0.67	37 C
			3.16%	1 P:z	-5.263	0.67	40 C
			2.55%	1 P:y	-9.066	1.33	5 O
			2.39%	1 P:y	-5.263	0.67	23 C
			2.05%	1 P:z	-5.263	0.67	37 C
			1.86%	1 P:z	-9.066	1.33	8 O
			1.79%	1 P:x	-9.066	1.33	9 O
			1.74%	1 P:y	-5.263	0.67	22 C
			1.65%	1 S	-6.532	1.00	64 H
			1.64%	1 S	-6.532	1.00	68 H
			1.59%	1 P:y	-9.066	1.33	7 O
			1.41%	1 S	-6.532	1.00	66 H
			1.39%	1 P:x	-5.263	0.67	30 C
			1.32%	1 S	-6.532	1.00	94 H
			1.30%	1 P:x	-9.066	1.33	5 O
			1.28%	1 P:y	-5.263	0.67	35 C
			1.26%	1 S	-6.532	1.00	75 H
			1.19%	1 P:x	-5.263	0.67	23 C
			1.18%	1 P:x	-5.263	0.67	21 C
			1.08%	1 S	-6.532	1.00	73 H
			1.06%	1 P:y	-9.066	1.33	2 O
			1.06%	1 S	-6.532	1.00	62 H
-8.738	2.00	96 A	4.76%	1 S	-6.532	1.00	56 H
			4.03%	1 S	-6.532	1.00	72 H
			3.37%	1 P:z	-9.066	1.33	8 O
			3.13%	1 P:y	-5.263	0.67	20 C
			2.92%	1 P:y	-9.066	1.33	7 O
			2.48%	1 S	-6.532	1.00	90 H
			2.41%	1 S	-6.532	1.00	94 H
			2.20%	1 P:y	-5.263	0.67	36 C

			2.06%	1 S	-6.532	1.00	49 H
			2.04%	1 P:z	-5.263	0.67	15 C
			2.00%	1 P:y	-5.263	0.67	18 C
			1.97%	1 P:x	-5.263	0.67	20 C
			1.91%	1 S	-6.532	1.00	80 H
			1.83%	1 S	-6.532	1.00	83 H
			1.82%	1 P:y	-9.066	1.33	6 O
			1.79%	1 P:y	-5.263	0.67	24 C
			1.67%	1 S	-6.532	1.00	71 H
			1.65%	1 P:y	-5.263	0.67	19 C
			1.57%	1 P:y	-5.263	0.67	26 C
			1.56%	1 P:y	-9.066	1.33	1 O
			1.49%	1 P:z	-5.263	0.67	32 C
			1.49%	1 P:z	-5.263	0.67	36 C
			1.28%	1 P:x	-9.066	1.33	1 O
			1.28%	1 P:z	-9.066	1.33	6 O
			1.16%	1 P:z	-5.263	0.67	35 C
			1.07%	1 P:y	-5.263	0.67	13 C
			1.06%	1 P:z	-5.263	0.67	24 C
			1.04%	1 P:z	-5.263	0.67	17 C
			1.02%	1 P:y	-7.073	1.00	43 N
-8.698	2.00	97 A	3.98%	1 P:y	-5.263	0.67	28 C
			3.56%	1 P:y	-9.066	1.33	7 O
			3.32%	1 S	-6.532	1.00	80 H
			3.29%	1 P:y	-5.263	0.67	13 C
			2.61%	1 P:y	-9.066	1.33	3 O
			2.59%	1 P:y	-5.263	0.67	26 C
			2.32%	1 S	-6.532	1.00	77 H
			2.26%	1 S	-6.532	1.00	87 H
			2.25%	1 P:x	-9.066	1.33	1 O
			2.21%	1 S	-6.532	1.00	83 H

			2.16%	1 S	-6.532	1.00	56 H		
			2.08%	1 P:y	-9.066	1.33	6 O		
			2.07%	1 S	-6.532	1.00	69 H		
			1.95%	1 P:z	-5.263	0.67	38 C		
			1.88%	1 P:z	-9.066	1.33	8 O		
			1.68%	1 S	-6.532	1.00	70 H		
			1.65%	1 P:y	-5.263	0.67	12 C		
			1.43%	1 P:y	-7.073	1.00	43 N		
			1.33%	1 P:z	-9.066	1.33	7 O		
			1.31%	1 P:y	-9.066	1.33	10 O		
			1.29%	1 P:x	-5.263	0.67	27 C		
			1.29%	1 P:y	-5.263	0.67	18 C		
			1.29%	1 S	-6.532	1.00	72 H		
			1.21%	1 P:z	-5.263	0.67	24 C		
			1.18%	1 P:y	-5.263	0.67	38 C		
			1.15%	1 P:z	-7.073	1.00	43 N		
			1.14%	1 P:x	-9.066	1.33	7 O		
			1.13%	1 P:z	-5.263	0.67	32 C		
			1.03%	1 P:y	-9.066	1.33	1 O		
			1.03%	1 P:y	-5.263	0.67	24 C		
			1.01%	1 P:z	-9.066	1.33	6 O		
			1.01%	1 S	-6.532	1.00	96 H		
-8.637	2.00	98 A	9.06%	1 S	-6.532	1.00	69 H		
			7.51%	1 S	-6.532	1.00	70 H		
			4.67%	1 S	-6.532	1.00	90 H		
			4.58%	1 P:y	-9.066	1.33	1 O		
			4.34%	1 S	-6.532	1.00	94 H		
			4.22%	1 P:z	-5.263	0.67	20 C		
			4.17%	1 P:z	-5.263	0.67	24 C		
			2.74%	1 P:y	-5.263	0.67	17 C		
			2.51%	1 P:y	-5.263	0.67	32 C		

			2.45%	1 S	-6.532	1.00	72 H
			2.35%	1 P:y	-5.263	0.67	18 C
			2.06%	1 P:z	-9.066	1.33	6 O
			2.00%	1 S	-6.532	1.00	86 H
			1.96%	1 P:z	-9.066	1.33	3 O
			1.62%	1 P:x	-9.066	1.33	6 O
			1.40%	1 P:z	-5.263	0.67	18 C
			1.39%	1 P:y	-5.263	0.67	14 C
			1.17%	1 S	-6.532	1.00	55 H
			1.14%	1 P:y	-5.263	0.67	20 C
			1.13%	1 P:y	-5.263	0.67	19 C
			1.08%	1 P:y	-9.066	1.33	6 O
			1.04%	1 S	-6.532	1.00	48 H
-8.579	2.00	99 A	8.23%	1 P:x	-9.066	1.33	5 O
			7.61%	1 S	-6.532	1.00	62 H
			5.48%	1 S	-6.532	1.00	64 H
			4.11%	1 P:y	-9.066	1.33	9 O
			3.92%	1 P:y	-5.263	0.67	33 C
			3.84%	1 P:x	-5.263	0.67	34 C
			3.82%	1 S	-6.532	1.00	60 H
			3.27%	1 S	-6.532	1.00	44 H
			3.15%	1 P:x	-9.066	1.33	2 O
			2.90%	1 P:z	-5.263	0.67	37 C
			2.75%	1 P:y	-5.263	0.67	34 C
			2.70%	1 S	-6.532	1.00	46 H
			2.60%	1 P:y	-5.263	0.67	37 C
			2.53%	1 S	-6.532	1.00	45 H
			2.40%	1 S	-6.532	1.00	65 H
			2.32%	1 S	-6.532	1.00	47 H
			1.99%	1 P:y	-5.263	0.67	40 C
			1.94%	1 P:x	-5.263	0.67	22 C

			1.77%	1 P:x	-5.263	0.67	40 C	
			1.64%	1 P:z	-5.263	0.67	40 C	
			1.36%	2 P:y	4.578	0.00	33 C	
			1.30%	1 P:x	-5.263	0.67	37 C	
			1.29%	1 P:x	-5.263	0.67	31 C	
-8.536	2.00	100 A	8.53%	1 P:x	-9.066	1.33	10 O	
			5.33%	1 S	-6.532	1.00	75 H	
			5.28%	1 P:z	-5.263	0.67	28 C	
			5.28%	1 S	-6.532	1.00	78 H	
			4.28%	1 P:x	-5.263	0.67	30 C	
			4.03%	1 P:x	-9.066	1.33	3 O	
			3.63%	1 P:x	-5.263	0.67	28 C	
			3.42%	1 S	-6.532	1.00	77 H	
			3.38%	1 P:x	-5.263	0.67	29 C	
			3.35%	1 S	-6.532	1.00	84 H	
			2.94%	1 S	-6.532	1.00	76 H	
			2.85%	1 P:y	-5.263	0.67	13 C	
			2.64%	1 P:y	-9.066	1.33	7 O	
			2.30%	1 P:x	-5.263	0.67	13 C	
			2.28%	1 P:x	-7.073	1.00	43 N	
			2.05%	1 P:x	-5.263	0.67	38 C	
			2.00%	1 P:z	-5.263	0.67	13 C	
			1.83%	1 S	-6.532	1.00	73 H	
			1.51%	1 P:x	-5.263	0.67	41 C	
			1.44%	1 P:x	-5.263	0.67	12 C	
			1.32%	1 P:z	-9.066	1.33	3 O	
			1.28%	1 S	-6.532	1.00	81 H	
			1.16%	1 S	-6.532	1.00	88 H	
			1.15%	1 P:z	-5.263	0.67	12 C	
			1.14%	1 S	-6.532	1.00	91 H	
			1.06%	1 P:y	-5.263	0.67	28 C	

-8.462	2.00	101 A	8.06%	1 P:z	-5.263	0.67	37 C
			7.40%	1 S	-6.532	1.00	62 H
			5.37%	1 S	-6.532	1.00	63 H
			5.29%	1 S	-6.532	1.00	61 H
			5.02%	1 P:z	-5.263	0.67	40 C
			4.80%	1 S	-6.532	1.00	64 H
			4.74%	1 P:x	-9.066	1.33	5 O
			2.10%	1 P:z	-9.066	1.33	9 O
			2.02%	1 P:z	-9.066	1.33	6 O
			1.75%	1 P:y	-5.263	0.67	20 C
			1.58%	1 P:z	-5.263	0.67	24 C
			1.50%	1 S	-6.532	1.00	60 H
			1.38%	1 S	-6.532	1.00	66 H
			1.36%	1 P:y	-9.066	1.33	9 O
			1.34%	1 P:y	-5.263	0.67	21 C
			1.24%	2 P:z	4.578	0.00	37 C
			1.23%	1 P:z	-5.263	0.67	20 C
			1.18%	1 P:y	-5.263	0.67	40 C
			1.16%	1 S	-6.532	1.00	65 H
			1.13%	1 P:y	-9.066	1.33	6 O
			1.12%	1 P:y	-9.066	1.33	8 O
			1.12%	1 S	-6.532	1.00	68 H
			1.09%	1 P:y	-9.066	1.33	2 O
			1.08%	1 P:x	-5.263	0.67	33 C
			1.04%	1 S	-6.532	1.00	49 H
-8.351	2.00	102 A	7.33%	1 P:z	-9.066	1.33	8 O
			6.14%	1 P:x	-9.066	1.33	8 O
			4.17%	1 P:z	-5.263	0.67	35 C
			3.61%	1 S	-6.532	1.00	53 H
			3.61%	1 P:x	-5.263	0.67	19 C
			3.38%	1 S	-6.532	1.00	55 H

			2.96%	1 S	-6.532	1.00	46 H
			2.66%	1 P:z	-9.066	1.33	4 O
			2.59%	1 P:y	-9.066	1.33	1 O
			2.57%	1 S	-6.532	1.00	59 H
			2.51%	1 P:z	-9.066	1.33	3 O
			2.47%	1 P:y	-5.263	0.67	27 C
			2.24%	1 P:x	-5.263	0.67	36 C
			2.17%	1 P:y	-5.263	0.67	34 C
			2.03%	1 P:y	-9.066	1.33	4 O
			2.02%	1 P:x	-5.263	0.67	27 C
			1.98%	1 P:y	-5.263	0.67	19 C
			1.90%	1 S	-6.532	1.00	54 H
			1.70%	1 P:y	-5.263	0.67	36 C
			1.27%	1 S	-6.532	1.00	49 H
			1.25%	1 P:z	-5.263	0.67	39 C
			1.21%	2 P:x	4.578	0.00	19 C
			1.16%	1 P:x	-9.066	1.33	4 O
			1.11%	1 P:z	-5.263	0.67	15 C
-8.332	2.00	103 A	8.10%	1 P:y	-9.066	1.33	6 O
			6.25%	1 P:z	-9.066	1.33	6 O
			3.43%	1 S	-6.532	1.00	50 H
			3.26%	1 S	-6.532	1.00	49 H
			2.63%	1 P:z	-5.263	0.67	24 C
			2.61%	1 P:z	-5.263	0.67	15 C
			2.44%	1 P:z	-5.263	0.67	20 C
			2.22%	1 S	-6.532	1.00	61 H
			2.12%	1 S	-6.532	1.00	90 H
			1.85%	1 P:x	-5.263	0.67	35 C
			1.76%	1 P:z	-5.263	0.67	17 C
			1.70%	1 P:y	-9.066	1.33	8 O
			1.67%	1 P:z	-5.263	0.67	27 C

			1.59%	1 P:z	-5.263	0.67	32 C	
			1.59%	1 P:y	-5.263	0.67	15 C	
			1.51%	1 S	-6.532	1.00	63 H	
			1.51%	1 P:z	-5.263	0.67	37 C	
			1.47%	1 P:y	-5.263	0.67	37 C	
			1.39%	1 S	-6.532	1.00	69 H	
			1.21%	1 S	-6.532	1.00	65 H	
			1.17%	1 P:z	-9.066	1.33	8 O	
			1.15%	1 P:z	-5.263	0.67	14 C	
			1.15%	1 S	-6.532	1.00	55 H	
			1.13%	1 P:z	-9.066	1.33	4 O	
			1.10%	1 P:y	-5.263	0.67	14 C	
			1.03%	1 P:x	-5.263	0.67	31 C	
-8.236	2.00	104 A	10.51%	1 S	-6.532	1.00	88 H	
			9.84%	1 S	-6.532	1.00	87 H	
			6.58%	1 P:z	-5.263	0.67	13 C	
			5.81%	1 P:z	-9.066	1.33	7 O	
			5.61%	1 P:y	-9.066	1.33	7 O	
			5.09%	1 P:z	-5.263	0.67	12 C	
			4.58%	1 P:x	-9.066	1.33	10 O	
			2.83%	1 P:y	-9.066	1.33	3 O	
			2.29%	1 P:x	-7.073	1.00	43 N	
			2.14%	1 P:z	-9.066	1.33	3 O	
			1.98%	1 P:y	-5.263	0.67	12 C	
			1.86%	1 P:x	-5.263	0.67	12 C	
			1.71%	1 P:x	-5.263	0.67	13 C	
			1.69%	1 P:x	-5.263	0.67	26 C	
			1.66%	1 P:y	-5.263	0.67	30 C	
			1.46%	1 S	-23.822	2.00	7 O	
			1.44%	1 S	-6.532	1.00	77 H	
			1.35%	1 P:y	-5.263	0.67	29 C	

			1.11%	1 P:y	-5.263	0.67	26 C		
			1.03%	2 P:z	4.578	0.00	13 C		
-8.170	2.00	105 A	7.48%	1 S	-6.532	1.00	50 H		
			5.06%	1 P:y	-9.066	1.33	8 O		
			3.62%	1 P:x	-5.263	0.67	35 C		
			3.19%	1 P:z	-9.066	1.33	6 O		
			3.07%	1 P:y	-9.066	1.33	6 O		
			3.05%	1 P:z	-9.066	1.33	8 O		
			2.97%	1 P:x	-5.263	0.67	15 C		
			2.28%	1 P:z	-9.066	1.33	4 O		
			2.05%	1 P:z	-5.263	0.67	24 C		
			2.00%	1 P:z	-5.263	0.67	20 C		
			1.99%	1 S	-6.532	1.00	56 H		
			1.97%	1 S	-6.532	1.00	55 H		
			1.93%	1 S	-6.532	1.00	46 H		
			1.55%	1 P:y	-5.263	0.67	13 C		
			1.53%	1 P:y	-5.263	0.67	15 C		
			1.50%	1 P:x	-9.066	1.33	8 O		
			1.49%	1 P:z	-5.263	0.67	36 C		
			1.48%	1 P:y	-5.263	0.67	28 C		
			1.43%	1 S	-6.532	1.00	58 H		
			1.42%	1 S	-6.532	1.00	77 H		
			1.39%	1 P:y	-5.263	0.67	34 C		
			1.38%	1 S	-6.532	1.00	49 H		
			1.25%	1 P:y	-5.263	0.67	35 C		
			1.18%	1 P:y	-5.263	0.67	20 C		
			1.18%	1 P:z	-5.263	0.67	39 C		
			1.16%	1 P:x	-5.263	0.67	19 C		
			1.08%	1 P:z	-5.263	0.67	19 C		
			1.07%	1 P:x	-9.066	1.33	1 O		
			1.04%	1 P:z	-5.263	0.67	15 C		

-8.071	2.00	106 A	13.61%	1 P:y	-9.066	1.33	5 O
			8.82%	1 P:x	-9.066	1.33	2 O
			6.52%	1 P:x	-9.066	1.33	9 O
			5.25%	1 P:y	-5.263	0.67	37 C
			3.13%	1 S	-6.532	1.00	61 H
			3.07%	1 S	-6.532	1.00	51 H
			2.71%	1 P:y	-5.263	0.67	33 C
			2.64%	1 P:z	-9.066	1.33	8 O
			2.13%	1 S	-6.532	1.00	44 H
			2.12%	1 S	-6.532	1.00	60 H
			1.87%	1 S	-6.532	1.00	48 H
			1.41%	1 P:y	-5.263	0.67	22 C
			1.33%	1 S	-6.532	1.00	52 H
			1.32%	1 P:y	-5.263	0.67	15 C
			1.28%	1 P:y	-5.263	0.67	23 C
			1.20%	1 P:x	-5.263	0.67	34 C
			1.17%	1 P:z	-9.066	1.33	9 O
			1.14%	1 P:y	-5.263	0.67	25 C
			1.13%	1 P:x	-5.263	0.67	17 C
			1.06%	1 S	-23.822	2.00	2 O
			1.03%	1 P:x	-5.263	0.67	33 C
			1.02%	2 P:y	4.578	0.00	33 C
			1.00%	1 S	-6.532	1.00	63 H
-7.992	2.00	107 A	6.21%	1 S	-6.532	1.00	51 H
			4.85%	1 P:y	-5.263	0.67	15 C
			4.80%	1 P:y	-5.263	0.67	25 C
			4.10%	1 S	-6.532	1.00	48 H
			3.59%	1 S	-6.532	1.00	52 H
			3.31%	1 P:y	-9.066	1.33	6 O
			3.28%	1 P:x	-9.066	1.33	9 O
			3.20%	1 P:z	-9.066	1.33	4 O

			2.69%	1 P:x	-9.066	1.33	1	O
			2.33%	1 P:y	-5.263	0.67	27	C
			2.30%	1 P:y	-9.066	1.33	5	O
			2.26%	1 P:y	-9.066	1.33	4	O
			1.86%	1 P:x	-9.066	1.33	2	O
			1.70%	1 S	-6.532	1.00	53	H
			1.48%	1 P:x	-5.263	0.67	25	C
			1.34%	1 P:z	-9.066	1.33	8	O
			1.34%	1 P:y	-5.263	0.67	13	C
			1.30%	1 P:y	-5.263	0.67	28	C
			1.29%	1 P:z	-9.066	1.33	1	O
			1.15%	1 S	-6.532	1.00	77	H
			1.08%	1 S	-6.532	1.00	76	H
			1.08%	1 P:x	-9.066	1.33	7	O
			1.04%	1 P:y	-5.263	0.67	30	C
			1.04%	1 S	-6.532	1.00	44	H
			1.02%	1 P:z	-9.066	1.33	6	O
-7.910	2.00	108 A	5.60%	1 P:z	-9.066	1.33	3	O
			4.14%	1 P:z	-5.263	0.67	27	C
			3.38%	1 S	-6.532	1.00	53	H
			3.36%	1 P:z	-5.263	0.67	25	C
			3.31%	1 S	-6.532	1.00	86	H
			3.28%	1 P:y	-9.066	1.33	3	O
			3.06%	1 S	-6.532	1.00	49	H
			2.97%	1 P:z	-9.066	1.33	1	O
			2.78%	1 P:z	-9.066	1.33	8	O
			2.75%	1 P:y	-9.066	1.33	1	O
			2.75%	1 P:x	-9.066	1.33	1	O
			2.42%	1 P:z	-5.263	0.67	15	C
			2.14%	1 S	-6.532	1.00	85	H
			2.00%	1 P:y	-9.066	1.33	7	O

			1.58%	1 S	-6.532	1.00	87 H
			1.56%	1 P:x	-9.066	1.33	8 O
			1.52%	1 P:z	-9.066	1.33	4 O
			1.44%	1 P:y	-5.263	0.67	15 C
			1.43%	1 P:x	-5.263	0.67	28 C
			1.33%	1 P:y	-5.263	0.67	27 C
			1.26%	1 S	-6.532	1.00	76 H
			1.25%	1 P:y	-5.263	0.67	29 C
			1.23%	1 S	-6.532	1.00	78 H
			1.23%	1 P:y	-5.263	0.67	30 C
			1.15%	1 S	-23.822	2.00	3 O
			1.13%	1 P:z	-5.263	0.67	18 C
			1.10%	2 P:z	4.578	0.00	25 C
-7.853	2.00	109 A	4.68%	1 P:y	-9.066	1.33	3 O
			3.59%	1 S	-6.532	1.00	53 H
			3.43%	1 P:y	-5.263	0.67	15 C
			3.40%	1 P:y	-5.263	0.67	25 C
			3.22%	1 P:y	-5.263	0.67	30 C
			2.95%	1 P:x	-9.066	1.33	7 O
			2.76%	1 P:y	-5.263	0.67	29 C
			2.74%	1 S	-6.532	1.00	52 H
			2.74%	1 P:x	-9.066	1.33	1 O
			2.68%	1 S	-6.532	1.00	51 H
			2.53%	1 S	-6.532	1.00	76 H
			2.49%	1 S	-6.532	1.00	86 H
			2.37%	1 P:y	-5.263	0.67	27 C
			2.08%	1 P:y	-5.263	0.67	28 C
			2.02%	1 P:y	-5.263	0.67	13 C
			1.93%	1 S	-6.532	1.00	49 H
			1.87%	1 P:y	-9.066	1.33	7 O
			1.80%	1 P:z	-9.066	1.33	3 O

			1.67%	1 P:z	-5.263	0.67	15 C
			1.53%	1 P:y	-9.066	1.33	4 O
			1.52%	1 P:z	-5.263	0.67	19 C
			1.50%	1 P:z	-5.263	0.67	18 C
			1.42%	1 S	-6.532	1.00	77 H
			1.25%	1 P:z	-5.263	0.67	27 C
			1.22%	1 S	-6.532	1.00	56 H
			1.10%	1 P:x	-5.263	0.67	27 C
			1.03%	1 P:z	-9.066	1.33	1 O
-7.704	2.00	110 A	11.69%	1 P:x	-9.066	1.33	1 O
			9.08%	1 P:z	-9.066	1.33	1 O
			5.11%	1 P:z	-5.263	0.67	25 C
			4.37%	1 S	-6.532	1.00	85 H
			4.15%	1 S	-6.532	1.00	49 H
			3.87%	1 P:z	-5.263	0.67	27 C
			3.34%	1 S	-6.532	1.00	51 H
			3.29%	1 P:x	-5.263	0.67	27 C
			2.99%	1 S	-6.532	1.00	53 H
			2.84%	1 P:z	-5.263	0.67	15 C
			2.83%	1 S	-6.532	1.00	86 H
			2.51%	1 P:z	-9.066	1.33	8 O
			2.22%	1 S	-6.532	1.00	48 H
			1.76%	1 S	-6.532	1.00	52 H
			1.60%	1 S	-23.822	2.00	1 O
			1.56%	1 P:z	-9.066	1.33	3 O
			1.41%	1 P:x	-9.066	1.33	3 O
			1.38%	1 P:x	-5.263	0.67	25 C
			1.37%	1 P:x	-5.263	0.67	18 C
			1.20%	1 P:x	-5.263	0.67	28 C
			1.19%	2 P:z	4.578	0.00	27 C
			1.05%	1 P:y	-5.263	0.67	27 C

			1.03%	1 P:y	-9.066	1.33	5 O
			1.00%	1 P:z	-5.263	0.67	11 C
-7.462	2.00	111 A	16.66%	1 P:y	-9.066	1.33	9 O
			13.58%	1 P:x	-9.066	1.33	5 O
			9.41%	1 P:y	-9.066	1.33	5 O
			5.84%	1 P:x	-5.263	0.67	23 C
			5.39%	1 P:y	-9.066	1.33	2 O
			5.03%	1 P:x	-9.066	1.33	2 O
			3.76%	1 P:z	-9.066	1.33	9 O
			3.13%	1 P:x	-5.263	0.67	33 C
			2.61%	1 P:x	-9.066	1.33	9 O
			2.15%	1 P:x	-5.263	0.67	21 C
			2.11%	1 P:y	-5.263	0.67	37 C
			1.69%	1 S	-6.532	1.00	68 H
			1.60%	1 P:z	-5.263	0.67	21 C
			1.54%	2 P:x	4.578	0.00	23 C
			1.47%	1 P:y	-5.263	0.67	33 C
			1.16%	1 S	-23.822	2.00	2 O
			1.10%	1 P:x	-5.263	0.67	17 C
-7.292	2.00	112 A	31.28%	1 P:x	-9.066	1.33	7 O
			14.66%	1 P:x	-9.066	1.33	10 O
			6.12%	1 P:y	-9.066	1.33	3 O
			6.08%	1 P:z	-9.066	1.33	10 O
			4.23%	1 P:x	-9.066	1.33	3 O
			3.89%	1 S	-6.532	1.00	76 H
			3.41%	1 P:z	-9.066	1.33	7 O
			3.21%	1 P:z	-9.066	1.33	3 O
			1.94%	1 P:x	-5.263	0.67	11 C
			1.43%	1 P:y	-9.066	1.33	10 O
			1.12%	1 P:x	-5.263	0.67	12 C
			1.09%	1 P:x	-9.066	1.33	1 O

-7.248	2.00	113 A	21.57%	1 P:y	-9.066	1.33	4 O
			10.11%	1 P:z	-9.066	1.33	4 O
			5.98%	1 P:y	-5.263	0.67	36 C
			3.86%	1 P:z	-5.263	0.67	19 C
			2.81%	1 S	-6.532	1.00	57 H
			2.80%	1 P:y	-9.066	1.33	1 O
			2.79%	1 S	-6.532	1.00	59 H
			2.66%	1 S	-23.822	2.00	4 O
			2.59%	1 P:y	-5.263	0.67	39 C
			2.27%	1 P:z	-5.263	0.67	27 C
			2.26%	1 P:y	-5.263	0.67	19 C
			2.14%	1 S	-6.532	1.00	55 H
			2.05%	1 P:z	-5.263	0.67	36 C
			2.00%	1 P:y	-5.263	0.67	27 C
			1.97%	1 P:z	-9.066	1.33	8 O
			1.72%	1 S	-6.532	1.00	53 H
			1.70%	2 P:z	4.578	0.00	19 C
			1.43%	1 S	-6.532	1.00	58 H
			1.42%	1 P:x	-9.066	1.33	7 O
			1.38%	1 S	-6.532	1.00	51 H
			1.34%	1 P:z	-5.263	0.67	39 C
			1.17%	1 P:y	-5.263	0.67	15 C
			1.11%	1 P:z	-5.263	0.67	25 C
			1.08%	1 S	-6.532	1.00	52 H
			1.07%	2 P:y	4.578	0.00	19 C
			1.03%	1 P:y	-5.263	0.67	25 C
-6.761	2.00	114 A	45.64%	1 P:y	-9.066	1.33	1 O
			5.58%	1 P:y	-5.263	0.67	24 C
			5.02%	1 P:z	-9.066	1.33	4 O
			3.05%	1 P:y	-9.066	1.33	3 O
			2.75%	1 P:y	-9.066	1.33	4 O

			2.75%	1 S	-6.532	1.00	86 H
			2.47%	1 P:x	-9.066	1.33	6 O
			1.70%	1 P:y	-5.263	0.67	18 C
			1.44%	1 S	-6.532	1.00	85 H
			1.33%	1 S	-6.532	1.00	58 H
			1.27%	1 S	-6.532	1.00	57 H
			1.21%	1 S	-6.532	1.00	69 H
			1.20%	1 P:x	-9.066	1.33	4 O
			1.19%	1 P:y	-9.066	1.33	6 O
			1.08%	2 P:y	4.578	0.00	18 C
			1.06%	1 P:y	-5.263	0.67	19 C
			1.00%	1 P:y	-5.263	0.67	27 C
-6.738	2.00	115 A	37.47%	1 P:z	-9.066	1.33	9 O
			10.79%	1 P:x	-9.066	1.33	9 O
			7.99%	1 P:y	-9.066	1.33	2 O
			5.48%	1 P:z	-9.066	1.33	5 O
			4.18%	1 P:y	-9.066	1.33	9 O
			3.58%	1 P:x	-9.066	1.33	2 O
			3.05%	1 P:x	-9.066	1.33	5 O
			2.52%	1 P:x	-5.263	0.67	17 C
			1.53%	1 S	-6.532	1.00	95 H
			1.44%	1 P:z	-9.066	1.33	2 O
			1.13%	1 P:x	-5.263	0.67	32 C
			1.03%	1 D:z2	33.808	0.00	16 C
-6.456	2.00	116 A	22.18%	1 P:y	-9.066	1.33	10 O
			19.97%	1 P:z	-9.066	1.33	10 O
			7.34%	1 P:y	-9.066	1.33	2 O
			6.91%	1 P:z	-5.263	0.67	22 C
			5.32%	1 P:z	-5.263	0.67	31 C
			4.72%	1 P:x	-9.066	1.33	2 O
			4.21%	1 P:z	-9.066	1.33	7 O

			3.12%	1 P:y	-5.263	0.67	42 C
			1.43%	1 P:x	-9.066	1.33	8 O
			1.35%	1 P:y	-5.263	0.67	22 C
			1.32%	1 P:x	-9.066	1.33	10 O
			1.27%	1 P:z	-9.066	1.33	2 O
			1.03%	1 S	-6.532	1.00	92 H
-6.403	2.00	117 A	20.90%	1 P:y	-9.066	1.33	2 O
			14.52%	1 P:z	-9.066	1.33	10 O
			13.42%	1 P:y	-9.066	1.33	10 O
			9.70%	1 P:x	-9.066	1.33	2 O
			4.21%	1 P:z	-5.263	0.67	22 C
			2.84%	1 P:y	-5.263	0.67	22 C
			2.62%	1 P:z	-9.066	1.33	7 O
			2.48%	1 P:z	-5.263	0.67	31 C
			2.31%	1 P:z	-9.066	1.33	2 O
			2.09%	1 P:y	-5.263	0.67	42 C
			1.43%	1 P:x	-9.066	1.33	8 O
			1.18%	1 S	-6.532	1.00	47 H
			1.17%	1 P:x	-5.263	0.67	33 C
			1.02%	1 P:z	-5.263	0.67	34 C
-6.174	2.00	118 A	52.87%	1 P:x	-9.066	1.33	3 O
			6.26%	1 S	-6.532	1.00	85 H
			4.24%	1 S	-6.532	1.00	76 H
			4.19%	1 P:x	-5.263	0.67	12 C
			3.21%	1 P:x	-9.066	1.33	7 O
			3.12%	1 P:x	-5.263	0.67	28 C
			1.76%	1 P:x	-9.066	1.33	10 O
			1.75%	1 P:z	-9.066	1.33	3 O
			1.71%	1 P:x	-5.263	0.67	11 C
			1.39%	1 P:y	-9.066	1.33	3 O
			1.34%	1 P:x	-5.263	0.67	29 C

			1.28%	1 S	-6.532	1.00	78 H
			1.09%	1 P:x	-9.066	1.33	6 O
			1.04%	2 P:x	4.578	0.00	11 C
-6.026	2.00	119 A	20.89%	1 P:z	-9.066	1.33	4 O
			15.86%	1 P:y	-9.066	1.33	4 O
			7.41%	1 P:x	-9.066	1.33	6 O
			6.23%	1 P:y	-9.066	1.33	6 O
			4.18%	1 P:z	-5.263	0.67	18 C
			3.61%	1 S	-6.532	1.00	58 H
			2.99%	1 P:y	-9.066	1.33	2 O
			2.93%	1 S	-6.532	1.00	57 H
			2.88%	1 P:x	-9.066	1.33	4 O
			1.85%	1 P:z	-5.263	0.67	22 C
			1.85%	1 P:y	-9.066	1.33	1 O
			1.62%	1 P:z	-5.263	0.67	31 C
			1.59%	1 P:z	-5.263	0.67	19 C
			1.30%	1 P:z	-9.066	1.33	6 O
			1.25%	1 P:x	-5.263	0.67	27 C
			1.16%	1 P:x	-5.263	0.67	24 C
			1.03%	1 P:y	-5.263	0.67	36 C
-5.989	2.00	120 A	22.36%	1 P:z	-5.263	0.67	22 C
			18.83%	1 P:z	-5.263	0.67	31 C
			14.31%	1 P:y	-9.066	1.33	2 O
			3.76%	1 P:x	-9.066	1.33	2 O
			3.21%	1 P:z	-9.066	1.33	8 O
			2.86%	1 P:x	-9.066	1.33	6 O
			2.33%	1 P:y	-9.066	1.33	8 O
			2.24%	1 P:x	-5.263	0.67	22 C
			2.06%	1 S	-6.532	1.00	45 H
			1.71%	1 P:y	-9.066	1.33	6 O
			1.60%	1 S	-6.532	1.00	47 H

			1.35%	1 P:x	-5.263	0.67	33 C
			1.30%	1 P:x	-5.263	0.67	31 C
			1.19%	1 P:z	-9.066	1.33	6 O
			1.15%	1 P:z	-5.263	0.67	34 C
-5.862	2.00	121 A	19.61%	1 P:x	-9.066	1.33	6 O
			14.41%	1 P:z	-9.066	1.33	6 O
			8.57%	1 P:y	-9.066	1.33	1 O
			6.08%	1 P:y	-9.066	1.33	6 O
			6.00%	1 P:x	-5.263	0.67	24 C
			5.76%	1 P:z	-9.066	1.33	4 O
			5.33%	1 P:y	-9.066	1.33	4 O
			2.79%	1 P:y	-5.263	0.67	17 C
			2.48%	1 P:z	-5.263	0.67	17 C
			1.77%	1 P:y	-5.263	0.67	18 C
			1.58%	1 P:x	-5.263	0.67	17 C
			1.00%	1 S	-6.532	1.00	58 H
-5.296	2.00	122 A	47.26%	1 P:y	-9.066	1.33	8 O
			15.74%	1 P:x	-9.066	1.33	8 O
			7.22%	1 P:y	-5.263	0.67	31 C
			6.31%	1 P:x	-5.263	0.67	25 C
			1.93%	1 P:z	-5.263	0.67	25 C
			1.43%	1 D:x2-y2	33.808	0.00	35 C
			1.39%	1 P:y	-5.263	0.67	25 C
			1.26%	1 S	-13.631	2.00	31 C
			1.18%	1 P:y	-5.263	0.67	35 C
			1.11%	2 P:y	4.578	0.00	35 C
			1.10%	1 P:y	-9.066	1.33	2 O
			1.07%	1 P:z	-9.066	1.33	8 O
-4.806	2.00	123 A	49.37%	1 P:z	-7.073	1.00	43 N
			11.97%	1 P:y	-7.073	1.00	43 N
			7.76%	1 S	-6.532	1.00	82 H

			7.11%	1 S	-6.532	1.00	79 H		
			3.89%	1 S	-18.438	2.00	43 N		
			3.64%	1 P:z	-5.263	0.67	12 C		
			1.53%	1 P:x	-7.073	1.00	43 N		
			1.50%	1 P:y	-5.263	0.67	28 C		
			1.39%	1 S	-6.532	1.00	84 H		
			1.12%	1 S	-6.532	1.00	81 H		
-1.892	0.00	124 A	27.22%	1 P:y	-5.263	0.67	14 C		
			22.05%	1 P:z	-5.263	0.67	14 C		
			14.09%	1 P:y	-9.066	1.33	6 O		
			12.18%	1 P:z	-9.066	1.33	6 O		
			5.50%	1 S	-6.532	1.00	90 H		
			2.54%	1 P:z	-5.263	0.67	20 C		
			-1.53%	2 P:y	4.578	0.00	14 C		
			1.24%	1 P:y	-5.263	0.67	18 C		
			1.23%	1 S	-6.532	1.00	71 H		
			1.15%	1 P:z	-5.263	0.67	35 C		
			-1.14%	2 P:z	9.420	0.00	6 O		
			1.11%	1 P:z	-5.263	0.67	22 C		
			-1.07%	2 P:y	9.420	0.00	6 O		
			1.03%	1 P:x	-5.263	0.67	32 C		
-1.865	0.00	125 A	23.20%	1 P:z	-5.263	0.67	35 C		
			18.44%	1 P:z	-5.263	0.67	22 C		
			15.29%	1 P:z	-9.066	1.33	8 O		
			10.60%	1 P:z	-5.263	0.67	31 C		
			4.78%	1 P:x	-5.263	0.67	35 C		
			4.05%	1 P:x	-9.066	1.33	8 O		
			3.76%	1 P:x	-5.263	0.67	22 C		
			-1.46%	2 P:z	4.578	0.00	31 C		
			1.33%	1 P:z	-5.263	0.67	14 C		
			1.26%	1 P:y	-5.263	0.67	14 C		

			-1.25%	2 P:z	9.420	0.00	8 O
-1.708	0.00	126 A	30.91%	1 P:y	-5.263	0.67	16 C
			19.44%	1 P:x	-5.263	0.67	16 C
			13.45%	1 P:y	-9.066	1.33	9 O
			9.69%	1 S	-6.532	1.00	90 H
			7.90%	1 P:x	-9.066	1.33	9 O
			5.17%	1 P:y	-9.066	1.33	5 O
			5.04%	1 P:x	-9.066	1.33	5 O
			1.69%	1 P:y	-5.263	0.67	14 C
			-1.67%	2 P:x	4.578	0.00	16 C
			-1.59%	2 P:y	4.578	0.00	16 C
			1.46%	1 S	-13.631	2.00	14 C
			-1.30%	2 P:y	9.420	0.00	9 O
-1.066	0.00	127 A	41.15%	1 P:x	-5.263	0.67	41 C
			20.27%	1 P:x	-9.066	1.33	10 O
			9.80%	1 S	-6.532	1.00	93 H
			9.13%	1 P:z	-5.263	0.67	41 C
			6.84%	1 P:x	-9.066	1.33	7 O
			4.44%	1 P:z	-9.066	1.33	10 O
			3.62%	1 P:y	-5.263	0.67	41 C
			-2.65%	2 P:x	4.578	0.00	41 C
			2.14%	1 S	-6.532	1.00	91 H
			-1.89%	2 P:x	9.420	0.00	10 O
			1.59%	1 P:z	-9.066	1.33	7 O
			1.55%	1 S	-6.532	1.00	92 H
			1.28%	1 P:y	-9.066	1.33	10 O
			-1.21%	2 S	2.394	0.00	93 H
			-1.08%	2 S	2.394	0.00	91 H
-0.508	0.00	128 A	12.45%	2 S	2.394	0.00	64 H
			11.44%	2 S	2.394	0.00	60 H
			7.43%	2 S	2.394	0.00	96 H

			6.36%	1 S	-6.532	1.00	60 H
			5.91%	2 S	2.394	0.00	65 H
			5.83%	2 S	2.394	0.00	90 H
			5.52%	2 S	2.394	0.00	67 H
			5.37%	2 S	2.394	0.00	61 H
			5.18%	1 S	-6.532	1.00	64 H
			4.33%	2 S	2.394	0.00	63 H
			3.12%	1 S	-6.532	1.00	96 H
			2.92%	1 S	-6.532	1.00	67 H
			2.86%	1 S	-6.532	1.00	61 H
			2.50%	1 S	-6.532	1.00	65 H
			2.46%	1 P:z	-5.263	0.67	33 C
			-2.24%	2 S	11.741	0.00	40 C
			2.12%	2 S	2.394	0.00	51 H
			2.10%	2 S	2.394	0.00	44 H
			1.95%	1 S	-6.532	1.00	63 H
			1.91%	1 S	-6.532	1.00	90 H
			1.86%	2 S	2.394	0.00	92 H
			1.68%	1 S	-6.532	1.00	51 H
			1.58%	2 S	2.394	0.00	66 H
			1.54%	2 S	2.394	0.00	72 H
			1.44%	2 S	2.394	0.00	91 H
			1.32%	2 S	2.394	0.00	94 H
			-1.26%	2 S	2.394	0.00	62 H
			-1.14%	2 P:x	4.578	0.00	31 C
			-1.13%	2 S	11.741	0.00	32 C
			-1.07%	1 S	-6.532	1.00	62 H
			-1.00%	2 S	2.394	0.00	71 H
-0.349	0.00	129 A	17.96%	2 S	2.394	0.00	76 H
			9.45%	1 S	-6.532	1.00	76 H
			8.21%	2 S	2.394	0.00	70 H

7.69%	2 S	2.394	0.00	88 H
6.02%	2 S	2.394	0.00	78 H
5.44%	2 S	2.394	0.00	83 H
5.04%	2 S	2.394	0.00	74 H
3.98%	1 S	-6.532	1.00	78 H
3.80%	2 S	2.394	0.00	60 H
3.80%	2 S	2.394	0.00	75 H
3.75%	1 P:z	-5.263	0.67	31 C
3.63%	1 S	-6.532	1.00	88 H
3.63%	1 S	-6.532	1.00	70 H
3.30%	2 S	2.394	0.00	67 H
2.69%	2 S	2.394	0.00	86 H
2.68%	2 S	2.394	0.00	85 H
2.63%	1 S	-6.532	1.00	74 H
2.49%	1 S	-6.532	1.00	83 H
2.41%	1 S	-6.532	1.00	60 H
-2.27%	2 S	2.394	0.00	77 H
2.05%	1 P:z	-5.263	0.67	35 C
2.03%	2 S	2.394	0.00	61 H
1.99%	2 S	2.394	0.00	64 H
-1.80%	2 P:z	4.578	0.00	28 C
-1.70%	2 S	2.394	0.00	71 H
1.69%	1 S	-6.532	1.00	75 H
-1.67%	2 P:z	4.578	0.00	12 C
1.62%	2 S	2.394	0.00	92 H
-1.61%	2 S	2.394	0.00	73 H
-1.60%	2 P:x	4.578	0.00	29 C
1.57%	1 S	-6.532	1.00	86 H
1.56%	1 P:z	-5.263	0.67	22 C
-1.55%	2 S	2.394	0.00	87 H
-1.52%	2 P:z	4.578	0.00	13 C

			-1.49%	2 P:z	4.578	0.00	33 C
			1.39%	2 S	2.394	0.00	44 H
			1.38%	2 S	2.394	0.00	55 H
			1.38%	1 S	-6.532	1.00	61 H
			-1.37%	2 P:z	4.578	0.00	31 C
			-1.36%	2 P:z	4.578	0.00	18 C
			1.35%	1 S	-6.532	1.00	67 H
			1.27%	1 P:x	-5.263	0.67	35 C
			1.04%	1 S	-6.532	1.00	87 H
			-1.03%	2 P:z	4.578	0.00	29 C
			-1.03%	2 P:x	4.578	0.00	28 C
			1.02%	1 S	-6.532	1.00	85 H
			-1.01%	2 S	11.741	0.00	30 C
			1.01%	1 P:z	-9.066	1.33	8 O
-0.301	0.00	130 A	17.81%	1 P:z	-5.263	0.67	31 C
			11.73%	1 P:z	-5.263	0.67	35 C
			8.30%	1 P:z	-5.263	0.67	22 C
			5.77%	2 S	2.394	0.00	76 H
			5.73%	1 P:z	-9.066	1.33	8 O
			-5.07%	2 P:z	4.578	0.00	31 C
			4.86%	1 P:x	-5.263	0.67	35 C
			3.60%	2 S	2.394	0.00	74 H
			3.22%	2 S	2.394	0.00	78 H
			2.82%	1 S	-6.532	1.00	47 H
			2.80%	1 S	-6.532	1.00	76 H
			2.63%	2 S	2.394	0.00	75 H
			2.52%	1 P:x	-5.263	0.67	31 C
			2.38%	2 S	2.394	0.00	67 H
			2.22%	1 S	-6.532	1.00	78 H
			1.87%	1 S	-6.532	1.00	74 H
			1.62%	1 S	-6.532	1.00	45 H

			1.54%	2 S	2.394	0.00	60 H
			-1.39%	2 S	2.394	0.00	51 H
			1.32%	2 S	2.394	0.00	88 H
			1.28%	2 S	2.394	0.00	44 H
			1.25%	2 S	2.394	0.00	55 H
			1.22%	1 S	-6.532	1.00	75 H
			1.14%	1 P:z	-5.263	0.67	27 C
			-1.08%	2 P:z	4.578	0.00	33 C
			1.07%	1 P:x	-9.066	1.33	8 O
			1.06%	1 S	-6.532	1.00	66 H
-0.187	0.00	131 A	13.00%	2 S	2.394	0.00	92 H
			6.98%	2 S	2.394	0.00	83 H
			6.01%	1 S	-6.532	1.00	92 H
			5.30%	2 S	2.394	0.00	75 H
			5.25%	2 S	2.394	0.00	91 H
			4.74%	2 S	2.394	0.00	82 H
			4.69%	2 S	2.394	0.00	84 H
			4.37%	2 S	2.394	0.00	88 H
			3.44%	2 S	2.394	0.00	55 H
			3.19%	2 S	2.394	0.00	81 H
			3.16%	1 S	-6.532	1.00	83 H
			-3.06%	2 S	2.394	0.00	93 H
			-2.94%	2 P:y	4.578	0.00	13 C
			2.83%	2 S	2.394	0.00	73 H
			2.74%	1 S	-6.532	1.00	75 H
			2.52%	1 S	-6.532	1.00	91 H
			2.43%	1 S	-6.532	1.00	88 H
			2.30%	2 S	2.394	0.00	74 H
			2.12%	2 S	2.394	0.00	57 H
			2.11%	2 S	2.394	0.00	79 H
			2.09%	2 S	2.394	0.00	72 H

			2.06%	1 S	-6.532	1.00	84 H
			-1.93%	2 P:y	4.578	0.00	29 C
			1.81%	2 S	2.394	0.00	89 H
			1.66%	2 S	2.394	0.00	59 H
			1.65%	1 S	-6.532	1.00	82 H
			1.64%	1 S	-6.532	1.00	81 H
			1.62%	2 S	2.394	0.00	58 H
			1.53%	1 P:z	-5.263	0.67	31 C
			-1.49%	2 S	11.741	0.00	42 C
			-1.46%	2 S	11.741	0.00	38 C
			1.43%	2 S	2.394	0.00	86 H
			-1.40%	2 P:y	4.578	0.00	18 C
			1.38%	1 S	-6.532	1.00	55 H
			1.24%	1 S	-6.532	1.00	86 H
			1.21%	2 S	2.394	0.00	95 H
			1.20%	1 S	-6.532	1.00	73 H
			-1.20%	2 P:y	4.578	0.00	26 C
			1.19%	1 S	-6.532	1.00	74 H
			1.18%	1 S	-6.532	1.00	47 H
			1.17%	2 S	2.394	0.00	50 H
			1.17%	2 S	2.394	0.00	47 H
			1.17%	2 S	2.394	0.00	96 H
			1.11%	2 S	2.394	0.00	94 H
			-1.01%	1 S	-6.532	1.00	93 H
-0.121	0.00	132 A	9.21%	2 S	2.394	0.00	73 H
			8.59%	2 S	2.394	0.00	77 H
			8.18%	2 S	2.394	0.00	80 H
			5.31%	1 S	-6.532	1.00	77 H
			4.87%	2 S	2.394	0.00	81 H
			4.56%	1 S	-6.532	1.00	73 H
			4.08%	1 S	-6.532	1.00	80 H

-3.38%	2 P:y	4.578	0.00	29 C
3.27%	2 S	2.394	0.00	54 H
3.12%	2 S	2.394	0.00	70 H
3.10%	1 S	-6.532	1.00	76 H
2.92%	2 S	2.394	0.00	90 H
2.72%	1 S	-6.532	1.00	85 H
2.71%	2 S	2.394	0.00	87 H
2.62%	2 S	2.394	0.00	84 H
2.58%	2 S	2.394	0.00	65 H
2.48%	2 S	2.394	0.00	78 H
2.43%	2 S	2.394	0.00	74 H
2.40%	2 S	2.394	0.00	91 H
2.26%	1 S	-6.532	1.00	81 H
2.08%	2 S	2.394	0.00	63 H
-1.97%	2 P:x	4.578	0.00	13 C
1.97%	1 S	-6.532	1.00	90 H
1.90%	1 S	-6.532	1.00	87 H
-1.77%	2 P:y	4.578	0.00	30 C
-1.77%	2 P:x	4.578	0.00	11 C
1.62%	2 S	2.394	0.00	44 H
1.59%	1 S	-6.532	1.00	70 H
1.56%	1 S	-6.532	1.00	88 H
-1.56%	2 P:y	4.578	0.00	13 C
-1.56%	2 P:y	4.578	0.00	28 C
1.55%	2 S	2.394	0.00	57 H
-1.55%	2 P:x	4.578	0.00	29 C
1.54%	1 S	-6.532	1.00	54 H
1.54%	2 S	2.394	0.00	52 H
-1.49%	2 P:x	4.578	0.00	12 C
1.45%	2 S	2.394	0.00	48 H
1.44%	2 S	2.394	0.00	82 H

			1.41%	2 S	2.394	0.00	85 H
			-1.26%	2 P:x	4.578	0.00	28 C
			1.25%	2 S	2.394	0.00	49 H
			1.21%	2 S	2.394	0.00	55 H
			1.21%	1 S	-6.532	1.00	65 H
			-1.20%	2 S	11.741	0.00	26 C
			1.16%	1 S	-6.532	1.00	63 H
			1.16%	2 S	2.394	0.00	79 H
			1.14%	2 S	2.394	0.00	56 H
			-1.12%	2 P:y	4.578	0.00	11 C
			1.10%	1 S	-6.532	1.00	74 H
			-1.00%	2 S	2.394	0.00	53 H
-0.001	0.00	133 A	8.47%	2 S	2.394	0.00	65 H
			8.00%	2 S	2.394	0.00	90 H
			7.74%	2 S	2.394	0.00	63 H
			5.94%	2 S	2.394	0.00	67 H
			5.47%	2 S	2.394	0.00	44 H
			5.15%	1 S	-6.532	1.00	90 H
			4.94%	2 S	2.394	0.00	77 H
			4.87%	2 S	2.394	0.00	80 H
			4.62%	1 S	-6.532	1.00	60 H
			4.59%	1 S	-6.532	1.00	63 H
			4.16%	1 S	-6.532	1.00	65 H
			3.99%	1 S	-6.532	1.00	67 H
			3.96%	2 S	2.394	0.00	73 H
			3.65%	1 S	-6.532	1.00	44 H
			3.54%	2 S	2.394	0.00	60 H
			3.36%	1 S	-6.532	1.00	77 H
			2.62%	2 S	2.394	0.00	81 H
			2.62%	2 S	2.394	0.00	49 H
			2.54%	1 S	-6.532	1.00	80 H

			2.29%	1 S	-6.532	1.00	76 H
			-2.25%	2 P:y	4.578	0.00	32 C
			2.16%	1 S	-6.532	1.00	73 H
			-2.02%	2 P:z	4.578	0.00	33 C
			1.98%	2 S	2.394	0.00	96 H
			-1.84%	2 P:y	4.578	0.00	33 C
			1.72%	2 S	2.394	0.00	94 H
			1.63%	1 S	-6.532	1.00	51 H
			1.63%	1 S	-6.532	1.00	96 H
			1.63%	1 P:z	-5.263	0.67	22 C
			-1.50%	2 P:y	4.578	0.00	29 C
			1.49%	2 S	2.394	0.00	51 H
			1.45%	2 S	2.394	0.00	78 H
			-1.36%	2 S	11.741	0.00	40 C
			1.32%	1 S	-6.532	1.00	94 H
			-1.31%	2 P:y	4.578	0.00	37 C
			-1.29%	2 P:x	4.578	0.00	29 C
			1.29%	2 S	2.394	0.00	62 H
			-1.26%	2 P:x	4.578	0.00	40 C
			1.26%	1 P:z	-5.263	0.67	31 C
			-1.26%	2 P:x	4.578	0.00	37 C
			1.22%	1 S	-6.532	1.00	81 H
			-1.15%	2 P:x	4.578	0.00	17 C
			1.13%	2 S	2.394	0.00	84 H
			1.11%	1 S	-6.532	1.00	85 H
			-1.06%	2 P:z	4.578	0.00	21 C
			-1.05%	2 P:x	4.578	0.00	28 C
			-1.03%	2 P:x	4.578	0.00	13 C
			1.03%	2 S	2.394	0.00	74 H
0.006	0.00	134 A	8.55%	2 S	2.394	0.00	57 H
			7.05%	2 S	2.394	0.00	59 H

6.15%	2 S	2.394	0.00	61 H
5.92%	2 S	2.394	0.00	95 H
4.69%	2 S	2.394	0.00	74 H
3.98%	1 S	-6.532	1.00	61 H
3.96%	2 S	2.394	0.00	90 H
3.74%	1 S	-6.532	1.00	57 H
3.70%	2 S	2.394	0.00	78 H
3.34%	2 S	2.394	0.00	67 H
3.34%	2 S	2.394	0.00	50 H
3.19%	1 S	-6.532	1.00	59 H
2.73%	2 S	2.394	0.00	64 H
2.61%	1 S	-6.532	1.00	95 H
2.54%	1 S	-6.532	1.00	78 H
2.48%	2 S	2.394	0.00	92 H
2.44%	1 S	-6.532	1.00	74 H
2.34%	2 S	2.394	0.00	66 H
2.31%	2 S	2.394	0.00	60 H
2.29%	2 S	2.394	0.00	56 H
2.29%	2 S	2.394	0.00	58 H
2.12%	1 S	-6.532	1.00	90 H
2.06%	1 S	-6.532	1.00	60 H
-1.98%	2 S	11.741	0.00	39 C
1.90%	1 P:z	-5.263	0.67	31 C
1.73%	1 S	-6.532	1.00	64 H
1.71%	2 S	2.394	0.00	49 H
1.70%	1 S	-6.532	1.00	67 H
-1.61%	2 P:y	4.578	0.00	22 C
1.59%	2 S	2.394	0.00	55 H
1.54%	2 S	2.394	0.00	65 H
1.47%	2 S	2.394	0.00	76 H
-1.41%	2 P:z	4.578	0.00	33 C

			-1.36%	2 P:z	4.578	0.00	40 C
			1.23%	1 P:z	-5.263	0.67	22 C
			1.17%	1 S	-6.532	1.00	92 H
			1.13%	1 S	-6.532	1.00	50 H
			1.11%	1 S	-13.631	2.00	39 C
			-1.05%	2 P:z	4.578	0.00	28 C
			1.00%	2 S	2.394	0.00	72 H
0.146	0.00	135 A	5.95%	2 S	2.394	0.00	75 H
			4.86%	2 S	2.394	0.00	83 H
			4.74%	2 S	2.394	0.00	62 H
			4.47%	2 S	2.394	0.00	81 H
			4.16%	2 S	2.394	0.00	84 H
			3.95%	2 S	2.394	0.00	96 H
			3.90%	2 S	2.394	0.00	91 H
			3.86%	2 S	2.394	0.00	66 H
			3.82%	1 S	-6.532	1.00	62 H
			3.50%	2 S	2.394	0.00	72 H
			3.49%	2 S	2.394	0.00	55 H
			3.43%	2 S	2.394	0.00	88 H
			3.38%	2 S	2.394	0.00	94 H
			3.12%	1 S	-6.532	1.00	75 H
			3.04%	2 S	2.394	0.00	89 H
			3.02%	2 S	2.394	0.00	68 H
			2.98%	1 S	-6.532	1.00	88 H
			2.84%	1 S	-6.532	1.00	83 H
			2.71%	1 S	-6.532	1.00	81 H
			2.61%	2 S	2.394	0.00	78 H
			2.32%	1 S	-6.532	1.00	91 H
			2.23%	1 S	-6.532	1.00	94 H
			2.15%	2 S	2.394	0.00	92 H
			2.12%	1 S	-6.532	1.00	60 H

			1.97%	2 S	2.394	0.00	61 H		
			1.94%	1 S	-6.532	1.00	55 H		
			1.88%	2 S	2.394	0.00	46 H		
			1.84%	1 S	-6.532	1.00	84 H		
			1.80%	2 S	2.394	0.00	73 H		
			1.80%	1 S	-6.532	1.00	78 H		
			1.78%	1 S	-6.532	1.00	72 H		
			1.76%	1 S	-6.532	1.00	89 H		
			1.66%	1 S	-6.532	1.00	68 H		
			-1.63%	2 S	2.394	0.00	71 H		
			1.56%	2 S	2.394	0.00	47 H		
			1.55%	1 S	-6.532	1.00	96 H		
			1.50%	2 S	2.394	0.00	60 H		
			1.50%	1 S	-6.532	1.00	66 H		
			-1.37%	2 P:y	4.578	0.00	29 C		
			1.37%	1 S	-6.532	1.00	44 H		
			1.36%	2 S	2.394	0.00	44 H		
			1.33%	1 S	-6.532	1.00	51 H		
			-1.32%	2 P:y	4.578	0.00	37 C		
			-1.29%	2 P:z	4.578	0.00	17 C		
			1.29%	2 S	2.394	0.00	51 H		
			1.27%	2 S	2.394	0.00	86 H		
			-1.26%	2 P:z	4.578	0.00	13 C		
			-1.23%	2 P:y	4.578	0.00	22 C		
			-1.17%	2 P:z	4.578	0.00	12 C		
			1.12%	2 S	2.394	0.00	70 H		
			-1.10%	2 P:z	4.578	0.00	33 C		
			-1.10%	2 P:x	4.578	0.00	30 C		
			-1.03%	2 P:x	4.578	0.00	25 C		
			-1.00%	2 S	2.394	0.00	45 H		
0.325	0.00	136 A	7.82%	2 S	2.394	0.00	61 H		

7.80%	2 S	2.394	0.00	57 H
6.91%	2 S	2.394	0.00	65 H
6.05%	2 S	2.394	0.00	90 H
5.70%	1 S	-6.532	1.00	61 H
5.52%	2 S	2.394	0.00	44 H
4.60%	1 S	-6.532	1.00	65 H
4.18%	1 S	-6.532	1.00	44 H
4.08%	1 S	-6.532	1.00	57 H
3.86%	1 S	-6.532	1.00	90 H
3.68%	2 S	2.394	0.00	84 H
-2.95%	2 P:y	4.578	0.00	37 C
2.75%	2 S	2.394	0.00	72 H
2.69%	2 S	2.394	0.00	74 H
2.62%	2 S	2.394	0.00	55 H
2.62%	2 S	2.394	0.00	66 H
2.52%	2 S	2.394	0.00	81 H
-2.51%	2 P:y	4.578	0.00	40 C
2.42%	2 S	2.394	0.00	64 H
-2.31%	2 P:y	4.578	0.00	33 C
2.13%	2 S	2.394	0.00	83 H
1.97%	2 S	2.394	0.00	56 H
-1.96%	2 P:y	4.578	0.00	22 C
1.89%	1 S	-6.532	1.00	84 H
-1.73%	2 P:y	4.578	0.00	23 C
-1.67%	2 P:x	4.578	0.00	36 C
1.59%	1 S	-6.532	1.00	56 H
1.58%	1 P:x	-5.263	0.67	39 C
1.56%	1 S	-13.631	2.00	39 C
1.54%	1 S	-6.532	1.00	74 H
1.50%	1 S	-6.532	1.00	64 H
1.50%	1 S	-6.532	1.00	55 H

			1.46%	1 S	-6.532	1.00	81 H
			1.35%	2 S	2.394	0.00	51 H
			1.27%	2 S	2.394	0.00	59 H
			1.25%	1 S	-6.532	1.00	83 H
			-1.24%	2 S	11.741	0.00	39 C
			1.19%	1 S	-6.532	1.00	66 H
			1.19%	2 S	2.394	0.00	73 H
			1.09%	1 S	-6.532	1.00	72 H
			-1.09%	2 P:y	4.578	0.00	21 C
			1.08%	2 S	2.394	0.00	96 H
			1.07%	2 P:x	4.578	0.00	39 C
			1.01%	2 S	2.394	0.00	58 H
			-1.00%	2 P:z	4.578	0.00	40 C
0.489	0.00	137 A	7.96%	2 S	2.394	0.00	48 H
			7.04%	2 S	2.394	0.00	49 H
			5.65%	1 S	-6.532	1.00	60 H
			5.42%	2 S	2.394	0.00	50 H
			4.51%	2 S	2.394	0.00	65 H
			4.51%	2 S	2.394	0.00	75 H
			4.33%	1 S	-6.532	1.00	48 H
			3.87%	2 S	2.394	0.00	56 H
			3.48%	2 S	2.394	0.00	52 H
			3.29%	2 S	2.394	0.00	62 H
			3.01%	1 S	-6.532	1.00	62 H
			2.94%	1 S	-6.532	1.00	65 H
			2.90%	2 S	2.394	0.00	60 H
			2.87%	1 S	-6.532	1.00	75 H
			2.87%	1 S	-6.532	1.00	49 H
			2.57%	2 S	2.394	0.00	95 H
			-2.44%	2 P:z	4.578	0.00	33 C
			2.36%	1 S	-6.532	1.00	52 H

2.33%	1 S	-6.532	1.00	50 H
2.11%	1 P:y	-5.263	0.67	39 C
2.07%	1 S	-6.532	1.00	88 H
-1.91%	2 S	11.741	0.00	15 C
-1.90%	2 P:y	4.578	0.00	40 C
1.90%	1 S	-6.532	1.00	95 H
1.89%	1 S	-6.532	1.00	54 H
1.79%	2 S	2.394	0.00	88 H
1.78%	2 S	2.394	0.00	96 H
1.69%	2 S	2.394	0.00	92 H
1.57%	2 S	2.394	0.00	54 H
1.47%	1 S	-6.532	1.00	56 H
-1.47%	2 P:y	4.578	0.00	37 C
1.46%	2 S	2.394	0.00	91 H
-1.38%	2 P:x	4.578	0.00	32 C
1.36%	2 S	2.394	0.00	82 H
-1.33%	2 P:y	4.578	0.00	15 C
-1.31%	2 P:z	4.578	0.00	40 C
-1.31%	2 P:y	4.578	0.00	29 C
1.25%	1 P:x	-5.263	0.67	39 C
1.21%	2 S	2.394	0.00	74 H
1.21%	2 S	2.394	0.00	58 H
-1.19%	2 P:x	4.578	0.00	36 C
-1.14%	2 P:z	4.578	0.00	36 C
1.13%	2 S	2.394	0.00	61 H
1.11%	2 S	2.394	0.00	64 H
1.11%	2 S	2.394	0.00	57 H
1.08%	1 P:x	-5.263	0.67	19 C
-1.05%	2 P:z	4.578	0.00	19 C
1.00%	1 S	-6.532	1.00	64 H
1.00%	1 S	-6.532	1.00	57 H

0.567	0.00	138 A	7.61%	2 S	2.394	0.00	70 H
			6.99%	2 S	2.394	0.00	80 H
			6.21%	2 S	2.394	0.00	90 H
			5.92%	2 S	2.394	0.00	74 H
			5.90%	2 S	2.394	0.00	65 H
			5.81%	2 S	2.394	0.00	92 H
			5.81%	2 S	2.394	0.00	67 H
			5.78%	2 S	2.394	0.00	81 H
			5.72%	1 S	-6.532	1.00	90 H
			4.84%	1 S	-6.532	1.00	65 H
			4.26%	1 S	-6.532	1.00	70 H
			3.94%	1 S	-6.532	1.00	67 H
			3.73%	1 S	-6.532	1.00	92 H
			3.71%	1 S	-6.532	1.00	74 H
			3.58%	1 S	-6.532	1.00	80 H
			3.23%	1 S	-6.532	1.00	81 H
			-2.23%	2 P:y	4.578	0.00	40 C
			2.10%	2 S	2.394	0.00	77 H
			1.90%	2 S	2.394	0.00	91 H
			1.77%	2 S	2.394	0.00	72 H
			1.71%	1 S	-6.532	1.00	77 H
			-1.68%	2 P:y	4.578	0.00	26 C
			1.59%	2 S	2.394	0.00	82 H
			-1.45%	2 P:x	4.578	0.00	38 C
			1.43%	2 S	2.394	0.00	83 H
			1.41%	1 S	-6.532	1.00	85 H
			1.40%	1 S	-6.532	1.00	72 H
			1.37%	1 S	-6.532	1.00	82 H
			1.35%	2 S	2.394	0.00	85 H
			1.32%	1 S	-6.532	1.00	69 H
			-1.32%	2 P:z	4.578	0.00	17 C

			1.29%	2 S	2.394	0.00	75 H
			1.27%	2 S	2.394	0.00	68 H
			-1.26%	2 P:z	4.578	0.00	32 C
			-1.21%	2 P:z	4.578	0.00	24 C
			1.20%	1 S	-6.532	1.00	94 H
			-1.20%	2 S	11.741	0.00	26 C
			-1.18%	2 P:z	4.578	0.00	21 C
			-1.16%	2 P:z	4.578	0.00	30 C
			1.12%	1 S	-6.532	1.00	68 H
			1.09%	1 S	-6.532	1.00	60 H
			1.09%	2 S	2.394	0.00	95 H
			-1.07%	2 P:y	4.578	0.00	41 C
			-1.03%	2 P:x	4.578	0.00	24 C
			-1.01%	2 P:x	4.578	0.00	17 C
0.638	0.00	139 A	10.59%	2 S	2.394	0.00	78 H
			7.72%	1 S	-6.532	1.00	78 H
			5.83%	2 S	2.394	0.00	68 H
			-5.67%	2 P:z	4.578	0.00	28 C
			5.44%	2 S	2.394	0.00	74 H
			4.66%	2 S	2.394	0.00	59 H
			4.64%	2 S	2.394	0.00	95 H
			4.47%	2 S	2.394	0.00	70 H
			4.45%	2 S	2.394	0.00	86 H
			-4.12%	2 P:z	4.578	0.00	30 C
			3.98%	1 S	-6.532	1.00	74 H
			3.94%	1 S	-6.532	1.00	68 H
			3.35%	1 S	-6.532	1.00	70 H
			3.07%	1 S	-6.532	1.00	86 H
			3.00%	1 S	-6.532	1.00	59 H
			2.80%	2 S	2.394	0.00	85 H
			2.75%	2 S	2.394	0.00	75 H

2.70%	2 S	2.394	0.00	54 H
2.62%	2 S	2.394	0.00	90 H
2.52%	2 S	2.394	0.00	64 H
2.39%	1 S	-6.532	1.00	95 H
2.23%	2 S	2.394	0.00	55 H
2.21%	1 P:z	-5.263	0.67	33 C
2.10%	2 S	2.394	0.00	47 H
1.98%	1 S	-6.532	1.00	60 H
1.94%	2 S	2.394	0.00	52 H
1.93%	1 S	-6.532	1.00	85 H
1.92%	2 S	2.394	0.00	66 H
-1.83%	2 P:z	4.578	0.00	39 C
1.81%	1 S	-6.532	1.00	52 H
1.70%	1 S	-6.532	1.00	75 H
1.70%	1 S	-6.532	1.00	62 H
1.65%	1 S	-6.532	1.00	64 H
-1.60%	2 P:x	4.578	0.00	30 C
1.60%	1 S	-6.532	1.00	54 H
1.59%	1 S	-6.532	1.00	90 H
-1.58%	2 P:z	4.578	0.00	33 C
1.48%	2 S	2.394	0.00	67 H
1.36%	2 S	2.394	0.00	87 H
-1.35%	2 P:z	4.578	0.00	20 C
-1.33%	2 P:z	4.578	0.00	13 C
1.30%	2 S	2.394	0.00	48 H
1.24%	1 S	-6.532	1.00	55 H
1.19%	2 S	2.394	0.00	92 H
1.15%	1 S	-6.532	1.00	88 H
-1.10%	2 P:z	4.578	0.00	36 C
-1.10%	2 P:z	4.578	0.00	18 C
-1.07%	2 P:x	4.578	0.00	23 C

			-1.05%	2 S	2.394	0.00	94 H
0.653	0.00	140 A	8.63%	1 S	-6.532	1.00	61 H
			8.56%	1 S	-6.532	1.00	64 H
			8.56%	2 S	2.394	0.00	64 H
			6.36%	1 S	-6.532	1.00	66 H
			5.49%	2 S	2.394	0.00	56 H
			-4.82%	2 P:y	4.578	0.00	21 C
			4.66%	2 S	2.394	0.00	61 H
			4.60%	2 S	2.394	0.00	66 H
			3.88%	1 S	-6.532	1.00	56 H
			3.80%	2 S	2.394	0.00	59 H
			3.64%	2 S	2.394	0.00	48 H
			-3.36%	2 P:y	4.578	0.00	40 C
			-3.01%	2 P:y	4.578	0.00	39 C
			2.97%	2 S	2.394	0.00	95 H
			2.96%	1 S	-6.532	1.00	59 H
			2.92%	2 S	2.394	0.00	94 H
			2.90%	1 S	-6.532	1.00	60 H
			-2.79%	2 P:y	4.578	0.00	32 C
			2.72%	1 S	-6.532	1.00	94 H
			2.64%	1 P:y	-5.263	0.67	33 C
			-2.49%	2 P:x	4.578	0.00	37 C
			-2.40%	2 P:z	4.578	0.00	40 C
			2.37%	1 S	-6.532	1.00	51 H
			2.22%	2 S	2.394	0.00	60 H
			2.11%	1 S	-6.532	1.00	95 H
			2.09%	1 S	-13.631	2.00	33 C
			-1.99%	2 P:x	4.578	0.00	40 C
			1.85%	1 S	-6.532	1.00	48 H
			1.64%	1 P:x	-5.263	0.67	33 C
			1.55%	2 S	2.394	0.00	63 H

			1.51%	1 P:y	-5.263	0.67	21 C
			-1.50%	2 P:x	4.578	0.00	39 C
			1.50%	2 S	2.394	0.00	62 H
			1.49%	2 S	2.394	0.00	58 H
			1.36%	2 S	2.394	0.00	51 H
			-1.34%	2 P:x	4.578	0.00	33 C
			1.30%	1 S	-6.532	1.00	57 H
			1.26%	1 S	-6.532	1.00	58 H
			1.26%	2 S	2.394	0.00	67 H
			1.25%	1 S	-6.532	1.00	86 H
			1.23%	2 S	2.394	0.00	86 H
			1.16%	1 P:x	-5.263	0.67	39 C
			1.13%	2 S	2.394	0.00	83 H
			-1.08%	2 P:y	4.578	0.00	33 C
			1.05%	1 S	-6.532	1.00	63 H
			1.04%	1 S	-6.532	1.00	67 H
			1.04%	2 S	2.394	0.00	81 H
			1.02%	1 S	-23.822	2.00	5 O
			-1.01%	2 P:y	4.578	0.00	25 C
0.731	0.00	141 A	11.78%	2 S	2.394	0.00	47 H
			7.99%	2 S	2.394	0.00	68 H
			7.84%	1 S	-6.532	1.00	68 H
			7.01%	2 S	2.394	0.00	72 H
			5.20%	1 S	-6.532	1.00	72 H
			5.17%	1 S	-6.532	1.00	47 H
			4.27%	1 P:z	-5.263	0.67	33 C
			3.63%	2 S	2.394	0.00	91 H
			-3.59%	2 P:x	4.578	0.00	21 C
			3.55%	1 S	-6.532	1.00	60 H
			3.42%	2 S	2.394	0.00	96 H
			3.08%	1 S	-6.532	1.00	76 H

-2.67%	2 P:z	4.578	0.00	42 C
2.65%	1 S	-6.532	1.00	96 H
-2.38%	2 P:x	4.578	0.00	31 C
2.38%	2 S	2.394	0.00	73 H
2.27%	1 S	-6.532	1.00	61 H
2.18%	1 S	-6.532	1.00	91 H
-2.07%	2 P:y	4.578	0.00	20 C
1.93%	1 S	-6.532	1.00	73 H
1.87%	1 S	-6.532	1.00	86 H
1.82%	2 S	2.394	0.00	86 H
1.74%	2 S	2.394	0.00	79 H
1.63%	1 S	-6.532	1.00	52 H
1.52%	2 S	2.394	0.00	92 H
-1.48%	2 P:x	4.578	0.00	34 C
-1.45%	2 P:z	4.578	0.00	33 C
1.37%	2 S	2.394	0.00	76 H
1.35%	2 S	2.394	0.00	81 H
-1.34%	2 P:x	4.578	0.00	22 C
-1.34%	2 P:x	4.578	0.00	30 C
1.29%	1 P:x	-5.263	0.67	39 C
1.27%	2 S	2.394	0.00	52 H
-1.22%	2 P:x	4.578	0.00	23 C
1.19%	2 S	2.394	0.00	46 H
1.17%	1 P:z	-5.263	0.67	18 C
1.14%	2 S	2.394	0.00	64 H
-1.11%	2 P:y	4.578	0.00	39 C
1.10%	1 S	-6.532	1.00	56 H
1.10%	2 S	2.394	0.00	83 H
-1.09%	2 P:x	4.578	0.00	38 C
1.08%	1 S	-6.532	1.00	57 H
1.05%	1 S	-6.532	1.00	92 H

			-1.00%	2	P:x	4.578	0.00	29	C
0.807	0.00	142 A	15.67%	2	S	2.394	0.00	84	H
			11.73%	1	S	-6.532	1.00	84	H
			4.85%	2	S	2.394	0.00	81	H
			-4.81%	2	P:x	4.578	0.00	38	C
			4.79%	2	S	2.394	0.00	80	H
			4.63%	1	S	-6.532	1.00	90	H
			4.59%	2	S	2.394	0.00	75	H
			4.47%	1	S	-6.532	1.00	80	H
			-4.28%	2	P:y	4.578	0.00	26	C
			4.06%	2	S	2.394	0.00	77	H
			3.99%	1	S	-6.532	1.00	77	H
			3.84%	2	S	2.394	0.00	90	H
			3.72%	2	S	2.394	0.00	59	H
			-3.50%	2	P:x	4.578	0.00	26	C
			3.48%	1	S	-6.532	1.00	81	H
			3.20%	1	S	-6.532	1.00	59	H
			-2.92%	2	P:x	4.578	0.00	30	C
			2.78%	1	S	-6.532	1.00	75	H
			2.48%	2	S	2.394	0.00	92	H
			2.30%	2	S	2.394	0.00	88	H
			-2.13%	2	P:x	4.578	0.00	13	C
			2.11%	2	S	2.394	0.00	55	H
			2.10%	2	S	2.394	0.00	70	H
			2.03%	1	S	-6.532	1.00	70	H
			1.98%	2	S	2.394	0.00	64	H
			1.96%	1	S	-6.532	1.00	55	H
			1.91%	1	P:x	-5.263	0.67	26	C
			-1.79%	2	S	2.394	0.00	93	H
			1.63%	2	S	2.394	0.00	87	H
			1.54%	2	S	2.394	0.00	95	H

			-1.48%	2 P:z	4.578	0.00	39 C
			1.47%	1 S	-6.532	1.00	92 H
			1.39%	2 S	2.394	0.00	49 H
			1.39%	2 S	2.394	0.00	44 H
			-1.37%	2 P:z	4.578	0.00	40 C
			1.35%	1 S	-6.532	1.00	64 H
			1.32%	2 S	2.394	0.00	91 H
			1.29%	2 S	2.394	0.00	96 H
			1.15%	1 S	-6.532	1.00	49 H
			1.12%	1 S	-6.532	1.00	44 H
			1.09%	1 S	-6.532	1.00	88 H
0.888	0.00	143 A	13.24%	2 S	2.394	0.00	83 H
			10.21%	1 S	-6.532	1.00	83 H
			7.24%	2 S	2.394	0.00	81 H
			5.00%	2 S	2.394	0.00	49 H
			4.51%	1 S	-6.532	1.00	81 H
			4.32%	2 S	2.394	0.00	54 H
			4.06%	1 S	-6.532	1.00	54 H
			-4.00%	2 P:x	4.578	0.00	38 C
			3.24%	2 S	2.394	0.00	65 H
			2.93%	1 S	-6.532	1.00	49 H
			2.80%	2 S	2.394	0.00	66 H
			2.78%	2 S	2.394	0.00	55 H
			-2.51%	2 P:x	4.578	0.00	26 C
			2.49%	1 S	-6.532	1.00	88 H
			-2.47%	2 P:z	4.578	0.00	38 C
			2.47%	2 S	2.394	0.00	88 H
			2.41%	1 S	-6.532	1.00	65 H
			2.25%	1 S	-6.532	1.00	52 H
			2.24%	1 S	-6.532	1.00	66 H
			2.22%	2 S	2.394	0.00	91 H

			-2.21%	2 P:y	4.578	0.00	13 C
			2.07%	2 S	2.394	0.00	95 H
			-2.04%	2 P:z	4.578	0.00	36 C
			1.98%	2 S	2.394	0.00	79 H
			1.98%	2 S	2.394	0.00	74 H
			1.98%	1 S	-6.532	1.00	91 H
			1.95%	1 S	-6.532	1.00	79 H
			1.92%	2 S	2.394	0.00	73 H
			1.88%	1 S	-6.532	1.00	55 H
			1.86%	1 S	-6.532	1.00	74 H
			-1.76%	2 P:z	4.578	0.00	42 C
			1.71%	1 S	-6.532	1.00	73 H
			1.65%	1 S	-6.532	1.00	76 H
			-1.61%	2 P:z	4.578	0.00	30 C
			1.58%	2 S	2.394	0.00	90 H
			1.55%	2 S	2.394	0.00	58 H
			1.32%	2 S	2.394	0.00	50 H
			-1.29%	2 P:z	4.578	0.00	15 C
			1.25%	1 P:x	-5.263	0.67	18 C
			1.24%	1 S	-6.532	1.00	95 H
			1.19%	1 S	-6.532	1.00	90 H
			-1.15%	2 P:z	4.578	0.00	37 C
			1.13%	2 S	2.394	0.00	78 H
			1.12%	1 S	-6.532	1.00	58 H
			1.10%	1 P:x	-5.263	0.67	19 C
			-1.10%	2 P:x	4.578	0.00	13 C
			-1.09%	2 P:y	4.578	0.00	40 C
0.987	0.00	144 A	12.61%	1 S	-6.532	1.00	62 H
			-8.83%	2 P:z	4.578	0.00	37 C
			7.49%	2 S	2.394	0.00	62 H
			5.66%	1 S	-6.532	1.00	61 H

4.92%	1 P:z	-5.263	0.67	33	C
-3.24%	2 S	2.394	0.00	61	H
3.13%	2 S	2.394	0.00	48	H
2.98%	1 P:x	-5.263	0.67	33	C
2.95%	1 S	-6.532	1.00	91	H
2.82%	2 S	2.394	0.00	91	H
2.81%	1 P:y	-5.263	0.67	33	C
2.51%	1 P:z	-5.263	0.67	37	C
2.49%	2 S	2.394	0.00	56	H
2.31%	2 S	2.394	0.00	65	H
2.19%	2 P:z	4.578	0.00	40	C
2.12%	1 S	-6.532	1.00	56	H
2.06%	2 S	2.394	0.00	52	H
2.03%	1 S	-6.532	1.00	51	H
2.01%	1 S	-6.532	1.00	66	H
1.98%	2 S	2.394	0.00	45	H
1.88%	1 P:y	-9.066	1.33	5	O
1.87%	2 S	2.394	0.00	47	H
1.79%	1 S	-6.532	1.00	90	H
-1.77%	2 P:z	4.578	0.00	42	C
1.64%	2 S	2.394	0.00	72	H
-1.54%	2 P:y	4.578	0.00	21	C
1.54%	1 S	-23.822	2.00	5	O
-1.47%	2 P:z	4.578	0.00	30	C
1.42%	1 S	-13.631	2.00	33	C
1.41%	1 S	-6.532	1.00	52	H
1.37%	1 S	-6.532	1.00	94	H
1.36%	1 P:y	-5.263	0.67	21	C
1.36%	1 S	-6.532	1.00	48	H
1.35%	2 S	2.394	0.00	59	H
1.34%	1 P:x	-9.066	1.33	5	O

			1.31%	1 S	-6.532	1.00	74 H
			1.26%	2 S	2.394	0.00	89 H
			1.25%	2 S	2.394	0.00	94 H
			1.25%	2 S	2.394	0.00	79 H
			1.22%	1 S	-6.532	1.00	59 H
			1.13%	2 S	2.394	0.00	51 H
			-1.13%	2 P:x	4.578	0.00	31 C
			-1.11%	2 P:x	4.578	0.00	25 C
			1.08%	1 S	-6.532	1.00	79 H
			1.01%	1 S	-6.532	1.00	95 H
			1.00%	1 S	-6.532	1.00	78 H
1.039	0.00	145 A	5.79%	1 S	-6.532	1.00	55 H
			4.65%	2 S	2.394	0.00	55 H
			4.15%	1 S	-6.532	1.00	69 H
			3.84%	1 S	-6.532	1.00	88 H
			3.23%	1 S	-6.532	1.00	89 H
			3.22%	2 S	2.394	0.00	58 H
			2.93%	2 S	2.394	0.00	73 H
			2.88%	2 S	2.394	0.00	84 H
			2.83%	1 S	-6.532	1.00	73 H
			2.77%	2 S	2.394	0.00	74 H
			2.75%	2 S	2.394	0.00	49 H
			2.49%	1 S	-6.532	1.00	58 H
			2.48%	2 S	2.394	0.00	89 H
			2.26%	2 S	2.394	0.00	93 H
			2.22%	1 S	-6.532	1.00	68 H
			2.16%	2 S	2.394	0.00	82 H
			2.11%	1 S	-6.532	1.00	84 H
			-2.06%	2 P:x	4.578	0.00	36 C
			2.02%	1 P:z	-5.263	0.67	33 C
			1.97%	1 S	-6.532	1.00	53 H

			1.94%	2 S	2.394	0.00	70 H
			1.93%	1 S	-6.532	1.00	93 H
			1.83%	2 S	2.394	0.00	69 H
			-1.63%	2 P:z	4.578	0.00	37 C
			1.61%	1 S	-6.532	1.00	74 H
			-1.56%	2 P:z	4.578	0.00	24 C
			1.54%	1 P:x	-5.263	0.67	18 C
			-1.50%	2 P:z	4.578	0.00	39 C
			-1.44%	2 P:z	4.578	0.00	42 C
			1.43%	1 S	-6.532	1.00	70 H
			1.36%	2 S	2.394	0.00	78 H
			1.36%	1 S	-6.532	1.00	60 H
			1.36%	1 S	-6.532	1.00	85 H
			1.35%	2 S	2.394	0.00	88 H
			1.33%	1 S	-6.532	1.00	78 H
			1.33%	2 S	2.394	0.00	66 H
			1.33%	1 S	-6.532	1.00	82 H
			1.32%	2 P:y	4.578	0.00	38 C
			1.14%	1 P:z	-5.263	0.67	11 C
			1.12%	2 S	2.394	0.00	68 H
			1.11%	2 S	2.394	0.00	50 H
			1.11%	1 S	-6.532	1.00	49 H
			1.10%	1 P:z	-5.263	0.67	18 C
			-1.08%	2 P:x	4.578	0.00	38 C
			1.04%	2 P:z	4.578	0.00	21 C
			1.03%	2 S	2.394	0.00	62 H
			1.02%	2 S	2.394	0.00	80 H
1.052	0.00	146 A	6.97%	1 S	-6.532	1.00	75 H
			6.74%	1 S	-6.532	1.00	78 H
			6.52%	1 S	-6.532	1.00	88 H
			6.51%	2 S	2.394	0.00	75 H

4.26%	1 P:y	-5.263	0.67	12 C
3.49%	2 S	2.394	0.00	73 H
3.43%	2 S	2.394	0.00	88 H
3.39%	1 P:z	-5.263	0.67	29 C
3.24%	1 S	-6.532	1.00	73 H
-2.92%	2 S	2.394	0.00	74 H
2.76%	1 P:x	-5.263	0.67	13 C
2.68%	2 S	2.394	0.00	47 H
-2.27%	2 P:y	4.578	0.00	13 C
-2.20%	2 P:z	4.578	0.00	30 C
2.12%	1 S	-6.532	1.00	92 H
2.11%	1 S	-6.532	1.00	84 H
2.07%	1 S	-6.532	1.00	83 H
1.90%	1 S	-6.532	1.00	44 H
1.89%	2 S	2.394	0.00	92 H
1.82%	1 P:y	-5.263	0.67	11 C
-1.81%	2 P:z	4.578	0.00	28 C
-1.80%	2 P:x	4.578	0.00	38 C
1.78%	1 S	-6.532	1.00	52 H
1.76%	1 P:z	-5.263	0.67	28 C
1.74%	1 S	-6.532	1.00	80 H
1.70%	2 S	2.394	0.00	80 H
1.65%	1 S	-6.532	1.00	74 H
1.64%	1 P:z	-5.263	0.67	30 C
1.60%	2 S	2.394	0.00	84 H
1.40%	1 S	-6.532	1.00	54 H
1.31%	2 S	2.394	0.00	44 H
1.29%	1 P:y	-5.263	0.67	38 C
1.26%	1 S	-6.532	1.00	47 H
1.24%	2 S	2.394	0.00	49 H
1.22%	1 S	-6.532	1.00	93 H

			1.06%	1 S	-6.532	1.00	51 H
			1.06%	1 P:y	-9.066	1.33	7 O
			1.05%	2 S	2.394	0.00	68 H
			1.04%	1 S	-6.532	1.00	65 H
1.204	0.00	147 A	7.55%	1 S	-6.532	1.00	81 H
			5.87%	2 S	2.394	0.00	81 H
			5.55%	2 S	2.394	0.00	47 H
			5.13%	1 S	-6.532	1.00	86 H
			4.78%	1 S	-6.532	1.00	65 H
			4.53%	2 S	2.394	0.00	65 H
			4.41%	1 S	-6.532	1.00	49 H
			4.39%	2 S	2.394	0.00	49 H
			4.32%	2 S	2.394	0.00	83 H
			4.00%	1 S	-6.532	1.00	83 H
			-3.61%	2 P:z	4.578	0.00	15 C
			3.54%	1 S	-6.532	1.00	47 H
			3.30%	1 S	-6.532	1.00	78 H
			-3.16%	2 P:x	4.578	0.00	26 C
			-3.00%	2 P:x	4.578	0.00	29 C
			2.88%	1 P:x	-5.263	0.67	18 C
			-2.58%	2 P:x	4.578	0.00	38 C
			-2.50%	2 P:y	4.578	0.00	40 C
			2.41%	2 S	2.394	0.00	66 H
			2.36%	1 S	-6.532	1.00	77 H
			-2.36%	2 P:x	4.578	0.00	28 C
			2.33%	1 S	-6.532	1.00	96 H
			2.26%	1 S	-6.532	1.00	76 H
			2.19%	2 S	2.394	0.00	44 H
			2.15%	2 S	2.394	0.00	96 H
			2.09%	2 S	2.394	0.00	75 H
			2.02%	1 S	-6.532	1.00	44 H

			2.00%	2 S	2.394	0.00	86 H		
			1.87%	2 S	2.394	0.00	77 H		
			1.79%	1 S	-6.532	1.00	66 H		
			1.74%	2 S	2.394	0.00	56 H		
			1.74%	2 S	2.394	0.00	58 H		
			1.69%	1 S	-6.532	1.00	50 H		
			1.53%	2 S	2.394	0.00	78 H		
			1.51%	1 S	-6.532	1.00	75 H		
			1.40%	1 P:x	-5.263	0.67	39 C		
			1.29%	1 P:y	-5.263	0.67	39 C		
			1.29%	2 S	2.394	0.00	76 H		
			-1.29%	2 P:y	4.578	0.00	22 C		
			1.16%	1 S	-6.532	1.00	57 H		
			-1.11%	2 P:x	4.578	0.00	18 C		
			-1.10%	2 P:x	4.578	0.00	15 C		
			1.09%	1 S	-6.532	1.00	58 H		
			1.06%	1 S	-6.532	1.00	80 H		
			-1.01%	2 P:z	4.578	0.00	18 C		
			1.01%	2 S	2.394	0.00	74 H		
1.285	0.00	148 A	-6.27%	2 P:x	4.578	0.00	15 C		
			6.26%	2 S	2.394	0.00	48 H		
			6.19%	1 S	-6.532	1.00	91 H		
			5.87%	2 S	2.394	0.00	91 H		
			5.48%	1 S	-6.532	1.00	57 H		
			4.91%	2 S	2.394	0.00	52 H		
			4.71%	1 S	-6.532	1.00	50 H		
			4.41%	2 S	2.394	0.00	57 H		
			-4.34%	2 P:x	4.578	0.00	42 C		
			4.12%	2 S	2.394	0.00	95 H		
			4.05%	1 S	-6.532	1.00	48 H		
			3.73%	1 S	-6.532	1.00	69 H		

			-3.52%	2 P:y	4.578	0.00	39 C
			3.29%	1 S	-6.532	1.00	95 H
			2.80%	1 P:y	-5.263	0.67	39 C
			2.74%	2 S	2.394	0.00	46 H
			2.67%	1 S	-6.532	1.00	52 H
			2.61%	2 P:x	4.578	0.00	34 C
			2.34%	1 S	-6.532	1.00	49 H
			2.15%	1 S	-6.532	1.00	46 H
			2.02%	1 P:x	-5.263	0.67	18 C
			1.99%	2 S	2.394	0.00	69 H
			1.85%	1 S	-6.532	1.00	93 H
			1.78%	2 S	2.394	0.00	87 H
			1.75%	2 S	2.394	0.00	54 H
			1.71%	1 S	-6.532	1.00	62 H
			-1.71%	2 P:y	4.578	0.00	34 C
			1.68%	2 S	2.394	0.00	49 H
			1.49%	2 S	2.394	0.00	93 H
			1.49%	1 S	-6.532	1.00	77 H
			1.40%	1 S	-6.532	1.00	90 H
			1.39%	2 S	2.394	0.00	62 H
			1.35%	1 S	-6.532	1.00	59 H
			-1.31%	2 P:z	4.578	0.00	24 C
			-1.27%	2 P:x	4.578	0.00	24 C
			-1.17%	2 P:x	4.578	0.00	19 C
			1.13%	1 P:x	-5.263	0.67	15 C
			1.03%	2 S	2.394	0.00	68 H
1.346	0.00	149 A	6.08%	1 S	-6.532	1.00	76 H
			5.52%	1 S	-6.532	1.00	64 H
			5.16%	2 S	2.394	0.00	78 H
			-4.80%	2 P:z	4.578	0.00	40 C
			4.23%	2 S	2.394	0.00	70 H

3.85%	1 S	-6.532	1.00	58	H
3.40%	2 S	2.394	0.00	58	H
3.23%	1 P:x	-5.263	0.67	39	C
3.12%	2 S	2.394	0.00	72	H
-3.07%	2 P:z	4.578	0.00	28	C
2.96%	1 S	-6.532	1.00	78	H
2.91%	2 P:y	4.578	0.00	38	C
2.79%	1 S	-6.532	1.00	63	H
2.76%	1 P:y	-5.263	0.67	38	C
2.57%	2 S	2.394	0.00	64	H
2.52%	1 S	-6.532	1.00	70	H
2.47%	1 P:y	-5.263	0.67	12	C
2.40%	1 S	-6.532	1.00	96	H
2.34%	1 S	-6.532	1.00	67	H
2.30%	2 S	2.394	0.00	76	H
2.17%	1 S	-6.532	1.00	71	H
2.01%	2 S	2.394	0.00	96	H
1.81%	2 S	2.394	0.00	63	H
1.78%	2 S	2.394	0.00	74	H
1.68%	1 S	-6.532	1.00	74	H
-1.67%	2 P:x	4.578	0.00	29	C
1.63%	2 S	2.394	0.00	86	H
1.60%	1 S	-13.631	2.00	29	C
-1.60%	2 S	11.741	0.00	29	C
-1.59%	2 S	16.116	0.00	43	N
-1.59%	2 P:z	4.578	0.00	30	C
1.58%	1 S	-6.532	1.00	86	H
1.52%	2 S	2.394	0.00	71	H
1.50%	1 S	-18.438	2.00	43	N
1.34%	2 S	2.394	0.00	67	H
-1.33%	2 P:z	4.578	0.00	39	C

			1.29%	1 S	-6.532	1.00	52 H		
			1.22%	1 P:z	-5.263	0.67	40 C		
			-1.21%	2 S	2.394	0.00	69 H		
			1.19%	2 P:z	4.578	0.00	26 C		
			-1.18%	2 P:y	4.578	0.00	32 C		
			1.11%	1 S	-6.532	1.00	72 H		
			1.06%	1 S	-6.532	1.00	91 H		
			1.06%	1 P:x	-5.263	0.67	33 C		
1.357	0.00	150 A	5.33%	2 S	2.394	0.00	46 H		
			4.34%	1 S	-6.532	1.00	84 H		
			3.93%	1 S	-6.532	1.00	46 H		
			-3.28%	2 P:z	4.578	0.00	38 C		
			3.25%	2 S	2.394	0.00	54 H		
			2.96%	2 S	2.394	0.00	84 H		
			2.83%	2 S	2.394	0.00	82 H		
			2.75%	1 S	-6.532	1.00	76 H		
			2.71%	1 S	-6.532	1.00	91 H		
			2.63%	2 S	2.394	0.00	49 H		
			2.58%	1 S	-6.532	1.00	69 H		
			2.56%	1 S	-6.532	1.00	92 H		
			2.51%	1 S	-6.532	1.00	90 H		
			2.46%	2 S	2.394	0.00	89 H		
			2.41%	2 S	2.394	0.00	62 H		
			2.41%	2 S	2.394	0.00	79 H		
			-2.39%	2 P:z	4.578	0.00	37 C		
			2.38%	2 P:y	4.578	0.00	42 C		
			2.27%	1 S	-6.532	1.00	82 H		
			2.19%	1 S	-6.532	1.00	49 H		
			-2.19%	2 P:y	4.578	0.00	34 C		
			1.94%	1 S	-6.532	1.00	54 H		
			1.85%	1 P:x	-5.263	0.67	42 C		

			1.84%	1 P:z	-5.263	0.67	33 C
			1.83%	1 S	-6.532	1.00	89 H
			1.73%	1 S	-6.532	1.00	53 H
			1.70%	2 S	2.394	0.00	56 H
			1.65%	2 S	2.394	0.00	96 H
			1.62%	2 S	2.394	0.00	90 H
			-1.60%	2 P:y	4.578	0.00	27 C
			1.58%	1 S	-6.532	1.00	78 H
			1.56%	1 S	-6.532	1.00	80 H
			1.55%	2 S	2.394	0.00	91 H
			1.49%	1 S	-6.532	1.00	64 H
			1.39%	1 S	-6.532	1.00	62 H
			1.38%	2 S	2.394	0.00	47 H
			1.38%	1 S	-6.532	1.00	56 H
			1.34%	2 S	2.394	0.00	73 H
			1.27%	1 S	-6.532	1.00	79 H
			1.24%	1 S	-6.532	1.00	61 H
			-1.24%	2 S	2.394	0.00	45 H
			-1.21%	2 P:z	4.578	0.00	15 C
			1.21%	1 S	-6.532	1.00	96 H
			1.14%	1 P:x	-5.263	0.67	33 C
			1.13%	2 P:x	4.578	0.00	34 C
			1.11%	1 P:y	-5.263	0.67	42 C
			1.11%	2 P:y	4.578	0.00	38 C
			1.10%	1 P:x	-5.263	0.67	17 C
			1.07%	1 P:x	-5.263	0.67	18 C
			1.05%	2 S	2.394	0.00	80 H
			-1.04%	2 P:z	4.578	0.00	42 C
1.383	0.00	151 A	8.42%	1 S	-6.532	1.00	69 H
			4.86%	2 S	2.394	0.00	91 H
			4.57%	2 S	2.394	0.00	69 H

4.43%	1 S	-6.532	1.00	53 H
-4.40%	2 P:x	4.578	0.00	42 C
4.37%	1 S	-6.532	1.00	62 H
4.04%	1 S	-6.532	1.00	91 H
3.95%	2 S	2.394	0.00	70 H
3.74%	1 S	-6.532	1.00	47 H
3.62%	2 S	2.394	0.00	47 H
3.56%	1 S	-6.532	1.00	70 H
-3.03%	2 P:y	4.578	0.00	27 C
2.97%	2 S	2.394	0.00	68 H
2.97%	2 S	2.394	0.00	62 H
2.89%	1 S	-6.532	1.00	93 H
2.47%	2 S	2.394	0.00	53 H
2.41%	2 S	2.394	0.00	79 H
-2.36%	2 P:x	4.578	0.00	24 C
2.30%	2 S	2.394	0.00	48 H
2.27%	1 S	-6.532	1.00	84 H
2.23%	1 S	-6.532	1.00	68 H
2.23%	1 S	-6.532	1.00	48 H
1.95%	1 S	-6.532	1.00	76 H
1.84%	1 P:z	-5.263	0.67	24 C
1.79%	1 S	-6.532	1.00	90 H
-1.75%	2 P:z	4.578	0.00	37 C
1.75%	2 S	2.394	0.00	73 H
1.75%	1 S	-6.532	1.00	60 H
1.63%	1 S	-6.532	1.00	95 H
-1.54%	2 P:y	4.578	0.00	24 C
-1.52%	2 P:x	4.578	0.00	38 C
1.47%	2 S	2.394	0.00	93 H
1.47%	1 S	-6.532	1.00	85 H
1.46%	1 S	-6.532	1.00	52 H

			1.44%	1 S	-6.532	1.00	79 H
			1.44%	1 S	-6.532	1.00	73 H
			1.43%	2 S	2.394	0.00	64 H
			-1.43%	2 P:x	4.578	0.00	14 C
			-1.37%	2 P:x	4.578	0.00	21 C
			1.32%	2 P:y	4.578	0.00	42 C
			1.31%	1 S	-6.532	1.00	57 H
			-1.23%	2 P:y	4.578	0.00	18 C
			1.18%	1 S	-6.532	1.00	86 H
			1.17%	1 S	-6.532	1.00	87 H
			1.17%	2 S	2.394	0.00	49 H
			1.14%	2 S	2.394	0.00	90 H
			1.14%	2 P:x	4.578	0.00	34 C
			-1.13%	2 P:x	4.578	0.00	15 C
			1.12%	2 S	2.394	0.00	95 H
			1.11%	2 S	2.394	0.00	87 H
			-1.10%	2 S	2.394	0.00	46 H
			1.10%	1 P:x	-5.263	0.67	34 C
			1.08%	1 S	-6.532	1.00	64 H
			-1.07%	2 P:z	4.578	0.00	34 C
			1.07%	1 P:x	-5.263	0.67	33 C
			1.01%	1 P:x	-5.263	0.67	42 C
			1.00%	2 S	2.394	0.00	52 H
			1.00%	1 S	-6.532	1.00	96 H
1.447	0.00	152 A	10.54%	2 S	2.394	0.00	49 H
			8.42%	1 S	-6.532	1.00	49 H
			7.82%	1 S	-6.532	1.00	96 H
			5.92%	2 S	2.394	0.00	96 H
			5.09%	1 S	-6.532	1.00	65 H
			4.55%	2 S	2.394	0.00	65 H
			4.34%	2 S	2.394	0.00	66 H

4.07%	1 S	-6.532	1.00	86 H
3.60%	1 S	-6.532	1.00	66 H
3.58%	2 S	2.394	0.00	57 H
3.48%	1 S	-6.532	1.00	57 H
-2.93%	2 P:z	4.578	0.00	15 C
2.85%	2 S	2.394	0.00	48 H
2.76%	2 S	2.394	0.00	47 H
-2.58%	2 P:y	4.578	0.00	32 C
2.57%	1 S	-6.532	1.00	87 H
-2.57%	2 P:y	4.578	0.00	40 C
-2.51%	2 P:y	4.578	0.00	21 C
2.32%	1 S	-6.532	1.00	74 H
2.14%	1 S	-6.532	1.00	80 H
2.07%	1 S	-6.532	1.00	47 H
1.98%	2 S	2.394	0.00	87 H
1.98%	2 S	2.394	0.00	59 H
1.90%	1 S	-6.532	1.00	52 H
1.87%	1 S	-6.532	1.00	77 H
1.72%	2 S	2.394	0.00	45 H
1.68%	1 S	-6.532	1.00	48 H
1.63%	1 S	-6.532	1.00	44 H
1.61%	1 S	-6.532	1.00	45 H
1.61%	1 S	-6.532	1.00	54 H
-1.56%	2 P:y	4.578	0.00	26 C
1.45%	2 S	2.394	0.00	52 H
1.45%	2 S	2.394	0.00	86 H
-1.44%	2 P:z	4.578	0.00	39 C
1.35%	2 S	2.394	0.00	79 H
1.31%	2 S	2.394	0.00	74 H
-1.30%	2 P:y	4.578	0.00	25 C
-1.29%	2 P:x	4.578	0.00	18 C

			1.26%	1 S	-6.532	1.00	89 H
			1.25%	2 S	2.394	0.00	78 H
			1.23%	1 S	-6.532	1.00	59 H
			-1.15%	2 P:y	4.578	0.00	34 C
			1.12%	1 S	-6.532	1.00	60 H
			1.10%	2 S	2.394	0.00	89 H
			1.09%	2 S	2.394	0.00	44 H
			-1.09%	2 P:z	4.578	0.00	32 C
			1.08%	2 S	2.394	0.00	77 H
			-1.03%	2 S	11.741	0.00	15 C
			-1.03%	2 P:x	4.578	0.00	32 C
1.503	0.00	153 A	5.91%	1 P:x	-5.263	0.67	18 C
			5.69%	1 S	-6.532	1.00	95 H
			-5.56%	2 P:z	4.578	0.00	32 C
			4.39%	1 S	-6.532	1.00	94 H
			4.08%	2 S	2.394	0.00	49 H
			4.01%	1 S	-6.532	1.00	73 H
			3.96%	2 S	2.394	0.00	55 H
			3.87%	2 S	2.394	0.00	95 H
			3.47%	1 S	-6.532	1.00	84 H
			3.15%	1 S	-6.532	1.00	78 H
			2.93%	2 S	2.394	0.00	84 H
			2.83%	1 S	-6.532	1.00	55 H
			2.46%	1 S	-6.532	1.00	48 H
			2.37%	2 S	2.394	0.00	48 H
			2.16%	1 S	-6.532	1.00	79 H
			-2.15%	2 P:x	4.578	0.00	32 C
			2.09%	1 S	-6.532	1.00	49 H
			1.98%	1 P:z	-5.263	0.67	11 C
			1.97%	1 S	-6.532	1.00	66 H
			1.83%	1 S	-6.532	1.00	86 H

			1.79%	2 S	2.394	0.00	79 H	
			1.65%	1 P:z	-5.263	0.67	32 C	
			1.58%	1 S	-6.532	1.00	96 H	
			1.56%	1 S	-6.532	1.00	70 H	
			1.52%	2 S	2.394	0.00	80 H	
			1.49%	2 S	2.394	0.00	66 H	
			1.47%	1 S	-6.532	1.00	51 H	
			1.44%	1 S	-6.532	1.00	63 H	
			1.44%	2 S	2.394	0.00	94 H	
			1.40%	1 P:z	-5.263	0.67	29 C	
			-1.37%	2 P:x	4.578	0.00	38 C	
			-1.35%	2 P:y	4.578	0.00	18 C	
			1.34%	1 S	-23.822	2.00	1 O	
			1.18%	1 S	-6.532	1.00	85 H	
			-1.14%	2 P:y	4.578	0.00	25 C	
			1.11%	1 S	-6.532	1.00	80 H	
			-1.11%	2 P:z	4.578	0.00	26 C	
			1.09%	1 S	-13.631	2.00	29 C	
			1.08%	1 S	-6.532	1.00	65 H	
			1.07%	1 S	-6.532	1.00	91 H	
			1.05%	2 S	2.394	0.00	86 H	
			-1.04%	2 P:z	4.578	0.00	15 C	
			1.04%	2 S	2.394	0.00	96 H	
			1.02%	1 S	-6.532	1.00	46 H	
			-1.02%	2 P:y	4.578	0.00	40 C	
			1.00%	1 P:x	-9.066	1.33	1 O	
1.574	0.00	154 A	14.51%	1 S	-6.532	1.00	92 H	
			-12.66%	2 P:z	4.578	0.00	42 C	
			8.22%	2 S	2.394	0.00	92 H	
			4.44%	1 S	-6.532	1.00	91 H	
			4.12%	1 S	-6.532	1.00	93 H	

-4.09%	2 P:z	4.578	0.00	30	C
3.94%	1 P:z	-5.263	0.67	42	C
3.87%	1 S	-6.532	1.00	83	H
3.58%	1 S	-6.532	1.00	73	H
3.50%	1 S	-6.532	1.00	81	H
2.69%	2 S	2.394	0.00	67	H
2.60%	1 S	-6.532	1.00	67	H
2.58%	1 S	-6.532	1.00	74	H
2.57%	2 S	2.394	0.00	83	H
2.23%	2 S	2.394	0.00	72	H
2.10%	2 S	2.394	0.00	73	H
2.09%	2 P:z	4.578	0.00	20	C
1.71%	2 S	2.394	0.00	91	H
1.68%	1 S	-6.532	1.00	55	H
1.49%	1 P:x	-5.263	0.67	33	C
1.41%	1 S	-6.532	1.00	69	H
1.40%	2 S	2.394	0.00	81	H
1.38%	1 S	-6.532	1.00	53	H
1.36%	1 P:z	-5.263	0.67	11	C
1.34%	2 S	2.394	0.00	68	H
1.34%	1 S	-6.532	1.00	58	H
-1.30%	2 P:x	4.578	0.00	26	C
1.30%	1 P:z	-5.263	0.67	20	C
1.25%	1 P:z	-5.263	0.67	30	C
1.24%	2 S	2.394	0.00	71	H
1.18%	2 S	2.394	0.00	53	H
1.17%	2 S	2.394	0.00	76	H
-1.13%	2 P:x	4.578	0.00	15	C
1.13%	2 S	2.394	0.00	69	H
1.11%	1 S	-6.532	1.00	62	H
1.08%	2 S	2.394	0.00	84	H

			1.06%	2 S	2.394	0.00	74 H
			-1.05%	2 S	11.741	0.00	24 C
			-1.04%	2 P:z	4.578	0.00	39 C
			1.03%	1 S	-6.532	1.00	48 H
1.654	0.00	155 A	23.82%	1 S	-6.532	1.00	67 H
			13.91%	2 S	2.394	0.00	67 H
			-7.86%	2 P:z	4.578	0.00	21 C
			6.09%	1 S	-6.532	1.00	65 H
			5.93%	1 S	-6.532	1.00	64 H
			-4.51%	2 P:y	4.578	0.00	40 C
			4.38%	2 S	2.394	0.00	47 H
			3.60%	1 S	-6.532	1.00	72 H
			3.30%	1 S	-6.532	1.00	61 H
			3.05%	1 S	-6.532	1.00	47 H
			2.92%	1 S	-6.532	1.00	70 H
			2.90%	2 S	2.394	0.00	65 H
			-2.86%	2 P:z	4.578	0.00	40 C
			-2.69%	2 P:y	4.578	0.00	20 C
			-2.15%	2 P:y	4.578	0.00	21 C
			1.88%	2 S	2.394	0.00	72 H
			1.85%	1 P:x	-5.263	0.67	21 C
			1.79%	1 P:x	-5.263	0.67	39 C
			1.61%	1 P:z	-5.263	0.67	20 C
			1.56%	1 S	-6.532	1.00	50 H
			-1.56%	2 S	2.394	0.00	68 H
			1.44%	1 P:z	-5.263	0.67	21 C
			1.41%	2 S	2.394	0.00	64 H
			1.26%	2 S	2.394	0.00	61 H
			1.22%	1 P:y	-5.263	0.67	40 C
			1.22%	2 S	2.394	0.00	70 H
			1.21%	1 P:y	-5.263	0.67	39 C

			1.20%	1 S	-6.532	1.00	86 H
			1.20%	3 S	40.877	0.00	67 H
			-1.17%	2 P:z	4.578	0.00	20 C
			1.15%	1 S	-6.532	1.00	44 H
			1.13%	1 S	-6.532	1.00	93 H
			1.07%	2 S	2.394	0.00	86 H
			-1.04%	2 P:x	4.578	0.00	21 C
			1.03%	1 P:y	-5.263	0.67	21 C
			-1.02%	2 P:z	4.578	0.00	15 C
			1.01%	1 S	-6.532	1.00	55 H
			-1.01%	2 P:y	4.578	0.00	22 C
			1.01%	1 S	-6.532	1.00	49 H
1.742	0.00	156 A	5.66%	1 S	-6.532	1.00	83 H
			3.87%	1 S	-6.532	1.00	52 H
			3.82%	2 S	2.394	0.00	83 H
			3.71%	2 S	2.394	0.00	47 H
			3.71%	1 S	-6.532	1.00	47 H
			-3.30%	2 P:z	4.578	0.00	38 C
			3.21%	1 S	-6.532	1.00	91 H
			3.13%	2 S	2.394	0.00	73 H
			2.92%	2 S	2.394	0.00	91 H
			2.48%	2 S	2.394	0.00	54 H
			2.43%	1 S	-6.532	1.00	50 H
			2.40%	1 S	-6.532	1.00	80 H
			2.29%	1 S	-6.532	1.00	88 H
			2.27%	2 S	2.394	0.00	55 H
			2.22%	1 S	-6.532	1.00	44 H
			2.17%	1 P:z	-5.263	0.67	33 C
			2.03%	2 P:z	4.578	0.00	30 C
			2.01%	1 S	-6.532	1.00	55 H
			-2.00%	2 P:x	4.578	0.00	31 C

			2.00%	1 S	-6.532	1.00	78 H
			1.97%	1 S	-6.532	1.00	73 H
			1.93%	1 S	-6.532	1.00	75 H
			1.84%	2 S	2.394	0.00	74 H
			1.84%	1 S	-6.532	1.00	87 H
			1.69%	1 S	-6.532	1.00	86 H
			1.67%	1 S	-6.532	1.00	82 H
			1.60%	1 S	-6.532	1.00	89 H
			-1.60%	2 P:x	4.578	0.00	30 C
			1.50%	2 S	2.394	0.00	56 H
			1.49%	1 P:y	-5.263	0.67	12 C
			1.35%	1 S	-6.532	1.00	57 H
			1.31%	1 S	-6.532	1.00	95 H
			-1.30%	2 P:x	4.578	0.00	22 C
			1.29%	2 S	2.394	0.00	57 H
			1.28%	2 S	2.394	0.00	89 H
			1.27%	1 S	-6.532	1.00	72 H
			-1.17%	2 P:y	4.578	0.00	12 C
			1.12%	2 S	2.394	0.00	52 H
			1.11%	2 S	2.394	0.00	59 H
			1.11%	2 S	2.394	0.00	80 H
			1.10%	1 P:x	-5.263	0.67	33 C
			1.09%	2 S	2.394	0.00	75 H
			1.08%	2 S	2.394	0.00	65 H
			-1.08%	2 P:x	4.578	0.00	17 C
			1.06%	1 P:x	-7.073	1.00	43 N
			-1.03%	2 P:x	4.578	0.00	34 C
			1.03%	1 S	-6.532	1.00	65 H
			1.02%	1 P:x	-5.263	0.67	39 C
			1.01%	1 P:y	-5.263	0.67	38 C
1.805	0.00	157 A	10.12%	1 S	-6.532	1.00	76 H

9.91%	1 S	-6.532	1.00	74 H
6.02%	2 S	2.394	0.00	76 H
3.24%	1 S	-6.532	1.00	85 H
3.22%	2 S	2.394	0.00	78 H
2.98%	1 S	-6.532	1.00	72 H
2.86%	1 S	-6.532	1.00	77 H
2.77%	1 P:y	-5.263	0.67	11 C
2.59%	2 S	2.394	0.00	79 H
-2.53%	2 P:z	4.578	0.00	30 C
2.50%	2 P:y	4.578	0.00	30 C
2.41%	1 S	-6.532	1.00	83 H
2.32%	1 S	-6.532	1.00	75 H
2.32%	1 S	-13.631	2.00	29 C
2.14%	1 S	-6.532	1.00	88 H
2.06%	2 S	2.394	0.00	74 H
2.01%	1 S	-6.532	1.00	62 H
2.01%	1 S	-6.532	1.00	79 H
1.98%	1 P:x	-5.263	0.67	26 C
1.97%	1 S	-6.532	1.00	92 H
1.96%	1 P:x	-5.263	0.67	29 C
-1.87%	2 S	11.741	0.00	29 C
1.84%	1 P:z	-5.263	0.67	30 C
1.75%	1 S	-6.532	1.00	78 H
-1.69%	2 P:y	4.578	0.00	13 C
-1.69%	2 P:y	4.578	0.00	29 C
1.65%	2 P:x	4.578	0.00	26 C
-1.65%	2 P:x	4.578	0.00	29 C
1.65%	2 S	2.394	0.00	49 H
1.64%	1 S	-6.532	1.00	49 H
-1.50%	2 P:y	4.578	0.00	20 C
1.47%	1 S	-6.532	1.00	96 H

			-1.45%	2 P:y	4.578	0.00	11 C	
			1.40%	1 P:y	-5.263	0.67	26 C	
			-1.37%	2 P:z	4.578	0.00	15 C	
			-1.37%	2 S	16.116	0.00	43 N	
			1.35%	2 S	2.394	0.00	62 H	
			1.26%	1 S	-6.532	1.00	70 H	
			-1.26%	2 P:z	4.578	0.00	42 C	
			1.25%	2 S	2.394	0.00	92 H	
			1.25%	1 P:y	-5.263	0.67	28 C	
			1.22%	1 S	-18.438	2.00	43 N	
			1.20%	2 S	2.394	0.00	72 H	
			-1.18%	2 P:x	4.578	0.00	38 C	
			-1.16%	2 S	2.394	0.00	88 H	
			1.10%	1 S	-6.532	1.00	84 H	
			1.03%	1 P:z	-9.066	1.33	3 O	
			1.00%	1 P:x	-5.263	0.67	18 C	
1.868	0.00	158 A	10.02%	1 S	-6.532	1.00	95 H	
			6.40%	2 S	2.394	0.00	95 H	
			5.92%	1 S	-6.532	1.00	75 H	
			5.41%	1 S	-6.532	1.00	63 H	
			-5.19%	2 P:x	4.578	0.00	30 C	
			5.11%	1 S	-6.532	1.00	56 H	
			-3.81%	2 P:y	4.578	0.00	40 C	
			3.78%	1 P:y	-5.263	0.67	12 C	
			3.45%	1 S	-6.532	1.00	65 H	
			3.41%	1 S	-6.532	1.00	84 H	
			3.03%	1 S	-6.532	1.00	73 H	
			2.64%	1 S	-6.532	1.00	87 H	
			2.29%	2 S	2.394	0.00	75 H	
			2.00%	2 S	2.394	0.00	63 H	
			1.93%	1 S	-6.532	1.00	50 H	

1.91%	1 S	-6.532	1.00	58 H
1.90%	1 S	-6.532	1.00	83 H
-1.84%	2 P:z	4.578	0.00	15 C
1.80%	1 P:x	-5.263	0.67	30 C
1.77%	2 S	2.394	0.00	58 H
-1.76%	2 P:z	4.578	0.00	32 C
1.71%	1 P:y	-5.263	0.67	40 C
1.69%	2 S	2.394	0.00	52 H
-1.69%	2 P:y	4.578	0.00	32 C
1.68%	1 S	-6.532	1.00	52 H
1.65%	1 P:x	-5.263	0.67	39 C
-1.63%	2 P:x	4.578	0.00	38 C
1.62%	2 S	2.394	0.00	76 H
1.59%	1 S	-6.532	1.00	66 H
1.54%	1 S	-6.532	1.00	49 H
1.53%	2 S	2.394	0.00	87 H
1.41%	2 S	2.394	0.00	57 H
1.38%	2 S	2.394	0.00	49 H
1.28%	1 P:z	-5.263	0.67	36 C
1.27%	1 P:x	-5.263	0.67	38 C
1.22%	1 S	-6.532	1.00	47 H
1.21%	1 S	-13.631	2.00	39 C
-1.20%	2 P:x	4.578	0.00	15 C
-1.19%	2 P:x	4.578	0.00	32 C
1.18%	2 P:y	4.578	0.00	39 C
-1.13%	2 S	11.741	0.00	39 C
1.11%	1 S	-6.532	1.00	80 H
1.06%	1 P:y	-5.263	0.67	11 C
1.06%	2 S	2.394	0.00	66 H
1.03%	2 P:x	4.578	0.00	39 C
1.02%	2 P:z	4.578	0.00	17 C

			1.02%	1 S	-6.532	1.00	70 H
			1.01%	2 S	2.394	0.00	56 H
1.934	0.00	159 A	5.66%	1 S	-6.532	1.00	96 H
			5.65%	1 S	-6.532	1.00	62 H
			4.13%	1 S	-6.532	1.00	59 H
			3.77%	2 S	2.394	0.00	96 H
			3.74%	2 S	2.394	0.00	62 H
			3.42%	1 S	-6.532	1.00	64 H
			3.07%	1 S	-6.532	1.00	70 H
			2.90%	1 S	-6.532	1.00	49 H
			2.68%	1 S	-6.532	1.00	60 H
			2.59%	1 S	-6.532	1.00	88 H
			2.32%	1 S	-6.532	1.00	82 H
			2.32%	2 S	2.394	0.00	49 H
			2.29%	1 S	-6.532	1.00	47 H
			-2.29%	2 P:z	4.578	0.00	15 C
			2.18%	2 S	2.394	0.00	91 H
			2.02%	2 S	2.394	0.00	47 H
			1.99%	2 S	2.394	0.00	59 H
			1.90%	2 S	2.394	0.00	70 H
			1.89%	2 P:y	4.578	0.00	42 C
			1.87%	1 P:y	-5.263	0.67	12 C
			1.86%	1 S	-6.532	1.00	81 H
			-1.81%	2 S	11.741	0.00	37 C
			1.77%	1 S	-6.532	1.00	67 H
			1.73%	2 S	2.394	0.00	56 H
			1.67%	1 S	-6.532	1.00	56 H
			1.62%	2 S	2.394	0.00	64 H
			1.56%	1 P:y	-5.263	0.67	11 C
			1.50%	1 S	-6.532	1.00	72 H
			1.50%	2 S	2.394	0.00	60 H

			-1.44%	2 P:y	4.578	0.00	34 C
			1.42%	2 S	2.394	0.00	82 H
			1.39%	1 S	-6.532	1.00	45 H
			-1.33%	2 P:z	4.578	0.00	38 C
			1.31%	1 S	-6.532	1.00	54 H
			1.30%	1 S	-13.631	2.00	37 C
			1.27%	1 S	-6.532	1.00	91 H
			1.25%	1 S	-6.532	1.00	77 H
			1.25%	2 S	2.394	0.00	67 H
			1.24%	1 P:y	-5.263	0.67	40 C
			-1.24%	2 P:z	4.578	0.00	20 C
			1.22%	1 S	-6.532	1.00	48 H
			1.18%	2 P:y	4.578	0.00	37 C
			1.10%	1 P:y	-9.066	1.33	7 O
			-1.07%	2 P:y	4.578	0.00	32 C
			1.07%	1 P:z	-5.263	0.67	26 C
			-1.06%	2 P:z	4.578	0.00	37 C
			1.05%	1 S	-6.532	1.00	44 H
			-1.04%	2 P:x	4.578	0.00	32 C
			1.01%	2 S	2.394	0.00	87 H
			-1.01%	2 P:y	4.578	0.00	20 C
1.989	0.00	160 A	6.28%	1 S	-6.532	1.00	47 H
			6.17%	1 S	-6.532	1.00	92 H
			5.63%	1 S	-6.532	1.00	87 H
			4.80%	1 S	-6.532	1.00	73 H
			4.64%	2 S	2.394	0.00	47 H
			3.82%	1 P:y	-5.263	0.67	38 C
			3.56%	2 S	2.394	0.00	73 H
			-3.54%	2 P:z	4.578	0.00	30 C
			3.20%	1 S	-6.532	1.00	63 H
			3.17%	1 S	-6.532	1.00	74 H

3.05%	1 S	-6.532	1.00	88 H
-2.88%	2 P:z	4.578	0.00	42 C
-2.52%	2 P:y	4.578	0.00	40 C
2.39%	1 S	-6.532	1.00	65 H
2.37%	2 S	2.394	0.00	72 H
2.32%	1 S	-6.532	1.00	81 H
2.30%	1 S	-6.532	1.00	85 H
2.24%	1 S	-6.532	1.00	91 H
2.24%	1 S	-6.532	1.00	44 H
2.23%	1 S	-6.532	1.00	62 H
2.22%	1 S	-6.532	1.00	83 H
2.19%	1 S	-6.532	1.00	59 H
-2.18%	2 P:z	4.578	0.00	38 C
2.08%	2 S	2.394	0.00	83 H
2.06%	1 S	-6.532	1.00	82 H
2.01%	1 P:x	-5.263	0.67	26 C
1.88%	1 S	-6.532	1.00	86 H
1.77%	1 P:y	-5.263	0.67	12 C
-1.62%	2 P:x	4.578	0.00	34 C
-1.58%	2 P:y	4.578	0.00	12 C
1.51%	1 P:z	-5.263	0.67	33 C
1.51%	2 P:y	4.578	0.00	38 C
1.47%	1 S	-18.438	2.00	43 N
1.45%	1 P:z	-5.263	0.67	30 C
1.43%	1 S	-6.532	1.00	72 H
1.43%	2 S	2.394	0.00	59 H
-1.39%	2 P:z	4.578	0.00	12 C
-1.38%	2 P:x	4.578	0.00	31 C
-1.28%	2 S	16.116	0.00	43 N
1.26%	2 P:x	4.578	0.00	40 C
1.21%	2 S	2.394	0.00	92 H

			1.19%	2 S	2.394	0.00	84 H		
			1.19%	2 S	2.394	0.00	62 H		
			1.19%	2 S	2.394	0.00	70 H		
			-1.13%	2 P:x	4.578	0.00	22 C		
			1.13%	1 P:z	-5.263	0.67	42 C		
			-1.10%	2 S	11.741	0.00	37 C		
			1.09%	2 S	2.394	0.00	66 H		
			1.06%	1 P:y	-5.263	0.67	40 C		
			-1.04%	2 S	2.394	0.00	91 H		
			1.02%	1 P:x	-5.263	0.67	34 C		
			1.00%	2 P:z	4.578	0.00	26 C		
2.070	0.00	161 A	15.79%	1 S	-6.532	1.00	48 H		
			10.47%	2 S	2.394	0.00	48 H		
			8.75%	1 S	-6.532	1.00	87 H		
			-6.39%	2 P:x	4.578	0.00	15 C		
			6.22%	2 S	2.394	0.00	87 H		
			-6.00%	2 P:z	4.578	0.00	15 C		
			5.75%	1 S	-6.532	1.00	57 H		
			5.52%	1 S	-6.532	1.00	49 H		
			4.92%	1 S	-6.532	1.00	59 H		
			-4.83%	2 P:x	4.578	0.00	32 C		
			-3.88%	2 P:y	4.578	0.00	39 C		
			3.72%	1 P:y	-5.263	0.67	39 C		
			3.54%	1 S	-6.532	1.00	94 H		
			3.17%	1 S	-6.532	1.00	95 H		
			3.03%	1 S	-6.532	1.00	69 H		
			2.98%	1 S	-6.532	1.00	96 H		
			2.79%	1 P:x	-5.263	0.67	15 C		
			2.64%	1 S	-6.532	1.00	63 H		
			2.29%	1 S	-6.532	1.00	54 H		
			2.04%	2 S	2.394	0.00	49 H		

			1.67%	2 S	2.394	0.00	95 H
			1.44%	2 S	2.394	0.00	96 H
			-1.43%	2 P:y	4.578	0.00	15 C
			1.37%	1 S	-6.532	1.00	92 H
			-1.36%	2 S	2.394	0.00	57 H
			-1.34%	2 P:x	4.578	0.00	12 C
			1.32%	3 S	40.877	0.00	48 H
			1.32%	1 P:z	-5.263	0.67	15 C
			1.31%	2 S	2.394	0.00	56 H
			-1.29%	2 P:x	4.578	0.00	14 C
			1.24%	2 P:z	4.578	0.00	17 C
			1.23%	1 S	-6.532	1.00	56 H
			-1.22%	2 S	2.394	0.00	85 H
			1.22%	2 S	2.394	0.00	59 H
			-1.22%	2 P:x	4.578	0.00	11 C
			1.19%	1 P:x	-5.263	0.67	36 C
			1.16%	1 P:z	-5.263	0.67	19 C
			-1.13%	2 P:x	4.578	0.00	39 C
			-1.13%	2 P:z	4.578	0.00	12 C
			1.10%	2 S	2.394	0.00	94 H
			1.02%	1 S	-6.532	1.00	50 H
2.124	0.00	162 A	7.44%	2 S	2.394	0.00	79 H
			7.14%	1 S	-6.532	1.00	79 H
			6.70%	1 S	-6.532	1.00	72 H
			6.54%	2 S	2.394	0.00	72 H
			5.23%	1 S	-6.532	1.00	95 H
			-4.79%	2 P:z	4.578	0.00	26 C
			4.09%	1 P:y	-5.263	0.67	12 C
			3.64%	1 S	-6.532	1.00	92 H
			3.34%	1 S	-6.532	1.00	75 H
			3.07%	1 P:z	-5.263	0.67	26 C

-2.57%	2 P:y	4.578	0.00	20 C
2.42%	1 S	-6.532	1.00	85 H
2.42%	1 P:y	-5.263	0.67	38 C
2.10%	1 S	-6.532	1.00	82 H
-2.08%	2 P:x	4.578	0.00	12 C
1.98%	1 S	-6.532	1.00	81 H
1.85%	1 P:z	-5.263	0.67	42 C
1.78%	1 S	-6.532	1.00	78 H
1.76%	2 S	2.394	0.00	92 H
1.75%	1 S	-6.532	1.00	96 H
1.75%	1 S	-6.532	1.00	70 H
1.71%	1 S	-6.532	1.00	59 H
1.62%	1 P:z	-5.263	0.67	29 C
1.61%	1 P:y	-9.066	1.33	7 O
1.59%	2 S	2.394	0.00	95 H
-1.57%	2 P:x	4.578	0.00	30 C
1.51%	1 P:y	-5.263	0.67	32 C
1.41%	1 S	-6.532	1.00	74 H
1.40%	2 S	2.394	0.00	82 H
1.39%	2 S	2.394	0.00	70 H
1.30%	2 S	2.394	0.00	56 H
1.25%	2 S	2.394	0.00	81 H
-1.23%	2 S	2.394	0.00	93 H
1.22%	2 P:y	4.578	0.00	42 C
1.20%	1 S	-6.532	1.00	46 H
1.18%	2 S	2.394	0.00	80 H
1.11%	2 P:x	4.578	0.00	42 C
1.07%	2 S	2.394	0.00	87 H
1.06%	1 S	-6.532	1.00	44 H
1.03%	2 S	2.394	0.00	85 H

2.186 0.00 163 A 14.05% 1 S -6.532 1.00 75 H

-9.30%	2 P:x	4.578	0.00	30 C
7.38%	1 S	-6.532	1.00	73 H
6.47%	1 P:x	-5.263	0.67	30 C
5.06%	1 S	-6.532	1.00	85 H
3.63%	1 S	-6.532	1.00	86 H
3.49%	2 S	2.394	0.00	68 H
3.43%	1 S	-6.532	1.00	91 H
3.07%	1 S	-6.532	1.00	58 H
3.06%	2 S	2.394	0.00	79 H
-2.82%	2 P:z	4.578	0.00	39 C
2.49%	2 S	2.394	0.00	75 H
2.27%	1 S	-6.532	1.00	79 H
2.10%	1 S	-6.532	1.00	68 H
1.96%	1 S	-6.532	1.00	63 H
1.95%	1 S	-6.532	1.00	56 H
-1.89%	2 P:y	4.578	0.00	26 C
1.86%	2 P:z	4.578	0.00	26 C
1.80%	1 S	-6.532	1.00	64 H
1.79%	1 S	-6.532	1.00	61 H
-1.65%	2 P:z	4.578	0.00	40 C
-1.61%	2 S	2.394	0.00	73 H
1.60%	1 S	-6.532	1.00	84 H
1.58%	2 P:x	4.578	0.00	28 C
1.55%	1 P:y	-5.263	0.67	29 C
-1.52%	2 P:z	4.578	0.00	37 C
1.49%	1 S	-6.532	1.00	76 H
1.44%	1 S	-6.532	1.00	59 H
1.38%	1 S	-6.532	1.00	82 H
1.36%	1 P:z	-5.263	0.67	40 C
1.30%	2 S	2.394	0.00	58 H
1.29%	1 S	-6.532	1.00	80 H

			1.27%	1 P:x	-5.263	0.67	13 C
			1.14%	1 S	-6.532	1.00	52 H
			-1.13%	2 P:x	4.578	0.00	12 C
			1.05%	1 P:x	-5.263	0.67	33 C
			1.02%	2 S	2.394	0.00	45 H
2.273	0.00	164 A	5.78%	1 S	-6.532	1.00	44 H
			5.32%	1 S	-6.532	1.00	59 H
			3.94%	1 P:x	-5.263	0.67	39 C
			3.72%	1 S	-6.532	1.00	49 H
			3.51%	1 S	-6.532	1.00	71 H
			3.32%	1 S	-6.532	1.00	60 H
			3.13%	1 S	-6.532	1.00	52 H
			3.01%	1 S	-6.532	1.00	48 H
			2.77%	1 S	-6.532	1.00	57 H
			2.68%	1 S	-6.532	1.00	75 H
			2.66%	1 S	-6.532	1.00	70 H
			2.49%	1 S	-6.532	1.00	64 H
			-2.42%	2 P:x	4.578	0.00	20 C
			2.41%	1 P:y	-5.263	0.67	12 C
			2.34%	1 S	-6.532	1.00	95 H
			2.32%	1 S	-6.532	1.00	55 H
			-2.23%	2 P:x	4.578	0.00	15 C
			1.97%	1 S	-6.532	1.00	51 H
			1.95%	1 P:y	-5.263	0.67	30 C
			1.89%	2 S	2.394	0.00	52 H
			-1.88%	2 P:z	4.578	0.00	15 C
			1.85%	1 P:z	-5.263	0.67	29 C
			1.84%	1 P:y	-5.263	0.67	15 C
			-1.65%	2 S	2.394	0.00	59 H
			1.65%	2 S	2.394	0.00	71 H
			1.53%	1 P:y	-5.263	0.67	38 C

			1.45%	1 S	-6.532	1.00	76 H
			1.42%	2 P:y	4.578	0.00	42 C
			1.41%	2 S	2.394	0.00	64 H
			-1.35%	2 S	2.394	0.00	85 H
			1.27%	1 P:z	-5.263	0.67	27 C
			1.27%	1 P:z	-5.263	0.67	21 C
			-1.26%	2 S	2.394	0.00	70 H
			1.26%	2 P:x	4.578	0.00	40 C
			-1.26%	2 P:x	4.578	0.00	30 C
			1.26%	2 S	2.394	0.00	55 H
			1.25%	2 S	2.394	0.00	76 H
			-1.22%	2 P:y	4.578	0.00	39 C
			-1.22%	2 S	11.741	0.00	25 C
			1.17%	1 S	-6.532	1.00	73 H
			-1.15%	2 P:y	4.578	0.00	22 C
			1.15%	1 S	-6.532	1.00	53 H
			1.13%	1 S	-6.532	1.00	89 H
			1.12%	1 P:x	-5.263	0.67	20 C
			1.08%	2 P:y	4.578	0.00	30 C
			-1.08%	2 P:x	4.578	0.00	22 C
			1.06%	1 P:x	-5.263	0.67	21 C
			-1.04%	2 P:z	4.578	0.00	36 C
			1.03%	2 S	2.394	0.00	48 H
			1.03%	1 S	-13.631	2.00	25 C
			1.03%	1 S	-6.532	1.00	86 H
			1.02%	1 P:x	-5.263	0.67	22 C
2.305	0.00	165 A	10.54%	1 S	-6.532	1.00	64 H
			6.87%	1 S	-6.532	1.00	60 H
			6.46%	1 S	-6.532	1.00	65 H
			3.64%	1 S	-6.532	1.00	47 H
			3.57%	1 P:y	-5.263	0.67	40 C

-3.51%	2 P:z	4.578	0.00	40 C
3.25%	1 S	-6.532	1.00	96 H
3.04%	1 S	-6.532	1.00	62 H
2.65%	1 S	-13.631	2.00	33 C
-2.49%	2 P:y	4.578	0.00	40 C
-2.45%	2 P:z	4.578	0.00	37 C
2.44%	1 P:y	-5.263	0.67	12 C
2.32%	1 S	-6.532	1.00	81 H
2.30%	1 S	-6.532	1.00	52 H
2.25%	2 S	2.394	0.00	47 H
2.24%	1 S	-6.532	1.00	92 H
2.18%	1 S	-6.532	1.00	67 H
2.07%	1 P:z	-5.263	0.67	40 C
2.03%	1 S	-6.532	1.00	86 H
-1.99%	2 S	2.394	0.00	65 H
1.88%	2 S	2.394	0.00	60 H
1.76%	2 S	2.394	0.00	55 H
1.63%	1 S	-6.532	1.00	59 H
-1.62%	2 S	11.741	0.00	33 C
-1.56%	2 P:z	4.578	0.00	34 C
1.55%	1 P:x	-5.263	0.67	33 C
1.54%	1 S	-6.532	1.00	68 H
1.41%	1 S	-6.532	1.00	55 H
-1.35%	2 P:y	4.578	0.00	33 C
1.24%	1 S	-6.532	1.00	79 H
-1.24%	2 P:y	4.578	0.00	32 C
1.22%	1 S	-6.532	1.00	58 H
1.21%	1 S	-6.532	1.00	95 H
1.12%	2 S	2.394	0.00	62 H
1.10%	1 S	-6.532	1.00	46 H
1.07%	1 S	-6.532	1.00	69 H

			1.05%	1 P:x	-5.263	0.67	39 C
			1.04%	1 S	-6.532	1.00	66 H
			1.03%	2 S	2.394	0.00	56 H
			-1.02%	2 P:y	4.578	0.00	34 C
			1.01%	2 S	2.394	0.00	96 H
2.402	0.00	166 A	13.79%	1 S	-6.532	1.00	68 H
			7.51%	2 S	2.394	0.00	68 H
			-7.17%	2 P:x	4.578	0.00	21 C
			5.21%	1 S	-6.532	1.00	73 H
			4.03%	1 S	-6.532	1.00	60 H
			3.83%	2 S	2.394	0.00	45 H
			3.75%	1 S	-6.532	1.00	82 H
			3.68%	1 S	-6.532	1.00	91 H
			-3.34%	2 P:y	4.578	0.00	34 C
			3.02%	1 S	-6.532	1.00	67 H
			3.00%	1 P:x	-5.263	0.67	21 C
			2.96%	1 S	-6.532	1.00	80 H
			2.78%	1 P:y	-5.263	0.67	26 C
			2.69%	1 S	-6.532	1.00	74 H
			2.55%	1 S	-6.532	1.00	47 H
			2.47%	1 P:z	-5.263	0.67	33 C
			2.30%	1 S	-6.532	1.00	46 H
			-2.28%	2 P:z	4.578	0.00	28 C
			2.07%	2 S	2.394	0.00	82 H
			2.07%	1 S	-6.532	1.00	96 H
			1.89%	1 P:z	-5.263	0.67	26 C
			1.76%	2 S	2.394	0.00	47 H
			1.74%	1 P:y	-5.263	0.67	38 C
			1.62%	2 S	2.394	0.00	61 H
			1.59%	1 S	-6.532	1.00	87 H
			-1.49%	2 S	2.394	0.00	73 H

			1.46%	1 P:x	-5.263	0.67	33 C	
			-1.44%	2 P:z	4.578	0.00	30 C	
			1.44%	1 S	-6.532	1.00	45 H	
			-1.44%	2 P:z	4.578	0.00	37 C	
			1.44%	2 S	2.394	0.00	87 H	
			1.41%	1 P:z	-5.263	0.67	30 C	
			1.38%	1 S	-6.532	1.00	61 H	
			1.28%	1 S	-6.532	1.00	72 H	
			1.27%	1 S	-6.532	1.00	78 H	
			1.21%	1 S	-6.532	1.00	86 H	
			1.21%	1 S	-18.438	2.00	43 N	
			-1.19%	2 P:y	4.578	0.00	37 C	
			-1.16%	2 S	16.116	0.00	43 N	
			-1.03%	2 P:z	4.578	0.00	38 C	
			1.01%	1 S	-6.532	1.00	54 H	
			1.01%	3 S	40.877	0.00	68 H	
2.487	0.00	167 A	6.14%	1 S	-6.532	1.00	87 H	
			5.70%	1 S	-6.532	1.00	74 H	
			4.57%	1 S	-6.532	1.00	78 H	
			4.01%	1 S	-6.532	1.00	47 H	
			3.89%	2 S	2.394	0.00	78 H	
			3.63%	1 P:z	-5.263	0.67	12 C	
			-3.54%	2 P:z	4.578	0.00	28 C	
			3.43%	1 S	-6.532	1.00	56 H	
			3.29%	1 S	-6.532	1.00	52 H	
			2.83%	1 S	-6.532	1.00	63 H	
			2.64%	1 S	-6.532	1.00	85 H	
			2.53%	2 S	2.394	0.00	87 H	
			2.48%	1 S	-6.532	1.00	61 H	
			2.48%	1 P:y	-5.263	0.67	26 C	
			2.43%	1 S	-6.532	1.00	57 H	

-2.26%	2 P:z	4.578	0.00	30 C
2.19%	2 S	2.394	0.00	68 H
2.16%	1 S	-6.532	1.00	46 H
2.02%	1 S	-6.532	1.00	68 H
1.92%	1 S	-6.532	1.00	73 H
-1.82%	2 P:y	4.578	0.00	34 C
1.77%	1 P:x	-5.263	0.67	13 C
1.77%	1 S	-6.532	1.00	49 H
1.75%	1 P:z	-5.263	0.67	30 C
1.69%	1 S	-6.532	1.00	72 H
1.66%	1 S	-6.532	1.00	80 H
1.55%	1 S	-6.532	1.00	86 H
-1.54%	2 P:y	4.578	0.00	37 C
1.49%	2 S	2.394	0.00	47 H
-1.43%	2 S	2.394	0.00	85 H
1.41%	1 P:y	-5.263	0.67	40 C
1.41%	1 P:y	-5.263	0.67	20 C
1.39%	1 P:z	-5.263	0.67	38 C
1.39%	1 S	-6.532	1.00	84 H
-1.33%	2 P:x	4.578	0.00	23 C
1.30%	1 P:z	-5.263	0.67	36 C
1.29%	2 P:z	4.578	0.00	21 C
-1.28%	2 P:x	4.578	0.00	12 C
-1.24%	2 S	16.116	0.00	43 N
1.23%	1 P:x	-5.263	0.67	26 C
1.23%	2 P:x	4.578	0.00	30 C
-1.23%	2 P:z	4.578	0.00	12 C
1.17%	1 S	-6.532	1.00	75 H
1.13%	1 S	-6.532	1.00	94 H
1.12%	2 S	2.394	0.00	66 H
-1.12%	2 P:z	4.578	0.00	15 C

			1.11%	2 S	2.394	0.00	57 H
			1.10%	1 S	-18.438	2.00	43 N
			1.02%	2 S	2.394	0.00	61 H
2.544	0.00	168 A	16.17%	1 S	-6.532	1.00	70 H
			6.55%	1 S	-6.532	1.00	72 H
			5.80%	1 S	-6.532	1.00	85 H
			4.98%	1 S	-6.532	1.00	84 H
			4.75%	1 S	-6.532	1.00	88 H
			4.29%	1 S	-6.532	1.00	77 H
			3.89%	1 S	-6.532	1.00	58 H
			3.77%	1 S	-6.532	1.00	54 H
			-3.42%	2 P:z	4.578	0.00	28 C
			3.11%	2 S	2.394	0.00	81 H
			-2.83%	2 P:z	4.578	0.00	20 C
			2.82%	1 P:z	-5.263	0.67	20 C
			-2.70%	2 P:z	4.578	0.00	39 C
			-2.67%	2 P:x	4.578	0.00	20 C
			2.41%	1 P:y	-5.263	0.67	20 C
			2.35%	1 P:y	-5.263	0.67	36 C
			2.31%	2 S	2.394	0.00	88 H
			2.27%	1 S	-6.532	1.00	83 H
			-2.18%	2 P:y	4.578	0.00	20 C
			2.16%	1 S	-6.532	1.00	81 H
			2.03%	1 S	-6.532	1.00	50 H
			-2.00%	2 S	2.394	0.00	70 H
			1.97%	2 S	2.394	0.00	77 H
			1.96%	1 S	-6.532	1.00	63 H
			-1.94%	2 P:x	4.578	0.00	38 C
			1.83%	2 P:y	4.578	0.00	36 C
			1.68%	2 S	2.394	0.00	85 H
			-1.67%	2 P:y	4.578	0.00	13 C

			1.62%	1 S	-6.532	1.00	56 H
			1.59%	1 S	-6.532	1.00	92 H
			1.59%	1 P:z	-5.263	0.67	13 C
			1.55%	1 P:x	-5.263	0.67	38 C
			1.52%	1 P:x	-5.263	0.67	11 C
			1.49%	2 S	2.394	0.00	71 H
			1.41%	1 P:z	-5.263	0.67	39 C
			1.40%	1 S	-6.532	1.00	78 H
			-1.36%	2 S	2.394	0.00	72 H
			1.35%	1 P:y	-5.263	0.67	19 C
			-1.26%	2 S	2.394	0.00	76 H
			1.22%	1 S	-6.532	1.00	61 H
			1.20%	2 P:x	4.578	0.00	30 C
			1.16%	1 S	-6.532	1.00	52 H
			1.15%	2 S	2.394	0.00	58 H
			1.14%	1 P:x	-5.263	0.67	18 C
			1.13%	1 S	-6.532	1.00	95 H
			-1.12%	2 S	11.741	0.00	11 C
			1.07%	1 S	-6.532	1.00	71 H
			1.06%	1 P:z	-5.263	0.67	28 C
			1.04%	2 S	2.394	0.00	54 H
			1.03%	1 S	-6.532	1.00	69 H
			-1.03%	2 S	2.394	0.00	56 H
			1.03%	1 S	-6.532	1.00	59 H
2.588	0.00	169 A	9.30%	1 S	-6.532	1.00	78 H
			8.85%	1 S	-6.532	1.00	87 H
			-5.27%	2 P:z	4.578	0.00	28 C
			4.57%	1 S	-6.532	1.00	80 H
			4.18%	1 S	-6.532	1.00	72 H
			3.91%	1 S	-6.532	1.00	96 H
			3.88%	1 S	-6.532	1.00	74 H

3.54%	1 P:y	-5.263	0.67	26 C
3.37%	1 S	-6.532	1.00	63 H
3.30%	1 S	-6.532	1.00	75 H
2.95%	1 S	-6.532	1.00	57 H
2.92%	1 P:z	-5.263	0.67	28 C
2.80%	2 S	2.394	0.00	78 H
-2.78%	2 P:y	4.578	0.00	20 C
2.65%	1 S	-6.532	1.00	76 H
2.58%	1 S	-6.532	1.00	79 H
2.21%	1 S	-6.532	1.00	50 H
2.04%	1 S	-6.532	1.00	95 H
2.03%	1 S	-6.532	1.00	55 H
1.85%	1 S	-6.532	1.00	62 H
-1.83%	2 P:y	4.578	0.00	26 C
1.68%	1 S	-6.532	1.00	82 H
1.54%	2 S	2.394	0.00	87 H
1.52%	1 P:z	-5.263	0.67	29 C
1.51%	1 S	-6.532	1.00	59 H
1.49%	2 S	2.394	0.00	57 H
1.48%	1 P:y	-5.263	0.67	32 C
1.46%	2 S	2.394	0.00	72 H
1.45%	1 P:y	-5.263	0.67	40 C
-1.44%	2 P:z	4.578	0.00	37 C
1.39%	1 S	-6.532	1.00	56 H
-1.38%	2 P:x	4.578	0.00	29 C
-1.37%	2 S	2.394	0.00	74 H
-1.36%	2 P:y	4.578	0.00	40 C
1.30%	1 P:z	-5.263	0.67	36 C
1.29%	1 S	-6.532	1.00	54 H
-1.26%	2 P:z	4.578	0.00	30 C
1.25%	2 P:y	4.578	0.00	15 C

			1.22%	2 S	2.394	0.00	50 H
			1.21%	1 S	-6.532	1.00	77 H
			-1.21%	2 P:x	4.578	0.00	15 C
			-1.17%	2 P:z	4.578	0.00	36 C
			1.17%	2 S	2.394	0.00	81 H
			1.15%	1 P:x	-5.263	0.67	30 C
			-1.15%	2 S	2.394	0.00	56 H
			-1.14%	2 S	2.394	0.00	80 H
			1.13%	1 P:y	-5.263	0.67	20 C
			-1.06%	2 P:x	4.578	0.00	12 C
			-1.05%	2 S	2.394	0.00	77 H
			1.05%	1 P:x	-5.263	0.67	12 C
			1.03%	1 S	-6.532	1.00	68 H
			1.03%	2 S	2.394	0.00	79 H
			1.02%	1 S	-6.532	1.00	70 H
			1.01%	1 P:y	-5.263	0.67	12 C
			-1.01%	2 P:z	4.578	0.00	32 C
			-1.00%	2 P:z	4.578	0.00	39 C
2.640	0.00	170 A	20.63%	1 P:z	-5.263	0.67	29 C
			-6.68%	2 P:z	4.578	0.00	30 C
			6.62%	1 S	-6.532	1.00	55 H
			3.80%	1 P:z	-9.066	1.33	3 O
			3.68%	1 S	-6.532	1.00	48 H
			3.42%	2 S	2.394	0.00	73 H
			3.35%	1 S	-6.532	1.00	76 H
			3.29%	1 P:x	-5.263	0.67	39 C
			3.28%	1 S	-6.532	1.00	49 H
			2.76%	1 S	-6.532	1.00	70 H
			2.61%	1 S	-6.532	1.00	73 H
			2.51%	1 S	-6.532	1.00	79 H
			-2.29%	2 S	2.394	0.00	88 H

			2.26%	2 S	2.394	0.00	74 H
			2.12%	1 P:y	-5.263	0.67	13 C
			1.94%	2 S	2.394	0.00	55 H
			-1.75%	2 S	24.573	0.00	3 O
			1.65%	1 P:y	-5.263	0.67	30 C
			1.64%	1 P:x	-9.066	1.33	4 O
			1.64%	2 S	2.394	0.00	75 H
			1.58%	1 S	-23.822	2.00	3 O
			1.54%	1 P:z	-5.263	0.67	15 C
			1.50%	1 P:z	-5.263	0.67	26 C
			-1.49%	2 P:z	4.578	0.00	15 C
			1.47%	1 P:y	-5.263	0.67	12 C
			-1.42%	2 P:y	4.578	0.00	17 C
			-1.37%	2 P:z	9.420	0.00	3 O
			1.22%	2 S	2.394	0.00	76 H
			-1.12%	2 P:x	4.578	0.00	20 C
			1.10%	2 S	2.394	0.00	83 H
			1.10%	1 P:y	-5.263	0.67	39 C
			1.09%	2 P:y	4.578	0.00	38 C
			1.08%	1 S	-13.631	2.00	12 C
			1.08%	1 S	-6.532	1.00	52 H
			1.05%	1 P:y	-5.263	0.67	18 C
2.706	0.00	171 A	10.62%	1 S	-6.532	1.00	62 H
			-8.96%	2 P:z	4.578	0.00	37 C
			5.19%	1 P:y	-5.263	0.67	42 C
			4.37%	1 S	-6.532	1.00	89 H
			4.29%	2 S	2.394	0.00	62 H
			4.09%	1 S	-6.532	1.00	79 H
			3.99%	1 S	-6.532	1.00	91 H
			3.41%	2 P:z	4.578	0.00	40 C
			2.94%	1 S	-6.532	1.00	90 H

2.93%	1 S	-6.532	1.00	81 H
2.92%	1 S	-6.532	1.00	69 H
2.71%	1 S	-6.532	1.00	71 H
2.64%	1 S	-6.532	1.00	55 H
-2.52%	2 P:x	4.578	0.00	36 C
2.50%	1 S	-13.631	2.00	41 C
2.28%	1 P:z	-5.263	0.67	37 C
2.25%	1 S	-6.532	1.00	93 H
-2.18%	2 S	2.394	0.00	91 H
2.12%	1 S	-6.532	1.00	52 H
2.05%	1 S	-6.532	1.00	48 H
2.04%	1 S	-6.532	1.00	54 H
2.01%	1 S	-6.532	1.00	64 H
2.01%	1 S	-6.532	1.00	94 H
1.93%	1 S	-6.532	1.00	65 H
1.92%	1 S	-6.532	1.00	84 H
1.87%	1 S	-6.532	1.00	56 H
1.87%	1 S	-6.532	1.00	95 H
-1.87%	2 P:y	4.578	0.00	40 C
-1.84%	2 P:x	4.578	0.00	42 C
1.76%	1 S	-6.532	1.00	61 H
-1.74%	2 P:z	4.578	0.00	26 C
1.56%	2 P:y	4.578	0.00	42 C
1.51%	1 P:z	-5.263	0.67	32 C
1.46%	1 S	-6.532	1.00	44 H
1.42%	1 S	-6.532	1.00	85 H
-1.31%	2 P:x	4.578	0.00	17 C
1.30%	1 S	-6.532	1.00	47 H
1.27%	2 S	2.394	0.00	79 H
-1.27%	2 P:x	4.578	0.00	26 C
1.23%	1 P:z	-5.263	0.67	40 C

			-1.23%	2 S	11.741	0.00	41 C
			1.21%	1 P:x	-5.263	0.67	42 C
			-1.20%	2 P:x	4.578	0.00	31 C
			-1.16%	2 P:z	4.578	0.00	32 C
			1.15%	2 S	2.394	0.00	93 H
			1.12%	2 S	2.394	0.00	81 H
			1.09%	1 P:z	-5.263	0.67	15 C
			1.06%	1 P:x	-5.263	0.67	36 C
			1.04%	2 P:z	4.578	0.00	42 C
			1.03%	1 P:x	-5.263	0.67	27 C
			1.01%	1 S	-6.532	1.00	49 H
2.720	0.00	172 A	7.56%	1 S	-6.532	1.00	72 H
			5.56%	1 S	-6.532	1.00	79 H
			3.74%	1 S	-6.532	1.00	90 H
			3.69%	1 S	-6.532	1.00	61 H
			3.64%	1 S	-6.532	1.00	69 H
			3.44%	1 P:x	-5.263	0.67	24 C
			3.35%	1 S	-6.532	1.00	95 H
			3.20%	1 S	-6.532	1.00	53 H
			2.66%	1 S	-6.532	1.00	48 H
			2.55%	1 P:z	-5.263	0.67	26 C
			2.53%	1 P:y	-5.263	0.67	37 C
			2.44%	1 S	-6.532	1.00	54 H
			2.35%	1 P:z	-5.263	0.67	40 C
			2.31%	1 S	-6.532	1.00	49 H
			2.18%	1 S	-6.532	1.00	63 H
			-2.16%	2 P:z	4.578	0.00	37 C
			-2.10%	2 P:z	4.578	0.00	15 C
			-2.07%	2 P:z	4.578	0.00	26 C
			2.06%	2 S	2.394	0.00	58 H
			2.01%	2 P:z	4.578	0.00	14 C

2.00%	1 P:x	-5.263	0.67	32 C
1.99%	1 P:y	-5.263	0.67	42 C
1.93%	1 S	-6.532	1.00	71 H
-1.92%	2 P:y	4.578	0.00	20 C
-1.80%	2 S	2.394	0.00	81 H
1.80%	1 S	-6.532	1.00	45 H
1.77%	1 S	-6.532	1.00	58 H
1.74%	1 P:x	-5.263	0.67	20 C
1.74%	1 S	-6.532	1.00	64 H
1.66%	2 S	2.394	0.00	66 H
1.65%	2 S	2.394	0.00	45 H
1.60%	1 S	-6.532	1.00	81 H
1.55%	2 P:y	4.578	0.00	42 C
1.50%	2 S	2.394	0.00	79 H
-1.42%	2 S	2.394	0.00	61 H
1.40%	1 S	-6.532	1.00	55 H
1.35%	1 S	-6.532	1.00	68 H
1.33%	1 P:y	-5.263	0.67	20 C
-1.33%	2 P:z	4.578	0.00	34 C
1.31%	1 P:x	-5.263	0.67	19 C
-1.25%	2 S	2.394	0.00	55 H
1.24%	1 S	-6.532	1.00	88 H
-1.23%	2 P:x	4.578	0.00	32 C
1.16%	2 P:x	4.578	0.00	41 C
1.13%	2 S	2.394	0.00	95 H
-1.12%	2 S	2.394	0.00	68 H
-1.10%	2 P:z	4.578	0.00	40 C
1.10%	2 P:x	4.578	0.00	27 C
-1.08%	2 S	2.394	0.00	71 H
1.06%	1 P:x	-5.263	0.67	36 C
1.02%	1 S	-6.532	1.00	94 H

			-1.01%	2 P:y	4.578	0.00	17 C	
			1.00%	1 S	-6.532	1.00	85 H	
2.815	0.00	173 A	7.76%	1 S	-6.532	1.00	45 H	
			-7.56%	2 P:z	4.578	0.00	34 C	
			5.83%	1 S	-6.532	1.00	63 H	
			5.79%	1 S	-6.532	1.00	46 H	
			5.46%	1 S	-6.532	1.00	47 H	
			4.20%	1 P:z	-5.263	0.67	34 C	
			3.54%	1 S	-6.532	1.00	79 H	
			3.30%	1 S	-6.532	1.00	56 H	
			2.89%	2 P:z	4.578	0.00	21 C	
			2.76%	2 S	2.394	0.00	45 H	
			2.73%	2 S	2.394	0.00	46 H	
			2.42%	1 P:y	-5.263	0.67	40 C	
			2.41%	1 P:z	-5.263	0.67	27 C	
			2.40%	1 S	-6.532	1.00	53 H	
			2.30%	1 P:z	-5.263	0.67	21 C	
			2.15%	1 S	-6.532	1.00	54 H	
			1.94%	2 P:x	4.578	0.00	40 C	
			1.88%	1 P:z	-5.263	0.67	26 C	
			-1.82%	2 S	2.394	0.00	65 H	
			1.77%	1 S	-6.532	1.00	90 H	
			1.69%	1 S	-6.532	1.00	69 H	
			-1.66%	2 P:z	4.578	0.00	36 C	
			1.65%	1 P:x	-5.263	0.67	21 C	
			1.58%	1 S	-6.532	1.00	59 H	
			1.53%	1 P:x	-5.263	0.67	39 C	
			1.49%	1 S	-6.532	1.00	87 H	
			1.47%	1 S	-6.532	1.00	75 H	
			1.45%	1 S	-6.532	1.00	82 H	
			-1.40%	2 P:z	4.578	0.00	40 C	

			1.35%	1 S	-6.532	1.00	68 H	
			-1.30%	2 P:z	4.578	0.00	26 C	
			1.29%	2 P:x	4.578	0.00	27 C	
			1.29%	1 S	-6.532	1.00	96 H	
			1.26%	1 S	-6.532	1.00	78 H	
			-1.18%	2 P:z	4.578	0.00	38 C	
			-1.17%	2 P:x	4.578	0.00	25 C	
			1.16%	2 S	2.394	0.00	63 H	
			1.15%	1 S	-6.532	1.00	95 H	
			1.14%	1 P:z	-5.263	0.67	23 C	
			1.14%	1 S	-6.532	1.00	65 H	
			-1.06%	2 S	11.741	0.00	23 C	
			-1.04%	2 P:x	4.578	0.00	21 C	
			1.03%	2 P:x	4.578	0.00	33 C	
2.949	0.00	174 A	7.40%	1 S	-6.532	1.00	59 H	
			6.22%	1 S	-6.532	1.00	66 H	
			6.19%	1 S	-6.532	1.00	56 H	
			5.46%	1 S	-6.532	1.00	94 H	
			5.18%	1 S	-6.532	1.00	82 H	
			3.78%	2 P:z	4.578	0.00	21 C	
			3.19%	1 P:z	-5.263	0.67	23 C	
			3.18%	1 S	-6.532	1.00	57 H	
			3.06%	1 S	-6.532	1.00	44 H	
			-2.96%	2 P:y	4.578	0.00	21 C	
			2.85%	1 P:y	-5.263	0.67	21 C	
			-2.73%	2 P:z	4.578	0.00	38 C	
			2.71%	1 S	-6.532	1.00	62 H	
			2.44%	1 S	-6.532	1.00	84 H	
			-2.35%	2 P:y	4.578	0.00	39 C	
			2.23%	1 S	-6.532	1.00	81 H	
			2.11%	1 S	-6.532	1.00	55 H	

			2.10%	1 S	-6.532	1.00	75 H
			2.03%	1 S	-6.532	1.00	68 H
			2.02%	2 P:x	4.578	0.00	40 C
			1.84%	1 S	-6.532	1.00	87 H
			-1.75%	2 P:x	4.578	0.00	36 C
			1.67%	1 S	-6.532	1.00	47 H
			1.53%	1 P:z	-5.263	0.67	21 C
			1.50%	1 S	-6.532	1.00	54 H
			1.40%	1 P:z	-5.263	0.67	38 C
			1.39%	1 S	-6.532	1.00	46 H
			-1.38%	2 S	2.394	0.00	68 H
			1.34%	1 S	-6.532	1.00	45 H
			-1.32%	2 P:z	4.578	0.00	34 C
			-1.31%	2 P:x	4.578	0.00	39 C
			1.29%	2 P:x	4.578	0.00	37 C
			-1.28%	2 S	11.741	0.00	32 C
			1.28%	1 P:y	-5.263	0.67	39 C
			-1.25%	2 S	2.394	0.00	96 H
			1.22%	1 P:y	-5.263	0.67	36 C
			-1.16%	2 S	2.394	0.00	56 H
			-1.13%	2 S	11.741	0.00	23 C
			-1.12%	2 S	2.394	0.00	81 H
			1.12%	2 S	2.394	0.00	54 H
			1.09%	1 S	-6.532	1.00	79 H
			1.09%	1 P:x	-5.263	0.67	32 C
			1.06%	1 S	-6.532	1.00	48 H
			1.06%	2 S	2.394	0.00	67 H
			1.05%	2 S	2.394	0.00	82 H
			1.04%	1 S	-6.532	1.00	91 H
3.024	0.00	175 A	20.90%	1 S	-6.532	1.00	66 H
			11.16%	1 S	-6.532	1.00	68 H

-10.54%	2 P:y	4.578	0.00	21 C
9.37%	1 S	-6.532	1.00	61 H
-4.03%	2 P:x	4.578	0.00	21 C
3.98%	1 P:y	-5.263	0.67	21 C
-3.59%	2 P:z	4.578	0.00	37 C
3.22%	1 S	-6.532	1.00	60 H
3.18%	1 P:x	-5.263	0.67	21 C
3.15%	1 S	-6.532	1.00	47 H
3.05%	2 P:x	4.578	0.00	40 C
2.96%	2 S	2.394	0.00	66 H
2.83%	2 P:z	4.578	0.00	40 C
2.09%	1 S	-6.532	1.00	92 H
-1.91%	2 S	11.741	0.00	37 C
1.87%	1 S	-6.532	1.00	96 H
1.82%	3 S	40.877	0.00	66 H
-1.68%	2 S	2.394	0.00	68 H
1.67%	1 S	-6.532	1.00	46 H
1.63%	1 S	-6.532	1.00	95 H
1.62%	1 S	-6.532	1.00	80 H
1.58%	1 S	-13.631	2.00	37 C
1.54%	1 S	-6.532	1.00	81 H
1.49%	1 S	-6.532	1.00	82 H
-1.45%	2 P:y	4.578	0.00	34 C
1.44%	1 P:y	-5.263	0.67	15 C
1.36%	1 S	-6.532	1.00	84 H
1.33%	3 S	40.877	0.00	61 H
1.33%	2 P:z	4.578	0.00	34 C
1.21%	2 S	2.394	0.00	63 H
1.20%	1 S	-13.631	2.00	33 C
1.19%	1 S	-6.532	1.00	52 H
1.19%	2 S	2.394	0.00	45 H

			-1.13%	2 P:y	4.578	0.00	37 C
			1.09%	2 P:y	4.578	0.00	15 C
3.071	0.00	176 A	8.59%	1 S	-6.532	1.00	56 H
			5.54%	1 S	-6.532	1.00	90 H
			5.47%	1 S	-6.532	1.00	95 H
			5.21%	1 S	-6.532	1.00	58 H
			4.40%	1 P:y	-5.263	0.67	17 C
			-3.97%	2 P:y	4.578	0.00	17 C
			3.57%	1 S	-6.532	1.00	63 H
			3.25%	1 S	-6.532	1.00	59 H
			3.24%	1 S	-6.532	1.00	48 H
			-2.89%	2 P:z	4.578	0.00	39 C
			2.73%	1 P:x	-5.263	0.67	32 C
			2.68%	1 P:z	-5.263	0.67	15 C
			-2.64%	2 S	2.394	0.00	59 H
			2.53%	1 P:x	-5.263	0.67	24 C
			-2.45%	2 S	2.394	0.00	49 H
			2.44%	1 S	-6.532	1.00	93 H
			2.31%	2 S	2.394	0.00	58 H
			2.18%	1 P:y	-5.263	0.67	36 C
			2.12%	1 S	-6.532	1.00	92 H
			2.03%	1 S	-6.532	1.00	79 H
			1.99%	2 S	2.394	0.00	90 H
			1.99%	1 P:z	-5.263	0.67	42 C
			1.99%	1 S	-6.532	1.00	49 H
			1.97%	1 P:z	-5.263	0.67	39 C
			1.93%	1 P:z	-5.263	0.67	40 C
			1.89%	1 S	-6.532	1.00	52 H
			1.85%	1 S	-6.532	1.00	72 H
			1.72%	1 S	-6.532	1.00	54 H
			1.60%	2 S	2.394	0.00	71 H

			-1.60%	2 P:z	4.578	0.00	17 C
			1.54%	1 S	-6.532	1.00	64 H
			1.48%	1 S	-6.532	1.00	82 H
			-1.45%	2 P:z	4.578	0.00	36 C
			1.43%	1 S	-13.631	2.00	18 C
			1.42%	1 P:x	-5.263	0.67	18 C
			-1.41%	2 P:x	4.578	0.00	32 C
			-1.36%	2 P:y	4.578	0.00	36 C
			1.31%	1 S	-13.631	2.00	14 C
			1.29%	1 S	-6.532	1.00	50 H
			1.27%	1 P:x	-5.263	0.67	27 C
			1.21%	1 P:z	-5.263	0.67	19 C
			-1.21%	2 S	2.394	0.00	54 H
			1.21%	1 S	-13.631	2.00	19 C
			1.20%	2 P:z	4.578	0.00	32 C
			1.20%	2 P:y	4.578	0.00	42 C
			-1.19%	2 S	2.394	0.00	64 H
			-1.19%	2 P:z	4.578	0.00	40 C
			1.08%	1 P:z	-5.263	0.67	26 C
			1.05%	2 P:y	4.578	0.00	30 C
			1.00%	1 P:x	-9.066	1.33	1 O
3.106	0.00	177 A	7.85%	1 S	-6.532	1.00	80 H
			6.95%	1 S	-6.532	1.00	77 H
			6.77%	1 P:y	-5.263	0.67	30 C
			4.89%	1 S	-6.532	1.00	58 H
			4.54%	1 S	-6.532	1.00	83 H
			-4.32%	2 P:y	4.578	0.00	29 C
			4.10%	1 S	-6.532	1.00	78 H
			3.80%	2 P:x	4.578	0.00	30 C
			3.52%	1 P:y	-5.263	0.67	29 C
			3.40%	2 S	2.394	0.00	80 H

3.40%	1 S	-6.532	1.00	60 H
3.39%	1 S	-6.532	1.00	81 H
-3.24%	2 P:y	4.578	0.00	26 C
3.13%	1 S	-6.532	1.00	74 H
-3.13%	2 P:z	4.578	0.00	28 C
3.12%	1 S	-6.532	1.00	67 H
2.93%	1 S	-6.532	1.00	46 H
2.87%	1 P:z	-5.263	0.67	28 C
2.71%	1 P:z	-5.263	0.67	21 C
2.45%	2 P:y	4.578	0.00	30 C
-2.45%	2 S	2.394	0.00	81 H
2.19%	1 P:x	-5.263	0.67	30 C
2.00%	1 S	-6.532	1.00	53 H
-1.97%	2 P:x	4.578	0.00	38 C
-1.91%	2 S	2.394	0.00	78 H
-1.89%	2 S	2.394	0.00	77 H
1.85%	1 S	-6.532	1.00	55 H
-1.81%	2 S	11.741	0.00	29 C
1.69%	2 P:y	4.578	0.00	28 C
1.63%	1 S	-6.532	1.00	84 H
1.53%	1 S	-6.532	1.00	93 H
1.52%	1 S	-6.532	1.00	70 H
1.46%	1 S	-6.532	1.00	52 H
1.46%	1 P:y	-5.263	0.67	12 C
1.43%	1 P:x	-5.263	0.67	13 C
1.39%	1 P:z	-5.263	0.67	30 C
1.39%	1 P:x	-5.263	0.67	12 C
1.38%	1 S	-6.532	1.00	96 H
1.37%	1 S	-13.631	2.00	29 C
1.33%	1 P:z	-5.263	0.67	11 C
1.31%	1 S	-6.532	1.00	72 H

			1.27%	1 P:x	-5.263	0.67	38 C
			1.26%	1 P:x	-5.263	0.67	19 C
			-1.26%	2 S	2.394	0.00	67 H
			1.25%	1 P:x	-5.263	0.67	28 C
			1.22%	2 S	2.394	0.00	46 H
			1.22%	1 P:z	-5.263	0.67	39 C
			1.18%	1 P:y	-5.263	0.67	27 C
			1.16%	1 P:x	-5.263	0.67	26 C
			-1.15%	2 P:x	4.578	0.00	23 C
			-1.14%	2 P:z	4.578	0.00	39 C
			-1.12%	2 P:y	4.578	0.00	39 C
			1.11%	1 P:y	-5.263	0.67	36 C
			-1.11%	2 P:y	4.578	0.00	27 C
			1.11%	1 S	-6.532	1.00	57 H
			1.01%	1 P:y	-5.263	0.67	13 C
			-1.01%	2 P:x	4.578	0.00	26 C
3.153	0.00	178 A	7.44%	1 P:z	-5.263	0.67	21 C
			5.76%	1 P:y	-5.263	0.67	37 C
			4.38%	1 P:x	-5.263	0.67	23 C
			4.17%	1 S	-6.532	1.00	82 H
			3.71%	1 P:x	-5.263	0.67	40 C
			3.41%	1 S	-6.532	1.00	81 H
			3.31%	1 S	-6.532	1.00	84 H
			3.15%	2 P:x	4.578	0.00	32 C
			3.13%	1 S	-6.532	1.00	79 H
			3.12%	1 S	-6.532	1.00	59 H
			3.07%	1 S	-6.532	1.00	71 H
			3.03%	1 S	-6.532	1.00	62 H
			2.84%	1 P:y	-5.263	0.67	23 C
			2.53%	1 P:x	-5.263	0.67	32 C
			-2.47%	2 P:y	4.578	0.00	17 C

2.42%	1 S	-13.631	2.00	23 C
2.38%	1 P:y	-5.263	0.67	33 C
2.15%	1 P:y	-5.263	0.67	17 C
2.08%	1 S	-6.532	1.00	56 H
-2.05%	2 S	11.741	0.00	23 C
2.04%	1 S	-6.532	1.00	66 H
-1.99%	2 P:x	4.578	0.00	37 C
-1.95%	2 P:x	4.578	0.00	23 C
-1.88%	2 P:z	4.578	0.00	38 C
1.78%	1 P:z	-5.263	0.67	32 C
-1.72%	2 P:x	4.578	0.00	20 C
1.71%	1 P:y	-5.263	0.67	21 C
-1.67%	2 P:y	4.578	0.00	37 C
-1.62%	2 P:z	4.578	0.00	37 C
1.59%	1 S	-6.532	1.00	63 H
1.56%	1 S	-6.532	1.00	60 H
1.49%	2 P:z	4.578	0.00	21 C
-1.48%	2 P:x	4.578	0.00	38 C
1.48%	1 P:z	-9.066	1.33	2 O
1.48%	2 S	2.394	0.00	62 H
1.47%	1 S	-6.532	1.00	50 H
1.46%	2 P:x	4.578	0.00	40 C
1.44%	1 P:z	-5.263	0.67	38 C
1.43%	1 S	-6.532	1.00	87 H
1.41%	1 P:x	-5.263	0.67	37 C
1.39%	1 P:y	-5.263	0.67	40 C
1.35%	1 P:z	-5.263	0.67	40 C
1.23%	1 S	-6.532	1.00	67 H
1.21%	1 S	-6.532	1.00	92 H
-1.19%	2 S	2.394	0.00	60 H
1.18%	1 P:x	-5.263	0.67	20 C

			1.16%	1 S	-13.631	2.00	14 C
			1.14%	1 P:x	-5.263	0.67	26 C
			1.14%	1 S	-6.532	1.00	49 H
			-1.13%	2 P:z	4.578	0.00	26 C
			1.11%	1 S	-6.532	1.00	65 H
			1.09%	1 S	-13.631	2.00	37 C
			-1.06%	2 S	2.394	0.00	81 H
			-1.03%	2 S	2.394	0.00	66 H
			-1.02%	2 S	2.394	0.00	96 H
3.194	0.00	179 A	7.13%	1 S	-6.532	1.00	58 H
			5.89%	1 S	-6.532	1.00	46 H
			5.29%	1 S	-6.532	1.00	94 H
			4.72%	1 S	-6.532	1.00	83 H
			3.92%	1 S	-6.532	1.00	88 H
			3.45%	1 S	-6.532	1.00	48 H
			3.42%	1 S	-6.532	1.00	78 H
			2.90%	1 S	-6.532	1.00	67 H
			-2.65%	2 P:z	4.578	0.00	39 C
			2.62%	1 S	-6.532	1.00	54 H
			2.56%	1 P:z	-5.263	0.67	18 C
			2.47%	2 S	2.394	0.00	46 H
			2.41%	2 P:y	4.578	0.00	30 C
			2.35%	1 P:y	-5.263	0.67	38 C
			2.26%	1 P:z	-5.263	0.67	20 C
			2.20%	1 S	-6.532	1.00	68 H
			2.19%	1 P:z	-5.263	0.67	39 C
			2.15%	1 P:z	-5.263	0.67	28 C
			-2.14%	2 P:x	4.578	0.00	13 C
			2.08%	1 S	-6.532	1.00	44 H
			-2.05%	2 S	2.394	0.00	67 H
			1.87%	1 S	-13.631	2.00	13 C

1.82%	1 S	-6.532	1.00	57 H
1.70%	1 P:y	-5.263	0.67	28 C
1.67%	1 S	-6.532	1.00	86 H
-1.65%	2 S	2.394	0.00	92 H
-1.57%	2 P:y	4.578	0.00	34 C
-1.57%	2 S	2.394	0.00	78 H
1.54%	1 P:y	-5.263	0.67	30 C
1.54%	1 S	-6.532	1.00	63 H
1.53%	1 S	-6.532	1.00	59 H
-1.51%	2 P:z	4.578	0.00	18 C
1.49%	1 S	-6.532	1.00	95 H
1.49%	1 P:z	-5.263	0.67	21 C
1.48%	1 P:z	-5.263	0.67	26 C
1.44%	2 P:z	4.578	0.00	26 C
-1.42%	2 S	11.741	0.00	13 C
-1.41%	2 S	2.394	0.00	94 H
1.41%	2 P:y	4.578	0.00	28 C
1.40%	1 S	-6.532	1.00	79 H
1.39%	1 S	-6.532	1.00	55 H
1.36%	1 P:y	-5.263	0.67	36 C
1.35%	1 P:x	-5.263	0.67	32 C
1.31%	1 S	-13.631	2.00	19 C
-1.23%	2 P:x	4.578	0.00	15 C
1.21%	1 P:y	-5.263	0.67	15 C
-1.19%	2 P:x	4.578	0.00	36 C
1.15%	1 P:z	-5.263	0.67	19 C
1.14%	1 P:z	-5.263	0.67	42 C
1.13%	1 S	-6.532	1.00	71 H
1.12%	1 P:y	-5.263	0.67	42 C
1.12%	1 P:z	-5.263	0.67	12 C
1.06%	1 P:z	-5.263	0.67	32 C

			-1.06%	2 P:y	4.578	0.00	29 C		
			-1.06%	2 P:x	7.645	0.00	43 N		
			1.05%	1 P:x	-5.263	0.67	19 C		
			1.05%	1 S	-6.532	1.00	92 H		
			-1.04%	2 S	2.394	0.00	95 H		
			-1.04%	2 P:x	4.578	0.00	21 C		
			-1.02%	2 P:z	4.578	0.00	28 C		
			1.01%	1 S	-6.532	1.00	91 H		
3.246	0.00	180 A	16.63%	1 S	-6.532	1.00	71 H		
			4.33%	1 S	-6.532	1.00	58 H		
			3.88%	1 S	-6.532	1.00	45 H		
			3.74%	1 S	-6.532	1.00	53 H		
			-3.67%	2 P:x	4.578	0.00	20 C		
			3.54%	1 S	-6.532	1.00	56 H		
			3.45%	1 S	-6.532	1.00	79 H		
			3.20%	1 P:x	-5.263	0.67	24 C		
			3.04%	1 P:x	-5.263	0.67	20 C		
			2.89%	2 P:x	4.578	0.00	42 C		
			2.76%	2 P:z	4.578	0.00	21 C		
			-2.75%	2 P:z	4.578	0.00	36 C		
			2.72%	1 S	-6.532	1.00	81 H		
			2.71%	2 P:y	4.578	0.00	30 C		
			2.68%	1 S	-6.532	1.00	84 H		
			-2.65%	2 S	2.394	0.00	72 H		
			2.47%	1 S	-6.532	1.00	72 H		
			2.40%	1 S	-6.532	1.00	46 H		
			2.23%	1 S	-13.631	2.00	41 C		
			2.07%	1 S	-6.532	1.00	54 H		
			2.05%	1 P:z	-5.263	0.67	21 C		
			2.00%	2 S	2.394	0.00	91 H		
			2.00%	1 P:y	-5.263	0.67	42 C		

			-1.97%	2 S	2.394	0.00	81 H		
			1.94%	1 S	-6.532	1.00	86 H		
			1.75%	1 P:y	-5.263	0.67	17 C		
			1.66%	1 P:z	-5.263	0.67	36 C		
			-1.58%	2 P:y	4.578	0.00	34 C		
			-1.51%	2 P:z	4.578	0.00	34 C		
			1.48%	1 S	-6.532	1.00	89 H		
			-1.46%	2 P:z	4.578	0.00	39 C		
			1.45%	1 S	-13.631	2.00	14 C		
			1.42%	1 S	-6.532	1.00	87 H		
			1.42%	1 S	-6.532	1.00	69 H		
			-1.39%	2 S	11.741	0.00	14 C		
			-1.24%	2 P:x	4.578	0.00	24 C		
			1.20%	1 S	-6.532	1.00	48 H		
			-1.19%	2 S	2.394	0.00	56 H		
			1.17%	1 P:z	-5.263	0.67	39 C		
			-1.16%	2 P:x	4.578	0.00	38 C		
			1.14%	1 P:x	-5.263	0.67	38 C		
			1.09%	2 P:z	4.578	0.00	38 C		
			1.06%	1 P:x	-5.263	0.67	34 C		
			1.02%	2 S	2.394	0.00	53 H		
3.272	0.00	181 A	9.63%	1 P:x	-5.263	0.67	23 C		
			5.88%	2 P:y	4.578	0.00	21 C		
			-5.25%	2 P:x	4.578	0.00	23 C		
			4.74%	1 P:y	-5.263	0.67	21 C		
			4.54%	1 P:z	-5.263	0.67	21 C		
			4.51%	1 S	-6.532	1.00	82 H		
			3.65%	1 S	-6.532	1.00	48 H		
			3.51%	1 S	-6.532	1.00	89 H		
			3.21%	1 P:y	-5.263	0.67	17 C		
			3.13%	2 P:x	4.578	0.00	34 C		

2.72%	1 S	-6.532	1.00	49 H
2.69%	1 S	-6.532	1.00	52 H
-2.68%	2 P:y	4.578	0.00	17 C
2.55%	1 P:x	-5.263	0.67	33 C
-2.47%	2 P:z	4.578	0.00	38 C
-2.32%	2 P:z	4.578	0.00	23 C
2.27%	1 S	-6.532	1.00	79 H
2.17%	2 S	2.394	0.00	44 H
2.14%	2 P:y	4.578	0.00	40 C
1.97%	2 S	2.394	0.00	45 H
1.83%	2 P:y	4.578	0.00	35 C
1.81%	1 S	-6.532	1.00	66 H
1.77%	1 S	-6.532	1.00	45 H
1.69%	1 S	-6.532	1.00	91 H
1.69%	1 P:x	-5.263	0.67	32 C
1.68%	1 P:z	-5.263	0.67	38 C
1.67%	1 S	-6.532	1.00	93 H
-1.67%	2 P:x	4.578	0.00	42 C
-1.64%	2 S	2.394	0.00	60 H
1.55%	1 S	-13.631	2.00	14 C
1.54%	1 P:y	-5.263	0.67	30 C
-1.53%	2 S	2.394	0.00	66 H
1.48%	1 S	-6.532	1.00	64 H
1.44%	1 P:z	-5.263	0.67	15 C
1.44%	2 S	2.394	0.00	67 H
-1.44%	2 S	2.394	0.00	49 H
-1.32%	2 P:x	4.578	0.00	25 C
1.31%	2 P:x	4.578	0.00	32 C
1.30%	1 S	-6.532	1.00	51 H
1.30%	1 P:y	-5.263	0.67	36 C
1.16%	2 P:z	4.578	0.00	35 C

			1.13%	1	P:x	-5.263	0.67	42	C
			1.09%	2	P:y	4.578	0.00	30	C
			-1.08%	2	P:x	4.578	0.00	35	C
			1.08%	1	S	-6.532	1.00	46	H
			1.04%	1	S	-6.532	1.00	83	H
3.321	0.00	182 A	9.00%	1	P:y	-5.263	0.67	20	C
			6.62%	1	S	-6.532	1.00	72	H
			6.45%	1	S	-6.532	1.00	52	H
			5.14%	1	S	-6.532	1.00	85	H
			4.75%	1	S	-6.532	1.00	87	H
			3.79%	1	S	-6.532	1.00	81	H
			3.68%	1	P:z	-5.263	0.67	24	C
			3.47%	1	S	-6.532	1.00	45	H
			-2.79%	2	P:z	4.578	0.00	18	C
			-2.53%	2	P:z	4.578	0.00	24	C
			2.38%	1	S	-6.532	1.00	58	H
			2.31%	1	S	-6.532	1.00	83	H
			2.23%	1	P:x	-5.263	0.67	26	C
			2.08%	1	P:z	-5.263	0.67	18	C
			-2.01%	2	S	2.394	0.00	81	H
			1.89%	1	P:y	-5.263	0.67	15	C
			-1.87%	2	S	2.394	0.00	72	H
			1.86%	2	P:y	4.578	0.00	15	C
			1.85%	1	P:z	-5.263	0.67	20	C
			1.82%	1	S	-6.532	1.00	44	H
			1.68%	1	S	-6.532	1.00	66	H
			1.66%	1	S	-6.532	1.00	50	H
			1.66%	1	P:x	-5.263	0.67	18	C
			-1.56%	2	P:x	4.578	0.00	27	C
			1.55%	1	S	-6.532	1.00	60	H
			-1.51%	2	P:z	4.578	0.00	34	C

			1.50%	1 S	-13.631	2.00	11 C
			1.45%	1 S	-6.532	1.00	86 H
			-1.42%	2 P:z	4.578	0.00	13 C
			1.39%	1 P:z	-5.263	0.67	11 C
			1.38%	1 P:z	-5.263	0.67	39 C
			1.36%	1 P:x	-5.263	0.67	27 C
			1.34%	1 P:z	-5.263	0.67	19 C
			1.32%	1 S	-6.532	1.00	96 H
			1.31%	1 S	-6.532	1.00	88 H
			1.22%	1 S	-6.532	1.00	84 H
			-1.22%	2 S	2.394	0.00	44 H
			-1.21%	2 S	11.741	0.00	24 C
			1.20%	1 P:y	-9.066	1.33	3 O
			1.17%	1 P:x	-5.263	0.67	32 C
			-1.16%	2 P:z	4.578	0.00	19 C
			-1.15%	2 P:z	4.578	0.00	11 C
			1.15%	2 P:x	4.578	0.00	17 C
			1.10%	2 P:y	4.578	0.00	30 C
			1.07%	1 S	-13.631	2.00	24 C
			-1.03%	2 P:y	4.578	0.00	21 C
			1.02%	1 P:x	-9.066	1.33	1 O
3.383	0.00	183 A	6.92%	1 S	-6.532	1.00	54 H
			6.44%	1 S	-6.532	1.00	46 H
			6.11%	1 S	-6.532	1.00	55 H
			5.75%	1 S	-6.532	1.00	69 H
			-4.13%	2 P:x	4.578	0.00	36 C
			3.36%	1 S	-6.532	1.00	93 H
			3.34%	1 P:x	-5.263	0.67	40 C
			2.83%	1 S	-6.532	1.00	68 H
			-2.81%	2 S	2.394	0.00	55 H
			2.61%	1 P:x	-5.263	0.67	36 C

2.42%	1 P:y	-5.263	0.67	37 C
2.39%	1 S	-6.532	1.00	87 H
2.34%	1 S	-6.532	1.00	58 H
2.31%	1 S	-6.532	1.00	75 H
2.23%	1 S	-6.532	1.00	88 H
2.13%	1 P:x	-5.263	0.67	21 C
2.06%	1 P:x	-5.263	0.67	26 C
-1.96%	2 P:y	4.578	0.00	34 C
1.92%	1 S	-6.532	1.00	56 H
1.90%	1 P:x	-5.263	0.67	30 C
1.89%	1 P:x	-5.263	0.67	24 C
1.88%	1 P:y	-5.263	0.67	13 C
1.87%	1 S	-6.532	1.00	73 H
1.84%	1 S	-6.532	1.00	82 H
-1.83%	2 P:x	4.578	0.00	37 C
1.83%	1 S	-6.532	1.00	84 H
1.77%	1 P:y	-5.263	0.67	33 C
1.77%	1 S	-6.532	1.00	95 H
1.71%	1 P:y	-5.263	0.67	34 C
1.62%	1 S	-6.532	1.00	61 H
1.60%	1 S	-6.532	1.00	51 H
-1.57%	2 S	2.394	0.00	88 H
1.53%	1 P:x	-5.263	0.67	25 C
-1.46%	2 S	2.394	0.00	75 H
1.46%	1 P:z	-5.263	0.67	12 C
-1.45%	2 P:z	4.578	0.00	24 C
1.43%	2 P:x	4.578	0.00	40 C
1.43%	1 P:z	-5.263	0.67	29 C
1.36%	1 S	-6.532	1.00	81 H
-1.35%	2 P:y	4.578	0.00	37 C
1.30%	1 P:x	-5.263	0.67	37 C

			1.30%	1 P:y	-5.263	0.67	28 C		
			1.26%	2 P:x	4.578	0.00	31 C		
			-1.22%	2 P:x	4.578	0.00	38 C		
			-1.21%	2 S	2.394	0.00	68 H		
			1.19%	2 P:x	4.578	0.00	22 C		
			1.15%	1 S	-6.532	1.00	45 H		
			1.14%	1 P:x	-5.263	0.67	19 C		
			-1.13%	2 S	2.394	0.00	45 H		
			1.10%	2 S	2.394	0.00	70 H		
			1.09%	2 P:z	4.578	0.00	14 C		
			1.08%	1 P:z	-5.263	0.67	11 C		
			1.06%	1 P:z	-5.263	0.67	42 C		
			1.05%	1 P:z	-5.263	0.67	38 C		
			-1.04%	2 S	2.394	0.00	92 H		
			-1.03%	2 P:x	4.578	0.00	14 C		
			-1.00%	2 P:y	4.578	0.00	13 C		
3.472	0.00	184 A	14.17%	1 S	-6.532	1.00	94 H		
			8.96%	1 S	-6.532	1.00	50 H		
			6.84%	1 S	-6.532	1.00	57 H		
			6.52%	1 S	-6.532	1.00	51 H		
			-3.89%	2 P:x	4.578	0.00	25 C		
			3.79%	1 P:z	-5.263	0.67	29 C		
			3.66%	2 S	2.394	0.00	54 H		
			3.45%	1 P:y	-5.263	0.67	39 C		
			3.32%	1 P:z	-5.263	0.67	30 C		
			3.16%	1 P:x	-5.263	0.67	25 C		
			-2.70%	2 S	2.394	0.00	94 H		
			2.59%	1 P:y	-5.263	0.67	32 C		
			-2.28%	2 P:y	4.578	0.00	32 C		
			2.14%	2 P:y	4.578	0.00	15 C		
			2.02%	1 S	-6.532	1.00	64 H		

			1.91%	2 P:z	4.578	0.00	21 C
			1.84%	1 P:z	-5.263	0.67	21 C
			1.82%	1 P:z	-5.263	0.67	32 C
			-1.78%	2 P:z	4.578	0.00	29 C
			1.68%	1 S	-6.532	1.00	74 H
			1.63%	1 P:y	-5.263	0.67	15 C
			1.62%	1 S	-6.532	1.00	60 H
			-1.60%	2 P:z	4.578	0.00	33 C
			1.57%	2 S	2.394	0.00	50 H
			-1.54%	2 P:y	4.578	0.00	39 C
			-1.49%	2 S	11.741	0.00	23 C
			1.42%	1 S	-13.631	2.00	23 C
			-1.40%	2 S	2.394	0.00	74 H
			-1.38%	2 S	11.741	0.00	25 C
			1.31%	1 S	-6.532	1.00	87 H
			1.28%	1 S	-13.631	2.00	35 C
			-1.21%	2 S	11.741	0.00	35 C
			1.20%	2 S	2.394	0.00	52 H
			-1.20%	2 S	2.394	0.00	56 H
			1.17%	1 S	-6.532	1.00	44 H
			1.17%	2 P:z	4.578	0.00	39 C
			1.05%	1 P:x	-5.263	0.67	37 C
			1.04%	1 S	-6.532	1.00	66 H
			-1.02%	2 P:x	4.578	0.00	27 C
			1.01%	1 P:y	-5.263	0.67	22 C
3.513	0.00	185 A	6.01%	1 P:z	-5.263	0.67	32 C
			4.55%	2 P:x	4.578	0.00	20 C
			4.39%	1 P:x	-5.263	0.67	40 C
			4.29%	1 S	-6.532	1.00	96 H
			4.12%	1 S	-6.532	1.00	62 H
			3.92%	1 S	-6.532	1.00	93 H

3.72%	1 P:z	-5.263	0.67	17 C
3.67%	1 S	-6.532	1.00	88 H
3.45%	1 P:x	-5.263	0.67	32 C
-3.30%	2 P:x	4.578	0.00	37 C
2.98%	1 P:x	-5.263	0.67	37 C
2.88%	1 S	-6.532	1.00	85 H
2.85%	2 P:x	4.578	0.00	32 C
-2.69%	2 P:y	4.578	0.00	17 C
2.57%	1 P:y	-5.263	0.67	37 C
2.35%	1 S	-6.532	1.00	94 H
2.30%	1 P:y	-5.263	0.67	24 C
2.18%	1 P:z	-5.263	0.67	42 C
1.98%	1 P:y	-5.263	0.67	23 C
1.96%	1 P:z	-5.263	0.67	20 C
1.87%	1 S	-6.532	1.00	46 H
1.85%	1 S	-6.532	1.00	91 H
1.83%	2 S	2.394	0.00	70 H
-1.80%	2 S	2.394	0.00	90 H
1.74%	1 P:z	-5.263	0.67	23 C
1.67%	1 S	-6.532	1.00	66 H
1.58%	1 P:z	-5.263	0.67	29 C
1.55%	2 P:x	4.578	0.00	22 C
1.51%	1 P:y	-5.263	0.67	21 C
1.40%	1 S	-6.532	1.00	67 H
1.37%	1 S	-6.532	1.00	83 H
1.36%	2 S	2.394	0.00	68 H
-1.35%	2 S	11.741	0.00	17 C
-1.34%	2 P:z	4.578	0.00	32 C
1.33%	1 S	-6.532	1.00	90 H
1.32%	1 S	-6.532	1.00	64 H
-1.24%	2 S	2.394	0.00	96 H

-1.22%	2 P:x	4.578	0.00	24 C
-1.20%	2 S	2.394	0.00	92 H
1.15%	2 P:z	4.578	0.00	24 C
-1.13%	2 P:y	4.578	0.00	23 C
1.12%	2 P:x	4.578	0.00	34 C
1.12%	1 S	-13.631	2.00	17 C
1.10%	1 P:x	-5.263	0.67	18 C
1.09%	1 S	-6.532	1.00	77 H
-1.09%	2 P:x	4.578	0.00	40 C
1.07%	2 P:z	4.578	0.00	40 C
-1.02%	2 S	2.394	0.00	44 H
-1.01%	2 P:z	4.578	0.00	23 C

1

B O N D I N G   E N E R G Y *** (decomposition) ***

*** IMPORTANT NOTE ***

The bond energy is computed as an energy difference between molecule and fragments. In particular when the fragments are single atoms, they are usually computed as SPHERICALLY SYMMETRIC and SPIN-RESTRICTED. Obviously, this usually does NOT represent the true atomic groundstate.

To obtain the 'real' bond energy, (atomic) correction terms must be applied for the true (multiplet) fragment ground state. See ref: E.J.Baerends, V.Branchadell, M.Sodupe, Chem.Phys.Lett.265 (1997) 481

General theoretical background on the bond energy decomposition scheme used here (Morokuma-Ziegler) can be found in the review paper:

F.M. Bickelhaupt and E.J. Baerends,

"Kohn-Sham Density Functional Theory: Predicting and Understanding Chemistry"

In: Rev. Comput. Chem.; Lipkowitz, K. B. and Boyd, D. B., Eds.;

Wiley-VCH: New York, 2000, Vol. 15, 1-86.

Symbols used in the Bickelhaupt-Baerends (BB) paper are given below to make the direct connection to that paper, where detailed explanations can be found on the meaning of the various terms.

	hartree	eV	kcal/mol	kJ/mol
	--------------------	-----------	----------	-----------
**Pauli Repulsion**				
Kinetic (Delta T^0):	210.096755374791854	5717.0236	131837.72	551608.95
Delta V^Pauli Coulomb:	-110.863532369576575	-3016.7502	-69567.92	-291072.16
Delta V^Pauli LDA-XC:	-26.078724479557291	-709.6382	-16364.65	-68469.68
Delta V^Pauli GGA-Exchange:	1.464761026067094	39.8582	919.15	3845.73
Delta V^Pauli GGA-Correlation:	-1.928641243220374	-52.4810	-1210.24	-5063.65
	--------------------	-----------	----------	-----------
Total Pauli Repulsion:	72.690618308504725	1978.0124	45614.06	190849.19

(Total Pauli Repulsion =
Delta E^Pauli in BB paper)

Steric Interaction

	hartree	eV	kcal/mol	kJ/mol
Pauli Repulsion (Delta E^Pauli):	72.690618308504725	1978.0124	45614.06	190849.19
Electrostatic Interaction:	-15.081070277219581	-410.3768	-9463.52	-39595.34

(Electrostatic Interaction =
Delta V_elstat in the BB paper)

-------------------- ----------- ---------- -----------

Total Steric Interaction: 57.609548031285144 1567.6356 36150.54 151253.85

(Total Steric Interaction =

Delta E^0 in the BB paper)

Orbital Interactions

A: -77.092287067045746 -2097.7879 -48376.15 -202405.77

-------------------- ----------- ---------- -----------

Total Orbital Interactions: -77.099310275179633 -2097.9790 -48380.55 -202424.21

Alternative Decomposition Orb.Int.

Kinetic: -191.530167694215436 -5211.8010 -120187.01 -502862.38

Coulomb: 105.855611308942699 2880.4777 66425.41 277923.87

XC: 8.575246110093056 233.3443 5381.05 22514.31

-------------------- ----------- ---------- -----------

Total Orbital Interactions: -77.099310275179676 -2097.9790 -48380.55 -202424.21

Residu (E=Steric+OrbInt+Res): -0.000440393086981 -0.0120 -0.28 -1.16

Dispersion Energy: -0.185089018260984 -5.0365 -116.15 -485.95

Total Bonding Energy: -19.675291655242457 -535.3919 -12346.43 -51657.47

Summary of Bonding Energy (energy terms are taken from the energy decomposition above)

==
====

Electrostatic Energy: -15.081070277219581 -410.3768 -9463.52 -39595.34

Kinetic Energy: 18.566587680576419 505.2226 11650.71 48746.57

Coulomb (Steric+OrbInt) Energy: -5.008361453720852 -136.2844 -3142.79 -13149.45

XC Energy: -17.967358586617515 -488.9167 -11274.69 -47173.29

Dispersion Energy:	-0.185089018260984	-5.0365	-116.15	-485.95
	--------------------	-----------	----------	-----------
Total Bonding Energy:	-19.675291655242514	-535.3919	-12346.43	-51657.47

Correction terms (incorporated in energies above; only for test purposes):

1. Indication of fit-quality: 1st-order fit-correction used in the energy (hartree): 0.0016618607
2. Electrostatic (Fit correction): 0.0000000000

F R A G M E N T E N E R G Y T E R M S *** (summed over all fragments) ***

The energy terms below are (parts of) the Total Energy of the fragments from which the molecule is built.

Exchange and Correlation

Exchange LDA:	-228.215821310933677	-6210.0685	-143207.61	-599180.55
Exchange GGA:	-29.540333570171811	-803.8334	-18536.84	-77558.13
Correlation LDA:	0.000000000000000	0.0000	0.00	0.00
Correlation GGA:	-9.308822383076050	-253.3059	-5841.37	-24440.31
	--------------------	-----------	----------	-----------
Total XC:	-267.064977264181550	-7267.2078	-167585.82	-701179.00

Electrostatic potential at the Nuclei due to electrons and other nuclei

Atom		Potential
----		---------
1)	O	22.29069508
2)	C	14.60697343
3)	C	14.65801017
4)	C	14.67289030
5)	O	22.31812907
6)	N	18.33987770
7)	O	22.29916328
8)	C	14.62535388
9)	C	14.74009723
10)	C	14.57557526
11)	O	22.29895110
12)	C	14.67781719
13)	O	22.25131894
14)	C	14.65523524
15)	C	14.64698567
16)	O	22.31691375
17)	C	14.72223795
18)	C	14.72137017
19)	O	22.26717367
20)	C	14.72148376
21)	C	14.63595188
22)	C	14.69784040
23)	C	14.71321991
24)	C	14.70356933
25)	C	14.72313623
26)	C	14.71389628

27)	C	14.65230129
28)	C	14.72126785
29)	C	14.71201481
30)	C	14.71656044
31)	O	22.33696264
32)	C	14.63889670
33)	C	14.73933624
34)	C	14.65318390
35)	C	14.73295942
36)	C	14.70492427
37)	C	14.70332984
38)	C	14.67785599
39)	C	14.71602415
40)	H	1.10522026
41)	H	1.12927144
42)	H	1.13395732
43)	H	1.12819453
44)	H	1.13588093
45)	H	1.13489028
46)	H	1.13242874
47)	H	1.12323457
48)	H	1.12890751
49)	H	1.12807103
50)	H	1.12252659
51)	H	1.12128147
52)	H	1.12060494
53)	H	1.11541781
54)	H	1.11737413
55)	H	1.11223427
56)	H	1.09249186
57)	H	1.10585883

58)	H	1.11593609
59)	H	1.11340427
60)	H	1.11256388
61)	H	1.11242733
62)	H	1.11351213
63)	H	1.10979578
64)	H	1.11431068
65)	H	1.10943904
66)	H	1.11669957
67)	H	1.11733813
68)	H	1.11871795
69)	C	14.59457553
70)	H	1.11421183
71)	H	1.10856308
72)	H	1.11435117
73)	H	1.11083818
74)	H	1.11276316
75)	H	1.11121459
76)	H	1.12781507
77)	H	1.11735598
78)	H	1.12036637
79)	H	1.12861212
80)	H	1.11764976
81)	H	1.12139429
82)	H	1.10200967
83)	H	1.10796192
84)	H	1.10345800
85)	H	1.10859844
86)	H	1.01002580
87)	O	22.30474357
88)	H	1.07842200

89) H 1.10183642

90) H 1.09978144

91) H 1.09057245

92) C 14.71103685

93) O 22.32468109

94) H 1.11538544

95) H 1.11455295

96) H 1.11253445

=== CALCULATE ANALYTICAL SECOND DERIVATIVES OF THE ENERGY ===

This Analytical Second Derivatives Progam

was written for ADF by

Stephen K. Wolff

Scientific Computing & Modelling

Vrije University

Theoretical Chemistry

De Boelelaan 1083

1081 HV Amsterdam

The Netherlands

References:

```
                ===========

   Attila Berces, Ross M. Dickson, Liangyou Fan
   Heiko Jacobsen, David Swerhone, Tom Ziegler,
   Computer Physics Communications 100 (1997) 247.

   Heiko Jacobsen, Attila Berces
   David Swerhone, Tom Ziegler,
   Computer Physics Communications 100 (1997) 263.

   Stephen K. Wolff
   Int. J. Quantum Chem. 104 (2005) 645.

  -------------------------------------------------

<><><><><><><><><><><><><><><><><><><><><><><><><>

  SECOND DERIVATIVES INFORMATION:
  -------------------------------

  === PRINT

   EIGS,  lprint_eigbas =  F
   U1,    lprint_u1     =  F
   PARTS, lprint_parts  =  F

  === DEBUG

   ALL       =  F
   B1        =  F
   DENSITIES  =  F
```

FIT = F

HESSIAN = F

NUMBERS = F

SYMMETRY = F

U1 = F

=== CPKS

MAX_CPKS_ITERATIONS = 20

U1_ACCURACY PARAMETER = 5.00

U1 ACCURACY = 0.0000100

Estimated peak private memory usage 512 MB per process

shared memory usage 6108 MB per node

<><><><><><><><><><><><><><><><><><><><><><><><><>

Handling 96 atoms per pass in write_gradb1

wr_gradb1 5531369472.00000 1048576000.00000

######## LOOPING OVER NUCLEI ########

NUCLEUS: 1 O

=================== solving CPKS equations ===================

NUCLEUS COORD. ITERATION ACCURACY

------- ------ --------- --------

1 1 1 0.47614049D-02

1	1	2	0.15323121D-02
1	1	3	0.82541419D-03
1	1	4	0.22933135D-03
1	1	5	0.68970622D-04
1	1	6	0.24101316D-04
1	1	7	0.71947301D-05

*** CPKS converged.

1	2	1	0.83263930D-02
1	2	2	0.28178845D-02
1	2	3	0.68944824D-03
1	2	4	0.12105811D-03
1	2	5	0.58512736D-04
1	2	6	0.29987359D-04
1	2	7	0.55354621D-05

*** CPKS converged.

1	3	1	0.65045488D-02
1	3	2	0.18659029D-02
1	3	3	0.10667065D-02
1	3	4	0.18950732D-03
1	3	5	0.65171366D-04
1	3	6	0.46447649D-04
1	3	7	0.11917163D-04
1	3	8	0.39810285D-05

*** CPKS converged.

NUCLEUS: 5 O

NUCLEUS COORD. ITERATION ACCURACY

------- ------ --------- --------

5	1	1	0.79432224D-02
5	1	2	0.26581579D-02
5	1	3	0.13208978D-02
5	1	4	0.20158503D-03
5	1	5	0.62884095D-04
5	1	6	0.36716026D-04
5	1	7	0.53623098D-05

*** CPKS converged.

5	2	1	0.13967476D-01
5	2	2	0.29538487D-02
5	2	3	0.33549743D-03
5	2	4	0.15556631D-03
5	2	5	0.81997438D-04
5	2	6	0.26290028D-04
5	2	7	0.85203131D-05

*** CPKS converged.

5	3	1	0.15509485D-01
5	3	2	0.29503504D-02
5	3	3	0.15709882D-02
5	3	4	0.10900070D-03
5	3	5	0.72637675D-04
5	3	6	0.24196091D-04

5 3 7 0.14489229D-04

5 3 8 0.62196311D-05

*** CPKS converged.

NUCLEUS: 7 O

NUCLEUS	COORD.	ITERATION	ACCURACY
7	1	1	0.33478110D-02
7	1	2	0.66761329D-03
7	1	3	0.54636113D-03
7	1	4	0.20861947D-03
7	1	5	0.62156615D-04
7	1	6	0.19497782D-04
7	1	7	0.97246250D-05

*** CPKS converged.

7	2	1	0.70356471D-02
7	2	2	0.24607940D-02
7	2	3	0.13290717D-02
7	2	4	0.13226665D-03
7	2	5	0.43355544D-04
7	2	6	0.29705923D-04
7	2	7	0.10789482D-04

7 2 8 0.27138630D-05

*** CPKS converged.

----

7 3 1 0.35037944D-02

7 3 2 0.21113924D-02

7 3 3 0.96887220D-03

7 3 4 0.24850277D-03

7 3 5 0.45952996D-04

7 3 6 0.10485617D-04

7 3 7 0.46555557D-05

*** CPKS converged.

NUCLEUS: 11 O

NUCLEUS	COORD.	ITERATION	ACCURACY
11	1	1	0.63325900D-02
11	1	2	0.16362197D-02
11	1	3	0.30876097D-03
11	1	4	0.26038620D-03
11	1	5	0.11098122D-03
11	1	6	0.29704367D-04
11	1	7	0.54914721D-05

*** CPKS converged.

11	2	1	0.89534808D-02
11	2	2	0.25588139D-02
11	2	3	0.13926529D-02
11	2	4	0.13641341D-03
11	2	5	0.58752866D-04
11	2	6	0.29112463D-04
11	2	7	0.61794859D-05

*** CPKS converged.

11	3	1	0.12931899D-01
11	3	2	0.17810128D-02
11	3	3	0.59787117D-03
11	3	4	0.15832000D-03
11	3	5	0.54477248D-04
11	3	6	0.24753209D-04
11	3	7	0.12680726D-04
11	3	8	0.15879521D-05

*** CPKS converged.

NUCLEUS: 13 O

NUCLEUS COORD. ITERATION ACCURACY

------- ------ --------- --------

13	1	1	0.31170237D-01
13	1	2	0.14775143D-02
13	1	3	0.12785544D-02
13	1	4	0.37103097D-03
13	1	5	0.63967150D-04
13	1	6	0.23129923D-04
13	1	7	0.89930176D-05

*** CPKS converged.

13	2	1	0.25214386D-01
13	2	2	0.16266777D-02
13	2	3	0.86287008D-03
13	2	4	0.23211020D-03
13	2	5	0.90233594D-04
13	2	6	0.24776555D-04
13	2	7	0.81162222D-05

*** CPKS converged.

13	3	1	0.30528206D-01
13	3	2	0.52765185D-02
13	3	3	0.11941721D-02
13	3	4	0.58793895D-03
13	3	5	0.15034224D-03
13	3	6	0.58780925D-04
13	3	7	0.10873725D-04
13	3	8	0.58113481D-05

*** CPKS converged.

NUCLEUS: 16 O

NUCLEUS COORD.   ITERATION   ACCURACY

------- ------   ---------   --------

16	1	1	0.38442684D-01
16	1	2	0.30454679D-02
16	1	3	0.10040337D-02
16	1	4	0.52467852D-03
16	1	5	0.11017006D-03
16	1	6	0.24456589D-04
16	1	7	0.13683870D-04
16	1	8	0.71333038D-05

*** CPKS converged.

16	2	1	0.24780201D-01
16	2	2	0.32349415D-02
16	2	3	0.16557911D-02
16	2	4	0.38966914D-03
16	2	5	0.10266563D-03
16	2	6	0.18188038D-04
16	2	7	0.96579988D-05

*** CPKS converged.

16	3	1	0.27452792D-01
16	3	2	0.31215058D-02
16	3	3	0.14715632D-02
16	3	4	0.43870679D-03

16 3 5 0.10018950D-03

16 3 6 0.19486929D-04

16 3 7 0.13452570D-04

16 3 8 0.52718987D-05

*** CPKS converged.

NUCLEUS: 19 O

NUCLEUS	COORD.	ITERATION	ACCURACY
19	1	1	0.22154646D-01
19	1	2	0.24940400D-02
19	1	3	0.86978806D-03
19	1	4	0.22878742D-03
19	1	5	0.11340751D-03
19	1	6	0.28181018D-04
19	1	7	0.94302477D-05

*** CPKS converged.

19	2	1	0.38912179D-01
19	2	2	0.18988092D-02
19	2	3	0.79599225D-03
19	2	4	0.24512385D-03
19	2	5	0.10731754D-03

19 2 6 0.27169688D-04

19 2 7 0.51957853D-05

*** CPKS converged.

----

19 3 1 0.27960013D-01

19 3 2 0.51165367D-02

19 3 3 0.15737540D-02

19 3 4 0.53352058D-03

19 3 5 0.13580206D-03

19 3 6 0.50277498D-04

19 3 7 0.56436088D-05

*** CPKS converged.

NUCLEUS: 31 O

NUCLEUS COORD. ITERATION ACCURACY

------- ------ --------- --------

31 1 1 0.40068761D-01

31 1 2 0.27810980D-02

31 1 3 0.10004312D-02

31 1 4 0.77378580D-03

31 1 5 0.12421887D-03

31 1 6 0.37393867D-04

31 1 7 0.13947597D-04

31 1 8 0.41158334D-05

*** CPKS converged.

----

31 2 1 0.23361670D-01

31 2 2 0.30818143D-02

31 2 3 0.11133254D-02

31 2 4 0.10712843D-02

31 2 5 0.82334888D-04

31 2 6 0.40990341D-04

31 2 7 0.11654744D-04

31 2 8 0.25748965D-05

*** CPKS converged.

----

31 3 1 0.24047440D-01

31 3 2 0.36407042D-02

31 3 3 0.14143774D-02

31 3 4 0.66804684D-03

31 3 5 0.67312294D-04

31 3 6 0.31542706D-04

31 3 7 0.12105395D-04

31 3 8 0.30805016D-05

*** CPKS converged.

NUCLEUS: 87 O

NUCLEUS COORD. ITERATION ACCURACY

NUCLEUS	COORD.	ITERATION	ACCURACY
-------	------	---------	--------
87	1	1	0.40870213D-01
87	1	2	0.28743533D-02
87	1	3	0.16470647D-02
87	1	4	0.57770050D-03
87	1	5	0.17341705D-03
87	1	6	0.26964137D-04
87	1	7	0.13484730D-04
87	1	8	0.45720483D-05

*** CPKS converged.

87	2	1	0.36266058D-01
87	2	2	0.40140741D-02
87	2	3	0.11855571D-02
87	2	4	0.48099693D-03
87	2	5	0.17559422D-03
87	2	6	0.25002380D-04
87	2	7	0.13428169D-04
87	2	8	0.53230741D-05

*** CPKS converged.

87	3	1	0.54578336D-01
87	3	2	0.22013492D-02
87	3	3	0.11325419D-02
87	3	4	0.38543069D-03
87	3	5	0.84244758D-04
87	3	6	0.20232715D-04
87	3	7	0.12872830D-04
87	3	8	0.40069014D-05

*** CPKS converged.

NUCLEUS: 93 O

NUCLEUS COORD. ITERATION ACCURACY

------- ------ --------- --------

93	1	1	0.42467251D-01
93	1	2	0.27716499D-02
93	1	3	0.95429260D-03
93	1	4	0.40754788D-03
93	1	5	0.13928656D-03
93	1	6	0.18336258D-04
93	1	7	0.78081586D-05

*** CPKS converged.

93	2	1	0.53755946D-01
93	2	2	0.27486805D-02
93	2	3	0.12517308D-02
93	2	4	0.66045691D-03
93	2	5	0.21212075D-03
93	2	6	0.26901936D-04
93	2	7	0.89399936D-05

*** CPKS converged.

93	3	1	0.55271759D-01
93	3	2	0.31918492D-02
93	3	3	0.10412778D-02
93	3	4	0.38364168D-03
93	3	5	0.10905292D-03
93	3	6	0.21348616D-04
93	3	7	0.96423266D-05

*** CPKS converged.

NUCLEUS: 2 C

NUCLEUS	COORD.	ITERATION	ACCURACY
2	1	1	0.15586500D-01
2	1	2	0.42037485D-02
2	1	3	0.18618897D-02
2	1	4	0.25356767D-03
2	1	5	0.91116577D-04
2	1	6	0.48821763D-04
2	1	7	0.20044797D-04
2	1	8	0.49584014D-05

*** CPKS converged.

2	2	1	0.11471871D-01

2	2	2	0.42582842D-02
2	2	3	0.20531956D-02
2	2	4	0.22472134D-03
2	2	5	0.86124944D-04
2	2	6	0.50887636D-04
2	2	7	0.20200450D-04
2	2	8	0.53573853D-05

*** CPKS converged.

2	3	1	0.13584260D-01
2	3	2	0.20855508D-02
2	3	3	0.10130807D-02
2	3	4	0.33107754D-03
2	3	5	0.88800414D-04
2	3	6	0.38610426D-04
2	3	7	0.16507295D-04
2	3	8	0.49565422D-05

*** CPKS converged.

NUCLEUS: 3 C

NUCLEUS	COORD.	ITERATION	ACCURACY
-------	------	---------	--------
3	1	1	0.57155946D-02

3	1	2	0.84822229D-03
3	1	3	0.44747128D-03
3	1	4	0.19955421D-03
3	1	5	0.73989679D-04
3	1	6	0.14567265D-04
3	1	7	0.44058097D-05

*** CPKS converged.

3	2	1	0.58668564D-01
3	2	2	0.46868997D-02
3	2	3	0.85904655D-03
3	2	4	0.33397195D-03
3	2	5	0.16219643D-03
3	2	6	0.53061302D-04
3	2	7	0.80144636D-05

*** CPKS converged.

3	3	1	0.17565128D-01
3	3	2	0.61943818D-02
3	3	3	0.13016983D-02
3	3	4	0.24156231D-03
3	3	5	0.13327817D-03
3	3	6	0.65786008D-04
3	3	7	0.12926703D-04
3	3	8	0.34020105D-05

*** CPKS converged.

NUCLEUS: 4 C

NUCLEUS COORD.   ITERATION   ACCURACY

------- ------   ---------   --------

4	1	1	0.10844800D-01
4	1	2	0.19169342D-02
4	1	3	0.87390680D-03
4	1	4	0.37798611D-03
4	1	5	0.84354479D-04
4	1	6	0.11914998D-04
4	1	7	0.68505146D-05

*** CPKS converged.

4	2	1	0.64195055D-02
4	2	2	0.21706054D-02
4	2	3	0.12599240D-02
4	2	4	0.36117333D-03
4	2	5	0.54135223D-04
4	2	6	0.18828012D-04
4	2	7	0.13148075D-04
4	2	8	0.38115664D-05

*** CPKS converged.

4	3	1	0.96184832D-02
4	3	2	0.32200353D-02
4	3	3	0.12811652D-02
4	3	4	0.29824697D-03
4	3	5	0.51498084D-04

4 3 6 0.40005203D-04

4 3 7 0.16997855D-04

4 3 8 0.32428429D-05

*** CPKS converged.

NUCLEUS: 8 C

NUCLEUS	COORD.	ITERATION	ACCURACY
8	1	1	0.63202342D-01
8	1	2	0.26396162D-02
8	1	3	0.16705930D-02
8	1	4	0.67662404D-03
8	1	5	0.19266110D-03
8	1	6	0.43084776D-04
8	1	7	0.15839655D-04
8	1	8	0.10406137D-04
8	1	9	0.21610362D-05

*** CPKS converged.

8	2	1	0.39339878D-01
8	2	2	0.29693096D-02
8	2	3	0.13989133D-02
8	2	4	0.58316720D-03

8 2 5 0.16850632D-03

8 2 6 0.28589130D-04

8 2 7 0.13480371D-04

8 2 8 0.79708516D-05

*** CPKS converged.

----

8 3 1 0.44541777D-01

8 3 2 0.33239111D-02

8 3 3 0.13555898D-02

8 3 4 0.66140858D-03

8 3 5 0.14307655D-03

8 3 6 0.42249079D-04

8 3 7 0.16283029D-04

8 3 8 0.86374912D-05

*** CPKS converged.

NUCLEUS: 9 C

NUCLEUS COORD. ITERATION ACCURACY

------- ------ --------- --------

9 1 1 0.11233778D-01

9 1 2 0.43822936D-02

9 1 3 0.39521993D-03

9 1 4 0.18639616D-03

9 1 5 0.96241550D-04

9 1 6 0.24831234D-04

9 1 7 0.91681399D-05

*** CPKS converged.

----

9 2 1 0.66999035D-02

9 2 2 0.33830808D-02

9 2 3 0.16811299D-02

9 2 4 0.34564473D-03

9 2 5 0.12514000D-03

9 2 6 0.32296964D-04

9 2 7 0.12223967D-04

9 2 8 0.53060496D-05

*** CPKS converged.

----

9 3 1 0.50888696D-02

9 3 2 0.26200947D-02

9 3 3 0.10397763D-02

9 3 4 0.36228688D-03

9 3 5 0.13130464D-03

9 3 6 0.30897505D-04

9 3 7 0.11857557D-04

9 3 8 0.52194133D-05

*** CPKS converged.

NUCLEUS: 10 C

NUCLEUS COORD.   ITERATION   ACCURACY

------- ------   ---------   --------

10	1	1	0.64775727D-01
10	1	2	0.57640523D-02
10	1	3	0.23009847D-02
10	1	4	0.86724218D-03
10	1	5	0.27368234D-03
10	1	6	0.47186476D-04
10	1	7	0.19524164D-04
10	1	8	0.52817424D-05

*** CPKS converged.

10	2	1	0.49786249D-01
10	2	2	0.45540126D-02
10	2	3	0.16384320D-02
10	2	4	0.68485047D-03
10	2	5	0.26532933D-03
10	2	6	0.34417746D-04
10	2	7	0.15841130D-04
10	2	8	0.55667946D-05

*** CPKS converged.

10	3	1	0.10931969D+00
10	3	2	0.52100903D-02
10	3	3	0.22656014D-02
10	3	4	0.78745432D-03
10	3	5	0.30104641D-03
10	3	6	0.88819993D-04

10	3	7	0.23666182D-04
10	3	8	0.81137092D-05

*** CPKS converged.

NUCLEUS: 12 C

NUCLEUS	COORD.	ITERATION	ACCURACY
12	1	1	0.12325106D-01
12	1	2	0.30888102D-02
12	1	3	0.12964292D-02
12	1	4	0.31289861D-03
12	1	5	0.10802892D-03
12	1	6	0.32398698D-04
12	1	7	0.13041486D-04
12	1	8	0.23520620D-05

*** CPKS converged.

12	2	1	0.19137399D-01
12	2	2	0.36026891D-02
12	2	3	0.15205147D-02
12	2	4	0.26495868D-03
12	2	5	0.86577407D-04
12	2	6	0.36885804D-04

12	2	7	0.12223188D-04
12	2	8	0.47200721D-05

*** CPKS converged.

12	3	1	0.19978233D-01
12	3	2	0.37795454D-02
12	3	3	0.10032973D-02
12	3	4	0.34462104D-03
12	3	5	0.11767922D-03
12	3	6	0.45722013D-04
12	3	7	0.87325577D-05

*** CPKS converged.

NUCLEUS: 14 C

NUCLEUS	COORD.	ITERATION	ACCURACY
-------	------	---------	--------
14	1	1	0.98204180D-02
14	1	2	0.18045874D-02
14	1	3	0.11069952D-02
14	1	4	0.27629253D-03
14	1	5	0.85007685D-04
14	1	6	0.28272695D-04
14	1	7	0.10798103D-04

14 1 8 0.46747903D-05

*** CPKS converged.

----

14 2 1 0.10265304D-01

14 2 2 0.33512594D-02

14 2 3 0.77441274D-03

14 2 4 0.16771756D-03

14 2 5 0.10817335D-03

14 2 6 0.41683097D-04

14 2 7 0.73793530D-05

*** CPKS converged.

----

14 3 1 0.53750713D-02

14 3 2 0.25181354D-02

14 3 3 0.73462824D-03

14 3 4 0.15820968D-03

14 3 5 0.56439266D-04

14 3 6 0.43071898D-04

14 3 7 0.12988518D-04

14 3 8 0.36225613D-05

*** CPKS converged.

NUCLEUS: 15 C

NUCLEUS COORD.   ITERATION   ACCURACY

-------	------	---------	--------
15	1	1	0.23481607D-01
15	1	2	0.21756450D-02
15	1	3	0.90424131D-03
15	1	4	0.21830157D-03
15	1	5	0.99411049D-04
15	1	6	0.36614063D-04
15	1	7	0.10591097D-04
15	1	8	0.62639126D-05

*** CPKS converged.

15	2	1	0.62644633D-02
15	2	2	0.23936316D-02
15	2	3	0.69752067D-03
15	2	4	0.26630976D-03
15	2	5	0.76886520D-04
15	2	6	0.34348271D-04
15	2	7	0.11930672D-04
15	2	8	0.23967830D-05

*** CPKS converged.

15	3	1	0.15612963D-01
15	3	2	0.25314259D-02
15	3	3	0.88940843D-03
15	3	4	0.27227143D-03
15	3	5	0.10902386D-03
15	3	6	0.31603589D-04
15	3	7	0.13299930D-04
15	3	8	0.34334282D-05

*** CPKS converged.

NUCLEUS: 17 C

NUCLEUS COORD. ITERATION ACCURACY

NUCLEUS	COORD.	ITERATION	ACCURACY
17	1	1	0.77546691D-02
17	1	2	0.30276073D-02
17	1	3	0.11324622D-02
17	1	4	0.22543718D-03
17	1	5	0.66151610D-04
17	1	6	0.26334399D-04
17	1	7	0.10394252D-04
17	1	8	0.59358746D-05

*** CPKS converged.

17	2	1	0.87282199D-02
17	2	2	0.36285161D-02
17	2	3	0.10811696D-02
17	2	4	0.17690782D-03
17	2	5	0.15268882D-03
17	2	6	0.39170302D-04
17	2	7	0.98627619D-05

*** CPKS converged.

17	3	1	0.11709599D-01
17	3	2	0.43648558D-02
17	3	3	0.79896810D-03
17	3	4	0.18863244D-03
17	3	5	0.10320984D-03
17	3	6	0.36465273D-04
17	3	7	0.11530816D-04
17	3	8	0.49335276D-05

*** CPKS converged.

NUCLEUS: 18 C

NUCLEUS	COORD.	ITERATION	ACCURACY
18	1	1	0.17829151D-01
18	1	2	0.63676705D-02
18	1	3	0.67706607D-03
18	1	4	0.27087565D-03
18	1	5	0.12160353D-03
18	1	6	0.18187728D-04
18	1	7	0.13648861D-04
18	1	8	0.27034693D-05

*** CPKS converged.

----

18 2 1 0.74703134D-02

18 2 2 0.60563954D-02

18 2 3 0.10430756D-02

18 2 4 0.21097826D-03

18 2 5 0.11748630D-03

18 2 6 0.25386625D-04

18 2 7 0.73381994D-05

*** CPKS converged.

----

18 3 1 0.21640842D-01

18 3 2 0.74542779D-02

18 3 3 0.92034618D-03

18 3 4 0.26028074D-03

18 3 5 0.74194573D-04

18 3 6 0.29766966D-04

18 3 7 0.84323992D-05

*** CPKS converged.

NUCLEUS: 20 C

NUCLEUS COORD. ITERATION ACCURACY

------- ------ --------- --------

20 1 1 0.49635836D-01

20 1 2 0.43875616D-02

20	1	3	0.95985510D-03
20	1	4	0.62635608D-03
20	1	5	0.17546028D-03
20	1	6	0.57721721D-04
20	1	7	0.13622509D-04
20	1	8	0.56750050D-05

*** CPKS converged.

20	2	1	0.33603500D-01
20	2	2	0.67643757D-02
20	2	3	0.61650832D-03
20	2	4	0.53658262D-03
20	2	5	0.12653261D-03
20	2	6	0.35654779D-04
20	2	7	0.15709369D-04
20	2	8	0.46583924D-05

*** CPKS converged.

20	3	1	0.11065232D-01
20	3	2	0.28226697D-02
20	3	3	0.61830446D-03
20	3	4	0.21015794D-03
20	3	5	0.94689250D-04
20	3	6	0.14993513D-04
20	3	7	0.50390907D-05

*** CPKS converged.

NUCLEUS: 21 C

NUCLEUS COORD.   ITERATION   ACCURACY

------- ------   ---------   --------

21	1	1	0.76286000D-02
21	1	2	0.17841002D-02
21	1	3	0.10147649D-02
21	1	4	0.34185931D-03
21	1	5	0.74429576D-04
21	1	6	0.31378172D-04
21	1	7	0.56552623D-05

*** CPKS converged.

21	2	1	0.65217638D-01
21	2	2	0.50012001D-02
21	2	3	0.96095379D-03
21	2	4	0.34349796D-03
21	2	5	0.14821337D-03
21	2	6	0.38915442D-04
21	2	7	0.19368909D-04
21	2	8	0.56268160D-05

*** CPKS converged.

21	3	1	0.14161368D-01
21	3	2	0.38161725D-02
21	3	3	0.21483550D-02
21	3	4	0.32093050D-03
21	3	5	0.13732291D-03

```
  21     3       6     0.83665295D-04
  21     3       7     0.10704295D-04
  21     3       8     0.38316868D-05
*** CPKS converged.
----------------------------------------------


###### NUCLEUS:    22  C


NUCLEUS  COORD.    ITERATION   ACCURACY
-------  ------    ---------   --------
  22     1       1     0.18975466D-01
  22     1       2     0.43126158D-02
  22     1       3     0.11035986D-02
  22     1       4     0.35442988D-03
  22     1       5     0.10734205D-03
  22     1       6     0.28815063D-04
  22     1       7     0.78376826D-05
*** CPKS converged.
----------------------------------------------
  22     2       1     0.21426004D-01
  22     2       2     0.29296410D-02
  22     2       3     0.11497581D-02
  22     2       4     0.19139003D-03
  22     2       5     0.99312036D-04
  22     2       6     0.48052779D-04
```

22 2 7 0.76742430D-05

*** CPKS converged.

22	3	1	0.13955544D-01
22	3	2	0.24293808D-02
22	3	3	0.73922018D-03
22	3	4	0.14619089D-03
22	3	5	0.81237792D-04
22	3	6	0.41443541D-04
22	3	7	0.11337600D-04
22	3	8	0.28768272D-05

*** CPKS converged.

NUCLEUS: 23 C

NUCLEUS	COORD.	ITERATION	ACCURACY
-------	------	---------	--------
23	1	1	0.19210433D-01
23	1	2	0.37000689D-02
23	1	3	0.22880363D-02
23	1	4	0.50196005D-03
23	1	5	0.11876243D-03
23	1	6	0.43206327D-04
23	1	7	0.18242769D-04

23	1	8	0.50983249D-05

*** CPKS converged.

23	2	1	0.75433218D-02
23	2	2	0.26426819D-02
23	2	3	0.71675811D-03
23	2	4	0.39537926D-03
23	2	5	0.16501764D-03
23	2	6	0.17639415D-04
23	2	7	0.14003103D-04
23	2	8	0.51361126D-05

*** CPKS converged.

23	3	1	0.16890556D-01
23	3	2	0.20788711D-02
23	3	3	0.12141856D-02
23	3	4	0.22779116D-03
23	3	5	0.20003152D-03
23	3	6	0.44902484D-04
23	3	7	0.92278346D-05

*** CPKS converged.

NUCLEUS: 24 C

NUCLEUS COORD. ITERATION ACCURACY

NUCLEUS	COORD.	ITERATION	ACCURACY
24	1	1	0.11606647D-01
24	1	2	0.24189015D-02
24	1	3	0.14706286D-02
24	1	4	0.33480895D-03
24	1	5	0.42931744D-04
24	1	6	0.25694627D-04
24	1	7	0.13910148D-04
24	1	8	0.33532593D-05

*** CPKS converged.

24	2	1	0.89463529D-02
24	2	2	0.29673857D-02
24	2	3	0.17104185D-02
24	2	4	0.17460646D-03
24	2	5	0.43026299D-04
24	2	6	0.27373058D-04
24	2	7	0.13552390D-04
24	2	8	0.18649505D-05

*** CPKS converged.

24	3	1	0.89817438D-02
24	3	2	0.20133164D-02
24	3	3	0.10717890D-02
24	3	4	0.47630311D-03
24	3	5	0.72930048D-04
24	3	6	0.17033714D-04
24	3	7	0.69567714D-05

*** CPKS converged.

NUCLEUS: 25 C

NUCLEUS	COORD.	ITERATION	ACCURACY
25	1	1	0.87985335D-02
25	1	2	0.42316024D-02
25	1	3	0.14154676D-02
25	1	4	0.21620790D-03
25	1	5	0.91367706D-04
25	1	6	0.32417129D-04
25	1	7	0.10074183D-04
25	1	8	0.49928438D-05

*** CPKS converged.

25	2	1	0.67258974D-02
25	2	2	0.22910800D-02
25	2	3	0.11883084D-02
25	2	4	0.13258755D-03
25	2	5	0.10450355D-03
25	2	6	0.26073866D-04
25	2	7	0.64804852D-05

*** CPKS converged.

25	3	1	0.13373661D-01

25	3	2	0.33285766D-02
25	3	3	0.11037666D-02
25	3	4	0.51711826D-03
25	3	5	0.12579425D-03
25	3	6	0.39557594D-04
25	3	7	0.12906678D-04
25	3	8	0.58671997D-05

*** CPKS converged.

NUCLEUS: 26 C

NUCLEUS	COORD.	ITERATION	ACCURACY
26	1	1	0.61303786D-02
26	1	2	0.11830958D-02
26	1	3	0.26835446D-03
26	1	4	0.15115014D-03
26	1	5	0.68443388D-04
26	1	6	0.11106010D-04
26	1	7	0.67683993D-05

*** CPKS converged.

26	2	1	0.60035672D-02
26	2	2	0.23555200D-02

26	2	3	0.12074715D-02
26	2	4	0.20756025D-03
26	2	5	0.33985113D-04
26	2	6	0.13870974D-04
26	2	7	0.57169273D-05

*** CPKS converged.

26	3	1	0.45251805D-02
26	3	2	0.81746039D-03
26	3	3	0.88881743D-03
26	3	4	0.35742160D-03
26	3	5	0.51443717D-04
26	3	6	0.84804161D-05

*** CPKS converged.

NUCLEUS: 27 C

NUCLEUS	COORD.	ITERATION	ACCURACY
-------	------	---------	--------
27	1	1	0.50660126D-02
27	1	2	0.10285484D-02
27	1	3	0.58533486D-03
27	1	4	0.20122151D-03
27	1	5	0.76112456D-04

27	1	6	0.14301186D-04
27	1	7	0.69570878D-05

*** CPKS converged.

27	2	1	0.60725040D-02
27	2	2	0.19809881D-02
27	2	3	0.92039537D-03
27	2	4	0.29332890D-03
27	2	5	0.90465702D-04
27	2	6	0.22175449D-04
27	2	7	0.37871084D-05

*** CPKS converged.

27	3	1	0.96357041D-02
27	3	2	0.23960526D-02
27	3	3	0.13627549D-02
27	3	4	0.38708410D-03
27	3	5	0.58547251D-04
27	3	6	0.24156213D-04
27	3	7	0.12927816D-04
27	3	8	0.58568815D-05

*** CPKS converged.

NUCLEUS: 28 C

```
NUCLEUS  COORD.   ITERATION   ACCURACY
-------  ------    ---------   --------
  28      1        1     0.76826826D-02
  28      1        2     0.98903132D-03
  28      1        3     0.79053357D-03
  28      1        4     0.25336494D-03
  28      1        5     0.45580349D-04
  28      1        6     0.10240197D-04
  28      1        7     0.59791095D-05
*** CPKS converged.
----------------------------------------------
  28      2        1     0.65604467D-02
  28      2        2     0.14376814D-02
  28      2        3     0.33691980D-03
  28      2        4     0.14791935D-03
  28      2        5     0.72659571D-04
  28      2        6     0.95611607D-05
*** CPKS converged.
----------------------------------------------
  28      3        1     0.80819663D-02
  28      3        2     0.79132979D-03
  28      3        3     0.71317221D-03
  28      3        4     0.29656210D-03
  28      3        5     0.43550423D-04
  28      3        6     0.11851166D-04
  28      3        7     0.35343653D-05
*** CPKS converged.
----------------------------------------------
```

NUCLEUS: 29 C

NUCLEUS	COORD.	ITERATION	ACCURACY
-------	------	---------	--------
29	1	1	0.51122102D-01
29	1	2	0.71176682D-02
29	1	3	0.11901203D-02
29	1	4	0.44130112D-03
29	1	5	0.62623265D-04
29	1	6	0.31297238D-04
29	1	7	0.90112282D-05

*** CPKS converged.

29	2	1	0.69951762D-01
29	2	2	0.46288093D-02
29	2	3	0.11463919D-02
29	2	4	0.52491025D-03
29	2	5	0.56985856D-04
29	2	6	0.38696156D-04
29	2	7	0.13063461D-04
29	2	8	0.68465759D-05

*** CPKS converged.

29	3	1	0.21250882D-01
29	3	2	0.25429794D-02
29	3	3	0.13937592D-02

29	3	4	0.43503784D-03
29	3	5	0.15489601D-03
29	3	6	0.25576356D-04
29	3	7	0.70721328D-05

*** CPKS converged.

NUCLEUS: 30 C

NUCLEUS	COORD.	ITERATION	ACCURACY
30	1	1	0.78298631D-02
30	1	2	0.40506395D-02
30	1	3	0.10938767D-02
30	1	4	0.27895625D-03
30	1	5	0.14232018D-03
30	1	6	0.27301605D-04
30	1	7	0.95597698D-05

*** CPKS converged.

30	2	1	0.71118146D-02
30	2	2	0.34878262D-02
30	2	3	0.96904821D-03
30	2	4	0.32672174D-03
30	2	5	0.12473242D-03

30	2	6	0.38333577D-04
30	2	7	0.16721876D-04
30	2	8	0.54692690D-05

*** CPKS converged.

30	3	1	0.52978206D-02
30	3	2	0.30071685D-02
30	3	3	0.12937503D-02
30	3	4	0.16121573D-03
30	3	5	0.11963105D-03
30	3	6	0.61649179D-04
30	3	7	0.15154951D-04
30	3	8	0.36679855D-05

*** CPKS converged.

NUCLEUS: 32 C

NUCLEUS	COORD.	ITERATION	ACCURACY
-------	------	---------	--------
32	1	1	0.32012324D-01
32	1	2	0.50300367D-02
32	1	3	0.14357845D-02
32	1	4	0.73335182D-03
32	1	5	0.15666786D-03

32 1 6 0.37491121D-04

32 1 7 0.14584973D-04

32 1 8 0.53734818D-05

*** CPKS converged.

----

32 2 1 0.35765967D-01

32 2 2 0.48029750D-02

32 2 3 0.89821157D-03

32 2 4 0.30629317D-03

32 2 5 0.83202689D-04

32 2 6 0.41111852D-04

32 2 7 0.92175419D-05

*** CPKS converged.

----

32 3 1 0.26553092D-01

32 3 2 0.34148708D-02

32 3 3 0.96004918D-03

32 3 4 0.38440277D-03

32 3 5 0.20169674D-03

32 3 6 0.32248824D-04

32 3 7 0.74642646D-05

*** CPKS converged.

NUCLEUS: 33 C

NUCLEUS COORD. ITERATION ACCURACY

------- ------ --------- --------

33	1	1	0.29692788D-01
33	1	2	0.58229499D-02
33	1	3	0.18250757D-02
33	1	4	0.42579673D-03
33	1	5	0.10245394D-03
33	1	6	0.22224791D-04
33	1	7	0.66367892D-05

*** CPKS converged.

33	2	1	0.28240902D-01
33	2	2	0.32918795D-02
33	2	3	0.76085634D-03
33	2	4	0.33154444D-03
33	2	5	0.57975610D-04
33	2	6	0.17254213D-04
33	2	7	0.16117170D-04
33	2	8	0.55350532D-05

*** CPKS converged.

33	3	1	0.17594181D-01
33	3	2	0.75224110D-02
33	3	3	0.67629788D-03
33	3	4	0.22213616D-03
33	3	5	0.10809813D-03
33	3	6	0.52663348D-04
33	3	7	0.12657417D-04
33	3	8	0.42745794D-05

*** CPKS converged.

NUCLEUS: 34 C

NUCLEUS COORD. ITERATION ACCURACY

NUCLEUS	COORD.	ITERATION	ACCURACY
34	1	1	0.59377574D-01
34	1	2	0.48002100D-02
34	1	3	0.11838174D-02
34	1	4	0.84960908D-03
34	1	5	0.19912438D-03
34	1	6	0.58260180D-04
34	1	7	0.20352064D-04
34	1	8	0.53888725D-05

*** CPKS converged.

34	2	1	0.49028535D-01
34	2	2	0.46488715D-02
34	2	3	0.15709547D-02
34	2	4	0.15993945D-02
34	2	5	0.94420376D-04
34	2	6	0.62762752D-04
34	2	7	0.19222846D-04
34	2	8	0.35603195D-05

*** CPKS converged.

34	3	1	0.37073175D-01
34	3	2	0.46566132D-02
34	3	3	0.11715243D-02
34	3	4	0.58678530D-03
34	3	5	0.11268419D-03
34	3	6	0.46267375D-04
34	3	7	0.12347756D-04
34	3	8	0.29112750D-05

*** CPKS converged.

NUCLEUS: 35 C

NUCLEUS	COORD.	ITERATION	ACCURACY
-------	------	---------	--------
35	1	1	0.39172176D-02
35	1	2	0.22527305D-02
35	1	3	0.72754892D-03
35	1	4	0.17330539D-03
35	1	5	0.67199518D-04
35	1	6	0.18977402D-04
35	1	7	0.61015042D-05

*** CPKS converged.

----

35 2 1 0.80939754D-02

35 2 2 0.24487261D-02

35 2 3 0.14051677D-02

35 2 4 0.17040932D-03

35 2 5 0.49679725D-04

35 2 6 0.32262045D-04

35 2 7 0.14378045D-04

35 2 8 0.42262193D-05

*** CPKS converged.

----

35 3 1 0.48015215D-02

35 3 2 0.12567940D-02

35 3 3 0.73503028D-03

35 3 4 0.15363241D-03

35 3 5 0.40540458D-04

35 3 6 0.23593154D-04

35 3 7 0.96099097D-05

*** CPKS converged.

NUCLEUS: 36 C

NUCLEUS COORD. ITERATION ACCURACY

------- ------ --------- --------

36 1 1 0.59319851D-02

36	1	2	0.32355763D-02
36	1	3	0.17073303D-02
36	1	4	0.18049219D-03
36	1	5	0.58485507D-04
36	1	6	0.22171021D-04
36	1	7	0.52972563D-05

*** CPKS converged.

36	2	1	0.10335587D-01
36	2	2	0.45699885D-02
36	2	3	0.11924360D-02
36	2	4	0.17529875D-03
36	2	5	0.11668734D-03
36	2	6	0.46576716D-04
36	2	7	0.84427655D-05

*** CPKS converged.

36	3	1	0.90095879D-02
36	3	2	0.39790078D-02
36	3	3	0.39980776D-03
36	3	4	0.19596616D-03
36	3	5	0.64355301D-04
36	3	6	0.22513127D-04
36	3	7	0.62099527D-05

*** CPKS converged.

NUCLEUS: 37 C

NUCLEUS COORD. ITERATION ACCURACY

------- ------ --------- --------

37	1	1	0.75198222D-02
37	1	2	0.43239601D-02
37	1	3	0.11493980D-02
37	1	4	0.15047758D-03
37	1	5	0.45007225D-04
37	1	6	0.28521958D-04
37	1	7	0.13359283D-04
37	1	8	0.16296753D-05

*** CPKS converged.

37	2	1	0.13404084D-01
37	2	2	0.16953860D-02
37	2	3	0.31038548D-03
37	2	4	0.17986862D-03
37	2	5	0.68312166D-04
37	2	6	0.12835722D-04
37	2	7	0.48961038D-05

*** CPKS converged.

37	3	1	0.55227969D-02
37	3	2	0.37050714D-02
37	3	3	0.12670088D-02
37	3	4	0.24718799D-03
37	3	5	0.74335938D-04
37	3	6	0.48890389D-04

37	3	7	0.15521665D-04
37	3	8	0.21000586D-05

*** CPKS converged.

NUCLEUS: 38 C

NUCLEUS	COORD.	ITERATION	ACCURACY
-------	------	---------	--------
38	1	1	0.35831737D-01
38	1	2	0.31543409D-02
38	1	3	0.15322327D-02
38	1	4	0.33807419D-03
38	1	5	0.97012914D-04
38	1	6	0.39152562D-04
38	1	7	0.12504738D-04
38	1	8	0.40868664D-05

*** CPKS converged.

38	2	1	0.11874701D-01
38	2	2	0.43319475D-02
38	2	3	0.13965732D-02
38	2	4	0.26020597D-03
38	2	5	0.87255712D-04
38	2	6	0.42304425D-04

38	2	7	0.13769548D-04
38	2	8	0.22877979D-05

*** CPKS converged.

38	3	1	0.10553349D-01
38	3	2	0.26629136D-02
38	3	3	0.73971503D-03
38	3	4	0.25587074D-03
38	3	5	0.10162771D-03
38	3	6	0.33257431D-04
38	3	7	0.10131726D-04
38	3	8	0.21965612D-05

*** CPKS converged.

NUCLEUS: 39 C

NUCLEUS	COORD.	ITERATION	ACCURACY
39	1	1	0.62889411D-02
39	1	2	0.16782214D-02
39	1	3	0.42411591D-03
39	1	4	0.19803605D-03
39	1	5	0.26163677D-04
39	1	6	0.14611300D-04

39	1	7	0.61328941D-05

*** CPKS converged.

39	2	1	0.64299461D-02
39	2	2	0.39064752D-02
39	2	3	0.65450256D-03
39	2	4	0.13749592D-03
39	2	5	0.82721149D-04
39	2	6	0.24045452D-04
39	2	7	0.73414304D-05

*** CPKS converged.

39	3	1	0.53979649D-02
39	3	2	0.12668088D-02
39	3	3	0.47509850D-03
39	3	4	0.13166675D-03
39	3	5	0.44900217D-04
39	3	6	0.24522325D-04
39	3	7	0.55788989D-05

*** CPKS converged.

NUCLEUS: 69 C

NUCLEUS COORD. ITERATION ACCURACY

------- ------ --------- --------

69	1	1	0.70854962D-01
69	1	2	0.35179046D-02
69	1	3	0.15084517D-02
69	1	4	0.50046861D-03
69	1	5	0.12748095D-03
69	1	6	0.31332143D-04
69	1	7	0.10960311D-04
69	1	8	0.33057681D-05

*** CPKS converged.

69	2	1	0.72674695D-01
69	2	2	0.80424313D-02
69	2	3	0.21102138D-02
69	2	4	0.51389026D-03
69	2	5	0.28458194D-03
69	2	6	0.48597390D-04
69	2	7	0.11483667D-04
69	2	8	0.42174747D-05

*** CPKS converged.

69	3	1	0.10660798D+00
69	3	2	0.53161475D-02
69	3	3	0.28595354D-02
69	3	4	0.74161507D-03
69	3	5	0.27838062D-03
69	3	6	0.89351407D-04
69	3	7	0.16585491D-04
69	3	8	0.50561157D-05

*** CPKS converged.

NUCLEUS: 92 C

NUCLEUS COORD.   ITERATION   ACCURACY

------- ------ --------- --------

92	1	1	0.17937495D-01
92	1	2	0.23292727D-02
92	1	3	0.83837387D-03
92	1	4	0.22091334D-03
92	1	5	0.99402964D-04
92	1	6	0.39682352D-04
92	1	7	0.10020188D-04
92	1	8	0.30430713D-05

*** CPKS converged.

92	2	1	0.37314026D-01
92	2	2	0.77338420D-02
92	2	3	0.22331074D-02
92	2	4	0.24365789D-03
92	2	5	0.17017203D-03
92	2	6	0.72212283D-04
92	2	7	0.10693470D-04
92	2	8	0.39779777D-05

*** CPKS converged.

----

92 3 1 0.17798862D-01

92 3 2 0.29063643D-02

92 3 3 0.76303474D-03

92 3 4 0.16785619D-03

92 3 5 0.55202243D-04

92 3 6 0.36044749D-04

92 3 7 0.14845802D-04

92 3 8 0.37667294D-05

*** CPKS converged.

NUCLEUS: 6 N

NUCLEUS COORD. ITERATION ACCURACY

------- ------ --------- --------

6 1 1 0.29145994D-02

6 1 2 0.91208106D-03

6 1 3 0.70509585D-03

6 1 4 0.26441342D-03

6 1 5 0.55747913D-04

6 1 6 0.14077910D-04

6 1 7 0.83046558D-05

*** CPKS converged.

----

6 2 1 0.37716692D-02

6	2	2	0.16661691D-02
6	2	3	0.97449450D-03
6	2	4	0.17193805D-03
6	2	5	0.38035691D-04
6	2	6	0.15800642D-04
6	2	7	0.99907919D-05

*** CPKS converged.

6	3	1	0.57966158D-02
6	3	2	0.14014167D-02
6	3	3	0.10712168D-02
6	3	4	0.17172671D-03
6	3	5	0.40916086D-04
6	3	6	0.28597307D-04
6	3	7	0.93567924D-05

*** CPKS converged.

NUCLEUS: 40 H

NUCLEUS	COORD.	ITERATION	ACCURACY
40	1	1	0.31775072D-01
40	1	2	0.12116200D-02
40	1	3	0.90206621D-03

40	1	4	0.13542007D-03
40	1	5	0.85241659D-04
40	1	6	0.17351558D-04
40	1	7	0.69458425D-05

*** CPKS converged.

40	2	1	0.56449308D-02
40	2	2	0.18773757D-02
40	2	3	0.26969280D-03
40	2	4	0.11825193D-03
40	2	5	0.78641964D-04
40	2	6	0.99999492D-05

*** CPKS converged.

40	3	1	0.86812228D-02
40	3	2	0.12169387D-02
40	3	3	0.20404371D-03
40	3	4	0.16203763D-03
40	3	5	0.42540301D-04
40	3	6	0.12988006D-04
40	3	7	0.31453961D-05

*** CPKS converged.

NUCLEUS: 41 H

NUCLEUS COORD.   ITERATION   ACCURACY

------- ------   ---------   --------

41	1	1	0.45559708D-02
41	1	2	0.10948884D-02
41	1	3	0.51380297D-03
41	1	4	0.61467505D-04
41	1	5	0.32802366D-04
41	1	6	0.80355230D-05

*** CPKS converged.

41	2	1	0.11264786D-01
41	2	2	0.10899391D-02
41	2	3	0.27181366D-03
41	2	4	0.14033431D-03
41	2	5	0.33219972D-04
41	2	6	0.56825363D-05

*** CPKS converged.

41	3	1	0.83794300D-02
41	3	2	0.26466874D-02
41	3	3	0.53299525D-03
41	3	4	0.21037804D-03
41	3	5	0.81888902D-04
41	3	6	0.22106223D-04
41	3	7	0.72186809D-05

*** CPKS converged.

NUCLEUS: 42 H

NUCLEUS COORD.   ITERATION   ACCURACY

------- ------ --------- --------

42	1	1	0.52627867D-02
42	1	2	0.10717493D-02
42	1	3	0.43582316D-03
42	1	4	0.15214307D-03
42	1	5	0.28854522D-04
42	1	6	0.65505965D-05

*** CPKS converged.

42	2	1	0.55580472D-02
42	2	2	0.33621940D-02
42	2	3	0.41319543D-03
42	2	4	0.18839837D-03
42	2	5	0.91138530D-04
42	2	6	0.18185203D-04
42	2	7	0.10151378D-04
42	2	8	0.18126097D-05

*** CPKS converged.

42	3	1	0.17464416D-02
42	3	2	0.11906248D-02
42	3	3	0.18638561D-03
42	3	4	0.65234110D-04
42	3	5	0.31004442D-04

42 3 6 0.10931474D-04

42 3 7 0.26425788D-05

*** CPKS converged.

NUCLEUS: 43 H

NUCLEUS COORD. ITERATION ACCURACY

------- ------ --------- --------

43 1 1 0.82382511D-02

43 1 2 0.20356370D-02

43 1 3 0.55703633D-03

43 1 4 0.12406586D-03

43 1 5 0.86191570D-04

43 1 6 0.16154473D-04

43 1 7 0.68448536D-05

*** CPKS converged.

----

43 2 1 0.10546942D-01

43 2 2 0.96622127D-03

43 2 3 0.25383670D-03

43 2 4 0.12450829D-03

43 2 5 0.27386352D-04

43 2 6 0.55568727D-05

*** CPKS converged.

43	3	1	0.75920631D-02
43	3	2	0.26342272D-02
43	3	3	0.31380258D-03
43	3	4	0.16254476D-03
43	3	5	0.53773407D-04
43	3	6	0.23150978D-04
43	3	7	0.48056252D-05

*** CPKS converged.

NUCLEUS: 44 H

NUCLEUS	COORD.	ITERATION	ACCURACY
44	1	1	0.52993429D-02
44	1	2	0.21629682D-02
44	1	3	0.28848705D-03
44	1	4	0.10991766D-03
44	1	5	0.56909437D-04
44	1	6	0.12765769D-04
44	1	7	0.48017454D-05

*** CPKS converged.

44	2	1	0.22921070D-02

44 2 2 0.11156352D-02

44 2 3 0.39567863D-03

44 2 4 0.17868047D-03

44 2 5 0.74313387D-04

44 2 6 0.80470516D-05

*** CPKS converged.

----

44 3 1 0.18133071D-02

44 3 2 0.65073485D-03

44 3 3 0.34874481D-03

44 3 4 0.89714133D-04

44 3 5 0.36842116D-04

44 3 6 0.92986627D-05

*** CPKS converged.

NUCLEUS: 45 H

NUCLEUS COORD. ITERATION ACCURACY

------- ------ --------- --------

45 1 1 0.43959556D-02

45 1 2 0.97517331D-03

45 1 3 0.13912978D-03

45 1 4 0.62520526D-04

45 1 5 0.25253277D-04

45 1 6 0.72449781D-05

*** CPKS converged.

----

45 2 1 0.17554904D-02

45 2 2 0.85328760D-03

45 2 3 0.37301960D-03

45 2 4 0.13167296D-03

45 2 5 0.37631723D-04

45 2 6 0.76616020D-05

*** CPKS converged.

----

45 3 1 0.22743536D-02

45 3 2 0.11734000D-02

45 3 3 0.37840056D-03

45 3 4 0.17843367D-03

45 3 5 0.64001206D-04

45 3 6 0.22394109D-04

45 3 7 0.66951995D-05

*** CPKS converged.

NUCLEUS: 46 H

NUCLEUS COORD. ITERATION ACCURACY

------- ------ --------- --------

46	1	1	0.35588736D-02
46	1	2	0.26040797D-02
46	1	3	0.38650501D-03
46	1	4	0.10963658D-03
46	1	5	0.55851413D-04
46	1	6	0.18927027D-04
46	1	7	0.73677139D-05

*** CPKS converged.

46	2	1	0.28160458D-02
46	2	2	0.12938136D-02
46	2	3	0.63943278D-03
46	2	4	0.20231800D-03
46	2	5	0.43699527D-04
46	2	6	0.99562008D-05

*** CPKS converged.

46	3	1	0.17878892D-02
46	3	2	0.72350604D-03
46	3	3	0.23735229D-03
46	3	4	0.11302769D-03
46	3	5	0.59247204D-04
46	3	6	0.10519597D-04
46	3	7	0.28368316D-05

*** CPKS converged.

NUCLEUS: 47 H

NUCLEUS COORD. ITERATION ACCURACY

------- ------ --------- --------

47	1	1	0.96931503D-02
47	1	2	0.34594822D-02
47	1	3	0.64287423D-03
47	1	4	0.16456993D-03
47	1	5	0.77391569D-04
47	1	6	0.20703344D-04
47	1	7	0.80225600D-05

*** CPKS converged.

47	2	1	0.49246143D-02
47	2	2	0.15202822D-02
47	2	3	0.44218673D-03
47	2	4	0.25585056D-03
47	2	5	0.58855163D-04
47	2	6	0.12034264D-04
47	2	7	0.75107082D-05

*** CPKS converged.

47	3	1	0.24020233D-02
47	3	2	0.68983012D-03
47	3	3	0.26636771D-03
47	3	4	0.61934530D-04
47	3	5	0.18633026D-04
47	3	6	0.40599996D-05

*** CPKS converged.

NUCLEUS: 48 H

NUCLEUS COORD.   ITERATION   ACCURACY

-------	------	---------	--------
48	1	1	0.47625613D-02
48	1	2	0.20883493D-02
48	1	3	0.38593627D-03
48	1	4	0.15708522D-03
48	1	5	0.44798197D-04
48	1	6	0.16855035D-04
48	1	7	0.57052900D-05

*** CPKS converged.

48	2	1	0.17458904D-02
48	2	2	0.77740730D-03
48	2	3	0.29844850D-03
48	2	4	0.11396612D-03
48	2	5	0.29586564D-04
48	2	6	0.10728961D-04
48	2	7	0.37130139D-05

*** CPKS converged.

48	3	1	0.17129103D-02

48 3 2 0.76861286D-03

48 3 3 0.24922152D-03

48 3 4 0.94449106D-04

48 3 5 0.31536346D-04

48 3 6 0.98158236D-05

*** CPKS converged.

NUCLEUS: 49 H

NUCLEUS	COORD.	ITERATION	ACCURACY
49	1	1	0.45204545D-02
49	1	2	0.13904985D-02
49	1	3	0.35638152D-03
49	1	4	0.76454310D-04
49	1	5	0.31506155D-04
49	1	6	0.11579417D-04
49	1	7	0.27737778D-05

*** CPKS converged.

49	2	1	0.44323743D-02
49	2	2	0.12923017D-02
49	2	3	0.43804524D-03
49	2	4	0.29095893D-03

49 2 5 0.13258499D-03

49 2 6 0.16840850D-04

49 2 7 0.76562599D-05

*** CPKS converged.

----

49 3 1 0.20096437D-02

49 3 2 0.11758916D-02

49 3 3 0.42560270D-03

49 3 4 0.55444698D-04

49 3 5 0.25714908D-04

49 3 6 0.91476433D-05

*** CPKS converged.

NUCLEUS: 50 H

NUCLEUS	COORD.	ITERATION	ACCURACY
-------	------	---------	--------
50	1	1	0.24718841D-02
50	1	2	0.86245311D-03
50	1	3	0.47699511D-03
50	1	4	0.13378436D-03
50	1	5	0.45215652D-04
50	1	6	0.12316717D-04
50	1	7	0.63948348D-05

*** CPKS converged.

50	2	1	0.18517906D-02
50	2	2	0.65147901D-03
50	2	3	0.28005844D-03
50	2	4	0.27401442D-04
50	2	5	0.20035011D-04
50	2	6	0.97350963D-05

*** CPKS converged.

50	3	1	0.20476565D-02
50	3	2	0.86569508D-03
50	3	3	0.34374967D-03
50	3	4	0.93735185D-04
50	3	5	0.34961548D-04
50	3	6	0.13242026D-04
50	3	7	0.51398109D-05

*** CPKS converged.

NUCLEUS: 51 H

NUCLEUS	COORD.	ITERATION	ACCURACY
51	1	1	0.17662944D-02

51	1	2	0.88405536D-03
51	1	3	0.34495966D-03
51	1	4	0.92987182D-04
51	1	5	0.36166908D-04
51	1	6	0.12402709D-04
51	1	7	0.36556855D-05

*** CPKS converged.

51	2	1	0.18498252D-02
51	2	2	0.71876116D-03
51	2	3	0.38660301D-03
51	2	4	0.34094077D-04
51	2	5	0.20036696D-04
51	2	6	0.12370705D-04
51	2	7	0.37250820D-05

*** CPKS converged.

51	3	1	0.17189521D-02
51	3	2	0.61172609D-03
51	3	3	0.35644129D-03
51	3	4	0.90839526D-04
51	3	5	0.35689325D-04
51	3	6	0.13403265D-04
51	3	7	0.50561436D-05

*** CPKS converged.

NUCLEUS: 52 H

NUCLEUS	COORD.	ITERATION	ACCURACY
52	1	1	0.13386519D-02
52	1	2	0.75334461D-03
52	1	3	0.22066617D-03
52	1	4	0.65485927D-04
52	1	5	0.17339843D-04
52	1	6	0.71502551D-05

*** CPKS converged.

52	2	1	0.20535790D-02
52	2	2	0.92900829D-03
52	2	3	0.39582872D-03
52	2	4	0.86891194D-04
52	2	5	0.29493025D-04
52	2	6	0.15206946D-04
52	2	7	0.47559248D-05

*** CPKS converged.

52	3	1	0.25230971D-02
52	3	2	0.75866311D-03
52	3	3	0.23646391D-03
52	3	4	0.74003912D-04
52	3	5	0.48757054D-04
52	3	6	0.18001087D-04
52	3	7	0.48159383D-05

*** CPKS converged.

NUCLEUS: 53 H

NUCLEUS COORD.   ITERATION   ACCURACY

------- ------   ---------   --------

53	1	1	0.67915771D-02
53	1	2	0.73365635D-03
53	1	3	0.31118855D-03
53	1	4	0.76598255D-04
53	1	5	0.26379707D-04
53	1	6	0.11250641D-04
53	1	7	0.25088900D-05

*** CPKS converged.

53	2	1	0.25632732D-02
53	2	2	0.22136671D-02
53	2	3	0.64327472D-03
53	2	4	0.22101784D-03
53	2	5	0.43469561D-04
53	2	6	0.19362238D-04
53	2	7	0.82365613D-05

*** CPKS converged.

53	3	1	0.30286920D-02

53	3	2	0.84640596D-03
53	3	3	0.20408321D-03
53	3	4	0.72476869D-04
53	3	5	0.24262383D-04
53	3	6	0.94275346D-05

*** CPKS converged.

NUCLEUS: 54 H

NUCLEUS	COORD.	ITERATION	ACCURACY
54	1	1	0.54279053D-02
54	1	2	0.70881603D-03
54	1	3	0.37646444D-03
54	1	4	0.53623443D-04
54	1	5	0.33058994D-04
54	1	6	0.11453489D-04
54	1	7	0.28463883D-05

*** CPKS converged.

54	2	1	0.26043775D-02
54	2	2	0.90775612D-03
54	2	3	0.25669467D-03
54	2	4	0.63490907D-04

54	2	5	0.18993651D-04
54	2	6	0.10866281D-04
54	2	7	0.22258941D-05

*** CPKS converged.

54	3	1	0.22575408D-02
54	3	2	0.16826042D-02
54	3	3	0.32887703D-03
54	3	4	0.18315974D-03
54	3	5	0.10042006D-03
54	3	6	0.17688985D-04
54	3	7	0.63262017D-05

*** CPKS converged.

NUCLEUS: 55 H

NUCLEUS	COORD.	ITERATION	ACCURACY
-------	------	---------	--------
55	1	1	0.62179506D-02
55	1	2	0.11084524D-02
55	1	3	0.58858517D-03
55	1	4	0.94673632D-04
55	1	5	0.42870580D-04
55	1	6	0.18399551D-04

55 1 7 0.41926111D-05

*** CPKS converged.

----

55 2 1 0.18797427D-02

55 2 2 0.10280038D-02

55 2 3 0.38316272D-03

55 2 4 0.11900975D-03

55 2 5 0.19431120D-04

55 2 6 0.10898205D-04

55 2 7 0.52162754D-05

*** CPKS converged.

----

55 3 1 0.32729579D-02

55 3 2 0.87382252D-03

55 3 3 0.26061704D-03

55 3 4 0.77905060D-04

55 3 5 0.31121287D-04

55 3 6 0.11149490D-04

55 3 7 0.41560917D-05

*** CPKS converged.

NUCLEUS: 56 H

NUCLEUS COORD. ITERATION ACCURACY

```
-------  ------    ---------   --------
  56      1        1      0.65570345D-02
  56      1        2      0.15528691D-02
  56      1        3      0.46179723D-03
  56      1        4      0.21623815D-03
  56      1        5      0.44776323D-04
  56      1        6      0.11081361D-04
  56      1        7      0.36450747D-05
*** CPKS converged.
---------------------------------------------
  56      2        1      0.68794173D-02
  56      2        2      0.11060555D-02
  56      2        3      0.22979583D-03
  56      2        4      0.97876139D-04
  56      2        5      0.38035385D-04
  56      2        6      0.94784270D-05
*** CPKS converged.
---------------------------------------------
  56      3        1      0.40596054D-02
  56      3        2      0.19868445D-02
  56      3        3      0.11403150D-02
  56      3        4      0.16356755D-03
  56      3        5      0.12908627D-03
  56      3        6      0.30963274D-04
  56      3        7      0.93378418D-05
*** CPKS converged.
---------------------------------------------
```

NUCLEUS: 57 H

NUCLEUS COORD.   ITERATION   ACCURACY

------- ------   ---------   --------

57	1	1	0.25767786D-02
57	1	2	0.14343699D-02
57	1	3	0.55832981D-03
57	1	4	0.10653499D-03
57	1	5	0.81747036D-04
57	1	6	0.17313413D-04
57	1	7	0.31525373D-05

*** CPKS converged.

57	2	1	0.51896703D-02
57	2	2	0.20492410D-02
57	2	3	0.38117730D-03
57	2	4	0.86305709D-04
57	2	5	0.33451044D-04
57	2	6	0.11626284D-04
57	2	7	0.35083526D-05

*** CPKS converged.

57	3	1	0.25574916D-02
57	3	2	0.57263406D-03
57	3	3	0.24420702D-03
57	3	4	0.98325243D-04
57	3	5	0.26453498D-04
57	3	6	0.16885547D-04

57 3 7 0.87330910D-05

*** CPKS converged.

NUCLEUS: 58 H

NUCLEUS	COORD.	ITERATION	ACCURACY
58	1	1	0.22107524D-02
58	1	2	0.13215999D-02
58	1	3	0.35742151D-03
58	1	4	0.10684234D-03
58	1	5	0.56289086D-04
58	1	6	0.13865789D-04
58	1	7	0.26089007D-05

*** CPKS converged.

58	2	1	0.44472709D-02
58	2	2	0.15734719D-02
58	2	3	0.24936477D-03
58	2	4	0.57696133D-04
58	2	5	0.30822441D-04
58	2	6	0.69594789D-05

*** CPKS converged.

58	3	1	0.34956152D-02
58	3	2	0.77706252D-03
58	3	3	0.32423837D-03
58	3	4	0.10924977D-03
58	3	5	0.24605289D-04
58	3	6	0.16303192D-04
58	3	7	0.63252220D-05

*** CPKS converged.

NUCLEUS: 59 H

NUCLEUS	COORD.	ITERATION	ACCURACY
59	1	1	0.21833502D-02
59	1	2	0.74784852D-03
59	1	3	0.54310674D-03
59	1	4	0.10348832D-03
59	1	5	0.69076410D-04
59	1	6	0.18621582D-04
59	1	7	0.29357128D-05

*** CPKS converged.

59	2	1	0.25661800D-02
59	2	2	0.13156327D-02

59	2	3	0.37053309D-03
59	2	4	0.69547670D-04
59	2	5	0.27990544D-04
59	2	6	0.78259804D-05

*** CPKS converged.

59	3	1	0.23189418D-02
59	3	2	0.59828197D-03
59	3	3	0.18176959D-03
59	3	4	0.87375405D-04
59	3	5	0.18183329D-04
59	3	6	0.17861355D-04
59	3	7	0.47626836D-05

*** CPKS converged.

NUCLEUS: 60 H

NUCLEUS	COORD.	ITERATION	ACCURACY
-------	------	---------	--------
60	1	1	0.18093414D-02
60	1	2	0.89875315D-03
60	1	3	0.39220598D-03
60	1	4	0.80870438D-04
60	1	5	0.43283922D-04

60	1	6	0.13458755D-04
60	1	7	0.19201661D-05

*** CPKS converged.

60	2	1	0.27807294D-02
60	2	2	0.13008371D-02
60	2	3	0.15663010D-03
60	2	4	0.58239363D-04
60	2	5	0.20973523D-04
60	2	6	0.78816095D-05

*** CPKS converged.

60	3	1	0.26925474D-02
60	3	2	0.10903516D-02
60	3	3	0.55396646D-03
60	3	4	0.10526832D-03
60	3	5	0.33889788D-04
60	3	6	0.18171172D-04
60	3	7	0.27921735D-05

*** CPKS converged.

NUCLEUS: 61 H

NUCLEUS COORD. ITERATION ACCURACY

------- ------ --------- --------

61 1 1 0.23117792D-02

61 1 2 0.10943232D-02

61 1 3 0.42200249D-03

61 1 4 0.27408528D-04

61 1 5 0.19023046D-04

61 1 6 0.56948771D-05

*** CPKS converged.

----

61 2 1 0.22759251D-02

61 2 2 0.17986448D-02

61 2 3 0.51392166D-03

61 2 4 0.75493240D-04

61 2 5 0.47430608D-04

61 2 6 0.23486663D-04

61 2 7 0.37338564D-05

*** CPKS converged.

----

61 3 1 0.98474045D-03

61 3 2 0.51972880D-03

61 3 3 0.11852599D-03

61 3 4 0.35664521D-04

61 3 5 0.10192184D-04

61 3 6 0.69379300D-05

*** CPKS converged.

NUCLEUS: 62 H

```
NUCLEUS  COORD.   ITERATION   ACCURACY
-------  ------    ---------   --------
   62      1         1      0.54524613D-02
   62      1         2      0.12262924D-02
   62      1         3      0.11023836D-03
   62      1         4      0.10309482D-03
   62      1         5      0.44554207D-04
   62      1         6      0.44924692D-05
*** CPKS converged.
----------------------------------------------
   62      2         1      0.39794549D-02
   62      2         2      0.25317361D-02
   62      2         3      0.64928138D-03
   62      2         4      0.14743743D-03
   62      2         5      0.57912115D-04
   62      2         6      0.12267577D-04
   62      2         7      0.47007758D-05
*** CPKS converged.
----------------------------------------------
   62      3         1      0.57300182D-02
   62      3         2      0.15443846D-02
   62      3         3      0.15953964D-03
   62      3         4      0.62520311D-04
   62      3         5      0.18231369D-04
   62      3         6      0.65048615D-05
*** CPKS converged.
----------------------------------------------
```

NUCLEUS: 63 H

NUCLEUS COORD.   ITERATION   ACCURACY

------- ------   ---------   --------

63   1   1   0.50339452D-02

63   1   2   0.16390499D-02

63   1   3   0.22309509D-03

63   1   4   0.11515377D-03

63   1   5   0.54836653D-04

63   1   6   0.10242070D-04

63   1   7   0.25609890D-05

*** CPKS converged.

----

63   2   1   0.46742136D-02

63   2   2   0.20014216D-02

63   2   3   0.20869023D-03

63   2   4   0.89717081D-04

63   2   5   0.49051285D-04

63   2   6   0.10552460D-04

63   2   7   0.33963573D-05

*** CPKS converged.

----

63   3   1   0.31378740D-02

63   3   2   0.16380171D-02

63	3	3	0.72112037D-03
63	3	4	0.72996531D-04
63	3	5	0.47600280D-04
63	3	6	0.15250043D-04
63	3	7	0.51680634D-05

*** CPKS converged.

NUCLEUS: 64 H

NUCLEUS	COORD.	ITERATION	ACCURACY
64	1	1	0.27379289D-02
64	1	2	0.20972151D-02
64	1	3	0.45296065D-03
64	1	4	0.13341342D-03
64	1	5	0.72510559D-04
64	1	6	0.24548586D-04
64	1	7	0.48450310D-05

*** CPKS converged.

64	2	1	0.51659159D-02
64	2	2	0.16067955D-02
64	2	3	0.17581575D-03
64	2	4	0.87031657D-04

64 2 5 0.34631657D-04

64 2 6 0.82010786D-05

*** CPKS converged.

----

64 3 1 0.53731628D-02

64 3 2 0.14843251D-02

64 3 3 0.12224109D-03

64 3 4 0.78039473D-04

64 3 5 0.27383693D-04

64 3 6 0.48546647D-05

*** CPKS converged.

NUCLEUS: 65 H

NUCLEUS	COORD.	ITERATION	ACCURACY
65	1	1	0.37050633D-02
65	1	2	0.10868233D-02
65	1	3	0.33828066D-03
65	1	4	0.81457967D-04
65	1	5	0.24447678D-04
65	1	6	0.13096178D-04
65	1	7	0.44177118D-05

*** CPKS converged.

----

65 2 1 0.39840981D-02

65 2 2 0.96216961D-03

65 2 3 0.24653176D-03

65 2 4 0.10457723D-03

65 2 5 0.49123171D-04

65 2 6 0.11605827D-04

65 2 7 0.24431463D-05

*** CPKS converged.

----

65 3 1 0.30841918D-02

65 3 2 0.17596378D-02

65 3 3 0.50813010D-03

65 3 4 0.15993434D-03

65 3 5 0.66694862D-04

65 3 6 0.20883822D-04

65 3 7 0.58821501D-05

*** CPKS converged.

NUCLEUS: 66 H

NUCLEUS COORD. ITERATION ACCURACY

------- ------ --------- --------

66 1 1 0.27685293D-02

66	1	2	0.99463851D-03
66	1	3	0.44852445D-03
66	1	4	0.71178616D-04
66	1	5	0.24909494D-04
66	1	6	0.10231887D-04
66	1	7	0.53563395D-05

*** CPKS converged.

66	2	1	0.18012632D-02
66	2	2	0.85352243D-03
66	2	3	0.27391198D-03
66	2	4	0.70538265D-04
66	2	5	0.55725125D-04
66	2	6	0.14106298D-04
66	2	7	0.31794222D-05

*** CPKS converged.

66	3	1	0.24728342D-02
66	3	2	0.16582701D-02
66	3	3	0.36140997D-03
66	3	4	0.87333322D-04
66	3	5	0.41628125D-04
66	3	6	0.18208093D-04
66	3	7	0.41489376D-05

*** CPKS converged.

NUCLEUS: 67 H

NUCLEUS COORD.   ITERATION   ACCURACY

------- ------   ---------   --------

67	1	1	0.54826086D-02
67	1	2	0.10359744D-02
67	1	3	0.55037142D-03
67	1	4	0.12013331D-03
67	1	5	0.54777992D-04
67	1	6	0.15895349D-04
67	1	7	0.62865232D-05

*** CPKS converged.

67	2	1	0.14255689D-02
67	2	2	0.76441057D-03
67	2	3	0.17366252D-03
67	2	4	0.37971206D-04
67	2	5	0.21837166D-04
67	2	6	0.78248770D-05

*** CPKS converged.

67	3	1	0.14751312D-02
67	3	2	0.11758803D-02
67	3	3	0.28249697D-03
67	3	4	0.48691512D-04
67	3	5	0.27432603D-04
67	3	6	0.76444165D-05

*** CPKS converged.

NUCLEUS: 68 H

NUCLEUS COORD.   ITERATION   ACCURACY

------- ------   ---------   --------

68	1	1	0.25176421D-02
68	1	2	0.10426309D-02
68	1	3	0.35468916D-03
68	1	4	0.71291928D-04
68	1	5	0.24191968D-04
68	1	6	0.13070973D-04
68	1	7	0.43051340D-05

*** CPKS converged.

68	2	1	0.36230353D-02
68	2	2	0.16554275D-02
68	2	3	0.62785805D-03
68	2	4	0.14652762D-03
68	2	5	0.59725536D-04
68	2	6	0.21520846D-04
68	2	7	0.76870085D-05

*** CPKS converged.

68	3	1	0.24659930D-02
68	3	2	0.99433709D-03

68	3	3	0.27215036D-03
68	3	4	0.73824853D-04
68	3	5	0.39107281D-04
68	3	6	0.85805806D-05

*** CPKS converged.

NUCLEUS: 70 H

NUCLEUS	COORD.	ITERATION	ACCURACY
70	1	1	0.19440757D-02
70	1	2	0.45013157D-03
70	1	3	0.31862279D-03
70	1	4	0.12888547D-03
70	1	5	0.32435414D-04
70	1	6	0.46290537D-05

*** CPKS converged.

70	2	1	0.30646661D-02
70	2	2	0.69861789D-03
70	2	3	0.42132684D-03
70	2	4	0.12393234D-03
70	2	5	0.28730672D-04
70	2	6	0.43728884D-05

*** CPKS converged.

70	3	1	0.25063600D-02
70	3	2	0.57634753D-03
70	3	3	0.33422632D-03
70	3	4	0.16544556D-03
70	3	5	0.57704649D-04
70	3	6	0.48833494D-05

*** CPKS converged.

NUCLEUS: 71 H

NUCLEUS	COORD.	ITERATION	ACCURACY
-------	------	---------	--------
71	1	1	0.16233593D-02
71	1	2	0.38521009D-03
71	1	3	0.20255610D-03
71	1	4	0.66590985D-04
71	1	5	0.14962211D-04
71	1	6	0.29807908D-05

*** CPKS converged.

71	2	1	0.18663136D-02
71	2	2	0.49121429D-03

71 2 3 0.17903653D-03

71 2 4 0.68861926D-04

71 2 5 0.25303617D-04

71 2 6 0.54887728D-05

*** CPKS converged.

----

71 3 1 0.61216310D-02

71 3 2 0.77900823D-03

71 3 3 0.22586391D-03

71 3 4 0.19489643D-03

71 3 5 0.10230906D-03

71 3 6 0.87071964D-05

*** CPKS converged.

NUCLEUS: 72 H

NUCLEUS	COORD.	ITERATION	ACCURACY
72	1	1	0.39434697D-02
72	1	2	0.63540083D-03
72	1	3	0.19205424D-03
72	1	4	0.11832893D-03
72	1	5	0.28001488D-04
72	1	6	0.71587502D-05

*** CPKS converged.

72	2	1	0.15637033D-02
72	2	2	0.37566436D-03
72	2	3	0.20779482D-03
72	2	4	0.75469815D-04
72	2	5	0.21603103D-04
72	2	6	0.39580390D-05

*** CPKS converged.

72	3	1	0.17114668D-02
72	3	2	0.29389388D-03
72	3	3	0.23731104D-03
72	3	4	0.12807895D-03
72	3	5	0.32286828D-04
72	3	6	0.36608243D-05

*** CPKS converged.

NUCLEUS: 73 H

NUCLEUS	COORD.	ITERATION	ACCURACY
-------	------	---------	--------
73	1	1	0.18567312D-02
73	1	2	0.46305127D-03

73	1	3	0.35891375D-03
73	1	4	0.12084245D-03
73	1	5	0.33612840D-04
73	1	6	0.80158654D-05

*** CPKS converged.

73	2	1	0.23122102D-02
73	2	2	0.10618245D-02
73	2	3	0.54704518D-03
73	2	4	0.13035528D-03
73	2	5	0.32766235D-04
73	2	6	0.75168604D-05

*** CPKS converged.

73	3	1	0.25930796D-02
73	3	2	0.65620999D-03
73	3	3	0.54171353D-03
73	3	4	0.20865944D-03
73	3	5	0.32669514D-04
73	3	6	0.66318107D-05

*** CPKS converged.

NUCLEUS: 74 H

NUCLEUS	COORD.	ITERATION	ACCURACY
74	1	1	0.21446672D-02
74	1	2	0.50343755D-03
74	1	3	0.37457030D-03
74	1	4	0.13795042D-03
74	1	5	0.43563169D-04
74	1	6	0.41813049D-05

*** CPKS converged.

74	2	1	0.25653417D-02
74	2	2	0.18873450D-02
74	2	3	0.72105865D-03
74	2	4	0.66336265D-04
74	2	5	0.24514061D-04
74	2	6	0.12918822D-04
74	2	7	0.64087685D-05

*** CPKS converged.

74	3	1	0.15894184D-02
74	3	2	0.29541262D-03
74	3	3	0.24797185D-03
74	3	4	0.14283405D-03
74	3	5	0.45346212D-04
74	3	6	0.67951187D-05

*** CPKS converged.

NUCLEUS: 75 H

NUCLEUS COORD.   ITERATION   ACCURACY

------- ------   ---------   --------

75	1	1	0.13779876D-02
75	1	2	0.32314677D-03
75	1	3	0.10301266D-03
75	1	4	0.52807157D-04
75	1	5	0.20914400D-04
75	1	6	0.33751451D-05

*** CPKS converged.

75	2	1	0.24386362D-02
75	2	2	0.11681338D-02
75	2	3	0.25143285D-03
75	2	4	0.44449654D-04
75	2	5	0.17556401D-04
75	2	6	0.10219448D-04
75	2	7	0.37945387D-05

*** CPKS converged.

75	3	1	0.31838074D-02
75	3	2	0.11274731D-02
75	3	3	0.59336465D-03
75	3	4	0.20001200D-03
75	3	5	0.72736122D-04
75	3	6	0.12313227D-04
75	3	7	0.75914555D-05

*** CPKS converged.

NUCLEUS: 76 H

NUCLEUS	COORD.	ITERATION	ACCURACY
76	1	1	0.19879635D-02
76	1	2	0.78097468D-03
76	1	3	0.38563726D-03
76	1	4	0.55554625D-04
76	1	5	0.12079522D-04
76	1	6	0.54756599D-05

*** CPKS converged.

76	2	1	0.19549565D-02
76	2	2	0.88993918D-03
76	2	3	0.53533386D-03
76	2	4	0.85311487D-04
76	2	5	0.17605185D-04
76	2	6	0.97574623D-05

*** CPKS converged.

76	3	1	0.32853914D-02
76	3	2	0.11916179D-02

76	3	3	0.32309387D-03
76	3	4	0.16643734D-03
76	3	5	0.75222829D-04
76	3	6	0.11964929D-04
76	3	7	0.56493187D-05

*** CPKS converged.

NUCLEUS: 77 H

NUCLEUS	COORD.	ITERATION	ACCURACY
77	1	1	0.18656523D-02
77	1	2	0.69294414D-03
77	1	3	0.39309389D-03
77	1	4	0.92262997D-04
77	1	5	0.19118183D-04
77	1	6	0.79168226D-05

*** CPKS converged.

77	2	1	0.24193700D-02
77	2	2	0.13604035D-02
77	2	3	0.84493265D-03
77	2	4	0.15077184D-03
77	2	5	0.38900234D-04

77 2 6 0.82668532D-05

*** CPKS converged.

----

77 3 1 0.21477590D-02

77 3 2 0.57838881D-03

77 3 3 0.37825090D-03

77 3 4 0.11541568D-03

77 3 5 0.11404748D-04

77 3 6 0.50970295D-05

*** CPKS converged.

NUCLEUS: 78 H

NUCLEUS	COORD.	ITERATION	ACCURACY
78	1	1	0.30182320D-02
78	1	2	0.11681633D-02
78	1	3	0.72062893D-03
78	1	4	0.21756971D-03
78	1	5	0.36573458D-04
78	1	6	0.61637509D-05

*** CPKS converged.

----

78 2 1 0.10336696D-02

78 2 2 0.81054749D-03

78 2 3 0.38488254D-03

78 2 4 0.53280664D-04

78 2 5 0.10316867D-04

78 2 6 0.73557458D-05

*** CPKS converged.

----

78 3 1 0.30957010D-02

78 3 2 0.55218513D-03

78 3 3 0.37930421D-03

78 3 4 0.19300234D-03

78 3 5 0.19692298D-04

78 3 6 0.51499477D-05

*** CPKS converged.

NUCLEUS: 79 H

NUCLEUS	COORD.	ITERATION	ACCURACY
79	1	1	0.20053316D-02
79	1	2	0.10109397D-02
79	1	3	0.27929926D-03
79	1	4	0.46782893D-04
79	1	5	0.11569590D-04

79 1 6 0.66728681D-05

*** CPKS converged.

----

79 2 1 0.27120554D-02

79 2 2 0.99345407D-03

79 2 3 0.60912850D-03

79 2 4 0.16631229D-03

79 2 5 0.44307486D-04

79 2 6 0.82898026D-05

*** CPKS converged.

----

79 3 1 0.27771032D-02

79 3 2 0.10478866D-02

79 3 3 0.63941246D-03

79 3 4 0.10847387D-03

79 3 5 0.35974891D-04

79 3 6 0.14465516D-04

79 3 7 0.66430855D-05

*** CPKS converged.

NUCLEUS: 80 H

NUCLEUS COORD. ITERATION ACCURACY

------- ------ --------- --------

80	1	1	0.26851172D-02
80	1	2	0.18014411D-02
80	1	3	0.57236721D-03
80	1	4	0.90254966D-04
80	1	5	0.29881082D-04
80	1	6	0.12889250D-04
80	1	7	0.59812790D-05

*** CPKS converged.

80	2	1	0.23920606D-02
80	2	2	0.67853242D-03
80	2	3	0.36164999D-03
80	2	4	0.12186826D-03
80	2	5	0.21840390D-04
80	2	6	0.33156008D-05

*** CPKS converged.

80	3	1	0.19940999D-02
80	3	2	0.10269980D-02
80	3	3	0.57283863D-03
80	3	4	0.10048312D-03
80	3	5	0.29919259D-04
80	3	6	0.17481973D-04
80	3	7	0.67768106D-05

*** CPKS converged.

NUCLEUS: 81 H

NUCLEUS COORD. ITERATION ACCURACY

------- ------ --------- --------

81	1	1	0.22676901D-02
81	1	2	0.12898969D-02
81	1	3	0.63319403D-03
81	1	4	0.17326637D-03
81	1	5	0.38492749D-04
81	1	6	0.76911001D-05

*** CPKS converged.

81	2	1	0.27549964D-02
81	2	2	0.40525194D-03
81	2	3	0.29950834D-03
81	2	4	0.72285360D-04
81	2	5	0.92794652D-05

*** CPKS converged.

81	3	1	0.12881078D-02
81	3	2	0.12207939D-02
81	3	3	0.45854881D-03
81	3	4	0.57507455D-04
81	3	5	0.30939059D-04
81	3	6	0.16217146D-04
81	3	7	0.51820207D-05

*** CPKS converged.

NUCLEUS: 82 H

NUCLEUS COORD.   ITERATION   ACCURACY

------- ------   ---------   --------

82	1	1	0.15419604D-02
82	1	2	0.56488482D-03
82	1	3	0.30871268D-03
82	1	4	0.69721222D-04
82	1	5	0.30134105D-04
82	1	6	0.13549865D-04
82	1	7	0.44840530D-05

*** CPKS converged.

82	2	1	0.47349663D-02
82	2	2	0.18997907D-02
82	2	3	0.78462353D-03
82	2	4	0.12993276D-03
82	2	5	0.32379022D-04
82	2	6	0.21511803D-04
82	2	7	0.90927542D-05

*** CPKS converged.

82	3	1	0.24919687D-02
82	3	2	0.89425181D-03
82	3	3	0.49850529D-03

82 3 4 0.15769109D-03

82 3 5 0.29090230D-04

82 3 6 0.13957252D-04

82 3 7 0.57412237D-05

*** CPKS converged.

NUCLEUS: 83 H

NUCLEUS	COORD.	ITERATION	ACCURACY
83	1	1	0.21899496D-02
83	1	2	0.71066866D-03
83	1	3	0.41330326D-03
83	1	4	0.10784686D-03
83	1	5	0.37140406D-04
83	1	6	0.14380124D-04
83	1	7	0.49299663D-05

*** CPKS converged.

83	2	1	0.34816903D-02
83	2	2	0.11861973D-02
83	2	3	0.19066827D-03
83	2	4	0.52887979D-04
83	2	5	0.37533676D-04

83 2 6 0.13936307D-04

83 2 7 0.28030371D-05

*** CPKS converged.

----

83 3 1 0.26776238D-02

83 3 2 0.10061780D-02

83 3 3 0.31562489D-03

83 3 4 0.93625606D-04

83 3 5 0.37717186D-04

83 3 6 0.14796125D-04

83 3 7 0.57534253D-05

*** CPKS converged.

NUCLEUS: 84 H

NUCLEUS COORD. ITERATION ACCURACY

------- ------ --------- --------

84 1 1 0.30273322D-02

84 1 2 0.13378176D-02

84 1 3 0.71578234D-03

84 1 4 0.56781935D-04

84 1 5 0.33056172D-04

84 1 6 0.20547753D-04

84 1 7 0.57965202D-05

*** CPKS converged.

84	2	1	0.11889444D-01
84	2	2	0.25093117D-02
84	2	3	0.32711785D-03
84	2	4	0.11327824D-03
84	2	5	0.66130535D-04
84	2	6	0.24227409D-04
84	2	7	0.44070657D-05

*** CPKS converged.

84	3	1	0.51431850D-02
84	3	2	0.20378912D-02
84	3	3	0.66069198D-03
84	3	4	0.75280808D-04
84	3	5	0.38158957D-04
84	3	6	0.24755508D-04
84	3	7	0.85508205D-05

*** CPKS converged.

NUCLEUS: 85 H

NUCLEUS COORD. ITERATION ACCURACY

------- ------ --------- --------

85	1	1	0.20430270D-02
85	1	2	0.73570043D-03
85	1	3	0.39783591D-03
85	1	4	0.16604953D-03
85	1	5	0.35589568D-04
85	1	6	0.53014985D-05

*** CPKS converged.

85	2	1	0.43222519D-02
85	2	2	0.22124830D-02
85	2	3	0.50830291D-03
85	2	4	0.10237566D-03
85	2	5	0.32347991D-04
85	2	6	0.16997801D-04
85	2	7	0.67824938D-05

*** CPKS converged.

85	3	1	0.18728087D-02
85	3	2	0.57688132D-03
85	3	3	0.24331975D-03
85	3	4	0.11684960D-03
85	3	5	0.29172343D-04
85	3	6	0.13592166D-04
85	3	7	0.74770934D-05

*** CPKS converged.

NUCLEUS: 86 H

NUCLEUS COORD.   ITERATION   ACCURACY

------- ------ --------- --------

86	1	1	0.14279402D-01
86	1	2	0.29864888D-02
86	1	3	0.91311908D-03
86	1	4	0.33922287D-03
86	1	5	0.79528429D-04
86	1	6	0.26121478D-04
86	1	7	0.51755961D-05

*** CPKS converged.

86	2	1	0.42043859D-02
86	2	2	0.12491530D-02
86	2	3	0.23290639D-03
86	2	4	0.47494867D-04
86	2	5	0.36210119D-04
86	2	6	0.97386499D-05

*** CPKS converged.

86	3	1	0.16315947D-01
86	3	2	0.31489095D-02
86	3	3	0.13729236D-02
86	3	4	0.17247656D-03
86	3	5	0.54757398D-04
86	3	6	0.31569279D-04
86	3	7	0.73484182D-05

*** CPKS converged.

NUCLEUS: 88 H

NUCLEUS	COORD.	ITERATION	ACCURACY
88	1	1	0.37751571D-02
88	1	2	0.93595291D-03
88	1	3	0.67689139D-03
88	1	4	0.14765026D-03
88	1	5	0.55645779D-04
88	1	6	0.29592215D-04
88	1	7	0.55963114D-05

*** CPKS converged.

88	2	1	0.52684772D-02
88	2	2	0.11749767D-02
88	2	3	0.65065767D-03
88	2	4	0.16663781D-03
88	2	5	0.63547179D-04
88	2	6	0.30516700D-04
88	2	7	0.96947694D-05

*** CPKS converged.

88	3	1	0.43904989D-02

88	3	2	0.23789325D-02
88	3	3	0.46778160D-03
88	3	4	0.14508358D-03
88	3	5	0.55121206D-04
88	3	6	0.20769399D-04
88	3	7	0.74813800D-05

*** CPKS converged.

NUCLEUS: 89 H

NUCLEUS	COORD.	ITERATION	ACCURACY
89	1	1	0.31774693D-02
89	1	2	0.13985391D-02
89	1	3	0.52815983D-03
89	1	4	0.16894766D-03
89	1	5	0.75325360D-04
89	1	6	0.84638114D-05

*** CPKS converged.

89	2	1	0.46683277D-02
89	2	2	0.90178525D-03
89	2	3	0.42797505D-03
89	2	4	0.69143895D-04

89	2	5	0.45145698D-04
89	2	6	0.12843971D-04
89	2	7	0.32333377D-05

*** CPKS converged.

89	3	1	0.47557336D-02
89	3	2	0.25755953D-02
89	3	3	0.31739002D-03
89	3	4	0.11258774D-03
89	3	5	0.58803561D-04
89	3	6	0.11955626D-04
89	3	7	0.61779930D-05

*** CPKS converged.

NUCLEUS: 90 H

NUCLEUS	COORD.	ITERATION	ACCURACY
90	1	1	0.37724685D-02
90	1	2	0.11588223D-02
90	1	3	0.13417850D-03
90	1	4	0.51093075D-04
90	1	5	0.81683601D-05

*** CPKS converged.

90	2	1	0.42167029D-02
90	2	2	0.21791098D-02
90	2	3	0.30812262D-03
90	2	4	0.68086306D-04
90	2	5	0.48319795D-04
90	2	6	0.13847798D-04
90	2	7	0.32100089D-05

*** CPKS converged.

90	3	1	0.54532934D-02
90	3	2	0.29136540D-02
90	3	3	0.28149359D-03
90	3	4	0.11933253D-03
90	3	5	0.67531924D-04
90	3	6	0.13054230D-04
90	3	7	0.45507504D-05

*** CPKS converged.

NUCLEUS: 91 H

NUCLEUS	COORD.	ITERATION	ACCURACY
-------	------	---------	--------
91	1	1	0.79056951D-02

91	1	2	0.25201092D-02
91	1	3	0.65135905D-03
91	1	4	0.18734463D-03
91	1	5	0.56828271D-04
91	1	6	0.26835856D-04
91	1	7	0.11244885D-04
91	1	8	0.14889307D-05

*** CPKS converged.

91	2	1	0.23885746D-02
91	2	2	0.75440076D-03
91	2	3	0.19713679D-03
91	2	4	0.30984045D-04
91	2	5	0.12199065D-04
91	2	6	0.36483647D-05

*** CPKS converged.

91	3	1	0.46838735D-02
91	3	2	0.20093212D-02
91	3	3	0.47918261D-03
91	3	4	0.15376608D-03
91	3	5	0.43289543D-04
91	3	6	0.70458669D-05

*** CPKS converged.

NUCLEUS: 94 H

```
NUCLEUS  COORD.   ITERATION   ACCURACY
-------  ------    ---------   --------
  94      1         1       0.20159727D-02
  94      1         2       0.12736314D-02
  94      1         3       0.38329351D-03
  94      1         4       0.74770874D-04
  94      1         5       0.52252997D-04
  94      1         6       0.95498460D-05
*** CPKS converged.
---------------------------------------------
  94      2         1       0.22756230D-02
  94      2         2       0.75205460D-03
  94      2         3       0.40249592D-03
  94      2         4       0.72610234D-04
  94      2         5       0.21027992D-04
  94      2         6       0.14153924D-04
  94      2         7       0.47667851D-05
*** CPKS converged.
---------------------------------------------
  94      3         1       0.27944941D-02
  94      3         2       0.16487425D-02
  94      3         3       0.52595608D-03
  94      3         4       0.79078614D-04
  94      3         5       0.43514717D-04
  94      3         6       0.14077153D-04
  94      3         7       0.47119653D-05
*** CPKS converged.
---------------------------------------------
```

NUCLEUS: 95 H

NUCLEUS COORD. ITERATION ACCURACY

------- ------ --------- --------

95	1	1	0.20818605D-02
95	1	2	0.16399776D-02
95	1	3	0.32554584D-03
95	1	4	0.10497699D-03
95	1	5	0.50702215D-04
95	1	6	0.12926382D-04
95	1	7	0.35902823D-05

*** CPKS converged.

95	2	1	0.22005194D-02
95	2	2	0.76599112D-03
95	2	3	0.36450872D-03
95	2	4	0.63917931D-04
95	2	5	0.34940046D-04
95	2	6	0.88327452D-05

*** CPKS converged.

95	3	1	0.22348327D-02
95	3	2	0.10905508D-02
95	3	3	0.35953308D-03

95	3	4	0.51423516D-04
95	3	5	0.43889172D-04
95	3	6	0.17237812D-04
95	3	7	0.50908234D-05

*** CPKS converged.

NUCLEUS: 96 H

NUCLEUS	COORD.	ITERATION	ACCURACY
96	1	1	0.17150817D-02
96	1	2	0.12013848D-02
96	1	3	0.39017119D-03
96	1	4	0.66752378D-04
96	1	5	0.46546113D-04
96	1	6	0.96027238D-05

*** CPKS converged.

96	2	1	0.34078793D-02
96	2	2	0.14364374D-02
96	2	3	0.41714069D-03
96	2	4	0.14368436D-03
96	2	5	0.57366077D-04
96	2	6	0.13911259D-04

```
  96      2         7      0.64507307D-05
*** CPKS converged.
---------------------------------------------
  96      3         1      0.32424287D-02
  96      3         2      0.19149061D-02
  96      3         3      0.43660340D-03
  96      3         4      0.97040174D-04
  96      3         5      0.41389408D-04
  96      3         6      0.13171379D-04
  96      3         7      0.70107703D-05
*** CPKS converged.
---------------------------------------------

===================  end of solving CPKS equations  ===================

*********************************************************************************
****************

                  *****************************************
                  * F R E Q U E N C Y   A N A L Y S I S *
                  *****************************************

 Atomic Masses:
 ---------------
   1. O          15.99491400
   2. C          12.00000000
   3. C          12.00000000
```

4. C 12.00000000

5. O 15.99491400

6. N 14.00307400

7. O 15.99491400

8. C 12.00000000

9. C 12.00000000

10. C 12.00000000

11. O 15.99491400

12. C 12.00000000

13. O 15.99491400

14. C 12.00000000

15. C 12.00000000

16. O 15.99491400

17. C 12.00000000

18. C 12.00000000

19. O 15.99491400

20. C 12.00000000

21. C 12.00000000

22. C 12.00000000

23. C 12.00000000

24. C 12.00000000

25. C 12.00000000

26. C 12.00000000

27. C 12.00000000

28. C 12.00000000

29. C 12.00000000

30. C 12.00000000

31. O 15.99491400

32. C 12.00000000

33. C 12.00000000

34. C 12.00000000

35. C	12.00000000
36. C	12.00000000
37. C	12.00000000
38. C	12.00000000
39. C	12.00000000
40. H	1.00782500
41. H	1.00782500
42. H	1.00782500
43. H	1.00782500
44. H	1.00782500
45. H	1.00782500
46. H	1.00782500
47. H	1.00782500
48. H	1.00782500
49. H	1.00782500
50. H	1.00782500
51. H	1.00782500
52. H	1.00782500
53. H	1.00782500
54. H	1.00782500
55. H	1.00782500
56. H	1.00782500
57. H	1.00782500
58. H	1.00782500
59. H	1.00782500
60. H	1.00782500
61. H	1.00782500
62. H	1.00782500
63. H	1.00782500
64. H	1.00782500
65. H	1.00782500

66. H	1.00782500
67. H	1.00782500
68. H	1.00782500
69. C	12.00000000
70. H	1.00782500
71. H	1.00782500
72. H	1.00782500
73. H	1.00782500
74. H	1.00782500
75. H	1.00782500
76. H	1.00782500
77. H	1.00782500
78. H	1.00782500
79. H	1.00782500
80. H	1.00782500
81. H	1.00782500
82. H	1.00782500
83. H	1.00782500
84. H	1.00782500
85. H	1.00782500
86. H	1.00782500
87. O	15.99491400
88. H	1.00782500
89. H	1.00782500
90. H	1.00782500
91. H	1.00782500
92. C	12.00000000
93. O	15.99491400
94. H	1.00782500
95. H	1.00782500
96. H	1.00782500

Normal Modes in Symmetry Displacements *** (cartesians, not mass-weighted) ***

=== A ===

Symmetry Displacements

	1			2			3		
1.O	0.246	0.000	0.000	0.000	0.246	0.001	0.000	0.000	0.246
2.C	-0.009	0.000	0.000	0.000	-0.009	0.001	0.000	0.002	-0.008
3.C	-0.009	0.001	0.001	0.000	-0.009	0.001	0.000	0.002	-0.007
4.C	-0.010	0.001	0.001	0.000	-0.010	0.001	0.000	0.002	-0.007
5.O	-0.003	0.001	0.000	0.000	-0.005	-0.002	-0.001	-0.002	-0.005
6.N	-0.010	0.002	0.002	0.000	-0.010	0.000	0.000	0.002	-0.007
7.O	-0.010	-0.000	0.000	0.000	-0.009	0.002	0.001	0.002	-0.008
8.C	-0.007	-0.002	-0.002	0.000	-0.007	-0.000	-0.000	0.001	-0.006
9.C	-0.007	0.000	0.000	-0.000	-0.004	0.002	0.000	-0.002	-0.009
10.C	-0.004	-0.001	-0.001	0.000	-0.006	-0.001	-0.000	-0.000	-0.005
11.O	-0.007	-0.001	-0.001	-0.000	-0.006	0.001	0.000	-0.000	-0.008
12.C	-0.005	-0.002	-0.002	0.000	-0.006	-0.001	-0.000	0.000	-0.006
13.O	-0.004	-0.001	-0.001	-0.000	-0.005	-0.001	-0.000	-0.001	-0.005
14.C	-0.008	-0.001	-0.001	0.000	-0.007	0.001	0.000	0.001	-0.007
15.C	-0.008	-0.001	-0.000	-0.000	-0.006	0.002	0.000	-0.000	-0.008

16.O	-0.007	-0.003	-0.002	0.000	-0.008	0.000	0.000	0.001	-0.007
17.C	-0.007	-0.001	-0.001	0.000	-0.008	-0.001	-0.000	0.001	-0.006
18.C	-0.002	0.001	0.000	-0.000	-0.003	-0.002	-0.001	-0.003	-0.005
19.O	-0.008	0.001	0.001	0.000	-0.008	-0.000	0.000	0.001	-0.006
20.C	-0.004	0.000	-0.000	-0.000	-0.004	-0.001	-0.000	-0.002	-0.006
21.C	-0.003	0.000	-0.000	-0.000	-0.004	-0.002	-0.001	-0.002	-0.005
22.C	-0.007	-0.001	-0.001	0.000	-0.007	-0.000	-0.000	0.001	-0.006
23.C	-0.006	0.000	0.000	-0.000	-0.005	0.001	0.000	-0.002	-0.008
24.C	-0.010	0.003	0.003	0.000	-0.009	0.001	0.000	0.002	-0.008
25.C	-0.007	0.000	0.000	-0.000	-0.006	0.001	0.000	-0.001	-0.008
26.C	-0.011	0.001	0.001	0.000	-0.010	0.002	0.001	0.003	-0.008
27.C	-0.011	-0.000	0.001	0.000	-0.010	0.002	0.001	0.003	-0.008
28.C	-0.012	-0.000	0.000	0.000	-0.010	0.003	0.001	0.003	-0.009
29.C	-0.005	0.001	0.001	-0.000	-0.004	-0.000	-0.000	-0.002	-0.006
30.C	-0.005	-0.003	-0.002	-0.000	-0.006	-0.000	-0.000	-0.000	-0.006
31.O	-0.006	0.002	0.001	-0.000	-0.004	0.001	0.000	-0.003	-0.008
32.C	-0.003	-0.001	-0.001	-0.000	-0.004	-0.002	-0.001	-0.002	-0.005
33.C	-0.005	0.002	0.001	-0.000	-0.005	-0.001	-0.000	-0.002	-0.006
34.C	-0.005	0.001	0.001	-0.000	-0.004	0.001	0.000	-0.002	-0.007
35.C	-0.009	-0.001	-0.000	-0.000	-0.007	0.003	0.001	0.000	-0.009
36.C	-0.002	-0.001	-0.001	0.000	-0.004	-0.003	-0.001	-0.002	-0.004
37.C	-0.009	0.002	0.002	0.000	-0.010	-0.001	0.000	0.003	-0.006
38.C	-0.008	-0.002	-0.002	-0.000	-0.006	0.002	0.000	0.000	-0.008
39.C	-0.002	-0.002	-0.002	0.000	-0.004	-0.003	-0.001	-0.002	-0.004
40.H	-0.004	-0.000	-0.000	-0.000	-0.004	-0.000	-0.000	-0.002	-0.006
41.H	-0.005	0.002	0.002	-0.000	-0.005	-0.001	-0.000	-0.002	-0.006
42.H	-0.004	0.002	0.001	-0.000	-0.005	-0.002	-0.000	-0.002	-0.005
43.H	-0.004	0.002	0.002	-0.000	-0.004	-0.001	-0.000	-0.003	-0.006
44.H	-0.007	0.001	0.001	-0.000	-0.005	0.003	0.000	-0.002	-0.009
45.H	-0.006	0.000	0.000	-0.000	-0.004	0.002	0.000	-0.003	-0.009
46.H	-0.007	-0.000	-0.000	-0.000	-0.005	0.003	0.000	-0.002	-0.009

47.H	-0.006	-0.000	-0.000	-0.000	-0.005	0.001	0.000	-0.002	-0.007
48.H	-0.008	0.001	0.001	-0.000	-0.006	0.002	0.000	-0.001	-0.008
49.H	-0.007	0.000	0.000	-0.000	-0.006	0.000	0.000	-0.001	-0.007
50.H	-0.009	-0.000	0.000	-0.000	-0.007	0.003	0.001	0.000	-0.009
51.H	-0.009	-0.001	-0.001	-0.000	-0.007	0.003	0.001	0.001	-0.009
52.H	-0.009	-0.001	-0.000	-0.000	-0.006	0.003	0.001	-0.000	-0.009
53.H	-0.008	-0.002	-0.002	-0.000	-0.006	0.003	0.001	-0.000	-0.009
54.H	-0.008	-0.002	-0.002	-0.000	-0.007	0.002	0.000	0.001	-0.008
55.H	-0.007	-0.003	-0.002	-0.000	-0.006	0.002	0.000	-0.000	-0.008
56.H	-0.002	-0.001	-0.001	-0.000	-0.003	-0.002	-0.001	-0.003	-0.005
57.H	-0.001	-0.000	-0.001	0.000	-0.004	-0.004	-0.001	-0.002	-0.003
58.H	-0.002	-0.001	-0.001	0.000	-0.005	-0.003	-0.001	-0.001	-0.004
59.H	-0.002	-0.002	-0.002	0.000	-0.005	-0.003	-0.001	-0.001	-0.004
60.H	-0.001	-0.002	-0.002	-0.000	-0.004	-0.003	-0.001	-0.002	-0.004
61.H	-0.001	-0.002	-0.002	0.000	-0.004	-0.004	-0.001	-0.002	-0.003
62.H	-0.001	0.001	-0.000	-0.000	-0.003	-0.003	-0.001	-0.003	-0.004
63.H	-0.002	0.001	-0.000	-0.000	-0.003	-0.002	-0.001	-0.004	-0.005
64.H	-0.002	0.002	0.001	-0.000	-0.003	-0.002	-0.001	-0.003	-0.005
65.H	-0.006	-0.001	-0.001	0.000	-0.007	-0.000	-0.000	0.000	-0.006
66.H	-0.008	-0.001	-0.001	0.000	-0.009	-0.001	-0.000	0.002	-0.006
67.H	-0.007	-0.000	-0.000	0.000	-0.008	-0.001	-0.000	0.001	-0.006
68.H	-0.006	-0.001	-0.001	0.000	-0.008	-0.002	-0.000	0.001	-0.005
69.C	-0.007	0.002	0.001	0.000	-0.007	-0.001	-0.000	0.000	-0.006
70.H	-0.012	0.000	0.001	0.000	-0.010	0.004	0.001	0.003	-0.010
71.H	-0.013	-0.000	0.001	0.000	-0.011	0.003	0.001	0.004	-0.009
72.H	-0.012	-0.001	-0.000	0.000	-0.010	0.003	0.001	0.003	-0.009
73.H	-0.011	-0.000	0.000	0.000	-0.010	0.002	0.001	0.003	-0.008
74.H	-0.011	0.001	0.002	0.000	-0.010	0.002	0.001	0.002	-0.009
75.H	-0.012	0.001	0.001	0.000	-0.011	0.002	0.001	0.003	-0.008
76.H	-0.009	0.003	0.003	0.000	-0.008	0.000	0.000	0.001	-0.007
77.H	-0.010	0.003	0.003	0.000	-0.009	0.001	0.001	0.002	-0.008

78.H	-0.010	0.003	0.003	0.000	-0.009	0.001	0.000	0.002	-0.008
79.H	-0.008	0.002	0.002	0.000	-0.009	-0.001	-0.000	0.002	-0.006
80.H	-0.009	0.002	0.002	0.000	-0.011	-0.001	0.000	0.003	-0.006
81.H	-0.009	0.003	0.003	0.000	-0.010	-0.001	0.000	0.003	-0.006
82.H	-0.009	-0.000	-0.000	0.000	-0.009	0.001	0.000	0.002	-0.007
83.H	-0.009	-0.001	-0.001	0.000	-0.008	0.001	0.000	0.001	-0.008
84.H	-0.009	0.001	0.002	0.000	-0.008	0.001	0.000	0.001	-0.008
85.H	-0.010	0.001	0.001	0.000	-0.010	0.000	0.000	0.003	-0.007
86.H	-0.004	0.000	-0.000	0.000	-0.005	-0.002	-0.001	-0.001	-0.005
87.O	-0.004	-0.001	-0.001	0.000	-0.006	-0.002	-0.000	-0.000	-0.005
88.H	-0.005	-0.003	-0.002	0.000	-0.007	-0.001	-0.000	0.001	-0.005
89.H	-0.005	0.002	0.002	0.000	-0.007	-0.002	-0.000	-0.000	-0.005
90.H	-0.006	0.001	0.001	0.000	-0.008	-0.002	-0.000	0.001	-0.005
91.H	-0.006	0.001	0.001	0.000	-0.007	-0.002	-0.000	0.000	-0.005
92.C	-0.006	0.001	0.001	0.000	-0.007	-0.002	-0.000	0.000	-0.005
93.O	-0.007	0.002	0.002	0.000	-0.007	-0.000	0.000	-0.000	-0.007
94.H	-0.005	-0.002	-0.002	-0.000	-0.005	0.000	-0.000	-0.001	-0.006
95.H	-0.006	-0.003	-0.003	-0.000	-0.006	0.000	-0.000	0.000	-0.006
96.H	-0.004	-0.003	-0.003	-0.000	-0.005	-0.001	-0.000	-0.001	-0.006

	4			5			6		
1.O	0.000	0.000	0.000	0.000	0.000	0.000	0.000	0.000	0.000
2.C	-0.002	0.000	-0.001	0.000	-0.003	-0.002	0.001	-0.005	-0.005
3.C	-0.003	-0.003	-0.002	-0.000	-0.005	-0.001	0.001	-0.004	-0.006
4.C	-0.002	-0.003	-0.002	-0.000	-0.003	-0.001	0.002	-0.007	-0.006
5.O	0.243	-0.002	0.001	0.000	0.245	0.005	0.000	0.000	0.243
6.N	-0.002	-0.006	-0.003	-0.001	-0.004	-0.000	0.002	-0.006	-0.008
7.O	0.001	0.001	-0.001	0.001	-0.003	-0.004	0.000	-0.006	-0.002
8.C	-0.008	0.006	0.002	-0.001	-0.004	-0.000	0.001	-0.003	-0.006

9.C	-0.002	-0.001	-0.001	0.004	-0.011	-0.004	-0.004	0.006	-0.001
10.C	-0.012	0.004	0.002	-0.002	-0.007	0.002	0.001	0.001	-0.010
11.O	-0.004	0.004	0.001	0.002	-0.006	-0.003	-0.002	0.001	-0.002
12.C	-0.010	0.007	0.003	-0.001	-0.005	0.001	0.001	-0.001	-0.007
13.O	-0.013	0.004	0.002	-0.001	-0.008	0.002	-0.000	0.003	-0.010
14.C	-0.004	0.003	0.000	0.001	-0.005	-0.002	-0.000	-0.002	-0.004
15.C	-0.003	0.002	-0.000	0.002	-0.006	-0.003	-0.002	-0.000	-0.002
16.O	-0.006	0.008	0.002	-0.000	-0.002	-0.001	0.001	-0.004	-0.004
17.C	-0.008	0.003	0.001	-0.002	-0.003	0.001	0.002	-0.004	-0.008
18.C	-0.017	-0.003	0.001	-0.002	-0.014	0.005	-0.001	0.009	-0.013
19.O	-0.006	-0.003	-0.001	-0.001	-0.005	0.001	0.002	-0.003	-0.009
20.C	-0.011	-0.002	0.000	-0.000	-0.012	0.002	-0.001	0.007	-0.009
21.C	-0.015	-0.001	0.001	-0.001	-0.011	0.004	-0.000	0.007	-0.012
22.C	-0.007	0.003	0.001	-0.001	-0.005	-0.000	0.001	-0.002	-0.006
23.C	-0.005	-0.001	-0.001	0.002	-0.010	-0.002	-0.003	0.005	-0.004
24.C	-0.002	-0.008	-0.004	0.000	-0.006	-0.000	0.001	-0.004	-0.007
25.C	-0.004	-0.001	-0.001	0.002	-0.009	-0.002	-0.002	0.002	-0.004
26.C	0.002	-0.002	-0.002	0.001	-0.002	-0.003	0.001	-0.008	-0.003
27.C	0.002	0.001	-0.001	0.001	-0.001	-0.004	0.001	-0.008	-0.002
28.C	0.005	0.001	-0.002	0.002	-0.000	-0.006	0.000	-0.009	0.001
29.C	-0.010	-0.004	-0.001	0.000	-0.012	0.001	-0.002	0.007	-0.008
30.C	-0.010	0.009	0.003	-0.000	-0.005	-0.000	-0.000	0.000	-0.006
31.O	-0.006	-0.006	-0.002	0.003	-0.014	-0.002	-0.003	0.009	-0.005
32.C	-0.015	0.002	0.002	-0.002	-0.010	0.004	-0.000	0.006	-0.012
33.C	-0.011	-0.006	-0.001	-0.000	-0.013	0.002	-0.001	0.007	-0.010
34.C	-0.007	-0.004	-0.001	0.002	-0.012	-0.001	-0.003	0.007	-0.006
35.C	0.001	0.002	-0.001	0.003	-0.006	-0.005	-0.002	-0.001	0.000
36.C	-0.018	0.003	0.003	-0.003	-0.010	0.006	0.001	0.005	-0.014
37.C	-0.005	-0.006	-0.002	-0.002	-0.004	0.002	0.004	-0.007	-0.010
38.C	-0.002	0.007	0.001	0.002	-0.005	-0.004	-0.002	-0.001	-0.000
39.C	-0.019	0.006	0.004	-0.003	-0.009	0.005	0.001	0.004	-0.014

40.H	-0.011	0.000	0.001	0.000	-0.011	0.001	-0.002	0.007	-0.008
41.H	-0.009	-0.007	-0.002	0.000	-0.012	0.001	-0.001	0.005	-0.010
42.H	-0.013	-0.006	-0.001	-0.001	-0.013	0.003	-0.000	0.007	-0.012
43.H	-0.010	-0.008	-0.002	0.000	-0.014	0.002	-0.002	0.009	-0.010
44.H	-0.001	-0.002	-0.002	0.004	-0.011	-0.005	-0.004	0.006	-0.000
45.H	-0.003	-0.001	-0.001	0.004	-0.012	-0.004	-0.005	0.008	-0.001
46.H	-0.001	0.001	-0.001	0.004	-0.010	-0.005	-0.004	0.005	0.001
47.H	-0.006	0.001	0.000	0.002	-0.010	-0.002	-0.003	0.005	-0.004
48.H	-0.003	-0.002	-0.001	0.002	-0.009	-0.003	-0.002	0.002	-0.003
49.H	-0.006	-0.001	-0.000	0.001	-0.008	-0.001	-0.001	0.002	-0.006
50.H	0.001	0.000	-0.001	0.003	-0.006	-0.005	-0.002	-0.001	-0.000
51.H	0.002	0.004	-0.000	0.003	-0.004	-0.006	-0.002	-0.003	0.001
52.H	0.001	0.002	-0.001	0.004	-0.007	-0.006	-0.003	0.001	0.001
53.H	-0.000	0.007	0.001	0.003	-0.005	-0.006	-0.003	-0.000	0.002
54.H	-0.002	0.008	0.001	0.002	-0.003	-0.004	-0.002	-0.003	0.000
55.H	-0.003	0.008	0.002	0.002	-0.005	-0.004	-0.002	-0.000	-0.000
56.H	-0.015	0.002	0.002	-0.001	-0.011	0.003	-0.001	0.007	-0.011
57.H	-0.020	0.001	0.003	-0.003	-0.011	0.006	0.001	0.007	-0.016
58.H	-0.018	0.002	0.003	-0.004	-0.009	0.006	0.002	0.004	-0.015
59.H	-0.018	0.007	0.004	-0.003	-0.007	0.005	0.001	0.002	-0.013
60.H	-0.019	0.006	0.004	-0.003	-0.009	0.005	0.000	0.006	-0.013
61.H	-0.021	0.006	0.004	-0.004	-0.008	0.007	0.002	0.004	-0.016
62.H	-0.019	-0.003	0.001	-0.002	-0.014	0.006	0.000	0.009	-0.015
63.H	-0.016	-0.003	0.001	-0.001	-0.015	0.004	-0.001	0.011	-0.012
64.H	-0.016	-0.005	-0.000	-0.001	-0.015	0.005	-0.001	0.010	-0.014
65.H	-0.008	0.002	0.001	-0.001	-0.006	0.001	0.000	-0.000	-0.008
66.H	-0.007	0.005	0.001	-0.002	-0.002	0.001	0.002	-0.006	-0.007
67.H	-0.008	0.001	0.001	-0.002	-0.004	0.002	0.002	-0.004	-0.009
68.H	-0.010	0.004	0.002	-0.002	-0.003	0.003	0.003	-0.004	-0.010
69.C	-0.007	-0.005	-0.001	-0.001	-0.007	0.002	0.001	-0.001	-0.010
70.H	0.006	-0.000	-0.002	0.003	-0.002	-0.007	-0.001	-0.007	0.002

71.H	0.006	0.001	-0.002	0.002	0.001	-0.006	0.001	-0.011	0.001
72.H	0.006	0.003	-0.001	0.003	0.000	-0.006	-0.000	-0.009	0.002
73.H	0.001	0.002	-0.001	0.000	0.000	-0.003	0.002	-0.010	-0.003
74.H	0.003	-0.004	-0.003	0.002	-0.003	-0.004	0.000	-0.006	-0.002
75.H	0.002	-0.002	-0.002	0.001	-0.001	-0.003	0.002	-0.010	-0.003
76.H	-0.003	-0.009	-0.003	-0.000	-0.007	0.000	0.001	-0.002	-0.008
77.H	0.001	-0.008	-0.004	0.001	-0.006	-0.002	0.001	-0.004	-0.005
78.H	-0.001	-0.010	-0.004	-0.000	-0.006	-0.000	0.002	-0.004	-0.008
79.H	-0.007	-0.007	-0.002	-0.002	-0.005	0.003	0.003	-0.005	-0.012
80.H	-0.005	-0.005	-0.002	-0.003	-0.002	0.002	0.004	-0.008	-0.011
81.H	-0.005	-0.008	-0.003	-0.002	-0.004	0.002	0.004	-0.007	-0.011
82.H	-0.003	0.002	-0.000	-0.000	-0.002	-0.001	0.002	-0.006	-0.005
83.H	-0.002	0.004	0.001	0.001	-0.003	-0.003	-0.000	-0.004	-0.003
84.H	-0.002	-0.004	-0.002	0.001	-0.006	-0.001	0.000	-0.003	-0.005
85.H	-0.003	-0.002	-0.001	-0.001	-0.002	-0.000	0.003	-0.008	-0.007
86.H	-0.015	-0.001	0.001	-0.002	-0.009	0.004	0.001	0.003	-0.013
87.O	-0.013	0.003	0.002	-0.002	-0.006	0.003	0.002	0.000	-0.011
88.H	-0.011	0.008	0.003	-0.002	-0.004	0.002	0.001	-0.002	-0.008
89.H	-0.012	-0.006	-0.001	-0.002	-0.009	0.004	0.002	0.002	-0.013
90.H	-0.011	-0.004	-0.000	-0.003	-0.006	0.004	0.003	-0.002	-0.013
91.H	-0.011	-0.003	-0.000	-0.002	-0.008	0.003	0.002	0.000	-0.012
92.C	-0.011	-0.005	-0.001	-0.002	-0.008	0.003	0.002	0.000	-0.012
93.O	-0.006	-0.006	-0.002	-0.000	-0.009	0.001	0.000	0.001	-0.008
94.H	-0.008	0.007	0.002	0.001	-0.007	-0.001	-0.001	0.002	-0.005
95.H	-0.008	0.010	0.003	0.000	-0.004	-0.001	-0.000	-0.001	-0.004
96.H	-0.011	0.009	0.004	-0.001	-0.006	0.001	-0.000	0.001	-0.007

	7			8			9		
1.O	0.000	0.000	0.000	0.000	0.000	0.000	0.000	0.000	0.000

2.C	-0.012	0.000	0.001	0.000	-0.012	0.002	0.000	0.004	-0.009
3.C	-0.012	0.002	0.003	0.000	-0.011	0.001	0.000	0.004	-0.008
4.C	-0.014	0.003	0.003	0.001	-0.014	0.001	0.000	0.006	-0.009
5.O	0.000	0.000	0.000	0.000	0.000	0.000	0.000	0.000	0.000
6.N	-0.014	0.005	0.005	0.001	-0.014	0.000	0.000	0.006	-0.008
7.O	0.243	-0.000	0.001	0.000	0.244	0.004	0.000	0.000	0.245
8.C	-0.007	-0.004	-0.004	0.000	-0.009	-0.000	-0.000	0.002	-0.006
9.C	-0.008	0.000	0.000	-0.002	-0.002	0.005	0.002	-0.005	-0.012
10.C	-0.002	-0.003	-0.003	0.000	-0.005	-0.003	-0.001	-0.001	-0.004
11.O	-0.008	-0.003	-0.002	-0.001	-0.006	0.003	0.001	-0.001	-0.010
12.C	-0.004	-0.005	-0.005	0.000	-0.007	-0.002	-0.001	0.000	-0.005
13.O	-0.000	-0.003	-0.003	0.000	-0.003	-0.003	-0.001	-0.003	-0.004
14.C	-0.010	-0.002	-0.001	-0.000	-0.009	0.002	0.000	0.002	-0.009
15.C	-0.010	-0.002	-0.001	-0.001	-0.007	0.004	0.001	0.000	-0.010
16.O	-0.008	-0.006	-0.005	0.000	-0.010	0.001	0.000	0.003	-0.007
17.C	-0.008	-0.002	-0.002	0.001	-0.011	-0.002	-0.001	0.003	-0.005
18.C	0.004	0.002	0.000	0.000	0.001	-0.006	-0.001	-0.007	-0.002
19.O	-0.010	0.003	0.003	0.001	-0.011	-0.001	-0.000	0.003	-0.006
20.C	-0.001	0.001	-0.000	-0.000	-0.001	-0.002	-0.000	-0.005	-0.005
21.C	0.001	0.001	-0.001	0.000	-0.001	-0.005	-0.001	-0.005	-0.003
22.C	-0.007	-0.002	-0.002	0.000	-0.009	-0.000	-0.000	0.002	-0.007
23.C	-0.006	0.001	0.000	-0.001	-0.002	0.003	0.001	-0.004	-0.010
24.C	-0.014	0.006	0.006	0.000	-0.012	0.001	0.000	0.004	-0.009
25.C	-0.008	0.000	0.000	-0.001	-0.005	0.003	0.001	-0.002	-0.009
26.C	-0.017	0.002	0.003	0.000	-0.015	0.004	0.001	0.006	-0.011
27.C	-0.017	-0.000	0.001	0.000	-0.015	0.005	0.001	0.007	-0.011
28.C	-0.019	-0.001	0.001	-0.000	-0.015	0.007	0.002	0.007	-0.013
29.C	-0.002	0.002	0.001	-0.000	-0.001	-0.001	-0.000	-0.005	-0.006
30.C	-0.004	-0.006	-0.006	-0.000	-0.005	-0.001	-0.000	-0.001	-0.006
31.O	-0.005	0.004	0.003	-0.001	0.000	0.002	0.001	-0.006	-0.009
32.C	0.002	-0.001	-0.002	0.000	-0.001	-0.005	-0.001	-0.005	-0.002

33.C	-0.002	0.004	0.003	-0.000	-0.002	-0.002	-0.000	-0.005	-0.005
34.C	-0.004	0.002	0.002	-0.001	-0.001	0.001	0.001	-0.005	-0.008
35.C	-0.012	-0.001	-0.000	-0.001	-0.008	0.006	0.001	0.001	-0.012
36.C	0.004	-0.002	-0.003	0.001	-0.002	-0.007	-0.002	-0.004	-0.000
37.C	-0.012	0.005	0.005	0.001	-0.014	-0.002	-0.001	0.006	-0.006
38.C	-0.010	-0.005	-0.004	-0.001	-0.007	0.005	0.001	0.000	-0.011
39.C	0.004	-0.004	-0.005	0.001	-0.002	-0.008	-0.002	-0.004	0.000
40.H	-0.001	-0.000	-0.001	-0.001	-0.001	-0.001	-0.000	-0.005	-0.006
41.H	-0.004	0.005	0.003	-0.000	-0.003	-0.002	-0.000	-0.004	-0.006
42.H	-0.001	0.004	0.003	0.000	-0.002	-0.004	-0.001	-0.005	-0.004
43.H	-0.002	0.006	0.004	-0.000	-0.000	-0.002	-0.000	-0.006	-0.006
44.H	-0.009	0.002	0.002	-0.002	-0.002	0.006	0.002	-0.005	-0.013
45.H	-0.006	0.001	0.000	-0.002	0.000	0.005	0.002	-0.006	-0.012
46.H	-0.009	-0.001	-0.001	-0.002	-0.002	0.006	0.002	-0.004	-0.012
47.H	-0.005	-0.001	-0.001	-0.001	-0.002	0.002	0.001	-0.004	-0.009
48.H	-0.009	0.002	0.002	-0.001	-0.005	0.003	0.001	-0.002	-0.010
49.H	-0.007	0.001	0.001	-0.000	-0.005	0.001	0.000	-0.002	-0.008
50.H	-0.013	-0.000	0.001	-0.001	-0.008	0.006	0.002	0.001	-0.012
51.H	-0.014	-0.003	-0.001	-0.001	-0.009	0.007	0.002	0.002	-0.013
52.H	-0.012	-0.001	-0.000	-0.001	-0.006	0.007	0.002	-0.001	-0.013
53.H	-0.011	-0.005	-0.003	-0.001	-0.006	0.007	0.002	-0.000	-0.012
54.H	-0.011	-0.006	-0.004	-0.001	-0.009	0.005	0.001	0.002	-0.011
55.H	-0.008	-0.006	-0.005	-0.001	-0.006	0.004	0.001	-0.000	-0.010
56.H	0.003	-0.002	-0.003	-0.000	0.000	-0.004	-0.001	-0.006	-0.003
57.H	0.006	-0.001	-0.003	0.001	-0.000	-0.009	-0.002	-0.005	0.001
58.H	0.003	-0.002	-0.003	0.001	-0.003	-0.008	-0.002	-0.003	0.000
59.H	0.003	-0.005	-0.006	0.001	-0.004	-0.007	-0.002	-0.002	-0.001
60.H	0.005	-0.005	-0.005	0.001	-0.001	-0.007	-0.002	-0.005	-0.000
61.H	0.006	-0.004	-0.006	0.001	-0.002	-0.009	-0.002	-0.003	0.002
62.H	0.005	0.002	-0.000	0.000	0.001	-0.008	-0.002	-0.007	-0.001
63.H	0.004	0.002	-0.000	-0.000	0.003	-0.005	-0.001	-0.008	-0.003

64.H	0.003	0.004	0.002	-0.000	0.001	-0.006	-0.001	-0.008	-0.002
65.H	-0.006	-0.001	-0.001	0.000	-0.007	-0.001	-0.000	0.000	-0.006
66.H	-0.009	-0.003	-0.002	0.001	-0.012	-0.001	-0.001	0.005	-0.006
67.H	-0.008	-0.001	-0.000	0.001	-0.011	-0.002	-0.001	0.003	-0.005
68.H	-0.006	-0.003	-0.002	0.001	-0.010	-0.004	-0.001	0.003	-0.004
69.C	-0.008	0.004	0.003	0.000	-0.008	-0.002	-0.000	0.001	-0.006
70.H	-0.020	0.000	0.002	-0.001	-0.014	0.008	0.002	0.006	-0.014
71.H	-0.021	-0.001	0.001	-0.000	-0.017	0.008	0.002	0.009	-0.014
72.H	-0.019	-0.002	-0.000	-0.000	-0.015	0.008	0.002	0.007	-0.014
73.H	-0.017	-0.001	0.000	0.000	-0.016	0.004	0.001	0.008	-0.010
74.H	-0.017	0.003	0.004	-0.000	-0.014	0.005	0.001	0.005	-0.012
75.H	-0.018	0.002	0.003	0.000	-0.016	0.004	0.001	0.008	-0.011
76.H	-0.012	0.007	0.006	0.000	-0.010	0.000	0.000	0.002	-0.008
77.H	-0.015	0.006	0.006	0.000	-0.012	0.003	0.001	0.004	-0.010
78.H	-0.014	0.008	0.007	0.001	-0.013	0.001	0.000	0.004	-0.009
79.H	-0.010	0.005	0.005	0.001	-0.013	-0.003	-0.001	0.005	-0.005
80.H	-0.013	0.004	0.004	0.001	-0.016	-0.002	-0.001	0.007	-0.005
81.H	-0.013	0.006	0.006	0.001	-0.015	-0.002	-0.001	0.006	-0.006
82.H	-0.012	-0.001	-0.000	0.000	-0.013	0.001	0.000	0.005	-0.008
83.H	-0.011	-0.003	-0.002	-0.000	-0.010	0.003	0.001	0.003	-0.009
84.H	-0.012	0.003	0.003	-0.000	-0.010	0.002	0.001	0.002	-0.009
85.H	-0.014	0.002	0.002	0.001	-0.015	0.000	-0.000	0.007	-0.008
86.H	-0.000	0.000	-0.001	0.001	-0.004	-0.006	-0.001	-0.002	-0.002
87.O	-0.002	-0.002	-0.003	0.001	-0.006	-0.005	-0.001	-0.000	-0.003
88.H	-0.004	-0.006	-0.005	0.001	-0.008	-0.003	-0.001	0.002	-0.004
89.H	-0.004	0.005	0.003	0.001	-0.006	-0.005	-0.001	-0.001	-0.003
90.H	-0.005	0.003	0.002	0.001	-0.009	-0.005	-0.001	0.002	-0.003
91.H	-0.004	0.002	0.001	0.001	-0.007	-0.004	-0.001	0.000	-0.004
92.C	-0.005	0.003	0.003	0.001	-0.008	-0.004	-0.001	0.000	-0.004
93.O	-0.008	0.005	0.004	0.000	-0.007	-0.000	0.000	-0.000	-0.007
94.H	-0.004	-0.005	-0.005	-0.000	-0.004	0.001	0.000	-0.002	-0.007

95.H	-0.005	-0.007	-0.006	-0.000	-0.007	0.000	-0.000	0.001	-0.007
96.H	-0.002	-0.007	-0.006	0.000	-0.005	-0.002	-0.001	-0.001	-0.005

	10			11			12		
1.O	0.000	0.000	0.000	0.000	0.000	0.000	0.000	0.000	0.000
2.C	-0.007	-0.000	0.000	-0.001	-0.006	-0.000	-0.003	0.003	-0.008
3.C	-0.006	0.002	-0.001	-0.001	-0.004	-0.001	-0.003	0.002	-0.006
4.C	-0.005	0.002	-0.002	-0.000	-0.003	-0.001	-0.005	0.004	-0.005
5.O	0.000	0.000	0.000	0.000	0.000	0.000	0.000	0.000	0.000
6.N	-0.004	0.004	-0.003	0.000	-0.000	-0.001	-0.006	0.003	-0.003
7.O	0.000	0.000	0.000	0.000	0.000	0.000	0.000	0.000	0.000
8.C	-0.006	-0.005	0.002	0.001	-0.012	0.001	-0.001	0.003	-0.011
9.C	-0.015	0.001	0.001	-0.006	-0.008	-0.000	0.007	-0.004	-0.011
10.C	-0.005	-0.003	0.001	0.003	-0.011	0.001	0.000	0.000	-0.008
11.O	0.245	-0.003	0.003	0.000	0.245	0.000	0.000	0.000	0.244
12.C	-0.006	-0.005	0.002	0.002	-0.013	0.001	-0.000	0.002	-0.010
13.O	-0.006	-0.003	0.001	0.003	-0.011	0.001	0.002	-0.002	-0.008
14.C	-0.008	-0.002	0.001	-0.002	-0.009	0.000	-0.000	0.002	-0.010
15.C	-0.011	-0.002	0.002	-0.003	-0.009	-0.000	0.002	0.000	-0.011
16.O	-0.007	-0.006	0.003	0.000	-0.013	0.001	-0.001	0.004	-0.013
17.C	-0.004	-0.003	0.001	0.002	-0.009	0.001	-0.004	0.003	-0.007
18.C	-0.006	0.003	-0.002	0.004	-0.007	0.000	0.004	-0.006	-0.002
19.O	-0.004	0.002	-0.002	0.001	-0.003	-0.001	-0.004	0.002	-0.004
20.C	-0.008	0.002	-0.001	0.001	-0.008	0.000	0.004	-0.004	-0.006
21.C	-0.006	0.001	-0.001	0.003	-0.008	0.000	0.003	-0.004	-0.004
22.C	-0.006	-0.003	0.001	0.001	-0.009	0.001	-0.001	0.002	-0.009
23.C	-0.012	0.001	0.001	-0.004	-0.008	-0.000	0.005	-0.003	-0.010
24.C	-0.006	0.006	-0.004	-0.001	0.001	-0.002	-0.004	0.001	-0.002
25.C	-0.011	0.001	0.001	-0.003	-0.007	-0.000	0.003	-0.001	-0.009

26.C	-0.007	0.001	-0.001	-0.003	-0.003	-0.001	-0.005	0.005	-0.008
27.C	-0.008	-0.001	0.001	-0.003	-0.006	-0.001	-0.004	0.005	-0.010
28.C	-0.010	-0.002	0.002	-0.005	-0.006	-0.001	-0.004	0.006	-0.012
29.C	-0.009	0.003	-0.001	-0.000	-0.006	-0.000	0.004	-0.005	-0.005
30.C	-0.007	-0.006	0.003	0.001	-0.015	0.002	0.002	0.001	-0.012
31.O	-0.013	0.005	-0.001	-0.004	-0.005	-0.001	0.007	-0.006	-0.006
32.C	-0.005	-0.001	0.000	0.004	-0.010	0.001	0.003	-0.003	-0.006
33.C	-0.008	0.005	-0.003	0.001	-0.004	-0.001	0.003	-0.005	-0.002
34.C	-0.012	0.003	-0.001	-0.003	-0.006	-0.001	0.005	-0.005	-0.007
35.C	-0.013	-0.002	0.002	-0.006	-0.009	-0.000	0.002	0.001	-0.013
36.C	-0.003	-0.001	-0.000	0.006	-0.011	0.001	0.001	-0.003	-0.004
37.C	-0.002	0.004	-0.004	0.002	0.000	-0.001	-0.007	0.003	-0.001
38.C	-0.012	-0.005	0.004	-0.004	-0.013	0.001	0.003	0.002	-0.015
39.C	-0.003	-0.004	0.001	0.007	-0.013	0.002	0.001	-0.002	-0.006
40.H	-0.009	0.000	0.000	-0.000	-0.009	0.000	0.005	-0.004	-0.007
41.H	-0.008	0.005	-0.003	0.000	-0.003	-0.001	0.002	-0.004	-0.003
42.H	-0.006	0.005	-0.003	0.002	-0.004	-0.000	0.002	-0.005	-0.001
43.H	-0.009	0.007	-0.003	-0.000	-0.003	-0.001	0.004	-0.007	-0.002
44.H	-0.016	0.002	0.001	-0.007	-0.007	-0.001	0.007	-0.004	-0.011
45.H	-0.016	0.002	0.001	-0.007	-0.008	-0.001	0.008	-0.005	-0.011
46.H	-0.015	-0.001	0.002	-0.007	-0.009	-0.000	0.007	-0.003	-0.013
47.H	-0.012	-0.000	0.001	-0.003	-0.009	0.000	0.005	-0.003	-0.010
48.H	-0.011	0.002	0.000	-0.004	-0.006	-0.001	0.003	-0.002	-0.009
49.H	-0.009	0.001	-0.000	-0.001	-0.007	-0.000	0.002	-0.001	-0.007
50.H	-0.012	-0.000	0.002	-0.006	-0.007	-0.001	0.002	0.001	-0.012
51.H	-0.013	-0.003	0.003	-0.006	-0.010	-0.000	0.002	0.002	-0.014
52.H	-0.014	-0.001	0.003	-0.007	-0.009	-0.000	0.004	-0.000	-0.014
53.H	-0.014	-0.005	0.004	-0.006	-0.013	0.000	0.004	0.001	-0.016
54.H	-0.011	-0.006	0.004	-0.004	-0.013	0.001	0.001	0.003	-0.016
55.H	-0.012	-0.006	0.005	-0.004	-0.014	0.001	0.004	0.001	-0.016
56.H	-0.007	-0.001	0.000	0.003	-0.011	0.001	0.004	-0.004	-0.006

57.H	-0.003	-0.000	-0.001	0.007	-0.010	0.001	0.002	-0.004	-0.003
58.H	-0.002	-0.001	-0.001	0.007	-0.010	0.001	-0.000	-0.002	-0.003
59.H	-0.003	-0.005	0.002	0.006	-0.014	0.002	0.001	-0.000	-0.007
60.H	-0.004	-0.004	0.001	0.006	-0.014	0.002	0.003	-0.003	-0.007
61.H	-0.001	-0.004	0.001	0.008	-0.013	0.002	0.000	-0.001	-0.005
62.H	-0.005	0.003	-0.002	0.005	-0.007	0.001	0.003	-0.006	-0.001
63.H	-0.008	0.003	-0.001	0.003	-0.008	0.000	0.006	-0.007	-0.003
64.H	-0.007	0.005	-0.003	0.003	-0.005	-0.000	0.004	-0.007	-0.001
65.H	-0.006	-0.001	0.000	0.001	-0.009	0.000	-0.000	0.001	-0.008
66.H	-0.003	-0.004	0.001	0.002	-0.010	0.001	-0.005	0.005	-0.009
67.H	-0.003	-0.001	-0.000	0.003	-0.007	0.000	-0.004	0.003	-0.006
68.H	-0.002	-0.003	0.000	0.004	-0.009	0.001	-0.004	0.003	-0.007
69.C	-0.005	0.004	-0.003	0.001	-0.003	-0.001	-0.002	-0.000	-0.003
70.H	-0.011	-0.000	0.001	-0.007	-0.005	-0.001	-0.002	0.005	-0.012
71.H	-0.009	-0.002	0.002	-0.005	-0.006	-0.001	-0.005	0.007	-0.012
72.H	-0.010	-0.003	0.003	-0.006	-0.008	-0.001	-0.003	0.006	-0.014
73.H	-0.006	-0.002	0.001	-0.002	-0.006	-0.000	-0.006	0.006	-0.010
74.H	-0.009	0.002	-0.001	-0.004	-0.002	-0.001	-0.004	0.004	-0.008
75.H	-0.006	0.001	-0.001	-0.002	-0.003	-0.001	-0.006	0.006	-0.007
76.H	-0.006	0.006	-0.004	-0.001	0.001	-0.002	-0.003	0.000	-0.002
77.H	-0.008	0.006	-0.003	-0.003	0.001	-0.002	-0.003	0.001	-0.004
78.H	-0.005	0.007	-0.004	-0.001	0.003	-0.002	-0.005	0.001	-0.001
79.H	-0.002	0.005	-0.004	0.003	-0.000	-0.001	-0.007	0.002	-0.000
80.H	-0.001	0.003	-0.003	0.003	-0.001	-0.001	-0.009	0.005	-0.001
81.H	-0.001	0.006	-0.005	0.003	0.002	-0.001	-0.008	0.003	0.001
82.H	-0.005	-0.002	0.000	-0.000	-0.007	-0.000	-0.004	0.004	-0.008
83.H	-0.008	-0.003	0.002	-0.002	-0.010	0.000	-0.001	0.003	-0.012
84.H	-0.008	0.003	-0.002	-0.002	-0.003	-0.001	-0.002	0.001	-0.006
85.H	-0.004	0.001	-0.001	0.001	-0.004	-0.001	-0.007	0.005	-0.005
86.H	-0.003	0.001	-0.001	0.005	-0.007	0.001	-0.000	-0.002	-0.003
87.O	-0.003	-0.002	0.000	0.004	-0.010	0.001	-0.001	0.000	-0.006

88.H	-0.004	-0.006	0.003	0.003	-0.014	0.002	-0.001	0.003	-0.010
89.H	-0.003	0.005	-0.004	0.004	-0.002	-0.001	-0.002	-0.002	0.000
90.H	-0.001	0.003	-0.003	0.005	-0.003	-0.000	-0.004	0.001	-0.001
91.H	-0.004	0.002	-0.002	0.003	-0.005	-0.000	-0.002	-0.001	-0.003
92.C	-0.003	0.003	-0.003	0.003	-0.003	-0.000	-0.003	-0.001	-0.001
93.O	-0.007	0.005	-0.003	-0.000	-0.002	-0.001	-0.001	-0.001	-0.003
94.H	-0.009	-0.005	0.003	-0.001	-0.014	0.001	0.003	0.000	-0.013
95.H	-0.008	-0.008	0.004	-0.000	-0.016	0.002	0.001	0.002	-0.014
96.H	-0.007	-0.007	0.004	0.002	-0.015	0.002	0.002	0.001	-0.012

	13			14			15		
1.O	0.000	0.000	0.000	0.000	0.000	0.000	0.000	0.000	0.000
2.C	-0.003	-0.000	-0.001	-0.001	-0.004	-0.002	-0.003	-0.003	-0.005
3.C	-0.003	-0.002	-0.002	-0.001	-0.001	-0.002	-0.003	-0.003	-0.004
4.C	-0.001	-0.002	-0.003	-0.001	0.001	-0.002	-0.005	-0.005	-0.004
5.O	0.000	0.000	0.000	0.000	0.000	0.000	0.000	0.000	0.000
6.N	-0.001	-0.004	-0.004	-0.000	0.003	-0.002	-0.005	-0.005	-0.002
7.O	0.000	0.000	0.000	0.000	0.000	0.000	0.000	0.000	0.000
8.C	-0.008	0.004	0.003	0.001	-0.011	0.001	-0.000	-0.000	-0.011
9.C	-0.007	-0.000	-0.000	-0.005	-0.011	-0.004	0.004	0.003	-0.004
10.C	-0.012	0.003	0.003	0.003	-0.012	0.003	0.002	0.002	-0.013
11.O	0.000	0.000	0.000	0.000	0.000	0.000	0.000	0.000	0.000
12.C	-0.010	0.004	0.004	0.002	-0.013	0.002	0.001	0.001	-0.013
13.O	0.244	0.002	0.003	0.000	0.243	0.003	0.000	0.000	0.244
14.C	-0.005	0.002	0.001	-0.001	-0.008	-0.001	-0.001	-0.001	-0.008
15.C	-0.005	0.001	0.001	-0.003	-0.009	-0.002	0.001	0.000	-0.007
16.O	-0.006	0.005	0.004	0.000	-0.012	0.001	-0.001	-0.001	-0.012
17.C	-0.007	0.002	0.002	0.002	-0.007	0.002	-0.002	-0.002	-0.010
18.C	-0.017	-0.002	-0.000	0.004	-0.012	0.003	0.005	0.005	-0.010

19.O	-0.005	-0.002	-0.002	0.001	-0.002	-0.000	-0.003	-0.003	-0.005
20.C	-0.013	-0.001	0.000	0.001	-0.011	0.001	0.004	0.004	-0.009
21.C	-0.014	-0.000	0.000	0.003	-0.011	0.003	0.004	0.004	-0.010
22.C	-0.007	0.002	0.002	0.001	-0.009	0.001	-0.001	-0.001	-0.010
23.C	-0.008	-0.000	-0.000	-0.003	-0.011	-0.002	0.003	0.003	-0.006
24.C	-0.001	-0.005	-0.005	-0.001	0.004	-0.003	-0.004	-0.004	-0.001
25.C	-0.007	-0.000	-0.000	-0.003	-0.009	-0.002	0.002	0.001	-0.006
26.C	0.001	-0.002	-0.003	-0.003	0.001	-0.003	-0.005	-0.005	-0.002
27.C	0.001	0.000	-0.001	-0.003	-0.002	-0.003	-0.005	-0.005	-0.004
28.C	0.003	0.001	-0.001	-0.005	-0.002	-0.005	-0.005	-0.005	-0.003
29.C	-0.011	-0.002	-0.001	0.000	-0.009	0.000	0.004	0.003	-0.007
30.C	-0.010	0.005	0.005	0.001	-0.016	0.002	0.002	0.002	-0.013
31.O	-0.010	-0.003	-0.002	-0.003	-0.009	-0.002	0.005	0.004	-0.003
32.C	-0.015	0.001	0.002	0.004	-0.014	0.004	0.004	0.004	-0.012
33.C	-0.012	-0.003	-0.003	0.001	-0.007	0.001	0.003	0.003	-0.006
34.C	-0.010	-0.002	-0.001	-0.002	-0.010	-0.001	0.004	0.003	-0.005
35.C	-0.003	0.001	0.001	-0.005	-0.009	-0.004	0.000	-0.000	-0.005
36.C	-0.017	0.002	0.003	0.006	-0.014	0.005	0.004	0.004	-0.014
37.C	-0.003	-0.004	-0.004	0.002	0.004	-0.000	-0.006	-0.005	-0.003
38.C	-0.005	0.004	0.003	-0.004	-0.013	-0.002	0.001	0.001	-0.009
39.C	-0.017	0.004	0.004	0.006	-0.016	0.006	0.004	0.004	-0.016
40.H	-0.012	0.000	0.001	0.000	-0.013	0.001	0.004	0.004	-0.009
41.H	-0.010	-0.004	-0.003	0.000	-0.005	-0.000	0.002	0.002	-0.005
42.H	-0.013	-0.003	-0.003	0.002	-0.007	0.002	0.003	0.003	-0.007
43.H	-0.012	-0.005	-0.004	0.000	-0.007	0.000	0.004	0.004	-0.004
44.H	-0.006	-0.001	-0.001	-0.006	-0.010	-0.004	0.004	0.003	-0.003
45.H	-0.008	-0.000	-0.000	-0.005	-0.013	-0.004	0.005	0.005	-0.004
46.H	-0.006	0.001	0.001	-0.006	-0.012	-0.004	0.004	0.003	-0.005
47.H	-0.009	0.001	0.001	-0.002	-0.012	-0.001	0.004	0.003	-0.007
48.H	-0.006	-0.001	-0.001	-0.003	-0.007	-0.003	0.001	0.001	-0.004
49.H	-0.008	-0.001	-0.000	-0.001	-0.008	-0.001	0.001	0.001	-0.006

50.H	-0.002	0.000	-0.000	-0.005	-0.007	-0.004	-0.001	-0.001	-0.004
51.H	-0.002	0.002	0.001	-0.005	-0.009	-0.004	-0.001	-0.001	-0.006
52.H	-0.003	0.001	0.000	-0.006	-0.010	-0.005	0.001	0.001	-0.004
53.H	-0.004	0.004	0.003	-0.005	-0.013	-0.003	0.001	0.001	-0.008
54.H	-0.004	0.005	0.004	-0.003	-0.013	-0.002	-0.000	-0.000	-0.009
55.H	-0.006	0.005	0.004	-0.003	-0.015	-0.002	0.002	0.002	-0.010
56.H	-0.016	0.002	0.002	0.003	-0.015	0.003	0.005	0.005	-0.012
57.H	-0.018	0.001	0.002	0.007	-0.014	0.006	0.005	0.004	-0.014
58.H	-0.016	0.001	0.002	0.007	-0.012	0.006	0.003	0.003	-0.014
59.H	-0.016	0.004	0.005	0.006	-0.016	0.006	0.003	0.003	-0.016
60.H	-0.018	0.004	0.005	0.006	-0.018	0.006	0.005	0.005	-0.016
61.H	-0.018	0.004	0.005	0.008	-0.016	0.007	0.004	0.004	-0.018
62.H	-0.018	-0.001	-0.000	0.005	-0.012	0.005	0.005	0.005	-0.011
63.H	-0.017	-0.001	0.000	0.003	-0.013	0.003	0.006	0.006	-0.010
64.H	-0.016	-0.003	-0.001	0.003	-0.010	0.003	0.005	0.005	-0.009
65.H	-0.008	0.001	0.001	0.001	-0.009	0.001	0.000	0.000	-0.009
66.H	-0.005	0.003	0.002	0.002	-0.007	0.001	-0.003	-0.003	-0.011
67.H	-0.006	0.001	0.000	0.002	-0.005	0.001	-0.002	-0.002	-0.009
68.H	-0.008	0.002	0.002	0.004	-0.008	0.003	-0.002	-0.002	-0.012
69.C	-0.007	-0.003	-0.003	0.001	-0.002	0.000	-0.002	-0.001	-0.005
70.H	0.003	-0.000	-0.002	-0.006	-0.001	-0.006	-0.005	-0.004	-0.001
71.H	0.005	0.001	-0.001	-0.005	-0.000	-0.005	-0.007	-0.006	-0.002
72.H	0.003	0.002	0.000	-0.006	-0.003	-0.005	-0.005	-0.005	-0.004
73.H	0.001	0.001	-0.000	-0.002	-0.002	-0.003	-0.006	-0.005	-0.005
74.H	0.001	-0.003	-0.003	-0.004	0.001	-0.004	-0.005	-0.005	-0.001
75.H	0.002	-0.002	-0.003	-0.003	0.002	-0.003	-0.007	-0.006	-0.002
76.H	-0.003	-0.005	-0.005	-0.001	0.003	-0.002	-0.003	-0.003	-0.001
77.H	-0.000	-0.005	-0.005	-0.003	0.003	-0.004	-0.004	-0.004	0.000
78.H	-0.001	-0.006	-0.006	-0.001	0.006	-0.003	-0.005	-0.005	0.000
79.H	-0.004	-0.004	-0.004	0.003	0.003	0.001	-0.005	-0.004	-0.004
80.H	-0.002	-0.003	-0.003	0.002	0.004	0.000	-0.007	-0.006	-0.005

81.H	-0.002	-0.005	-0.005	0.002	0.006	-0.000	-0.006	-0.006	-0.002
82.H	-0.003	0.001	0.000	-0.000	-0.004	-0.001	-0.004	-0.003	-0.007
83.H	-0.004	0.002	0.002	-0.002	-0.008	-0.002	-0.002	-0.002	-0.008
84.H	-0.003	-0.003	-0.003	-0.002	-0.001	-0.002	-0.003	-0.003	-0.003
85.H	-0.001	-0.001	-0.002	0.000	0.001	-0.001	-0.006	-0.005	-0.005
86.H	-0.013	-0.000	0.000	0.004	-0.009	0.004	0.002	0.002	-0.011
87.O	-0.012	0.002	0.002	0.004	-0.011	0.004	0.001	0.001	-0.012
88.H	-0.010	0.005	0.005	0.003	-0.013	0.003	0.000	0.000	-0.014
89.H	-0.010	-0.004	-0.003	0.003	-0.003	0.002	-0.000	-0.000	-0.006
90.H	-0.009	-0.002	-0.002	0.004	-0.002	0.003	-0.002	-0.002	-0.008
91.H	-0.010	-0.002	-0.001	0.003	-0.005	0.002	-0.000	-0.000	-0.008
92.C	-0.009	-0.003	-0.002	0.003	-0.003	0.002	-0.001	-0.001	-0.007
93.O	-0.006	-0.004	-0.003	-0.000	-0.002	-0.001	-0.001	-0.001	-0.004
94.H	-0.010	0.004	0.004	-0.000	-0.016	0.001	0.003	0.003	-0.012
95.H	-0.009	0.006	0.006	0.000	-0.016	0.001	0.001	0.001	-0.014
96.H	-0.012	0.006	0.006	0.002	-0.017	0.003	0.003	0.003	-0.015

	16			17			18		
1.O	0.000	0.000	0.000	0.000	0.000	0.000	0.000	0.000	0.000
2.C	-0.009	0.000	0.000	-0.002	-0.010	0.001	-0.005	0.001	-0.007
3.C	-0.009	0.000	0.001	-0.001	-0.005	-0.000	-0.006	-0.000	-0.004
4.C	-0.010	0.000	0.001	-0.000	-0.006	-0.000	-0.009	0.000	-0.003
5.O	0.000	0.000	0.000	0.000	0.000	0.000	0.000	0.000	0.000
6.N	-0.010	0.000	0.002	0.001	-0.001	-0.002	-0.010	-0.001	0.000
7.O	0.000	0.000	0.000	0.000	0.000	0.000	0.000	0.000	0.000
8.C	-0.008	0.000	-0.002	0.002	-0.019	0.002	-0.001	0.003	-0.015
9.C	-0.006	-0.000	-0.000	-0.014	-0.005	0.004	0.010	-0.001	-0.010
10.C	-0.007	0.000	-0.001	0.007	-0.014	-0.001	0.001	0.001	-0.012
11.O	0.000	0.000	0.000	0.000	0.000	0.000	0.000	0.000	0.000

12.C	-0.007	0.000	-0.002	0.004	-0.019	0.001	0.001	0.003	-0.015
13.O	0.000	0.000	0.000	0.000	0.000	0.000	0.000	0.000	0.000
14.C	-0.008	0.000	-0.001	-0.003	-0.013	0.003	-0.001	0.001	-0.011
15.C	-0.007	0.000	-0.001	-0.007	-0.012	0.004	0.002	0.001	-0.011
16.O	0.246	0.000	-0.002	0.000	0.239	0.003	0.000	0.000	0.242
17.C	-0.009	0.000	-0.001	0.006	-0.015	-0.000	-0.005	0.002	-0.010
18.C	-0.005	-0.000	0.001	0.007	0.000	-0.005	0.008	-0.002	-0.005
19.O	-0.009	0.000	0.001	0.003	-0.004	-0.002	-0.006	-0.000	-0.003
20.C	-0.006	-0.000	0.000	0.001	-0.003	-0.002	0.007	-0.001	-0.007
21.C	-0.006	-0.000	0.000	0.007	-0.004	-0.003	0.006	-0.001	-0.007
22.C	-0.008	0.000	-0.001	0.002	-0.014	0.001	-0.002	0.002	-0.011
23.C	-0.006	-0.000	-0.000	-0.008	-0.005	0.002	0.008	-0.001	-0.009
24.C	-0.010	-0.000	0.002	-0.002	0.003	-0.002	-0.008	-0.002	0.002
25.C	-0.007	-0.000	-0.000	-0.007	-0.006	0.002	0.004	-0.000	-0.008
26.C	-0.010	0.000	0.001	-0.005	-0.008	0.002	-0.009	0.001	-0.005
27.C	-0.010	0.000	0.000	-0.006	-0.012	0.003	-0.008	0.002	-0.008
28.C	-0.010	0.000	-0.000	-0.011	-0.014	0.006	-0.008	0.002	-0.009
29.C	-0.006	-0.000	0.001	-0.001	0.000	-0.002	0.007	-0.002	-0.005
30.C	-0.007	0.000	-0.002	0.001	-0.021	0.003	0.003	0.003	-0.018
31.O	-0.006	-0.000	0.001	-0.009	0.004	0.001	0.010	-0.003	-0.004
32.C	-0.006	-0.000	-0.001	0.008	-0.009	-0.003	0.006	-0.000	-0.010
33.C	-0.006	-0.000	0.001	0.001	0.004	-0.003	0.005	-0.003	-0.002
34.C	-0.006	-0.000	0.000	-0.006	0.000	0.000	0.008	-0.002	-0.006
35.C	-0.007	0.000	-0.001	-0.012	-0.012	0.005	0.003	0.001	-0.012
36.C	-0.006	-0.000	-0.001	0.013	-0.010	-0.004	0.004	0.000	-0.010
37.C	-0.010	0.000	0.002	0.006	-0.001	-0.004	-0.012	-0.001	0.001
38.C	-0.007	0.000	-0.002	-0.009	-0.019	0.006	0.004	0.003	-0.017
39.C	-0.006	0.000	-0.001	0.014	-0.015	-0.004	0.004	0.001	-0.014
40.H	-0.006	-0.000	-0.000	-0.001	-0.006	-0.000	0.008	-0.001	-0.009
41.H	-0.007	-0.000	0.001	0.000	0.004	-0.003	0.004	-0.003	-0.001
42.H	-0.006	-0.000	0.001	0.005	0.004	-0.005	0.004	-0.003	-0.001

43.H	-0.006	-0.000	0.002	-0.001	0.008	-0.003	0.007	-0.004	-0.000
44.H	-0.006	-0.000	0.000	-0.016	-0.002	0.005	0.010	-0.002	-0.008
45.H	-0.005	-0.000	-0.000	-0.015	-0.003	0.004	0.012	-0.002	-0.010
46.H	-0.006	-0.000	-0.001	-0.015	-0.008	0.006	0.010	-0.000	-0.012
47.H	-0.006	-0.000	-0.001	-0.007	-0.008	0.002	0.008	-0.000	-0.011
48.H	-0.007	-0.000	0.000	-0.008	-0.004	0.002	0.004	-0.001	-0.007
49.H	-0.007	-0.000	0.000	-0.003	-0.006	0.001	0.003	-0.001	-0.007
50.H	-0.008	0.000	-0.000	-0.012	-0.009	0.005	0.001	0.001	-0.010
51.H	-0.008	0.000	-0.001	-0.013	-0.015	0.007	0.001	0.002	-0.014
52.H	-0.007	0.000	-0.001	-0.015	-0.010	0.006	0.005	0.001	-0.012
53.H	-0.007	0.000	-0.002	-0.013	-0.018	0.007	0.005	0.002	-0.017
54.H	-0.008	0.000	-0.002	-0.008	-0.022	0.006	0.001	0.003	-0.018
55.H	-0.007	0.000	-0.002	-0.009	-0.021	0.006	0.005	0.003	-0.019
56.H	-0.006	-0.000	-0.001	0.006	-0.009	-0.002	0.008	-0.000	-0.011
57.H	-0.006	-0.000	-0.000	0.014	-0.007	-0.006	0.005	-0.001	-0.009
58.H	-0.007	-0.000	-0.001	0.015	-0.010	-0.005	0.002	0.000	-0.009
59.H	-0.007	0.000	-0.002	0.013	-0.018	-0.002	0.003	0.002	-0.015
60.H	-0.006	0.000	-0.002	0.012	-0.015	-0.003	0.007	0.001	-0.015
61.H	-0.006	0.000	-0.001	0.018	-0.016	-0.005	0.003	0.002	-0.013
62.H	-0.006	-0.000	0.001	0.011	0.000	-0.006	0.008	-0.002	-0.005
63.H	-0.005	-0.000	0.000	0.005	0.000	-0.004	0.011	-0.003	-0.006
64.H	-0.005	-0.000	0.001	0.007	0.004	-0.005	0.008	-0.003	-0.003
65.H	-0.008	0.000	-0.000	0.002	-0.011	-0.000	-0.000	0.001	-0.010
66.H	-0.009	0.000	-0.001	0.005	-0.018	0.000	-0.007	0.003	-0.012
67.H	-0.009	0.000	-0.000	0.006	-0.012	-0.001	-0.006	0.001	-0.008
68.H	-0.009	0.000	-0.001	0.009	-0.016	-0.002	-0.006	0.002	-0.011
69.C	-0.008	-0.000	0.001	0.003	-0.001	-0.003	-0.003	-0.001	-0.002
70.H	-0.009	0.000	0.000	-0.014	-0.011	0.006	-0.006	0.001	-0.008
71.H	-0.010	0.000	-0.000	-0.011	-0.015	0.006	-0.010	0.002	-0.009
72.H	-0.010	0.000	-0.001	-0.012	-0.017	0.007	-0.007	0.003	-0.012
73.H	-0.010	0.000	-0.000	-0.004	-0.015	0.003	-0.010	0.002	-0.009

74.H	-0.010	0.000	0.001	-0.008	-0.005	0.002	-0.007	-0.000	-0.003
75.H	-0.010	0.000	0.001	-0.004	-0.008	0.002	-0.011	0.001	-0.004
76.H	-0.009	-0.000	0.002	-0.001	0.005	-0.003	-0.006	-0.002	0.003
77.H	-0.009	-0.000	0.002	-0.005	0.003	-0.000	-0.007	-0.002	0.001
78.H	-0.010	-0.000	0.003	-0.001	0.006	-0.003	-0.009	-0.002	0.005
79.H	-0.010	-0.000	0.002	0.008	0.000	-0.005	-0.010	-0.001	0.002
80.H	-0.011	0.000	0.002	0.008	-0.004	-0.004	-0.014	0.000	-0.000
81.H	-0.010	-0.000	0.003	0.007	0.002	-0.005	-0.013	-0.001	0.004
82.H	-0.009	0.000	-0.000	0.000	-0.013	0.001	-0.007	0.002	-0.009
83.H	-0.008	0.000	-0.001	-0.004	-0.017	0.004	-0.003	0.002	-0.013
84.H	-0.009	-0.000	0.001	-0.004	-0.002	0.000	-0.004	-0.001	-0.003
85.H	-0.010	0.000	0.001	0.003	-0.009	-0.001	-0.011	0.001	-0.004
86.H	-0.007	-0.000	0.000	0.010	-0.006	-0.004	0.001	-0.001	-0.007
87.O	-0.007	0.000	-0.001	0.010	-0.013	-0.002	-0.001	0.001	-0.010
88.H	-0.008	0.000	-0.002	0.007	-0.022	0.000	-0.001	0.003	-0.016
89.H	-0.008	-0.000	0.002	0.008	0.002	-0.006	-0.002	-0.002	0.000
90.H	-0.009	-0.000	0.001	0.011	-0.003	-0.005	-0.006	-0.001	-0.002
91.H	-0.008	-0.000	0.001	0.008	-0.004	-0.004	-0.002	-0.001	-0.004
92.C	-0.008	-0.000	0.001	0.008	-0.001	-0.005	-0.004	-0.001	-0.002
93.O	-0.008	-0.000	0.002	-0.000	0.002	-0.002	-0.001	-0.002	-0.001
94.H	-0.006	0.000	-0.002	-0.002	-0.019	0.003	0.005	0.002	-0.017
95.H	-0.007	0.000	-0.003	-0.000	-0.025	0.004	0.002	0.004	-0.020
96.H	-0.007	0.000	-0.002	0.003	-0.022	0.002	0.004	0.003	-0.019

	19			20			21		
1.O	0.000	0.000	0.000	0.000	0.000	0.000	0.000	0.000	0.000
2.C	-0.011	0.001	-0.000	0.001	-0.011	0.002	0.003	-0.001	-0.008
3.C	-0.012	-0.001	0.002	0.001	-0.013	0.001	0.003	-0.000	-0.010
4.C	-0.014	-0.000	0.003	0.001	-0.016	0.002	0.005	-0.001	-0.010

5.O	0.000	0.000	0.000	0.000	0.000	0.000	0.000	0.000	0.000
6.N	-0.015	-0.002	0.005	0.000	-0.017	0.001	0.005	-0.000	-0.012
7.O	0.000	0.000	0.000	0.000	0.000	0.000	0.000	0.000	0.000
8.C	-0.009	0.003	-0.005	-0.001	-0.005	-0.001	0.001	-0.002	-0.004
9.C	0.000	-0.001	-0.002	0.004	-0.004	0.004	-0.005	0.001	-0.006
10.C	-0.008	0.002	-0.003	-0.002	-0.003	-0.003	-0.000	-0.001	-0.006
11.O	0.000	0.000	0.000	0.000	0.000	0.000	0.000	0.000	0.000
12.C	-0.008	0.003	-0.005	-0.002	-0.003	-0.002	-0.000	-0.001	-0.004
13.O	0.000	0.000	0.000	0.000	0.000	0.000	0.000	0.000	0.000
14.C	-0.008	0.001	-0.003	0.001	-0.008	0.001	0.001	-0.001	-0.006
15.C	-0.006	0.001	-0.003	0.002	-0.006	0.002	-0.001	-0.000	-0.006
16.O	0.000	0.000	0.000	0.000	0.000	0.000	0.000	0.000	0.000
17.C	-0.012	0.002	-0.002	-0.002	-0.009	-0.002	0.003	-0.001	-0.007
18.C	-0.003	-0.002	0.002	-0.003	-0.003	-0.004	-0.004	0.002	-0.010
19.O	0.244	-0.001	0.003	0.000	0.244	-0.000	0.000	0.000	0.245
20.C	-0.003	-0.001	0.000	-0.001	-0.004	-0.001	-0.003	0.001	-0.008
21.C	-0.005	-0.001	0.001	-0.003	-0.004	-0.003	-0.003	0.001	-0.009
22.C	-0.009	0.002	-0.003	-0.000	-0.007	-0.001	0.001	-0.001	-0.006
23.C	-0.002	-0.001	-0.001	0.002	-0.004	0.002	-0.004	0.001	-0.007
24.C	-0.013	-0.003	0.007	0.001	-0.018	0.002	0.004	0.000	-0.013
25.C	-0.005	-0.000	-0.001	0.002	-0.006	0.002	-0.002	0.000	-0.007
26.C	-0.014	0.000	0.002	0.002	-0.016	0.003	0.005	-0.001	-0.009
27.C	-0.013	0.001	-0.000	0.003	-0.014	0.003	0.004	-0.002	-0.007
28.C	-0.012	0.002	-0.001	0.004	-0.014	0.005	0.004	-0.002	-0.006
29.C	-0.003	-0.002	0.002	-0.000	-0.005	-0.000	-0.003	0.002	-0.009
30.C	-0.006	0.003	-0.007	-0.001	-0.001	-0.002	-0.002	-0.001	-0.003
31.O	-0.000	-0.003	0.002	0.002	-0.006	0.002	-0.005	0.002	-0.009
32.C	-0.005	0.000	-0.002	-0.003	-0.002	-0.004	-0.003	0.001	-0.007
33.C	-0.004	-0.003	0.004	-0.001	-0.007	-0.001	-0.003	0.002	-0.011
34.C	-0.002	-0.002	0.001	0.002	-0.005	0.001	-0.004	0.002	-0.009
35.C	-0.005	0.001	-0.003	0.004	-0.007	0.004	-0.001	-0.001	-0.005

36.C	-0.006	0.001	-0.002	-0.005	-0.002	-0.006	-0.002	0.000	-0.007
37.C	-0.017	-0.002	0.006	-0.001	-0.018	-0.000	0.006	-0.000	-0.013
38.C	-0.004	0.003	-0.007	0.003	-0.003	0.002	-0.002	-0.001	-0.003
39.C	-0.007	0.002	-0.004	-0.005	0.000	-0.006	-0.002	-0.000	-0.006
40.H	-0.003	-0.000	-0.001	-0.000	-0.002	-0.001	-0.004	0.001	-0.007
41.H	-0.005	-0.003	0.004	-0.000	-0.009	-0.000	-0.002	0.002	-0.011
42.H	-0.006	-0.003	0.004	-0.002	-0.008	-0.002	-0.002	0.002	-0.012
43.H	-0.003	-0.004	0.005	-0.000	-0.007	-0.000	-0.004	0.003	-0.012
44.H	0.000	-0.001	-0.001	0.005	-0.005	0.004	-0.005	0.001	-0.007
45.H	0.002	-0.001	-0.002	0.004	-0.002	0.004	-0.006	0.002	-0.006
46.H	-0.000	0.000	-0.003	0.004	-0.003	0.004	-0.005	0.001	-0.005
47.H	-0.002	0.000	-0.002	0.002	-0.003	0.001	-0.004	0.001	-0.006
48.H	-0.005	-0.001	0.000	0.003	-0.008	0.003	-0.002	0.001	-0.008
49.H	-0.006	-0.000	0.000	0.001	-0.007	0.001	-0.001	0.000	-0.008
50.H	-0.006	0.000	-0.002	0.004	-0.008	0.004	-0.001	-0.000	-0.006
51.H	-0.006	0.002	-0.004	0.004	-0.007	0.004	-0.001	-0.001	-0.004
52.H	-0.003	0.001	-0.003	0.005	-0.006	0.005	-0.003	-0.000	-0.005
53.H	-0.003	0.003	-0.007	0.004	-0.003	0.004	-0.003	-0.001	-0.002
54.H	-0.006	0.004	-0.007	0.003	-0.004	0.002	-0.001	-0.002	-0.002
55.H	-0.004	0.003	-0.008	0.003	-0.001	0.002	-0.003	-0.001	-0.002
56.H	-0.003	0.000	-0.002	-0.002	-0.000	-0.004	-0.004	0.001	-0.007
57.H	-0.006	-0.000	-0.001	-0.005	-0.001	-0.006	-0.002	0.001	-0.008
58.H	-0.008	0.001	-0.001	-0.005	-0.003	-0.006	-0.000	0.000	-0.008
59.H	-0.007	0.003	-0.005	-0.004	-0.000	-0.006	-0.001	-0.001	-0.005
60.H	-0.005	0.002	-0.005	-0.004	0.002	-0.006	-0.003	0.000	-0.005
61.H	-0.008	0.002	-0.004	-0.006	0.000	-0.007	-0.001	-0.000	-0.006
62.H	-0.004	-0.002	0.002	-0.004	-0.003	-0.005	-0.003	0.002	-0.010
63.H	-0.001	-0.002	0.001	-0.002	-0.002	-0.003	-0.005	0.002	-0.009
64.H	-0.003	-0.003	0.003	-0.003	-0.004	-0.003	-0.004	0.003	-0.011
65.H	-0.008	0.001	-0.002	-0.001	-0.007	-0.001	0.000	-0.000	-0.007
66.H	-0.013	0.003	-0.003	-0.001	-0.009	-0.001	0.004	-0.002	-0.006

67.H	-0.012	0.001	-0.000	-0.002	-0.010	-0.001	0.003	-0.001	-0.008
68.H	-0.013	0.002	-0.002	-0.003	-0.008	-0.003	0.003	-0.002	-0.007
69.C	-0.011	-0.002	0.004	-0.001	-0.012	-0.000	0.002	0.001	-0.011
70.H	-0.010	0.001	-0.001	0.005	-0.014	0.006	0.003	-0.002	-0.007
71.H	-0.014	0.002	-0.001	0.004	-0.015	0.005	0.005	-0.002	-0.006
72.H	-0.011	0.002	-0.003	0.004	-0.012	0.005	0.003	-0.002	-0.005
73.H	-0.014	0.002	-0.001	0.002	-0.014	0.003	0.005	-0.002	-0.007
74.H	-0.012	-0.001	0.003	0.003	-0.016	0.004	0.004	-0.001	-0.009
75.H	-0.015	0.000	0.002	0.002	-0.017	0.003	0.006	-0.002	-0.009
76.H	-0.012	-0.003	0.007	0.001	-0.016	0.002	0.003	0.001	-0.013
77.H	-0.012	-0.003	0.006	0.002	-0.017	0.003	0.003	0.000	-0.012
78.H	-0.014	-0.003	0.008	0.001	-0.019	0.002	0.005	0.001	-0.014
79.H	-0.016	-0.002	0.006	-0.002	-0.017	-0.001	0.005	0.000	-0.013
80.H	-0.018	-0.001	0.005	-0.002	-0.018	-0.001	0.007	-0.001	-0.012
81.H	-0.017	-0.002	0.008	-0.002	-0.020	-0.000	0.007	-0.000	-0.014
82.H	-0.013	0.001	-0.001	0.000	-0.011	0.001	0.004	-0.002	-0.007
83.H	-0.009	0.002	-0.004	0.002	-0.008	0.002	0.001	-0.002	-0.005
84.H	-0.010	-0.001	0.003	0.001	-0.013	0.002	0.002	0.000	-0.010
85.H	-0.016	0.000	0.002	-0.000	-0.016	0.001	0.006	-0.001	-0.010
86.H	-0.008	-0.000	0.001	-0.003	-0.006	-0.004	-0.000	0.001	-0.009
87.O	-0.009	0.001	-0.002	-0.003	-0.005	-0.004	0.001	-0.001	-0.007
88.H	-0.010	0.004	-0.006	-0.002	-0.003	-0.003	0.001	-0.002	-0.004
89.H	-0.010	-0.002	0.005	-0.003	-0.012	-0.002	0.001	0.001	-0.012
90.H	-0.013	-0.001	0.004	-0.003	-0.012	-0.003	0.003	0.000	-0.011
91.H	-0.010	-0.001	0.003	-0.002	-0.010	-0.002	0.001	0.000	-0.010
92.C	-0.011	-0.002	0.004	-0.002	-0.011	-0.002	0.002	0.001	-0.011
93.O	-0.009	-0.002	0.005	0.000	-0.012	0.001	0.001	0.001	-0.011
94.H	-0.004	0.003	-0.007	0.000	-0.001	-0.001	-0.003	-0.001	-0.003
95.H	-0.006	0.004	-0.009	-0.000	-0.001	-0.001	-0.001	-0.002	-0.002
96.H	-0.005	0.004	-0.008	-0.001	0.001	-0.002	-0.002	-0.001	-0.003

	22			23			24		
	------------------------			------------------------			------------------------		
1.O	0.000	0.000	0.000	0.000	0.000	0.000	0.000	0.000	0.000
2.C	-0.004	-0.001	0.000	0.001	-0.001	-0.003	0.003	0.002	-0.010
3.C	-0.002	0.002	-0.004	0.000	-0.004	-0.001	0.003	0.003	-0.011
4.C	0.001	0.002	-0.005	-0.001	-0.001	-0.001	0.004	0.004	-0.011
5.O	0.000	0.000	0.000	0.000	0.000	0.000	0.000	0.000	0.000
6.N	0.003	0.005	-0.009	-0.002	-0.004	0.001	0.005	0.004	-0.013
7.O	0.000	0.000	0.000	0.000	0.000	0.000	0.000	0.000	0.000
8.C	-0.006	-0.007	0.008	-0.001	0.001	-0.001	0.000	-0.000	-0.005
9.C	-0.024	0.001	0.003	0.011	-0.016	-0.009	-0.004	-0.003	-0.010
10.C	-0.007	-0.005	0.005	-0.004	-0.006	0.004	-0.001	-0.001	-0.004
11.O	0.000	0.000	0.000	0.000	0.000	0.000	0.000	0.000	0.000
12.C	-0.007	-0.008	0.009	-0.003	-0.001	0.001	-0.001	-0.001	-0.003
13.O	0.000	0.000	0.000	0.000	0.000	0.000	0.000	0.000	0.000
14.C	-0.008	-0.004	0.004	0.002	-0.002	-0.004	0.001	0.001	-0.008
15.C	-0.014	-0.003	0.005	0.006	-0.006	-0.007	-0.001	-0.000	-0.009
16.O	0.000	0.000	0.000	0.000	0.000	0.000	0.000	0.000	0.000
17.C	-0.000	-0.005	0.003	-0.005	0.001	0.002	0.002	0.001	-0.006
18.C	-0.014	0.005	-0.004	-0.004	-0.023	0.009	-0.005	-0.004	-0.006
19.O	0.000	0.000	0.000	0.000	0.000	0.000	0.000	0.000	0.000
20.C	-0.015	0.002	-0.001	0.000	-0.018	0.003	-0.003	-0.003	-0.007
21.C	-0.011	0.002	-0.001	-0.004	-0.017	0.007	-0.003	-0.003	-0.006
22.C	-0.006	-0.004	0.004	-0.001	-0.002	-0.001	0.001	0.000	-0.006
23.C	-0.019	0.001	0.002	0.007	-0.015	-0.005	-0.003	-0.002	-0.009
24.C	-0.001	0.008	-0.011	0.000	-0.009	0.000	0.004	0.004	-0.014
25.C	-0.015	0.001	0.001	0.005	-0.011	-0.005	-0.001	-0.001	-0.009
26.C	-0.001	0.001	-0.003	0.002	0.001	-0.005	0.005	0.004	-0.012
27.C	-0.002	-0.002	0.001	0.004	0.003	-0.008	0.004	0.004	-0.011
28.C	-0.005	-0.003	0.003	0.007	0.005	-0.012	0.005	0.004	-0.012

29.C	-0.016	0.005	-0.003	0.002	-0.020	0.002	-0.003	-0.003	-0.009
30.C	-0.011	-0.009	0.012	-0.000	-0.001	-0.002	-0.002	-0.002	-0.003
31.O	0.239	0.007	-0.004	0.000	0.239	-0.003	0.000	0.000	0.244
32.C	-0.011	-0.001	0.002	-0.005	-0.014	0.007	-0.003	-0.003	-0.004
33.C	-0.013	0.007	-0.007	0.000	-0.022	0.005	-0.003	-0.002	-0.010
34.C	-0.019	0.004	-0.002	0.006	-0.020	-0.002	-0.004	-0.003	-0.010
35.C	-0.016	-0.003	0.006	0.009	-0.005	-0.011	-0.000	-0.000	-0.010
36.C	-0.007	-0.002	0.002	-0.008	-0.012	0.011	-0.003	-0.003	-0.002
37.C	0.007	0.005	-0.011	-0.006	-0.004	0.005	0.005	0.004	-0.012
38.C	-0.016	-0.008	0.012	0.007	-0.001	-0.010	-0.001	-0.001	-0.007
39.C	-0.007	-0.005	0.006	-0.009	-0.009	0.010	-0.003	-0.003	-0.001
40.H	-0.017	0.001	0.002	0.002	-0.016	0.001	-0.004	-0.003	-0.007
41.H	-0.012	0.007	-0.008	0.000	-0.020	0.004	-0.002	-0.001	-0.011
42.H	-0.011	0.007	-0.008	-0.003	-0.022	0.008	-0.003	-0.002	-0.009
43.H	-0.016	0.009	-0.009	0.001	-0.026	0.005	-0.004	-0.003	-0.011
44.H	-0.025	0.003	0.001	0.012	-0.017	-0.010	-0.003	-0.002	-0.012
45.H	-0.027	0.002	0.003	0.012	-0.019	-0.009	-0.005	-0.004	-0.010
46.H	-0.024	-0.001	0.006	0.012	-0.013	-0.011	-0.003	-0.003	-0.010
47.H	-0.019	-0.000	0.004	0.006	-0.014	-0.004	-0.003	-0.003	-0.008
48.H	-0.016	0.002	-0.001	0.007	-0.013	-0.005	-0.001	-0.001	-0.011
49.H	-0.012	0.001	-0.000	0.003	-0.011	-0.001	-0.001	-0.001	-0.009
50.H	-0.015	-0.001	0.003	0.009	-0.006	-0.010	0.000	0.000	-0.011
51.H	-0.015	-0.005	0.008	0.009	-0.001	-0.012	0.000	0.000	-0.009
52.H	-0.020	-0.002	0.006	0.011	-0.008	-0.012	-0.001	-0.001	-0.010
53.H	-0.019	-0.007	0.012	0.010	-0.002	-0.013	-0.001	-0.001	-0.008
54.H	-0.013	-0.009	0.012	0.006	0.002	-0.010	-0.000	-0.000	-0.006
55.H	-0.017	-0.009	0.013	0.007	-0.001	-0.010	-0.002	-0.002	-0.005
56.H	-0.014	-0.001	0.003	-0.003	-0.016	0.006	-0.005	-0.004	-0.004
57.H	-0.008	-0.000	0.000	-0.009	-0.016	0.013	-0.004	-0.004	-0.003
58.H	-0.004	-0.002	0.001	-0.010	-0.011	0.012	-0.002	-0.002	-0.003
59.H	-0.006	-0.007	0.008	-0.008	-0.005	0.008	-0.002	-0.003	-0.000

60.H	-0.010	-0.006	0.007	-0.007	-0.011	0.009	-0.004	-0.004	-0.000
61.H	-0.004	-0.006	0.006	-0.012	-0.008	0.013	-0.003	-0.003	0.000
62.H	-0.011	0.004	-0.004	-0.007	-0.023	0.012	-0.005	-0.004	-0.005
63.H	-0.017	0.004	-0.002	-0.002	-0.024	0.008	-0.006	-0.005	-0.006
64.H	-0.014	0.007	-0.006	-0.003	-0.025	0.010	-0.005	-0.004	-0.007
65.H	-0.007	-0.002	0.002	-0.001	-0.006	0.001	-0.000	-0.000	-0.007
66.H	0.001	-0.007	0.005	-0.004	0.004	0.001	0.003	0.002	-0.005
67.H	0.000	-0.002	0.000	-0.005	-0.001	0.003	0.002	0.002	-0.007
68.H	0.001	-0.005	0.003	-0.007	0.001	0.005	0.002	0.001	-0.005
69.C	-0.003	0.005	-0.007	-0.003	-0.011	0.004	0.001	0.001	-0.010
70.H	-0.008	-0.002	0.002	0.009	0.002	-0.013	0.004	0.004	-0.013
71.H	-0.002	-0.004	0.003	0.007	0.008	-0.012	0.006	0.005	-0.012
72.H	-0.006	-0.006	0.006	0.008	0.007	-0.013	0.004	0.004	-0.011
73.H	0.001	-0.004	0.002	0.001	0.007	-0.006	0.005	0.004	-0.010
74.H	-0.004	0.002	-0.004	0.005	-0.002	-0.007	0.004	0.004	-0.013
75.H	0.002	0.000	-0.004	0.002	0.003	-0.006	0.006	0.005	-0.012
76.H	-0.002	0.008	-0.012	-0.000	-0.012	0.002	0.003	0.003	-0.014
77.H	-0.004	0.007	-0.010	0.003	-0.009	-0.003	0.004	0.003	-0.015
78.H	0.001	0.009	-0.014	-0.000	-0.010	0.001	0.004	0.004	-0.015
79.H	0.006	0.006	-0.011	-0.006	-0.007	0.007	0.004	0.004	-0.011
80.H	0.010	0.003	-0.009	-0.007	-0.000	0.005	0.006	0.005	-0.011
81.H	0.008	0.007	-0.013	-0.006	-0.005	0.006	0.006	0.005	-0.013
82.H	-0.001	-0.003	0.001	-0.001	0.002	-0.002	0.003	0.003	-0.008
83.H	-0.007	-0.006	0.006	0.003	0.001	-0.006	0.002	0.001	-0.007
84.H	-0.005	0.004	-0.005	0.002	-0.008	-0.002	0.002	0.002	-0.012
85.H	0.004	-0.000	-0.004	-0.003	0.002	0.000	0.005	0.004	-0.010
86.H	-0.005	0.001	-0.002	-0.007	-0.012	0.009	-0.001	-0.001	-0.005
87.O	-0.004	-0.003	0.003	-0.007	-0.006	0.007	-0.001	-0.001	-0.004
88.H	-0.004	-0.009	0.010	-0.005	0.002	0.002	-0.000	-0.001	-0.002
89.H	-0.002	0.007	-0.010	-0.006	-0.015	0.009	0.000	0.000	-0.009
90.H	0.003	0.004	-0.008	-0.008	-0.009	0.009	0.002	0.002	-0.008

91.H	-0.002	0.003	-0.005	-0.005	-0.010	0.007	0.000	0.000	-0.008
92.C	-0.001	0.004	-0.007	-0.006	-0.011	0.008	0.001	0.001	-0.009
93.O	-0.007	0.006	-0.008	0.000	-0.014	0.002	0.001	0.001	-0.011
94.H	-0.014	-0.008	0.011	0.002	-0.004	-0.003	-0.002	-0.002	-0.004
95.H	-0.011	-0.011	0.014	0.000	0.002	-0.004	-0.001	-0.001	-0.002
96.H	-0.011	-0.010	0.013	-0.002	-0.002	-0.000	-0.002	-0.002	-0.002

	25			26			27		
	------------------------			------------------------			------------------------		
1.O	0.000	0.000	0.000	0.000	0.000	0.000	0.000	0.000	0.000
2.C	-0.006	0.001	-0.001	-0.001	-0.008	-0.000	-0.002	-0.005	-0.006
3.C	-0.007	-0.003	-0.000	-0.000	-0.006	-0.001	-0.002	-0.004	-0.005
4.C	-0.006	-0.003	-0.000	-0.000	-0.005	-0.001	-0.004	-0.007	-0.005
5.O	0.000	0.000	0.000	0.000	0.000	0.000	0.000	0.000	0.000
6.N	-0.007	-0.006	0.000	0.000	-0.003	-0.001	-0.004	-0.007	-0.004
7.O	0.000	0.000	0.000	0.000	0.000	0.000	0.000	0.000	0.000
8.C	-0.010	0.008	-0.000	0.001	-0.013	0.001	-0.000	-0.001	-0.012
9.C	0.000	-0.001	-0.002	-0.005	-0.009	-0.000	0.002	0.006	-0.003
10.C	-0.014	0.005	0.001	0.003	-0.012	0.001	0.002	0.002	-0.014
11.O	0.000	0.000	0.000	0.000	0.000	0.000	0.000	0.000	0.000
12.C	-0.012	0.008	0.000	0.002	-0.013	0.001	0.001	0.000	-0.014
13.O	0.000	0.000	0.000	0.000	0.000	0.000	0.000	0.000	0.000
14.C	-0.006	0.004	-0.001	-0.001	-0.010	0.000	-0.001	-0.002	-0.007
15.C	-0.003	0.003	-0.001	-0.003	-0.010	0.000	-0.000	0.000	-0.006
16.O	0.000	0.000	0.000	0.000	0.000	0.000	0.000	0.000	0.000
17.C	-0.012	0.004	0.000	0.002	-0.010	0.001	-0.001	-0.003	-0.012
18.C	-0.016	-0.004	0.002	0.003	-0.008	0.000	0.005	0.008	-0.013
19.O	0.000	0.000	0.000	0.000	0.000	0.000	0.000	0.000	0.000
20.C	-0.011	-0.002	0.001	0.001	-0.009	0.000	0.003	0.006	-0.010
21.C	-0.015	-0.002	0.002	0.003	-0.009	0.000	0.003	0.006	-0.013

22.C	-0.009	0.004	-0.000	0.001	-0.011	0.001	-0.000	-0.001	-0.010
23.C	-0.003	-0.001	-0.001	-0.003	-0.009	-0.000	0.002	0.005	-0.006
24.C	-0.006	-0.009	0.000	-0.001	-0.002	-0.002	-0.003	-0.005	-0.003
25.C	-0.004	-0.001	-0.001	-0.003	-0.009	-0.000	0.001	0.002	-0.006
26.C	-0.003	-0.002	-0.001	-0.002	-0.005	-0.001	-0.004	-0.008	-0.003
27.C	-0.002	0.002	-0.002	-0.003	-0.007	-0.000	-0.004	-0.008	-0.003
28.C	0.002	0.003	-0.003	-0.004	-0.008	-0.001	-0.005	-0.008	-0.001
29.C	-0.009	-0.005	0.000	-0.000	-0.007	-0.000	0.003	0.006	-0.008
30.C	-0.010	0.010	-0.000	0.001	-0.015	0.002	0.002	0.002	-0.013
31.O	0.000	0.000	0.000	0.000	0.000	0.000	0.000	0.000	0.000
32.C	-0.015	0.002	0.001	0.003	-0.011	0.001	0.004	0.006	-0.015
33.C	-0.010	-0.008	0.001	0.001	-0.005	-0.001	0.002	0.005	-0.008
34.C	-0.005	-0.005	-0.000	-0.002	-0.007	-0.000	0.002	0.006	-0.006
35.C	0.000	0.003	-0.002	-0.005	-0.010	-0.000	-0.001	-0.000	-0.003
36.C	-0.019	0.003	0.002	0.005	-0.012	0.001	0.004	0.005	-0.018
37.C	-0.011	-0.007	0.001	0.003	-0.002	-0.001	-0.004	-0.007	-0.007
38.C	-0.002	0.008	-0.002	-0.004	-0.014	0.001	-0.000	0.000	-0.007
39.C	-0.020	0.007	0.002	0.006	-0.014	0.002	0.004	0.005	-0.019
40.H	-0.009	-0.000	0.000	-0.000	-0.010	0.000	0.003	0.006	-0.010
41.H	-0.009	-0.008	0.001	0.000	-0.005	-0.001	0.002	0.004	-0.007
42.H	-0.013	-0.008	0.002	0.002	-0.005	-0.001	0.003	0.005	-0.010
43.H	-0.009	-0.010	0.001	-0.000	-0.004	-0.001	0.003	0.007	-0.007
44.H	0.002	-0.003	-0.002	-0.006	-0.008	-0.001	0.001	0.005	-0.002
45.H	0.000	-0.002	-0.002	-0.006	-0.009	-0.000	0.002	0.007	-0.003
46.H	0.001	0.001	-0.002	-0.006	-0.010	-0.000	0.001	0.005	-0.003
47.H	-0.005	0.001	-0.001	-0.003	-0.010	0.000	0.002	0.005	-0.007
48.H	-0.003	-0.003	-0.001	-0.003	-0.007	-0.001	0.000	0.002	-0.004
49.H	-0.007	-0.001	-0.000	-0.001	-0.008	-0.000	0.001	0.002	-0.007
50.H	0.001	0.001	-0.002	-0.005	-0.009	-0.000	-0.001	-0.001	-0.002
51.H	0.001	0.005	-0.003	-0.005	-0.011	0.000	-0.002	-0.002	-0.003
52.H	0.002	0.002	-0.003	-0.006	-0.010	-0.000	-0.001	0.001	-0.002

53.H	0.001	0.008	-0.003	-0.005	-0.013	0.001	-0.000	0.001	-0.005
54.H	-0.002	0.010	-0.002	-0.003	-0.014	0.001	-0.001	-0.001	-0.007
55.H	-0.003	0.010	-0.002	-0.003	-0.015	0.001	0.001	0.001	-0.008
56.H	-0.014	0.002	0.001	0.002	-0.012	0.001	0.004	0.007	-0.015
57.H	-0.020	0.001	0.003	0.006	-0.011	0.001	0.005	0.007	-0.018
58.H	-0.020	0.002	0.003	0.006	-0.011	0.001	0.003	0.004	-0.018
59.H	-0.019	0.008	0.002	0.005	-0.014	0.002	0.003	0.004	-0.019
60.H	-0.019	0.007	0.002	0.005	-0.014	0.002	0.005	0.007	-0.019
61.H	-0.023	0.007	0.003	0.007	-0.014	0.002	0.004	0.005	-0.021
62.H	-0.018	-0.004	0.003	0.005	-0.008	0.000	0.005	0.008	-0.015
63.H	-0.014	-0.004	0.002	0.002	-0.009	0.000	0.005	0.010	-0.013
64.H	-0.015	-0.007	0.002	0.003	-0.007	-0.000	0.004	0.008	-0.012
65.H	-0.010	0.002	0.000	0.001	-0.010	0.000	0.000	0.000	-0.010
66.H	-0.011	0.006	0.000	0.002	-0.011	0.001	-0.002	-0.005	-0.011
67.H	-0.012	0.002	0.001	0.002	-0.009	0.000	-0.001	-0.004	-0.011
68.H	-0.014	0.005	0.001	0.004	-0.011	0.001	-0.001	-0.003	-0.013
69.C	-0.010	-0.006	0.001	0.001	-0.005	-0.001	-0.001	-0.001	-0.008
70.H	0.003	0.001	-0.003	-0.006	-0.007	-0.001	-0.005	-0.007	0.001
71.H	0.002	0.003	-0.003	-0.004	-0.007	-0.001	-0.006	-0.010	-0.000
72.H	0.002	0.005	-0.003	-0.005	-0.009	-0.000	-0.005	-0.008	-0.001
73.H	-0.004	0.004	-0.001	-0.001	-0.008	-0.000	-0.004	-0.009	-0.005
74.H	-0.001	-0.004	-0.001	-0.003	-0.004	-0.001	-0.004	-0.007	-0.001
75.H	-0.003	-0.002	-0.001	-0.002	-0.005	-0.001	-0.005	-0.009	-0.002
76.H	-0.007	-0.010	0.000	-0.000	-0.002	-0.002	-0.002	-0.004	-0.003
77.H	-0.003	-0.009	-0.001	-0.002	-0.002	-0.002	-0.003	-0.005	-0.001
78.H	-0.006	-0.012	0.000	-0.000	-0.000	-0.002	-0.004	-0.006	-0.002
79.H	-0.012	-0.007	0.001	0.003	-0.003	-0.001	-0.003	-0.006	-0.008
80.H	-0.012	-0.005	0.001	0.003	-0.003	-0.001	-0.004	-0.009	-0.008
81.H	-0.011	-0.009	0.001	0.003	-0.001	-0.001	-0.004	-0.008	-0.006
82.H	-0.007	0.003	-0.001	0.000	-0.008	-0.000	-0.003	-0.006	-0.007
83.H	-0.005	0.006	-0.001	-0.002	-0.011	0.000	-0.002	-0.003	-0.007

84.H	-0.005	-0.005	-0.001	-0.001	-0.005	-0.001	-0.002	-0.003	-0.004
85.H	-0.008	-0.001	0.000	0.001	-0.006	-0.001	-0.004	-0.008	-0.006
86.H	-0.016	-0.001	0.002	0.004	-0.009	0.000	0.002	0.003	-0.014
87.O	0.242	0.004	0.001	0.000	0.245	0.001	0.000	0.000	0.243
88.H	-0.014	0.010	0.000	0.003	-0.014	0.002	0.001	-0.001	-0.015
89.H	-0.014	-0.008	0.002	0.003	-0.004	-0.001	0.001	0.001	-0.010
90.H	-0.016	-0.004	0.002	0.004	-0.005	-0.000	-0.000	-0.002	-0.011
91.H	-0.014	-0.003	0.001	0.003	-0.007	-0.000	0.000	-0.000	-0.011
92.C	-0.014	-0.005	0.002	0.003	-0.005	-0.000	-0.000	-0.001	-0.010
93.O	-0.008	-0.007	0.000	-0.000	-0.004	-0.001	-0.000	-0.000	-0.006
94.H	-0.008	0.009	-0.001	-0.001	-0.014	0.001	0.002	0.003	-0.012
95.H	-0.009	0.013	-0.001	0.000	-0.016	0.002	0.001	0.001	-0.013
96.H	-0.012	0.011	-0.000	0.001	-0.016	0.002	0.002	0.003	-0.015

	28			29			30		
1.O	0.000	0.000	0.000	0.000	0.000	0.000	0.000	0.000	0.000
2.C	-0.010	0.000	0.000	0.002	-0.009	-0.000	0.004	-0.000	-0.009
3.C	-0.010	0.000	0.001	0.001	-0.013	0.001	0.005	0.001	-0.012
4.C	-0.010	0.000	0.001	-0.000	-0.013	0.001	0.008	0.001	-0.013
5.O	0.000	0.000	0.000	0.000	0.000	0.000	0.000	0.000	0.000
6.N	-0.010	0.000	0.001	-0.001	-0.017	0.002	0.009	0.002	-0.016
7.O	0.000	0.000	0.000	0.000	0.000	0.000	0.000	0.000	0.000
8.C	-0.009	-0.000	-0.001	-0.002	-0.000	-0.002	0.001	-0.002	-0.003
9.C	-0.008	-0.000	-0.000	0.011	-0.009	-0.001	-0.009	0.001	-0.007
10.C	-0.008	-0.000	-0.001	-0.005	-0.002	-0.001	-0.001	-0.001	-0.004
11.O	0.000	0.000	0.000	0.000	0.000	0.000	0.000	0.000	0.000
12.C	-0.008	-0.000	-0.001	-0.004	0.001	-0.002	-0.000	-0.002	-0.002
13.O	0.000	0.000	0.000	0.000	0.000	0.000	0.000	0.000	0.000
14.C	-0.009	0.000	-0.000	0.002	-0.005	-0.001	0.001	-0.001	-0.006

15.C	-0.009	-0.000	-0.000	0.006	-0.006	-0.002	-0.002	-0.001	-0.006
16.O	0.000	0.000	0.000	0.000	0.000	0.000	0.000	0.000	0.000
17.C	-0.009	0.000	-0.000	-0.005	-0.004	-0.001	0.004	-0.001	-0.006
18.C	-0.007	-0.000	0.000	-0.006	-0.012	0.002	-0.007	0.002	-0.011
19.O	0.000	0.000	0.000	0.000	0.000	0.000	0.000	0.000	0.000
20.C	-0.008	-0.000	0.000	-0.001	-0.010	0.000	-0.006	0.001	-0.009
21.C	-0.008	-0.000	0.000	-0.005	-0.010	0.001	-0.005	0.001	-0.009
22.C	-0.009	0.000	-0.001	-0.001	-0.004	-0.001	0.001	-0.001	-0.006
23.C	-0.008	-0.000	-0.000	0.007	-0.009	-0.001	-0.007	0.001	-0.008
24.C	-0.010	0.000	0.001	0.001	-0.020	0.003	0.006	0.003	-0.017
25.C	-0.008	-0.000	-0.000	0.005	-0.009	-0.001	-0.004	0.000	-0.008
26.C	-0.010	0.000	0.001	0.004	-0.012	0.000	0.008	0.000	-0.012
27.C	-0.010	0.000	0.000	0.005	-0.008	-0.001	0.007	-0.001	-0.009
28.C	-0.010	0.000	-0.000	0.009	-0.007	-0.001	0.006	-0.001	-0.008
29.C	-0.008	-0.000	0.000	0.001	-0.013	0.001	-0.006	0.002	-0.011
30.C	-0.008	-0.000	-0.001	-0.001	0.003	-0.003	-0.003	-0.003	-0.000
31.O	0.000	0.000	0.000	0.000	0.000	0.000	0.000	0.000	0.000
32.C	-0.007	-0.000	-0.000	-0.006	-0.006	-0.000	-0.005	-0.000	-0.006
33.C	-0.008	-0.000	0.001	-0.001	-0.016	0.002	-0.005	0.002	-0.014
34.C	-0.008	-0.000	0.000	0.005	-0.013	0.000	-0.007	0.002	-0.010
35.C	-0.009	-0.000	-0.000	0.010	-0.006	-0.002	-0.002	-0.001	-0.006
36.C	-0.008	-0.000	-0.001	-0.010	-0.005	0.000	-0.003	-0.001	-0.006
37.C	-0.010	0.000	0.001	-0.005	-0.018	0.003	0.010	0.002	-0.016
38.C	-0.008	-0.000	-0.001	0.007	0.001	-0.003	-0.003	-0.002	-0.001
39.C	-0.008	-0.000	-0.001	-0.011	-0.000	-0.001	-0.003	-0.002	-0.003
40.H	-0.007	-0.000	-0.000	0.001	-0.008	-0.000	-0.007	0.000	-0.007
41.H	-0.008	-0.000	0.001	-0.000	-0.017	0.002	-0.003	0.002	-0.014
42.H	-0.008	-0.000	0.001	-0.004	-0.016	0.002	-0.004	0.002	-0.014
43.H	-0.008	-0.000	0.001	0.000	-0.019	0.002	-0.006	0.003	-0.015
44.H	-0.008	-0.000	0.000	0.012	-0.011	-0.001	-0.009	0.001	-0.009
45.H	-0.007	-0.000	-0.000	0.012	-0.009	-0.001	-0.011	0.001	-0.007

46.H	-0.008	-0.000	-0.000	0.012	-0.006	-0.002	-0.008	-0.000	-0.006
47.H	-0.008	-0.000	-0.000	0.005	-0.007	-0.001	-0.007	-0.000	-0.006
48.H	-0.008	-0.000	0.000	0.007	-0.011	-0.000	-0.003	0.001	-0.010
49.H	-0.008	-0.000	0.000	0.002	-0.010	-0.000	-0.002	0.000	-0.009
50.H	-0.009	0.000	-0.000	0.009	-0.008	-0.001	-0.001	-0.000	-0.008
51.H	-0.009	0.000	-0.001	0.010	-0.003	-0.003	-0.001	-0.001	-0.004
52.H	-0.008	-0.000	-0.000	0.012	-0.006	-0.002	-0.005	-0.001	-0.006
53.H	-0.008	-0.000	-0.001	0.011	0.000	-0.004	-0.005	-0.002	-0.001
54.H	-0.009	-0.000	-0.001	0.006	0.002	-0.004	-0.001	-0.003	-0.001
55.H	-0.008	-0.000	-0.001	0.007	0.003	-0.004	-0.004	-0.003	0.000
56.H	-0.007	-0.000	-0.001	-0.005	-0.005	-0.000	-0.007	-0.000	-0.006
57.H	-0.007	-0.000	-0.000	-0.011	-0.006	0.001	-0.004	-0.000	-0.007
58.H	-0.008	-0.000	-0.000	-0.012	-0.005	0.001	-0.001	-0.001	-0.007
59.H	-0.008	-0.000	-0.001	-0.010	0.001	-0.001	-0.002	-0.002	-0.002
60.H	-0.007	-0.000	-0.001	-0.009	0.000	-0.001	-0.005	-0.002	-0.002
61.H	-0.008	-0.000	-0.001	-0.014	0.000	-0.000	-0.002	-0.002	-0.003
62.H	-0.007	-0.000	0.000	-0.009	-0.012	0.002	-0.006	0.002	-0.011
63.H	-0.007	-0.000	0.000	-0.004	-0.011	0.001	-0.009	0.001	-0.010
64.H	-0.007	-0.000	0.001	-0.005	-0.015	0.002	-0.007	0.002	-0.013
65.H	-0.009	-0.000	-0.000	-0.002	-0.006	-0.000	0.000	-0.001	-0.007
66.H	-0.010	0.000	-0.001	-0.004	-0.002	-0.001	0.006	-0.002	-0.005
67.H	-0.009	0.000	-0.000	-0.005	-0.007	0.000	0.005	-0.001	-0.008
68.H	-0.009	0.000	-0.000	-0.007	-0.004	-0.000	0.005	-0.002	-0.006
69.C	-0.009	0.000	0.001	-0.003	-0.015	0.002	0.003	0.002	-0.014
70.H	-0.010	0.000	0.000	0.011	-0.009	-0.001	0.005	-0.000	-0.009
71.H	-0.011	0.000	-0.000	0.008	-0.007	-0.001	0.008	-0.001	-0.008
72.H	-0.010	0.000	-0.000	0.009	-0.005	-0.002	0.006	-0.002	-0.006
73.H	-0.010	0.000	-0.000	0.003	-0.006	-0.001	0.008	-0.001	-0.008
74.H	-0.010	0.000	0.001	0.006	-0.014	0.001	0.006	0.001	-0.013
75.H	-0.011	0.000	0.001	0.003	-0.012	0.000	0.010	0.000	-0.012
76.H	-0.010	0.000	0.001	0.000	-0.021	0.003	0.005	0.003	-0.018

77.H	-0.010	0.000	0.001	0.004	-0.020	0.002	0.005	0.002	-0.017
78.H	-0.010	0.000	0.002	0.000	-0.023	0.003	0.007	0.003	-0.019
79.H	-0.010	0.000	0.001	-0.006	-0.018	0.003	0.009	0.002	-0.017
80.H	-0.011	0.000	0.001	-0.007	-0.015	0.002	0.012	0.001	-0.015
81.H	-0.010	0.000	0.002	-0.006	-0.020	0.004	0.011	0.002	-0.018
82.H	-0.010	0.000	-0.000	-0.000	-0.007	-0.000	0.006	-0.001	-0.008
83.H	-0.009	0.000	-0.001	0.003	-0.003	-0.002	0.002	-0.002	-0.005
84.H	-0.009	0.000	0.001	0.003	-0.015	0.001	0.003	0.001	-0.013
85.H	-0.010	0.000	0.001	-0.002	-0.011	0.001	0.009	0.000	-0.012
86.H	-0.008	-0.000	0.000	-0.008	-0.009	0.001	-0.001	0.000	-0.009
87.O	0.000	0.000	0.000	0.000	0.000	0.000	0.000	0.000	0.000
88.H	-0.009	-0.000	-0.001	-0.006	0.003	-0.002	0.001	-0.003	-0.001
89.H	-0.009	0.000	0.001	-0.007	-0.017	0.003	0.002	0.002	-0.015
90.H	-0.009	0.000	0.001	-0.009	-0.014	0.002	0.005	0.001	-0.014
91.H	-0.009	0.000	0.000	-0.006	-0.012	0.002	0.002	0.001	-0.012
92.C	-0.009	0.000	0.001	-0.006	-0.015	0.002	0.003	0.001	-0.014
93.O	0.246	0.000	0.001	0.000	0.242	0.002	0.000	0.000	0.243
94.H	-0.008	-0.000	-0.001	0.001	0.001	-0.003	-0.005	-0.002	-0.001
95.H	-0.008	-0.000	-0.002	-0.000	0.005	-0.004	-0.002	-0.004	0.001
96.H	-0.008	-0.000	-0.002	-0.003	0.004	-0.003	-0.004	-0.003	0.001

	31			32			33		
1.O	0.000	0.000	0.000	0.000	0.000	0.000	0.000	0.000	0.000
2.C	0.283	0.000	0.001	0.000	0.283	0.002	0.000	0.000	0.284
3.C	-0.012	0.001	0.002	0.000	-0.012	0.001	0.001	0.002	-0.009
4.C	-0.014	0.001	0.003	0.000	-0.014	0.001	0.001	0.003	-0.009
5.O	0.000	0.000	0.000	0.000	0.000	0.000	0.000	0.000	0.000
6.N	-0.014	0.002	0.005	0.001	-0.014	0.001	0.001	0.003	-0.009
7.O	0.000	0.000	0.000	0.000	0.000	0.000	0.000	0.000	0.000

8.C	-0.008	-0.002	-0.004	0.000	-0.009	-0.000	-0.000	0.001	-0.007
9.C	-0.005	-0.000	-0.000	-0.001	-0.003	0.005	0.000	-0.002	-0.010
10.C	-0.005	-0.001	-0.003	0.000	-0.006	-0.003	-0.001	-0.001	-0.006
11.O	0.000	0.000	0.000	0.000	0.000	0.000	0.000	0.000	0.000
12.C	-0.006	-0.002	-0.005	0.000	-0.007	-0.002	-0.000	0.000	-0.006
13.O	0.000	0.000	0.000	0.000	0.000	0.000	0.000	0.000	0.000
14.C	-0.010	-0.001	-0.001	-0.000	-0.009	0.002	0.000	0.001	-0.008
15.C	-0.008	-0.001	-0.001	-0.000	-0.008	0.003	0.000	-0.000	-0.009
16.O	0.000	0.000	0.000	0.000	0.000	0.000	0.000	0.000	0.000
17.C	-0.010	-0.001	-0.002	0.001	-0.011	-0.002	0.000	0.002	-0.007
18.C	-0.000	0.000	0.001	-0.000	-0.001	-0.005	-0.001	-0.003	-0.006
19.O	0.000	0.000	0.000	0.000	0.000	0.000	0.000	0.000	0.000
20.C	-0.003	0.000	0.000	-0.000	-0.003	-0.002	-0.001	-0.002	-0.007
21.C	-0.002	0.000	-0.000	-0.000	-0.003	-0.004	-0.001	-0.002	-0.006
22.C	-0.008	-0.001	-0.002	0.000	-0.009	-0.000	0.000	0.001	-0.007
23.C	-0.005	-0.000	-0.000	-0.001	-0.004	0.003	-0.000	-0.002	-0.009
24.C	-0.013	0.002	0.006	0.000	-0.012	0.001	0.001	0.002	-0.009
25.C	-0.007	0.000	0.000	-0.001	-0.006	0.002	0.000	-0.001	-0.009
26.C	-0.015	0.001	0.003	0.000	-0.014	0.003	0.001	0.003	-0.010
27.C	-0.015	0.000	0.001	0.000	-0.014	0.004	0.001	0.003	-0.010
28.C	-0.016	-0.000	0.000	-0.000	-0.015	0.006	0.001	0.003	-0.011
29.C	-0.003	0.001	0.001	-0.000	-0.003	-0.001	-0.000	-0.002	-0.008
30.C	-0.005	-0.002	-0.006	-0.000	-0.006	-0.000	-0.000	-0.000	-0.007
31.O	0.000	0.000	0.000	0.000	0.000	0.000	0.000	0.000	0.000
32.C	-0.002	-0.001	-0.002	0.000	-0.003	-0.004	-0.001	-0.002	-0.005
33.C	-0.004	0.001	0.003	-0.000	-0.003	-0.002	-0.001	-0.002	-0.007
34.C	-0.004	0.001	0.001	-0.001	-0.003	0.001	-0.000	-0.003	-0.009
35.C	-0.010	-0.001	-0.001	-0.001	-0.008	0.005	0.001	0.000	-0.010
36.C	-0.001	-0.001	-0.003	0.000	-0.003	-0.006	-0.001	-0.002	-0.004
37.C	-0.014	0.002	0.005	0.001	-0.014	-0.002	0.001	0.003	-0.008
38.C	-0.008	-0.002	-0.004	-0.001	-0.007	0.004	0.000	0.000	-0.009

39.C	-0.001	-0.002	-0.004	0.000	-0.003	-0.007	-0.001	-0.002	-0.004
40.H	-0.003	-0.000	-0.001	-0.000	-0.002	-0.001	-0.001	-0.003	-0.007
41.H	-0.005	0.001	0.004	-0.000	-0.005	-0.001	-0.000	-0.002	-0.008
42.H	-0.003	0.001	0.003	-0.000	-0.003	-0.003	-0.001	-0.002	-0.007
43.H	-0.003	0.002	0.004	-0.000	-0.002	-0.002	-0.001	-0.003	-0.008
44.H	-0.006	0.000	0.001	-0.001	-0.003	0.005	0.000	-0.002	-0.011
45.H	-0.004	-0.000	-0.000	-0.001	-0.001	0.005	0.000	-0.003	-0.010
46.H	-0.006	-0.001	-0.001	-0.001	-0.004	0.005	0.000	-0.002	-0.010
47.H	-0.005	-0.000	-0.001	-0.001	-0.004	0.002	-0.000	-0.002	-0.008
48.H	-0.008	0.000	0.001	-0.001	-0.006	0.003	0.000	-0.001	-0.009
49.H	-0.007	0.000	0.000	-0.000	-0.006	0.001	-0.000	-0.001	-0.008
50.H	-0.010	-0.000	-0.000	-0.001	-0.009	0.005	0.001	0.001	-0.010
51.H	-0.011	-0.001	-0.002	-0.001	-0.009	0.006	0.001	0.001	-0.010
52.H	-0.009	-0.000	-0.001	-0.001	-0.007	0.006	0.001	-0.000	-0.011
53.H	-0.008	-0.002	-0.004	-0.001	-0.007	0.006	0.001	-0.000	-0.010
54.H	-0.009	-0.002	-0.005	-0.000	-0.009	0.004	0.000	0.001	-0.009
55.H	-0.007	-0.002	-0.005	-0.001	-0.007	0.004	0.000	-0.000	-0.009
56.H	-0.001	-0.001	-0.002	-0.000	-0.001	-0.004	-0.001	-0.003	-0.006
57.H	0.000	-0.001	-0.002	0.000	-0.002	-0.007	-0.001	-0.003	-0.004
58.H	-0.002	-0.001	-0.002	0.001	-0.005	-0.007	-0.001	-0.001	-0.004
59.H	-0.002	-0.002	-0.005	0.000	-0.005	-0.006	-0.001	-0.001	-0.004
60.H	0.000	-0.002	-0.005	0.000	-0.002	-0.006	-0.001	-0.002	-0.004
61.H	-0.000	-0.002	-0.005	0.001	-0.004	-0.008	-0.002	-0.002	-0.003
62.H	0.001	0.000	0.001	0.000	-0.001	-0.006	-0.001	-0.004	-0.005
63.H	0.001	0.000	0.000	-0.000	0.001	-0.004	-0.001	-0.004	-0.006
64.H	-0.000	0.001	0.002	-0.000	-0.001	-0.005	-0.001	-0.004	-0.006
65.H	-0.007	-0.000	-0.001	0.000	-0.008	-0.001	-0.000	0.000	-0.007
66.H	-0.011	-0.001	-0.002	0.001	-0.012	-0.001	0.000	0.002	-0.007
67.H	-0.010	-0.000	-0.000	0.001	-0.011	-0.002	0.000	0.002	-0.007
68.H	-0.009	-0.001	-0.002	0.001	-0.010	-0.003	-0.000	0.001	-0.006
69.C	-0.009	0.001	0.003	0.000	-0.009	-0.001	0.000	0.000	-0.008

70.H	-0.016	0.000	0.001	-0.000	-0.014	0.007	0.001	0.003	-0.011
71.H	-0.018	0.000	0.000	0.000	-0.016	0.007	0.002	0.004	-0.011
72.H	-0.016	-0.001	-0.001	-0.000	-0.015	0.007	0.001	0.003	-0.011
73.H	-0.015	-0.000	-0.000	0.000	-0.015	0.003	0.001	0.004	-0.009
74.H	-0.015	0.001	0.003	0.000	-0.014	0.004	0.001	0.003	-0.010
75.H	-0.017	0.001	0.003	0.000	-0.016	0.003	0.001	0.004	-0.010
76.H	-0.012	0.002	0.006	0.000	-0.011	0.000	0.001	0.001	-0.009
77.H	-0.014	0.002	0.006	0.000	-0.012	0.003	0.001	0.002	-0.010
78.H	-0.014	0.003	0.007	0.000	-0.013	0.001	0.001	0.002	-0.009
79.H	-0.012	0.002	0.005	0.001	-0.013	-0.002	0.000	0.002	-0.007
80.H	-0.015	0.002	0.004	0.001	-0.015	-0.002	0.001	0.003	-0.007
81.H	-0.014	0.002	0.006	0.001	-0.015	-0.002	0.001	0.003	-0.008
82.H	-0.012	-0.000	-0.000	0.000	-0.013	0.001	0.001	0.002	-0.008
83.H	-0.011	-0.001	-0.002	-0.000	-0.011	0.003	0.001	0.002	-0.009
84.H	-0.012	0.001	0.003	0.000	-0.011	0.002	0.001	0.001	-0.009
85.H	-0.014	0.001	0.002	0.001	-0.015	0.000	0.001	0.003	-0.008
86.H	-0.004	0.000	-0.000	0.000	-0.005	-0.005	-0.001	-0.001	-0.005
87.O	0.000	0.000	0.000	0.000	0.000	0.000	0.000	0.000	0.000
88.H	-0.006	-0.002	-0.005	0.000	-0.008	-0.003	-0.000	0.001	-0.006
89.H	-0.007	0.002	0.004	0.001	-0.007	-0.004	-0.000	-0.000	-0.006
90.H	-0.009	0.001	0.003	0.001	-0.010	-0.004	-0.000	0.001	-0.006
91.H	-0.007	0.001	0.002	0.000	-0.008	-0.003	-0.000	0.000	-0.006
92.C	-0.008	0.001	0.003	0.001	-0.009	-0.003	-0.000	0.000	-0.007
93.O	0.000	0.000	0.000	0.000	0.000	0.000	0.000	0.000	0.000
94.H	-0.005	-0.002	-0.005	-0.000	-0.005	0.000	-0.000	-0.001	-0.007
95.H	-0.006	-0.003	-0.007	-0.000	-0.007	0.000	-0.000	0.000	-0.007
96.H	-0.004	-0.002	-0.006	-0.000	-0.005	-0.001	-0.001	-0.001	-0.006

34 35 36

------------------------ ------------------------ ------------------------

1.O	0.000	0.000	0.000	0.000	0.000	0.000	0.000	0.000	0.000
2.C	0.000	0.000	0.000	0.000	0.000	0.000	0.000	0.000	0.000
3.C	0.282	0.001	0.003	0.000	0.282	0.002	0.000	0.000	0.284
4.C	-0.015	0.001	0.003	0.001	-0.016	0.002	0.004	0.002	-0.011
5.O	0.000	0.000	0.000	0.000	0.000	0.000	0.000	0.000	0.000
6.N	-0.016	0.001	0.006	0.000	-0.018	0.002	0.005	0.003	-0.012
7.O	0.000	0.000	0.000	0.000	0.000	0.000	0.000	0.000	0.000
8.C	-0.009	-0.000	-0.005	-0.000	-0.006	-0.001	0.001	-0.000	-0.005
9.C	-0.004	-0.000	-0.001	0.004	-0.004	0.004	-0.004	-0.001	-0.009
10.C	-0.006	-0.000	-0.003	-0.002	-0.004	-0.003	-0.001	-0.001	-0.005
11.O	0.000	0.000	0.000	0.000	0.000	0.000	0.000	0.000	0.000
12.C	-0.007	-0.001	-0.005	-0.001	-0.004	-0.002	-0.000	-0.001	-0.004
13.O	0.000	0.000	0.000	0.000	0.000	0.000	0.000	0.000	0.000
14.C	-0.010	-0.000	-0.002	0.001	-0.008	0.001	0.001	0.000	-0.007
15.C	-0.008	-0.000	-0.002	0.002	-0.007	0.003	-0.001	-0.000	-0.008
16.O	0.000	0.000	0.000	0.000	0.000	0.000	0.000	0.000	0.000
17.C	-0.011	-0.000	-0.002	-0.001	-0.009	-0.002	0.002	0.000	-0.006
18.C	-0.000	-0.000	0.001	-0.003	-0.002	-0.004	-0.004	-0.002	-0.007
19.O	0.000	0.000	0.000	0.000	0.000	0.000	0.000	0.000	0.000
20.C	-0.003	-0.000	0.000	-0.001	-0.004	-0.002	-0.003	-0.001	-0.008
21.C	-0.002	-0.000	0.000	-0.002	-0.003	-0.004	-0.003	-0.001	-0.007
22.C	-0.009	-0.000	-0.002	-0.000	-0.007	-0.001	0.001	-0.000	-0.006
23.C	-0.004	-0.000	-0.000	0.002	-0.004	0.002	-0.003	-0.001	-0.009
24.C	-0.014	0.001	0.007	0.001	-0.018	0.002	0.004	0.003	-0.013
25.C	-0.006	-0.000	-0.000	0.002	-0.007	0.002	-0.002	-0.000	-0.009
26.C	-0.016	0.001	0.003	0.002	-0.017	0.004	0.005	0.002	-0.011
27.C	-0.016	0.000	0.001	0.002	-0.015	0.004	0.004	0.002	-0.010
28.C	-0.016	0.000	-0.000	0.004	-0.015	0.006	0.004	0.002	-0.010
29.C	-0.003	-0.000	0.002	-0.000	-0.005	-0.000	-0.003	-0.001	-0.009
30.C	-0.005	-0.001	-0.007	-0.001	-0.001	-0.002	-0.002	-0.002	-0.003
31.O	0.000	0.000	0.000	0.000	0.000	0.000	0.000	0.000	0.000

32.C	-0.002	-0.001	-0.002	-0.003	-0.002	-0.004	-0.003	-0.002	-0.005
33.C	-0.004	0.000	0.004	-0.001	-0.007	-0.001	-0.003	-0.000	-0.010
34.C	-0.003	-0.000	0.002	0.001	-0.005	0.002	-0.004	-0.001	-0.010
35.C	-0.009	-0.000	-0.002	0.004	-0.007	0.005	-0.001	-0.000	-0.008
36.C	-0.002	-0.001	-0.003	-0.004	-0.002	-0.006	-0.002	-0.002	-0.004
37.C	-0.016	0.001	0.006	-0.001	-0.019	-0.000	0.005	0.003	-0.012
38.C	-0.007	-0.001	-0.006	0.003	-0.003	0.003	-0.001	-0.001	-0.005
39.C	-0.002	-0.001	-0.005	-0.004	-0.000	-0.007	-0.002	-0.002	-0.003
40.H	-0.002	-0.000	-0.001	-0.000	-0.002	-0.001	-0.004	-0.002	-0.007
41.H	-0.005	0.000	0.004	-0.000	-0.009	-0.000	-0.002	-0.000	-0.011
42.H	-0.004	0.000	0.004	-0.002	-0.007	-0.002	-0.002	-0.000	-0.010
43.H	-0.003	0.000	0.005	-0.000	-0.007	-0.000	-0.003	-0.001	-0.011
44.H	-0.004	-0.000	0.001	0.004	-0.005	0.005	-0.004	-0.001	-0.010
45.H	-0.002	-0.000	-0.001	0.004	-0.002	0.004	-0.005	-0.002	-0.009
46.H	-0.004	-0.000	-0.002	0.004	-0.003	0.005	-0.004	-0.001	-0.008
47.H	-0.004	-0.000	-0.002	0.002	-0.003	0.001	-0.003	-0.001	-0.007
48.H	-0.007	0.000	0.001	0.002	-0.008	0.003	-0.001	-0.000	-0.010
49.H	-0.007	-0.000	0.000	0.001	-0.007	0.001	-0.001	-0.000	-0.009
50.H	-0.010	0.000	-0.000	0.004	-0.009	0.005	-0.000	0.000	-0.009
51.H	-0.010	-0.000	-0.003	0.004	-0.007	0.005	0.000	-0.000	-0.008
52.H	-0.008	-0.000	-0.002	0.004	-0.006	0.005	-0.002	-0.000	-0.009
53.H	-0.007	-0.001	-0.005	0.004	-0.003	0.004	-0.002	-0.001	-0.006
54.H	-0.009	-0.001	-0.006	0.002	-0.005	0.003	-0.000	-0.001	-0.005
55.H	-0.006	-0.001	-0.007	0.002	-0.002	0.002	-0.002	-0.001	-0.004
56.H	-0.001	-0.001	-0.003	-0.002	-0.000	-0.004	-0.004	-0.002	-0.005
57.H	-0.001	-0.001	-0.002	-0.005	-0.001	-0.007	-0.003	-0.002	-0.005
58.H	-0.004	-0.000	-0.002	-0.005	-0.003	-0.007	-0.001	-0.001	-0.005
59.H	-0.003	-0.001	-0.006	-0.004	-0.001	-0.006	-0.002	-0.002	-0.002
60.H	-0.001	-0.001	-0.005	-0.004	0.002	-0.007	-0.003	-0.002	-0.002
61.H	-0.002	-0.001	-0.005	-0.005	-0.000	-0.008	-0.002	-0.002	-0.002
62.H	-0.000	-0.000	0.001	-0.004	-0.002	-0.006	-0.004	-0.002	-0.007

63.H	0.001	-0.000	0.001	-0.002	-0.001	-0.004	-0.005	-0.002	-0.007
64.H	-0.000	-0.000	0.003	-0.002	-0.004	-0.004	-0.004	-0.001	-0.009
65.H	-0.008	-0.000	-0.001	-0.001	-0.007	-0.001	0.000	-0.000	-0.007
66.H	-0.012	-0.000	-0.003	-0.001	-0.010	-0.001	0.003	0.001	-0.006
67.H	-0.011	0.000	-0.000	-0.002	-0.011	-0.002	0.003	0.001	-0.007
68.H	-0.010	-0.000	-0.002	-0.002	-0.009	-0.003	0.002	0.000	-0.006
69.C	-0.010	0.001	0.004	-0.001	-0.012	-0.001	0.002	0.001	-0.010
70.H	-0.015	0.000	0.001	0.004	-0.015	0.007	0.003	0.002	-0.011
71.H	-0.018	0.001	0.000	0.004	-0.016	0.006	0.005	0.002	-0.010
72.H	-0.016	0.000	-0.002	0.004	-0.013	0.006	0.004	0.002	-0.009
73.H	-0.016	0.000	-0.000	0.002	-0.015	0.003	0.005	0.002	-0.009
74.H	-0.015	0.001	0.004	0.003	-0.017	0.005	0.004	0.002	-0.012
75.H	-0.018	0.001	0.003	0.002	-0.018	0.004	0.006	0.003	-0.011
76.H	-0.013	0.001	0.007	0.001	-0.017	0.002	0.003	0.002	-0.013
77.H	-0.014	0.001	0.007	0.002	-0.018	0.004	0.003	0.002	-0.014
78.H	-0.015	0.001	0.009	0.001	-0.020	0.002	0.004	0.003	-0.014
79.H	-0.014	0.001	0.006	-0.002	-0.018	-0.001	0.005	0.002	-0.012
80.H	-0.017	0.001	0.005	-0.002	-0.019	-0.001	0.006	0.003	-0.011
81.H	-0.016	0.001	0.008	-0.001	-0.020	-0.000	0.006	0.003	-0.013
82.H	-0.013	0.000	-0.000	0.000	-0.012	0.001	0.003	0.001	-0.008
83.H	-0.011	-0.000	-0.003	0.001	-0.009	0.002	0.001	0.000	-0.007
84.H	-0.012	0.001	0.004	0.001	-0.014	0.002	0.002	0.001	-0.011
85.H	-0.016	0.001	0.003	-0.000	-0.017	0.001	0.005	0.002	-0.010
86.H	-0.005	-0.000	0.000	-0.003	-0.006	-0.004	-0.001	-0.001	-0.007
87.O	0.000	0.000	0.000	0.000	0.000	0.000	0.000	0.000	0.000
88.H	-0.007	-0.001	-0.006	-0.002	-0.004	-0.003	0.000	-0.001	-0.003
89.H	-0.008	0.001	0.005	-0.002	-0.012	-0.003	0.001	0.001	-0.010
90.H	-0.010	0.000	0.004	-0.003	-0.013	-0.003	0.003	0.001	-0.009
91.H	-0.008	0.000	0.002	-0.002	-0.010	-0.003	0.001	0.000	-0.009
92.C	-0.009	0.000	0.004	-0.002	-0.012	-0.002	0.001	0.001	-0.010
93.O	0.000	0.000	0.000	0.000	0.000	0.000	0.000	0.000	0.000

94.H	-0.004	-0.001	-0.006	0.000	-0.001	-0.001	-0.002	-0.002	-0.004
95.H	-0.006	-0.001	-0.008	-0.000	-0.001	-0.001	-0.001	-0.002	-0.003
96.H	-0.004	-0.001	-0.007	-0.001	0.000	-0.003	-0.002	-0.002	-0.002

	37			38			39		
1.O	0.000	0.000	0.000	0.000	0.000	0.000	0.000	0.000	0.000
2.C	0.000	0.000	0.000	0.000	0.000	0.000	0.000	0.000	0.000
3.C	0.000	0.000	0.000	0.000	0.000	0.000	0.000	0.000	0.000
4.C	0.279	0.001	0.006	0.000	0.278	0.003	0.000	0.000	0.283
5.O	0.000	0.000	0.000	0.000	0.000	0.000	0.000	0.000	0.000
6.N	-0.020	0.001	0.009	0.000	-0.023	0.002	0.007	0.003	-0.014
7.O	0.000	0.000	0.000	0.000	0.000	0.000	0.000	0.000	0.000
8.C	-0.009	0.000	-0.007	-0.001	-0.006	-0.001	0.001	-0.001	-0.004
9.C	-0.000	-0.001	-0.001	0.004	-0.001	0.007	-0.005	-0.001	-0.010
10.C	-0.005	-0.000	-0.005	-0.002	-0.003	-0.005	-0.001	-0.001	-0.004
11.O	0.000	0.000	0.000	0.000	0.000	0.000	0.000	0.000	0.000
12.C	-0.007	-0.000	-0.008	-0.002	-0.003	-0.004	-0.000	-0.001	-0.003
13.O	0.000	0.000	0.000	0.000	0.000	0.000	0.000	0.000	0.000
14.C	-0.011	0.000	-0.003	0.001	-0.009	0.002	0.001	0.000	-0.007
15.C	-0.008	-0.000	-0.003	0.002	-0.007	0.004	-0.001	-0.000	-0.008
16.O	0.000	0.000	0.000	0.000	0.000	0.000	0.000	0.000	0.000
17.C	-0.013	0.000	-0.003	-0.002	-0.011	-0.003	0.003	0.001	-0.006
18.C	0.003	-0.001	0.002	-0.003	0.001	-0.007	-0.005	-0.002	-0.008
19.O	0.000	0.000	0.000	0.000	0.000	0.000	0.000	0.000	0.000
20.C	-0.000	-0.001	0.001	-0.001	-0.001	-0.003	-0.004	-0.001	-0.008
21.C	-0.000	-0.001	0.001	-0.003	-0.001	-0.006	-0.004	-0.001	-0.007
22.C	-0.010	0.000	-0.004	-0.000	-0.008	-0.001	0.001	-0.000	-0.006
23.C	-0.002	-0.001	-0.000	0.002	-0.003	0.004	-0.004	-0.001	-0.009
24.C	-0.018	0.001	0.012	0.001	-0.023	0.004	0.005	0.003	-0.015

25.C	-0.006	-0.000	-0.000	0.002	-0.006	0.004	-0.002	-0.000	-0.009
26.C	-0.021	0.001	0.005	0.002	-0.022	0.006	0.006	0.003	-0.012
27.C	-0.020	0.001	0.001	0.003	-0.019	0.007	0.006	0.002	-0.010
28.C	-0.021	0.001	-0.000	0.004	-0.020	0.010	0.006	0.002	-0.011
29.C	-0.001	-0.001	0.003	-0.000	-0.003	-0.001	-0.004	-0.001	-0.010
30.C	-0.004	-0.000	-0.011	-0.001	0.000	-0.002	-0.002	-0.002	-0.002
31.O	0.000	0.000	0.000	0.000	0.000	0.000	0.000	0.000	0.000
32.C	0.000	-0.001	-0.003	-0.003	0.001	-0.007	-0.004	-0.002	-0.005
33.C	-0.002	-0.001	0.007	-0.001	-0.005	-0.002	-0.003	-0.000	-0.011
34.C	-0.000	-0.001	0.003	0.001	-0.003	0.002	-0.005	-0.001	-0.010
35.C	-0.009	0.000	-0.003	0.004	-0.008	0.008	-0.001	-0.000	-0.009
36.C	-0.000	-0.001	-0.004	-0.005	0.001	-0.010	-0.003	-0.002	-0.004
37.C	-0.021	0.001	0.010	-0.001	-0.024	-0.001	0.007	0.004	-0.013
38.C	-0.007	-0.000	-0.009	0.003	-0.003	0.005	-0.002	-0.001	-0.005
39.C	-0.000	-0.001	-0.007	-0.005	0.003	-0.011	-0.003	-0.002	-0.002
40.H	0.001	-0.001	-0.002	-0.000	0.000	-0.002	-0.005	-0.002	-0.007
41.H	-0.004	-0.000	0.007	-0.000	-0.008	-0.001	-0.002	0.000	-0.012
42.H	-0.002	-0.001	0.007	-0.002	-0.006	-0.004	-0.003	-0.000	-0.011
43.H	-0.000	-0.001	0.008	-0.000	-0.005	-0.001	-0.004	-0.000	-0.012
44.H	-0.001	-0.001	0.001	0.005	-0.003	0.008	-0.005	-0.001	-0.011
45.H	0.002	-0.001	-0.001	0.004	0.001	0.007	-0.007	-0.002	-0.010
46.H	-0.001	-0.001	-0.003	0.004	-0.001	0.008	-0.005	-0.002	-0.008
47.H	-0.001	-0.001	-0.003	0.002	-0.001	0.003	-0.004	-0.002	-0.008
48.H	-0.006	-0.000	0.002	0.003	-0.008	0.005	-0.002	0.000	-0.011
49.H	-0.006	-0.000	0.001	0.001	-0.007	0.001	-0.001	-0.000	-0.009
50.H	-0.010	0.000	-0.001	0.004	-0.010	0.008	0.000	0.000	-0.010
51.H	-0.010	0.000	-0.005	0.004	-0.008	0.008	0.000	-0.000	-0.008
52.H	-0.007	-0.000	-0.003	0.005	-0.006	0.009	-0.002	-0.001	-0.009
53.H	-0.006	-0.000	-0.009	0.004	-0.003	0.008	-0.002	-0.001	-0.005
54.H	-0.009	0.000	-0.010	0.003	-0.005	0.005	-0.000	-0.001	-0.004
55.H	-0.005	-0.000	-0.011	0.003	-0.001	0.004	-0.002	-0.002	-0.004

56.H	0.003	-0.001	-0.004	-0.003	0.003	-0.006	-0.005	-0.003	-0.005
57.H	0.001	-0.001	-0.002	-0.005	0.002	-0.011	-0.004	-0.002	-0.004
58.H	-0.003	-0.000	-0.003	-0.005	-0.002	-0.011	-0.001	-0.001	-0.004
59.H	-0.002	-0.000	-0.009	-0.005	0.002	-0.010	-0.002	-0.002	-0.001
60.H	0.002	-0.001	-0.008	-0.005	0.005	-0.010	-0.004	-0.003	-0.001
61.H	-0.000	-0.001	-0.008	-0.006	0.003	-0.013	-0.002	-0.003	-0.001
62.H	0.003	-0.001	0.002	-0.004	0.001	-0.009	-0.005	-0.002	-0.007
63.H	0.005	-0.001	0.001	-0.003	0.003	-0.006	-0.006	-0.002	-0.007
64.H	0.003	-0.001	0.005	-0.003	-0.000	-0.006	-0.005	-0.001	-0.009
65.H	-0.008	0.000	-0.002	-0.001	-0.007	-0.002	0.000	-0.000	-0.007
66.H	-0.015	0.001	-0.004	-0.001	-0.013	-0.002	0.004	0.001	-0.005
67.H	-0.014	0.000	-0.000	-0.002	-0.013	-0.003	0.004	0.001	-0.007
68.H	-0.013	0.000	-0.003	-0.003	-0.011	-0.005	0.003	0.000	-0.005
69.C	-0.012	0.000	0.007	-0.001	-0.014	-0.001	0.002	0.002	-0.012
70.H	-0.019	0.001	0.001	0.005	-0.019	0.011	0.005	0.002	-0.012
71.H	-0.023	0.001	0.000	0.005	-0.022	0.010	0.007	0.003	-0.011
72.H	-0.020	0.001	-0.003	0.005	-0.018	0.010	0.005	0.002	-0.009
73.H	-0.021	0.001	-0.001	0.002	-0.020	0.005	0.007	0.002	-0.009
74.H	-0.019	0.001	0.006	0.003	-0.021	0.008	0.005	0.003	-0.013
75.H	-0.023	0.001	0.005	0.002	-0.024	0.006	0.008	0.004	-0.013
76.H	-0.016	0.000	0.012	0.001	-0.020	0.002	0.004	0.003	-0.015
77.H	-0.018	0.001	0.011	0.002	-0.022	0.006	0.005	0.003	-0.016
78.H	-0.019	0.001	0.014	0.001	-0.025	0.003	0.006	0.004	-0.017
79.H	-0.019	0.001	0.010	-0.002	-0.022	-0.002	0.006	0.003	-0.013
80.H	-0.023	0.001	0.008	-0.002	-0.025	-0.002	0.008	0.004	-0.012
81.H	-0.022	0.001	0.013	-0.001	-0.026	-0.001	0.008	0.004	-0.015
82.H	-0.017	0.001	-0.001	0.001	-0.016	0.002	0.005	0.002	-0.008
83.H	-0.013	0.000	-0.005	0.002	-0.010	0.003	0.002	0.000	-0.007
84.H	-0.014	0.000	0.006	0.002	-0.017	0.004	0.003	0.002	-0.013
85.H	-0.021	0.001	0.004	0.000	-0.022	0.001	0.007	0.003	-0.011
86.H	-0.005	-0.000	0.001	-0.003	-0.005	-0.007	-0.001	-0.001	-0.007

87.O	0.000	0.000	0.000	0.000	0.000	0.000	0.000	0.000	0.000
88.H	-0.008	0.000	-0.009	-0.002	-0.004	-0.005	0.001	-0.001	-0.002
89.H	-0.009	-0.000	0.008	-0.003	-0.013	-0.005	0.001	0.001	-0.011
90.H	-0.013	0.000	0.006	-0.003	-0.015	-0.006	0.004	0.002	-0.010
91.H	-0.010	0.000	0.004	-0.002	-0.011	-0.004	0.001	0.001	-0.009
92.C	-0.011	0.000	0.006	-0.002	-0.013	-0.004	0.002	0.001	-0.011
93.O	0.000	0.000	0.000	0.000	0.000	0.000	0.000	0.000	0.000
94.H	-0.003	-0.000	-0.010	0.000	0.001	-0.001	-0.003	-0.002	-0.003
95.H	-0.006	-0.000	-0.013	-0.000	-0.000	-0.001	-0.001	-0.002	-0.001
96.H	-0.003	-0.000	-0.012	-0.001	0.002	-0.004	-0.002	-0.002	-0.001

	40			41			42		
	------------------------			------------------------			------------------------		
1.O	0.000	0.000	0.000	0.000	0.000	0.000	0.000	0.000	0.000
2.C	0.000	0.000	0.000	0.000	0.000	0.000	0.000	0.000	0.000
3.C	0.000	0.000	0.000	0.000	0.000	0.000	0.000	0.000	0.000
4.C	0.000	0.000	0.000	0.000	0.000	0.000	0.000	0.000	0.000
5.O	0.000	0.000	0.000	0.000	0.000	0.000	0.000	0.000	0.000
6.N	-0.010	-0.001	0.002	0.001	-0.003	-0.002	-0.008	-0.002	-0.002
7.O	0.000	0.000	0.000	0.000	0.000	0.000	0.000	0.000	0.000
8.C	0.284	0.001	-0.001	0.000	0.280	0.002	0.000	0.000	0.282
9.C	-0.006	-0.000	-0.000	-0.011	-0.006	0.003	0.007	0.000	-0.009
10.C	-0.009	0.001	-0.001	0.005	-0.014	-0.000	0.001	0.001	-0.012
11.O	0.000	0.000	0.000	0.000	0.000	0.000	0.000	0.000	0.000
12.C	-0.009	0.002	-0.001	0.003	-0.017	0.001	0.000	0.002	-0.014
13.O	0.000	0.000	0.000	0.000	0.000	0.000	0.000	0.000	0.000
14.C	-0.008	0.001	-0.001	-0.002	-0.012	0.002	-0.001	0.001	-0.010
15.C	-0.007	0.000	-0.001	-0.006	-0.011	0.003	0.001	0.001	-0.010
16.O	0.000	0.000	0.000	0.000	0.000	0.000	0.000	0.000	0.000
17.C	-0.010	0.001	-0.000	0.005	-0.014	-0.000	-0.004	0.001	-0.011

18.C	-0.008	-0.001	0.001	0.005	-0.002	-0.004	0.006	-0.001	-0.007
19.O	0.000	0.000	0.000	0.000	0.000	0.000	0.000	0.000	0.000
20.C	-0.008	-0.001	0.000	0.001	-0.005	-0.001	0.005	-0.000	-0.008
21.C	-0.008	-0.000	0.000	0.005	-0.005	-0.003	0.004	-0.000	-0.008
22.C	-0.009	0.001	-0.001	0.001	-0.013	0.001	-0.002	0.001	-0.011
23.C	-0.007	-0.000	-0.000	-0.007	-0.006	0.002	0.005	-0.000	-0.009
24.C	-0.010	-0.002	0.002	-0.001	0.000	-0.002	-0.007	-0.003	0.000
25.C	-0.007	-0.000	-0.000	-0.005	-0.007	0.001	0.003	-0.000	-0.008
26.C	-0.010	-0.000	0.001	-0.003	-0.008	0.001	-0.008	-0.001	-0.005
27.C	-0.009	0.000	-0.000	-0.004	-0.011	0.002	-0.007	-0.000	-0.008
28.C	-0.009	0.001	-0.000	-0.008	-0.013	0.004	-0.007	0.000	-0.008
29.C	-0.007	-0.001	0.001	-0.001	-0.002	-0.001	0.005	-0.001	-0.006
30.C	-0.008	0.002	-0.002	0.001	-0.019	0.002	0.002	0.003	-0.016
31.O	0.000	0.000	0.000	0.000	0.000	0.000	0.000	0.000	0.000
32.C	-0.008	0.000	-0.000	0.006	-0.009	-0.002	0.004	0.001	-0.011
33.C	-0.008	-0.002	0.001	0.001	0.001	-0.003	0.004	-0.002	-0.004
34.C	-0.007	-0.001	0.000	-0.005	-0.002	0.000	0.006	-0.001	-0.006
35.C	-0.007	0.000	-0.001	-0.009	-0.011	0.004	0.001	0.001	-0.010
36.C	-0.009	0.000	-0.000	0.010	-0.010	-0.003	0.003	0.001	-0.012
37.C	-0.011	-0.001	0.002	0.005	-0.003	-0.003	-0.010	-0.002	-0.001
38.C	-0.007	0.002	-0.002	-0.007	-0.017	0.005	0.003	0.002	-0.015
39.C	-0.009	0.001	-0.001	0.010	-0.014	-0.002	0.003	0.002	-0.014
40.H	-0.007	-0.000	-0.000	-0.001	-0.007	-0.000	0.006	0.000	-0.010
41.H	-0.008	-0.002	0.001	0.000	0.001	-0.003	0.002	-0.002	-0.003
42.H	-0.008	-0.002	0.001	0.004	0.001	-0.004	0.003	-0.002	-0.004
43.H	-0.007	-0.002	0.002	-0.001	0.003	-0.003	0.005	-0.002	-0.003
44.H	-0.005	-0.001	-0.000	-0.012	-0.004	0.003	0.007	-0.000	-0.007
45.H	-0.005	-0.000	-0.000	-0.011	-0.005	0.003	0.009	-0.000	-0.009
46.H	-0.006	0.000	-0.001	-0.012	-0.009	0.004	0.007	0.001	-0.011
47.H	-0.007	0.000	-0.001	-0.005	-0.008	0.002	0.006	0.000	-0.010
48.H	-0.007	-0.001	0.000	-0.006	-0.005	0.001	0.003	-0.001	-0.007

49.H	-0.008	-0.000	0.000	-0.002	-0.007	0.000	0.002	-0.000	-0.008
50.H	-0.007	0.000	-0.000	-0.009	-0.009	0.003	0.001	0.000	-0.009
51.H	-0.007	0.001	-0.001	-0.010	-0.014	0.005	0.001	0.001	-0.012
52.H	-0.006	0.000	-0.001	-0.011	-0.010	0.005	0.003	0.001	-0.010
53.H	-0.006	0.001	-0.002	-0.010	-0.016	0.006	0.004	0.002	-0.014
54.H	-0.007	0.002	-0.002	-0.006	-0.019	0.005	0.001	0.002	-0.015
55.H	-0.007	0.002	-0.002	-0.007	-0.018	0.005	0.003	0.002	-0.016
56.H	-0.008	0.000	-0.000	0.004	-0.009	-0.001	0.006	0.001	-0.012
57.H	-0.009	0.000	0.000	0.011	-0.008	-0.004	0.004	0.001	-0.011
58.H	-0.010	0.000	0.000	0.011	-0.010	-0.003	0.001	0.001	-0.011
59.H	-0.009	0.001	-0.001	0.010	-0.016	-0.001	0.002	0.002	-0.015
60.H	-0.009	0.001	-0.001	0.009	-0.014	-0.002	0.005	0.002	-0.015
61.H	-0.010	0.001	-0.001	0.013	-0.015	-0.003	0.002	0.002	-0.014
62.H	-0.008	-0.001	0.001	0.008	-0.002	-0.004	0.006	-0.001	-0.007
63.H	-0.007	-0.001	0.001	0.003	-0.003	-0.003	0.008	-0.000	-0.008
64.H	-0.008	-0.001	0.001	0.005	0.000	-0.004	0.006	-0.001	-0.005
65.H	-0.009	0.000	-0.000	0.002	-0.011	0.000	-0.001	0.001	-0.010
66.H	-0.010	0.001	-0.001	0.004	-0.016	0.000	-0.006	0.001	-0.012
67.H	-0.010	0.000	0.000	0.005	-0.011	-0.001	-0.005	0.000	-0.009
68.H	-0.010	0.001	-0.000	0.007	-0.014	-0.001	-0.005	0.001	-0.011
69.C	-0.009	-0.001	0.001	0.003	-0.003	-0.002	-0.003	-0.001	-0.004
70.H	-0.008	0.000	-0.000	-0.010	-0.010	0.004	-0.005	-0.000	-0.007
71.H	-0.009	0.001	-0.000	-0.008	-0.013	0.004	-0.008	-0.000	-0.008
72.H	-0.008	0.001	-0.001	-0.009	-0.015	0.005	-0.006	0.001	-0.010
73.H	-0.010	0.001	-0.000	-0.002	-0.014	0.002	-0.008	0.000	-0.009
74.H	-0.009	-0.001	0.001	-0.005	-0.006	0.001	-0.006	-0.001	-0.004
75.H	-0.010	-0.000	0.001	-0.003	-0.008	0.001	-0.009	-0.001	-0.005
76.H	-0.009	-0.002	0.002	-0.000	0.001	-0.003	-0.005	-0.003	0.000
77.H	-0.009	-0.002	0.002	-0.004	0.000	-0.001	-0.006	-0.003	-0.000
78.H	-0.010	-0.002	0.002	-0.000	0.003	-0.003	-0.007	-0.003	0.002
79.H	-0.011	-0.001	0.002	0.006	-0.002	-0.004	-0.008	-0.002	-0.001

80.H	-0.012	-0.001	0.002	0.007	-0.005	-0.003	-0.011	-0.002	-0.003
81.H	-0.011	-0.002	0.002	0.006	-0.001	-0.004	-0.011	-0.003	0.000
82.H	-0.010	0.001	-0.000	0.001	-0.012	0.001	-0.006	0.000	-0.009
83.H	-0.009	0.001	-0.001	-0.003	-0.015	0.003	-0.002	0.001	-0.012
84.H	-0.009	-0.001	0.001	-0.002	-0.004	-0.000	-0.004	-0.001	-0.004
85.H	-0.011	-0.000	0.001	0.002	-0.009	-0.001	-0.009	-0.001	-0.005
86.H	-0.009	-0.000	0.000	0.008	-0.007	-0.003	0.001	-0.000	-0.008
87.O	0.000	0.000	0.000	0.000	0.000	0.000	0.000	0.000	0.000
88.H	-0.010	0.002	-0.001	0.006	-0.019	0.001	-0.001	0.002	-0.015
89.H	-0.010	-0.001	0.002	0.007	-0.000	-0.004	-0.002	-0.002	-0.003
90.H	-0.011	-0.001	0.001	0.009	-0.004	-0.004	-0.005	-0.001	-0.004
91.H	-0.010	-0.001	0.001	0.006	-0.005	-0.003	-0.002	-0.001	-0.006
92.C	-0.010	-0.001	0.001	0.006	-0.003	-0.004	-0.003	-0.001	-0.004
93.O	0.000	0.000	0.000	0.000	0.000	0.000	0.000	0.000	0.000
94.H	-0.008	0.002	-0.002	-0.001	-0.017	0.003	0.004	0.002	-0.015
95.H	-0.008	0.002	-0.002	-0.000	-0.021	0.003	0.001	0.003	-0.018
96.H	-0.008	0.002	-0.002	0.002	-0.019	0.002	0.003	0.003	-0.017

	43			44			45		
	------------------------			------------------------			------------------------		
1.O	0.000	0.000	0.000	0.000	0.000	0.000	0.000	0.000	0.000
2.C	0.000	0.000	0.000	0.000	0.000	0.000	0.000	0.000	0.000
3.C	0.000	0.000	0.000	0.000	0.000	0.000	0.000	0.000	0.000
4.C	0.000	0.000	0.000	0.000	0.000	0.000	0.000	0.000	0.000
5.O	0.000	0.000	0.000	0.000	0.000	0.000	0.000	0.000	0.000
6.N	0.002	0.009	-0.010	-0.001	-0.002	-0.001	-0.002	0.007	-0.009
7.O	0.000	0.000	0.000	0.000	0.000	0.000	0.000	0.000	0.000
8.C	0.000	0.000	0.000	0.000	0.000	0.000	0.000	0.000	0.000
9.C	0.274	0.002	0.004	0.000	0.282	-0.006	0.000	0.000	0.281
10.C	-0.004	-0.008	0.004	-0.001	-0.010	0.003	-0.001	-0.001	-0.005

11.O	0.000	0.000	0.000	0.000	0.000	0.000	0.000	0.000	0.000
12.C	-0.005	-0.012	0.008	-0.000	-0.008	0.002	-0.001	0.001	-0.008
13.O	0.000	0.000	0.000	0.000	0.000	0.000	0.000	0.000	0.000
14.C	-0.010	-0.005	0.004	0.000	-0.006	-0.002	0.000	0.003	-0.012
15.C	-0.017	-0.004	0.005	0.001	-0.008	-0.004	0.002	0.001	-0.014
16.O	0.000	0.000	0.000	0.000	0.000	0.000	0.000	0.000	0.000
17.C	0.001	-0.007	0.002	-0.001	-0.004	0.002	-0.002	0.005	-0.007
18.C	-0.010	0.007	-0.005	-0.000	-0.017	0.006	-0.000	-0.010	-0.002
19.O	0.000	0.000	0.000	0.000	0.000	0.000	0.000	0.000	0.000
20.C	-0.014	0.004	-0.002	0.000	-0.015	0.002	0.001	-0.007	-0.006
21.C	-0.008	0.003	-0.003	-0.000	-0.015	0.005	-0.000	-0.007	-0.003
22.C	-0.005	-0.006	0.004	-0.000	-0.006	0.000	-0.001	0.003	-0.009
23.C	-0.022	0.002	0.002	0.002	-0.013	-0.003	0.003	-0.005	-0.012
24.C	-0.003	0.013	-0.012	-0.000	-0.004	-0.001	-0.001	0.005	-0.009
25.C	-0.018	0.001	0.001	0.001	-0.011	-0.003	0.002	-0.002	-0.012
26.C	-0.004	0.002	-0.003	-0.000	-0.001	-0.004	-0.000	0.009	-0.013
27.C	-0.007	-0.003	0.001	0.000	-0.000	-0.005	0.000	0.009	-0.015
28.C	-0.011	-0.004	0.004	0.001	0.000	-0.008	0.001	0.010	-0.018
29.C	-0.015	0.007	-0.004	0.001	-0.015	0.001	0.001	-0.007	-0.007
30.C	-0.010	-0.015	0.011	0.000	-0.009	0.000	0.000	0.000	-0.009
31.O	0.000	0.000	0.000	0.000	0.000	0.000	0.000	0.000	0.000
32.C	-0.007	-0.002	0.001	-0.001	-0.014	0.005	-0.001	-0.006	-0.003
33.C	-0.012	0.012	-0.008	0.000	-0.015	0.002	0.001	-0.007	-0.006
34.C	-0.021	0.007	-0.002	0.001	-0.015	-0.002	0.002	-0.007	-0.010
35.C	-0.021	-0.004	0.006	0.002	-0.008	-0.007	0.003	0.001	-0.016
36.C	-0.001	-0.004	0.001	-0.001	-0.014	0.008	-0.002	-0.005	-0.001
37.C	0.008	0.010	-0.012	-0.002	-0.001	0.002	-0.003	0.007	-0.006
38.C	-0.020	-0.012	0.012	0.001	-0.008	-0.006	0.002	0.001	-0.016
39.C	-0.000	-0.009	0.004	-0.001	-0.013	0.008	-0.002	-0.004	-0.001
40.H	-0.016	0.001	0.001	0.001	-0.015	0.001	0.001	-0.007	-0.008
41.H	-0.012	0.012	-0.008	0.000	-0.013	0.002	0.000	-0.006	-0.007

42.H	-0.008	0.012	-0.009	-0.000	-0.014	0.004	-0.000	-0.007	-0.004
43.H	-0.016	0.015	-0.010	0.001	-0.016	0.002	0.001	-0.009	-0.006
44.H	-0.031	0.005	0.002	0.003	-0.014	-0.007	0.004	-0.006	-0.016
45.H	-0.032	0.003	0.004	0.003	-0.016	-0.006	0.005	-0.008	-0.015
46.H	-0.030	-0.002	0.007	0.003	-0.013	-0.007	0.004	-0.005	-0.017
47.H	-0.021	-0.001	0.004	0.001	-0.013	-0.002	0.002	-0.005	-0.012
48.H	-0.019	0.004	-0.000	0.001	-0.010	-0.004	0.002	-0.002	-0.013
49.H	-0.013	0.002	-0.000	0.001	-0.010	-0.001	0.001	-0.002	-0.010
50.H	-0.020	-0.001	0.004	0.002	-0.007	-0.007	0.003	0.002	-0.016
51.H	-0.021	-0.008	0.009	0.002	-0.006	-0.007	0.003	0.003	-0.017
52.H	-0.026	-0.003	0.007	0.002	-0.009	-0.008	0.004	-0.000	-0.018
53.H	-0.025	-0.012	0.013	0.002	-0.009	-0.007	0.003	0.001	-0.018
54.H	-0.017	-0.014	0.013	0.001	-0.006	-0.005	0.002	0.004	-0.016
55.H	-0.020	-0.015	0.014	0.001	-0.009	-0.005	0.002	0.001	-0.015
56.H	-0.011	-0.003	0.002	-0.000	-0.016	0.004	0.000	-0.007	-0.004
57.H	-0.001	-0.001	-0.001	-0.001	-0.015	0.009	-0.002	-0.007	0.001
58.H	0.003	-0.003	-0.001	-0.002	-0.012	0.008	-0.003	-0.003	0.000
59.H	0.000	-0.012	0.007	-0.001	-0.011	0.007	-0.002	-0.002	-0.002
60.H	-0.004	-0.010	0.006	-0.001	-0.015	0.007	-0.001	-0.006	-0.002
61.H	0.004	-0.010	0.004	-0.002	-0.013	0.010	-0.003	-0.004	0.001
62.H	-0.006	0.007	-0.006	-0.001	-0.017	0.008	-0.001	-0.010	0.000
63.H	-0.014	0.006	-0.004	0.000	-0.019	0.005	0.001	-0.011	-0.003
64.H	-0.010	0.011	-0.008	-0.000	-0.018	0.006	-0.000	-0.010	-0.002
65.H	-0.006	-0.003	0.002	-0.000	-0.008	0.001	-0.000	0.001	-0.008
66.H	0.002	-0.010	0.004	-0.001	-0.003	0.001	-0.002	0.007	-0.008
67.H	0.002	-0.003	-0.001	-0.001	-0.004	0.002	-0.002	0.005	-0.007
68.H	0.004	-0.008	0.002	-0.001	-0.005	0.004	-0.002	0.005	-0.005
69.C	-0.003	0.008	-0.008	-0.001	-0.007	0.002	-0.001	0.001	-0.006
70.H	-0.015	-0.001	0.003	0.001	-0.001	-0.009	0.002	0.008	-0.019
71.H	-0.009	-0.005	0.004	0.001	0.002	-0.008	0.001	0.012	-0.018
72.H	-0.013	-0.008	0.007	0.001	0.000	-0.008	0.001	0.010	-0.019

73.H	-0.003	-0.006	0.003	-0.000	0.001	-0.004	-0.001	0.011	-0.014
74.H	-0.008	0.005	-0.004	0.000	-0.002	-0.005	0.000	0.007	-0.014
75.H	-0.002	0.002	-0.003	-0.000	0.001	-0.004	-0.001	0.011	-0.013
76.H	-0.004	0.015	-0.012	-0.000	-0.005	-0.000	-0.001	0.002	-0.008
77.H	-0.007	0.013	-0.010	0.000	-0.004	-0.003	0.000	0.004	-0.012
78.H	-0.001	0.017	-0.015	-0.001	-0.003	-0.001	-0.001	0.005	-0.009
79.H	0.007	0.010	-0.012	-0.002	-0.003	0.003	-0.003	0.006	-0.005
80.H	0.011	0.006	-0.010	-0.002	0.000	0.003	-0.003	0.009	-0.005
81.H	0.009	0.013	-0.014	-0.002	-0.001	0.003	-0.003	0.008	-0.005
82.H	-0.002	-0.004	0.001	-0.001	-0.002	-0.001	-0.001	0.007	-0.010
83.H	-0.010	-0.009	0.007	0.000	-0.004	-0.003	0.000	0.005	-0.013
84.H	-0.008	0.007	-0.005	0.000	-0.005	-0.002	0.000	0.003	-0.011
85.H	0.004	0.001	-0.004	-0.001	-0.000	-0.000	-0.002	0.009	-0.009
86.H	-0.001	0.001	-0.003	-0.001	-0.011	0.006	-0.002	-0.003	-0.002
87.O	0.000	0.000	0.000	0.000	0.000	0.000	0.000	0.000	0.000
88.H	-0.001	-0.015	0.009	-0.001	-0.007	0.003	-0.002	0.003	-0.006
89.H	0.001	0.011	-0.011	-0.001	-0.009	0.005	-0.002	-0.002	-0.003
90.H	0.006	0.006	-0.009	-0.002	-0.006	0.005	-0.003	0.002	-0.002
91.H	0.000	0.005	-0.006	-0.001	-0.008	0.004	-0.002	-0.000	-0.004
92.C	0.002	0.008	-0.009	-0.001	-0.008	0.004	-0.002	0.000	-0.004
93.O	0.000	0.000	0.000	0.000	0.000	0.000	0.000	0.000	0.000
94.H	-0.015	-0.012	0.011	0.001	-0.011	-0.001	0.001	-0.001	-0.011
95.H	-0.011	-0.018	0.014	0.000	-0.008	-0.001	0.000	0.002	-0.011
96.H	-0.009	-0.016	0.012	-0.000	-0.010	0.001	-0.000	-0.001	-0.008

	46			47			48		
1.O	0.000	0.000	0.000	0.000	0.000	0.000	0.000	0.000	0.000
2.C	0.000	0.000	0.000	0.000	0.000	0.000	0.000	0.000	0.000
3.C	0.000	0.000	0.000	0.000	0.000	0.000	0.000	0.000	0.000

4.C	0.000	0.000	0.000	0.000	0.000	0.000	0.000	0.000	0.000
5.O	0.000	0.000	0.000	0.000	0.000	0.000	0.000	0.000	0.000
6.N	-0.006	-0.005	-0.001	0.001	-0.001	-0.002	-0.006	-0.006	-0.003
7.O	0.000	0.000	0.000	0.000	0.000	0.000	0.000	0.000	0.000
8.C	0.000	0.000	0.000	0.000	0.000	0.000	0.000	0.000	0.000
9.C	0.000	0.000	0.000	0.000	0.000	0.000	0.000	0.000	0.000
10.C	0.282	0.004	0.001	0.000	0.282	0.002	0.000	0.000	0.281
11.O	0.000	0.000	0.000	0.000	0.000	0.000	0.000	0.000	0.000
12.C	-0.011	0.006	0.001	0.002	-0.016	0.002	0.001	0.001	-0.015
13.O	0.000	0.000	0.000	0.000	0.000	0.000	0.000	0.000	0.000
14.C	-0.007	0.002	-0.000	-0.002	-0.011	0.000	-0.001	-0.001	-0.009
15.C	-0.005	0.002	-0.001	-0.004	-0.011	0.000	0.001	0.000	-0.008
16.O	0.000	0.000	0.000	0.000	0.000	0.000	0.000	0.000	0.000
17.C	-0.010	0.003	0.001	0.003	-0.011	0.001	-0.003	-0.002	-0.012
18.C	-0.015	-0.003	0.001	0.004	-0.009	0.000	0.006	0.005	-0.012
19.O	0.000	0.000	0.000	0.000	0.000	0.000	0.000	0.000	0.000
20.C	-0.012	-0.001	0.000	0.001	-0.010	0.000	0.004	0.004	-0.010
21.C	-0.014	-0.001	0.001	0.004	-0.010	0.001	0.004	0.004	-0.012
22.C	-0.009	0.003	0.000	0.001	-0.012	0.001	-0.001	-0.001	-0.011
23.C	-0.006	-0.001	-0.001	-0.005	-0.010	-0.000	0.004	0.003	-0.007
24.C	-0.006	-0.007	-0.002	-0.001	0.001	-0.003	-0.005	-0.005	-0.001
25.C	-0.006	-0.001	-0.001	-0.004	-0.009	-0.000	0.002	0.001	-0.007
26.C	-0.003	-0.002	-0.001	-0.003	-0.004	-0.002	-0.006	-0.006	-0.003
27.C	-0.003	0.001	-0.001	-0.003	-0.007	-0.001	-0.006	-0.005	-0.005
28.C	-0.000	0.001	-0.002	-0.006	-0.007	-0.001	-0.006	-0.006	-0.004
29.C	-0.010	-0.003	-0.000	-0.001	-0.007	-0.001	0.004	0.003	-0.008
30.C	-0.011	0.007	0.001	0.001	-0.018	0.002	0.002	0.003	-0.016
31.O	0.000	0.000	0.000	0.000	0.000	0.000	0.000	0.000	0.000
32.C	-0.015	0.001	0.001	0.004	-0.013	0.001	0.004	0.004	-0.014
33.C	-0.011	-0.005	-0.000	0.000	-0.005	-0.001	0.003	0.003	-0.007
34.C	-0.008	-0.003	-0.001	-0.004	-0.008	-0.001	0.004	0.003	-0.006

35.C	-0.003	0.002	-0.001	-0.007	-0.011	-0.000	0.000	-0.000	-0.006
36.C	-0.017	0.002	0.002	0.007	-0.013	0.002	0.004	0.004	-0.016
37.C	-0.009	-0.005	-0.001	0.004	0.000	-0.002	-0.007	-0.006	-0.004
38.C	-0.005	0.006	-0.000	-0.005	-0.016	0.001	0.001	0.001	-0.011
39.C	-0.018	0.004	0.002	0.008	-0.016	0.003	0.004	0.004	-0.019
40.H	-0.011	0.000	0.000	-0.001	-0.011	0.001	0.005	0.004	-0.011
41.H	-0.010	-0.005	-0.001	0.000	-0.004	-0.001	0.002	0.001	-0.006
42.H	-0.013	-0.005	0.000	0.003	-0.005	-0.001	0.003	0.002	-0.008
43.H	-0.011	-0.007	-0.001	-0.001	-0.003	-0.002	0.004	0.003	-0.005
44.H	-0.003	-0.002	-0.002	-0.009	-0.008	-0.001	0.004	0.003	-0.004
45.H	-0.004	-0.001	-0.001	-0.009	-0.010	-0.001	0.006	0.005	-0.006
46.H	-0.003	0.001	-0.001	-0.009	-0.012	-0.000	0.004	0.003	-0.006
47.H	-0.007	0.001	-0.000	-0.004	-0.012	0.000	0.004	0.003	-0.009
48.H	-0.005	-0.002	-0.001	-0.005	-0.007	-0.001	0.001	0.001	-0.005
49.H	-0.008	-0.001	-0.000	-0.002	-0.009	-0.000	0.001	0.001	-0.008
50.H	-0.003	0.000	-0.001	-0.007	-0.009	-0.001	-0.000	-0.001	-0.005
51.H	-0.002	0.003	-0.001	-0.007	-0.012	-0.000	-0.001	-0.001	-0.007
52.H	-0.002	0.001	-0.001	-0.009	-0.011	-0.001	0.002	0.001	-0.006
53.H	-0.003	0.005	-0.001	-0.008	-0.016	0.001	0.002	0.002	-0.009
54.H	-0.005	0.007	-0.000	-0.005	-0.016	0.001	-0.000	0.000	-0.011
55.H	-0.006	0.007	0.000	-0.005	-0.018	0.002	0.002	0.002	-0.012
56.H	-0.015	0.002	0.001	0.003	-0.014	0.002	0.006	0.005	-0.014
57.H	-0.018	0.001	0.002	0.008	-0.013	0.002	0.005	0.005	-0.016
58.H	-0.018	0.002	0.002	0.008	-0.013	0.002	0.002	0.003	-0.016
59.H	-0.017	0.006	0.002	0.007	-0.017	0.003	0.003	0.004	-0.019
60.H	-0.017	0.005	0.002	0.007	-0.017	0.003	0.005	0.005	-0.019
61.H	-0.020	0.005	0.003	0.010	-0.017	0.003	0.003	0.004	-0.020
62.H	-0.017	-0.003	0.001	0.006	-0.009	0.001	0.006	0.005	-0.013
63.H	-0.015	-0.002	0.001	0.003	-0.010	0.000	0.007	0.006	-0.012
64.H	-0.015	-0.005	0.001	0.003	-0.007	-0.000	0.006	0.005	-0.010
65.H	-0.010	0.002	0.000	0.001	-0.011	0.001	-0.000	0.000	-0.011

66.H	-0.010	0.004	0.001	0.003	-0.012	0.001	-0.004	-0.003	-0.012
67.H	-0.010	0.001	0.000	0.003	-0.009	0.000	-0.003	-0.002	-0.011
68.H	-0.012	0.003	0.001	0.005	-0.012	0.001	-0.003	-0.002	-0.014
69.C	-0.010	-0.004	-0.000	0.002	-0.004	-0.001	-0.002	-0.002	-0.006
70.H	0.001	0.000	-0.002	-0.008	-0.006	-0.002	-0.005	-0.005	-0.002
71.H	0.000	0.002	-0.002	-0.006	-0.007	-0.001	-0.007	-0.007	-0.003
72.H	0.000	0.003	-0.001	-0.007	-0.010	-0.001	-0.006	-0.005	-0.005
73.H	-0.004	0.002	-0.001	-0.002	-0.008	-0.001	-0.007	-0.006	-0.007
74.H	-0.002	-0.003	-0.002	-0.004	-0.003	-0.002	-0.006	-0.005	-0.002
75.H	-0.003	-0.001	-0.002	-0.002	-0.003	-0.002	-0.008	-0.007	-0.003
76.H	-0.007	-0.007	-0.002	-0.000	0.001	-0.003	-0.004	-0.004	-0.001
77.H	-0.004	-0.006	-0.002	-0.003	0.001	-0.003	-0.005	-0.005	-0.000
78.H	-0.006	-0.008	-0.002	-0.000	0.003	-0.003	-0.006	-0.006	0.000
79.H	-0.010	-0.005	-0.001	0.004	-0.000	-0.002	-0.006	-0.005	-0.005
80.H	-0.009	-0.003	-0.000	0.005	-0.001	-0.001	-0.008	-0.007	-0.006
81.H	-0.009	-0.006	-0.001	0.004	0.002	-0.002	-0.008	-0.007	-0.003
82.H	-0.007	0.002	-0.000	0.000	-0.009	-0.000	-0.005	-0.004	-0.008
83.H	-0.006	0.004	-0.000	-0.002	-0.012	0.001	-0.002	-0.002	-0.010
84.H	-0.006	-0.003	-0.001	-0.002	-0.003	-0.002	-0.003	-0.003	-0.004
85.H	-0.007	-0.001	-0.001	0.002	-0.004	-0.001	-0.007	-0.006	-0.006
86.H	-0.015	-0.001	0.001	0.006	-0.009	0.001	0.001	0.002	-0.013
87.O	0.000	0.000	0.000	0.000	0.000	0.000	0.000	0.000	0.000
88.H	-0.013	0.007	0.002	0.004	-0.017	0.003	-0.000	0.001	-0.017
89.H	-0.013	-0.005	0.000	0.005	-0.003	-0.001	-0.001	-0.001	-0.007
90.H	-0.013	-0.003	0.000	0.006	-0.004	-0.001	-0.003	-0.002	-0.009
91.H	-0.012	-0.002	0.000	0.004	-0.006	-0.000	-0.001	-0.001	-0.009
92.C	-0.012	-0.004	0.000	0.004	-0.004	-0.001	-0.002	-0.001	-0.008
93.O	0.000	0.000	0.000	0.000	0.000	0.000	0.000	0.000	0.000
94.H	-0.009	0.006	0.001	-0.001	-0.017	0.002	0.003	0.003	-0.014
95.H	-0.010	0.008	0.001	-0.000	-0.020	0.003	0.001	0.002	-0.016
96.H	-0.012	0.007	0.002	0.002	-0.019	0.003	0.003	0.003	-0.017

	49			50			51		
1.O	0.000	0.000	0.000	0.000	0.000	0.000	0.000	0.000	0.000
2.C	0.000	0.000	0.000	0.000	0.000	0.000	0.000	0.000	0.000
3.C	0.000	0.000	0.000	0.000	0.000	0.000	0.000	0.000	0.000
4.C	0.000	0.000	0.000	0.000	0.000	0.000	0.000	0.000	0.000
5.O	0.000	0.000	0.000	0.000	0.000	0.000	0.000	0.000	0.000
6.N	-0.008	-0.003	-0.000	0.002	0.001	-0.003	-0.010	-0.005	-0.001
7.O	0.000	0.000	0.000	0.000	0.000	0.000	0.000	0.000	0.000
8.C	0.000	0.000	0.000	0.000	0.000	0.000	0.000	0.000	0.000
9.C	0.000	0.000	0.000	0.000	0.000	0.000	0.000	0.000	0.000
10.C	0.000	0.000	0.000	0.000	0.000	0.000	0.000	0.000	0.000
11.O	0.000	0.000	0.000	0.000	0.000	0.000	0.000	0.000	0.000
12.C	0.283	0.004	0.000	0.000	0.278	0.003	0.000	0.000	0.280
13.O	0.000	0.000	0.000	0.000	0.000	0.000	0.000	0.000	0.000
14.C	-0.008	0.002	-0.000	-0.003	-0.014	0.002	-0.002	0.000	-0.012
15.C	-0.007	0.001	-0.000	-0.007	-0.013	0.002	0.002	0.001	-0.011
16.O	0.000	0.000	0.000	0.000	0.000	0.000	0.000	0.000	0.000
17.C	-0.011	0.002	0.000	0.006	-0.015	0.001	-0.005	-0.000	-0.013
18.C	-0.013	-0.002	0.001	0.006	-0.005	-0.002	0.008	0.002	-0.010
19.O	0.000	0.000	0.000	0.000	0.000	0.000	0.000	0.000	0.000
20.C	-0.011	-0.001	0.000	0.001	-0.008	-0.001	0.007	0.002	-0.010
21.C	-0.013	-0.001	0.001	0.006	-0.008	-0.001	0.006	0.002	-0.011
22.C	-0.010	0.002	0.000	0.002	-0.015	0.001	-0.002	0.001	-0.013
23.C	-0.007	-0.000	-0.000	-0.008	-0.009	0.001	0.007	0.002	-0.009
24.C	-0.007	-0.004	-0.001	-0.001	0.004	-0.004	-0.008	-0.005	0.002
25.C	-0.007	-0.000	-0.000	-0.007	-0.009	0.001	0.004	0.001	-0.008
26.C	-0.006	-0.001	-0.001	-0.004	-0.005	-0.001	-0.009	-0.004	-0.004
27.C	-0.006	0.001	-0.001	-0.006	-0.010	0.001	-0.009	-0.003	-0.007

28.C	-0.004	0.001	-0.001	-0.010	-0.011	0.002	-0.008	-0.003	-0.007
29.C	-0.010	-0.002	0.000	-0.001	-0.004	-0.001	0.007	0.001	-0.007
30.C	-0.010	0.005	0.000	0.001	-0.024	0.004	0.003	0.004	-0.020
31.O	0.000	0.000	0.000	0.000	0.000	0.000	0.000	0.000	0.000
32.C	-0.013	0.001	0.001	0.007	-0.013	0.000	0.006	0.003	-0.015
33.C	-0.010	-0.004	0.000	0.001	-0.000	-0.003	0.005	-0.000	-0.004
34.C	-0.008	-0.002	-0.000	-0.006	-0.005	-0.001	0.008	0.001	-0.006
35.C	-0.005	0.001	-0.001	-0.012	-0.013	0.002	0.002	0.001	-0.010
36.C	-0.015	0.001	0.001	0.012	-0.015	-0.000	0.004	0.003	-0.016
37.C	-0.010	-0.003	-0.000	0.007	0.001	-0.004	-0.012	-0.005	-0.001
38.C	-0.006	0.004	-0.001	-0.009	-0.021	0.004	0.003	0.003	-0.016
39.C	-0.015	0.003	0.001	0.013	-0.020	0.001	0.004	0.004	-0.020
40.H	-0.010	0.000	0.000	-0.001	-0.011	0.001	0.008	0.003	-0.012
41.H	-0.010	-0.004	-0.000	0.000	0.001	-0.003	0.004	-0.001	-0.003
42.H	-0.012	-0.004	0.000	0.004	-0.000	-0.003	0.004	-0.000	-0.005
43.H	-0.010	-0.005	0.000	-0.001	0.003	-0.003	0.007	-0.000	-0.002
44.H	-0.005	-0.001	-0.001	-0.015	-0.006	0.001	0.009	0.001	-0.006
45.H	-0.006	-0.001	-0.001	-0.015	-0.009	0.002	0.012	0.003	-0.008
46.H	-0.005	0.001	-0.001	-0.015	-0.012	0.003	0.009	0.002	-0.010
47.H	-0.008	0.001	-0.000	-0.007	-0.012	0.002	0.007	0.002	-0.011
48.H	-0.007	-0.001	-0.001	-0.008	-0.006	0.000	0.003	-0.000	-0.006
49.H	-0.009	-0.001	-0.000	-0.003	-0.008	0.000	0.002	0.000	-0.008
50.H	-0.005	0.000	-0.001	-0.012	-0.010	0.002	0.001	-0.000	-0.008
51.H	-0.005	0.002	-0.001	-0.012	-0.016	0.003	0.001	0.001	-0.012
52.H	-0.004	0.001	-0.001	-0.015	-0.013	0.003	0.004	0.001	-0.009
53.H	-0.005	0.004	-0.001	-0.013	-0.021	0.005	0.005	0.003	-0.015
54.H	-0.006	0.005	-0.000	-0.008	-0.023	0.005	0.001	0.003	-0.017
55.H	-0.007	0.005	-0.000	-0.008	-0.023	0.005	0.005	0.004	-0.018
56.H	-0.013	0.001	0.001	0.005	-0.014	0.001	0.008	0.004	-0.015
57.H	-0.015	0.001	0.001	0.013	-0.012	-0.001	0.006	0.003	-0.015
58.H	-0.015	0.001	0.001	0.014	-0.014	-0.001	0.002	0.002	-0.015

59.H	-0.014	0.004	0.001	0.012	-0.022	0.002	0.003	0.004	-0.021
60.H	-0.015	0.003	0.001	0.011	-0.020	0.002	0.007	0.005	-0.020
61.H	-0.016	0.003	0.002	0.017	-0.020	0.001	0.003	0.004	-0.021
62.H	-0.014	-0.002	0.001	0.010	-0.006	-0.002	0.008	0.002	-0.010
63.H	-0.013	-0.002	0.001	0.004	-0.006	-0.001	0.011	0.003	-0.010
64.H	-0.013	-0.003	0.001	0.006	-0.002	-0.003	0.008	0.001	-0.007
65.H	-0.010	0.001	0.000	0.002	-0.013	0.001	-0.000	0.001	-0.012
66.H	-0.010	0.003	0.000	0.006	-0.017	0.001	-0.007	-0.000	-0.014
67.H	-0.011	0.001	0.000	0.006	-0.011	-0.000	-0.006	-0.001	-0.011
68.H	-0.012	0.002	0.001	0.009	-0.016	0.001	-0.005	0.000	-0.014
69.C	-0.010	-0.003	0.000	0.003	-0.002	-0.003	-0.004	-0.002	-0.004
70.H	-0.003	0.000	-0.001	-0.013	-0.009	0.001	-0.006	-0.003	-0.005
71.H	-0.004	0.001	-0.001	-0.010	-0.011	0.002	-0.010	-0.003	-0.006
72.H	-0.004	0.002	-0.001	-0.011	-0.014	0.003	-0.008	-0.002	-0.009
73.H	-0.006	0.002	-0.001	-0.003	-0.012	0.001	-0.010	-0.003	-0.009
74.H	-0.005	-0.002	-0.001	-0.007	-0.003	-0.001	-0.008	-0.004	-0.002
75.H	-0.006	-0.001	-0.001	-0.003	-0.005	-0.001	-0.012	-0.005	-0.003
76.H	-0.008	-0.005	-0.000	-0.000	0.005	-0.004	-0.006	-0.005	0.002
77.H	-0.006	-0.004	-0.001	-0.005	0.004	-0.003	-0.007	-0.005	0.002
78.H	-0.008	-0.005	-0.001	-0.000	0.008	-0.005	-0.009	-0.006	0.004
79.H	-0.010	-0.003	0.000	0.008	0.002	-0.004	-0.010	-0.005	-0.001
80.H	-0.010	-0.002	0.000	0.009	-0.001	-0.003	-0.014	-0.005	-0.003
81.H	-0.010	-0.004	-0.000	0.008	0.005	-0.005	-0.013	-0.006	0.001
82.H	-0.008	0.001	-0.000	0.001	-0.012	0.001	-0.007	-0.002	-0.010
83.H	-0.007	0.003	-0.000	-0.004	-0.017	0.002	-0.003	0.000	-0.013
84.H	-0.007	-0.002	-0.001	-0.003	-0.002	-0.002	-0.004	-0.003	-0.003
85.H	-0.009	-0.001	-0.000	0.003	-0.006	-0.001	-0.011	-0.004	-0.005
86.H	-0.013	-0.000	0.001	0.009	-0.009	-0.001	0.001	0.001	-0.011
87.O	0.000	0.000	0.000	0.000	0.000	0.000	0.000	0.000	0.000
88.H	-0.012	0.005	0.001	0.007	-0.023	0.003	-0.001	0.003	-0.020
89.H	-0.012	-0.004	0.000	0.008	0.001	-0.004	-0.002	-0.002	-0.004

90.H	-0.012	-0.002	0.001	0.011	-0.003	-0.003	-0.006	-0.002	-0.006
91.H	-0.012	-0.001	0.000	0.007	-0.005	-0.002	-0.002	-0.001	-0.007
92.C	-0.012	-0.002	0.000	0.008	-0.002	-0.003	-0.004	-0.002	-0.005
93.O	0.000	0.000	0.000	0.000	0.000	0.000	0.000	0.000	0.000
94.H	-0.009	0.004	0.000	-0.002	-0.022	0.004	0.005	0.004	-0.018
95.H	-0.010	0.006	0.000	-0.000	-0.027	0.005	0.002	0.004	-0.021
96.H	-0.011	0.005	0.001	0.003	-0.025	0.004	0.004	0.005	-0.021

	52			53			54		
1.O	0.000	0.000	0.000	0.000	0.000	0.000	0.000	0.000	0.000
2.C	0.000	0.000	0.000	0.000	0.000	0.000	0.000	0.000	0.000
3.C	0.000	0.000	0.000	0.000	0.000	0.000	0.000	0.000	0.000
4.C	0.000	0.000	0.000	0.000	0.000	0.000	0.000	0.000	0.000
5.O	0.000	0.000	0.000	0.000	0.000	0.000	0.000	0.000	0.000
6.N	-0.012	0.002	0.001	0.001	-0.009	-0.001	-0.005	0.002	-0.007
7.O	0.000	0.000	0.000	0.000	0.000	0.000	0.000	0.000	0.000
8.C	0.000	0.000	0.000	0.000	0.000	0.000	0.000	0.000	0.000
9.C	0.000	0.000	0.000	0.000	0.000	0.000	0.000	0.000	0.000
10.C	0.000	0.000	0.000	0.000	0.000	0.000	0.000	0.000	0.000
11.O	0.000	0.000	0.000	0.000	0.000	0.000	0.000	0.000	0.000
12.C	0.000	0.000	0.000	0.000	0.000	0.000	0.000	0.000	0.000
13.O	0.000	0.000	0.000	0.000	0.000	0.000	0.000	0.000	0.000
14.C	0.283	-0.001	-0.000	0.000	0.282	0.002	0.000	0.000	0.283
15.C	-0.011	-0.001	-0.000	-0.003	-0.011	0.003	0.002	0.001	-0.013
16.O	0.000	0.000	0.000	0.000	0.000	0.000	0.000	0.000	0.000
17.C	-0.009	-0.002	-0.001	0.003	-0.014	-0.000	-0.003	0.003	-0.010
18.C	-0.005	0.001	-0.000	0.003	-0.004	-0.003	0.003	-0.004	-0.006
19.O	0.000	0.000	0.000	0.000	0.000	0.000	0.000	0.000	0.000
20.C	-0.007	0.001	-0.000	0.000	-0.006	-0.001	0.003	-0.003	-0.009

21.C	-0.006	0.000	-0.001	0.003	-0.007	-0.003	0.002	-0.003	-0.007
22.C	-0.010	-0.002	-0.001	0.001	-0.013	0.001	-0.001	0.002	-0.011
23.C	-0.010	0.000	0.000	-0.004	-0.007	0.002	0.004	-0.002	-0.012
24.C	-0.012	0.003	0.002	-0.000	-0.006	-0.001	-0.003	0.001	-0.006
25.C	-0.011	0.000	0.000	-0.003	-0.009	0.002	0.002	-0.001	-0.011
26.C	-0.014	0.001	0.001	-0.002	-0.012	0.002	-0.003	0.003	-0.010
27.C	-0.014	-0.000	0.001	-0.002	-0.014	0.003	-0.003	0.004	-0.012
28.C	-0.015	-0.001	0.001	-0.005	-0.015	0.004	-0.002	0.005	-0.014
29.C	-0.008	0.001	0.000	-0.001	-0.005	-0.001	0.003	-0.003	-0.008
30.C	-0.008	-0.004	-0.002	0.000	-0.015	0.002	0.001	0.002	-0.014
31.O	0.000	0.000	0.000	0.000	0.000	0.000	0.000	0.000	0.000
32.C	-0.006	-0.001	-0.001	0.003	-0.009	-0.002	0.002	-0.002	-0.009
33.C	-0.008	0.003	0.001	0.000	-0.003	-0.002	0.002	-0.004	-0.006
34.C	-0.009	0.001	0.001	-0.003	-0.005	0.001	0.004	-0.003	-0.010
35.C	-0.013	-0.001	0.000	-0.006	-0.012	0.004	0.002	0.001	-0.014
36.C	-0.004	-0.001	-0.002	0.006	-0.010	-0.003	0.000	-0.001	-0.008
37.C	-0.011	0.003	0.001	0.003	-0.009	-0.002	-0.006	0.002	-0.005
38.C	-0.012	-0.003	-0.001	-0.004	-0.015	0.004	0.002	0.002	-0.016
39.C	-0.004	-0.003	-0.002	0.006	-0.012	-0.003	0.000	-0.001	-0.009
40.H	-0.008	-0.000	-0.000	-0.001	-0.008	-0.000	0.003	-0.003	-0.010
41.H	-0.009	0.003	0.001	-0.000	-0.004	-0.002	0.001	-0.003	-0.006
42.H	-0.007	0.003	0.001	0.002	-0.003	-0.003	0.001	-0.004	-0.005
43.H	-0.008	0.003	0.001	-0.001	-0.001	-0.002	0.003	-0.005	-0.006
44.H	-0.012	0.001	0.001	-0.007	-0.006	0.004	0.005	-0.003	-0.013
45.H	-0.011	0.000	0.000	-0.007	-0.006	0.003	0.007	-0.004	-0.013
46.H	-0.012	-0.001	0.000	-0.007	-0.009	0.004	0.005	-0.002	-0.014
47.H	-0.010	-0.001	-0.000	-0.003	-0.009	0.002	0.004	-0.002	-0.012
48.H	-0.011	0.001	0.001	-0.004	-0.008	0.002	0.002	-0.001	-0.011
49.H	-0.010	0.000	0.000	-0.001	-0.009	0.001	0.001	-0.001	-0.010
50.H	-0.013	-0.000	0.001	-0.005	-0.011	0.004	0.002	0.001	-0.013
51.H	-0.013	-0.002	-0.000	-0.006	-0.014	0.005	0.002	0.002	-0.015

52.H	-0.013	-0.001	0.000	-0.007	-0.011	0.005	0.004	0.000	-0.015
53.H	-0.012	-0.003	-0.001	-0.006	-0.014	0.005	0.003	0.002	-0.017
54.H	-0.012	-0.004	-0.001	-0.004	-0.016	0.004	0.001	0.003	-0.016
55.H	-0.011	-0.004	-0.001	-0.004	-0.015	0.004	0.003	0.002	-0.016
56.H	-0.005	-0.001	-0.001	0.002	-0.009	-0.002	0.003	-0.003	-0.009
57.H	-0.004	-0.001	-0.001	0.006	-0.008	-0.004	0.001	-0.003	-0.007
58.H	-0.004	-0.001	-0.001	0.007	-0.010	-0.004	-0.001	-0.001	-0.007
59.H	-0.005	-0.003	-0.002	0.006	-0.014	-0.002	-0.000	0.000	-0.010
60.H	-0.004	-0.003	-0.002	0.005	-0.012	-0.002	0.001	-0.001	-0.010
61.H	-0.003	-0.003	-0.002	0.008	-0.012	-0.004	-0.001	-0.000	-0.008
62.H	-0.004	0.001	-0.001	0.004	-0.004	-0.004	0.002	-0.004	-0.005
63.H	-0.005	0.001	-0.000	0.002	-0.004	-0.003	0.004	-0.005	-0.007
64.H	-0.005	0.002	0.000	0.002	-0.003	-0.004	0.003	-0.005	-0.005
65.H	-0.009	-0.001	-0.001	0.001	-0.011	-0.000	-0.000	0.001	-0.010
66.H	-0.010	-0.002	-0.001	0.003	-0.016	0.000	-0.004	0.004	-0.011
67.H	-0.009	-0.001	-0.000	0.003	-0.013	-0.001	-0.003	0.002	-0.009
68.H	-0.008	-0.002	-0.001	0.004	-0.014	-0.001	-0.003	0.003	-0.009
69.C	-0.010	0.002	0.001	0.002	-0.007	-0.002	-0.002	-0.000	-0.007
70.H	-0.016	-0.000	0.001	-0.006	-0.013	0.005	-0.001	0.004	-0.014
71.H	-0.016	-0.001	0.001	-0.004	-0.015	0.004	-0.003	0.005	-0.014
72.H	-0.015	-0.002	0.000	-0.005	-0.016	0.005	-0.002	0.005	-0.015
73.H	-0.014	-0.001	0.000	-0.001	-0.015	0.002	-0.004	0.005	-0.012
74.H	-0.014	0.001	0.001	-0.003	-0.010	0.002	-0.002	0.002	-0.010
75.H	-0.014	0.001	0.001	-0.001	-0.012	0.002	-0.004	0.004	-0.010
76.H	-0.012	0.004	0.002	-0.000	-0.005	-0.001	-0.003	-0.000	-0.006
77.H	-0.013	0.003	0.002	-0.002	-0.006	0.000	-0.002	0.001	-0.007
78.H	-0.012	0.004	0.002	0.000	-0.005	-0.001	-0.004	0.000	-0.005
79.H	-0.010	0.003	0.001	0.004	-0.008	-0.003	-0.005	0.001	-0.005
80.H	-0.011	0.002	0.001	0.004	-0.011	-0.002	-0.007	0.003	-0.006
81.H	-0.011	0.003	0.002	0.004	-0.008	-0.003	-0.006	0.002	-0.004
82.H	-0.012	-0.001	-0.000	0.000	-0.014	0.001	-0.003	0.003	-0.011

83.H	-0.012	-0.002	-0.000	-0.002	-0.015	0.003	-0.001	0.003	-0.013
84.H	-0.012	0.002	0.001	-0.001	-0.008	0.000	-0.001	0.001	-0.009
85.H	-0.012	0.000	0.001	0.002	-0.012	-0.000	-0.005	0.004	-0.008
86.H	-0.006	0.000	-0.001	0.004	-0.008	-0.003	-0.001	-0.001	-0.007
87.O	0.000	0.000	0.000	0.000	0.000	0.000	0.000	0.000	0.000
88.H	-0.008	-0.004	-0.002	0.003	-0.016	-0.000	-0.001	0.003	-0.012
89.H	-0.008	0.003	0.001	0.004	-0.005	-0.004	-0.002	-0.002	-0.004
90.H	-0.008	0.002	0.000	0.005	-0.008	-0.004	-0.004	0.000	-0.005
91.H	-0.008	0.001	0.000	0.003	-0.008	-0.003	-0.002	-0.000	-0.006
92.C	-0.008	0.002	0.000	0.004	-0.007	-0.003	-0.002	-0.001	-0.006
93.O	0.000	0.000	0.000	0.000	0.000	0.000	0.000	0.000	0.000
94.H	-0.009	-0.003	-0.002	-0.001	-0.014	0.002	0.002	0.001	-0.014
95.H	-0.009	-0.005	-0.002	-0.000	-0.017	0.002	0.001	0.003	-0.015
96.H	-0.007	-0.004	-0.002	0.001	-0.015	0.001	0.001	0.001	-0.014

	55			56			57		
1.O	0.000	0.000	0.000	0.000	0.000	0.000	0.000	0.000	0.000
2.C	0.000	0.000	0.000	0.000	0.000	0.000	0.000	0.000	0.000
3.C	0.000	0.000	0.000	0.000	0.000	0.000	0.000	0.000	0.000
4.C	0.000	0.000	0.000	0.000	0.000	0.000	0.000	0.000	0.000
5.O	0.000	0.000	0.000	0.000	0.000	0.000	0.000	0.000	0.000
6.N	-0.008	0.005	-0.002	0.001	-0.006	-0.001	-0.005	0.004	-0.007
7.O	0.000	0.000	0.000	0.000	0.000	0.000	0.000	0.000	0.000
8.C	0.000	0.000	0.000	0.000	0.000	0.000	0.000	0.000	0.000
9.C	0.000	0.000	0.000	0.000	0.000	0.000	0.000	0.000	0.000
10.C	0.000	0.000	0.000	0.000	0.000	0.000	0.000	0.000	0.000
11.O	0.000	0.000	0.000	0.000	0.000	0.000	0.000	0.000	0.000
12.C	0.000	0.000	0.000	0.000	0.000	0.000	0.000	0.000	0.000
13.O	0.000	0.000	0.000	0.000	0.000	0.000	0.000	0.000	0.000

14.C	0.000	0.000	0.000	0.000	0.000	0.000	0.000	0.000	0.000
15.C	0.281	-0.003	0.002	0.000	0.283	0.001	0.000	0.000	0.281
16.O	0.000	0.000	0.000	0.000	0.000	0.000	0.000	0.000	0.000
17.C	-0.006	-0.004	0.001	0.002	-0.012	0.000	-0.003	0.005	-0.010
18.C	-0.006	0.004	-0.002	0.002	-0.008	-0.001	0.002	-0.007	-0.005
19.O	0.000	0.000	0.000	0.000	0.000	0.000	0.000	0.000	0.000
20.C	-0.010	0.002	-0.001	0.000	-0.009	-0.000	0.003	-0.005	-0.008
21.C	-0.007	0.001	-0.001	0.002	-0.010	-0.000	0.002	-0.005	-0.006
22.C	-0.009	-0.004	0.001	0.001	-0.012	0.001	-0.001	0.003	-0.012
23.C	-0.016	0.001	0.001	-0.004	-0.010	0.000	0.005	-0.004	-0.013
24.C	-0.010	0.008	-0.003	-0.000	-0.005	-0.001	-0.003	0.002	-0.007
25.C	-0.015	0.000	0.001	-0.003	-0.010	0.000	0.003	-0.001	-0.013
26.C	-0.012	0.001	0.000	-0.002	-0.009	-0.000	-0.003	0.006	-0.012
27.C	-0.013	-0.002	0.001	-0.002	-0.010	0.000	-0.003	0.007	-0.014
28.C	-0.016	-0.003	0.002	-0.004	-0.011	0.001	-0.002	0.008	-0.017
29.C	-0.011	0.004	-0.001	-0.001	-0.008	-0.000	0.003	-0.005	-0.008
30.C	-0.010	-0.010	0.003	0.000	-0.016	0.002	0.001	0.002	-0.014
31.O	0.000	0.000	0.000	0.000	0.000	0.000	0.000	0.000	0.000
32.C	-0.006	-0.002	-0.000	0.003	-0.012	0.000	0.001	-0.004	-0.007
33.C	-0.010	0.007	-0.002	0.000	-0.006	-0.001	0.002	-0.006	-0.006
34.C	-0.015	0.004	-0.000	-0.003	-0.008	-0.000	0.005	-0.006	-0.011
35.C	-0.018	-0.003	0.003	-0.005	-0.012	0.001	0.003	0.002	-0.017
36.C	-0.002	-0.003	-0.001	0.005	-0.012	-0.000	-0.000	-0.003	-0.006
37.C	-0.005	0.006	-0.003	0.003	-0.006	-0.002	-0.007	0.004	-0.005
38.C	-0.016	-0.008	0.004	-0.004	-0.015	0.002	0.003	0.002	-0.018
39.C	-0.002	-0.006	0.000	0.005	-0.014	0.000	-0.000	-0.002	-0.007
40.H	-0.011	-0.000	0.000	-0.001	-0.011	0.000	0.004	-0.005	-0.010
41.H	-0.010	0.007	-0.002	-0.000	-0.006	-0.001	0.002	-0.005	-0.006
42.H	-0.007	0.007	-0.003	0.002	-0.006	-0.001	0.001	-0.006	-0.004
43.H	-0.011	0.009	-0.003	-0.001	-0.005	-0.001	0.003	-0.008	-0.005
44.H	-0.021	0.002	0.002	-0.006	-0.009	0.001	0.007	-0.005	-0.015

45.H	-0.021	0.001	0.002	-0.006	-0.010	0.001	0.008	-0.006	-0.015
46.H	-0.020	-0.002	0.003	-0.006	-0.011	0.001	0.007	-0.003	-0.017
47.H	-0.015	-0.001	0.002	-0.003	-0.011	0.001	0.005	-0.003	-0.013
48.H	-0.016	0.002	0.001	-0.004	-0.009	0.000	0.003	-0.002	-0.013
49.H	-0.012	0.001	0.000	-0.001	-0.010	0.000	0.002	-0.001	-0.011
50.H	-0.018	-0.001	0.002	-0.005	-0.011	0.001	0.003	0.002	-0.016
51.H	-0.018	-0.005	0.004	-0.005	-0.013	0.002	0.003	0.003	-0.018
52.H	-0.020	-0.002	0.003	-0.006	-0.011	0.001	0.005	0.000	-0.018
53.H	-0.019	-0.008	0.005	-0.005	-0.015	0.002	0.005	0.002	-0.020
54.H	-0.015	-0.009	0.004	-0.003	-0.015	0.002	0.002	0.004	-0.019
55.H	-0.016	-0.009	0.005	-0.004	-0.016	0.002	0.004	0.002	-0.019
56.H	-0.007	-0.002	0.000	0.002	-0.012	0.000	0.003	-0.005	-0.008
57.H	-0.002	-0.001	-0.002	0.005	-0.011	-0.000	0.000	-0.005	-0.004
58.H	-0.001	-0.003	-0.001	0.006	-0.012	-0.000	-0.002	-0.002	-0.005
59.H	-0.002	-0.008	0.001	0.005	-0.015	0.001	-0.001	0.000	-0.008
60.H	-0.003	-0.007	0.001	0.004	-0.014	0.001	0.001	-0.003	-0.008
61.H	0.001	-0.007	-0.000	0.007	-0.014	0.000	-0.002	-0.001	-0.005
62.H	-0.004	0.003	-0.003	0.004	-0.008	-0.001	0.002	-0.007	-0.003
63.H	-0.008	0.003	-0.002	0.001	-0.009	-0.001	0.004	-0.008	-0.006
64.H	-0.007	0.006	-0.003	0.002	-0.007	-0.001	0.003	-0.008	-0.004
65.H	-0.009	-0.003	0.001	0.001	-0.012	0.000	-0.000	0.001	-0.010
66.H	-0.006	-0.006	0.001	0.002	-0.013	0.001	-0.004	0.006	-0.011
67.H	-0.006	-0.002	-0.000	0.002	-0.011	-0.000	-0.004	0.004	-0.009
68.H	-0.004	-0.005	0.000	0.004	-0.013	0.000	-0.004	0.004	-0.009
69.C	-0.008	0.005	-0.002	0.001	-0.007	-0.001	-0.002	0.000	-0.006
70.H	-0.018	-0.001	0.002	-0.005	-0.010	0.001	-0.000	0.006	-0.017
71.H	-0.016	-0.003	0.002	-0.004	-0.011	0.001	-0.003	0.009	-0.017
72.H	-0.017	-0.005	0.003	-0.005	-0.012	0.001	-0.001	0.008	-0.018
73.H	-0.011	-0.004	0.002	-0.001	-0.012	0.001	-0.004	0.008	-0.014
74.H	-0.014	0.003	-0.000	-0.003	-0.008	-0.000	-0.002	0.005	-0.012
75.H	-0.011	0.001	-0.000	-0.001	-0.009	-0.000	-0.005	0.008	-0.012

76.H	-0.010	0.009	-0.003	-0.000	-0.004	-0.002	-0.003	0.000	-0.006
77.H	-0.013	0.008	-0.002	-0.002	-0.005	-0.001	-0.002	0.002	-0.009
78.H	-0.010	0.010	-0.004	-0.000	-0.003	-0.002	-0.004	0.002	-0.006
79.H	-0.004	0.006	-0.003	0.003	-0.006	-0.002	-0.006	0.003	-0.004
80.H	-0.003	0.004	-0.003	0.003	-0.007	-0.001	-0.008	0.006	-0.005
81.H	-0.004	0.008	-0.004	0.003	-0.005	-0.002	-0.007	0.004	-0.003
82.H	-0.009	-0.003	0.001	0.000	-0.011	0.000	-0.003	0.006	-0.012
83.H	-0.012	-0.006	0.002	-0.002	-0.013	0.001	-0.001	0.005	-0.015
84.H	-0.012	0.004	-0.001	-0.001	-0.007	-0.001	-0.001	0.002	-0.010
85.H	-0.007	0.001	-0.001	0.001	-0.009	-0.000	-0.006	0.007	-0.009
86.H	-0.004	0.000	-0.002	0.004	-0.010	-0.001	-0.001	-0.002	-0.005
87.O	0.000	0.000	0.000	0.000	0.000	0.000	0.000	0.000	0.000
88.H	-0.005	-0.010	0.002	0.003	-0.016	0.001	-0.002	0.004	-0.012
89.H	-0.005	0.006	-0.003	0.003	-0.006	-0.002	-0.003	-0.002	-0.003
90.H	-0.003	0.004	-0.003	0.004	-0.008	-0.001	-0.005	0.001	-0.004
91.H	-0.005	0.002	-0.002	0.003	-0.008	-0.001	-0.002	-0.000	-0.005
92.C	-0.005	0.004	-0.003	0.003	-0.007	-0.001	-0.003	-0.000	-0.004
93.O	0.000	0.000	0.000	0.000	0.000	0.000	0.000	0.000	0.000
94.H	-0.012	-0.008	0.003	-0.001	-0.015	0.002	0.003	0.001	-0.015
95.H	-0.010	-0.012	0.004	-0.000	-0.017	0.002	0.001	0.004	-0.016
96.H	-0.008	-0.010	0.003	0.001	-0.016	0.002	0.001	0.001	-0.014

	58			59			60		
	------------------------			------------------------			------------------------		
1.O	0.000	0.000	0.000	0.000	0.000	0.000	0.000	0.000	0.000
2.C	0.000	0.000	0.000	0.000	0.000	0.000	0.000	0.000	0.000
3.C	0.000	0.000	0.000	0.000	0.000	0.000	0.000	0.000	0.000
4.C	0.000	0.000	0.000	0.000	0.000	0.000	0.000	0.000	0.000
5.O	0.000	0.000	0.000	0.000	0.000	0.000	0.000	0.000	0.000
6.N	-0.017	-0.004	0.006	0.002	-0.011	-0.001	-0.005	-0.003	-0.006

7.O	0.000	0.000	0.000	0.000	0.000	0.000	0.000	0.000	0.000
8.C	0.000	0.000	0.000	0.000	0.000	0.000	0.000	0.000	0.000
9.C	0.000	0.000	0.000	0.000	0.000	0.000	0.000	0.000	0.000
10.C	0.000	0.000	0.000	0.000	0.000	0.000	0.000	0.000	0.000
11.O	0.000	0.000	0.000	0.000	0.000	0.000	0.000	0.000	0.000
12.C	0.000	0.000	0.000	0.000	0.000	0.000	0.000	0.000	0.000
13.O	0.000	0.000	0.000	0.000	0.000	0.000	0.000	0.000	0.000
14.C	0.000	0.000	0.000	0.000	0.000	0.000	0.000	0.000	0.000
15.C	0.000	0.000	0.000	0.000	0.000	0.000	0.000	0.000	0.000
16.O	0.000	0.000	0.000	0.000	0.000	0.000	0.000	0.000	0.000
17.C	0.281	0.004	-0.002	0.000	0.280	-0.001	0.000	0.000	0.283
18.C	-0.008	-0.003	0.002	0.004	-0.001	-0.006	0.004	0.002	-0.012
19.O	0.000	0.000	0.000	0.000	0.000	0.000	0.000	0.000	0.000
20.C	-0.006	-0.001	0.000	0.000	-0.005	-0.002	0.003	0.002	-0.011
21.C	-0.009	-0.001	0.001	0.003	-0.005	-0.004	0.003	0.002	-0.012
22.C	-0.012	0.004	-0.003	0.001	-0.016	0.001	-0.001	-0.000	-0.012
23.C	-0.002	-0.001	-0.002	-0.006	-0.007	0.004	0.003	0.001	-0.010
24.C	-0.015	-0.006	0.007	-0.000	-0.008	-0.001	-0.004	-0.003	-0.005
25.C	-0.005	-0.000	-0.001	-0.005	-0.009	0.003	0.001	0.001	-0.010
26.C	-0.014	-0.001	0.002	-0.002	-0.015	0.003	-0.004	-0.003	-0.007
27.C	-0.013	0.002	-0.001	-0.003	-0.018	0.005	-0.004	-0.003	-0.008
28.C	-0.011	0.003	-0.002	-0.006	-0.019	0.008	-0.004	-0.003	-0.008
29.C	-0.006	-0.003	0.002	-0.001	-0.003	-0.001	0.003	0.001	-0.010
30.C	-0.009	0.008	-0.008	0.000	-0.018	0.002	0.001	0.002	-0.015
31.O	0.000	0.000	0.000	0.000	0.000	0.000	0.000	0.000	0.000
32.C	-0.010	0.001	-0.002	0.004	-0.008	-0.004	0.003	0.002	-0.014
33.C	-0.007	-0.005	0.004	0.000	-0.001	-0.003	0.002	0.001	-0.009
34.C	-0.003	-0.003	0.001	-0.005	-0.003	0.001	0.003	0.002	-0.009
35.C	-0.004	0.002	-0.004	-0.008	-0.014	0.007	0.000	0.000	-0.010
36.C	-0.012	0.002	-0.002	0.007	-0.009	-0.006	0.002	0.002	-0.015
37.C	-0.020	-0.004	0.007	0.005	-0.011	-0.004	-0.005	-0.004	-0.007

38.C	-0.004	0.006	-0.008	-0.006	-0.017	0.007	0.001	0.001	-0.013
39.C	-0.013	0.005	-0.004	0.008	-0.012	-0.005	0.002	0.003	-0.016
40.H	-0.005	0.000	-0.002	-0.001	-0.006	-0.000	0.003	0.002	-0.012
41.H	-0.008	-0.005	0.005	-0.000	-0.002	-0.003	0.001	0.000	-0.008
42.H	-0.009	-0.005	0.005	0.002	-0.001	-0.005	0.002	0.001	-0.009
43.H	-0.005	-0.007	0.006	-0.001	0.002	-0.003	0.003	0.001	-0.008
44.H	0.002	-0.002	-0.001	-0.011	-0.005	0.007	0.004	0.001	-0.008
45.H	0.003	-0.001	-0.003	-0.010	-0.004	0.006	0.005	0.002	-0.009
46.H	0.001	0.001	-0.004	-0.010	-0.009	0.008	0.003	0.002	-0.010
47.H	-0.003	0.001	-0.003	-0.005	-0.008	0.003	0.003	0.002	-0.011
48.H	-0.005	-0.002	-0.000	-0.006	-0.008	0.004	0.001	0.000	-0.008
49.H	-0.008	-0.001	-0.000	-0.002	-0.009	0.001	0.001	0.000	-0.010
50.H	-0.005	0.001	-0.003	-0.008	-0.013	0.007	-0.000	-0.000	-0.009
51.H	-0.005	0.004	-0.006	-0.008	-0.016	0.008	-0.000	-0.000	-0.010
52.H	-0.002	0.002	-0.004	-0.010	-0.012	0.008	0.001	0.001	-0.009
53.H	-0.002	0.006	-0.008	-0.009	-0.017	0.009	0.002	0.001	-0.012
54.H	-0.006	0.007	-0.009	-0.005	-0.020	0.007	0.000	0.001	-0.013
55.H	-0.004	0.007	-0.009	-0.006	-0.018	0.007	0.002	0.002	-0.014
56.H	-0.007	0.002	-0.002	0.003	-0.007	-0.003	0.004	0.003	-0.014
57.H	-0.012	0.001	-0.000	0.008	-0.007	-0.007	0.003	0.002	-0.015
58.H	-0.015	0.002	-0.001	0.009	-0.010	-0.007	0.001	0.002	-0.015
59.H	-0.013	0.006	-0.005	0.007	-0.015	-0.004	0.002	0.002	-0.017
60.H	-0.011	0.005	-0.005	0.007	-0.012	-0.005	0.003	0.003	-0.017
61.H	-0.015	0.005	-0.004	0.010	-0.013	-0.007	0.002	0.003	-0.017
62.H	-0.010	-0.003	0.003	0.006	-0.002	-0.008	0.004	0.002	-0.012
63.H	-0.005	-0.003	0.001	0.002	-0.001	-0.005	0.005	0.003	-0.012
64.H	-0.007	-0.005	0.004	0.003	0.001	-0.006	0.004	0.002	-0.010
65.H	-0.011	0.002	-0.002	0.001	-0.013	-0.000	-0.000	0.000	-0.012
66.H	-0.017	0.005	-0.003	0.004	-0.020	0.000	-0.003	-0.001	-0.013
67.H	-0.016	0.002	-0.000	0.004	-0.015	-0.002	-0.003	-0.001	-0.011
68.H	-0.017	0.004	-0.002	0.006	-0.017	-0.002	-0.002	-0.001	-0.013

69.C	-0.014	-0.003	0.004	0.002	-0.008	-0.003	-0.002	-0.001	-0.008
70.H	-0.009	0.001	-0.002	-0.008	-0.017	0.009	-0.003	-0.003	-0.007
71.H	-0.013	0.003	-0.002	-0.006	-0.021	0.008	-0.005	-0.003	-0.008
72.H	-0.010	0.004	-0.004	-0.007	-0.021	0.009	-0.004	-0.002	-0.009
73.H	-0.015	0.003	-0.002	-0.001	-0.020	0.004	-0.005	-0.003	-0.009
74.H	-0.012	-0.002	0.002	-0.004	-0.013	0.004	-0.004	-0.003	-0.006
75.H	-0.016	-0.000	0.002	-0.001	-0.017	0.003	-0.005	-0.004	-0.007
76.H	-0.014	-0.006	0.007	0.000	-0.006	-0.002	-0.003	-0.003	-0.005
77.H	-0.013	-0.006	0.006	-0.003	-0.008	0.001	-0.003	-0.003	-0.005
78.H	-0.016	-0.007	0.009	0.000	-0.006	-0.002	-0.004	-0.004	-0.004
79.H	-0.020	-0.004	0.007	0.006	-0.010	-0.005	-0.004	-0.003	-0.007
80.H	-0.022	-0.002	0.006	0.006	-0.014	-0.004	-0.006	-0.004	-0.008
81.H	-0.021	-0.005	0.009	0.006	-0.010	-0.004	-0.006	-0.004	-0.006
82.H	-0.015	0.003	-0.001	0.001	-0.018	0.002	-0.003	-0.002	-0.010
83.H	-0.011	0.005	-0.005	-0.002	-0.018	0.005	-0.001	-0.001	-0.011
84.H	-0.012	-0.003	0.003	-0.002	-0.010	0.001	-0.002	-0.002	-0.007
85.H	-0.018	0.000	0.002	0.003	-0.016	-0.000	-0.005	-0.003	-0.009
86.H	-0.013	-0.000	0.001	0.006	-0.008	-0.005	0.001	0.001	-0.012
87.O	0.000	0.000	0.000	0.000	0.000	0.000	0.000	0.000	0.000
88.H	-0.014	0.008	-0.007	0.005	-0.020	-0.000	-0.000	0.001	-0.016
89.H	-0.015	-0.005	0.006	0.005	-0.005	-0.006	-0.001	-0.001	-0.009
90.H	-0.018	-0.003	0.005	0.007	-0.009	-0.006	-0.002	-0.001	-0.010
91.H	-0.015	-0.002	0.003	0.005	-0.009	-0.004	-0.001	-0.001	-0.010
92.C	-0.016	-0.003	0.005	0.005	-0.008	-0.005	-0.001	-0.001	-0.009
93.O	0.000	0.000	0.000	0.000	0.000	0.000	0.000	0.000	0.000
94.H	-0.006	0.006	-0.007	-0.002	-0.016	0.003	0.002	0.002	-0.014
95.H	-0.009	0.009	-0.010	-0.000	-0.021	0.004	0.001	0.002	-0.016
96.H	-0.009	0.008	-0.008	0.002	-0.018	0.001	0.002	0.002	-0.016

61 62 63

	------------------------			------------------------			------------------------		
1.O	0.000	0.000	0.000	0.000	0.000	0.000	0.000	0.000	0.000
2.C	0.000	0.000	0.000	0.000	0.000	0.000	0.000	0.000	0.000
3.C	0.000	0.000	0.000	0.000	0.000	0.000	0.000	0.000	0.000
4.C	0.000	0.000	0.000	0.000	0.000	0.000	0.000	0.000	0.000
5.O	0.000	0.000	0.000	0.000	0.000	0.000	0.000	0.000	0.000
6.N	0.002	-0.005	-0.008	-0.002	-0.001	-0.001	0.002	-0.007	-0.011
7.O	0.000	0.000	0.000	0.000	0.000	0.000	0.000	0.000	0.000
8.C	0.000	0.000	0.000	0.000	0.000	0.000	0.000	0.000	0.000
9.C	0.000	0.000	0.000	0.000	0.000	0.000	0.000	0.000	0.000
10.C	0.000	0.000	0.000	0.000	0.000	0.000	0.000	0.000	0.000
11.O	0.000	0.000	0.000	0.000	0.000	0.000	0.000	0.000	0.000
12.C	0.000	0.000	0.000	0.000	0.000	0.000	0.000	0.000	0.000
13.O	0.000	0.000	0.000	0.000	0.000	0.000	0.000	0.000	0.000
14.C	0.000	0.000	0.000	0.000	0.000	0.000	0.000	0.000	0.000
15.C	0.000	0.000	0.000	0.000	0.000	0.000	0.000	0.000	0.000
16.O	0.000	0.000	0.000	0.000	0.000	0.000	0.000	0.000	0.000
17.C	0.000	0.000	0.000	0.000	0.000	0.000	0.000	0.000	0.000
18.C	0.275	-0.002	0.000	0.000	0.276	0.010	0.000	0.000	0.280
19.O	0.000	0.000	0.000	0.000	0.000	0.000	0.000	0.000	0.000
20.C	-0.021	-0.001	0.001	0.000	-0.021	0.003	-0.001	0.008	-0.013
21.C	-0.024	-0.000	0.002	-0.002	-0.021	0.008	0.001	0.008	-0.017
22.C	-0.009	0.004	0.004	-0.001	-0.005	-0.001	0.001	-0.004	-0.010
23.C	-0.013	0.000	0.000	0.005	-0.018	-0.006	-0.004	0.006	-0.006
24.C	0.001	-0.007	-0.011	-0.000	-0.005	-0.002	0.001	-0.004	-0.010
25.C	-0.010	0.000	0.000	0.003	-0.014	-0.006	-0.002	0.002	-0.007
26.C	0.006	-0.002	-0.005	0.001	0.003	-0.007	0.001	-0.010	-0.006
27.C	0.006	0.001	-0.001	0.002	0.004	-0.010	0.000	-0.010	-0.004
28.C	0.010	0.002	-0.001	0.004	0.006	-0.014	-0.001	-0.011	-0.000
29.C	-0.019	-0.003	-0.002	0.001	-0.022	0.002	-0.002	0.008	-0.012
30.C	-0.016	0.009	0.011	-0.000	-0.008	-0.001	-0.000	-0.001	-0.009

31.O	0.000	0.000	0.000	0.000	0.000	0.000	0.000	0.000	0.000
32.C	-0.025	0.003	0.005	-0.003	-0.019	0.008	0.001	0.006	-0.016
33.C	-0.019	-0.005	-0.005	0.000	-0.023	0.004	-0.001	0.009	-0.014
34.C	-0.016	-0.003	-0.002	0.004	-0.022	-0.003	-0.003	0.008	-0.009
35.C	-0.003	0.003	0.002	0.006	-0.008	-0.012	-0.004	-0.002	-0.002
36.C	-0.027	0.003	0.006	-0.005	-0.017	0.013	0.002	0.005	-0.020
37.C	0.000	-0.006	-0.008	-0.004	-0.000	0.004	0.004	-0.008	-0.014
38.C	-0.007	0.007	0.008	0.005	-0.007	-0.011	-0.003	-0.002	-0.002
39.C	-0.028	0.006	0.010	-0.006	-0.015	0.013	0.003	0.004	-0.019
40.H	-0.020	0.001	0.003	0.001	-0.021	0.001	-0.002	0.007	-0.011
41.H	-0.015	-0.005	-0.006	0.000	-0.020	0.003	-0.001	0.007	-0.013
42.H	-0.020	-0.005	-0.005	-0.002	-0.022	0.008	0.000	0.008	-0.017
43.H	-0.020	-0.007	-0.007	0.001	-0.026	0.004	-0.002	0.011	-0.014
44.H	-0.010	-0.001	-0.002	0.008	-0.020	-0.012	-0.006	0.007	-0.002
45.H	-0.014	-0.000	0.000	0.008	-0.023	-0.011	-0.006	0.009	-0.003
46.H	-0.010	0.002	0.002	0.008	-0.017	-0.013	-0.006	0.005	-0.001
47.H	-0.015	0.002	0.003	0.004	-0.018	-0.005	-0.003	0.005	-0.007
48.H	-0.008	-0.001	-0.002	0.004	-0.014	-0.007	-0.003	0.003	-0.006
49.H	-0.011	-0.000	-0.000	0.002	-0.013	-0.002	-0.001	0.002	-0.009
50.H	-0.002	0.001	-0.000	0.006	-0.007	-0.012	-0.003	-0.002	-0.002
51.H	-0.001	0.005	0.004	0.006	-0.004	-0.014	-0.003	-0.004	-0.000
52.H	-0.004	0.002	0.002	0.007	-0.011	-0.015	-0.005	0.000	-0.000
53.H	-0.006	0.007	0.007	0.007	-0.008	-0.014	-0.004	-0.001	0.000
54.H	-0.005	0.008	0.009	0.004	-0.003	-0.011	-0.002	-0.005	-0.002
55.H	-0.009	0.009	0.010	0.004	-0.007	-0.010	-0.003	-0.002	-0.003
56.H	-0.026	0.003	0.006	-0.002	-0.021	0.007	-0.000	0.008	-0.015
57.H	-0.030	0.002	0.005	-0.006	-0.021	0.015	0.003	0.008	-0.021
58.H	-0.026	0.003	0.006	-0.007	-0.015	0.014	0.003	0.004	-0.021
59.H	-0.025	0.008	0.011	-0.006	-0.012	0.011	0.003	0.002	-0.018
60.H	-0.029	0.007	0.011	-0.005	-0.018	0.012	0.002	0.006	-0.018
61.H	-0.030	0.007	0.011	-0.008	-0.015	0.016	0.004	0.004	-0.022

62.H	-0.030	-0.002	0.001	-0.004	-0.026	0.014	0.001	0.011	-0.021
63.H	-0.029	-0.002	0.001	-0.001	-0.028	0.009	-0.001	0.013	-0.017
64.H	-0.027	-0.004	-0.002	-0.002	-0.028	0.010	0.000	0.012	-0.018
65.H	-0.012	0.002	0.003	-0.001	-0.009	0.001	0.001	-0.001	-0.011
66.H	-0.006	0.005	0.005	-0.003	0.002	0.001	0.003	-0.009	-0.011
67.H	-0.008	0.002	0.002	-0.004	-0.002	0.003	0.003	-0.006	-0.013
68.H	-0.011	0.004	0.005	-0.005	-0.002	0.006	0.003	-0.006	-0.014
69.C	-0.009	-0.004	-0.005	-0.002	-0.010	0.003	0.002	-0.001	-0.014
70.H	0.010	0.000	-0.003	0.006	0.003	-0.016	-0.002	-0.009	0.001
71.H	0.012	0.002	-0.001	0.004	0.009	-0.015	-0.001	-0.014	-0.000
72.H	0.009	0.004	0.001	0.005	0.007	-0.016	-0.002	-0.012	0.001
73.H	0.006	0.003	0.000	0.000	0.007	-0.008	0.001	-0.012	-0.005
74.H	0.006	-0.003	-0.006	0.003	-0.000	-0.009	-0.000	-0.008	-0.005
75.H	0.009	-0.002	-0.005	0.000	0.006	-0.008	0.001	-0.012	-0.006
76.H	-0.002	-0.008	-0.011	-0.000	-0.008	-0.000	0.001	-0.002	-0.011
77.H	0.003	-0.007	-0.011	0.002	-0.005	-0.005	0.000	-0.004	-0.008
78.H	0.002	-0.009	-0.013	-0.001	-0.005	-0.001	0.002	-0.004	-0.011
79.H	-0.003	-0.006	-0.008	-0.005	-0.003	0.006	0.004	-0.006	-0.016
80.H	0.001	-0.004	-0.006	-0.006	0.003	0.005	0.005	-0.010	-0.015
81.H	0.001	-0.007	-0.010	-0.005	-0.000	0.005	0.005	-0.008	-0.015
82.H	-0.001	0.002	0.001	-0.001	0.001	-0.003	0.002	-0.008	-0.009
83.H	-0.003	0.005	0.004	0.001	-0.001	-0.007	-0.000	-0.006	-0.005
84.H	-0.002	-0.004	-0.006	0.001	-0.006	-0.004	-0.000	-0.003	-0.008
85.H	0.002	-0.001	-0.003	-0.003	0.004	-0.001	0.003	-0.010	-0.010
86.H	-0.020	0.000	0.002	-0.004	-0.015	0.010	0.002	0.003	-0.018
87.O	0.000	0.000	0.000	0.000	0.000	0.000	0.000	0.000	0.000
88.H	-0.015	0.009	0.011	-0.004	-0.004	0.004	0.002	-0.004	-0.013
89.H	-0.014	-0.005	-0.006	-0.004	-0.014	0.009	0.003	0.002	-0.018
90.H	-0.012	-0.003	-0.004	-0.006	-0.008	0.010	0.004	-0.002	-0.018
91.H	-0.014	-0.002	-0.002	-0.004	-0.011	0.007	0.002	0.000	-0.016
92.C	-0.012	-0.004	-0.004	-0.004	-0.011	0.008	0.003	-0.000	-0.017

93.O	0.000	0.000	0.000	0.000	0.000	0.000	0.000	0.000	0.000
94.H	-0.016	0.008	0.010	0.001	-0.011	-0.003	-0.001	0.001	-0.008
95.H	-0.014	0.011	0.013	0.000	-0.005	-0.003	-0.000	-0.003	-0.007
96.H	-0.019	0.010	0.013	-0.001	-0.009	0.001	0.000	-0.000	-0.011

	64			65			66		
	------------------------			------------------------			------------------------		
1.O	0.000	0.000	0.000	0.000	0.000	0.000	0.000	0.000	0.000
2.C	0.000	0.000	0.000	0.000	0.000	0.000	0.000	0.000	0.000
3.C	0.000	0.000	0.000	0.000	0.000	0.000	0.000	0.000	0.000
4.C	0.000	0.000	0.000	0.000	0.000	0.000	0.000	0.000	0.000
5.O	0.000	0.000	0.000	0.000	0.000	0.000	0.000	0.000	0.000
6.N	-0.001	-0.001	-0.007	-0.001	-0.002	-0.001	-0.000	-0.003	-0.010
7.O	0.000	0.000	0.000	0.000	0.000	0.000	0.000	0.000	0.000
8.C	0.000	0.000	0.000	0.000	0.000	0.000	0.000	0.000	0.000
9.C	0.000	0.000	0.000	0.000	0.000	0.000	0.000	0.000	0.000
10.C	0.000	0.000	0.000	0.000	0.000	0.000	0.000	0.000	0.000
11.O	0.000	0.000	0.000	0.000	0.000	0.000	0.000	0.000	0.000
12.C	0.000	0.000	0.000	0.000	0.000	0.000	0.000	0.000	0.000
13.O	0.000	0.000	0.000	0.000	0.000	0.000	0.000	0.000	0.000
14.C	0.000	0.000	0.000	0.000	0.000	0.000	0.000	0.000	0.000
15.C	0.000	0.000	0.000	0.000	0.000	0.000	0.000	0.000	0.000
16.O	0.000	0.000	0.000	0.000	0.000	0.000	0.000	0.000	0.000
17.C	0.000	0.000	0.000	0.000	0.000	0.000	0.000	0.000	0.000
18.C	0.000	0.000	0.000	0.000	0.000	0.000	0.000	0.000	0.000
19.O	0.000	0.000	0.000	0.000	0.000	0.000	0.000	0.000	0.000
20.C	0.279	0.000	0.000	0.000	0.279	0.003	0.000	0.000	0.283
21.C	-0.019	0.000	0.000	-0.001	-0.019	0.007	0.001	0.003	-0.013
22.C	-0.010	0.000	0.004	-0.001	-0.008	0.000	0.000	-0.001	-0.011
23.C	-0.017	0.000	0.001	0.002	-0.018	-0.004	-0.001	0.002	-0.010

24.C	-0.002	-0.002	-0.010	-0.000	-0.005	-0.002	-0.000	-0.002	-0.010
25.C	-0.013	0.000	0.001	0.002	-0.014	-0.004	-0.001	0.001	-0.010
26.C	0.000	-0.001	-0.003	0.000	-0.001	-0.006	-0.001	-0.004	-0.009
27.C	-0.000	-0.000	-0.000	0.001	-0.000	-0.007	-0.001	-0.004	-0.009
28.C	0.001	-0.000	0.001	0.002	0.001	-0.011	-0.001	-0.004	-0.007
29.C	-0.018	0.000	-0.002	0.001	-0.020	0.002	0.000	0.003	-0.012
30.C	-0.015	0.001	0.010	0.000	-0.012	0.000	0.000	0.000	-0.012
31.O	0.000	0.000	0.000	0.000	0.000	0.000	0.000	0.000	0.000
32.C	-0.020	0.001	0.004	-0.001	-0.018	0.007	0.001	0.003	-0.014
33.C	-0.017	-0.000	-0.005	0.000	-0.020	0.003	0.000	0.003	-0.012
34.C	-0.018	0.000	-0.002	0.002	-0.020	-0.002	-0.000	0.003	-0.010
35.C	-0.010	0.000	0.003	0.003	-0.010	-0.009	-0.001	-0.000	-0.008
36.C	-0.020	0.001	0.004	-0.002	-0.018	0.010	0.001	0.002	-0.015
37.C	-0.000	-0.001	-0.008	-0.002	-0.002	0.003	0.000	-0.003	-0.012
38.C	-0.012	0.001	0.008	0.002	-0.010	-0.008	-0.001	-0.000	-0.009
39.C	-0.020	0.001	0.008	-0.003	-0.017	0.010	0.001	0.002	-0.015
40.H	-0.020	0.001	0.002	0.001	-0.019	0.001	0.000	0.003	-0.012
41.H	-0.015	-0.001	-0.006	0.000	-0.018	0.002	0.000	0.002	-0.012
42.H	-0.017	-0.000	-0.005	-0.001	-0.019	0.006	0.001	0.003	-0.013
43.H	-0.019	-0.001	-0.007	0.001	-0.022	0.003	0.000	0.004	-0.012
44.H	-0.016	0.000	-0.000	0.004	-0.019	-0.009	-0.001	0.003	-0.008
45.H	-0.020	0.000	0.001	0.004	-0.021	-0.008	-0.001	0.004	-0.008
46.H	-0.017	0.001	0.003	0.004	-0.017	-0.009	-0.001	0.002	-0.008
47.H	-0.017	0.001	0.003	0.002	-0.017	-0.003	-0.000	0.002	-0.010
48.H	-0.013	-0.000	-0.001	0.002	-0.014	-0.005	-0.001	0.001	-0.009
49.H	-0.013	0.000	-0.000	0.001	-0.014	-0.001	-0.000	0.001	-0.011
50.H	-0.009	0.000	0.001	0.003	-0.009	-0.009	-0.001	-0.001	-0.008
51.H	-0.008	0.001	0.005	0.003	-0.008	-0.010	-0.001	-0.001	-0.008
52.H	-0.012	0.000	0.003	0.004	-0.012	-0.011	-0.001	0.000	-0.007
53.H	-0.012	0.001	0.008	0.003	-0.011	-0.010	-0.001	-0.000	-0.008
54.H	-0.010	0.001	0.009	0.002	-0.007	-0.008	-0.001	-0.001	-0.009

55.H	-0.014	0.001	0.010	0.002	-0.011	-0.007	-0.001	-0.000	-0.009
56.H	-0.022	0.001	0.005	-0.000	-0.021	0.006	0.001	0.003	-0.014
57.H	-0.022	0.001	0.003	-0.003	-0.020	0.012	0.002	0.003	-0.016
58.H	-0.018	0.001	0.004	-0.003	-0.016	0.011	0.001	0.002	-0.015
59.H	-0.018	0.001	0.009	-0.002	-0.014	0.009	0.001	0.001	-0.015
60.H	-0.022	0.001	0.009	-0.002	-0.019	0.010	0.001	0.003	-0.015
61.H	-0.020	0.001	0.008	-0.003	-0.016	0.013	0.002	0.002	-0.016
62.H	-0.023	0.000	-0.001	-0.002	-0.023	0.011	0.001	0.004	-0.015
63.H	-0.024	0.000	-0.000	-0.000	-0.025	0.007	0.001	0.005	-0.014
64.H	-0.022	-0.000	-0.003	-0.001	-0.024	0.008	0.001	0.005	-0.014
65.H	-0.012	0.000	0.003	-0.000	-0.010	0.001	0.000	-0.000	-0.012
66.H	-0.005	0.000	0.005	-0.002	-0.003	0.001	0.000	-0.003	-0.012
67.H	-0.007	-0.000	0.001	-0.002	-0.005	0.003	0.000	-0.002	-0.012
68.H	-0.008	0.000	0.004	-0.002	-0.006	0.005	0.001	-0.002	-0.013
69.C	-0.008	-0.001	-0.005	-0.001	-0.010	0.002	0.000	-0.000	-0.012
70.H	-0.000	-0.000	-0.001	0.002	-0.001	-0.012	-0.001	-0.004	-0.007
71.H	0.003	-0.000	0.000	0.001	0.003	-0.011	-0.001	-0.005	-0.007
72.H	0.000	-0.000	0.003	0.002	0.001	-0.012	-0.001	-0.004	-0.007
73.H	0.001	-0.000	0.001	-0.000	0.002	-0.006	-0.001	-0.005	-0.009
74.H	-0.001	-0.001	-0.005	0.001	-0.002	-0.007	-0.001	-0.003	-0.008
75.H	0.003	-0.001	-0.004	-0.000	0.002	-0.006	-0.001	-0.005	-0.009
76.H	-0.005	-0.001	-0.010	-0.000	-0.008	-0.000	-0.000	-0.001	-0.010
77.H	-0.002	-0.001	-0.009	0.000	-0.005	-0.004	-0.001	-0.002	-0.009
78.H	-0.001	-0.002	-0.012	-0.001	-0.005	-0.001	-0.000	-0.002	-0.010
79.H	-0.003	-0.001	-0.008	-0.003	-0.004	0.004	0.000	-0.002	-0.012
80.H	0.001	-0.001	-0.006	-0.003	0.000	0.004	0.000	-0.004	-0.012
81.H	0.001	-0.002	-0.010	-0.003	-0.001	0.004	0.000	-0.003	-0.012
82.H	-0.004	-0.000	0.001	-0.001	-0.002	-0.002	-0.000	-0.003	-0.010
83.H	-0.007	0.000	0.005	0.001	-0.005	-0.005	-0.000	-0.002	-0.010
84.H	-0.006	-0.001	-0.005	0.000	-0.007	-0.003	-0.000	-0.001	-0.010
85.H	-0.000	-0.001	-0.003	-0.002	0.000	-0.000	-0.000	-0.004	-0.011

86.H	-0.016	0.000	0.000	-0.002	-0.015	0.008	0.001	0.001	-0.014
87.O	0.000	0.000	0.000	0.000	0.000	0.000	0.000	0.000	0.000
88.H	-0.012	0.001	0.009	-0.002	-0.008	0.004	0.001	-0.001	-0.013
89.H	-0.011	-0.001	-0.006	-0.002	-0.013	0.007	0.001	0.001	-0.013
90.H	-0.008	-0.001	-0.005	-0.003	-0.008	0.007	0.001	-0.001	-0.014
91.H	-0.011	-0.000	-0.003	-0.002	-0.011	0.006	0.001	0.000	-0.013
92.C	-0.010	-0.001	-0.005	-0.002	-0.011	0.006	0.001	-0.000	-0.013
93.O	0.000	0.000	0.000	0.000	0.000	0.000	0.000	0.000	0.000
94.H	-0.016	0.001	0.009	0.001	-0.013	-0.001	0.000	0.001	-0.011
95.H	-0.014	0.002	0.012	0.000	-0.010	-0.001	0.000	-0.001	-0.011
96.H	-0.017	0.002	0.011	-0.000	-0.013	0.002	0.000	0.001	-0.012

	67			68			69		
1.O	0.000	0.000	0.000	0.000	0.000	0.000	0.000	0.000	0.000
2.C	0.000	0.000	0.000	0.000	0.000	0.000	0.000	0.000	0.000
3.C	0.000	0.000	0.000	0.000	0.000	0.000	0.000	0.000	0.000
4.C	0.000	0.000	0.000	0.000	0.000	0.000	0.000	0.000	0.000
5.O	0.000	0.000	0.000	0.000	0.000	0.000	0.000	0.000	0.000
6.N	-0.001	-0.005	-0.007	-0.001	-0.002	-0.001	0.000	-0.007	-0.010
7.O	0.000	0.000	0.000	0.000	0.000	0.000	0.000	0.000	0.000
8.C	0.000	0.000	0.000	0.000	0.000	0.000	0.000	0.000	0.000
9.C	0.000	0.000	0.000	0.000	0.000	0.000	0.000	0.000	0.000
10.C	0.000	0.000	0.000	0.000	0.000	0.000	0.000	0.000	0.000
11.O	0.000	0.000	0.000	0.000	0.000	0.000	0.000	0.000	0.000
12.C	0.000	0.000	0.000	0.000	0.000	0.000	0.000	0.000	0.000
13.O	0.000	0.000	0.000	0.000	0.000	0.000	0.000	0.000	0.000
14.C	0.000	0.000	0.000	0.000	0.000	0.000	0.000	0.000	0.000
15.C	0.000	0.000	0.000	0.000	0.000	0.000	0.000	0.000	0.000
16.O	0.000	0.000	0.000	0.000	0.000	0.000	0.000	0.000	0.000

17.C	0.000	0.000	0.000	0.000	0.000	0.000	0.000	0.000	0.000
18.C	0.000	0.000	0.000	0.000	0.000	0.000	0.000	0.000	0.000
19.O	0.000	0.000	0.000	0.000	0.000	0.000	0.000	0.000	0.000
20.C	0.000	0.000	0.000	0.000	0.000	0.000	0.000	0.000	0.000
21.C	0.277	-0.001	0.002	0.000	0.278	0.007	0.000	0.000	0.280
22.C	-0.011	0.004	0.004	-0.001	-0.009	0.000	0.000	-0.002	-0.012
23.C	-0.014	-0.000	0.000	0.002	-0.019	-0.005	-0.002	0.006	-0.008
24.C	-0.002	-0.007	-0.009	-0.000	-0.005	-0.002	-0.001	-0.004	-0.009
25.C	-0.011	-0.000	-0.000	0.002	-0.015	-0.004	-0.001	0.003	-0.008
26.C	0.002	-0.002	-0.004	0.000	-0.000	-0.006	-0.001	-0.009	-0.007
27.C	0.002	0.001	-0.001	0.000	-0.000	-0.007	-0.002	-0.009	-0.006
28.C	0.006	0.002	-0.001	0.001	0.001	-0.011	-0.003	-0.010	-0.002
29.C	-0.019	-0.003	-0.001	0.001	-0.021	0.002	0.000	0.008	-0.013
30.C	-0.017	0.009	0.009	0.000	-0.013	0.000	0.001	0.000	-0.013
31.O	0.000	0.000	0.000	0.000	0.000	0.000	0.000	0.000	0.000
32.C	-0.025	0.002	0.005	-0.001	-0.020	0.008	0.002	0.007	-0.018
33.C	-0.019	-0.006	-0.004	0.000	-0.021	0.004	0.001	0.008	-0.014
34.C	-0.016	-0.003	-0.002	0.002	-0.021	-0.002	-0.001	0.008	-0.010
35.C	-0.005	0.003	0.001	0.003	-0.011	-0.010	-0.003	-0.001	-0.004
36.C	-0.028	0.003	0.006	-0.002	-0.019	0.011	0.003	0.006	-0.021
37.C	-0.004	-0.005	-0.007	-0.002	-0.001	0.003	0.001	-0.007	-0.014
38.C	-0.009	0.007	0.006	0.002	-0.011	-0.008	-0.002	-0.001	-0.006
39.C	-0.028	0.006	0.009	-0.002	-0.018	0.012	0.003	0.005	-0.022
40.H	-0.021	0.001	0.002	0.001	-0.021	0.002	0.000	0.008	-0.013
41.H	-0.016	-0.006	-0.005	0.000	-0.019	0.002	0.000	0.006	-0.013
42.H	-0.021	-0.006	-0.003	-0.001	-0.020	0.006	0.001	0.008	-0.016
43.H	-0.020	-0.007	-0.005	0.001	-0.023	0.003	0.000	0.010	-0.014
44.H	-0.010	-0.002	-0.002	0.004	-0.020	-0.010	-0.003	0.007	-0.004
45.H	-0.014	-0.001	-0.000	0.004	-0.023	-0.008	-0.003	0.009	-0.005
46.H	-0.010	0.002	0.001	0.004	-0.019	-0.010	-0.003	0.006	-0.004
47.H	-0.015	0.001	0.002	0.002	-0.019	-0.003	-0.001	0.006	-0.009

48.H	-0.009	-0.002	-0.002	0.002	-0.015	-0.005	-0.002	0.003	-0.007
49.H	-0.013	-0.000	-0.000	0.001	-0.015	-0.001	-0.000	0.003	-0.010
50.H	-0.004	0.001	-0.001	0.002	-0.010	-0.010	-0.003	-0.001	-0.004
51.H	-0.003	0.005	0.003	0.003	-0.008	-0.011	-0.003	-0.003	-0.003
52.H	-0.005	0.002	0.001	0.003	-0.013	-0.011	-0.003	0.001	-0.003
53.H	-0.007	0.007	0.006	0.003	-0.012	-0.011	-0.003	-0.000	-0.004
54.H	-0.007	0.008	0.007	0.002	-0.008	-0.008	-0.002	-0.003	-0.006
55.H	-0.011	0.009	0.008	0.002	-0.012	-0.007	-0.002	-0.000	-0.007
56.H	-0.026	0.003	0.005	-0.000	-0.022	0.007	0.002	0.009	-0.017
57.H	-0.030	0.002	0.005	-0.002	-0.022	0.013	0.004	0.008	-0.022
58.H	-0.026	0.003	0.005	-0.003	-0.017	0.012	0.004	0.004	-0.022
59.H	-0.026	0.007	0.010	-0.002	-0.016	0.010	0.003	0.003	-0.021
60.H	-0.030	0.006	0.009	-0.002	-0.021	0.011	0.003	0.007	-0.021
61.H	-0.030	0.006	0.010	-0.003	-0.018	0.014	0.004	0.005	-0.024
62.H	-0.030	-0.002	0.001	-0.001	-0.025	0.012	0.003	0.011	-0.021
63.H	-0.028	-0.002	0.001	0.000	-0.027	0.008	0.002	0.012	-0.018
64.H	-0.027	-0.005	-0.002	-0.001	-0.025	0.009	0.002	0.012	-0.018
65.H	-0.014	0.002	0.003	-0.000	-0.011	0.001	0.001	-0.000	-0.013
66.H	-0.009	0.005	0.004	-0.002	-0.003	0.002	0.001	-0.007	-0.013
67.H	-0.010	0.002	0.002	-0.002	-0.006	0.003	0.001	-0.004	-0.014
68.H	-0.013	0.004	0.004	-0.002	-0.006	0.005	0.002	-0.004	-0.016
69.C	-0.011	-0.004	-0.004	-0.001	-0.010	0.003	0.001	-0.001	-0.013
70.H	0.006	0.001	-0.003	0.002	-0.001	-0.013	-0.003	-0.008	-0.001
71.H	0.008	0.002	-0.002	0.001	0.004	-0.012	-0.003	-0.012	-0.002
72.H	0.006	0.004	0.001	0.002	0.001	-0.012	-0.003	-0.010	-0.002
73.H	0.002	0.003	0.000	-0.000	0.002	-0.006	-0.001	-0.011	-0.007
74.H	0.003	-0.003	-0.006	0.001	-0.002	-0.008	-0.002	-0.007	-0.005
75.H	0.004	-0.001	-0.004	-0.000	0.002	-0.006	-0.001	-0.011	-0.006
76.H	-0.005	-0.008	-0.009	-0.000	-0.008	-0.001	-0.000	-0.002	-0.010
77.H	-0.000	-0.007	-0.009	0.000	-0.005	-0.005	-0.001	-0.004	-0.007
78.H	-0.001	-0.009	-0.011	-0.001	-0.005	-0.001	-0.000	-0.005	-0.010

79.H	-0.007	-0.006	-0.006	-0.002	-0.004	0.005	0.002	-0.005	-0.015
80.H	-0.003	-0.004	-0.005	-0.003	0.001	0.004	0.002	-0.009	-0.014
81.H	-0.003	-0.007	-0.008	-0.003	-0.001	0.004	0.001	-0.008	-0.014
82.H	-0.004	0.002	0.001	-0.001	-0.003	-0.002	-0.000	-0.007	-0.010
83.H	-0.006	0.005	0.003	0.001	-0.006	-0.005	-0.001	-0.005	-0.008
84.H	-0.004	-0.004	-0.005	0.000	-0.007	-0.003	-0.001	-0.003	-0.009
85.H	-0.002	-0.001	-0.003	-0.002	0.000	-0.000	0.000	-0.009	-0.011
86.H	-0.022	-0.000	0.002	-0.002	-0.016	0.009	0.002	0.004	-0.019
87.O	0.000	0.000	0.000	0.000	0.000	0.000	0.000	0.000	0.000
88.H	-0.017	0.009	0.009	-0.002	-0.009	0.004	0.002	-0.003	-0.016
89.H	-0.016	-0.006	-0.004	-0.002	-0.013	0.007	0.002	0.002	-0.017
90.H	-0.014	-0.003	-0.003	-0.003	-0.009	0.008	0.002	-0.002	-0.018
91.H	-0.016	-0.002	-0.001	-0.002	-0.012	0.006	0.002	0.001	-0.016
92.C	-0.015	-0.004	-0.003	-0.002	-0.011	0.006	0.002	0.000	-0.016
93.O	0.000	0.000	0.000	0.000	0.000	0.000	0.000	0.000	0.000
94.H	-0.017	0.008	0.008	0.001	-0.015	-0.001	-0.000	0.002	-0.011
95.H	-0.015	0.011	0.011	0.000	-0.011	-0.001	0.000	-0.001	-0.012
96.H	-0.020	0.010	0.010	-0.000	-0.014	0.002	0.001	0.001	-0.014

	70			71			72		
1.O	0.000	0.000	0.000	0.000	0.000	0.000	0.000	0.000	0.000
2.C	0.000	0.000	0.000	0.000	0.000	0.000	0.000	0.000	0.000
3.C	0.000	0.000	0.000	0.000	0.000	0.000	0.000	0.000	0.000
4.C	0.000	0.000	0.000	0.000	0.000	0.000	0.000	0.000	0.000
5.O	0.000	0.000	0.000	0.000	0.000	0.000	0.000	0.000	0.000
6.N	-0.014	-0.001	0.001	0.002	-0.009	-0.002	-0.006	-0.002	-0.007
7.O	0.000	0.000	0.000	0.000	0.000	0.000	0.000	0.000	0.000
8.C	0.000	0.000	0.000	0.000	0.000	0.000	0.000	0.000	0.000
9.C	0.000	0.000	0.000	0.000	0.000	0.000	0.000	0.000	0.000

10.C	0.000	0.000	0.000	0.000	0.000	0.000	0.000	0.000	0.000
11.O	0.000	0.000	0.000	0.000	0.000	0.000	0.000	0.000	0.000
12.C	0.000	0.000	0.000	0.000	0.000	0.000	0.000	0.000	0.000
13.O	0.000	0.000	0.000	0.000	0.000	0.000	0.000	0.000	0.000
14.C	0.000	0.000	0.000	0.000	0.000	0.000	0.000	0.000	0.000
15.C	0.000	0.000	0.000	0.000	0.000	0.000	0.000	0.000	0.000
16.O	0.000	0.000	0.000	0.000	0.000	0.000	0.000	0.000	0.000
17.C	0.000	0.000	0.000	0.000	0.000	0.000	0.000	0.000	0.000
18.C	0.000	0.000	0.000	0.000	0.000	0.000	0.000	0.000	0.000
19.O	0.000	0.000	0.000	0.000	0.000	0.000	0.000	0.000	0.000
20.C	0.000	0.000	0.000	0.000	0.000	0.000	0.000	0.000	0.000
21.C	0.000	0.000	0.000	0.000	0.000	0.000	0.000	0.000	0.000
22.C	0.282	0.001	-0.001	0.000	0.280	0.001	0.000	0.000	0.281
23.C	-0.009	-0.000	-0.001	-0.005	-0.010	0.002	0.005	0.001	-0.012
24.C	-0.013	-0.002	0.002	-0.000	-0.006	-0.002	-0.005	-0.002	-0.006
25.C	-0.010	-0.000	-0.000	-0.004	-0.011	0.002	0.003	0.000	-0.012
26.C	-0.013	-0.000	0.000	-0.002	-0.013	0.001	-0.005	-0.001	-0.009
27.C	-0.012	0.001	-0.000	-0.003	-0.016	0.002	-0.005	-0.001	-0.011
28.C	-0.011	0.001	-0.001	-0.006	-0.017	0.004	-0.005	-0.000	-0.012
29.C	-0.011	-0.001	0.001	-0.001	-0.007	-0.001	0.004	0.000	-0.011
30.C	-0.012	0.003	-0.002	0.001	-0.021	0.002	0.002	0.002	-0.019
31.O	0.000	0.000	0.000	0.000	0.000	0.000	0.000	0.000	0.000
32.C	-0.012	0.000	-0.000	0.005	-0.013	-0.002	0.004	0.001	-0.015
33.C	-0.011	-0.002	0.001	0.001	-0.004	-0.002	0.004	-0.001	-0.009
34.C	-0.010	-0.001	0.000	-0.004	-0.007	0.000	0.005	0.000	-0.011
35.C	-0.010	0.001	-0.001	-0.008	-0.015	0.004	0.002	0.001	-0.013
36.C	-0.013	0.001	-0.000	0.008	-0.014	-0.003	0.003	0.001	-0.016
37.C	-0.015	-0.001	0.002	0.005	-0.008	-0.003	-0.007	-0.002	-0.007
38.C	-0.010	0.002	-0.002	-0.006	-0.020	0.005	0.002	0.002	-0.017
39.C	-0.014	0.002	-0.001	0.009	-0.017	-0.002	0.003	0.002	-0.018
40.H	-0.011	-0.000	-0.000	-0.001	-0.011	-0.000	0.005	0.001	-0.014

41.H	-0.011	-0.002	0.001	0.000	-0.004	-0.002	0.003	-0.001	-0.009
42.H	-0.012	-0.002	0.002	0.003	-0.004	-0.004	0.003	-0.001	-0.009
43.H	-0.011	-0.003	0.002	-0.000	-0.002	-0.003	0.005	-0.001	-0.008
44.H	-0.008	-0.001	-0.001	-0.010	-0.008	0.003	0.006	0.000	-0.011
45.H	-0.008	-0.001	-0.001	-0.010	-0.009	0.003	0.008	0.001	-0.012
46.H	-0.008	0.000	-0.001	-0.010	-0.012	0.004	0.006	0.001	-0.014
47.H	-0.010	0.000	-0.001	-0.004	-0.012	0.002	0.005	0.001	-0.014
48.H	-0.010	-0.001	-0.000	-0.005	-0.010	0.002	0.002	-0.000	-0.011
49.H	-0.011	-0.000	-0.000	-0.002	-0.011	0.000	0.002	0.000	-0.012
50.H	-0.010	0.000	-0.001	-0.007	-0.013	0.004	0.001	0.000	-0.012
51.H	-0.010	0.002	-0.002	-0.008	-0.017	0.005	0.001	0.001	-0.015
52.H	-0.009	0.001	-0.001	-0.010	-0.014	0.005	0.003	0.001	-0.013
53.H	-0.009	0.002	-0.003	-0.008	-0.019	0.006	0.003	0.002	-0.017
54.H	-0.010	0.003	-0.002	-0.005	-0.021	0.005	0.001	0.002	-0.017
55.H	-0.010	0.003	-0.003	-0.005	-0.021	0.005	0.003	0.002	-0.018
56.H	-0.012	0.000	-0.000	0.004	-0.013	-0.001	0.005	0.002	-0.015
57.H	-0.013	0.000	0.000	0.009	-0.012	-0.004	0.004	0.001	-0.015
58.H	-0.014	0.001	0.000	0.010	-0.014	-0.004	0.002	0.001	-0.015
59.H	-0.014	0.002	-0.001	0.008	-0.019	-0.002	0.002	0.002	-0.019
60.H	-0.013	0.002	-0.001	0.008	-0.017	-0.002	0.004	0.002	-0.018
61.H	-0.014	0.002	-0.001	0.012	-0.017	-0.003	0.002	0.002	-0.018
62.H	-0.013	-0.001	0.001	0.007	-0.007	-0.004	0.005	0.001	-0.012
63.H	-0.011	-0.001	0.001	0.003	-0.007	-0.003	0.007	0.001	-0.012
64.H	-0.012	-0.002	0.001	0.004	-0.004	-0.004	0.005	0.000	-0.010
65.H	-0.012	0.001	-0.000	0.002	-0.015	-0.000	0.000	0.001	-0.014
66.H	-0.014	0.002	-0.001	0.004	-0.019	0.000	-0.004	0.001	-0.015
67.H	-0.014	0.001	0.000	0.004	-0.015	-0.001	-0.003	0.000	-0.013
68.H	-0.014	0.002	-0.000	0.006	-0.018	-0.001	-0.003	0.001	-0.015
69.C	-0.013	-0.001	0.001	0.003	-0.008	-0.002	-0.002	-0.001	-0.009
70.H	-0.011	0.001	-0.001	-0.008	-0.015	0.004	-0.004	-0.001	-0.011
71.H	-0.012	0.001	-0.001	-0.006	-0.017	0.004	-0.006	-0.001	-0.011

72.H	-0.011	0.002	-0.002	-0.007	-0.019	0.005	-0.004	-0.000	-0.013
73.H	-0.013	0.001	-0.001	-0.002	-0.018	0.002	-0.006	-0.000	-0.012
74.H	-0.012	-0.001	0.000	-0.004	-0.011	0.001	-0.004	-0.001	-0.008
75.H	-0.013	-0.000	0.000	-0.002	-0.013	0.001	-0.007	-0.001	-0.009
76.H	-0.013	-0.002	0.002	0.000	-0.005	-0.002	-0.003	-0.002	-0.006
77.H	-0.012	-0.002	0.002	-0.003	-0.006	-0.001	-0.004	-0.002	-0.006
78.H	-0.013	-0.003	0.002	0.000	-0.004	-0.002	-0.005	-0.003	-0.004
79.H	-0.015	-0.002	0.002	0.006	-0.008	-0.004	-0.006	-0.002	-0.007
80.H	-0.015	-0.001	0.002	0.006	-0.011	-0.003	-0.008	-0.002	-0.008
81.H	-0.015	-0.002	0.002	0.005	-0.007	-0.004	-0.008	-0.003	-0.006
82.H	-0.013	0.001	-0.000	0.001	-0.016	0.001	-0.004	-0.000	-0.013
83.H	-0.012	0.002	-0.001	-0.002	-0.018	0.003	-0.001	0.001	-0.015
84.H	-0.012	-0.001	0.001	-0.002	-0.009	-0.000	-0.002	-0.001	-0.009
85.H	-0.014	0.000	0.001	0.002	-0.013	-0.001	-0.007	-0.001	-0.010
86.H	-0.013	-0.000	0.001	0.007	-0.011	-0.003	0.001	0.000	-0.013
87.O	0.000	0.000	0.000	0.000	0.000	0.000	0.000	0.000	0.000
88.H	-0.013	0.003	-0.002	0.005	-0.022	0.001	-0.000	0.002	-0.018
89.H	-0.014	-0.002	0.002	0.006	-0.006	-0.004	-0.001	-0.001	-0.008
90.H	-0.015	-0.001	0.002	0.008	-0.009	-0.004	-0.003	-0.001	-0.010
91.H	-0.014	-0.001	0.001	0.005	-0.010	-0.003	-0.001	-0.000	-0.011
92.C	-0.014	-0.001	0.001	0.006	-0.008	-0.003	-0.002	-0.001	-0.010
93.O	0.000	0.000	0.000	0.000	0.000	0.000	0.000	0.000	0.000
94.H	-0.011	0.002	-0.002	-0.001	-0.019	0.002	0.003	0.002	-0.018
95.H	-0.012	0.003	-0.003	0.000	-0.023	0.003	0.002	0.003	-0.020
96.H	-0.012	0.003	-0.002	0.002	-0.021	0.002	0.003	0.003	-0.020

	73			74			75		
1.O	0.000	0.000	0.000	0.000	0.000	0.000	0.000	0.000	0.000
2.C	0.000	0.000	0.000	0.000	0.000	0.000	0.000	0.000	0.000

3.C	0.000	0.000	0.000	0.000	0.000	0.000	0.000	0.000	0.000
4.C	0.000	0.000	0.000	0.000	0.000	0.000	0.000	0.000	0.000
5.O	0.000	0.000	0.000	0.000	0.000	0.000	0.000	0.000	0.000
6.N	-0.001	0.005	-0.009	-0.001	-0.004	-0.000	-0.002	0.004	-0.011
7.O	0.000	0.000	0.000	0.000	0.000	0.000	0.000	0.000	0.000
8.C	0.000	0.000	0.000	0.000	0.000	0.000	0.000	0.000	0.000
9.C	0.000	0.000	0.000	0.000	0.000	0.000	0.000	0.000	0.000
10.C	0.000	0.000	0.000	0.000	0.000	0.000	0.000	0.000	0.000
11.O	0.000	0.000	0.000	0.000	0.000	0.000	0.000	0.000	0.000
12.C	0.000	0.000	0.000	0.000	0.000	0.000	0.000	0.000	0.000
13.O	0.000	0.000	0.000	0.000	0.000	0.000	0.000	0.000	0.000
14.C	0.000	0.000	0.000	0.000	0.000	0.000	0.000	0.000	0.000
15.C	0.000	0.000	0.000	0.000	0.000	0.000	0.000	0.000	0.000
16.O	0.000	0.000	0.000	0.000	0.000	0.000	0.000	0.000	0.000
17.C	0.000	0.000	0.000	0.000	0.000	0.000	0.000	0.000	0.000
18.C	0.000	0.000	0.000	0.000	0.000	0.000	0.000	0.000	0.000
19.O	0.000	0.000	0.000	0.000	0.000	0.000	0.000	0.000	0.000
20.C	0.000	0.000	0.000	0.000	0.000	0.000	0.000	0.000	0.000
21.C	0.000	0.000	0.000	0.000	0.000	0.000	0.000	0.000	0.000
22.C	0.000	0.000	0.000	0.000	0.000	0.000	0.000	0.000	0.000
23.C	0.276	0.001	0.003	0.000	0.279	-0.004	0.000	0.000	0.281
24.C	-0.005	0.009	-0.011	-0.000	-0.006	-0.001	-0.001	0.002	-0.011
25.C	-0.021	0.000	0.002	0.001	-0.016	-0.003	0.002	-0.002	-0.015
26.C	-0.006	-0.000	-0.002	-0.000	-0.003	-0.005	-0.001	0.005	-0.015
27.C	-0.008	-0.004	0.002	0.000	-0.003	-0.006	-0.001	0.006	-0.016
28.C	-0.011	-0.005	0.005	0.001	-0.002	-0.009	0.000	0.006	-0.018
29.C	-0.021	0.005	-0.003	0.001	-0.020	0.002	0.002	-0.005	-0.011
30.C	-0.016	-0.012	0.013	0.000	-0.015	0.001	0.001	0.001	-0.014
31.O	0.000	0.000	0.000	0.000	0.000	0.000	0.000	0.000	0.000
32.C	-0.015	-0.002	0.002	-0.000	-0.020	0.007	0.000	-0.004	-0.009
33.C	-0.018	0.009	-0.007	0.000	-0.020	0.003	0.001	-0.006	-0.010

34.C	-0.025	0.005	-0.001	0.001	-0.021	-0.002	0.003	-0.005	-0.013
35.C	-0.022	-0.004	0.007	0.001	-0.012	-0.008	0.003	0.001	-0.018
36.C	-0.010	-0.003	0.002	-0.001	-0.020	0.010	-0.001	-0.003	-0.008
37.C	0.003	0.005	-0.011	-0.002	-0.003	0.003	-0.004	0.004	-0.009
38.C	-0.022	-0.010	0.013	0.001	-0.013	-0.006	0.002	0.001	-0.018
39.C	-0.009	-0.007	0.006	-0.001	-0.019	0.010	-0.001	-0.002	-0.008
40.H	-0.022	0.000	0.003	0.001	-0.021	0.002	0.002	-0.005	-0.012
41.H	-0.017	0.009	-0.008	0.000	-0.018	0.002	0.001	-0.005	-0.010
42.H	-0.015	0.009	-0.008	-0.000	-0.020	0.006	0.000	-0.005	-0.008
43.H	-0.021	0.011	-0.009	0.001	-0.022	0.003	0.002	-0.007	-0.010
44.H	-0.032	0.004	0.003	0.002	-0.020	-0.008	0.005	-0.005	-0.017
45.H	-0.034	0.003	0.005	0.003	-0.023	-0.007	0.005	-0.006	-0.017
46.H	-0.031	-0.001	0.008	0.002	-0.019	-0.008	0.005	-0.003	-0.018
47.H	-0.025	-0.001	0.005	0.001	-0.019	-0.003	0.003	-0.004	-0.015
48.H	-0.022	0.003	0.000	0.001	-0.015	-0.004	0.002	-0.002	-0.015
49.H	-0.017	0.001	0.001	0.001	-0.015	-0.001	0.001	-0.002	-0.013
50.H	-0.021	-0.002	0.005	0.001	-0.011	-0.008	0.002	0.001	-0.017
51.H	-0.021	-0.007	0.010	0.001	-0.011	-0.009	0.002	0.002	-0.019
52.H	-0.027	-0.003	0.008	0.002	-0.014	-0.009	0.004	-0.001	-0.019
53.H	-0.026	-0.009	0.014	0.002	-0.014	-0.009	0.003	0.001	-0.020
54.H	-0.019	-0.012	0.014	0.001	-0.011	-0.006	0.002	0.003	-0.018
55.H	-0.023	-0.011	0.015	0.001	-0.014	-0.006	0.003	0.001	-0.018
56.H	-0.018	-0.002	0.004	0.000	-0.022	0.006	0.001	-0.005	-0.010
57.H	-0.011	-0.001	0.000	-0.001	-0.022	0.011	-0.001	-0.005	-0.007
58.H	-0.006	-0.003	0.001	-0.001	-0.018	0.011	-0.002	-0.002	-0.007
59.H	-0.009	-0.009	0.008	-0.001	-0.017	0.009	-0.001	-0.001	-0.009
60.H	-0.013	-0.007	0.008	-0.001	-0.021	0.009	-0.000	-0.004	-0.009
61.H	-0.006	-0.008	0.006	-0.002	-0.019	0.013	-0.002	-0.002	-0.007
62.H	-0.015	0.005	-0.005	-0.000	-0.024	0.010	0.000	-0.007	-0.006
63.H	-0.021	0.005	-0.002	0.000	-0.026	0.007	0.002	-0.008	-0.009
64.H	-0.018	0.008	-0.007	0.000	-0.024	0.007	0.001	-0.008	-0.007

65.H	-0.011	-0.003	0.003	-0.000	-0.013	0.002	-0.000	0.000	-0.012
66.H	-0.003	-0.009	0.006	-0.001	-0.006	0.002	-0.002	0.005	-0.013
67.H	-0.003	-0.004	0.001	-0.001	-0.008	0.003	-0.002	0.003	-0.011
68.H	-0.002	-0.007	0.003	-0.001	-0.009	0.005	-0.003	0.003	-0.011
69.C	-0.007	0.005	-0.007	-0.001	-0.011	0.002	-0.001	-0.000	-0.010
70.H	-0.014	-0.003	0.003	0.001	-0.004	-0.011	0.001	0.005	-0.019
71.H	-0.008	-0.006	0.004	0.000	0.000	-0.010	-0.000	0.008	-0.018
72.H	-0.012	-0.008	0.008	0.001	-0.002	-0.010	0.000	0.007	-0.019
73.H	-0.004	-0.006	0.003	-0.000	-0.001	-0.005	-0.002	0.007	-0.016
74.H	-0.009	0.002	-0.003	0.000	-0.004	-0.006	-0.000	0.004	-0.015
75.H	-0.003	-0.001	-0.003	-0.000	-0.001	-0.005	-0.002	0.006	-0.015
76.H	-0.007	0.010	-0.012	-0.000	-0.008	-0.000	-0.001	0.000	-0.011
77.H	-0.009	0.008	-0.010	0.000	-0.007	-0.004	-0.001	0.002	-0.013
78.H	-0.004	0.011	-0.014	-0.001	-0.006	-0.001	-0.002	0.002	-0.011
79.H	0.002	0.006	-0.012	-0.002	-0.005	0.004	-0.003	0.003	-0.008
80.H	0.006	0.003	-0.009	-0.002	-0.002	0.003	-0.004	0.005	-0.009
81.H	0.005	0.008	-0.014	-0.002	-0.003	0.003	-0.004	0.004	-0.008
82.H	-0.005	-0.005	0.002	-0.001	-0.005	-0.001	-0.002	0.005	-0.014
83.H	-0.012	-0.008	0.008	0.000	-0.008	-0.004	0.000	0.003	-0.016
84.H	-0.011	0.004	-0.005	0.000	-0.009	-0.003	-0.000	0.001	-0.013
85.H	0.000	-0.001	-0.004	-0.001	-0.002	-0.000	-0.003	0.005	-0.012
86.H	-0.008	0.001	-0.002	-0.001	-0.017	0.008	-0.001	-0.002	-0.008
87.O	0.000	0.000	0.000	0.000	0.000	0.000	0.000	0.000	0.000
88.H	-0.007	-0.012	0.011	-0.001	-0.012	0.004	-0.001	0.002	-0.012
89.H	-0.006	0.008	-0.010	-0.001	-0.014	0.006	-0.002	-0.002	-0.007
90.H	-0.000	0.004	-0.008	-0.002	-0.010	0.007	-0.003	0.001	-0.007
91.H	-0.006	0.003	-0.005	-0.001	-0.013	0.005	-0.002	-0.001	-0.009
92.C	-0.005	0.005	-0.008	-0.001	-0.012	0.006	-0.002	-0.001	-0.008
93.O	0.000	0.000	0.000	0.000	0.000	0.000	0.000	0.000	0.000
94.H	-0.019	-0.010	0.012	0.001	-0.017	-0.001	0.002	-0.001	-0.015
95.H	-0.015	-0.014	0.016	0.000	-0.013	-0.000	0.001	0.002	-0.016

96.H	-0.015	-0.012	0.014	0.000	-0.016	0.002	0.001	0.000	-0.014

	76			77			78		
1.O	0.000	0.000	0.000	0.000	0.000	0.000	0.000	0.000	0.000
2.C	0.000	0.000	0.000	0.000	0.000	0.000	0.000	0.000	0.000
3.C	0.000	0.000	0.000	0.000	0.000	0.000	0.000	0.000	0.000
4.C	0.000	0.000	0.000	0.000	0.000	0.000	0.000	0.000	0.000
5.O	0.000	0.000	0.000	0.000	0.000	0.000	0.000	0.000	0.000
6.N	-0.024	0.001	0.008	-0.001	-0.033	0.004	0.015	0.006	-0.025
7.O	0.000	0.000	0.000	0.000	0.000	0.000	0.000	0.000	0.000
8.C	0.000	0.000	0.000	0.000	0.000	0.000	0.000	0.000	0.000
9.C	0.000	0.000	0.000	0.000	0.000	0.000	0.000	0.000	0.000
10.C	0.000	0.000	0.000	0.000	0.000	0.000	0.000	0.000	0.000
11.O	0.000	0.000	0.000	0.000	0.000	0.000	0.000	0.000	0.000
12.C	0.000	0.000	0.000	0.000	0.000	0.000	0.000	0.000	0.000
13.O	0.000	0.000	0.000	0.000	0.000	0.000	0.000	0.000	0.000
14.C	0.000	0.000	0.000	0.000	0.000	0.000	0.000	0.000	0.000
15.C	0.000	0.000	0.000	0.000	0.000	0.000	0.000	0.000	0.000
16.O	0.000	0.000	0.000	0.000	0.000	0.000	0.000	0.000	0.000
17.C	0.000	0.000	0.000	0.000	0.000	0.000	0.000	0.000	0.000
18.C	0.000	0.000	0.000	0.000	0.000	0.000	0.000	0.000	0.000
19.O	0.000	0.000	0.000	0.000	0.000	0.000	0.000	0.000	0.000
20.C	0.000	0.000	0.000	0.000	0.000	0.000	0.000	0.000	0.000
21.C	0.000	0.000	0.000	0.000	0.000	0.000	0.000	0.000	0.000
22.C	0.000	0.000	0.000	0.000	0.000	0.000	0.000	0.000	0.000
23.C	0.000	0.000	0.000	0.000	0.000	0.000	0.000	0.000	0.000
24.C	0.278	0.002	0.011	0.000	0.271	0.006	0.000	0.000	0.274
25.C	-0.010	-0.000	-0.001	0.008	-0.010	0.002	-0.007	-0.001	-0.013
26.C	-0.025	0.001	0.004	0.006	-0.027	0.005	0.014	0.004	-0.020

27.C	-0.025	0.000	-0.000	0.007	-0.020	0.004	0.012	0.002	-0.015
28.C	-0.026	0.000	-0.001	0.013	-0.019	0.006	0.012	0.002	-0.014
29.C	-0.004	-0.000	0.002	0.001	-0.011	-0.000	-0.011	-0.000	-0.016
30.C	-0.008	-0.002	-0.012	-0.002	0.009	-0.006	-0.006	-0.006	0.004
31.O	0.000	0.000	0.000	0.000	0.000	0.000	0.000	0.000	0.000
32.C	-0.003	-0.001	-0.005	-0.009	0.000	-0.007	-0.010	-0.004	-0.005
33.C	-0.005	0.000	0.005	-0.001	-0.017	0.000	-0.009	0.001	-0.020
34.C	-0.004	-0.000	0.002	0.007	-0.011	0.002	-0.014	-0.000	-0.016
35.C	-0.014	-0.000	-0.004	0.014	-0.007	0.004	-0.004	-0.001	-0.010
36.C	-0.003	-0.001	-0.005	-0.015	0.001	-0.009	-0.007	-0.004	-0.003
37.C	-0.025	0.001	0.009	-0.007	-0.035	0.002	0.018	0.006	-0.026
38.C	-0.011	-0.001	-0.010	0.011	0.004	-0.000	-0.006	-0.005	-0.001
39.C	-0.002	-0.001	-0.009	-0.016	0.007	-0.011	-0.007	-0.006	0.002
40.H	-0.003	-0.001	-0.003	0.001	-0.003	-0.002	-0.013	-0.003	-0.009
41.H	-0.008	0.000	0.006	-0.000	-0.020	0.001	-0.006	0.002	-0.022
42.H	-0.005	0.000	0.006	-0.006	-0.018	-0.001	-0.007	0.002	-0.020
43.H	-0.003	0.000	0.007	0.001	-0.019	0.002	-0.012	0.002	-0.023
44.H	-0.006	-0.000	0.000	0.018	-0.009	0.006	-0.015	-0.001	-0.016
45.H	-0.002	-0.001	-0.002	0.017	-0.004	0.004	-0.020	-0.002	-0.012
46.H	-0.006	-0.001	-0.004	0.017	-0.002	0.004	-0.015	-0.003	-0.009
47.H	-0.005	-0.001	-0.004	0.008	-0.003	0.000	-0.013	-0.003	-0.009
48.H	-0.011	-0.000	0.001	0.010	-0.014	0.004	-0.006	0.000	-0.016
49.H	-0.010	-0.000	-0.000	0.003	-0.011	0.001	-0.005	-0.000	-0.013
50.H	-0.015	-0.000	-0.002	0.014	-0.012	0.005	-0.002	-0.000	-0.013
51.H	-0.015	-0.001	-0.006	0.015	-0.005	0.003	-0.002	-0.002	-0.007
52.H	-0.012	-0.000	-0.004	0.017	-0.006	0.005	-0.008	-0.002	-0.010
53.H	-0.011	-0.001	-0.010	0.015	0.004	0.001	-0.008	-0.005	-0.001
54.H	-0.014	-0.001	-0.011	0.009	0.004	-0.001	-0.003	-0.005	0.001
55.H	-0.009	-0.001	-0.012	0.010	0.008	-0.002	-0.008	-0.006	0.002
56.H	-0.000	-0.001	-0.005	-0.007	0.003	-0.006	-0.014	-0.004	-0.004
57.H	-0.001	-0.001	-0.004	-0.017	0.000	-0.009	-0.009	-0.004	-0.005

58.H	-0.005	-0.001	-0.004	-0.017	-0.002	-0.009	-0.004	-0.003	-0.005
59.H	-0.004	-0.001	-0.010	-0.015	0.008	-0.011	-0.005	-0.006	0.004
60.H	-0.000	-0.002	-0.010	-0.014	0.010	-0.011	-0.011	-0.006	0.003
61.H	-0.002	-0.001	-0.009	-0.021	0.008	-0.013	-0.006	-0.006	0.003
62.H	0.001	-0.001	0.001	-0.013	-0.007	-0.006	-0.013	-0.002	-0.012
63.H	0.002	-0.001	-0.000	-0.006	-0.004	-0.004	-0.017	-0.002	-0.012
64.H	0.000	-0.000	0.004	-0.008	-0.011	-0.003	-0.014	-0.000	-0.017
65.H	-0.012	-0.000	-0.003	-0.003	-0.007	-0.002	-0.000	-0.001	-0.008
66.H	-0.019	-0.000	-0.006	-0.006	-0.008	-0.004	0.010	-0.001	-0.005
67.H	-0.017	0.000	-0.002	-0.007	-0.014	-0.003	0.008	0.000	-0.011
68.H	-0.016	-0.000	-0.005	-0.011	-0.007	-0.005	0.008	-0.001	-0.005
69.C	-0.015	0.001	0.005	-0.004	-0.024	0.001	0.005	0.003	-0.021
70.H	-0.024	0.001	0.000	0.016	-0.021	0.008	0.009	0.002	-0.017
71.H	-0.028	0.001	-0.001	0.013	-0.021	0.006	0.015	0.002	-0.015
72.H	-0.025	0.000	-0.004	0.014	-0.015	0.005	0.010	0.001	-0.011
73.H	-0.026	0.000	-0.002	0.004	-0.018	0.003	0.015	0.002	-0.012
74.H	-0.024	0.001	0.005	0.009	-0.028	0.007	0.011	0.004	-0.022
75.H	-0.028	0.001	0.004	0.005	-0.029	0.005	0.017	0.005	-0.021
76.H	-0.020	0.001	0.011	0.001	-0.035	0.005	0.009	0.006	-0.029
77.H	-0.022	0.001	0.010	0.006	-0.035	0.007	0.010	0.006	-0.029
78.H	-0.024	0.002	0.013	0.001	-0.041	0.007	0.013	0.008	-0.033
79.H	-0.022	0.001	0.009	-0.009	-0.033	0.001	0.015	0.006	-0.025
80.H	-0.026	0.001	0.007	-0.010	-0.033	0.001	0.021	0.006	-0.023
81.H	-0.026	0.002	0.011	-0.008	-0.039	0.003	0.020	0.008	-0.029
82.H	-0.021	0.000	-0.002	-0.000	-0.016	0.000	0.011	0.001	-0.011
83.H	-0.017	-0.000	-0.006	0.005	-0.006	-0.000	0.004	-0.002	-0.006
84.H	-0.019	0.001	0.005	0.004	-0.025	0.004	0.006	0.004	-0.021
85.H	-0.025	0.001	0.003	-0.003	-0.026	0.002	0.017	0.004	-0.018
86.H	-0.007	-0.000	-0.001	-0.012	-0.009	-0.005	-0.003	-0.001	-0.011
87.O	0.000	0.000	0.000	0.000	0.000	0.000	0.000	0.000	0.000
88.H	-0.011	-0.001	-0.011	-0.009	0.005	-0.008	0.001	-0.005	0.003

89.H	-0.012	0.001	0.007	-0.010	-0.024	-0.001	0.003	0.003	-0.022
90.H	-0.016	0.001	0.005	-0.013	-0.023	-0.002	0.009	0.003	-0.019
91.H	-0.013	0.000	0.003	-0.009	-0.018	-0.002	0.003	0.001	-0.016
92.C	-0.014	0.001	0.005	-0.009	-0.022	-0.001	0.005	0.003	-0.019
93.O	0.000	0.000	0.000	0.000	0.000	0.000	0.000	0.000	0.000
94.H	-0.006	-0.001	-0.011	0.002	0.008	-0.004	-0.009	-0.006	0.002
95.H	-0.009	-0.002	-0.014	-0.000	0.011	-0.006	-0.004	-0.007	0.007
96.H	-0.006	-0.002	-0.013	-0.004	0.012	-0.008	-0.007	-0.007	0.006

	79			80			81		
1.O	0.000	0.000	0.000	0.000	0.000	0.000	0.000	0.000	0.000
2.C	0.000	0.000	0.000	0.000	0.000	0.000	0.000	0.000	0.000
3.C	0.000	0.000	0.000	0.000	0.000	0.000	0.000	0.000	0.000
4.C	0.000	0.000	0.000	0.000	0.000	0.000	0.000	0.000	0.000
5.O	0.000	0.000	0.000	0.000	0.000	0.000	0.000	0.000	0.000
6.N	-0.007	0.005	-0.006	-0.000	-0.009	-0.000	-0.001	0.004	-0.013
7.O	0.000	0.000	0.000	0.000	0.000	0.000	0.000	0.000	0.000
8.C	0.000	0.000	0.000	0.000	0.000	0.000	0.000	0.000	0.000
9.C	0.000	0.000	0.000	0.000	0.000	0.000	0.000	0.000	0.000
10.C	0.000	0.000	0.000	0.000	0.000	0.000	0.000	0.000	0.000
11.O	0.000	0.000	0.000	0.000	0.000	0.000	0.000	0.000	0.000
12.C	0.000	0.000	0.000	0.000	0.000	0.000	0.000	0.000	0.000
13.O	0.000	0.000	0.000	0.000	0.000	0.000	0.000	0.000	0.000
14.C	0.000	0.000	0.000	0.000	0.000	0.000	0.000	0.000	0.000
15.C	0.000	0.000	0.000	0.000	0.000	0.000	0.000	0.000	0.000
16.O	0.000	0.000	0.000	0.000	0.000	0.000	0.000	0.000	0.000
17.C	0.000	0.000	0.000	0.000	0.000	0.000	0.000	0.000	0.000
18.C	0.000	0.000	0.000	0.000	0.000	0.000	0.000	0.000	0.000
19.O	0.000	0.000	0.000	0.000	0.000	0.000	0.000	0.000	0.000

20.C	0.000	0.000	0.000	0.000	0.000	0.000	0.000	0.000	0.000
21.C	0.000	0.000	0.000	0.000	0.000	0.000	0.000	0.000	0.000
22.C	0.000	0.000	0.000	0.000	0.000	0.000	0.000	0.000	0.000
23.C	0.000	0.000	0.000	0.000	0.000	0.000	0.000	0.000	0.000
24.C	0.000	0.000	0.000	0.000	0.000	0.000	0.000	0.000	0.000
25.C	0.279	0.001	0.001	0.000	0.281	-0.002	0.000	0.000	0.281
26.C	-0.011	0.001	-0.001	-0.000	-0.009	-0.003	-0.000	0.005	-0.016
27.C	-0.013	-0.002	0.001	0.000	-0.009	-0.003	0.000	0.006	-0.017
28.C	-0.016	-0.003	0.003	0.000	-0.008	-0.005	0.001	0.006	-0.019
29.C	-0.019	0.005	-0.002	0.000	-0.018	0.001	0.001	-0.005	-0.013
30.C	-0.015	-0.010	0.008	0.000	-0.015	0.000	0.000	0.000	-0.014
31.O	0.000	0.000	0.000	0.000	0.000	0.000	0.000	0.000	0.000
32.C	-0.013	-0.002	0.001	-0.000	-0.017	0.003	-0.000	-0.004	-0.010
33.C	-0.017	0.008	-0.005	0.000	-0.018	0.002	0.001	-0.005	-0.011
34.C	-0.023	0.005	-0.001	0.001	-0.018	-0.001	0.002	-0.005	-0.015
35.C	-0.023	-0.003	0.005	0.001	-0.013	-0.004	0.002	0.000	-0.019
36.C	-0.009	-0.003	0.001	-0.001	-0.017	0.005	-0.001	-0.004	-0.008
37.C	-0.003	0.006	-0.007	-0.001	-0.009	0.001	-0.002	0.004	-0.011
38.C	-0.022	-0.009	0.009	0.001	-0.014	-0.003	0.002	0.001	-0.018
39.C	-0.008	-0.006	0.003	-0.001	-0.017	0.005	-0.001	-0.003	-0.008
40.H	-0.019	0.000	0.001	0.000	-0.018	0.001	0.001	-0.005	-0.013
41.H	-0.016	0.008	-0.005	0.000	-0.017	0.001	0.000	-0.004	-0.012
42.H	-0.014	0.008	-0.006	-0.000	-0.018	0.003	-0.000	-0.005	-0.010
43.H	-0.019	0.010	-0.006	0.000	-0.019	0.001	0.001	-0.007	-0.012
44.H	-0.029	0.003	0.002	0.002	-0.017	-0.004	0.003	-0.004	-0.019
45.H	-0.030	0.002	0.003	0.002	-0.019	-0.004	0.004	-0.006	-0.018
46.H	-0.028	-0.001	0.005	0.001	-0.017	-0.004	0.003	-0.003	-0.019
47.H	-0.023	-0.001	0.003	0.001	-0.017	-0.001	0.002	-0.004	-0.016
48.H	-0.021	0.003	0.000	0.001	-0.015	-0.002	0.002	-0.002	-0.016
49.H	-0.017	0.001	0.000	0.000	-0.015	-0.001	0.001	-0.002	-0.014
50.H	-0.022	-0.001	0.003	0.001	-0.013	-0.004	0.002	0.001	-0.019

51.H	-0.022	-0.006	0.006	0.001	-0.013	-0.005	0.002	0.002	-0.020
52.H	-0.026	-0.002	0.005	0.001	-0.014	-0.005	0.003	-0.001	-0.020
53.H	-0.025	-0.008	0.009	0.001	-0.014	-0.005	0.003	0.000	-0.020
54.H	-0.020	-0.010	0.009	0.001	-0.013	-0.003	0.001	0.002	-0.018
55.H	-0.022	-0.010	0.010	0.001	-0.014	-0.003	0.002	0.000	-0.018
56.H	-0.016	-0.002	0.002	-0.000	-0.019	0.003	0.000	-0.005	-0.011
57.H	-0.009	-0.001	-0.001	-0.001	-0.018	0.006	-0.001	-0.005	-0.007
58.H	-0.006	-0.002	-0.000	-0.001	-0.016	0.005	-0.002	-0.003	-0.008
59.H	-0.008	-0.008	0.005	-0.001	-0.016	0.004	-0.001	-0.002	-0.009
60.H	-0.011	-0.007	0.004	-0.001	-0.018	0.005	-0.001	-0.004	-0.009
61.H	-0.005	-0.007	0.003	-0.001	-0.017	0.006	-0.002	-0.003	-0.007
62.H	-0.012	0.005	-0.004	-0.000	-0.019	0.005	-0.001	-0.007	-0.008
63.H	-0.017	0.004	-0.002	0.000	-0.020	0.003	0.001	-0.008	-0.010
64.H	-0.015	0.007	-0.005	-0.000	-0.020	0.004	0.000	-0.007	-0.009
65.H	-0.013	-0.003	0.002	-0.000	-0.014	0.001	-0.000	0.000	-0.013
66.H	-0.007	-0.007	0.003	-0.001	-0.010	0.001	-0.001	0.005	-0.013
67.H	-0.007	-0.003	-0.000	-0.001	-0.011	0.001	-0.001	0.003	-0.012
68.H	-0.005	-0.006	0.002	-0.001	-0.012	0.002	-0.002	0.003	-0.011
69.C	-0.010	0.005	-0.005	-0.000	-0.013	0.001	-0.001	0.000	-0.012
70.H	-0.019	-0.001	0.002	0.001	-0.009	-0.006	0.001	0.005	-0.020
71.H	-0.014	-0.004	0.003	0.000	-0.007	-0.005	0.001	0.008	-0.020
72.H	-0.017	-0.006	0.005	0.001	-0.008	-0.005	0.001	0.006	-0.020
73.H	-0.010	-0.004	0.002	-0.000	-0.008	-0.003	-0.001	0.007	-0.017
74.H	-0.014	0.003	-0.002	0.000	-0.010	-0.003	0.000	0.004	-0.017
75.H	-0.010	0.001	-0.002	-0.000	-0.008	-0.003	-0.001	0.006	-0.016
76.H	-0.011	0.009	-0.008	-0.000	-0.012	-0.000	-0.000	0.001	-0.013
77.H	-0.013	0.008	-0.006	0.000	-0.011	-0.002	0.000	0.002	-0.015
78.H	-0.009	0.011	-0.009	-0.000	-0.011	-0.001	-0.001	0.002	-0.013
79.H	-0.003	0.006	-0.008	-0.001	-0.010	0.002	-0.002	0.003	-0.011
80.H	-0.001	0.004	-0.006	-0.001	-0.008	0.002	-0.003	0.006	-0.011
81.H	-0.002	0.008	-0.009	-0.001	-0.009	0.002	-0.002	0.004	-0.011

82.H	-0.010	-0.003	0.001	-0.000	-0.010	-0.001	-0.001	0.004	-0.015
83.H	-0.015	-0.006	0.005	0.000	-0.011	-0.002	0.000	0.003	-0.017
84.H	-0.014	0.004	-0.003	0.000	-0.012	-0.001	0.000	0.001	-0.015
85.H	-0.006	0.000	-0.002	-0.001	-0.009	-0.000	-0.002	0.006	-0.014
86.H	-0.009	0.001	-0.002	-0.001	-0.016	0.004	-0.001	-0.002	-0.009
87.O	0.000	0.000	0.000	0.000	0.000	0.000	0.000	0.000	0.000
88.H	-0.009	-0.010	0.006	-0.001	-0.013	0.002	-0.001	0.002	-0.012
89.H	-0.008	0.007	-0.007	-0.001	-0.014	0.003	-0.001	-0.002	-0.009
90.H	-0.004	0.004	-0.006	-0.001	-0.013	0.003	-0.002	0.001	-0.009
91.H	-0.008	0.003	-0.004	-0.001	-0.014	0.003	-0.001	-0.001	-0.010
92.C	-0.007	0.005	-0.005	-0.001	-0.013	0.003	-0.001	-0.000	-0.010
93.O	0.000	0.000	0.000	0.000	0.000	0.000	0.000	0.000	0.000
94.H	-0.018	-0.009	0.008	0.000	-0.015	-0.000	0.001	-0.001	-0.015
95.H	-0.015	-0.013	0.010	0.000	-0.014	-0.000	0.000	0.001	-0.015
96.H	-0.015	-0.011	0.008	-0.000	-0.015	0.001	0.000	-0.001	-0.013

	82			83			84		
1.O	0.000	0.000	0.000	0.000	0.000	0.000	0.000	0.000	0.000
2.C	0.000	0.000	0.000	0.000	0.000	0.000	0.000	0.000	0.000
3.C	0.000	0.000	0.000	0.000	0.000	0.000	0.000	0.000	0.000
4.C	0.000	0.000	0.000	0.000	0.000	0.000	0.000	0.000	0.000
5.O	0.000	0.000	0.000	0.000	0.000	0.000	0.000	0.000	0.000
6.N	-0.031	0.005	0.012	0.001	-0.033	0.003	0.006	0.009	-0.021
7.O	0.000	0.000	0.000	0.000	0.000	0.000	0.000	0.000	0.000
8.C	0.000	0.000	0.000	0.000	0.000	0.000	0.000	0.000	0.000
9.C	0.000	0.000	0.000	0.000	0.000	0.000	0.000	0.000	0.000
10.C	0.000	0.000	0.000	0.000	0.000	0.000	0.000	0.000	0.000
11.O	0.000	0.000	0.000	0.000	0.000	0.000	0.000	0.000	0.000
12.C	0.000	0.000	0.000	0.000	0.000	0.000	0.000	0.000	0.000

13.O	0.000	0.000	0.000	0.000	0.000	0.000	0.000	0.000	0.000
14.C	0.000	0.000	0.000	0.000	0.000	0.000	0.000	0.000	0.000
15.C	0.000	0.000	0.000	0.000	0.000	0.000	0.000	0.000	0.000
16.O	0.000	0.000	0.000	0.000	0.000	0.000	0.000	0.000	0.000
17.C	0.000	0.000	0.000	0.000	0.000	0.000	0.000	0.000	0.000
18.C	0.000	0.000	0.000	0.000	0.000	0.000	0.000	0.000	0.000
19.O	0.000	0.000	0.000	0.000	0.000	0.000	0.000	0.000	0.000
20.C	0.000	0.000	0.000	0.000	0.000	0.000	0.000	0.000	0.000
21.C	0.000	0.000	0.000	0.000	0.000	0.000	0.000	0.000	0.000
22.C	0.000	0.000	0.000	0.000	0.000	0.000	0.000	0.000	0.000
23.C	0.000	0.000	0.000	0.000	0.000	0.000	0.000	0.000	0.000
24.C	0.000	0.000	0.000	0.000	0.000	0.000	0.000	0.000	0.000
25.C	0.000	0.000	0.000	0.000	0.000	0.000	0.000	0.000	0.000
26.C	0.272	0.002	0.006	0.000	0.273	0.009	0.000	0.000	0.278
27.C	-0.033	-0.000	0.001	0.002	-0.030	0.010	0.006	0.009	-0.021
28.C	-0.037	-0.001	0.001	0.003	-0.031	0.015	0.007	0.010	-0.023
29.C	-0.003	0.002	0.003	-0.000	-0.004	-0.002	-0.004	-0.007	-0.015
30.C	-0.007	-0.007	-0.015	-0.001	-0.005	-0.002	-0.002	-0.003	-0.007
31.O	0.000	0.000	0.000	0.000	0.000	0.000	0.000	0.000	0.000
32.C	0.002	-0.002	-0.006	-0.002	-0.000	-0.010	-0.005	-0.007	-0.006
33.C	-0.004	0.004	0.007	-0.001	-0.006	-0.003	-0.004	-0.006	-0.015
34.C	-0.005	0.002	0.003	0.001	-0.003	0.003	-0.004	-0.007	-0.018
35.C	-0.020	-0.002	-0.004	0.003	-0.013	0.012	0.001	0.000	-0.020
36.C	0.004	-0.003	-0.008	-0.003	-0.001	-0.015	-0.005	-0.007	-0.003
37.C	-0.029	0.005	0.012	-0.000	-0.034	-0.002	0.006	0.009	-0.018
38.C	-0.015	-0.006	-0.012	0.002	-0.008	0.009	-0.001	-0.001	-0.015
39.C	0.005	-0.005	-0.013	-0.003	0.001	-0.016	-0.005	-0.006	-0.000
40.H	-0.001	-0.001	-0.003	-0.001	-0.000	-0.002	-0.005	-0.008	-0.012
41.H	-0.007	0.004	0.008	-0.000	-0.010	-0.002	-0.002	-0.004	-0.017
42.H	-0.002	0.004	0.007	-0.001	-0.007	-0.007	-0.004	-0.006	-0.013
43.H	-0.002	0.005	0.010	-0.001	-0.004	-0.002	-0.005	-0.008	-0.017

44.H	-0.010	0.001	0.002	0.003	-0.004	0.013	-0.003	-0.006	-0.023
45.H	-0.005	0.000	-0.001	0.002	0.001	0.011	-0.005	-0.009	-0.020
46.H	-0.010	-0.002	-0.004	0.002	-0.003	0.012	-0.003	-0.006	-0.020
47.H	-0.006	-0.001	-0.004	0.001	-0.003	0.004	-0.004	-0.006	-0.015
48.H	-0.014	0.001	0.003	0.002	-0.011	0.008	-0.001	-0.002	-0.020
49.H	-0.012	0.000	0.000	0.000	-0.011	0.002	-0.001	-0.002	-0.016
50.H	-0.022	-0.001	-0.000	0.003	-0.016	0.012	0.002	0.001	-0.022
51.H	-0.022	-0.003	-0.006	0.003	-0.015	0.013	0.002	0.002	-0.020
52.H	-0.018	-0.002	-0.003	0.003	-0.010	0.014	-0.000	-0.001	-0.022
53.H	-0.016	-0.005	-0.011	0.003	-0.008	0.012	-0.001	-0.002	-0.017
54.H	-0.018	-0.006	-0.013	0.002	-0.012	0.009	0.001	0.001	-0.014
55.H	-0.012	-0.007	-0.014	0.002	-0.006	0.007	-0.001	-0.002	-0.013
56.H	0.005	-0.002	-0.007	-0.002	0.003	-0.009	-0.006	-0.009	-0.006
57.H	0.007	-0.002	-0.006	-0.004	0.001	-0.017	-0.006	-0.008	-0.002
58.H	0.001	-0.002	-0.006	-0.003	-0.004	-0.017	-0.004	-0.005	-0.002
59.H	0.001	-0.006	-0.014	-0.003	-0.002	-0.015	-0.004	-0.005	-0.000
60.H	0.007	-0.005	-0.014	-0.003	0.004	-0.015	-0.007	-0.008	-0.000
61.H	0.006	-0.005	-0.013	-0.004	0.000	-0.020	-0.005	-0.006	0.002
62.H	0.008	0.001	0.000	-0.003	0.002	-0.015	-0.007	-0.010	-0.006
63.H	0.009	0.001	-0.000	-0.002	0.005	-0.010	-0.008	-0.012	-0.009
64.H	0.006	0.003	0.004	-0.002	0.001	-0.010	-0.007	-0.010	-0.010
65.H	-0.012	-0.002	-0.004	-0.000	-0.013	-0.002	-0.000	-0.000	-0.012
66.H	-0.022	-0.004	-0.007	-0.000	-0.022	-0.003	0.003	0.006	-0.010
67.H	-0.019	-0.001	-0.002	-0.001	-0.021	-0.004	0.002	0.004	-0.011
68.H	-0.016	-0.003	-0.006	-0.001	-0.018	-0.008	0.002	0.004	-0.008
69.C	-0.017	0.003	0.008	-0.000	-0.020	-0.002	0.002	0.002	-0.016
70.H	-0.036	0.000	0.003	0.004	-0.029	0.017	0.006	0.009	-0.026
71.H	-0.040	-0.001	0.001	0.004	-0.034	0.016	0.008	0.012	-0.024
72.H	-0.036	-0.002	-0.003	0.004	-0.029	0.016	0.007	0.010	-0.022
73.H	-0.034	-0.001	-0.001	0.002	-0.031	0.008	0.007	0.010	-0.019
74.H	-0.033	0.003	0.008	0.003	-0.031	0.011	0.006	0.008	-0.024

75.H	-0.038	0.002	0.007	0.002	-0.036	0.009	0.008	0.012	-0.022
76.H	-0.025	0.006	0.015	0.001	-0.027	0.003	0.004	0.005	-0.022
77.H	-0.030	0.006	0.015	0.002	-0.030	0.008	0.005	0.007	-0.025
78.H	-0.030	0.008	0.018	0.001	-0.033	0.004	0.006	0.008	-0.024
79.H	-0.026	0.005	0.012	-0.001	-0.031	-0.004	0.005	0.008	-0.017
80.H	-0.031	0.004	0.010	-0.000	-0.036	-0.004	0.007	0.011	-0.016
81.H	-0.031	0.006	0.015	-0.000	-0.036	-0.003	0.007	0.010	-0.019
82.H	-0.026	-0.001	-0.001	0.001	-0.025	0.003	0.004	0.007	-0.015
83.H	-0.022	-0.003	-0.007	0.001	-0.018	0.006	0.002	0.004	-0.015
84.H	-0.024	0.003	0.008	0.001	-0.023	0.005	0.003	0.004	-0.021
85.H	-0.031	0.001	0.005	0.001	-0.032	0.001	0.006	0.010	-0.017
86.H	-0.004	-0.000	-0.001	-0.002	-0.008	-0.011	-0.003	-0.003	-0.008
87.O	0.000	0.000	0.000	0.000	0.000	0.000	0.000	0.000	0.000
88.H	-0.010	-0.006	-0.014	-0.001	-0.010	-0.007	-0.001	0.000	-0.005
89.H	-0.011	0.004	0.009	-0.002	-0.017	-0.008	-0.000	-0.000	-0.013
90.H	-0.016	0.003	0.006	-0.002	-0.021	-0.009	0.002	0.003	-0.012
91.H	-0.012	0.002	0.004	-0.001	-0.016	-0.007	0.000	0.000	-0.012
92.C	-0.014	0.003	0.007	-0.001	-0.018	-0.007	0.001	0.001	-0.013
93.O	0.000	0.000	0.000	0.000	0.000	0.000	0.000	0.000	0.000
94.H	-0.006	-0.006	-0.014	-0.000	-0.003	-0.000	-0.003	-0.004	-0.009
95.H	-0.010	-0.008	-0.018	-0.000	-0.006	-0.001	-0.002	-0.001	-0.007
96.H	-0.004	-0.007	-0.017	-0.001	-0.002	-0.005	-0.003	-0.004	-0.005

	85			86			87		
1.O	0.000	0.000	0.000	0.000	0.000	0.000	0.000	0.000	0.000
2.C	0.000	0.000	0.000	0.000	0.000	0.000	0.000	0.000	0.000
3.C	0.000	0.000	0.000	0.000	0.000	0.000	0.000	0.000	0.000
4.C	0.000	0.000	0.000	0.000	0.000	0.000	0.000	0.000	0.000
5.O	0.000	0.000	0.000	0.000	0.000	0.000	0.000	0.000	0.000

6.N	-0.034	0.007	0.012	0.001	-0.031	0.002	0.001	0.011	-0.018
7.O	0.000	0.000	0.000	0.000	0.000	0.000	0.000	0.000	0.000
8.C	0.000	0.000	0.000	0.000	0.000	0.000	0.000	0.000	0.000
9.C	0.000	0.000	0.000	0.000	0.000	0.000	0.000	0.000	0.000
10.C	0.000	0.000	0.000	0.000	0.000	0.000	0.000	0.000	0.000
11.O	0.000	0.000	0.000	0.000	0.000	0.000	0.000	0.000	0.000
12.C	0.000	0.000	0.000	0.000	0.000	0.000	0.000	0.000	0.000
13.O	0.000	0.000	0.000	0.000	0.000	0.000	0.000	0.000	0.000
14.C	0.000	0.000	0.000	0.000	0.000	0.000	0.000	0.000	0.000
15.C	0.000	0.000	0.000	0.000	0.000	0.000	0.000	0.000	0.000
16.O	0.000	0.000	0.000	0.000	0.000	0.000	0.000	0.000	0.000
17.C	0.000	0.000	0.000	0.000	0.000	0.000	0.000	0.000	0.000
18.C	0.000	0.000	0.000	0.000	0.000	0.000	0.000	0.000	0.000
19.O	0.000	0.000	0.000	0.000	0.000	0.000	0.000	0.000	0.000
20.C	0.000	0.000	0.000	0.000	0.000	0.000	0.000	0.000	0.000
21.C	0.000	0.000	0.000	0.000	0.000	0.000	0.000	0.000	0.000
22.C	0.000	0.000	0.000	0.000	0.000	0.000	0.000	0.000	0.000
23.C	0.000	0.000	0.000	0.000	0.000	0.000	0.000	0.000	0.000
24.C	0.000	0.000	0.000	0.000	0.000	0.000	0.000	0.000	0.000
25.C	0.000	0.000	0.000	0.000	0.000	0.000	0.000	0.000	0.000
26.C	0.000	0.000	0.000	0.000	0.000	0.000	0.000	0.000	0.000
27.C	0.270	-0.001	0.002	0.000	0.272	0.012	0.000	0.000	0.277
28.C	-0.042	-0.002	0.002	-0.002	-0.035	0.018	0.004	0.015	-0.028
29.C	-0.004	0.004	0.003	-0.001	-0.001	-0.002	0.000	-0.010	-0.014
30.C	-0.008	-0.010	-0.015	-0.000	-0.013	-0.000	-0.000	-0.000	-0.014
31.O	0.000	0.000	0.000	0.000	0.000	0.000	0.000	0.000	0.000
32.C	0.003	-0.003	-0.007	0.001	-0.003	-0.011	-0.002	-0.008	-0.007
33.C	-0.005	0.007	0.008	-0.001	-0.002	-0.005	-0.000	-0.010	-0.013
34.C	-0.007	0.003	0.004	-0.003	-0.001	0.004	0.001	-0.010	-0.019
35.C	-0.024	-0.003	-0.002	-0.004	-0.017	0.016	0.004	0.002	-0.026
36.C	0.006	-0.003	-0.008	0.003	-0.004	-0.017	-0.004	-0.007	-0.003

37.C	-0.031	0.007	0.012	0.003	-0.032	-0.004	-0.001	0.012	-0.014
38.C	-0.019	-0.008	-0.011	-0.003	-0.016	0.013	0.003	0.002	-0.023
39.C	0.007	-0.007	-0.013	0.003	-0.005	-0.018	-0.004	-0.006	-0.002
40.H	-0.002	-0.001	-0.003	-0.001	-0.001	-0.002	-0.000	-0.010	-0.014
41.H	-0.009	0.007	0.009	-0.001	-0.005	-0.003	-0.000	-0.007	-0.014
42.H	-0.003	0.007	0.007	0.001	-0.002	-0.009	-0.001	-0.009	-0.010
43.H	-0.003	0.009	0.010	-0.001	0.002	-0.004	-0.000	-0.013	-0.014
44.H	-0.015	0.002	0.003	-0.006	-0.003	0.016	0.004	-0.008	-0.027
45.H	-0.009	0.001	0.000	-0.006	0.002	0.014	0.004	-0.012	-0.025
46.H	-0.015	-0.002	-0.003	-0.005	-0.005	0.016	0.004	-0.007	-0.026
47.H	-0.009	-0.002	-0.003	-0.003	-0.004	0.006	0.002	-0.007	-0.019
48.H	-0.018	0.002	0.003	-0.003	-0.011	0.009	0.002	-0.003	-0.022
49.H	-0.014	0.001	0.001	-0.001	-0.011	0.003	0.001	-0.002	-0.018
50.H	-0.027	-0.001	0.001	-0.004	-0.018	0.016	0.004	0.003	-0.026
51.H	-0.027	-0.005	-0.005	-0.004	-0.021	0.017	0.004	0.005	-0.027
52.H	-0.023	-0.002	-0.002	-0.005	-0.013	0.018	0.004	-0.001	-0.028
53.H	-0.021	-0.008	-0.010	-0.004	-0.015	0.017	0.004	0.001	-0.026
54.H	-0.022	-0.009	-0.012	-0.003	-0.021	0.013	0.003	0.005	-0.023
55.H	-0.016	-0.010	-0.014	-0.003	-0.014	0.012	0.002	0.001	-0.022
56.H	0.006	-0.003	-0.008	0.000	0.001	-0.010	-0.002	-0.011	-0.008
57.H	0.010	-0.002	-0.007	0.003	-0.000	-0.020	-0.004	-0.010	-0.001
58.H	0.003	-0.003	-0.007	0.004	-0.007	-0.019	-0.004	-0.005	-0.002
59.H	0.003	-0.008	-0.015	0.003	-0.009	-0.015	-0.004	-0.003	-0.003
60.H	0.009	-0.007	-0.015	0.002	-0.001	-0.016	-0.004	-0.009	-0.003
61.H	0.009	-0.007	-0.014	0.004	-0.005	-0.022	-0.005	-0.006	0.001
62.H	0.010	0.003	-0.000	0.002	0.005	-0.017	-0.003	-0.014	-0.003
63.H	0.009	0.002	-0.001	-0.000	0.008	-0.011	-0.002	-0.016	-0.008
64.H	0.006	0.005	0.004	0.001	0.005	-0.013	-0.002	-0.015	-0.007
65.H	-0.014	-0.002	-0.004	0.000	-0.016	-0.002	-0.000	0.001	-0.014
66.H	-0.023	-0.006	-0.007	0.002	-0.029	-0.002	-0.001	0.011	-0.013
67.H	-0.020	-0.002	-0.002	0.002	-0.025	-0.004	-0.001	0.007	-0.012

68.H	-0.016	-0.005	-0.006	0.003	-0.024	-0.008	-0.002	0.007	-0.009
69.C	-0.019	0.005	0.008	0.001	-0.018	-0.003	-0.000	0.002	-0.014
70.H	-0.042	-0.000	0.004	-0.003	-0.032	0.021	0.005	0.013	-0.030
71.H	-0.046	-0.002	0.002	-0.002	-0.040	0.019	0.004	0.018	-0.029
72.H	-0.042	-0.004	-0.002	-0.003	-0.036	0.020	0.004	0.015	-0.029
73.H	-0.038	-0.003	-0.000	0.000	-0.037	0.010	0.002	0.016	-0.022
74.H	-0.038	0.004	0.009	-0.001	-0.031	0.013	0.003	0.011	-0.025
75.H	-0.042	0.002	0.008	0.000	-0.038	0.010	0.002	0.016	-0.023
76.H	-0.028	0.010	0.016	0.000	-0.023	0.002	0.001	0.005	-0.018
77.H	-0.034	0.009	0.015	-0.001	-0.026	0.008	0.002	0.007	-0.023
78.H	-0.033	0.011	0.019	0.001	-0.028	0.003	0.001	0.008	-0.019
79.H	-0.027	0.007	0.012	0.003	-0.028	-0.006	-0.001	0.009	-0.012
80.H	-0.032	0.005	0.010	0.004	-0.036	-0.005	-0.001	0.014	-0.013
81.H	-0.032	0.009	0.016	0.003	-0.033	-0.005	-0.001	0.012	-0.013
82.H	-0.029	-0.002	-0.001	0.001	-0.030	0.004	0.001	0.011	-0.018
83.H	-0.025	-0.005	-0.006	-0.001	-0.024	0.008	0.002	0.007	-0.021
84.H	-0.027	0.005	0.008	-0.001	-0.023	0.006	0.002	0.005	-0.020
85.H	-0.034	0.002	0.005	0.002	-0.035	0.002	0.000	0.014	-0.017
86.H	-0.003	0.000	-0.002	0.002	-0.009	-0.013	-0.003	-0.004	-0.006
87.O	0.000	0.000	0.000	0.000	0.000	0.000	0.000	0.000	0.000
88.H	-0.010	-0.010	-0.015	0.002	-0.019	-0.006	-0.002	0.004	-0.010
89.H	-0.011	0.007	0.009	0.002	-0.013	-0.011	-0.002	-0.002	-0.009
90.H	-0.015	0.004	0.006	0.004	-0.020	-0.012	-0.002	0.004	-0.008
91.H	-0.012	0.003	0.003	0.002	-0.016	-0.009	-0.002	0.000	-0.009
92.C	-0.014	0.005	0.007	0.002	-0.017	-0.009	-0.002	0.001	-0.010
93.O	0.000	0.000	0.000	0.000	0.000	0.000	0.000	0.000	0.000
94.H	-0.008	-0.009	-0.013	-0.001	-0.010	0.002	0.000	-0.002	-0.016
95.H	-0.011	-0.012	-0.018	-0.001	-0.016	0.002	0.000	0.002	-0.015
96.H	-0.005	-0.011	-0.017	0.000	-0.011	-0.003	-0.001	-0.002	-0.012

	88			89			90		
	------------------------			------------------------			------------------------		
1.O	0.000	0.000	0.000	0.000	0.000	0.000	0.000	0.000	0.000
2.C	0.000	0.000	0.000	0.000	0.000	0.000	0.000	0.000	0.000
3.C	0.000	0.000	0.000	0.000	0.000	0.000	0.000	0.000	0.000
4.C	0.000	0.000	0.000	0.000	0.000	0.000	0.000	0.000	0.000
5.O	0.000	0.000	0.000	0.000	0.000	0.000	0.000	0.000	0.000
6.N	-0.041	0.012	0.014	0.001	-0.036	0.003	-0.001	0.021	-0.020
7.O	0.000	0.000	0.000	0.000	0.000	0.000	0.000	0.000	0.000
8.C	0.000	0.000	0.000	0.000	0.000	0.000	0.000	0.000	0.000
9.C	0.000	0.000	0.000	0.000	0.000	0.000	0.000	0.000	0.000
10.C	0.000	0.000	0.000	0.000	0.000	0.000	0.000	0.000	0.000
11.O	0.000	0.000	0.000	0.000	0.000	0.000	0.000	0.000	0.000
12.C	0.000	0.000	0.000	0.000	0.000	0.000	0.000	0.000	0.000
13.O	0.000	0.000	0.000	0.000	0.000	0.000	0.000	0.000	0.000
14.C	0.000	0.000	0.000	0.000	0.000	0.000	0.000	0.000	0.000
15.C	0.000	0.000	0.000	0.000	0.000	0.000	0.000	0.000	0.000
16.O	0.000	0.000	0.000	0.000	0.000	0.000	0.000	0.000	0.000
17.C	0.000	0.000	0.000	0.000	0.000	0.000	0.000	0.000	0.000
18.C	0.000	0.000	0.000	0.000	0.000	0.000	0.000	0.000	0.000
19.O	0.000	0.000	0.000	0.000	0.000	0.000	0.000	0.000	0.000
20.C	0.000	0.000	0.000	0.000	0.000	0.000	0.000	0.000	0.000
21.C	0.000	0.000	0.000	0.000	0.000	0.000	0.000	0.000	0.000
22.C	0.000	0.000	0.000	0.000	0.000	0.000	0.000	0.000	0.000
23.C	0.000	0.000	0.000	0.000	0.000	0.000	0.000	0.000	0.000
24.C	0.000	0.000	0.000	0.000	0.000	0.000	0.000	0.000	0.000
25.C	0.000	0.000	0.000	0.000	0.000	0.000	0.000	0.000	0.000
26.C	0.000	0.000	0.000	0.000	0.000	0.000	0.000	0.000	0.000
27.C	0.000	0.000	0.000	0.000	0.000	0.000	0.000	0.000	0.000
28.C	0.261	-0.005	0.005	0.000	0.268	0.025	0.000	0.000	0.269
29.C	-0.005	0.008	0.004	-0.002	0.001	-0.002	0.002	-0.016	-0.015

30.C	-0.009	-0.020	-0.017	-0.000	-0.017	0.001	0.000	0.003	-0.017
31.O	0.000	0.000	0.000	0.000	0.000	0.000	0.000	0.000	0.000
32.C	0.008	-0.004	-0.009	0.003	-0.002	-0.014	-0.003	-0.013	-0.003
33.C	-0.005	0.014	0.008	-0.001	0.001	-0.005	0.001	-0.017	-0.011
34.C	-0.012	0.008	0.005	-0.005	0.002	0.006	0.005	-0.017	-0.023
35.C	-0.036	-0.006	-0.000	-0.007	-0.021	0.021	0.008	0.005	-0.038
36.C	0.014	-0.006	-0.012	0.006	-0.004	-0.021	-0.006	-0.011	0.005
37.C	-0.035	0.013	0.014	0.004	-0.037	-0.004	-0.004	0.022	-0.012
38.C	-0.028	-0.016	-0.011	-0.006	-0.020	0.018	0.006	0.006	-0.034
39.C	0.016	-0.013	-0.017	0.006	-0.006	-0.022	-0.007	-0.009	0.005
40.H	-0.002	-0.001	-0.004	-0.002	0.000	-0.001	0.002	-0.016	-0.015
41.H	-0.010	0.014	0.010	-0.001	-0.003	-0.003	0.001	-0.012	-0.014
42.H	-0.000	0.014	0.007	0.001	0.001	-0.011	-0.002	-0.016	-0.006
43.H	-0.003	0.018	0.011	-0.002	0.007	-0.004	0.002	-0.022	-0.013
44.H	-0.026	0.005	0.006	-0.010	-0.001	0.021	0.010	-0.014	-0.038
45.H	-0.018	0.003	0.002	-0.010	0.005	0.019	0.010	-0.020	-0.035
46.H	-0.025	-0.003	-0.001	-0.010	-0.005	0.022	0.010	-0.010	-0.038
47.H	-0.014	-0.003	-0.003	-0.005	-0.004	0.009	0.005	-0.011	-0.025
48.H	-0.026	0.004	0.005	-0.006	-0.011	0.013	0.006	-0.004	-0.030
49.H	-0.019	0.002	0.002	-0.003	-0.012	0.005	0.003	-0.003	-0.021
50.H	-0.039	-0.002	0.003	-0.007	-0.022	0.021	0.008	0.007	-0.038
51.H	-0.040	-0.010	-0.003	-0.008	-0.026	0.023	0.008	0.010	-0.040
52.H	-0.036	-0.005	0.000	-0.009	-0.016	0.025	0.010	0.001	-0.041
53.H	-0.032	-0.016	-0.009	-0.008	-0.019	0.023	0.009	0.004	-0.040
54.H	-0.032	-0.019	-0.012	-0.005	-0.027	0.017	0.005	0.012	-0.034
55.H	-0.024	-0.019	-0.014	-0.006	-0.019	0.016	0.006	0.004	-0.032
56.H	0.010	-0.005	-0.010	0.001	0.003	-0.012	-0.002	-0.018	-0.005
57.H	0.020	-0.003	-0.010	0.006	0.001	-0.025	-0.007	-0.016	0.008
58.H	0.012	-0.005	-0.011	0.007	-0.008	-0.023	-0.007	-0.007	0.007
59.H	0.011	-0.017	-0.019	0.006	-0.011	-0.019	-0.006	-0.004	0.002
60.H	0.018	-0.014	-0.019	0.005	-0.002	-0.020	-0.005	-0.013	0.003

61.H	0.021	-0.014	-0.019	0.008	-0.007	-0.027	-0.009	-0.008	0.011
62.H	0.019	0.007	-0.002	0.004	0.009	-0.022	-0.005	-0.024	0.005
63.H	0.015	0.006	-0.002	0.001	0.012	-0.013	-0.001	-0.028	-0.003
64.H	0.012	0.012	0.003	0.002	0.010	-0.015	-0.002	-0.025	-0.001
65.H	-0.016	-0.005	-0.004	0.000	-0.019	-0.001	-0.000	0.004	-0.015
66.H	-0.025	-0.012	-0.008	0.003	-0.036	-0.002	-0.003	0.021	-0.015
67.H	-0.021	-0.004	-0.002	0.003	-0.030	-0.005	-0.003	0.015	-0.012
68.H	-0.015	-0.010	-0.008	0.005	-0.030	-0.009	-0.005	0.015	-0.007
69.C	-0.021	0.010	0.009	0.001	-0.020	-0.003	-0.001	0.005	-0.013
70.H	-0.059	-0.001	0.008	-0.007	-0.039	0.028	0.008	0.024	-0.045
71.H	-0.062	-0.005	0.006	-0.005	-0.049	0.026	0.006	0.034	-0.042
72.H	-0.058	-0.009	0.001	-0.006	-0.044	0.027	0.007	0.029	-0.043
73.H	-0.049	-0.007	0.002	-0.002	-0.046	0.014	0.002	0.031	-0.030
74.H	-0.051	0.007	0.013	-0.004	-0.036	0.017	0.004	0.021	-0.034
75.H	-0.054	0.003	0.010	-0.002	-0.046	0.014	0.002	0.030	-0.031
76.H	-0.034	0.019	0.018	-0.000	-0.024	0.003	0.001	0.009	-0.020
77.H	-0.045	0.017	0.019	-0.003	-0.029	0.011	0.003	0.014	-0.028
78.H	-0.041	0.022	0.022	-0.000	-0.030	0.004	0.000	0.015	-0.021
79.H	-0.029	0.014	0.013	0.004	-0.032	-0.008	-0.004	0.017	-0.009
80.H	-0.036	0.009	0.011	0.005	-0.042	-0.006	-0.005	0.027	-0.011
81.H	-0.036	0.017	0.017	0.004	-0.038	-0.006	-0.004	0.022	-0.011
82.H	-0.035	-0.005	-0.000	0.000	-0.037	0.006	0.000	0.021	-0.022
83.H	-0.033	-0.011	-0.005	-0.003	-0.031	0.011	0.003	0.016	-0.028
84.H	-0.035	0.009	0.011	-0.002	-0.026	0.008	0.002	0.010	-0.025
85.H	-0.040	0.002	0.007	0.002	-0.042	0.003	-0.002	0.026	-0.020
86.H	0.002	0.001	-0.003	0.004	-0.010	-0.016	-0.005	-0.006	-0.001
87.O	0.000	0.000	0.000	0.000	0.000	0.000	0.000	0.000	0.000
88.H	-0.008	-0.020	-0.017	0.003	-0.025	-0.007	-0.003	0.010	-0.009
89.H	-0.009	0.014	0.009	0.004	-0.013	-0.013	-0.004	-0.002	-0.004
90.H	-0.013	0.008	0.005	0.006	-0.023	-0.014	-0.006	0.008	-0.002
91.H	-0.011	0.005	0.003	0.004	-0.017	-0.010	-0.004	0.002	-0.006

92.C	-0.013	0.009	0.006	0.004	-0.018	-0.011	-0.004	0.003	-0.006
93.O	0.000	0.000	0.000	0.000	0.000	0.000	0.000	0.000	0.000
94.H	-0.010	-0.017	-0.015	-0.002	-0.013	0.004	0.002	-0.002	-0.020
95.H	-0.014	-0.024	-0.020	-0.001	-0.022	0.004	0.001	0.007	-0.020
96.H	-0.003	-0.021	-0.020	0.001	-0.015	-0.003	-0.001	-0.000	-0.013

	91			92			93		
1.O	0.000	0.000	0.000	0.000	0.000	0.000	0.000	0.000	0.000
2.C	0.000	0.000	0.000	0.000	0.000	0.000	0.000	0.000	0.000
3.C	0.000	0.000	0.000	0.000	0.000	0.000	0.000	0.000	0.000
4.C	0.000	0.000	0.000	0.000	0.000	0.000	0.000	0.000	0.000
5.O	0.000	0.000	0.000	0.000	0.000	0.000	0.000	0.000	0.000
6.N	-0.003	0.001	-0.010	-0.000	-0.007	-0.002	0.005	0.001	-0.021
7.O	0.000	0.000	0.000	0.000	0.000	0.000	0.000	0.000	0.000
8.C	0.000	0.000	0.000	0.000	0.000	0.000	0.000	0.000	0.000
9.C	0.000	0.000	0.000	0.000	0.000	0.000	0.000	0.000	0.000
10.C	0.000	0.000	0.000	0.000	0.000	0.000	0.000	0.000	0.000
11.O	0.000	0.000	0.000	0.000	0.000	0.000	0.000	0.000	0.000
12.C	0.000	0.000	0.000	0.000	0.000	0.000	0.000	0.000	0.000
13.O	0.000	0.000	0.000	0.000	0.000	0.000	0.000	0.000	0.000
14.C	0.000	0.000	0.000	0.000	0.000	0.000	0.000	0.000	0.000
15.C	0.000	0.000	0.000	0.000	0.000	0.000	0.000	0.000	0.000
16.O	0.000	0.000	0.000	0.000	0.000	0.000	0.000	0.000	0.000
17.C	0.000	0.000	0.000	0.000	0.000	0.000	0.000	0.000	0.000
18.C	0.000	0.000	0.000	0.000	0.000	0.000	0.000	0.000	0.000
19.O	0.000	0.000	0.000	0.000	0.000	0.000	0.000	0.000	0.000
20.C	0.000	0.000	0.000	0.000	0.000	0.000	0.000	0.000	0.000
21.C	0.000	0.000	0.000	0.000	0.000	0.000	0.000	0.000	0.000
22.C	0.000	0.000	0.000	0.000	0.000	0.000	0.000	0.000	0.000

23.C	0.000	0.000	0.000	0.000	0.000	0.000	0.000	0.000	0.000
24.C	0.000	0.000	0.000	0.000	0.000	0.000	0.000	0.000	0.000
25.C	0.000	0.000	0.000	0.000	0.000	0.000	0.000	0.000	0.000
26.C	0.000	0.000	0.000	0.000	0.000	0.000	0.000	0.000	0.000
27.C	0.000	0.000	0.000	0.000	0.000	0.000	0.000	0.000	0.000
28.C	0.000	0.000	0.000	0.000	0.000	0.000	0.000	0.000	0.000
29.C	0.277	0.002	-0.003	0.000	0.274	0.000	0.000	0.000	0.280
30.C	-0.020	-0.004	0.013	0.000	-0.009	-0.003	-0.001	-0.001	-0.012
31.O	0.000	0.000	0.000	0.000	0.000	0.000	0.000	0.000	0.000
32.C	-0.023	-0.000	0.004	-0.004	-0.024	0.008	-0.003	-0.000	-0.015
33.C	-0.023	0.003	-0.007	0.001	-0.032	0.003	-0.003	0.001	-0.020
34.C	-0.027	0.002	-0.002	0.007	-0.031	-0.005	-0.004	0.001	-0.018
35.C	-0.019	-0.001	0.005	0.011	-0.012	-0.016	-0.001	-0.000	-0.015
36.C	-0.022	-0.000	0.004	-0.009	-0.022	0.013	-0.002	-0.000	-0.015
37.C	-0.001	0.001	-0.011	-0.004	-0.007	0.003	0.006	0.001	-0.021
38.C	-0.021	-0.003	0.012	0.009	-0.008	-0.014	-0.001	-0.001	-0.013
39.C	-0.022	-0.002	0.008	-0.009	-0.019	0.013	-0.002	-0.001	-0.013
40.H	-0.026	0.001	0.002	0.002	-0.027	-0.000	-0.004	0.000	-0.016
41.H	-0.021	0.003	-0.008	0.001	-0.030	0.002	-0.002	0.001	-0.020
42.H	-0.022	0.003	-0.008	-0.002	-0.032	0.007	-0.002	0.001	-0.020
43.H	-0.025	0.004	-0.009	0.002	-0.037	0.003	-0.003	0.001	-0.020
44.H	-0.028	0.002	0.001	0.014	-0.028	-0.016	-0.004	0.000	-0.017
45.H	-0.031	0.001	0.003	0.013	-0.031	-0.014	-0.006	0.000	-0.016
46.H	-0.028	-0.000	0.006	0.014	-0.023	-0.017	-0.004	-0.000	-0.015
47.H	-0.026	0.000	0.004	0.007	-0.024	-0.007	-0.003	-0.000	-0.016
48.H	-0.021	0.001	-0.001	0.008	-0.021	-0.010	-0.001	0.000	-0.018
49.H	-0.020	0.000	-0.000	0.004	-0.020	-0.004	-0.001	0.000	-0.017
50.H	-0.018	-0.001	0.003	0.011	-0.012	-0.016	-0.000	-0.000	-0.016
51.H	-0.018	-0.002	0.008	0.011	-0.007	-0.018	-0.000	-0.001	-0.014
52.H	-0.022	-0.001	0.005	0.013	-0.015	-0.019	-0.002	-0.000	-0.015
53.H	-0.022	-0.003	0.012	0.012	-0.010	-0.018	-0.002	-0.001	-0.013

54.H	-0.018	-0.004	0.012	0.008	-0.004	-0.014	-0.000	-0.001	-0.012
55.H	-0.022	-0.004	0.014	0.008	-0.009	-0.013	-0.002	-0.001	-0.012
56.H	-0.027	0.000	0.005	-0.003	-0.027	0.007	-0.004	-0.000	-0.015
57.H	-0.023	0.000	0.002	-0.010	-0.027	0.016	-0.003	-0.000	-0.016
58.H	-0.019	-0.001	0.003	-0.010	-0.020	0.015	-0.001	-0.000	-0.015
59.H	-0.020	-0.003	0.010	-0.009	-0.014	0.011	-0.002	-0.001	-0.013
60.H	-0.025	-0.002	0.009	-0.008	-0.021	0.012	-0.003	-0.001	-0.013
61.H	-0.020	-0.002	0.008	-0.012	-0.018	0.017	-0.002	-0.001	-0.013
62.H	-0.026	0.002	-0.003	-0.007	-0.035	0.015	-0.004	0.000	-0.018
63.H	-0.030	0.003	-0.002	-0.002	-0.037	0.009	-0.005	0.000	-0.017
64.H	-0.027	0.003	-0.006	-0.003	-0.037	0.010	-0.004	0.001	-0.019
65.H	-0.016	-0.001	0.003	-0.000	-0.013	-0.000	0.000	-0.000	-0.016
66.H	-0.008	-0.003	0.005	-0.003	0.000	-0.000	0.004	-0.001	-0.015
67.H	-0.009	-0.002	0.001	-0.004	-0.006	0.002	0.003	-0.000	-0.017
68.H	-0.010	-0.003	0.004	-0.006	-0.005	0.005	0.003	-0.001	-0.015
69.C	-0.012	0.001	-0.007	-0.001	-0.017	0.002	0.002	0.001	-0.020
70.H	-0.009	-0.002	0.001	0.012	-0.001	-0.020	0.004	-0.000	-0.017
71.H	-0.004	-0.003	0.002	0.009	0.006	-0.019	0.006	-0.000	-0.017
72.H	-0.007	-0.003	0.005	0.010	0.005	-0.020	0.004	-0.001	-0.015
73.H	-0.004	-0.003	0.002	0.004	0.005	-0.011	0.005	-0.000	-0.016
74.H	-0.007	0.000	-0.005	0.007	-0.006	-0.012	0.004	0.000	-0.019
75.H	-0.002	-0.001	-0.004	0.004	0.001	-0.010	0.006	0.000	-0.019
76.H	-0.009	0.003	-0.012	0.002	-0.017	-0.002	0.003	0.001	-0.022
77.H	-0.008	0.002	-0.011	0.005	-0.014	-0.008	0.004	0.001	-0.022
78.H	-0.005	0.003	-0.015	0.001	-0.014	-0.003	0.005	0.001	-0.023
79.H	-0.003	0.001	-0.011	-0.005	-0.011	0.005	0.005	0.001	-0.021
80.H	0.001	-0.000	-0.009	-0.006	-0.003	0.004	0.007	0.000	-0.020
81.H	-0.000	0.002	-0.014	-0.005	-0.008	0.004	0.007	0.001	-0.022
82.H	-0.007	-0.002	0.001	0.001	-0.002	-0.005	0.004	-0.000	-0.016
83.H	-0.013	-0.003	0.006	0.004	-0.004	-0.010	0.002	-0.001	-0.015
84.H	-0.011	0.001	-0.006	0.004	-0.013	-0.006	0.002	0.001	-0.019

85.H	-0.002	-0.001	-0.004	-0.001	-0.001	-0.003	0.006	0.000	-0.019
86.H	-0.018	0.000	-0.001	-0.006	-0.021	0.010	-0.001	0.000	-0.017
87.O	0.000	0.000	0.000	0.000	0.000	0.000	0.000	0.000	0.000
88.H	-0.015	-0.004	0.011	-0.005	-0.004	0.003	0.001	-0.001	-0.012
89.H	-0.013	0.002	-0.009	-0.005	-0.023	0.009	0.001	0.001	-0.020
90.H	-0.009	0.001	-0.007	-0.008	-0.015	0.010	0.003	0.000	-0.019
91.H	-0.013	0.001	-0.004	-0.005	-0.018	0.007	0.001	0.000	-0.018
92.C	-0.012	0.001	-0.007	-0.005	-0.018	0.007	0.002	0.001	-0.020
93.O	0.000	0.000	0.000	0.000	0.000	0.000	0.000	0.000	0.000
94.H	-0.023	-0.003	0.012	0.003	-0.013	-0.005	-0.002	-0.001	-0.012
95.H	-0.019	-0.005	0.015	0.001	-0.005	-0.005	-0.001	-0.001	-0.011
96.H	-0.021	-0.004	0.014	-0.001	-0.010	-0.000	-0.002	-0.001	-0.011

	94			95			96		
1.O	0.000	0.000	0.000	0.000	0.000	0.000	0.000	0.000	0.000
2.C	0.000	0.000	0.000	0.000	0.000	0.000	0.000	0.000	0.000
3.C	0.000	0.000	0.000	0.000	0.000	0.000	0.000	0.000	0.000
4.C	0.000	0.000	0.000	0.000	0.000	0.000	0.000	0.000	0.000
5.O	0.000	0.000	0.000	0.000	0.000	0.000	0.000	0.000	0.000
6.N	-0.011	-0.000	-0.005	-0.002	0.001	-0.004	-0.024	-0.002	-0.001
7.O	0.000	0.000	0.000	0.000	0.000	0.000	0.000	0.000	0.000
8.C	0.000	0.000	0.000	0.000	0.000	0.000	0.000	0.000	0.000
9.C	0.000	0.000	0.000	0.000	0.000	0.000	0.000	0.000	0.000
10.C	0.000	0.000	0.000	0.000	0.000	0.000	0.000	0.000	0.000
11.O	0.000	0.000	0.000	0.000	0.000	0.000	0.000	0.000	0.000
12.C	0.000	0.000	0.000	0.000	0.000	0.000	0.000	0.000	0.000
13.O	0.000	0.000	0.000	0.000	0.000	0.000	0.000	0.000	0.000
14.C	0.000	0.000	0.000	0.000	0.000	0.000	0.000	0.000	0.000
15.C	0.000	0.000	0.000	0.000	0.000	0.000	0.000	0.000	0.000

16.O	0.000	0.000	0.000	0.000	0.000	0.000	0.000	0.000	0.000
17.C	0.000	0.000	0.000	0.000	0.000	0.000	0.000	0.000	0.000
18.C	0.000	0.000	0.000	0.000	0.000	0.000	0.000	0.000	0.000
19.O	0.000	0.000	0.000	0.000	0.000	0.000	0.000	0.000	0.000
20.C	0.000	0.000	0.000	0.000	0.000	0.000	0.000	0.000	0.000
21.C	0.000	0.000	0.000	0.000	0.000	0.000	0.000	0.000	0.000
22.C	0.000	0.000	0.000	0.000	0.000	0.000	0.000	0.000	0.000
23.C	0.000	0.000	0.000	0.000	0.000	0.000	0.000	0.000	0.000
24.C	0.000	0.000	0.000	0.000	0.000	0.000	0.000	0.000	0.000
25.C	0.000	0.000	0.000	0.000	0.000	0.000	0.000	0.000	0.000
26.C	0.000	0.000	0.000	0.000	0.000	0.000	0.000	0.000	0.000
27.C	0.000	0.000	0.000	0.000	0.000	0.000	0.000	0.000	0.000
28.C	0.000	0.000	0.000	0.000	0.000	0.000	0.000	0.000	0.000
29.C	0.000	0.000	0.000	0.000	0.000	0.000	0.000	0.000	0.000
30.C	0.279	-0.000	0.006	0.000	0.267	0.005	0.000	0.000	0.269
31.O	0.000	0.000	0.000	0.000	0.000	0.000	0.000	0.000	0.000
32.C	-0.022	0.000	0.002	0.012	-0.027	0.001	0.013	0.001	-0.025
33.C	-0.021	0.001	-0.004	-0.000	-0.002	-0.003	0.012	-0.005	-0.006
34.C	-0.022	0.000	-0.001	-0.013	-0.011	-0.001	0.018	-0.003	-0.014
35.C	-0.018	-0.000	0.002	-0.025	-0.026	0.003	0.004	0.002	-0.026
36.C	-0.022	0.000	0.002	0.021	-0.029	0.000	0.010	0.002	-0.026
37.C	-0.010	-0.000	-0.006	0.007	0.003	-0.005	-0.027	-0.003	0.001
38.C	-0.019	-0.000	0.005	-0.020	-0.041	0.006	0.007	0.005	-0.038
39.C	-0.022	0.000	0.004	0.023	-0.038	0.002	0.010	0.004	-0.034
40.H	-0.023	0.000	0.001	-0.004	-0.022	0.001	0.018	-0.000	-0.023
41.H	-0.020	0.000	-0.004	-0.002	-0.001	-0.003	0.008	-0.004	-0.005
42.H	-0.021	0.001	-0.004	0.006	-0.002	-0.004	0.010	-0.004	-0.005
43.H	-0.022	0.001	-0.005	-0.003	0.003	-0.004	0.016	-0.006	-0.002
44.H	-0.022	0.000	-0.000	-0.030	-0.014	0.001	0.020	-0.002	-0.018
45.H	-0.024	0.000	0.001	-0.028	-0.019	0.002	0.026	-0.001	-0.022
46.H	-0.022	0.000	0.002	-0.029	-0.025	0.003	0.020	0.001	-0.027

47.H	-0.022	0.000	0.002	-0.014	-0.024	0.002	0.016	0.001	-0.025
48.H	-0.019	0.000	-0.001	-0.018	-0.013	0.000	0.007	-0.001	-0.016
49.H	-0.019	0.000	-0.000	-0.008	-0.017	0.000	0.005	-0.000	-0.018
50.H	-0.017	-0.000	0.001	-0.025	-0.020	0.002	0.001	0.001	-0.022
51.H	-0.017	-0.000	0.003	-0.026	-0.031	0.004	0.001	0.004	-0.031
52.H	-0.019	-0.000	0.002	-0.030	-0.025	0.004	0.010	0.002	-0.026
53.H	-0.019	-0.000	0.005	-0.027	-0.040	0.006	0.010	0.005	-0.038
54.H	-0.017	-0.000	0.006	-0.018	-0.043	0.006	0.002	0.006	-0.040
55.H	-0.020	-0.000	0.007	-0.018	-0.045	0.007	0.010	0.006	-0.042
56.H	-0.024	0.000	0.002	0.008	-0.029	0.001	0.019	0.001	-0.027
57.H	-0.023	0.000	0.001	0.023	-0.026	-0.000	0.013	0.000	-0.023
58.H	-0.020	0.000	0.002	0.024	-0.027	-0.000	0.005	0.002	-0.024
59.H	-0.021	-0.000	0.005	0.020	-0.042	0.003	0.007	0.005	-0.037
60.H	-0.023	0.000	0.005	0.019	-0.040	0.003	0.015	0.004	-0.036
61.H	-0.022	0.000	0.004	0.029	-0.040	0.002	0.008	0.004	-0.034
62.H	-0.024	0.001	-0.001	0.018	-0.013	-0.002	0.017	-0.003	-0.014
63.H	-0.025	0.001	-0.001	0.007	-0.015	-0.001	0.024	-0.003	-0.016
64.H	-0.024	0.001	-0.003	0.010	-0.006	-0.003	0.019	-0.004	-0.009
65.H	-0.018	-0.000	0.001	0.001	-0.025	0.001	-0.001	0.002	-0.024
66.H	-0.014	-0.001	0.003	0.006	-0.032	0.002	-0.016	0.005	-0.028
67.H	-0.014	-0.000	0.000	0.007	-0.022	0.000	-0.013	0.002	-0.020
68.H	-0.015	-0.000	0.002	0.013	-0.030	0.001	-0.012	0.004	-0.026
69.C	-0.016	0.000	-0.004	0.002	-0.004	-0.003	-0.008	-0.002	-0.006
70.H	-0.012	-0.001	-0.000	-0.029	-0.016	0.002	-0.015	0.002	-0.018
71.H	-0.010	-0.001	0.000	-0.024	-0.020	0.002	-0.024	0.003	-0.020
72.H	-0.011	-0.001	0.002	-0.026	-0.027	0.004	-0.017	0.004	-0.026
73.H	-0.010	-0.001	0.001	-0.011	-0.023	0.002	-0.023	0.004	-0.021
74.H	-0.012	-0.000	-0.003	-0.018	-0.005	-0.001	-0.018	-0.001	-0.008
75.H	-0.009	-0.001	-0.003	-0.012	-0.009	-0.001	-0.027	0.001	-0.010
76.H	-0.014	0.000	-0.007	-0.005	0.009	-0.005	-0.014	-0.005	0.004
77.H	-0.012	-0.000	-0.006	-0.014	0.007	-0.004	-0.016	-0.004	0.003

78.H	-0.011	-0.000	-0.008	-0.006	0.014	-0.006	-0.021	-0.005	0.009
79.H	-0.012	-0.000	-0.006	0.010	0.003	-0.005	-0.023	-0.003	0.001
80.H	-0.009	-0.000	-0.005	0.010	-0.002	-0.004	-0.031	-0.001	-0.002
81.H	-0.010	-0.000	-0.007	0.008	0.009	-0.006	-0.029	-0.004	0.006
82.H	-0.013	-0.001	0.001	-0.003	-0.022	0.001	-0.017	0.003	-0.021
83.H	-0.015	-0.000	0.003	-0.011	-0.031	0.003	-0.007	0.004	-0.029
84.H	-0.014	-0.000	-0.003	-0.010	-0.005	-0.002	-0.010	-0.002	-0.007
85.H	-0.010	-0.001	-0.002	0.000	-0.011	-0.002	-0.025	0.001	-0.011
86.H	-0.020	0.000	-0.000	0.015	-0.019	-0.001	0.003	-0.000	-0.018
87.O	0.000	0.000	0.000	0.000	0.000	0.000	0.000	0.000	0.000
88.H	-0.018	-0.000	0.006	0.010	-0.044	0.004	-0.003	0.006	-0.039
89.H	-0.017	0.000	-0.005	0.012	-0.000	-0.005	-0.005	-0.004	-0.002
90.H	-0.015	-0.000	-0.003	0.016	-0.006	-0.004	-0.013	-0.002	-0.006
91.H	-0.017	0.000	-0.002	0.010	-0.011	-0.002	-0.005	-0.001	-0.011
92.C	-0.016	0.000	-0.003	0.011	-0.005	-0.004	-0.008	-0.002	-0.006
93.O	0.000	0.000	0.000	0.000	0.000	0.000	0.000	0.000	0.000
94.H	-0.021	-0.000	0.006	-0.005	-0.042	0.005	0.011	0.005	-0.039
95.H	-0.019	-0.000	0.008	-0.003	-0.051	0.007	0.005	0.008	-0.046
96.H	-0.021	-0.000	0.007	0.003	-0.049	0.006	0.009	0.007	-0.043

	97			98			99		
1.O	0.000	0.000	0.000	0.000	0.000	0.000	0.000	0.000	0.000
2.C	0.000	0.000	0.000	0.000	0.000	0.000	0.000	0.000	0.000
3.C	0.000	0.000	0.000	0.000	0.000	0.000	0.000	0.000	0.000
4.C	0.000	0.000	0.000	0.000	0.000	0.000	0.000	0.000	0.000
5.O	0.000	0.000	0.000	0.000	0.000	0.000	0.000	0.000	0.000
6.N	-0.000	-0.006	-0.011	-0.001	0.002	-0.005	-0.010	-0.012	-0.009
7.O	0.000	0.000	0.000	0.000	0.000	0.000	0.000	0.000	0.000
8.C	0.000	0.000	0.000	0.000	0.000	0.000	0.000	0.000	0.000

9.C	0.000	0.000	0.000	0.000	0.000	0.000	0.000	0.000	0.000
10.C	0.000	0.000	0.000	0.000	0.000	0.000	0.000	0.000	0.000
11.O	0.000	0.000	0.000	0.000	0.000	0.000	0.000	0.000	0.000
12.C	0.000	0.000	0.000	0.000	0.000	0.000	0.000	0.000	0.000
13.O	0.000	0.000	0.000	0.000	0.000	0.000	0.000	0.000	0.000
14.C	0.000	0.000	0.000	0.000	0.000	0.000	0.000	0.000	0.000
15.C	0.000	0.000	0.000	0.000	0.000	0.000	0.000	0.000	0.000
16.O	0.000	0.000	0.000	0.000	0.000	0.000	0.000	0.000	0.000
17.C	0.000	0.000	0.000	0.000	0.000	0.000	0.000	0.000	0.000
18.C	0.000	0.000	0.000	0.000	0.000	0.000	0.000	0.000	0.000
19.O	0.000	0.000	0.000	0.000	0.000	0.000	0.000	0.000	0.000
20.C	0.000	0.000	0.000	0.000	0.000	0.000	0.000	0.000	0.000
21.C	0.000	0.000	0.000	0.000	0.000	0.000	0.000	0.000	0.000
22.C	0.000	0.000	0.000	0.000	0.000	0.000	0.000	0.000	0.000
23.C	0.000	0.000	0.000	0.000	0.000	0.000	0.000	0.000	0.000
24.C	0.000	0.000	0.000	0.000	0.000	0.000	0.000	0.000	0.000
25.C	0.000	0.000	0.000	0.000	0.000	0.000	0.000	0.000	0.000
26.C	0.000	0.000	0.000	0.000	0.000	0.000	0.000	0.000	0.000
27.C	0.000	0.000	0.000	0.000	0.000	0.000	0.000	0.000	0.000
28.C	0.000	0.000	0.000	0.000	0.000	0.000	0.000	0.000	0.000
29.C	0.000	0.000	0.000	0.000	0.000	0.000	0.000	0.000	0.000
30.C	0.000	0.000	0.000	0.000	0.000	0.000	0.000	0.000	0.000
31.O	0.000	0.000	0.000	0.000	0.000	0.000	0.000	0.000	0.000
32.C	0.271	0.003	0.008	0.000	0.273	0.008	0.000	0.000	0.274
33.C	-0.029	-0.008	-0.007	0.001	-0.025	0.001	0.005	0.009	-0.017
34.C	-0.025	-0.004	-0.003	-0.002	-0.029	-0.005	0.005	0.010	-0.013
35.C	-0.007	0.004	0.001	-0.005	-0.020	-0.013	-0.003	-0.001	-0.010
36.C	-0.041	0.004	0.010	0.006	-0.033	0.013	0.009	0.009	-0.036
37.C	-0.004	-0.007	-0.011	0.001	0.003	0.000	-0.009	-0.013	-0.013
38.C	-0.012	0.011	0.010	-0.004	-0.027	-0.010	-0.000	-0.000	-0.017
39.C	-0.042	0.009	0.014	0.006	-0.036	0.014	0.009	0.008	-0.039

40.H	-0.031	0.001	0.004	0.000	-0.033	0.000	0.007	0.010	-0.022
41.H	-0.024	-0.008	-0.008	0.000	-0.021	-0.000	0.003	0.007	-0.015
42.H	-0.031	-0.008	-0.006	0.002	-0.024	0.005	0.006	0.009	-0.020
43.H	-0.030	-0.011	-0.009	0.000	-0.026	0.000	0.007	0.012	-0.014
44.H	-0.015	-0.003	-0.004	-0.006	-0.028	-0.014	0.003	0.009	-0.006
45.H	-0.022	-0.001	-0.001	-0.005	-0.034	-0.012	0.006	0.013	-0.009
46.H	-0.016	0.003	0.002	-0.006	-0.031	-0.014	0.003	0.008	-0.010
47.H	-0.023	0.002	0.003	-0.002	-0.031	-0.006	0.005	0.008	-0.017
48.H	-0.014	-0.002	-0.004	-0.004	-0.021	-0.009	0.000	0.003	-0.010
49.H	-0.019	-0.000	-0.001	-0.001	-0.022	-0.004	0.001	0.003	-0.016
50.H	-0.005	0.002	-0.001	-0.006	-0.017	-0.014	-0.004	-0.002	-0.008
51.H	-0.004	0.007	0.004	-0.006	-0.019	-0.014	-0.004	-0.004	-0.010
52.H	-0.008	0.003	0.001	-0.006	-0.023	-0.016	-0.001	0.001	-0.008
53.H	-0.010	0.010	0.009	-0.006	-0.027	-0.014	-0.001	0.001	-0.014
54.H	-0.009	0.012	0.011	-0.004	-0.024	-0.010	-0.002	-0.003	-0.018
55.H	-0.016	0.013	0.013	-0.004	-0.030	-0.009	0.001	0.001	-0.020
56.H	-0.040	0.004	0.008	0.003	-0.037	0.007	0.010	0.012	-0.030
57.H	-0.045	0.002	0.008	0.007	-0.035	0.015	0.011	0.011	-0.036
58.H	-0.039	0.004	0.009	0.006	-0.029	0.014	0.007	0.006	-0.036
59.H	-0.038	0.011	0.016	0.005	-0.034	0.013	0.008	0.005	-0.039
60.H	-0.044	0.009	0.015	0.006	-0.039	0.013	0.011	0.011	-0.039
61.H	-0.045	0.009	0.016	0.008	-0.035	0.018	0.010	0.008	-0.043
62.H	-0.045	-0.004	0.002	0.005	-0.034	0.013	0.011	0.014	-0.030
63.H	-0.043	-0.003	0.002	0.003	-0.038	0.007	0.012	0.017	-0.026
64.H	-0.041	-0.007	-0.003	0.004	-0.032	0.008	0.010	0.015	-0.024
65.H	-0.020	0.004	0.004	0.000	-0.021	0.000	0.000	-0.001	-0.022
66.H	-0.011	0.008	0.007	0.001	-0.013	0.001	-0.005	-0.010	-0.025
67.H	-0.014	0.003	0.002	0.001	-0.012	0.002	-0.004	-0.007	-0.023
68.H	-0.019	0.007	0.007	0.003	-0.016	0.006	-0.002	-0.006	-0.028
69.C	-0.015	-0.005	-0.007	0.000	-0.011	0.000	-0.002	-0.003	-0.016
70.H	0.011	0.002	-0.005	-0.007	-0.004	-0.019	-0.012	-0.013	-0.002

71.H	0.014	0.004	-0.003	-0.007	0.001	-0.017	-0.014	-0.018	-0.004
72.H	0.010	0.007	0.000	-0.007	-0.005	-0.017	-0.012	-0.015	-0.006
73.H	0.005	0.005	-0.000	-0.004	-0.002	-0.009	-0.011	-0.016	-0.012
74.H	0.006	-0.004	-0.010	-0.005	-0.001	-0.013	-0.011	-0.012	-0.004
75.H	0.009	-0.001	-0.008	-0.004	0.004	-0.011	-0.013	-0.017	-0.006
76.H	-0.006	-0.011	-0.015	-0.002	-0.003	-0.005	-0.006	-0.006	-0.007
77.H	0.001	-0.010	-0.015	-0.004	-0.000	-0.010	-0.009	-0.009	-0.003
78.H	-0.000	-0.013	-0.018	-0.002	0.003	-0.006	-0.009	-0.010	-0.004
79.H	-0.008	-0.008	-0.011	0.001	-0.000	0.002	-0.007	-0.010	-0.015
80.H	-0.003	-0.004	-0.009	0.001	0.004	0.001	-0.010	-0.016	-0.015
81.H	-0.002	-0.010	-0.014	0.001	0.006	0.000	-0.010	-0.014	-0.011
82.H	-0.005	0.004	0.001	-0.001	-0.008	-0.004	-0.007	-0.011	-0.017
83.H	-0.007	0.008	0.005	-0.003	-0.016	-0.007	-0.004	-0.007	-0.017
84.H	-0.005	-0.005	-0.008	-0.003	-0.008	-0.007	-0.006	-0.006	-0.009
85.H	-0.000	-0.000	-0.005	-0.001	-0.000	-0.003	-0.010	-0.014	-0.014
86.H	-0.032	0.000	0.003	0.004	-0.025	0.009	0.005	0.004	-0.028
87.O	0.000	0.000	0.000	0.000	0.000	0.000	0.000	0.000	0.000
88.H	-0.024	0.013	0.015	0.003	-0.025	0.006	0.001	-0.002	-0.033
89.H	-0.024	-0.008	-0.007	0.003	-0.014	0.006	0.001	0.001	-0.020
90.H	-0.020	-0.004	-0.005	0.003	-0.010	0.007	-0.002	-0.004	-0.022
91.H	-0.022	-0.003	-0.002	0.002	-0.016	0.005	0.000	-0.001	-0.022
92.C	-0.021	-0.005	-0.005	0.002	-0.013	0.005	-0.001	-0.002	-0.020
93.O	0.000	0.000	0.000	0.000	0.000	0.000	0.000	0.000	0.000
94.H	-0.025	0.011	0.013	-0.001	-0.033	-0.001	0.004	0.004	-0.026
95.H	-0.022	0.016	0.017	-0.000	-0.030	-0.001	0.002	-0.000	-0.029
96.H	-0.029	0.014	0.017	0.002	-0.034	0.003	0.005	0.004	-0.032

	100			101			102		
	------------------------			------------------------			------------------------		
1.O	0.000	0.000	0.000	0.000	0.000	0.000	0.000	0.000	0.000

2.C	0.000	0.000	0.000	0.000	0.000	0.000	0.000	0.000	0.000
3.C	0.000	0.000	0.000	0.000	0.000	0.000	0.000	0.000	0.000
4.C	0.000	0.000	0.000	0.000	0.000	0.000	0.000	0.000	0.000
5.O	0.000	0.000	0.000	0.000	0.000	0.000	0.000	0.000	0.000
6.N	-0.005	-0.001	-0.010	0.000	-0.014	-0.002	0.011	-0.002	-0.028
7.O	0.000	0.000	0.000	0.000	0.000	0.000	0.000	0.000	0.000
8.C	0.000	0.000	0.000	0.000	0.000	0.000	0.000	0.000	0.000
9.C	0.000	0.000	0.000	0.000	0.000	0.000	0.000	0.000	0.000
10.C	0.000	0.000	0.000	0.000	0.000	0.000	0.000	0.000	0.000
11.O	0.000	0.000	0.000	0.000	0.000	0.000	0.000	0.000	0.000
12.C	0.000	0.000	0.000	0.000	0.000	0.000	0.000	0.000	0.000
13.O	0.000	0.000	0.000	0.000	0.000	0.000	0.000	0.000	0.000
14.C	0.000	0.000	0.000	0.000	0.000	0.000	0.000	0.000	0.000
15.C	0.000	0.000	0.000	0.000	0.000	0.000	0.000	0.000	0.000
16.O	0.000	0.000	0.000	0.000	0.000	0.000	0.000	0.000	0.000
17.C	0.000	0.000	0.000	0.000	0.000	0.000	0.000	0.000	0.000
18.C	0.000	0.000	0.000	0.000	0.000	0.000	0.000	0.000	0.000
19.O	0.000	0.000	0.000	0.000	0.000	0.000	0.000	0.000	0.000
20.C	0.000	0.000	0.000	0.000	0.000	0.000	0.000	0.000	0.000
21.C	0.000	0.000	0.000	0.000	0.000	0.000	0.000	0.000	0.000
22.C	0.000	0.000	0.000	0.000	0.000	0.000	0.000	0.000	0.000
23.C	0.000	0.000	0.000	0.000	0.000	0.000	0.000	0.000	0.000
24.C	0.000	0.000	0.000	0.000	0.000	0.000	0.000	0.000	0.000
25.C	0.000	0.000	0.000	0.000	0.000	0.000	0.000	0.000	0.000
26.C	0.000	0.000	0.000	0.000	0.000	0.000	0.000	0.000	0.000
27.C	0.000	0.000	0.000	0.000	0.000	0.000	0.000	0.000	0.000
28.C	0.000	0.000	0.000	0.000	0.000	0.000	0.000	0.000	0.000
29.C	0.000	0.000	0.000	0.000	0.000	0.000	0.000	0.000	0.000
30.C	0.000	0.000	0.000	0.000	0.000	0.000	0.000	0.000	0.000
31.O	0.000	0.000	0.000	0.000	0.000	0.000	0.000	0.000	0.000
32.C	0.000	0.000	0.000	0.000	0.000	0.000	0.000	0.000	0.000

33.C	0.274	0.000	-0.007	0.000	0.267	0.005	0.000	0.000	0.275
34.C	-0.031	0.000	-0.002	0.011	-0.041	-0.006	-0.011	0.005	-0.021
35.C	-0.020	0.000	0.005	0.019	-0.013	-0.021	-0.004	-0.001	-0.012
36.C	-0.032	0.001	0.007	-0.017	-0.026	0.018	-0.005	0.002	-0.019
37.C	-0.005	-0.001	-0.011	-0.007	-0.014	0.005	0.014	-0.002	-0.031
38.C	-0.023	0.000	0.013	0.015	-0.005	-0.019	-0.006	-0.003	-0.006
39.C	-0.032	0.001	0.012	-0.018	-0.019	0.017	-0.005	0.001	-0.016
40.H	-0.033	0.001	0.004	0.003	-0.033	-0.000	-0.010	0.004	-0.018
41.H	-0.026	0.000	-0.008	0.001	-0.042	0.003	-0.005	0.005	-0.027
42.H	-0.029	0.000	-0.007	-0.005	-0.044	0.011	-0.006	0.006	-0.028
43.H	-0.032	0.000	-0.009	0.003	-0.052	0.005	-0.010	0.008	-0.029
44.H	-0.030	0.000	0.000	0.024	-0.036	-0.020	-0.013	0.004	-0.016
45.H	-0.034	0.001	0.003	0.023	-0.039	-0.018	-0.016	0.005	-0.014
46.H	-0.030	0.001	0.006	0.023	-0.027	-0.021	-0.013	0.002	-0.011
47.H	-0.030	0.001	0.005	0.011	-0.028	-0.009	-0.010	0.002	-0.015
48.H	-0.023	0.000	-0.001	0.014	-0.028	-0.012	-0.006	0.002	-0.019
49.H	-0.024	0.000	0.001	0.006	-0.025	-0.005	-0.004	0.001	-0.019
50.H	-0.018	-0.000	0.002	0.019	-0.015	-0.020	-0.003	-0.001	-0.014
51.H	-0.018	0.000	0.008	0.020	-0.006	-0.023	-0.003	-0.003	-0.009
52.H	-0.023	0.000	0.005	0.023	-0.018	-0.024	-0.007	-0.000	-0.011
53.H	-0.024	0.000	0.013	0.021	-0.007	-0.024	-0.008	-0.002	-0.005
54.H	-0.020	0.000	0.014	0.014	0.001	-0.019	-0.003	-0.005	-0.005
55.H	-0.025	0.001	0.016	0.014	-0.005	-0.018	-0.007	-0.003	-0.004
56.H	-0.035	0.001	0.007	-0.007	-0.032	0.009	-0.010	0.003	-0.017
57.H	-0.034	0.001	0.005	-0.019	-0.032	0.021	-0.007	0.004	-0.022
58.H	-0.029	0.000	0.006	-0.019	-0.023	0.020	-0.002	0.001	-0.021
59.H	-0.030	0.001	0.014	-0.016	-0.013	0.014	-0.004	-0.001	-0.013
60.H	-0.035	0.001	0.013	-0.015	-0.022	0.015	-0.008	0.001	-0.014
61.H	-0.032	0.001	0.012	-0.023	-0.018	0.022	-0.004	0.000	-0.016
62.H	-0.036	0.001	-0.001	-0.014	-0.045	0.019	-0.009	0.006	-0.026
63.H	-0.039	0.001	0.000	-0.005	-0.048	0.012	-0.013	0.007	-0.023

64.H	-0.036	0.001	-0.004	-0.008	-0.050	0.014	-0.010	0.007	-0.027
65.H	-0.021	0.000	0.004	-0.001	-0.015	-0.000	-0.001	-0.001	-0.017
66.H	-0.012	-0.000	0.007	-0.006	0.004	-0.000	0.008	-0.006	-0.015
67.H	-0.014	-0.000	0.002	-0.007	-0.007	0.003	0.006	-0.003	-0.020
68.H	-0.016	-0.000	0.006	-0.011	-0.003	0.007	0.006	-0.004	-0.017
69.C	-0.017	-0.000	-0.007	-0.003	-0.025	0.004	0.003	0.001	-0.027
70.H	-0.007	-0.001	-0.000	0.022	-0.002	-0.026	0.006	-0.005	-0.015
71.H	-0.001	-0.001	0.001	0.018	0.008	-0.024	0.011	-0.007	-0.014
72.H	-0.005	-0.001	0.005	0.019	0.008	-0.026	0.007	-0.007	-0.011
73.H	-0.004	-0.001	0.002	0.007	0.007	-0.014	0.011	-0.007	-0.016
74.H	-0.006	-0.001	-0.006	0.013	-0.011	-0.015	0.008	-0.003	-0.022
75.H	-0.001	-0.001	-0.005	0.008	-0.001	-0.013	0.013	-0.005	-0.022
76.H	-0.011	-0.001	-0.014	0.003	-0.028	-0.001	0.006	0.001	-0.031
77.H	-0.008	-0.001	-0.013	0.010	-0.023	-0.009	0.007	0.000	-0.028
78.H	-0.007	-0.001	-0.016	0.003	-0.026	-0.002	0.010	0.001	-0.033
79.H	-0.008	-0.001	-0.011	-0.009	-0.018	0.008	0.012	-0.001	-0.031
80.H	-0.003	-0.001	-0.009	-0.010	-0.007	0.006	0.016	-0.004	-0.029
81.H	-0.003	-0.001	-0.014	-0.008	-0.017	0.007	0.015	-0.002	-0.033
82.H	-0.010	-0.000	0.002	0.001	-0.001	-0.006	0.008	-0.005	-0.017
83.H	-0.015	-0.000	0.008	0.008	-0.002	-0.012	0.002	-0.004	-0.012
84.H	-0.013	-0.000	-0.006	0.007	-0.020	-0.007	0.004	-0.000	-0.024
85.H	-0.005	-0.001	-0.004	-0.002	-0.003	-0.003	0.013	-0.004	-0.023
86.H	-0.026	0.000	0.001	-0.012	-0.027	0.013	-0.002	0.002	-0.023
87.O	0.000	0.000	0.000	0.000	0.000	0.000	0.000	0.000	0.000
88.H	-0.021	0.000	0.014	-0.009	0.000	0.004	0.001	-0.004	-0.010
89.H	-0.020	-0.000	-0.009	-0.010	-0.033	0.013	0.002	0.003	-0.030
90.H	-0.015	-0.000	-0.006	-0.014	-0.022	0.013	0.007	0.000	-0.029
91.H	-0.020	-0.000	-0.003	-0.009	-0.024	0.009	0.002	0.001	-0.025
92.C	-0.018	-0.000	-0.006	-0.009	-0.026	0.010	0.004	0.001	-0.028
93.O	0.000	0.000	0.000	0.000	0.000	0.000	0.000	0.000	0.000
94.H	-0.028	0.001	0.014	0.004	-0.011	-0.007	-0.007	-0.001	-0.008

95.H	-0.024	0.001	0.018	0.002	0.001	-0.007	-0.004	-0.004	-0.005
96.H	-0.028	0.001	0.017	-0.003	-0.006	-0.001	-0.006	-0.003	-0.007

	103			104			105		
1.O	0.000	0.000	0.000	0.000	0.000	0.000	0.000	0.000	0.000
2.C	0.000	0.000	0.000	0.000	0.000	0.000	0.000	0.000	0.000
3.C	0.000	0.000	0.000	0.000	0.000	0.000	0.000	0.000	0.000
4.C	0.000	0.000	0.000	0.000	0.000	0.000	0.000	0.000	0.000
5.O	0.000	0.000	0.000	0.000	0.000	0.000	0.000	0.000	0.000
6.N	-0.003	0.006	-0.016	-0.000	-0.009	-0.003	0.004	0.006	-0.027
7.O	0.000	0.000	0.000	0.000	0.000	0.000	0.000	0.000	0.000
8.C	0.000	0.000	0.000	0.000	0.000	0.000	0.000	0.000	0.000
9.C	0.000	0.000	0.000	0.000	0.000	0.000	0.000	0.000	0.000
10.C	0.000	0.000	0.000	0.000	0.000	0.000	0.000	0.000	0.000
11.O	0.000	0.000	0.000	0.000	0.000	0.000	0.000	0.000	0.000
12.C	0.000	0.000	0.000	0.000	0.000	0.000	0.000	0.000	0.000
13.O	0.000	0.000	0.000	0.000	0.000	0.000	0.000	0.000	0.000
14.C	0.000	0.000	0.000	0.000	0.000	0.000	0.000	0.000	0.000
15.C	0.000	0.000	0.000	0.000	0.000	0.000	0.000	0.000	0.000
16.O	0.000	0.000	0.000	0.000	0.000	0.000	0.000	0.000	0.000
17.C	0.000	0.000	0.000	0.000	0.000	0.000	0.000	0.000	0.000
18.C	0.000	0.000	0.000	0.000	0.000	0.000	0.000	0.000	0.000
19.O	0.000	0.000	0.000	0.000	0.000	0.000	0.000	0.000	0.000
20.C	0.000	0.000	0.000	0.000	0.000	0.000	0.000	0.000	0.000
21.C	0.000	0.000	0.000	0.000	0.000	0.000	0.000	0.000	0.000
22.C	0.000	0.000	0.000	0.000	0.000	0.000	0.000	0.000	0.000
23.C	0.000	0.000	0.000	0.000	0.000	0.000	0.000	0.000	0.000
24.C	0.000	0.000	0.000	0.000	0.000	0.000	0.000	0.000	0.000
25.C	0.000	0.000	0.000	0.000	0.000	0.000	0.000	0.000	0.000

26.C	0.000	0.000	0.000	0.000	0.000	0.000	0.000	0.000	0.000
27.C	0.000	0.000	0.000	0.000	0.000	0.000	0.000	0.000	0.000
28.C	0.000	0.000	0.000	0.000	0.000	0.000	0.000	0.000	0.000
29.C	0.000	0.000	0.000	0.000	0.000	0.000	0.000	0.000	0.000
30.C	0.000	0.000	0.000	0.000	0.000	0.000	0.000	0.000	0.000
31.O	0.000	0.000	0.000	0.000	0.000	0.000	0.000	0.000	0.000
32.C	0.000	0.000	0.000	0.000	0.000	0.000	0.000	0.000	0.000
33.C	0.000	0.000	0.000	0.000	0.000	0.000	0.000	0.000	0.000
34.C	0.266	0.008	-0.002	0.000	0.267	-0.007	0.000	0.000	0.276
35.C	-0.038	-0.005	0.013	0.015	-0.018	-0.024	0.002	0.000	-0.029
36.C	-0.026	-0.003	0.005	-0.011	-0.035	0.022	-0.005	-0.006	-0.012
37.C	0.004	0.006	-0.020	-0.006	-0.009	0.006	0.003	0.007	-0.024
38.C	-0.039	-0.013	0.025	0.012	-0.014	-0.021	0.001	-0.000	-0.025
39.C	-0.025	-0.008	0.013	-0.012	-0.030	0.022	-0.006	-0.005	-0.011
40.H	-0.042	0.002	0.005	0.004	-0.041	0.001	-0.003	-0.008	-0.021
41.H	-0.033	0.013	-0.013	0.002	-0.044	0.004	-0.002	-0.006	-0.024
42.H	-0.031	0.013	-0.014	-0.002	-0.047	0.012	-0.003	-0.007	-0.021
43.H	-0.041	0.017	-0.016	0.004	-0.055	0.005	-0.003	-0.009	-0.024
44.H	-0.055	0.006	0.005	0.020	-0.042	-0.023	-0.000	-0.007	-0.030
45.H	-0.060	0.005	0.008	0.019	-0.047	-0.021	-0.002	-0.009	-0.029
46.H	-0.054	-0.001	0.014	0.019	-0.035	-0.025	-0.000	-0.006	-0.029
47.H	-0.046	-0.000	0.009	0.010	-0.036	-0.010	-0.002	-0.006	-0.024
48.H	-0.038	0.004	0.001	0.011	-0.031	-0.014	0.001	-0.003	-0.028
49.H	-0.033	0.002	0.001	0.006	-0.030	-0.005	-0.000	-0.003	-0.024
50.H	-0.035	-0.002	0.008	0.015	-0.019	-0.023	0.003	0.001	-0.030
51.H	-0.036	-0.009	0.018	0.015	-0.012	-0.026	0.003	0.002	-0.029
52.H	-0.045	-0.003	0.014	0.018	-0.024	-0.027	0.002	-0.002	-0.030
53.H	-0.044	-0.012	0.026	0.017	-0.016	-0.027	0.001	-0.001	-0.027
54.H	-0.033	-0.016	0.026	0.011	-0.007	-0.021	0.002	0.002	-0.024
55.H	-0.041	-0.015	0.028	0.012	-0.015	-0.019	0.000	-0.001	-0.023
56.H	-0.039	-0.001	0.008	-0.003	-0.042	0.011	-0.005	-0.008	-0.016

57.H	-0.028	0.000	0.001	-0.012	-0.042	0.026	-0.006	-0.008	-0.012
58.H	-0.019	-0.003	0.003	-0.013	-0.031	0.024	-0.005	-0.005	-0.012
59.H	-0.023	-0.012	0.016	-0.011	-0.024	0.018	-0.005	-0.004	-0.011
60.H	-0.032	-0.008	0.015	-0.009	-0.034	0.019	-0.006	-0.007	-0.011
61.H	-0.021	-0.009	0.012	-0.016	-0.029	0.028	-0.006	-0.005	-0.008
62.H	-0.034	0.009	-0.008	-0.008	-0.053	0.023	-0.006	-0.010	-0.015
63.H	-0.044	0.009	-0.004	-0.002	-0.056	0.014	-0.006	-0.012	-0.017
64.H	-0.038	0.013	-0.012	-0.004	-0.056	0.017	-0.005	-0.011	-0.018
65.H	-0.023	-0.004	0.006	-0.000	-0.020	0.000	-0.001	-0.000	-0.021
66.H	-0.007	-0.012	0.011	-0.004	-0.000	0.001	0.002	0.006	-0.019
67.H	-0.009	-0.005	0.002	-0.005	-0.009	0.004	0.001	0.004	-0.020
68.H	-0.008	-0.009	0.007	-0.008	-0.007	0.009	-0.000	0.003	-0.017
69.C	-0.016	0.007	-0.012	-0.002	-0.025	0.004	0.001	0.000	-0.023
70.H	-0.021	-0.005	0.006	0.016	-0.001	-0.030	0.006	0.007	-0.033
71.H	-0.011	-0.009	0.008	0.012	0.010	-0.028	0.007	0.011	-0.032
72.H	-0.018	-0.011	0.014	0.014	0.007	-0.030	0.006	0.009	-0.031
73.H	-0.007	-0.009	0.006	0.005	0.007	-0.015	0.005	0.009	-0.027
74.H	-0.014	0.002	-0.007	0.009	-0.007	-0.018	0.005	0.006	-0.031
75.H	-0.004	-0.002	-0.005	0.005	0.004	-0.015	0.006	0.010	-0.029
76.H	-0.013	0.013	-0.022	0.002	-0.024	-0.002	0.003	0.002	-0.028
77.H	-0.015	0.010	-0.018	0.007	-0.018	-0.011	0.004	0.004	-0.031
78.H	-0.007	0.014	-0.026	0.002	-0.019	-0.003	0.004	0.004	-0.029
79.H	0.001	0.008	-0.021	-0.007	-0.014	0.009	0.002	0.005	-0.023
80.H	0.009	0.002	-0.016	-0.008	-0.002	0.007	0.004	0.008	-0.023
81.H	0.007	0.009	-0.025	-0.007	-0.009	0.008	0.004	0.007	-0.025
82.H	-0.010	-0.007	0.004	0.001	-0.002	-0.007	0.003	0.006	-0.024
83.H	-0.022	-0.010	0.014	0.006	-0.006	-0.014	0.002	0.004	-0.024
84.H	-0.019	0.005	-0.009	0.005	-0.019	-0.009	0.003	0.002	-0.028
85.H	-0.000	-0.002	-0.006	-0.002	-0.000	-0.003	0.004	0.008	-0.025
86.H	-0.021	0.002	-0.003	-0.008	-0.032	0.016	-0.003	-0.004	-0.016
87.O	0.000	0.000	0.000	0.000	0.000	0.000	0.000	0.000	0.000

88.H	-0.018	-0.016	0.020	-0.006	-0.009	0.006	-0.002	0.001	-0.015
89.H	-0.015	0.011	-0.018	-0.007	-0.033	0.015	-0.001	-0.002	-0.020
90.H	-0.006	0.005	-0.014	-0.010	-0.021	0.016	-0.000	0.001	-0.019
91.H	-0.015	0.004	-0.008	-0.006	-0.026	0.011	-0.001	-0.001	-0.020
92.C	-0.013	0.007	-0.013	-0.007	-0.026	0.012	-0.001	-0.000	-0.020
93.O	0.000	0.000	0.000	0.000	0.000	0.000	0.000	0.000	0.000
94.H	-0.037	-0.012	0.023	0.005	-0.022	-0.006	-0.002	-0.003	-0.019
95.H	-0.030	-0.019	0.029	0.002	-0.010	-0.007	-0.001	0.000	-0.018
96.H	-0.032	-0.016	0.026	-0.001	-0.018	0.001	-0.003	-0.002	-0.016

	106			107			108		
1.O	0.000	0.000	0.000	0.000	0.000	0.000	0.000	0.000	0.000
2.C	0.000	0.000	0.000	0.000	0.000	0.000	0.000	0.000	0.000
3.C	0.000	0.000	0.000	0.000	0.000	0.000	0.000	0.000	0.000
4.C	0.000	0.000	0.000	0.000	0.000	0.000	0.000	0.000	0.000
5.O	0.000	0.000	0.000	0.000	0.000	0.000	0.000	0.000	0.000
6.N	-0.025	0.014	-0.005	-0.001	-0.018	-0.001	-0.007	0.017	-0.024
7.O	0.000	0.000	0.000	0.000	0.000	0.000	0.000	0.000	0.000
8.C	0.000	0.000	0.000	0.000	0.000	0.000	0.000	0.000	0.000
9.C	0.000	0.000	0.000	0.000	0.000	0.000	0.000	0.000	0.000
10.C	0.000	0.000	0.000	0.000	0.000	0.000	0.000	0.000	0.000
11.O	0.000	0.000	0.000	0.000	0.000	0.000	0.000	0.000	0.000
12.C	0.000	0.000	0.000	0.000	0.000	0.000	0.000	0.000	0.000
13.O	0.000	0.000	0.000	0.000	0.000	0.000	0.000	0.000	0.000
14.C	0.000	0.000	0.000	0.000	0.000	0.000	0.000	0.000	0.000
15.C	0.000	0.000	0.000	0.000	0.000	0.000	0.000	0.000	0.000
16.O	0.000	0.000	0.000	0.000	0.000	0.000	0.000	0.000	0.000
17.C	0.000	0.000	0.000	0.000	0.000	0.000	0.000	0.000	0.000
18.C	0.000	0.000	0.000	0.000	0.000	0.000	0.000	0.000	0.000

19.O	0.000	0.000	0.000	0.000	0.000	0.000	0.000	0.000	0.000
20.C	0.000	0.000	0.000	0.000	0.000	0.000	0.000	0.000	0.000
21.C	0.000	0.000	0.000	0.000	0.000	0.000	0.000	0.000	0.000
22.C	0.000	0.000	0.000	0.000	0.000	0.000	0.000	0.000	0.000
23.C	0.000	0.000	0.000	0.000	0.000	0.000	0.000	0.000	0.000
24.C	0.000	0.000	0.000	0.000	0.000	0.000	0.000	0.000	0.000
25.C	0.000	0.000	0.000	0.000	0.000	0.000	0.000	0.000	0.000
26.C	0.000	0.000	0.000	0.000	0.000	0.000	0.000	0.000	0.000
27.C	0.000	0.000	0.000	0.000	0.000	0.000	0.000	0.000	0.000
28.C	0.000	0.000	0.000	0.000	0.000	0.000	0.000	0.000	0.000
29.C	0.000	0.000	0.000	0.000	0.000	0.000	0.000	0.000	0.000
30.C	0.000	0.000	0.000	0.000	0.000	0.000	0.000	0.000	0.000
31.O	0.000	0.000	0.000	0.000	0.000	0.000	0.000	0.000	0.000
32.C	0.000	0.000	0.000	0.000	0.000	0.000	0.000	0.000	0.000
33.C	0.000	0.000	0.000	0.000	0.000	0.000	0.000	0.000	0.000
34.C	0.000	0.000	0.000	0.000	0.000	0.000	0.000	0.000	0.000
35.C	0.261	-0.009	0.015	0.000	0.276	0.002	0.000	0.000	0.261
36.C	-0.002	-0.005	-0.007	0.007	-0.027	-0.001	-0.003	-0.018	-0.003
37.C	-0.013	0.016	-0.009	0.002	-0.017	-0.002	-0.013	0.018	-0.013
38.C	-0.052	-0.026	0.018	-0.009	-0.032	0.003	0.017	0.006	-0.058
39.C	0.000	-0.017	-0.005	0.007	-0.031	0.000	-0.004	-0.013	-0.005
40.H	-0.034	0.004	0.002	-0.003	-0.025	0.000	0.013	-0.026	-0.027
41.H	-0.030	0.027	-0.006	-0.002	-0.017	-0.001	0.008	-0.026	-0.017
42.H	-0.020	0.027	-0.010	0.001	-0.017	-0.002	0.004	-0.031	-0.007
43.H	-0.033	0.036	-0.008	-0.003	-0.015	-0.002	0.013	-0.040	-0.014
44.H	-0.067	0.013	0.012	-0.013	-0.022	0.002	0.029	-0.025	-0.054
45.H	-0.066	0.011	0.012	-0.012	-0.023	0.002	0.031	-0.032	-0.053
46.H	-0.066	-0.001	0.016	-0.012	-0.026	0.003	0.028	-0.018	-0.059
47.H	-0.047	0.000	0.008	-0.007	-0.025	0.001	0.019	-0.019	-0.041
48.H	-0.050	0.009	0.006	-0.008	-0.022	0.001	0.016	-0.012	-0.042
49.H	-0.037	0.005	0.003	-0.004	-0.023	0.000	0.010	-0.010	-0.033

50.H	-0.057	-0.003	0.013	-0.010	-0.025	0.002	0.017	0.003	-0.055
51.H	-0.058	-0.017	0.018	-0.011	-0.029	0.003	0.018	0.011	-0.062
52.H	-0.065	-0.006	0.017	-0.012	-0.026	0.003	0.024	-0.004	-0.062
53.H	-0.061	-0.024	0.021	-0.011	-0.032	0.004	0.022	0.004	-0.065
54.H	-0.049	-0.032	0.019	-0.008	-0.034	0.004	0.014	0.015	-0.058
55.H	-0.050	-0.030	0.019	-0.008	-0.034	0.004	0.018	0.005	-0.057
56.H	-0.019	-0.002	-0.002	0.002	-0.027	0.000	0.008	-0.027	-0.015
57.H	0.001	0.001	-0.010	0.008	-0.026	-0.001	-0.003	-0.026	0.003
58.H	0.003	-0.005	-0.009	0.008	-0.027	-0.001	-0.008	-0.013	0.001
59.H	-0.002	-0.024	-0.002	0.007	-0.033	0.001	-0.005	-0.005	-0.010
60.H	-0.005	-0.017	-0.003	0.006	-0.031	0.001	0.000	-0.018	-0.009
61.H	0.009	-0.019	-0.007	0.010	-0.031	-0.000	-0.009	-0.012	0.002
62.H	-0.007	0.018	-0.013	0.005	-0.021	-0.002	0.002	-0.038	0.003
63.H	-0.021	0.019	-0.007	0.001	-0.021	-0.001	0.011	-0.043	-0.008
64.H	-0.017	0.027	-0.011	0.002	-0.018	-0.002	0.007	-0.042	-0.003
65.H	-0.025	-0.008	0.002	-0.001	-0.027	0.001	0.002	0.001	-0.027
66.H	-0.016	-0.024	0.004	0.002	-0.030	0.001	-0.007	0.025	-0.029
67.H	-0.015	-0.009	-0.001	0.002	-0.026	-0.000	-0.007	0.014	-0.021
68.H	-0.008	-0.018	-0.001	0.004	-0.029	0.000	-0.009	0.016	-0.018
69.C	-0.022	0.016	-0.006	0.000	-0.019	-0.001	-0.002	-0.003	-0.017
70.H	-0.059	-0.007	0.015	-0.012	-0.024	0.002	0.012	0.025	-0.063
71.H	-0.052	-0.016	0.015	-0.010	-0.026	0.002	0.005	0.039	-0.061
72.H	-0.055	-0.021	0.018	-0.010	-0.028	0.003	0.009	0.034	-0.064
73.H	-0.035	-0.017	0.009	-0.005	-0.027	0.001	-0.002	0.035	-0.046
74.H	-0.045	0.007	0.006	-0.007	-0.020	0.000	0.004	0.019	-0.045
75.H	-0.036	-0.002	0.005	-0.005	-0.022	0.000	-0.003	0.032	-0.041
76.H	-0.030	0.028	-0.007	-0.003	-0.014	-0.002	-0.001	-0.000	-0.021
77.H	-0.040	0.024	-0.002	-0.006	-0.015	-0.001	0.003	0.007	-0.033
78.H	-0.029	0.031	-0.008	-0.003	-0.013	-0.002	-0.004	0.006	-0.021
79.H	-0.010	0.018	-0.011	0.003	-0.017	-0.002	-0.013	0.012	-0.008
80.H	-0.008	0.008	-0.009	0.003	-0.019	-0.002	-0.017	0.026	-0.012

81.H	-0.011	0.022	-0.012	0.003	-0.015	-0.003	-0.015	0.017	-0.009
82.H	-0.027	-0.012	0.005	-0.002	-0.026	0.001	-0.003	0.023	-0.035
83.H	-0.038	-0.020	0.011	-0.005	-0.029	0.002	0.006	0.017	-0.046
84.H	-0.037	0.012	0.001	-0.005	-0.019	-0.000	0.004	0.005	-0.033
85.H	-0.021	-0.002	-0.001	-0.000	-0.022	-0.000	-0.009	0.027	-0.027
86.H	-0.008	0.004	-0.008	0.005	-0.024	-0.001	-0.004	-0.013	-0.006
87.O	0.000	0.000	0.000	0.000	0.000	0.000	0.000	0.000	0.000
88.H	-0.013	-0.032	0.005	0.003	-0.034	0.002	-0.004	0.013	-0.026
89.H	-0.011	0.023	-0.013	0.004	-0.017	-0.002	-0.006	-0.013	-0.003
90.H	-0.004	0.012	-0.012	0.005	-0.020	-0.002	-0.012	0.001	-0.003
91.H	-0.012	0.009	-0.008	0.003	-0.021	-0.001	-0.005	-0.005	-0.010
92.C	-0.011	0.015	-0.010	0.003	-0.019	-0.002	-0.006	-0.005	-0.007
93.O	0.000	0.000	0.000	0.000	0.000	0.000	0.000	0.000	0.000
94.H	-0.035	-0.025	0.011	-0.003	-0.033	0.003	0.011	-0.003	-0.041
95.H	-0.030	-0.039	0.014	-0.002	-0.036	0.003	0.007	0.011	-0.043
96.H	-0.023	-0.032	0.009	0.000	-0.035	0.002	0.006	0.001	-0.033

	109			110			111		
1.O	0.000	0.000	0.000	0.000	0.000	0.000	0.000	0.000	0.000
2.C	0.000	0.000	0.000	0.000	0.000	0.000	0.000	0.000	0.000
3.C	0.000	0.000	0.000	0.000	0.000	0.000	0.000	0.000	0.000
4.C	0.000	0.000	0.000	0.000	0.000	0.000	0.000	0.000	0.000
5.O	0.000	0.000	0.000	0.000	0.000	0.000	0.000	0.000	0.000
6.N	-0.001	-0.012	-0.016	-0.003	0.000	-0.008	-0.012	-0.019	-0.009
7.O	0.000	0.000	0.000	0.000	0.000	0.000	0.000	0.000	0.000
8.C	0.000	0.000	0.000	0.000	0.000	0.000	0.000	0.000	0.000
9.C	0.000	0.000	0.000	0.000	0.000	0.000	0.000	0.000	0.000
10.C	0.000	0.000	0.000	0.000	0.000	0.000	0.000	0.000	0.000
11.O	0.000	0.000	0.000	0.000	0.000	0.000	0.000	0.000	0.000

12.C	0.000	0.000	0.000	0.000	0.000	0.000	0.000	0.000	0.000
13.O	0.000	0.000	0.000	0.000	0.000	0.000	0.000	0.000	0.000
14.C	0.000	0.000	0.000	0.000	0.000	0.000	0.000	0.000	0.000
15.C	0.000	0.000	0.000	0.000	0.000	0.000	0.000	0.000	0.000
16.O	0.000	0.000	0.000	0.000	0.000	0.000	0.000	0.000	0.000
17.C	0.000	0.000	0.000	0.000	0.000	0.000	0.000	0.000	0.000
18.C	0.000	0.000	0.000	0.000	0.000	0.000	0.000	0.000	0.000
19.O	0.000	0.000	0.000	0.000	0.000	0.000	0.000	0.000	0.000
20.C	0.000	0.000	0.000	0.000	0.000	0.000	0.000	0.000	0.000
21.C	0.000	0.000	0.000	0.000	0.000	0.000	0.000	0.000	0.000
22.C	0.000	0.000	0.000	0.000	0.000	0.000	0.000	0.000	0.000
23.C	0.000	0.000	0.000	0.000	0.000	0.000	0.000	0.000	0.000
24.C	0.000	0.000	0.000	0.000	0.000	0.000	0.000	0.000	0.000
25.C	0.000	0.000	0.000	0.000	0.000	0.000	0.000	0.000	0.000
26.C	0.000	0.000	0.000	0.000	0.000	0.000	0.000	0.000	0.000
27.C	0.000	0.000	0.000	0.000	0.000	0.000	0.000	0.000	0.000
28.C	0.000	0.000	0.000	0.000	0.000	0.000	0.000	0.000	0.000
29.C	0.000	0.000	0.000	0.000	0.000	0.000	0.000	0.000	0.000
30.C	0.000	0.000	0.000	0.000	0.000	0.000	0.000	0.000	0.000
31.O	0.000	0.000	0.000	0.000	0.000	0.000	0.000	0.000	0.000
32.C	0.000	0.000	0.000	0.000	0.000	0.000	0.000	0.000	0.000
33.C	0.000	0.000	0.000	0.000	0.000	0.000	0.000	0.000	0.000
34.C	0.000	0.000	0.000	0.000	0.000	0.000	0.000	0.000	0.000
35.C	0.000	0.000	0.000	0.000	0.000	0.000	0.000	0.000	0.000
36.C	0.257	0.006	0.017	0.000	0.265	0.019	0.000	0.000	0.261
37.C	-0.010	-0.013	-0.014	-0.000	0.002	-0.000	-0.009	-0.020	-0.018
38.C	-0.012	0.022	0.008	-0.005	-0.040	-0.017	-0.003	0.001	-0.014
39.C	-0.069	0.015	0.023	0.008	-0.053	0.020	0.021	0.020	-0.064
40.H	-0.043	-0.002	0.004	0.001	-0.051	-0.001	0.011	0.028	-0.029
41.H	-0.035	-0.021	-0.010	0.001	-0.035	-0.001	0.006	0.024	-0.020
42.H	-0.048	-0.021	-0.006	0.003	-0.039	0.007	0.012	0.029	-0.031

43.H	-0.042	-0.027	-0.011	0.001	-0.043	-0.000	0.011	0.036	-0.020
44.H	-0.012	-0.009	-0.011	-0.006	-0.044	-0.023	-0.002	0.026	0.004
45.H	-0.021	-0.007	-0.006	-0.005	-0.053	-0.020	0.003	0.034	-0.002
46.H	-0.013	0.002	-0.004	-0.006	-0.047	-0.023	-0.001	0.021	-0.001
47.H	-0.029	0.001	0.001	-0.002	-0.047	-0.010	0.005	0.022	-0.017
48.H	-0.014	-0.007	-0.009	-0.004	-0.033	-0.015	-0.002	0.013	-0.006
49.H	-0.025	-0.003	-0.003	-0.001	-0.035	-0.007	0.002	0.012	-0.018
50.H	0.001	0.003	-0.008	-0.007	-0.027	-0.023	-0.010	-0.001	0.002
51.H	0.002	0.015	-0.002	-0.007	-0.029	-0.024	-0.010	-0.005	-0.001
52.H	-0.001	0.006	-0.006	-0.007	-0.036	-0.025	-0.008	0.007	0.003
53.H	-0.006	0.020	0.005	-0.006	-0.041	-0.023	-0.006	0.003	-0.006
54.H	-0.008	0.026	0.009	-0.005	-0.035	-0.017	-0.006	-0.007	-0.015
55.H	-0.017	0.025	0.011	-0.004	-0.044	-0.015	-0.001	0.004	-0.019
56.H	-0.060	0.003	0.013	0.005	-0.056	0.010	0.019	0.031	-0.046
57.H	-0.073	0.001	0.016	0.008	-0.053	0.022	0.023	0.029	-0.061
58.H	-0.065	0.006	0.016	0.008	-0.045	0.021	0.019	0.017	-0.060
59.H	-0.063	0.021	0.024	0.007	-0.050	0.017	0.018	0.013	-0.062
60.H	-0.070	0.016	0.024	0.007	-0.059	0.018	0.023	0.025	-0.063
61.H	-0.075	0.017	0.026	0.010	-0.053	0.026	0.024	0.019	-0.072
62.H	-0.071	-0.013	0.007	0.007	-0.054	0.018	0.023	0.039	-0.051
63.H	-0.064	-0.013	0.005	0.005	-0.058	0.010	0.021	0.044	-0.041
64.H	-0.062	-0.020	-0.000	0.005	-0.051	0.011	0.019	0.041	-0.039
65.H	-0.030	0.007	0.005	0.001	-0.032	-0.001	0.003	0.003	-0.030
66.H	-0.020	0.019	0.009	0.000	-0.019	0.000	-0.003	-0.019	-0.034
67.H	-0.024	0.007	0.003	0.001	-0.020	0.002	-0.001	-0.011	-0.032
68.H	-0.033	0.015	0.011	0.003	-0.024	0.007	0.003	-0.011	-0.042
69.C	-0.024	-0.012	-0.009	0.000	-0.019	-0.000	-0.001	0.002	-0.021
70.H	0.024	0.005	-0.014	-0.009	-0.007	-0.029	-0.022	-0.023	0.013
71.H	0.027	0.012	-0.011	-0.009	0.001	-0.027	-0.024	-0.035	0.009
72.H	0.022	0.017	-0.007	-0.009	-0.008	-0.027	-0.021	-0.030	0.006
73.H	0.009	0.013	-0.005	-0.005	-0.004	-0.016	-0.017	-0.031	-0.008

74.H	0.013	-0.006	-0.017	-0.007	-0.004	-0.020	-0.018	-0.019	0.006
75.H	0.015	0.000	-0.014	-0.006	0.004	-0.017	-0.020	-0.031	0.000
76.H	-0.008	-0.023	-0.020	-0.002	-0.008	-0.008	-0.008	-0.004	-0.006
77.H	0.005	-0.019	-0.023	-0.005	-0.004	-0.016	-0.014	-0.010	0.004
78.H	-0.000	-0.026	-0.025	-0.003	0.001	-0.010	-0.013	-0.011	-0.001
79.H	-0.017	-0.015	-0.013	0.001	-0.003	0.003	-0.006	-0.014	-0.022
80.H	-0.009	-0.007	-0.010	0.000	0.004	0.002	-0.010	-0.027	-0.022
81.H	-0.008	-0.018	-0.018	-0.000	0.006	0.000	-0.010	-0.021	-0.016
82.H	-0.007	0.010	-0.001	-0.002	-0.013	-0.008	-0.009	-0.019	-0.019
83.H	-0.008	0.017	0.003	-0.003	-0.025	-0.012	-0.007	-0.012	-0.016
84.H	-0.006	-0.010	-0.013	-0.004	-0.014	-0.012	-0.009	-0.005	-0.006
85.H	-0.003	0.001	-0.008	-0.002	-0.002	-0.006	-0.012	-0.026	-0.016
86.H	-0.052	-0.002	0.007	0.005	-0.038	0.013	0.013	0.015	-0.045
87.O	0.000	0.000	0.000	0.000	0.000	0.000	0.000	0.000	0.000
88.H	-0.039	0.027	0.020	0.003	-0.038	0.007	0.007	-0.005	-0.048
89.H	-0.039	-0.018	-0.007	0.003	-0.024	0.009	0.006	0.011	-0.032
90.H	-0.035	-0.009	-0.003	0.004	-0.018	0.010	0.004	-0.002	-0.036
91.H	-0.037	-0.007	-0.001	0.003	-0.026	0.007	0.005	0.005	-0.034
92.C	-0.034	-0.012	-0.005	0.003	-0.022	0.007	0.004	0.004	-0.032
93.O	0.000	0.000	0.000	0.000	0.000	0.000	0.000	0.000	0.000
94.H	-0.034	0.021	0.015	-0.000	-0.048	-0.004	0.007	0.011	-0.033
95.H	-0.031	0.032	0.020	0.000	-0.045	-0.003	0.004	-0.001	-0.037
96.H	-0.043	0.027	0.022	0.002	-0.050	0.003	0.010	0.008	-0.045

	112			113			114		
1.O	0.000	0.000	0.000	0.000	0.000	0.000	0.000	0.000	0.000
2.C	0.000	0.000	0.000	0.000	0.000	0.000	0.000	0.000	0.000
3.C	0.000	0.000	0.000	0.000	0.000	0.000	0.000	0.000	0.000
4.C	0.000	0.000	0.000	0.000	0.000	0.000	0.000	0.000	0.000

5.O	0.000	0.000	0.000	0.000	0.000	0.000	0.000	0.000	0.000
6.N	-0.054	-0.002	0.020	0.003	-0.067	0.009	0.024	0.001	-0.047
7.O	0.000	0.000	0.000	0.000	0.000	0.000	0.000	0.000	0.000
8.C	0.000	0.000	0.000	0.000	0.000	0.000	0.000	0.000	0.000
9.C	0.000	0.000	0.000	0.000	0.000	0.000	0.000	0.000	0.000
10.C	0.000	0.000	0.000	0.000	0.000	0.000	0.000	0.000	0.000
11.O	0.000	0.000	0.000	0.000	0.000	0.000	0.000	0.000	0.000
12.C	0.000	0.000	0.000	0.000	0.000	0.000	0.000	0.000	0.000
13.O	0.000	0.000	0.000	0.000	0.000	0.000	0.000	0.000	0.000
14.C	0.000	0.000	0.000	0.000	0.000	0.000	0.000	0.000	0.000
15.C	0.000	0.000	0.000	0.000	0.000	0.000	0.000	0.000	0.000
16.O	0.000	0.000	0.000	0.000	0.000	0.000	0.000	0.000	0.000
17.C	0.000	0.000	0.000	0.000	0.000	0.000	0.000	0.000	0.000
18.C	0.000	0.000	0.000	0.000	0.000	0.000	0.000	0.000	0.000
19.O	0.000	0.000	0.000	0.000	0.000	0.000	0.000	0.000	0.000
20.C	0.000	0.000	0.000	0.000	0.000	0.000	0.000	0.000	0.000
21.C	0.000	0.000	0.000	0.000	0.000	0.000	0.000	0.000	0.000
22.C	0.000	0.000	0.000	0.000	0.000	0.000	0.000	0.000	0.000
23.C	0.000	0.000	0.000	0.000	0.000	0.000	0.000	0.000	0.000
24.C	0.000	0.000	0.000	0.000	0.000	0.000	0.000	0.000	0.000
25.C	0.000	0.000	0.000	0.000	0.000	0.000	0.000	0.000	0.000
26.C	0.000	0.000	0.000	0.000	0.000	0.000	0.000	0.000	0.000
27.C	0.000	0.000	0.000	0.000	0.000	0.000	0.000	0.000	0.000
28.C	0.000	0.000	0.000	0.000	0.000	0.000	0.000	0.000	0.000
29.C	0.000	0.000	0.000	0.000	0.000	0.000	0.000	0.000	0.000
30.C	0.000	0.000	0.000	0.000	0.000	0.000	0.000	0.000	0.000
31.O	0.000	0.000	0.000	0.000	0.000	0.000	0.000	0.000	0.000
32.C	0.000	0.000	0.000	0.000	0.000	0.000	0.000	0.000	0.000
33.C	0.000	0.000	0.000	0.000	0.000	0.000	0.000	0.000	0.000
34.C	0.000	0.000	0.000	0.000	0.000	0.000	0.000	0.000	0.000
35.C	0.000	0.000	0.000	0.000	0.000	0.000	0.000	0.000	0.000

36.C	0.000	0.000	0.000	0.000	0.000	0.000	0.000	0.000	0.000
37.C	0.260	-0.003	0.024	0.000	0.256	0.001	0.000	0.000	0.264
38.C	-0.011	0.008	-0.032	0.013	-0.004	0.015	-0.014	-0.006	-0.000
39.C	-0.011	0.004	-0.022	-0.016	0.011	-0.028	-0.015	-0.002	-0.012
40.H	0.001	-0.003	-0.009	0.001	0.002	-0.002	-0.025	0.004	-0.022
41.H	-0.012	-0.009	0.016	0.001	-0.025	0.001	-0.013	0.009	-0.044
42.H	-0.010	-0.010	0.016	-0.005	-0.019	-0.008	-0.015	0.009	-0.044
43.H	-0.001	-0.013	0.019	0.001	-0.017	0.000	-0.023	0.012	-0.047
44.H	0.007	-0.005	-0.006	0.019	-0.009	0.024	-0.029	0.006	-0.024
45.H	0.015	-0.005	-0.010	0.018	0.003	0.020	-0.037	0.006	-0.020
46.H	0.006	-0.001	-0.018	0.019	-0.002	0.023	-0.029	0.002	-0.013
47.H	0.001	-0.001	-0.013	0.009	-0.002	0.009	-0.024	0.002	-0.018
48.H	-0.011	-0.003	-0.001	0.012	-0.022	0.016	-0.013	0.003	-0.028
49.H	-0.015	-0.002	-0.003	0.005	-0.019	0.006	-0.011	0.002	-0.027
50.H	-0.019	0.001	-0.010	0.017	-0.027	0.023	-0.007	-0.001	-0.020
51.H	-0.019	0.006	-0.023	0.017	-0.020	0.023	-0.007	-0.005	-0.009
52.H	-0.007	0.002	-0.017	0.020	-0.015	0.026	-0.017	-0.001	-0.014
53.H	-0.007	0.007	-0.033	0.017	-0.003	0.021	-0.017	-0.005	0.000
54.H	-0.019	0.010	-0.034	0.012	-0.009	0.014	-0.007	-0.008	0.002
55.H	-0.008	0.009	-0.037	0.012	0.003	0.012	-0.017	-0.007	0.004
56.H	0.002	-0.001	-0.014	-0.007	0.011	-0.015	-0.026	0.003	-0.018
57.H	-0.007	-0.002	-0.007	-0.017	0.006	-0.028	-0.018	0.003	-0.025
58.H	-0.019	0.001	-0.008	-0.017	-0.003	-0.027	-0.008	0.001	-0.023
59.H	-0.015	0.006	-0.026	-0.015	0.009	-0.025	-0.011	-0.004	-0.007
60.H	-0.004	0.004	-0.025	-0.014	0.018	-0.026	-0.021	-0.001	-0.009
61.H	-0.014	0.005	-0.021	-0.021	0.011	-0.034	-0.012	-0.002	-0.012
62.H	-0.001	-0.008	0.005	-0.013	0.002	-0.023	-0.024	0.008	-0.035
63.H	0.009	-0.008	0.001	-0.007	0.008	-0.014	-0.032	0.009	-0.031
64.H	0.002	-0.010	0.012	-0.008	-0.003	-0.014	-0.026	0.010	-0.041
65.H	-0.024	0.002	-0.010	-0.001	-0.019	-0.002	-0.003	-0.002	-0.021
66.H	-0.045	0.009	-0.016	-0.003	-0.031	-0.004	0.015	-0.008	-0.015

67.H	-0.041	0.004	-0.005	-0.004	-0.035	-0.005	0.012	-0.003	-0.026
68.H	-0.040	0.006	-0.013	-0.008	-0.026	-0.012	0.011	-0.005	-0.019
69.C	-0.034	-0.004	0.014	-0.001	-0.042	-0.000	0.006	0.003	-0.042
70.H	-0.041	0.004	-0.006	0.020	-0.052	0.032	0.013	-0.004	-0.023
71.H	-0.053	0.008	-0.009	0.018	-0.059	0.029	0.024	-0.008	-0.021
72.H	-0.044	0.009	-0.017	0.018	-0.047	0.028	0.016	-0.008	-0.014
73.H	-0.053	0.008	-0.009	0.008	-0.053	0.015	0.024	-0.008	-0.021
74.H	-0.046	-0.000	0.009	0.013	-0.060	0.023	0.017	-0.001	-0.037
75.H	-0.058	0.003	0.007	0.010	-0.067	0.019	0.028	-0.004	-0.035
76.H	-0.041	-0.008	0.027	0.004	-0.060	0.010	0.013	0.006	-0.054
77.H	-0.043	-0.006	0.024	0.010	-0.064	0.019	0.015	0.004	-0.050
78.H	-0.050	-0.008	0.033	0.005	-0.072	0.013	0.021	0.006	-0.059
79.H	-0.055	-0.004	0.025	-0.005	-0.064	-0.003	0.025	0.002	-0.052
80.H	-0.065	0.000	0.019	-0.005	-0.070	-0.002	0.034	-0.002	-0.047
81.H	-0.063	-0.005	0.031	-0.004	-0.076	0.001	0.032	0.002	-0.057
82.H	-0.045	0.005	-0.007	0.003	-0.042	0.006	0.016	-0.005	-0.023
83.H	-0.031	0.007	-0.020	0.008	-0.025	0.010	0.004	-0.006	-0.012
84.H	-0.035	-0.003	0.011	0.007	-0.047	0.013	0.008	0.002	-0.039
85.H	-0.057	0.003	0.007	0.001	-0.060	0.006	0.027	-0.004	-0.035
86.H	-0.020	-0.002	-0.000	-0.011	-0.014	-0.017	-0.007	0.002	-0.030
87.O	0.000	0.000	0.000	0.000	0.000	0.000	0.000	0.000	0.000
88.H	-0.028	0.010	-0.030	-0.007	-0.006	-0.013	0.000	-0.008	-0.004
89.H	-0.031	-0.007	0.020	-0.008	-0.038	-0.010	0.003	0.006	-0.048
90.H	-0.042	-0.003	0.015	-0.010	-0.043	-0.012	0.013	0.002	-0.043
91.H	-0.031	-0.003	0.008	-0.007	-0.031	-0.009	0.003	0.002	-0.037
92.C	-0.035	-0.004	0.014	-0.007	-0.038	-0.008	0.006	0.004	-0.043
93.O	0.000	0.000	0.000	0.000	0.000	0.000	0.000	0.000	0.000
94.H	-0.007	0.007	-0.032	0.003	0.007	-0.001	-0.018	-0.005	-0.001
95.H	-0.016	0.012	-0.041	0.001	0.005	-0.002	-0.010	-0.009	0.006
96.H	-0.011	0.009	-0.037	-0.003	0.011	-0.009	-0.015	-0.007	0.003

	115			116			117		
	------------------------			------------------------			------------------------		
1.O	0.000	0.000	0.000	0.000	0.000	0.000	0.000	0.000	0.000
2.C	0.000	0.000	0.000	0.000	0.000	0.000	0.000	0.000	0.000
3.C	0.000	0.000	0.000	0.000	0.000	0.000	0.000	0.000	0.000
4.C	0.000	0.000	0.000	0.000	0.000	0.000	0.000	0.000	0.000
5.O	0.000	0.000	0.000	0.000	0.000	0.000	0.000	0.000	0.000
6.N	-0.025	0.015	-0.011	-0.005	-0.005	-0.007	-0.030	0.018	-0.013
7.O	0.000	0.000	0.000	0.000	0.000	0.000	0.000	0.000	0.000
8.C	0.000	0.000	0.000	0.000	0.000	0.000	0.000	0.000	0.000
9.C	0.000	0.000	0.000	0.000	0.000	0.000	0.000	0.000	0.000
10.C	0.000	0.000	0.000	0.000	0.000	0.000	0.000	0.000	0.000
11.O	0.000	0.000	0.000	0.000	0.000	0.000	0.000	0.000	0.000
12.C	0.000	0.000	0.000	0.000	0.000	0.000	0.000	0.000	0.000
13.O	0.000	0.000	0.000	0.000	0.000	0.000	0.000	0.000	0.000
14.C	0.000	0.000	0.000	0.000	0.000	0.000	0.000	0.000	0.000
15.C	0.000	0.000	0.000	0.000	0.000	0.000	0.000	0.000	0.000
16.O	0.000	0.000	0.000	0.000	0.000	0.000	0.000	0.000	0.000
17.C	0.000	0.000	0.000	0.000	0.000	0.000	0.000	0.000	0.000
18.C	0.000	0.000	0.000	0.000	0.000	0.000	0.000	0.000	0.000
19.O	0.000	0.000	0.000	0.000	0.000	0.000	0.000	0.000	0.000
20.C	0.000	0.000	0.000	0.000	0.000	0.000	0.000	0.000	0.000
21.C	0.000	0.000	0.000	0.000	0.000	0.000	0.000	0.000	0.000
22.C	0.000	0.000	0.000	0.000	0.000	0.000	0.000	0.000	0.000
23.C	0.000	0.000	0.000	0.000	0.000	0.000	0.000	0.000	0.000
24.C	0.000	0.000	0.000	0.000	0.000	0.000	0.000	0.000	0.000
25.C	0.000	0.000	0.000	0.000	0.000	0.000	0.000	0.000	0.000
26.C	0.000	0.000	0.000	0.000	0.000	0.000	0.000	0.000	0.000
27.C	0.000	0.000	0.000	0.000	0.000	0.000	0.000	0.000	0.000
28.C	0.000	0.000	0.000	0.000	0.000	0.000	0.000	0.000	0.000

29.C	0.000	0.000	0.000	0.000	0.000	0.000	0.000	0.000	0.000
30.C	0.000	0.000	0.000	0.000	0.000	0.000	0.000	0.000	0.000
31.O	0.000	0.000	0.000	0.000	0.000	0.000	0.000	0.000	0.000
32.C	0.000	0.000	0.000	0.000	0.000	0.000	0.000	0.000	0.000
33.C	0.000	0.000	0.000	0.000	0.000	0.000	0.000	0.000	0.000
34.C	0.000	0.000	0.000	0.000	0.000	0.000	0.000	0.000	0.000
35.C	0.000	0.000	0.000	0.000	0.000	0.000	0.000	0.000	0.000
36.C	0.000	0.000	0.000	0.000	0.000	0.000	0.000	0.000	0.000
37.C	0.000	0.000	0.000	0.000	0.000	0.000	0.000	0.000	0.000
38.C	0.259	-0.027	0.025	0.000	0.257	0.012	0.000	0.000	0.244
39.C	-0.013	-0.018	0.001	0.029	-0.064	0.004	0.010	-0.009	-0.029
40.H	-0.049	0.005	0.003	-0.011	-0.039	0.002	0.041	-0.029	-0.043
41.H	-0.042	0.029	-0.011	-0.008	-0.007	-0.006	0.024	-0.036	-0.010
42.H	-0.033	0.029	-0.015	0.005	-0.008	-0.007	0.021	-0.042	0.001
43.H	-0.048	0.038	-0.014	-0.010	-0.001	-0.007	0.039	-0.055	-0.004
44.H	-0.083	0.014	0.012	-0.051	-0.026	0.004	0.064	-0.031	-0.070
45.H	-0.084	0.012	0.014	-0.049	-0.033	0.005	0.073	-0.039	-0.073
46.H	-0.082	-0.001	0.019	-0.050	-0.043	0.008	0.063	-0.019	-0.085
47.H	-0.061	0.001	0.010	-0.027	-0.042	0.005	0.047	-0.021	-0.062
48.H	-0.061	0.010	0.006	-0.032	-0.026	0.001	0.034	-0.015	-0.053
49.H	-0.048	0.005	0.003	-0.017	-0.031	0.001	0.024	-0.012	-0.044
50.H	-0.066	-0.004	0.015	-0.041	-0.037	0.005	0.029	0.006	-0.076
51.H	-0.068	-0.018	0.023	-0.043	-0.054	0.009	0.029	0.019	-0.094
52.H	-0.077	-0.006	0.020	-0.050	-0.044	0.008	0.046	-0.001	-0.089
53.H	-0.073	-0.025	0.028	-0.045	-0.066	0.013	0.045	0.013	-0.106
54.H	-0.058	-0.033	0.026	-0.031	-0.072	0.013	0.025	0.028	-0.100
55.H	-0.063	-0.032	0.027	-0.032	-0.075	0.013	0.038	0.015	-0.101
56.H	-0.035	-0.001	0.001	0.007	-0.048	0.002	0.035	-0.029	-0.034
57.H	-0.013	0.002	-0.009	0.030	-0.044	-0.001	0.015	-0.029	-0.008
58.H	-0.008	-0.005	-0.007	0.032	-0.047	-0.001	0.000	-0.012	-0.011
59.H	-0.014	-0.025	0.005	0.026	-0.070	0.005	0.006	0.002	-0.039

60.H	-0.019	-0.018	0.003	0.024	-0.066	0.005	0.022	-0.015	-0.036
61.H	-0.003	-0.019	-0.002	0.040	-0.065	0.003	0.002	-0.007	-0.021
62.H	-0.023	0.020	-0.014	0.021	-0.025	-0.005	0.026	-0.048	0.004
63.H	-0.039	0.020	-0.008	0.005	-0.027	-0.003	0.045	-0.054	-0.013
64.H	-0.033	0.029	-0.015	0.009	-0.014	-0.006	0.034	-0.055	0.002
65.H	-0.034	-0.008	0.004	-0.002	-0.044	0.002	0.007	0.005	-0.044
66.H	-0.020	-0.025	0.007	0.007	-0.056	0.004	-0.022	0.037	-0.051
67.H	-0.019	-0.010	-0.001	0.008	-0.040	0.000	-0.018	0.021	-0.033
68.H	-0.013	-0.019	0.002	0.017	-0.052	0.002	-0.021	0.025	-0.036
69.C	-0.028	0.017	-0.011	0.001	-0.013	-0.006	-0.006	-0.006	-0.011
70.H	-0.063	-0.008	0.015	-0.046	-0.033	0.005	0.004	0.033	-0.080
71.H	-0.052	-0.017	0.016	-0.038	-0.039	0.005	-0.015	0.051	-0.080
72.H	-0.057	-0.023	0.021	-0.041	-0.049	0.008	-0.003	0.048	-0.091
73.H	-0.036	-0.019	0.010	-0.018	-0.042	0.004	-0.023	0.047	-0.063
74.H	-0.048	0.007	0.002	-0.029	-0.015	-0.002	-0.009	0.022	-0.046
75.H	-0.036	-0.002	0.002	-0.020	-0.022	-0.001	-0.028	0.039	-0.045
76.H	-0.034	0.030	-0.015	-0.010	0.007	-0.009	-0.011	-0.006	-0.004
77.H	-0.043	0.025	-0.009	-0.023	0.004	-0.007	-0.009	0.003	-0.021
78.H	-0.031	0.033	-0.018	-0.010	0.014	-0.011	-0.022	0.000	0.001
79.H	-0.011	0.019	-0.018	0.013	-0.002	-0.010	-0.037	0.010	0.007
80.H	-0.006	0.008	-0.015	0.015	-0.011	-0.008	-0.051	0.030	-0.002
81.H	-0.009	0.023	-0.021	0.011	0.005	-0.011	-0.047	0.016	0.012
82.H	-0.030	-0.013	0.006	-0.007	-0.041	0.002	-0.017	0.032	-0.050
83.H	-0.045	-0.022	0.015	-0.020	-0.055	0.007	0.005	0.027	-0.073
84.H	-0.042	0.013	-0.003	-0.018	-0.014	-0.003	-0.001	0.004	-0.032
85.H	-0.021	-0.003	-0.003	-0.001	-0.025	-0.003	-0.034	0.034	-0.029
86.H	-0.018	0.005	-0.008	0.019	-0.034	-0.003	0.003	-0.015	-0.012
87.O	0.000	0.000	0.000	0.000	0.000	0.000	0.000	0.000	0.000
88.H	-0.021	-0.034	0.012	0.012	-0.074	0.008	-0.003	0.026	-0.060
89.H	-0.018	0.025	-0.019	0.015	-0.006	-0.009	-0.008	-0.020	0.010
90.H	-0.008	0.013	-0.016	0.022	-0.016	-0.007	-0.025	0.000	0.005

91.H	-0.020	0.010	-0.011	0.012	-0.023	-0.005	-0.007	-0.007	-0.009
92.C	-0.018	0.016	-0.014	0.013	-0.014	-0.007	-0.012	-0.008	-0.000
93.O	0.000	0.000	0.000	0.000	0.000	0.000	0.000	0.000	0.000
94.H	-0.048	-0.026	0.019	-0.013	-0.070	0.010	0.032	0.005	-0.078
95.H	-0.041	-0.041	0.024	-0.009	-0.084	0.013	0.020	0.024	-0.089
96.H	-0.036	-0.034	0.018	0.001	-0.080	0.011	0.022	0.011	-0.074

	118			119			120		
1.O	0.000	0.000	0.000	0.000	0.000	0.000	0.000	0.000	0.000
2.C	0.000	0.000	0.000	0.000	0.000	0.000	0.000	0.000	0.000
3.C	0.000	0.000	0.000	0.000	0.000	0.000	0.000	0.000	0.000
4.C	0.000	0.000	0.000	0.000	0.000	0.000	0.000	0.000	0.000
5.O	0.000	0.000	0.000	0.000	0.000	0.000	0.000	0.000	0.000
6.N	0.000	-0.020	-0.022	-0.007	0.015	-0.011	-0.033	-0.033	0.000
7.O	0.000	0.000	0.000	0.000	0.000	0.000	0.000	0.000	0.000
8.C	0.000	0.000	0.000	0.000	0.000	0.000	0.000	0.000	0.000
9.C	0.000	0.000	0.000	0.000	0.000	0.000	0.000	0.000	0.000
10.C	0.000	0.000	0.000	0.000	0.000	0.000	0.000	0.000	0.000
11.O	0.000	0.000	0.000	0.000	0.000	0.000	0.000	0.000	0.000
12.C	0.000	0.000	0.000	0.000	0.000	0.000	0.000	0.000	0.000
13.O	0.000	0.000	0.000	0.000	0.000	0.000	0.000	0.000	0.000
14.C	0.000	0.000	0.000	0.000	0.000	0.000	0.000	0.000	0.000
15.C	0.000	0.000	0.000	0.000	0.000	0.000	0.000	0.000	0.000
16.O	0.000	0.000	0.000	0.000	0.000	0.000	0.000	0.000	0.000
17.C	0.000	0.000	0.000	0.000	0.000	0.000	0.000	0.000	0.000
18.C	0.000	0.000	0.000	0.000	0.000	0.000	0.000	0.000	0.000
19.O	0.000	0.000	0.000	0.000	0.000	0.000	0.000	0.000	0.000
20.C	0.000	0.000	0.000	0.000	0.000	0.000	0.000	0.000	0.000
21.C	0.000	0.000	0.000	0.000	0.000	0.000	0.000	0.000	0.000

22.C	0.000	0.000	0.000	0.000	0.000	0.000	0.000	0.000	0.000
23.C	0.000	0.000	0.000	0.000	0.000	0.000	0.000	0.000	0.000
24.C	0.000	0.000	0.000	0.000	0.000	0.000	0.000	0.000	0.000
25.C	0.000	0.000	0.000	0.000	0.000	0.000	0.000	0.000	0.000
26.C	0.000	0.000	0.000	0.000	0.000	0.000	0.000	0.000	0.000
27.C	0.000	0.000	0.000	0.000	0.000	0.000	0.000	0.000	0.000
28.C	0.000	0.000	0.000	0.000	0.000	0.000	0.000	0.000	0.000
29.C	0.000	0.000	0.000	0.000	0.000	0.000	0.000	0.000	0.000
30.C	0.000	0.000	0.000	0.000	0.000	0.000	0.000	0.000	0.000
31.O	0.000	0.000	0.000	0.000	0.000	0.000	0.000	0.000	0.000
32.C	0.000	0.000	0.000	0.000	0.000	0.000	0.000	0.000	0.000
33.C	0.000	0.000	0.000	0.000	0.000	0.000	0.000	0.000	0.000
34.C	0.000	0.000	0.000	0.000	0.000	0.000	0.000	0.000	0.000
35.C	0.000	0.000	0.000	0.000	0.000	0.000	0.000	0.000	0.000
36.C	0.000	0.000	0.000	0.000	0.000	0.000	0.000	0.000	0.000
37.C	0.000	0.000	0.000	0.000	0.000	0.000	0.000	0.000	0.000
38.C	0.000	0.000	0.000	0.000	0.000	0.000	0.000	0.000	0.000
39.C	0.241	0.030	0.035	0.000	0.242	0.026	0.000	0.000	0.237
40.H	-0.064	0.003	0.008	0.001	-0.079	0.001	0.041	0.045	-0.052
41.H	-0.051	-0.029	-0.013	0.001	-0.031	-0.003	0.024	0.032	-0.020
42.H	-0.072	-0.028	-0.007	0.016	-0.039	0.006	0.034	0.039	-0.038
43.H	-0.062	-0.038	-0.015	0.001	-0.036	-0.003	0.039	0.049	-0.018
44.H	-0.014	-0.009	-0.012	-0.044	-0.060	-0.025	0.027	0.039	0.004
45.H	-0.028	-0.005	-0.006	-0.039	-0.076	-0.021	0.040	0.052	-0.009
46.H	-0.015	0.009	-0.002	-0.042	-0.078	-0.023	0.027	0.036	-0.012
47.H	-0.041	0.007	0.004	-0.018	-0.077	-0.010	0.032	0.037	-0.035
48.H	-0.018	-0.007	-0.010	-0.026	-0.045	-0.017	0.013	0.019	-0.009
49.H	-0.035	-0.001	-0.002	-0.010	-0.052	-0.007	0.016	0.019	-0.030
50.H	0.005	0.008	-0.008	-0.039	-0.047	-0.024	-0.003	0.002	-0.003
51.H	0.008	0.026	0.002	-0.041	-0.064	-0.024	-0.004	-0.002	-0.016
52.H	0.003	0.013	-0.004	-0.046	-0.064	-0.026	0.008	0.015	-0.005

53.H	-0.005	0.037	0.011	-0.040	-0.090	-0.021	0.012	0.014	-0.031
54.H	-0.009	0.045	0.017	-0.027	-0.087	-0.014	0.003	0.000	-0.046
55.H	-0.022	0.045	0.020	-0.026	-0.101	-0.012	0.017	0.016	-0.053
56.H	-0.090	0.012	0.020	0.021	-0.094	0.013	0.051	0.051	-0.082
57.H	-0.112	0.007	0.024	0.045	-0.086	0.026	0.052	0.048	-0.101
58.H	-0.099	0.014	0.024	0.044	-0.078	0.025	0.037	0.030	-0.100
59.H	-0.096	0.038	0.036	0.039	-0.105	0.024	0.038	0.029	-0.115
60.H	-0.107	0.032	0.036	0.039	-0.113	0.025	0.053	0.047	-0.116
61.H	-0.116	0.032	0.039	0.054	-0.105	0.032	0.047	0.037	-0.128
62.H	-0.108	-0.014	0.010	0.037	-0.071	0.020	0.056	0.058	-0.077
63.H	-0.097	-0.013	0.008	0.021	-0.078	0.012	0.060	0.066	-0.064
64.H	-0.093	-0.026	0.000	0.024	-0.058	0.012	0.053	0.058	-0.054
65.H	-0.043	0.014	0.009	0.004	-0.060	0.001	0.011	0.009	-0.053
66.H	-0.029	0.032	0.015	0.008	-0.053	0.003	-0.013	-0.022	-0.063
67.H	-0.036	0.014	0.007	0.011	-0.040	0.003	-0.007	-0.013	-0.053
68.H	-0.049	0.026	0.017	0.021	-0.057	0.010	-0.001	-0.010	-0.074
69.C	-0.035	-0.017	-0.011	0.004	-0.016	-0.002	-0.001	-0.000	-0.024
70.H	0.041	0.009	-0.017	-0.050	-0.018	-0.032	-0.036	-0.033	0.018
71.H	0.045	0.018	-0.013	-0.044	-0.013	-0.029	-0.048	-0.050	0.010
72.H	0.038	0.027	-0.006	-0.045	-0.033	-0.029	-0.038	-0.039	-0.000
73.H	0.017	0.021	-0.004	-0.022	-0.022	-0.016	-0.038	-0.043	-0.017
74.H	0.023	-0.009	-0.022	-0.033	0.001	-0.024	-0.033	-0.031	0.016
75.H	0.026	-0.000	-0.018	-0.025	0.005	-0.020	-0.045	-0.047	0.006
76.H	-0.010	-0.035	-0.028	-0.010	0.015	-0.013	-0.017	-0.012	0.010
77.H	0.011	-0.031	-0.031	-0.025	0.017	-0.021	-0.027	-0.021	0.023
78.H	0.002	-0.041	-0.035	-0.012	0.032	-0.015	-0.030	-0.025	0.021
79.H	-0.025	-0.024	-0.018	0.013	0.013	0.000	-0.024	-0.026	-0.018
80.H	-0.013	-0.012	-0.014	0.012	0.015	-0.001	-0.037	-0.043	-0.022
81.H	-0.012	-0.030	-0.025	0.009	0.031	-0.004	-0.036	-0.038	-0.005
82.H	-0.009	0.017	0.001	-0.008	-0.033	-0.008	-0.021	-0.025	-0.033
83.H	-0.010	0.029	0.008	-0.018	-0.059	-0.011	-0.008	-0.011	-0.038

84.H	-0.006	-0.014	-0.017	-0.017	-0.011	-0.015	-0.014	-0.010	-0.002
85.H	-0.002	0.002	-0.009	-0.003	-0.004	-0.008	-0.034	-0.039	-0.020
86.H	-0.078	0.001	0.011	0.029	-0.059	0.015	0.027	0.025	-0.072
87.O	0.000	0.000	0.000	0.000	0.000	0.000	0.000	0.000	0.000
88.H	-0.059	0.046	0.031	0.019	-0.091	0.012	0.014	0.004	-0.094
89.H	-0.059	-0.026	-0.010	0.021	-0.017	0.007	0.011	0.012	-0.037
90.H	-0.053	-0.013	-0.004	0.026	-0.017	0.010	-0.001	-0.005	-0.047
91.H	-0.055	-0.008	-0.001	0.018	-0.034	0.007	0.009	0.008	-0.048
92.C	-0.052	-0.016	-0.006	0.019	-0.021	0.006	0.005	0.004	-0.040
93.O	0.000	0.000	0.000	0.000	0.000	0.000	0.000	0.000	0.000
94.H	-0.050	0.039	0.024	-0.004	-0.103	-0.000	0.028	0.026	-0.072
95.H	-0.045	0.056	0.032	-0.001	-0.109	0.002	0.019	0.012	-0.085
96.H	-0.065	0.049	0.034	0.011	-0.113	0.009	0.031	0.024	-0.095

	121			122			123		
1.O	0.000	0.000	0.000	0.000	0.000	0.000	0.000	0.000	0.000
2.C	0.000	0.000	0.000	0.000	0.000	0.000	0.000	0.000	0.000
3.C	0.000	0.000	0.000	0.000	0.000	0.000	0.000	0.000	0.000
4.C	0.000	0.000	0.000	0.000	0.000	0.000	0.000	0.000	0.000
5.O	0.000	0.000	0.000	0.000	0.000	0.000	0.000	0.000	0.000
6.N	-0.042	-0.003	0.002	0.001	-0.051	0.002	0.013	-0.002	-0.049
7.O	0.000	0.000	0.000	0.000	0.000	0.000	0.000	0.000	0.000
8.C	0.000	0.000	0.000	0.000	0.000	0.000	0.000	0.000	0.000
9.C	0.000	0.000	0.000	0.000	0.000	0.000	0.000	0.000	0.000
10.C	0.000	0.000	0.000	0.000	0.000	0.000	0.000	0.000	0.000
11.O	0.000	0.000	0.000	0.000	0.000	0.000	0.000	0.000	0.000
12.C	0.000	0.000	0.000	0.000	0.000	0.000	0.000	0.000	0.000
13.O	0.000	0.000	0.000	0.000	0.000	0.000	0.000	0.000	0.000
14.C	0.000	0.000	0.000	0.000	0.000	0.000	0.000	0.000	0.000

15.C	0.000	0.000	0.000	0.000	0.000	0.000	0.000	0.000	0.000
16.O	0.000	0.000	0.000	0.000	0.000	0.000	0.000	0.000	0.000
17.C	0.000	0.000	0.000	0.000	0.000	0.000	0.000	0.000	0.000
18.C	0.000	0.000	0.000	0.000	0.000	0.000	0.000	0.000	0.000
19.O	0.000	0.000	0.000	0.000	0.000	0.000	0.000	0.000	0.000
20.C	0.000	0.000	0.000	0.000	0.000	0.000	0.000	0.000	0.000
21.C	0.000	0.000	0.000	0.000	0.000	0.000	0.000	0.000	0.000
22.C	0.000	0.000	0.000	0.000	0.000	0.000	0.000	0.000	0.000
23.C	0.000	0.000	0.000	0.000	0.000	0.000	0.000	0.000	0.000
24.C	0.000	0.000	0.000	0.000	0.000	0.000	0.000	0.000	0.000
25.C	0.000	0.000	0.000	0.000	0.000	0.000	0.000	0.000	0.000
26.C	0.000	0.000	0.000	0.000	0.000	0.000	0.000	0.000	0.000
27.C	0.000	0.000	0.000	0.000	0.000	0.000	0.000	0.000	0.000
28.C	0.000	0.000	0.000	0.000	0.000	0.000	0.000	0.000	0.000
29.C	0.000	0.000	0.000	0.000	0.000	0.000	0.000	0.000	0.000
30.C	0.000	0.000	0.000	0.000	0.000	0.000	0.000	0.000	0.000
31.O	0.000	0.000	0.000	0.000	0.000	0.000	0.000	0.000	0.000
32.C	0.000	0.000	0.000	0.000	0.000	0.000	0.000	0.000	0.000
33.C	0.000	0.000	0.000	0.000	0.000	0.000	0.000	0.000	0.000
34.C	0.000	0.000	0.000	0.000	0.000	0.000	0.000	0.000	0.000
35.C	0.000	0.000	0.000	0.000	0.000	0.000	0.000	0.000	0.000
36.C	0.000	0.000	0.000	0.000	0.000	0.000	0.000	0.000	0.000
37.C	0.000	0.000	0.000	0.000	0.000	0.000	0.000	0.000	0.000
38.C	0.000	0.000	0.000	0.000	0.000	0.000	0.000	0.000	0.000
39.C	0.000	0.000	0.000	0.000	0.000	0.000	0.000	0.000	0.000
40.H	-0.037	0.001	-0.002	0.002	-0.035	-0.002	-0.018	0.006	-0.034
41.H	-0.038	-0.005	0.002	0.001	-0.053	0.002	-0.011	0.008	-0.048
42.H	-0.041	-0.005	0.002	-0.006	-0.052	0.003	-0.011	0.009	-0.050
43.H	-0.037	-0.007	0.002	0.002	-0.057	0.003	-0.017	0.012	-0.050
44.H	-0.029	-0.001	-0.003	0.025	-0.042	-0.005	-0.022	0.007	-0.032
45.H	-0.029	-0.001	-0.003	0.024	-0.039	-0.005	-0.027	0.008	-0.029

46.H	-0.029	0.002	-0.005	0.024	-0.033	-0.006	-0.022	0.004	-0.025
47.H	-0.034	0.002	-0.003	0.012	-0.034	-0.004	-0.018	0.004	-0.030
48.H	-0.034	-0.001	-0.002	0.015	-0.042	-0.003	-0.012	0.003	-0.036
49.H	-0.037	0.000	-0.001	0.006	-0.039	-0.002	-0.009	0.003	-0.036
50.H	-0.033	0.002	-0.004	0.021	-0.035	-0.005	-0.008	-0.001	-0.029
51.H	-0.033	0.006	-0.005	0.022	-0.025	-0.007	-0.008	-0.004	-0.022
52.H	-0.031	0.003	-0.005	0.025	-0.031	-0.007	-0.015	-0.000	-0.025
53.H	-0.031	0.007	-0.007	0.022	-0.019	-0.009	-0.015	-0.004	-0.016
54.H	-0.035	0.009	-0.007	0.015	-0.015	-0.008	-0.008	-0.007	-0.017
55.H	-0.034	0.009	-0.007	0.015	-0.014	-0.008	-0.014	-0.005	-0.015
56.H	-0.040	0.002	-0.001	-0.008	-0.030	-0.001	-0.018	0.005	-0.034
57.H	-0.045	0.002	0.000	-0.021	-0.033	0.002	-0.012	0.005	-0.040
58.H	-0.046	0.003	0.000	-0.022	-0.030	0.002	-0.006	0.002	-0.039
59.H	-0.045	0.007	-0.003	-0.019	-0.017	-0.001	-0.008	-0.002	-0.028
60.H	-0.043	0.006	-0.002	-0.018	-0.020	-0.001	-0.014	0.002	-0.029
61.H	-0.047	0.006	-0.001	-0.026	-0.020	0.001	-0.008	0.000	-0.033
62.H	-0.043	-0.002	0.002	-0.016	-0.044	0.004	-0.016	0.010	-0.046
63.H	-0.039	-0.002	0.001	-0.007	-0.043	0.002	-0.022	0.011	-0.042
64.H	-0.040	-0.005	0.002	-0.009	-0.050	0.003	-0.018	0.012	-0.048
65.H	-0.040	0.003	-0.002	-0.002	-0.031	-0.002	-0.004	-0.001	-0.034
66.H	-0.044	0.007	-0.003	-0.006	-0.023	-0.003	0.008	-0.008	-0.030
67.H	-0.044	0.003	-0.001	-0.007	-0.032	-0.000	0.006	-0.004	-0.037
68.H	-0.045	0.006	-0.001	-0.012	-0.026	-0.001	0.006	-0.005	-0.034
69.C	0.269	-0.002	0.002	0.000	0.265	0.002	0.000	0.000	0.266
70.H	-0.034	0.003	-0.004	0.024	-0.036	-0.006	0.004	-0.006	-0.030
71.H	-0.036	0.005	-0.004	0.020	-0.032	-0.006	0.011	-0.010	-0.029
72.H	-0.035	0.006	-0.005	0.022	-0.027	-0.007	0.006	-0.010	-0.024
73.H	-0.040	0.005	-0.003	0.009	-0.030	-0.004	0.012	-0.009	-0.031
74.H	-0.037	-0.001	-0.001	0.015	-0.046	-0.002	0.007	-0.003	-0.040
75.H	-0.040	0.001	-0.001	0.010	-0.041	-0.002	0.015	-0.007	-0.040
76.H	-0.040	-0.006	0.003	0.004	-0.059	0.003	0.006	0.003	-0.053

77.H	-0.038	-0.005	0.002	0.011	-0.057	0.001	0.006	0.001	-0.050
78.H	-0.041	-0.007	0.004	0.004	-0.062	0.003	0.011	0.002	-0.056
79.H	-0.046	-0.003	0.003	-0.009	-0.053	0.004	0.014	-0.000	-0.054
80.H	-0.047	-0.001	0.002	-0.010	-0.047	0.003	0.020	-0.005	-0.051
81.H	-0.046	-0.004	0.004	-0.008	-0.057	0.004	0.019	-0.001	-0.057
82.H	-0.041	0.004	-0.002	0.002	-0.031	-0.002	0.008	-0.006	-0.034
83.H	-0.038	0.006	-0.004	0.009	-0.024	-0.005	-0.000	-0.006	-0.026
84.H	-0.039	-0.002	0.000	0.008	-0.047	-0.000	0.002	0.000	-0.043
85.H	-0.043	0.001	0.000	-0.001	-0.040	-0.000	0.015	-0.006	-0.042
86.H	-0.044	0.001	0.001	-0.014	-0.038	0.002	-0.005	0.003	-0.042
87.O	0.000	0.000	0.000	0.000	0.000	0.000	0.000	0.000	0.000
88.H	-0.043	0.009	-0.004	-0.010	-0.014	-0.004	-0.001	-0.006	-0.024
89.H	-0.044	-0.004	0.003	-0.011	-0.052	0.004	0.001	0.005	-0.053
90.H	-0.046	-0.002	0.003	-0.015	-0.046	0.004	0.007	0.001	-0.050
91.H	-0.044	-0.001	0.001	-0.010	-0.043	0.002	0.001	0.002	-0.045
92.C	-0.044	-0.002	0.002	-0.010	-0.048	0.003	0.003	0.003	-0.049
93.O	0.000	0.000	0.000	0.000	0.000	0.000	0.000	0.000	0.000
94.H	-0.037	0.008	-0.005	0.004	-0.017	-0.006	-0.014	-0.002	-0.021
95.H	-0.039	0.011	-0.006	0.002	-0.009	-0.007	-0.008	-0.007	-0.016
96.H	-0.040	0.010	-0.005	-0.004	-0.012	-0.005	-0.011	-0.004	-0.019

	124			125			126		
	------------------------			------------------------			------------------------		
1.O	0.000	0.000	0.000	0.000	0.000	0.000	0.000	0.000	0.000
2.C	0.000	0.000	0.000	0.000	0.000	0.000	0.000	0.000	0.000
3.C	0.000	0.000	0.000	0.000	0.000	0.000	0.000	0.000	0.000
4.C	0.000	0.000	0.000	0.000	0.000	0.000	0.000	0.000	0.000
5.O	0.000	0.000	0.000	0.000	0.000	0.000	0.000	0.000	0.000
6.N	-0.046	-0.013	0.001	-0.001	-0.057	0.001	0.015	-0.013	-0.056
7.O	0.000	0.000	0.000	0.000	0.000	0.000	0.000	0.000	0.000

8.C	0.000	0.000	0.000	0.000	0.000	0.000	0.000	0.000	0.000
9.C	0.000	0.000	0.000	0.000	0.000	0.000	0.000	0.000	0.000
10.C	0.000	0.000	0.000	0.000	0.000	0.000	0.000	0.000	0.000
11.O	0.000	0.000	0.000	0.000	0.000	0.000	0.000	0.000	0.000
12.C	0.000	0.000	0.000	0.000	0.000	0.000	0.000	0.000	0.000
13.O	0.000	0.000	0.000	0.000	0.000	0.000	0.000	0.000	0.000
14.C	0.000	0.000	0.000	0.000	0.000	0.000	0.000	0.000	0.000
15.C	0.000	0.000	0.000	0.000	0.000	0.000	0.000	0.000	0.000
16.O	0.000	0.000	0.000	0.000	0.000	0.000	0.000	0.000	0.000
17.C	0.000	0.000	0.000	0.000	0.000	0.000	0.000	0.000	0.000
18.C	0.000	0.000	0.000	0.000	0.000	0.000	0.000	0.000	0.000
19.O	0.000	0.000	0.000	0.000	0.000	0.000	0.000	0.000	0.000
20.C	0.000	0.000	0.000	0.000	0.000	0.000	0.000	0.000	0.000
21.C	0.000	0.000	0.000	0.000	0.000	0.000	0.000	0.000	0.000
22.C	0.000	0.000	0.000	0.000	0.000	0.000	0.000	0.000	0.000
23.C	0.000	0.000	0.000	0.000	0.000	0.000	0.000	0.000	0.000
24.C	0.000	0.000	0.000	0.000	0.000	0.000	0.000	0.000	0.000
25.C	0.000	0.000	0.000	0.000	0.000	0.000	0.000	0.000	0.000
26.C	0.000	0.000	0.000	0.000	0.000	0.000	0.000	0.000	0.000
27.C	0.000	0.000	0.000	0.000	0.000	0.000	0.000	0.000	0.000
28.C	0.000	0.000	0.000	0.000	0.000	0.000	0.000	0.000	0.000
29.C	0.000	0.000	0.000	0.000	0.000	0.000	0.000	0.000	0.000
30.C	0.000	0.000	0.000	0.000	0.000	0.000	0.000	0.000	0.000
31.O	0.000	0.000	0.000	0.000	0.000	0.000	0.000	0.000	0.000
32.C	0.000	0.000	0.000	0.000	0.000	0.000	0.000	0.000	0.000
33.C	0.000	0.000	0.000	0.000	0.000	0.000	0.000	0.000	0.000
34.C	0.000	0.000	0.000	0.000	0.000	0.000	0.000	0.000	0.000
35.C	0.000	0.000	0.000	0.000	0.000	0.000	0.000	0.000	0.000
36.C	0.000	0.000	0.000	0.000	0.000	0.000	0.000	0.000	0.000
37.C	0.000	0.000	0.000	0.000	0.000	0.000	0.000	0.000	0.000
38.C	0.000	0.000	0.000	0.000	0.000	0.000	0.000	0.000	0.000

39.C	0.000	0.000	0.000	0.000	0.000	0.000	0.000	0.000	0.000
40.H	-0.047	0.002	-0.001	0.001	-0.048	-0.001	-0.019	0.027	-0.044
41.H	-0.048	-0.022	0.003	-0.001	-0.067	0.003	-0.011	0.029	-0.060
42.H	-0.060	-0.022	0.007	-0.009	-0.067	0.007	-0.008	0.033	-0.070
43.H	-0.047	-0.030	0.004	0.000	-0.074	0.004	-0.018	0.042	-0.064
44.H	-0.011	-0.007	-0.010	0.028	-0.055	-0.010	-0.034	0.027	-0.020
45.H	-0.014	-0.004	-0.010	0.026	-0.053	-0.010	-0.038	0.034	-0.020
46.H	-0.013	0.007	-0.012	0.027	-0.044	-0.012	-0.033	0.020	-0.013
47.H	-0.032	0.006	-0.006	0.012	-0.045	-0.006	-0.024	0.021	-0.030
48.H	-0.027	-0.005	-0.006	0.016	-0.053	-0.006	-0.018	0.015	-0.032
49.H	-0.040	0.000	-0.003	0.005	-0.049	-0.003	-0.012	0.013	-0.041
50.H	-0.017	0.008	-0.011	0.023	-0.042	-0.010	-0.016	-0.001	-0.018
51.H	-0.016	0.022	-0.014	0.024	-0.031	-0.013	-0.017	-0.008	-0.008
52.H	-0.011	0.011	-0.013	0.028	-0.040	-0.013	-0.025	0.006	-0.010
53.H	-0.015	0.030	-0.015	0.025	-0.025	-0.015	-0.024	-0.002	-0.002
54.H	-0.026	0.037	-0.013	0.016	-0.019	-0.012	-0.013	-0.013	-0.008
55.H	-0.027	0.037	-0.013	0.016	-0.020	-0.012	-0.020	-0.004	-0.008
56.H	-0.063	0.009	0.003	-0.012	-0.043	0.003	-0.014	0.028	-0.054
57.H	-0.083	0.006	0.010	-0.027	-0.046	0.009	-0.002	0.028	-0.073
58.H	-0.083	0.012	0.009	-0.028	-0.041	0.008	0.005	0.015	-0.070
59.H	-0.078	0.031	0.004	-0.024	-0.026	0.004	0.001	0.007	-0.055
60.H	-0.077	0.025	0.005	-0.023	-0.031	0.004	-0.006	0.019	-0.057
61.H	-0.090	0.026	0.009	-0.033	-0.030	0.008	0.004	0.013	-0.068
62.H	-0.075	-0.012	0.010	-0.021	-0.060	0.009	-0.009	0.040	-0.076
63.H	-0.062	-0.011	0.006	-0.010	-0.060	0.005	-0.019	0.045	-0.064
64.H	-0.065	-0.021	0.008	-0.013	-0.067	0.008	-0.014	0.044	-0.073
65.H	-0.051	0.013	-0.001	-0.004	-0.039	-0.001	-0.002	0.001	-0.044
66.H	-0.057	0.028	-0.002	-0.009	-0.026	-0.002	0.013	-0.023	-0.041
67.H	-0.059	0.013	0.001	-0.010	-0.038	0.001	0.011	-0.012	-0.051
68.H	-0.067	0.022	0.002	-0.016	-0.030	0.002	0.013	-0.014	-0.052
69.C	0.000	0.000	0.000	0.000	0.000	0.000	0.000	0.000	0.000

70.H	-0.011	0.010	-0.013	0.027	-0.039	-0.013	-0.005	-0.022	-0.012
71.H	-0.016	0.018	-0.013	0.022	-0.032	-0.012	0.005	-0.036	-0.013
72.H	-0.015	0.024	-0.014	0.023	-0.028	-0.014	-0.001	-0.032	-0.008
73.H	-0.034	0.020	-0.008	0.008	-0.031	-0.007	0.011	-0.032	-0.027
74.H	-0.025	-0.005	-0.007	0.015	-0.051	-0.006	0.003	-0.015	-0.033
75.H	-0.032	0.003	-0.006	0.009	-0.044	-0.005	0.013	-0.028	-0.035
76.H	-0.043	-0.026	0.002	0.002	-0.068	0.002	0.006	0.004	-0.060
77.H	-0.031	-0.022	-0.002	0.011	-0.064	-0.002	0.003	-0.003	-0.048
78.H	-0.041	-0.030	0.003	0.003	-0.070	0.003	0.011	-0.002	-0.062
79.H	-0.062	-0.017	0.007	-0.013	-0.060	0.006	0.020	-0.008	-0.071
80.H	-0.062	-0.007	0.005	-0.014	-0.051	0.005	0.027	-0.022	-0.066
81.H	-0.059	-0.021	0.007	-0.012	-0.063	0.007	0.025	-0.013	-0.072
82.H	-0.045	0.016	-0.004	0.000	-0.035	-0.004	0.009	-0.020	-0.037
83.H	-0.035	0.025	-0.008	0.008	-0.029	-0.008	-0.003	-0.015	-0.024
84.H	-0.036	-0.009	-0.002	0.007	-0.055	-0.002	0.000	-0.001	-0.044
85.H	-0.049	0.004	-0.001	-0.004	-0.043	-0.000	0.018	-0.024	-0.048
86.H	-0.071	0.001	0.007	-0.019	-0.048	0.006	0.002	0.016	-0.066
87.O	0.000	0.000	0.000	0.000	0.000	0.000	0.000	0.000	0.000
88.H	-0.064	0.038	-0.001	-0.014	-0.019	-0.002	0.004	-0.011	-0.040
89.H	-0.066	-0.020	0.008	-0.015	-0.064	0.008	0.007	0.016	-0.075
90.H	-0.071	-0.009	0.008	-0.020	-0.055	0.008	0.015	0.002	-0.073
91.H	-0.064	-0.005	0.005	-0.014	-0.053	0.005	0.006	0.008	-0.065
92.C	0.258	-0.011	0.007	0.000	0.261	0.006	0.000	0.000	0.256
93.O	0.000	0.000	0.000	0.000	0.000	0.000	0.000	0.000	0.000
94.H	-0.044	0.032	-0.007	0.003	-0.025	-0.007	-0.015	0.004	-0.025
95.H	-0.047	0.045	-0.008	-0.000	-0.014	-0.008	-0.009	-0.009	-0.021
96.H	-0.056	0.040	-0.004	-0.007	-0.019	-0.004	-0.009	0.000	-0.031

127 128 129

------------------------ ------------------------ ------------------------

1.O	0.000	0.000	0.000	0.000	0.000	0.000	0.000	0.000	0.000
2.C	0.000	0.000	0.000	0.000	0.000	0.000	0.000	0.000	0.000
3.C	0.000	0.000	0.000	0.000	0.000	0.000	0.000	0.000	0.000
4.C	0.000	0.000	0.000	0.000	0.000	0.000	0.000	0.000	0.000
5.O	0.000	0.000	0.000	0.000	0.000	0.000	0.000	0.000	0.000
6.N	0.216	0.001	0.037	0.000	0.205	0.011	0.000	0.000	0.220
7.O	0.000	0.000	0.000	0.000	0.000	0.000	0.000	0.000	0.000
8.C	0.000	0.000	0.000	0.000	0.000	0.000	0.000	0.000	0.000
9.C	0.000	0.000	0.000	0.000	0.000	0.000	0.000	0.000	0.000
10.C	0.000	0.000	0.000	0.000	0.000	0.000	0.000	0.000	0.000
11.O	0.000	0.000	0.000	0.000	0.000	0.000	0.000	0.000	0.000
12.C	0.000	0.000	0.000	0.000	0.000	0.000	0.000	0.000	0.000
13.O	0.000	0.000	0.000	0.000	0.000	0.000	0.000	0.000	0.000
14.C	0.000	0.000	0.000	0.000	0.000	0.000	0.000	0.000	0.000
15.C	0.000	0.000	0.000	0.000	0.000	0.000	0.000	0.000	0.000
16.O	0.000	0.000	0.000	0.000	0.000	0.000	0.000	0.000	0.000
17.C	0.000	0.000	0.000	0.000	0.000	0.000	0.000	0.000	0.000
18.C	0.000	0.000	0.000	0.000	0.000	0.000	0.000	0.000	0.000
19.O	0.000	0.000	0.000	0.000	0.000	0.000	0.000	0.000	0.000
20.C	0.000	0.000	0.000	0.000	0.000	0.000	0.000	0.000	0.000
21.C	0.000	0.000	0.000	0.000	0.000	0.000	0.000	0.000	0.000
22.C	0.000	0.000	0.000	0.000	0.000	0.000	0.000	0.000	0.000
23.C	0.000	0.000	0.000	0.000	0.000	0.000	0.000	0.000	0.000
24.C	0.000	0.000	0.000	0.000	0.000	0.000	0.000	0.000	0.000
25.C	0.000	0.000	0.000	0.000	0.000	0.000	0.000	0.000	0.000
26.C	0.000	0.000	0.000	0.000	0.000	0.000	0.000	0.000	0.000
27.C	0.000	0.000	0.000	0.000	0.000	0.000	0.000	0.000	0.000
28.C	0.000	0.000	0.000	0.000	0.000	0.000	0.000	0.000	0.000
29.C	0.000	0.000	0.000	0.000	0.000	0.000	0.000	0.000	0.000
30.C	0.000	0.000	0.000	0.000	0.000	0.000	0.000	0.000	0.000
31.O	0.000	0.000	0.000	0.000	0.000	0.000	0.000	0.000	0.000

32.C	0.000	0.000	0.000	0.000	0.000	0.000	0.000	0.000	0.000
33.C	0.000	0.000	0.000	0.000	0.000	0.000	0.000	0.000	0.000
34.C	0.000	0.000	0.000	0.000	0.000	0.000	0.000	0.000	0.000
35.C	0.000	0.000	0.000	0.000	0.000	0.000	0.000	0.000	0.000
36.C	0.000	0.000	0.000	0.000	0.000	0.000	0.000	0.000	0.000
37.C	0.000	0.000	0.000	0.000	0.000	0.000	0.000	0.000	0.000
38.C	0.000	0.000	0.000	0.000	0.000	0.000	0.000	0.000	0.000
39.C	0.000	0.000	0.000	0.000	0.000	0.000	0.000	0.000	0.000
40.H	-0.009	-0.004	-0.018	-0.002	-0.005	-0.011	-0.045	-0.006	-0.048
41.H	-0.033	-0.006	0.027	-0.003	-0.064	-0.004	-0.022	0.007	-0.092
42.H	-0.025	-0.006	0.025	-0.018	-0.053	-0.021	-0.027	0.006	-0.087
43.H	-0.013	-0.008	0.033	-0.002	-0.051	-0.005	-0.042	0.007	-0.098
44.H	-0.014	-0.004	-0.006	0.044	-0.029	0.038	-0.049	-0.001	-0.069
45.H	0.003	-0.005	-0.015	0.040	-0.005	0.030	-0.065	-0.005	-0.059
46.H	-0.015	-0.003	-0.028	0.042	-0.010	0.033	-0.049	-0.007	-0.048
47.H	-0.016	-0.003	-0.023	0.017	-0.011	0.008	-0.042	-0.006	-0.047
48.H	-0.042	-0.002	0.000	0.025	-0.054	0.022	-0.019	0.002	-0.072
49.H	-0.042	-0.002	-0.006	0.008	-0.046	0.004	-0.015	0.000	-0.063
50.H	-0.060	0.000	-0.015	0.037	-0.059	0.035	-0.004	0.000	-0.061
51.H	-0.060	0.002	-0.036	0.039	-0.041	0.034	-0.004	-0.005	-0.040
52.H	-0.041	-0.000	-0.026	0.045	-0.035	0.039	-0.024	-0.004	-0.050
53.H	-0.038	0.002	-0.056	0.039	-0.006	0.030	-0.026	-0.012	-0.020
54.H	-0.056	0.004	-0.060	0.025	-0.015	0.017	-0.006	-0.012	-0.014
55.H	-0.035	0.003	-0.065	0.024	0.007	0.013	-0.026	-0.015	-0.008
56.H	-0.001	-0.004	-0.030	-0.023	0.015	-0.035	-0.048	-0.011	-0.032
57.H	-0.009	-0.004	-0.020	-0.049	0.004	-0.058	-0.035	-0.008	-0.037
58.H	-0.031	-0.002	-0.022	-0.049	-0.012	-0.056	-0.015	-0.007	-0.035
59.H	-0.026	0.001	-0.054	-0.043	0.017	-0.054	-0.020	-0.015	-0.005
60.H	-0.005	-0.001	-0.051	-0.042	0.033	-0.056	-0.041	-0.017	-0.007
61.H	-0.019	-0.000	-0.047	-0.058	0.020	-0.070	-0.024	-0.015	-0.009
62.H	-0.000	-0.007	0.003	-0.039	-0.009	-0.048	-0.046	-0.003	-0.061

63.H	0.012	-0.007	-0.002	-0.022	0.003	-0.032	-0.061	-0.005	-0.059
64.H	-0.000	-0.008	0.016	-0.025	-0.023	-0.032	-0.049	0.001	-0.076
65.H	-0.054	0.000	-0.020	-0.007	-0.042	-0.011	-0.001	-0.003	-0.046
66.H	-0.091	0.005	-0.032	-0.014	-0.062	-0.015	0.035	-0.003	-0.034
67.H	-0.084	0.002	-0.011	-0.016	-0.073	-0.016	0.029	0.001	-0.054
68.H	-0.079	0.003	-0.026	-0.026	-0.054	-0.029	0.026	-0.003	-0.037
69.C	0.000	0.000	0.000	0.000	0.000	0.000	0.000	0.000	0.000
70.H	-0.104	0.004	-0.005	0.047	-0.107	0.051	0.036	0.007	-0.073
71.H	-0.125	0.006	-0.010	0.040	-0.119	0.045	0.057	0.007	-0.067
72.H	-0.109	0.006	-0.025	0.042	-0.093	0.044	0.041	0.002	-0.052
73.H	-0.118	0.006	-0.013	0.017	-0.106	0.020	0.055	0.005	-0.059
74.H	-0.107	0.002	0.020	0.028	-0.127	0.035	0.042	0.013	-0.094
75.H	-0.127	0.004	0.015	0.020	-0.139	0.028	0.064	0.013	-0.087
76.H	-0.091	-0.002	0.050	0.005	-0.134	0.013	0.032	0.018	-0.117
77.H	-0.100	-0.001	0.045	0.020	-0.141	0.029	0.037	0.018	-0.116
78.H	-0.109	-0.002	0.061	0.007	-0.160	0.018	0.048	0.023	-0.129
79.H	-0.108	-0.000	0.042	-0.019	-0.138	-0.010	0.053	0.017	-0.105
80.H	-0.128	0.003	0.033	-0.019	-0.147	-0.009	0.072	0.016	-0.097
81.H	-0.125	0.000	0.054	-0.016	-0.163	-0.004	0.069	0.021	-0.118
82.H	-0.097	0.004	-0.012	0.003	-0.087	0.004	0.038	0.003	-0.056
83.H	-0.075	0.004	-0.034	0.014	-0.051	0.011	0.015	-0.004	-0.037
84.H	-0.083	-0.001	0.021	0.014	-0.104	0.017	0.022	0.010	-0.091
85.H	-0.118	0.004	0.012	-0.003	-0.124	0.004	0.059	0.011	-0.080
86.H	-0.039	-0.002	-0.005	-0.033	-0.036	-0.037	-0.010	-0.002	-0.055
87.O	0.000	0.000	0.000	0.000	0.000	0.000	0.000	0.000	0.000
88.H	-0.056	0.004	-0.057	-0.023	-0.009	-0.032	0.004	-0.013	-0.006
89.H	-0.061	-0.004	0.033	-0.026	-0.089	-0.024	0.009	0.010	-0.094
90.H	-0.081	-0.001	0.023	-0.032	-0.096	-0.028	0.029	0.009	-0.083
91.H	-0.062	-0.002	0.011	-0.023	-0.072	-0.023	0.010	0.005	-0.073
92.C	0.000	0.000	0.000	0.000	0.000	0.000	0.000	0.000	0.000
93.O	0.000	0.000	0.000	0.000	0.000	0.000	0.000	0.000	0.000

94.H	-0.026	0.001	-0.058	0.002	0.013	-0.009	-0.030	-0.015	-0.010
95.H	-0.041	0.004	-0.074	-0.001	0.015	-0.013	-0.014	-0.018	0.006
96.H	-0.027	0.002	-0.069	-0.013	0.024	-0.026	-0.025	-0.018	0.004

	130			131			132		
	------------------------			------------------------			------------------------		
1.O	0.000	0.000	0.000	0.000	0.000	0.000	0.000	0.000	0.000
2.C	0.000	0.000	0.000	0.000	0.000	0.000	0.000	0.000	0.000
3.C	0.000	0.000	0.000	0.000	0.000	0.000	0.000	0.000	0.000
4.C	0.000	0.000	0.000	0.000	0.000	0.000	0.000	0.000	0.000
5.O	0.000	0.000	0.000	0.000	0.000	0.000	0.000	0.000	0.000
6.N	0.000	0.000	0.000	0.000	0.000	0.000	0.000	0.000	0.000
7.O	0.000	0.000	0.000	0.000	0.000	0.000	0.000	0.000	0.000
8.C	0.000	0.000	0.000	0.000	0.000	0.000	0.000	0.000	0.000
9.C	0.000	0.000	0.000	0.000	0.000	0.000	0.000	0.000	0.000
10.C	0.000	0.000	0.000	0.000	0.000	0.000	0.000	0.000	0.000
11.O	0.000	0.000	0.000	0.000	0.000	0.000	0.000	0.000	0.000
12.C	0.000	0.000	0.000	0.000	0.000	0.000	0.000	0.000	0.000
13.O	0.000	0.000	0.000	0.000	0.000	0.000	0.000	0.000	0.000
14.C	0.000	0.000	0.000	0.000	0.000	0.000	0.000	0.000	0.000
15.C	0.000	0.000	0.000	0.000	0.000	0.000	0.000	0.000	0.000
16.O	0.000	0.000	0.000	0.000	0.000	0.000	0.000	0.000	0.000
17.C	0.000	0.000	0.000	0.000	0.000	0.000	0.000	0.000	0.000
18.C	0.000	0.000	0.000	0.000	0.000	0.000	0.000	0.000	0.000
19.O	0.000	0.000	0.000	0.000	0.000	0.000	0.000	0.000	0.000
20.C	0.000	0.000	0.000	0.000	0.000	0.000	0.000	0.000	0.000
21.C	0.000	0.000	0.000	0.000	0.000	0.000	0.000	0.000	0.000
22.C	0.000	0.000	0.000	0.000	0.000	0.000	0.000	0.000	0.000
23.C	0.000	0.000	0.000	0.000	0.000	0.000	0.000	0.000	0.000
24.C	0.000	0.000	0.000	0.000	0.000	0.000	0.000	0.000	0.000

25.C	0.000	0.000	0.000	0.000	0.000	0.000	0.000	0.000	0.000
26.C	0.000	0.000	0.000	0.000	0.000	0.000	0.000	0.000	0.000
27.C	0.000	0.000	0.000	0.000	0.000	0.000	0.000	0.000	0.000
28.C	0.000	0.000	0.000	0.000	0.000	0.000	0.000	0.000	0.000
29.C	0.000	0.000	0.000	0.000	0.000	0.000	0.000	0.000	0.000
30.C	0.000	0.000	0.000	0.000	0.000	0.000	0.000	0.000	0.000
31.O	0.000	0.000	0.000	0.000	0.000	0.000	0.000	0.000	0.000
32.C	0.000	0.000	0.000	0.000	0.000	0.000	0.000	0.000	0.000
33.C	0.000	0.000	0.000	0.000	0.000	0.000	0.000	0.000	0.000
34.C	0.000	0.000	0.000	0.000	0.000	0.000	0.000	0.000	0.000
35.C	0.000	0.000	0.000	0.000	0.000	0.000	0.000	0.000	0.000
36.C	0.000	0.000	0.000	0.000	0.000	0.000	0.000	0.000	0.000
37.C	0.000	0.000	0.000	0.000	0.000	0.000	0.000	0.000	0.000
38.C	0.000	0.000	0.000	0.000	0.000	0.000	0.000	0.000	0.000
39.C	0.000	0.000	0.000	0.000	0.000	0.000	0.000	0.000	0.000
40.H	0.979	0.001	0.003	0.000	0.979	0.003	0.000	0.000	0.986
41.H	-0.027	0.001	-0.012	0.000	-0.027	0.003	0.001	0.001	-0.017
42.H	-0.029	0.002	-0.011	0.000	-0.030	0.008	0.002	0.002	-0.018
43.H	-0.033	0.002	-0.014	0.001	-0.033	0.003	0.003	0.002	-0.017
44.H	-0.033	0.001	-0.002	0.001	-0.031	-0.014	0.002	0.002	-0.016
45.H	-0.038	0.001	0.002	0.001	-0.036	-0.012	0.003	0.003	-0.017
46.H	-0.033	0.000	0.005	0.001	-0.030	-0.014	0.002	0.002	-0.017
47.H	-0.032	0.000	0.004	0.001	-0.031	-0.005	0.002	0.002	-0.019
48.H	-0.024	0.001	-0.003	0.001	-0.023	-0.008	0.001	0.001	-0.017
49.H	-0.024	0.000	-0.001	0.000	-0.023	-0.002	0.001	0.001	-0.018
50.H	-0.018	-0.000	0.001	0.000	-0.017	-0.014	-0.000	-0.000	-0.017
51.H	-0.018	-0.001	0.008	0.000	-0.016	-0.015	-0.000	-0.000	-0.018
52.H	-0.024	-0.000	0.005	0.001	-0.022	-0.016	0.001	0.001	-0.017
53.H	-0.025	-0.001	0.014	0.001	-0.023	-0.014	0.001	0.001	-0.019
54.H	-0.019	-0.001	0.016	0.000	-0.018	-0.010	0.000	0.000	-0.020
55.H	-0.026	-0.001	0.018	0.001	-0.024	-0.009	0.001	0.001	-0.021

56.H	-0.037	0.001	0.007	0.001	-0.037	0.010	0.003	0.003	-0.022
57.H	-0.034	0.001	0.004	0.000	-0.035	0.019	0.003	0.003	-0.023
58.H	-0.028	0.000	0.005	-0.000	-0.028	0.018	0.002	0.002	-0.023
59.H	-0.029	-0.001	0.015	0.000	-0.029	0.016	0.002	0.002	-0.025
60.H	-0.036	-0.000	0.014	0.000	-0.036	0.016	0.003	0.003	-0.025
61.H	-0.031	-0.000	0.013	-0.000	-0.032	0.022	0.002	0.002	-0.025
62.H	-0.037	0.002	-0.004	0.001	-0.038	0.017	0.004	0.003	-0.021
63.H	-0.041	0.002	-0.002	0.001	-0.041	0.011	0.004	0.004	-0.021
64.H	-0.037	0.002	-0.008	0.001	-0.037	0.012	0.003	0.003	-0.020
65.H	-0.020	-0.000	0.003	-0.000	-0.020	0.002	0.000	0.000	-0.020
66.H	-0.008	-0.001	0.007	-0.001	-0.009	0.003	-0.002	-0.002	-0.021
67.H	-0.010	-0.001	0.000	-0.001	-0.011	0.005	-0.002	-0.001	-0.020
68.H	-0.012	-0.001	0.006	-0.001	-0.013	0.008	-0.001	-0.001	-0.022
69.C	0.000	0.000	0.000	0.000	0.000	0.000	0.000	0.000	0.000
70.H	-0.004	-0.001	-0.002	-0.000	-0.003	-0.019	-0.003	-0.003	-0.015
71.H	0.003	-0.001	-0.000	-0.001	0.003	-0.017	-0.004	-0.004	-0.016
72.H	-0.002	-0.001	0.005	-0.000	-0.002	-0.017	-0.003	-0.003	-0.017
73.H	0.000	-0.001	0.001	-0.001	0.000	-0.008	-0.004	-0.003	-0.018
74.H	-0.003	-0.000	-0.010	-0.001	-0.003	-0.012	-0.003	-0.003	-0.015
75.H	0.004	-0.001	-0.008	-0.001	0.003	-0.009	-0.004	-0.004	-0.016
76.H	-0.008	0.001	-0.019	-0.001	-0.009	-0.002	-0.002	-0.002	-0.015
77.H	-0.005	0.001	-0.018	-0.000	-0.006	-0.008	-0.003	-0.003	-0.014
78.H	-0.002	0.001	-0.023	-0.001	-0.004	-0.003	-0.003	-0.003	-0.014
79.H	-0.003	0.000	-0.016	-0.001	-0.004	0.006	-0.003	-0.003	-0.017
80.H	0.004	-0.000	-0.013	-0.001	0.002	0.005	-0.004	-0.004	-0.017
81.H	0.003	0.000	-0.020	-0.001	0.001	0.004	-0.004	-0.004	-0.016
82.H	-0.006	-0.001	0.001	-0.001	-0.007	-0.003	-0.002	-0.002	-0.019
83.H	-0.013	-0.001	0.008	-0.000	-0.013	-0.006	-0.001	-0.001	-0.019
84.H	-0.011	0.000	-0.010	-0.000	-0.011	-0.005	-0.002	-0.002	-0.016
85.H	0.001	-0.001	-0.007	-0.001	-0.001	-0.001	-0.004	-0.003	-0.017
86.H	-0.025	0.000	-0.001	-0.000	-0.026	0.013	0.001	0.001	-0.021

87.O	0.000	0.000	0.000	0.000	0.000	0.000	0.000	0.000	0.000
88.H	-0.019	-0.001	0.016	-0.000	-0.020	0.008	0.000	0.000	-0.023
89.H	-0.018	0.001	-0.013	-0.000	-0.019	0.010	-0.000	-0.000	-0.018
90.H	-0.012	0.000	-0.010	-0.001	-0.013	0.011	-0.001	-0.001	-0.019
91.H	-0.017	0.000	-0.006	-0.000	-0.018	0.008	-0.000	-0.000	-0.019
92.C	0.000	0.000	0.000	0.000	0.000	0.000	0.000	0.000	0.000
93.O	0.000	0.000	0.000	0.000	0.000	0.000	0.000	0.000	0.000
94.H	-0.029	-0.001	0.016	0.001	-0.028	-0.000	0.002	0.002	-0.022
95.H	-0.024	-0.001	0.021	0.000	-0.023	-0.000	0.001	0.001	-0.023
96.H	-0.029	-0.001	0.019	0.000	-0.028	0.005	0.002	0.002	-0.024

	133			134			135		
1.O	0.000	0.000	0.000	0.000	0.000	0.000	0.000	0.000	0.000
2.C	0.000	0.000	0.000	0.000	0.000	0.000	0.000	0.000	0.000
3.C	0.000	0.000	0.000	0.000	0.000	0.000	0.000	0.000	0.000
4.C	0.000	0.000	0.000	0.000	0.000	0.000	0.000	0.000	0.000
5.O	0.000	0.000	0.000	0.000	0.000	0.000	0.000	0.000	0.000
6.N	0.000	0.000	0.000	0.000	0.000	0.000	0.000	0.000	0.000
7.O	0.000	0.000	0.000	0.000	0.000	0.000	0.000	0.000	0.000
8.C	0.000	0.000	0.000	0.000	0.000	0.000	0.000	0.000	0.000
9.C	0.000	0.000	0.000	0.000	0.000	0.000	0.000	0.000	0.000
10.C	0.000	0.000	0.000	0.000	0.000	0.000	0.000	0.000	0.000
11.O	0.000	0.000	0.000	0.000	0.000	0.000	0.000	0.000	0.000
12.C	0.000	0.000	0.000	0.000	0.000	0.000	0.000	0.000	0.000
13.O	0.000	0.000	0.000	0.000	0.000	0.000	0.000	0.000	0.000
14.C	0.000	0.000	0.000	0.000	0.000	0.000	0.000	0.000	0.000
15.C	0.000	0.000	0.000	0.000	0.000	0.000	0.000	0.000	0.000
16.O	0.000	0.000	0.000	0.000	0.000	0.000	0.000	0.000	0.000
17.C	0.000	0.000	0.000	0.000	0.000	0.000	0.000	0.000	0.000

18.C	0.000	0.000	0.000	0.000	0.000	0.000	0.000	0.000	0.000
19.O	0.000	0.000	0.000	0.000	0.000	0.000	0.000	0.000	0.000
20.C	0.000	0.000	0.000	0.000	0.000	0.000	0.000	0.000	0.000
21.C	0.000	0.000	0.000	0.000	0.000	0.000	0.000	0.000	0.000
22.C	0.000	0.000	0.000	0.000	0.000	0.000	0.000	0.000	0.000
23.C	0.000	0.000	0.000	0.000	0.000	0.000	0.000	0.000	0.000
24.C	0.000	0.000	0.000	0.000	0.000	0.000	0.000	0.000	0.000
25.C	0.000	0.000	0.000	0.000	0.000	0.000	0.000	0.000	0.000
26.C	0.000	0.000	0.000	0.000	0.000	0.000	0.000	0.000	0.000
27.C	0.000	0.000	0.000	0.000	0.000	0.000	0.000	0.000	0.000
28.C	0.000	0.000	0.000	0.000	0.000	0.000	0.000	0.000	0.000
29.C	0.000	0.000	0.000	0.000	0.000	0.000	0.000	0.000	0.000
30.C	0.000	0.000	0.000	0.000	0.000	0.000	0.000	0.000	0.000
31.O	0.000	0.000	0.000	0.000	0.000	0.000	0.000	0.000	0.000
32.C	0.000	0.000	0.000	0.000	0.000	0.000	0.000	0.000	0.000
33.C	0.000	0.000	0.000	0.000	0.000	0.000	0.000	0.000	0.000
34.C	0.000	0.000	0.000	0.000	0.000	0.000	0.000	0.000	0.000
35.C	0.000	0.000	0.000	0.000	0.000	0.000	0.000	0.000	0.000
36.C	0.000	0.000	0.000	0.000	0.000	0.000	0.000	0.000	0.000
37.C	0.000	0.000	0.000	0.000	0.000	0.000	0.000	0.000	0.000
38.C	0.000	0.000	0.000	0.000	0.000	0.000	0.000	0.000	0.000
39.C	0.000	0.000	0.000	0.000	0.000	0.000	0.000	0.000	0.000
40.H	0.000	0.000	0.000	0.000	0.000	0.000	0.000	0.000	0.000
41.H	0.984	-0.000	-0.006	0.000	0.977	0.005	0.000	0.000	0.982
42.H	-0.025	-0.000	-0.005	-0.006	-0.040	0.009	-0.007	0.006	-0.030
43.H	-0.027	0.000	-0.007	0.001	-0.046	0.006	-0.011	0.007	-0.031
44.H	-0.025	0.000	-0.001	0.021	-0.032	-0.010	-0.015	0.004	-0.018
45.H	-0.028	0.000	0.001	0.020	-0.032	-0.010	-0.019	0.004	-0.016
46.H	-0.025	0.000	0.003	0.020	-0.024	-0.012	-0.015	0.002	-0.012
47.H	-0.026	0.000	0.002	0.009	-0.024	-0.005	-0.012	0.002	-0.015
48.H	-0.021	0.000	-0.002	0.011	-0.027	-0.005	-0.006	0.002	-0.021

49.H	-0.022	0.000	-0.001	0.004	-0.025	-0.001	-0.005	0.002	-0.020
50.H	-0.018	-0.000	0.000	0.016	-0.018	-0.011	-0.003	-0.000	-0.016
51.H	-0.018	0.000	0.004	0.017	-0.009	-0.014	-0.003	-0.003	-0.010
52.H	-0.021	0.000	0.002	0.020	-0.018	-0.013	-0.008	-0.000	-0.012
53.H	-0.022	0.000	0.007	0.018	-0.008	-0.015	-0.008	-0.003	-0.005
54.H	-0.019	0.000	0.008	0.011	-0.002	-0.012	-0.003	-0.004	-0.005
55.H	-0.022	0.001	0.009	0.012	-0.005	-0.011	-0.008	-0.003	-0.003
56.H	-0.029	0.001	0.004	-0.007	-0.025	0.006	-0.012	0.002	-0.016
57.H	-0.029	0.000	0.002	-0.018	-0.026	0.014	-0.008	0.003	-0.020
58.H	-0.025	0.000	0.003	-0.020	-0.020	0.013	-0.003	0.001	-0.019
59.H	-0.026	0.001	0.008	-0.017	-0.010	0.008	-0.004	-0.002	-0.011
60.H	-0.029	0.001	0.007	-0.015	-0.016	0.009	-0.010	0.000	-0.011
61.H	-0.027	0.001	0.007	-0.023	-0.014	0.013	-0.005	-0.001	-0.014
62.H	-0.030	0.000	-0.002	-0.014	-0.037	0.014	-0.011	0.006	-0.026
63.H	-0.031	0.000	-0.001	-0.006	-0.038	0.009	-0.016	0.006	-0.023
64.H	-0.029	0.000	-0.004	-0.008	-0.042	0.011	-0.012	0.007	-0.028
65.H	-0.020	0.000	0.002	-0.003	-0.016	0.001	-0.000	-0.001	-0.017
66.H	-0.014	-0.000	0.004	-0.008	-0.003	0.001	0.009	-0.005	-0.014
67.H	-0.016	-0.000	0.000	-0.008	-0.013	0.004	0.008	-0.002	-0.020
68.H	-0.017	0.000	0.003	-0.012	-0.008	0.005	0.007	-0.003	-0.017
69.C	0.000	0.000	0.000	0.000	0.000	0.000	0.000	0.000	0.000
70.H	-0.011	-0.000	-0.001	0.018	-0.011	-0.014	0.008	-0.003	-0.017
71.H	-0.007	-0.000	-0.000	0.014	-0.004	-0.013	0.014	-0.005	-0.016
72.H	-0.010	-0.000	0.002	0.015	-0.002	-0.014	0.009	-0.005	-0.012
73.H	-0.009	-0.000	0.000	0.004	-0.004	-0.007	0.014	-0.005	-0.017
74.H	-0.011	-0.001	-0.005	0.010	-0.019	-0.006	0.010	-0.001	-0.025
75.H	-0.007	-0.001	-0.004	0.005	-0.012	-0.005	0.016	-0.003	-0.024
76.H	-0.014	-0.001	-0.010	0.001	-0.034	0.003	0.008	0.003	-0.034
77.H	-0.012	-0.001	-0.009	0.007	-0.030	-0.001	0.009	0.002	-0.032
78.H	-0.011	-0.001	-0.011	0.000	-0.034	0.003	0.012	0.003	-0.037
79.H	-0.012	-0.001	-0.008	-0.011	-0.026	0.009	0.014	0.001	-0.034

80.H	-0.008	-0.001	-0.007	-0.012	-0.018	0.007	0.019	-0.001	-0.031
81.H	-0.009	-0.001	-0.010	-0.010	-0.027	0.008	0.018	0.001	-0.037
82.H	-0.013	-0.000	0.000	-0.001	-0.009	-0.002	0.010	-0.003	-0.018
83.H	-0.016	0.000	0.004	0.005	-0.007	-0.007	0.003	-0.003	-0.012
84.H	-0.015	-0.000	-0.005	0.005	-0.025	-0.001	0.005	0.001	-0.026
85.H	-0.010	-0.001	-0.003	-0.004	-0.013	0.001	0.015	-0.003	-0.025
86.H	-0.023	0.000	-0.000	-0.013	-0.025	0.010	-0.002	0.002	-0.023
87.O	0.000	0.000	0.000	0.000	0.000	0.000	0.000	0.000	0.000
88.H	-0.020	0.000	0.008	-0.010	-0.002	0.002	0.001	-0.004	-0.008
89.H	-0.019	-0.000	-0.007	-0.011	-0.034	0.011	0.003	0.004	-0.032
90.H	-0.016	-0.000	-0.005	-0.015	-0.025	0.011	0.008	0.001	-0.030
91.H	-0.019	-0.000	-0.003	-0.010	-0.026	0.008	0.003	0.002	-0.026
92.C	0.000	0.000	0.000	0.000	0.000	0.000	0.000	0.000	0.000
93.O	0.000	0.000	0.000	0.000	0.000	0.000	0.000	0.000	0.000
94.H	-0.024	0.001	0.008	0.003	-0.009	-0.004	-0.008	-0.002	-0.007
95.H	-0.022	0.001	0.010	0.000	0.001	-0.005	-0.004	-0.005	-0.003
96.H	-0.025	0.001	0.010	-0.004	-0.004	-0.001	-0.007	-0.003	-0.005

	136			137			138		
	------------------------			------------------------			------------------------		
1.O	0.000	0.000	0.000	0.000	0.000	0.000	0.000	0.000	0.000
2.C	0.000	0.000	0.000	0.000	0.000	0.000	0.000	0.000	0.000
3.C	0.000	0.000	0.000	0.000	0.000	0.000	0.000	0.000	0.000
4.C	0.000	0.000	0.000	0.000	0.000	0.000	0.000	0.000	0.000
5.O	0.000	0.000	0.000	0.000	0.000	0.000	0.000	0.000	0.000
6.N	0.000	0.000	0.000	0.000	0.000	0.000	0.000	0.000	0.000
7.O	0.000	0.000	0.000	0.000	0.000	0.000	0.000	0.000	0.000
8.C	0.000	0.000	0.000	0.000	0.000	0.000	0.000	0.000	0.000
9.C	0.000	0.000	0.000	0.000	0.000	0.000	0.000	0.000	0.000
10.C	0.000	0.000	0.000	0.000	0.000	0.000	0.000	0.000	0.000

11.O	0.000	0.000	0.000	0.000	0.000	0.000	0.000	0.000	0.000
12.C	0.000	0.000	0.000	0.000	0.000	0.000	0.000	0.000	0.000
13.O	0.000	0.000	0.000	0.000	0.000	0.000	0.000	0.000	0.000
14.C	0.000	0.000	0.000	0.000	0.000	0.000	0.000	0.000	0.000
15.C	0.000	0.000	0.000	0.000	0.000	0.000	0.000	0.000	0.000
16.O	0.000	0.000	0.000	0.000	0.000	0.000	0.000	0.000	0.000
17.C	0.000	0.000	0.000	0.000	0.000	0.000	0.000	0.000	0.000
18.C	0.000	0.000	0.000	0.000	0.000	0.000	0.000	0.000	0.000
19.O	0.000	0.000	0.000	0.000	0.000	0.000	0.000	0.000	0.000
20.C	0.000	0.000	0.000	0.000	0.000	0.000	0.000	0.000	0.000
21.C	0.000	0.000	0.000	0.000	0.000	0.000	0.000	0.000	0.000
22.C	0.000	0.000	0.000	0.000	0.000	0.000	0.000	0.000	0.000
23.C	0.000	0.000	0.000	0.000	0.000	0.000	0.000	0.000	0.000
24.C	0.000	0.000	0.000	0.000	0.000	0.000	0.000	0.000	0.000
25.C	0.000	0.000	0.000	0.000	0.000	0.000	0.000	0.000	0.000
26.C	0.000	0.000	0.000	0.000	0.000	0.000	0.000	0.000	0.000
27.C	0.000	0.000	0.000	0.000	0.000	0.000	0.000	0.000	0.000
28.C	0.000	0.000	0.000	0.000	0.000	0.000	0.000	0.000	0.000
29.C	0.000	0.000	0.000	0.000	0.000	0.000	0.000	0.000	0.000
30.C	0.000	0.000	0.000	0.000	0.000	0.000	0.000	0.000	0.000
31.O	0.000	0.000	0.000	0.000	0.000	0.000	0.000	0.000	0.000
32.C	0.000	0.000	0.000	0.000	0.000	0.000	0.000	0.000	0.000
33.C	0.000	0.000	0.000	0.000	0.000	0.000	0.000	0.000	0.000
34.C	0.000	0.000	0.000	0.000	0.000	0.000	0.000	0.000	0.000
35.C	0.000	0.000	0.000	0.000	0.000	0.000	0.000	0.000	0.000
36.C	0.000	0.000	0.000	0.000	0.000	0.000	0.000	0.000	0.000
37.C	0.000	0.000	0.000	0.000	0.000	0.000	0.000	0.000	0.000
38.C	0.000	0.000	0.000	0.000	0.000	0.000	0.000	0.000	0.000
39.C	0.000	0.000	0.000	0.000	0.000	0.000	0.000	0.000	0.000
40.H	0.000	0.000	0.000	0.000	0.000	0.000	0.000	0.000	0.000
41.H	0.000	0.000	0.000	0.000	0.000	0.000	0.000	0.000	0.000

42.H	0.981	-0.006	-0.006	0.000	0.975	0.012	0.000	0.000	0.979
43.H	-0.030	-0.008	-0.008	0.001	-0.050	0.007	-0.011	0.016	-0.033
44.H	-0.020	-0.002	-0.003	0.022	-0.035	-0.014	-0.017	0.010	-0.012
45.H	-0.025	-0.001	-0.001	0.021	-0.037	-0.012	-0.020	0.012	-0.011
46.H	-0.020	0.001	0.002	0.021	-0.027	-0.015	-0.016	0.007	-0.007
47.H	-0.025	0.001	0.002	0.009	-0.028	-0.006	-0.013	0.007	-0.014
48.H	-0.018	-0.002	-0.003	0.012	-0.029	-0.007	-0.007	0.005	-0.018
49.H	-0.022	-0.001	-0.001	0.004	-0.027	-0.002	-0.005	0.004	-0.020
50.H	-0.012	0.000	-0.001	0.016	-0.018	-0.014	-0.005	-0.001	-0.010
51.H	-0.011	0.004	0.003	0.017	-0.010	-0.017	-0.005	-0.004	-0.004
52.H	-0.014	0.001	0.001	0.020	-0.020	-0.017	-0.010	0.001	-0.006
53.H	-0.016	0.006	0.007	0.018	-0.009	-0.018	-0.010	-0.002	0.000
54.H	-0.015	0.007	0.009	0.011	-0.002	-0.014	-0.004	-0.006	-0.001
55.H	-0.020	0.008	0.010	0.012	-0.007	-0.014	-0.009	-0.002	-0.000
56.H	-0.037	0.002	0.005	-0.007	-0.030	0.009	-0.011	0.010	-0.021
57.H	-0.041	0.001	0.005	-0.019	-0.031	0.018	-0.006	0.010	-0.029
58.H	-0.036	0.002	0.005	-0.020	-0.024	0.017	-0.001	0.005	-0.028
59.H	-0.036	0.007	0.011	-0.017	-0.013	0.012	-0.002	0.002	-0.019
60.H	-0.040	0.006	0.011	-0.016	-0.021	0.013	-0.008	0.006	-0.020
61.H	-0.041	0.006	0.010	-0.023	-0.018	0.019	-0.002	0.004	-0.024
62.H	-0.041	-0.003	-0.000	-0.014	-0.043	0.018	-0.009	0.015	-0.034
63.H	-0.040	-0.002	-0.000	-0.006	-0.044	0.012	-0.015	0.016	-0.029
64.H	-0.038	-0.005	-0.004	-0.008	-0.047	0.014	-0.011	0.016	-0.034
65.H	-0.022	0.002	0.002	-0.003	-0.017	0.002	-0.000	-0.000	-0.019
66.H	-0.015	0.004	0.005	-0.008	-0.002	0.001	0.010	-0.009	-0.016
67.H	-0.018	0.001	0.001	-0.009	-0.012	0.005	0.008	-0.005	-0.023
68.H	-0.021	0.003	0.004	-0.013	-0.007	0.007	0.008	-0.006	-0.021
69.C	0.000	0.000	0.000	0.000	0.000	0.000	0.000	0.000	0.000
70.H	0.000	-0.001	-0.004	0.018	-0.008	-0.018	0.005	-0.009	-0.010
71.H	0.003	0.001	-0.002	0.014	0.000	-0.017	0.011	-0.014	-0.009
72.H	0.000	0.003	0.001	0.015	0.001	-0.019	0.007	-0.013	-0.005

73.H	-0.003	0.002	-0.001	0.004	-0.000	-0.009	0.013	-0.013	-0.014
74.H	-0.003	-0.004	-0.008	0.010	-0.016	-0.009	0.008	-0.006	-0.020
75.H	-0.000	-0.003	-0.006	0.005	-0.008	-0.007	0.015	-0.011	-0.020
76.H	-0.011	-0.009	-0.012	0.000	-0.032	0.003	0.007	0.001	-0.034
77.H	-0.006	-0.008	-0.012	0.006	-0.028	-0.003	0.008	-0.001	-0.029
78.H	-0.007	-0.010	-0.015	0.000	-0.031	0.003	0.012	-0.001	-0.036
79.H	-0.013	-0.007	-0.010	-0.011	-0.024	0.010	0.015	-0.003	-0.037
80.H	-0.008	-0.005	-0.008	-0.012	-0.014	0.008	0.020	-0.009	-0.034
81.H	-0.008	-0.008	-0.012	-0.011	-0.023	0.010	0.019	-0.005	-0.039
82.H	-0.011	0.001	0.000	-0.001	-0.007	-0.003	0.009	-0.008	-0.018
83.H	-0.013	0.004	0.004	0.005	-0.006	-0.008	0.002	-0.007	-0.010
84.H	-0.011	-0.005	-0.007	0.004	-0.024	-0.003	0.004	-0.001	-0.024
85.H	-0.007	-0.002	-0.005	-0.005	-0.010	0.001	0.015	-0.010	-0.025
86.H	-0.031	-0.001	0.001	-0.013	-0.027	0.013	-0.001	0.005	-0.029
87.O	0.000	0.000	0.000	0.000	0.000	0.000	0.000	0.000	0.000
88.H	-0.025	0.008	0.010	-0.010	-0.003	0.004	0.002	-0.005	-0.013
89.H	-0.025	-0.006	-0.007	-0.012	-0.035	0.014	0.004	0.006	-0.037
90.H	-0.022	-0.004	-0.005	-0.015	-0.025	0.014	0.010	0.000	-0.035
91.H	-0.024	-0.003	-0.003	-0.010	-0.026	0.010	0.004	0.002	-0.030
92.C	0.000	0.000	0.000	0.000	0.000	0.000	0.000	0.000	0.000
93.O	0.000	0.000	0.000	0.000	0.000	0.000	0.000	0.000	0.000
94.H	-0.026	0.007	0.010	0.003	-0.012	-0.005	-0.008	0.001	-0.007
95.H	-0.024	0.010	0.013	0.000	-0.001	-0.005	-0.004	-0.005	-0.004
96.H	-0.029	0.009	0.012	-0.004	-0.007	-0.000	-0.006	-0.001	-0.008

	139			140			141		
	------------------------			------------------------			------------------------		
1.O	0.000	0.000	0.000	0.000	0.000	0.000	0.000	0.000	0.000
2.C	0.000	0.000	0.000	0.000	0.000	0.000	0.000	0.000	0.000
3.C	0.000	0.000	0.000	0.000	0.000	0.000	0.000	0.000	0.000

4.C	0.000	0.000	0.000	0.000	0.000	0.000	0.000	0.000	0.000
5.O	0.000	0.000	0.000	0.000	0.000	0.000	0.000	0.000	0.000
6.N	0.000	0.000	0.000	0.000	0.000	0.000	0.000	0.000	0.000
7.O	0.000	0.000	0.000	0.000	0.000	0.000	0.000	0.000	0.000
8.C	0.000	0.000	0.000	0.000	0.000	0.000	0.000	0.000	0.000
9.C	0.000	0.000	0.000	0.000	0.000	0.000	0.000	0.000	0.000
10.C	0.000	0.000	0.000	0.000	0.000	0.000	0.000	0.000	0.000
11.O	0.000	0.000	0.000	0.000	0.000	0.000	0.000	0.000	0.000
12.C	0.000	0.000	0.000	0.000	0.000	0.000	0.000	0.000	0.000
13.O	0.000	0.000	0.000	0.000	0.000	0.000	0.000	0.000	0.000
14.C	0.000	0.000	0.000	0.000	0.000	0.000	0.000	0.000	0.000
15.C	0.000	0.000	0.000	0.000	0.000	0.000	0.000	0.000	0.000
16.O	0.000	0.000	0.000	0.000	0.000	0.000	0.000	0.000	0.000
17.C	0.000	0.000	0.000	0.000	0.000	0.000	0.000	0.000	0.000
18.C	0.000	0.000	0.000	0.000	0.000	0.000	0.000	0.000	0.000
19.O	0.000	0.000	0.000	0.000	0.000	0.000	0.000	0.000	0.000
20.C	0.000	0.000	0.000	0.000	0.000	0.000	0.000	0.000	0.000
21.C	0.000	0.000	0.000	0.000	0.000	0.000	0.000	0.000	0.000
22.C	0.000	0.000	0.000	0.000	0.000	0.000	0.000	0.000	0.000
23.C	0.000	0.000	0.000	0.000	0.000	0.000	0.000	0.000	0.000
24.C	0.000	0.000	0.000	0.000	0.000	0.000	0.000	0.000	0.000
25.C	0.000	0.000	0.000	0.000	0.000	0.000	0.000	0.000	0.000
26.C	0.000	0.000	0.000	0.000	0.000	0.000	0.000	0.000	0.000
27.C	0.000	0.000	0.000	0.000	0.000	0.000	0.000	0.000	0.000
28.C	0.000	0.000	0.000	0.000	0.000	0.000	0.000	0.000	0.000
29.C	0.000	0.000	0.000	0.000	0.000	0.000	0.000	0.000	0.000
30.C	0.000	0.000	0.000	0.000	0.000	0.000	0.000	0.000	0.000
31.O	0.000	0.000	0.000	0.000	0.000	0.000	0.000	0.000	0.000
32.C	0.000	0.000	0.000	0.000	0.000	0.000	0.000	0.000	0.000
33.C	0.000	0.000	0.000	0.000	0.000	0.000	0.000	0.000	0.000
34.C	0.000	0.000	0.000	0.000	0.000	0.000	0.000	0.000	0.000

35.C	0.000	0.000	0.000	0.000	0.000	0.000	0.000	0.000	0.000
36.C	0.000	0.000	0.000	0.000	0.000	0.000	0.000	0.000	0.000
37.C	0.000	0.000	0.000	0.000	0.000	0.000	0.000	0.000	0.000
38.C	0.000	0.000	0.000	0.000	0.000	0.000	0.000	0.000	0.000
39.C	0.000	0.000	0.000	0.000	0.000	0.000	0.000	0.000	0.000
40.H	0.000	0.000	0.000	0.000	0.000	0.000	0.000	0.000	0.000
41.H	0.000	0.000	0.000	0.000	0.000	0.000	0.000	0.000	0.000
42.H	0.000	0.000	0.000	0.000	0.000	0.000	0.000	0.000	0.000
43.H	0.979	0.002	-0.014	0.000	0.965	0.010	0.000	0.000	0.979
44.H	-0.034	0.001	-0.002	0.029	-0.042	-0.018	-0.019	0.006	-0.018
45.H	-0.040	0.001	0.001	0.028	-0.045	-0.017	-0.024	0.007	-0.016
46.H	-0.034	0.001	0.005	0.028	-0.031	-0.021	-0.019	0.003	-0.011
47.H	-0.034	0.001	0.004	0.013	-0.033	-0.007	-0.016	0.003	-0.015
48.H	-0.026	0.001	-0.004	0.016	-0.034	-0.009	-0.008	0.004	-0.022
49.H	-0.026	0.000	-0.002	0.006	-0.030	-0.002	-0.006	0.003	-0.021
50.H	-0.019	-0.000	0.001	0.022	-0.019	-0.019	-0.004	-0.000	-0.015
51.H	-0.019	-0.001	0.008	0.023	-0.007	-0.023	-0.004	-0.003	-0.008
52.H	-0.025	0.000	0.005	0.028	-0.021	-0.023	-0.011	0.000	-0.011
53.H	-0.027	-0.000	0.014	0.025	-0.007	-0.025	-0.011	-0.003	-0.002
54.H	-0.021	-0.001	0.016	0.015	0.003	-0.019	-0.004	-0.006	-0.002
55.H	-0.028	-0.001	0.017	0.016	-0.003	-0.018	-0.011	-0.004	-0.000
56.H	-0.039	0.001	0.006	-0.009	-0.035	0.012	-0.016	0.004	-0.017
57.H	-0.037	0.001	0.003	-0.025	-0.036	0.026	-0.010	0.004	-0.023
58.H	-0.030	0.000	0.004	-0.027	-0.026	0.024	-0.004	0.002	-0.022
59.H	-0.031	-0.000	0.014	-0.023	-0.012	0.017	-0.006	-0.002	-0.011
60.H	-0.038	0.000	0.014	-0.021	-0.023	0.018	-0.013	0.001	-0.012
61.H	-0.034	0.000	0.012	-0.031	-0.018	0.026	-0.006	-0.000	-0.015
62.H	-0.040	0.002	-0.004	-0.019	-0.052	0.025	-0.014	0.008	-0.030
63.H	-0.044	0.002	-0.003	-0.008	-0.055	0.016	-0.020	0.009	-0.026
64.H	-0.040	0.002	-0.009	-0.011	-0.059	0.020	-0.016	0.010	-0.032
65.H	-0.022	-0.000	0.003	-0.004	-0.018	0.003	-0.001	-0.000	-0.018

66.H	-0.010	-0.001	0.007	-0.011	0.004	0.002	0.012	-0.006	-0.014
67.H	-0.012	-0.001	0.000	-0.012	-0.010	0.007	0.010	-0.003	-0.022
68.H	-0.014	-0.001	0.005	-0.017	-0.004	0.010	0.009	-0.004	-0.018
69.C	0.000	0.000	0.000	0.000	0.000	0.000	0.000	0.000	0.000
70.H	-0.005	-0.001	-0.002	0.024	-0.005	-0.025	0.009	-0.004	-0.017
71.H	0.002	-0.002	-0.001	0.018	0.007	-0.023	0.016	-0.007	-0.016
72.H	-0.004	-0.001	0.004	0.020	0.007	-0.025	0.011	-0.007	-0.011
73.H	-0.001	-0.001	0.001	0.005	0.006	-0.012	0.017	-0.007	-0.017
74.H	-0.004	-0.001	-0.010	0.013	-0.016	-0.012	0.012	-0.001	-0.027
75.H	0.002	-0.001	-0.009	0.006	-0.005	-0.010	0.019	-0.004	-0.026
76.H	-0.010	0.000	-0.020	0.000	-0.037	0.005	0.009	0.004	-0.039
77.H	-0.007	0.000	-0.018	0.009	-0.032	-0.004	0.011	0.003	-0.036
78.H	-0.004	0.000	-0.023	0.000	-0.036	0.004	0.015	0.004	-0.043
79.H	-0.004	-0.000	-0.017	-0.015	-0.026	0.015	0.018	0.001	-0.039
80.H	0.002	-0.001	-0.014	-0.017	-0.013	0.012	0.024	-0.002	-0.036
81.H	0.001	-0.000	-0.021	-0.015	-0.025	0.014	0.023	0.001	-0.042
82.H	-0.008	-0.001	0.000	-0.002	-0.003	-0.003	0.012	-0.004	-0.019
83.H	-0.015	-0.001	0.007	0.007	-0.002	-0.011	0.003	-0.004	-0.011
84.H	-0.012	0.000	-0.010	0.006	-0.026	-0.003	0.006	0.001	-0.029
85.H	-0.001	-0.001	-0.007	-0.006	-0.007	0.002	0.019	-0.004	-0.027
86.H	-0.027	0.001	-0.001	-0.018	-0.031	0.018	-0.003	0.003	-0.026
87.O	0.000	0.000	0.000	0.000	0.000	0.000	0.000	0.000	0.000
88.H	-0.021	-0.001	0.015	-0.014	0.002	0.005	0.002	-0.005	-0.007
89.H	-0.020	0.001	-0.014	-0.016	-0.041	0.019	0.003	0.005	-0.037
90.H	-0.014	0.000	-0.011	-0.021	-0.028	0.019	0.010	0.002	-0.034
91.H	-0.019	0.000	-0.007	-0.014	-0.030	0.015	0.004	0.003	-0.030
92.C	0.000	0.000	0.000	0.000	0.000	0.000	0.000	0.000	0.000
93.O	0.000	0.000	0.000	0.000	0.000	0.000	0.000	0.000	0.000
94.H	-0.031	-0.000	0.015	0.004	-0.010	-0.006	-0.011	-0.002	-0.005
95.H	-0.026	-0.001	0.021	0.000	0.004	-0.007	-0.006	-0.006	0.000
96.H	-0.031	-0.000	0.019	-0.005	-0.004	-0.000	-0.009	-0.004	-0.003

	142			143			144		
	------------------------			------------------------			------------------------		
1.O	0.000	0.000	0.000	0.000	0.000	0.000	0.000	0.000	0.000
2.C	0.000	0.000	0.000	0.000	0.000	0.000	0.000	0.000	0.000
3.C	0.000	0.000	0.000	0.000	0.000	0.000	0.000	0.000	0.000
4.C	0.000	0.000	0.000	0.000	0.000	0.000	0.000	0.000	0.000
5.O	0.000	0.000	0.000	0.000	0.000	0.000	0.000	0.000	0.000
6.N	0.000	0.000	0.000	0.000	0.000	0.000	0.000	0.000	0.000
7.O	0.000	0.000	0.000	0.000	0.000	0.000	0.000	0.000	0.000
8.C	0.000	0.000	0.000	0.000	0.000	0.000	0.000	0.000	0.000
9.C	0.000	0.000	0.000	0.000	0.000	0.000	0.000	0.000	0.000
10.C	0.000	0.000	0.000	0.000	0.000	0.000	0.000	0.000	0.000
11.O	0.000	0.000	0.000	0.000	0.000	0.000	0.000	0.000	0.000
12.C	0.000	0.000	0.000	0.000	0.000	0.000	0.000	0.000	0.000
13.O	0.000	0.000	0.000	0.000	0.000	0.000	0.000	0.000	0.000
14.C	0.000	0.000	0.000	0.000	0.000	0.000	0.000	0.000	0.000
15.C	0.000	0.000	0.000	0.000	0.000	0.000	0.000	0.000	0.000
16.O	0.000	0.000	0.000	0.000	0.000	0.000	0.000	0.000	0.000
17.C	0.000	0.000	0.000	0.000	0.000	0.000	0.000	0.000	0.000
18.C	0.000	0.000	0.000	0.000	0.000	0.000	0.000	0.000	0.000
19.O	0.000	0.000	0.000	0.000	0.000	0.000	0.000	0.000	0.000
20.C	0.000	0.000	0.000	0.000	0.000	0.000	0.000	0.000	0.000
21.C	0.000	0.000	0.000	0.000	0.000	0.000	0.000	0.000	0.000
22.C	0.000	0.000	0.000	0.000	0.000	0.000	0.000	0.000	0.000
23.C	0.000	0.000	0.000	0.000	0.000	0.000	0.000	0.000	0.000
24.C	0.000	0.000	0.000	0.000	0.000	0.000	0.000	0.000	0.000
25.C	0.000	0.000	0.000	0.000	0.000	0.000	0.000	0.000	0.000
26.C	0.000	0.000	0.000	0.000	0.000	0.000	0.000	0.000	0.000
27.C	0.000	0.000	0.000	0.000	0.000	0.000	0.000	0.000	0.000

28.C	0.000	0.000	0.000	0.000	0.000	0.000	0.000	0.000	0.000
29.C	0.000	0.000	0.000	0.000	0.000	0.000	0.000	0.000	0.000
30.C	0.000	0.000	0.000	0.000	0.000	0.000	0.000	0.000	0.000
31.O	0.000	0.000	0.000	0.000	0.000	0.000	0.000	0.000	0.000
32.C	0.000	0.000	0.000	0.000	0.000	0.000	0.000	0.000	0.000
33.C	0.000	0.000	0.000	0.000	0.000	0.000	0.000	0.000	0.000
34.C	0.000	0.000	0.000	0.000	0.000	0.000	0.000	0.000	0.000
35.C	0.000	0.000	0.000	0.000	0.000	0.000	0.000	0.000	0.000
36.C	0.000	0.000	0.000	0.000	0.000	0.000	0.000	0.000	0.000
37.C	0.000	0.000	0.000	0.000	0.000	0.000	0.000	0.000	0.000
38.C	0.000	0.000	0.000	0.000	0.000	0.000	0.000	0.000	0.000
39.C	0.000	0.000	0.000	0.000	0.000	0.000	0.000	0.000	0.000
40.H	0.000	0.000	0.000	0.000	0.000	0.000	0.000	0.000	0.000
41.H	0.000	0.000	0.000	0.000	0.000	0.000	0.000	0.000	0.000
42.H	0.000	0.000	0.000	0.000	0.000	0.000	0.000	0.000	0.000
43.H	0.000	0.000	0.000	0.000	0.000	0.000	0.000	0.000	0.000
44.H	0.965	0.012	0.003	0.000	0.978	-0.014	0.000	0.000	0.978
45.H	-0.067	0.009	0.006	0.012	-0.040	-0.012	0.002	-0.019	-0.033
46.H	-0.063	-0.000	0.012	0.012	-0.031	-0.015	0.003	-0.012	-0.035
47.H	-0.047	0.001	0.006	0.006	-0.032	-0.005	0.000	-0.013	-0.025
48.H	-0.042	0.010	-0.002	0.007	-0.029	-0.007	0.002	-0.007	-0.030
49.H	-0.032	0.006	-0.002	0.003	-0.027	-0.002	0.000	-0.006	-0.023
50.H	-0.043	0.000	0.006	0.009	-0.020	-0.014	0.004	0.001	-0.035
51.H	-0.044	-0.012	0.016	0.009	-0.015	-0.016	0.005	0.003	-0.035
52.H	-0.055	-0.003	0.012	0.012	-0.023	-0.017	0.004	-0.004	-0.037
53.H	-0.052	-0.019	0.024	0.010	-0.018	-0.017	0.004	-0.002	-0.034
54.H	-0.038	-0.025	0.024	0.006	-0.012	-0.013	0.003	0.003	-0.029
55.H	-0.044	-0.024	0.026	0.007	-0.017	-0.012	0.002	-0.003	-0.027
56.H	-0.028	-0.002	0.003	-0.003	-0.036	0.010	-0.005	-0.018	-0.008
57.H	-0.009	0.001	-0.004	-0.009	-0.036	0.020	-0.008	-0.017	0.001
58.H	-0.002	-0.003	-0.003	-0.010	-0.028	0.019	-0.007	-0.010	0.000

59.H	-0.007	-0.020	0.011	-0.008	-0.023	0.015	-0.006	-0.008	-0.001
60.H	-0.015	-0.015	0.010	-0.007	-0.031	0.015	-0.007	-0.016	-0.001
61.H	0.000	-0.016	0.006	-0.012	-0.027	0.021	-0.008	-0.012	0.005
62.H	-0.018	0.016	-0.013	-0.006	-0.043	0.019	-0.007	-0.022	-0.003
63.H	-0.033	0.015	-0.008	-0.002	-0.046	0.012	-0.005	-0.025	-0.009
64.H	-0.027	0.023	-0.017	-0.003	-0.046	0.014	-0.005	-0.023	-0.009
65.H	-0.018	-0.004	0.002	-0.001	-0.021	0.002	-0.001	-0.001	-0.017
66.H	-0.001	-0.016	0.007	-0.005	-0.007	0.003	-0.000	0.011	-0.016
67.H	-0.002	-0.004	-0.002	-0.005	-0.013	0.005	-0.001	0.007	-0.015
68.H	0.002	-0.012	0.003	-0.007	-0.012	0.008	-0.002	0.007	-0.011
69.C	0.000	0.000	0.000	0.000	0.000	0.000	0.000	0.000	0.000
70.H	-0.033	-0.001	0.004	0.009	-0.007	-0.019	0.007	0.015	-0.041
71.H	-0.021	-0.007	0.006	0.007	0.000	-0.017	0.007	0.022	-0.039
72.H	-0.028	-0.013	0.012	0.008	-0.002	-0.019	0.007	0.018	-0.039
73.H	-0.010	-0.009	0.004	0.001	-0.002	-0.009	0.004	0.019	-0.029
74.H	-0.021	0.011	-0.009	0.005	-0.012	-0.010	0.005	0.013	-0.035
75.H	-0.008	0.005	-0.008	0.002	-0.004	-0.008	0.005	0.020	-0.032
76.H	-0.013	0.029	-0.025	-0.000	-0.023	0.001	0.002	0.004	-0.026
77.H	-0.018	0.026	-0.021	0.003	-0.019	-0.005	0.004	0.008	-0.032
78.H	-0.007	0.033	-0.030	-0.000	-0.020	0.001	0.003	0.009	-0.027
79.H	0.009	0.021	-0.025	-0.007	-0.016	0.009	-0.000	0.010	-0.017
80.H	0.016	0.013	-0.021	-0.008	-0.008	0.008	0.001	0.018	-0.017
81.H	0.013	0.025	-0.030	-0.007	-0.013	0.008	0.001	0.015	-0.019
82.H	-0.009	-0.006	0.001	-0.001	-0.008	-0.002	0.002	0.012	-0.023
83.H	-0.024	-0.014	0.012	0.003	-0.011	-0.007	0.002	0.007	-0.026
84.H	-0.020	0.015	-0.012	0.002	-0.020	-0.004	0.002	0.005	-0.028
85.H	0.002	0.004	-0.010	-0.003	-0.007	0.000	0.002	0.017	-0.023
86.H	-0.008	0.005	-0.008	-0.007	-0.029	0.014	-0.005	-0.008	-0.007
87.O	0.000	0.000	0.000	0.000	0.000	0.000	0.000	0.000	0.000
88.H	-0.008	-0.025	0.016	-0.005	-0.013	0.006	-0.003	0.002	-0.010
89.H	-0.005	0.023	-0.023	-0.006	-0.030	0.013	-0.003	-0.005	-0.012

90.H	0.006	0.014	-0.019	-0.008	-0.021	0.014	-0.003	0.003	-0.010
91.H	-0.006	0.011	-0.013	-0.005	-0.025	0.010	-0.002	-0.002	-0.012
92.C	0.000	0.000	0.000	0.000	0.000	0.000	0.000	0.000	0.000
93.O	0.000	0.000	0.000	0.000	0.000	0.000	0.000	0.000	0.000
94.H	-0.034	-0.020	0.020	0.002	-0.022	-0.003	-0.001	-0.007	-0.019
95.H	-0.026	-0.031	0.026	0.001	-0.014	-0.003	-0.001	-0.001	-0.017
96.H	-0.024	-0.027	0.022	-0.001	-0.020	0.002	-0.003	-0.006	-0.012

	145			146			147		
1.O	0.000	0.000	0.000	0.000	0.000	0.000	0.000	0.000	0.000
2.C	0.000	0.000	0.000	0.000	0.000	0.000	0.000	0.000	0.000
3.C	0.000	0.000	0.000	0.000	0.000	0.000	0.000	0.000	0.000
4.C	0.000	0.000	0.000	0.000	0.000	0.000	0.000	0.000	0.000
5.O	0.000	0.000	0.000	0.000	0.000	0.000	0.000	0.000	0.000
6.N	0.000	0.000	0.000	0.000	0.000	0.000	0.000	0.000	0.000
7.O	0.000	0.000	0.000	0.000	0.000	0.000	0.000	0.000	0.000
8.C	0.000	0.000	0.000	0.000	0.000	0.000	0.000	0.000	0.000
9.C	0.000	0.000	0.000	0.000	0.000	0.000	0.000	0.000	0.000
10.C	0.000	0.000	0.000	0.000	0.000	0.000	0.000	0.000	0.000
11.O	0.000	0.000	0.000	0.000	0.000	0.000	0.000	0.000	0.000
12.C	0.000	0.000	0.000	0.000	0.000	0.000	0.000	0.000	0.000
13.O	0.000	0.000	0.000	0.000	0.000	0.000	0.000	0.000	0.000
14.C	0.000	0.000	0.000	0.000	0.000	0.000	0.000	0.000	0.000
15.C	0.000	0.000	0.000	0.000	0.000	0.000	0.000	0.000	0.000
16.O	0.000	0.000	0.000	0.000	0.000	0.000	0.000	0.000	0.000
17.C	0.000	0.000	0.000	0.000	0.000	0.000	0.000	0.000	0.000
18.C	0.000	0.000	0.000	0.000	0.000	0.000	0.000	0.000	0.000
19.O	0.000	0.000	0.000	0.000	0.000	0.000	0.000	0.000	0.000
20.C	0.000	0.000	0.000	0.000	0.000	0.000	0.000	0.000	0.000

21.C	0.000	0.000	0.000	0.000	0.000	0.000	0.000	0.000	0.000
22.C	0.000	0.000	0.000	0.000	0.000	0.000	0.000	0.000	0.000
23.C	0.000	0.000	0.000	0.000	0.000	0.000	0.000	0.000	0.000
24.C	0.000	0.000	0.000	0.000	0.000	0.000	0.000	0.000	0.000
25.C	0.000	0.000	0.000	0.000	0.000	0.000	0.000	0.000	0.000
26.C	0.000	0.000	0.000	0.000	0.000	0.000	0.000	0.000	0.000
27.C	0.000	0.000	0.000	0.000	0.000	0.000	0.000	0.000	0.000
28.C	0.000	0.000	0.000	0.000	0.000	0.000	0.000	0.000	0.000
29.C	0.000	0.000	0.000	0.000	0.000	0.000	0.000	0.000	0.000
30.C	0.000	0.000	0.000	0.000	0.000	0.000	0.000	0.000	0.000
31.O	0.000	0.000	0.000	0.000	0.000	0.000	0.000	0.000	0.000
32.C	0.000	0.000	0.000	0.000	0.000	0.000	0.000	0.000	0.000
33.C	0.000	0.000	0.000	0.000	0.000	0.000	0.000	0.000	0.000
34.C	0.000	0.000	0.000	0.000	0.000	0.000	0.000	0.000	0.000
35.C	0.000	0.000	0.000	0.000	0.000	0.000	0.000	0.000	0.000
36.C	0.000	0.000	0.000	0.000	0.000	0.000	0.000	0.000	0.000
37.C	0.000	0.000	0.000	0.000	0.000	0.000	0.000	0.000	0.000
38.C	0.000	0.000	0.000	0.000	0.000	0.000	0.000	0.000	0.000
39.C	0.000	0.000	0.000	0.000	0.000	0.000	0.000	0.000	0.000
40.H	0.000	0.000	0.000	0.000	0.000	0.000	0.000	0.000	0.000
41.H	0.000	0.000	0.000	0.000	0.000	0.000	0.000	0.000	0.000
42.H	0.000	0.000	0.000	0.000	0.000	0.000	0.000	0.000	0.000
43.H	0.000	0.000	0.000	0.000	0.000	0.000	0.000	0.000	0.000
44.H	0.000	0.000	0.000	0.000	0.000	0.000	0.000	0.000	0.000
45.H	0.957	0.010	0.007	0.000	0.972	-0.018	0.000	0.000	0.980
46.H	-0.071	0.001	0.015	0.011	-0.038	-0.021	0.007	-0.012	-0.035
47.H	-0.055	0.002	0.008	0.006	-0.039	-0.007	0.004	-0.012	-0.026
48.H	-0.047	0.011	-0.004	0.006	-0.031	-0.011	0.004	-0.007	-0.029
49.H	-0.036	0.007	-0.003	0.003	-0.030	-0.003	0.002	-0.006	-0.023
50.H	-0.045	0.000	0.007	0.007	-0.020	-0.020	0.005	0.000	-0.035
51.H	-0.046	-0.012	0.020	0.008	-0.016	-0.023	0.005	0.003	-0.036

52.H	-0.059	-0.003	0.015	0.010	-0.026	-0.024	0.007	-0.004	-0.037
53.H	-0.058	-0.019	0.031	0.009	-0.023	-0.023	0.006	-0.002	-0.036
54.H	-0.040	-0.025	0.032	0.005	-0.015	-0.017	0.004	0.004	-0.032
55.H	-0.050	-0.025	0.034	0.006	-0.023	-0.015	0.004	-0.002	-0.032
56.H	-0.038	-0.001	0.006	-0.001	-0.046	0.014	-0.001	-0.017	-0.012
57.H	-0.019	0.002	-0.003	-0.006	-0.044	0.028	-0.004	-0.016	-0.002
58.H	-0.008	-0.003	-0.001	-0.007	-0.034	0.026	-0.005	-0.009	-0.003
59.H	-0.014	-0.019	0.018	-0.006	-0.031	0.021	-0.004	-0.007	-0.007
60.H	-0.025	-0.014	0.016	-0.005	-0.041	0.022	-0.003	-0.014	-0.006
61.H	-0.007	-0.015	0.011	-0.009	-0.036	0.030	-0.006	-0.010	-0.001
62.H	-0.029	0.017	-0.016	-0.004	-0.052	0.025	-0.003	-0.021	-0.004
63.H	-0.046	0.017	-0.010	0.000	-0.056	0.016	-0.000	-0.024	-0.010
64.H	-0.037	0.025	-0.021	-0.001	-0.054	0.019	-0.001	-0.022	-0.008
65.H	-0.021	-0.004	0.004	-0.001	-0.023	0.003	-0.000	-0.001	-0.019
66.H	0.002	-0.017	0.010	-0.004	-0.005	0.004	-0.003	0.011	-0.019
67.H	0.000	-0.004	-0.003	-0.004	-0.011	0.007	-0.003	0.006	-0.016
68.H	0.004	-0.012	0.005	-0.006	-0.012	0.012	-0.004	0.006	-0.013
69.C	0.000	0.000	0.000	0.000	0.000	0.000	0.000	0.000	0.000
70.H	-0.028	-0.001	0.003	0.007	-0.002	-0.027	0.005	0.013	-0.039
71.H	-0.013	-0.008	0.006	0.004	0.009	-0.025	0.003	0.020	-0.038
72.H	-0.023	-0.014	0.015	0.006	0.004	-0.026	0.004	0.016	-0.039
73.H	-0.002	-0.010	0.004	0.000	0.005	-0.012	0.000	0.017	-0.029
74.H	-0.015	0.011	-0.014	0.003	-0.005	-0.016	0.002	0.011	-0.032
75.H	0.001	0.005	-0.012	0.000	0.005	-0.012	0.000	0.017	-0.029
76.H	-0.009	0.030	-0.035	-0.000	-0.018	-0.000	-0.000	0.002	-0.021
77.H	-0.014	0.026	-0.030	0.002	-0.012	-0.009	0.001	0.006	-0.027
78.H	-0.001	0.033	-0.041	-0.001	-0.011	-0.001	-0.001	0.006	-0.022
79.H	0.015	0.021	-0.033	-0.006	-0.009	0.011	-0.004	0.008	-0.014
80.H	0.025	0.013	-0.028	-0.007	0.001	0.009	-0.005	0.015	-0.015
81.H	0.021	0.025	-0.040	-0.006	-0.003	0.009	-0.004	0.012	-0.015
82.H	-0.005	-0.006	0.001	-0.001	-0.005	-0.004	-0.001	0.011	-0.024

83.H	-0.023	-0.014	0.015	0.002	-0.011	-0.010	0.002	0.007	-0.028
84.H	-0.018	0.015	-0.017	0.002	-0.016	-0.006	0.001	0.003	-0.025
85.H	0.010	0.003	-0.013	-0.003	0.001	-0.000	-0.002	0.014	-0.021
86.H	-0.013	0.006	-0.009	-0.005	-0.033	0.019	-0.003	-0.008	-0.009
87.O	0.000	0.000	0.000	0.000	0.000	0.000	0.000	0.000	0.000
88.H	-0.010	-0.026	0.023	-0.004	-0.017	0.009	-0.003	0.003	-0.015
89.H	-0.006	0.024	-0.030	-0.005	-0.029	0.017	-0.003	-0.006	-0.010
90.H	0.008	0.015	-0.024	-0.007	-0.019	0.018	-0.005	0.001	-0.009
91.H	-0.007	0.012	-0.017	-0.004	-0.025	0.013	-0.003	-0.003	-0.012
92.C	0.000	0.000	0.000	0.000	0.000	0.000	0.000	0.000	0.000
93.O	0.000	0.000	0.000	0.000	0.000	0.000	0.000	0.000	0.000
94.H	-0.041	-0.020	0.027	0.003	-0.029	-0.003	0.002	-0.006	-0.023
95.H	-0.031	-0.032	0.036	0.001	-0.020	-0.003	0.001	0.001	-0.023
96.H	-0.031	-0.027	0.031	-0.000	-0.027	0.004	-0.000	-0.004	-0.018

	148			149			150		
	------------------------			------------------------			------------------------		
1.O	0.000	0.000	0.000	0.000	0.000	0.000	0.000	0.000	0.000
2.C	0.000	0.000	0.000	0.000	0.000	0.000	0.000	0.000	0.000
3.C	0.000	0.000	0.000	0.000	0.000	0.000	0.000	0.000	0.000
4.C	0.000	0.000	0.000	0.000	0.000	0.000	0.000	0.000	0.000
5.O	0.000	0.000	0.000	0.000	0.000	0.000	0.000	0.000	0.000
6.N	0.000	0.000	0.000	0.000	0.000	0.000	0.000	0.000	0.000
7.O	0.000	0.000	0.000	0.000	0.000	0.000	0.000	0.000	0.000
8.C	0.000	0.000	0.000	0.000	0.000	0.000	0.000	0.000	0.000
9.C	0.000	0.000	0.000	0.000	0.000	0.000	0.000	0.000	0.000
10.C	0.000	0.000	0.000	0.000	0.000	0.000	0.000	0.000	0.000
11.O	0.000	0.000	0.000	0.000	0.000	0.000	0.000	0.000	0.000
12.C	0.000	0.000	0.000	0.000	0.000	0.000	0.000	0.000	0.000
13.O	0.000	0.000	0.000	0.000	0.000	0.000	0.000	0.000	0.000

14.C	0.000	0.000	0.000	0.000	0.000	0.000	0.000	0.000	0.000
15.C	0.000	0.000	0.000	0.000	0.000	0.000	0.000	0.000	0.000
16.O	0.000	0.000	0.000	0.000	0.000	0.000	0.000	0.000	0.000
17.C	0.000	0.000	0.000	0.000	0.000	0.000	0.000	0.000	0.000
18.C	0.000	0.000	0.000	0.000	0.000	0.000	0.000	0.000	0.000
19.O	0.000	0.000	0.000	0.000	0.000	0.000	0.000	0.000	0.000
20.C	0.000	0.000	0.000	0.000	0.000	0.000	0.000	0.000	0.000
21.C	0.000	0.000	0.000	0.000	0.000	0.000	0.000	0.000	0.000
22.C	0.000	0.000	0.000	0.000	0.000	0.000	0.000	0.000	0.000
23.C	0.000	0.000	0.000	0.000	0.000	0.000	0.000	0.000	0.000
24.C	0.000	0.000	0.000	0.000	0.000	0.000	0.000	0.000	0.000
25.C	0.000	0.000	0.000	0.000	0.000	0.000	0.000	0.000	0.000
26.C	0.000	0.000	0.000	0.000	0.000	0.000	0.000	0.000	0.000
27.C	0.000	0.000	0.000	0.000	0.000	0.000	0.000	0.000	0.000
28.C	0.000	0.000	0.000	0.000	0.000	0.000	0.000	0.000	0.000
29.C	0.000	0.000	0.000	0.000	0.000	0.000	0.000	0.000	0.000
30.C	0.000	0.000	0.000	0.000	0.000	0.000	0.000	0.000	0.000
31.O	0.000	0.000	0.000	0.000	0.000	0.000	0.000	0.000	0.000
32.C	0.000	0.000	0.000	0.000	0.000	0.000	0.000	0.000	0.000
33.C	0.000	0.000	0.000	0.000	0.000	0.000	0.000	0.000	0.000
34.C	0.000	0.000	0.000	0.000	0.000	0.000	0.000	0.000	0.000
35.C	0.000	0.000	0.000	0.000	0.000	0.000	0.000	0.000	0.000
36.C	0.000	0.000	0.000	0.000	0.000	0.000	0.000	0.000	0.000
37.C	0.000	0.000	0.000	0.000	0.000	0.000	0.000	0.000	0.000
38.C	0.000	0.000	0.000	0.000	0.000	0.000	0.000	0.000	0.000
39.C	0.000	0.000	0.000	0.000	0.000	0.000	0.000	0.000	0.000
40.H	0.000	0.000	0.000	0.000	0.000	0.000	0.000	0.000	0.000
41.H	0.000	0.000	0.000	0.000	0.000	0.000	0.000	0.000	0.000
42.H	0.000	0.000	0.000	0.000	0.000	0.000	0.000	0.000	0.000
43.H	0.000	0.000	0.000	0.000	0.000	0.000	0.000	0.000	0.000
44.H	0.000	0.000	0.000	0.000	0.000	0.000	0.000	0.000	0.000

45.H	0.000	0.000	0.000	0.000	0.000	0.000	0.000	0.000	0.000
46.H	0.961	0.001	0.015	0.000	0.979	-0.014	0.000	0.000	0.976
47.H	-0.054	0.002	0.008	0.001	-0.035	-0.005	0.010	-0.015	-0.029
48.H	-0.048	0.012	-0.002	0.000	-0.027	-0.008	0.008	-0.010	-0.030
49.H	-0.036	0.008	-0.002	0.000	-0.027	-0.002	0.005	-0.009	-0.024
50.H	-0.049	0.001	0.008	0.000	-0.021	-0.014	0.008	0.000	-0.039
51.H	-0.050	-0.013	0.019	0.000	-0.021	-0.015	0.008	0.004	-0.043
52.H	-0.062	-0.003	0.015	0.001	-0.026	-0.016	0.012	-0.005	-0.043
53.H	-0.060	-0.020	0.028	0.001	-0.027	-0.014	0.012	-0.001	-0.045
54.H	-0.043	-0.027	0.028	0.000	-0.022	-0.010	0.006	0.006	-0.041
55.H	-0.051	-0.027	0.031	0.001	-0.029	-0.009	0.009	-0.001	-0.040
56.H	-0.033	-0.001	0.004	0.001	-0.041	0.010	0.005	-0.021	-0.012
57.H	-0.012	0.002	-0.005	0.001	-0.039	0.019	-0.001	-0.020	0.000
58.H	-0.003	-0.003	-0.003	0.001	-0.033	0.018	-0.004	-0.011	-0.001
59.H	-0.009	-0.021	0.013	0.001	-0.034	0.015	-0.003	-0.007	-0.009
60.H	-0.019	-0.016	0.012	0.001	-0.040	0.016	0.001	-0.016	-0.008
61.H	-0.001	-0.016	0.007	0.001	-0.036	0.021	-0.005	-0.011	-0.001
62.H	-0.022	0.019	-0.015	0.001	-0.042	0.016	0.002	-0.027	0.000
63.H	-0.039	0.018	-0.010	0.001	-0.045	0.011	0.007	-0.030	-0.008
64.H	-0.032	0.027	-0.019	0.001	-0.041	0.012	0.004	-0.029	-0.004
65.H	-0.021	-0.004	0.003	0.000	-0.024	0.002	0.000	-0.002	-0.020
66.H	-0.001	-0.018	0.008	-0.001	-0.013	0.003	-0.006	0.014	-0.021
67.H	-0.002	-0.004	-0.003	-0.000	-0.015	0.004	-0.006	0.007	-0.016
68.H	0.002	-0.013	0.003	-0.000	-0.017	0.008	-0.007	0.008	-0.014
69.C	0.000	0.000	0.000	0.000	0.000	0.000	0.000	0.000	0.000
70.H	-0.037	-0.001	0.006	-0.001	-0.007	-0.019	0.004	0.015	-0.043
71.H	-0.023	-0.009	0.007	-0.001	-0.001	-0.017	-0.001	0.024	-0.042
72.H	-0.031	-0.015	0.015	-0.001	-0.006	-0.017	0.002	0.021	-0.045
73.H	-0.010	-0.010	0.005	-0.001	-0.004	-0.009	-0.004	0.021	-0.032
74.H	-0.023	0.013	-0.010	-0.001	-0.007	-0.012	-0.000	0.012	-0.031
75.H	-0.008	0.005	-0.009	-0.001	-0.001	-0.009	-0.005	0.020	-0.029

76.H	-0.014	0.033	-0.029	-0.001	-0.013	-0.002	-0.003	0.000	-0.016
77.H	-0.021	0.029	-0.024	-0.001	-0.010	-0.008	-0.001	0.005	-0.024
78.H	-0.008	0.036	-0.034	-0.001	-0.007	-0.003	-0.005	0.005	-0.016
79.H	0.010	0.024	-0.029	-0.001	-0.008	0.006	-0.010	0.008	-0.007
80.H	0.018	0.014	-0.024	-0.001	-0.002	0.005	-0.012	0.017	-0.010
81.H	0.015	0.028	-0.034	-0.001	-0.003	0.004	-0.011	0.012	-0.008
82.H	-0.010	-0.007	0.002	-0.001	-0.011	-0.003	-0.004	0.013	-0.025
83.H	-0.027	-0.015	0.014	-0.000	-0.017	-0.006	0.001	0.009	-0.033
84.H	-0.023	0.017	-0.013	-0.000	-0.015	-0.005	0.000	0.002	-0.024
85.H	0.003	0.004	-0.011	-0.001	-0.005	-0.001	-0.008	0.017	-0.020
86.H	-0.010	0.007	-0.009	0.000	-0.030	0.012	-0.002	-0.011	-0.006
87.O	0.000	0.000	0.000	0.000	0.000	0.000	0.000	0.000	0.000
88.H	-0.010	-0.028	0.019	0.000	-0.024	0.007	-0.003	0.005	-0.019
89.H	-0.006	0.026	-0.027	0.000	-0.023	0.010	-0.004	-0.009	-0.004
90.H	0.007	0.016	-0.022	-0.000	-0.017	0.011	-0.008	0.000	-0.004
91.H	-0.007	0.013	-0.015	-0.000	-0.022	0.008	-0.004	-0.005	-0.009
92.C	0.000	0.000	0.000	0.000	0.000	0.000	0.000	0.000	0.000
93.O	0.000	0.000	0.000	0.000	0.000	0.000	0.000	0.000	0.000
94.H	-0.040	-0.022	0.023	0.001	-0.032	-0.001	0.006	-0.006	-0.029
95.H	-0.031	-0.034	0.031	0.000	-0.028	-0.000	0.003	0.003	-0.031
96.H	-0.029	-0.029	0.026	0.001	-0.032	0.004	0.003	-0.004	-0.024

	151			152			153		
	------------------------			------------------------			------------------------		
1.O	0.000	0.000	0.000	0.000	0.000	0.000	0.000	0.000	0.000
2.C	0.000	0.000	0.000	0.000	0.000	0.000	0.000	0.000	0.000
3.C	0.000	0.000	0.000	0.000	0.000	0.000	0.000	0.000	0.000
4.C	0.000	0.000	0.000	0.000	0.000	0.000	0.000	0.000	0.000
5.O	0.000	0.000	0.000	0.000	0.000	0.000	0.000	0.000	0.000
6.N	0.000	0.000	0.000	0.000	0.000	0.000	0.000	0.000	0.000

7.O	0.000	0.000	0.000	0.000	0.000	0.000	0.000	0.000	0.000
8.C	0.000	0.000	0.000	0.000	0.000	0.000	0.000	0.000	0.000
9.C	0.000	0.000	0.000	0.000	0.000	0.000	0.000	0.000	0.000
10.C	0.000	0.000	0.000	0.000	0.000	0.000	0.000	0.000	0.000
11.O	0.000	0.000	0.000	0.000	0.000	0.000	0.000	0.000	0.000
12.C	0.000	0.000	0.000	0.000	0.000	0.000	0.000	0.000	0.000
13.O	0.000	0.000	0.000	0.000	0.000	0.000	0.000	0.000	0.000
14.C	0.000	0.000	0.000	0.000	0.000	0.000	0.000	0.000	0.000
15.C	0.000	0.000	0.000	0.000	0.000	0.000	0.000	0.000	0.000
16.O	0.000	0.000	0.000	0.000	0.000	0.000	0.000	0.000	0.000
17.C	0.000	0.000	0.000	0.000	0.000	0.000	0.000	0.000	0.000
18.C	0.000	0.000	0.000	0.000	0.000	0.000	0.000	0.000	0.000
19.O	0.000	0.000	0.000	0.000	0.000	0.000	0.000	0.000	0.000
20.C	0.000	0.000	0.000	0.000	0.000	0.000	0.000	0.000	0.000
21.C	0.000	0.000	0.000	0.000	0.000	0.000	0.000	0.000	0.000
22.C	0.000	0.000	0.000	0.000	0.000	0.000	0.000	0.000	0.000
23.C	0.000	0.000	0.000	0.000	0.000	0.000	0.000	0.000	0.000
24.C	0.000	0.000	0.000	0.000	0.000	0.000	0.000	0.000	0.000
25.C	0.000	0.000	0.000	0.000	0.000	0.000	0.000	0.000	0.000
26.C	0.000	0.000	0.000	0.000	0.000	0.000	0.000	0.000	0.000
27.C	0.000	0.000	0.000	0.000	0.000	0.000	0.000	0.000	0.000
28.C	0.000	0.000	0.000	0.000	0.000	0.000	0.000	0.000	0.000
29.C	0.000	0.000	0.000	0.000	0.000	0.000	0.000	0.000	0.000
30.C	0.000	0.000	0.000	0.000	0.000	0.000	0.000	0.000	0.000
31.O	0.000	0.000	0.000	0.000	0.000	0.000	0.000	0.000	0.000
32.C	0.000	0.000	0.000	0.000	0.000	0.000	0.000	0.000	0.000
33.C	0.000	0.000	0.000	0.000	0.000	0.000	0.000	0.000	0.000
34.C	0.000	0.000	0.000	0.000	0.000	0.000	0.000	0.000	0.000
35.C	0.000	0.000	0.000	0.000	0.000	0.000	0.000	0.000	0.000
36.C	0.000	0.000	0.000	0.000	0.000	0.000	0.000	0.000	0.000
37.C	0.000	0.000	0.000	0.000	0.000	0.000	0.000	0.000	0.000

38.C	0.000	0.000	0.000	0.000	0.000	0.000	0.000	0.000	0.000
39.C	0.000	0.000	0.000	0.000	0.000	0.000	0.000	0.000	0.000
40.H	0.000	0.000	0.000	0.000	0.000	0.000	0.000	0.000	0.000
41.H	0.000	0.000	0.000	0.000	0.000	0.000	0.000	0.000	0.000
42.H	0.000	0.000	0.000	0.000	0.000	0.000	0.000	0.000	0.000
43.H	0.000	0.000	0.000	0.000	0.000	0.000	0.000	0.000	0.000
44.H	0.000	0.000	0.000	0.000	0.000	0.000	0.000	0.000	0.000
45.H	0.000	0.000	0.000	0.000	0.000	0.000	0.000	0.000	0.000
46.H	0.000	0.000	0.000	0.000	0.000	0.000	0.000	0.000	0.000
47.H	0.973	0.002	0.006	0.000	0.978	-0.006	0.000	0.000	0.983
48.H	-0.040	0.007	-0.003	0.001	-0.029	-0.009	0.005	-0.004	-0.025
49.H	-0.034	0.004	-0.002	0.001	-0.029	-0.003	0.003	-0.004	-0.023
50.H	-0.037	0.000	0.005	0.001	-0.022	-0.015	0.004	-0.000	-0.029
51.H	-0.038	-0.007	0.015	0.001	-0.021	-0.017	0.004	0.002	-0.032
52.H	-0.047	-0.001	0.010	0.002	-0.027	-0.018	0.007	-0.002	-0.031
53.H	-0.047	-0.011	0.023	0.002	-0.028	-0.017	0.006	0.000	-0.033
54.H	-0.035	-0.014	0.024	0.001	-0.022	-0.012	0.003	0.003	-0.032
55.H	-0.043	-0.014	0.026	0.002	-0.029	-0.011	0.005	0.001	-0.032
56.H	-0.040	0.001	0.005	0.001	-0.043	0.010	0.004	-0.008	-0.019
57.H	-0.027	0.002	-0.001	0.000	-0.041	0.020	0.001	-0.008	-0.014
58.H	-0.019	-0.001	0.000	-0.001	-0.034	0.019	-0.001	-0.004	-0.015
59.H	-0.023	-0.011	0.014	-0.000	-0.034	0.016	0.000	-0.002	-0.019
60.H	-0.032	-0.007	0.013	0.000	-0.042	0.017	0.002	-0.006	-0.019
61.H	-0.020	-0.008	0.010	-0.000	-0.037	0.023	-0.001	-0.004	-0.016
62.H	-0.034	0.011	-0.011	0.001	-0.045	0.018	0.003	-0.011	-0.013
63.H	-0.045	0.011	-0.007	0.002	-0.048	0.011	0.006	-0.012	-0.016
64.H	-0.039	0.016	-0.015	0.001	-0.044	0.013	0.004	-0.012	-0.014
65.H	-0.024	-0.002	0.003	0.000	-0.025	0.002	0.001	-0.001	-0.022
66.H	-0.007	-0.010	0.008	-0.001	-0.013	0.003	-0.004	0.006	-0.023
67.H	-0.009	-0.002	-0.001	-0.001	-0.016	0.005	-0.003	0.003	-0.020
68.H	-0.008	-0.007	0.004	-0.001	-0.017	0.008	-0.004	0.003	-0.020

69.C	0.000	0.000	0.000	0.000	0.000	0.000	0.000	0.000	0.000
70.H	-0.023	-0.001	0.002	0.000	-0.007	-0.021	0.000	0.006	-0.030
71.H	-0.012	-0.006	0.004	-0.000	-0.000	-0.019	-0.002	0.009	-0.030
72.H	-0.019	-0.009	0.010	0.000	-0.005	-0.020	-0.001	0.008	-0.032
73.H	-0.007	-0.006	0.003	-0.001	-0.003	-0.010	-0.004	0.008	-0.026
74.H	-0.015	0.006	-0.011	-0.000	-0.007	-0.013	-0.002	0.004	-0.024
75.H	-0.004	0.002	-0.009	-0.001	-0.001	-0.010	-0.004	0.007	-0.024
76.H	-0.014	0.017	-0.026	-0.001	-0.014	-0.002	-0.002	-0.001	-0.017
77.H	-0.015	0.015	-0.022	-0.000	-0.011	-0.009	-0.002	0.001	-0.020
78.H	-0.007	0.019	-0.031	-0.001	-0.009	-0.003	-0.004	0.001	-0.016
79.H	0.002	0.012	-0.024	-0.002	-0.009	0.006	-0.006	0.002	-0.014
80.H	0.010	0.007	-0.020	-0.002	-0.002	0.005	-0.008	0.006	-0.015
81.H	0.007	0.014	-0.029	-0.002	-0.004	0.005	-0.007	0.003	-0.014
82.H	-0.010	-0.004	0.001	-0.001	-0.010	-0.003	-0.003	0.005	-0.023
83.H	-0.023	-0.008	0.011	0.000	-0.017	-0.008	0.000	0.004	-0.027
84.H	-0.020	0.009	-0.012	-0.000	-0.016	-0.006	-0.000	0.000	-0.021
85.H	0.000	0.001	-0.010	-0.002	-0.004	-0.002	-0.005	0.006	-0.020
86.H	-0.021	0.004	-0.006	-0.000	-0.031	0.013	-0.000	-0.004	-0.016
87.O	0.000	0.000	0.000	0.000	0.000	0.000	0.000	0.000	0.000
88.H	-0.018	-0.015	0.018	-0.000	-0.024	0.007	-0.001	0.003	-0.023
89.H	-0.015	0.014	-0.022	-0.001	-0.025	0.011	-0.002	-0.004	-0.013
90.H	-0.005	0.008	-0.017	-0.001	-0.018	0.012	-0.004	-0.001	-0.014
91.H	-0.015	0.007	-0.012	-0.001	-0.024	0.009	-0.002	-0.002	-0.016
92.C	0.000	0.000	0.000	0.000	0.000	0.000	0.000	0.000	0.000
93.O	0.000	0.000	0.000	0.000	0.000	0.000	0.000	0.000	0.000
94.H	-0.038	-0.011	0.021	0.001	-0.033	-0.002	0.004	-0.002	-0.027
95.H	-0.031	-0.018	0.027	0.001	-0.027	-0.001	0.002	0.002	-0.029
96.H	-0.033	-0.015	0.024	0.001	-0.033	0.004	0.003	-0.001	-0.026

154 155 156

	------------------------			------------------------			------------------------		
1.O	0.000	0.000	0.000	0.000	0.000	0.000	0.000	0.000	0.000
2.C	0.000	0.000	0.000	0.000	0.000	0.000	0.000	0.000	0.000
3.C	0.000	0.000	0.000	0.000	0.000	0.000	0.000	0.000	0.000
4.C	0.000	0.000	0.000	0.000	0.000	0.000	0.000	0.000	0.000
5.O	0.000	0.000	0.000	0.000	0.000	0.000	0.000	0.000	0.000
6.N	0.000	0.000	0.000	0.000	0.000	0.000	0.000	0.000	0.000
7.O	0.000	0.000	0.000	0.000	0.000	0.000	0.000	0.000	0.000
8.C	0.000	0.000	0.000	0.000	0.000	0.000	0.000	0.000	0.000
9.C	0.000	0.000	0.000	0.000	0.000	0.000	0.000	0.000	0.000
10.C	0.000	0.000	0.000	0.000	0.000	0.000	0.000	0.000	0.000
11.O	0.000	0.000	0.000	0.000	0.000	0.000	0.000	0.000	0.000
12.C	0.000	0.000	0.000	0.000	0.000	0.000	0.000	0.000	0.000
13.O	0.000	0.000	0.000	0.000	0.000	0.000	0.000	0.000	0.000
14.C	0.000	0.000	0.000	0.000	0.000	0.000	0.000	0.000	0.000
15.C	0.000	0.000	0.000	0.000	0.000	0.000	0.000	0.000	0.000
16.O	0.000	0.000	0.000	0.000	0.000	0.000	0.000	0.000	0.000
17.C	0.000	0.000	0.000	0.000	0.000	0.000	0.000	0.000	0.000
18.C	0.000	0.000	0.000	0.000	0.000	0.000	0.000	0.000	0.000
19.O	0.000	0.000	0.000	0.000	0.000	0.000	0.000	0.000	0.000
20.C	0.000	0.000	0.000	0.000	0.000	0.000	0.000	0.000	0.000
21.C	0.000	0.000	0.000	0.000	0.000	0.000	0.000	0.000	0.000
22.C	0.000	0.000	0.000	0.000	0.000	0.000	0.000	0.000	0.000
23.C	0.000	0.000	0.000	0.000	0.000	0.000	0.000	0.000	0.000
24.C	0.000	0.000	0.000	0.000	0.000	0.000	0.000	0.000	0.000
25.C	0.000	0.000	0.000	0.000	0.000	0.000	0.000	0.000	0.000
26.C	0.000	0.000	0.000	0.000	0.000	0.000	0.000	0.000	0.000
27.C	0.000	0.000	0.000	0.000	0.000	0.000	0.000	0.000	0.000
28.C	0.000	0.000	0.000	0.000	0.000	0.000	0.000	0.000	0.000
29.C	0.000	0.000	0.000	0.000	0.000	0.000	0.000	0.000	0.000
30.C	0.000	0.000	0.000	0.000	0.000	0.000	0.000	0.000	0.000

31.O	0.000	0.000	0.000	0.000	0.000	0.000	0.000	0.000	0.000
32.C	0.000	0.000	0.000	0.000	0.000	0.000	0.000	0.000	0.000
33.C	0.000	0.000	0.000	0.000	0.000	0.000	0.000	0.000	0.000
34.C	0.000	0.000	0.000	0.000	0.000	0.000	0.000	0.000	0.000
35.C	0.000	0.000	0.000	0.000	0.000	0.000	0.000	0.000	0.000
36.C	0.000	0.000	0.000	0.000	0.000	0.000	0.000	0.000	0.000
37.C	0.000	0.000	0.000	0.000	0.000	0.000	0.000	0.000	0.000
38.C	0.000	0.000	0.000	0.000	0.000	0.000	0.000	0.000	0.000
39.C	0.000	0.000	0.000	0.000	0.000	0.000	0.000	0.000	0.000
40.H	0.000	0.000	0.000	0.000	0.000	0.000	0.000	0.000	0.000
41.H	0.000	0.000	0.000	0.000	0.000	0.000	0.000	0.000	0.000
42.H	0.000	0.000	0.000	0.000	0.000	0.000	0.000	0.000	0.000
43.H	0.000	0.000	0.000	0.000	0.000	0.000	0.000	0.000	0.000
44.H	0.000	0.000	0.000	0.000	0.000	0.000	0.000	0.000	0.000
45.H	0.000	0.000	0.000	0.000	0.000	0.000	0.000	0.000	0.000
46.H	0.000	0.000	0.000	0.000	0.000	0.000	0.000	0.000	0.000
47.H	0.000	0.000	0.000	0.000	0.000	0.000	0.000	0.000	0.000
48.H	0.977	0.008	-0.000	0.000	0.981	-0.005	0.000	0.000	0.981
49.H	-0.032	0.005	-0.001	0.004	-0.028	-0.001	-0.001	-0.005	-0.025
50.H	-0.041	0.000	0.005	0.010	-0.023	-0.010	0.002	-0.000	-0.033
51.H	-0.042	-0.008	0.012	0.011	-0.018	-0.012	0.002	0.001	-0.032
52.H	-0.049	-0.002	0.009	0.013	-0.024	-0.012	0.001	-0.004	-0.034
53.H	-0.047	-0.013	0.017	0.012	-0.018	-0.013	0.000	-0.003	-0.030
54.H	-0.037	-0.017	0.017	0.008	-0.014	-0.010	0.001	0.001	-0.026
55.H	-0.041	-0.017	0.018	0.008	-0.017	-0.010	-0.001	-0.004	-0.025
56.H	-0.028	-0.000	0.002	-0.002	-0.031	0.005	-0.006	-0.014	-0.014
57.H	-0.015	0.002	-0.004	-0.008	-0.032	0.012	-0.007	-0.013	-0.008
58.H	-0.010	-0.002	-0.003	-0.009	-0.027	0.011	-0.005	-0.008	-0.008
59.H	-0.014	-0.013	0.007	-0.008	-0.021	0.007	-0.005	-0.007	-0.007
60.H	-0.019	-0.010	0.006	-0.006	-0.026	0.008	-0.007	-0.012	-0.007
61.H	-0.008	-0.010	0.003	-0.011	-0.024	0.011	-0.007	-0.009	-0.003

62.H	-0.021	0.012	-0.009	-0.006	-0.039	0.012	-0.007	-0.016	-0.012
63.H	-0.031	0.012	-0.006	-0.001	-0.040	0.007	-0.007	-0.018	-0.016
64.H	-0.027	0.018	-0.011	-0.002	-0.041	0.009	-0.007	-0.016	-0.016
65.H	-0.023	-0.002	0.002	-0.000	-0.023	0.001	-0.001	-0.002	-0.020
66.H	-0.012	-0.011	0.005	-0.004	-0.013	0.000	0.002	0.007	-0.019
67.H	-0.012	-0.003	-0.001	-0.004	-0.018	0.003	0.001	0.004	-0.019
68.H	-0.009	-0.008	0.002	-0.006	-0.016	0.004	-0.000	0.003	-0.015
69.C	0.000	0.000	0.000	0.000	0.000	0.000	0.000	0.000	0.000
70.H	-0.035	-0.001	0.004	0.010	-0.016	-0.012	0.006	0.009	-0.038
71.H	-0.027	-0.006	0.005	0.007	-0.010	-0.012	0.008	0.014	-0.036
72.H	-0.032	-0.009	0.010	0.009	-0.010	-0.013	0.006	0.011	-0.035
73.H	-0.019	-0.007	0.004	0.002	-0.011	-0.006	0.006	0.012	-0.029
74.H	-0.026	0.008	-0.004	0.006	-0.020	-0.006	0.005	0.009	-0.035
75.H	-0.018	0.003	-0.004	0.002	-0.015	-0.005	0.007	0.013	-0.032
76.H	-0.020	0.021	-0.016	0.001	-0.030	0.002	0.003	0.003	-0.030
77.H	-0.024	0.018	-0.012	0.004	-0.027	-0.002	0.004	0.006	-0.034
78.H	-0.017	0.023	-0.018	0.000	-0.029	0.002	0.004	0.007	-0.032
79.H	-0.005	0.015	-0.016	-0.006	-0.025	0.007	0.003	0.007	-0.023
80.H	-0.001	0.009	-0.013	-0.007	-0.019	0.006	0.004	0.012	-0.023
81.H	-0.003	0.018	-0.019	-0.006	-0.024	0.007	0.004	0.010	-0.026
82.H	-0.017	-0.004	0.001	-0.000	-0.015	-0.002	0.003	0.007	-0.025
83.H	-0.027	-0.010	0.008	0.004	-0.015	-0.006	0.002	0.004	-0.026
84.H	-0.025	0.011	-0.007	0.003	-0.025	-0.002	0.003	0.003	-0.030
85.H	-0.010	0.003	-0.006	-0.003	-0.016	0.000	0.004	0.011	-0.026
86.H	-0.015	0.004	-0.005	-0.006	-0.029	0.008	-0.004	-0.006	-0.014
87.O	0.000	0.000	0.000	0.000	0.000	0.000	0.000	0.000	0.000
88.H	-0.015	-0.018	0.010	-0.005	-0.014	0.001	-0.002	-0.000	-0.013
89.H	-0.013	0.017	-0.015	-0.005	-0.033	0.009	-0.002	-0.003	-0.019
90.H	-0.006	0.010	-0.012	-0.008	-0.026	0.009	-0.000	0.002	-0.018
91.H	-0.014	0.008	-0.009	-0.005	-0.028	0.007	-0.001	-0.002	-0.019
92.C	0.000	0.000	0.000	0.000	0.000	0.000	0.000	0.000	0.000

93.O	0.000	0.000	0.000	0.000	0.000	0.000	0.000	0.000	0.000
94.H	-0.033	-0.014	0.013	0.003	-0.020	-0.004	-0.003	-0.006	-0.019
95.H	-0.028	-0.022	0.017	0.002	-0.014	-0.005	-0.002	-0.002	-0.017
96.H	-0.026	-0.019	0.014	-0.000	-0.017	-0.001	-0.004	-0.006	-0.014

	157			158			159		
1.O	0.000	0.000	0.000	0.000	0.000	0.000	0.000	0.000	0.000
2.C	0.000	0.000	0.000	0.000	0.000	0.000	0.000	0.000	0.000
3.C	0.000	0.000	0.000	0.000	0.000	0.000	0.000	0.000	0.000
4.C	0.000	0.000	0.000	0.000	0.000	0.000	0.000	0.000	0.000
5.O	0.000	0.000	0.000	0.000	0.000	0.000	0.000	0.000	0.000
6.N	0.000	0.000	0.000	0.000	0.000	0.000	0.000	0.000	0.000
7.O	0.000	0.000	0.000	0.000	0.000	0.000	0.000	0.000	0.000
8.C	0.000	0.000	0.000	0.000	0.000	0.000	0.000	0.000	0.000
9.C	0.000	0.000	0.000	0.000	0.000	0.000	0.000	0.000	0.000
10.C	0.000	0.000	0.000	0.000	0.000	0.000	0.000	0.000	0.000
11.O	0.000	0.000	0.000	0.000	0.000	0.000	0.000	0.000	0.000
12.C	0.000	0.000	0.000	0.000	0.000	0.000	0.000	0.000	0.000
13.O	0.000	0.000	0.000	0.000	0.000	0.000	0.000	0.000	0.000
14.C	0.000	0.000	0.000	0.000	0.000	0.000	0.000	0.000	0.000
15.C	0.000	0.000	0.000	0.000	0.000	0.000	0.000	0.000	0.000
16.O	0.000	0.000	0.000	0.000	0.000	0.000	0.000	0.000	0.000
17.C	0.000	0.000	0.000	0.000	0.000	0.000	0.000	0.000	0.000
18.C	0.000	0.000	0.000	0.000	0.000	0.000	0.000	0.000	0.000
19.O	0.000	0.000	0.000	0.000	0.000	0.000	0.000	0.000	0.000
20.C	0.000	0.000	0.000	0.000	0.000	0.000	0.000	0.000	0.000
21.C	0.000	0.000	0.000	0.000	0.000	0.000	0.000	0.000	0.000
22.C	0.000	0.000	0.000	0.000	0.000	0.000	0.000	0.000	0.000
23.C	0.000	0.000	0.000	0.000	0.000	0.000	0.000	0.000	0.000

24.C	0.000	0.000	0.000	0.000	0.000	0.000	0.000	0.000	0.000
25.C	0.000	0.000	0.000	0.000	0.000	0.000	0.000	0.000	0.000
26.C	0.000	0.000	0.000	0.000	0.000	0.000	0.000	0.000	0.000
27.C	0.000	0.000	0.000	0.000	0.000	0.000	0.000	0.000	0.000
28.C	0.000	0.000	0.000	0.000	0.000	0.000	0.000	0.000	0.000
29.C	0.000	0.000	0.000	0.000	0.000	0.000	0.000	0.000	0.000
30.C	0.000	0.000	0.000	0.000	0.000	0.000	0.000	0.000	0.000
31.O	0.000	0.000	0.000	0.000	0.000	0.000	0.000	0.000	0.000
32.C	0.000	0.000	0.000	0.000	0.000	0.000	0.000	0.000	0.000
33.C	0.000	0.000	0.000	0.000	0.000	0.000	0.000	0.000	0.000
34.C	0.000	0.000	0.000	0.000	0.000	0.000	0.000	0.000	0.000
35.C	0.000	0.000	0.000	0.000	0.000	0.000	0.000	0.000	0.000
36.C	0.000	0.000	0.000	0.000	0.000	0.000	0.000	0.000	0.000
37.C	0.000	0.000	0.000	0.000	0.000	0.000	0.000	0.000	0.000
38.C	0.000	0.000	0.000	0.000	0.000	0.000	0.000	0.000	0.000
39.C	0.000	0.000	0.000	0.000	0.000	0.000	0.000	0.000	0.000
40.H	0.000	0.000	0.000	0.000	0.000	0.000	0.000	0.000	0.000
41.H	0.000	0.000	0.000	0.000	0.000	0.000	0.000	0.000	0.000
42.H	0.000	0.000	0.000	0.000	0.000	0.000	0.000	0.000	0.000
43.H	0.000	0.000	0.000	0.000	0.000	0.000	0.000	0.000	0.000
44.H	0.000	0.000	0.000	0.000	0.000	0.000	0.000	0.000	0.000
45.H	0.000	0.000	0.000	0.000	0.000	0.000	0.000	0.000	0.000
46.H	0.000	0.000	0.000	0.000	0.000	0.000	0.000	0.000	0.000
47.H	0.000	0.000	0.000	0.000	0.000	0.000	0.000	0.000	0.000
48.H	0.000	0.000	0.000	0.000	0.000	0.000	0.000	0.000	0.000
49.H	0.981	0.003	-0.001	0.000	0.982	-0.002	0.000	0.000	0.984
50.H	-0.032	0.000	0.003	0.007	-0.023	-0.009	0.000	-0.000	-0.026
51.H	-0.032	-0.004	0.008	0.007	-0.020	-0.011	0.000	0.000	-0.026
52.H	-0.037	-0.001	0.006	0.009	-0.025	-0.011	-0.000	-0.001	-0.026
53.H	-0.037	-0.006	0.012	0.008	-0.022	-0.012	-0.001	-0.001	-0.025
54.H	-0.031	-0.008	0.012	0.005	-0.018	-0.009	-0.000	-0.000	-0.023

55.H	-0.034	-0.008	0.013	0.006	-0.021	-0.009	-0.001	-0.002	-0.023
56.H	-0.032	0.000	0.003	-0.001	-0.033	0.005	-0.003	-0.005	-0.020
57.H	-0.025	0.001	-0.001	-0.005	-0.033	0.011	-0.003	-0.004	-0.018
58.H	-0.021	-0.001	-0.000	-0.006	-0.028	0.011	-0.002	-0.003	-0.018
59.H	-0.023	-0.006	0.007	-0.005	-0.025	0.007	-0.002	-0.003	-0.017
60.H	-0.027	-0.004	0.006	-0.004	-0.029	0.008	-0.003	-0.004	-0.017
61.H	-0.021	-0.005	0.005	-0.007	-0.027	0.012	-0.003	-0.003	-0.016
62.H	-0.029	0.006	-0.006	-0.003	-0.038	0.011	-0.003	-0.005	-0.020
63.H	-0.034	0.006	-0.003	0.000	-0.039	0.007	-0.004	-0.006	-0.021
64.H	-0.031	0.009	-0.008	-0.001	-0.040	0.008	-0.003	-0.005	-0.021
65.H	-0.024	-0.001	0.002	-0.000	-0.024	0.001	-0.001	-0.001	-0.022
66.H	-0.016	-0.006	0.004	-0.003	-0.015	0.001	0.001	0.002	-0.021
67.H	-0.016	-0.001	-0.001	-0.003	-0.019	0.003	0.001	0.001	-0.022
68.H	-0.016	-0.004	0.002	-0.004	-0.018	0.004	0.000	0.001	-0.020
69.C	0.000	0.000	0.000	0.000	0.000	0.000	0.000	0.000	0.000
70.H	-0.025	-0.001	0.001	0.007	-0.016	-0.012	0.002	0.003	-0.028
71.H	-0.020	-0.003	0.002	0.005	-0.011	-0.011	0.003	0.005	-0.028
72.H	-0.023	-0.005	0.006	0.006	-0.012	-0.012	0.003	0.004	-0.027
73.H	-0.016	-0.003	0.002	0.001	-0.012	-0.006	0.003	0.004	-0.025
74.H	-0.021	0.004	-0.005	0.004	-0.019	-0.006	0.002	0.003	-0.028
75.H	-0.015	0.001	-0.004	0.001	-0.014	-0.005	0.003	0.005	-0.027
76.H	-0.019	0.010	-0.013	0.000	-0.026	0.001	0.001	0.001	-0.027
77.H	-0.021	0.009	-0.011	0.003	-0.024	-0.003	0.002	0.002	-0.028
78.H	-0.016	0.011	-0.015	-0.000	-0.024	0.001	0.002	0.003	-0.027
79.H	-0.011	0.007	-0.012	-0.004	-0.022	0.006	0.002	0.003	-0.024
80.H	-0.007	0.004	-0.010	-0.005	-0.017	0.005	0.003	0.004	-0.024
81.H	-0.008	0.008	-0.015	-0.004	-0.020	0.005	0.003	0.004	-0.025
82.H	-0.018	-0.002	0.001	-0.000	-0.016	-0.002	0.002	0.002	-0.024
83.H	-0.024	-0.005	0.006	0.003	-0.018	-0.006	0.001	0.001	-0.024
84.H	-0.023	0.005	-0.006	0.002	-0.024	-0.002	0.001	0.001	-0.026
85.H	-0.012	0.001	-0.005	-0.002	-0.016	-0.000	0.002	0.004	-0.025

86.H	-0.022	0.002	-0.003	-0.004	-0.029	0.008	-0.002	-0.002	-0.020
87.O	0.000	0.000	0.000	0.000	0.000	0.000	0.000	0.000	0.000
88.H	-0.021	-0.008	0.009	-0.003	-0.018	0.002	-0.001	-0.000	-0.019
89.H	-0.019	0.008	-0.011	-0.003	-0.030	0.008	-0.000	-0.001	-0.023
90.H	-0.014	0.005	-0.009	-0.005	-0.025	0.008	0.000	0.001	-0.022
91.H	-0.019	0.004	-0.006	-0.003	-0.027	0.006	-0.000	-0.001	-0.022
92.C	0.000	0.000	0.000	0.000	0.000	0.000	0.000	0.000	0.000
93.O	0.000	0.000	0.000	0.000	0.000	0.000	0.000	0.000	0.000
94.H	-0.032	-0.006	0.010	0.002	-0.024	-0.003	-0.002	-0.002	-0.021
95.H	-0.028	-0.010	0.014	0.001	-0.019	-0.003	-0.001	-0.001	-0.020
96.H	-0.028	-0.009	0.012	-0.000	-0.022	-0.000	-0.002	-0.002	-0.019

	160			161			162		
	------------------------			------------------------			------------------------		
1.O	0.000	0.000	0.000	0.000	0.000	0.000	0.000	0.000	0.000
2.C	0.000	0.000	0.000	0.000	0.000	0.000	0.000	0.000	0.000
3.C	0.000	0.000	0.000	0.000	0.000	0.000	0.000	0.000	0.000
4.C	0.000	0.000	0.000	0.000	0.000	0.000	0.000	0.000	0.000
5.O	0.000	0.000	0.000	0.000	0.000	0.000	0.000	0.000	0.000
6.N	0.000	0.000	0.000	0.000	0.000	0.000	0.000	0.000	0.000
7.O	0.000	0.000	0.000	0.000	0.000	0.000	0.000	0.000	0.000
8.C	0.000	0.000	0.000	0.000	0.000	0.000	0.000	0.000	0.000
9.C	0.000	0.000	0.000	0.000	0.000	0.000	0.000	0.000	0.000
10.C	0.000	0.000	0.000	0.000	0.000	0.000	0.000	0.000	0.000
11.O	0.000	0.000	0.000	0.000	0.000	0.000	0.000	0.000	0.000
12.C	0.000	0.000	0.000	0.000	0.000	0.000	0.000	0.000	0.000
13.O	0.000	0.000	0.000	0.000	0.000	0.000	0.000	0.000	0.000
14.C	0.000	0.000	0.000	0.000	0.000	0.000	0.000	0.000	0.000
15.C	0.000	0.000	0.000	0.000	0.000	0.000	0.000	0.000	0.000
16.O	0.000	0.000	0.000	0.000	0.000	0.000	0.000	0.000	0.000

17.C	0.000	0.000	0.000	0.000	0.000	0.000	0.000	0.000	0.000
18.C	0.000	0.000	0.000	0.000	0.000	0.000	0.000	0.000	0.000
19.O	0.000	0.000	0.000	0.000	0.000	0.000	0.000	0.000	0.000
20.C	0.000	0.000	0.000	0.000	0.000	0.000	0.000	0.000	0.000
21.C	0.000	0.000	0.000	0.000	0.000	0.000	0.000	0.000	0.000
22.C	0.000	0.000	0.000	0.000	0.000	0.000	0.000	0.000	0.000
23.C	0.000	0.000	0.000	0.000	0.000	0.000	0.000	0.000	0.000
24.C	0.000	0.000	0.000	0.000	0.000	0.000	0.000	0.000	0.000
25.C	0.000	0.000	0.000	0.000	0.000	0.000	0.000	0.000	0.000
26.C	0.000	0.000	0.000	0.000	0.000	0.000	0.000	0.000	0.000
27.C	0.000	0.000	0.000	0.000	0.000	0.000	0.000	0.000	0.000
28.C	0.000	0.000	0.000	0.000	0.000	0.000	0.000	0.000	0.000
29.C	0.000	0.000	0.000	0.000	0.000	0.000	0.000	0.000	0.000
30.C	0.000	0.000	0.000	0.000	0.000	0.000	0.000	0.000	0.000
31.O	0.000	0.000	0.000	0.000	0.000	0.000	0.000	0.000	0.000
32.C	0.000	0.000	0.000	0.000	0.000	0.000	0.000	0.000	0.000
33.C	0.000	0.000	0.000	0.000	0.000	0.000	0.000	0.000	0.000
34.C	0.000	0.000	0.000	0.000	0.000	0.000	0.000	0.000	0.000
35.C	0.000	0.000	0.000	0.000	0.000	0.000	0.000	0.000	0.000
36.C	0.000	0.000	0.000	0.000	0.000	0.000	0.000	0.000	0.000
37.C	0.000	0.000	0.000	0.000	0.000	0.000	0.000	0.000	0.000
38.C	0.000	0.000	0.000	0.000	0.000	0.000	0.000	0.000	0.000
39.C	0.000	0.000	0.000	0.000	0.000	0.000	0.000	0.000	0.000
40.H	0.000	0.000	0.000	0.000	0.000	0.000	0.000	0.000	0.000
41.H	0.000	0.000	0.000	0.000	0.000	0.000	0.000	0.000	0.000
42.H	0.000	0.000	0.000	0.000	0.000	0.000	0.000	0.000	0.000
43.H	0.000	0.000	0.000	0.000	0.000	0.000	0.000	0.000	0.000
44.H	0.000	0.000	0.000	0.000	0.000	0.000	0.000	0.000	0.000
45.H	0.000	0.000	0.000	0.000	0.000	0.000	0.000	0.000	0.000
46.H	0.000	0.000	0.000	0.000	0.000	0.000	0.000	0.000	0.000
47.H	0.000	0.000	0.000	0.000	0.000	0.000	0.000	0.000	0.000

48.H	0.000	0.000	0.000	0.000	0.000	0.000	0.000	0.000	0.000
49.H	0.000	0.000	0.000	0.000	0.000	0.000	0.000	0.000	0.000
50.H	0.971	0.001	0.007	0.000	0.984	-0.001	0.000	0.000	0.975
51.H	-0.051	-0.011	0.012	0.001	-0.023	-0.001	0.008	0.003	-0.045
52.H	-0.057	-0.002	0.010	0.001	-0.023	-0.001	0.010	-0.006	-0.046
53.H	-0.054	-0.018	0.015	0.001	-0.023	-0.001	0.009	-0.003	-0.045
54.H	-0.043	-0.023	0.014	0.001	-0.023	-0.001	0.006	0.004	-0.040
55.H	-0.045	-0.023	0.014	0.001	-0.023	-0.001	0.006	-0.003	-0.039
56.H	-0.019	-0.001	-0.001	-0.000	-0.024	0.000	-0.000	-0.023	-0.012
57.H	-0.002	0.002	-0.007	-0.001	-0.024	0.001	-0.005	-0.022	-0.000
58.H	0.000	-0.002	-0.006	-0.001	-0.024	0.001	-0.006	-0.013	-0.001
59.H	-0.004	-0.018	0.001	-0.001	-0.023	0.000	-0.005	-0.010	-0.006
60.H	-0.007	-0.014	-0.000	-0.001	-0.023	0.000	-0.004	-0.019	-0.005
61.H	0.005	-0.014	-0.003	-0.001	-0.023	0.001	-0.008	-0.014	0.002
62.H	-0.009	0.017	-0.010	-0.001	-0.025	0.001	-0.003	-0.029	-0.002
63.H	-0.021	0.016	-0.006	-0.000	-0.025	0.000	0.000	-0.033	-0.010
64.H	-0.018	0.024	-0.010	-0.000	-0.025	0.001	-0.001	-0.031	-0.008
65.H	-0.023	-0.003	0.001	-0.000	-0.023	0.000	0.000	-0.003	-0.022
66.H	-0.014	-0.015	0.003	-0.000	-0.023	-0.000	-0.003	0.013	-0.022
67.H	-0.014	-0.003	-0.002	-0.000	-0.023	0.000	-0.003	0.007	-0.019
68.H	-0.008	-0.011	-0.001	-0.001	-0.023	0.000	-0.005	0.007	-0.015
69.C	0.000	0.000	0.000	0.000	0.000	0.000	0.000	0.000	0.000
70.H	-0.051	-0.000	0.008	0.001	-0.023	-0.001	0.007	0.016	-0.049
71.H	-0.044	-0.007	0.008	0.001	-0.023	-0.001	0.005	0.025	-0.047
72.H	-0.047	-0.012	0.011	0.001	-0.023	-0.001	0.006	0.021	-0.048
73.H	-0.030	-0.008	0.005	0.000	-0.023	-0.000	0.001	0.022	-0.036
74.H	-0.039	0.012	0.000	0.001	-0.024	-0.000	0.004	0.013	-0.039
75.H	-0.031	0.006	0.000	0.000	-0.023	-0.000	0.002	0.022	-0.036
76.H	-0.026	0.029	-0.010	0.000	-0.024	0.000	0.001	0.001	-0.025
77.H	-0.035	0.026	-0.006	0.000	-0.024	0.000	0.003	0.006	-0.033
78.H	-0.025	0.033	-0.011	0.000	-0.024	0.000	0.000	0.007	-0.026

79.H	-0.009	0.022	-0.012	-0.001	-0.024	0.001	-0.005	0.009	-0.015
80.H	-0.007	0.013	-0.010	-0.001	-0.024	0.000	-0.005	0.018	-0.017
81.H	-0.009	0.026	-0.013	-0.001	-0.024	0.001	-0.005	0.014	-0.017
82.H	-0.024	-0.005	0.002	0.000	-0.023	-0.000	-0.000	0.013	-0.028
83.H	-0.034	-0.013	0.007	0.000	-0.023	-0.000	0.003	0.008	-0.034
84.H	-0.032	0.015	-0.003	0.000	-0.024	-0.000	0.002	0.003	-0.031
85.H	-0.018	0.005	-0.003	-0.000	-0.023	0.000	-0.002	0.018	-0.025
86.H	-0.009	0.006	-0.007	-0.001	-0.024	0.000	-0.004	-0.012	-0.008
87.O	0.000	0.000	0.000	0.000	0.000	0.000	0.000	0.000	0.000
88.H	-0.012	-0.024	0.005	-0.000	-0.023	-0.000	-0.003	0.003	-0.017
89.H	-0.010	0.024	-0.012	-0.000	-0.024	0.001	-0.004	-0.009	-0.010
90.H	-0.004	0.015	-0.011	-0.001	-0.024	0.001	-0.006	0.000	-0.009
91.H	-0.012	0.012	-0.008	-0.000	-0.024	0.000	-0.003	-0.005	-0.013
92.C	0.000	0.000	0.000	0.000	0.000	0.000	0.000	0.000	0.000
93.O	0.000	0.000	0.000	0.000	0.000	0.000	0.000	0.000	0.000
94.H	-0.032	-0.019	0.009	0.000	-0.023	-0.000	0.003	-0.008	-0.027
95.H	-0.028	-0.030	0.012	0.000	-0.022	-0.000	0.002	0.000	-0.027
96.H	-0.022	-0.026	0.009	-0.000	-0.023	-0.000	0.000	-0.007	-0.021

	163			164			165		
	------------------------			------------------------			------------------------		
1.O	0.000	0.000	0.000	0.000	0.000	0.000	0.000	0.000	0.000
2.C	0.000	0.000	0.000	0.000	0.000	0.000	0.000	0.000	0.000
3.C	0.000	0.000	0.000	0.000	0.000	0.000	0.000	0.000	0.000
4.C	0.000	0.000	0.000	0.000	0.000	0.000	0.000	0.000	0.000
5.O	0.000	0.000	0.000	0.000	0.000	0.000	0.000	0.000	0.000
6.N	0.000	0.000	0.000	0.000	0.000	0.000	0.000	0.000	0.000
7.O	0.000	0.000	0.000	0.000	0.000	0.000	0.000	0.000	0.000
8.C	0.000	0.000	0.000	0.000	0.000	0.000	0.000	0.000	0.000
9.C	0.000	0.000	0.000	0.000	0.000	0.000	0.000	0.000	0.000

10.C	0.000	0.000	0.000	0.000	0.000	0.000	0.000	0.000	0.000
11.O	0.000	0.000	0.000	0.000	0.000	0.000	0.000	0.000	0.000
12.C	0.000	0.000	0.000	0.000	0.000	0.000	0.000	0.000	0.000
13.O	0.000	0.000	0.000	0.000	0.000	0.000	0.000	0.000	0.000
14.C	0.000	0.000	0.000	0.000	0.000	0.000	0.000	0.000	0.000
15.C	0.000	0.000	0.000	0.000	0.000	0.000	0.000	0.000	0.000
16.O	0.000	0.000	0.000	0.000	0.000	0.000	0.000	0.000	0.000
17.C	0.000	0.000	0.000	0.000	0.000	0.000	0.000	0.000	0.000
18.C	0.000	0.000	0.000	0.000	0.000	0.000	0.000	0.000	0.000
19.O	0.000	0.000	0.000	0.000	0.000	0.000	0.000	0.000	0.000
20.C	0.000	0.000	0.000	0.000	0.000	0.000	0.000	0.000	0.000
21.C	0.000	0.000	0.000	0.000	0.000	0.000	0.000	0.000	0.000
22.C	0.000	0.000	0.000	0.000	0.000	0.000	0.000	0.000	0.000
23.C	0.000	0.000	0.000	0.000	0.000	0.000	0.000	0.000	0.000
24.C	0.000	0.000	0.000	0.000	0.000	0.000	0.000	0.000	0.000
25.C	0.000	0.000	0.000	0.000	0.000	0.000	0.000	0.000	0.000
26.C	0.000	0.000	0.000	0.000	0.000	0.000	0.000	0.000	0.000
27.C	0.000	0.000	0.000	0.000	0.000	0.000	0.000	0.000	0.000
28.C	0.000	0.000	0.000	0.000	0.000	0.000	0.000	0.000	0.000
29.C	0.000	0.000	0.000	0.000	0.000	0.000	0.000	0.000	0.000
30.C	0.000	0.000	0.000	0.000	0.000	0.000	0.000	0.000	0.000
31.O	0.000	0.000	0.000	0.000	0.000	0.000	0.000	0.000	0.000
32.C	0.000	0.000	0.000	0.000	0.000	0.000	0.000	0.000	0.000
33.C	0.000	0.000	0.000	0.000	0.000	0.000	0.000	0.000	0.000
34.C	0.000	0.000	0.000	0.000	0.000	0.000	0.000	0.000	0.000
35.C	0.000	0.000	0.000	0.000	0.000	0.000	0.000	0.000	0.000
36.C	0.000	0.000	0.000	0.000	0.000	0.000	0.000	0.000	0.000
37.C	0.000	0.000	0.000	0.000	0.000	0.000	0.000	0.000	0.000
38.C	0.000	0.000	0.000	0.000	0.000	0.000	0.000	0.000	0.000
39.C	0.000	0.000	0.000	0.000	0.000	0.000	0.000	0.000	0.000
40.H	0.000	0.000	0.000	0.000	0.000	0.000	0.000	0.000	0.000

41.H	0.000	0.000	0.000	0.000	0.000	0.000	0.000	0.000	0.000
42.H	0.000	0.000	0.000	0.000	0.000	0.000	0.000	0.000	0.000
43.H	0.000	0.000	0.000	0.000	0.000	0.000	0.000	0.000	0.000
44.H	0.000	0.000	0.000	0.000	0.000	0.000	0.000	0.000	0.000
45.H	0.000	0.000	0.000	0.000	0.000	0.000	0.000	0.000	0.000
46.H	0.000	0.000	0.000	0.000	0.000	0.000	0.000	0.000	0.000
47.H	0.000	0.000	0.000	0.000	0.000	0.000	0.000	0.000	0.000
48.H	0.000	0.000	0.000	0.000	0.000	0.000	0.000	0.000	0.000
49.H	0.000	0.000	0.000	0.000	0.000	0.000	0.000	0.000	0.000
50.H	0.000	0.000	0.000	0.000	0.000	0.000	0.000	0.000	0.000
51.H	0.969	-0.012	0.013	0.000	0.982	0.005	0.000	0.000	0.969
52.H	-0.062	-0.002	0.012	-0.015	-0.025	0.005	0.020	-0.006	-0.054
53.H	-0.058	-0.020	0.017	-0.014	-0.032	0.007	0.019	-0.000	-0.058
54.H	-0.046	-0.026	0.015	-0.009	-0.034	0.006	0.011	0.009	-0.053
55.H	-0.048	-0.025	0.016	-0.010	-0.034	0.006	0.016	0.000	-0.053
56.H	-0.019	-0.001	-0.001	0.002	-0.024	-0.000	0.010	-0.026	-0.015
57.H	-0.001	0.002	-0.008	0.009	-0.023	-0.003	0.001	-0.026	0.001
58.H	0.002	-0.003	-0.007	0.010	-0.025	-0.003	-0.004	-0.015	-0.001
59.H	-0.003	-0.020	0.000	0.008	-0.032	0.000	-0.001	-0.008	-0.013
60.H	-0.006	-0.015	-0.000	0.007	-0.030	-0.000	0.004	-0.019	-0.011
61.H	0.007	-0.016	-0.004	0.012	-0.030	-0.002	-0.004	-0.014	-0.002
62.H	-0.008	0.018	-0.011	0.006	-0.017	-0.004	0.006	-0.036	0.003
63.H	-0.022	0.018	-0.007	0.001	-0.017	-0.003	0.014	-0.040	-0.008
64.H	-0.018	0.027	-0.011	0.002	-0.014	-0.004	0.010	-0.039	-0.002
65.H	-0.024	-0.003	0.001	-0.000	-0.025	0.000	0.002	-0.003	-0.025
66.H	-0.015	-0.017	0.003	0.003	-0.031	0.001	-0.008	0.018	-0.026
67.H	-0.014	-0.003	-0.002	0.004	-0.025	-0.001	-0.007	0.009	-0.019
68.H	-0.008	-0.012	-0.001	0.006	-0.029	-0.000	-0.009	0.010	-0.017
69.C	0.000	0.000	0.000	0.000	0.000	0.000	0.000	0.000	0.000
70.H	-0.055	-0.000	0.009	-0.013	-0.024	0.004	0.006	0.018	-0.053
71.H	-0.048	-0.007	0.009	-0.010	-0.027	0.004	-0.001	0.030	-0.052

72.H	-0.051	-0.013	0.012	-0.011	-0.029	0.005	0.003	0.026	-0.056
73.H	-0.032	-0.009	0.005	-0.003	-0.028	0.002	-0.006	0.026	-0.040
74.H	-0.042	0.013	0.001	-0.007	-0.019	0.000	-0.000	0.013	-0.036
75.H	-0.033	0.006	0.001	-0.004	-0.022	0.000	-0.008	0.024	-0.034
76.H	-0.028	0.032	-0.010	-0.002	-0.011	-0.004	-0.003	-0.003	-0.015
77.H	-0.037	0.029	-0.006	-0.005	-0.013	-0.002	-0.001	0.002	-0.025
78.H	-0.027	0.036	-0.011	-0.001	-0.010	-0.005	-0.007	0.002	-0.014
79.H	-0.009	0.024	-0.013	0.006	-0.015	-0.005	-0.014	0.007	-0.005
80.H	-0.007	0.015	-0.010	0.007	-0.018	-0.004	-0.019	0.019	-0.008
81.H	-0.009	0.028	-0.014	0.006	-0.013	-0.006	-0.017	0.012	-0.004
82.H	-0.025	-0.006	0.002	-0.001	-0.026	0.001	-0.005	0.016	-0.030
83.H	-0.036	-0.014	0.008	-0.005	-0.030	0.003	0.003	0.011	-0.041
84.H	-0.035	0.017	-0.003	-0.004	-0.017	-0.001	0.001	0.001	-0.027
85.H	-0.019	0.005	-0.003	0.002	-0.022	-0.001	-0.012	0.020	-0.022
86.H	-0.008	0.007	-0.007	0.006	-0.021	-0.003	-0.002	-0.015	-0.006
87.O	0.000	0.000	0.000	0.000	0.000	0.000	0.000	0.000	0.000
88.H	-0.013	-0.026	0.005	0.004	-0.035	0.002	-0.003	0.007	-0.026
89.H	-0.010	0.026	-0.013	0.005	-0.014	-0.005	-0.005	-0.014	-0.001
90.H	-0.004	0.016	-0.012	0.008	-0.018	-0.005	-0.011	-0.002	-0.001
91.H	-0.012	0.013	-0.008	0.005	-0.019	-0.003	-0.004	-0.008	-0.008
92.C	0.000	0.000	0.000	0.000	0.000	0.000	0.000	0.000	0.000
93.O	0.000	0.000	0.000	0.000	0.000	0.000	0.000	0.000	0.000
94.H	-0.034	-0.021	0.010	-0.004	-0.032	0.004	0.012	-0.006	-0.038
95.H	-0.029	-0.033	0.013	-0.002	-0.037	0.005	0.007	0.005	-0.041
96.H	-0.023	-0.028	0.009	0.000	-0.035	0.004	0.007	-0.003	-0.033

	166			167			168		
	------------------------			------------------------			------------------------		
1.O	0.000	0.000	0.000	0.000	0.000	0.000	0.000	0.000	0.000
2.C	0.000	0.000	0.000	0.000	0.000	0.000	0.000	0.000	0.000

3.C	0.000	0.000	0.000	0.000	0.000	0.000	0.000	0.000	0.000
4.C	0.000	0.000	0.000	0.000	0.000	0.000	0.000	0.000	0.000
5.O	0.000	0.000	0.000	0.000	0.000	0.000	0.000	0.000	0.000
6.N	0.000	0.000	0.000	0.000	0.000	0.000	0.000	0.000	0.000
7.O	0.000	0.000	0.000	0.000	0.000	0.000	0.000	0.000	0.000
8.C	0.000	0.000	0.000	0.000	0.000	0.000	0.000	0.000	0.000
9.C	0.000	0.000	0.000	0.000	0.000	0.000	0.000	0.000	0.000
10.C	0.000	0.000	0.000	0.000	0.000	0.000	0.000	0.000	0.000
11.O	0.000	0.000	0.000	0.000	0.000	0.000	0.000	0.000	0.000
12.C	0.000	0.000	0.000	0.000	0.000	0.000	0.000	0.000	0.000
13.O	0.000	0.000	0.000	0.000	0.000	0.000	0.000	0.000	0.000
14.C	0.000	0.000	0.000	0.000	0.000	0.000	0.000	0.000	0.000
15.C	0.000	0.000	0.000	0.000	0.000	0.000	0.000	0.000	0.000
16.O	0.000	0.000	0.000	0.000	0.000	0.000	0.000	0.000	0.000
17.C	0.000	0.000	0.000	0.000	0.000	0.000	0.000	0.000	0.000
18.C	0.000	0.000	0.000	0.000	0.000	0.000	0.000	0.000	0.000
19.O	0.000	0.000	0.000	0.000	0.000	0.000	0.000	0.000	0.000
20.C	0.000	0.000	0.000	0.000	0.000	0.000	0.000	0.000	0.000
21.C	0.000	0.000	0.000	0.000	0.000	0.000	0.000	0.000	0.000
22.C	0.000	0.000	0.000	0.000	0.000	0.000	0.000	0.000	0.000
23.C	0.000	0.000	0.000	0.000	0.000	0.000	0.000	0.000	0.000
24.C	0.000	0.000	0.000	0.000	0.000	0.000	0.000	0.000	0.000
25.C	0.000	0.000	0.000	0.000	0.000	0.000	0.000	0.000	0.000
26.C	0.000	0.000	0.000	0.000	0.000	0.000	0.000	0.000	0.000
27.C	0.000	0.000	0.000	0.000	0.000	0.000	0.000	0.000	0.000
28.C	0.000	0.000	0.000	0.000	0.000	0.000	0.000	0.000	0.000
29.C	0.000	0.000	0.000	0.000	0.000	0.000	0.000	0.000	0.000
30.C	0.000	0.000	0.000	0.000	0.000	0.000	0.000	0.000	0.000
31.O	0.000	0.000	0.000	0.000	0.000	0.000	0.000	0.000	0.000
32.C	0.000	0.000	0.000	0.000	0.000	0.000	0.000	0.000	0.000
33.C	0.000	0.000	0.000	0.000	0.000	0.000	0.000	0.000	0.000

34.C	0.000	0.000	0.000	0.000	0.000	0.000	0.000	0.000	0.000
35.C	0.000	0.000	0.000	0.000	0.000	0.000	0.000	0.000	0.000
36.C	0.000	0.000	0.000	0.000	0.000	0.000	0.000	0.000	0.000
37.C	0.000	0.000	0.000	0.000	0.000	0.000	0.000	0.000	0.000
38.C	0.000	0.000	0.000	0.000	0.000	0.000	0.000	0.000	0.000
39.C	0.000	0.000	0.000	0.000	0.000	0.000	0.000	0.000	0.000
40.H	0.000	0.000	0.000	0.000	0.000	0.000	0.000	0.000	0.000
41.H	0.000	0.000	0.000	0.000	0.000	0.000	0.000	0.000	0.000
42.H	0.000	0.000	0.000	0.000	0.000	0.000	0.000	0.000	0.000
43.H	0.000	0.000	0.000	0.000	0.000	0.000	0.000	0.000	0.000
44.H	0.000	0.000	0.000	0.000	0.000	0.000	0.000	0.000	0.000
45.H	0.000	0.000	0.000	0.000	0.000	0.000	0.000	0.000	0.000
46.H	0.000	0.000	0.000	0.000	0.000	0.000	0.000	0.000	0.000
47.H	0.000	0.000	0.000	0.000	0.000	0.000	0.000	0.000	0.000
48.H	0.000	0.000	0.000	0.000	0.000	0.000	0.000	0.000	0.000
49.H	0.000	0.000	0.000	0.000	0.000	0.000	0.000	0.000	0.000
50.H	0.000	0.000	0.000	0.000	0.000	0.000	0.000	0.000	0.000
51.H	0.000	0.000	0.000	0.000	0.000	0.000	0.000	0.000	0.000
52.H	0.958	-0.003	0.018	0.000	0.982	-0.007	0.000	0.000	0.968
53.H	-0.073	-0.025	0.029	-0.003	-0.030	-0.006	0.018	-0.002	-0.060
54.H	-0.056	-0.034	0.028	-0.002	-0.028	-0.004	0.011	0.008	-0.053
55.H	-0.061	-0.033	0.029	-0.002	-0.031	-0.004	0.014	-0.001	-0.052
56.H	-0.030	0.000	0.001	0.001	-0.034	0.004	0.006	-0.030	-0.014
57.H	-0.005	0.004	-0.009	0.002	-0.033	0.008	-0.003	-0.029	0.003
58.H	0.002	-0.003	-0.007	0.002	-0.031	0.007	-0.006	-0.017	0.002
59.H	-0.005	-0.026	0.007	0.002	-0.033	0.007	-0.004	-0.011	-0.008
60.H	-0.012	-0.019	0.006	0.002	-0.035	0.007	-0.000	-0.023	-0.007
61.H	0.007	-0.020	0.000	0.003	-0.033	0.009	-0.007	-0.017	0.003
62.H	-0.016	0.025	-0.016	0.002	-0.033	0.006	0.001	-0.040	0.003
63.H	-0.035	0.024	-0.010	0.001	-0.035	0.004	0.009	-0.044	-0.008
64.H	-0.028	0.036	-0.018	0.001	-0.032	0.004	0.006	-0.043	-0.003

65.H	-0.027	-0.005	0.003	0.000	-0.027	0.000	0.002	-0.004	-0.025
66.H	-0.010	-0.023	0.007	0.000	-0.022	0.001	-0.006	0.019	-0.026
67.H	-0.010	-0.005	-0.002	0.001	-0.022	0.001	-0.006	0.009	-0.020
68.H	-0.003	-0.016	0.001	0.001	-0.024	0.003	-0.008	0.010	-0.016
69.C	0.000	0.000	0.000	0.000	0.000	0.000	0.000	0.000	0.000
70.H	-0.060	-0.001	0.012	-0.003	-0.018	-0.009	0.010	0.020	-0.059
71.H	-0.047	-0.011	0.013	-0.003	-0.016	-0.008	0.004	0.033	-0.057
72.H	-0.053	-0.019	0.019	-0.003	-0.019	-0.008	0.007	0.028	-0.060
73.H	-0.028	-0.013	0.008	-0.001	-0.018	-0.004	-0.002	0.029	-0.043
74.H	-0.042	0.016	-0.003	-0.002	-0.017	-0.006	0.003	0.015	-0.042
75.H	-0.028	0.006	-0.003	-0.002	-0.015	-0.005	-0.003	0.027	-0.039
76.H	-0.027	0.041	-0.021	-0.001	-0.018	-0.003	-0.001	-0.002	-0.021
77.H	-0.037	0.037	-0.016	-0.002	-0.017	-0.005	0.002	0.004	-0.032
78.H	-0.023	0.046	-0.025	-0.001	-0.015	-0.003	-0.003	0.004	-0.021
79.H	0.000	0.030	-0.024	0.001	-0.017	0.001	-0.011	0.009	-0.009
80.H	0.006	0.018	-0.019	0.001	-0.014	0.001	-0.014	0.022	-0.012
81.H	0.002	0.035	-0.027	0.001	-0.014	0.000	-0.013	0.014	-0.010
82.H	-0.023	-0.008	0.004	-0.001	-0.020	-0.002	-0.003	0.018	-0.033
83.H	-0.040	-0.019	0.015	-0.001	-0.024	-0.003	0.005	0.012	-0.042
84.H	-0.037	0.021	-0.008	-0.001	-0.020	-0.003	0.003	0.001	-0.032
85.H	-0.011	0.005	-0.007	-0.000	-0.017	-0.002	-0.008	0.022	-0.026
86.H	-0.010	0.009	-0.010	0.002	-0.028	0.005	-0.003	-0.017	-0.005
87.O	0.000	0.000	0.000	0.000	0.000	0.000	0.000	0.000	0.000
88.H	-0.012	-0.035	0.014	0.001	-0.029	0.003	-0.003	0.007	-0.023
89.H	-0.009	0.034	-0.023	0.001	-0.023	0.003	-0.005	-0.015	-0.003
90.H	0.002	0.021	-0.020	0.001	-0.021	0.004	-0.010	-0.002	-0.003
91.H	-0.011	0.017	-0.013	0.001	-0.024	0.003	-0.004	-0.008	-0.010
92.C	0.000	0.000	0.000	0.000	0.000	0.000	0.000	0.000	0.000
93.O	0.000	0.000	0.000	0.000	0.000	0.000	0.000	0.000	0.000
94.H	-0.044	-0.027	0.020	-0.001	-0.032	-0.000	0.009	-0.008	-0.037
95.H	-0.036	-0.043	0.026	-0.000	-0.031	0.000	0.006	0.004	-0.039

96.H	-0.030	-0.036	0.021	0.000	-0.033	0.002	0.005	-0.005	-0.030

	169			170			171		
	------------------------			------------------------			------------------------		
1.O	0.000	0.000	0.000	0.000	0.000	0.000	0.000	0.000	0.000
2.C	0.000	0.000	0.000	0.000	0.000	0.000	0.000	0.000	0.000
3.C	0.000	0.000	0.000	0.000	0.000	0.000	0.000	0.000	0.000
4.C	0.000	0.000	0.000	0.000	0.000	0.000	0.000	0.000	0.000
5.O	0.000	0.000	0.000	0.000	0.000	0.000	0.000	0.000	0.000
6.N	0.000	0.000	0.000	0.000	0.000	0.000	0.000	0.000	0.000
7.O	0.000	0.000	0.000	0.000	0.000	0.000	0.000	0.000	0.000
8.C	0.000	0.000	0.000	0.000	0.000	0.000	0.000	0.000	0.000
9.C	0.000	0.000	0.000	0.000	0.000	0.000	0.000	0.000	0.000
10.C	0.000	0.000	0.000	0.000	0.000	0.000	0.000	0.000	0.000
11.O	0.000	0.000	0.000	0.000	0.000	0.000	0.000	0.000	0.000
12.C	0.000	0.000	0.000	0.000	0.000	0.000	0.000	0.000	0.000
13.O	0.000	0.000	0.000	0.000	0.000	0.000	0.000	0.000	0.000
14.C	0.000	0.000	0.000	0.000	0.000	0.000	0.000	0.000	0.000
15.C	0.000	0.000	0.000	0.000	0.000	0.000	0.000	0.000	0.000
16.O	0.000	0.000	0.000	0.000	0.000	0.000	0.000	0.000	0.000
17.C	0.000	0.000	0.000	0.000	0.000	0.000	0.000	0.000	0.000
18.C	0.000	0.000	0.000	0.000	0.000	0.000	0.000	0.000	0.000
19.O	0.000	0.000	0.000	0.000	0.000	0.000	0.000	0.000	0.000
20.C	0.000	0.000	0.000	0.000	0.000	0.000	0.000	0.000	0.000
21.C	0.000	0.000	0.000	0.000	0.000	0.000	0.000	0.000	0.000
22.C	0.000	0.000	0.000	0.000	0.000	0.000	0.000	0.000	0.000
23.C	0.000	0.000	0.000	0.000	0.000	0.000	0.000	0.000	0.000
24.C	0.000	0.000	0.000	0.000	0.000	0.000	0.000	0.000	0.000
25.C	0.000	0.000	0.000	0.000	0.000	0.000	0.000	0.000	0.000
26.C	0.000	0.000	0.000	0.000	0.000	0.000	0.000	0.000	0.000

27.C	0.000	0.000	0.000	0.000	0.000	0.000	0.000	0.000	0.000
28.C	0.000	0.000	0.000	0.000	0.000	0.000	0.000	0.000	0.000
29.C	0.000	0.000	0.000	0.000	0.000	0.000	0.000	0.000	0.000
30.C	0.000	0.000	0.000	0.000	0.000	0.000	0.000	0.000	0.000
31.O	0.000	0.000	0.000	0.000	0.000	0.000	0.000	0.000	0.000
32.C	0.000	0.000	0.000	0.000	0.000	0.000	0.000	0.000	0.000
33.C	0.000	0.000	0.000	0.000	0.000	0.000	0.000	0.000	0.000
34.C	0.000	0.000	0.000	0.000	0.000	0.000	0.000	0.000	0.000
35.C	0.000	0.000	0.000	0.000	0.000	0.000	0.000	0.000	0.000
36.C	0.000	0.000	0.000	0.000	0.000	0.000	0.000	0.000	0.000
37.C	0.000	0.000	0.000	0.000	0.000	0.000	0.000	0.000	0.000
38.C	0.000	0.000	0.000	0.000	0.000	0.000	0.000	0.000	0.000
39.C	0.000	0.000	0.000	0.000	0.000	0.000	0.000	0.000	0.000
40.H	0.000	0.000	0.000	0.000	0.000	0.000	0.000	0.000	0.000
41.H	0.000	0.000	0.000	0.000	0.000	0.000	0.000	0.000	0.000
42.H	0.000	0.000	0.000	0.000	0.000	0.000	0.000	0.000	0.000
43.H	0.000	0.000	0.000	0.000	0.000	0.000	0.000	0.000	0.000
44.H	0.000	0.000	0.000	0.000	0.000	0.000	0.000	0.000	0.000
45.H	0.000	0.000	0.000	0.000	0.000	0.000	0.000	0.000	0.000
46.H	0.000	0.000	0.000	0.000	0.000	0.000	0.000	0.000	0.000
47.H	0.000	0.000	0.000	0.000	0.000	0.000	0.000	0.000	0.000
48.H	0.000	0.000	0.000	0.000	0.000	0.000	0.000	0.000	0.000
49.H	0.000	0.000	0.000	0.000	0.000	0.000	0.000	0.000	0.000
50.H	0.000	0.000	0.000	0.000	0.000	0.000	0.000	0.000	0.000
51.H	0.000	0.000	0.000	0.000	0.000	0.000	0.000	0.000	0.000
52.H	0.000	0.000	0.000	0.000	0.000	0.000	0.000	0.000	0.000
53.H	0.959	-0.025	0.031	0.000	0.974	0.003	0.000	0.000	0.959
54.H	-0.057	-0.033	0.030	-0.018	-0.046	0.005	0.020	0.015	-0.070
55.H	-0.064	-0.032	0.032	-0.019	-0.049	0.006	0.029	0.006	-0.071
56.H	-0.034	0.001	0.002	0.002	-0.038	0.004	0.023	-0.029	-0.021
57.H	-0.010	0.005	-0.008	0.016	-0.035	0.005	0.008	-0.029	-0.000

58.H	-0.002	-0.003	-0.007	0.017	-0.035	0.005	-0.002	-0.016	-0.003
59.H	-0.009	-0.025	0.009	0.014	-0.048	0.009	0.003	-0.005	-0.022
60.H	-0.017	-0.018	0.007	0.012	-0.048	0.009	0.013	-0.018	-0.020
61.H	0.002	-0.019	0.002	0.021	-0.046	0.009	-0.001	-0.012	-0.008
62.H	-0.021	0.025	-0.017	0.010	-0.024	0.001	0.016	-0.044	0.006
63.H	-0.040	0.025	-0.010	0.000	-0.027	0.000	0.029	-0.048	-0.007
64.H	-0.033	0.036	-0.019	0.003	-0.018	-0.002	0.022	-0.049	0.004
65.H	-0.030	-0.004	0.004	-0.002	-0.030	0.001	0.006	-0.003	-0.030
66.H	-0.011	-0.023	0.008	0.005	-0.034	0.003	-0.013	0.023	-0.034
67.H	-0.011	-0.005	-0.002	0.006	-0.025	0.001	-0.011	0.010	-0.021
68.H	-0.004	-0.016	0.002	0.010	-0.033	0.004	-0.014	0.014	-0.022
69.C	0.000	0.000	0.000	0.000	0.000	0.000	0.000	0.000	0.000
70.H	-0.059	-0.002	0.012	-0.025	-0.018	-0.005	0.008	0.020	-0.060
71.H	-0.046	-0.012	0.014	-0.019	-0.020	-0.004	-0.005	0.034	-0.059
72.H	-0.053	-0.019	0.020	-0.021	-0.027	-0.002	0.003	0.031	-0.067
73.H	-0.028	-0.014	0.009	-0.008	-0.023	-0.002	-0.012	0.031	-0.046
74.H	-0.042	0.015	-0.003	-0.015	-0.008	-0.007	-0.002	0.011	-0.036
75.H	-0.027	0.006	-0.003	-0.009	-0.010	-0.006	-0.015	0.025	-0.034
76.H	-0.028	0.040	-0.023	-0.005	0.003	-0.010	-0.005	-0.010	-0.005
77.H	-0.037	0.036	-0.017	-0.011	0.002	-0.010	-0.002	-0.003	-0.018
78.H	-0.023	0.045	-0.027	-0.004	0.009	-0.012	-0.012	-0.005	-0.002
79.H	-0.000	0.029	-0.026	0.009	-0.001	-0.007	-0.024	0.002	0.005
80.H	0.007	0.017	-0.021	0.011	-0.004	-0.006	-0.033	0.018	-0.001
81.H	0.003	0.034	-0.030	0.009	0.005	-0.009	-0.030	0.007	0.008
82.H	-0.023	-0.009	0.004	-0.003	-0.024	-0.001	-0.009	0.019	-0.035
83.H	-0.041	-0.019	0.016	-0.011	-0.034	0.002	0.006	0.015	-0.051
84.H	-0.037	0.021	-0.008	-0.009	-0.010	-0.006	0.002	-0.003	-0.024
85.H	-0.011	0.004	-0.007	0.002	-0.013	-0.004	-0.021	0.021	-0.021
86.H	-0.013	0.009	-0.010	0.010	-0.026	0.001	0.001	-0.018	-0.005
87.O	0.000	0.000	0.000	0.000	0.000	0.000	0.000	0.000	0.000
88.H	-0.015	-0.034	0.016	0.007	-0.047	0.008	-0.002	0.014	-0.038

89.H	-0.011	0.033	-0.025	0.008	-0.008	-0.005	-0.005	-0.021	0.008
90.H	0.001	0.020	-0.021	0.013	-0.012	-0.003	-0.017	-0.006	0.005
91.H	-0.013	0.016	-0.014	0.007	-0.017	-0.002	-0.005	-0.011	-0.005
92.C	0.000	0.000	0.000	0.000	0.000	0.000	0.000	0.000	0.000
93.O	0.000	0.000	0.000	0.000	0.000	0.000	0.000	0.000	0.000
94.H	-0.048	-0.026	0.023	-0.009	-0.048	0.007	0.023	-0.003	-0.053
95.H	-0.039	-0.042	0.029	-0.005	-0.054	0.009	0.014	0.013	-0.059
96.H	-0.034	-0.036	0.024	-0.001	-0.053	0.009	0.015	0.002	-0.048

	172			173			174		
1.O	0.000	0.000	0.000	0.000	0.000	0.000	0.000	0.000	0.000
2.C	0.000	0.000	0.000	0.000	0.000	0.000	0.000	0.000	0.000
3.C	0.000	0.000	0.000	0.000	0.000	0.000	0.000	0.000	0.000
4.C	0.000	0.000	0.000	0.000	0.000	0.000	0.000	0.000	0.000
5.O	0.000	0.000	0.000	0.000	0.000	0.000	0.000	0.000	0.000
6.N	0.000	0.000	0.000	0.000	0.000	0.000	0.000	0.000	0.000
7.O	0.000	0.000	0.000	0.000	0.000	0.000	0.000	0.000	0.000
8.C	0.000	0.000	0.000	0.000	0.000	0.000	0.000	0.000	0.000
9.C	0.000	0.000	0.000	0.000	0.000	0.000	0.000	0.000	0.000
10.C	0.000	0.000	0.000	0.000	0.000	0.000	0.000	0.000	0.000
11.O	0.000	0.000	0.000	0.000	0.000	0.000	0.000	0.000	0.000
12.C	0.000	0.000	0.000	0.000	0.000	0.000	0.000	0.000	0.000
13.O	0.000	0.000	0.000	0.000	0.000	0.000	0.000	0.000	0.000
14.C	0.000	0.000	0.000	0.000	0.000	0.000	0.000	0.000	0.000
15.C	0.000	0.000	0.000	0.000	0.000	0.000	0.000	0.000	0.000
16.O	0.000	0.000	0.000	0.000	0.000	0.000	0.000	0.000	0.000
17.C	0.000	0.000	0.000	0.000	0.000	0.000	0.000	0.000	0.000
18.C	0.000	0.000	0.000	0.000	0.000	0.000	0.000	0.000	0.000
19.O	0.000	0.000	0.000	0.000	0.000	0.000	0.000	0.000	0.000

20.C	0.000	0.000	0.000	0.000	0.000	0.000	0.000	0.000	0.000
21.C	0.000	0.000	0.000	0.000	0.000	0.000	0.000	0.000	0.000
22.C	0.000	0.000	0.000	0.000	0.000	0.000	0.000	0.000	0.000
23.C	0.000	0.000	0.000	0.000	0.000	0.000	0.000	0.000	0.000
24.C	0.000	0.000	0.000	0.000	0.000	0.000	0.000	0.000	0.000
25.C	0.000	0.000	0.000	0.000	0.000	0.000	0.000	0.000	0.000
26.C	0.000	0.000	0.000	0.000	0.000	0.000	0.000	0.000	0.000
27.C	0.000	0.000	0.000	0.000	0.000	0.000	0.000	0.000	0.000
28.C	0.000	0.000	0.000	0.000	0.000	0.000	0.000	0.000	0.000
29.C	0.000	0.000	0.000	0.000	0.000	0.000	0.000	0.000	0.000
30.C	0.000	0.000	0.000	0.000	0.000	0.000	0.000	0.000	0.000
31.O	0.000	0.000	0.000	0.000	0.000	0.000	0.000	0.000	0.000
32.C	0.000	0.000	0.000	0.000	0.000	0.000	0.000	0.000	0.000
33.C	0.000	0.000	0.000	0.000	0.000	0.000	0.000	0.000	0.000
34.C	0.000	0.000	0.000	0.000	0.000	0.000	0.000	0.000	0.000
35.C	0.000	0.000	0.000	0.000	0.000	0.000	0.000	0.000	0.000
36.C	0.000	0.000	0.000	0.000	0.000	0.000	0.000	0.000	0.000
37.C	0.000	0.000	0.000	0.000	0.000	0.000	0.000	0.000	0.000
38.C	0.000	0.000	0.000	0.000	0.000	0.000	0.000	0.000	0.000
39.C	0.000	0.000	0.000	0.000	0.000	0.000	0.000	0.000	0.000
40.H	0.000	0.000	0.000	0.000	0.000	0.000	0.000	0.000	0.000
41.H	0.000	0.000	0.000	0.000	0.000	0.000	0.000	0.000	0.000
42.H	0.000	0.000	0.000	0.000	0.000	0.000	0.000	0.000	0.000
43.H	0.000	0.000	0.000	0.000	0.000	0.000	0.000	0.000	0.000
44.H	0.000	0.000	0.000	0.000	0.000	0.000	0.000	0.000	0.000
45.H	0.000	0.000	0.000	0.000	0.000	0.000	0.000	0.000	0.000
46.H	0.000	0.000	0.000	0.000	0.000	0.000	0.000	0.000	0.000
47.H	0.000	0.000	0.000	0.000	0.000	0.000	0.000	0.000	0.000
48.H	0.000	0.000	0.000	0.000	0.000	0.000	0.000	0.000	0.000
49.H	0.000	0.000	0.000	0.000	0.000	0.000	0.000	0.000	0.000
50.H	0.000	0.000	0.000	0.000	0.000	0.000	0.000	0.000	0.000

51.H	0.000	0.000	0.000	0.000	0.000	0.000	0.000	0.000	0.000
52.H	0.000	0.000	0.000	0.000	0.000	0.000	0.000	0.000	0.000
53.H	0.000	0.000	0.000	0.000	0.000	0.000	0.000	0.000	0.000
54.H	0.971	-0.025	0.020	0.000	0.969	0.016	0.000	0.000	0.961
55.H	-0.053	-0.024	0.021	-0.029	-0.055	0.017	0.032	0.009	-0.073
56.H	-0.029	0.000	0.001	0.001	-0.031	0.001	0.027	-0.023	-0.026
57.H	-0.011	0.003	-0.006	0.020	-0.027	-0.004	0.011	-0.024	-0.008
58.H	-0.007	-0.002	-0.005	0.023	-0.032	-0.003	0.000	-0.012	-0.010
59.H	-0.012	-0.019	0.004	0.018	-0.050	0.004	0.005	-0.000	-0.031
60.H	-0.016	-0.014	0.003	0.015	-0.046	0.004	0.017	-0.012	-0.028
61.H	-0.003	-0.014	-0.001	0.029	-0.046	0.001	0.002	-0.007	-0.018
62.H	-0.019	0.018	-0.011	0.012	-0.011	-0.008	0.020	-0.038	0.001
63.H	-0.033	0.018	-0.006	-0.002	-0.012	-0.005	0.035	-0.042	-0.010
64.H	-0.028	0.026	-0.012	0.002	-0.002	-0.009	0.027	-0.044	0.001
65.H	-0.029	-0.004	0.003	-0.003	-0.031	0.002	0.007	-0.001	-0.032
66.H	-0.018	-0.017	0.005	0.007	-0.044	0.005	-0.015	0.022	-0.038
67.H	-0.017	-0.004	-0.001	0.008	-0.030	-0.001	-0.012	0.010	-0.024
68.H	-0.012	-0.012	0.001	0.014	-0.039	0.001	-0.014	0.014	-0.027
69.C	0.000	0.000	0.000	0.000	0.000	0.000	0.000	0.000	0.000
70.H	-0.054	-0.002	0.010	-0.035	-0.025	0.007	0.007	0.017	-0.055
71.H	-0.046	-0.009	0.011	-0.027	-0.032	0.008	-0.007	0.030	-0.056
72.H	-0.050	-0.015	0.015	-0.030	-0.039	0.011	0.002	0.028	-0.064
73.H	-0.032	-0.011	0.007	-0.011	-0.034	0.005	-0.014	0.028	-0.045
74.H	-0.042	0.011	0.000	-0.021	-0.011	-0.001	-0.003	0.008	-0.032
75.H	-0.032	0.004	0.000	-0.012	-0.018	-0.001	-0.018	0.020	-0.031
76.H	-0.030	0.029	-0.013	-0.007	0.008	-0.011	-0.006	-0.013	-0.002
77.H	-0.038	0.026	-0.008	-0.016	0.005	-0.008	-0.004	-0.007	-0.013
78.H	-0.028	0.032	-0.015	-0.005	0.013	-0.013	-0.014	-0.009	0.003
79.H	-0.011	0.021	-0.015	0.013	-0.001	-0.013	-0.026	-0.001	0.006
80.H	-0.007	0.012	-0.012	0.016	-0.009	-0.010	-0.036	0.013	-0.001
81.H	-0.010	0.024	-0.017	0.013	0.005	-0.015	-0.033	0.002	0.010

82.H	-0.027	-0.007	0.004	-0.004	-0.032	0.003	-0.010	0.017	-0.036
83.H	-0.039	-0.014	0.011	-0.015	-0.041	0.009	0.007	0.015	-0.053
84.H	-0.037	0.015	-0.004	-0.014	-0.009	-0.003	0.002	-0.004	-0.022
85.H	-0.019	0.003	-0.004	0.003	-0.020	-0.003	-0.024	0.017	-0.020
86.H	-0.016	0.006	-0.007	0.013	-0.021	-0.005	0.003	-0.015	-0.010
87.O	0.000	0.000	0.000	0.000	0.000	0.000	0.000	0.000	0.000
88.H	-0.018	-0.026	0.010	0.009	-0.055	0.009	-0.001	0.016	-0.045
89.H	-0.016	0.024	-0.015	0.011	-0.000	-0.012	-0.005	-0.021	0.007
90.H	-0.008	0.014	-0.013	0.018	-0.010	-0.010	-0.018	-0.007	0.003
91.H	-0.017	0.012	-0.009	0.009	-0.014	-0.007	-0.004	-0.011	-0.007
92.C	0.000	0.000	0.000	0.000	0.000	0.000	0.000	0.000	0.000
93.O	0.000	0.000	0.000	0.000	0.000	0.000	0.000	0.000	0.000
94.H	-0.041	-0.020	0.014	-0.014	-0.051	0.012	0.026	0.002	-0.057
95.H	-0.035	-0.032	0.018	-0.009	-0.063	0.015	0.017	0.016	-0.065
96.H	-0.031	-0.027	0.014	-0.003	-0.058	0.012	0.018	0.007	-0.055

	175			176			177		
1.O	0.000	0.000	0.000	0.000	0.000	0.000	0.000	0.000	0.000
2.C	0.000	0.000	0.000	0.000	0.000	0.000	0.000	0.000	0.000
3.C	0.000	0.000	0.000	0.000	0.000	0.000	0.000	0.000	0.000
4.C	0.000	0.000	0.000	0.000	0.000	0.000	0.000	0.000	0.000
5.O	0.000	0.000	0.000	0.000	0.000	0.000	0.000	0.000	0.000
6.N	0.000	0.000	0.000	0.000	0.000	0.000	0.000	0.000	0.000
7.O	0.000	0.000	0.000	0.000	0.000	0.000	0.000	0.000	0.000
8.C	0.000	0.000	0.000	0.000	0.000	0.000	0.000	0.000	0.000
9.C	0.000	0.000	0.000	0.000	0.000	0.000	0.000	0.000	0.000
10.C	0.000	0.000	0.000	0.000	0.000	0.000	0.000	0.000	0.000
11.O	0.000	0.000	0.000	0.000	0.000	0.000	0.000	0.000	0.000
12.C	0.000	0.000	0.000	0.000	0.000	0.000	0.000	0.000	0.000

13.O	0.000	0.000	0.000	0.000	0.000	0.000	0.000	0.000	0.000
14.C	0.000	0.000	0.000	0.000	0.000	0.000	0.000	0.000	0.000
15.C	0.000	0.000	0.000	0.000	0.000	0.000	0.000	0.000	0.000
16.O	0.000	0.000	0.000	0.000	0.000	0.000	0.000	0.000	0.000
17.C	0.000	0.000	0.000	0.000	0.000	0.000	0.000	0.000	0.000
18.C	0.000	0.000	0.000	0.000	0.000	0.000	0.000	0.000	0.000
19.O	0.000	0.000	0.000	0.000	0.000	0.000	0.000	0.000	0.000
20.C	0.000	0.000	0.000	0.000	0.000	0.000	0.000	0.000	0.000
21.C	0.000	0.000	0.000	0.000	0.000	0.000	0.000	0.000	0.000
22.C	0.000	0.000	0.000	0.000	0.000	0.000	0.000	0.000	0.000
23.C	0.000	0.000	0.000	0.000	0.000	0.000	0.000	0.000	0.000
24.C	0.000	0.000	0.000	0.000	0.000	0.000	0.000	0.000	0.000
25.C	0.000	0.000	0.000	0.000	0.000	0.000	0.000	0.000	0.000
26.C	0.000	0.000	0.000	0.000	0.000	0.000	0.000	0.000	0.000
27.C	0.000	0.000	0.000	0.000	0.000	0.000	0.000	0.000	0.000
28.C	0.000	0.000	0.000	0.000	0.000	0.000	0.000	0.000	0.000
29.C	0.000	0.000	0.000	0.000	0.000	0.000	0.000	0.000	0.000
30.C	0.000	0.000	0.000	0.000	0.000	0.000	0.000	0.000	0.000
31.O	0.000	0.000	0.000	0.000	0.000	0.000	0.000	0.000	0.000
32.C	0.000	0.000	0.000	0.000	0.000	0.000	0.000	0.000	0.000
33.C	0.000	0.000	0.000	0.000	0.000	0.000	0.000	0.000	0.000
34.C	0.000	0.000	0.000	0.000	0.000	0.000	0.000	0.000	0.000
35.C	0.000	0.000	0.000	0.000	0.000	0.000	0.000	0.000	0.000
36.C	0.000	0.000	0.000	0.000	0.000	0.000	0.000	0.000	0.000
37.C	0.000	0.000	0.000	0.000	0.000	0.000	0.000	0.000	0.000
38.C	0.000	0.000	0.000	0.000	0.000	0.000	0.000	0.000	0.000
39.C	0.000	0.000	0.000	0.000	0.000	0.000	0.000	0.000	0.000
40.H	0.000	0.000	0.000	0.000	0.000	0.000	0.000	0.000	0.000
41.H	0.000	0.000	0.000	0.000	0.000	0.000	0.000	0.000	0.000
42.H	0.000	0.000	0.000	0.000	0.000	0.000	0.000	0.000	0.000
43.H	0.000	0.000	0.000	0.000	0.000	0.000	0.000	0.000	0.000

44.H	0.000	0.000	0.000	0.000	0.000	0.000	0.000	0.000	0.000
45.H	0.000	0.000	0.000	0.000	0.000	0.000	0.000	0.000	0.000
46.H	0.000	0.000	0.000	0.000	0.000	0.000	0.000	0.000	0.000
47.H	0.000	0.000	0.000	0.000	0.000	0.000	0.000	0.000	0.000
48.H	0.000	0.000	0.000	0.000	0.000	0.000	0.000	0.000	0.000
49.H	0.000	0.000	0.000	0.000	0.000	0.000	0.000	0.000	0.000
50.H	0.000	0.000	0.000	0.000	0.000	0.000	0.000	0.000	0.000
51.H	0.000	0.000	0.000	0.000	0.000	0.000	0.000	0.000	0.000
52.H	0.000	0.000	0.000	0.000	0.000	0.000	0.000	0.000	0.000
53.H	0.000	0.000	0.000	0.000	0.000	0.000	0.000	0.000	0.000
54.H	0.000	0.000	0.000	0.000	0.000	0.000	0.000	0.000	0.000
55.H	0.964	-0.030	0.036	0.000	0.965	0.012	0.000	0.000	0.956
56.H	-0.043	0.001	0.005	0.000	-0.044	0.006	0.034	-0.023	-0.030
57.H	-0.020	0.004	-0.006	0.021	-0.039	0.005	0.015	-0.023	-0.010
58.H	-0.012	-0.003	-0.004	0.024	-0.040	0.005	0.002	-0.011	-0.013
59.H	-0.018	-0.023	0.013	0.019	-0.061	0.013	0.008	0.003	-0.037
60.H	-0.027	-0.016	0.011	0.015	-0.060	0.012	0.021	-0.010	-0.035
61.H	-0.009	-0.017	0.006	0.030	-0.058	0.012	0.003	-0.004	-0.024
62.H	-0.031	0.023	-0.015	0.013	-0.023	-0.001	0.026	-0.040	0.001
63.H	-0.050	0.023	-0.008	-0.003	-0.026	-0.000	0.043	-0.044	-0.011
64.H	-0.042	0.032	-0.018	0.002	-0.014	-0.005	0.033	-0.047	0.002
65.H	-0.034	-0.005	0.006	-0.004	-0.035	0.003	0.010	0.000	-0.036
66.H	-0.014	-0.022	0.011	0.007	-0.043	0.006	-0.017	0.025	-0.042
67.H	-0.014	-0.006	-0.000	0.008	-0.029	0.001	-0.014	0.011	-0.026
68.H	-0.009	-0.016	0.005	0.015	-0.040	0.005	-0.016	0.016	-0.031
69.C	0.000	0.000	0.000	0.000	0.000	0.000	0.000	0.000	0.000
70.H	-0.055	-0.004	0.013	-0.039	-0.020	-0.003	0.009	0.018	-0.058
71.H	-0.041	-0.014	0.015	-0.030	-0.023	-0.002	-0.008	0.032	-0.059
72.H	-0.049	-0.020	0.022	-0.034	-0.034	0.002	0.003	0.031	-0.069
73.H	-0.026	-0.015	0.010	-0.013	-0.028	-0.000	-0.017	0.030	-0.048
74.H	-0.039	0.011	-0.003	-0.024	-0.005	-0.009	-0.003	0.007	-0.031

75.H	-0.024	0.002	-0.003	-0.014	-0.008	-0.007	-0.021	0.020	-0.031
76.H	-0.028	0.034	-0.024	-0.008	0.013	-0.015	-0.006	-0.017	0.003
77.H	-0.036	0.029	-0.018	-0.018	0.012	-0.014	-0.004	-0.010	-0.009
78.H	-0.022	0.037	-0.029	-0.007	0.021	-0.018	-0.016	-0.014	0.009
79.H	-0.002	0.023	-0.026	0.013	0.007	-0.012	-0.030	-0.004	0.010
80.H	0.006	0.012	-0.021	0.016	0.001	-0.010	-0.043	0.011	0.002
81.H	0.002	0.027	-0.031	0.013	0.015	-0.015	-0.039	-0.001	0.015
82.H	-0.023	-0.010	0.006	-0.005	-0.028	0.000	-0.012	0.018	-0.039
83.H	-0.041	-0.019	0.019	-0.018	-0.042	0.005	0.009	0.018	-0.058
84.H	-0.038	0.017	-0.008	-0.016	-0.006	-0.008	0.003	-0.006	-0.021
85.H	-0.011	0.001	-0.007	0.002	-0.012	-0.005	-0.027	0.017	-0.020
86.H	-0.020	0.008	-0.008	0.013	-0.028	0.001	0.005	-0.016	-0.011
87.O	0.000	0.000	0.000	0.000	0.000	0.000	0.000	0.000	0.000
88.H	-0.021	-0.032	0.020	0.009	-0.061	0.013	-0.000	0.020	-0.052
89.H	-0.017	0.029	-0.025	0.011	-0.001	-0.009	-0.005	-0.024	0.010
90.H	-0.004	0.016	-0.020	0.019	-0.008	-0.006	-0.020	-0.008	0.005
91.H	-0.018	0.013	-0.013	0.009	-0.015	-0.004	-0.004	-0.012	-0.006
92.C	0.000	0.000	0.000	0.000	0.000	0.000	0.000	0.000	0.000
93.O	0.000	0.000	0.000	0.000	0.000	0.000	0.000	0.000	0.000
94.H	-0.052	-0.024	0.027	-0.016	-0.061	0.012	0.033	0.005	-0.065
95.H	-0.043	-0.038	0.034	-0.010	-0.072	0.016	0.021	0.021	-0.075
96.H	-0.040	-0.032	0.028	-0.004	-0.069	0.015	0.023	0.011	-0.064

	178			179			180		
1.O	0.000	0.000	0.000	0.000	0.000	0.000	0.000	0.000	0.000
2.C	0.000	0.000	0.000	0.000	0.000	0.000	0.000	0.000	0.000
3.C	0.000	0.000	0.000	0.000	0.000	0.000	0.000	0.000	0.000
4.C	0.000	0.000	0.000	0.000	0.000	0.000	0.000	0.000	0.000
5.O	0.000	0.000	0.000	0.000	0.000	0.000	0.000	0.000	0.000

6.N	0.000	0.000	0.000	0.000	0.000	0.000	0.000	0.000	0.000
7.O	0.000	0.000	0.000	0.000	0.000	0.000	0.000	0.000	0.000
8.C	0.000	0.000	0.000	0.000	0.000	0.000	0.000	0.000	0.000
9.C	0.000	0.000	0.000	0.000	0.000	0.000	0.000	0.000	0.000
10.C	0.000	0.000	0.000	0.000	0.000	0.000	0.000	0.000	0.000
11.O	0.000	0.000	0.000	0.000	0.000	0.000	0.000	0.000	0.000
12.C	0.000	0.000	0.000	0.000	0.000	0.000	0.000	0.000	0.000
13.O	0.000	0.000	0.000	0.000	0.000	0.000	0.000	0.000	0.000
14.C	0.000	0.000	0.000	0.000	0.000	0.000	0.000	0.000	0.000
15.C	0.000	0.000	0.000	0.000	0.000	0.000	0.000	0.000	0.000
16.O	0.000	0.000	0.000	0.000	0.000	0.000	0.000	0.000	0.000
17.C	0.000	0.000	0.000	0.000	0.000	0.000	0.000	0.000	0.000
18.C	0.000	0.000	0.000	0.000	0.000	0.000	0.000	0.000	0.000
19.O	0.000	0.000	0.000	0.000	0.000	0.000	0.000	0.000	0.000
20.C	0.000	0.000	0.000	0.000	0.000	0.000	0.000	0.000	0.000
21.C	0.000	0.000	0.000	0.000	0.000	0.000	0.000	0.000	0.000
22.C	0.000	0.000	0.000	0.000	0.000	0.000	0.000	0.000	0.000
23.C	0.000	0.000	0.000	0.000	0.000	0.000	0.000	0.000	0.000
24.C	0.000	0.000	0.000	0.000	0.000	0.000	0.000	0.000	0.000
25.C	0.000	0.000	0.000	0.000	0.000	0.000	0.000	0.000	0.000
26.C	0.000	0.000	0.000	0.000	0.000	0.000	0.000	0.000	0.000
27.C	0.000	0.000	0.000	0.000	0.000	0.000	0.000	0.000	0.000
28.C	0.000	0.000	0.000	0.000	0.000	0.000	0.000	0.000	0.000
29.C	0.000	0.000	0.000	0.000	0.000	0.000	0.000	0.000	0.000
30.C	0.000	0.000	0.000	0.000	0.000	0.000	0.000	0.000	0.000
31.O	0.000	0.000	0.000	0.000	0.000	0.000	0.000	0.000	0.000
32.C	0.000	0.000	0.000	0.000	0.000	0.000	0.000	0.000	0.000
33.C	0.000	0.000	0.000	0.000	0.000	0.000	0.000	0.000	0.000
34.C	0.000	0.000	0.000	0.000	0.000	0.000	0.000	0.000	0.000
35.C	0.000	0.000	0.000	0.000	0.000	0.000	0.000	0.000	0.000
36.C	0.000	0.000	0.000	0.000	0.000	0.000	0.000	0.000	0.000

37.C	0.000	0.000	0.000	0.000	0.000	0.000	0.000	0.000	0.000
38.C	0.000	0.000	0.000	0.000	0.000	0.000	0.000	0.000	0.000
39.C	0.000	0.000	0.000	0.000	0.000	0.000	0.000	0.000	0.000
40.H	0.000	0.000	0.000	0.000	0.000	0.000	0.000	0.000	0.000
41.H	0.000	0.000	0.000	0.000	0.000	0.000	0.000	0.000	0.000
42.H	0.000	0.000	0.000	0.000	0.000	0.000	0.000	0.000	0.000
43.H	0.000	0.000	0.000	0.000	0.000	0.000	0.000	0.000	0.000
44.H	0.000	0.000	0.000	0.000	0.000	0.000	0.000	0.000	0.000
45.H	0.000	0.000	0.000	0.000	0.000	0.000	0.000	0.000	0.000
46.H	0.000	0.000	0.000	0.000	0.000	0.000	0.000	0.000	0.000
47.H	0.000	0.000	0.000	0.000	0.000	0.000	0.000	0.000	0.000
48.H	0.000	0.000	0.000	0.000	0.000	0.000	0.000	0.000	0.000
49.H	0.000	0.000	0.000	0.000	0.000	0.000	0.000	0.000	0.000
50.H	0.000	0.000	0.000	0.000	0.000	0.000	0.000	0.000	0.000
51.H	0.000	0.000	0.000	0.000	0.000	0.000	0.000	0.000	0.000
52.H	0.000	0.000	0.000	0.000	0.000	0.000	0.000	0.000	0.000
53.H	0.000	0.000	0.000	0.000	0.000	0.000	0.000	0.000	0.000
54.H	0.000	0.000	0.000	0.000	0.000	0.000	0.000	0.000	0.000
55.H	0.000	0.000	0.000	0.000	0.000	0.000	0.000	0.000	0.000
56.H	0.963	0.005	0.013	0.000	0.963	0.013	0.000	0.000	0.978
57.H	-0.063	0.005	0.006	0.006	-0.065	0.030	0.011	0.012	-0.041
58.H	-0.050	0.003	0.007	0.005	-0.053	0.028	0.007	0.008	-0.041
59.H	-0.052	0.002	0.026	0.005	-0.056	0.022	0.008	0.010	-0.046
60.H	-0.065	0.004	0.025	0.006	-0.068	0.024	0.012	0.014	-0.046
61.H	-0.056	0.003	0.021	0.006	-0.060	0.034	0.009	0.011	-0.048
62.H	-0.068	0.007	-0.008	0.006	-0.069	0.026	0.013	0.013	-0.034
63.H	-0.075	0.008	-0.004	0.006	-0.075	0.015	0.016	0.015	-0.032
64.H	-0.068	0.008	-0.015	0.006	-0.067	0.017	0.013	0.012	-0.028
65.H	-0.036	0.001	0.008	0.001	-0.036	-0.002	0.003	0.003	-0.030
66.H	-0.014	-0.004	0.015	-0.001	-0.015	-0.001	-0.004	-0.003	-0.033
67.H	-0.018	-0.002	0.002	-0.001	-0.019	0.003	-0.003	-0.002	-0.029

68.H	-0.021	-0.002	0.011	0.000	-0.023	0.009	-0.002	-0.001	-0.035
69.C	0.000	0.000	0.000	0.000	0.000	0.000	0.000	0.000	0.000
70.H	-0.006	-0.004	0.002	-0.005	-0.003	-0.041	-0.006	-0.007	-0.013
71.H	0.006	-0.006	0.005	-0.006	0.009	-0.038	-0.010	-0.011	-0.015
72.H	-0.004	-0.005	0.014	-0.005	-0.001	-0.039	-0.007	-0.007	-0.018
73.H	0.002	-0.005	0.006	-0.005	0.003	-0.022	-0.009	-0.009	-0.021
74.H	-0.005	-0.002	-0.014	-0.005	-0.002	-0.027	-0.007	-0.008	-0.012
75.H	0.008	-0.005	-0.011	-0.006	0.009	-0.023	-0.011	-0.012	-0.015
76.H	-0.014	0.001	-0.033	-0.002	-0.011	-0.008	-0.004	-0.006	-0.012
77.H	-0.009	-0.000	-0.029	-0.004	-0.005	-0.020	-0.005	-0.008	-0.009
78.H	-0.003	-0.000	-0.039	-0.004	-0.000	-0.010	-0.007	-0.010	-0.009
79.H	-0.003	-0.001	-0.029	-0.002	-0.003	0.006	-0.008	-0.008	-0.019
80.H	0.008	-0.004	-0.023	-0.004	0.008	0.004	-0.012	-0.012	-0.020
81.H	0.006	-0.002	-0.036	-0.004	0.007	0.004	-0.011	-0.012	-0.015
82.H	-0.010	-0.003	0.004	-0.003	-0.010	-0.011	-0.005	-0.005	-0.025
83.H	-0.024	-0.002	0.018	-0.002	-0.023	-0.019	-0.001	-0.001	-0.027
84.H	-0.019	-0.000	-0.015	-0.002	-0.016	-0.015	-0.002	-0.004	-0.016
85.H	0.002	-0.004	-0.010	-0.004	0.003	-0.008	-0.010	-0.009	-0.021
86.H	-0.045	0.003	-0.002	0.003	-0.046	0.018	0.006	0.006	-0.033
87.O	0.000	0.000	0.000	0.000	0.000	0.000	0.000	0.000	0.000
88.H	-0.035	-0.002	0.030	0.002	-0.037	0.007	0.002	0.005	-0.041
89.H	-0.032	0.003	-0.024	0.001	-0.031	0.014	0.001	0.001	-0.023
90.H	-0.020	0.000	-0.018	0.000	-0.021	0.016	-0.003	-0.002	-0.026
91.H	-0.031	0.002	-0.011	0.001	-0.031	0.011	0.001	0.001	-0.027
92.C	0.000	0.000	0.000	0.000	0.000	0.000	0.000	0.000	0.000
93.O	0.000	0.000	0.000	0.000	0.000	0.000	0.000	0.000	0.000
94.H	-0.053	0.001	0.031	0.003	-0.053	-0.008	0.009	0.009	-0.036
95.H	-0.044	-0.001	0.041	0.002	-0.045	-0.008	0.006	0.007	-0.040
96.H	-0.052	0.000	0.037	0.003	-0.054	0.001	0.008	0.010	-0.042

	181			182			183		
	------------------------			------------------------			------------------------		
1.O	0.000	0.000	0.000	0.000	0.000	0.000	0.000	0.000	0.000
2.C	0.000	0.000	0.000	0.000	0.000	0.000	0.000	0.000	0.000
3.C	0.000	0.000	0.000	0.000	0.000	0.000	0.000	0.000	0.000
4.C	0.000	0.000	0.000	0.000	0.000	0.000	0.000	0.000	0.000
5.O	0.000	0.000	0.000	0.000	0.000	0.000	0.000	0.000	0.000
6.N	0.000	0.000	0.000	0.000	0.000	0.000	0.000	0.000	0.000
7.O	0.000	0.000	0.000	0.000	0.000	0.000	0.000	0.000	0.000
8.C	0.000	0.000	0.000	0.000	0.000	0.000	0.000	0.000	0.000
9.C	0.000	0.000	0.000	0.000	0.000	0.000	0.000	0.000	0.000
10.C	0.000	0.000	0.000	0.000	0.000	0.000	0.000	0.000	0.000
11.O	0.000	0.000	0.000	0.000	0.000	0.000	0.000	0.000	0.000
12.C	0.000	0.000	0.000	0.000	0.000	0.000	0.000	0.000	0.000
13.O	0.000	0.000	0.000	0.000	0.000	0.000	0.000	0.000	0.000
14.C	0.000	0.000	0.000	0.000	0.000	0.000	0.000	0.000	0.000
15.C	0.000	0.000	0.000	0.000	0.000	0.000	0.000	0.000	0.000
16.O	0.000	0.000	0.000	0.000	0.000	0.000	0.000	0.000	0.000
17.C	0.000	0.000	0.000	0.000	0.000	0.000	0.000	0.000	0.000
18.C	0.000	0.000	0.000	0.000	0.000	0.000	0.000	0.000	0.000
19.O	0.000	0.000	0.000	0.000	0.000	0.000	0.000	0.000	0.000
20.C	0.000	0.000	0.000	0.000	0.000	0.000	0.000	0.000	0.000
21.C	0.000	0.000	0.000	0.000	0.000	0.000	0.000	0.000	0.000
22.C	0.000	0.000	0.000	0.000	0.000	0.000	0.000	0.000	0.000
23.C	0.000	0.000	0.000	0.000	0.000	0.000	0.000	0.000	0.000
24.C	0.000	0.000	0.000	0.000	0.000	0.000	0.000	0.000	0.000
25.C	0.000	0.000	0.000	0.000	0.000	0.000	0.000	0.000	0.000
26.C	0.000	0.000	0.000	0.000	0.000	0.000	0.000	0.000	0.000
27.C	0.000	0.000	0.000	0.000	0.000	0.000	0.000	0.000	0.000
28.C	0.000	0.000	0.000	0.000	0.000	0.000	0.000	0.000	0.000
29.C	0.000	0.000	0.000	0.000	0.000	0.000	0.000	0.000	0.000

30.C	0.000	0.000	0.000	0.000	0.000	0.000	0.000	0.000	0.000
31.O	0.000	0.000	0.000	0.000	0.000	0.000	0.000	0.000	0.000
32.C	0.000	0.000	0.000	0.000	0.000	0.000	0.000	0.000	0.000
33.C	0.000	0.000	0.000	0.000	0.000	0.000	0.000	0.000	0.000
34.C	0.000	0.000	0.000	0.000	0.000	0.000	0.000	0.000	0.000
35.C	0.000	0.000	0.000	0.000	0.000	0.000	0.000	0.000	0.000
36.C	0.000	0.000	0.000	0.000	0.000	0.000	0.000	0.000	0.000
37.C	0.000	0.000	0.000	0.000	0.000	0.000	0.000	0.000	0.000
38.C	0.000	0.000	0.000	0.000	0.000	0.000	0.000	0.000	0.000
39.C	0.000	0.000	0.000	0.000	0.000	0.000	0.000	0.000	0.000
40.H	0.000	0.000	0.000	0.000	0.000	0.000	0.000	0.000	0.000
41.H	0.000	0.000	0.000	0.000	0.000	0.000	0.000	0.000	0.000
42.H	0.000	0.000	0.000	0.000	0.000	0.000	0.000	0.000	0.000
43.H	0.000	0.000	0.000	0.000	0.000	0.000	0.000	0.000	0.000
44.H	0.000	0.000	0.000	0.000	0.000	0.000	0.000	0.000	0.000
45.H	0.000	0.000	0.000	0.000	0.000	0.000	0.000	0.000	0.000
46.H	0.000	0.000	0.000	0.000	0.000	0.000	0.000	0.000	0.000
47.H	0.000	0.000	0.000	0.000	0.000	0.000	0.000	0.000	0.000
48.H	0.000	0.000	0.000	0.000	0.000	0.000	0.000	0.000	0.000
49.H	0.000	0.000	0.000	0.000	0.000	0.000	0.000	0.000	0.000
50.H	0.000	0.000	0.000	0.000	0.000	0.000	0.000	0.000	0.000
51.H	0.000	0.000	0.000	0.000	0.000	0.000	0.000	0.000	0.000
52.H	0.000	0.000	0.000	0.000	0.000	0.000	0.000	0.000	0.000
53.H	0.000	0.000	0.000	0.000	0.000	0.000	0.000	0.000	0.000
54.H	0.000	0.000	0.000	0.000	0.000	0.000	0.000	0.000	0.000
55.H	0.000	0.000	0.000	0.000	0.000	0.000	0.000	0.000	0.000
56.H	0.000	0.000	0.000	0.000	0.000	0.000	0.000	0.000	0.000
57.H	0.958	0.004	0.011	0.000	0.963	0.031	0.000	0.000	0.967
58.H	-0.069	0.006	0.012	0.002	-0.054	0.029	0.011	0.021	-0.057
59.H	-0.067	0.020	0.023	0.003	-0.055	0.022	0.010	0.018	-0.055
60.H	-0.077	0.017	0.023	0.005	-0.068	0.024	0.011	0.030	-0.056

61.H	-0.079	0.017	0.023	0.003	-0.060	0.034	0.013	0.023	-0.065
62.H	-0.077	-0.007	0.001	0.005	-0.073	0.027	0.011	0.042	-0.053
63.H	-0.073	-0.006	0.001	0.007	-0.079	0.016	0.008	0.047	-0.043
64.H	-0.069	-0.014	-0.006	0.006	-0.072	0.019	0.008	0.043	-0.043
65.H	-0.034	0.005	0.005	0.002	-0.037	-0.002	0.001	0.007	-0.029
66.H	-0.022	0.013	0.010	-0.002	-0.015	-0.001	-0.000	-0.015	-0.031
67.H	-0.026	0.003	0.003	-0.001	-0.020	0.003	0.001	-0.007	-0.032
68.H	-0.034	0.010	0.010	-0.001	-0.023	0.009	0.003	-0.007	-0.040
69.C	0.000	0.000	0.000	0.000	0.000	0.000	0.000	0.000	0.000
70.H	0.019	-0.001	-0.008	-0.001	-0.005	-0.042	-0.013	-0.020	0.011
71.H	0.025	0.004	-0.005	-0.003	0.008	-0.039	-0.013	-0.032	0.008
72.H	0.018	0.009	0.000	-0.002	-0.001	-0.041	-0.013	-0.026	0.007
73.H	0.008	0.005	-0.001	-0.003	0.002	-0.023	-0.008	-0.028	-0.008
74.H	0.010	-0.011	-0.015	-0.002	-0.005	-0.027	-0.010	-0.016	-0.000
75.H	0.014	-0.007	-0.013	-0.004	0.007	-0.023	-0.009	-0.028	-0.005
76.H	-0.011	-0.024	-0.024	-0.001	-0.017	-0.007	-0.004	-0.002	-0.015
77.H	0.002	-0.022	-0.024	-0.002	-0.011	-0.018	-0.008	-0.008	-0.005
78.H	-0.002	-0.028	-0.029	-0.003	-0.007	-0.008	-0.005	-0.010	-0.013
79.H	-0.017	-0.018	-0.018	-0.004	-0.008	0.008	0.000	-0.012	-0.030
80.H	-0.008	-0.013	-0.014	-0.006	0.005	0.006	-0.001	-0.024	-0.029
81.H	-0.007	-0.023	-0.023	-0.005	0.002	0.006	-0.002	-0.019	-0.026
82.H	-0.009	0.004	0.001	-0.002	-0.011	-0.011	-0.004	-0.016	-0.019
83.H	-0.013	0.012	0.008	0.001	-0.023	-0.020	-0.005	-0.008	-0.014
84.H	-0.010	-0.012	-0.013	-0.000	-0.020	-0.015	-0.005	-0.003	-0.012
85.H	-0.003	-0.005	-0.008	-0.004	0.000	-0.007	-0.004	-0.023	-0.020
86.H	-0.056	-0.001	0.003	0.002	-0.048	0.019	0.007	0.019	-0.046
87.O	0.000	0.000	0.000	0.000	0.000	0.000	0.000	0.000	0.000
88.H	-0.043	0.023	0.022	0.001	-0.036	0.006	0.004	0.000	-0.040
89.H	-0.042	-0.017	-0.013	0.000	-0.036	0.016	0.004	0.013	-0.038
90.H	-0.037	-0.011	-0.008	-0.002	-0.024	0.017	0.004	0.001	-0.041
91.H	-0.040	-0.007	-0.005	0.000	-0.034	0.012	0.004	0.008	-0.037

92.C	0.000	0.000	0.000	0.000	0.000	0.000	0.000	0.000	0.000
93.O	0.000	0.000	0.000	0.000	0.000	0.000	0.000	0.000	0.000
94.H	-0.042	0.020	0.019	0.005	-0.052	-0.010	0.000	0.015	-0.026
95.H	-0.037	0.028	0.026	0.003	-0.043	-0.010	0.000	0.004	-0.028
96.H	-0.050	0.026	0.026	0.004	-0.052	-0.000	0.004	0.013	-0.036

	184			185			186		
	------------------------			------------------------			------------------------		
1.O	0.000	0.000	0.000	0.000	0.000	0.000	0.000	0.000	0.000
2.C	0.000	0.000	0.000	0.000	0.000	0.000	0.000	0.000	0.000
3.C	0.000	0.000	0.000	0.000	0.000	0.000	0.000	0.000	0.000
4.C	0.000	0.000	0.000	0.000	0.000	0.000	0.000	0.000	0.000
5.O	0.000	0.000	0.000	0.000	0.000	0.000	0.000	0.000	0.000
6.N	0.000	0.000	0.000	0.000	0.000	0.000	0.000	0.000	0.000
7.O	0.000	0.000	0.000	0.000	0.000	0.000	0.000	0.000	0.000
8.C	0.000	0.000	0.000	0.000	0.000	0.000	0.000	0.000	0.000
9.C	0.000	0.000	0.000	0.000	0.000	0.000	0.000	0.000	0.000
10.C	0.000	0.000	0.000	0.000	0.000	0.000	0.000	0.000	0.000
11.O	0.000	0.000	0.000	0.000	0.000	0.000	0.000	0.000	0.000
12.C	0.000	0.000	0.000	0.000	0.000	0.000	0.000	0.000	0.000
13.O	0.000	0.000	0.000	0.000	0.000	0.000	0.000	0.000	0.000
14.C	0.000	0.000	0.000	0.000	0.000	0.000	0.000	0.000	0.000
15.C	0.000	0.000	0.000	0.000	0.000	0.000	0.000	0.000	0.000
16.O	0.000	0.000	0.000	0.000	0.000	0.000	0.000	0.000	0.000
17.C	0.000	0.000	0.000	0.000	0.000	0.000	0.000	0.000	0.000
18.C	0.000	0.000	0.000	0.000	0.000	0.000	0.000	0.000	0.000
19.O	0.000	0.000	0.000	0.000	0.000	0.000	0.000	0.000	0.000
20.C	0.000	0.000	0.000	0.000	0.000	0.000	0.000	0.000	0.000
21.C	0.000	0.000	0.000	0.000	0.000	0.000	0.000	0.000	0.000
22.C	0.000	0.000	0.000	0.000	0.000	0.000	0.000	0.000	0.000

23.C	0.000	0.000	0.000	0.000	0.000	0.000	0.000	0.000	0.000
24.C	0.000	0.000	0.000	0.000	0.000	0.000	0.000	0.000	0.000
25.C	0.000	0.000	0.000	0.000	0.000	0.000	0.000	0.000	0.000
26.C	0.000	0.000	0.000	0.000	0.000	0.000	0.000	0.000	0.000
27.C	0.000	0.000	0.000	0.000	0.000	0.000	0.000	0.000	0.000
28.C	0.000	0.000	0.000	0.000	0.000	0.000	0.000	0.000	0.000
29.C	0.000	0.000	0.000	0.000	0.000	0.000	0.000	0.000	0.000
30.C	0.000	0.000	0.000	0.000	0.000	0.000	0.000	0.000	0.000
31.O	0.000	0.000	0.000	0.000	0.000	0.000	0.000	0.000	0.000
32.C	0.000	0.000	0.000	0.000	0.000	0.000	0.000	0.000	0.000
33.C	0.000	0.000	0.000	0.000	0.000	0.000	0.000	0.000	0.000
34.C	0.000	0.000	0.000	0.000	0.000	0.000	0.000	0.000	0.000
35.C	0.000	0.000	0.000	0.000	0.000	0.000	0.000	0.000	0.000
36.C	0.000	0.000	0.000	0.000	0.000	0.000	0.000	0.000	0.000
37.C	0.000	0.000	0.000	0.000	0.000	0.000	0.000	0.000	0.000
38.C	0.000	0.000	0.000	0.000	0.000	0.000	0.000	0.000	0.000
39.C	0.000	0.000	0.000	0.000	0.000	0.000	0.000	0.000	0.000
40.H	0.000	0.000	0.000	0.000	0.000	0.000	0.000	0.000	0.000
41.H	0.000	0.000	0.000	0.000	0.000	0.000	0.000	0.000	0.000
42.H	0.000	0.000	0.000	0.000	0.000	0.000	0.000	0.000	0.000
43.H	0.000	0.000	0.000	0.000	0.000	0.000	0.000	0.000	0.000
44.H	0.000	0.000	0.000	0.000	0.000	0.000	0.000	0.000	0.000
45.H	0.000	0.000	0.000	0.000	0.000	0.000	0.000	0.000	0.000
46.H	0.000	0.000	0.000	0.000	0.000	0.000	0.000	0.000	0.000
47.H	0.000	0.000	0.000	0.000	0.000	0.000	0.000	0.000	0.000
48.H	0.000	0.000	0.000	0.000	0.000	0.000	0.000	0.000	0.000
49.H	0.000	0.000	0.000	0.000	0.000	0.000	0.000	0.000	0.000
50.H	0.000	0.000	0.000	0.000	0.000	0.000	0.000	0.000	0.000
51.H	0.000	0.000	0.000	0.000	0.000	0.000	0.000	0.000	0.000
52.H	0.000	0.000	0.000	0.000	0.000	0.000	0.000	0.000	0.000
53.H	0.000	0.000	0.000	0.000	0.000	0.000	0.000	0.000	0.000

54.H	0.000	0.000	0.000	0.000	0.000	0.000	0.000	0.000	0.000
55.H	0.000	0.000	0.000	0.000	0.000	0.000	0.000	0.000	0.000
56.H	0.000	0.000	0.000	0.000	0.000	0.000	0.000	0.000	0.000
57.H	0.000	0.000	0.000	0.000	0.000	0.000	0.000	0.000	0.000
58.H	0.962	0.006	0.011	0.000	0.972	0.021	0.000	0.000	0.966
59.H	-0.065	0.023	0.016	0.006	-0.054	0.018	0.011	0.019	-0.059
60.H	-0.071	0.019	0.016	0.007	-0.062	0.019	0.013	0.031	-0.060
61.H	-0.078	0.019	0.018	0.008	-0.057	0.026	0.015	0.025	-0.069
62.H	-0.070	-0.013	0.006	0.006	-0.058	0.019	0.013	0.042	-0.055
63.H	-0.061	-0.012	0.004	0.004	-0.063	0.011	0.011	0.048	-0.044
64.H	-0.060	-0.021	0.001	0.005	-0.056	0.012	0.010	0.044	-0.044
65.H	-0.033	0.006	0.003	0.001	-0.037	0.000	0.002	0.008	-0.031
66.H	-0.028	0.018	0.005	0.000	-0.024	0.001	-0.000	-0.014	-0.033
67.H	-0.032	0.005	0.002	0.001	-0.025	0.003	0.001	-0.007	-0.034
68.H	-0.040	0.014	0.007	0.002	-0.029	0.008	0.003	-0.006	-0.042
69.C	0.000	0.000	0.000	0.000	0.000	0.000	0.000	0.000	0.000
70.H	0.018	0.001	-0.011	-0.008	-0.013	-0.028	-0.014	-0.019	0.010
71.H	0.019	0.008	-0.009	-0.008	-0.006	-0.026	-0.014	-0.032	0.006
72.H	0.016	0.014	-0.006	-0.007	-0.014	-0.026	-0.013	-0.025	0.005
73.H	0.000	0.010	-0.004	-0.004	-0.010	-0.014	-0.009	-0.027	-0.010
74.H	0.006	-0.012	-0.012	-0.006	-0.010	-0.019	-0.010	-0.016	-0.001
75.H	0.005	-0.006	-0.010	-0.005	-0.003	-0.016	-0.010	-0.028	-0.005
76.H	-0.015	-0.030	-0.013	-0.002	-0.015	-0.007	-0.004	-0.002	-0.015
77.H	-0.002	-0.027	-0.015	-0.004	-0.011	-0.014	-0.008	-0.008	-0.005
78.H	-0.009	-0.034	-0.016	-0.003	-0.006	-0.008	-0.006	-0.010	-0.012
79.H	-0.027	-0.022	-0.008	0.000	-0.010	0.004	-0.001	-0.012	-0.030
80.H	-0.022	-0.014	-0.006	-0.000	-0.003	0.003	-0.003	-0.024	-0.029
81.H	-0.020	-0.026	-0.011	-0.001	-0.002	0.002	-0.003	-0.019	-0.026
82.H	-0.015	0.007	-0.001	-0.002	-0.019	-0.007	-0.004	-0.015	-0.021
83.H	-0.013	0.016	0.001	-0.003	-0.029	-0.011	-0.004	-0.007	-0.016
84.H	-0.011	-0.014	-0.009	-0.003	-0.021	-0.010	-0.005	-0.003	-0.012

85.H	-0.013	-0.004	-0.005	-0.002	-0.008	-0.005	-0.005	-0.023	-0.021
86.H	-0.055	-0.003	0.005	0.004	-0.043	0.013	0.008	0.019	-0.048
87.O	0.000	0.000	0.000	0.000	0.000	0.000	0.000	0.000	0.000
88.H	-0.044	0.028	0.013	0.003	-0.042	0.007	0.005	0.002	-0.044
89.H	-0.044	-0.023	-0.004	0.003	-0.030	0.009	0.005	0.013	-0.039
90.H	-0.043	-0.014	-0.001	0.003	-0.024	0.011	0.004	0.001	-0.042
91.H	-0.042	-0.010	-0.000	0.002	-0.032	0.008	0.004	0.009	-0.039
92.C	0.000	0.000	0.000	0.000	0.000	0.000	0.000	0.000	0.000
93.O	0.000	0.000	0.000	0.000	0.000	0.000	0.000	0.000	0.000
94.H	-0.034	0.024	0.009	0.000	-0.052	-0.003	0.002	0.017	-0.029
95.H	-0.033	0.035	0.012	0.000	-0.048	-0.003	0.002	0.006	-0.032
96.H	-0.044	0.031	0.014	0.002	-0.054	0.004	0.005	0.015	-0.040

	187			188			189		
	------------------------			------------------------			------------------------		
1.O	0.000	0.000	0.000	0.000	0.000	0.000	0.000	0.000	0.000
2.C	0.000	0.000	0.000	0.000	0.000	0.000	0.000	0.000	0.000
3.C	0.000	0.000	0.000	0.000	0.000	0.000	0.000	0.000	0.000
4.C	0.000	0.000	0.000	0.000	0.000	0.000	0.000	0.000	0.000
5.O	0.000	0.000	0.000	0.000	0.000	0.000	0.000	0.000	0.000
6.N	0.000	0.000	0.000	0.000	0.000	0.000	0.000	0.000	0.000
7.O	0.000	0.000	0.000	0.000	0.000	0.000	0.000	0.000	0.000
8.C	0.000	0.000	0.000	0.000	0.000	0.000	0.000	0.000	0.000
9.C	0.000	0.000	0.000	0.000	0.000	0.000	0.000	0.000	0.000
10.C	0.000	0.000	0.000	0.000	0.000	0.000	0.000	0.000	0.000
11.O	0.000	0.000	0.000	0.000	0.000	0.000	0.000	0.000	0.000
12.C	0.000	0.000	0.000	0.000	0.000	0.000	0.000	0.000	0.000
13.O	0.000	0.000	0.000	0.000	0.000	0.000	0.000	0.000	0.000
14.C	0.000	0.000	0.000	0.000	0.000	0.000	0.000	0.000	0.000
15.C	0.000	0.000	0.000	0.000	0.000	0.000	0.000	0.000	0.000

16.O	0.000	0.000	0.000	0.000	0.000	0.000	0.000	0.000	0.000
17.C	0.000	0.000	0.000	0.000	0.000	0.000	0.000	0.000	0.000
18.C	0.000	0.000	0.000	0.000	0.000	0.000	0.000	0.000	0.000
19.O	0.000	0.000	0.000	0.000	0.000	0.000	0.000	0.000	0.000
20.C	0.000	0.000	0.000	0.000	0.000	0.000	0.000	0.000	0.000
21.C	0.000	0.000	0.000	0.000	0.000	0.000	0.000	0.000	0.000
22.C	0.000	0.000	0.000	0.000	0.000	0.000	0.000	0.000	0.000
23.C	0.000	0.000	0.000	0.000	0.000	0.000	0.000	0.000	0.000
24.C	0.000	0.000	0.000	0.000	0.000	0.000	0.000	0.000	0.000
25.C	0.000	0.000	0.000	0.000	0.000	0.000	0.000	0.000	0.000
26.C	0.000	0.000	0.000	0.000	0.000	0.000	0.000	0.000	0.000
27.C	0.000	0.000	0.000	0.000	0.000	0.000	0.000	0.000	0.000
28.C	0.000	0.000	0.000	0.000	0.000	0.000	0.000	0.000	0.000
29.C	0.000	0.000	0.000	0.000	0.000	0.000	0.000	0.000	0.000
30.C	0.000	0.000	0.000	0.000	0.000	0.000	0.000	0.000	0.000
31.O	0.000	0.000	0.000	0.000	0.000	0.000	0.000	0.000	0.000
32.C	0.000	0.000	0.000	0.000	0.000	0.000	0.000	0.000	0.000
33.C	0.000	0.000	0.000	0.000	0.000	0.000	0.000	0.000	0.000
34.C	0.000	0.000	0.000	0.000	0.000	0.000	0.000	0.000	0.000
35.C	0.000	0.000	0.000	0.000	0.000	0.000	0.000	0.000	0.000
36.C	0.000	0.000	0.000	0.000	0.000	0.000	0.000	0.000	0.000
37.C	0.000	0.000	0.000	0.000	0.000	0.000	0.000	0.000	0.000
38.C	0.000	0.000	0.000	0.000	0.000	0.000	0.000	0.000	0.000
39.C	0.000	0.000	0.000	0.000	0.000	0.000	0.000	0.000	0.000
40.H	0.000	0.000	0.000	0.000	0.000	0.000	0.000	0.000	0.000
41.H	0.000	0.000	0.000	0.000	0.000	0.000	0.000	0.000	0.000
42.H	0.000	0.000	0.000	0.000	0.000	0.000	0.000	0.000	0.000
43.H	0.000	0.000	0.000	0.000	0.000	0.000	0.000	0.000	0.000
44.H	0.000	0.000	0.000	0.000	0.000	0.000	0.000	0.000	0.000
45.H	0.000	0.000	0.000	0.000	0.000	0.000	0.000	0.000	0.000
46.H	0.000	0.000	0.000	0.000	0.000	0.000	0.000	0.000	0.000

47.H	0.000	0.000	0.000	0.000	0.000	0.000	0.000	0.000	0.000
48.H	0.000	0.000	0.000	0.000	0.000	0.000	0.000	0.000	0.000
49.H	0.000	0.000	0.000	0.000	0.000	0.000	0.000	0.000	0.000
50.H	0.000	0.000	0.000	0.000	0.000	0.000	0.000	0.000	0.000
51.H	0.000	0.000	0.000	0.000	0.000	0.000	0.000	0.000	0.000
52.H	0.000	0.000	0.000	0.000	0.000	0.000	0.000	0.000	0.000
53.H	0.000	0.000	0.000	0.000	0.000	0.000	0.000	0.000	0.000
54.H	0.000	0.000	0.000	0.000	0.000	0.000	0.000	0.000	0.000
55.H	0.000	0.000	0.000	0.000	0.000	0.000	0.000	0.000	0.000
56.H	0.000	0.000	0.000	0.000	0.000	0.000	0.000	0.000	0.000
57.H	0.000	0.000	0.000	0.000	0.000	0.000	0.000	0.000	0.000
58.H	0.000	0.000	0.000	0.000	0.000	0.000	0.000	0.000	0.000
59.H	0.963	0.020	0.020	0.000	0.958	0.023	0.000	0.000	0.959
60.H	-0.075	0.017	0.020	0.020	-0.083	0.023	0.031	0.034	-0.075
61.H	-0.080	0.017	0.021	0.033	-0.077	0.028	0.024	0.028	-0.081
62.H	-0.075	-0.010	0.004	0.018	-0.051	0.014	0.033	0.032	-0.048
63.H	-0.069	-0.009	0.003	0.004	-0.057	0.009	0.040	0.037	-0.042
64.H	-0.067	-0.017	-0.002	0.008	-0.042	0.007	0.034	0.030	-0.035
65.H	-0.037	0.006	0.005	-0.001	-0.045	0.003	0.009	0.009	-0.039
66.H	-0.028	0.015	0.008	0.007	-0.041	0.006	-0.008	-0.005	-0.045
67.H	-0.032	0.004	0.003	0.008	-0.031	0.004	-0.005	-0.003	-0.037
68.H	-0.040	0.012	0.009	0.015	-0.043	0.010	-0.003	0.001	-0.051
69.C	0.000	0.000	0.000	0.000	0.000	0.000	0.000	0.000	0.000
70.H	0.014	0.001	-0.009	-0.035	-0.015	-0.022	-0.012	-0.015	0.001
71.H	0.017	0.006	-0.007	-0.028	-0.013	-0.019	-0.022	-0.023	-0.004
72.H	0.012	0.011	-0.002	-0.031	-0.027	-0.017	-0.015	-0.015	-0.011
73.H	-0.000	0.008	-0.002	-0.012	-0.019	-0.009	-0.020	-0.019	-0.019
74.H	0.003	-0.010	-0.013	-0.022	-0.001	-0.019	-0.014	-0.017	0.003
75.H	0.005	-0.005	-0.011	-0.014	0.001	-0.016	-0.024	-0.025	-0.003
76.H	-0.016	-0.025	-0.018	-0.007	0.010	-0.014	-0.008	-0.011	0.003
77.H	-0.004	-0.023	-0.019	-0.017	0.011	-0.020	-0.011	-0.015	0.010

78.H	-0.009	-0.029	-0.022	-0.006	0.022	-0.017	-0.016	-0.020	0.010
79.H	-0.025	-0.019	-0.012	0.012	0.008	-0.005	-0.017	-0.017	-0.013
80.H	-0.018	-0.012	-0.010	0.014	0.009	-0.004	-0.026	-0.024	-0.016
81.H	-0.017	-0.023	-0.016	0.011	0.021	-0.008	-0.024	-0.025	-0.004
82.H	-0.016	0.006	0.000	-0.004	-0.026	-0.004	-0.011	-0.010	-0.027
83.H	-0.016	0.013	0.005	-0.014	-0.045	-0.004	0.000	0.001	-0.032
84.H	-0.014	-0.012	-0.011	-0.013	-0.010	-0.012	-0.004	-0.006	-0.007
85.H	-0.011	-0.004	-0.006	0.001	-0.005	-0.006	-0.021	-0.020	-0.017
86.H	-0.058	-0.002	0.005	0.016	-0.044	0.011	0.015	0.016	-0.046
87.O	0.000	0.000	0.000	0.000	0.000	0.000	0.000	0.000	0.000
88.H	-0.046	0.024	0.018	0.011	-0.069	0.015	0.008	0.012	-0.065
89.H	-0.046	-0.019	-0.008	0.013	-0.013	0.001	0.005	0.004	-0.023
90.H	-0.042	-0.011	-0.004	0.019	-0.014	0.005	-0.004	-0.003	-0.030
91.H	-0.044	-0.008	-0.002	0.011	-0.026	0.004	0.005	0.005	-0.032
92.C	0.000	0.000	0.000	0.000	0.000	0.000	0.000	0.000	0.000
93.O	0.000	0.000	0.000	0.000	0.000	0.000	0.000	0.000	0.000
94.H	-0.041	0.021	0.014	-0.010	-0.076	0.007	0.023	0.023	-0.053
95.H	-0.038	0.030	0.019	-0.006	-0.082	0.010	0.015	0.018	-0.062
96.H	-0.050	0.027	0.020	0.001	-0.084	0.014	0.021	0.024	-0.067

	190			191			192		
1.O	0.000	0.000	0.000	0.000	0.000	0.000	0.000	0.000	0.000
2.C	0.000	0.000	0.000	0.000	0.000	0.000	0.000	0.000	0.000
3.C	0.000	0.000	0.000	0.000	0.000	0.000	0.000	0.000	0.000
4.C	0.000	0.000	0.000	0.000	0.000	0.000	0.000	0.000	0.000
5.O	0.000	0.000	0.000	0.000	0.000	0.000	0.000	0.000	0.000
6.N	0.000	0.000	0.000	0.000	0.000	0.000	0.000	0.000	0.000
7.O	0.000	0.000	0.000	0.000	0.000	0.000	0.000	0.000	0.000
8.C	0.000	0.000	0.000	0.000	0.000	0.000	0.000	0.000	0.000

9.C	0.000	0.000	0.000	0.000	0.000	0.000	0.000	0.000	0.000
10.C	0.000	0.000	0.000	0.000	0.000	0.000	0.000	0.000	0.000
11.O	0.000	0.000	0.000	0.000	0.000	0.000	0.000	0.000	0.000
12.C	0.000	0.000	0.000	0.000	0.000	0.000	0.000	0.000	0.000
13.O	0.000	0.000	0.000	0.000	0.000	0.000	0.000	0.000	0.000
14.C	0.000	0.000	0.000	0.000	0.000	0.000	0.000	0.000	0.000
15.C	0.000	0.000	0.000	0.000	0.000	0.000	0.000	0.000	0.000
16.O	0.000	0.000	0.000	0.000	0.000	0.000	0.000	0.000	0.000
17.C	0.000	0.000	0.000	0.000	0.000	0.000	0.000	0.000	0.000
18.C	0.000	0.000	0.000	0.000	0.000	0.000	0.000	0.000	0.000
19.O	0.000	0.000	0.000	0.000	0.000	0.000	0.000	0.000	0.000
20.C	0.000	0.000	0.000	0.000	0.000	0.000	0.000	0.000	0.000
21.C	0.000	0.000	0.000	0.000	0.000	0.000	0.000	0.000	0.000
22.C	0.000	0.000	0.000	0.000	0.000	0.000	0.000	0.000	0.000
23.C	0.000	0.000	0.000	0.000	0.000	0.000	0.000	0.000	0.000
24.C	0.000	0.000	0.000	0.000	0.000	0.000	0.000	0.000	0.000
25.C	0.000	0.000	0.000	0.000	0.000	0.000	0.000	0.000	0.000
26.C	0.000	0.000	0.000	0.000	0.000	0.000	0.000	0.000	0.000
27.C	0.000	0.000	0.000	0.000	0.000	0.000	0.000	0.000	0.000
28.C	0.000	0.000	0.000	0.000	0.000	0.000	0.000	0.000	0.000
29.C	0.000	0.000	0.000	0.000	0.000	0.000	0.000	0.000	0.000
30.C	0.000	0.000	0.000	0.000	0.000	0.000	0.000	0.000	0.000
31.O	0.000	0.000	0.000	0.000	0.000	0.000	0.000	0.000	0.000
32.C	0.000	0.000	0.000	0.000	0.000	0.000	0.000	0.000	0.000
33.C	0.000	0.000	0.000	0.000	0.000	0.000	0.000	0.000	0.000
34.C	0.000	0.000	0.000	0.000	0.000	0.000	0.000	0.000	0.000
35.C	0.000	0.000	0.000	0.000	0.000	0.000	0.000	0.000	0.000
36.C	0.000	0.000	0.000	0.000	0.000	0.000	0.000	0.000	0.000
37.C	0.000	0.000	0.000	0.000	0.000	0.000	0.000	0.000	0.000
38.C	0.000	0.000	0.000	0.000	0.000	0.000	0.000	0.000	0.000
39.C	0.000	0.000	0.000	0.000	0.000	0.000	0.000	0.000	0.000

40.H	0.000	0.000	0.000	0.000	0.000	0.000	0.000	0.000	0.000
41.H	0.000	0.000	0.000	0.000	0.000	0.000	0.000	0.000	0.000
42.H	0.000	0.000	0.000	0.000	0.000	0.000	0.000	0.000	0.000
43.H	0.000	0.000	0.000	0.000	0.000	0.000	0.000	0.000	0.000
44.H	0.000	0.000	0.000	0.000	0.000	0.000	0.000	0.000	0.000
45.H	0.000	0.000	0.000	0.000	0.000	0.000	0.000	0.000	0.000
46.H	0.000	0.000	0.000	0.000	0.000	0.000	0.000	0.000	0.000
47.H	0.000	0.000	0.000	0.000	0.000	0.000	0.000	0.000	0.000
48.H	0.000	0.000	0.000	0.000	0.000	0.000	0.000	0.000	0.000
49.H	0.000	0.000	0.000	0.000	0.000	0.000	0.000	0.000	0.000
50.H	0.000	0.000	0.000	0.000	0.000	0.000	0.000	0.000	0.000
51.H	0.000	0.000	0.000	0.000	0.000	0.000	0.000	0.000	0.000
52.H	0.000	0.000	0.000	0.000	0.000	0.000	0.000	0.000	0.000
53.H	0.000	0.000	0.000	0.000	0.000	0.000	0.000	0.000	0.000
54.H	0.000	0.000	0.000	0.000	0.000	0.000	0.000	0.000	0.000
55.H	0.000	0.000	0.000	0.000	0.000	0.000	0.000	0.000	0.000
56.H	0.000	0.000	0.000	0.000	0.000	0.000	0.000	0.000	0.000
57.H	0.000	0.000	0.000	0.000	0.000	0.000	0.000	0.000	0.000
58.H	0.000	0.000	0.000	0.000	0.000	0.000	0.000	0.000	0.000
59.H	0.000	0.000	0.000	0.000	0.000	0.000	0.000	0.000	0.000
60.H	0.949	0.019	0.033	0.000	0.945	0.038	0.000	0.000	0.955
61.H	-0.097	0.018	0.032	0.031	-0.095	0.048	0.028	0.034	-0.092
62.H	-0.098	-0.006	0.002	0.019	-0.079	0.031	0.037	0.041	-0.056
63.H	-0.095	-0.004	0.003	0.008	-0.088	0.019	0.044	0.046	-0.048
64.H	-0.090	-0.012	-0.008	0.011	-0.071	0.019	0.038	0.039	-0.040
65.H	-0.045	0.006	0.010	0.001	-0.053	0.004	0.011	0.012	-0.042
66.H	-0.025	0.014	0.017	0.006	-0.036	0.007	-0.007	-0.006	-0.049
67.H	-0.031	0.004	0.005	0.007	-0.030	0.008	-0.004	-0.003	-0.041
68.H	-0.041	0.011	0.016	0.013	-0.043	0.017	-0.001	0.001	-0.056
69.C	0.000	0.000	0.000	0.000	0.000	0.000	0.000	0.000	0.000
70.H	0.018	-0.000	-0.007	-0.031	-0.007	-0.043	-0.013	-0.017	0.005

71.H	0.027	0.004	-0.003	-0.026	0.003	-0.038	-0.024	-0.027	-0.001
72.H	0.018	0.009	0.005	-0.028	-0.015	-0.037	-0.016	-0.018	-0.008
73.H	0.009	0.006	0.002	-0.012	-0.006	-0.020	-0.020	-0.022	-0.018
74.H	0.009	-0.010	-0.018	-0.019	0.006	-0.031	-0.015	-0.019	0.005
75.H	0.017	-0.006	-0.015	-0.014	0.015	-0.026	-0.025	-0.029	-0.001
76.H	-0.014	-0.023	-0.032	-0.006	0.008	-0.015	-0.007	-0.011	0.004
77.H	0.000	-0.021	-0.031	-0.015	0.013	-0.027	-0.012	-0.016	0.012
78.H	-0.002	-0.027	-0.039	-0.006	0.025	-0.019	-0.016	-0.021	0.012
79.H	-0.017	-0.017	-0.025	0.009	0.012	0.003	-0.016	-0.018	-0.015
80.H	-0.005	-0.012	-0.020	0.010	0.020	0.002	-0.026	-0.028	-0.018
81.H	-0.005	-0.022	-0.032	0.008	0.029	-0.002	-0.025	-0.027	-0.005
82.H	-0.012	0.005	0.004	-0.004	-0.020	-0.008	-0.011	-0.011	-0.028
83.H	-0.019	0.013	0.015	-0.012	-0.044	-0.013	0.001	0.001	-0.033
84.H	-0.014	-0.011	-0.016	-0.011	-0.011	-0.018	-0.003	-0.006	-0.006
85.H	-0.002	-0.005	-0.010	-0.000	0.005	-0.008	-0.021	-0.022	-0.018
86.H	-0.069	0.000	0.005	0.016	-0.058	0.023	0.018	0.021	-0.052
87.O	0.000	0.000	0.000	0.000	0.000	0.000	0.000	0.000	0.000
88.H	-0.053	0.023	0.033	0.011	-0.073	0.020	0.010	0.014	-0.071
89.H	-0.051	-0.016	-0.018	0.012	-0.024	0.013	0.007	0.007	-0.027
90.H	-0.042	-0.010	-0.011	0.016	-0.017	0.016	-0.003	-0.002	-0.035
91.H	-0.049	-0.006	-0.006	0.011	-0.034	0.012	0.007	0.007	-0.036
92.C	0.000	0.000	0.000	0.000	0.000	0.000	0.000	0.000	0.000
93.O	0.000	0.000	0.000	0.000	0.000	0.000	0.000	0.000	0.000
94.H	-0.057	0.021	0.031	-0.006	-0.090	0.003	0.025	0.028	-0.057
95.H	-0.050	0.029	0.040	-0.003	-0.089	0.006	0.017	0.021	-0.066
96.H	-0.065	0.027	0.039	0.004	-0.096	0.016	0.024	0.029	-0.072

	193			194			195		
1.O	0.000	0.000	0.000	0.000	0.000	0.000	0.000	0.000	0.000

2.C	0.000	0.000	0.000	0.000	0.000	0.000	0.000	0.000	0.000
3.C	0.000	0.000	0.000	0.000	0.000	0.000	0.000	0.000	0.000
4.C	0.000	0.000	0.000	0.000	0.000	0.000	0.000	0.000	0.000
5.O	0.000	0.000	0.000	0.000	0.000	0.000	0.000	0.000	0.000
6.N	0.000	0.000	0.000	0.000	0.000	0.000	0.000	0.000	0.000
7.O	0.000	0.000	0.000	0.000	0.000	0.000	0.000	0.000	0.000
8.C	0.000	0.000	0.000	0.000	0.000	0.000	0.000	0.000	0.000
9.C	0.000	0.000	0.000	0.000	0.000	0.000	0.000	0.000	0.000
10.C	0.000	0.000	0.000	0.000	0.000	0.000	0.000	0.000	0.000
11.O	0.000	0.000	0.000	0.000	0.000	0.000	0.000	0.000	0.000
12.C	0.000	0.000	0.000	0.000	0.000	0.000	0.000	0.000	0.000
13.O	0.000	0.000	0.000	0.000	0.000	0.000	0.000	0.000	0.000
14.C	0.000	0.000	0.000	0.000	0.000	0.000	0.000	0.000	0.000
15.C	0.000	0.000	0.000	0.000	0.000	0.000	0.000	0.000	0.000
16.O	0.000	0.000	0.000	0.000	0.000	0.000	0.000	0.000	0.000
17.C	0.000	0.000	0.000	0.000	0.000	0.000	0.000	0.000	0.000
18.C	0.000	0.000	0.000	0.000	0.000	0.000	0.000	0.000	0.000
19.O	0.000	0.000	0.000	0.000	0.000	0.000	0.000	0.000	0.000
20.C	0.000	0.000	0.000	0.000	0.000	0.000	0.000	0.000	0.000
21.C	0.000	0.000	0.000	0.000	0.000	0.000	0.000	0.000	0.000
22.C	0.000	0.000	0.000	0.000	0.000	0.000	0.000	0.000	0.000
23.C	0.000	0.000	0.000	0.000	0.000	0.000	0.000	0.000	0.000
24.C	0.000	0.000	0.000	0.000	0.000	0.000	0.000	0.000	0.000
25.C	0.000	0.000	0.000	0.000	0.000	0.000	0.000	0.000	0.000
26.C	0.000	0.000	0.000	0.000	0.000	0.000	0.000	0.000	0.000
27.C	0.000	0.000	0.000	0.000	0.000	0.000	0.000	0.000	0.000
28.C	0.000	0.000	0.000	0.000	0.000	0.000	0.000	0.000	0.000
29.C	0.000	0.000	0.000	0.000	0.000	0.000	0.000	0.000	0.000
30.C	0.000	0.000	0.000	0.000	0.000	0.000	0.000	0.000	0.000
31.O	0.000	0.000	0.000	0.000	0.000	0.000	0.000	0.000	0.000
32.C	0.000	0.000	0.000	0.000	0.000	0.000	0.000	0.000	0.000

33.C	0.000	0.000	0.000	0.000	0.000	0.000	0.000	0.000	0.000
34.C	0.000	0.000	0.000	0.000	0.000	0.000	0.000	0.000	0.000
35.C	0.000	0.000	0.000	0.000	0.000	0.000	0.000	0.000	0.000
36.C	0.000	0.000	0.000	0.000	0.000	0.000	0.000	0.000	0.000
37.C	0.000	0.000	0.000	0.000	0.000	0.000	0.000	0.000	0.000
38.C	0.000	0.000	0.000	0.000	0.000	0.000	0.000	0.000	0.000
39.C	0.000	0.000	0.000	0.000	0.000	0.000	0.000	0.000	0.000
40.H	0.000	0.000	0.000	0.000	0.000	0.000	0.000	0.000	0.000
41.H	0.000	0.000	0.000	0.000	0.000	0.000	0.000	0.000	0.000
42.H	0.000	0.000	0.000	0.000	0.000	0.000	0.000	0.000	0.000
43.H	0.000	0.000	0.000	0.000	0.000	0.000	0.000	0.000	0.000
44.H	0.000	0.000	0.000	0.000	0.000	0.000	0.000	0.000	0.000
45.H	0.000	0.000	0.000	0.000	0.000	0.000	0.000	0.000	0.000
46.H	0.000	0.000	0.000	0.000	0.000	0.000	0.000	0.000	0.000
47.H	0.000	0.000	0.000	0.000	0.000	0.000	0.000	0.000	0.000
48.H	0.000	0.000	0.000	0.000	0.000	0.000	0.000	0.000	0.000
49.H	0.000	0.000	0.000	0.000	0.000	0.000	0.000	0.000	0.000
50.H	0.000	0.000	0.000	0.000	0.000	0.000	0.000	0.000	0.000
51.H	0.000	0.000	0.000	0.000	0.000	0.000	0.000	0.000	0.000
52.H	0.000	0.000	0.000	0.000	0.000	0.000	0.000	0.000	0.000
53.H	0.000	0.000	0.000	0.000	0.000	0.000	0.000	0.000	0.000
54.H	0.000	0.000	0.000	0.000	0.000	0.000	0.000	0.000	0.000
55.H	0.000	0.000	0.000	0.000	0.000	0.000	0.000	0.000	0.000
56.H	0.000	0.000	0.000	0.000	0.000	0.000	0.000	0.000	0.000
57.H	0.000	0.000	0.000	0.000	0.000	0.000	0.000	0.000	0.000
58.H	0.000	0.000	0.000	0.000	0.000	0.000	0.000	0.000	0.000
59.H	0.000	0.000	0.000	0.000	0.000	0.000	0.000	0.000	0.000
60.H	0.000	0.000	0.000	0.000	0.000	0.000	0.000	0.000	0.000
61.H	0.938	0.034	0.033	0.000	0.948	0.046	0.000	0.000	0.939
62.H	-0.109	-0.017	0.010	0.023	-0.076	0.028	0.043	0.068	-0.075
63.H	-0.095	-0.015	0.008	0.009	-0.084	0.018	0.047	0.077	-0.061

64.H	-0.093	-0.029	0.001	0.012	-0.066	0.017	0.041	0.068	-0.055
65.H	-0.046	0.012	0.008	0.002	-0.056	0.005	0.012	0.019	-0.047
66.H	-0.035	0.030	0.013	0.008	-0.044	0.009	-0.005	-0.012	-0.055
67.H	-0.042	0.010	0.006	0.010	-0.035	0.008	-0.002	-0.004	-0.048
68.H	-0.056	0.024	0.015	0.017	-0.049	0.017	0.002	-0.000	-0.067
69.C	0.000	0.000	0.000	0.000	0.000	0.000	0.000	0.000	0.000
70.H	0.038	0.004	-0.014	-0.036	-0.012	-0.036	-0.019	-0.026	0.023
71.H	0.041	0.013	-0.011	-0.029	-0.005	-0.033	-0.028	-0.044	0.016
72.H	0.035	0.023	-0.005	-0.031	-0.023	-0.031	-0.020	-0.031	0.008
73.H	0.011	0.016	-0.003	-0.012	-0.014	-0.016	-0.022	-0.036	-0.011
74.H	0.019	-0.016	-0.018	-0.022	0.003	-0.027	-0.018	-0.026	0.016
75.H	0.019	-0.007	-0.015	-0.014	0.010	-0.022	-0.027	-0.043	0.007
76.H	-0.014	-0.044	-0.022	-0.006	0.009	-0.015	-0.008	-0.009	0.005
77.H	0.007	-0.040	-0.025	-0.016	0.013	-0.025	-0.014	-0.018	0.019
78.H	-0.004	-0.051	-0.027	-0.006	0.027	-0.019	-0.017	-0.024	0.014
79.H	-0.033	-0.032	-0.013	0.013	0.011	0.001	-0.014	-0.022	-0.023
80.H	-0.023	-0.020	-0.010	0.014	0.016	0.001	-0.023	-0.039	-0.025
81.H	-0.021	-0.040	-0.019	0.012	0.028	-0.004	-0.022	-0.036	-0.011
82.H	-0.014	0.013	0.001	-0.003	-0.026	-0.005	-0.010	-0.017	-0.028
83.H	-0.012	0.027	0.006	-0.013	-0.051	-0.008	-0.001	-0.001	-0.029
84.H	-0.009	-0.020	-0.014	-0.012	-0.012	-0.016	-0.004	-0.005	-0.002
85.H	-0.010	-0.005	-0.007	0.002	-0.000	-0.007	-0.020	-0.033	-0.019
86.H	-0.082	-0.002	0.010	0.020	-0.059	0.022	0.023	0.035	-0.068
87.O	0.000	0.000	0.000	0.000	0.000	0.000	0.000	0.000	0.000
88.H	-0.063	0.047	0.026	0.014	-0.081	0.021	0.013	0.017	-0.082
89.H	-0.064	-0.033	-0.007	0.016	-0.022	0.010	0.011	0.018	-0.040
90.H	-0.060	-0.019	-0.002	0.021	-0.018	0.014	0.002	0.001	-0.049
91.H	-0.060	-0.013	0.001	0.014	-0.035	0.011	0.010	0.015	-0.047
92.C	0.000	0.000	0.000	0.000	0.000	0.000	0.000	0.000	0.000
93.O	0.000	0.000	0.000	0.000	0.000	0.000	0.000	0.000	0.000
94.H	-0.050	0.040	0.020	-0.007	-0.095	0.006	0.025	0.040	-0.059

95.H	-0.046	0.058	0.027	-0.003	-0.097	0.010	0.018	0.027	-0.069
96.H	-0.066	0.052	0.028	0.005	-0.103	0.018	0.026	0.040	-0.080

	196			197			198		
	------------------------			------------------------			------------------------		
1.O	0.000	0.000	0.000	0.000	0.000	0.000	0.000	0.000	0.000
2.C	0.000	0.000	0.000	0.000	0.000	0.000	0.000	0.000	0.000
3.C	0.000	0.000	0.000	0.000	0.000	0.000	0.000	0.000	0.000
4.C	0.000	0.000	0.000	0.000	0.000	0.000	0.000	0.000	0.000
5.O	0.000	0.000	0.000	0.000	0.000	0.000	0.000	0.000	0.000
6.N	0.000	0.000	0.000	0.000	0.000	0.000	0.000	0.000	0.000
7.O	0.000	0.000	0.000	0.000	0.000	0.000	0.000	0.000	0.000
8.C	0.000	0.000	0.000	0.000	0.000	0.000	0.000	0.000	0.000
9.C	0.000	0.000	0.000	0.000	0.000	0.000	0.000	0.000	0.000
10.C	0.000	0.000	0.000	0.000	0.000	0.000	0.000	0.000	0.000
11.O	0.000	0.000	0.000	0.000	0.000	0.000	0.000	0.000	0.000
12.C	0.000	0.000	0.000	0.000	0.000	0.000	0.000	0.000	0.000
13.O	0.000	0.000	0.000	0.000	0.000	0.000	0.000	0.000	0.000
14.C	0.000	0.000	0.000	0.000	0.000	0.000	0.000	0.000	0.000
15.C	0.000	0.000	0.000	0.000	0.000	0.000	0.000	0.000	0.000
16.O	0.000	0.000	0.000	0.000	0.000	0.000	0.000	0.000	0.000
17.C	0.000	0.000	0.000	0.000	0.000	0.000	0.000	0.000	0.000
18.C	0.000	0.000	0.000	0.000	0.000	0.000	0.000	0.000	0.000
19.O	0.000	0.000	0.000	0.000	0.000	0.000	0.000	0.000	0.000
20.C	0.000	0.000	0.000	0.000	0.000	0.000	0.000	0.000	0.000
21.C	0.000	0.000	0.000	0.000	0.000	0.000	0.000	0.000	0.000
22.C	0.000	0.000	0.000	0.000	0.000	0.000	0.000	0.000	0.000
23.C	0.000	0.000	0.000	0.000	0.000	0.000	0.000	0.000	0.000
24.C	0.000	0.000	0.000	0.000	0.000	0.000	0.000	0.000	0.000
25.C	0.000	0.000	0.000	0.000	0.000	0.000	0.000	0.000	0.000

26.C	0.000	0.000	0.000	0.000	0.000	0.000	0.000	0.000	0.000
27.C	0.000	0.000	0.000	0.000	0.000	0.000	0.000	0.000	0.000
28.C	0.000	0.000	0.000	0.000	0.000	0.000	0.000	0.000	0.000
29.C	0.000	0.000	0.000	0.000	0.000	0.000	0.000	0.000	0.000
30.C	0.000	0.000	0.000	0.000	0.000	0.000	0.000	0.000	0.000
31.O	0.000	0.000	0.000	0.000	0.000	0.000	0.000	0.000	0.000
32.C	0.000	0.000	0.000	0.000	0.000	0.000	0.000	0.000	0.000
33.C	0.000	0.000	0.000	0.000	0.000	0.000	0.000	0.000	0.000
34.C	0.000	0.000	0.000	0.000	0.000	0.000	0.000	0.000	0.000
35.C	0.000	0.000	0.000	0.000	0.000	0.000	0.000	0.000	0.000
36.C	0.000	0.000	0.000	0.000	0.000	0.000	0.000	0.000	0.000
37.C	0.000	0.000	0.000	0.000	0.000	0.000	0.000	0.000	0.000
38.C	0.000	0.000	0.000	0.000	0.000	0.000	0.000	0.000	0.000
39.C	0.000	0.000	0.000	0.000	0.000	0.000	0.000	0.000	0.000
40.H	0.000	0.000	0.000	0.000	0.000	0.000	0.000	0.000	0.000
41.H	0.000	0.000	0.000	0.000	0.000	0.000	0.000	0.000	0.000
42.H	0.000	0.000	0.000	0.000	0.000	0.000	0.000	0.000	0.000
43.H	0.000	0.000	0.000	0.000	0.000	0.000	0.000	0.000	0.000
44.H	0.000	0.000	0.000	0.000	0.000	0.000	0.000	0.000	0.000
45.H	0.000	0.000	0.000	0.000	0.000	0.000	0.000	0.000	0.000
46.H	0.000	0.000	0.000	0.000	0.000	0.000	0.000	0.000	0.000
47.H	0.000	0.000	0.000	0.000	0.000	0.000	0.000	0.000	0.000
48.H	0.000	0.000	0.000	0.000	0.000	0.000	0.000	0.000	0.000
49.H	0.000	0.000	0.000	0.000	0.000	0.000	0.000	0.000	0.000
50.H	0.000	0.000	0.000	0.000	0.000	0.000	0.000	0.000	0.000
51.H	0.000	0.000	0.000	0.000	0.000	0.000	0.000	0.000	0.000
52.H	0.000	0.000	0.000	0.000	0.000	0.000	0.000	0.000	0.000
53.H	0.000	0.000	0.000	0.000	0.000	0.000	0.000	0.000	0.000
54.H	0.000	0.000	0.000	0.000	0.000	0.000	0.000	0.000	0.000
55.H	0.000	0.000	0.000	0.000	0.000	0.000	0.000	0.000	0.000
56.H	0.000	0.000	0.000	0.000	0.000	0.000	0.000	0.000	0.000

57.H	0.000	0.000	0.000	0.000	0.000	0.000	0.000	0.000	0.000
58.H	0.000	0.000	0.000	0.000	0.000	0.000	0.000	0.000	0.000
59.H	0.000	0.000	0.000	0.000	0.000	0.000	0.000	0.000	0.000
60.H	0.000	0.000	0.000	0.000	0.000	0.000	0.000	0.000	0.000
61.H	0.000	0.000	0.000	0.000	0.000	0.000	0.000	0.000	0.000
62.H	0.934	-0.007	0.006	0.000	0.933	0.063	0.000	0.000	0.958
63.H	-0.126	-0.006	0.007	0.002	-0.142	0.040	0.000	0.078	-0.062
64.H	-0.118	-0.015	-0.007	-0.002	-0.135	0.046	0.002	0.073	-0.066
65.H	-0.058	0.009	0.017	0.001	-0.055	0.006	-0.000	0.016	-0.038
66.H	-0.031	0.019	0.026	-0.007	-0.007	0.007	0.005	-0.017	-0.039
67.H	-0.039	0.007	0.010	-0.007	-0.024	0.016	0.005	-0.005	-0.044
68.H	-0.051	0.015	0.025	-0.011	-0.025	0.028	0.008	-0.005	-0.053
69.C	0.000	0.000	0.000	0.000	0.000	0.000	0.000	0.000	0.000
70.H	0.026	0.002	-0.007	0.020	0.002	-0.075	-0.015	-0.023	0.019
71.H	0.039	0.007	-0.002	0.014	0.029	-0.069	-0.011	-0.042	0.015
72.H	0.026	0.014	0.010	0.017	0.016	-0.072	-0.013	-0.033	0.017
73.H	0.015	0.010	0.005	0.004	0.020	-0.036	-0.004	-0.036	-0.008
74.H	0.014	-0.010	-0.023	0.011	-0.006	-0.045	-0.008	-0.018	-0.002
75.H	0.026	-0.005	-0.018	0.005	0.020	-0.037	-0.004	-0.036	-0.007
76.H	-0.017	-0.027	-0.041	0.002	-0.039	-0.005	-0.002	0.006	-0.029
77.H	0.002	-0.024	-0.041	0.008	-0.025	-0.028	-0.006	-0.004	-0.013
78.H	0.000	-0.032	-0.051	0.001	-0.022	-0.009	-0.001	-0.006	-0.027
79.H	-0.019	-0.020	-0.031	-0.011	-0.017	0.025	0.008	-0.010	-0.050
80.H	-0.002	-0.013	-0.024	-0.014	0.011	0.020	0.009	-0.029	-0.047
81.H	-0.002	-0.025	-0.041	-0.012	-0.001	0.019	0.008	-0.021	-0.046
82.H	-0.013	0.008	0.008	0.000	-0.006	-0.013	-0.000	-0.018	-0.024
83.H	-0.024	0.017	0.023	0.008	-0.023	-0.030	-0.006	-0.006	-0.013
84.H	-0.017	-0.012	-0.020	0.007	-0.035	-0.020	-0.005	0.003	-0.019
85.H	0.001	-0.003	-0.011	-0.005	0.009	-0.005	0.003	-0.028	-0.030
86.H	-0.090	0.001	0.010	-0.009	-0.081	0.046	0.007	0.035	-0.066
87.O	0.000	0.000	0.000	0.000	0.000	0.000	0.000	0.000	0.000

88.H	-0.068	0.030	0.049	-0.007	-0.039	0.022	0.005	0.005	-0.049
89.H	-0.066	-0.019	-0.022	-0.009	-0.070	0.040	0.007	0.027	-0.061
90.H	-0.053	-0.011	-0.013	-0.014	-0.043	0.043	0.010	0.008	-0.064
91.H	-0.063	-0.007	-0.005	-0.008	-0.059	0.032	0.006	0.020	-0.056
92.C	0.000	0.000	0.000	0.000	0.000	0.000	0.000	0.000	0.000
93.O	0.000	0.000	0.000	0.000	0.000	0.000	0.000	0.000	0.000
94.H	-0.075	0.027	0.046	0.008	-0.072	-0.009	-0.005	0.028	-0.027
95.H	-0.065	0.037	0.059	0.005	-0.048	-0.009	-0.003	0.011	-0.028
96.H	-0.086	0.034	0.057	0.001	-0.067	0.010	-0.000	0.025	-0.041

	199			200			201		
	------------------------			------------------------			------------------------		
1.O	0.000	0.000	0.000	0.000	0.000	0.000	0.000	0.000	0.000
2.C	0.000	0.000	0.000	0.000	0.000	0.000	0.000	0.000	0.000
3.C	0.000	0.000	0.000	0.000	0.000	0.000	0.000	0.000	0.000
4.C	0.000	0.000	0.000	0.000	0.000	0.000	0.000	0.000	0.000
5.O	0.000	0.000	0.000	0.000	0.000	0.000	0.000	0.000	0.000
6.N	0.000	0.000	0.000	0.000	0.000	0.000	0.000	0.000	0.000
7.O	0.000	0.000	0.000	0.000	0.000	0.000	0.000	0.000	0.000
8.C	0.000	0.000	0.000	0.000	0.000	0.000	0.000	0.000	0.000
9.C	0.000	0.000	0.000	0.000	0.000	0.000	0.000	0.000	0.000
10.C	0.000	0.000	0.000	0.000	0.000	0.000	0.000	0.000	0.000
11.O	0.000	0.000	0.000	0.000	0.000	0.000	0.000	0.000	0.000
12.C	0.000	0.000	0.000	0.000	0.000	0.000	0.000	0.000	0.000
13.O	0.000	0.000	0.000	0.000	0.000	0.000	0.000	0.000	0.000
14.C	0.000	0.000	0.000	0.000	0.000	0.000	0.000	0.000	0.000
15.C	0.000	0.000	0.000	0.000	0.000	0.000	0.000	0.000	0.000
16.O	0.000	0.000	0.000	0.000	0.000	0.000	0.000	0.000	0.000
17.C	0.000	0.000	0.000	0.000	0.000	0.000	0.000	0.000	0.000
18.C	0.000	0.000	0.000	0.000	0.000	0.000	0.000	0.000	0.000

19.O	0.000	0.000	0.000	0.000	0.000	0.000	0.000	0.000	0.000
20.C	0.000	0.000	0.000	0.000	0.000	0.000	0.000	0.000	0.000
21.C	0.000	0.000	0.000	0.000	0.000	0.000	0.000	0.000	0.000
22.C	0.000	0.000	0.000	0.000	0.000	0.000	0.000	0.000	0.000
23.C	0.000	0.000	0.000	0.000	0.000	0.000	0.000	0.000	0.000
24.C	0.000	0.000	0.000	0.000	0.000	0.000	0.000	0.000	0.000
25.C	0.000	0.000	0.000	0.000	0.000	0.000	0.000	0.000	0.000
26.C	0.000	0.000	0.000	0.000	0.000	0.000	0.000	0.000	0.000
27.C	0.000	0.000	0.000	0.000	0.000	0.000	0.000	0.000	0.000
28.C	0.000	0.000	0.000	0.000	0.000	0.000	0.000	0.000	0.000
29.C	0.000	0.000	0.000	0.000	0.000	0.000	0.000	0.000	0.000
30.C	0.000	0.000	0.000	0.000	0.000	0.000	0.000	0.000	0.000
31.O	0.000	0.000	0.000	0.000	0.000	0.000	0.000	0.000	0.000
32.C	0.000	0.000	0.000	0.000	0.000	0.000	0.000	0.000	0.000
33.C	0.000	0.000	0.000	0.000	0.000	0.000	0.000	0.000	0.000
34.C	0.000	0.000	0.000	0.000	0.000	0.000	0.000	0.000	0.000
35.C	0.000	0.000	0.000	0.000	0.000	0.000	0.000	0.000	0.000
36.C	0.000	0.000	0.000	0.000	0.000	0.000	0.000	0.000	0.000
37.C	0.000	0.000	0.000	0.000	0.000	0.000	0.000	0.000	0.000
38.C	0.000	0.000	0.000	0.000	0.000	0.000	0.000	0.000	0.000
39.C	0.000	0.000	0.000	0.000	0.000	0.000	0.000	0.000	0.000
40.H	0.000	0.000	0.000	0.000	0.000	0.000	0.000	0.000	0.000
41.H	0.000	0.000	0.000	0.000	0.000	0.000	0.000	0.000	0.000
42.H	0.000	0.000	0.000	0.000	0.000	0.000	0.000	0.000	0.000
43.H	0.000	0.000	0.000	0.000	0.000	0.000	0.000	0.000	0.000
44.H	0.000	0.000	0.000	0.000	0.000	0.000	0.000	0.000	0.000
45.H	0.000	0.000	0.000	0.000	0.000	0.000	0.000	0.000	0.000
46.H	0.000	0.000	0.000	0.000	0.000	0.000	0.000	0.000	0.000
47.H	0.000	0.000	0.000	0.000	0.000	0.000	0.000	0.000	0.000
48.H	0.000	0.000	0.000	0.000	0.000	0.000	0.000	0.000	0.000
49.H	0.000	0.000	0.000	0.000	0.000	0.000	0.000	0.000	0.000

50.H	0.000	0.000	0.000	0.000	0.000	0.000	0.000	0.000	0.000
51.H	0.000	0.000	0.000	0.000	0.000	0.000	0.000	0.000	0.000
52.H	0.000	0.000	0.000	0.000	0.000	0.000	0.000	0.000	0.000
53.H	0.000	0.000	0.000	0.000	0.000	0.000	0.000	0.000	0.000
54.H	0.000	0.000	0.000	0.000	0.000	0.000	0.000	0.000	0.000
55.H	0.000	0.000	0.000	0.000	0.000	0.000	0.000	0.000	0.000
56.H	0.000	0.000	0.000	0.000	0.000	0.000	0.000	0.000	0.000
57.H	0.000	0.000	0.000	0.000	0.000	0.000	0.000	0.000	0.000
58.H	0.000	0.000	0.000	0.000	0.000	0.000	0.000	0.000	0.000
59.H	0.000	0.000	0.000	0.000	0.000	0.000	0.000	0.000	0.000
60.H	0.000	0.000	0.000	0.000	0.000	0.000	0.000	0.000	0.000
61.H	0.000	0.000	0.000	0.000	0.000	0.000	0.000	0.000	0.000
62.H	0.000	0.000	0.000	0.000	0.000	0.000	0.000	0.000	0.000
63.H	0.922	0.003	0.005	0.000	0.907	0.057	0.000	0.000	0.967
64.H	-0.141	-0.001	-0.016	-0.001	-0.177	0.065	0.006	0.061	-0.060
65.H	-0.069	0.004	0.023	0.001	-0.070	0.011	0.002	0.016	-0.040
66.H	-0.027	0.006	0.037	-0.008	-0.006	0.013	0.003	-0.011	-0.042
67.H	-0.037	0.002	0.013	-0.008	-0.028	0.024	0.004	-0.002	-0.045
68.H	-0.047	0.005	0.032	-0.013	-0.030	0.041	0.006	-0.001	-0.053
69.C	0.000	0.000	0.000	0.000	0.000	0.000	0.000	0.000	0.000
70.H	0.009	-0.001	-0.001	0.024	0.009	-0.099	-0.012	-0.017	0.007
71.H	0.032	-0.000	0.006	0.017	0.045	-0.091	-0.010	-0.032	0.003
72.H	0.013	0.003	0.024	0.020	0.026	-0.094	-0.011	-0.024	0.004
73.H	0.015	0.001	0.012	0.005	0.032	-0.046	-0.005	-0.026	-0.016
74.H	0.007	-0.005	-0.028	0.013	-0.000	-0.059	-0.008	-0.013	-0.008
75.H	0.028	-0.004	-0.021	0.005	0.034	-0.047	-0.005	-0.027	-0.013
76.H	-0.021	-0.010	-0.060	0.003	-0.043	-0.005	-0.002	0.005	-0.029
77.H	-0.005	-0.010	-0.056	0.010	-0.025	-0.037	-0.005	-0.002	-0.016
78.H	0.001	-0.012	-0.073	0.001	-0.019	-0.010	-0.002	-0.004	-0.026
79.H	-0.008	-0.008	-0.048	-0.014	-0.014	0.036	0.004	-0.007	-0.046
80.H	0.016	-0.007	-0.038	-0.017	0.023	0.029	0.004	-0.022	-0.044

81.H	0.013	-0.011	-0.063	-0.014	0.008	0.028	0.004	-0.016	-0.042
82.H	-0.014	0.002	0.013	0.000	-0.003	-0.015	-0.001	-0.012	-0.029
83.H	-0.037	0.006	0.037	0.010	-0.027	-0.037	-0.004	-0.002	-0.021
84.H	-0.027	-0.004	-0.027	0.008	-0.041	-0.025	-0.004	0.004	-0.022
85.H	0.009	-0.003	-0.015	-0.006	0.018	-0.004	0.000	-0.020	-0.032
86.H	-0.097	0.002	0.009	-0.011	-0.105	0.066	0.008	0.031	-0.062
87.O	0.000	0.000	0.000	0.000	0.000	0.000	0.000	0.000	0.000
88.H	-0.073	0.012	0.068	-0.008	-0.051	0.033	0.006	0.008	-0.052
89.H	-0.068	-0.005	-0.037	-0.010	-0.087	0.057	0.006	0.023	-0.056
90.H	-0.046	-0.004	-0.024	-0.016	-0.051	0.061	0.008	0.008	-0.058
91.H	-0.065	-0.001	-0.011	-0.009	-0.074	0.046	0.006	0.018	-0.053
92.C	0.000	0.000	0.000	0.000	0.000	0.000	0.000	0.000	0.000
93.O	0.000	0.000	0.000	0.000	0.000	0.000	0.000	0.000	0.000
94.H	-0.100	0.012	0.067	0.011	-0.096	-0.009	-0.001	0.026	-0.034
95.H	-0.083	0.015	0.086	0.006	-0.064	-0.009	0.000	0.013	-0.035
96.H	-0.104	0.014	0.082	0.002	-0.090	0.018	0.003	0.024	-0.046

	202			203			204		
	------------------------			------------------------			------------------------		
1.O	0.000	0.000	0.000	0.000	0.000	0.000	0.000	0.000	0.000
2.C	0.000	0.000	0.000	0.000	0.000	0.000	0.000	0.000	0.000
3.C	0.000	0.000	0.000	0.000	0.000	0.000	0.000	0.000	0.000
4.C	0.000	0.000	0.000	0.000	0.000	0.000	0.000	0.000	0.000
5.O	0.000	0.000	0.000	0.000	0.000	0.000	0.000	0.000	0.000
6.N	0.000	0.000	0.000	0.000	0.000	0.000	0.000	0.000	0.000
7.O	0.000	0.000	0.000	0.000	0.000	0.000	0.000	0.000	0.000
8.C	0.000	0.000	0.000	0.000	0.000	0.000	0.000	0.000	0.000
9.C	0.000	0.000	0.000	0.000	0.000	0.000	0.000	0.000	0.000
10.C	0.000	0.000	0.000	0.000	0.000	0.000	0.000	0.000	0.000
11.O	0.000	0.000	0.000	0.000	0.000	0.000	0.000	0.000	0.000

12.C	0.000	0.000	0.000	0.000	0.000	0.000	0.000	0.000	0.000
13.O	0.000	0.000	0.000	0.000	0.000	0.000	0.000	0.000	0.000
14.C	0.000	0.000	0.000	0.000	0.000	0.000	0.000	0.000	0.000
15.C	0.000	0.000	0.000	0.000	0.000	0.000	0.000	0.000	0.000
16.O	0.000	0.000	0.000	0.000	0.000	0.000	0.000	0.000	0.000
17.C	0.000	0.000	0.000	0.000	0.000	0.000	0.000	0.000	0.000
18.C	0.000	0.000	0.000	0.000	0.000	0.000	0.000	0.000	0.000
19.O	0.000	0.000	0.000	0.000	0.000	0.000	0.000	0.000	0.000
20.C	0.000	0.000	0.000	0.000	0.000	0.000	0.000	0.000	0.000
21.C	0.000	0.000	0.000	0.000	0.000	0.000	0.000	0.000	0.000
22.C	0.000	0.000	0.000	0.000	0.000	0.000	0.000	0.000	0.000
23.C	0.000	0.000	0.000	0.000	0.000	0.000	0.000	0.000	0.000
24.C	0.000	0.000	0.000	0.000	0.000	0.000	0.000	0.000	0.000
25.C	0.000	0.000	0.000	0.000	0.000	0.000	0.000	0.000	0.000
26.C	0.000	0.000	0.000	0.000	0.000	0.000	0.000	0.000	0.000
27.C	0.000	0.000	0.000	0.000	0.000	0.000	0.000	0.000	0.000
28.C	0.000	0.000	0.000	0.000	0.000	0.000	0.000	0.000	0.000
29.C	0.000	0.000	0.000	0.000	0.000	0.000	0.000	0.000	0.000
30.C	0.000	0.000	0.000	0.000	0.000	0.000	0.000	0.000	0.000
31.O	0.000	0.000	0.000	0.000	0.000	0.000	0.000	0.000	0.000
32.C	0.000	0.000	0.000	0.000	0.000	0.000	0.000	0.000	0.000
33.C	0.000	0.000	0.000	0.000	0.000	0.000	0.000	0.000	0.000
34.C	0.000	0.000	0.000	0.000	0.000	0.000	0.000	0.000	0.000
35.C	0.000	0.000	0.000	0.000	0.000	0.000	0.000	0.000	0.000
36.C	0.000	0.000	0.000	0.000	0.000	0.000	0.000	0.000	0.000
37.C	0.000	0.000	0.000	0.000	0.000	0.000	0.000	0.000	0.000
38.C	0.000	0.000	0.000	0.000	0.000	0.000	0.000	0.000	0.000
39.C	0.000	0.000	0.000	0.000	0.000	0.000	0.000	0.000	0.000
40.H	0.000	0.000	0.000	0.000	0.000	0.000	0.000	0.000	0.000
41.H	0.000	0.000	0.000	0.000	0.000	0.000	0.000	0.000	0.000
42.H	0.000	0.000	0.000	0.000	0.000	0.000	0.000	0.000	0.000

43.H	0.000	0.000	0.000	0.000	0.000	0.000	0.000	0.000	0.000
44.H	0.000	0.000	0.000	0.000	0.000	0.000	0.000	0.000	0.000
45.H	0.000	0.000	0.000	0.000	0.000	0.000	0.000	0.000	0.000
46.H	0.000	0.000	0.000	0.000	0.000	0.000	0.000	0.000	0.000
47.H	0.000	0.000	0.000	0.000	0.000	0.000	0.000	0.000	0.000
48.H	0.000	0.000	0.000	0.000	0.000	0.000	0.000	0.000	0.000
49.H	0.000	0.000	0.000	0.000	0.000	0.000	0.000	0.000	0.000
50.H	0.000	0.000	0.000	0.000	0.000	0.000	0.000	0.000	0.000
51.H	0.000	0.000	0.000	0.000	0.000	0.000	0.000	0.000	0.000
52.H	0.000	0.000	0.000	0.000	0.000	0.000	0.000	0.000	0.000
53.H	0.000	0.000	0.000	0.000	0.000	0.000	0.000	0.000	0.000
54.H	0.000	0.000	0.000	0.000	0.000	0.000	0.000	0.000	0.000
55.H	0.000	0.000	0.000	0.000	0.000	0.000	0.000	0.000	0.000
56.H	0.000	0.000	0.000	0.000	0.000	0.000	0.000	0.000	0.000
57.H	0.000	0.000	0.000	0.000	0.000	0.000	0.000	0.000	0.000
58.H	0.000	0.000	0.000	0.000	0.000	0.000	0.000	0.000	0.000
59.H	0.000	0.000	0.000	0.000	0.000	0.000	0.000	0.000	0.000
60.H	0.000	0.000	0.000	0.000	0.000	0.000	0.000	0.000	0.000
61.H	0.000	0.000	0.000	0.000	0.000	0.000	0.000	0.000	0.000
62.H	0.000	0.000	0.000	0.000	0.000	0.000	0.000	0.000	0.000
63.H	0.000	0.000	0.000	0.000	0.000	0.000	0.000	0.000	0.000
64.H	0.924	-0.007	-0.014	0.000	0.894	0.080	0.000	0.000	0.957
65.H	-0.076	0.006	0.024	-0.000	-0.077	0.014	-0.007	0.024	-0.039
66.H	-0.035	0.011	0.038	-0.015	0.003	0.015	0.007	-0.016	-0.038
67.H	-0.045	0.004	0.014	-0.016	-0.030	0.030	0.006	-0.000	-0.049
68.H	-0.056	0.009	0.034	-0.026	-0.027	0.049	0.009	-0.001	-0.055
69.C	0.000	0.000	0.000	0.000	0.000	0.000	0.000	0.000	0.000
70.H	0.013	0.000	-0.003	0.047	0.009	-0.114	-0.017	-0.021	0.023
71.H	0.035	0.003	0.004	0.035	0.053	-0.105	-0.007	-0.044	0.019
72.H	0.016	0.007	0.021	0.040	0.036	-0.110	-0.013	-0.034	0.024
73.H	0.013	0.005	0.011	0.011	0.040	-0.052	0.002	-0.036	-0.007

74.H	0.007	-0.007	-0.029	0.026	-0.010	-0.065	-0.007	-0.013	-0.007
75.H	0.027	-0.005	-0.022	0.013	0.033	-0.052	0.003	-0.034	-0.012
76.H	-0.025	-0.017	-0.059	0.005	-0.070	-0.000	-0.001	0.017	-0.045
77.H	-0.006	-0.016	-0.057	0.019	-0.047	-0.037	-0.005	0.005	-0.026
78.H	-0.002	-0.021	-0.073	0.003	-0.046	-0.005	0.003	0.004	-0.045
79.H	-0.016	-0.013	-0.047	-0.024	-0.032	0.047	0.015	-0.002	-0.068
80.H	0.008	-0.009	-0.036	-0.028	0.015	0.039	0.021	-0.026	-0.062
81.H	0.006	-0.017	-0.061	-0.024	-0.010	0.040	0.019	-0.014	-0.066
82.H	-0.018	0.004	0.013	0.001	-0.001	-0.016	0.002	-0.015	-0.025
83.H	-0.039	0.011	0.036	0.018	-0.023	-0.043	-0.010	-0.003	-0.007
84.H	-0.030	-0.008	-0.027	0.015	-0.057	-0.026	-0.007	0.012	-0.027
85.H	0.004	-0.003	-0.015	-0.009	0.016	-0.002	0.011	-0.025	-0.037
86.H	-0.108	0.002	0.011	-0.024	-0.122	0.079	0.000	0.047	-0.075
87.O	0.000	0.000	0.000	0.000	0.000	0.000	0.000	0.000	0.000
88.H	-0.082	0.019	0.069	-0.018	-0.042	0.038	0.001	0.008	-0.044
89.H	-0.077	-0.011	-0.034	-0.021	-0.115	0.071	0.004	0.041	-0.078
90.H	-0.057	-0.007	-0.022	-0.032	-0.068	0.076	0.012	0.018	-0.078
91.H	-0.074	-0.004	-0.009	-0.019	-0.092	0.058	0.003	0.030	-0.067
92.C	0.000	0.000	0.000	0.000	0.000	0.000	0.000	0.000	0.000
93.O	0.000	0.000	0.000	0.000	0.000	0.000	0.000	0.000	0.000
94.H	-0.105	0.018	0.067	0.016	-0.095	-0.012	-0.020	0.035	-0.018
95.H	-0.089	0.024	0.086	0.008	-0.052	-0.013	-0.013	0.014	-0.015
96.H	-0.111	0.022	0.082	-0.001	-0.084	0.018	-0.012	0.030	-0.032

	205			206			207		
1.O	0.000	0.000	0.000	0.000	0.000	0.000	0.000	0.000	0.000
2.C	0.000	0.000	0.000	0.000	0.000	0.000	0.000	0.000	0.000
3.C	0.000	0.000	0.000	0.000	0.000	0.000	0.000	0.000	0.000
4.C	0.000	0.000	0.000	0.000	0.000	0.000	0.000	0.000	0.000

5.O	0.000	0.000	0.000	0.000	0.000	0.000	0.000	0.000	0.000
6.N	0.000	0.000	0.000	0.000	0.000	0.000	0.000	0.000	0.000
7.O	0.000	0.000	0.000	0.000	0.000	0.000	0.000	0.000	0.000
8.C	0.000	0.000	0.000	0.000	0.000	0.000	0.000	0.000	0.000
9.C	0.000	0.000	0.000	0.000	0.000	0.000	0.000	0.000	0.000
10.C	0.000	0.000	0.000	0.000	0.000	0.000	0.000	0.000	0.000
11.O	0.000	0.000	0.000	0.000	0.000	0.000	0.000	0.000	0.000
12.C	0.000	0.000	0.000	0.000	0.000	0.000	0.000	0.000	0.000
13.O	0.000	0.000	0.000	0.000	0.000	0.000	0.000	0.000	0.000
14.C	0.000	0.000	0.000	0.000	0.000	0.000	0.000	0.000	0.000
15.C	0.000	0.000	0.000	0.000	0.000	0.000	0.000	0.000	0.000
16.O	0.000	0.000	0.000	0.000	0.000	0.000	0.000	0.000	0.000
17.C	0.000	0.000	0.000	0.000	0.000	0.000	0.000	0.000	0.000
18.C	0.000	0.000	0.000	0.000	0.000	0.000	0.000	0.000	0.000
19.O	0.000	0.000	0.000	0.000	0.000	0.000	0.000	0.000	0.000
20.C	0.000	0.000	0.000	0.000	0.000	0.000	0.000	0.000	0.000
21.C	0.000	0.000	0.000	0.000	0.000	0.000	0.000	0.000	0.000
22.C	0.000	0.000	0.000	0.000	0.000	0.000	0.000	0.000	0.000
23.C	0.000	0.000	0.000	0.000	0.000	0.000	0.000	0.000	0.000
24.C	0.000	0.000	0.000	0.000	0.000	0.000	0.000	0.000	0.000
25.C	0.000	0.000	0.000	0.000	0.000	0.000	0.000	0.000	0.000
26.C	0.000	0.000	0.000	0.000	0.000	0.000	0.000	0.000	0.000
27.C	0.000	0.000	0.000	0.000	0.000	0.000	0.000	0.000	0.000
28.C	0.000	0.000	0.000	0.000	0.000	0.000	0.000	0.000	0.000
29.C	0.000	0.000	0.000	0.000	0.000	0.000	0.000	0.000	0.000
30.C	0.000	0.000	0.000	0.000	0.000	0.000	0.000	0.000	0.000
31.O	0.000	0.000	0.000	0.000	0.000	0.000	0.000	0.000	0.000
32.C	0.000	0.000	0.000	0.000	0.000	0.000	0.000	0.000	0.000
33.C	0.000	0.000	0.000	0.000	0.000	0.000	0.000	0.000	0.000
34.C	0.000	0.000	0.000	0.000	0.000	0.000	0.000	0.000	0.000
35.C	0.000	0.000	0.000	0.000	0.000	0.000	0.000	0.000	0.000

36.C	0.000	0.000	0.000	0.000	0.000	0.000	0.000	0.000	0.000
37.C	0.000	0.000	0.000	0.000	0.000	0.000	0.000	0.000	0.000
38.C	0.000	0.000	0.000	0.000	0.000	0.000	0.000	0.000	0.000
39.C	0.000	0.000	0.000	0.000	0.000	0.000	0.000	0.000	0.000
40.H	0.000	0.000	0.000	0.000	0.000	0.000	0.000	0.000	0.000
41.H	0.000	0.000	0.000	0.000	0.000	0.000	0.000	0.000	0.000
42.H	0.000	0.000	0.000	0.000	0.000	0.000	0.000	0.000	0.000
43.H	0.000	0.000	0.000	0.000	0.000	0.000	0.000	0.000	0.000
44.H	0.000	0.000	0.000	0.000	0.000	0.000	0.000	0.000	0.000
45.H	0.000	0.000	0.000	0.000	0.000	0.000	0.000	0.000	0.000
46.H	0.000	0.000	0.000	0.000	0.000	0.000	0.000	0.000	0.000
47.H	0.000	0.000	0.000	0.000	0.000	0.000	0.000	0.000	0.000
48.H	0.000	0.000	0.000	0.000	0.000	0.000	0.000	0.000	0.000
49.H	0.000	0.000	0.000	0.000	0.000	0.000	0.000	0.000	0.000
50.H	0.000	0.000	0.000	0.000	0.000	0.000	0.000	0.000	0.000
51.H	0.000	0.000	0.000	0.000	0.000	0.000	0.000	0.000	0.000
52.H	0.000	0.000	0.000	0.000	0.000	0.000	0.000	0.000	0.000
53.H	0.000	0.000	0.000	0.000	0.000	0.000	0.000	0.000	0.000
54.H	0.000	0.000	0.000	0.000	0.000	0.000	0.000	0.000	0.000
55.H	0.000	0.000	0.000	0.000	0.000	0.000	0.000	0.000	0.000
56.H	0.000	0.000	0.000	0.000	0.000	0.000	0.000	0.000	0.000
57.H	0.000	0.000	0.000	0.000	0.000	0.000	0.000	0.000	0.000
58.H	0.000	0.000	0.000	0.000	0.000	0.000	0.000	0.000	0.000
59.H	0.000	0.000	0.000	0.000	0.000	0.000	0.000	0.000	0.000
60.H	0.000	0.000	0.000	0.000	0.000	0.000	0.000	0.000	0.000
61.H	0.000	0.000	0.000	0.000	0.000	0.000	0.000	0.000	0.000
62.H	0.000	0.000	0.000	0.000	0.000	0.000	0.000	0.000	0.000
63.H	0.000	0.000	0.000	0.000	0.000	0.000	0.000	0.000	0.000
64.H	0.000	0.000	0.000	0.000	0.000	0.000	0.000	0.000	0.000
65.H	0.970	0.002	0.010	0.000	0.967	0.007	0.000	0.000	0.974
66.H	-0.036	0.002	0.015	0.002	-0.039	0.009	-0.000	-0.000	-0.049

67.H	-0.040	0.001	0.005	0.002	-0.041	0.012	0.002	0.001	-0.044
68.H	-0.044	0.002	0.013	0.004	-0.047	0.019	0.004	0.003	-0.052
69.C	0.000	0.000	0.000	0.000	0.000	0.000	0.000	0.000	0.000
70.H	-0.021	-0.000	-0.000	-0.007	-0.023	-0.036	-0.006	-0.005	-0.023
71.H	-0.011	-0.000	0.003	-0.007	-0.011	-0.033	-0.012	-0.009	-0.026
72.H	-0.019	0.001	0.010	-0.007	-0.022	-0.033	-0.007	-0.006	-0.030
73.H	-0.018	0.000	0.005	-0.003	-0.017	-0.015	-0.009	-0.007	-0.034
74.H	-0.022	-0.002	-0.012	-0.005	-0.019	-0.022	-0.007	-0.005	-0.021
75.H	-0.013	-0.002	-0.009	-0.004	-0.008	-0.018	-0.012	-0.009	-0.024
76.H	-0.034	-0.004	-0.025	-0.001	-0.027	-0.004	-0.001	-0.001	-0.020
77.H	-0.027	-0.004	-0.024	-0.003	-0.021	-0.016	-0.004	-0.003	-0.016
78.H	-0.024	-0.005	-0.031	-0.002	-0.015	-0.006	-0.006	-0.005	-0.015
79.H	-0.028	-0.003	-0.021	0.002	-0.019	0.013	-0.005	-0.004	-0.029
80.H	-0.018	-0.003	-0.016	0.001	-0.008	0.011	-0.011	-0.009	-0.031
81.H	-0.019	-0.005	-0.027	0.001	-0.007	0.009	-0.011	-0.008	-0.024
82.H	-0.031	0.001	0.005	-0.001	-0.031	-0.003	-0.003	-0.002	-0.038
83.H	-0.040	0.002	0.016	-0.002	-0.047	-0.010	0.003	0.003	-0.042
84.H	-0.036	-0.002	-0.011	-0.002	-0.035	-0.010	0.001	0.001	-0.026
85.H	-0.021	-0.001	-0.006	-0.001	-0.016	-0.001	-0.009	-0.007	-0.032
86.H	-0.065	0.001	0.003	0.006	-0.070	0.028	0.015	0.012	-0.049
87.O	0.000	0.000	0.000	0.000	0.000	0.000	0.000	0.000	0.000
88.H	-0.055	0.005	0.028	0.004	-0.064	0.018	0.010	0.008	-0.060
89.H	-0.053	-0.002	-0.016	0.005	-0.051	0.021	0.009	0.007	-0.035
90.H	-0.044	-0.001	-0.011	0.005	-0.040	0.024	0.003	0.002	-0.039
91.H	-0.052	-0.000	-0.005	0.004	-0.052	0.019	0.008	0.006	-0.040
92.C	0.000	0.000	0.000	0.000	0.000	0.000	0.000	0.000	0.000
93.O	0.000	0.000	0.000	0.000	0.000	0.000	0.000	0.000	0.000
94.H	-0.067	0.005	0.028	0.002	-0.081	0.002	0.018	0.014	-0.054
95.H	-0.060	0.006	0.036	0.002	-0.074	0.003	0.014	0.010	-0.059
96.H	-0.068	0.006	0.034	0.004	-0.083	0.013	0.018	0.014	-0.062

	208			209			210		
	------------------------			------------------------			------------------------		
1.O	0.000	0.000	0.000	0.000	0.000	0.000	0.000	0.000	0.000
2.C	0.000	0.000	0.000	0.000	0.000	0.000	0.000	0.000	0.000
3.C	0.000	0.000	0.000	0.000	0.000	0.000	0.000	0.000	0.000
4.C	0.000	0.000	0.000	0.000	0.000	0.000	0.000	0.000	0.000
5.O	0.000	0.000	0.000	0.000	0.000	0.000	0.000	0.000	0.000
6.N	0.000	0.000	0.000	0.000	0.000	0.000	0.000	0.000	0.000
7.O	0.000	0.000	0.000	0.000	0.000	0.000	0.000	0.000	0.000
8.C	0.000	0.000	0.000	0.000	0.000	0.000	0.000	0.000	0.000
9.C	0.000	0.000	0.000	0.000	0.000	0.000	0.000	0.000	0.000
10.C	0.000	0.000	0.000	0.000	0.000	0.000	0.000	0.000	0.000
11.O	0.000	0.000	0.000	0.000	0.000	0.000	0.000	0.000	0.000
12.C	0.000	0.000	0.000	0.000	0.000	0.000	0.000	0.000	0.000
13.O	0.000	0.000	0.000	0.000	0.000	0.000	0.000	0.000	0.000
14.C	0.000	0.000	0.000	0.000	0.000	0.000	0.000	0.000	0.000
15.C	0.000	0.000	0.000	0.000	0.000	0.000	0.000	0.000	0.000
16.O	0.000	0.000	0.000	0.000	0.000	0.000	0.000	0.000	0.000
17.C	0.000	0.000	0.000	0.000	0.000	0.000	0.000	0.000	0.000
18.C	0.000	0.000	0.000	0.000	0.000	0.000	0.000	0.000	0.000
19.O	0.000	0.000	0.000	0.000	0.000	0.000	0.000	0.000	0.000
20.C	0.000	0.000	0.000	0.000	0.000	0.000	0.000	0.000	0.000
21.C	0.000	0.000	0.000	0.000	0.000	0.000	0.000	0.000	0.000
22.C	0.000	0.000	0.000	0.000	0.000	0.000	0.000	0.000	0.000
23.C	0.000	0.000	0.000	0.000	0.000	0.000	0.000	0.000	0.000
24.C	0.000	0.000	0.000	0.000	0.000	0.000	0.000	0.000	0.000
25.C	0.000	0.000	0.000	0.000	0.000	0.000	0.000	0.000	0.000
26.C	0.000	0.000	0.000	0.000	0.000	0.000	0.000	0.000	0.000
27.C	0.000	0.000	0.000	0.000	0.000	0.000	0.000	0.000	0.000
28.C	0.000	0.000	0.000	0.000	0.000	0.000	0.000	0.000	0.000

29.C	0.000	0.000	0.000	0.000	0.000	0.000	0.000	0.000	0.000
30.C	0.000	0.000	0.000	0.000	0.000	0.000	0.000	0.000	0.000
31.O	0.000	0.000	0.000	0.000	0.000	0.000	0.000	0.000	0.000
32.C	0.000	0.000	0.000	0.000	0.000	0.000	0.000	0.000	0.000
33.C	0.000	0.000	0.000	0.000	0.000	0.000	0.000	0.000	0.000
34.C	0.000	0.000	0.000	0.000	0.000	0.000	0.000	0.000	0.000
35.C	0.000	0.000	0.000	0.000	0.000	0.000	0.000	0.000	0.000
36.C	0.000	0.000	0.000	0.000	0.000	0.000	0.000	0.000	0.000
37.C	0.000	0.000	0.000	0.000	0.000	0.000	0.000	0.000	0.000
38.C	0.000	0.000	0.000	0.000	0.000	0.000	0.000	0.000	0.000
39.C	0.000	0.000	0.000	0.000	0.000	0.000	0.000	0.000	0.000
40.H	0.000	0.000	0.000	0.000	0.000	0.000	0.000	0.000	0.000
41.H	0.000	0.000	0.000	0.000	0.000	0.000	0.000	0.000	0.000
42.H	0.000	0.000	0.000	0.000	0.000	0.000	0.000	0.000	0.000
43.H	0.000	0.000	0.000	0.000	0.000	0.000	0.000	0.000	0.000
44.H	0.000	0.000	0.000	0.000	0.000	0.000	0.000	0.000	0.000
45.H	0.000	0.000	0.000	0.000	0.000	0.000	0.000	0.000	0.000
46.H	0.000	0.000	0.000	0.000	0.000	0.000	0.000	0.000	0.000
47.H	0.000	0.000	0.000	0.000	0.000	0.000	0.000	0.000	0.000
48.H	0.000	0.000	0.000	0.000	0.000	0.000	0.000	0.000	0.000
49.H	0.000	0.000	0.000	0.000	0.000	0.000	0.000	0.000	0.000
50.H	0.000	0.000	0.000	0.000	0.000	0.000	0.000	0.000	0.000
51.H	0.000	0.000	0.000	0.000	0.000	0.000	0.000	0.000	0.000
52.H	0.000	0.000	0.000	0.000	0.000	0.000	0.000	0.000	0.000
53.H	0.000	0.000	0.000	0.000	0.000	0.000	0.000	0.000	0.000
54.H	0.000	0.000	0.000	0.000	0.000	0.000	0.000	0.000	0.000
55.H	0.000	0.000	0.000	0.000	0.000	0.000	0.000	0.000	0.000
56.H	0.000	0.000	0.000	0.000	0.000	0.000	0.000	0.000	0.000
57.H	0.000	0.000	0.000	0.000	0.000	0.000	0.000	0.000	0.000
58.H	0.000	0.000	0.000	0.000	0.000	0.000	0.000	0.000	0.000
59.H	0.000	0.000	0.000	0.000	0.000	0.000	0.000	0.000	0.000

60.H	0.000	0.000	0.000	0.000	0.000	0.000	0.000	0.000	0.000
61.H	0.000	0.000	0.000	0.000	0.000	0.000	0.000	0.000	0.000
62.H	0.000	0.000	0.000	0.000	0.000	0.000	0.000	0.000	0.000
63.H	0.000	0.000	0.000	0.000	0.000	0.000	0.000	0.000	0.000
64.H	0.000	0.000	0.000	0.000	0.000	0.000	0.000	0.000	0.000
65.H	0.000	0.000	0.000	0.000	0.000	0.000	0.000	0.000	0.000
66.H	0.975	0.011	-0.001	0.000	0.966	0.001	0.000	0.000	0.967
67.H	-0.044	0.003	0.001	0.013	-0.045	-0.004	0.002	0.002	-0.049
68.H	-0.047	0.008	0.001	0.021	-0.054	-0.006	0.005	0.004	-0.061
69.C	0.000	0.000	0.000	0.000	0.000	0.000	0.000	0.000	0.000
70.H	-0.021	0.003	-0.008	-0.037	-0.044	0.019	-0.008	-0.006	-0.022
71.H	-0.025	0.008	-0.008	-0.029	-0.055	0.018	-0.019	-0.011	-0.027
72.H	-0.023	0.010	-0.010	-0.032	-0.060	0.021	-0.011	-0.006	-0.034
73.H	-0.033	0.008	-0.005	-0.010	-0.056	0.010	-0.015	-0.008	-0.037
74.H	-0.028	-0.005	-0.003	-0.021	-0.029	0.008	-0.010	-0.007	-0.017
75.H	-0.033	-0.000	-0.002	-0.012	-0.039	0.006	-0.020	-0.012	-0.021
76.H	-0.036	-0.016	0.005	-0.004	-0.004	-0.007	-0.002	-0.003	-0.011
77.H	-0.031	-0.014	0.002	-0.015	-0.009	-0.000	-0.006	-0.006	-0.007
78.H	-0.036	-0.018	0.005	-0.004	-0.002	-0.008	-0.011	-0.008	-0.004
79.H	-0.046	-0.011	0.007	0.018	-0.016	-0.014	-0.010	-0.006	-0.023
80.H	-0.048	-0.005	0.006	0.019	-0.029	-0.011	-0.020	-0.011	-0.027
81.H	-0.046	-0.012	0.008	0.016	-0.013	-0.014	-0.019	-0.011	-0.015
82.H	-0.037	0.005	-0.002	-0.001	-0.049	0.004	-0.005	-0.002	-0.042
83.H	-0.031	0.009	-0.006	-0.013	-0.057	0.011	0.006	0.004	-0.050
84.H	-0.032	-0.008	0.000	-0.011	-0.021	0.001	0.002	0.000	-0.023
85.H	-0.041	0.000	0.001	0.005	-0.039	-0.002	-0.015	-0.008	-0.031
86.H	-0.047	-0.005	0.005	0.022	-0.027	-0.013	0.025	0.014	-0.054
87.O	0.000	0.000	0.000	0.000	0.000	0.000	0.000	0.000	0.000
88.H	-0.044	0.015	-0.002	0.016	-0.068	0.000	0.016	0.011	-0.076
89.H	-0.046	-0.014	0.008	0.019	-0.007	-0.017	0.013	0.007	-0.032
90.H	-0.049	-0.008	0.008	0.025	-0.021	-0.016	0.004	0.002	-0.038

91.H	-0.045	-0.007	0.005	0.017	-0.023	-0.012	0.013	0.007	-0.042
92.C	0.000	0.000	0.000	0.000	0.000	0.000	0.000	0.000	0.000
93.O	0.000	0.000	0.000	0.000	0.000	0.000	0.000	0.000	0.000
94.H	-0.032	0.010	-0.006	-0.007	-0.058	0.009	0.031	0.018	-0.068
95.H	-0.035	0.018	-0.007	-0.003	-0.074	0.011	0.023	0.015	-0.077
96.H	-0.039	0.015	-0.005	0.005	-0.067	0.005	0.030	0.019	-0.079

	211			212			213		
1.O	0.000	0.000	0.000	0.000	0.000	0.000	0.000	0.000	0.000
2.C	0.000	0.000	0.000	0.000	0.000	0.000	0.000	0.000	0.000
3.C	0.000	0.000	0.000	0.000	0.000	0.000	0.000	0.000	0.000
4.C	0.000	0.000	0.000	0.000	0.000	0.000	0.000	0.000	0.000
5.O	0.000	0.000	0.000	0.000	0.000	0.000	0.000	0.000	0.000
6.N	0.000	0.000	0.000	0.000	0.000	0.000	0.000	0.000	0.000
7.O	0.000	0.000	0.000	0.000	0.000	0.000	0.000	0.000	0.000
8.C	0.000	0.000	0.000	0.000	0.000	0.000	0.000	0.000	0.000
9.C	0.000	0.000	0.000	0.000	0.000	0.000	0.000	0.000	0.000
10.C	0.000	0.000	0.000	0.000	0.000	0.000	0.000	0.000	0.000
11.O	0.000	0.000	0.000	0.000	0.000	0.000	0.000	0.000	0.000
12.C	0.000	0.000	0.000	0.000	0.000	0.000	0.000	0.000	0.000
13.O	0.000	0.000	0.000	0.000	0.000	0.000	0.000	0.000	0.000
14.C	0.000	0.000	0.000	0.000	0.000	0.000	0.000	0.000	0.000
15.C	0.000	0.000	0.000	0.000	0.000	0.000	0.000	0.000	0.000
16.O	0.000	0.000	0.000	0.000	0.000	0.000	0.000	0.000	0.000
17.C	0.000	0.000	0.000	0.000	0.000	0.000	0.000	0.000	0.000
18.C	0.000	0.000	0.000	0.000	0.000	0.000	0.000	0.000	0.000
19.O	0.000	0.000	0.000	0.000	0.000	0.000	0.000	0.000	0.000
20.C	0.000	0.000	0.000	0.000	0.000	0.000	0.000	0.000	0.000
21.C	0.000	0.000	0.000	0.000	0.000	0.000	0.000	0.000	0.000

22.C	0.000	0.000	0.000	0.000	0.000	0.000	0.000	0.000	0.000
23.C	0.000	0.000	0.000	0.000	0.000	0.000	0.000	0.000	0.000
24.C	0.000	0.000	0.000	0.000	0.000	0.000	0.000	0.000	0.000
25.C	0.000	0.000	0.000	0.000	0.000	0.000	0.000	0.000	0.000
26.C	0.000	0.000	0.000	0.000	0.000	0.000	0.000	0.000	0.000
27.C	0.000	0.000	0.000	0.000	0.000	0.000	0.000	0.000	0.000
28.C	0.000	0.000	0.000	0.000	0.000	0.000	0.000	0.000	0.000
29.C	0.000	0.000	0.000	0.000	0.000	0.000	0.000	0.000	0.000
30.C	0.000	0.000	0.000	0.000	0.000	0.000	0.000	0.000	0.000
31.O	0.000	0.000	0.000	0.000	0.000	0.000	0.000	0.000	0.000
32.C	0.000	0.000	0.000	0.000	0.000	0.000	0.000	0.000	0.000
33.C	0.000	0.000	0.000	0.000	0.000	0.000	0.000	0.000	0.000
34.C	0.000	0.000	0.000	0.000	0.000	0.000	0.000	0.000	0.000
35.C	0.000	0.000	0.000	0.000	0.000	0.000	0.000	0.000	0.000
36.C	0.000	0.000	0.000	0.000	0.000	0.000	0.000	0.000	0.000
37.C	0.000	0.000	0.000	0.000	0.000	0.000	0.000	0.000	0.000
38.C	0.000	0.000	0.000	0.000	0.000	0.000	0.000	0.000	0.000
39.C	0.000	0.000	0.000	0.000	0.000	0.000	0.000	0.000	0.000
40.H	0.000	0.000	0.000	0.000	0.000	0.000	0.000	0.000	0.000
41.H	0.000	0.000	0.000	0.000	0.000	0.000	0.000	0.000	0.000
42.H	0.000	0.000	0.000	0.000	0.000	0.000	0.000	0.000	0.000
43.H	0.000	0.000	0.000	0.000	0.000	0.000	0.000	0.000	0.000
44.H	0.000	0.000	0.000	0.000	0.000	0.000	0.000	0.000	0.000
45.H	0.000	0.000	0.000	0.000	0.000	0.000	0.000	0.000	0.000
46.H	0.000	0.000	0.000	0.000	0.000	0.000	0.000	0.000	0.000
47.H	0.000	0.000	0.000	0.000	0.000	0.000	0.000	0.000	0.000
48.H	0.000	0.000	0.000	0.000	0.000	0.000	0.000	0.000	0.000
49.H	0.000	0.000	0.000	0.000	0.000	0.000	0.000	0.000	0.000
50.H	0.000	0.000	0.000	0.000	0.000	0.000	0.000	0.000	0.000
51.H	0.000	0.000	0.000	0.000	0.000	0.000	0.000	0.000	0.000
52.H	0.000	0.000	0.000	0.000	0.000	0.000	0.000	0.000	0.000

53.H	0.000	0.000	0.000	0.000	0.000	0.000	0.000	0.000	0.000
54.H	0.000	0.000	0.000	0.000	0.000	0.000	0.000	0.000	0.000
55.H	0.000	0.000	0.000	0.000	0.000	0.000	0.000	0.000	0.000
56.H	0.000	0.000	0.000	0.000	0.000	0.000	0.000	0.000	0.000
57.H	0.000	0.000	0.000	0.000	0.000	0.000	0.000	0.000	0.000
58.H	0.000	0.000	0.000	0.000	0.000	0.000	0.000	0.000	0.000
59.H	0.000	0.000	0.000	0.000	0.000	0.000	0.000	0.000	0.000
60.H	0.000	0.000	0.000	0.000	0.000	0.000	0.000	0.000	0.000
61.H	0.000	0.000	0.000	0.000	0.000	0.000	0.000	0.000	0.000
62.H	0.000	0.000	0.000	0.000	0.000	0.000	0.000	0.000	0.000
63.H	0.000	0.000	0.000	0.000	0.000	0.000	0.000	0.000	0.000
64.H	0.000	0.000	0.000	0.000	0.000	0.000	0.000	0.000	0.000
65.H	0.000	0.000	0.000	0.000	0.000	0.000	0.000	0.000	0.000
66.H	0.000	0.000	0.000	0.000	0.000	0.000	0.000	0.000	0.000
67.H	0.973	0.004	0.003	0.000	0.975	0.001	0.000	0.000	0.971
68.H	-0.052	0.010	0.005	0.007	-0.046	0.002	0.006	0.002	-0.059
69.C	0.000	0.000	0.000	0.000	0.000	0.000	0.000	0.000	0.000
70.H	-0.016	0.004	-0.010	-0.012	-0.039	-0.001	-0.010	-0.010	-0.013
71.H	-0.019	0.009	-0.009	-0.010	-0.040	-0.001	-0.012	-0.019	-0.017
72.H	-0.018	0.013	-0.009	-0.011	-0.044	0.000	-0.010	-0.014	-0.019
73.H	-0.030	0.010	-0.004	-0.004	-0.041	0.000	-0.007	-0.016	-0.031
74.H	-0.025	-0.005	-0.006	-0.007	-0.033	-0.002	-0.007	-0.007	-0.020
75.H	-0.028	0.000	-0.004	-0.004	-0.034	-0.001	-0.010	-0.017	-0.024
76.H	-0.037	-0.019	-0.000	-0.001	-0.028	-0.003	-0.001	0.004	-0.029
77.H	-0.029	-0.017	-0.004	-0.005	-0.028	-0.003	-0.005	-0.001	-0.020
78.H	-0.035	-0.021	-0.001	-0.001	-0.024	-0.003	-0.005	-0.003	-0.025
79.H	-0.048	-0.013	0.004	0.006	-0.030	-0.002	-0.001	-0.004	-0.042
80.H	-0.047	-0.006	0.003	0.006	-0.031	-0.001	-0.004	-0.014	-0.043
81.H	-0.045	-0.015	0.003	0.005	-0.026	-0.002	-0.004	-0.010	-0.038
82.H	-0.038	0.007	-0.001	-0.000	-0.042	0.001	-0.002	-0.006	-0.039
83.H	-0.033	0.012	-0.003	-0.004	-0.047	0.001	0.000	0.001	-0.038

84.H	-0.033	-0.009	-0.002	-0.004	-0.034	-0.001	-0.001	0.004	-0.029
85.H	-0.039	0.000	0.000	0.002	-0.036	-0.001	-0.005	-0.012	-0.037
86.H	-0.057	-0.005	0.007	0.008	-0.042	0.001	0.014	0.023	-0.062
87.O	0.000	0.000	0.000	0.000	0.000	0.000	0.000	0.000	0.000
88.H	-0.052	0.019	0.004	0.006	-0.054	0.004	0.009	0.009	-0.063
89.H	-0.053	-0.017	0.006	0.007	-0.033	-0.001	0.009	0.017	-0.050
90.H	-0.055	-0.009	0.007	0.009	-0.035	-0.000	0.006	0.007	-0.054
91.H	-0.051	-0.008	0.005	0.006	-0.038	0.000	0.008	0.014	-0.052
92.C	0.000	0.000	0.000	0.000	0.000	0.000	0.000	0.000	0.000
93.O	0.000	0.000	0.000	0.000	0.000	0.000	0.000	0.000	0.000
94.H	-0.041	0.014	0.000	-0.002	-0.054	0.003	0.010	0.021	-0.051
95.H	-0.042	0.023	0.000	-0.000	-0.058	0.004	0.008	0.013	-0.055
96.H	-0.048	0.019	0.003	0.003	-0.057	0.004	0.013	0.020	-0.061

	214			215			216		
	------------------------			------------------------			------------------------		
1.O	0.000	0.000	0.000	0.000	0.000	0.000	0.000	0.000	0.000
2.C	0.000	0.000	0.000	0.000	0.000	0.000	0.000	0.000	0.000
3.C	0.000	0.000	0.000	0.000	0.000	0.000	0.000	0.000	0.000
4.C	0.000	0.000	0.000	0.000	0.000	0.000	0.000	0.000	0.000
5.O	0.000	0.000	0.000	0.000	0.000	0.000	0.000	0.000	0.000
6.N	0.000	0.000	0.000	0.000	0.000	0.000	0.000	0.000	0.000
7.O	0.000	0.000	0.000	0.000	0.000	0.000	0.000	0.000	0.000
8.C	0.000	0.000	0.000	0.000	0.000	0.000	0.000	0.000	0.000
9.C	0.000	0.000	0.000	0.000	0.000	0.000	0.000	0.000	0.000
10.C	0.000	0.000	0.000	0.000	0.000	0.000	0.000	0.000	0.000
11.O	0.000	0.000	0.000	0.000	0.000	0.000	0.000	0.000	0.000
12.C	0.000	0.000	0.000	0.000	0.000	0.000	0.000	0.000	0.000
13.O	0.000	0.000	0.000	0.000	0.000	0.000	0.000	0.000	0.000
14.C	0.000	0.000	0.000	0.000	0.000	0.000	0.000	0.000	0.000

15.C	0.000	0.000	0.000	0.000	0.000	0.000	0.000	0.000	0.000
16.O	0.000	0.000	0.000	0.000	0.000	0.000	0.000	0.000	0.000
17.C	0.000	0.000	0.000	0.000	0.000	0.000	0.000	0.000	0.000
18.C	0.000	0.000	0.000	0.000	0.000	0.000	0.000	0.000	0.000
19.O	0.000	0.000	0.000	0.000	0.000	0.000	0.000	0.000	0.000
20.C	0.000	0.000	0.000	0.000	0.000	0.000	0.000	0.000	0.000
21.C	0.000	0.000	0.000	0.000	0.000	0.000	0.000	0.000	0.000
22.C	0.000	0.000	0.000	0.000	0.000	0.000	0.000	0.000	0.000
23.C	0.000	0.000	0.000	0.000	0.000	0.000	0.000	0.000	0.000
24.C	0.000	0.000	0.000	0.000	0.000	0.000	0.000	0.000	0.000
25.C	0.000	0.000	0.000	0.000	0.000	0.000	0.000	0.000	0.000
26.C	0.000	0.000	0.000	0.000	0.000	0.000	0.000	0.000	0.000
27.C	0.000	0.000	0.000	0.000	0.000	0.000	0.000	0.000	0.000
28.C	0.000	0.000	0.000	0.000	0.000	0.000	0.000	0.000	0.000
29.C	0.000	0.000	0.000	0.000	0.000	0.000	0.000	0.000	0.000
30.C	0.000	0.000	0.000	0.000	0.000	0.000	0.000	0.000	0.000
31.O	0.000	0.000	0.000	0.000	0.000	0.000	0.000	0.000	0.000
32.C	0.000	0.000	0.000	0.000	0.000	0.000	0.000	0.000	0.000
33.C	0.000	0.000	0.000	0.000	0.000	0.000	0.000	0.000	0.000
34.C	0.000	0.000	0.000	0.000	0.000	0.000	0.000	0.000	0.000
35.C	0.000	0.000	0.000	0.000	0.000	0.000	0.000	0.000	0.000
36.C	0.000	0.000	0.000	0.000	0.000	0.000	0.000	0.000	0.000
37.C	0.000	0.000	0.000	0.000	0.000	0.000	0.000	0.000	0.000
38.C	0.000	0.000	0.000	0.000	0.000	0.000	0.000	0.000	0.000
39.C	0.000	0.000	0.000	0.000	0.000	0.000	0.000	0.000	0.000
40.H	0.000	0.000	0.000	0.000	0.000	0.000	0.000	0.000	0.000
41.H	0.000	0.000	0.000	0.000	0.000	0.000	0.000	0.000	0.000
42.H	0.000	0.000	0.000	0.000	0.000	0.000	0.000	0.000	0.000
43.H	0.000	0.000	0.000	0.000	0.000	0.000	0.000	0.000	0.000
44.H	0.000	0.000	0.000	0.000	0.000	0.000	0.000	0.000	0.000
45.H	0.000	0.000	0.000	0.000	0.000	0.000	0.000	0.000	0.000

46.H	0.000	0.000	0.000	0.000	0.000	0.000	0.000	0.000	0.000
47.H	0.000	0.000	0.000	0.000	0.000	0.000	0.000	0.000	0.000
48.H	0.000	0.000	0.000	0.000	0.000	0.000	0.000	0.000	0.000
49.H	0.000	0.000	0.000	0.000	0.000	0.000	0.000	0.000	0.000
50.H	0.000	0.000	0.000	0.000	0.000	0.000	0.000	0.000	0.000
51.H	0.000	0.000	0.000	0.000	0.000	0.000	0.000	0.000	0.000
52.H	0.000	0.000	0.000	0.000	0.000	0.000	0.000	0.000	0.000
53.H	0.000	0.000	0.000	0.000	0.000	0.000	0.000	0.000	0.000
54.H	0.000	0.000	0.000	0.000	0.000	0.000	0.000	0.000	0.000
55.H	0.000	0.000	0.000	0.000	0.000	0.000	0.000	0.000	0.000
56.H	0.000	0.000	0.000	0.000	0.000	0.000	0.000	0.000	0.000
57.H	0.000	0.000	0.000	0.000	0.000	0.000	0.000	0.000	0.000
58.H	0.000	0.000	0.000	0.000	0.000	0.000	0.000	0.000	0.000
59.H	0.000	0.000	0.000	0.000	0.000	0.000	0.000	0.000	0.000
60.H	0.000	0.000	0.000	0.000	0.000	0.000	0.000	0.000	0.000
61.H	0.000	0.000	0.000	0.000	0.000	0.000	0.000	0.000	0.000
62.H	0.000	0.000	0.000	0.000	0.000	0.000	0.000	0.000	0.000
63.H	0.000	0.000	0.000	0.000	0.000	0.000	0.000	0.000	0.000
64.H	0.000	0.000	0.000	0.000	0.000	0.000	0.000	0.000	0.000
65.H	0.000	0.000	0.000	0.000	0.000	0.000	0.000	0.000	0.000
66.H	0.000	0.000	0.000	0.000	0.000	0.000	0.000	0.000	0.000
67.H	0.000	0.000	0.000	0.000	0.000	0.000	0.000	0.000	0.000
68.H	0.964	0.017	0.010	0.000	0.967	0.004	0.000	0.000	0.957
69.C	0.000	0.000	0.000	0.000	0.000	0.000	0.000	0.000	0.000
70.H	-0.002	0.006	-0.016	-0.029	-0.041	-0.001	-0.017	-0.016	0.001
71.H	-0.006	0.016	-0.014	-0.023	-0.044	-0.000	-0.025	-0.032	-0.006
72.H	-0.006	0.021	-0.013	-0.025	-0.053	0.002	-0.018	-0.022	-0.012
73.H	-0.025	0.017	-0.006	-0.008	-0.048	0.001	-0.016	-0.025	-0.030
74.H	-0.017	-0.009	-0.010	-0.016	-0.028	-0.003	-0.014	-0.013	-0.006
75.H	-0.022	0.000	-0.008	-0.009	-0.031	-0.003	-0.021	-0.028	-0.014
76.H	-0.037	-0.032	-0.003	-0.002	-0.013	-0.006	-0.002	0.005	-0.017

77.H	-0.024	-0.027	-0.008	-0.011	-0.015	-0.006	-0.009	-0.003	-0.004
78.H	-0.034	-0.035	-0.004	-0.002	-0.007	-0.008	-0.010	-0.007	-0.009
79.H	-0.055	-0.021	0.005	0.015	-0.019	-0.005	-0.004	-0.008	-0.041
80.H	-0.053	-0.009	0.004	0.016	-0.024	-0.003	-0.013	-0.024	-0.043
81.H	-0.050	-0.025	0.003	0.014	-0.012	-0.006	-0.012	-0.019	-0.031
82.H	-0.039	0.011	-0.001	0.000	-0.048	0.002	-0.004	-0.009	-0.045
83.H	-0.032	0.019	-0.003	-0.009	-0.060	0.004	0.003	0.004	-0.045
84.H	-0.032	-0.015	-0.004	-0.008	-0.029	-0.003	-0.000	0.006	-0.022
85.H	-0.041	0.001	-0.000	0.005	-0.034	-0.001	-0.012	-0.021	-0.037
86.H	-0.074	-0.007	0.013	0.020	-0.048	0.002	0.029	0.038	-0.081
87.O	0.000	0.000	0.000	0.000	0.000	0.000	0.000	0.000	0.000
88.H	-0.064	0.032	0.011	0.015	-0.076	0.008	0.020	0.017	-0.092
89.H	-0.066	-0.027	0.009	0.017	-0.025	-0.003	0.018	0.027	-0.056
90.H	-0.068	-0.015	0.010	0.022	-0.031	-0.002	0.011	0.011	-0.064
91.H	-0.063	-0.012	0.009	0.015	-0.038	-0.000	0.017	0.022	-0.062
92.C	0.000	0.000	0.000	0.000	0.000	0.000	0.000	0.000	0.000
93.O	0.000	0.000	0.000	0.000	0.000	0.000	0.000	0.000	0.000
94.H	-0.047	0.023	0.003	-0.003	-0.076	0.008	0.027	0.037	-0.071
95.H	-0.049	0.038	0.004	-0.000	-0.085	0.010	0.022	0.024	-0.080
96.H	-0.059	0.032	0.009	0.007	-0.083	0.010	0.030	0.036	-0.090

	217			218			219		
1.O	0.000	0.000	0.000	0.000	0.000	0.000	0.000	0.000	0.000
2.C	0.000	0.000	0.000	0.000	0.000	0.000	0.000	0.000	0.000
3.C	0.000	0.000	0.000	0.000	0.000	0.000	0.000	0.000	0.000
4.C	0.000	0.000	0.000	0.000	0.000	0.000	0.000	0.000	0.000
5.O	0.000	0.000	0.000	0.000	0.000	0.000	0.000	0.000	0.000
6.N	0.000	0.000	0.000	0.000	0.000	0.000	0.000	0.000	0.000
7.O	0.000	0.000	0.000	0.000	0.000	0.000	0.000	0.000	0.000

8.C	0.000	0.000	0.000	0.000	0.000	0.000	0.000	0.000	0.000
9.C	0.000	0.000	0.000	0.000	0.000	0.000	0.000	0.000	0.000
10.C	0.000	0.000	0.000	0.000	0.000	0.000	0.000	0.000	0.000
11.O	0.000	0.000	0.000	0.000	0.000	0.000	0.000	0.000	0.000
12.C	0.000	0.000	0.000	0.000	0.000	0.000	0.000	0.000	0.000
13.O	0.000	0.000	0.000	0.000	0.000	0.000	0.000	0.000	0.000
14.C	0.000	0.000	0.000	0.000	0.000	0.000	0.000	0.000	0.000
15.C	0.000	0.000	0.000	0.000	0.000	0.000	0.000	0.000	0.000
16.O	0.000	0.000	0.000	0.000	0.000	0.000	0.000	0.000	0.000
17.C	0.000	0.000	0.000	0.000	0.000	0.000	0.000	0.000	0.000
18.C	0.000	0.000	0.000	0.000	0.000	0.000	0.000	0.000	0.000
19.O	0.000	0.000	0.000	0.000	0.000	0.000	0.000	0.000	0.000
20.C	0.000	0.000	0.000	0.000	0.000	0.000	0.000	0.000	0.000
21.C	0.000	0.000	0.000	0.000	0.000	0.000	0.000	0.000	0.000
22.C	0.000	0.000	0.000	0.000	0.000	0.000	0.000	0.000	0.000
23.C	0.000	0.000	0.000	0.000	0.000	0.000	0.000	0.000	0.000
24.C	0.000	0.000	0.000	0.000	0.000	0.000	0.000	0.000	0.000
25.C	0.000	0.000	0.000	0.000	0.000	0.000	0.000	0.000	0.000
26.C	0.000	0.000	0.000	0.000	0.000	0.000	0.000	0.000	0.000
27.C	0.000	0.000	0.000	0.000	0.000	0.000	0.000	0.000	0.000
28.C	0.000	0.000	0.000	0.000	0.000	0.000	0.000	0.000	0.000
29.C	0.000	0.000	0.000	0.000	0.000	0.000	0.000	0.000	0.000
30.C	0.000	0.000	0.000	0.000	0.000	0.000	0.000	0.000	0.000
31.O	0.000	0.000	0.000	0.000	0.000	0.000	0.000	0.000	0.000
32.C	0.000	0.000	0.000	0.000	0.000	0.000	0.000	0.000	0.000
33.C	0.000	0.000	0.000	0.000	0.000	0.000	0.000	0.000	0.000
34.C	0.000	0.000	0.000	0.000	0.000	0.000	0.000	0.000	0.000
35.C	0.000	0.000	0.000	0.000	0.000	0.000	0.000	0.000	0.000
36.C	0.000	0.000	0.000	0.000	0.000	0.000	0.000	0.000	0.000
37.C	0.000	0.000	0.000	0.000	0.000	0.000	0.000	0.000	0.000
38.C	0.000	0.000	0.000	0.000	0.000	0.000	0.000	0.000	0.000

39.C	0.000	0.000	0.000	0.000	0.000	0.000	0.000	0.000	0.000
40.H	0.000	0.000	0.000	0.000	0.000	0.000	0.000	0.000	0.000
41.H	0.000	0.000	0.000	0.000	0.000	0.000	0.000	0.000	0.000
42.H	0.000	0.000	0.000	0.000	0.000	0.000	0.000	0.000	0.000
43.H	0.000	0.000	0.000	0.000	0.000	0.000	0.000	0.000	0.000
44.H	0.000	0.000	0.000	0.000	0.000	0.000	0.000	0.000	0.000
45.H	0.000	0.000	0.000	0.000	0.000	0.000	0.000	0.000	0.000
46.H	0.000	0.000	0.000	0.000	0.000	0.000	0.000	0.000	0.000
47.H	0.000	0.000	0.000	0.000	0.000	0.000	0.000	0.000	0.000
48.H	0.000	0.000	0.000	0.000	0.000	0.000	0.000	0.000	0.000
49.H	0.000	0.000	0.000	0.000	0.000	0.000	0.000	0.000	0.000
50.H	0.000	0.000	0.000	0.000	0.000	0.000	0.000	0.000	0.000
51.H	0.000	0.000	0.000	0.000	0.000	0.000	0.000	0.000	0.000
52.H	0.000	0.000	0.000	0.000	0.000	0.000	0.000	0.000	0.000
53.H	0.000	0.000	0.000	0.000	0.000	0.000	0.000	0.000	0.000
54.H	0.000	0.000	0.000	0.000	0.000	0.000	0.000	0.000	0.000
55.H	0.000	0.000	0.000	0.000	0.000	0.000	0.000	0.000	0.000
56.H	0.000	0.000	0.000	0.000	0.000	0.000	0.000	0.000	0.000
57.H	0.000	0.000	0.000	0.000	0.000	0.000	0.000	0.000	0.000
58.H	0.000	0.000	0.000	0.000	0.000	0.000	0.000	0.000	0.000
59.H	0.000	0.000	0.000	0.000	0.000	0.000	0.000	0.000	0.000
60.H	0.000	0.000	0.000	0.000	0.000	0.000	0.000	0.000	0.000
61.H	0.000	0.000	0.000	0.000	0.000	0.000	0.000	0.000	0.000
62.H	0.000	0.000	0.000	0.000	0.000	0.000	0.000	0.000	0.000
63.H	0.000	0.000	0.000	0.000	0.000	0.000	0.000	0.000	0.000
64.H	0.000	0.000	0.000	0.000	0.000	0.000	0.000	0.000	0.000
65.H	0.000	0.000	0.000	0.000	0.000	0.000	0.000	0.000	0.000
66.H	0.000	0.000	0.000	0.000	0.000	0.000	0.000	0.000	0.000
67.H	0.000	0.000	0.000	0.000	0.000	0.000	0.000	0.000	0.000
68.H	0.000	0.000	0.000	0.000	0.000	0.000	0.000	0.000	0.000
69.C	0.000	0.000	0.000	0.000	0.000	0.000	0.000	0.000	0.000

70.H	0.942	-0.012	0.027	0.000	0.969	0.031	0.000	0.000	0.934
71.H	-0.105	-0.029	0.024	-0.010	-0.067	0.028	0.022	0.068	-0.122
72.H	-0.104	-0.039	0.023	-0.011	-0.062	0.029	0.026	0.053	-0.125
73.H	-0.070	-0.031	0.009	-0.003	-0.063	0.013	0.007	0.057	-0.079
74.H	-0.085	0.014	0.017	-0.007	-0.049	0.017	0.017	0.021	-0.091
75.H	-0.077	-0.002	0.013	-0.004	-0.061	0.013	0.008	0.055	-0.080
76.H	-0.049	0.055	0.005	-0.001	-0.032	-0.001	0.003	-0.023	-0.040
77.H	-0.072	0.046	0.014	-0.005	-0.039	0.009	0.012	-0.005	-0.069
78.H	-0.055	0.060	0.007	-0.001	-0.039	0.000	0.002	-0.002	-0.044
79.H	-0.018	0.035	-0.009	0.007	-0.043	-0.015	-0.016	0.006	-0.002
80.H	-0.023	0.015	-0.008	0.007	-0.056	-0.013	-0.019	0.042	-0.008
81.H	-0.027	0.042	-0.005	0.006	-0.049	-0.013	-0.017	0.025	-0.008
82.H	-0.047	-0.021	0.000	-0.000	-0.051	0.002	0.000	0.024	-0.050
83.H	-0.058	-0.035	0.004	-0.005	-0.045	0.010	0.012	0.004	-0.071
84.H	-0.059	0.025	0.007	-0.004	-0.036	0.006	0.010	-0.017	-0.059
85.H	-0.043	-0.003	-0.000	0.002	-0.056	-0.002	-0.006	0.041	-0.040
86.H	0.016	0.012	-0.024	0.008	-0.017	-0.025	-0.016	-0.072	0.026
87.O	0.000	0.000	0.000	0.000	0.000	0.000	0.000	0.000	0.000
88.H	-0.001	-0.057	-0.020	0.006	-0.039	-0.014	-0.012	-0.014	-0.006
89.H	0.001	0.047	-0.016	0.006	-0.019	-0.022	-0.014	-0.061	0.018
90.H	0.005	0.025	-0.019	0.009	-0.032	-0.024	-0.021	-0.026	0.022
91.H	-0.003	0.020	-0.016	0.006	-0.025	-0.019	-0.013	-0.046	0.008
92.C	0.000	0.000	0.000	0.000	0.000	0.000	0.000	0.000	0.000
93.O	0.000	0.000	0.000	0.000	0.000	0.000	0.000	0.000	0.000
94.H	-0.031	-0.042	-0.007	-0.003	-0.024	0.000	0.010	-0.056	-0.046
95.H	-0.029	-0.068	-0.009	-0.002	-0.036	0.000	0.006	-0.024	-0.045
96.H	-0.009	-0.056	-0.017	0.001	-0.027	-0.009	-0.001	-0.049	-0.021

220 221 222

------------------------ ------------------------ ------------------------

1.O	0.000	0.000	0.000	0.000	0.000	0.000	0.000	0.000	0.000
2.C	0.000	0.000	0.000	0.000	0.000	0.000	0.000	0.000	0.000
3.C	0.000	0.000	0.000	0.000	0.000	0.000	0.000	0.000	0.000
4.C	0.000	0.000	0.000	0.000	0.000	0.000	0.000	0.000	0.000
5.O	0.000	0.000	0.000	0.000	0.000	0.000	0.000	0.000	0.000
6.N	0.000	0.000	0.000	0.000	0.000	0.000	0.000	0.000	0.000
7.O	0.000	0.000	0.000	0.000	0.000	0.000	0.000	0.000	0.000
8.C	0.000	0.000	0.000	0.000	0.000	0.000	0.000	0.000	0.000
9.C	0.000	0.000	0.000	0.000	0.000	0.000	0.000	0.000	0.000
10.C	0.000	0.000	0.000	0.000	0.000	0.000	0.000	0.000	0.000
11.O	0.000	0.000	0.000	0.000	0.000	0.000	0.000	0.000	0.000
12.C	0.000	0.000	0.000	0.000	0.000	0.000	0.000	0.000	0.000
13.O	0.000	0.000	0.000	0.000	0.000	0.000	0.000	0.000	0.000
14.C	0.000	0.000	0.000	0.000	0.000	0.000	0.000	0.000	0.000
15.C	0.000	0.000	0.000	0.000	0.000	0.000	0.000	0.000	0.000
16.O	0.000	0.000	0.000	0.000	0.000	0.000	0.000	0.000	0.000
17.C	0.000	0.000	0.000	0.000	0.000	0.000	0.000	0.000	0.000
18.C	0.000	0.000	0.000	0.000	0.000	0.000	0.000	0.000	0.000
19.O	0.000	0.000	0.000	0.000	0.000	0.000	0.000	0.000	0.000
20.C	0.000	0.000	0.000	0.000	0.000	0.000	0.000	0.000	0.000
21.C	0.000	0.000	0.000	0.000	0.000	0.000	0.000	0.000	0.000
22.C	0.000	0.000	0.000	0.000	0.000	0.000	0.000	0.000	0.000
23.C	0.000	0.000	0.000	0.000	0.000	0.000	0.000	0.000	0.000
24.C	0.000	0.000	0.000	0.000	0.000	0.000	0.000	0.000	0.000
25.C	0.000	0.000	0.000	0.000	0.000	0.000	0.000	0.000	0.000
26.C	0.000	0.000	0.000	0.000	0.000	0.000	0.000	0.000	0.000
27.C	0.000	0.000	0.000	0.000	0.000	0.000	0.000	0.000	0.000
28.C	0.000	0.000	0.000	0.000	0.000	0.000	0.000	0.000	0.000
29.C	0.000	0.000	0.000	0.000	0.000	0.000	0.000	0.000	0.000
30.C	0.000	0.000	0.000	0.000	0.000	0.000	0.000	0.000	0.000
31.O	0.000	0.000	0.000	0.000	0.000	0.000	0.000	0.000	0.000

32.C	0.000	0.000	0.000	0.000	0.000	0.000	0.000	0.000	0.000
33.C	0.000	0.000	0.000	0.000	0.000	0.000	0.000	0.000	0.000
34.C	0.000	0.000	0.000	0.000	0.000	0.000	0.000	0.000	0.000
35.C	0.000	0.000	0.000	0.000	0.000	0.000	0.000	0.000	0.000
36.C	0.000	0.000	0.000	0.000	0.000	0.000	0.000	0.000	0.000
37.C	0.000	0.000	0.000	0.000	0.000	0.000	0.000	0.000	0.000
38.C	0.000	0.000	0.000	0.000	0.000	0.000	0.000	0.000	0.000
39.C	0.000	0.000	0.000	0.000	0.000	0.000	0.000	0.000	0.000
40.H	0.000	0.000	0.000	0.000	0.000	0.000	0.000	0.000	0.000
41.H	0.000	0.000	0.000	0.000	0.000	0.000	0.000	0.000	0.000
42.H	0.000	0.000	0.000	0.000	0.000	0.000	0.000	0.000	0.000
43.H	0.000	0.000	0.000	0.000	0.000	0.000	0.000	0.000	0.000
44.H	0.000	0.000	0.000	0.000	0.000	0.000	0.000	0.000	0.000
45.H	0.000	0.000	0.000	0.000	0.000	0.000	0.000	0.000	0.000
46.H	0.000	0.000	0.000	0.000	0.000	0.000	0.000	0.000	0.000
47.H	0.000	0.000	0.000	0.000	0.000	0.000	0.000	0.000	0.000
48.H	0.000	0.000	0.000	0.000	0.000	0.000	0.000	0.000	0.000
49.H	0.000	0.000	0.000	0.000	0.000	0.000	0.000	0.000	0.000
50.H	0.000	0.000	0.000	0.000	0.000	0.000	0.000	0.000	0.000
51.H	0.000	0.000	0.000	0.000	0.000	0.000	0.000	0.000	0.000
52.H	0.000	0.000	0.000	0.000	0.000	0.000	0.000	0.000	0.000
53.H	0.000	0.000	0.000	0.000	0.000	0.000	0.000	0.000	0.000
54.H	0.000	0.000	0.000	0.000	0.000	0.000	0.000	0.000	0.000
55.H	0.000	0.000	0.000	0.000	0.000	0.000	0.000	0.000	0.000
56.H	0.000	0.000	0.000	0.000	0.000	0.000	0.000	0.000	0.000
57.H	0.000	0.000	0.000	0.000	0.000	0.000	0.000	0.000	0.000
58.H	0.000	0.000	0.000	0.000	0.000	0.000	0.000	0.000	0.000
59.H	0.000	0.000	0.000	0.000	0.000	0.000	0.000	0.000	0.000
60.H	0.000	0.000	0.000	0.000	0.000	0.000	0.000	0.000	0.000
61.H	0.000	0.000	0.000	0.000	0.000	0.000	0.000	0.000	0.000
62.H	0.000	0.000	0.000	0.000	0.000	0.000	0.000	0.000	0.000

63.H	0.000	0.000	0.000	0.000	0.000	0.000	0.000	0.000	0.000
64.H	0.000	0.000	0.000	0.000	0.000	0.000	0.000	0.000	0.000
65.H	0.000	0.000	0.000	0.000	0.000	0.000	0.000	0.000	0.000
66.H	0.000	0.000	0.000	0.000	0.000	0.000	0.000	0.000	0.000
67.H	0.000	0.000	0.000	0.000	0.000	0.000	0.000	0.000	0.000
68.H	0.000	0.000	0.000	0.000	0.000	0.000	0.000	0.000	0.000
69.C	0.000	0.000	0.000	0.000	0.000	0.000	0.000	0.000	0.000
70.H	0.000	0.000	0.000	0.000	0.000	0.000	0.000	0.000	0.000
71.H	0.937	-0.027	0.020	0.000	0.944	0.071	0.000	0.000	0.930
72.H	-0.114	-0.036	0.013	-0.034	-0.096	0.074	0.031	0.064	-0.143
73.H	-0.086	-0.029	0.004	-0.012	-0.098	0.034	0.007	0.068	-0.091
74.H	-0.096	0.012	0.026	-0.023	-0.061	0.044	0.020	0.026	-0.100
75.H	-0.096	-0.003	0.020	-0.014	-0.091	0.035	0.007	0.064	-0.089
76.H	-0.055	0.050	0.025	-0.005	-0.017	-0.000	0.005	-0.025	-0.041
77.H	-0.081	0.042	0.032	-0.017	-0.033	0.026	0.015	-0.005	-0.074
78.H	-0.068	0.054	0.032	-0.005	-0.033	0.003	0.002	-0.003	-0.044
79.H	-0.033	0.032	0.008	0.017	-0.044	-0.034	-0.020	0.008	0.001
80.H	-0.045	0.013	0.005	0.018	-0.077	-0.028	-0.025	0.049	-0.007
81.H	-0.048	0.037	0.017	0.015	-0.058	-0.028	-0.022	0.028	-0.006
82.H	-0.055	-0.020	-0.005	-0.002	-0.069	0.008	0.001	0.031	-0.059
83.H	-0.057	-0.033	-0.009	-0.014	-0.056	0.027	0.016	0.010	-0.084
84.H	-0.061	0.022	0.016	-0.013	-0.028	0.017	0.013	-0.016	-0.065
85.H	-0.061	-0.004	0.005	0.004	-0.080	-0.001	-0.009	0.048	-0.044
86.H	0.027	0.011	-0.026	0.021	0.015	-0.058	-0.014	-0.076	0.026
87.O	0.000	0.000	0.000	0.000	0.000	0.000	0.000	0.000	0.000
88.H	0.005	-0.052	-0.043	0.015	-0.045	-0.031	-0.011	-0.008	-0.014
89.H	0.005	0.043	-0.003	0.018	0.013	-0.051	-0.014	-0.066	0.021
90.H	0.001	0.022	-0.010	0.024	-0.020	-0.055	-0.024	-0.026	0.025
91.H	0.000	0.019	-0.011	0.016	-0.005	-0.042	-0.012	-0.048	0.008
92.C	0.000	0.000	0.000	0.000	0.000	0.000	0.000	0.000	0.000
93.O	0.000	0.000	0.000	0.000	0.000	0.000	0.000	0.000	0.000

94.H	-0.011	-0.038	-0.031	-0.009	-0.007	0.004	0.018	-0.056	-0.058
95.H	-0.015	-0.062	-0.039	-0.004	-0.039	0.004	0.011	-0.019	-0.059
96.H	0.008	-0.052	-0.045	0.004	-0.016	-0.018	0.005	-0.047	-0.032

	223			224			225		
1.O	0.000	0.000	0.000	0.000	0.000	0.000	0.000	0.000	0.000
2.C	0.000	0.000	0.000	0.000	0.000	0.000	0.000	0.000	0.000
3.C	0.000	0.000	0.000	0.000	0.000	0.000	0.000	0.000	0.000
4.C	0.000	0.000	0.000	0.000	0.000	0.000	0.000	0.000	0.000
5.O	0.000	0.000	0.000	0.000	0.000	0.000	0.000	0.000	0.000
6.N	0.000	0.000	0.000	0.000	0.000	0.000	0.000	0.000	0.000
7.O	0.000	0.000	0.000	0.000	0.000	0.000	0.000	0.000	0.000
8.C	0.000	0.000	0.000	0.000	0.000	0.000	0.000	0.000	0.000
9.C	0.000	0.000	0.000	0.000	0.000	0.000	0.000	0.000	0.000
10.C	0.000	0.000	0.000	0.000	0.000	0.000	0.000	0.000	0.000
11.O	0.000	0.000	0.000	0.000	0.000	0.000	0.000	0.000	0.000
12.C	0.000	0.000	0.000	0.000	0.000	0.000	0.000	0.000	0.000
13.O	0.000	0.000	0.000	0.000	0.000	0.000	0.000	0.000	0.000
14.C	0.000	0.000	0.000	0.000	0.000	0.000	0.000	0.000	0.000
15.C	0.000	0.000	0.000	0.000	0.000	0.000	0.000	0.000	0.000
16.O	0.000	0.000	0.000	0.000	0.000	0.000	0.000	0.000	0.000
17.C	0.000	0.000	0.000	0.000	0.000	0.000	0.000	0.000	0.000
18.C	0.000	0.000	0.000	0.000	0.000	0.000	0.000	0.000	0.000
19.O	0.000	0.000	0.000	0.000	0.000	0.000	0.000	0.000	0.000
20.C	0.000	0.000	0.000	0.000	0.000	0.000	0.000	0.000	0.000
21.C	0.000	0.000	0.000	0.000	0.000	0.000	0.000	0.000	0.000
22.C	0.000	0.000	0.000	0.000	0.000	0.000	0.000	0.000	0.000
23.C	0.000	0.000	0.000	0.000	0.000	0.000	0.000	0.000	0.000
24.C	0.000	0.000	0.000	0.000	0.000	0.000	0.000	0.000	0.000

25.C	0.000	0.000	0.000	0.000	0.000	0.000	0.000	0.000	0.000
26.C	0.000	0.000	0.000	0.000	0.000	0.000	0.000	0.000	0.000
27.C	0.000	0.000	0.000	0.000	0.000	0.000	0.000	0.000	0.000
28.C	0.000	0.000	0.000	0.000	0.000	0.000	0.000	0.000	0.000
29.C	0.000	0.000	0.000	0.000	0.000	0.000	0.000	0.000	0.000
30.C	0.000	0.000	0.000	0.000	0.000	0.000	0.000	0.000	0.000
31.O	0.000	0.000	0.000	0.000	0.000	0.000	0.000	0.000	0.000
32.C	0.000	0.000	0.000	0.000	0.000	0.000	0.000	0.000	0.000
33.C	0.000	0.000	0.000	0.000	0.000	0.000	0.000	0.000	0.000
34.C	0.000	0.000	0.000	0.000	0.000	0.000	0.000	0.000	0.000
35.C	0.000	0.000	0.000	0.000	0.000	0.000	0.000	0.000	0.000
36.C	0.000	0.000	0.000	0.000	0.000	0.000	0.000	0.000	0.000
37.C	0.000	0.000	0.000	0.000	0.000	0.000	0.000	0.000	0.000
38.C	0.000	0.000	0.000	0.000	0.000	0.000	0.000	0.000	0.000
39.C	0.000	0.000	0.000	0.000	0.000	0.000	0.000	0.000	0.000
40.H	0.000	0.000	0.000	0.000	0.000	0.000	0.000	0.000	0.000
41.H	0.000	0.000	0.000	0.000	0.000	0.000	0.000	0.000	0.000
42.H	0.000	0.000	0.000	0.000	0.000	0.000	0.000	0.000	0.000
43.H	0.000	0.000	0.000	0.000	0.000	0.000	0.000	0.000	0.000
44.H	0.000	0.000	0.000	0.000	0.000	0.000	0.000	0.000	0.000
45.H	0.000	0.000	0.000	0.000	0.000	0.000	0.000	0.000	0.000
46.H	0.000	0.000	0.000	0.000	0.000	0.000	0.000	0.000	0.000
47.H	0.000	0.000	0.000	0.000	0.000	0.000	0.000	0.000	0.000
48.H	0.000	0.000	0.000	0.000	0.000	0.000	0.000	0.000	0.000
49.H	0.000	0.000	0.000	0.000	0.000	0.000	0.000	0.000	0.000
50.H	0.000	0.000	0.000	0.000	0.000	0.000	0.000	0.000	0.000
51.H	0.000	0.000	0.000	0.000	0.000	0.000	0.000	0.000	0.000
52.H	0.000	0.000	0.000	0.000	0.000	0.000	0.000	0.000	0.000
53.H	0.000	0.000	0.000	0.000	0.000	0.000	0.000	0.000	0.000
54.H	0.000	0.000	0.000	0.000	0.000	0.000	0.000	0.000	0.000
55.H	0.000	0.000	0.000	0.000	0.000	0.000	0.000	0.000	0.000

56.H	0.000	0.000	0.000	0.000	0.000	0.000	0.000	0.000	0.000
57.H	0.000	0.000	0.000	0.000	0.000	0.000	0.000	0.000	0.000
58.H	0.000	0.000	0.000	0.000	0.000	0.000	0.000	0.000	0.000
59.H	0.000	0.000	0.000	0.000	0.000	0.000	0.000	0.000	0.000
60.H	0.000	0.000	0.000	0.000	0.000	0.000	0.000	0.000	0.000
61.H	0.000	0.000	0.000	0.000	0.000	0.000	0.000	0.000	0.000
62.H	0.000	0.000	0.000	0.000	0.000	0.000	0.000	0.000	0.000
63.H	0.000	0.000	0.000	0.000	0.000	0.000	0.000	0.000	0.000
64.H	0.000	0.000	0.000	0.000	0.000	0.000	0.000	0.000	0.000
65.H	0.000	0.000	0.000	0.000	0.000	0.000	0.000	0.000	0.000
66.H	0.000	0.000	0.000	0.000	0.000	0.000	0.000	0.000	0.000
67.H	0.000	0.000	0.000	0.000	0.000	0.000	0.000	0.000	0.000
68.H	0.000	0.000	0.000	0.000	0.000	0.000	0.000	0.000	0.000
69.C	0.000	0.000	0.000	0.000	0.000	0.000	0.000	0.000	0.000
70.H	0.000	0.000	0.000	0.000	0.000	0.000	0.000	0.000	0.000
71.H	0.000	0.000	0.000	0.000	0.000	0.000	0.000	0.000	0.000
72.H	0.934	-0.050	0.032	0.000	0.943	0.077	0.000	0.000	0.907
73.H	-0.090	-0.042	0.015	-0.023	-0.108	0.037	0.006	0.094	-0.116
74.H	-0.106	0.011	0.030	-0.039	-0.057	0.046	0.027	0.035	-0.120
75.H	-0.099	-0.010	0.024	-0.026	-0.089	0.037	0.004	0.084	-0.107
76.H	-0.062	0.060	0.016	-0.011	-0.003	0.000	0.008	-0.035	-0.036
77.H	-0.090	0.049	0.028	-0.030	-0.020	0.026	0.021	-0.009	-0.080
78.H	-0.071	0.064	0.021	-0.012	-0.014	0.003	-0.000	-0.008	-0.037
79.H	-0.028	0.036	-0.005	0.022	-0.034	-0.032	-0.033	0.009	0.016
80.H	-0.036	0.010	-0.004	0.024	-0.071	-0.026	-0.044	0.063	0.003
81.H	-0.040	0.042	0.002	0.019	-0.043	-0.027	-0.038	0.033	0.011
82.H	-0.060	-0.028	0.001	-0.007	-0.080	0.012	0.001	0.047	-0.075
83.H	-0.070	-0.042	0.005	-0.024	-0.075	0.031	0.028	0.022	-0.113
84.H	-0.072	0.027	0.016	-0.022	-0.025	0.019	0.022	-0.020	-0.075
85.H	-0.059	-0.010	0.004	0.002	-0.081	0.002	-0.018	0.065	-0.050
86.H	0.021	0.017	-0.032	0.033	0.005	-0.054	-0.009	-0.093	0.036

87.O	0.000	0.000	0.000	0.000	0.000	0.000	0.000	0.000	0.000
88.H	-0.001	-0.064	-0.033	0.023	-0.075	-0.024	-0.008	0.002	-0.029
89.H	0.001	0.055	-0.016	0.026	0.018	-0.049	-0.014	-0.086	0.040
90.H	0.003	0.027	-0.021	0.036	-0.019	-0.052	-0.032	-0.032	0.041
91.H	-0.004	0.025	-0.018	0.023	-0.008	-0.039	-0.011	-0.059	0.017
92.C	0.000	0.000	0.000	0.000	0.000	0.000	0.000	0.000	0.000
93.O	0.000	0.000	0.000	0.000	0.000	0.000	0.000	0.000	0.000
94.H	-0.032	-0.044	-0.015	-0.013	-0.037	0.011	0.041	-0.059	-0.086
95.H	-0.031	-0.076	-0.020	-0.007	-0.075	0.011	0.028	-0.009	-0.092
96.H	-0.007	-0.060	-0.029	0.007	-0.051	-0.010	0.021	-0.046	-0.055

	226			227			228		
1.O	0.000	0.000	0.000	0.000	0.000	0.000	0.000	0.000	0.000
2.C	0.000	0.000	0.000	0.000	0.000	0.000	0.000	0.000	0.000
3.C	0.000	0.000	0.000	0.000	0.000	0.000	0.000	0.000	0.000
4.C	0.000	0.000	0.000	0.000	0.000	0.000	0.000	0.000	0.000
5.O	0.000	0.000	0.000	0.000	0.000	0.000	0.000	0.000	0.000
6.N	0.000	0.000	0.000	0.000	0.000	0.000	0.000	0.000	0.000
7.O	0.000	0.000	0.000	0.000	0.000	0.000	0.000	0.000	0.000
8.C	0.000	0.000	0.000	0.000	0.000	0.000	0.000	0.000	0.000
9.C	0.000	0.000	0.000	0.000	0.000	0.000	0.000	0.000	0.000
10.C	0.000	0.000	0.000	0.000	0.000	0.000	0.000	0.000	0.000
11.O	0.000	0.000	0.000	0.000	0.000	0.000	0.000	0.000	0.000
12.C	0.000	0.000	0.000	0.000	0.000	0.000	0.000	0.000	0.000
13.O	0.000	0.000	0.000	0.000	0.000	0.000	0.000	0.000	0.000
14.C	0.000	0.000	0.000	0.000	0.000	0.000	0.000	0.000	0.000
15.C	0.000	0.000	0.000	0.000	0.000	0.000	0.000	0.000	0.000
16.O	0.000	0.000	0.000	0.000	0.000	0.000	0.000	0.000	0.000
17.C	0.000	0.000	0.000	0.000	0.000	0.000	0.000	0.000	0.000

18.C	0.000	0.000	0.000	0.000	0.000	0.000	0.000	0.000	0.000
19.O	0.000	0.000	0.000	0.000	0.000	0.000	0.000	0.000	0.000
20.C	0.000	0.000	0.000	0.000	0.000	0.000	0.000	0.000	0.000
21.C	0.000	0.000	0.000	0.000	0.000	0.000	0.000	0.000	0.000
22.C	0.000	0.000	0.000	0.000	0.000	0.000	0.000	0.000	0.000
23.C	0.000	0.000	0.000	0.000	0.000	0.000	0.000	0.000	0.000
24.C	0.000	0.000	0.000	0.000	0.000	0.000	0.000	0.000	0.000
25.C	0.000	0.000	0.000	0.000	0.000	0.000	0.000	0.000	0.000
26.C	0.000	0.000	0.000	0.000	0.000	0.000	0.000	0.000	0.000
27.C	0.000	0.000	0.000	0.000	0.000	0.000	0.000	0.000	0.000
28.C	0.000	0.000	0.000	0.000	0.000	0.000	0.000	0.000	0.000
29.C	0.000	0.000	0.000	0.000	0.000	0.000	0.000	0.000	0.000
30.C	0.000	0.000	0.000	0.000	0.000	0.000	0.000	0.000	0.000
31.O	0.000	0.000	0.000	0.000	0.000	0.000	0.000	0.000	0.000
32.C	0.000	0.000	0.000	0.000	0.000	0.000	0.000	0.000	0.000
33.C	0.000	0.000	0.000	0.000	0.000	0.000	0.000	0.000	0.000
34.C	0.000	0.000	0.000	0.000	0.000	0.000	0.000	0.000	0.000
35.C	0.000	0.000	0.000	0.000	0.000	0.000	0.000	0.000	0.000
36.C	0.000	0.000	0.000	0.000	0.000	0.000	0.000	0.000	0.000
37.C	0.000	0.000	0.000	0.000	0.000	0.000	0.000	0.000	0.000
38.C	0.000	0.000	0.000	0.000	0.000	0.000	0.000	0.000	0.000
39.C	0.000	0.000	0.000	0.000	0.000	0.000	0.000	0.000	0.000
40.H	0.000	0.000	0.000	0.000	0.000	0.000	0.000	0.000	0.000
41.H	0.000	0.000	0.000	0.000	0.000	0.000	0.000	0.000	0.000
42.H	0.000	0.000	0.000	0.000	0.000	0.000	0.000	0.000	0.000
43.H	0.000	0.000	0.000	0.000	0.000	0.000	0.000	0.000	0.000
44.H	0.000	0.000	0.000	0.000	0.000	0.000	0.000	0.000	0.000
45.H	0.000	0.000	0.000	0.000	0.000	0.000	0.000	0.000	0.000
46.H	0.000	0.000	0.000	0.000	0.000	0.000	0.000	0.000	0.000
47.H	0.000	0.000	0.000	0.000	0.000	0.000	0.000	0.000	0.000
48.H	0.000	0.000	0.000	0.000	0.000	0.000	0.000	0.000	0.000

49.H	0.000	0.000	0.000	0.000	0.000	0.000	0.000	0.000	0.000
50.H	0.000	0.000	0.000	0.000	0.000	0.000	0.000	0.000	0.000
51.H	0.000	0.000	0.000	0.000	0.000	0.000	0.000	0.000	0.000
52.H	0.000	0.000	0.000	0.000	0.000	0.000	0.000	0.000	0.000
53.H	0.000	0.000	0.000	0.000	0.000	0.000	0.000	0.000	0.000
54.H	0.000	0.000	0.000	0.000	0.000	0.000	0.000	0.000	0.000
55.H	0.000	0.000	0.000	0.000	0.000	0.000	0.000	0.000	0.000
56.H	0.000	0.000	0.000	0.000	0.000	0.000	0.000	0.000	0.000
57.H	0.000	0.000	0.000	0.000	0.000	0.000	0.000	0.000	0.000
58.H	0.000	0.000	0.000	0.000	0.000	0.000	0.000	0.000	0.000
59.H	0.000	0.000	0.000	0.000	0.000	0.000	0.000	0.000	0.000
60.H	0.000	0.000	0.000	0.000	0.000	0.000	0.000	0.000	0.000
61.H	0.000	0.000	0.000	0.000	0.000	0.000	0.000	0.000	0.000
62.H	0.000	0.000	0.000	0.000	0.000	0.000	0.000	0.000	0.000
63.H	0.000	0.000	0.000	0.000	0.000	0.000	0.000	0.000	0.000
64.H	0.000	0.000	0.000	0.000	0.000	0.000	0.000	0.000	0.000
65.H	0.000	0.000	0.000	0.000	0.000	0.000	0.000	0.000	0.000
66.H	0.000	0.000	0.000	0.000	0.000	0.000	0.000	0.000	0.000
67.H	0.000	0.000	0.000	0.000	0.000	0.000	0.000	0.000	0.000
68.H	0.000	0.000	0.000	0.000	0.000	0.000	0.000	0.000	0.000
69.C	0.000	0.000	0.000	0.000	0.000	0.000	0.000	0.000	0.000
70.H	0.000	0.000	0.000	0.000	0.000	0.000	0.000	0.000	0.000
71.H	0.000	0.000	0.000	0.000	0.000	0.000	0.000	0.000	0.000
72.H	0.000	0.000	0.000	0.000	0.000	0.000	0.000	0.000	0.000
73.H	0.954	-0.022	0.002	0.000	0.932	0.056	0.000	0.000	0.949
74.H	-0.090	0.005	0.023	-0.043	-0.073	0.068	0.017	0.024	-0.097
75.H	-0.094	-0.006	0.018	-0.027	-0.116	0.056	0.002	0.056	-0.089
76.H	-0.061	0.029	0.026	-0.013	-0.008	0.006	0.005	-0.020	-0.043
77.H	-0.079	0.023	0.031	-0.033	-0.030	0.042	0.014	-0.004	-0.071
78.H	-0.073	0.031	0.033	-0.013	-0.027	0.011	-0.001	-0.004	-0.044
79.H	-0.050	0.017	0.012	0.024	-0.047	-0.038	-0.022	0.008	-0.011

80.H	-0.062	0.004	0.009	0.027	-0.094	-0.031	-0.029	0.042	-0.020
81.H	-0.063	0.020	0.021	0.022	-0.063	-0.031	-0.025	0.023	-0.014
82.H	-0.062	-0.015	-0.005	-0.008	-0.094	0.022	0.000	0.033	-0.070
83.H	-0.059	-0.022	-0.011	-0.028	-0.080	0.047	0.019	0.018	-0.095
84.H	-0.063	0.012	0.015	-0.026	-0.029	0.031	0.015	-0.010	-0.069
85.H	-0.070	-0.006	0.006	0.002	-0.102	0.008	-0.012	0.044	-0.054
86.H	0.002	0.007	-0.020	0.032	0.021	-0.068	-0.005	-0.055	-0.000
87.O	0.000	0.000	0.000	0.000	0.000	0.000	0.000	0.000	0.000
88.H	-0.016	-0.033	-0.040	0.022	-0.072	-0.029	-0.004	0.006	-0.043
89.H	-0.017	0.026	0.003	0.026	0.027	-0.061	-0.008	-0.052	0.003
90.H	-0.023	0.012	-0.004	0.037	-0.020	-0.065	-0.021	-0.018	0.004
91.H	-0.020	0.011	-0.007	0.023	-0.001	-0.048	-0.007	-0.034	-0.012
92.C	0.000	0.000	0.000	0.000	0.000	0.000	0.000	0.000	0.000
93.O	0.000	0.000	0.000	0.000	0.000	0.000	0.000	0.000	0.000
94.H	-0.020	-0.023	-0.031	-0.019	-0.021	0.018	0.028	-0.033	-0.079
95.H	-0.026	-0.039	-0.039	-0.011	-0.067	0.019	0.019	-0.000	-0.084
96.H	-0.008	-0.031	-0.043	0.003	-0.036	-0.011	0.015	-0.024	-0.060

	229			230			231		
	------------------------			------------------------			------------------------		
1.O	0.000	0.000	0.000	0.000	0.000	0.000	0.000	0.000	0.000
2.C	0.000	0.000	0.000	0.000	0.000	0.000	0.000	0.000	0.000
3.C	0.000	0.000	0.000	0.000	0.000	0.000	0.000	0.000	0.000
4.C	0.000	0.000	0.000	0.000	0.000	0.000	0.000	0.000	0.000
5.O	0.000	0.000	0.000	0.000	0.000	0.000	0.000	0.000	0.000
6.N	0.000	0.000	0.000	0.000	0.000	0.000	0.000	0.000	0.000
7.O	0.000	0.000	0.000	0.000	0.000	0.000	0.000	0.000	0.000
8.C	0.000	0.000	0.000	0.000	0.000	0.000	0.000	0.000	0.000
9.C	0.000	0.000	0.000	0.000	0.000	0.000	0.000	0.000	0.000
10.C	0.000	0.000	0.000	0.000	0.000	0.000	0.000	0.000	0.000

11.O	0.000	0.000	0.000	0.000	0.000	0.000	0.000	0.000	0.000
12.C	0.000	0.000	0.000	0.000	0.000	0.000	0.000	0.000	0.000
13.O	0.000	0.000	0.000	0.000	0.000	0.000	0.000	0.000	0.000
14.C	0.000	0.000	0.000	0.000	0.000	0.000	0.000	0.000	0.000
15.C	0.000	0.000	0.000	0.000	0.000	0.000	0.000	0.000	0.000
16.O	0.000	0.000	0.000	0.000	0.000	0.000	0.000	0.000	0.000
17.C	0.000	0.000	0.000	0.000	0.000	0.000	0.000	0.000	0.000
18.C	0.000	0.000	0.000	0.000	0.000	0.000	0.000	0.000	0.000
19.O	0.000	0.000	0.000	0.000	0.000	0.000	0.000	0.000	0.000
20.C	0.000	0.000	0.000	0.000	0.000	0.000	0.000	0.000	0.000
21.C	0.000	0.000	0.000	0.000	0.000	0.000	0.000	0.000	0.000
22.C	0.000	0.000	0.000	0.000	0.000	0.000	0.000	0.000	0.000
23.C	0.000	0.000	0.000	0.000	0.000	0.000	0.000	0.000	0.000
24.C	0.000	0.000	0.000	0.000	0.000	0.000	0.000	0.000	0.000
25.C	0.000	0.000	0.000	0.000	0.000	0.000	0.000	0.000	0.000
26.C	0.000	0.000	0.000	0.000	0.000	0.000	0.000	0.000	0.000
27.C	0.000	0.000	0.000	0.000	0.000	0.000	0.000	0.000	0.000
28.C	0.000	0.000	0.000	0.000	0.000	0.000	0.000	0.000	0.000
29.C	0.000	0.000	0.000	0.000	0.000	0.000	0.000	0.000	0.000
30.C	0.000	0.000	0.000	0.000	0.000	0.000	0.000	0.000	0.000
31.O	0.000	0.000	0.000	0.000	0.000	0.000	0.000	0.000	0.000
32.C	0.000	0.000	0.000	0.000	0.000	0.000	0.000	0.000	0.000
33.C	0.000	0.000	0.000	0.000	0.000	0.000	0.000	0.000	0.000
34.C	0.000	0.000	0.000	0.000	0.000	0.000	0.000	0.000	0.000
35.C	0.000	0.000	0.000	0.000	0.000	0.000	0.000	0.000	0.000
36.C	0.000	0.000	0.000	0.000	0.000	0.000	0.000	0.000	0.000
37.C	0.000	0.000	0.000	0.000	0.000	0.000	0.000	0.000	0.000
38.C	0.000	0.000	0.000	0.000	0.000	0.000	0.000	0.000	0.000
39.C	0.000	0.000	0.000	0.000	0.000	0.000	0.000	0.000	0.000
40.H	0.000	0.000	0.000	0.000	0.000	0.000	0.000	0.000	0.000
41.H	0.000	0.000	0.000	0.000	0.000	0.000	0.000	0.000	0.000

42.H	0.000	0.000	0.000	0.000	0.000	0.000	0.000	0.000	0.000
43.H	0.000	0.000	0.000	0.000	0.000	0.000	0.000	0.000	0.000
44.H	0.000	0.000	0.000	0.000	0.000	0.000	0.000	0.000	0.000
45.H	0.000	0.000	0.000	0.000	0.000	0.000	0.000	0.000	0.000
46.H	0.000	0.000	0.000	0.000	0.000	0.000	0.000	0.000	0.000
47.H	0.000	0.000	0.000	0.000	0.000	0.000	0.000	0.000	0.000
48.H	0.000	0.000	0.000	0.000	0.000	0.000	0.000	0.000	0.000
49.H	0.000	0.000	0.000	0.000	0.000	0.000	0.000	0.000	0.000
50.H	0.000	0.000	0.000	0.000	0.000	0.000	0.000	0.000	0.000
51.H	0.000	0.000	0.000	0.000	0.000	0.000	0.000	0.000	0.000
52.H	0.000	0.000	0.000	0.000	0.000	0.000	0.000	0.000	0.000
53.H	0.000	0.000	0.000	0.000	0.000	0.000	0.000	0.000	0.000
54.H	0.000	0.000	0.000	0.000	0.000	0.000	0.000	0.000	0.000
55.H	0.000	0.000	0.000	0.000	0.000	0.000	0.000	0.000	0.000
56.H	0.000	0.000	0.000	0.000	0.000	0.000	0.000	0.000	0.000
57.H	0.000	0.000	0.000	0.000	0.000	0.000	0.000	0.000	0.000
58.H	0.000	0.000	0.000	0.000	0.000	0.000	0.000	0.000	0.000
59.H	0.000	0.000	0.000	0.000	0.000	0.000	0.000	0.000	0.000
60.H	0.000	0.000	0.000	0.000	0.000	0.000	0.000	0.000	0.000
61.H	0.000	0.000	0.000	0.000	0.000	0.000	0.000	0.000	0.000
62.H	0.000	0.000	0.000	0.000	0.000	0.000	0.000	0.000	0.000
63.H	0.000	0.000	0.000	0.000	0.000	0.000	0.000	0.000	0.000
64.H	0.000	0.000	0.000	0.000	0.000	0.000	0.000	0.000	0.000
65.H	0.000	0.000	0.000	0.000	0.000	0.000	0.000	0.000	0.000
66.H	0.000	0.000	0.000	0.000	0.000	0.000	0.000	0.000	0.000
67.H	0.000	0.000	0.000	0.000	0.000	0.000	0.000	0.000	0.000
68.H	0.000	0.000	0.000	0.000	0.000	0.000	0.000	0.000	0.000
69.C	0.000	0.000	0.000	0.000	0.000	0.000	0.000	0.000	0.000
70.H	0.000	0.000	0.000	0.000	0.000	0.000	0.000	0.000	0.000
71.H	0.000	0.000	0.000	0.000	0.000	0.000	0.000	0.000	0.000
72.H	0.000	0.000	0.000	0.000	0.000	0.000	0.000	0.000	0.000

73.H	0.000	0.000	0.000	0.000	0.000	0.000	0.000	0.000	0.000
74.H	0.941	0.002	0.038	0.000	0.959	0.035	0.000	0.000	0.929
75.H	-0.109	-0.020	0.030	0.004	-0.092	0.029	0.037	0.083	-0.123
76.H	-0.072	0.051	0.021	0.001	-0.060	0.008	0.015	-0.022	-0.082
77.H	-0.099	0.040	0.036	0.002	-0.069	0.024	0.033	0.001	-0.119
78.H	-0.082	0.052	0.028	0.002	-0.076	0.011	0.020	0.003	-0.091
79.H	-0.041	0.027	-0.002	0.002	-0.073	-0.014	-0.009	0.017	-0.029
80.H	-0.050	-0.001	-0.002	0.003	-0.089	-0.011	-0.005	0.065	-0.033
81.H	-0.053	0.029	0.006	0.003	-0.087	-0.010	-0.002	0.039	-0.042
82.H	-0.070	-0.033	0.004	0.001	-0.064	0.010	0.013	0.047	-0.073
83.H	-0.079	-0.043	0.008	-0.000	-0.046	0.021	0.021	0.024	-0.093
84.H	-0.081	0.021	0.021	0.001	-0.054	0.017	0.022	-0.010	-0.097
85.H	-0.071	-0.019	0.007	0.003	-0.082	0.006	0.011	0.065	-0.068
86.H	0.010	0.019	-0.034	-0.004	-0.011	-0.032	-0.037	-0.078	0.024
87.O	0.000	0.000	0.000	0.000	0.000	0.000	0.000	0.000	0.000
88.H	-0.012	-0.061	-0.036	-0.002	-0.026	-0.017	-0.023	0.004	0.001
89.H	-0.010	0.051	-0.016	-0.002	-0.031	-0.026	-0.025	-0.069	0.000
90.H	-0.010	0.022	-0.022	-0.001	-0.047	-0.029	-0.028	-0.022	0.010
91.H	-0.015	0.022	-0.018	-0.002	-0.032	-0.021	-0.022	-0.046	-0.005
92.C	0.000	0.000	0.000	0.000	0.000	0.000	0.000	0.000	0.000
93.O	0.000	0.000	0.000	0.000	0.000	0.000	0.000	0.000	0.000
94.H	-0.039	-0.037	-0.015	-0.004	-0.003	0.005	-0.001	-0.052	-0.051
95.H	-0.040	-0.070	-0.021	-0.003	-0.015	0.004	-0.002	-0.008	-0.045
96.H	-0.016	-0.053	-0.033	-0.004	-0.002	-0.009	-0.017	-0.041	-0.014

	232			233			234		
1.O	0.000	0.000	0.000	0.000	0.000	0.000	0.000	0.000	0.000
2.C	0.000	0.000	0.000	0.000	0.000	0.000	0.000	0.000	0.000
3.C	0.000	0.000	0.000	0.000	0.000	0.000	0.000	0.000	0.000

4.C	0.000	0.000	0.000	0.000	0.000	0.000	0.000	0.000	0.000
5.O	0.000	0.000	0.000	0.000	0.000	0.000	0.000	0.000	0.000
6.N	0.000	0.000	0.000	0.000	0.000	0.000	0.000	0.000	0.000
7.O	0.000	0.000	0.000	0.000	0.000	0.000	0.000	0.000	0.000
8.C	0.000	0.000	0.000	0.000	0.000	0.000	0.000	0.000	0.000
9.C	0.000	0.000	0.000	0.000	0.000	0.000	0.000	0.000	0.000
10.C	0.000	0.000	0.000	0.000	0.000	0.000	0.000	0.000	0.000
11.O	0.000	0.000	0.000	0.000	0.000	0.000	0.000	0.000	0.000
12.C	0.000	0.000	0.000	0.000	0.000	0.000	0.000	0.000	0.000
13.O	0.000	0.000	0.000	0.000	0.000	0.000	0.000	0.000	0.000
14.C	0.000	0.000	0.000	0.000	0.000	0.000	0.000	0.000	0.000
15.C	0.000	0.000	0.000	0.000	0.000	0.000	0.000	0.000	0.000
16.O	0.000	0.000	0.000	0.000	0.000	0.000	0.000	0.000	0.000
17.C	0.000	0.000	0.000	0.000	0.000	0.000	0.000	0.000	0.000
18.C	0.000	0.000	0.000	0.000	0.000	0.000	0.000	0.000	0.000
19.O	0.000	0.000	0.000	0.000	0.000	0.000	0.000	0.000	0.000
20.C	0.000	0.000	0.000	0.000	0.000	0.000	0.000	0.000	0.000
21.C	0.000	0.000	0.000	0.000	0.000	0.000	0.000	0.000	0.000
22.C	0.000	0.000	0.000	0.000	0.000	0.000	0.000	0.000	0.000
23.C	0.000	0.000	0.000	0.000	0.000	0.000	0.000	0.000	0.000
24.C	0.000	0.000	0.000	0.000	0.000	0.000	0.000	0.000	0.000
25.C	0.000	0.000	0.000	0.000	0.000	0.000	0.000	0.000	0.000
26.C	0.000	0.000	0.000	0.000	0.000	0.000	0.000	0.000	0.000
27.C	0.000	0.000	0.000	0.000	0.000	0.000	0.000	0.000	0.000
28.C	0.000	0.000	0.000	0.000	0.000	0.000	0.000	0.000	0.000
29.C	0.000	0.000	0.000	0.000	0.000	0.000	0.000	0.000	0.000
30.C	0.000	0.000	0.000	0.000	0.000	0.000	0.000	0.000	0.000
31.O	0.000	0.000	0.000	0.000	0.000	0.000	0.000	0.000	0.000
32.C	0.000	0.000	0.000	0.000	0.000	0.000	0.000	0.000	0.000
33.C	0.000	0.000	0.000	0.000	0.000	0.000	0.000	0.000	0.000
34.C	0.000	0.000	0.000	0.000	0.000	0.000	0.000	0.000	0.000

35.C	0.000	0.000	0.000	0.000	0.000	0.000	0.000	0.000	0.000
36.C	0.000	0.000	0.000	0.000	0.000	0.000	0.000	0.000	0.000
37.C	0.000	0.000	0.000	0.000	0.000	0.000	0.000	0.000	0.000
38.C	0.000	0.000	0.000	0.000	0.000	0.000	0.000	0.000	0.000
39.C	0.000	0.000	0.000	0.000	0.000	0.000	0.000	0.000	0.000
40.H	0.000	0.000	0.000	0.000	0.000	0.000	0.000	0.000	0.000
41.H	0.000	0.000	0.000	0.000	0.000	0.000	0.000	0.000	0.000
42.H	0.000	0.000	0.000	0.000	0.000	0.000	0.000	0.000	0.000
43.H	0.000	0.000	0.000	0.000	0.000	0.000	0.000	0.000	0.000
44.H	0.000	0.000	0.000	0.000	0.000	0.000	0.000	0.000	0.000
45.H	0.000	0.000	0.000	0.000	0.000	0.000	0.000	0.000	0.000
46.H	0.000	0.000	0.000	0.000	0.000	0.000	0.000	0.000	0.000
47.H	0.000	0.000	0.000	0.000	0.000	0.000	0.000	0.000	0.000
48.H	0.000	0.000	0.000	0.000	0.000	0.000	0.000	0.000	0.000
49.H	0.000	0.000	0.000	0.000	0.000	0.000	0.000	0.000	0.000
50.H	0.000	0.000	0.000	0.000	0.000	0.000	0.000	0.000	0.000
51.H	0.000	0.000	0.000	0.000	0.000	0.000	0.000	0.000	0.000
52.H	0.000	0.000	0.000	0.000	0.000	0.000	0.000	0.000	0.000
53.H	0.000	0.000	0.000	0.000	0.000	0.000	0.000	0.000	0.000
54.H	0.000	0.000	0.000	0.000	0.000	0.000	0.000	0.000	0.000
55.H	0.000	0.000	0.000	0.000	0.000	0.000	0.000	0.000	0.000
56.H	0.000	0.000	0.000	0.000	0.000	0.000	0.000	0.000	0.000
57.H	0.000	0.000	0.000	0.000	0.000	0.000	0.000	0.000	0.000
58.H	0.000	0.000	0.000	0.000	0.000	0.000	0.000	0.000	0.000
59.H	0.000	0.000	0.000	0.000	0.000	0.000	0.000	0.000	0.000
60.H	0.000	0.000	0.000	0.000	0.000	0.000	0.000	0.000	0.000
61.H	0.000	0.000	0.000	0.000	0.000	0.000	0.000	0.000	0.000
62.H	0.000	0.000	0.000	0.000	0.000	0.000	0.000	0.000	0.000
63.H	0.000	0.000	0.000	0.000	0.000	0.000	0.000	0.000	0.000
64.H	0.000	0.000	0.000	0.000	0.000	0.000	0.000	0.000	0.000
65.H	0.000	0.000	0.000	0.000	0.000	0.000	0.000	0.000	0.000

66.H	0.000	0.000	0.000	0.000	0.000	0.000	0.000	0.000	0.000
67.H	0.000	0.000	0.000	0.000	0.000	0.000	0.000	0.000	0.000
68.H	0.000	0.000	0.000	0.000	0.000	0.000	0.000	0.000	0.000
69.C	0.000	0.000	0.000	0.000	0.000	0.000	0.000	0.000	0.000
70.H	0.000	0.000	0.000	0.000	0.000	0.000	0.000	0.000	0.000
71.H	0.000	0.000	0.000	0.000	0.000	0.000	0.000	0.000	0.000
72.H	0.000	0.000	0.000	0.000	0.000	0.000	0.000	0.000	0.000
73.H	0.000	0.000	0.000	0.000	0.000	0.000	0.000	0.000	0.000
74.H	0.000	0.000	0.000	0.000	0.000	0.000	0.000	0.000	0.000
75.H	0.933	-0.016	0.033	0.000	0.921	0.081	0.000	0.000	0.931
76.H	-0.080	0.043	0.044	-0.010	-0.044	0.023	0.017	-0.019	-0.089
77.H	-0.108	0.034	0.054	-0.023	-0.070	0.067	0.037	0.006	-0.129
78.H	-0.098	0.045	0.055	-0.009	-0.076	0.030	0.022	0.007	-0.098
79.H	-0.062	0.023	0.019	0.014	-0.086	-0.035	-0.009	0.023	-0.033
80.H	-0.080	0.001	0.014	0.017	-0.138	-0.027	-0.006	0.073	-0.038
81.H	-0.082	0.025	0.032	0.014	-0.115	-0.024	-0.002	0.046	-0.046
82.H	-0.082	-0.027	-0.003	-0.007	-0.106	0.033	0.015	0.055	-0.083
83.H	-0.078	-0.036	-0.012	-0.021	-0.074	0.063	0.026	0.031	-0.105
84.H	-0.084	0.018	0.028	-0.019	-0.050	0.050	0.026	-0.006	-0.107
85.H	-0.093	-0.014	0.012	0.001	-0.134	0.019	0.012	0.075	-0.076
86.H	0.018	0.015	-0.033	0.017	0.037	-0.080	-0.035	-0.079	0.022
87.O	0.000	0.000	0.000	0.000	0.000	0.000	0.000	0.000	0.000
88.H	-0.010	-0.051	-0.062	0.011	-0.044	-0.038	-0.022	0.009	-0.006
89.H	-0.011	0.042	0.003	0.014	0.016	-0.065	-0.024	-0.069	-0.002
90.H	-0.021	0.019	-0.008	0.022	-0.036	-0.072	-0.028	-0.018	0.007
91.H	-0.016	0.018	-0.011	0.011	-0.004	-0.052	-0.020	-0.045	-0.009
92.C	0.000	0.000	0.000	0.000	0.000	0.000	0.000	0.000	0.000
93.O	0.000	0.000	0.000	0.000	0.000	0.000	0.000	0.000	0.000
94.H	-0.018	-0.032	-0.044	-0.017	0.020	0.022	0.004	-0.049	-0.061
95.H	-0.027	-0.059	-0.057	-0.011	-0.027	0.020	0.002	-0.002	-0.056
96.H	0.001	-0.046	-0.065	-0.003	0.010	-0.016	-0.014	-0.038	-0.023

	235			236			237		
1.O	0.000	0.000	0.000	0.000	0.000	0.000	0.000	0.000	0.000
2.C	0.000	0.000	0.000	0.000	0.000	0.000	0.000	0.000	0.000
3.C	0.000	0.000	0.000	0.000	0.000	0.000	0.000	0.000	0.000
4.C	0.000	0.000	0.000	0.000	0.000	0.000	0.000	0.000	0.000
5.O	0.000	0.000	0.000	0.000	0.000	0.000	0.000	0.000	0.000
6.N	0.000	0.000	0.000	0.000	0.000	0.000	0.000	0.000	0.000
7.O	0.000	0.000	0.000	0.000	0.000	0.000	0.000	0.000	0.000
8.C	0.000	0.000	0.000	0.000	0.000	0.000	0.000	0.000	0.000
9.C	0.000	0.000	0.000	0.000	0.000	0.000	0.000	0.000	0.000
10.C	0.000	0.000	0.000	0.000	0.000	0.000	0.000	0.000	0.000
11.O	0.000	0.000	0.000	0.000	0.000	0.000	0.000	0.000	0.000
12.C	0.000	0.000	0.000	0.000	0.000	0.000	0.000	0.000	0.000
13.O	0.000	0.000	0.000	0.000	0.000	0.000	0.000	0.000	0.000
14.C	0.000	0.000	0.000	0.000	0.000	0.000	0.000	0.000	0.000
15.C	0.000	0.000	0.000	0.000	0.000	0.000	0.000	0.000	0.000
16.O	0.000	0.000	0.000	0.000	0.000	0.000	0.000	0.000	0.000
17.C	0.000	0.000	0.000	0.000	0.000	0.000	0.000	0.000	0.000
18.C	0.000	0.000	0.000	0.000	0.000	0.000	0.000	0.000	0.000
19.O	0.000	0.000	0.000	0.000	0.000	0.000	0.000	0.000	0.000
20.C	0.000	0.000	0.000	0.000	0.000	0.000	0.000	0.000	0.000
21.C	0.000	0.000	0.000	0.000	0.000	0.000	0.000	0.000	0.000
22.C	0.000	0.000	0.000	0.000	0.000	0.000	0.000	0.000	0.000
23.C	0.000	0.000	0.000	0.000	0.000	0.000	0.000	0.000	0.000
24.C	0.000	0.000	0.000	0.000	0.000	0.000	0.000	0.000	0.000
25.C	0.000	0.000	0.000	0.000	0.000	0.000	0.000	0.000	0.000
26.C	0.000	0.000	0.000	0.000	0.000	0.000	0.000	0.000	0.000
27.C	0.000	0.000	0.000	0.000	0.000	0.000	0.000	0.000	0.000

28.C	0.000	0.000	0.000	0.000	0.000	0.000	0.000	0.000	0.000
29.C	0.000	0.000	0.000	0.000	0.000	0.000	0.000	0.000	0.000
30.C	0.000	0.000	0.000	0.000	0.000	0.000	0.000	0.000	0.000
31.O	0.000	0.000	0.000	0.000	0.000	0.000	0.000	0.000	0.000
32.C	0.000	0.000	0.000	0.000	0.000	0.000	0.000	0.000	0.000
33.C	0.000	0.000	0.000	0.000	0.000	0.000	0.000	0.000	0.000
34.C	0.000	0.000	0.000	0.000	0.000	0.000	0.000	0.000	0.000
35.C	0.000	0.000	0.000	0.000	0.000	0.000	0.000	0.000	0.000
36.C	0.000	0.000	0.000	0.000	0.000	0.000	0.000	0.000	0.000
37.C	0.000	0.000	0.000	0.000	0.000	0.000	0.000	0.000	0.000
38.C	0.000	0.000	0.000	0.000	0.000	0.000	0.000	0.000	0.000
39.C	0.000	0.000	0.000	0.000	0.000	0.000	0.000	0.000	0.000
40.H	0.000	0.000	0.000	0.000	0.000	0.000	0.000	0.000	0.000
41.H	0.000	0.000	0.000	0.000	0.000	0.000	0.000	0.000	0.000
42.H	0.000	0.000	0.000	0.000	0.000	0.000	0.000	0.000	0.000
43.H	0.000	0.000	0.000	0.000	0.000	0.000	0.000	0.000	0.000
44.H	0.000	0.000	0.000	0.000	0.000	0.000	0.000	0.000	0.000
45.H	0.000	0.000	0.000	0.000	0.000	0.000	0.000	0.000	0.000
46.H	0.000	0.000	0.000	0.000	0.000	0.000	0.000	0.000	0.000
47.H	0.000	0.000	0.000	0.000	0.000	0.000	0.000	0.000	0.000
48.H	0.000	0.000	0.000	0.000	0.000	0.000	0.000	0.000	0.000
49.H	0.000	0.000	0.000	0.000	0.000	0.000	0.000	0.000	0.000
50.H	0.000	0.000	0.000	0.000	0.000	0.000	0.000	0.000	0.000
51.H	0.000	0.000	0.000	0.000	0.000	0.000	0.000	0.000	0.000
52.H	0.000	0.000	0.000	0.000	0.000	0.000	0.000	0.000	0.000
53.H	0.000	0.000	0.000	0.000	0.000	0.000	0.000	0.000	0.000
54.H	0.000	0.000	0.000	0.000	0.000	0.000	0.000	0.000	0.000
55.H	0.000	0.000	0.000	0.000	0.000	0.000	0.000	0.000	0.000
56.H	0.000	0.000	0.000	0.000	0.000	0.000	0.000	0.000	0.000
57.H	0.000	0.000	0.000	0.000	0.000	0.000	0.000	0.000	0.000
58.H	0.000	0.000	0.000	0.000	0.000	0.000	0.000	0.000	0.000

59.H	0.000	0.000	0.000	0.000	0.000	0.000	0.000	0.000	0.000
60.H	0.000	0.000	0.000	0.000	0.000	0.000	0.000	0.000	0.000
61.H	0.000	0.000	0.000	0.000	0.000	0.000	0.000	0.000	0.000
62.H	0.000	0.000	0.000	0.000	0.000	0.000	0.000	0.000	0.000
63.H	0.000	0.000	0.000	0.000	0.000	0.000	0.000	0.000	0.000
64.H	0.000	0.000	0.000	0.000	0.000	0.000	0.000	0.000	0.000
65.H	0.000	0.000	0.000	0.000	0.000	0.000	0.000	0.000	0.000
66.H	0.000	0.000	0.000	0.000	0.000	0.000	0.000	0.000	0.000
67.H	0.000	0.000	0.000	0.000	0.000	0.000	0.000	0.000	0.000
68.H	0.000	0.000	0.000	0.000	0.000	0.000	0.000	0.000	0.000
69.C	0.000	0.000	0.000	0.000	0.000	0.000	0.000	0.000	0.000
70.H	0.000	0.000	0.000	0.000	0.000	0.000	0.000	0.000	0.000
71.H	0.000	0.000	0.000	0.000	0.000	0.000	0.000	0.000	0.000
72.H	0.000	0.000	0.000	0.000	0.000	0.000	0.000	0.000	0.000
73.H	0.000	0.000	0.000	0.000	0.000	0.000	0.000	0.000	0.000
74.H	0.000	0.000	0.000	0.000	0.000	0.000	0.000	0.000	0.000
75.H	0.000	0.000	0.000	0.000	0.000	0.000	0.000	0.000	0.000
76.H	0.962	0.023	0.015	0.000	0.937	-0.002	0.000	0.000	0.942
77.H	-0.083	0.017	0.023	0.052	-0.111	-0.015	0.031	0.006	-0.124
78.H	-0.076	0.022	0.019	0.029	-0.128	-0.003	0.033	0.008	-0.125
79.H	-0.056	0.010	0.002	-0.018	-0.097	0.013	0.022	0.012	-0.083
80.H	-0.061	-0.004	0.002	-0.018	-0.070	0.009	0.040	0.029	-0.077
81.H	-0.063	0.010	0.007	-0.012	-0.105	0.011	0.039	0.021	-0.098
82.H	-0.070	-0.017	0.004	0.021	-0.024	-0.012	0.021	0.019	-0.057
83.H	-0.073	-0.021	0.005	0.041	-0.007	-0.023	0.006	0.008	-0.048
84.H	-0.074	0.009	0.014	0.040	-0.084	-0.013	0.013	-0.001	-0.095
85.H	-0.071	-0.012	0.006	0.012	-0.047	-0.006	0.038	0.028	-0.075
86.H	-0.029	0.011	-0.017	-0.043	-0.067	0.020	-0.045	-0.030	-0.021
87.O	0.000	0.000	0.000	0.000	0.000	0.000	0.000	0.000	0.000
88.H	-0.040	-0.027	-0.020	-0.027	0.021	0.002	-0.026	-0.003	0.011
89.H	-0.040	0.025	-0.006	-0.031	-0.112	0.020	-0.023	-0.023	-0.063

90.H	-0.040	0.010	-0.009	-0.041	-0.084	0.021	-0.008	-0.005	-0.050
91.H	-0.042	0.011	-0.008	-0.026	-0.078	0.014	-0.021	-0.016	-0.047
92.C	0.000	0.000	0.000	0.000	0.000	0.000	0.000	0.000	0.000
93.O	0.000	0.000	0.000	0.000	0.000	0.000	0.000	0.000	0.000
94.H	-0.052	-0.015	-0.008	0.019	0.001	-0.014	-0.042	-0.024	-0.010
95.H	-0.053	-0.031	-0.012	0.012	0.035	-0.016	-0.031	-0.008	0.006
96.H	-0.041	-0.022	-0.018	-0.009	0.020	-0.005	-0.048	-0.021	0.015

	238			239			240		
1.O	0.000	0.000	0.000	0.000	0.000	0.000	0.000	0.000	0.000
2.C	0.000	0.000	0.000	0.000	0.000	0.000	0.000	0.000	0.000
3.C	0.000	0.000	0.000	0.000	0.000	0.000	0.000	0.000	0.000
4.C	0.000	0.000	0.000	0.000	0.000	0.000	0.000	0.000	0.000
5.O	0.000	0.000	0.000	0.000	0.000	0.000	0.000	0.000	0.000
6.N	0.000	0.000	0.000	0.000	0.000	0.000	0.000	0.000	0.000
7.O	0.000	0.000	0.000	0.000	0.000	0.000	0.000	0.000	0.000
8.C	0.000	0.000	0.000	0.000	0.000	0.000	0.000	0.000	0.000
9.C	0.000	0.000	0.000	0.000	0.000	0.000	0.000	0.000	0.000
10.C	0.000	0.000	0.000	0.000	0.000	0.000	0.000	0.000	0.000
11.O	0.000	0.000	0.000	0.000	0.000	0.000	0.000	0.000	0.000
12.C	0.000	0.000	0.000	0.000	0.000	0.000	0.000	0.000	0.000
13.O	0.000	0.000	0.000	0.000	0.000	0.000	0.000	0.000	0.000
14.C	0.000	0.000	0.000	0.000	0.000	0.000	0.000	0.000	0.000
15.C	0.000	0.000	0.000	0.000	0.000	0.000	0.000	0.000	0.000
16.O	0.000	0.000	0.000	0.000	0.000	0.000	0.000	0.000	0.000
17.C	0.000	0.000	0.000	0.000	0.000	0.000	0.000	0.000	0.000
18.C	0.000	0.000	0.000	0.000	0.000	0.000	0.000	0.000	0.000
19.O	0.000	0.000	0.000	0.000	0.000	0.000	0.000	0.000	0.000
20.C	0.000	0.000	0.000	0.000	0.000	0.000	0.000	0.000	0.000

21.C	0.000	0.000	0.000	0.000	0.000	0.000	0.000	0.000	0.000
22.C	0.000	0.000	0.000	0.000	0.000	0.000	0.000	0.000	0.000
23.C	0.000	0.000	0.000	0.000	0.000	0.000	0.000	0.000	0.000
24.C	0.000	0.000	0.000	0.000	0.000	0.000	0.000	0.000	0.000
25.C	0.000	0.000	0.000	0.000	0.000	0.000	0.000	0.000	0.000
26.C	0.000	0.000	0.000	0.000	0.000	0.000	0.000	0.000	0.000
27.C	0.000	0.000	0.000	0.000	0.000	0.000	0.000	0.000	0.000
28.C	0.000	0.000	0.000	0.000	0.000	0.000	0.000	0.000	0.000
29.C	0.000	0.000	0.000	0.000	0.000	0.000	0.000	0.000	0.000
30.C	0.000	0.000	0.000	0.000	0.000	0.000	0.000	0.000	0.000
31.O	0.000	0.000	0.000	0.000	0.000	0.000	0.000	0.000	0.000
32.C	0.000	0.000	0.000	0.000	0.000	0.000	0.000	0.000	0.000
33.C	0.000	0.000	0.000	0.000	0.000	0.000	0.000	0.000	0.000
34.C	0.000	0.000	0.000	0.000	0.000	0.000	0.000	0.000	0.000
35.C	0.000	0.000	0.000	0.000	0.000	0.000	0.000	0.000	0.000
36.C	0.000	0.000	0.000	0.000	0.000	0.000	0.000	0.000	0.000
37.C	0.000	0.000	0.000	0.000	0.000	0.000	0.000	0.000	0.000
38.C	0.000	0.000	0.000	0.000	0.000	0.000	0.000	0.000	0.000
39.C	0.000	0.000	0.000	0.000	0.000	0.000	0.000	0.000	0.000
40.H	0.000	0.000	0.000	0.000	0.000	0.000	0.000	0.000	0.000
41.H	0.000	0.000	0.000	0.000	0.000	0.000	0.000	0.000	0.000
42.H	0.000	0.000	0.000	0.000	0.000	0.000	0.000	0.000	0.000
43.H	0.000	0.000	0.000	0.000	0.000	0.000	0.000	0.000	0.000
44.H	0.000	0.000	0.000	0.000	0.000	0.000	0.000	0.000	0.000
45.H	0.000	0.000	0.000	0.000	0.000	0.000	0.000	0.000	0.000
46.H	0.000	0.000	0.000	0.000	0.000	0.000	0.000	0.000	0.000
47.H	0.000	0.000	0.000	0.000	0.000	0.000	0.000	0.000	0.000
48.H	0.000	0.000	0.000	0.000	0.000	0.000	0.000	0.000	0.000
49.H	0.000	0.000	0.000	0.000	0.000	0.000	0.000	0.000	0.000
50.H	0.000	0.000	0.000	0.000	0.000	0.000	0.000	0.000	0.000
51.H	0.000	0.000	0.000	0.000	0.000	0.000	0.000	0.000	0.000

52.H	0.000	0.000	0.000	0.000	0.000	0.000	0.000	0.000	0.000
53.H	0.000	0.000	0.000	0.000	0.000	0.000	0.000	0.000	0.000
54.H	0.000	0.000	0.000	0.000	0.000	0.000	0.000	0.000	0.000
55.H	0.000	0.000	0.000	0.000	0.000	0.000	0.000	0.000	0.000
56.H	0.000	0.000	0.000	0.000	0.000	0.000	0.000	0.000	0.000
57.H	0.000	0.000	0.000	0.000	0.000	0.000	0.000	0.000	0.000
58.H	0.000	0.000	0.000	0.000	0.000	0.000	0.000	0.000	0.000
59.H	0.000	0.000	0.000	0.000	0.000	0.000	0.000	0.000	0.000
60.H	0.000	0.000	0.000	0.000	0.000	0.000	0.000	0.000	0.000
61.H	0.000	0.000	0.000	0.000	0.000	0.000	0.000	0.000	0.000
62.H	0.000	0.000	0.000	0.000	0.000	0.000	0.000	0.000	0.000
63.H	0.000	0.000	0.000	0.000	0.000	0.000	0.000	0.000	0.000
64.H	0.000	0.000	0.000	0.000	0.000	0.000	0.000	0.000	0.000
65.H	0.000	0.000	0.000	0.000	0.000	0.000	0.000	0.000	0.000
66.H	0.000	0.000	0.000	0.000	0.000	0.000	0.000	0.000	0.000
67.H	0.000	0.000	0.000	0.000	0.000	0.000	0.000	0.000	0.000
68.H	0.000	0.000	0.000	0.000	0.000	0.000	0.000	0.000	0.000
69.C	0.000	0.000	0.000	0.000	0.000	0.000	0.000	0.000	0.000
70.H	0.000	0.000	0.000	0.000	0.000	0.000	0.000	0.000	0.000
71.H	0.000	0.000	0.000	0.000	0.000	0.000	0.000	0.000	0.000
72.H	0.000	0.000	0.000	0.000	0.000	0.000	0.000	0.000	0.000
73.H	0.000	0.000	0.000	0.000	0.000	0.000	0.000	0.000	0.000
74.H	0.000	0.000	0.000	0.000	0.000	0.000	0.000	0.000	0.000
75.H	0.000	0.000	0.000	0.000	0.000	0.000	0.000	0.000	0.000
76.H	0.000	0.000	0.000	0.000	0.000	0.000	0.000	0.000	0.000
77.H	0.935	0.044	0.054	0.000	0.936	0.005	0.000	0.000	0.907
78.H	-0.106	0.057	0.043	0.030	-0.144	0.007	0.054	0.008	-0.166
79.H	-0.059	0.028	0.003	-0.011	-0.115	0.004	0.018	0.024	-0.078
80.H	-0.069	-0.007	0.003	-0.010	-0.103	0.003	0.038	0.079	-0.076
81.H	-0.073	0.028	0.014	-0.005	-0.132	0.005	0.039	0.050	-0.102
82.H	-0.092	-0.039	0.013	0.022	-0.046	-0.001	0.037	0.058	-0.090

83.H	-0.102	-0.047	0.018	0.038	-0.019	-0.003	0.031	0.032	-0.098
84.H	-0.105	0.025	0.035	0.038	-0.091	0.002	0.038	-0.007	-0.143
85.H	-0.093	-0.026	0.017	0.015	-0.078	0.001	0.050	0.079	-0.102
86.H	0.001	0.031	-0.039	-0.037	-0.049	-0.001	-0.067	-0.086	0.020
87.O	0.000	0.000	0.000	0.000	0.000	0.000	0.000	0.000	0.000
88.H	-0.024	-0.063	-0.040	-0.022	0.017	-0.006	-0.038	0.009	0.031
89.H	-0.023	0.064	-0.015	-0.025	-0.101	0.003	-0.036	-0.075	-0.037
90.H	-0.022	0.028	-0.023	-0.033	-0.087	0.002	-0.025	-0.020	-0.018
91.H	-0.028	0.031	-0.018	-0.021	-0.073	0.001	-0.031	-0.049	-0.027
92.C	0.000	0.000	0.000	0.000	0.000	0.000	0.000	0.000	0.000
93.O	0.000	0.000	0.000	0.000	0.000	0.000	0.000	0.000	0.000
94.H	-0.055	-0.032	-0.012	0.016	0.016	-0.006	-0.032	-0.055	-0.030
95.H	-0.056	-0.072	-0.019	0.011	0.037	-0.008	-0.023	-0.004	-0.011
96.H	-0.028	-0.051	-0.035	-0.008	0.032	-0.007	-0.052	-0.042	0.021

	241			242			243		
1.O	0.000	0.000	0.000	0.000	0.000	0.000	0.000	0.000	0.000
2.C	0.000	0.000	0.000	0.000	0.000	0.000	0.000	0.000	0.000
3.C	0.000	0.000	0.000	0.000	0.000	0.000	0.000	0.000	0.000
4.C	0.000	0.000	0.000	0.000	0.000	0.000	0.000	0.000	0.000
5.O	0.000	0.000	0.000	0.000	0.000	0.000	0.000	0.000	0.000
6.N	0.000	0.000	0.000	0.000	0.000	0.000	0.000	0.000	0.000
7.O	0.000	0.000	0.000	0.000	0.000	0.000	0.000	0.000	0.000
8.C	0.000	0.000	0.000	0.000	0.000	0.000	0.000	0.000	0.000
9.C	0.000	0.000	0.000	0.000	0.000	0.000	0.000	0.000	0.000
[truncated: 425,216 more chars]
